# Supplementary material for: Dysregulation of U12-Type Splicing in Lupus Neutrophils
Source: bioRxiv. 2025 Nov 7:2025.11.06.686965. Preprint. [Version 1] doi: 10.1101/2025.11.06.686965 (PMC12637451; doi:10.1101/2025.11.06.686965)
Supplement: Supplement 1 [file media-1.pdf]

Supplementary Material.

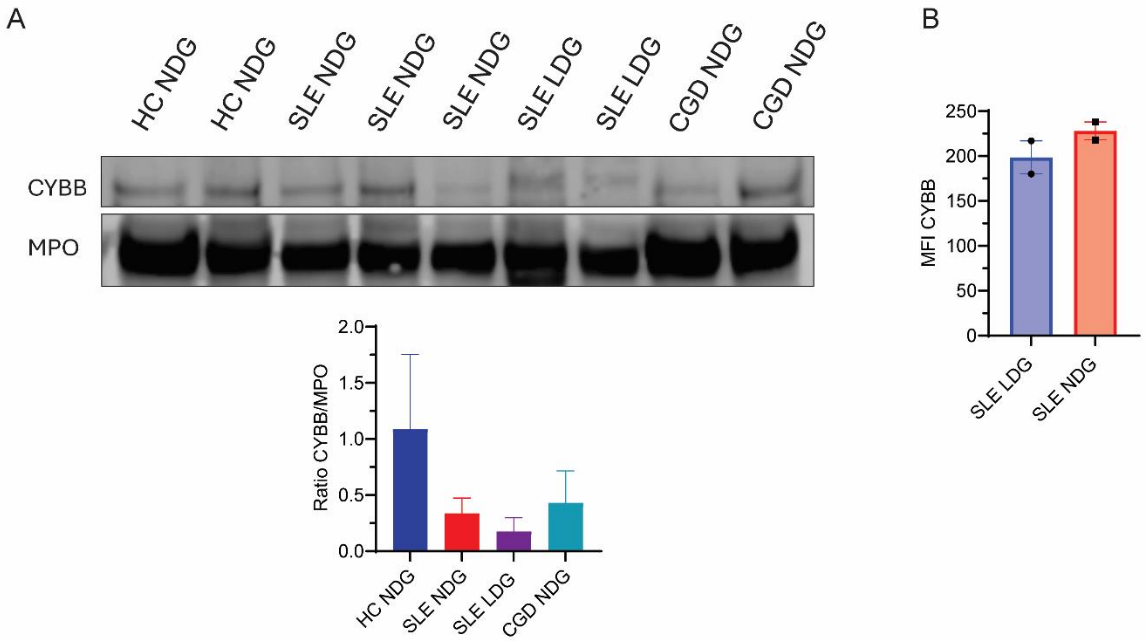

Figure 1S: Reduced expression of the NADPH oxidase component CYBB (NOX2) in SLE neutrophils. A) Western blot (top) and respective densitometry (Bottom) showing the expression of CYBB (NOX2) and MPO (as a housekeeping protein) in neutrophils from healthy controls or derived from SLE or CGD patients. B) Expression of NOX2 protein in neutrophils (NDGs and LDGs (from 2 SLE patients by flow cytometry. Shown average ± SEM.

Supplementary Data 1S
4_events_all
HC NDG versus SLE LDG

Splice_type	ID	Gene_ID	Gene_Symbol	Chromosome	Strand	LongExonStart_0base	LongExonEnd	ShortES	ShortEE	FlankingES	FlankingEE	Inclusion counts			Skipping counts			Inclusion counts		Skipping counts		IncFormLen	SkipFormLen	p-value	adjust_p	Inclusion_level(normalized)					Level_diff
												control_s3	control_s4	control_s10	control_s3	control_s4	control_s10	ldg_14	ldg_s16	ldg_s14	ldg_s16					IncLevel_s3	IncLevel_s4	IncLevel_s10	IncLevel14	IncLevel16	
SE	8881	CPEB2	CPEB2	chr4	+	15040463	15040487	15033160	15033211	15052413	15052584	14	15	2	1	0	0	0	1	5	1	73	49	1.71E-10	9.87E-07	0.904	1	1	0	0.402	0.767
SE	8677	MAEA	MAEA	chr4	+	1322380	1322503	1315396	1315600	1332756	1332865	11	5	2	0	4	0	0	0	1	1	98	49	5.28E-08	0.000152595	1	0.385	1	0	0	0.795
SE	12478	PQLC1	PQLC1	chr18	-	79933968	79934022	79919183	79919400	79943328	79943462	1	0	2	7	3	0	1	2	0	0	98	49	1.97E-07	0.000379884	0.067	0	1	1	1	-0.644
SE	10324	IRF7	IRF7	chr11	-	614173	614399	613784	613865	614475	614534	2	3	7	2	2	0	0	0	8	3	98	49	8.93E-07	0.00129026	0.333	0.429	1	0	0	0.587
A5SS	2439	LILRA6	LILRA6	chr19	-	54241575	54241878	54241661	54241878	54240830	54241127	33	12	2	6	1	4	0	0	2	1	98	49	1.04E-06	0.000361711	0.733	0.857	0.2	0	0	0.597
SE	8882	CPEB2	CPEB2	chr4	+	15059186	15059301	15058420	15058539	15062078	15062260	12	15	3	1	0	0	1	0	1	2	98	49	1.24E-06	0.001436638	0.857	1	1	0.333	0	0.786
SE	12726	ADGRE5	ADGRE5	chr19	+	14397076	14397223	14396341	14396473	14397657	14397804	34	9	2	10	6	4	2	8	0	0	98	49	4.31E-06	0.004149582	0.63	0.429	0.2	1	1	-0.58
RI	535	TNFRSF10B	TNFRSF10B	chr8	-	23028330	23028602	23028330	23028441	23028528	23028602	22	4	2	4	7	1	1	2	0	0	98	49	4.88E-06	0.000804867	0.733	0.222	0.5	1	1	-0.515
SE	17082	FAM193B	FAM193B	chr5	-	177531281	177531521	177524184	177525205	177532442	177532641	21	5	2	10	4	0	3	7	0	0	98	49	1.30E-05	0.01058422	0.512	0.385	1	1	1	-0.368
SE	3910	AGTRAP	AGTRAP	chr1	+	11746126	11746223	11745802	11745837	11747439	11747545	5	4	2	5	2	0	0	0	1	2	98	49	1.47E-05	0.01058422	0.333	0.5	1	0	0	0.611
SE	5664	WDFY3	WDFY3	chr4	-	84932269	84932363	84896910	84897010	84966208	84966391	36	18	3	4	11	1	2	13	0	0	98	49	2.47E-05	0.015846384	0.818	0.45	0.6	1	1	-0.377
A3SS	1115	PHF20L1	PHF20L1	chr8	+	132777791	132777911	132777795	132777911	132775357	132775645	32	9	5	10	5	3	0	1	3	3	53	49	2.54E-05	0.014675126	0.747	0.625	0.606	0	0.236	0.541
RI	948	BCL2L11	BCL2L11	chr2	+	111123732	111124139	111123732	111123869	111124049	111124139	7	6	0	6	0	3	2	4	0	0	98	49	2.87E-05	0.002370232	0.368	1	0	1	1	-0.544
SE	8257	TNFSF13B	TNFSF13B	chr13	+	108286802	108286859	108270339	108270424	108303252	108303365	38	21	5	4	4	4	1	21	0	0	98	49	3.11E-05	0.017945737	0.826	0.724	0.385	1	1	-0.355
A3SS	2900	PGS1	PGS1	chr17	+	78414878	78415027	78414951	78415027	78403567	78404089	27	19	7	4	4	6	1	5	0	0	98	49	5.08E-05	0.014675126	0.771	0.704	0.368	1	1	-0.386
SE	7275	STK40	STK40	chr1	-	36367847	36368084	36361220	36361340	36385722	36385927	7	13	4	19	7	3	1	1	0	0	98	49	5.15E-05	0.027036581	0.156	0.481	0.4	1	1	-0.654
SE	5316	GATAD2A	GATAD2A	chr19	+	19492305	19492438	19465339	19465614	19492580	19492712	19	12	3	4	5	0	7	4	0	0	98	49	5.83E-05	0.028081837	0.704	0.545	1	1	1	-0.25
SE	1880	EEF1D	EEF1D	chr8	-	143586218	143586290	143581228	143581328	143586728	143586849	65	17	14	16	9	0	3	12	0	0	98	49	6.77E-05	0.029618192	0.67	0.486	1	1	1	-0.281
SE	8102	DEDD2	DEDD2	chr19	-	42216679	42217045	42215132	42215252	42217631	42217792	20	8	7	6	4	0	2	1	0	0	98	49	7.18E-05	0.029618192	0.625	0.5	1	1	1	-0.292
SE	4618	KIF13A	KIF13A	chr6	-	17789871	17789910	17787775	17787875	17794248	17794395	10	6	4	1	1	2	0	1	2	1	88	49	9.19E-05	0.035411696	0.848	0.77	0.527	0	0.358	0.536
SE	10413	KLF6	KLF6	chr10	-	3780105	3780229	3775995	3779590	3781640	3782214	40	21	8	7	7	0	3	14	0	0	98	49	9.87E-05	0.035411696	0.741	0.6	1	1	1	-0.22
RI	167	WTAP	WTAP	chr6	+	159748190	159749421	159748190	159748369	159748589	159749421	39	9	9	12	2	0	1	13	0	0	98	49	0.000107296	0.005901273	0.619	0.692	1	1	1	-0.23
SE	14148	PSD4	PSD4	chr2	+	113198739	113198884	113197746	113197913	113199082	113199226	31	16	11	8	3	1	5	2	0	0	98	49	0.000108337	0.035411696	0.66	0.727	0.846	1	1	-0.256
SE	9645	MCTP1	MCTP1	chr5	-	94917895	94917973	94912805	94912976	94923961	94924021	17	27	15	13	3	1	2	3	0	0	98	49	0.000110298	0.035411696	0.395	0.818	0.882	1	1	-0.302
SE	3227	BANP	BANP	chr16	+	88004294	88004411	87984059	87984259	88006089	88006265	19	13	3	3	3	1	1	1	0	0	98	49	0.000123531	0.037572954	0.76	0.684	0.6	1	1	-0.319
A5SS	1191	PTPN22	PTPN22	chr1	-	113834308	113834439	113834392	113834439	113833110	113833138	16	24	53	8	1	10	1	10	0	0	98	49	0.000126122	0.020806987	0.5	0.923	0.726	1	1	-0.284
A3SS	3574	RALGAPB	RALGAPB	chr20	+	38535073	38535207	38535085	38535207	38532729	38532859	5	2	3	3	4	0	1	1	0	0	61	49	0.000130734	0.025188065	0.572	0.287	1	1	1	-0.38
SE	14188	ACOX1	ACOX1	chr17	-	75960214	75960375	75957458	75957566	75978533	75978693	35	8	22	9	4	0	1	11	0	0	98	49	0.000149043	0.043065865	0.66	0.5	1	1	1	-0.28
SE	24	G6PD	G6PD	chrX	-	154532941	154533128	154532566	154532802	154533575	154533669	22	14	5	4	4	0	1	4	0	0	98	49	0.000172952	0.046713957	0.733	0.636	1	1	1	-0.21
A5SS	237	LAT2	LAT2	chr7	+	74214821	74215010	74214821	74214940	74215946	74216069	39	16	7	4	4	2	1	5	0	0	98	49	0.000179371	0.020806987	0.83	0.667	0.636	1	1	-0.289
SE	5497	SYNE1	SYNE1	chr6	-	152300640	152300781	152293959	152294127	152301868	152302063	6	3	0	0	1	1	0	1	1	1	98	49	0.000184481	0.046713957	1	0.6	0	0	0.333	0.367
SE	3520	MTMR14	MTMR14	chr3	+	9689963	9690143	9688943	9689082	9701789	9702394	13	12	6	5	2	0	2	9	0	0	98	49	0.000189631	0.046713957	0.565	0.75	1	1	1	-0.228
SE	1735	VNN3	VNN3	chr6	-	132728740	132728979	132724605	132724979	132734252	132734383	44	14	6	7	6	0	1	2	0	0	98	49	0.000197256	0.046713957	0.759	0.538	1	1	1	-0.234
A3SS	3415	MAP4K4	MAP4K4	chr2	+	101866427	101866579	101866430	101866579	101864929	101865036	22	11	2	7	4	0	1	6	0	0	52	49	0.000199827	0.02887498	0.748	0.722	1	1	1	-0.177
SE	1533	WIPI2	WIPI2	chr7	+	5193117	5193171	5190203	5190493	5199575	5199658	3	1	0	10	7	2	1	3	0	1	98	49	0.000202085	0.046713957	0.13	0.067	0	1	0.6	-0.734
SE	6524	LYST	LYST	chr1	-	235728075	235728131	235724027	235724180	235729595	235729657	104	34	16	10	6	0	1	35	0	0	98	49	0.000210324	0.046748649	0.839	0.739	1	1	1	-0.141
SE	3723	ADGRG3	ADGRG3	chr16	+	57676199	57676338	57673320	57673468	57678169	57678316	109	54	15	13	11	0	2	14	0	0	98	49	0.000247814	0.051249962	0.807	0.711	1	1	1	-0.161
SE	12145	PGS1	PGS1	chr17	+	78414878	78415027	78403567	78404089	78419545	78419675	11	15	8	4	3	0	1	4	0	0	98	49	0.000253016	0.051249962	0.579	0.714	1	1	1	-0.236
SE	1733	VNN3	VNN3	chr6	-	132728740	132728933	132724605	132724979	132734252	132734383	51	21	4	7	6	0	1	5	0	0	98	49	0.000257181	0.051249962	0.785	0.636	1	1	1	-0.193
SE	12146	PGS1	PGS1	chr17	+	78414951	78415027	78403567	78404089	78419545	78419675	10	13	10	4	3	0	1	1	0	0	98	49	0.000268464	0.051715024	0.556	0.684	1	1	1	-0.253
SE	3786	RGS14	RGS14	chr5	+	177371474	177371589	177371349	177371396	177371872	177372598	22	11	8	4	3	0	1	10	0	0	98	49	0.000290269	0.052774163	0.733	0.647	1	1	1	-0.207
SE	8838	NR1H2	NR1H2	chr19	+	50378148	50378439	50377732	50377870	50378521	50378796	14	7	10	2	7	0	3	3	0	0	98	49	0.000293892	0.052774163	0.778	0.333	1	1	1	-0.296
SE	8860	GUK1	GUK1	chr1	+	228148370	228148456	228147614	228147699	228148664	228148954	32	21	5	0	1	0	0	8	1	3	98	49	0.000307457	0.052774163	1	0.913	1	0	0.571	0.686
RI	488	STX10	STX10	chr19	-	13144057	13144671	13144057	13144486	13144576	13144671	15	16	0	9	10	13	1	6	0	1	98	49	0.000313634	0.0129374	0.455	0.444	0	1	0.75	-0.575
SE	7426	CD300A	CD300A	chr17	+	74473535	74473874	74466382	74466743	74474531	74474685	41	19	9	2	3	0	0	1	1	1	98	49	0.000315774	0.052774163	0.911	0.76	1	0	0.333	0.724
SE	9633	ARIH1	ARIH1	chr15	+	72544819	72544964	72518066	72518134	72555270	72555363	17	7	5	2	7	0	1	2	0	0	98	49	0.000319622	0.052774163	0.81	0.333	1	1	1	-0.286
SE	6469	C1orf43	C1orf43	chr1	-	154219835	154219937	154214456	154214574	154220341	154220797	3	4	3	2	1	0	0	0	1	1	98	49	0.000389915	0.062553102	0.429	0.667	1	0	0.333	0.532
SE	12875	ZFAS1	ZFAS1	chr20	+	49280902	49280964	49280484	49280570	49289044	49289258	45	37	17	12	14	13	1	12	0	1	98	49	0.000400496	0.062553102	0.652	0.569	0.395	1	0.857	-0.39
SE	16934	IRF1	IRF1	chr5	-	132486250	132486373	132484361	132484497	132486556	132486686	65	18	29	3	6	3	2	12	0	0	98	49	0.000423508	0.064406706	0.915	0.6	0.829	1	1	-0.219
A5SS	2943	MBD4	MBD4	chr3	-	129432502	129432606	129432555	129432606	129430943	129431578	33	10	3	6	2	0	0	1	1	1	98	49	0.0004824	0.041700018	0.733	0.714	1	0	0.333	0.649
A3SS	3084	GMIP	GMIP	chr19	-	19636706	19636796	19636706	19636787	19636916	19637029	27	15	8	6	4	0	1	9	0	0	58	49	0.000488408	0.042850564	0.792	0.76	1	1	1	-0.149
A3SS	3087	CDV3	CDV3	chr3	+	133587158	133590274	133587895	133590274	133586562	133586722	17	15	11	11	1	0	1	2	0	0	98	49	0.000557132	0.042850564	0.436	0.882	1	1	1	-0.227
SE	2174	BAG6	BAG6	chr6	-	31647590	31647826	31646393	31646523	31648676	31648751	21	7	6	2	5	0	1	2	0	0	98	49	0.000561069	0.07959693	0.84	0.412	1	1	1	-0.249
SE	12189	HGS	HGS	chr17	+	81690181	81690234	81688703	81688827	81690673	81690742	32	18	10	2	9	0	1	1	0	0	98	49	0.000573304	0.07959693	0.889	0.5	1	1	1	-0.204
SE	16397	FAM13A-AS1	FAM13A-AS1	chr4	+	88722150	88722344	88711854	88712017	88723208	88723315	19	6	1	3	3	0	1	1	0	0	98	49	0.000577518	0.07959693	0.76	0.5	1	1	1	-0.247
SE	16816	AGTPBP1	AGTPBP1	chr9	-	85660935	85660973	85657434	85657643	85669484	85669578	44	26	26	8	5	0	1	9	0	0	87	49	0.000578486	0.07959693	0.756	0.745	1	1	1	-0.166
A3SS	4077	CNBP	CNBP	chr3	-	129171078	129171283	129171078	129171277	129171445	129171538	6	2	5	22	19	10	0	0	1	4	55	49	0.000580571	0.042850564	0.195	0.086	0.308	0	0	0.196
A3SS	971	TPT1	TPT1	chr13	-	45333470	45337553	45333470	45337388	45338659	45338776	38	18	5	326	83	97	0	2	3	72	98	49	0.000593087	0.042850564	0.055	0.098	0.025	0	0.014	0.052
A5SS	1426	TBXAS1	TBXAS1	chr7	+	139875584	139875666	139875584	139875637	139911224	139911321	20	11	2	53	44	9	0	1	1	15	78	49	0.000599138	0.041700018	0.192	0.136	0.122	0	0.04	0.13
SE	10242	LST1	LST1	chr6	+	31587640	31587733	31587199	31587318	31588562	31588909	49	29	24	35	11	14	0	2	1	6	98	49	0.000674599	0.090662994	0.412	0.569	0.462	0	0.143	0.409
RI	408	CPT1B	CPT1B	chr22	-	50568860	50569421	50568860	50569081	50569335	50569421	3	4	0	3	1	1	0	0	2	1	98	49	0.000688021	0.01974555	0.333	0.667	0	0	0	0.333
RI	529	APH1A	APH1A	chr1	-	150265398	150266656	150265398	150266194	150266532	150266656	29	4	2	3	2	1	2	7	0	0	98	49	0.00071802	0.01974555	0.829	0.5	0.5	1	1	-0.39
SE	9952	SEC14L1	SEC14L1	chr17	+	77196201	77196311	77194676	77194911	77200483	77200673	180	90	52	9	7	2	11	34	0	0	98	49	0.000738806	0.095494005	0.909	0.865	0.929	1	1	-0.099
SE	10018	SBF2	SBF2	chr11	-	9998265	9998379	9993920	9993998	10000913	10001022	44	20	1	4	3	0	1	3	0	0	98	49	0.000771438	0.095494005	0.846	0.769	1	1	1	-0.128
SE	6902	CASP1	CASP1	chr11	-	105029123	105029267	105026841	105026951	105029664	105029899	48	13	23	6	3	0	1	17	0	0	98	49	0.000776107	0.095494005	0.8	0.684	1	1	1	-0.172
SE	4285	PLXNC1	PLXNC1	chr12	+	94298631	94298795	94297315	94297423	94300909	94301057	56	42	23	8	3	0	4	20	0	0	98	49	0.000802256	0.095494005	0.778	0.875	1	1	1	-0.116
SE	5058	ARAP1	ARAP1	chr11	-	72692752	72692785	72688454	72688537	72693324	72693470	13	7	12	26	12	17	0	2	1	16	82	49	0.000807769	0.095494005	0.23	0.258	0.297	0	0.07	0.227
SE	5663	WDFY3	WDFY3	chr4	-	84836928	84837090	84831412	84831605	84841153	84841263	68	11	17	8	2	0	1	20	0	0	98	49	0.000809691	0.095494005	0.81	0.733	1	1	1	-0.152
A3SS	3089	CDV3	CDV3	chr3	+	133587161	133590274	133587895	133590274	133586562	133586722	10	15	10	11	1	0	1	2	0	0	98	49	0.000820412	0.052688694	0.312	0.882	1	1	1	-0.269
SE	494	ABCA1	ABCA1	chr9	-	104825682	104825887	104824464	104824578	104826947	104827169	31	8	7	9	0	0	4	6	0	0	98	49	0.001014686	0.117277454	0.633	1	1	1	1	-0.122
SE	2588	CDC42SE1	CDC42SE1	chr1	-	151056650	151056786	151055676	151055993	151059478	151059649	15	1	5	46	16	7	0	0	1	4	98	49	0.001070413	0.12129248	0.14	0.03	0.263	0	0	0.144
SE	1285	SAMD9L	SAMD9L	chr7	-	93147087	93147146	93145384	93146040	93148193	93148388	6	7	3	7	1	3	1	7	0	1	98	49	0.001129194	0.125492512	0.3	0.778	0.333	1	0.778	-0.419
SE	7935	SUPT6H	SUPT6H	chr17	+	28682941	28683092	28682726	28682856	28683267	28683422	4	6	1	0	0	0	0	1	2	0	98	49	0.001189757	0.127567419	1	1	1	0	1	0.5
SE	6307	CD82	CD82	chr11	+	44605354	44605429	44605057	44605182	44615271	44615373	44	22	10	0	0	0	0	1	2	0	98	49	0.00119201	0.127567419	1	1	1	0	1	0.5
SE	1284	SAMD9L	SAMD9L	chr7	-	93146882	93147146	93145384	93146040	93148193	93148388	7	3	6	7	1	3	2	5	0	1	98	49	0.001254048	0.131766276	0.333	0.6	0.5	1	0.714	-0.379
SE	14713	PLEKHG3	PLEKHG3	chr14	+	64738741	64738855	64737355	64737375	64741035	64742455	48	3	10	5	3	0	2	14	0	0	98	49	0.001325462	0.136355346	0.828	0.333	1	1	1	-0.28
SE	15669	UBA7	UBA7	chr3	-	49812647	49812738	49812407	49812543	49813061	49813168	14	7	0	0	3	2	1	2	0	0	98	49	0.001349513	0.136355346	1	0.538	0	1	1	-0.487
SE	16195	UBE2R2	UBE2R2	chr9	+	33911963	33912098	33900173	33900271	33917017	33920403	21	10	8	1	0	0	0	6	2	1	98	49	0.001368508	0.136355346	0.913	1	1	0	0.75	0.596
SE	15849	ARHGAP25	ARHGAP25	chr2	+	68813286	68813419	68807272	68807480	68816288	68816362	77	36	39	8	4	0	1	15	0	0	98	49	0.001401076	0.137234229	0.828	0.818	1	1	1	-0.118
SE	8647	SAMSN1	SAMSN1	chr21	-	14516891	14517041	14512443	14512573	14521149	14521221	26	9	10	0	1	0	1	12	1	3	98	49	0.001473673	0.141939264	1	0.818	1	0.333	0.667	0.439
A5SS	2947	CSF2RA	CSF2RA	chrX	+	1274754	1274842	1274754	1274818	1282677	1282779	43	28	3	6	3	0	1	3	0	0	73	49	0.001509767	0.07556475	0.828	0.862	1	1	1	-0.103
SE	12923	TYROBP	TYROBP	chr19	-	35907729	35907762	35907445	35907580	35908174	35908309	34	6	4	1	1	0	3	6	3	1	82	49	0.001510354	0.14308748	0.953	0.782	1	0.374	0.782	0.334
SE	6156	LIMK2	LIMK2	chr22	+	31225719	31225819	31212263	31212424	31258290	31258426	118	52	19	7	4	0	14	29	0	0	98	49	0.001567896	0.146143067	0.894	0.867	1	1	1	-0.08
SE	14889	PREX1	PREX1	chr20	-	48659918	48660061	48658135	48658228	48666282	48666355	169	90	61	4	14	0	1	31	0	0	98	49	0.001683429	0.154421214	0.955	0.763	1	1	1	-0.094
A5SS	1107	SERPINA1	SERPINA1	chr14	-	94388800	94389010	94388818	94389010	94388559	94388660	38	15	12	18	3	11	1	6	0	1	67	49	0.001715222	0.07556475	0.607	0.785	0.444	1	0.814	-0.295
SE	9038	DENND5A	DENND5A	chr11	-	9170626	9170777	9169855	9169949	9178131	9178366	72	31	10	4	0	5	2	10	0	0	98	49	0.001799469	0.162486423	0.9	1	0.5	1	1	-0.2
SE	13231	RBM23	RBM23	chr14	-	22905605	22905659	22905335	22905453	22906194	22906368	38	23	7	1	3	3	0	2	2	1	98	49	0.001845941	0.163565	0.95	0.793	0.538	0	0.5	0.51
SE	9349	RAB18	RAB18	chr10	+	27531528	27531615	27526827	27526889	27532506	27532579	8	4	0	20	15	7	0	0	1	4	98	49	0.001867923	0.163565	0.167	0.118	0	0	0	0.095
A5SS	2038	ABTB1	ABTB1	chr3	+	127674390	127674453	127674390	127674418	127674544	127674600	57	37	13	15	18	7	2	6	0	1	84	49	0.001873218	0.07556475	0.689	0.545	0.52	1	0.778	-0.304
SE	1093	MGAM	MGAM	chr7	+	142027114	142027227	142025049	142025149	142027609	142027735	159	45	13	12	1	0	1	27	0	0	98	49	0.00193449	0.166856958	0.869	0.957	1	1	1	-0.058
A3SS	3963	CNN2	CNN2	chr19	+	1036066	1036246	1036129	1036246	1032558	1032696	13	10	0	83	43	21	2	9	1	10	98	49	0.001940249	0.109742062	0.073	0.104	0	0.5	0.31	-0.346
A5SS	2352	TYROBP	TYROBP	chr19	-	35908167	35908309	35908174	35908309	35907445	35907580	4	4	1	1	1	0	0	5	3	1	56	49	0.001954261	0.07556475	0.778	0.778	1	0	0.814	0.445
SE	11375	LRRC75A-AS1	LRRC75A-AS1	chr17	+	16441073	16441130	16440110	16440253	16441367	16442026	23	8	9	5	1	0	1	8	0	0	98	49	0.001999476	0.169926028	0.697	0.8	1	1	1	-0.168
A3SS	168	TMEM71	TMEM71	chr8	-	132746941	132747114	132746941	132747057	132751784	132751997	107	66	50	4	3	5	5	22	0	0	98	49	0.002088517	0.109742062	0.93	0.917	0.833	1	1	-0.107
SE	2482	ARRB2	ARRB2	chr17	+	4716146	4716191	4715972	4716033	4716411	4716608	147	66	40	20	6	0	6	32	0	1	94	49	0.002174624	0.182132677	0.793	0.852	1	1	0.943	-0.09
SE	9910	PTP4A2	PTP4A2	chr1	-	31915894	31915987	31911695	31911826	31918969	31919658	21	15	12	4	1	1	4	13	0	0	98	49	0.002314755	0.189353778	0.724	0.882	0.857	1	1	-0.179
SE	2943	FCAR	FCAR	chr19	+	54888006	54888294	54885234	54885525	54889648	54891420	58	10	7	4	9	0	5	28	0	1	98	49	0.002326374	0.189353778	0.879	0.357	1	1	0.933	-0.221
A3SS	700	PCBP2	PCBP2	chr12	+	53462480	53462567	53462492	53462567	53461014	53461143	65	31	9	51	6	3	1	8	1	19	61	49	0.00238858	0.11504996	0.506	0.806	0.707	0.445	0.253	0.324
A5SS	760	FAM160A2	FAM160A2	chr11	-	6218557	6218763	6218599	6218763	6217370	6218150	1	0	1	0	1	0	0	1	2	0	91	49	0.002461806	0.085670833	1	0	1	0	1	0.167
SE	2034	TAP1	TAP1	chr6	-	32848989	32849118	32848651	32848840	32850319	32850517	61	38	37	6	2	0	2	34	0	0	98	49	0.002588194	0.207738482	0.836	0.905	1	1	1	-0.086
SE	9654	FAU	FAU	chr11	-	65120980	65121036	65120626	65120806	65121499	65121644	78	21	15	2	6	0	1	13	0	0	98	49	0.002676583	0.210269197	0.951	0.636	1	1	1	-0.138
SE	11777	FMNL1	FMNL1	chr17	+	45245631	45245733	45245252	45245416	45245877	45245973	108	41	29	8	3	1	5	21	3	6	98	49	0.002713341	0.210269197	0.871	0.872	0.935	0.455	0.636	0.347
SE	14886	PREX1	PREX1	chr20	-	48644408	48644497	48642406	48642489	48645850	48646057	122	56	51	0	3	0	5	22	0	5	98	49	0.002756217	0.210269197	1	0.903	1	1	0.687	0.124
SE	9504	HSH2D	HSH2D	chr19	+	16151446	16151578	16148723	16148875	16152551	16153208	12	4	0	44	18	3	0	1	4	15	98	49	0.002765264	0.210269197	0.12	0.1	0	0	0.032	0.057
SE	2825	MEFV	MEFV	chr16	-	3249430	3249780	3247015	3247246	3254157	3254790	46	22	18	1	0	0	0	11	4	0	98	49	0.002963845	0.22054525	0.958	1	1	0	1	0.486
SE	2763	PCBP2	PCBP2	chr12	+	53464759	53464852	53462480	53462567	53467220	53467293	54	17	19	0	1	0	0	13	1	3	98	49	0.002976731	0.22054525	1	0.895	1	0	0.684	0.623
SE	13280	RBM5	RBM5	chr3	+	50092042	50092208	50090381	50090451	50093719	50093875	82	17	14	4	4	0	1	2	0	0	98	49	0.003118328	0.2281116	0.911	0.68	1	1	1	-0.136
SE	16135	IL1RAP	IL1RAP	chr3	+	190556129	190556216	190514050	190514219	190564288	190564353	22	20	1	5	1	0	3	7	0	1	98	49	0.003473051	0.250884516	0.687	0.909	1	1	0.778	-0.024
SE	10388	ZSWIM8	ZSWIM8	chr10	+	73788669	73788823	73785623	73786086	73789095	73789190	24	5	2	1	1	0	2	3	5	0	98	49	0.003538822	0.252479651	0.923	1	1	0.167	1	0.391
RI	1085	UNC119	UNC119	chr17	-	28546706	28547849	28546706	28547409	28547676	28547849	36	22	3	3	3	6	1	10	0	1	98	49	0.003670791	0.086525783	0.857	0.786	0.2	1	0.833	-0.302
SE	10573	NFKBIA	NFKBIA	chr14	-	35402770	35402859	35402393	35402663	35403149	35403360	124	307	51	1	15	0	10	63	0	0	98	49	0.003690903	0.257474498	0.984	0.911	1	1	1	-0.035
SE	7428	CD300A	CD300A	chr17	+	74481725	74481833	74481288	74481326	74484000	74484798	38	29	35	5	6	3	4	15	0	1	98	49	0.003715696	0.257474498	0.792	0.707	0.854	1	0.882	-0.157
SE	6933	IL17RA	IL17RA	chr22	+	17105852	17105954	17105590	17105602	17107726	17107768	61	17	15	7	0	0	10	15	0	0	98	49	0.003761285	0.257474498	0.813	1	1	1	1	-0.062
SE	10301	NLRP6	NLRP6	chr11	+	280083	281839	279833	279872	282704	282797	10	11	4	1	1	3	1	2	0	0	98	49	0.003860898	0.257474498	0.833	0.846	0.4	1	1	-0.307
SE	1138	LINC-PINT	LINC-PINT	chr7	-	131002196	131002320	130984052	130984109	131052424	131052555	7	5	0	47	20	3	0	0	2	9	98	49	0.003875277	0.257474498	0.069	0.111	0	0	0	0.06
SE	10775	PTK2B	PTK2B	chr8	+	27445793	27445919	27444205	27444271	27450748	27450895	8	1	0	22	5	8	0	0	1	2	98	49	0.003876152	0.257474498	0.154	0.091	0	0	0	0.082
SE	10245	LST1	LST1	chr6	+	31587943	31587966	31587199	31587318	31588562	31588909	12	10	1	35	11	14	0	1	1	6	72	49	0.003941519	0.258841315	0.189	0.382	0.046	0	0.102	0.155
A3SS	805	BANP	BANP	chr16	+	88035322	88035394	88035331	88035394	88033108	88033245	11	8	7	8	0	0	1	2	0	0	58	49	0.004108642	0.171485553	0.537	1	1	1	1	-0.154
A3SS	2515	ZSWIM8	ZSWIM8	chr10	+	73794540	73794639	73794555	73794639	73794146	73794330	19	5	0	9	3	1	1	7	1	0	64	49	0.004153629	0.171485553	0.618	0.561	0	0.434	1	-0.324
SE	15293	CSF2RB	CSF2RB	chr22	+	36936548	36936652	36935629	36935687	36937376	36940437	151	73	46	7	4	0	7	34	0	0	98	49	0.004519392	0.293455815	0.915	0.901	1	1	1	-0.061
SE	1135	LINC-PINT	LINC-PINT	chr7	-	130959868	130959970	130944159	130945835	130984052	130984109	21	1	0	14	1	2	0	1	1	2	98	49	0.004626486	0.297071834	0.429	0.333	0	0	0.2	0.154
SE	2941	FCAR	FCAR	chr19	+	54888006	54888220	54885234	54885525	54889648	54891420	30	4	5	4	9	0	1	11	0	1	98	49	0.004730423	0.300407848	0.789	0.182	1	1	0.846	-0.266
A3SS	1029	OAS1	OAS1	chr12	+	112919388	112919907	112919486	112919907	112917546	112917700	1	1	9	0	0	0	3	5	0	4	98	49	0.004828658	0.186064281	1	1	1	1	0.385	0.307
SE	6536	RBM39	RBM39	chr20	-	35740524	35740597	35738967	35739017	35740823	35740887	63	33	36	23	8	1	2	8	0	1	98	49	0.004971951	0.312314156	0.578	0.673	0.947	1	0.8	-0.167
A3SS	4024	RICTOR	RICTOR	chr5	-	38945490	38945724	38945490	38945610	38946467	38946552	100	32	12	12	8	0	1	15	0	1	98	49	0.005321448	0.192237298	0.806	0.667	1	1	0.882	-0.117
SE	207	PIM2	PIM2	chrX	-	48914394	48914571	48913181	48914294	48915019	48915392	35	12	17	7	6	1	0	2	1	2	98	49	0.005370609	0.33372849	0.714	0.5	0.895	0	0.333	0.536
SE	7149	ATP1A1	ATP1A1	chr1	+	116395109	116395285	116393530	116393723	116396597	116396734	17	4	8	6	0	0	1	11	0	0	98	49	0.005599832	0.344270522	0.586	1	1	1	1	-0.138
SE	247	DDX3X	DDX3X	chrX	+	41347314	41347451	41346858	41347012	41347639	41350287	147	37	36	7	3	0	1	17	0	0	98	49	0.005759724	0.347607175	0.913	0.86	1	1	1	-0.076
SE	3044	STX16	STX16	chr20	+	58671153	58671297	58670511	58670603	58673630	58673711	37	27	7	4	4	0	1	16	0	1	98	49	0.005774405	0.347607175	0.822	0.659	1	1	0.889	-0.118
SE	8799	BROX	BROX	chr1	+	222731356	222731516	222730026	222730177	222732627	222735196	39	8	5	0	0	1	2	6	0	4	98	49	0.005905541	0.351836284	1	1	0.714	1	0.429	0.19
SE	11526	CHFR	CHFR	chr12	-	132870723	132870783	132861466	132861634	132872284	132872394	19	3	6	0	4	1	3	5	0	0	98	49	0.006223406	0.366990437	1	0.273	0.75	1	1	-0.326
SE	9240	SVIL	SVIL	chr10	-	29526960	29527056	29524471	29524715	29529704	29529844	8	0	1	33	14	2	0	0	3	8	98	49	0.00637626	0.372206119	0.108	0	0.2	0	0	0.103
SE	9931	MKNK1	MKNK1	chr1	-	46560233	46560277	46557406	46558800	46561477	46561642	16	9	2	5	2	0	0	2	1	2	93	49	0.006502959	0.375806004	0.628	0.703	1	0	0.345	0.605
SE	15577	SUN2	SUN2	chr22	-	38750897	38751035	38750224	38750320	38751209	38751373	43	11	9	5	1	0	1	6	0	0	98	49	0.00671086	0.383255085	0.811	0.846	1	1	1	-0.114
SE	4283	PLXNC1	PLXNC1	chr12	+	94209589	94209704	94186372	94186473	94220015	94220163	77	36	26	7	5	0	4	27	0	1	98	49	0.006764495	0.383255085	0.846	0.783	1	1	0.931	-0.089
SE	6938	ARID4B	ARID4B	chr1	-	235213768	235214026	235196030	235196115	235219792	235219968	11	1	1	9	3	5	0	1	1	2	98	49	0.006951843	0.387975679	0.379	0.143	0.091	0	0.2	0.104
SE	236	DDX3X	DDX3X	chrX	+	41339035	41339083	41337407	41337465	41341483	41341616	120	66	28	5	4	0	2	15	0	0	97	49	0.006990144	0.387975679	0.924	0.893	1	1	1	-0.061
SE	2824	MEFV	MEFV	chr16	-	3248908	3249004	3247015	3247246	3254157	3254790	48	18	35	1	0	0	2	18	4	0	98	49	0.007049221	0.387975679	0.96	1	1	0.2	1	0.387
SE	7813	PGD	PGD	chr1	+	10403070	10403136	10400392	10400572	10404160	10404279	93	46	31	1	0	0	7	13	4	0	98	49	0.007216115	0.39135749	0.979	1	1	0.467	1	0.259
SE	8990	GLYR1	GLYR1	chr16	-	4821546	4821597	4821379	4821453	4822874	4822931	21	4	15	0	0	0	0	9	3	0	98	49	0.007246107	0.39135749	1	1	1	0	1	0.5
SE	13299	TSEN34	TSEN34	chr19	+	54191720	54191964	54191213	54191607	54192115	54192373	62	31	46	3	7	3	5	17	0	1	98	49	0.007593268	0.406310123	0.912	0.689	0.885	1	0.895	-0.119
SE	12576	VAV1	VAV1	chr19	+	6847997	6848114	6843134	6843166	6850669	6850757	60	21	19	0	0	0	3	13	4	0	98	49	0.007817874	0.414490774	1	1	1	0.273	1	0.363
SE	245	DDX3X	DDX3X	chrX	+	41347311	41347451	41346858	41347012	41347639	41347915	173	52	46	7	3	0	1	19	0	0	98	49	0.008108954	0.426014944	0.925	0.897	1	1	1	-0.059
SE	3570	DIP2B	DIP2B	chr12	+	50692948	50693013	50691048	50691151	50695266	50695360	18	22	2	6	0	0	2	5	0	0	98	49	0.008244832	0.42925121	0.6	1	1	1	1	-0.133
SE	9036	DENND5A	DENND5A	chr11	-	9147029	9147151	9145669	9145815	9150080	9150209	66	31	24	7	0	0	3	13	0	0	98	49	0.008385906	0.432697767	0.825	1	1	1	1	-0.058
SE	10695	NPL	NPL	chr1	+	182822114	182822199	182818812	182818859	182825780	182825820	43	14	16	4	1	1	5	5	0	0	98	49	0.008492504	0.434320174	0.843	0.875	0.889	1	1	-0.131
SE	9897	ICAM3	ICAM3	chr19	-	10335065	10335353	10334527	10334782	10335670	10335976	209	102	117	19	7	4	1	23	0	1	98	49	0.008673586	0.439689953	0.846	0.879	0.936	1	0.92	-0.073
A5SS	523	BTNL8	BTNL8	chr5	+	180947511	180947755	180947511	180947625	180948354	180948375	13	4	0	4	0	3	2	2	0	1	98	49	0.009042031	0.277083706	0.619	1	0	1	0.5	-0.21
SE	1187	IFRD1	IFRD1	chr7	+	112462269	112462378	112462000	112462179	112467980	112468115	78	16	20	80	56	22	0	10	3	37	98	49	0.009088918	0.456737895	0.328	0.125	0.312	0	0.119	0.196
SE	6534	RBM39	RBM39	chr20	-	35729461	35729527	35729311	35729365	35731940	35732135	186	68	19	4	0	5	3	27	0	0	98	49	0.009727315	0.484604766	0.959	1	0.655	1	1	-0.129
A5SS	1150	MYD88	MYD88	chr3	+	38140387	38140592	38140387	38140568	38140756	38140848	1	0	0	9	1	3	0	4	1	7	73	49	0.009750777	0.277083706	0.069	0	0	0	0.277	-0.116

RI	1014	OSCAR	OSCAR	chr19	-	54094667	54096153	54094667	54095357	54095871	54096153	5	1	1	0	2	3	2	1	0	1	98	49	0.009785372	0.201823302	1	0.2	0.143	1	0.333	-0.219
A3SS	3474	CFLAR	CFLAR	chr2	+	201162995	201172688	201163834	201172688	201160431	201160942	36	9	8	132	56	24	0	4	3	38	98	49	0.009823696	0.330007041	0.12	0.074	0.143	0	0.05	0.087
SE	10365	AKNA	AKNA	chr9	-	114343707	114343803	114342008	114342125	114345862	114346009	63	18	18	2	4	0	0	10	2	2	98	49	0.009893908	0.48869139	0.94	0.692	1	0	0.714	0.52
SE	14778	PLAUR	PLAUR	chr19	-	43655438	43655573	43652224	43652371	43656478	43656640	65	27	18	3	0	0	8	42	0	6	98	49	0.010169452	0.498044606	0.915	1	1	1	0.778	0.083
A3SS	1048	DEF8	DEF8	chr16	+	89964169	89964310	89964220	89964310	89963362	89963443	19	8	3	2	2	3	3	3	0	1	98	49	0.010277036	0.330007041	0.826	0.667	0.333	1	0.6	-0.191
A5SS	762	PLEKHB2	PLEKHB2	chr2	+	131132901	131133015	131132901	131132991	131140166	131140275	1	0	0	18	5	2	0	4	1	3	73	49	0.010350828	0.277083706	0.036	0	0	0	0.472	-0.224
SE	237	DDX3X	DDX3X	chrX	+	41343215	41343351	41342736	41342836	41343736	41343822	212	92	26	8	6	4	2	31	0	1	98	49	0.01082339	0.521780013	0.93	0.885	0.765	1	0.939	-0.11
SE	12808	ELL	ELL	chr19	-	18465796	18465918	18465411	18465575	18472834	18472882	69	32	3	2	0	0	2	13	4	0	98	49	0.010834678	0.521780013	0.945	1	1	0.2	1	0.382
SE	11623	PSMB3	PSMB3	chr17	+	38760507	38760608	38755882	38755990	38762410	38762505	18	13	30	2	5	2	1	7	0	1	98	49	0.011365999	0.532195482	0.818	0.565	0.882	1	0.778	-0.134
SE	6535	RBM39	RBM39	chr20	-	35734185	35734257	35731940	35732135	35738967	35739017	2	2	8	56	49	9	0	0	2	9	98	49	0.011421734	0.532195482	0.018	0.02	0.308	0	0	0.115
SE	10891	ADGRE2	ADGRE2	chr19	-	14755653	14755877	14752328	14752526	14756237	14756345	6	6	12	0	0	0	0	2	1	1	98	49	0.011489901	0.532195482	1	1	1	0	0.5	0.75
SE	7028	TAOK3	TAOK3	chr12	-	118212913	118212995	118201295	118201463	118214016	118214110	28	5	5	3	3	0	2	4	0	1	98	49	0.011563355	0.532195482	0.824	0.455	1	1	0.667	-0.074
SE	4824	FRYL	FRYL	chr4	-	48512480	48512688	48510834	48510984	48515027	48515275	5	10	4	0	0	0	0	2	2	0	98	49	0.011681017	0.532195482	1	1	1	0	1	0.5
SE	2177	PRRC2A	PRRC2A	chr6	+	31622726	31622901	31620672	31620858	31624260	31624360	16	3	2	0	0	0	0	2	2	0	98	49	0.011681454	0.532195482	1	1	1	0	1	0.5
SE	16224	CIRBP	CIRBP	chr19	+	1271139	1271246	1270866	1271036	1271328	1271467	66	44	22	5	2	0	2	4	0	0	98	49	0.011717498	0.532195482	0.868	0.917	1	1	1	-0.072
SE	9303	PIK3CD	PIK3CD	chr1	+	9723968	9724092	9723124	9723292	9724275	9724421	67	52	19	2	5	0	3	12	0	0	98	49	0.011787683	0.532195482	0.944	0.839	1	1	1	-0.072
SE	16189	SH3BP2	SH3BP2	chr4	+	2831922	2831978	2831570	2831679	2832330	2832412	28	28	9	6	2	0	1	12	0	1	98	49	0.012193479	0.540043183	0.7	0.875	1	1	0.857	-0.07
SE	4830	CLEC7A	CLEC7A	chr12	-	10123244	10123363	10116780	10118590	10125086	10125173	96	47	30	2	0	1	0	38	2	1	98	49	0.012205539	0.540043183	0.96	1	0.937	0	0.95	0.491
SE	10339	FIP1L1	FIP1L1	chr4	+	53414614	53414722	53399729	53399839	53425871	53425965	11	12	7	3	0	2	1	0	0	2	98	49	0.012241851	0.540043183	0.647	1	0.636	1	0	0.261
SE	15687	MAPKAPK3	MAPKAPK3	chr3	+	50647122	50647203	50646739	50646825	50647893	50649297	43	30	7	5	1	0	2	11	0	0	98	49	0.012450865	0.545102635	0.811	0.937	1	1	1	-0.084
SE	9203	ACSL1	ACSL1	chr4	-	184773080	184773154	184770398	184770476	184773662	184773714	318	169	121	11	3	0	2	76	0	0	98	49	0.012737605	0.550988099	0.935	0.966	1	1	1	-0.033
SE	14776	PLAUR	PLAUR	chr19	-	43652224	43652371	43648570	43649143	43656478	43656640	23	4	2	1	4	0	2	23	0	0	98	49	0.012877679	0.550988099	0.92	0.333	1	1	1	-0.249
SE	12252	SMCHD1	SMCHD1	chr18	+	2778168	2778239	2777805	2777915	2784449	2784621	191	107	39	9	3	3	2	47	0	1	98	49	0.012958315	0.550988099	0.914	0.947	0.867	1	0.959	-0.07
SE	13224	FPR1	FPR1	chr19	-	51750202	51750253	51745769	51747005	51751813	51751897	47	21	16	13	7	3	1	2	0	1	98	49	0.012966669	0.550988099	0.644	0.6	0.727	1	0.5	-0.093
SE	7858	LRRK2	LRRK2	chr12	+	40305784	40305966	40303947	40304134	40308466	40308696	300	164	57	13	1	0	2	32	0	0	98	49	0.013129747	0.553845305	0.92	0.988	1	1	1	-0.031
SE	2764	PCBP2	PCBP2	chr12	+	53465931	53465973	53462480	53462567	53467220	53467293	72	31	6	0	1	0	2	30	1	3	91	49	0.013550016	0.567431451	1	0.943	1	0.519	0.843	0.3
SE	2605	PXN	PXN	chr12	-	120215559	120215661	120215102	120215273	120221622	120221758	10	6	2	31	12	16	0	1	1	3	98	49	0.013824713	0.574769886	0.139	0.2	0.059	0	0.143	0.061
SE	6756	SETD1B	SETD1B	chr12	+	121819403	121819895	121817798	121817904	121822489	121823749	8	9	3	0	0	0	3	1	0	3	98	49	0.014021703	0.578795883	1	1	1	1	0.143	0.428
SE	9544	GMPR2	GMPR2	chr14	+	24238245	24238405	24237519	24237562	24238588	24239241	27	12	7	3	2	4	0	1	2	0	98	49	0.014211036	0.582450894	0.818	0.75	0.467	0	1	0.178
SE	7719	CCDC88B	CCDC88B	chr11	+	64354003	64354170	64353714	64353813	64355193	64355400	36	17	6	0	2	0	4	4	1	2	98	49	0.014784184	0.600287427	1	0.81	1	0.667	0.5	0.353
SE	8321	RNF10	RNF10	chr12	+	120571190	120571291	120566824	120566980	120575630	120575688	35	27	8	4	4	0	3	6	0	1	98	49	0.014853972	0.600287427	0.814	0.771	1	1	0.75	-0.013
SE	12967	DDX60L	DDX60L	chr4	-	168448637	168448779	168441336	168441492	168453123	168453282	92	48	7	10	0	0	2	53	0	1	98	49	0.015096044	0.605727518	0.821	1	1	1	0.964	-0.042
SE	11622	PSMB3	PSMB3	chr17	+	38760430	38760608	38755882	38755990	38762410	38762505	32	23	41	2	5	2	1	12	0	1	98	49	0.015198216	0.605727518	0.889	0.697	0.911	1	0.857	-0.096
SE	4214	ZBTB37	ZBTB37	chr1	+	173873466	173873566	173870196	173871148	173885635	173886636	15	3	2	5	0	0	1	6	0	0	98	49	0.015703593	0.621582645	0.6	1	1	1	1	-0.133
A3SS	2442	DNM2	DNM2	chr19	+	10830977	10831910	10830980	10831910	10830126	10830378	4	9	3	4	2	1	2	1	0	1	52	49	0.015869094	0.482754544	0.485	0.809	0.739	1	0.485	-0.065
RI	113	DDAH2	DDAH2	chr6	-	31728864	31730265	31728864	31729225	31730050	31730265	10	3	2	4	3	0	1	0	0	3	98	49	0.01604331	0.294127359	0.556	0.333	1	1	0	0.13
SE	11055	THEMIS2	THEMIS2	chr1	+	27881970	27883043	27876587	27876728	27885294	27885451	25	14	1	3	2	6	0	2	3	1	98	49	0.016045206	0.627878418	0.806	0.778	0.077	0	0.5	0.304
SE	4833	CLEC7A	CLEC7A	chr12	-	10125086	10125173	10123244	10123363	10125296	10125448	29	13	14	50	26	13	1	25	0	8	98	49	0.016142926	0.627878418	0.225	0.2	0.35	1	0.61	-0.547
SE	8018	RNF19B	RNF19B	chr1	-	32949568	32949774	32948221	32948363	32964050	32964813	56	29	8	1	1	0	0	7	2	0	98	49	0.016354916	0.627878418	0.966	0.935	1	0	1	0.467
SE	10036	PRKCD	PRKCD	chr3	+	53185926	53186027	53185603	53185700	53186166	53186340	23	8	11	0	2	0	3	4	0	4	98	49	0.016356585	0.627878418	1	0.667	1	1	0.333	0.222
SE	5176	SLC12A9	SLC12A9	chr7	+	100861137	100861262	100860149	100860232	100861391	100861584	62	24	16	1	2	3	4	10	0	0	98	49	0.01640589	0.627878418	0.969	0.857	0.727	1	1	-0.149
A5SS	1792	MLKL	MLKL	chr16	-	74675323	74675404	74675354	74675404	74674959	74675100	6	1	6	7	2	0	1	1	5	3	80	49	0.016544743	0.411255051	0.344	0.234	1	0.109	0.17	0.386
A3SS	2481	LST1	LST1	chr6	+	31588496	31588909	31588562	31588909	31587943	31587966	67	45	41	5	2	0	4	10	0	0	98	49	0.016723378	0.482990798	0.87	0.918	1	1	1	-0.071
SE	14578	LRRFIP1	LRRFIP1	chr2	+	237751199	237751271	237749198	237749324	237753308	237753479	13	10	0	26	29	2	0	2	1	13	98	49	0.016726002	0.635918186	0.2	0.147	0	0	0.071	0.08
SE	10008	NT5C2	NT5C2	chr10	-	103106588	103106706	103105705	103105801	103139405	103139479	62	22	12	3	3	0	1	7	0	0	98	49	0.017233157	0.650917757	0.912	0.786	1	1	1	-0.101
SE	5342	SF3B1	SF3B1	chr2	-	197392971	197393188	197391973	197392461	197396055	197396328	221	87	41	2	6	2	5	39	0	0	98	49	0.017359502	0.651432222	0.982	0.879	0.911	1	1	-0.076
A3SS	2976	MKNK2	MKNK2	chr19	-	2037470	2039856	2037470	2037829	2040133	2040177	127	48	42	2	2	4	6	15	0	0	98	49	0.017548109	0.482990798	0.969	0.923	0.84	1	1	-0.089
SE	15943	CSF2RA	CSF2RA	chrX	+	1303922	1304019	1300490	1300626	1305445	1305527	20	7	17	8	0	0	1	11	0	1	98	49	0.017694655	0.659725219	0.556	1	1	1	0.846	-0.071
SE	12824	GMIP	GMIP	chr19	-	19635643	19635721	19635414	19635569	19636706	19636796	60	37	3	6	2	0	9	8	1	0	98	49	0.017993748	0.666576081	0.833	0.902	1	0.818	1	0.003
SE	14605	HDLBP	HDLBP	chr2	-	241255373	241255580	241255050	241255158	241256183	241256399	29	5	1	3	2	0	1	1	0	0	98	49	0.018618823	0.685338728	0.829	0.556	1	1	1	-0.205
SE	615	TLN1	TLN1	chr9	-	35722860	35722921	35722118	35722223	35723951	35724079	103	87	32	4	4	0	3	26	0	0	98	49	0.019607496	0.717162791	0.928	0.916	1	1	1	-0.052
SE	17072	GRK6	GRK6	chr5	+	177431994	177432107	177430871	177430967	177432232	177432310	62	21	28	7	0	0	7	9	0	1	98	49	0.019813105	0.720125358	0.816	1	1	1	0.818	0.03
SE	9108	MAN2C1	MAN2C1	chr15	-	75363998	75364188	75362641	75362748	75364487	75364665	10	0	3	0	1	0	2	0	0	2	98	49	0.020428857	0.737174147	1	0	1	1	0	0.167
SE	5207	PTPRJ	PTPRJ	chr11	+	48121002	48121266	48112746	48112983	48123612	48123870	98	47	29	7	3	0	2	25	0	1	98	49	0.020572324	0.737174147	0.875	0.887	1	1	0.926	-0.042
SE	7351	FAM157A	FAM157A	chr3	+	198169784	198169872	198167675	198167871	198180754	198180857	63	20	3	5	2	9	4	48	0	3	98	49	0.020664858	0.737174147	0.863	0.833	0.143	1	0.889	-0.332
SE	16115	SIRPA	SIRPA	chr20	+	1921394	1921712	1915098	1915455	1924763	1924877	7	11	1	4	5	0	1	0	0	1	98	49	0.020848898	0.739176561	0.467	0.524	1	1	0	0.164
A3SS	1722	MED11	MED11	chr17	+	4732938	4733610	4733049	4733610	4731775	4731906	12	8	4	5	0	0	1	4	0	0	98	49	0.020946769	0.550328744	0.545	1	1	1	1	-0.152
SE	5052	ST3GAL4	ST3GAL4	chr11	+	126409267	126409411	126408306	126408496	126413504	126413648	12	9	4	0	0	0	0	3	2	0	98	49	0.021635118	0.762374053	1	1	1	0	1	0.5
SE	11444	USP22	USP22	chr17	-	21019083	21019185	21017941	21018111	21021112	21021226	20	8	3	0	0	6	1	14	0	0	98	49	0.022240574	0.777124869	1	1	0.2	1	1	-0.267
SE	4936	TNFRSF1A	TNFRSF1A	chr12	-	6334090	6334244	6333736	6333865	6341775	6342117	130	53	58	2	4	0	2	17	3	1	98	49	0.022373377	0.777124869	0.97	0.869	1	0.25	0.895	0.374
SE	16935	IRF1	IRF1	chr5	-	132486250	132486373	132485666	132485716	132486556	132486686	113	50	55	8	2	0	2	36	0	1	98	49	0.022457147	0.777124869	0.876	0.926	1	1	0.947	-0.039
SE	14480	BAZ2A	BAZ2A	chr12	-	56605829	56606063	56605072	56605327	56606246	56606312	37	4	10	2	3	0	2	2	0	0	98	49	0.022681196	0.779565992	0.902	0.4	1	1	1	-0.233
SE	11938	MAP7D1	MAP7D1	chr1	+	36174897	36175008	36173363	36173478	36176198	36176321	24	17	3	8	1	1	0	4	2	2	98	49	0.022797483	0.779565992	0.6	0.895	0.6	0	0.5	0.448
SE	6610	AKAP13	AKAP13	chr15	+	85658536	85658590	85655416	85655787	85664562	85664755	80	39	4	3	5	0	2	13	0	1	98	49	0.023450224	0.789226928	0.93	0.796	1	1	0.867	-0.025
SE	4907	GABARAPL1	GABARAPL1	chr12	+	10218062	10218141	10212889	10213219	10220439	10220558	45	31	26	2	0	0	0	8	2	0	98	49	0.023507475	0.789226928	0.918	1	1	0	1	0.473
SE	3726	ADGRG3	ADGRG3	chr16	+	57684389	57684483	57680504	57680617	57685642	57685926	42	10	20	2	0	0	0	7	2	0	98	49	0.023598824	0.789226928	0.913	1	1	0	1	0.471
SE	1534	WIPI2	WIPI2	chr7	+	5193117	5193345	5190203	5190493	5199575	5199658	3	0	0	10	7	2	1	1	0	1	98	49	0.023699285	0.789226928	0.13	0	0	1	0.333	-0.623
SE	15851	SYNE2	SYNE2	chr14	+	64029894	64030059	64027483	64027793	64031015	64031357	33	20	7	2	0	0	0	5	2	0	98	49	0.023762846	0.789226928	0.892	1	1	0	1	0.464
A3SS	4045	SDCBP	SDCBP	chr8	+	58575899	58576061	58575902	58576061	58572204	58572314	53	62	38	10	10	8	3	20	0	2	52	49	0.024257968	0.609613283	0.833	0.854	0.817	1	0.904	-0.117
SE	7851	LRRK2	LRRK2	chr12	+	40298242	40298493	40295426	40295644	40299108	40299257	407	104	51	7	5	0	3	61	0	0	98	49	0.024357869	0.804366435	0.967	0.912	1	1	1	-0.04
A5SS	2848	TLE3	TLE3	chr15	-	70058158	70058291	70058185	70058291	70057458	70057658	31	10	15	5	2	1	1	4	0	1	76	49	0.024599774	0.570714761	0.8	0.763	0.906	1	0.721	-0.037
SE	15838	AOAH	AOAH	chr7	-	36686698	36686794	36673942	36674009	36724021	36724549	64	16	5	7	0	0	5	37	0	1	98	49	0.026174187	0.856138307	0.821	1	1	1	0.949	-0.034
SE	7209	NBEAL2	NBEAL2	chr3	+	47007065	47007155	47006332	47006449	47007240	47007350	85	26	16	1	1	4	2	16	0	0	98	49	0.026221921	0.856138307	0.977	0.929	0.667	1	1	-0.142
A3SS	658	PXN	PXN	chr12	-	120215102	120215417	120215102	120215273	120215559	120215661	20	9	4	3	1	1	1	7	0	1	98	49	0.026774513	0.644819526	0.769	0.818	0.667	1	0.778	-0.138
SE	3034	XPO6	XPO6	chr16	-	28180940	28181031	28177219	28177332	28211365	28211918	338	139	60	2	9	0	3	55	0	0	98	49	0.027362825	0.888369463	0.988	0.885	1	1	1	-0.042
A5SS	1247	CASS4	CASS4	chr20	+	56437163	56437586	56437163	56437424	56445899	56446001	44	9	8	1	1	3	2	10	0	0	98	49	0.027870677	0.606187224	0.957	0.818	0.571	1	1	-0.218
SE	16895	VASP	VASP	chr19	+	45521321	45521406	45517928	45518094	45522167	45522217	181	71	83	5	2	0	2	37	2	3	98	49	0.027956523	0.902539602	0.948	0.947	1	0.333	0.86	0.368
SE	12194	SP110	SP110	chr2	-	230178156	230178255	230176472	230176724	230183571	230183640	34	16	10	0	1	4	1	8	0	0	98	49	0.028111633	0.902539602	1	0.889	0.556	1	1	-0.185
SE	10325	IRF7	IRF7	chr11	-	614173	614399	613950	614037	614475	614534	3	3	7	8	8	0	1	2	7	10	98	49	0.029017678	0.926481559	0.158	0.158	1	0.067	0.091	0.36
SE	11878	SPAG9	SPAG9	chr17	-	50974770	50974947	50970706	50970856	50977107	50977221	35	17	6	3	2	0	1	3	0	0	98	49	0.030137537	0.956949587	0.854	0.81	1	1	1	-0.112
SE	389	RAPGEF1	RAPGEF1	chr9	-	131650128	131650242	131643238	131643417	131650809	131650949	18	3	1	4	2	0	2	1	0	1	98	49	0.030356618	0.958638776	0.692	0.429	1	1	0.333	0.04
SE	8171	PECAM1	PECAM1	chr17	-	64354932	64355040	64353490	64353518	64356110	64356398	174	76	71	9	3	0	1	38	0	8	98	49	0.031092019	0.96633516	0.906	0.927	1	1	0.704	0.092
SE	3127	CMIP	CMIP	chr16	+	81664268	81664349	81660883	81660946	81670141	81670245	27	11	4	0	0	0	0	5	2	0	98	49	0.031099492	0.96633516	1	1	1	0	1	0.5
SE	11704	CASC3	CASC3	chr17	+	40167849	40167948	40167497	40167612	40168202	40168417	28	11	7	0	0	0	0	5	2	0	98	49	0.03110198	0.96633516	1	1	1	0	1	0.5
A3SS	3526	SNRPB	SNRPB	chr20	-	2461634	2461939	2461634	2461793	2462635	2462761	22	10	1	7	1	0	0	3	1	2	98	49	0.031364389	0.725144665	0.611	0.833	1	0	0.429	0.6
SE	2485	ARRB2	ARRB2	chr17	+	4720392	4720472	4720211	4720299	4720585	4720640	118	56	50	3	13	0	2	26	1	0	98	49	0.031561151	0.97535771	0.952	0.683	1	0.5	1	0.128
SE	4342	ITPK1	ITPK1	chr14	-	93076594	93076619	93016675	93016801	93115068	93115184	55	27	12	4	2	0	4	6	0	0	74	49	0.032101744	0.984878326	0.901	0.899	1	1	1	-0.067
A5SS	2353	TYROBP	TYROBP	chr19	-	35908167	35908309	35908174	35908309	35907729	35907762	71	31	15	8	2	0	1	10	0	1	56	49	0.032118554	0.657485692	0.886	0.931	1	1	0.897	-0.009
SE	10543	ATF7IP	ATF7IP	chr12	+	14457206	14457295	14456560	14456634	14460494	14461133	27	8	3	0	0	5	1	3	0	0	98	49	0.032327505	0.984878326	1	1	0.231	1	1	-0.256
SE	8341	CASP8	CASP8	chr2	+	201272897	201272942	201272637	201272776	201274888	201274953	163	69	16	5	2	1	1	6	0	0	94	49	0.032380495	0.984878326	0.944	0.947	0.893	1	1	-0.072
A3SS	2482	LST1	LST1	chr6	+	31588517	31588909	31588562	31588909	31587943	31587966	78	54	55	5	2	0	3	16	0	0	94	49	0.032814021	0.729480928	0.89	0.934	1	1	1	-0.059
SE	12922	TYROBP	TYROBP	chr19	-	35907729	35907762	35907445	35907580	35908167	35908309	95	33	18	2	3	0	4	15	0	5	82	49	0.035068864	1	0.966	0.868	1	1	0.642	0.124
SE	5209	PTPRJ	PTPRJ	chr11	+	48146875	48146963	48144999	48145124	48149446	48149488	36	5	11	0	5	0	6	5	0	0	98	49	0.035659347	1	1	0.333	1	1	1	-0.222
SE	5658	WDFY3	WDFY3	chr4	-	84741760	84741921	84740186	84740416	84743699	84743799	44	17	5	4	1	0	3	11	0	0	98	49	0.036395704	1	0.846	0.895	1	1	1	-0.086
A3SS	710	TMBIM4	TMBIM4	chr12	-	66153339	66153448	66153339	66153410	66169854	66170072	20	11	18	1	0	0	0	4	2	0	87	49	0.036512779	0.781643942	0.918	1	1	0	1	0.473
A5SS	1620	RBM10	RBM10	chrX	+	47145217	47145516	47145217	47145485	47147356	47147498	5	4	2	9	1	2	0	2	2	3	80	49	0.037869553	0.728051467	0.254	0.71	0.38	0	0.29	0.303
SE	8098	DEDD2	DEDD2	chr19	-	42201988	42202072	42198592	42199829	42209699	42209840	2	0	1	16	25	11	1	2	3	5	98	49	0.037915884	1	0.059	0	0.043	0.143	0.167	-0.121
SE	3160	CTSA	CTSA	chr20	+	45893219	45893311	45892724	45892880	45893987	45894072	29	8	6	0	6	1	1	14	1	0	98	49	0.0382167	1	1	0.4	0.75	0.333	1	0.05
SE	15547	ATG16L2	ATG16L2	chr11	+	72827187	72827293	72826702	72826823	72828358	72828508	132	85	43	7	2	1	1	41	0	1	98	49	0.038578466	1	0.904	0.955	0.956	1	0.953	-0.038
SE	6790	JMJD1C	JMJD1C	chr10	-	63264650	63264764	63217206	63217331	63268662	63269223	152	34	22	6	1	0	1	15	0	0	98	49	0.039711108	1	0.927	0.944	1	1	1	-0.043
SE	11096	ARHGAP15	ARHGAP15	chr2	+	143436912	143437042	143435600	143435699	143487372	143487495	197	49	30	0	0	0	0	19	2	0	98	49	0.039732904	1	1	1	1	0	1	0.5
A5SS	1739	ADPGK	ADPGK	chr15	-	72783245	72783785	72783458	72783785	72774871	72775097	12	11	11	4	0	1	3	7	0	1	98	49	0.039749936	0.728051467	0.6	1	0.846	1	0.778	-0.074
SE	11726	BECN1	BECN1	chr17	-	42815907	42816054	42814523	42814673	42818220	42818415	14	14	6	0	0	0	2	7	0	3	98	49	0.040231039	1	1	1	1	1	0.538	0.231
A3SS	2480	LST1	LST1	chr6	+	31588496	31588909	31588517	31588909	31587943	31587966	21	3	5	16	11	17	1	0	0	6	70	49	0.041875868	0.864437565	0.479	0.16	0.171	1	0	-0.23
SE	5136	MYL6	MYL6	chr12	+	56160625	56160670	56160242	56160320	56161386	56161582	10	3	2	45	57	61	0	1	3	15	94	49	0.041972875	1	0.104	0.027	0.017	0	0.034	0.032
SE	8284	PLK3	PLK3	chr1	+	44801621	44801751	44801035	44801152	44801844	44801932	35	29	7	2	3	0	2	6	0	0	98	49	0.041980644	1	0.897	0.829	1	1	1	-0.091
SE	14777	PLAUR	PLAUR	chr19	-	43655438	43655573	43648566	43649143	43656478	43656640	35	17	4	1	4	0	8	20	0	0	98	49	0.04219046	1	0.946	0.68	1	1	1	-0.125
SE	7861	LRRK2	LRRK2	chr12	+	40313971	40314173	40310430	40310649	40315211	40315300	329	88	41	8	1	4	3	44	0	1	98	49	0.042582705	1	0.954	0.978	0.837	1	0.957	-0.055
SE	16936	IRF1	IRF1	chr5	-	132486794	132486844	132486556	132486686	132486953	132487130	173	106	45	11	1	0	2	21	0	1	98	49	0.042607206	1	0.887	0.981	1	1	0.913	-0.001
SE	2762	PCBP2	PCBP2	chr12	+	53464759	53464852	53462480	53462567	53465931	53465973	98	27	22	27	13	4	0	23	2	16	98	49	0.042684862	1	0.645	0.509	0.733	0	0.418	0.42
SE	9205	ACSL1	ACSL1	chr4	-	184776483	184776662	184773842	184773875	184776883	184776983	213	52	116	7	5	0	3	33	0	1	98	49	0.043517605	1	0.938	0.839	1	1	0.943	-0.046
RI	292	DDIT3	DDIT3	chr12	-	57517268	57517753	57517268	57517438	57517705	57517753	91	57	8	5	4	0	2	7	0	1	98	49	0.044574092	0.735472513	0.901	0.877	1	1	0.778	0.037
SE	7558	RFWD2	RFWD2	chr1	-	176175909	176176007	176163814	176163891	176184632	176184692	106	47	14	3	2	1	2	13	0	0	98	49	0.044718214	1	0.946	0.922	0.875	1	1	-0.086
SE	1246	LRCH4	LRCH4	chr7	-	100577085	100577154	100576901	100577005	100577272	100577389	34	27	13	2	3	0	4	5	0	0	98	49	0.045883646	1	0.895	0.818	1	1	1	-0.096
SE	8398	ATP2A3	ATP2A3	chr17	-	3953346	3953429	3951580	3951685	3953692	3953710	37	21	14	2	3	0	2	4	0	0	98	49	0.046992497	1	0.902	0.778	1	1	1	-0.107
SE	12877	LSM14A	LSM14A	chr19	+	34215123	34215300	34208928	34209051	34215595	34215661	31	12	8	5	0	0	1	7	0	0	98	49	0.047526789	1	0.756	1	1	1	1	-0.081
SE	15922	RPS6KA1	RPS6KA1	chr1	+	26555536	26555625	26555150	26555221	26556653	26556718	106	32	25	1	4	0	0	27	1	4	98	49	0.049990346	1	0.981	0.8	1	0	0.771	0.542
SE	5960	SORL1	SORL1	chr11	+	121570156	121570270	121566939	121567113	121574240	121574363	515	176	96	23	8	4	0	56	1	12	98	49	0.050255826	1	0.918	0.917	0.923	0	0.7	0.569
SE	12750	CYP4F3	CYP4F3	chr19	+	15645718	15645863	15641414	15641613	15647051	15647105	161	45	36	5	2	0	5	21	0	0	98	49	0.050941161	1	0.942	0.918	1	1	1	-0.047
SE	11819	PTPN6	PTPN6	chr12	+	6960805	6960945	6960343	6960435	6961125	6961316	199	83	40	8	3	0	1	28	0	1	98	49	0.05118402	1	0.926	0.933	1	1	0.933	-0.014
SE	5411	NCF2	NCF2	chr1	-	183567203	183567345	183565703	183565779	183569141	183569185	111	80	66	3	3	1	1	25	0	0	98	49	0.051634206	1	0.949	0.93	0.971	1	1	-0.05
SE	5375	MGEA5	MGEA5	chr10	-	101793912	101793998	101792838	101792943	101797979	101798154	19	7	7	8	3	0	0	3	2	1	98	49	0.052864731	1	0.543	0.538	1	0	0.6	0.394
SE	7210	NBEAL2	NBEAL2	chr3	+	47007240	47007350	47006332	47006449	47007524	47007697	24	9	9	4	2	0	1	7	1	0	98	49	0.052977721	1	0.75	0.692	1	0.333	1	0.148
SE	1938	CNPY3	CNPY3	chr6	+	42934474	42934598	42929121	42929721	42935573	42935670	37	18	18	1	4	0	3	10	0	0	98	49	0.053886737	1	0.949	0.692	1	1	1	-0.12
SE	52	IDS	IDS	chrX	-	149503311	149503489	149500948	149501037	149504156	149504293	31	20	4	5	0	0	4	7	0	0	98	49	0.054213286	1	0.756	1	1	1	1	-0.081
SE	1264	ZNF394	ZNF394	chr7	-	99498715	99498842	99493230	99494631	99499637	99500254	14	22	1	2	0	2	2	5	0	0	98	49	0.054854194	1	0.778	1	0.2	1	1	-0.341
SE	8832	DOCK2	DOCK2	chr5	+	169759704	169759775	169747395	169747504	169761518	169761625	103	48	17	3	3	0	3	30	0	0	98	49	0.054985887	1	0.945	0.889	1	1	1	-0.055
SE	15461	RAF1	RAF1	chr3	-	12611949	12612062	12609232	12609335	12618514	12618747	159	54	30	3	0	4	3	15	0	0	98	49	0.055208514	1	0.964	1	0.789	1	1	-0.082
SE	10771	RILPL2	RILPL2	chr12	-	123430507	123430659	123423043	123423157	123436081	123436733	25	15	11	4	4	3	1	12	4	4	98	49	0.055941552	1	0.758	0.652	0.647	0.111	0.6	0.33
SE	51	IDS	IDS	chrX	-	149503311	149503445	149500948	149501037	149504156	149504293	34	20	4	5	0	0	4	7	0	0	98	49	0.056974615	1	0.773	1	1	1	1	-0.076
A3SS	1540	FLII	FLII	chr17	-	18251679	18251816	18251679	18251813	18251998	18252146	8	2	6	18	8	8	2	6	2	3	52	49	0.058311108	1	0.295	0.191	0.414	0.485	0.653	-0.269
SE	5445	BCL6	BCL6	chr3	-	187726730	187726898	187725498	187725629	187728359	187728544	72	29	20	15	5	0	1	25	0	2	98	49	0.060336665	1	0.706	0.744	1	1	0.862	-0.114
SE	10383	PIK3R5	PIK3R5	chr17	-	8889972	8890126	8889138	8889222	8890737	8890912	39	10	7	4	0	1	5	10	0	1	98	49	0.060499246	1	0.83	1	0.778	1	0.833	-0.047
SE	9973	DUSP1	DUSP1	chr5	-	172770160	172770306	172769574	172769794	172770585	172771200	974	462	420	12	6	3	105	424	1	1	98	49	0.061055612	1	0.976	0.975	0.986	0.981	0.995	-0.009
SE	9605	GLT1D1	GLT1D1	chr12	+	128875913	128876062	128853535	128853649	128888638	128888744	58	26	14	4	1	0	3	18	0	0	98	49	0.061072609	1	0.879	0.929	1	1	1	-0.064
SE	9384	AMICA1	AMICA1	chr11	-	118205881	118205991	118203427	118203665	118210486	118210712	265	109	117	7	3	0	1	9	0	0	98	49	0.061322729	1	0.95	0.948	1	1	1	-0.034
SE	6836	GAK	GAK	chr4	-	877614	877809	877089	877207	881906	882040	8	7	5	1	3	0	2	6	0	0	98	49	0.061544568	1	0.8	0.538	1	1	1	-0.221
RI	637	NXF1	NXF1	chr11	-	62798538	62800486	62798538	62798575	62800376	62800486	28	8	2	7	1	0	0	4	1	2	98	49	0.061822068	0.927331017	0.667	0.8	1	0	0.5	0.572
A3SS	2903	TYMP	TYMP	chr22	-	50526000	50526156	50526000	50526141	50526245	50526476	4	5	0	13	14	13	2	0	5	13	64	49	0.062039036	1	0.191	0.215	0	0.234	0	0.018
SE	7830	CD44	CD44	chr11	+	35180273	35180407	35176574	35176740	35186831	35186900	58	6	1	0	0	0	4	16	2	1	98	49	0.0624059	1	1	1	1	0.5	0.889	0.305
SE	10823	HIF1A	HIF1A	chr14	+	61740754	61741188	61738086	61738373	61744704	61744813	35	38	6	0	0	0	1	13	0	3	98	49	0.063816629	1	1	1	1	1	0.684	0.158
A3SS	4076	CNBP	CNBP	chr3	-	129171078	129171280	129171078	129171277	129171445	129171538	18	9	9	22	19	10	0	2	1	4	52	49	0.066637907	1	0.435	0.309	0.459	0	0.32	0.241
A3SS	1970	FCGR3B	FCGR3B	chr1	-	161629777	161630035	161629777	161630032	161630367	161630388	57	9	5	6	0	0	3	1	0	0	52	49	0.067295016	1	0.9	1	1	1	1	-0.033
SE	16938	IRF1	IRF1	chr5	-	132487925	132488025	132486953	132487130	132490544	132490773	75	60	22	18	5	5	7	11	0	2	98	49	0.068165057	1	0.676	0.857	0.687	1	0.733	-0.127
SE	5705	SERPINA1	SERPINA1	chr14	-	94388800	94389010	94388559	94388663	94390456	94390692	66	13	9	28	18	5	1	17	0	4	98	49	0.068748003	1	0.541	0.265	0.474	1	0.68	-0.413
A3SS	3007	STXBP2	STXBP2	chr19	+	7640697	7640809	7640730	7640809	7639730	7639807	1	2	0	6	4	1	0	7	1	1	82	49	0.069429487	1	0.091	0.23	0	0	0.807	-0.297
SE	4942	KIAA1033	KIAA1033	chr12	+	105140293	105140401	105137885	105138011	105140898	105141045	34	14	3	3	0	2	3	12	0	1	98	49	0.069523012	1	0.85	1	0.429	1	0.857	-0.169
SE	12748	CYP4F3	CYP4F3	chr19	+	15643906	15644051	15641414	15641613	15645718	15645863	3	3	6	103	25	21	0	1	4	16	98	49	0.07067314	1	0.014	0.057	0.125	0	0.03	0.05
SE	495	ABCA1	ABCA1	chr9	-	104884426	104884568	104883038	104883157	104889101	104889195	46	21	3	13	2	0	3	20	1	1	98	49	0.070809818	1	0.639	0.84	1	0.6	0.909	0.072
SE	7502	SLC12A6	SLC12A6	chr15	-	34275344	34275389	34260925	34261020	34336409	34336601	155	85	27	2	2	3	6	36	0	0	94	49	0.072192319	1	0.976	0.957	0.824	1	1	-0.081
SE	16969	ETF1	ETF1	chr5	-	138517560	138517700	138513567	138513706	138518691	138518867	43	22	9	3	0	0	0	12	2	0	98	49	0.072739847	1	0.878	1	1	0	1	0.459
SE	5708	SERPINA1	SERPINA1	chr14	-	94388818	94389010	94388559	94388663	94390456	94390692	59	15	11	28	18	5	1	17	0	4	98	49	0.073470529	1	0.513	0.294	0.524	1	0.68	-0.396
RI	202	ULK3	ULK3	chr15	-	74840233	74840641	74840233	74840316	74840497	74840641	4	2	0	1	0	1	2	1	0	3	98	49	0.074113907	1	0.667	1	0	1	0.143	-0.016
A5SS	2605	ACOX1	ACOX1	chr17	-	75978790	75979434	75978964	75979434	75978533	75978693	5	1	0	36	10	4	0	0	2	2	98	49	0.075943129	1	0.065	0.048	0	0	0	0.038
SE	4782	CARD8	CARD8	chr19	-	48218870	48219012	48215339	48215384	48221729	48221855	62	26	16	3	2	0	2	6	0	0	98	49	0.076881264	1	0.912	0.867	1	1	1	-0.074

SE	16211	NSD1	NSD1	chr5	+	177238236	177238507	177235820	177235945	177239755	177239865	19	2	3	2	0	2	3	11	0	0	98	49	0.077838524	1	0.826	1	0.429	1	1	-0.248
SE	12158	CYTH1	CYTH1	chr17	-	78702537	78702604	78702121	78702240	78708196	78708261	32	12	16	6	6	0	0	6	1	3	98	49	0.079729274	1	0.727	0.5	1	0	0.5	0.492
SE	6674	ARHGAP4	ARHGAP4	chrX	-	153913777	153913879	153913408	153913600	153918831	153919053	42	16	19	5	0	0	1	11	0	0	98	49	0.081582192	1	0.808	1	1	1	1	-0.064
A5SS	2845	TLE3	TLE3	chr15	-	70057458	70057658	70057473	70057658	70056297	70056374	40	10	7	28	3	16	1	9	0	3	64	49	0.081597054	1	0.522	0.718	0.251	1	0.697	-0.351
SE	1987	SRPK1	SRPK1	chr6	-	35870280	35870494	35869481	35869901	35870933	35870959	19	4	7	8	10	0	0	4	1	5	98	49	0.082834818	1	0.543	0.167	1	0	0.286	0.427
SE	15984	RNF13	RNF13	chr3	+	149813924	149814026	149812687	149813353	149846010	149846140	4	0	0	25	6	2	0	4	1	2	98	49	0.083683236	1	0.074	0	0	0	0.5	-0.225
SE	3162	PATL2	PATL2	chr15	-	44675485	44675691	44674149	44674230	44676474	44676565	1	1	0	0	0	5	1	3	0	1	98	49	0.084376737	1	1	1	0	1	0.6	-0.133
RI	169	STX4	STX4	chr16	+	31034225	31034536	31034225	31034325	31034461	31034536	18	13	0	1	0	1	1	5	0	3	98	49	0.088362273	1	0.9	1	0	1	0.455	-0.094
A5SS	1475	USF1	USF1	chr1	-	161042554	161042670	161042585	161042670	161042115	161042217	43	24	10	15	2	0	0	9	2	2	80	49	0.088876961	1	0.637	0.88	1	0	0.734	0.472
SE	8679	MAEA	MAEA	chr4	+	1327626	1327703	1322380	1322503	1332756	1332865	13	8	8	0	3	1	3	2	0	0	98	49	0.089381693	1	1	0.571	0.8	1	1	-0.21
SE	14389	SRSF7	SRSF7	chr2	-	38746143	38746179	38743598	38745187	38746693	38746747	31	2	4	1	3	0	2	5	0	0	85	49	0.089682605	1	0.947	0.278	1	1	1	-0.258
SE	9050	STAT2	STAT2	chr12	-	56351291	56351450	56351097	56351190	56354465	56354614	10	6	1	2	2	0	1	7	0	0	98	49	0.09007975	1	0.714	0.6	1	1	1	-0.229
SE	12179	RNF213	RNF213	chr17	+	80389172	80389367	80388611	80388689	80389827	80389917	66	40	17	6	0	0	7	56	0	1	98	49	0.091311053	1	0.846	1	1	1	0.966	-0.034
SE	12247	SMCHD1	SMCHD1	chr18	+	2750042	2750122	2747521	2747647	2750349	2750507	354	198	64	10	13	2	2	54	0	8	98	49	0.091337788	1	0.947	0.884	0.941	1	0.771	0.038
SE	13736	SMG5	SMG5	chr1	-	156266540	156266678	156265780	156266380	156267469	156267678	12	6	2	3	1	0	1	3	0	1	98	49	0.092398999	1	0.667	0.75	1	1	0.6	0.006
SE	12130	SRSF2	SRSF2	chr17	-	76735771	76735875	76734114	76735158	76736153	76736464	26	9	1	16	3	2	1	2	2	3	98	49	0.092646108	1	0.448	0.6	0.2	0.2	0.25	0.191
SE	8463	MAST3	MAST3	chr19	+	18124641	18124774	18124264	18124366	18128399	18128458	64	24	10	1	2	0	1	11	0	3	98	49	0.092711196	1	0.97	0.857	1	1	0.647	0.119
SE	14429	CFLAR	CFLAR	chr2	+	201129728	201130146	201116103	201116481	201133028	201133134	356	120	62	4	2	3	1	63	0	0	98	49	0.093297333	1	0.978	0.968	0.912	1	1	-0.047
SE	5253	PTPRC	PTPRC	chr1	+	198699563	198699704	198692346	198692373	198703297	198703372	78	41	25	260	94	48	3	18	0	25	98	49	0.093994131	1	0.13	0.179	0.207	1	0.265	-0.461
A3SS	709	TMBIM4	TMBIM4	chr12	-	66138723	66138769	66138723	66138734	66145840	66145958	40	23	36	3	0	2	1	12	0	0	84	49	0.09406817	1	0.886	1	0.913	1	1	-0.067
SE	2868	NLRP12	NLRP12	chr19	-	53795858	53796029	53793600	53794136	53801226	53801397	19	6	5	1	3	0	2	2	0	0	98	49	0.095265544	1	0.905	0.5	1	1	1	-0.198
SE	12833	N4BP2L2	N4BP2L2	chr13	-	32527407	32527532	32522181	32522270	32535768	32537027	35	36	13	2	2	1	2	9	0	1	98	49	0.095937215	1	0.897	0.9	0.867	1	0.818	-0.021
SE	4184	GIT2	GIT2	chr12	-	109951211	109951316	109948061	109948846	109953091	109953234	19	5	5	1	1	2	1	2	0	0	98	49	0.096392161	1	0.905	0.714	0.556	1	1	-0.275
SE	12725	ADGRE5	ADGRE5	chr19	+	14396341	14396473	14390923	14391079	14397657	14397804	22	14	13	46	34	6	0	4	1	11	98	49	0.098212686	1	0.193	0.171	0.52	0	0.154	0.218
SE	12710	CBX6	CBX6	chr22	-	38871901	38871945	38871691	38871757	38872121	38872334	2	6	1	0	0	0	0	1	1	0	93	49	0.098245981	1	1	1	1	0	1	0.5
SE	7842	LRRK2	LRRK2	chr12	+	40243549	40243681	40240482	40240617	40249825	40249945	416	161	60	4	6	0	2	41	0	0	98	49	0.100960498	1	0.981	0.931	1	1	1	-0.029
SE	2182	PRRC2A	PRRC2A	chr6	+	31636732	31636945	31636509	31636608	31637041	31637136	7	5	1	0	0	0	0	2	1	0	98	49	0.101331953	1	1	1	1	0	1	0.5
SE	7319	VPS11	VPS11	chr11	+	119078564	119078704	119078172	119078334	119078794	119078997	6	1	2	0	0	0	0	2	1	0	98	49	0.101331953	1	1	1	1	0	1	0.5
SE	10160	BRMS1	BRMS1	chr11	-	66340120	66340213	66338720	66338785	66340773	66340870	21	4	9	0	0	0	0	2	1	0	98	49	0.101331953	1	1	1	1	0	1	0.5
SE	12459	TTN-AS1	TTN-AS1	chr2	+	178571299	178571449	178542221	178542361	178597595	178597681	4	4	3	0	0	0	0	2	1	0	98	49	0.101331953	1	1	1	1	0	1	0.5
SE	17031	PCGF3	PCGF3	chr4	+	764983	765064	761278	761416	766031	770639	30	12	2	0	0	0	0	2	1	0	98	49	0.101331953	1	1	1	1	0	1	0.5
SE	5503	SYNE1	SYNE1	chr6	-	152455885	152456044	152455425	152455590	152458756	152458930	7	7	5	0	0	0	0	1	1	0	98	49	0.101440565	1	1	1	1	0	1	0.5
SE	7688	TFIP11	TFIP11	chr22	-	26494138	26494304	26491928	26492368	26494796	26494939	15	12	4	0	0	0	1	0	0	1	98	49	0.101440565	1	1	1	1	1	0	0.5
SE	9457	ZNF467	ZNF467	chr7	-	149770439	149770556	149769089	149769200	149770998	149771074	17	1	9	0	0	0	0	1	1	0	98	49	0.101440565	1	1	1	1	0	1	0.5
SE	3517	MTMR14	MTMR14	chr3	+	9662266	9662375	9653620	9653769	9668718	9668794	34	19	7	0	0	0	0	1	1	0	98	49	0.101563741	1	1	1	1	0	1	0.5
A5SS	1110	SERPINA1	SERPINA1	chr14	-	94390419	94390692	94390456	94390692	94388818	94389010	85	62	16	44	13	5	4	5	1	15	86	49	0.103524947	1	0.524	0.731	0.646	0.695	0.16	0.206
RI	626	HSH2D	HSH2D	chr19	+	16152551	16153208	16152551	16152641	16153042	16153208	76	27	3	3	2	4	5	21	1	1	98	49	0.104386471	1	0.927	0.871	0.273	0.714	0.913	-0.123
A3SS	3069	LIME1	LIME1	chr20	+	63737972	63738060	63738007	63738060	63737820	63737902	2	0	3	2	1	0	0	1	1	0	84	49	0.10449659	1	0.368	0	1	0	1	-0.044
SE	16882	ALPL	ALPL	chr1	+	21561096	21561212	21560625	21560745	21563109	21563284	124	65	46	3	3	0	3	21	0	0	98	49	0.110341459	1	0.954	0.915	1	1	1	-0.044
SE	4346	OSBPL8	OSBPL8	chr12	-	76459858	76459895	76450850	76450988	76487509	76487618	1	4	4	15	11	1	1	0	0	2	86	49	0.110562647	1	0.037	0.172	0.695	1	0	-0.199
SE	5410	NCF2	NCF2	chr1	-	183566919	183566988	183565703	183565779	183569141	183569185	180	77	88	3	3	1	12	35	0	0	98	49	0.11452548	1	0.968	0.928	0.978	1	1	-0.042
SE	1601	TAGAP	TAGAP	chr6	-	159042077	159042244	159041353	159041515	159043588	159043655	143	66	40	6	1	0	4	19	0	1	98	49	0.116249145	1	0.923	0.971	1	1	0.905	0.012
A3SS	2499	NLRP6	NLRP6	chr11	+	282701	282797	282704	282797	280083	281839	1	0	3	7	8	2	0	0	1	1	52	49	0.116389539	1	0.119	0	0.586	0	0	0.235
A3SS	415	RXRB	RXRB	chr6	-	33195362	33195466	33195362	33195454	33195569	33195702	1	1	2	4	2	0	0	1	1	0	61	49	0.117574196	1	0.167	0.287	1	0	1	-0.015
A3SS	639	ARRB2	ARRB2	chr17	+	4720549	4720640	4720585	4720640	4720392	4720472	9	7	0	57	20	11	0	2	2	13	85	49	0.118308764	1	0.083	0.168	0	0	0.081	0.043
SE	3037	MSRB1	MSRB1	chr16	-	1940777	1940892	1938232	1939143	1941256	1941405	52	44	33	3	2	0	2	10	0	0	98	49	0.11831167	1	0.897	0.917	1	1	1	-0.062
A5SS	1477	IFI6	IFI6	chr1	-	27669220	27669346	27669244	27669346	27668457	27668535	1	2	0	6	10	7	0	5	1	20	73	49	0.119011446	1	0.101	0.118	0	0	0.144	0.001
A5SS	1435	U2AF2	U2AF2	chr19	+	55669082	55669181	55669082	55669169	55669443	55669692	3	0	1	5	4	2	0	0	2	1	61	49	0.119377499	1	0.325	0	0.287	0	0	0.204
A3SS	137	FGD3	FGD3	chr9	+	93034540	93034681	93034543	93034681	93032768	93032873	47	25	14	4	0	2	6	5	0	1	52	49	0.119826628	1	0.917	1	0.868	1	0.825	0.016
SE	12237	SMCHD1	SMCHD1	chr18	+	2694526	2694693	2688627	2688747	2697031	2697122	515	238	81	1	10	0	3	97	0	0	98	49	0.12013002	1	0.996	0.922	1	1	1	-0.027
SE	9959	HDAC7	HDAC7	chr12	-	47786584	47786703	47785751	47785885	47787711	47787809	20	7	4	1	0	0	0	4	1	1	98	49	0.121266089	1	0.909	1	1	0	0.667	0.636
SE	16445	CNN2	CNN2	chr19	+	1036129	1036246	1032558	1032696	1036415	1036562	138	85	62	3	3	1	1	25	0	1	98	49	0.121520206	1	0.958	0.934	0.969	1	0.926	-0.009
SE	3715	TNIP1	TNIP1	chr5	-	151064959	151065131	151063612	151063747	151080879	151081084	17	4	13	6	2	0	0	5	1	2	98	49	0.12328066	1	0.586	0.5	1	0	0.556	0.417
SE	3455	SLC15A3	SLC15A3	chr11	-	60941121	60941290	60939479	60939638	60942034	60942145	25	9	3	16	8	5	2	5	1	1	98	49	0.124092315	1	0.439	0.36	0.231	0.5	0.714	-0.264
SE	12826	GMIP	GMIP	chr19	-	19641809	19641867	19640445	19640571	19641975	19642051	98	64	17	2	0	0	1	27	0	3	98	49	0.1248425	1	0.961	1	1	1	0.818	0.078
SE	9165	CELF2	CELF2	chr10	+	11217424	11217507	11165485	11165682	11249152	11249201	150	68	22	14	3	1	1	31	0	2	98	49	0.125461496	1	0.843	0.919	0.917	1	0.886	-0.05
SE	9858	NADK	NADK	chr1	-	1756502	1756608	1756257	1756343	1757180	1757310	75	38	35	0	0	0	1	24	0	3	98	49	0.126801704	1	1	1	1	1	0.8	0.1
SE	1502	HNRNPA2B1	HNRNPA2B1	chr7	-	26197830	26197866	26197621	26197732	26200571	26200793	4	1	0	27	10	3	0	1	3	7	85	49	0.127514469	1	0.079	0.055	0	0	0.076	0.007
A5SS	1004	SNX17	SNX17	chr2	+	27371268	27371468	27371268	27371343	27372622	27372740	2	0	2	4	1	1	0	0	1	3	98	49	0.127869113	1	0.2	0	0.5	0	0	0.233
A3SS	659	PXN	PXN	chr12	-	120215102	120215417	120215102	120215273	120221622	120221758	21	9	5	31	12	16	1	7	1	3	98	49	0.127945795	1	0.253	0.273	0.135	0.333	0.538	-0.215
SE	15052	ABCG1	ABCG1	chr21	+	42276894	42276945	42273302	42273435	42282273	42282419	9	8	6	0	4	0	1	11	0	0	98	49	0.129179721	1	1	0.5	1	1	1	-0.167
SE	4911	ASAP1	ASAP1	chr8	-	130091972	130092143	130079901	130079971	130112093	130112322	12	4	3	6	0	0	1	1	0	1	98	49	0.129893977	1	0.5	1	1	1	0	0.333
SE	6639	CSF3R	CSF3R	chr1	-	36473434	36473622	36472516	36472686	36473763	36473887	1105	330	219	31	13	3	19	197	1	3	98	49	0.134838662	1	0.947	0.927	0.973	0.905	0.97	0.011
SE	10004	ADRBK1	ADRBK1	chr11	+	67283706	67283773	67283127	67283228	67283853	67283949	123	48	22	6	0	0	13	34	5	0	98	49	0.135600813	1	0.911	1	1	0.565	1	0.188
A5SS	102	PTBP3	PTBP3	chr9	-	112297831	112297916	112297840	112297916	112275843	112276013	19	5	5	13	7	0	0	3	2	2	58	49	0.136047271	1	0.553	0.376	1	0	0.559	0.363
SE	14398	STAT1	STAT1	chr2	-	190983641	190983740	190982382	190982518	190984309	190984393	88	37	23	4	1	0	2	25	0	0	98	49	0.136123209	1	0.917	0.949	1	1	1	-0.045
A3SS	3122	TYROBP	TYROBP	chr19	-	35907217	35907264	35907217	35907261	35907445	35907580	135	71	63	17	13	2	11	42	0	4	52	49	0.136563402	1	0.882	0.837	0.967	1	0.908	-0.059
SE	5704	SERPINA1	SERPINA1	chr14	-	94388800	94389010	94388559	94388660	94390456	94390692	46	13	6	33	5	10	1	17	1	3	98	49	0.137238291	1	0.411	0.565	0.231	0.333	0.739	-0.134
SE	12236	SMCHD1	SMCHD1	chr18	+	2674014	2674145	2673280	2673363	2688393	2688508	698	295	69	5	14	0	7	143	0	2	98	49	0.138416666	1	0.986	0.913	1	1	0.973	-0.02
SE	4372	SNRK	SNRK	chr3	+	43299753	43299815	43286511	43286675	43303097	43303792	81	42	6	1	3	1	1	7	0	0	98	49	0.141049021	1	0.976	0.875	0.75	1	1	-0.133
A3SS	317	GLIPR2	GLIPR2	chr9	+	36162231	36163913	36162361	36163913	36150871	36150949	17	3	2	51	13	20	1	6	0	5	98	49	0.14275805	1	0.143	0.103	0.048	1	0.375	-0.59
SE	3986	PSEN1	PSEN1	chr14	+	73198030	73198129	73192643	73192864	73206385	73206472	13	9	15	5	0	0	1	8	2	1	98	49	0.144842421	1	0.565	1	1	0.2	0.8	0.355
SE	14558	CLK3	CLK3	chr15	+	74625801	74625968	74624901	74625018	74627351	74627446	25	8	3	2	1	1	3	4	0	0	98	49	0.146907852	1	0.862	0.8	0.6	1	1	-0.246
A5SS	1914	ADAR	ADAR	chr1	-	154590183	154590409	154590261	154590409	154589756	154589928	69	26	17	20	1	4	4	24	0	3	98	49	0.147328922	1	0.633	0.929	0.68	1	0.8	-0.153
SE	3714	TNIP1	TNIP1	chr5	-	151064959	151065127	151063612	151063747	151080879	151081088	12	2	8	6	2	0	0	4	1	2	98	49	0.150224689	1	0.5	0.333	1	0	0.5	0.361
SE	4600	TUBA1A	TUBA1A	chr12	-	49186309	49186458	49184794	49185990	49186610	49186833	119	100	82	0	0	0	5	29	1	2	98	49	0.15233388	1	1	1	1	0.714	0.879	0.204
A3SS	2325	ARPC5	ARPC5	chr1	-	183633081	183633163	183633081	183633154	183635516	183635941	7	1	0	103	77	27	0	0	4	20	58	49	0.153712068	1	0.054	0.011	0	0	0	0.022
SE	7711	MDM4	MDM4	chr1	+	204537429	204537497	204532190	204532246	204538208	204538308	23	12	3	2	2	0	1	6	0	0	98	49	0.153839737	1	0.852	0.75	1	1	1	-0.133
SE	11696	OASL	OASL	chr12	-	121033460	121033743	121031441	121031617	121038773	121039242	15	18	2	2	5	0	0	16	2	2	98	49	0.154890071	1	0.789	0.643	1	0	0.8	0.411
A3SS	4117	GRK6	GRK6	chr5	+	177441734	177442849	177441736	177442849	177440918	177441053	8	2	7	7	1	0	1	6	0	2	51	49	0.158599923	1	0.523	0.658	1	1	0.742	-0.144
A5SS	2330	N4BP2L2	N4BP2L2	chr13	-	32527407	32527532	32527457	32527532	32522181	32522270	70	73	47	6	4	10	2	17	0	2	98	49	0.1604111	1	0.854	0.901	0.701	1	0.81	-0.086
SE	5959	SORL1	SORL1	chr11	+	121557313	121557405	121555186	121555318	121558590	121558837	519	211	71	15	4	3	10	73	0	2	98	49	0.164593154	1	0.945	0.963	0.922	1	0.948	-0.031
SE	3036	XPO6	XPO6	chr16	-	28208986	28209122	28180940	28181031	28211365	28211918	8	10	3	48	22	8	1	1	0	17	98	49	0.166415202	1	0.077	0.185	0.158	1	0.029	-0.374
SE	8074	VPS29	VPS29	chr12	-	110492995	110493231	110491522	110492122	110496011	110496094	24	8	11	0	4	0	1	7	0	0	98	49	0.167858835	1	1	0.5	1	1	1	-0.167
SE	11498	SSH2	SSH2	chr17	-	29650653	29650800	29648143	29648344	29655560	29655607	128	82	14	0	0	0	1	32	0	3	98	49	0.168540954	1	1	1	1	1	0.842	0.079
SE	16957	H2AFY	H2AFY	chr5	-	135352945	135353045	135345967	135346057	135360496	135360604	16	12	14	3	0	1	2	10	0	0	98	49	0.171430699	1	0.727	1	0.875	1	1	-0.133
A3SS	2200	STAT2	STAT2	chr12	-	56355707	56355803	56355707	56355791	56356131	56356285	14	3	1	2	0	0	4	17	0	3	61	49	0.174115106	1	0.849	1	1	1	0.82	0.04
A5SS	2171	PGS1	PGS1	chr17	+	78419545	78419725	78419545	78419675	78424060	78424659	61	42	16	4	0	2	10	32	0	1	98	49	0.175839321	1	0.884	1	0.8	1	0.941	-0.076
SE	9035	DENND5A	DENND5A	chr11	-	9145669	9145815	9144994	9145113	9147029	9147151	81	32	23	5	4	1	0	13	1	0	98	49	0.176386755	1	0.89	0.8	0.92	0	1	0.37
SE	6835	GAK	GAK	chr4	-	851749	851974	850935	851084	859605	859722	8	3	6	0	0	0	1	6	2	0	98	49	0.177931592	1	1	1	1	0.2	1	0.4
SE	3021	XPO6	XPO6	chr16	-	28117317	28117462	28112903	28113050	28121669	28121762	267	159	66	4	3	1	1	55	0	1	98	49	0.179229462	1	0.971	0.964	0.971	1	0.965	-0.014
SE	15911	CEP63	CEP63	chr3	+	134558141	134558347	134551925	134552012	134561376	134565028	11	12	1	2	4	3	2	0	0	4	98	49	0.179730224	1	0.733	0.6	0.143	1	0	-0.008
SE	15543	ATG16L2	ATG16L2	chr11	+	72822847	72822961	72822477	72822543	72824059	72824122	161	82	52	3	3	0	9	41	0	0	98	49	0.182129729	1	0.964	0.932	1	1	0.953	-0.011
SE	9063	SIPA1	SIPA1	chr11	+	65644954	65645129	65642462	65642639	65646220	65646378	37	11	11	0	0	0	1	11	2	0	98	49	0.18440821	1	1	1	1	0.2	1	0.4
SE	10876	PSMB4	PSMB4	chr1	+	151399980	151400187	151399564	151399727	151400441	151400588	24	9	14	2	2	0	1	3	0	0	98	49	0.185402396	1	0.857	0.692	1	1	1	-0.15
SE	8604	MAPKAPK2	MAPKAPK2	chr1	+	206731838	206731919	206731639	206731725	206732574	206734285	40	17	20	0	0	0	1	14	2	0	98	49	0.186541703	1	1	1	1	0.2	1	0.4
SE	11695	OASL	OASL	chr12	-	121027575	121027817	121023989	121024137	121031441	121031617	19	18	4	0	0	0	0	26	1	2	98	49	0.186693868	1	1	1	1	0	0.867	0.567
SE	2951	IQGAP1	IQGAP1	chr15	+	90439331	90439399	90433718	90433795	90440501	90440615	217	123	69	3	4	0	1	32	0	0	98	49	0.187913552	1	0.973	0.939	1	1	1	-0.029
SE	2540	MAP3K1	MAP3K1	chr5	+	56875031	56875310	56872824	56873005	56878979	56879101	66	10	4	8	0	0	0	5	1	1	98	49	0.188514258	1	0.805	1	1	0	0.714	0.578
SE	13009	ARHGEF1	ARHGEF1	chr19	+	41905174	41905261	41904948	41905036	41905759	41905827	55	13	18	1	0	0	1	10	2	0	98	49	0.190810353	1	0.965	1	1	0.2	1	0.388
A5SS	1108	SERPINA1	SERPINA1	chr14	-	94388800	94389010	94388818	94389010	94388559	94388663	58	15	15	15	2	6	1	6	0	2	67	49	0.193154337	1	0.739	0.846	0.646	1	0.687	-0.1
SE	4705	LCP1	LCP1	chr13	-	46158825	46158989	46158521	46158651	46159598	46159686	463	214	119	3	7	0	1	110	0	5	98	49	0.19318281	1	0.987	0.939	1	1	0.917	0.017
SE	13214	FES	FES	chr15	+	90889516	90889636	90889305	90889443	90889839	90889962	14	10	6	0	4	0	1	7	0	0	98	49	0.193843347	1	1	0.556	1	1	1	-0.148
RI	580	ATG3	ATG3	chr3	-	112532506	112534337	112532506	112532780	112534268	112534337	10	6	4	28	11	3	0	3	3	5	98	49	0.199919419	1	0.152	0.214	0.4	0	0.231	0.14
A3SS	381	MTCH1	MTCH1	chr6	-	36977633	36977784	36977633	36977691	36978077	36978155	18	5	1	18	5	4	1	1	0	3	98	49	0.205857876	1	0.333	0.333	0.111	1	0.143	-0.312
A3SS	2637	FERMT3	FERMT3	chr11	+	64219878	64220015	64219890	64220015	64219739	64219789	1	2	0	25	9	2	2	1	8	9	61	49	0.206700481	1	0.031	0.151	0	0.167	0.082	-0.064
SE	5948	SORL1	SORL1	chr11	+	121470006	121470123	121452202	121452616	121478117	121478243	354	200	103	3	5	1	9	67	0	1	98	49	0.20705801	1	0.983	0.952	0.981	1	0.971	-0.014
SE	7874	PAK1	PAK1	chr11	-	77325327	77325376	77322014	77323360	77332729	77332867	11	4	0	25	19	1	0	3	1	12	98	49	0.207915739	1	0.18	0.095	0	0	0.111	0.036
A5SS	579	FCAR	FCAR	chr19	+	54888006	54888294	54888006	54888228	54889648	54891420	75	27	8	7	3	0	5	40	2	6	98	49	0.20797773	1	0.843	0.818	1	0.556	0.769	0.224
SE	8769	ZCCHC2	ZCCHC2	chr18	+	62544801	62544873	62542497	62542574	62550347	62550460	26	30	4	0	0	0	0	29	1	2	98	49	0.211120851	1	1	1	1	0	0.879	0.56
SE	15206	ADORA2A	ADORA2A	chr22	+	24433130	24433736	24427561	24427726	24440582	24442361	6	0	1	0	2	0	1	1	0	0	98	49	0.21534978	1	1	0	1	1	1	-0.333
SE	8486	SCYL3	SCYL3	chr1	-	169870254	169870357	169868927	169869039	169873695	169873752	7	3	0	0	0	2	2	1	0	0	98	49	0.215368466	1	1	1	0	1	1	-0.333
SE	4182	GIT2	GIT2	chr12	-	109951166	109951316	109948061	109948846	109953091	109953234	31	17	5	1	1	2	1	3	0	0	98	49	0.215861986	1	0.939	0.895	0.556	1	1	-0.203
SE	11775	FMNL1	FMNL1	chr17	+	45236135	45236244	45234071	45234200	45237280	45237357	115	36	19	1	0	0	8	42	2	1	98	49	0.216566122	1	0.983	1	1	0.667	0.955	0.183
SE	3020	XPO6	XPO6	chr16	-	28111816	28112006	28107521	28107677	28112903	28113050	282	100	34	15	2	0	2	40	1	1	98	49	0.2195329	1	0.904	0.962	1	0.5	0.952	0.229
RI	603	ATP6V0B	ATP6V0B	chr1	+	43977025	43978300	43977025	43977216	43977980	43978300	21	7	1	73	31	55	0	3	4	15	98	49	0.221518601	1	0.126	0.101	0.009	0	0.091	0.033
A3SS	3186	STRN4	STRN4	chr19	-	46727451	46727567	46727451	46727546	46727893	46728007	7	3	1	6	5	0	1	1	0	4	70	49	0.22753096	1	0.45	0.296	1	1	0.149	0.007
SE	6722	TNFRSF1B	TNFRSF1B	chr1	+	12188795	12188895	12167002	12167169	12190956	12191085	127	70	38	8	2	0	7	37	2	3	98	49	0.227588142	1	0.888	0.946	1	0.636	0.86	0.197
A3SS	3372	GNAS	GNAS	chr20	+	58903527	58903585	58903530	58903585	58895611	58895684	37	18	9	10	3	1	3	4	0	2	52	49	0.228963552	1	0.777	0.85	0.895	1	0.653	0.014
SE	16897	VASP	VASP	chr19	+	45523840	45523877	45523643	45523695	45524096	45524142	213	112	95	2	0	0	5	37	0	3	86	49	0.231255615	1	0.984	1	1	1	0.875	0.057
A5SS	640	BANP	BANP	chr16	+	87975047	87975203	87975047	87975185	87981035	87981127	4	4	2	15	9	0	0	2	1	1	67	49	0.235724677	1	0.163	0.245	1	0	0.594	0.172
SE	701	SLC45A4	SLC45A4	chr8	-	141221576	141221765	141219649	141219829	141253988	141254629	26	19	4	2	3	0	0	5	1	0	98	49	0.236450153	1	0.867	0.76	1	0	1	0.376
SE	13642	EPSTI1	EPSTI1	chr13	-	42919299	42919332	42917540	42917624	42926335	42926429	0	1	1	5	3	0	0	4	4	14	82	49	0.238594202	1	0	0.166	1	0	0.146	0.316
SE	978	DOCK5	DOCK5	chr8	+	25407982	25408154	25403557	25403724	25408801	25408940	105	24	4	5	5	1	1	23	1	1	98	49	0.239086319	1	0.913	0.706	0.667	0.333	0.92	0.135
A3SS	2222	NFKB2	NFKB2	chr10	+	102402251	102402529	102402254	102402529	102402047	102402159	4	3	0	5	3	1	1	3	0	3	52	49	0.239433215	1	0.43	0.485	0	1	0.485	-0.437
SE	13860	FOXN2	FOXN2	chr2	+	48328560	48328702	48314655	48314814	48346200	48346751	4	2	0	2	0	1	1	2	0	1	98	49	0.243503547	1	0.5	1	0	1	0.5	-0.25
A5SS	916	TUBA1A	TUBA1A	chr12	-	49188416	49189324	49188976	49189324	49186610	49186833	18	4	1	41	18	9	1	1	1	3	98	49	0.24508394	1	0.18	0.1	0.053	0.333	0.143	-0.127
A5SS	917	TUBA1A	TUBA1A	chr12	-	49188888	49189324	49188976	49189324	49186610	49186833	18	4	1	41	18	9	1	1	1	3	98	49	0.24508394	1	0.18	0.1	0.053	0.333	0.143	-0.127
SE	9048	STAT2	STAT2	chr12	-	56349162	56349261	56348923	56349059	56349425	56349509	12	12	3	4	0	0	1	9	0	0	98	49	0.247044358	1	0.6	1	1	1	1	-0.133
SE	17106	NQO2	NQO2	chr6	+	3004494	3004651	2999815	3000085	3006467	3006559	15	4	1	17	7	0	0	3	5	2	98	49	0.248944006	1	0.306	0.222	1	0	0.429	0.295
SE	1581	IGF2R	IGF2R	chr6	+	160045744	160045882	160044513	160044657	160046497	160046645	374	98	67	2	0	0	0	35	1	1	98	49	0.251838884	1	0.989	1	1	0	0.946	0.523
A3SS	2478	LST1	LST1	chr6	+	31588496	31588909	31588562	31588909	31587640	31587733	66	44	40	10	5	2	3	10	0	2	98	49	0.255381106	1	0.767	0.815	0.909	1	0.714	-0.027
SE	6853	AMPD2	AMPD2	chr1	+	109628642	109628806	109628363	109628495	109629108	109629235	52	31	7	4	0	1	1	2	1	0	98	49	0.256881736	1	0.867	1	0.778	0.333	1	0.215
SE	13047	CHD2	CHD2	chr15	+	92901166	92901299	92900320	92900824	92924320	92924552	40	24	8	9	0	0	4	13	0	2	98	49	0.257739962	1	0.69	1	1	1	0.765	0.014
SE	10856	STAT6	STAT6	chr12	-	57107604	57107743	57107230	57107314	57108162	57108299	90	38	20	18	8	3	1	13	1	5	98	49	0.258500124	1	0.714	0.704	0.769	0.333	0.565	0.28
A3SS	2479	LST1	LST1	chr6	+	31588517	31588909	31588562	31588909	31587640	31587733	65	47	42	10	5	2	3	10	0	2	94	49	0.25902872	1	0.772	0.831	0.916	1	0.723	-0.022
SE	11921	VMP1	VMP1	chr17	+	59737452	59737543	59735337	59735473	59738836	59738947	176	163	135	7	2	1	4	43	0	4	98	49	0.259710227	1	0.926	0.976	0.985	1	0.843	0.041
SE	5188	SAP130	SAP130	chr2	-	127977984	127978089	127954985	127955344	127986784	127986962	1	2	4	0	0	3	2	2	0	0	98	49	0.260139245	1	1	1	0.4	1	1	-0.2
SE	14545	EIF4G2	EIF4G2	chr11	-	10801660	10801774	10800961	10801087	10802048	10802209	65	24	19	5	2	1	0	6	1	1	98	49	0.260458271	1	0.867	0.857	0.905	0	0.75	0.501
SE	12352	DDX17	DDX17	chr22	-	38495795	38495937	38494885	38495046	38498084	38498150	182	92	23	2	5	0	1	31	0	1	98	49	0.262772824	1	0.978	0.902	1	1	0.939	-0.01
SE	13235	RBM23	RBM23	chr14	-	22909482	22909595	22908332	22908380	22911327	22911403	80	34	30	2	3	0	2	4	0	1	98	49	0.265544407	1	0.952	0.85	1	1	0.667	0.101
SE	9302	PIK3CD	PIK3CD	chr1	+	9721443	9721587	9721126	9721248	9721760	9721860	72	39	29	1	3	0	1	19	0	3	98	49	0.265878817	1	0.973	0.867	1	1	0.76	0.067
SE	3576	ACTN1	ACTN1	chr14	-	68909954	68910042	68909317	68909396	68912155	68912242	151	86	54	7	5	0	9	25	2	0	98	49	0.266375307	1	0.915	0.896	1	0.692	1	0.091
SE	5241	CHI3L1	CHI3L1	chr1	-	203180469	203180652	203179760	203179877	203181161	203181285	82	30	43	1	1	2	4	12	0	0	98	49	0.270873952	1	0.976	0.937	0.915	1	1	-0.057
SE	2677	CEACAM3	CEACAM3	chr19	+	41809964	41810017	41808812	41808930	41810322	41810354	61	14	29	0	4	0	5	6	0	0	98	49	0.273594365	1	1	0.636	1	1	1	-0.121
A5SS	2581	HNRNPC	HNRNPC	chr14	-	21230957	21231072	21230996	21231072	21230318	21230366	65	22	16	60	15	22	6	6	1	5	88	49	0.276324142	1	0.376	0.45	0.288	0.77	0.401	-0.214
RI	1035	NCF4	NCF4	chr22	+	36875652	36876094	36875652	36875783	36876028	36876094	62	32	18	80	40	40	2	11	0	11	98	49	0.277848545	1	0.279	0.286	0.184	1	0.333	-0.417
SE	7102	ANKRD12	ANKRD12	chr18	+	9204475	9204544	9195550	9195698	9208656	9208803	28	7	0	20	8	3	1	3	0	2	98	49	0.279726536	1	0.412	0.304	0	1	0.429	-0.476
SE	15047	RAB11FIP1	RAB11FIP1	chr8	-	37871277	37873179	37870419	37870528	37874514	37875322	11	14	12	12	15	8	0	4	1	4	98	49	0.282239472	1	0.314	0.318	0.429	0	0.333	0.187
A3SS	3625	RPS3	RPS3	chr11	+	75402303	75402446	75402351	75402446	75401639	75401733	9	3	1	76	13	8	0	2	2	7	97	49	0.287169766	1	0.056	0.104	0.059	0	0.126	0.01
SE	6114	TSPAN2	TSPAN2	chr1	-	115060463	115060538	115058882	115058981	115062134	115062232	4	5	2	0	3	0	2	2	0	0	98	49	0.28728316	1	1	0.455	1	1	1	-0.182
SE	7849	LRRK2	LRRK2	chr12	+	40293544	40293663	40287350	40287539	40294844	40294914	513	192	66	3	6	0	2	74	0	0	98	49	0.292052716	1	0.988	0.941	1	1	1	-0.024
RI	51	GSTK1	GSTK1	chr7	+	143264991	143265296	143264991	143265092	143265260	143265296	26	2	5	15	4	4	2	3	1	6	98	49	0.297917554	1	0.464	0.2	0.385	0.5	0.2	0
SE	1947	CCND3	CCND3	chr6	-	41940369	41940585	41937234	41937394	42048500	42048894	13	20	6	3	0	1	1	23	0	1	98	49	0.297942063	1	0.684	1	0.75	1	0.92	-0.149
SE	13953	DYSF	DYSF	chr2	+	71656161	71656290	71643964	71644063	71658877	71659033	52	21	7	0	0	0	2	17	2	0	98	49	0.298053201	1	1	1	1	0.333	1	0.334

SE	10845 ITGAX	ITGAX	chr16	+	31371333	31371497	31371083	31371214	31371629	31371784	48	48	16	2	2	1	1	13	1	0	98	49	0.299662353	1	0.923	0.923	0.889	0.333	1	0.245
RI	401 CDK5RAP3	CDK5RAP3	chr17	+	47975513	47976013	47975513	47975653	47975868	47976013	40	14	4	4	0	0	2	6	0	0	98	49	0.300766182	1	0.833	1	1	1	1	-0.056
SE	7862 LRRK2	LRRK2	chr12	+	40319987	40320175	40315211	40315300	40321033	40321188	398	102	74	35	7	2	2	64	4	2	98	49	0.301827803	1	0.85	0.879	0.949	0.2	0.941	0.322
SE	7112 RGS18	RGS18	chr1	+	192159219	192159321	192158461	192158756	192160377	192160439	116	31	14	0	0	4	4	22	0	0	98	49	0.302925336	1	1	1	0.636	1	1	-0.121
SE	13218 SIGLEC5	SIGLEC5	chr19	-	51627461	51627746	51627148	51627248	51627833	51628091	15	19	18	0	0	0	2	8	2	0	98	49	0.30502781	1	1	1	1	0.333	1	0.334
SE	6675 ARHGAP4	ARHGAP4	chrX	-	153919566	153919686	153919154	153919283	153920625	153920808	4	4	2	21	15	2	2	0	2	2	98	49	0.305352645	1	0.087	0.118	0.333	0.333	0	0.013
SE	700 SLC45A4	SLC45A4	chr8	-	141217089	141217189	141215758	141215970	141218010	141219029	34	25	4	4	0	0	4	2	0	0	98	49	0.305687075	1	0.81	1	1	1	1	-0.063
SE	3766 CD47	CD47	chr3	-	108051938	108051970	108043093	108047292	108057476	108057569	14	6	6	1	0	0	0	5	1	1	81	49	0.308504515	1	0.894	1	1	0	0.752	0.589
A3SS	2631 HIF1A	HIF1A	chr14	+	61720378	61720572	61720381	61720572	61695400	61695839	25	8	3	40	22	5	1	4	1	14	52	49	0.310039241	1	0.371	0.255	0.361	0.485	0.212	-0.019
SE	3738 DDX58	DDX58	chr9	-	32500804	32500939	32493760	32493942	32526060	32526324	51	14	9	1	3	0	1	33	0	0	98	49	0.312908735	1	0.962	0.7	1	1	1	-0.113
SE	15478 SLC6A6	SLC6A6	chr3	+	14479084	14479185	14478465	14478568	14481670	14481841	129	53	23	4	1	0	3	12	0	0	98	49	0.313509545	1	0.942	0.964	1	1	1	-0.031
A3SS	3149 TMEM91	TMEM91	chr19	+	41383560	41384082	41383873	41384082	41382771	41382921	3	5	6	0	3	0	1	3	0	0	98	49	0.313679604	1	1	0.455	1	1	1	-0.182
SE	4183 GIT2	GIT2	chr12	-	109951211	109951316	109939164	109939247	109953091	109953234	17	5	5	0	0	3	1	2	0	0	98	49	0.315095602	1	1	1	0.455	1	1	-0.182
SE	8978 SAT1	SAT1	chrX	+	23784293	23784403	23783799	23783883	23785327	23785429	114	53	25	115	88	54	5	44	10	78	98	49	0.315758853	1	0.331	0.231	0.188	0.2	0.22	0.04
A5SS	2846 TLE3	TLE3	chr15	-	70058149	70058291	70058158	70058291	70057458	70057658	11	0	0	22	8	12	0	2	1	4	58	49	0.317935248	1	0.297	0	0	0	0.297	-0.05
A3SS	2087 MAPKAPK2	MAPKAPK2	chr1	+	206732037	206734285	206732574	206734285	206731838	206731919	19	8	0	21	9	11	0	4	1	9	98	49	0.319337215	1	0.311	0.308	0	0	0.182	0.115
SE	13061 FOSB	FOSB	chr19	+	45471193	45471301	45470628	45470949	45472550	45475179	62	18	1	9	4	0	3	9	2	0	98	49	0.319578232	1	0.775	0.692	1	0.429	1	0.108
SE	5706 SERPINA1	SERPINA1	chr14	-	94388818	94389010	94388559	94388660	94390456	94390692	62	16	16	33	5	10	1	16	1	3	98	49	0.319885457	1	0.484	0.615	0.444	0.333	0.727	-0.016
SE	11222 CASP4	CASP4	chr11	-	104950924	104951098	104949542	104949777	104954746	104955001	92	21	45	15	4	0	0	17	1	2	98	49	0.319912879	1	0.754	0.724	1	0	0.81	0.421
RI	295 DDIT3	DDIT3	chr12	-	57517268	57517753	57517268	57517595	57517705	57517753	97	35	5	3	0	0	2	10	0	0	98	49	0.322152533	1	0.942	1	1	1	1	-0.019
SE	4483 USP15	USP15	chr12	+	62325871	62325933	62321463	62321609	62349220	62349307	283	86	43	5	0	1	2	32	0	1	98	49	0.323933701	1	0.966	1	0.956	1	0.941	0.003
SE	10726 EIF1	EIF1	chr17	+	41689777	41689941	41688874	41689069	41690087	41690189	39	17	12	2	2	0	1	10	0	0	98	49	0.32396072	1	0.907	0.81	1	1	1	-0.094
SE	11056 THEMIS2	THEMIS2	chr1	+	27881970	27883043	27879643	27880054	27885294	27885451	71	18	19	2	0	1	1	19	0	1	98	49	0.326458678	1	0.947	1	0.905	1	0.905	-0.002
SE	5702 SERPINA1	SERPINA1	chr14	-	94388559	94388663	94382591	94383241	94390456	94390692	97	33	42	107	35	38	0	17	2	20	98	49	0.327279187	1	0.312	0.32	0.356	0	0.298	0.18
SE	12965 DDX60L	DDX60L	chr4	-	168419301	168419397	168416681	168416797	168420260	168420380	26	27	8	3	0	0	1	32	0	1	98	49	0.327799215	1	0.812	1	1	1	0.941	-0.033
SE	10741 MYO1F	MYO1F	chr19	-	8548035	8548122	8545649	8545736	8548236	8548317	301	188	125	0	0	0	9	61	0	2	98	49	0.329790459	1	1	1	1	1	0.938	0.031
SE	2766 PCBP2	PCBP2	chr12	+	53467804	53467843	53467220	53467293	53468776	53468832	60	29	10	14	0	2	1	9	0	7	88	49	0.331743206	1	0.705	1	0.736	1	0.417	0.105
SE	12248 SMCHD1	SMCHD1	chr18	+	2751277	2751393	2750349	2750507	2752487	2752552	188	121	48	0	6	0	8	40	0	1	98	49	0.332012882	1	1	0.91	1	1	0.952	-0.006
SE	12962 DDX60L	DDX60L	chr4	-	168395958	168396124	168394460	168394617	168400825	168400978	83	27	6	3	0	0	6	32	0	0	98	49	0.332424323	1	0.933	1	1	1	1	-0.022
SE	10367 AKNA	AKNA	chr9	-	114381059	114381446	114376465	114377532	114394356	114394405	88	34	19	3	0	0	1	14	0	0	98	49	0.332785768	1	0.936	1	1	1	1	-0.021
RI	773 MAP7D1	MAP7D1	chr1	+	36176198	36176581	36176198	36176321	36176417	36176581	28	18	2	6	1	4	1	8	1	1	98	49	0.333527727	1	0.7	0.9	0.2	0.333	0.8	0.033
A5SS	744 HAUS4	HAUS4	chr14	-	22956915	22957142	22956923	22957142	22955099	22955176	4	3	6	6	3	1	0	3	1	2	57	49	0.335219927	1	0.364	0.462	0.838	0	0.563	0.273
SE	10958 CR1	CR1	chr1	+	207609289	207609688	207607250	207607336	207611676	207611853	63	11	18	1	3	0	1	20	0	0	98	49	0.335963878	1	0.969	0.647	1	1	1	-0.128
SE	12631 ALOX5	ALOX5	chr10	+	45382482	45382681	45374175	45374429	45395854	45395936	196	76	31	0	0	0	6	67	0	2	98	49	0.338181246	1	1	1	1	1	0.944	0.028
SE	15588 CTNNB1	CTNNB1	chr3	+	41227207	41227352	41225659	41225861	41233340	41233444	34	12	26	4	0	0	1	9	0	0	98	49	0.339007481	1	0.81	1	1	1	1	-0.063
A5SS	1106 SERPINA1	SERPINA1	chr14	-	94390419	94390692	94390456	94390692	94382591	94383241	87	62	16	107	35	38	4	5	2	20	86	49	0.339487748	1	0.317	0.502	0.193	0.533	0.125	0.008
SE	7959 TNRC6B	TNRC6B	chr22	+	40125771	40125862	40117054	40117128	40156114	40156182	38	24	8	4	0	0	1	5	0	0	98	49	0.340420177	1	0.826	1	1	1	1	-0.058
SE	3287 RNASET2	RNASET2	chr6	-	166948569	166948625	166946681	166946739	166952487	166952548	287	68	83	3	2	2	8	51	1	0	98	49	0.341060884	1	0.98	0.944	0.954	0.8	1	0.059
A3SS	988 UBE2D3	UBE2D3	chr4	-	102826484	102826844	102826484	102826636	102827591	102827948	126	52	28	6	1	2	5	24	0	2	98	49	0.341867183	1	0.913	0.963	0.875	1	0.857	-0.012
SE	3141 EIF4A2	EIF4A2	chr3	+	186784961	186785101	186784563	186784696	186785882	186786051	32	7	7	25	4	0	1	6	2	3	98	49	0.342609716	1	0.39	0.467	1	0.2	0.5	0.269
SE	15542 LTB	LTB	chr6	-	31581813	31581859	31581558	31581630	31582255	31582425	30	15	4	4	1	2	0	5	3	0	95	49	0.345211048	1	0.795	0.886	0.508	0	1	0.23
SE	516 FGD3	FGD3	chr9	+	93002924	93003014	92976207	92976709	93004000	93004137	107	64	24	0	5	0	10	22	0	0	98	49	0.346206533	1	1	0.865	1	1	1	-0.045
SE	10737 MYO1F	MYO1F	chr19	-	8539946	8540028	8536948	8537055	8541905	8541991	210	144	78	0	0	0	6	55	0	2	98	49	0.346482776	1	1	1	1	1	0.932	0.034
SE	5964 SORL1	SORL1	chr11	+	121577280	121577400	121574240	121574363	121583457	121583583	414	148	108	3	0	0	8	61	0	3	98	49	0.346526483	1	0.986	1	1	1	0.91	0.04
SE	976 DOCK5	DOCK5	chr8	+	25368570	25368725	25366869	25366970	25369536	25369641	85	22	4	3	0	0	1	12	0	0	98	49	0.347461072	1	0.934	1	1	1	1	-0.022
SE	11431 MX1	MX1	chr21	+	41443787	41443866	41441715	41441914	41445447	41445570	50	61	8	0	0	0	4	62	0	2	98	49	0.348348103	1	1	1	1	1	0.939	0.03
SE	2179 PRRC2A	PRRC2A	chr6	+	31625791	31625871	31625459	31625611	31626019	31626162	22	1	6	1	2	0	4	4	0	1	98	49	0.352306411	1	0.917	0.2	1	1	0.667	-0.128
SE	3265 DGAT2	DGAT2	chr11	+	75784617	75784746	75768732	75769112	75790187	75790295	33	26	25	0	4	0	7	3	0	0	98	49	0.352706146	1	1	0.765	1	1	1	-0.078
SE	492 ABCA1	ABCA1	chr9	-	104810799	104810924	104809465	104809564	104812573	104812722	18	1	4	6	5	2	0	3	1	1	98	49	0.353286236	1	0.6	0.091	0.5	0	0.6	0.097
SE	5920 MYD88	MYD88	chr3	+	38140387	38140592	38139863	38139998	38140756	38140848	4	5	4	0	0	0	0	8	1	1	98	49	0.354202895	1	1	1	1	0	0.8	0.6
SE	4297 PACSIN2	PACSIN2	chr22	-	42876887	42877010	42876136	42876333	42879047	42879169	37	20	13	2	7	1	0	5	3	0	98	49	0.354244445	1	0.902	0.588	0.867	0	1	0.286
SE	4305 OAZ1	OAZ1	chr19	+	2271390	2271530	2269485	2269744	2271781	2271954	190	118	175	6	1	0	8	81	0	0	98	49	0.354607926	1	0.941	0.983	1	1	1	-0.025
SE	3027 XPO6	XPO6	chr16	-	28135215	28135324	28133840	28133933	28146093	28146203	317	187	67	1	0	0	5	50	0	2	98	49	0.357703086	1	0.994	1	1	1	0.926	0.035
SE	9881 BAZ2B	BAZ2B	chr2	-	159438302	159438695	159432756	159433363	159439008	159439212	112	30	9	3	0	0	1	18	0	0	98	49	0.35775207	1	0.949	1	1	1	1	-0.017
SE	33 FLNA	FLNA	chrX	-	154357250	154357274	154354824	154355072	154357433	154357623	18	6	0	23	5	3	1	4	7	5	73	49	0.359250106	1	0.344	0.446	0	0.087	0.349	0.045
SE	14571 ADIPOR1	ADIPOR1	chr1	-	202954240	202954307	202950929	202951164	202958184	202958396	2	5	0	4	2	2	3	1	1	1	98	49	0.361687671	1	0.2	0.556	0	0.6	0.333	-0.215
SE	7060 CHD1	CHD1	chr5	-	98881974	98882123	98881278	98881375	98883087	98883237	48	28	8	0	4	0	2	14	0	0	98	49	0.361850765	1	1	0.778	1	1	1	-0.074
SE	7781 CYLD	CYLD	chr16	+	50781245	50781411	50779664	50780044	50782324	50782466	72	46	22	1	2	0	1	18	0	0	98	49	0.364325029	1	0.973	0.92	1	1	1	-0.036
A3SS	3774 SUN2	SUN2	chr22	-	38750224	38750383	38750224	38750320	38750897	38751035	6	3	1	20	9	6	0	2	1	1	98	49	0.365156177	1	0.13	0.143	0.077	0	0.5	-0.133
A3SS	603 BTN3A1	BTN3A1	chr6	+	26407670	26407952	26407826	26407952	26405908	26406256	33	15	3	0	4	0	2	3	0	0	98	49	0.365827954	1	1	0.652	1	1	1	-0.116
A3SS	1073 OSBPL8	OSBPL8	chr12	-	76450850	76450988	76450850	76450979	76487509	76487618	30	24	9	8	8	3	0	3	1	1	58	49	0.367474353	1	0.76	0.717	0.717	0	0.717	0.373
SE	6612 AKAP13	AKAP13	chr15	+	85662387	85662453	85658536	85658590	85664562	85664755	4	1	1	22	14	3	0	2	2	11	98	49	0.367993412	1	0.083	0.034	0.143	0	0.083	0.045
A3SS	1953 RNF19B	RNF19B	chr1	-	32936445	32937309	32936445	32937259	32938396	32938528	40	11	15	42	10	11	1	9	0	5	98	49	0.368999566	1	0.323	0.355	0.405	1	0.474	-0.376
SE	316 WWC3	WWC3	chrX	+	10117123	10117689	10116417	10116507	10122618	10122739	40	23	14	4	0	0	1	4	0	0	98	49	0.373057457	1	0.833	1	1	1	1	-0.056
SE	2394 TMEM164	TMEM164	chrX	-	110067346	110067396	110003500	110004164	110109079	110109146	65	27	1	3	0	0	2	14	0	0	98	49	0.376246788	1	0.915	1	1	1	1	-0.028
SE	13855 GNAS	GNAS	chr20	+	58898940	58898985	58895611	58895684	58903530	58903585	18	7	2	10	3	1	1	3	0	2	94	49	0.377418069	1	0.484	0.549	0.51	1	0.439	-0.205
SE	7972 LCOR	LCOR	chr10	+	96944112	96944245	96907667	96907747	96949007	96949295	17	8	4	4	0	0	2	0	0	1	98	49	0.37804421	1	0.68	1	1	1	0	0.393
SE	15173 SHKBP1	SHKBP1	chr19	+	40589081	40589178	40588623	40588779	40590729	40590853	67	18	15	3	0	0	4	12	0	0	98	49	0.378060424	1	0.918	1	1	1	1	-0.027
SE	3824 PTPRE	PTPRE	chr10	+	128060938	128061015	128056122	128056213	128061678	128061715	118	64	26	2	2	0	3	11	0	0	98	49	0.378698267	1	0.967	0.941	1	1	1	-0.031
SE	282 SH3KBP1	SH3KBP1	chrX	-	19683822	19684028	19645399	19645475	19695611	19695741	59	21	14	1	0	3	3	13	0	0	98	49	0.379406837	1	0.967	1	0.7	1	1	-0.111
SE	12240 SMCHD1	SMCHD1	chr18	+	2718157	2718235	2707806	2707920	2718314	2718434	320	153	43	3	0	4	2	71	0	0	98	49	0.37951052	1	0.982	1	0.843	1	1	-0.058
SE	4221 DEF8	DEF8	chr16	+	89962011	89962125	89961736	89961864	89963362	89963443	32	31	5	2	1	0	3	5	0	0	98	49	0.379976534	1	0.889	0.939	1	1	1	-0.057
SE	2802 IFI44L	IFI44L	chr1	+	78629719	78629850	78628950	78628999	78635336	78635489	9	3	1	0	1	0	3	61	0	2	98	49	0.382224179	1	1	0.6	1	1	0.938	-0.102
RI	232 ZFP36L1	ZFP36L1	chr14	-	68787654	68790492	68787654	68788809	68789526	68790492	173	46	11	5	5	2	4	17	0	2	98	49	0.382258712	1	0.945	0.821	0.733	1	0.81	-0.072
SE	11429 MX1	MX1	chr21	+	41430532	41430608	41427754	41427866	41432049	41432175	41	24	6	0	0	0	6	38	0	2	98	49	0.382268666	1	1	1	1	1	0.905	0.047
SE	7863 LRRK2	LRRK2	chr12	+	40322034	40322181	40321033	40321188	40322318	40322510	353	137	46	0	0	0	1	45	0	2	98	49	0.388091869	1	1	1	1	1	0.918	0.041
SE	1579 IGF2R	IGF2R	chr6	+	160029549	160029655	160027184	160027314	160032550	160032713	344	145	49	7	5	0	14	45	0	4	98	49	0.389795203	1	0.961	0.935	1	1	0.849	0.041
A3SS	2558 SF1	SF1	chr11	-	64764603	64766155	64764603	64765518	64766899	64767079	16	4	1	4	0	0	3	7	0	0	98	49	0.390237161	1	0.667	1	1	1	1	-0.111
A3SS	3621 TPD52L2	TPD52L2	chr20	+	63882718	63882820	63882749	63882820	63875815	63875875	48	23	8	2	1	0	2	11	0	1	80	49	0.392016835	1	0.936	0.934	1	1	0.871	0.021
A3SS	3930 FBXL5	FBXL5	chr4	-	15644492	15644708	15644492	15644657	15655203	15655412	81	58	25	1	2	0	1	40	0	0	98	49	0.393596853	1	0.976	0.935	1	1	1	-0.03
SE	12020 KLHL2	KLHL2	chr4	+	165238777	165238899	165228806	165228913	165263196	165263359	14	0	6	4	3	1	0	4	1	2	98	49	0.39720417	1	0.636	0	0.75	0	0.5	0.212
SE	11956 CHD3	CHD3	chr17	+	7902936	7903061	7902609	7902727	7903271	7903503	9	7	2	0	0	0	1	3	0	2	98	49	0.39801607	1	1	1	1	1	0.429	0.285
A3SS	2329 GMPR2	GMPR2	chr14	+	24238245	24239241	24238588	24239241	24237519	24237562	23	13	4	3	2	4	0	3	2	0	98	49	0.398116415	1	0.793	0.765	0.333	0	1	0.13
A3SS	752 ISG20	ISG20	chr15	+	88650234	88652310	88652169	88652310	88639342	88639594	47	52	24	1	1	1	1	17	0	0	98	49	0.399153396	1	0.959	0.963	0.923	1	1	-0.052
SE	14416 ANKRD44	ANKRD44	chr2	-	197122649	197122792	197121331	197121544	197125380	197125468	151	81	24	2	2	0	4	11	0	0	98	49	0.399864693	1	0.974	0.953	1	1	1	-0.024
SE	6529 LYST	LYST	chr1	-	235791698	235792125	235788700	235788845	235793502	235793612	169	78	9	1	3	0	1	20	0	0	98	49	0.402422043	1	0.988	0.929	1	1	1	-0.028
SE	10735 MYO1F	MYO1F	chr19	-	8530458	8530573	8530195	8530365	8536251	8536396	77	70	51	1	3	0	3	27	0	0	98	49	0.403164003	1	0.975	0.921	1	1	1	-0.035
SE	17074 GRK6	GRK6	chr5	+	177433346	177433410	177433146	177433239	177433535	177433676	28	29	18	2	2	0	4	11	0	0	98	49	0.403207343	1	0.875	0.879	1	1	1	-0.082
SE	6005 NIN	NIN	chr14	-	50821873	50822077	50806736	50806818	50830463	50830517	61	31	6	3	1	0	3	8	0	1	98	49	0.403251294	1	0.91	0.939	1	1	0.8	0.05
SE	10818 HIF1A	HIF1A	chr14	+	61720381	61720572	61695400	61695839	61721508	61721654	109	71	25	1	3	0	6	35	0	0	98	49	0.404683891	1	0.982	0.922	1	1	1	-0.032
SE	15298 TRIOBP	TRIOBP	chr22	+	37769261	37769375	37768073	37768176	37771649	37771736	12	2	1	0	0	0	2	3	0	2	98	49	0.404694173	1	1	1	1	1	0.429	0.285
SE	7631 CSDE1	CSDE1	chr1	-	114739691	114739890	114737962	114738072	114749820	114750207	111	34	20	3	0	0	2	23	0	0	98	49	0.405048782	1	0.949	1	1	1	1	-0.017
SE	10003 ADRBK1	ADRBK1	chr11	+	67281092	67281184	67280731	67280783	67281458	67281558	152	66	46	2	2	0	2	32	0	0	98	49	0.405794829	1	0.974	0.943	1	1	1	-0.028
SE	15609 HIPK3	HIPK3	chr11	+	33348521	33348818	33347851	33348013	33349146	33349287	54	19	5	2	1	0	1	4	0	0	98	49	0.406461955	1	0.931	0.905	1	1	1	-0.055
SE	8973 FCGR2A	FCGR2A	chr1	+	161510833	161510956	161509819	161510074	161513894	161513932	349	205	165	1	3	0	1	66	0	2	98	49	0.40674608	1	0.994	0.972	1	1	0.943	0.017
SE	14733 HCK	HCK	chr20	+	32054215	32054281	32052187	32052486	32071661	32071782	19	4	8	135	59	44	0	5	9	33	98	49	0.408086651	1	0.066	0.033	0.083	0	0.07	0.026
SE	16016 CCNL1	CCNL1	chr3	-	157149286	157149385	157147555	157148589	157149484	157149596	172	75	20	2	2	0	1	41	0	0	98	49	0.408138584	1	0.977	0.949	1	1	1	-0.025
SE	2024 TAPBP	TAPBP	chr6	-	33303954	33303989	33299694	33301771	33304127	33304217	78	38	14	3	0	0	2	23	1	0	84	49	0.412371599	1	0.938	1	1	0.538	1	0.21
SE	15171 SHKBP1	SHKBP1	chr19	+	40582350	40582466	40580745	40580936	40583397	40583485	110	30	27	3	0	0	1	14	0	0	98	49	0.414401449	1	0.948	1	1	1	1	-0.017
SE	368 RXRA	RXRA	chr9	+	134417157	134417327	134408939	134409119	134421675	134421805	69	31	17	3	0	0	3	11	0	0	98	49	0.41476143	1	0.92	1	1	1	1	-0.027
SE	14779 PLAUR	PLAUR	chr19	-	43665315	43665459	43656478	43656640	43667580	43667691	119	68	19	2	2	0	13	65	0	0	98	49	0.415108943	1	0.967	0.944	1	1	1	-0.03
SE	12822 MCL1	MCL1	chr1	-	150578243	150578491	150574550	150577491	150578842	150578963	544	221	161	66	59	10	5	123	4	15	98	49	0.417762806	1	0.805	0.652	0.89	0.385	0.804	0.188
SE	6782 JMJD1C	JMJD1C	chr10	-	63186214	63186383	63185562	63185653	63189167	63189446	115	40	13	3	0	0	2	9	0	0	98	49	0.417962582	1	0.95	1	1	1	1	-0.017
SE	611 TLN1	TLN1	chr9	-	35704668	35704815	35704331	35704498	35705550	35705670	156	32	27	2	3	0	3	28	0	0	98	49	0.420055523	1	0.975	0.842	1	1	1	-0.061
SE	7706 TBXAS1	TBXAS1	chr7	+	139953367	139953456	139936190	139936307	139957633	139957764	90	67	13	3	0	0	1	32	0	0	98	49	0.420526793	1	0.938	1	1	1	1	-0.021
A3SS	2476 LST1	LST1	chr6	+	31588517	31588909	31588562	31588909	31587199	31587318	108	64	58	35	11	14	3	14	1	6	94	49	0.420817006	1	0.617	0.752	0.684	0.61	0.549	0.105
SE	8099 DEDD2	DEDD2	chr19	-	42202050	42202072	42198592	42199829	42209699	42209840	3	0	1	16	25	11	0	3	3	5	71	49	0.421373914	1	0.115	0	0.059	0	0.293	-0.088
SE	12176 RNF213	RNF213	chr17	+	80369767	80369867	80369501	80369671	80371873	80371985	109	38	8	0	1	0	10	63	0	2	98	49	0.423196935	1	1	0.95	1	1	0.94	0.013
SE	12195 SP110	SP110	chr2	-	230216780	230216928	230214949	230215118	230219873	230220112	27	29	6	1	2	0	5	11	0	0	98	49	0.423386143	1	0.931	0.879	1	1	1	-0.063
SE	10846 ITGAX	ITGAX	chr16	+	31372377	31372509	31371629	31371784	31372596	31372670	186	116	43	2	0	0	16	38	0	2	98	49	0.423798056	1	0.979	1	1	1	0.905	0.04
A5SS	2156 HN1	HN1	chr17	-	75147519	75147653	75147555	75147653	75146665	75146684	0	1	4	34	19	8	0	1	1	2	85	49	0.424216982	1	0	0.029	0.224	0	0.224	-0.028
SE	8525 NFKBIZ	NFKBIZ	chr3	+	101855394	101855458	101855061	101855208	101855732	101855902	124	40	7	3	0	0	1	28	0	0	98	49	0.426137175	1	0.954	1	1	1	1	-0.015
SE	3708 IRF9	IRF9	chr14	+	24163877	24163959	24163377	24163508	24164062	24164134	46	27	7	4	6	0	3	25	1	5	98	49	0.426518129	1	0.852	0.692	1	0.6	0.714	0.191
SE	9065 SIPA1	SIPA1	chr11	+	65649261	65649480	65647383	65647658	65649560	65649672	60	21	9	3	0	0	3	9	0	0	98	49	0.426604104	1	0.909	1	1	1	1	-0.03
A3SS	605 BTN3A1	BTN3A1	chr6	+	26412411	26415216	26413168	26415216	26411554	26411581	19	6	4	17	0	0	1	5	0	2	98	49	0.426625721	1	0.358	1	1	1	0.556	0.008
SE	13589 FAM129A	FAM129A	chr1	-	184803584	184803692	184798078	184798190	184805945	184806056	214	78	64	2	4	0	1	38	0	0	98	49	0.428739516	1	0.982	0.907	1	1	1	-0.037
SE	16420 RHOA	RHOA	chr3	-	49368427	49368548	49362495	49362626	49375433	49375591	157	74	57	3	1	0	3	34	0	0	98	49	0.431166683	1	0.963	0.974	1	1	1	-0.021
SE	5062 ARAP1	ARAP1	chr11	-	72699004	72699107	72697910	72698106	72699416	72699552	163	116	39	0	1	0	8	34	0	2	98	49	0.434451175	1	1	0.983	1	1	0.895	0.047
A3SS	4087 SPI1	SPI1	chr11	-	47375632	47375732	47375632	47375729	47378308	47378576	51	15	7	58	22	9	8	16	4	19	52	49	0.435975739	1	0.453	0.391	0.423	0.653	0.442	-0.125
SE	2306 HLA-A	HLA-A	chr6	+	29944499	29944616	29944121	29944397	29945058	29945091	734	514	291	1	4	0	41	187	1	2	98	49	0.436480895	1	0.997	0.985	1	0.953	0.979	0.028
SE	2689 STX4	STX4	chr16	+	31034969	31035040	31034225	31034536	31037925	31038034	13	13	1	0	0	2	1	3	0	0	98	49	0.436872697	1	1	1	0.2	1	1	-0.267
SE	14888 PREX1	PREX1	chr20	-	48650893	48651055	48649995	48650206	48651395	48651583	129	77	48	0	0	0	3	32	0	2	98	49	0.43772807	1	1	1	1	1	0.889	0.055
A3SS	606 BTN3A1	BTN3A1	chr6	+	26412704	26415216	26413168	26415216	26411554	26411581	21	8	4	17	0	0	1	5	0	2	98	49	0.439597639	1	0.382	1	1	1	0.556	0.016
SE	4482 HBP1	HBP1	chr7	+	107189291	107189448	107186568	107186681	107190172	107190317	29	13	19	4	0	0	1	1	0	1	98	49	0.439686131	1	0.784	1	1	1	0.333	0.261
A5SS	1551 OS9	OS9	chr12	+	57718145	57718421	57718145	57718376	57718992	57719182	99	50	22	1	2	0	6	6	0	0	94	49	0.443569573	1	0.981	0.929	1	1	1	-0.03
SE	14047 TMEM127	TMEM127	chr2	-	96265137	96265512	96254832	96254997	96265868	96266013	27	6	12	0	3	0	3	11	0	1	98	49	0.444110003	1	1	0.5	1	1	0.846	-0.09
SE	13049 CHD2	CHD2	chr15	+	92937517	92937625	92929029	92929091	92939577	92939718	65	24	3	4	0	0	1	7	0	0	98	49	0.446218154	1	0.89	1	1	1	1	-0.037
SE	12793 JAK3	JAK3	chr19	-	17835082	17835215	17834851	17835003	17835923	17836051	53	36	7	0	4	0	6	18	0	0	98	49	0.447951856	1	1	0.818	1	1	1	-0.061
RI	787 TYMP	TYMP	chr22	-	50529495	50530085	50529495	50529719	50529903	50530085	72	56	11	4	4	5	8	20	0	13	98	49	0.449170597	1	0.9	0.875	0.524	1	0.435	0.049
A5SS	313 EEF1D	EEF1D	chr8	-	143581228	143581328	143581285	143581328	143581053	143581154	89	43	46	3	0	0	8	16	0	0	98	49	0.449604503	1	0.937	1	1	1	1	-0.021
SE	7926 EPB41	EPB41	chr1	+	29060421	29060484	29053103	29053312	29064981	29065158	3	0	0	21	11	11	0	0	2	10	98	49	0.449822548	1	0.067	0	0	0	0	0.022
SE	9197 ACSL1	ACSL1	chr4	-	184764852	184764925	184763166	184763255	184766621	184766756	147	62	37	0	1	0	2	44	0	2	98	49	0.450670684	1	1	0.969	1	1	0.917	0.031
SE	15280 TOM1	TOM1	chr22	+	35321958	35322037	35317876	35317961	35323027	35323177	24	34	9	1	2	0	4	11	0	1	98	49	0.45078102	1	0.923	0.895	1	1	0.846	0.016
RI	1042 PLIN2	PLIN2	chr9	-	19126113	19126448	19126113	19126309	19126396	19126448	1	2	0	2	0	1	0	1	1	0	98	49	0.45174834	1	0.2	1	0	0	1	-0.1
SE	1939 UBR2	UBR2	chr6	+	42573933	42573993	42564021	42564397	42592150	42592229	156	72	10	0	4	1	5	9	0	0	98	49	0.452057563	1	1	0.9	0.833	1	1	-0.089
SE	16878 ALPL	ALPL	chr1	+	21553977	21554142	21509364	21509517	21560625	21560745	64	35	11	2	1	0	2	14	0	0	98	49	0.457733702	1	0.941	0.946	1	1	1	-0.038
SE	3002 R3HDM4	R3HDM4	chr19	-	901421	901546	900828	900952	901975	902130	128	109	50	5	0	0	6	19	0	1	98	49	0.458344066	1	0.928	1	1	1	0.905	0.023
SE	11816 PTPN6	PTPN6	chr12	+	6957918	6958073	6957653	6957785	6959926	6959994	138	67	28	23	4	3	4	43	4	3	98	49	0.458449304	1	0.75	0.893	0.824	0.333	0.878	0.217
SE	8817 ATXN2	ATXN2	chr12	-	111510384	111510582	111509890	111509998	111513356	111513539	7	5	0	2	0	1	0	2	1	0	98	49	0.460013403	1	0.636	1	0	0	1	0.045
SE	16308 HOTAIRM1	HOTAIRM1	chr7	+	27099097	27099366	27096093	27096388	27099778	27100258	5	0	0	21	27	5	0	0	1	1	98	49	0.464250946	1	0.106	0	0	0	0	0.035
SE	1582 IGF2R	IGF2R	chr6	+	160048343	160048543	160047791	160047907	160050472	160050652	233	104	38	0	2	0	3	42	0	2	98	49	0.464661849	1	1	0.963	1	1	0.913	0.031
SE	11898 TRIM25	TRIM25	chr17	-	56895925	56895952	56895520	56895604	56899114	56899180	36	14	12	2	2	0	1	3	0	1	76	49	0.46806809	1	0.921	0.819	1	1	0.659	0.084
A3SS	3409 TMEM127	TMEM127	chr2	-	96265137	96265512	96265137	96265489	96265868	96266013	21	3	3	10	7	1	4	2	3	4	72	49	0.469441269	1	0.588	0.226	0.671	0.476	0.254	0.13
SE	8049 ADAM8	ADAM8	chr10	-	133263687	133263765	133262415	133263233	133267351	133267417	100	41	18	16	3	2	4	10	2	2	98	49	0.469863379	1	0.758	0.872	0.818	0.5	0.714	0.209
A5SS	740 RNF31	RNF31	chr14	+	24148257	24148477	24148257	24148413	24148641	24148701	4	1	0	6	3	2	1	1	0	3	98	49	0.472635957	1	0.25	0.143	0	1	0.143	-0.441
SE	5917 MYD88	MYD88	chr3	+	38139863	38139998	38138477	38139028	38140756	38140848	50	12	5	3	0	0	6	12	0	0	98	49	0.473541925	1	0.893	1	1	1	1	-0.036
A5SS	2716 POLD4	POLD4	chr11	-	67353293	67353596	67353363	67353596	67352987	67353077	14	11	3	0	1	2	1	1	0	0	98	49	0.476503373	1	1	0.846	0.429	1	1	-0.242
SE	1136 LINC-PINT	LINC-PINT	chr7	-	130967388	130967517	130944159	130945835	130984052	130984109	36	7	0	14	1	2	0	5	1	2	98	49	0.476619732	1	0.562	0.778	0	0	0.556	0.169
SE	8831 DOCK2	DOCK2	chr5	+	169714359	169714457	169714027	169714211	169716212	169716302	109	62	13	3	0	0	13	24	0	0	98	49	0.477632073	1	0.948	1	1	1	1	-0.017
SE	5223 MX2	MX2	chr21	+	41380016	41380151	41377788	41377981	41382409	41382564	32	26	37	5	1	0	4	25	0	2	98	49	0.478098223	1	0.762	0.929	1	1	0.862	-0.034
SE	4593 PARP8	PARP8	chr5	+	50821212	50821338	50815431	50815524	50822334	50822400	53	30	6	2	2	0	2	9	0	1	98	49	0.478298961	1	0.93	0.882	1	1	0.818	0.028
SE	12594 STXBP2	STXBP2	chr19	+	7640730	7640809	7639730	7639807	7640899	7641003	28	16	11	2	1	0	1	7	0	0	98	49	0.478353872	1	0.875	0.889	1	1	1	-0.079
A3SS	3788 LPPR2	LPPR2	chr19	+	11359531	11359720	11359606	11359720	11357659	11357739	20	16	12	4	0	0	1	6	0	0	98	49	0.479378889	1	0.714	1	1	1	1	-0.095
SE	4419 ARHGAP9	ARHGAP9	chr12	-	57476870	57476963	57476589	57476651	57477155	57477269	192	72	60	7	5	0	2	25	0	2	98	49	0.480342678	1	0.932	0.878	1	1	0.862	0.006
SE	5177 SLC12A9	SLC12A9	chr7	+	100861736	100861911	100861391	100861584	100862680	100862827	53	23	12	2	1	0	2	16	0	0	98	49	0.480487308	1	0.93	0.92	1	1	1	-0.05

SE	12573 VAV1	VAV1	chr19	+	6822418	6822514	6822220	6822329	6825052	6825121	91	45	14	3	7	0	0	15	2	0	98	49	0.481387676	1	0.938	0.763	1	0	1	0.4
SE	13803 PARP14	PARP14	chr3	+	122699389	122701635	122695425	122695662	122703741	122703978	78	24	1	0	0	0	8	30	0	2	98	49	0.481745882	1	1	1	1	1	0.882	0.059
SE	8919 ATP6V0B	ATP6V0B	chr1	+	43977025	43977216	43976772	43976824	43977980	43978300	91	62	65	1	3	0	4	22	0	0	98	49	0.483149345	1	0.978	0.912	1	1	1	-0.037
SE	15965 DPEP2	DPEP2	chr16	-	67990046	67990131	67989322	67989398	67990820	67990997	105	42	37	3	0	0	1	15	0	0	98	49	0.483939387	1	0.946	1	1	1	1	-0.018
SE	5221 BCL2A1	BCL2A1	chr15	-	79967636	79967692	79960889	79961174	79970699	79971301	10	4	6	104	148	64	0	2	1	19	98	49	0.485539689	1	0.046	0.013	0.045	0	0.05	0.01
SE	15339 TLE3	TLE3	chr15	-	70059409	70059460	70058662	70058815	70060529	70060649	157	61	34	0	0	0	4	31	2	0	98	49	0.486682137	1	1	1	1	0.5	1	0.25
SE	14401 STAT1	STAT1	chr2	-	191013524	191013678	191009875	191010004	191014017	191014250	89	53	9	3	4	0	5	34	0	2	98	49	0.491119546	1	0.937	0.869	1	1	0.895	-0.012
SE	7748 ACAP1	ACAP1	chr17	+	7337311	7337369	7336528	7336787	7341947	7342067	24	18	5	2	1	0	1	3	0	0	98	49	0.491953713	1	0.857	0.9	1	1	1	-0.081
SE	1248 PILRA	PILRA	chr7	+	100389887	100390106	100374043	100374433	100397878	100397912	91	45	54	17	6	18	4	21	0	5	98	49	0.492450455	1	0.728	0.789	0.6	1	0.677	-0.133
SE	3064 DDX5	DDX5	chr17	-	64504676	64504842	64504221	64504318	64506075	64506366	293	110	22	1	4	0	2	38	0	0	98	49	0.493007314	1	0.993	0.932	1	1	1	-0.025
SE	14933 OSBPL2	OSBPL2	chr20	+	62259980	62260125	62256056	62256221	62263615	62263691	30	14	10	2	1	0	2	3	0	0	98	49	0.494721497	1	0.882	0.875	1	1	1	-0.081
SE	13839 PPM1B	PPM1B	chr2	+	44209209	44209327	44201185	44202045	44217966	44218078	27	9	12	0	3	0	3	7	0	0	98	49	0.494769315	1	1	0.6	1	1	1	-0.133
SE	10608 MICAL2	MICAL2	chr11	+	12204249	12204457	12162078	12162419	12208022	12208139	74	30	7	3	0	0	1	17	0	1	98	49	0.49895827	1	0.925	1	1	1	0.895	0.027
SE	7206 NBEAL2	NBEAL2	chr3	+	47003173	47003309	47002956	47003081	47003815	47003976	120	36	9	0	0	0	14	31	0	2	98	49	0.499363766	1	1	1	1	1	0.886	0.057
A3SS	3712 TOM1	TOM1	chr22	+	35322998	35323177	35323027	35323177	35321958	35322037	7	5	0	14	19	3	1	5	4	6	78	49	0.499951338	1	0.239	0.142	0	0.136	0.344	-0.113
SE	12634 ALOX5	ALOX5	chr10	+	45444115	45444286	45443727	45443828	45445507	45446119	96	37	30	0	1	0	2	41	0	2	98	49	0.500085279	1	1	0.949	1	1	0.911	0.027
A5SS	2045 RPL13A	RPL13A	chr19	+	49489849	49489922	49489849	49489912	49490231	49490297	99	13	1	3	1	0	3	11	0	1	59	49	0.501906011	1	0.965	0.915	1	1	0.901	0.009
SE	14397 STAT1	STAT1	chr2	-	190979771	190979866	190978855	190979001	190980619	190980669	41	25	5	2	4	0	1	20	1	2	98	49	0.502698128	1	0.911	0.758	1	0.333	0.833	0.307
SE	11487 FLOT2	FLOT2	chr17	-	28881191	28881375	28880712	28880862	28881813	28882028	144	64	62	0	0	0	3	27	0	2	98	49	0.502725055	1	1	1	1	1	0.871	0.065
SE	15289 NCF4	NCF4	chr22	+	36864044	36864129	36860987	36861203	36864918	36865072	197	128	96	5	0	0	8	28	0	0	98	49	0.502830419	1	0.952	1	1	1	1	-0.016
SE	2484 ARRB2	ARRB2	chr17	+	4718260	4718345	4717887	4718023	4718611	4718684	106	27	85	30	9	10	0	26	1	3	98	49	0.504310317	1	0.639	0.6	0.81	0	0.812	0.277
SE	14887 PREX1	PREX1	chr20	-	48649995	48650206	48649299	48649576	48650893	48651055	184	95	70	2	3	0	8	40	0	0	98	49	0.505945346	1	0.979	0.941	1	1	1	-0.027
A3SS	1764 GPBP1	GPBP1	chr5	+	57249408	57249576	57249453	57249576	57247074	57247215	42	25	25	2	0	1	1	5	0	0	94	49	0.506543634	1	0.916	1	0.929	1	1	-0.052
SE	7869 LRRK2	LRRK2	chr12	+	40354298	40354492	40351538	40351733	40356114	40356187	206	77	31	1	0	0	2	26	0	2	98	49	0.510946613	1	0.99	1	1	1	0.867	0.063
SE	14024 SMAD2	SMAD2	chr18	-	47870474	47870564	47869242	47869436	47896520	47896809	55	18	19	3	0	0	1	1	0	0	98	49	0.516332408	1	0.902	1	1	1	1	-0.033
SE	715 DENND3	DENND3	chr8	+	141190283	141190417	141188985	141189146	141192330	141192449	68	28	4	2	1	1	6	22	0	0	98	49	0.516511922	1	0.944	0.933	0.667	1	1	-0.152
SE	5699 SERPINA1	SERPINA1	chr14	-	94388559	94388660	94382591	94383241	94390456	94390692	102	20	47	107	35	38	1	16	2	20	98	49	0.517955822	1	0.323	0.222	0.382	0.2	0.286	0.066
SE	3934 TPT1	TPT1	chr13	-	45339496	45339602	45338659	45338776	45339993	45340184	544	187	145	4	4	0	6	79	0	3	98	49	0.518168375	1	0.986	0.959	1	1	0.929	0.017
SE	5918 MYD88	MYD88	chr3	+	38140387	38140568	38138477	38139028	38140756	38140848	12	1	3	3	0	0	3	7	0	0	98	49	0.522673916	1	0.667	1	1	1	1	-0.111
SE	5308 LDB1	LDB1	chr10	-	102110528	102110701	102109920	102110043	102110868	102110971	34	8	7	0	0	0	3	5	0	2	98	49	0.523770865	1	1	1	1	1	0.556	0.222
SE	2036 TAP1	TAP1	chr6	-	32850943	32851149	32850319	32850517	32852108	32852239	70	36	31	2	0	1	2	13	0	0	98	49	0.525221449	1	0.946	1	0.939	1	1	-0.038
SE	6288 TCEB2	TCEB2	chr16	-	2775456	2775556	2771413	2772102	2776992	2777127	35	36	31	1	0	3	1	12	0	0	98	49	0.525837001	1	0.946	1	0.838	1	1	-0.072
SE	11452 CARD16	CARD16	chr11	-	105043482	105043545	105041325	105041719	105044391	105044658	48	16	42	20	3	6	1	14	1	5	98	49	0.528217041	1	0.545	0.727	0.778	0.333	0.583	0.225
SE	4012 MTMR6	MTMR6	chr13	-	25251648	25251775	25246200	25249492	25251852	25251984	27	9	8	0	0	0	2	5	0	2	98	49	0.529097092	1	1	1	1	1	0.556	0.222
SE	16389 HERC5	HERC5	chr4	+	88462134	88462356	88460094	88460171	88463531	88463623	3	17	1	1	1	0	4	25	0	2	98	49	0.529188344	1	0.6	0.895	1	1	0.862	-0.099
SE	7026 ELOVL5	ELOVL5	chr6	-	53287861	53287942	53276178	53276256	53291775	53291963	1	1	2	47	19	21	0	0	2	5	98	49	0.530437106	1	0.011	0.026	0.045	0	0	0.027
A5SS	598 STX16	STX16	chr20	+	58651252	58652138	58651252	58652087	58667489	58667597	13	8	3	3	2	2	4	7	0	3	98	49	0.531451593	1	0.684	0.667	0.429	1	0.538	-0.176
SE	6530 LYST	LYST	chr1	-	235804503	235804665	235802907	235803064	235805742	235806772	186	55	10	0	0	0	4	25	0	2	98	49	0.533639671	1	1	1	1	1	0.862	0.069
SE	15466 STAT5A	STAT5A	chr17	+	42291971	42292061	42289865	42290022	42295618	42295793	9	5	6	0	2	1	1	2	2	0	98	49	0.535091642	1	1	0.556	0.75	0.2	1	0.169
SE	5626 CD74	CD74	chr5	-	150403120	150403312	150402562	150402625	150404679	150404767	21	2	0	114	6	7	2	7	2	32	98	49	0.537096648	1	0.084	0.143	0	0.333	0.099	-0.14
SE	14925 ZBP1	ZBP1	chr20	-	57616243	57616468	57615511	57615580	57620261	57620576	13	7	4	3	0	0	1	9	0	0	98	49	0.538885454	1	0.684	1	1	1	1	-0.105
SE	273 GK	GK	chrX	+	30724100	30724181	30720851	30720995	30728731	30731460	69	41	22	2	2	0	4	5	0	0	98	49	0.541146332	1	0.945	0.911	1	1	1	-0.048
SE	1731 VNN3	VNN3	chr6	-	132726721	132727012	132724605	132724979	132728740	132728933	79	48	5	22	9	1	0	11	1	1	98	49	0.54337128	1	0.642	0.727	0.714	0	0.846	0.271
SE	15760 LSP1	LSP1	chr11	+	1881431	1881596	1880086	1880224	1883418	1883560	223	169	106	1	1	3	7	58	0	0	98	49	0.544999045	1	0.991	0.988	0.946	1	1	-0.025
SE	2829 STK38L	STK38L	chr12	+	27308338	27308461	27302136	27302188	27309113	27309197	42	12	7	2	1	0	3	8	0	0	98	49	0.545126619	1	0.913	0.857	1	1	1	-0.077
SE	8154 CAP1	CAP1	chr1	+	40069689	40069874	40067539	40067717	40070158	40070282	72	34	18	0	4	0	2	10	0	0	98	49	0.545980056	1	1	0.81	1	1	1	-0.063
SE	12089 ARRDC3	ARRDC3	chr5	-	91376620	91376768	91375510	91375613	91378693	91378775	106	33	48	18	10	1	1	13	0	6	98	49	0.54624919	1	0.746	0.623	0.96	1	0.52	0.016
SE	352 NOTCH1	NOTCH1	chr9	-	136515980	136516094	136515482	136515716	136517271	136517385	59	30	12	2	1	0	1	10	0	0	98	49	0.546699365	1	0.937	0.937	1	1	1	-0.042
SE	10817 HIF1A	HIF1A	chr14	+	61720378	61720572	61695400	61695839	61721508	61721654	94	57	23	1	3	0	6	25	0	0	98	49	0.547705846	1	0.979	0.905	1	1	1	-0.039
SE	10243 LST1	LST1	chr6	+	31587943	31587966	31587199	31587318	31588496	31588909	9	9	2	3	2	0	1	1	0	3	72	49	0.547785373	1	0.671	0.754	1	1	0.185	0.216
SE	953 SARAF	SARAF	chr8	-	30073876	30074055	30069641	30070059	30082846	30083208	72	48	39	20	10	11	3	13	1	5	98	49	0.549549436	1	0.643	0.706	0.639	0.6	0.565	0.08
SE	12701 GLUL	GLUL	chr1	-	182387130	182387292	182386255	182386402	182388571	182388750	1143	456	144	3	3	0	25	173	0	3	98	49	0.553318418	1	0.995	0.987	1	1	0.966	0.011
SE	9556 HNRNPU	HNRNPU	chr1	-	244858010	244858274	244857597	244857717	244858728	244858841	82	35	16	1	2	0	2	18	0	2	98	49	0.553341978	1	0.976	0.897	1	1	0.818	0.049
SE	4125 RPLP0	RPLP0	chr12	-	120198553	120198739	120197321	120197462	120198853	120199000	41	5	8	3	0	0	1	3	0	0	98	49	0.555263141	1	0.872	1	1	1	1	-0.043
SE	8828 DOCK2	DOCK2	chr5	+	169684195	169684350	169681743	169681879	169689251	169689333	203	80	23	3	1	1	4	41	0	0	98	49	0.555294052	1	0.971	0.976	0.92	1	1	-0.044
SE	10034 PRKCD	PRKCD	chr3	+	53165103	53165215	53161206	53161428	53178403	53178537	8	0	2	5	1	0	4	3	2	1	98	49	0.558514749	1	0.444	0	1	0.5	0.6	-0.069
SE	517 FGD3	FGD3	chr9	+	93019830	93019861	93018135	93018215	93020316	93020424	85	35	25	2	1	0	3	10	0	1	80	49	0.559138062	1	0.963	0.955	1	1	0.86	0.043
SE	8751 NABP1	NABP1	chr2	+	191682491	191682595	191681945	191682017	191683728	191683804	42	11	10	48	10	4	2	4	0	5	98	49	0.559187208	1	0.304	0.355	0.556	1	0.286	-0.238
SE	4151 PSTPIP1	PSTPIP1	chr15	+	77032297	77032394	77031179	77031278	77035507	77035563	11	5	4	3	0	0	1	3	0	0	98	49	0.562425178	1	0.647	1	1	1	1	-0.118
SE	137 PGK1	PGK1	chrX	+	78124873	78125051	78123194	78123374	78125326	78125425	78	40	29	1	2	0	2	30	0	0	98	49	0.565144298	1	0.975	0.909	1	1	1	-0.039
A3SS	2905 TYMP	TYMP	chr22	-	50529495	50529754	50529495	50529719	50529903	50530085	38	29	7	4	4	5	3	9	0	13	84	49	0.565719428	1	0.847	0.809	0.45	1	0.288	0.058
SE	12963 DDX60L	DDX60L	chr4	-	168395958	168396127	168394460	168394617	168400825	168400978	62	58	6	3	0	0	4	29	0	0	98	49	0.566280009	1	0.912	1	1	1	1	-0.029
SE	6242 APBB1IP	APBB1IP	chr10	+	26536073	26536217	26533438	26533525	26541581	26541692	99	17	22	1	3	0	1	12	0	0	98	49	0.566991124	1	0.98	0.739	1	1	1	-0.094
SE	11052 THEMIS2	THEMIS2	chr1	+	27879643	27880054	27876587	27876728	27881970	27882125	155	32	32	0	5	0	7	42	0	1	98	49	0.56829042	1	1	0.762	1	1	0.955	-0.057
SE	2382 FAM65B	FAM65B	chr6	-	24848024	24848154	24842861	24843554	24849801	24849950	314	131	97	3	3	0	3	57	0	0	98	49	0.569484819	1	0.981	0.956	1	1	1	-0.021
SE	14618 USP32	USP32	chr17	-	60180544	60180637	60177329	60179428	60181323	60181748	67	31	5	3	0	0	0	12	1	0	98	49	0.569592115	1	0.918	1	1	0	1	0.473
SE	4932 TLE4	TLE4	chr9	+	79708592	79708786	79708117	79708250	79709622	79709699	41	4	11	3	0	0	3	7	0	0	98	49	0.571129463	1	0.872	1	1	1	1	-0.043
SE	12509 ABCA7	ABCA7	chr19	+	1063759	1063863	1063578	1063678	1064160	1064253	2	5	1	2	0	0	1	3	0	0	98	49	0.572393395	1	0.333	1	1	1	1	-0.222
SE	14792 PHF1	PHF1	chr6	+	33414954	33415144	33414724	33414829	33415234	33415329	2	5	1	2	0	0	1	1	0	0	98	49	0.572393395	1	0.333	1	1	1	1	-0.222
A3SS	2653 IFITM3	IFITM3	chr11	-	319672	319990	319672	319985	320564	320914	22	6	8	0	0	3	1	33	0	0	54	49	0.573839549	1	1	1	0.708	1	1	-0.097
SE	6426 RALGAPA2	RALGAPA2	chr20	-	20680690	20680801	20653529	20653587	20712374	20712623	23	10	2	2	1	0	2	7	0	0	98	49	0.574229202	1	0.852	0.833	1	1	1	-0.105
SE	9970 DUSP1	DUSP1	chr5	-	172769754	172769849	172768089	172769132	172770160	172770306	842	360	330	3	7	4	17	269	0	3	98	49	0.576118237	1	0.993	0.963	0.976	1	0.978	-0.012
A5SS	1592 TBC1D10C	TBC1D10C	chr11	+	67406626	67406738	67406626	67406692	67406826	67407016	38	9	8	8	5	0	1	2	0	4	95	49	0.577419673	1	0.71	0.481	1	1	0.205	0.128
SE	15458 RAF1	RAF1	chr3	-	12590797	12590974	12587590	12587637	12591707	12591792	180	64	32	2	0	0	1	24	0	2	98	49	0.579441471	1	0.978	1	1	1	0.857	0.064
SE	6704 LAPTM5	LAPTM5	chr1	-	30742455	30742549	30741639	30741716	30757658	30757836	90	35	46	1	0	0	3	22	0	2	98	49	0.580977501	1	0.978	1	1	1	0.846	0.07
A3SS	2839 PTPN6	PTPN6	chr12	+	6960662	6960945	6960805	6960945	6960343	6960435	26	31	0	95	46	27	1	8	1	17	98	49	0.581966841	1	0.12	0.252	0	0.333	0.19	-0.138
SE	9269 NFKBID	NFKBID	chr19	+	35897650	35897856	35896912	35897058	35898471	35898532	18	4	1	0	1	0	7	10	2	0	98	49	0.582524393	1	1	0.667	1	0.636	1	0.071
A3SS	985 ELF1	ELF1	chr13	-	40949805	40949973	40949805	40949901	40951328	40951436	43	12	9	3	0	0	3	15	0	0	98	49	0.582990467	1	0.878	1	1	1	1	-0.041
SE	14246 ITGAL	ITGAL	chr16	+	30489253	30489386	30489081	30489155	30494211	30494363	43	17	4	3	0	0	1	10	0	0	98	49	0.583106327	1	0.878	1	1	1	1	-0.041
SE	10074 CD53	CD53	chr1	+	110897808	110897892	110891391	110891471	110899123	110899936	98	50	56	3	0	0	2	18	0	0	98	49	0.583622234	1	0.942	1	1	1	1	-0.019
SE	15985 RNF13	RNF13	chr3	+	149911977	149912083	149902071	149902162	149921133	149921227	129	71	27	0	0	4	3	27	0	0	98	49	0.583653552	1	1	1	0.771	1	1	-0.076
SE	10080 CAMK2G	CAMK2G	chr10	-	73839538	73839601	73837467	73837511	73842168	73842211	1	0	3	17	10	5	0	0	1	4	98	49	0.584918649	1	0.029	0	0.231	0	0	0.087
SE	12997 SASH3	SASH3	chrX	+	129792327	129792476	129790939	129791081	129792626	129792836	51	31	9	3	0	0	3	17	0	0	98	49	0.586405367	1	0.895	1	1	1	1	-0.035
SE	5115 N4BP1	N4BP1	chr16	-	48551385	48551482	48548006	48548114	48553538	48553669	47	10	7	10	5	1	1	10	0	3	98	49	0.588328632	1	0.701	0.5	0.778	1	0.625	-0.153
SE	6964 HCLS1	HCLS1	chr3	-	121632331	121632563	121632100	121632184	121633066	121633171	174	114	99	2	2	0	10	51	0	2	98	49	0.591514184	1	0.978	0.966	1	1	0.927	0.018
SE	15923 RPS6KA1	RPS6KA1	chr1	+	26556653	26556718	26555150	26555221	26556997	26557100	45	24	23	3	1	0	4	8	0	0	98	49	0.593177157	1	0.882	0.923	1	1	1	-0.065
SE	5916 MYD88	MYD88	chr3	+	38139863	38139998	38138477	38139028	38140387	38140568	54	17	9	3	0	0	5	19	2	0	98	49	0.594277976	1	0.9	1	1	0.556	1	0.189
SE	4181 GIT2	GIT2	chr12	-	109951166	109951316	109947255	109947504	109953091	109953234	21	13	10	0	3	0	1	5	0	1	98	49	0.595484855	1	1	0.684	1	1	0.714	0.038
SE	5444 BCL6	BCL6	chr3	-	187724940	187725078	187721376	187722601	187725498	187725629	143	71	32	1	4	0	8	53	0	0	98	49	0.596641777	1	0.986	0.899	1	1	1	-0.038
SE	11633 FKBP8	FKBP8	chr19	-	18539550	18539720	18539459	[illegible]	18541678	18541995	119	51	36	3	0	0	2	16	0	0	98	49	0.59812016	1	0.952	1	1	1	1	-0.016
SE	11189 CD300LF	CD300LF	chr17	-	74705203	74705377	74704477	74704816	74712823	74713000	3	2	0	7	4	1	0	4	2	0	98	49	0.600055751	1	0.176	0.2	0	0	1	-0.375
SE	10847 ITGAX	ITGAX	chr16	+	31373248	31373390	31372596	31372670	31376798	31376915	156	142	31	10	6	0	12	40	0	5	98	49	0.600219064	1	0.886	0.922	1	1	0.8	0.036
SE	14417 ANKRD44	ANKRD44	chr2	-	197187022	197187106	197147026	197147105	197310577	197310797	189	78	20	2	3	0	3	25	0	0	98	49	0.600837139	1	0.979	0.929	1	1	1	-0.031
A3SS	1461 SERPINA1	SERPINA1	chr14	-	94388559	94388663	94388559	94388660	94390456	94390692	31	18	5	33	5	10	0	5	1	3	52	49	0.601284271	1	0.47	0.772	0.32	0	0.611	0.215
SE	1108 MKRN1	MKRN1	chr7	-	140474380	140474489	140471882	140472011	140479159	140479569	0	0	3	5	3	3	0	0	1	1	98	49	0.601469268	1	0	0	0.333	0	0	0.111
A3SS	1888 MDM4	MDM4	chr1	+	204537429	204537497	204537458	204537497	204532190	204532246	31	9	2	3	0	0	1	10	0	0	78	49	0.60228108	1	0.867	1	1	1	1	-0.044
SE	8583 RASSF3	RASSF3	chr12	+	64688215	64688453	64684786	64684894	64691469	64691579	91	31	62	1	0	0	1	21	0	2	98	49	0.603346308	1	0.978	1	1	1	0.84	0.073
SE	8824 AQP9	AQP9	chr15	+	58179127	58179345	58174917	58175036	58183960	58185911	229	142	134	4	2	0	1	35	0	0	98	49	0.604291852	1	0.966	0.973	1	1	1	-0.02
SE	9289 MBNL1	MBNL1	chr3	+	152456266	152456361	152455541	152455577	152459270	152459345	51	22	22	3	0	0	1	10	0	0	98	49	0.608449796	1	0.895	1	1	1	1	-0.035
SE	2787 ARHGAP27	ARHGAP27	chr17	-	45404268	45404334	45404028	45404096	45404444	45404528	39	9	5	18	5	1	2	4	0	3	98	49	0.608834548	1	0.52	0.474	0.714	1	0.4	-0.131
A5SS	808 UBE2D3	UBE2D3	chr4	-	102827426	102827948	102827591	102827948	102826484	102826636	25	11	1	6	1	2	2	2	0	2	98	49	0.609205648	1	0.676	0.846	0.2	1	0.333	-0.093
SE	8678 MAEA	MAEA	chr4	+	1327626	1327703	1315396	1315600	1332756	1332865	12	5	7	0	4	0	3	2	1	1	98	49	0.60937349	1	1	0.385	1	0.6	0.5	0.245
SE	4595 PARP8	PARP8	chr5	+	50834930	50835015	50833978	50834048	50841965	50846522	122	72	14	3	0	0	4	16	0	0	98	49	0.610101228	1	0.953	1	1	1	1	-0.016
SE	17053 LCP2	LCP2	chr5	-	170270718	170270917	170268384	170268482	170274300	170274338	71	18	28	2	2	0	1	19	0	0	98	49	0.611178802	1	0.947	0.818	1	1	1	-0.078
SE	12250 SMCHD1	SMCHD1	chr18	+	2762104	2762236	2760651	2760739	2763636	2763789	300	180	52	5	0	0	2	58	0	0	98	49	0.612583031	1	0.968	1	1	1	1	-0.011
A5SS	1651 GAPVD1	GAPVD1	chr9	+	125321432	125321562	125321432	125321499	125323797	125323923	15	4	1	3	0	0	3	3	0	0	98	49	0.614143158	1	0.714	1	1	1	1	-0.095
SE	15170 SHKBP1	SHKBP1	chr19	+	40580565	40580656	40580323	40580485	40580745	40580936	72	13	18	0	3	0	3	17	0	0	98	49	0.614265951	1	1	0.684	1	1	1	-0.105
SE	1103 MGAM	MGAM	chr7	+	142064383	142064522	142063498	142063586	142065334	142065468	133	36	29	1	1	0	4	8	0	2	98	49	0.617391375	1	0.985	0.947	1	1	0.667	0.144
SE	6445 IRAK3	IRAK3	chr12	+	66226722	66226837	66217170	66217235	66228251	66228370	52	18	13	0	0	3	1	30	0	0	98	49	0.617973784	1	1	1	0.684	1	1	-0.105
A5SS	2718 POLD4	POLD4	chr11	-	67353302	67353596	67353363	67353596	67352987	67353077	17	14	3	0	1	2	1	2	0	0	98	49	0.618730112	1	1	0.875	0.429	1	1	-0.232
A3SS	2904 TYMP	TYMP	chr22	-	50529495	50529731	50529495	50529719	50529903	50530085	17	12	5	4	4	5	1	6	0	13	61	49	0.61890226	1	0.773	0.707	0.445	1	0.27	0.007
SE	923 KAT6A	KAT6A	chr8	-	41942792	41943000	41940841	41941444	41943747	41943979	50	26	16	3	0	0	3	9	0	1	98	49	0.61992813	1	0.893	1	1	1	0.818	0.055
A3SS	2190 FCGR2A	FCGR2A	chr1	+	161506333	161506591	161506336	161506591	161505986	161506007	99	41	17	88	27	14	1	20	0	17	52	49	0.62141272	1	0.515	0.589	0.534	1	0.526	-0.217
SE	14085 MAP4K4	MAP4K4	chr2	+	101871493	101871685	101870294	101870415	101873646	101873764	23	5	4	12	6	6	0	7	1	4	98	49	0.622463836	1	0.489	0.294	0.25	0	0.467	0.111
A5SS	1478 IFI6	IFI6	chr1	-	27669232	27669346	27669244	27669346	27668457	27668535	0	2	0	6	10	7	0	2	1	20	61	49	0.62257771	1	0	0.138	0	0	0.074	0.009
SE	240 DDX3X	DDX3X	chrX	+	41343736	41343822	41343215	41343351	41344029	41344128	233	80	33	10	0	0	4	36	2	0	98	49	0.623534899	1	0.921	1	1	0.5	1	0.224
SE	12825 GMIP	GMIP	chr19	-	19640295	19640360	19640084	19640192	19640445	19640571	161	65	30	4	1	0	9	25	0	2	98	49	0.624364766	1	0.953	0.97	1	1	0.862	0.043
SE	9199 ACSL1	ACSL1	chr4	-	184770254	184770332	184768315	184768450	184773080	184773154	18	3	6	1	2	0	2	3	0	1	98	49	0.625913977	1	0.9	0.429	1	1	0.6	-0.024
SE	11778 FMNL1	FMNL1	chr17	+	45246504	45246604	45246209	45246330	45246866	45247316	2	2	0	58	31	6	1	0	1	15	98	49	0.626297292	1	0.017	0.031	0	0.333	0	-0.151
SE	9386 AMICA1	AMICA1	chr11	-	118214823	118214886	118212406	118212561	118224940	118225094	229	73	31	30	15	6	7	17	1	5	98	49	0.626361724	1	0.792	0.709	0.721	0.778	0.63	0.037
SE	457 MEGF9	MEGF9	chr9	-	120607740	120608010	120600917	120605641	120612395	120612539	152	50	27	1	3	0	4	25	0	0	98	49	0.628685621	1	0.987	0.893	1	1	1	-0.04
SE	7427 CD300A	CD300A	chr17	+	74481288	74481326	74477435	74477530	74484000	74484798	61	21	22	0	0	0	2	17	0	2	87	49	0.630034787	1	1	1	1	1	0.827	0.087
SE	15607 HIPK3	HIPK3	chr11	+	33348165	33348228	33347851	33348013	33348521	33348818	24	8	4	15	2	2	0	7	1	1	98	49	0.632723622	1	0.444	0.667	0.5	0	0.778	0.148
RI	441 SHISA5	SHISA5	chr3	-	48467797	48469573	48467797	48469186	48469360	48469573	4	1	2	31	7	10	3	1	5	13	98	49	0.634532702	1	0.061	0.067	0.091	0.231	0.037	-0.061
A3SS	3511 INPP5D	INPP5D	chr2	+	233125744	233125919	233125747	233125919	233122106	233122257	8	2	8	15	8	0	0	7	1	5	52	49	0.634793545	1	0.334	0.191	1	0	0.569	0.224
SE	11506 CPD	CPD	chr17	+	30438974	30439077	30431771	30431881	30442307	30442450	106	39	36	2	2	0	1	10	0	2	98	49	0.636049511	1	0.964	0.907	1	1	0.714	0.1
SE	1596 SOD2	SOD2	chr6	-	159688125	159688242	159684853	159685033	159692660	159692863	781	510	412	10	9	2	7	82	2	0	98	49	0.639136682	1	0.975	0.966	0.99	0.636	1	0.159
SE	12976 GMFG	GMFG	chr19	-	39335260	39335310	39333076	39333126	39335434	39335531	157	104	81	4	0	0	1	18	0	1	98	49	0.639414084	1	0.952	1	1	1	0.9	0.034
SE	4304 OAZ1	OAZ1	chr19	+	2271384	2271530	2269485	2269744	2271781	2271954	353	218	283	6	1	0	18	139	0	0	98	49	0.644652121	1	0.967	0.991	1	1	1	-0.014
SE	6125 NUMB	NUMB	chr14	-	73316389	73316422	73297210	73297285	73323129	73323204	4	0	0	35	28	6	0	2	3	8	82	49	0.644661198	1	0.064	0	0	0	0.13	-0.044
SE	4325 GTPBP1	GTPBP1	chr22	+	38727212	38727348	38726257	38726440	38727982	38728161	44	22	10	3	0	0	1	10	0	0	98	49	0.646483805	1	0.88	1	1	1	1	-0.04
SE	8390 FRY	FRY	chr13	+	32175545	32175632	32173366	32173549	32178176	32178436	62	14	4	0	0	0	2	7	0	2	98	49	0.646484019	1	1	1	1	1	0.636	0.182
SE	16511 HPCAL1	HPCAL1	chr2	+	10408561	10408734	10396834	10396920	10419733	10420135	0	0	4	3	3	5	0	2	2	7	98	49	0.647971128	1	0	0	0.286	0	0.125	0.033
SE	2734 RHOT1	RHOT1	chr17	+	32172936	32173060	32171042	32171101	32173830	32173912	16	2	18	0	0	3	5	3	0	0	98	49	0.648168093	1	1	1	0.75	1	1	-0.083
SE	16237 BST1	BST1	chr4	+	15715284	15715361	15711806	15711889	15715706	15715799	51	25	24	3	0	0	2	33	0	0	98	49	0.65116662	1	0.895	1	1	1	1	-0.035
SE	3274 USP4	USP4	chr3	-	49294406	49294598	49292509	49292598	49297869	49297964	19	7	13	0	0	0	1	7	0	2	98	49	0.652443727	1	1	1	1	1	0.636	0.182
SE	1206 NAMPT	NAMPT	chr7	-	106254363	106254504	106253016	106253151	106261587	106261707	1557	784	321	12	6	0	24	228	0	4	98	49	0.655738702	1	0.985	0.985	1	1	0.966	0.007
SE	5064 ARAP1	ARAP1	chr11	-	72711020	72711141	72710384	72710587	72711429	72711499	117	42	33	3	3	0	4	21	0	2	98	49	0.656734237	1	0.951	0.875	1	1	0.84	0.022
SE	6965 HCLS1	HCLS1	chr3	-	121637145	121637256	121636433	121636489	121642926	121642981	63	48	55	1	2	2	0	9	1	0	98	49	0.657047606	1	0.969	0.923	0.932	0	1	0.441
SE	12686 SLC38A2	SLC38A2	chr12	-	46370511	46370627	46367266	46367340	46370775	46370857	8	3	3	2	1	0	2	6	0	1	98	49	0.65813555	1	0.667	0.6	1	1	0.75	-0.119
SE	14005 SRSF3	SRSF3	chr6	+	36599820	36600276	36598848	36598983	36601151	36601190	18	4	7	16	2	0	1	11	0	4	98	49	0.658575842	1	0.36	0.5	1	1	0.579	-0.169
SE	3019 XPO6	XPO6	chr16	-	28104545	28104707	28101846	28101945	28106042	28106214	425	164	72	0	0	0	7	58	2	0	98	49	0.659208366	1	1	1	1	0.636	1	0.182
A5SS	327 MTCH1	MTCH1	chr6	-	36972651	36972796	36972702	36972796	36970646	36970694	33	34	13	24	16	7	2	9	0	8	98	49	0.661988709	1	0.407	0.515	0.481	1	0.36	-0.212
SE	2554 RTFDC1	RTFDC1	chr20	+	56472325	56472415	56468584	56468766	56473300	56473395	4	0	0	21	14	4	0	0	2	1	98	49	0.662218871	1	0.087	0	0	0	0	0.029
SE	10104 PICALM	PICALM	chr11	-	86026291	86026367	86014869	86014963	86031468	86031611	144	66	56	4	0	1	1	14	0	0	98	49	0.664770716	1	0.947	1	0.966	1	1	-0.029
SE	235 DDX3X	DDX3X	chrX	+	41337407	41337465	41333307	41334297	41341483	41341616	84	33	3	5	0	0	2	10	0	2	98	49	0.666543847	1	0.894	1	1	1	0.714	0.108
A3SS	1957 ADAM8	ADAM8	chr10	-	133273253	133273443	133273253	133273430	133273761	133273838	43	23	4	4	0	1	6	11	0	2	62	49	0.666688927	1	0.895	1	0.76	1	0.813	-0.021
SE	1587 IGF2R	IGF2R	chr6	+	160064803	160064901	160064400	160064531	160068248	160068385	112	38	21	6	5	0	2	17	0	3	98	49	0.671419462	1	0.903	0.792	1	1	0.739	0.029
SE	4832 CLEC7A	CLEC7A	chr12	-	10125086	10125173	10116780	10118590	10125296	10125448	26	2	10	12	2	1	3	18	0	8	98	49	0.675221418	1	0.52	0.333	0.833	1	0.529	-0.203
A5SS	274 GLIPR2	GLIPR2	chr9	+	36147785	36147916	36147785	36147894	36148546	36148650	9	1	0	20	9	9	0	3	2	3	71	49	0.675450629	1	0.237	0.071	0	0	0.408	-0.101
SE	12958 PSMD8	PSMD8	chr19	+	38379239	38379405	38376351	38376454	38380898	38380999	15	9	3	2	1	0	1	3	0	0	98	49	0.675480788	1	0.789	0.818	1	1	1	-0.131
SE	5040 APLP2	APLP2	chr11	+	130137255	130137291	130135562	130135715	130140397	130140483	28	8	7	27	9	10	1	13	1	11	85	49	0.680652741	1	0.374	0.339	0.288	0.366	0.405	-0.052
SE	14046 TMEM127	TMEM127	chr2	-	96265137	96265489	96254832	96254997	96265868	96266013	26	12	10	0	3	0	3	13	0	1	98	49	0.680667227	1	1	0.667	1	1	0.867	-0.045
SE	3197 CCNDBP1	CCNDBP1	chr15	+	43194414	43194461	43192742	43192803	43194726	43197177	86	68	25	0	4	0	1	28	0	0	96	49	0.681207238	1	1	0.897	1	1	1	-0.034
SE	14793 PHF1	PHF1	chr6	+	33414965	33415144	33414724	33414829	33415234	33415329	3	7	1	2	0	0	1	1	0	0	98	49	0.683136067	1	0.429	1	1	1	1	-0.19

SE	6430	LMBR1L	LMBR1L	chr12	-	49104447	49104551	49103686	49103813	49104745	49104885	9	3	2	0	2	0	1	2	0	0	98	49	0.683158228	1	1	0.429	1	1	1	-0.19
SE	7519	MKL1	MKL1	chr22	-	40424205	40424381	40423535	40423685	40429605	40429767	12	3	1	0	2	0	2	2	0	0	98	49	0.683169487	1	1	0.429	1	1	1	-0.19
SE	9962	HDAC7	HDAC7	chr12	-	47796206	47796298	47795586	47795767	47797016	47797142	15	3	2	0	2	0	1	1	0	0	98	49	0.683194702	1	1	0.429	1	1	1	-0.19
SE	2629	NLRC5	NLRC5	chr16	+	57046551	57046641	57045447	57045492	57047544	57047628	23	19	1	3	0	0	1	10	0	0	98	49	0.687431362	1	0.793	1	1	1	1	-0.069
SE	3026	XPO6	XPO6	chr16	-	28135215	28135324	28132333	28132403	28146093	28146203	125	73	21	2	1	0	1	25	0	0	98	49	0.688505845	1	0.969	0.973	1	1	1	-0.019
SE	12874	ZFAS1	ZFAS1	chr20	+	49280484	49280570	49279103	49279208	49289044	49289258	54	41	23	3	1	0	1	10	0	0	98	49	0.688940427	1	0.9	0.953	1	1	1	-0.049
A3SS	2605	LILRB2	LILRB2	chr19	-	54277549	54277600	54277549	54277597	54277888	54277939	24	5	2	11	3	0	1	2	0	6	52	49	0.69041571	1	0.673	0.611	1	1	0.239	0.142
RI	460	CSF3R	CSF3R	chr1	-	36466042	36466827	36466042	36466200	36466620	36466827	826	345	179	10	1	0	19	165	0	1	98	49	0.692461527	1	0.976	0.994	1	1	0.988	-0.004
SE	3651	ERO1A	ERO1A	chr14	-	52683787	52683907	52682324	52682408	52695367	52695931	39	20	6	3	0	0	1	2	0	0	98	49	0.694855347	1	0.867	1	1	1	1	-0.044
A3SS	241	LRCH4	LRCH4	chr7	-	100574010	100575304	100574010	100574356	100575704	100575782	12	5	3	19	3	7	1	2	0	4	98	49	0.69501919	1	0.24	0.455	0.176	1	0.2	-0.31
SE	7089	MEGF6	MEGF6	chr1	-	3496987	3497119	3496654	3496783	3497232	3497361	6	3	1	8	4	0	3	0	0	2	98	49	0.696658222	1	0.273	0.273	1	1	0	0.015
A5SS	1109	SERPINA1	SERPINA1	chr14	-	94390419	94390692	94390456	94390692	94388559	94388663	84	62	16	28	18	5	4	5	0	4	86	49	0.696859738	1	0.631	0.662	0.646	1	0.416	-0.062
SE	1945	CCND3	CCND3	chr6	-	41936558	41936695	41934932	41936107	41937234	41937394	26	8	17	1	2	0	3	19	2	0	98	49	0.696975016	1	0.929	0.667	1	0.429	1	0.151
SE	2180	PRRC2A	PRRC2A	chr6	+	31626771	31626862	31626019	31626162	31626981	31627198	33	12	2	3	0	0	1	5	0	0	98	49	0.697530193	1	0.846	1	1	1	1	-0.051
SE	2603	CYTH4	CYTH4	chr22	+	37310987	37311064	37309211	37309323	37311455	37311527	172	85	41	0	0	4	2	30	0	0	98	49	0.698071195	1	1	1	0.837	1	1	-0.054
RI	801	ARHGDIA	ARHGDIA	chr17	-	81869329	81869625	81869329	81869406	81869541	81869625	38	40	6	23	8	3	5	15	0	13	98	49	0.698940977	1	0.452	0.714	0.5	1	0.366	-0.128
SE	6126	NUMB	NUMB	chr14	-	73366896	73366981	73355625	73355766	73409936	73410068	74	37	18	2	0	2	1	10	0	1	98	49	0.70020625	1	0.949	1	0.818	1	0.833	0.006
SE	13659	CLK1	CLK1	chr2	-	200854615	200854695	200853902	200853993	200855003	200855086	42	36	20	0	0	0	1	8	0	2	98	49	0.700781312	1	1	1	1	1	0.667	0.166
SE	2871	NLRP12	NLRP12	chr19	-	53807494	53807668	53805279	53805450	53809586	53811288	20	9	11	0	0	0	1	8	0	2	98	49	0.701325341	1	1	1	1	1	0.667	0.166
SE	12702	GLUL	GLUL	chr1	-	182390496	182390852	182388571	182388750	182391678	182392206	20	4	4	397	115	12	2	1	5	18	98	49	0.701769816	1	0.025	0.017	0.143	0.167	0.027	-0.035
SE	5206	PTPRJ	PTPRJ	chr11	+	48112746	48112983	48110057	48110076	48121002	48121266	111	70	34	0	1	0	11	32	2	0	98	49	0.701904035	1	1	0.972	1	0.733	1	0.124
SE	2767	PCBP2	PCBP2	chr12	+	53467804	53467843	53467220	53467293	53468779	53468832	50	22	5	3	1	0	1	4	0	2	88	49	0.70273878	1	0.903	0.925	1	1	0.527	0.179
SE	9208	ACSL1	ACSL1	chr4	-	184803319	184803546	184788616	184788731	184825915	184826114	417	162	76	5	2	0	4	91	0	0	98	49	0.70655971	1	0.977	0.976	1	1	1	-0.016
A3SS	2717	ABTB1	ABTB1	chr3	+	127674344	127674418	127674390	127674418	127672937	127673081	13	22	4	35	12	8	0	8	3	0	95	49	0.707794314	1	0.161	0.486	0.205	0	1	-0.216
SE	12883	GRAMD1A	GRAMD1A	chr19	+	35013255	35013368	35011473	35011554	35013540	35013691	28	7	12	9	3	0	1	5	0	4	98	49	0.710171334	1	0.609	0.538	1	1	0.385	0.023
SE	4005	PYCARD	PYCARD	chr16	-	31202146	31202203	31201485	31201841	31202416	31202776	17	20	30	3	0	0	2	9	0	0	98	49	0.711092721	1	0.739	1	1	1	1	-0.087
SE	16704	MICAL1	MICAL1	chr6	-	109447874	109447963	109447680	109447722	109448202	109448393	21	6	9	3	0	0	2	9	0	0	98	49	0.712273023	1	0.778	1	1	1	1	-0.074
A3SS	3844	ARHGAP25	ARHGAP25	chr2	+	68807272	68807480	68807275	68807480	68787839	68787956	49	22	16	3	0	1	0	5	1	0	52	49	0.713208598	1	0.939	1	0.938	0	1	0.459
SE	12198	ARAP3	ARAP3	chr5	-	141655361	141655400	141653400	141654435	141655620	141655758	3	0	1	13	4	9	0	0	1	2	88	49	0.713267633	1	0.114	0	0.058	0	0	0.057
SE	8344	CASP8	CASP8	chr2	+	201274888	201274953	201272897	201272942	201276826	201276968	160	55	21	11	0	0	1	6	0	2	98	49	0.714017103	1	0.879	1	1	1	0.6	0.16
RI	506	RAB24	RAB24	chr5	-	177303171	177303744	177303171	177303320	177303406	177303744	23	19	8	7	1	2	6	10	2	1	98	49	0.714087862	1	0.622	0.905	0.667	0.6	0.833	0.015
SE	3730	ARID5A	ARID5A	chr2	+	96549320	96549459	96547401	96547517	96549752	96549805	12	3	4	8	8	0	2	2	0	3	98	49	0.715441443	1	0.429	0.158	1	1	0.25	-0.096
SE	13149	FCGRT	FCGRT	chr19	+	49513386	49513473	49512278	49512750	49513881	49514133	99	37	27	0	1	3	9	14	0	0	98	49	0.717316717	1	1	0.949	0.818	1	1	-0.078
SE	5532	NDE1	NDE1	chr16	+	15696708	15696860	15694164	15694256	15724190	15726351	21	28	5	3	0	0	1	4	0	0	98	49	0.717769788	1	0.778	1	1	1	1	-0.074
SE	3285	RNASET2	RNASET2	chr6	-	166934090	166934136	166931043	166931118	166938894	166939008	231	74	117	2	4	0	3	45	0	0	95	49	0.719143808	1	0.983	0.905	1	1	1	-0.037
A3SS	1695	CSF3R	CSF3R	chr1	-	36466042	36466908	36466042	36466827	36467229	36467311	99	28	16	479	165	105	3	22	17	96	98	49	0.71928138	1	0.094	0.078	0.071	0.081	0.103	-0.011
A3SS	3626	RPS3	RPS3	chr11	+	75405582	75406913	75405613	75406913	75404671	75404868	39	8	8	61	21	21	0	4	1	4	80	49	0.719477917	1	0.281	0.189	0.189	0	0.38	0.03
SE	13854	GNAS	GNAS	chr20	+	58898940	58898985	58895611	58895684	58903527	58903585	10	4	3	28	12	5	1	2	3	2	94	49	0.72037482	1	0.157	0.148	0.238	0.148	0.343	-0.065
SE	12234	SMCHD1	SMCHD1	chr18	+	2666156	2666232	2655886	2656261	2666869	2667031	415	229	66	4	0	3	20	98	0	0	98	49	0.720453135	1	0.981	1	0.917	1	1	-0.034
SE	12238	SMCHD1	SMCHD1	chr18	+	2703691	2703886	2700734	2700918	2705693	2705807	514	273	65	0	0	5	4	139	0	0	98	49	0.720467692	1	1	1	0.867	1	1	-0.044
SE	6636	CSF3R	CSF3R	chr1	-	36467821	36467962	36467557	36467651	36468074	36468221	634	178	131	4	3	0	15	149	0	0	98	49	0.721936982	1	0.988	0.967	1	1	1	-0.015
SE	9241	SVIL	SVIL	chr10	-	29531253	29531288	29530606	29530668	29550596	29551263	30	14	2	3	0	0	2	4	0	0	84	49	0.722024346	1	0.854	1	1	1	1	-0.049
SE	15940	CSF2RA	CSF2RA	chrX	+	1285777	1285920	1282677	1282779	1288518	1288642	56	49	23	2	0	2	1	5	0	0	98	49	0.724133416	1	0.933	1	0.852	1	1	-0.072
SE	8918	ATP6V0B	ATP6V0B	chr1	+	43975799	43975848	43974647	43975107	43976089	43976173	74	62	36	20	5	5	5	19	1	4	98	49	0.728043554	1	0.649	0.861	0.783	0.714	0.704	0.055
SE	5412	NCF2	NCF2	chr1	-	183567203	183567345	183566919	183566988	183569141	183569185	164	124	119	6	4	0	1	39	0	2	98	49	0.730433554	1	0.932	0.939	1	1	0.907	0.003
SE	970	DOCK5	DOCK5	chr8	+	25300575	25300657	25298943	25299101	25302324	25302454	135	60	13	0	4	0	3	24	0	0	98	49	0.730535785	1	1	0.882	1	1	1	-0.039
SE	13949	DYSF	DYSF	chr2	+	71549349	71549391	71539156	71539239	71551040	71551156	10	11	1	38	16	10	2	2	1	11	91	49	0.731283912	1	0.124	0.27	0.051	0.519	0.089	-0.156
SE	4341	ITPK1	ITPK1	chr14	-	92993879	92993997	92962750	92962849	93016675	93016801	54	46	22	1	0	0	2	10	0	2	98	49	0.731604336	1	0.964	1	1	1	0.714	0.131
SE	16958	H2AFY	H2AFY	chr5	-	135388921	135389126	135370035	135370142	135399061	135399238	38	12	15	3	0	0	2	8	0	0	98	49	0.732088065	1	0.864	1	1	1	1	-0.045
SE	5243	PFKFB3	PFKFB3	chr10	+	6216124	6216191	6215220	6215317	6216705	6216780	157	110	11	3	2	0	19	68	1	1	98	49	0.733268371	1	0.963	0.965	1	0.905	0.971	0.038
SE	6533	RBM39	RBM39	chr20	-	35713018	35713096	35709223	35709256	35714184	35714389	71	48	21	1	3	0	4	24	0	0	98	49	0.733711812	1	0.973	0.889	1	1	1	-0.046
A3SS	3083	RFXANK	RFXANK	chr19	+	19196962	19197046	19196965	19197046	19193938	19194133	2	1	0	6	4	2	0	1	1	1	52	49	0.734057817	1	0.239	0.191	0	0	0.485	-0.099
SE	16459	TMC6	TMC6	chr17	-	78124888	78124985	78124523	78124781	78125157	78125263	14	10	6	2	1	0	4	2	1	0	98	49	0.734348789	1	0.778	0.833	1	0.667	1	0.037
SE	523	SEMA4D	SEMA4D	chr9	-	89388635	89388792	89387385	89387608	89388871	89389047	58	18	32	0	3	0	4	14	0	0	98	49	0.738125132	1	1	0.75	1	1	1	-0.083
SE	3660	SOS2	SOS2	chr14	-	50158564	50158646	50156998	50157121	50159430	50160086	91	38	10	3	0	0	1	14	0	2	98	49	0.738538674	1	0.938	1	1	1	0.778	0.09
SE	16425	MANBA	MANBA	chr4	-	102714437	102714561	102690595	102690771	102722870	102723041	15	10	11	1	2	0	1	4	0	0	98	49	0.742914923	1	0.882	0.714	1	1	1	-0.135
A3SS	693	PRKCB	PRKCB	chr16	+	24214657	24220611	24219960	24220611	24191089	24191230	37	23	9	1	0	2	2	14	0	0	98	49	0.747697883	1	0.949	1	0.692	1	1	-0.12
SE	11424	ANKHD1	ANKHD1	chr5	+	140496519	140497278	140486957	140487060	140504820	140504966	20	7	2	2	3	0	2	6	0	3	98	49	0.747961443	1	0.833	0.538	1	1	0.5	0.04
SE	8606	TKT	TKT	chr3	-	53237796	53237820	53234982	53235174	53240250	53240348	5	0	11	75	47	49	2	1	1	33	73	49	0.748430679	1	0.043	0	0.131	0.573	0.02	-0.238
A3SS	937	FCGR3A	FCGR3A	chr1	-	161548420	161548678	161548420	161548675	161549010	161549031	2	9	10	0	0	0	1	5	0	2	52	49	0.749944571	1	1	1	1	1	0.702	0.149
SE	15835	AOAH	AOAH	chr7	-	36621709	36621780	36620780	36620829	36623189	36623250	55	30	4	0	3	0	4	25	0	0	98	49	0.750059818	1	1	0.833	1	1	1	-0.056
SE	4158	NLRP1	NLRP1	chr17	-	5520880	5521012	5517745	5517887	5521523	5521786	42	6	5	8	4	0	1	10	0	3	98	49	0.75216073	1	0.724	0.429	1	1	0.625	-0.095
SE	1131	MKLN1	MKLN1	chr7	+	131445773	131445903	131443480	131443702	131463216	131463364	34	17	1	1	2	0	1	9	1	0	98	49	0.754100352	1	0.944	0.81	1	0.333	1	0.252
A3SS	1384	RASGRP4	RASGRP4	chr19	-	38419859	38420013	38419859	38419971	38420130	38420262	54	25	21	0	0	3	5	20	0	0	91	49	0.75562353	1	1	1	0.79	1	1	-0.07
A3SS	2074	ITGAM	ITGAM	chr16	+	31297741	31297954	31297744	31297954	31297513	31297654	7	1	1	11	9	2	2	2	1	7	52	49	0.758089344	1	0.375	0.095	0.32	0.653	0.212	-0.169
SE	684	DOCK8	DOCK8	chr9	+	386330	386426	382512	382685	390470	390566	93	23	16	2	2	0	4	13	0	0	98	49	0.759194701	1	0.959	0.852	1	1	1	-0.063
SE	15578	SUN2	SUN2	chr22	-	38754641	38754706	38752506	38752665	38755762	38756019	13	5	3	14	1	3	0	7	1	2	98	49	0.760050741	1	0.317	0.714	0.333	0	0.636	0.137
SE	5254	PTPRC	PTPRC	chr1	+	198702386	198702530	198699563	198699704	198703297	198703372	16	0	8	35	24	9	0	4	3	6	98	49	0.762041288	1	0.186	0	0.308	0	0.25	0.04
SE	2792	USP25	USP25	chr21	+	15824038	15824166	15818697	15818846	15824965	15825061	22	16	1	3	0	0	1	3	0	2	98	49	0.762601432	1	0.786	1	1	1	0.429	0.214
SE	13744	NBN	NBN	chr8	-	89978219	89978323	89971172	89971290	89980733	89980893	34	16	5	3	0	0	2	17	0	0	98	49	0.763162175	1	0.85	1	1	1	1	-0.05
SE	2804	UBN1	UBN1	chr16	+	4868832	4868903	4860663	4861102	4870211	4870341	30	33	10	3	0	0	0	4	1	0	98	49	0.764528525	1	0.833	1	1	0	1	0.444
A3SS	3648	ITGB2	ITGB2	chr21	-	44910283	44910415	44910283	44910372	44910724	44910785	10	2	3	92	29	19	1	3	1	16	92	49	0.765684671	1	0.055	0.035	0.078	0.348	0.091	-0.163
SE	2761	CPPED1	CPPED1	chr16	-	12704623	12705049	12659798	12665115	12781184	12781403	87	37	15	1	1	4	1	12	0	3	98	49	0.766803703	1	0.978	0.949	0.652	1	0.667	0.026
A5SS	2776	ABCG1	ABCG1	chr21	+	42287888	42288073	42287888	42288037	42288210	42288312	21	10	4	2	2	0	2	3	0	2	85	49	0.767335084	1	0.858	0.742	1	1	0.464	0.135
SE	14190	ACOX1	ACOX1	chr17	-	75973624	75973785	75960214	75960375	75978533	75978693	10	3	1	16	3	10	0	3	1	2	98	49	0.767698723	1	0.238	0.333	0.048	0	0.429	-0.008
SE	12161	TYMP	TYMP	chr22	-	50528511	50528610	50527587	50527717	50529135	50529338	73	24	32	0	0	3	1	41	0	0	98	49	0.768765459	1	1	1	0.842	1	1	-0.053
SE	7501	SLC12A6	SLC12A6	chr15	-	34257641	34257788	34256228	34256283	34258812	34258944	64	22	9	10	11	3	1	11	0	4	98	49	0.771324392	1	0.762	0.5	0.6	1	0.579	-0.169
A3SS	1471	LILRB3	LILRB3	chr19	-	54217318	54217477	54217318	54217474	54218360	54218413	15	12	4	44	15	2	2	8	3	11	52	49	0.772420962	1	0.243	0.43	0.653	0.386	0.407	0.046
A3SS	1670	RBM39	RBM39	chr20	-	35709223	35709274	35709223	35709256	35713018	35713096	38	12	8	24	4	4	0	7	1	1	67	49	0.773059786	1	0.537	0.687	0.594	0	0.837	0.188
SE	5059	ARAP1	ARAP1	chr11	-	72695717	72695865	72695541	72695628	72696548	72696654	95	77	31	0	3	0	1	24	0	0	98	49	0.778635347	1	1	0.928	1	1	1	-0.024
SE	9620	GSK3B	GSK3B	chr3	-	119912703	119912810	119905754	119905852	119916043	119916174	40	5	10	1	2	0	2	8	0	2	98	49	0.780210798	1	0.952	0.556	1	1	0.667	0.002
SE	11293	MBD1	MBD1	chr18	-	50273333	50273471	50272823	50272955	50273563	50273860	4	4	2	2	0	0	1	1	0	1	98	49	0.780381124	1	0.5	1	1	1	0.333	0.167
SE	5415	NCF2	NCF2	chr1	-	183573184	183573292	183570779	183570839	183577598	183577707	304	147	80	3	2	0	5	53	0	1	98	49	0.783922926	1	0.981	0.974	1	1	0.964	0.003
SE	10100	PICALM	PICALM	chr11	-	85990249	85990399	85983865	85983973	85996825	85996929	104	59	9	71	25	20	1	25	0	21	98	49	0.784680554	1	0.423	0.541	0.184	1	0.373	-0.304
SE	7840	LRRK2	LRRK2	chr12	+	40232273	40232383	40225554	40225640	40235625	40235714	338	169	65	0	0	4	6	38	0	0	98	49	0.785217176	1	1	1	0.89	1	1	-0.037
SE	15545	ATG16L2	ATG16L2	chr11	+	72826517	72826589	72826172	72826243	72826702	72826823	222	145	48	1	3	0	12	63	0	0	98	49	0.785871628	1	0.991	0.96	1	1	1	-0.016
SE	11053	THEMIS2	THEMIS2	chr1	+	27879643	27880054	27876587	27876728	27885294	27885451	111	23	15	3	2	6	6	25	3	1	98	49	0.787595271	1	0.949	0.852	0.556	0.5	0.926	0.073
SE	9009	HLA-B	HLA-B	chr6	-	31355106	31355223	31354632	31354665	31355316	31355592	2250	754	621	31	2	9	46	261	2	4	98	49	0.793423683	1	0.973	0.995	0.972	0.92	0.97	0.035
A3SS	2818	STAT3	STAT3	chr17	-	42316788	42316901	42316788	42316851	42317181	42317224	101	42	24	13	0	8	2	12	0	4	98	49	0.79351202	1	0.795	1	0.6	1	0.6	-0.002
SE	9855	NADK	NADK	chr1	-	1754050	1754208	1753566	1753649	1754283	1754383	84	54	60	4	0	0	4	21	0	2	98	49	0.803552896	1	0.913	1	1	1	0.84	0.051
SE	14775	PLAUR	PLAUR	chr19	-	43652224	43652371	43648570	43649143	43655438	43655573	60	16	17	10	2	1	2	46	0	7	98	49	0.805341856	1	0.75	0.8	0.895	1	0.767	-0.068
SE	2870	NLRP12	NLRP12	chr19	-	53807494	53807665	53805279	53805450	53809586	53811288	37	15	17	0	0	0	1	13	0	2	98	49	0.807194084	1	1	1	1	1	0.765	0.117
A3SS	753	ISG20	ISG20	chr15	+	88652109	88652310	88652169	88652310	88639342	88639594	60	67	37	1	1	1	1	28	0	0	98	49	0.807205421	1	0.968	0.971	0.949	1	1	-0.037
SE	3709	IRF9	IRF9	chr14	+	24164613	24164955	24164062	24164134	24165846	24165962	41	31	26	3	0	0	2	13	0	0	98	49	0.810580629	1	0.872	1	1	1	1	-0.043
A3SS	521	BAG6	BAG6	chr6	-	31647590	31647844	31647590	31647826	31648676	31648751	1	2	0	10	5	1	0	0	1	2	67	49	0.813743282	1	0.068	0.226	0	0	0	0.098
SE	9198	ACSL1	ACSL1	chr4	-	184765890	184765986	184764852	184764925	184766621	184766756	240	107	64	5	2	2	2	59	0	3	98	49	0.815627037	1	0.96	0.964	0.941	1	0.908	0.001
A3SS	2810	FKBP8	FKBP8	chr19	-	18538215	18538436	18538215	18538433	18539370	18539459	42	29	29	38	13	14	2	2	0	5	52	49	0.817161819	1	0.51	0.678	0.661	1	0.274	-0.021
A3SS	2397	DBNL	DBNL	chr7	+	44058428	44058480	44058431	44058480	44058128	44058280	11	6	7	21	12	7	0	6	3	2	52	49	0.818390764	1	0.33	0.32	0.485	0	0.739	0.009
SE	266	GK	GK	chrX	+	30665510	30665584	30653358	30653615	30677374	30677452	21	23	15	1	0	2	1	4	0	1	98	49	0.819518177	1	0.913	1	0.789	1	0.667	0.067
SE	13300	RPS9	RPS9	chr19	+	54206275	54206462	54201486	54201609	54207397	54207647	149	40	46	1	3	0	3	56	0	1	98	49	0.824162941	1	0.987	0.87	1	1	0.966	-0.031
SE	9572	GNAI2	GNAI2	chr3	+	50253023	50253184	50252396	50252538	50256191	50256320	199	126	93	2	0	4	6	43	0	0	98	49	0.825744	1	0.98	1	0.921	1	1	-0.033
SE	10099	PICALM	PICALM	chr11	-	85990249	85990378	85983865	85983973	85996825	85996929	78	43	4	71	25	20	1	18	0	21	98	49	0.825899785	1	0.355	0.462	0.091	1	0.3	-0.347
SE	1589	IGF2R	IGF2R	chr6	+	160079579	160079787	160078200	160078362	160080128	160080275	264	84	44	4	0	1	4	40	0	1	98	49	0.826922372	1	0.971	1	0.957	1	0.952	0
RI	1017	POFUT2	POFUT2	chr21	-	45263927	45267713	45263927	45265635	45267589	45267713	6	7	0	5	3	1	0	3	1	0	98	49	0.827034304	1	0.375	0.538	0	0	1	-0.196
SE	12784	MYO9B	MYO9B	chr19	+	17156908	17157038	17154314	17154415	17159394	17159484	95	46	13	0	3	0	6	18	0	0	98	49	0.827566797	1	1	0.885	1	1	1	-0.038
SE	10738	MYO1F	MYO1F	chr19	-	8541905	8541991	8539946	8540028	8544296	8544464	163	108	78	2	9	0	11	31	0	3	98	49	0.83190545	1	0.976	0.857	1	1	0.838	0.025
SE	7211	NBEAL2	NBEAL2	chr3	+	47008069	47008186	47007815	47007910	47008282	47008441	97	42	14	0	3	0	9	40	0	0	98	49	0.832076213	1	1	0.875	1	1	1	-0.042
SE	1410	IKZF1	IKZF1	chr7	+	50387344	50387470	50382539	50382707	50391728	50391833	29	5	9	3	0	0	2	10	0	1	98	49	0.832726723	1	0.829	1	1	1	0.833	0.026
SE	16143	KDM5C	KDM5C	chrX	-	53218275	53218398	53217795	53217966	53220838	53220916	20	13	3	0	2	0	1	12	0	2	98	49	0.833157475	1	1	0.765	1	1	0.75	0.047
A3SS	3148	TMEM91	TMEM91	chr19	+	41383560	41384082	41383862	41384082	41382771	41382921	6	5	2	1	4	10	1	3	0	3	98	49	0.834081594	1	0.75	0.385	0.091	1	0.333	-0.258
SE	239	DDX3X	DDX3X	chrX	+	41343736	41343822	41342736	41342836	41344029	41344128	166	36	32	0	3	0	2	21	0	0	98	49	0.834518929	1	1	0.857	1	1	1	-0.048
SE	16805	ZFAND5	ZFAND5	chr9	-	72363469	72363606	72360627	72360787	72364695	72365247	17	4	1	10	5	9	0	4	1	0	98	49	0.835172265	1	0.459	0.286	0.053	0	1	-0.234
SE	12241	SMCHD1	SMCHD1	chr18	+	2722518	2722663	2718314	2718434	2724898	2724995	190	86	63	23	10	0	1	43	0	5	98	49	0.835663393	1	0.805	0.811	1	1	0.811	-0.033
A3SS	2475	LST1	LST1	chr6	+	31588496	31588909	31588562	31588909	31587199	31587318	69	46	40	35	11	14	3	13	1	6	98	49	0.836902443	1	0.496	0.676	0.588	0.6	0.52	0.027
SE	3024	XPO6	XPO6	chr16	-	28133840	28133933	28132333	28132403	28135215	28135324	329	207	71	4	1	0	6	49	0	0	98	49	0.84163499	1	0.976	0.99	1	1	1	-0.011
SE	16626	RTN4	RTN4	chr2	-	54974694	54974764	54973562	54973621	54982514	54982653	29	15	8	3	0	0	2	5	0	0	98	49	0.84261972	1	0.829	1	1	1	1	-0.057
SE	6519	ACAP2	ACAP2	chr3	-	195392089	195392147	195381902	195382022	195442794	195443088	69	42	25	0	3	0	5	20	0	0	98	49	0.844595526	1	1	0.875	1	1	1	-0.042
A5SS	234	SAMD9L	SAMD9L	chr7	-	93146882	93147146	93147087	93147146	93145384	93146040	18	1	3	3	4	0	4	10	0	6	98	49	0.844753468	1	0.75	0.111	1	1	0.455	-0.107
SE	17083	FAM193B	FAM193B	chr5	-	177531952	177532200	177531281	177531521	177532442	177532641	19	5	2	7	4	0	2	0	0	1	98	49	0.844990272	1	0.576	0.385	1	1	0	0.154
SE	16046	ECE1	ECE1	chr1	-	21255946	21256138	21247220	21247363	21257524	21257590	47	15	9	1	2	0	1	5	0	0	98	49	0.849544904	1	0.959	0.789	1	1	1	-0.084
SE	14665	CDS2	CDS2	chr20	+	5186686	5186839	5185757	5185826	5189066	5189186	34	16	10	3	0	0	3	8	0	0	98	49	0.850069193	1	0.85	1	1	1	1	-0.05
SE	238	DDX3X	DDX3X	chrX	+	41343215	41343351	41342736	41342836	41344029	41344128	147	42	21	0	3	0	2	15	0	0	98	49	0.854951073	1	1	0.875	1	1	1	-0.042
SE	15493	NAIP	NAIP	chr5	-	71011274	71011374	71003775	71003857	71012347	71012918	63	16	5	0	1	2	1	9	0	0	98	49	0.855433741	1	1	0.889	0.556	1	1	-0.185
A5SS	1405	RFWD2	RFWD2	chr1	-	176163814	176163891	176163826	176163891	176162868	176162988	95	45	5	33	11	1	1	17	1	5	61	49	0.856767086	1	0.698	0.767	0.801	0.445	0.732	0.167
SE	2881	SRRM2	SRRM2	chr16	+	2756333	2756606	2752328	2752846	2757471	2757579	30	25	2	3	0	0	0	7	1	0	98	49	0.857146712	1	0.833	1	1	0	1	0.444
SE	2723	PRKCB	PRKCB	chr16	+	24172269	24172361	24154683	24154857	24174517	24174580	105	58	23	1	3	0	9	27	0	1	98	49	0.85735006	1	0.981	0.906	1	1	0.931	-0.003
SE	14489	ARPC2	ARPC2	chr2	+	218245419	218245546	218239390	218239484	218249363	218249464	357	133	74	1	0	4	3	57	0	0	98	49	0.858266198	1	0.994	1	0.902	1	1	-0.035
SE	4306	DUSP6	DUSP6	chr12	-	89350587	89351025	89347824	89349561	89351639	89352859	41	7	7	4	0	2	3	21	2	1	98	49	0.860721438	1	0.837	1	0.636	0.429	0.913	0.153
SE	7308	MAX	MAX	chr14	-	65101545	65101572	65093707	65093815	65102303	65102544	17	5	3	2	3	3	1	2	0	3	76	49	0.861697781	1	0.846	0.518	0.392	1	0.301	-0.065
SE	5224	MX2	MX2	chr21	+	41390564	41390703	41382409	41382564	41395586	41395785	59	36	25	23	16	2	4	23	0	13	98	49	0.863802513	1	0.562	0.529	0.862	1	0.469	-0.083
SE	12966	DDX60L	DDX60L	chr4	-	168441336	168441492	168433009	168433115	168448637	168448779	96	56	4	0	3	0	3	44	0	0	98	49	0.864023371	1	1	0.903	1	1	1	-0.032
SE	10313	JAK1	JAK1	chr1	-	64873369	64873523	64869310	64869474	64879024	64879148	84	27	32	4	0	0	1	13	0	0	98	49	0.867059698	1	0.913	1	1	1	1	-0.029
SE	14738	HCK	HCK	chr20	+	32084390	32084543	32083893	32084043	32086627	32086807	173	75	64	19	6	2	3	55	1	5	98	49	0.868449242	1	0.82	0.862	0.941	0.6	0.846	0.151
A3SS	2398	DBNL	DBNL	chr7	+	44058877	44058983	44058901	44058983	44058428	44058480	3	2	1	42	19	9	0	2	1	3	73	49	0.869747429	1	0.046	0.066	0.069	0	0.309	-0.094
SE	10048	ADD3	ADD3	chr10	+	110122109	110122292	110119465	110119564	110124016	110124274	39	17	5	0	0	2	2	5	0	0	98	49	0.874751865	1	1	1	0.556	1	1	-0.148
SE	12305	FKBP1A	FKBP1A	chr20	-	1375490	1375603	1371711	1372240	1392833	1392881	12	6	5	0	0	2	1	1	0	0	98	49	0.874986718	1	1	1	0.556	1	1	-0.148
SE	7551	RFWD2	RFWD2	chr1	-	176136487	176136547	176135009	176135086	176149005	176149074	156	44	20	29	6	0	1	16	0	4	98	49	0.876427787	1	0.729	0.786	1	1	0.667	0.005
SE	5085	SUCO	SUCO	chr1	+	172557279	172557417	172555868	172556023	172557643	172557794	46	22	16	1	2	0	4	9	0	0	98	49	0.881731767	1	0.958	0.846	1	1	1	-0.065
SE	13947	DYSF	DYSF	chr2	+	71513239	71513332	71511806	71511921	71513715	71513921	53	16	16	1	2	0	1	10	0	1	98	49	0.881863743	1	0.964	0.8	1	1	0.833	0.005
SE	6855	AMPD2	AMPD2	chr1	+	109629795	109629916	109629326	109629490	109630232	109630406	51	15	17	4	14	0	1	7	0	3	98	49	0.883392835	1	0.864	0.349	1	1	0.538	-0.031
SE	4794	HNRNPK	HNRNPK	chr9	-	83973901	83973973	83973285	83973399	83974516	83974589	185	110	61	5	3	4	1	53	0	3	98	49	0.884489462	1	0.949	0.948	0.884	1	0.898	-0.022
SE	5373	RGS2	RGS2	chr1	+	192810369	192810431	192809038	192809181	192810980	192811147	620	414	275	2	5	1	6	89	0	0	98	49	0.885655317	1	0.994	0.976	0.993	1	1	-0.012
SE	8848	CORO1A	CORO1A	chr16	+	30187941	30188087	30187724	30187829	30188191	30188249	327	150	138	1	4	0	18	59	0	0	98	49	0.888014184	1	0.994	0.949	1	1	1	-0.019
RI	505	RAB24	RAB24	chr5	-	177302127	177302496	177302127	177302178	177302396	177302496	66	13	19	10	8	12	3	14	0	12	98	49	0.894120158	1	0.767	0.448	0.442	1	0.368	-0.132
A3SS	2270	YY1AP1	YY1AP1	chr1	-	155668626	155668777	155668626	155668717	155670319	155670464	12	1	2	7	1	2	2	1	0	3	98	49	0.898227086	1	0.462	0.333	0.333	1	0.143	-0.195
SE	12271	PPP4R1	PPP4R1	chr18	-	9549196	9549338	9546790	9547952	9550051	9550186	55	24	29	1	2	0	1	6	0	0	98	49	0.901736809	1	0.965	0.857	1	1	1	-0.059
SE	5178	EIF4A1	EIF4A1	chr17	+	7575118	7575258	7574545	7574678	7576523	7576692	70	11	4	2	1	0	2	33	0	0	98	49	0.904424499	1	0.946	0.846	1	1	1	-0.069
A3SS	2819	STAT3	STAT3	chr17	-	42348388	42348539	42348388	42348517	42388278	42388495	38	12	6	17	9	1	2	14	1	6	71	49	0.907213185	1	0.607	0.479	0.805	0.58	0.617	0.032
SE	16463	ZDHHC17	ZDHHC17	chr12	+	76797433	76797537	76764073	76764329	76805316	76805439	20	10	2	2	2	2	1	8	0	3	98	49	0.914990937	1	0.833	0.714	0.333	1	0.571	-0.159
A3SS	1181	HNRNPK	HNRNPK	chr9	-	83968082	83969440	83968082	83969380	83970161	83970331	60	57	29	1	0	2	1	13	0	0	98	49	0.918678513	1	0.968	1	0.879	1	1	-0.051
SE	2644	BRD2	BRD2	chr6	+	32975020	32975112	32974461	32974765	32975383	32975521	21	6	1	15	3	1	1	3	0	3	98	49	0.922814456	1	0.412	0.5	0.333	1	0.333	-0.252
SE	246	DDX3X	DDX3X	chrX	+	41347314	41347451	41346504	41346622	41347639	41350287	102	29	29	1	2	0	1	11	0	0	98	49	0.922903892	1	0.981	0.879	1	1	1	-0.047
SE	13592	FAM129A	FAM129A	chr1	-	184823637	184823742	184823166	184823329	184831846	184831962	229	124	60	16	5	3	2	32	0	4	98	49	0.92335623	1	0.877	0.925	0.909	1	0.8	0.004
SE	15746	ERBB2IP	ERBB2IP	chr5	+	66075023	66075230	66053405	66054951	66076315	66076408	22	11	7	5	5	0	0	2	1	0	98	49	0.923482868	1	0.687	0.524	1	0	1	0.237
SE	5657	WDFY3	WDFY3	chr4	-	84726860	84726911	84724425	84724594	84733381	84733609	32	7	3	4	1	3	5	2	0	3	98	49	0.925440524	1	0.8	0.778	0.333	1	0.25	0.012

A3SS	600	BTN2A1	BTN2A1	chr6	+	26467718	26469653	26467947	26469653	26466061	26466088	20	4	3	3	0	0	2	4	0	2	98	49	0.925814529	1	0.769	1	1	1	0.5	0.173
SE	4288	PLXNC1	PLXNC1	chr12	+	94300909	94301057	94298631	94298795	94303755	94303896	85	44	32	0	1	2	4	17	0	0	98	49	0.929311539	1	1	0.957	0.889	1	1	-0.051
RI	786	SRSF2	SRSF2	chr17	-	76734114	76736464	76734114	76735158	76736153	76736464	55	21	6	16	3	2	7	12	2	3	98	49	0.930389012	1	0.632	0.778	0.6	0.636	0.667	0.019
A3SS	3977	IGF1R	IGF1R	chr15	+	98929557	98929660	98929560	98929660	98924524	98924684	15	2	2	6	0	1	1	2	0	3	52	49	0.930997489	1	0.702	1	0.653	1	0.386	0.092
SE	10098	PICALM	PICALM	chr11	-	85978069	85978093	85976622	85976682	85981128	85981228	28	12	12	58	39	13	0	12	2	14	73	49	0.931040519	1	0.245	0.171	0.383	0	0.365	0.084
RI	473	CASP1	CASP1	chr11	-	105025509	105026356	105025509	105025600	105026225	105026356	20	12	13	44	9	13	0	15	2	6	98	49	0.931871799	1	0.185	0.4	0.333	0	0.556	0.028
SE	12122	ST6GALNAC2	ST6GALNAC2	chr17	-	76578755	76578816	76574364	76574539	76585683	76586063	11	1	7	3	3	1	2	2	0	4	98	49	0.932313057	1	0.647	0.143	0.778	1	0.2	-0.077
A3SS	4092	NDUFA2	NDUFA2	chr5	-	140645362	140645725	140645362	140645678	140647255	140647362	5	0	5	1	2	18	0	3	1	2	96	49	0.938368028	1	0.718	0	0.124	0	0.434	0.064
SE	11300	KDM6B	KDM6B	chr17	+	7845549	7845691	7845313	7845456	7845871	7845970	105	37	39	1	2	0	9	23	0	0	98	49	0.938610085	1	0.981	0.902	1	1	1	-0.039
SE	1213	NAMPT	NAMPT	chr7	-	106274945	106275049	106272529	106272658	106277022	106277179	2440	1189	341	16	7	0	15	313	0	2	98	49	0.94075831	1	0.987	0.988	1	1	0.987	-0.002
SE	13786	SPAST	SPAST	chr2	+	32126947	32127022	32116118	32116212	32128407	32128479	28	12	4	3	0	0	1	4	0	0	98	49	0.943048222	1	0.824	1	1	1	1	-0.059
SE	12508	ABCA7	ABCA7	chr19	+	1054569	1054694	1054192	1054341	1054779	1054878	13	12	1	7	4	0	1	4	0	3	98	49	0.949350037	1	0.481	0.6	1	1	0.4	-0.006
SE	15924	RPS6KA1	RPS6KA1	chr1	+	26560725	26560851	26558806	26558937	26561044	26561134	71	22	16	0	0	0	1	11	0	2	98	49	0.953350319	1	1	1	1	1	0.733	0.133
SE	11513	SBNO2	SBNO2	chr19	-	1112188	1112301	1111995	1112067	1112401	1112537	61	21	22	10	4	0	8	15	4	0	98	49	0.956284839	1	0.753	0.724	1	0.5	1	0.076
SE	169	IL2RG	IL2RG	chrX	-	71108598	71108695	71108276	71108346	71109227	71109390	47	21	14	6	5	1	1	4	2	0	98	49	0.959685424	1	0.797	0.677	0.875	0.2	1	0.183
SE	8401	C19orf38	C19orf38	chr19	+	10859914	10859958	10858315	10858343	10863169	10863207	26	4	3	0	0	0	2	11	0	2	93	49	0.969972169	1	1	1	1	1	0.743	0.129
SE	1585	IGF2R	IGF2R	chr6	+	160061752	160061928	160061502	160061646	160062531	160062619	243	80	87	0	4	0	5	27	0	1	98	49	0.980810341	1	1	0.909	1	1	0.931	0.004
SE	5919	MYD88	MYD88	chr3	+	38140387	38140568	38139863	38139998	38140756	38140848	13	6	7	0	0	0	1	15	1	1	98	49	0.983062405	1	1	1	1	0.333	0.882	0.392
SE	5962	SORL1	SORL1	chr11	+	121574240	121574363	121566939	121567113	121583457	121583583	26	8	4	3	0	0	1	15	0	0	98	49	0.98862453	1	0.812	1	1	1	1	-0.063
A3SS	1740	AP1G2	AP1G2	chr14	-	23564045	23564221	23564045	23564159	23564332	23564388	9	4	0	4	0	1	3	1	0	3	98	49	0.98876747	1	0.529	1	0	1	0.143	-0.062
SE	6589	SETD2	SETD2	chr3	-	47101457	47101555	47097954	47098081	47103345	47103423	38	23	16	3	0	0	1	10	0	0	98	49	0.989533371	1	0.864	1	1	1	1	-0.045
A3SS	5	FAM122B	FAM122B	chrX	-	134769565	134772283	134769565	134771354	134781820	134781917	2	0	0	0	2	0	0	3	0	0				1	1	0	NA	NA	1	-0.5
A3SS	6	FAM122B	FAM122B	chrX	-	134785885	134785950	134785885	134785947	134787471	134787533	2	0	0	1	0	0	0	2	0	0	52	49	1	1	0.653	NA	NA	NA	1	-0.347
A3SS	15	MORF4L2	MORF4L2	chrX	-	103678498	103678651	103678498	103678600	103684680	103684729	14	3	0	1	0	0	0	2	0	0	98	49	1	1	0.875	1	NA	NA	1	-0.062
A3SS	25	ARMCX3	ARMCX3	chrX	+	101623785	101623893	101623813	101623893	101623129	101623276	0	0	0	1	1	0	0	1	0	0	77	49	1	1	0	0	NA	NA	1	-1
A3SS	31	ABCB7	ABCB7	chrX	-	75114753	75114834	75114753	75114831	75156104	75156340	1	0	0	1	0	0	0	2	0	0	52	49	1	1	0.485	NA	NA	NA	1	-0.515
A3SS	50	TFE3	TFE3	chrX	-	49039106	49039410	49039106	49039345	49040454	49040568	34	15	5	1	2	0	0	4	0	3	98	49	1	1	0.944	0.789	1	NA	0.4	0.511
A3SS	52	FTSJ1	FTSJ1	chrX	+	48482402	48482506	48482408	48482506	48481631	48481715	3	0	0	1	0	0	0	0	0	2	55	49	1	1	0.728	NA	NA	NA	0	0.728
A3SS	59	DDX3X	DDX3X	chrX	+	41347311	41347451	41347314	41347451	41346858	41347012	77	27	18	45	10	7	0	8	0	6	52	49	1	1	0.617	0.718	0.708	NA	0.557	0.124
A3SS	60	BCOR	BCOR	chrX	-	40063607	40063952	40063607	40063850	40064335	40064599	2	0	0	0	0	2	2	0	0	0	98	49	1	1	1	NA	0	1	NA	-0.5
A3SS	66	REPS2	REPS2	chrX	+	17025058	17025185	17025061	17025185	17022122	17022271	3	0	3	9	2	6	0	1	0	0	52	49	1	1	0.239	0	0.32	NA	1	-0.814
A3SS	69	CD99	CD99	chrX	+	2738199	2738256	2738202	2738256	2726259	2726373	20	3	2	1	0	0	0	10	0	1	52	49	1	1	0.95	1	1	NA	0.904	0.079
A3SS	84	SEC16A	SEC16A	chr9	-	136446854	136447015	136446854	136446949	136447226	136447364	4	0	0	0	1	0	0	1	0	0	98	49	1	1	1	0	NA	NA	1	-0.5
A3SS	126	ZNF189	ZNF189	chr9	+	101399883	101400010	101399925	101400010	101398853	101399189	2	0	0	0	0	0	0	1	0	1	91	49	1	1	1	NA	NA	NA	0.35	0.65
A3SS	136	FAM120A	FAM120A	chr9	+	93515666	93515767	93515669	93515767	93498789	93498886	9	4	1	2	0	0	0	1	0	2	52	49	1	1	0.809	1	1	NA	0.32	0.616
A3SS	139	NOL8	NOL8	chr9	-	92299889	92300810	92299889	92300016	92301550	92301822	2	0	0	2	1	0	0	1	0	0	98	49	1	1	0.333	0	NA	NA	1	-0.834
A3SS	140	SECISBP2	SECISBP2	chr9	+	89325426	89325676	89325547	89325676	89319651	89319797	16	3	3	3	1	0	0	4	0	1	98	49	1	1	0.727	0.6	1	NA	0.667	0.109
A3SS	154	FAM219A	FAM219A	chr9	-	34398183	34401122	34398183	34401119	34401665	34401720	5	0	2	1	0	3	0	1	0	1	52	49	1	1	0.825	NA	0.386	0	NA	0.605
A3SS	160	KDM4C	KDM4C	chr9	+	6986324	6986666	6986343	6986666	6984165	6984404	2	0	0	0	2	0	0	0	1	2	68	49	1	1	1	0	NA	0	0	0.5
A3SS	182	INTS8	INTS8	chr8	+	94838462	94838618	94838493	94838618	94836523	94836631	11	0	1	2	0	0	0	0	0	1	80	49	1	1	0.771	NA	1	NA	0	0.885
A3SS	194	TM2D2	TM2D2	chr8	-	38995317	38995794	38995317	38995405	38996212	38996522	0	1	0	0	0	0	0	2	0	1	98	49	1	1	NA	1	NA	NA	0.5	0.5
A3SS	209	PRKAG2	PRKAG2	chr7	-	151595344	151595454	151595344	151595451	151632068	151632138	6	1	6	0	8	0	0	1	0	0	52	49	1	1	1	0.105	1	NA	1	-0.298
A3SS	228	PNPLA8	PNPLA8	chr7	-	108514435	108515574	108514435	108515447	108521475	108521521	32	28	0	0	2	0	0	10	0	0	98	49	1	1	1	0.875	NA	NA	1	-0.062
A3SS	239	SRRT	SRRT	chr7	+	100885212	100885370	100885215	100885370	100884922	100885040	3	1	0	2	0	0	0	0	0	1	52	49	1	1	0.586	1	NA	NA	0	0.793
A3SS	240	SRRT	SRRT	chr7	+	100888041	100888143	100888053	100888143	100887702	100887859	5	4	0	3	1	0	1	1	0	3	61	49	1	1	0.572	0.763	NA	1	0.211	0.062
A3SS	245	STAG3L5P-PVRIG2P-PILRB	STAG3L5P-PVRIG2P-PILRB	chr7	+	100358689	100359079	100358898	100359079	100358219	100358366	12	2	3	0	0	1	0	1	0	0	98	49	1	1	1	1	0.6	NA	1	-0.133
A3SS	251	SMURF1	SMURF1	chr7	-	99033036	99033121	99033036	99033112	99035514	99035716	3	3	0	1	0	0	2	1	0	0	58	49	1	1	0.717	1	NA	1	1	-0.141
A3SS	270	GAB3	GAB3	chrX	-	154713206	154713426	154713206	154713423	154716025	154716329	1	0	0	2	0	0	0	1	0	0	52	49	1	1	1	0	NA	NA	1	-0.5
A3SS	280	ZMIZ2	ZMIZ2	chr7	+	44756946	44757149	44757042	44757149	44756424	44756539	6	4	0	1	0	0	0	3	0	0	98	49	1	1	0.75	1	NA	NA	1	-0.125
A3SS	282	POLM	POLM	chr7	-	44072061	44073430	44072061	44073377	44073624	44073708	9	1	0	0	1	0	0	1	0	0	98	49	1	1	1	0.333	NA	NA	1	-0.334
A3SS	286	CDK13	CDK13	chr7	+	40092784	40093237	40092964	40093237	40088125	40088331	12	3	1	0	1	0	0	1	0	0	98	49	1	1	1	0.6	1	NA	1	-0.133
A3SS	298	RNF216	RNF216	chr7	-	5740972	5741815	5740972	5741644	5752845	5752979	21	9	0	3	1	0	0	4	0	0	98	49	1	1	0.778	0.818	NA	NA	1	-0.202
A3SS	312	IPCEF1	IPCEF1	chr6	-	154247448	154247488	154247448	154247485	154265911	154265964	2	3	0	6	0	0	0	0	0	1	52	49	1	1	0.239	1	NA	NA	0	0.619
A3SS	336	VNN3	VNN3	chr6	-	132728740	132728979	132728740	132728933	132734252	132734383	109	54	13	29	12	3	0	9	0	4	95	49	1	1	0.66	0.699	0.691	NA	0.537	0.146
A3SS	337	ARG1	ARG1	chr6	+	131579086	131579285	131579110	131579285	131576662	131576735	0	0	0	5	5	3	0	2	0	4	73	49	1	1	0	0	0	NA	0.251	-0.251
A3SS	352	FAM135A	FAM135A	chr6	+	70524342	70526698	70524930	70526698	70523966	70524121	0	0	0	1	1	0	0	1	0	0	98	49	1	1	0	0	NA	NA	1	-1
A3SS	358	HSP90AB1	HSP90AB1	chr6	+	44248616	44248776	44248629	44248776	44246957	44247195	0	0	0	3	0	0	0	2	0	0	62	49	1	1	0	NA	NA	NA	1	-1
A3SS	369	GBGT1	GBGT1	chr9	-	133155177	133155349	133155177	133155312	133155900	133155936	3	0	0	2	0	0	0	1	0	1	86	49	1	1	0.461	NA	NA	NA	0.363	0.098
A3SS	370	GBGT1	GBGT1	chr9	-	133156014	133156102	133156014	133156065	133161466	133161532	2	3	0	1	4	0	0	0	0	2	86	49	1	1	0.533	0.299	NA	NA	0	0.416
A3SS	383	STK38	STK38	chr6	-	36540071	36540207	36540071	36540204	36547189	36547516	4	1	2	8	3	6	0	0	0	1	52	49	1	1	0.32	0.239	0.239	NA	0	0.266
A3SS	384	SRPK1	SRPK1	chr6	-	35890894	35891041	35890894	35891013	35920467	35920528	5	2	0	5	2	0	0	0	1	2	77	49	1	1	0.389	0.389	NA	0	0	0.389
A3SS	439	PSMB8-AS1	PSMB8-AS1	chr6	+	32845329	32846500	32845417	32846500	32844085	32844274	67	37	39	7	0	0	0	17	0	0	98	49	1	1	0.827	1	1	NA	1	-0.058
A3SS	500	C6orf25	C6orf25	chr6	+	31724745	31724844	31724764	31724844	31724586	31724627	6	0	0	1	0	0	9	0	0	0	68	49	1	1	0.812	NA	NA	1	NA	-0.188
A3SS	528	PRRC2A	PRRC2A	chr6	+	31622689	31622901	31622726	31622901	31620672	31620858	8	0	0	16	2	2	0	2	0	2	86	49	1	1	0.222	0	0	NA	0.363	-0.289
A3SS	543	DDX39B	DDX39B	chr6	-	31540321	31540664	31540321	31540534	31541949	31542475	14	6	0	4	0	0	1	0	0	0	98	49	1	1	0.636	1	NA	NA	1 NA	-0.182
A3SS	582	TRIM26	TRIM26	chr6	-	30198665	30199264	30198665	30199153	30201034	30201138	4	2	0	4	1	0	0	2	0	1	98	49	1	1	0.333	0.5	NA	NA	0.5	-0.084
A3SS	604	BTN3A1	BTN3A1	chr6	+	26412411	26415216	26412704	26415216	26411554	26411581	14	0	8	6	2	3	0	1	0	0	98	49	1	1	0.538	0	0.571	NA	1	-0.63
A3SS	607	BTN3A2	BTN3A2	chr6	+	26367974	26368050	26367989	26368050	26365158	26365352	0	0	0	11	4	0	0	1	0	2	64	49	1	1	0	0	NA	NA	0.277	-0.277
A3SS	608	BTN3A2	BTN3A2	chr6	+	26375514	26378320	26375796	26378320	26374770	26374798	7	2	0	3	1	2	0	0	0	2	98	49	1	1	0.538	0.5	0	NA	0	0.346
A3SS	611	FAM65B	FAM65B	chr6	-	24839365	24840775	24839365	24839599	24842861	24843554	109	83	41	12	14	10	0	20	0	2	98	49	1	1	0.82	0.748	0.672	NA	0.833	-0.086
A3SS	620	PHF23	PHF23	chr17	-	7235929	7236767	7235929	7236566	7237384	7237477	10	7	3	1	0	0	1	1	0	0	98	49	1	1	0.833	1	1	1	1	-0.056
A3SS	622	C17orf49	C17orf49	chr17	+	7017256	7017524	7017260	7017524	7016909	7017014	4	0	0	6	0	0	0	0	0	2	53	49	1	1	0.381	NA	NA	NA	0	0.381
A3SS	632	EML3	EML3	chr11	-	62611086	62611347	62611086	62611344	62611424	62611596	1	0	0	4	0	0	0	1	0	0	52	49	1	1	0.191	NA	NA	NA	1	-0.809
A3SS	644	BSDC1	BSDC1	chr1	-	32383829	32384048	32383829	32383997	32386778	32386895	3	1	0	8	5	3	0	0	0	1	98	49	1	1	0.158	0.091	0	NA	0	0.083
A3SS	649	C12orf57	C12orf57	chr12	+	6944379	6944565	6944475	6944565	6943816	6944173	0	0	0	11	0	5	1	1	0	0	98	49	1	1	0	NA	0	1	1	-1
A3SS	654	PSME3	PSME3	chr17	+	42838691	42838799	42838730	42838799	42838092	42838205	1	0	0	17	9	5	0	1	0	4	88	49	1	1	0.032	0	0	NA	0.122	-0.111
A3SS	669	CIAPIN1	CIAPIN1	chr16	-	57439181	57439334	57439181	57439295	57440771	57440983	1	0	1	0	2	0	0	2	0	0	88	49	1	1	1	0	1	NA	1	-0.333
A3SS	680	STX4	STX4	chr16	+	31034203	31034325	31034225	31034325	31034012	31034114	2	0	0	4	5	0	0	3	1	2	71	49	1	1	0.257	0	NA	0	0.509	-0.126
A3SS	685	ATXN2L	ATXN2L	chr16	+	28832186	28832399	28832204	28832399	28830961	28831072	1	1	0	3	3	0	0	1	1	0	67	49	1	1	0.196	0.196	NA	0	1	-0.304
A3SS	697	WDTC1	WDTC1	chr1	+	27297047	27297156	27297050	27297156	27296325	27296401	19	6	10	1	0	0	0	4	0	1	52	49	1	1	0.947	1	1	NA	0.79	0.192
A3SS	701	PCBP2	PCBP2	chr12	+	53468776	53468832	53468779	53468832	53467220	53467293	15	1	2	3	1	0	0	7	0	2	52	49	1	1	0.825	0.485	1	NA	0.767	0.003
A3SS	702	PCBP2	PCBP2	chr12	+	53468776	53468832	53468779	53468832	53467804	53467843	28	9	7	14	1	2	0	5	0	0	52	49	1	1	0.653	0.895	0.767	NA	1	-0.228
A3SS	711	SBNO1	SBNO1	chr12	-	123345257	123345570	123345257	123345567	123348028	123348133	8	1	2	7	4	0	0	0	0	1	52	49	1	1	0.519	0.191	1	NA	0	0.57
A3SS	714	MEFV	MEFV	chr16	-	3243859	3244067	3243859	3243892	3244253	3244286	5	3	3	10	8	13	0	8	0	3	98	49	1	1	0.2	0.158	0.103	NA	0.571	-0.417
A3SS	719	ZC3H14	ZC3H14	chr14	+	88609711	88609803	88609714	88609803	88609266	88609403	0	4	0	1	0	0	0	1	0	0	52	49	1	1	0	1	NA	NA	1	-0.5
A3SS	727	TSC2	TSC2	chr16	+	2079031	2079196	2079034	2079196	2076490	2076585	0	4	0	5	0	0	0	1	0	0	52	49	1	1	0	1	NA	NA	1	-0.5
A3SS	732	ATG5	ATG5	chr6	-	106248149	106248244	106248149	106248240	106279660	106279823	7	9	3	1	0	0	0	3	0	0	53	49	1	1	0.866	1	1	NA	1	-0.045
A3SS	733	NLRP12	NLRP12	chr19	-	53807494	53807668	53807494	53807665	53809586	53811288	4	1	0	21	7	6	0	1	0	6	52	49	1	1	0.152	0.119	0	NA	0.136	-0.046
A3SS	736	SERF2	SERF2	chr15	+	43793575	43796089	43793709	43796089	43792974	43793083	16	1	0	76	14	13	0	0	0	9	98	49	1	1	0.095	0.034	0	NA	0	0.043
A3SS	737	SERF2	SERF2	chr15	+	43793686	43796089	43793709	43796089	43792974	43793083	12	1	0	76	14	13	0	0	0	9	72	49	1	1	0.097	0.046	0	NA	0	0.048
A3SS	748	AP3S2	AP3S2	chr15	-	89889048	89889140	89889048	89889136	89893880	89894385	6	3	0	0	3	0	0	2	0	0	53	49	1	1	1	0.48	NA	NA	1	-0.26
A3SS	751	ISG20	ISG20	chr15	+	88650234	88652310	88652109	88652310	88639342	88639594	3	5	1	13	15	13	0	2	0	11	98	49	1	1	0.103	0.143	0.037	NA	0.083	0.011
A3SS	782	ZNF143	ZNF143	chr11	+	9474549	9474633	9474552	9474633	9473940	9474024	9	1	0	4	0	0	0	1	0	3	52	49	1	1	0.68	1	NA	NA	0.239	0.601
A3SS	786	TRPM7	TRPM7	chr15	-	50583088	50583159	50583088	50583156	50586391	50586488	1	1	6	1	0	1	0	1	0	0	52	49	1	1	0.485	1	0.85	NA	1	-0.222
A3SS	790	PLAGL1	PLAGL1	chr6	-	143960468	143960556	143960468	143960542	143964786	143964818	1	4	0	1	0	0	2	0	0	1	63	49	1	1	0.438	1	NA	1	0	0.219
A3SS	791	PLAGL1	PLAGL1	chr6	-	143960468	143960580	143960468	143960542	143964786	143964818	1	3	0	1	0	0	2	0	0	1	87	49	1	1	0.36	1	NA	1	0	0.18
A3SS	831	KIAA0430	KIAA0430	chr16	-	15622933	15623126	15622933	15623123	15624768	15624927	0	2	0	13	1	0	0	1	0	0	52	49	1	1	0	0.653	NA	NA	1	-0.673
A3SS	832	KIAA0430	KIAA0430	chr16	-	15625371	15625800	15625371	15625797	15630331	15630504	13	5	0	2	0	2	0	2	0	0	52	49	1	1	0.86	1	0	NA	1	-0.38
A3SS	843	CIITA	CIITA	chr16	+	10898921	10899002	10898924	10899002	10898669	10898732	4	0	0	0	0	0	0	2	0	2	52	49	1	1	1	NA	NA	NA	0.485	0.515
A3SS	844	CIITA	CIITA	chr16	+	10908982	10909187	10909028	10909187	10906498	10908149	1	0	0	0	1	0	0	1	0	0	95	49	1	1	1	0	NA	NA	1	-0.5
A3SS	850	MMS19	MMS19	chr10	-	97466058	97466159	97466058	97466156	97466503	97466585	2	0	0	0	0	0	0	3	0	1	52	49	1	1	1	NA	NA	NA	0.739	0.261
A3SS	855	DLST	DLST	chr14	+	74889274	74889349	74889276	74889349	74889094	74889147	2	5	1	1	0	0	0	1	0	0	51	49	1	1	0.658	1	1	NA	1	-0.114
A3SS	858	ZNF410	ZNF410	chr14	+	73896315	73896534	73896347	73896534	73893796	73893932	4	5	1	1	0	0	0	3	0	0	81	49	1	1	0.708	1	1	NA	1	-0.097
A3SS	859	ZNF410	ZNF410	chr14	+	73904901	73905083	73904930	73905083	73903959	73904110	8	5	2	3	1	0	0	2	0	0	78	49	1	1	0.85	1	1	NA	1	-0.05
A3SS	862	PCNX	PCNX	chr14	+	71012984	71013202	71012999	71013202	71011491	71011549	67	27	10	5	8	0	0	12	0	4	64	49	1	1	0.911	0.721	1	NA	0.697	0.18
A3SS	870	NLRC3	NLRC3	chr16	-	3567242	3567385	3567242	3567324	3577148	3577404	1	0	0	2	0	0	0	1	0	0	98	49	1	1	0.2	NA	NA	NA	1	-0.8
A3SS	878	COPS2	COPS2	chr15	-	49137347	49137458	49137347	49137437	49139527	49139653	0	1	0	0	1	0	0	2	0	2	70	49	1	1	NA	0.412	NA	NA	0.412	0
A3SS	890	LTB4R2	LTB4R2	chr14	+	24310598	24312053	24310654	24312053	24310150	24310255	4	1	0	1	0	0	0	1	0	0	98	49	1	1	0.667	1	NA	NA	1	-0.166
A3SS	894	TNIP1	TNIP1	chr5	-	151029942	151030747	151029942	151030673	151032286	151032383	21	13	13	1	1	0	0	6	0	0	98	49	1	1	0.913	0.867	1	NA	1	-0.073
A3SS	899	ARHGEF40	ARHGEF40	chr14	+	21076535	21076643	21076562	21076643	21076359	21076456	3	1	0	5	1	2	0	0	0	4	76	49	1	1	0.279	0.392	0	NA	0	0.224
A3SS	906	NOP16	NOP16	chr5	-	176386839	176386943	176386839	176386909	176388234	176388343	3	0	0	0	0	0	0	0	0	0	83	49	1	1	1	NA	NA	NA	0	1
A3SS	918	PLEKHB2	PLEKHB2	chr2	+	131140166	131140275	131140169	131140275	131132901	131132991	23	5	3	2	0	0	1	3	0	0	52	49	1	1	0.916	1	1	1	1	-0.028
A3SS	919	PLEKHB2	PLEKHB2	chr2	+	131140166	131140275	131140169	131140275	131132901	131133015	5	0	1	1	0	0	0	2	0	0	52	49	1	1	0.825	NA	1	NA	1	-0.088
A3SS	921	CARKD	CARKD	chr13	+	110638241	110639995	110638377	110639995	110637128	110637249	3	0	0	0	0	0	0	0	0	1	98	49	1	1	1	NA	NA	NA	0	1
A3SS	939	TTC23	TTC23	chr15	-	99139316	99139467	99139316	99139399	99156147	99156297	0	1	0	0	0	0	0	0	0	1	98	49	1	1	NA	1	NA	NA	0	1
A3SS	942	USP16	USP16	chr21	+	29037275	29037463	29037278	29037463	29036270	29036374	8	5	0	0	0	1	0	3	0	0	52	49	1	1	1	1	0	NA	1	-0.333
A3SS	969	ZNF18	ZNF18	chr17	-	11983296	11983407	11983296	11983404	11984112	11984197	0	0	2	0	0	0	0	0	0	1	52	49	1	1	NA	NA	1	NA	0	1
A3SS	974	RBM26	RBM26	chr13	-	79365577	79365733	79365577	79365718	79366054	79366195	1	0	0	5	0	0	0	0	0	2	64	49	1	1	0.133	NA	NA	NA	0	0.133
A3SS	977	SUPT20H	SUPT20H	chr13	-	37022010	37022317	37022010	37022080	37024034	37024193	5	0	0	2	0	0	0	2	0	1	98	49	1	1	0.556	NA	NA	NA	0.5	0.056
A3SS	987	PPP4C	PPP4C	chr16	+	30081199	30081310	30081258	30081310	30076314	30076475	8	2	0	18	15	7	0	3	0	2	98	49	1	1	0.182	0.062	0	NA	0.429	-0.348
A3SS	990	PSPC1	PSPC1	chr13	-	19674751	19675653	19674751	19674984	19677723	19677823	2	1	1	2	1	1	0	0	0	1	98	49	1	1	0.333	0.333	0.333	NA	0	0.333
A3SS	1001	TRAPPC13	TRAPPC13	chr5	+	65664233	65664384	65664236	65664384	65662049	65662150	1	4	0	0	2	0	0	0	0	1	52	49	1	1	1	0.653	NA	NA	0	0.827
A3SS	1003	ARL6IP4	ARL6IP4	chr12	+	122981570	122981879	122981594	122981879	122981128	122981266	1	0	0	5	0	0	0	0	0	1	73	49	1	1	0.118	NA	NA	NA	0	0.118
A3SS	1004	ARL6IP4	ARL6IP4	chr12	+	122981570	122981879	122981594	122981879	122981128	122981299	3	0	0	6	1	2	0	0	0	1	73	49	1	1	0.251	0	0	NA	0	0.084
A3SS	1005	ARL6IP4	ARL6IP4	chr12	+	122981594	122981809	122981655	122981809	122981128	122981299	20	5	5	1	0	0	0	4	0	0	98	49	1	1	0.909	1	1	NA	1	-0.03
A3SS	1018	ATPIF1	ATPIF1	chr1	+	28237760	28238105	28237836	28238105	28236360	28236452	2	0	0	4	5	2	0	5	0	1	98	49	1	1	0.2	0	0	NA	0.714	-0.647
A3SS	1031	POLDIP3	POLDIP3	chr22	-	42601969	42602107	42601969	42602056	42602769	42603160	1	0	0	8	4	0	0	0	0	1	98	49	1	1	0.059	0	NA	NA	0	0.029
A3SS	1044	CCDC53	CCDC53	chr12	-	102039867	102039978	102039867	102039975	102044104	102044212	9	2	0	2	0	0	0	1	0	0	52	49	1	1	0.809	1	NA	NA	1	-0.095
A3SS	1049	MARK3	MARK3	chr14	+	103468032	103468186	103468080	103468186	103467078	103467191	28	19	4	0	0	1	0	13	0	0	97	49	1	1	1	1	0.669	NA	1	-0.11
A3SS	1064	OAZ1	OAZ1	chr19	+	2271384	2271530	2271390	2271530	2269485	2269744	172	107	112	1	0	0	10	66	0	0	55	49	1	1	0.994	1	1	1	1	-0.002
A3SS	1088	TMCC1	TMCC1	chr3	-	129827802	129828508	129827802	129828077	129832773	129832826	35	29	11	0	0	1	0	6	0	0	98	49	1	1	1	1	0.846	NA	1	-0.051
A3SS	1104	DDIT3	DDIT3	chr12	-	57517268	57517519	57517268	57517438	57517705	57517753	37	34	5	5	4	0	0	5	0	1	98	49	1	1	0.787	0.81	1	NA	0.714	0.152
A3SS	1105	DDIT3	DDIT3	chr12	-	57517268	57517542	57517268	57517438	57517705	57517753	37	34	5	5	4	0	0	5	0	1	98	49	1	1	0.787	0.81	1	NA	0.714	0.152
A3SS	1106	DDIT3	DDIT3	chr12	-	57517268	57517595	57517268	57517438	57517705	57517753	40	34	5	5	4	0	0	5	0	1	98	49	1	1	0.8	0.81	1	NA	0.714	0.156
A3SS	1113	STARD3	STARD3	chr17	+	39658708	39658820	39658721	39658820	39658404	39658522	0	1	0	4	5	0	0	0	1	1	62	49	1	1	0	0.136	NA	0	0	0.068
A3SS	1119	ESYT1	ESYT1	chr12	+	56134079	56134181	56134109	56134181	56133780	56133873	2	0	0	2	1	0	0	1	0	1	79	49	1	1	0.383	0	NA	NA	0.383	-0.192
A3SS	1126	APPL2	APPL2	chr12	-	105207970	105208047	105207970	105208029	105208157	105208199	2	2	0	11	1	3	0	0	0	5	67	49	1	1	0.117	0.594	0	NA	0	0.237
A3SS	1130	SURF4	SURF4	chr9	-	133367258	133367543	133367258	133367445	133375921	133376166	1	0	0	1	1	0	0	1	0	0	98	49	1	1	0.333	0	NA	0	NA	0.167
A3SS	1133	MDM1	MDM1	chr12	-	68325004	68327510	68325004	68327021	68331106	68331221	2	3	1	3	0	0	0	1	0	1	98	49	1	1	0.25	1	1	NA	0	0.75
A3SS	1143	TRIP12	TRIP12	chr2	-	229805729	229805883	229805729	229805802	229807707	229807864	25	6	4	1	1	0	0	8	0	0	98	49	1	1	0.926	0.75	1	NA	1	-0.108
A3SS	1157	FGD4	FGD4	chr12	+	32607937	32608095	32607956	32608095	32602160	32602317	6	3	0	24	7	15	0	0	0	4	68	49	1	1	0.153	0.236	0	NA	0	0.13
A3SS	1167	DLG1	DLG1	chr3	-	197076585	197076685	197076585	197076682	197081050	197081117	1	0	0	1	0	0	0	4	0	0	52	49	1	1	0.485	NA	NA	NA	1	-0.515
A3SS	1170	LRMP	LRMP	chr12	+	25063719	25063816	25063722	25063816	25062819	25062900	50	29	6	24	6	0	0	3	0	2	98	49	1	1	0.663	0.82	1	NA	0.586	0.242
A3SS	1178	CARD8	CARD8	chr19	-	48230437	48230700	48230437	48230697	48230776	48231006	25	9	7	5	2	0	0	4	0	0	52	49	1	1	0.825	0.809	1	NA	1	-0.122
A3SS	1188	QKI	QKI	chr6	+	163563419	163563719	163563443	163563719	163561981	163562069	39	22	18	1	2	0	0	7	0	0	73	49	1	1	0.963	0.881	1	NA	1	-0.052
A3SS	1189	QKI	QKI	chr6	+	163564666	163578596	163565945	163578596	163563419	163563719	14	4	4	8	0	0	0	3	0	0	98	49	1	1	0.467	1	1	NA	1	-0.178
A3SS	1190	QKI	QKI	chr6	+	163564666	163578596	163570693	163578596	163563419	163563719	8	0	3	1	0	0	0	7	0	0	98	49	1	1	0.8	NA	1	NA	1	-0.1
A3SS	1191	QKI	QKI	chr6	+	163565945	163578596	163570693	163578596	163563419	163563719	16	0	1	1	0	0	0	7	0	0	98	49	1	1	0.889	NA	1	NA	1	-0.055
A3SS	1197	CD163	CD163	chr12	-	7479859	7479996	7479859	7479913	7481160	7481256	0	0	0	2	0	0	0	5	0	3	98	49	1	1	0	NA	NA	NA	0.455	-0.455
A3SS	1200	C1RL	C1RL	chr12	-	7099685	7099807	7099685	7099760	7099900	7100026	3	1	0	12	3	2	0	2	0	2	96	49	1	1	0.113	0.145	0	NA	0.338	-0.252
A3SS	1205	TLE4	TLE4	chr9	+	79652589	79652794	79652592	79652794	79627373	79627448	3	0	3	6	1	0	0	0	0	2	52	49	1	1	0.32	0	1	NA	0	0.44
A3SS	1209	SLTM	SLTM	chr15	-	58899468	58899937	58899468	58899883	58901259	58901287	10	5	1	1	0	0	0	6	0	0	98	49	1	1	0.833	1	1	NA	1	-0.056
A3SS	1215	WNK1	WNK1	chr12	+	878211	878361	878214	878361	862082	862270	6	0	0	1	1	0	0	1	0	2	52	49	1	1	0.85	0	NA	NA	0.32	0.105
A3SS	1216	WNK1	WNK1	chr12	+	878211	878361	878214	878361	871264	871348	8	1	0	3	1	0	2	0	0	1	52	49	1	1	0.715	0.485	NA	1	0	0.1
A3SS	1221	KANSL3	KANSL3	chr2	-	96631311	96631543	96631311	96631482	96636920	96637185	4	2	0	1	0	0	0	3	0	0	98	49	1	1	0.667	1	NA	NA	1	-0.166

A3SS	1225	NFRKB	NFRKB	chr11	-	129884744	129884882	129884744	129884846	129885434	129885609	0	1	5	1	0	0	0	1	0	0	85	49	1	1	0	1	1 NA		1	-0.333
A3SS	1227	MEF2C	MEF2C	chr5	-	88718240	88722925	88718240	88722829	88728492	88728628	5	0	0	2	0	0	0	1	0	1	98	49	1	1	0.556 NA	NA	NA		0.333	0.223
A3SS	1228	MEF2C	MEF2C	chr5	-	88823734	88823930	88823734	88823927	88882954	88883466	2	2	0	3	0	2	0	2	0	1	52	49	1	1	0.386	1	0 NA		0.653	-0.191
A3SS	1229	ANAPC5	ANAPC5	chr12	-	121328315	121328497	121328315	121328458	121330582	121330672	9	1	0	0	0	1	0	3	0	0	88	49	1	1	1	1	0 NA		1	-0.333
A3SS	1235	TMEM218	TMEM218	chr11	-	125101200	125101303	125101200	125101256	125102733	125102809	0	1	0	2	0	0	0	1	0	0	96	49	1	1	0	1 NA	NA		1	-0.5
A3SS	1242	NRGN	NRGN	chr11	+	124745961	124745982	124745964	124745982	124745502	124745729	9	0	0	12	0	0	2	1	4	1	52	49	1	1	0.414 NA	NA		0.32	0.485	0.012
A3SS	1257	SLC25A45	SLC25A45	chr11	-	65380131	65380234	65380131	65380175	65380533	65380632	1	0	1	0	0	0	0	0	0	1	98	49	1	1	1 NA		1 NA		0	1
A3SS	1263	MPZL3	MPZL3	chr11	-	118240210	118240377	118240210	118240341	118252221	118252368	23	2	10	0	2	0	0	6	0	0	85	49	1	1	1	0.366	1 NA		1	-0.211
A3SS	1277	MARK2	MARK2	chr11	+	63902600	63902782	63902603	63902782	63902197	63902330	1	0	0	25	4	8	0	0	0	6	52	49	1	1	0.036	0	0 NA		0	0.012
A3SS	1363	CCL4L2	CCL4L2	chr17	+	36212292	36212878	36212496	36212878	36211775	36211890	10	10	6	0	0	0	0	0	0	1	98	49	1	1	1	1	1 NA		0	1
A3SS	1367	CCL4L2	CCL4L2	chr17	+	36212302	36212878	36212496	36212878	36211775	36211890	10	10	6	0	0	0	0	0	0	1	98	49	1	1	1	1	1 NA		0	1
A3SS	1370	CCL4L2	CCL4L2	chr17	+	36212312	36212878	36212496	36212878	36211775	36211890	10	10	6	0	0	0	0	0	0	1	98	49	1	1	1	1	1 NA		0	1
A3SS	1372	CCL4L2	CCL4L2	chr17	+	36212333	36212878	36212496	36212878	36211775	36211890	10	10	6	0	0	0	0	0	0	1	98	49	1	1	1	1	1 NA		0	1
A3SS	1373	CCL4L2	CCL4L2	chr17	+	36212363	36212878	36212496	36212878	36211775	36211890	10	10	6	0	0	0	0	0	0	1	98	49	1	1	1	1	1 NA		0	1
A3SS	1382	RASGRP4	RASGRP4	chr19	-	38414847	38415123	38414847	38414916	38417051	38417168	55	18	9	0	1	1	2	16	0	0	98	49	1	1	1	0.9	0.818	1	1	-0.094
A3SS	1393	RC3H1	RC3H1	chr1	-	173931083	173938874	173931083	173938871	173941264	173941380	6	6	2	8	2	4	0	0	0	4	52	49	1	1	0.414	0.739	0.32 NA		0	0.491
A3SS	1394	RC3H1	RC3H1	chr1	-	173943441	173943615	173943441	173943588	173946475	173946608	12	1	3	1	3	6	0	2	0	0	76	49	1	1	0.886	0.177	0.244 NA		1	-0.564
A3SS	1431	COPA	COPA	chr1	-	160305432	160305598	160305432	160305571	160305687	160305773	0	0	0	9	10	0	0	4	0	3	76	49	1	1	0	0 NA	NA		0.462	-0.462
A3SS	1434	KCNJ15	KCNJ15	chr21	+	38257045	38257185	38257103	38257185	38229914	38230023	44	6	10	2	0	0	0	1	0	0	98	49	1	1	0.917	1	1 NA		1	-0.028
A3SS	1440	SDF4	SDF4	chr1	-	1216907	1217804	1216907	1217688	1218457	1218633	0	1	0	15	4	3	0	0	0	4	98	49	1	1	0	0.111	0 NA		0	0.037
A3SS	1445	METTL9	METTL9	chr16	+	21655226	21657473	21655229	21657473	21624930	21625115	2	6	1	1	0	1	0	2	0	2	52	49	1	1	0.653	1	0.485 NA		0.485	0.228
A3SS	1459	SERPINA1	SERPINA1	chr14	-	94388559	94388663	94388559	94388660	94388800	94389010	25	0	4	2	0	1	0	3	0	2	52	49	1	1	0.922 NA		0.79 NA		0.586	0.27
A3SS	1460	SERPINA1	SERPINA1	chr14	-	94388559	94388663	94388559	94388660	94388818	94389010	18	2	6	18	3	11	0	3	0	1	52	49	1	1	0.485	0.386	0.339 NA		0.739	-0.336
A3SS	1493	SRSF10	SRSF10	chr1	-	23964346	23971439	23964346	23967723	23971572	23971623	4	1	0	4	0	2	0	1	0	0	98	49	1	1	0.333	1	0 NA		1	-0.556
A3SS	1494	SRSF10	SRSF10	chr1	-	23964346	23971439	23964346	23967956	23971572	23971623	4	4	0	0	1	0	0	1	0	0	98	49	1	1	1	0.667 NA	NA		1	-0.166
A3SS	1498	EDRF1	EDRF1	chr10	+	125730271	125730399	125730287	125730399	125729357	125729479	1	0	0	1	2	1	0	0	0	1	65	49	1	1	0.43	0	0 NA		0	0.143
A3SS	1525	FAM63A	FAM63A	chr1	-	151002164	151002706	151002164	151002323	151006311	151006909	24	4	3	2	0	0	3	4	0	1	98	49	1	1	0.857	1	1	1	0.667	0.119
A3SS	1532	CDIPT	CDIPT	chr16	-	29862585	29862720	29862585	29862635	29862814	29863288	23	8	11	1	0	0	0	1	0	0	98	49	1	1	0.92	1	1 NA		1	-0.027
A3SS	1554	ALPK1	ALPK1	chr4	+	112377677	112377898	112377729	112377898	112315800	112315852	58	24	5	9	3	0	0	7	0	0	98	49	1	1	0.763	0.8	1 NA		1	-0.146
A3SS	1576	TSC22D4	TSC22D4	chr7	-	100477276	100478204	100477276	100477459	100478793	100479279	27	8	9	1	1	2	0	9	0	0	98	49	1	1	0.931	0.8	0.692 NA		1	-0.192
A3SS	1577	TSC22D4	TSC22D4	chr7	-	100477276	100478307	100477276	100477459	100478793	100479279	27	10	14	1	1	2	0	11	0	0	98	49	1	1	0.931	0.833	0.778 NA		1	-0.153
A3SS	1580	TSC22D4	TSC22D4	chr7	-	100477276	100478307	100477276	100478204	100478793	100479279	11	6	7	2	0	1	0	2	0	0	98	49	1	1	0.733	1	0.778 NA		1	-0.163
A3SS	1592	BRD7	BRD7	chr16	-	50320662	50320777	50320662	50320774	50321981	50322038	0	1	0	2	0	0	0	1	0	0	52	49	1	1	0	1 NA	NA		1	-0.5
A3SS	1604	CEP170	CEP170	chr1	-	243156220	243156455	243156220	243156347	243164283	243166116	4	2	0	1	0	0	0	1	0	0	98	49	1	1	0.667	1 NA	NA		1	-0.166
A3SS	1607	MAP2K7	MAP2K7	chr19	+	7911414	7911578	7911435	7911578	7911249	7911330	3	1	0	1	3	0	0	0	1	2	70	49	1	1	0.677	0.189 NA	NA	0	0	0.433
A3SS	1609	ARHGEF2	ARHGEF2	chr1	-	155964987	155965131	155964987	155965128	155965302	155965412	16	4	0	15	5	0	0	2	0	4	52	49	1	1	0.501	0.43 NA	NA		0.32	0.146
A3SS	1610	TMEM59	TMEM59	chr1	-	54040755	54040840	54040755	54040837	54041723	54041805	11	10	5	30	16	18	0	0	0	2	52	49	1	1	0.257	0.371	0.207 NA		0	0.278
A3SS	1613	ADAT1	ADAT1	chr16	-	75618585	75618698	75618585	75618640	75620265	75620334	0	1	2	5	0	0	0	1	0	2	98	49	1	1	0	1	1	0 NA	0	0.667
A3SS	1616	SORT1	SORT1	chr1	-	109345750	109345881	109345750	109345878	109347482	109347532	5	0	2	3	0	0	0	5	0	2	52	49	1	1	0.611 NA		1 NA		0.702	0.104
A3SS	1620	DHDDS	DHDDS	chr1	+	26468891	26471304	26468894	26471304	26460036	26460144	0	0	0	2	0	1	0	1	0	0	52	49	1	1	0 NA		0 NA		1	-1
A3SS	1624	SOAT1	SOAT1	chr1	+	179342009	179342192	179342113	179342192	179341027	179341310	2	0	0	4	1	2	0	0	0	3	98	49	1	1	0.2	0	0 NA		0	0.067
A3SS	1639	TGFBR3	TGFBR3	chr1	-	91719892	91720230	91719892	91720227	91721954	91722144	1	0	0	0	0	0	0	0	0	1	52	49	1	1	1 NA	NA	NA		0	1
A3SS	1647	HDDC3	HDDC3	chr15	-	90930917	90931556	90930917	90931405	90931703	90931944	0	4	0	0	0	0	4	1	0	2	98	49	1	1 NA		1 NA		1	0.2	0.4
A3SS	1651	LMBR1L	LMBR1L	chr12	-	49101249	49101323	49101249	49101263	49101471	49101549	8	3	4	1	1	0	1	4	0	0	98	49	1	1	0.8	0.6	1	1	1	-0.2
A3SS	1663	SYTL1	SYTL1	chr1	+	27349079	27349152	27349115	27349152	27347966	27348012	6	4	5	1	1	0	0	3	0	0	85	49	1	1	0.776	0.698	1 NA		1	-0.175
A3SS	1666	WDR47	WDR47	chr1	-	109004591	109004718	109004591	109004715	109010915	109011718	1	0	0	0	1	0	0	1	0	0	52	49	1	1	1	0 NA	NA		1	-0.5
A3SS	1678	FDPS	FDPS	chr1	+	155310042	155310205	155310063	155310205	155308747	155308965	3	0	0	5	0	0	0	1	0	0	70	49	1	1	0.296 NA	NA	NA		1	-0.704
A3SS	1681	AKAP13	AKAP13	chr15	+	85710578	85710645	85710581	85710645	85708018	85708086	66	11	12	4	0	0	0	13	0	0	52	49	1	1	0.94	1	1 NA		1	-0.02
A3SS	1698	SRP19	SRP19	chr5	+	112867371	112868392	112867403	112868392	112864620	112864732	6	2	0	2	0	1	0	1	0	1	81	49	1	1	0.645	1	0 NA		0.377	0.171
A3SS	1702	TUG1	TUG1	chr22	+	30972854	30973601	30972923	30973601	30969210	30971779	13	3	5	2	1	2	0	1	0	0	98	49	1	1	0.765	0.6	0.556 NA		1	-0.36
A3SS	1704	TRIM5	TRIM5	chr11	-	5663194	5665395	5663194	5664173	5665655	5665682	8	0	0	1	0	0	0	1	0	0	98	49	1	1	0.8 NA	NA	NA		1	-0.2
A3SS	1715	DMXL2	DMXL2	chr15	-	51476588	51476719	51476588	51476716	51478270	51478347	16	1	0	14	2	0	0	8	0	2	52	49	1	1	0.519	0.32 NA	NA		0.79	-0.371
A3SS	1727	CASP1	CASP1	chr11	-	105025509	105026356	105025509	105025600	105026841	105026951	62	20	21	1	2	0	0	29	0	0	98	49	1	1	0.969	0.833	1 NA		1	-0.066
A3SS	1736	CD83	CD83	chr6	+	14135107	14136917	14135110	14136917	14133648	14133755	9	1	0	0	0	0	0	0	0	1	52	49	1	1	1	1 NA	NA		0	1
A3SS	1742	MVP	MVP	chr16	+	29830473	29830674	29830514	29830674	29820393	29820510	13	2	1	0	2	0	0	0	0	1	90	49	1	1	1	0.353	1 NA		0	0.784
A3SS	1761	SLC25A44	SLC25A44	chr1	+	156207861	156208013	156207885	156208013	156199834	156200472	1	0	0	8	9	2	0	0	0	1	73	49	1	1	0.077	0	0 NA		0	0.026
A3SS	1765	PAPD4	PAPD4	chr5	+	79642831	79642910	79642843	79642910	79641104	79641196	3	0	0	3	3	2	0	4	0	0	61	49	1	1	0.445	0	0.287 NA		1	-0.756
A3SS	1781	RBMX	RBMX	chrX	-	136879016	136879158	136879016	136879123	136879318	136879453	2	0	0	9	3	0	0	0	0	4	84	49	1	1	0.115	0 NA	NA		0	0.058
A3SS	1790	FAM217B	FAM217B	chr20	+	59943939	59948647	59944395	59948647	59942426	59942512	6	7	3	1	1	0	0	3	0	0	98	49	1	1	0.75	0.778	1 NA		1	-0.157
A3SS	1795	SREK1	SREK1	chr5	+	66177513	66177658	66177516	66177658	66174945	66175041	6	7	0	2	2	0	0	1	0	0	52	49	1	1	0.739	0.767 NA	NA		1	-0.247
A3SS	1807	STK40	STK40	chr1	-	36358736	36358837	36358736	36358822	36361220	36361340	2	0	0	11	6	12	0	1	0	1	64	49	1	1	0.122	0	0 NA		0.434	-0.393
A3SS	1810	SHC1	SHC1	chr1	-	154965945	154966080	154965945	154966077	154966161	154966231	1	0	0	0	0	0	0	0	0	3	52	49	1	1	1 NA	NA	NA		0	1
A3SS	1828	ATP11A	ATP11A	chr13	+	112881815	112887168	112881875	112887168	112875775	112875941	11	1	0	0	3	0	3	1	0	1	98	49	1	1	1	0.143 NA	NA	1	0.333	-0.095
A3SS	1842	TPM3	TPM3	chr1	-	154155303	154159048	154155303	154157721	154170399	154170469	15	10	3	75	46	36	0	1	0	20	98	49	1	1	0.091	0.098	0.04 NA		0.024	0.052
A3SS	1843	TPM3	TPM3	chr1	-	154170399	154170469	154170399	154170465	154170648	154170711	114	42	46	0	2	0	0	19	0	1	53	49	1	1	1	0.951	1 NA		0.946	0.038
A3SS	1845	CDK12	CDK12	chr17	+	39530603	39534565	39530630	39534565	39525863	39526316	6	1	0	2	0	0	0	1	0	0	76	49	1	1	0.659	1 NA	NA		1	-0.17
A3SS	1862	APTX	APTX	chr9	-	32988078	32988129	32988078	32988124	32989758	32989895	1	0	0	1	0	0	0	0	0	1	54	49	1	1	0.476 NA	NA	NA		0	0.476
A3SS	1898	SZRD1	SZRD1	chr1	+	16393227	16393482	16393230	16393482	16391374	16391424	0	0	1	1	0	0	1	0	0	0	52	49	1	1	0 NA		1	1 NA	1	-0.5
A3SS	1900	FUS	FUS	chr16	+	31183857	31184002	31183860	31184002	31182512	31182664	6	6	5	3	6	0	0	5	0	3	52	49	1	1	0.653	0.485	1 NA		0.611	0.102
A3SS	1901	ZBTB17	ZBTB17	chr1	-	15942330	15942441	15942330	15942420	15942528	15942738	2	3	0	2	1	0	0	0	0	2	70	49	1	1	0.412	0.677 NA	NA		0	0.544
A3SS	1902	ZBTB17	ZBTB17	chr1	-	15946153	15946316	15946153	15946294	15946934	15947123	0	1	0	3	0	0	0	1	1	2	71	49	1	1	0	1 NA		0	0.257	0.371
A3SS	1906	APH1A	APH1A	chr1	-	150267956	150268127	150267956	150267991	150268697	150269209	19	12	1	2	3	1	0	6	0	0	98	49	1	1	0.826	0.667	0.333 NA		1	-0.391
A3SS	1913	CD58	CD58	chr1	-	116519230	116519302	116519230	116519267	116521905	116521983	5	2	3	8	9	1	0	1	0	1	84	49	1	1	0.267	0.115	0.636 NA		0.368	-0.029
A3SS	1916	RBBP5	RBBP5	chr1	-	205114788	205114961	205114788	205114905	205115857	205115883	7	8	0	0	1	0	0	5	0	0	98	49	1	1	1	0.8 NA	NA		1	-0.1
A3SS	1928	CYB561D1	CYB561D1	chr1	+	109495678	109500441	109495755	109500441	109495142	109495180	2	2	0	1	0	0	0	0	0	1	98	49	1	1	0.5	1 NA	NA		0	0.75
A3SS	1938	RSRC2	RSRC2	chr12	-	122508217	122508450	122508217	122508447	122511108	122511188	0	1	0	2	1	0	0	0	0	1	98	49	1	1	0	0.485 NA	NA		0	0.242
A3SS	1939	RSRC2	RSRC2	chr12	-	122518838	122519051	122518838	122519029	122520503	122520581	0	1	0	1	0	0	0	0	1	1	71	49	1	1	0	1 NA		0	0	0.5
A3SS	1940	RSRC2	RSRC2	chr12	-	122518838	122519051	122518838	122519029	122521384	122521428	0	1	0	3	0	0	0	0	0	1	71	49	1	1	0	1 NA			0	0.5
A3SS	1954	RNF19B	RNF19B	chr1	-	32948221	32948366	32948221	32948363	32949568	32949774	23	10	2	36	23	4	0	1	0	2	52	49	1	1	0.376	0.291	0.32 NA		0.32	0.009
A3SS	1955	PITPNM1	PITPNM1	chr11	-	67496177	67496348	67496177	67496345	67497230	67497436	3	0	0	2	0	0	0	2	0	0	98	49	1	1	0.586 NA	NA	NA		1	-0.414
A3SS	1997	AES	AES	chr19	-	3052909	3054040	3052909	3054037	3054119	3054194	25	1	0	4	0	0	0	6	0	0	52	49	1	1	0.855	1 NA	NA		1	-0.073
A3SS	2001	SYNRG	SYNRG	chr17	-	37541971	37542565	37541971	37542430	37553114	37554059	16	7	0	0	1	0	1	0	0	0	98	49	1	1	1	0.778 NA		1 NA		-0.111
A3SS	2005	EWSR1	EWSR1	chr22	+	29291561	29291599	29291564	29291599	29288605	29288786	0	0	0	0	3	0	1	0	0	0	52	49	1	1 NA		0 NA		1 NA		-1
A3SS	2014	NUDT13	NUDT13	chr10	+	73126634	73126827	73126672	73126827	73125397	73125509	3	1	0	0	0	0	0	0	0	1	87	49	1	1	1	1 NA	NA		0	1
A3SS	2026	IRAK1	IRAK1	chrX	-	154013042	154013433	154013042	154013343	154014041	154014278	4	3	1	2	0	0	0	3	0	0	98	49	1	1	0.5	1	1 NA		1	-0.167
A3SS	2028	C11orf68	C11orf68	chr11	-	65916811	65918218	65916811	65918215	65918909	65919060	1	0	0	0	0	0	0	0	0	1	98	49	1	1	1 NA	NA	NA		0	1
A3SS	2032	IL4R	IL4R	chr16	+	27352539	27352696	27352544	27352696	27346466	27346618	39	6	13	1	0	0	0	4	0	0	54	49	1	1	0.973	1	1 NA		1	-0.009
A3SS	2033	ASPH	ASPH	chr8	-	61638321	61638363	61638321	61638360	61642887	61642920	4	2	1	4	0	0	0	3	0	0	52	49	1	1	0.485	1	1 NA		1	-0.172
A3SS	2034	AKTIP	AKTIP	chr16	-	53491039	53492522	53491039	53492519	53492692	53492753	0	1	1	0	0	1	0	0	0	1	52	49	1	1 NA		1	0.485 NA		0	0.742
A3SS	2046	OS9	OS9	chr12	+	57716088	57716193	57716091	57716193	57715759	57715970	7	1	0	4	1	0	0	2	0	1	52	49	1	1	0.623	0.485 NA	NA		0.653	-0.099
A3SS	2062	FOSL1	FOSL1	chr11	-	65894013	65894121	65894013	65894116	65896808	65897006	2	0	0	0	0	0	0	2	0	2	54	49	1	1	1 NA	NA	NA		0.476	0.524
A3SS	2069	ABI1	ABI1	chr10	-	26759061	26759238	26759061	26759235	26765217	26765318	4	2	1	1	3	0	0	1	0	2	52	49	1	1	0.79	0.386	1 NA		0.32	0.405
A3SS	2082	CNIH4	CNIH4	chr1	+	224371282	224371423	224371329	224371423	224365878	224365991	26	7	6	1	0	0	0	7	0	0	96	49	1	1	0.93	1	1 NA		1	-0.023
A3SS	2090	CTDSP1	CTDSP1	chr2	+	218402110	218402215	218402113	218402215	218401563	218401712	5	2	3	13	5	6	0	0	0	7	52	49	1	1	0.266	0.274	0.32 NA		0	0.287
A3SS	2100	MEF2D	MEF2D	chr1	-	156483238	156483430	156483238	156483418	156500485	156500842	12	8	1	1	1	0	5	4	0	0	61	49	1	1	0.906	0.865	1	1	1	-0.076
A3SS	2116	SNRNP70	SNRNP70	chr19	+	49107794	49108613	49107821	49108613	49107624	49107712	12	8	0	4	2	1	0	1	0	1	76	49	1	1	0.659	0.721	0 NA		0.392	0.068
A3SS	2132	TBC1D10C	TBC1D10C	chr11	+	67405902	67406017	67405906	67406017	67405594	67405701	29	7	9	1	6	0	0	7	0	2	53	49	1	1	0.964	0.519	1 NA		0.764	0.064
A3SS	2140	MRPS34	MRPS34	chr16	-	1771894	1772534	1771894	1772513	1772603	1772646	0	3	0	0	0	0	0	0	0	1	70	49	1	1 NA		1 NA	NA		0	1
A3SS	2149	ZBED5	ZBED5	chr11	-	10852703	10855118	10852703	10855086	10856143	10856257	1	2	0	2	0	0	0	1	0	0	81	49	1	1	0.232	1 NA	NA		1	-0.384
A3SS	2179	ATP6V0B	ATP6V0B	chr1	+	43977025	43978300	43977980	43978300	43976772	43976824	22	37	10	1	3	0	0	10	0	0	98	49	1	1	0.917	0.86	1 NA		1	-0.074
A3SS	2191	GLYR1	GLYR1	chr16	-	4814536	4814647	4814536	4814629	4817597	4817697	10	5	2	1	0	0	0	5	0	0	67	49	1	1	0.88	1	1 NA		1	-0.04
A3SS	2192	PRELID1	PRELID1	chr5	+	177306421	177306959	177306454	177306959	177306097	177306176	32	11	29	1	0	0	1	7	0	0	82	49	1	1	0.95	1	1	1	1	-0.017
A3SS	2193	ZDHHC20	ZDHHC20	chr13	-	21381433	21381549	21381433	21381546	21381900	21381945	1	0	0	1	0	0	0	2	0	0	52	49	1	1	0.485 NA	NA	NA		1	-0.515
A3SS	2194	ZDHHC20	ZDHHC20	chr13	-	21381433	21381549	21381433	21381546	21382919	21383009	7	0	0	1	0	0	0	6	0	3	52	49	1	1	0.868 NA	NA	NA		0.653	0.215
A3SS	2196	C8orf58	C8orf58	chr8	+	22602536	22602643	22602560	22602643	22602199	22602312	2	0	0	1	0	0	1	0	0	0	73	49	1	1	0.573 NA	NA	NA	1 NA		-0.427
A3SS	2225	CHD4	CHD4	chr12	-	6601959	6602175	6601959	6602154	6602375	6602497	18	5	0	4	3	0	0	2	0	1	70	49	1	1	0.759	0.538 NA	NA		0.583	0.066
A3SS	2226	CELF2	CELF2	chr10	+	11321188	11321386	11321206	11321386	11314138	11314258	9	8	0	6	13	8	0	1	0	3	67	49	1	1	0.523	0.31	0 NA		0.196	0.082
A3SS	2227	TIAL1	TIAL1	chr10	-	119582458	119582608	119582458	119582557	119588151	119588248	4	10	0	4	4	0	0	0	0	1	98	49	1	1	0.333	0.556 NA	NA		0	0.445
A3SS	2228	BUB3	BUB3	chr10	+	123163819	123165370	123165046	123165370	123162611	123162828	11	2	4	1	0	0	0	5	0	0	98	49	1	1	0.846	1	1 NA		1	-0.051
A3SS	2259	MGRN1	MGRN1	chr16	+	4686258	4690974	4688795	4690974	4683842	4683932	7	3	0	9	1	0	1	0	0	0	98	49	1	1	0.28	0.6 NA	NA	1 NA		-0.56
A3SS	2279	NFYC	NFYC	chr1	+	40770396	40771606	40770708	40771606	40769355	40769415	0	2	3	3	0	0	0	0	1	0	98	49	1	1	0	1	1	0 NA		0.667
A3SS	2282	TRMU	TRMU	chr22	+	46350290	46350463	46350345	46350463	46346421	46346544	3	1	0	0	1	0	0	1	0	0	98	49	1	1	1	0.333 NA	NA		1	-0.334
A3SS	2288	RTN3	RTN3	chr11	+	63718701	63721032	63718980	63721032	63681449	63681778	14	8	1	0	0	1	1	0	0	0	98	49	1	1	1	1	0.333	1 NA		-0.222
A3SS	2293	ZNF33A	ZNF33A	chr10	+	38034277	38034400	38034325	38034400	38017290	38017386	0	1	0	0	1	0	0	1	0	0	97	49	1	1 NA		0.336 NA	NA		1	-0.664
A3SS	2294	ZNF33A	ZNF33A	chr10	+	38054374	38060067	38054377	38060067	38017290	38017386	9	5	3	3	4	0	0	1	0	1	52	49	1	1	0.739	0.541	1 NA		0.485	0.275
A3SS	2296	PRF1	PRF1	chr10	-	70600363	70600932	70600363	70600906	70602644	70602775	1	3	0	0	0	0	0	0	1	0	75	49	1	1	1	1 NA		0 NA		1
A3SS	2306	ELP4	ELP4	chr11	+	31603764	31603907	31603767	31603907	31594769	31594901	1	0	0	8	0	0	0	0	0	3	52	49	1	1	0.105 NA	NA	NA		0	0.105
A3SS	2313	C10orf128	C10orf128	chr10	-	49154723	49156259	49154723	49155909	49161598	49161622	2	6	0	0	0	0	0	0	0	1	98	49	1	1	1	1 NA	NA		0	1
A3SS	2321	XRN1	XRN1	chr3	-	142332374	142332534	142332374	142332531	142332966	142333089	9	0	1	1	0	0	0	1	0	0	98	49	1	1	0.895 NA		1 NA		1	-0.052
A3SS	2328	GMPR2	GMPR2	chr14	+	24237041	24237152	24237070	24237152	24235966	24236140	19	9	0	13	5	8	0	0	0	1	78	49	1	1	0.479	0.531	0 NA		0	0.337
A3SS	2331	CIB1	CIB1	chr15	-	90232218	90232447	90232218	90232327	90233668	90233703	4	9	0	0	0	0	0	5	0	1	98	49	1	1	1	1 NA	NA		0.714	0.286
A3SS	2340	ADPGK	ADPGK	chr15	-	72751366	72752895	72751366	72752892	72755555	72755654	5	2	1	13	2	0	0	1	0	3	52	49	1	1	0.266	0.485	1 NA		0.239	0.345
A3SS	2342	TBC1D10A	TBC1D10A	chr22	-	30304530	30304651	30304530	30304630	30326672	30326966	0	0	1	3	1	0	0	1	0	0	70	49	1	1	0	0	1 NA		1	-0.667
A3SS	2348	FUT11	FUT11	chr10	+	73775418	73776218	73775540	73776218	73773189	73773815	1	0	0	2	0	0	0	0	3	0	98	49	1	1	0.2 NA	NA		0 NA		0.2
A3SS	2354	TRPC4AP	TRPC4AP	chr20	-	35021189	35021356	35021189	35021332	35035122	35035308	12	5	0	2	5	1	0	3	0	1	73	49	1	1	0.801	0.402	0 NA		0.668	-0.267
A3SS	2356	PNPLA6	PNPLA6	chr19	+	7553871	7554015	7553874	7554015	7551361	7551437	4	1	1	3	1	2	0	3	0	1	52	49	1	1	0.557	0.485	0.32 NA		0.739	-0.285
A3SS	2365	IFIT1	IFIT1	chr10	+	89399889	89400003	89399894	89400003	89392545	89392717	2	0	0	0	0	0	0	0	1	1	54	49	1	1	1 NA	NA		0	0	1
A3SS	2377	RNH1	RNH1	chr11	-	502061	502249	502061	502181	504823	504996	2	0	0	1	2	0	0	5	0	2	98	49	1	1	0.5	0 NA	NA		0.556	-0.306
A3SS	2379	RNH1	RNH1	chr11	-	502061	502249	502061	502181	507112	507283	2	0	0	0	1	0	0	5	0	0	98	49	1	1	1	0 NA	NA		1	-0.5
A3SS	2392	BAZ2B	BAZ2B	chr2	-	159448241	159448409	159448241	159448403	159453612	159453801	43	9	0	6	1	0	0	5	0	0	55	49	1	1	0.865	0.889 NA	NA		1	-0.123
A3SS	2393	BAZ2B	BAZ2B	chr2	-	159448241	159448409	159448241	159448403	159478574	159478721	8	7	0	2	0	0	0	5	0	0	55	49	1	1	0.781	1 NA	NA		1	-0.109
A3SS	2405	SEC14L1	SEC14L1	chr17	+	77213917	77217099	77216548	77217099	77211949	77212201	164	43	8	0	2	0	8	10	0	0	98	49	1	1	1	0.915	1	1	1	-0.028
A3SS	2406	CLEC16A	CLEC16A	chr16	+	10972931	10973061	10972937	10973061	10971124	10971230	0	0	0	5	3	0	0	2	0	0	55	49	1	1	0	0 NA	NA		1	-1
A3SS	2407	GBF1	GBF1	chr10	+	102359266	102359435	102359269	102359435	102358505	102358729	1	0	0	4	0	0	0	0	0	1	52	49	1	1	0.191 NA	NA	NA		0	0.191
A3SS	2413	CALHM2	CALHM2	chr10	-	103451082	103451285	103451082	103451182	103452155	103452405	0	0	0	2	0	0	0	2	0	2	98	49	1	1	0 NA	NA	NA		0.333	-0.333
A3SS	2417	ANKRD11	ANKRD11	chr16	-	89316932	89317078	89316932	89317075	89418283	89418368	0	2	0	0	5	0	1	2	0	0	52	49	1	1 NA		0.274 NA		1	1	-0.726
A3SS	2424	PICALM	PICALM	chr11	-	85990249	85990399	85990249	85990378	85996825	85996929	125	58	10	4	3	0	0	18	0	0	70	49	1	1	0.956	0.931	1 NA		0.926	0.036
A3SS	2426	FAM89B	FAM89B	chr11	+	65573352	65574198	65573362	65574198	65572348	65572960	3	3	0	1	1	2	0	1	0	1	59	49	1	1	0.714	0.714	0 NA		0.454	0.022
A3SS	2440	TBCE	TBCE	chr1	+	235419416	235419561	235419472	235419561	235414432	235414618	1	0	0	0	1	0	1	0	0	0	98	49	1	1	1	0 NA		1 NA		-0.5
A3SS	2474	LST1	LST1	chr6	+	31588496	31588909	31588517	31588909	31587199	31587318	23	4	4	46	21	20	0	3	0	4	70	49	1	1	0.259	0.118	0.123 NA		0.344	-0.177
A3SS	2495	TUBGCP2	TUBGCP2	chr10	-	133283077	133283221	133283077	133283184	133283881	133284002	14	3	4	1	0	0	1	6	0	0	86	49	1	1	0.889	1	1	1	1	-0.037
A3SS	2514	PIK3R5	PIK3R5	chr17	-	8888170	8888953	8888170	8888891	8889138	8889222	2	1	1	10	5	7	0	2	0	2	98	49	1	1	0.091	0.091	0.067 NA		0.333	-0.25
A3SS	2516	ZSWIM8	ZSWIM8	chr10	+	73801016	73801195	73801084	73801195	73800639	73800759	14	6	1	2	0	0	0	2	0	0	98	49	1	1	0.778	1	1 NA		1	-0.074
A3SS	2517	GCH1	GCH1	chr14	-	54842005	54844143	54842005	54843054	54845767	54845852	4	0	0	0	0	0	0	2	0	2	98	49	1	1	1 NA	NA	NA		0.333	0.667
A3SS	2532	LYPLAL1	LYPLAL1	chr1	+	219193081	219193251	219193129	219193251	219179146	219179246	7	2	3	0	2	0	0	1	0	0	97	49	1	1	1	0.336	1 NA		1	-0.221
A3SS	2560	PPP1CA	PPP1CA	chr11	-	67401067	67401232	67401067	67401199	67401727	67401905	2	0	0	18	9	4	0	0	1	4	82	49	1	1	0.062	0	0	0	0	0.021
A3SS	2564	ATF7IP	ATF7IP	chr12	+	14434336	14434423	14434339	14434423	14423908	14425473	25	2	2	6	1	0	0	6	0	3	52	49	1	1	0.797	0.653	1 NA		0.653	0.164
A3SS	2571	SLC25A29	SLC25A29	chr14	-	100295625	100295993	100295625	100295697	100298841	100298885	1	1	0	2	0	0	3	0	0	0	98	49	1	1	0.2	1 NA		1 NA		-0.4
A3SS	2573	KCNK7	KCNK7	chr11	-	65592854	65593383	65592854	65593210	65593475	65593874	8	4	0	3	0	0	0	5	0	0	98	49	1	1	0.571	1 NA	NA		1	-0.215
A3SS	2575	SUPT7L	SUPT7L	chr2	-	27660983	27661601	27660983	27661388	27662178	27662281	0	1	0	0	1	0	0	0	0	1	98	49	1	1 NA		0.333 NA	NA		0	0.333
A3SS	2583	ADAR	ADAR	chr1	-	154596804	154596995	154596804	154596938	154597122	154597267	120	43	27	1	0	0	2	42	0	0	98	49	1	1	0.984	1	1	1	1	-0.005
A3SS	2594	PTMA	PTMA	chr2	+	231711886	231711983	231711889	231711983	231711347	231711419	5	1	0	7	0	0	0	1	0	2	52	49	1	1	0.402	1 NA	NA		0.32	0.381
A3SS	2604	LILRB2	LILRB2	chr19	-	54276806	54276979	54276806	54276929	54277549	54277597	3	0	0	9	1	2	0	1	0	3	98	49	1	1	0.143	0	0 NA		0.143	-0.095
A3SS	2615	ANKRD42	ANKRD42	chr11	+	83212652	83215200	83212978	83215200	83211294	83211430	3	0	0	0	0	0	0	0	0	1	98	49	1	1	1 NA	NA	NA		0	1
A3SS	2623	TNFSF13	TNFSF13	chr17	+	7559845	7559893	7559850	7559893	7558291	7559297	1	1	0	2	0	0	0	0	0	1	54	49	1	1	0.312	1 NA	NA		0	0.656

A3SS	2624	TNFSF13	TNFSF13	chr17	+	7559847	7559893	7559850	7559893	7558291	7559297	0	1	0	2	0	0	0	0	0	1	52	49	1	1	0	1	NA	NA	0	0.5
A3SS	2633	SLC43A3	SLC43A3	chr11	-	57425988	57426389	57425988	57426352	57426587	57426652	0	0	0	0	1	0	0	2	0	0	86	49	1	1	NA	0	NA	NA	1	-1
A3SS	2638	FAM111A	FAM111A	chr11	+	59148796	59148953	59148835	59148953	59145826	59145856	9	0	3	2	2	0	0	0	1	0	88	49	1	1	0.715	0	1	0	NA	0.572
A3SS	2657	PPP6R3	PPP6R3	chr11	+	68587926	68588024	68587944	68588024	68575957	68576043	2	3	3	0	0	0	0	0	0	1	67	49	1	1	1	1	1	NA	0	1
A3SS	2658	PPP6R3	PPP6R3	chr11	+	68587926	68588024	68587944	68588024	68583042	68583129	10	9	3	4	0	0	0	3	0	0	67	49	1	1	0.646	1	1	NA	1	-0.118
A3SS	2665	IFIT3	IFIT3	chr10	+	89337420	89337542	89337424	89337542	89332478	89332627	2	4	0	0	1	0	0	3	0	0	53	49	1	1	1	0.787	NA	NA	1	-0.107
A3SS	2671	KANSL1	KANSL1	chr17	-	46034160	46034285	46034160	46034282	46038537	46038686	13	1	0	4	0	0	1	1	0	0	52	49	1	1	0.754	1	NA	1	1	-0.123
A3SS	2676	THEMIS2	THEMIS2	chr1	+	27881970	27883043	27882512	27883043	27879643	27880054	191	107	53	1	0	0	17	67	0	0	98	49	1	1	0.99	1	1	1	1	-0.003
A3SS	2677	PIGT	PIGT	chr20	+	45419472	45419590	45419503	45419590	45419294	45419395	1	0	0	1	2	0	0	0	0	2	80	49	1	1	0.38	0	NA	NA	0	0.19
A3SS	2686	ZYX	ZYX	chr7	+	143381556	143381779	143381559	143381779	143381266	143381409	1	0	4	1	0	0	1	1	1	0	52	49	1	1	0.485	NA	1	0.485	1	0
A3SS	2701	CD300LF	CD300LF	chr17	-	74703034	74703143	74703034	74703098	74704477	74704816	15	4	1	22	14	3	0	2	0	4	94	49	1	1	0.262	0.13	0.148	NA	0.207	-0.027
A3SS	2703	ANAPC15	ANAPC15	chr11	-	72111156	72111286	72111156	72111281	72111411	72111496	1	0	0	1	0	0	0	1	0	0	54	49	1	1	0.476	NA	NA	NA	1	-0.524
A3SS	2714	MAP4K2	MAP4K2	chr11	-	64797103	64797192	64797103	64797168	64797274	64797380	10	5	6	2	0	0	0	3	0	0	73	49	1	1	0.77	1	1	NA	1	-0.077
A3SS	2721	ZNF276	ZNF276	chr16	+	89737720	89737905	89737805	89737905	89733920	89734038	2	1	0	7	2	0	1	1	0	5	98	49	1	1	0.125	0.2	NA	1	0.091	-0.383
A3SS	2729	ST20	ST20	chr15	-	79907628	79907706	79907628	79907684	79914587	79915256	2	1	0	9	0	0	0	1	0	5	71	49	1	1	0.133	1	NA	NA	0.121	0.446
A3SS	2735	CTC1	CTC1	chr17	-	8237374	8237519	8237374	8237414	8238030	8238242	7	6	0	0	1	0	0	3	0	0	98	49	1	1	1	0.75	NA	NA	1	-0.125
A3SS	2738	PAM	PAM	chr5	+	103028181	103028238	103028184	103028238	103025130	103025334	0	0	0	1	0	0	0	1	0	0	52	49	1	1	0	NA	NA	NA	1	-1
A3SS	2749	LRRC75A-AS1	LRRC75A-AS1	chr17	+	16439527	16439703	16439659	16439703	16438986	16439414	28	10	8	2	0	0	1	4	0	0	98	49	1	1	0.875	1	1	1	1	-0.042
A3SS	2750	LRRC75A-AS1	LRRC75A-AS1	chr17	+	16439580	16439703	16439659	16439703	16438986	16439414	28	10	8	2	0	0	1	4	0	0	98	49	1	1	0.875	1	1	1	1	-0.042
A3SS	2751	LRRC75A-AS1	LRRC75A-AS1	chr17	+	16440110	16440253	16440184	16440253	16439326	16439414	5	0	0	1	1	0	0	2	0	1	98	49	1	1	0.714	0	NA	NA	0.5	-0.143
A3SS	2752	LRRC75A-AS1	LRRC75A-AS1	chr17	+	16440110	16440253	16440184	16440253	16439659	16439703	5	0	0	2	2	0	0	2	0	0	98	49	1	1	0.556	0	NA	NA	1	-0.722
A3SS	2763	MTA1	MTA1	chr14	+	105464415	105464567	105464462	105464567	105464031	105464147	2	0	0	0	1	0	0	1	0	0	96	49	1	1	1	0	NA	NA	1	-0.5
A3SS	2764	LGALS9	LGALS9	chr17	+	27640571	27640773	27640642	27640773	27638262	27638354	21	4	6	1	0	0	2	25	0	0	98	49	1	1	0.913	1	1	1	1	-0.029
A3SS	2788	LILRA1	LILRA1	chr19	+	54594196	54594476	54594440	54594476	54593575	54593781	0	3	1	11	6	4	0	0	0	3	98	49	1	1	0	0.2	0.111	NA	0	0.104
A3SS	2813	UBXN1	UBXN1	chr11	-	62676497	62677005	62676497	62676639	62677517	62677634	9	11	6	1	0	0	0	4	0	0	98	49	1	1	0.818	1	1	NA	1	-0.061
A3SS	2820	ATP6V0A1	ATP6V0A1	chr17	+	42477638	42477742	42477659	42477742	42470089	42470218	1	3	3	5	1	0	0	0	0	1	70	49	1	1	0.123	0.677	1	NA	0	0.6
A3SS	2826	BRCA1	BRCA1	chr17	-	43076487	43076614	43076487	43076611	43082403	43082575	1	4	0	0	0	0	0	0	0	1	52	49	1	1	1	1	NA	NA	0	1
A3SS	2843	PHOSPHO1	PHOSPHO1	chr17	-	49223369	49225131	49223369	49225004	49226646	49226758	42	14	11	5	1	8	0	4	0	0	98	49	1	1	0.808	0.875	0.407	NA	0.667	0.03
A3SS	2854	MAP7D1	MAP7D1	chr1	+	36179511	36179557	36179514	36179557	36179261	36179315	2	4	0	26	18	14	0	0	0	3	52	49	1	1	0.068	0.173	0	NA	0	0.08
A3SS	2881	ZNF200	ZNF200	chr16	-	3222324	3224613	3222324	3224610	3232420	3232547	2	3	0	0	2	0	0	1	0	0	52	49	1	1	1	0.586	NA	NA	1	-0.207
A3SS	2895	RHBDF2	RHBDF2	chr17	-	76479732	76479941	76479732	76479854	76481374	76481545	2	1	0	8	3	2	0	1	0	5	98	49	1	1	0.111	0.143	0	NA	0.091	-0.006
A3SS	2898	PGS1	PGS1	chr17	+	78392475	78392665	78392513	78392665	78378617	78378808	34	19	3	2	0	0	0	15	0	0	87	49	1	1	0.905	1	1	NA	1	-0.032
A3SS	2906	TYMP	TYMP	chr22	-	50529495	50529754	50529495	50529731	50529903	50530085	23	17	2	2	0	0	2	4	0	1	72	49	1	1	0.887	1	1	1	0.731	0.097
A3SS	2921	IL16	IL16	chr15	+	81306419	81306545	81306422	81306545	81305907	81306166	5	5	5	13	3	5	0	1	0	1	52	49	1	1	0.266	0.611	0.485	NA	0.485	-0.031
A3SS	2923	HSDL1	HSDL1	chr16	-	84129985	84130431	84129985	84130171	84131101	84131327	1	1	0	0	1	0	0	3	0	0	98	49	1	1	1	0.333	NA	NA	1	-0.334
A3SS	2929	HPS4	HPS4	chr22	-	26470718	26470922	26470718	26470813	26472301	26472418	1	0	0	1	1	0	1	0	0	0	98	49	1	1	0.333	0	NA	1	NA	-0.834
A3SS	2930	PPP4R1	PPP4R1	chr18	-	9595017	9595153	9595017	9595102	9614225	9614270	10	4	3	9	0	2	0	4	0	0	98	49	1	1	0.357	1	0.429	NA	1	-0.405
A3SS	2940	CARS	CARS	chr11	-	3040895	3041031	3040895	3040984	3042164	3042256	3	4	2	8	4	0	0	0	0	3	96	49	1	1	0.161	0.338	1	NA	0	0.5
A3SS	2966	DYRK1A	DYRK1A	chr21	+	37478180	37478300	37478207	37478300	37472683	37472880	6	4	0	24	4	2	0	0	0	3	76	49	1	1	0.139	0.392	0	NA	0	0.177
A3SS	2969	GPX4	GPX4	chr19	+	1106377	1106459	1106399	1106459	1106241	1106266	1	1	0	10	9	1	0	0	0	2	71	49	1	1	0.065	0.071	0	NA	0	0.045
A3SS	2980	RPL32	RPL32	chr3	-	12840141	12840341	12840141	12840242	12841493	12841582	7	2	0	1	0	1	0	1	0	1	98	49	1	1	0.778	1	0	NA	0.333	0.26
A3SS	3006	KIAA0226L	KIAA0226L	chr13	-	46371940	46372597	46371940	46372210	46387133	46387500	28	7	2	0	0	2	0	2	0	0	98	49	1	1	1	1	0.333	NA	1	-0.222
A3SS	3014	IRF5	IRF5	chr7	+	128947229	128947535	128947277	128947535	128947022	128947056	1	0	0	1	1	0	0	1	0	1	97	49	1	1	0.336	0	NA	NA	0.336	-0.168
A3SS	3025	ATG4D	ATG4D	chr19	+	10544708	10544866	10544782	10544866	10543893	10544325	3	2	0	1	0	0	2	1	0	0	98	49	1	1	0.6	1	NA	1	1	-0.2
A3SS	3031	PRKCSH	PRKCSH	chr19	+	11447692	11447789	11447722	11447789	11447438	11447618	7	1	1	1	0	0	0	1	0	0	79	49	1	1	0.813	1	1	NA	1	-0.062
A3SS	3040	TNPO2	TNPO2	chr19	-	12702071	12702177	12702071	12702147	12702822	12702918	0	0	0	2	0	0	1	0	0	0	79	49	1	1	0	NA	NA	1	NA	-1
A3SS	3066	AUP1	AUP1	chr2	-	74526940	74527121	74526940	74527059	74527247	74527363	7	1	0	6	1	1	0	0	0	3	98	49	1	1	0.368	0.333	0	NA	0	0.234
A3SS	3086	CDV3	CDV3	chr3	+	133587158	133590274	133587161	133590274	133586562	133586722	11	3	2	3	3	1	0	4	0	1	52	49	1	1	0.776	0.485	0.653	NA	0.79	-0.152
A3SS	3088	CDV3	CDV3	chr3	+	133587158	133590274	133588352	133590274	133586562	133586722	35	8	22	1	0	0	0	9	0	0	98	49	1	1	0.946	1	1	NA	1	-0.018
A3SS	3090	CDV3	CDV3	chr3	+	133587161	133590274	133588352	133590274	133586562	133586722	28	8	21	1	0	0	0	9	0	0	98	49	1	1	0.933	1	1	NA	1	-0.022
A3SS	3091	CDV3	CDV3	chr3	+	133587895	133590274	133588352	133590274	133586562	133586722	36	6	20	1	0	0	0	8	0	0	98	49	1	1	0.947	1	1	NA	1	-0.018
A3SS	3093	ZNF493	ZNF493	chr19	+	21405757	21405856	21405760	21405856	21405128	21405255	0	2	0	0	3	1	0	1	0	0	52	49	1	1	NA	0.386	0	NA	1	-0.807
A3SS	3094	ING4	ING4	chr12	-	6652661	6652770	6652661	6652767	6652935	6653050	2	0	0	2	2	0	0	2	0	2	52	49	1	1	0.485	0	NA	NA	0.485	-0.242
A3SS	3095	ING4	ING4	chr12	-	6652661	6652770	6652661	6652767	6652944	6653050	2	0	0	1	0	2	0	1	0	0	52	49	1	1	0	NA	0	NA	1	-1
A3SS	3096	TMEM150B	TMEM150B	chr19	-	55320968	55321093	55320968	55321044	55322647	55322743	1	0	0	0	0	0	0	0	0	1	98	49	1	1	1	NA	NA	NA	0	1
A3SS	3105	CUTA	CUTA	chr6	-	33417480	33417816	33417480	33417695	33417958	33418288	3	1	0	0	1	0	1	1	0	1	98	49	1	1	1	0.333	NA	1	0.333	0
A3SS	3119	AZI2	AZI2	chr3	-	28331688	28331954	28331688	28331950	28332368	28332427	1	0	0	2	0	0	0	2	0	0	53	49	1	1	0.316	NA	NA	NA	1	-0.684
A3SS	3121	HCST	HCST	chr19	+	35904119	35904271	35904122	35904271	35903771	35903903	11	2	0	3	0	0	1	5	0	0	52	49	1	1	0.776	1	NA	1	1	-0.112
A3SS	3128	INO80E	INO80E	chr16	+	30001211	30001530	30001413	30001530	30000928	30001040	4	1	1	5	0	0	0	1	0	0	98	49	1	1	0.286	1	1	NA	1	-0.238
A3SS	3140	DDX60L	DDX60L	chr4	-	168395958	168396127	168395958	168396124	168400825	168400978	17	31	0	38	0	0	0	9	2	12	52	49	1	1	0.297	1	NA	0	0.414	0.442
A3SS	3147	TMEM91	TMEM91	chr19	+	41383560	41384082	41383714	41384082	41382771	41382921	1	5	1	0	1	0	1	1	0	0	98	49	1	1	1	0.714	1	1	1	-0.095
A3SS	3151	TMEM91	TMEM91	chr19	+	41383714	41384082	41383862	41384082	41382771	41382921	5	1	1	1	4	10	0	2	0	3	98	49	1	1	0.714	0.111	0.048	NA	0.25	0.041
A3SS	3152	TMEM91	TMEM91	chr19	+	41383714	41384082	41383873	41384082	41382771	41382921	2	1	5	0	3	0	0	2	0	0	98	49	1	1	1	0.143	1	NA	1	-0.286
A3SS	3154	TMEM91	TMEM91	chr19	+	41383862	41384082	41383873	41384082	41382771	41382921	1	4	14	0	3	0	0	3	0	0	60	49	1	1	1	0.521	1	NA	1	-0.16
A3SS	3162	APBB3	APBB3	chr5	-	140561586	140561722	140561586	140561701	140562099	140562227	3	0	0	2	2	0	0	1	0	0	70	49	1	1	0.512	0	NA	NA	1	-0.744
A3SS	3163	APBB3	APBB3	chr5	-	140561586	140561728	140561586	140561701	140562099	140562227	5	0	0	2	2	0	0	1	0	0	76	49	1	1	0.617	0	NA	NA	1	-0.692
A3SS	3175	DGCR2	DGCR2	chr22	-	19064847	19065075	19064847	19065067	19068099	19068225	4	0	0	14	8	0	0	3	1	4	57	49	1	1	0.197	0	NA	0	0.392	-0.098
A3SS	3190	AGFG1	AGFG1	chr2	+	227553703	227553795	227553709	227553795	227551958	227552117	7	7	4	0	0	0	0	2	0	4	55	49	1	1	1	1	1	NA	0.308	0.692
A3SS	3194	RPL18	RPL18	chr19	-	48617315	48617423	48617315	48617415	48617790	48617877	41	10	1	1	0	0	0	7	0	0	57	49	1	1	0.972	1	1	NA	1	-0.009
A3SS	3200	PTOV1	PTOV1	chr19	+	49860565	49860744	49860613	49860744	49860267	49860336	9	4	0	6	1	2	0	3	0	3	97	49	1	1	0.431	0.669	0	NA	0.336	0.031
A3SS	3210	EIF2AK1	EIF2AK1	chr7	-	6042932	6042993	6042932	6042990	6044561	6044661	5	0	0	0	3	0	0	3	0	0	52	49	1	1	1	0	NA	NA	1	-0.5
A3SS	3213	ALG13	ALG13	chrX	+	111682131	111682294	111682235	111682294	111681169	111681424	2	5	0	0	0	0	0	0	2	0	98	49	1	1	1	1	NA	0	NA	1
A3SS	3216	LY6E	LY6E	chr8	+	143020882	143020991	143020887	143020991	143018484	143018586	1	2	0	0	0	0	0	2	0	1	54	49	1	1	1	1	NA	NA	0.645	0.355
A3SS	3218	ENTPD1	ENTPD1	chr10	+	95847445	95847706	95847568	95847706	95845356	95845596	82	29	11	0	0	0	4	26	0	1	98	49	1	1	1	1	1	1	0.929	0.035
A3SS	3257	LILRB1	LILRB1	chr19	+	54633970	54634021	54633979	54634021	54633637	54633688	4	1	0	1	0	0	0	0	0	3	52	49	1	1	0.79	1	NA	NA	0	0.895
A3SS	3319	EPN1	EPN1	chr19	+	55692950	55693037	55692953	55693037	55692685	55692796	5	2	0	2	0	0	0	1	0	0	98	49	1	1	0.702	1	NA	NA	1	-0.149
A3SS	3337	ODC1	ODC1	chr2	-	10444473	10444647	10444473	10444547	10444930	10445049	11	0	4	1	0	0	0	2	0	1	98	49	1	1	0.846	NA	1	NA	0.5	0.423
A3SS	3350	EDF1	EDF1	chr9	-	136862113	136862496	136862113	136862345	136862905	136862999	7	5	0	16	2	3	0	3	0	1	98	49	1	1	0.179	0.556	0	NA	0.6	-0.355
A3SS	3351	NCOA1	NCOA1	chr2	+	24768220	24770701	24768223	24770701	24762686	24762776	4	6	0	1	0	0	0	2	0	0	52	49	1	1	0.79	1	NA	NA	1	-0.105
A3SS	3352	NCOA1	NCOA1	chr2	+	24768220	24770701	24768223	24770701	24768078	24768135	17	7	0	2	3	0	0	5	0	0	52	49	1	1	0.889	0.687	NA	NA	1	-0.212
A3SS	3355	ACIN1	ACIN1	chr14	-	23064354	23064488	23064354	23064485	23065965	23066008	11	2	0	4	2	0	0	2	0	0	52	49	1	1	0.722	0.485	NA	NA	1	-0.397
A3SS	3358	BRD9	BRD9	chr5	-	889586	889766	889586	889647	891154	891287	9	1	0	1	3	0	2	2	0	2	98	49	1	1	0.818	0.143	NA	1	0.333	-0.186
A3SS	3367	PPM1B	PPM1B	chr2	+	44229992	44231473	44230412	44231473	44218479	44218537	5	2	0	3	1	0	0	3	0	2	98	49	1	1	0.455	0.5	NA	NA	0.429	0.049
A3SS	3368	FBXO7	FBXO7	chr22	+	32478980	32479275	32479132	32479275	32474719	32475124	8	6	0	0	1	0	1	1	0	0	98	49	1	1	1	0.75	NA	1	1	-0.125
A3SS	3370	SH2D2A	SH2D2A	chr1	-	156814204	156814324	156814204	156814294	156815036	156815221	1	0	0	2	0	0	0	2	0	1	79	49	1	1	0.237	NA	NA	NA	0.554	-0.317
A3SS	3373	GNAS	GNAS	chr20	+	58903527	58903585	58903530	58903585	58898940	58898985	9	8	5	8	5	0	0	2	0	1	52	49	1	1	0.515	0.601	1	NA	0.653	0.052
A3SS	3379	CCDC88A	CCDC88A	chr2	-	55328293	55328435	55328293	55328432	55332565	55332693	1	0	0	2	0	0	0	1	0	1	52	49	1	1	0.32	NA	NA	NA	0.485	-0.165
A3SS	3382	VPS54	VPS54	chr2	-	63981645	63981887	63981645	63981851	63983863	63984019	0	0	0	3	0	0	2	1	0	0	85	49	1	1	0	NA	NA	1	1	-1
A3SS	3384	AFTPH	AFTPH	chr2	+	64591884	64593004	64591887	64593004	64585421	64585545	5	1	1	2	4	0	0	1	0	0	52	49	1	1	0.702	0.191	1	NA	1	-0.369
A3SS	3395	PSMA3-AS1	PSMA3-AS1	chr14	-	58273978	58274135	58273978	58274010	58291634	58291707	12	6	4	1	3	1	0	0	0	2	98	49	1	1	0.857	0.5	0.667	NA	0	0.675
A3SS	3400	TGOLN2	TGOLN2	chr2	-	85318017	85322758	85318017	85322741	85324914	85324998	5	0	0	17	6	0	0	0	0	2	66	49	1	1	0.179	0	NA	NA	0	0.089
A3SS	3401	TGOLN2	TGOLN2	chr2	-	85318017	85322799	85318017	85322741	85324914	85324998	10	3	0	17	6	0	0	0	0	2	98	49	1	1	0.227	0.2	NA	NA	0	0.214
A3SS	3414	MAP4K4	MAP4K4	chr2	+	101859642	101859864	101859735	101859864	101858995	101859082	24	14	10	14	4	4	0	4	0	1	98	49	1	1	0.462	0.636	0.556	NA	0.667	-0.116
A3SS	3422	UXS1	UXS1	chr2	-	106164735	106164799	106164735	106164784	106166055	106166083	5	4	0	4	4	6	0	1	0	0	64	49	1	1	0.489	0.434	0	NA	1	-0.692
A3SS	3431	MICU1	MICU1	chr10	-	72508154	72508275	72508154	72508269	72533745	72533789	4	0	0	7	5	5	0	0	0	2	55	49	1	1	0.337	0	0	NA	0	0.112
A3SS	3441	ITGAL	ITGAL	chr16	+	30505243	30505300	30505246	30505300	30504174	30504264	9	2	7	4	11	0	0	8	0	2	52	49	1	1	0.68	0.146	1	NA	0.79	-0.181
A3SS	3448	SP3	SP3	chr2	-	173963760	173963883	173963760	173963874	173964404	173964553	10	5	1	0	0	0	0	2	0	1	58	49	1	1	1	1	1	NA	0.628	0.372
A3SS	3450	ATF2	ATF2	chr2	-	175072249	175074867	175072249	175074835	175080659	175080765	3	0	0	5	2	1	0	1	0	0	81	49	1	1	0.266	0	0	NA	1	-0.911
A3SS	3462	WDR13	WDR13	chrX	+	48598716	48598957	48598750	48598957	48597491	48598037	3	4	3	1	0	0	0	3	0	0	83	49	1	1	0.639	1	1	NA	1	-0.12
A3SS	3469	DCTN3	DCTN3	chr9	-	34614041	34614132	34614041	34614101	34614709	34614768	0	5	0	3	4	1	1	0	0	0	80	49	1	1	0	0.434	0	1	NA	-0.855
A3SS	3470	DCTN3	DCTN3	chr9	-	34614041	34614198	34614041	34614101	34614709	34614768	0	5	0	3	4	1	1	0	0	0	98	49	1	1	0	0.385	0	1	NA	-0.872
A3SS	3485	SPATA20	SPATA20	chr17	+	50547664	50547767	50547719	50547767	50547088	50547285	0	0	0	2	0	0	0	1	0	0	98	49	1	1	0	NA	NA	NA	1	-1
A3SS	3513	SERPINB1	SERPINB1	chr6	-	2835855	2836023	2835855	2836019	2836107	2836250	58	34	28	4	1	2	0	15	0	3	53	49	1	1	0.931	0.969	0.928	NA	0.822	0.121
A3SS	3514	USP19	USP19	chr3	-	49115723	49115944	49115723	49115938	49116046	49116162	3	1	0	1	3	0	0	3	0	0	55	49	1	1	0.728	0.229	NA	NA	1	-0.522
A3SS	3515	RFC1	RFC1	chr4	-	39306591	39306704	39306591	39306701	39308635	39309032	1	0	0	1	2	0	0	1	0	0	52	49	1	1	0.485	0	NA	NA	0	0.242
A3SS	3524	NSFL1C	NSFL1C	chr20	-	1454966	1455132	1454966	1455037	1458199	1458274	19	10	12	2	0	0	0	6	0	1	98	49	1	1	0.826	1	1	NA	0.75	0.192
A3SS	3566	HCK	HCK	chr20	+	32073715	32073818	32073718	32073818	32073318	32073361	45	35	39	12	4	8	0	6	0	0	52	49	1	1	0.779	0.892	0.821	NA	1	-0.169
A3SS	3575	RALGAPB	RALGAPB	chr20	+	38565355	38565478	38565358	38565478	38562531	38562697	1	2	1	0	0	0	0	0	0	2	52	49	1	1	1	1	1	NA	0	1
A3SS	3586	SH3BP5	SH3BP5	chr3	-	15304102	15304299	15304102	15304231	15330503	15330566	3	2	0	7	2	0	0	2	0	2	98	49	1	1	0.176	0.333	NA	NA	0.333	-0.079
A3SS	3587	ELMO2	ELMO2	chr20	-	46394404	46394532	46394404	46394503	46398696	46398771	5	2	0	1	2	0	0	0	0	1	78	49	1	1	0.759	0.386	NA	NA	0	0.573
A3SS	3591	NCOA3	NCOA3	chr20	+	47634017	47634195	47634047	47634195	47633495	47633636	11	3	0	12	2	1	0	0	0	1	79	49	1	1	0.362	0.482	0	NA	0	0.281
A3SS	3592	NCOA3	NCOA3	chr20	+	47639576	47639822	47639621	47639822	47639007	47639202	15	14	0	4	1	0	0	11	0	0	94	49	1	1	0.662	0.879	NA	NA	1	-0.23
A3SS	3596	STAU1	STAU1	chr20	-	49124374	49124605	49124374	49124587	49135832	49135931	4	1	0	8	1	1	0	3	0	0	67	49	1	1	0.268	0.422	0	NA	1	-0.77
A3SS	3603	ZBP1	ZBP1	chr20	-	57613162	57613330	57613162	57613327	57614886	57615060	27	4	5	0	0	0	0	23	0	2	52	49	1	1	1	1	1	NA	0.916	0.084
A3SS	3606	OSBPL2	OSBPL2	chr20	+	62259980	62260125	62260016	62260125	62256056	62256221	43	24	12	1	0	0	2	13	0	0	85	49	1	1	0.961	1	1	1	1	-0.013
A3SS	3608	PPP1R12C	PPP1R12C	chr19	-	55092782	55092868	55092782	55092865	55093015	55093076	2	2	0	8	5	0	0	0	0	1	52	49	1	1	0.191	0.274	NA	NA	0	0.233
A3SS	3609	PPP1R12C	PPP1R12C	chr19	-	55095444	55095603	55095444	55095600	55095866	55095940	7	0	0	6	2	2	0	1	0	0	52	49	1	1	0.524	0	0	NA	1	-0.825
A3SS	3623	PCMTD2	PCMTD2	chr20	+	64267866	64268010	64267967	64268010	64265257	64265429	14	0	4	3	0	0	0	1	0	0	98	49	1	1	0.7	NA	1	NA	1	-0.15
A3SS	3649	POFUT2	POFUT2	chr21	-	45263927	45267713	45263927	45265635	45269838	45270019	8	6	0	1	0	0	0	3	0	0	98	49	1	1	0.8	1	NA	NA	1	-0.1
A3SS	3684	GUCD1	GUCD1	chr22	-	24543841	24544083	24543841	24543954	24546913	24547005	23	17	7	2	0	0	2	9	0	0	98	49	1	1	0.852	1	1	1	1	-0.049
A3SS	3706	SIGLEC10	SIGLEC10	chr19	-	51416665	51416950	51416665	51416806	51417081	51417465	33	12	5	2	0	0	0	5	0	0	98	49	1	1	0.892	1	1	NA	1	-0.036
A3SS	3711	TOM1	TOM1	chr22	+	35322998	35323177	35323027	35323177	35317876	35317961	7	4	0	1	2	0	1	5	0	1	78	49	1	1	0.815	0.557	NA	1	0.759	-0.194
A3SS	3717	APOL3	APOL3	chr22	-	36149045	36149371	36149045	36149142	36160668	36160929	2	0	0	9	0	0	1	0	0	0	98	49	1	1	0.1	NA	NA	1	NA	-0.9
A3SS	3731	PPP6R2	PPP6R2	chr22	+	50419345	50419462	50419348	50419462	50418866	50418979	0	0	0	3	2	0	1	0	0	1	52	49	1	1	0	0	NA	1	0	-0.5
A3SS	3732	PPP6R2	PPP6R2	chr22	+	50439697	50439857	50439700	50439857	50438598	50438762	1	0	0	3	3	0	0	1	0	2	52	49	1	1	0.239	0	NA	NA	0.32	-0.201
A3SS	3756	SLC6A6	SLC6A6	chr3	+	14443623	14443863	14443627	14443863	14416411	14416453	106	28	36	6	0	0	0	19	0	1	53	49	1	1	0.942	1	1	NA	0.946	0.035
A3SS	3763	PDCD6IP	PDCD6IP	chr3	+	33828837	33828969	33828852	33828969	33826479	33826580	3	1	0	1	0	0	0	2	0	0	64	49	1	1	0.697	1	NA	NA	1	-0.151
A3SS	3777	CTNNB1	CTNNB1	chr3	+	41239659	41240448	41239818	41240448	41239133	41239354	18	10	6	1	0	1	0	11	0	0	98	49	1	1	0.9	1	0.75	NA	1	-0.117
A3SS	3797	NCOR1	NCOR1	chr17	-	16101249	16101805	16101249	16101757	16108785	16108912	29	5	2	1	0	0	1	2	0	0	97	49	1	1	0.936	1	1	1	1	-0.021
A3SS	3809	ENY2	ENY2	chr8	+	109340443	109340563	109340488	109340563	109339319	109339390	0	1	0	6	7	3	1	1	0	1	94	49	1	1	0	0.069	0	1	0.343	-0.648
A3SS	3810	ENY2	ENY2	chr8	+	109340443	109340563	109340490	109340563	109339319	109339390	0	2	0	3	0	0	1	2	0	0	96	49	1	1	0	1	NA	1	1	-0.5
A3SS	3811	ENY2	ENY2	chr8	+	109340488	109340563	109340490	109340563	109339319	109339390	6	8	3	3	0	0	0	2	0	0	51	49	1	1	0.658	1	1	NA	1	-0.114
A3SS	3815	FAM208A	FAM208A	chr3	-	56633043	56633966	56633043	56633843	56638705	56638765	9	0	0	3	0	0	0	2	0	0	98	49	1	1	0.6	NA	NA	NA	1	-0.4
A3SS	3829	CEP97	CEP97	chr3	+	101757633	101758423	101757810	101758423	101757062	101757196	1	1	0	1	0	0	0	1	0	0	98	49	1	1	1	0.333	NA	NA	1	-0.334
A3SS	3840	NAA50	NAA50	chr3	-	113723958	113724095	113723958	113724092	113745941	113746299	14	12	1	4	3	3	0	2	0	3	52	49	1	1	0.767	0.79	0.239	NA	0.386	0.213
A3SS	3845	SYNE2	SYNE2	chr14	+	64221533	64221704	64221575	64221704	64220436	64220637	2	1	0	4	0	0	0	2	0	0	91	49	1	1	0.212	1	NA	NA	1	-0.394
A3SS	3853	DGKZ	DGKZ	chr11	+	46379453	46379568	46379468	46379568	46379202	46379236	0	1	0	1	0	0	0	0	0	2	64	49	1	1	0	1	NA	NA	0	0.5
A3SS	3854	APRT	APRT	chr16	-	88809468	88809840	88809468	88809706	88810069	88810148	10	1	0	1	0	0	0	3	0	0	98	49	1	1	0.833	1	NA	NA	1	-0.084
A3SS	3857	RYK	RYK	chr3	-	134191848	134191983	134191848	134191974	134195081	134195182	2	2	0	2	0	3	0	1	0	0	58	49	1	1	0.458	1	0	NA	1	-0.514
A3SS	3864	CLINT1	CLINT1	chr5	-	157789362	157789567	157789362	157789513	157791702	157791995	1	0	0	3	0	0	0	2	0	3	98	49	1	1	0.143	NA	NA	NA	0.25	-0.107
A3SS	3865	ARMC8	ARMC8	chr3	+	138188400	138188530	138188454	138188530	138187247	138187599	1	0	0	0	0	0	0	1	0	2	98	49	1	1	1	NA	NA	NA	0.2	0.8
A3SS	3872	MFSD1	MFSD1	chr3	+	158804318	158804371	158804322	158804371	158801925	158802315	15	1	0	1	1	0	0	5	0	0	53	49	1	1	0.933	0.48	NA	NA	1	-0.293
A3SS	3877	ZNF124	ZNF124	chr1	-	247154988	247157403	247154988	247157217	247159005	247159066	3	3	0	2	0	0	0	0	0	2	98	49	1	1	0.429	1	NA	NA	0	0.715
A3SS	3880	TOR1AIP1	TOR1AIP1	chr1	+	179889309	179889369	179889312	179889369	179884691	179884769	1	1	0	5	1	0	0	0	0	3	52	49	1	1	0.159	0.485	NA	NA	0	0.322
A3SS	3891	EIF4G1	EIF4G1	chr3	+	184316071	184316218	184316131	184316218	184315762	184315856	6	0	0	1	0	0	0	2	0	0	98	49	1	1	0.75	NA	NA	NA	1	-0.25
A3SS	3892	EIF4G1	EIF4G1	chr3	+	184323404	184323593	184323407	184323593	184323082	184323241	5	1	0	13	0	0	0	2	0	3	52	49	1	1	0.266	1	NA	NA	0.386	0.247
A3SS	3906	KDM5C	KDM5C	chrX	-	53217142	53217277	53217142	53217274	53217795	53217966	13	6	3	4	0	0	0	5	0	0	52	49	1	1	0.754	1	1	NA	1	-0.082
A3SS	3914	MFSD7	MFSD7	chr4	-	684482	684602	684482	684599	684718	684856	5	0	0	1	3	0	2	0	0	2	52	49	1	1	0.825	0	NA	1	0	-0.088
A3SS	3918	TMEM175	TMEM175	chr4	+	951645	951717	951681	951717	951206	951258	2	2	0	10	0	3	0	1	0	1	85	49	1	1	0.103	1	0	NA	0.366	0.002
A3SS	3929	FBXL5	FBXL5	chr4	-	15636493	15636676	15636493	15636673	15638507	15638694	170	70	42	1	0	0	3	40	0	0	52	49	1	1	0.994	1	1	1	1	-0.002
A3SS	3951	CXCL1	CXCL1	chr4	+	73869645	73869792	73869668	73869792	73869391	73869570	12	12	4	12	14	1	0	0	0	3	72	49	1	1	0.405	0.368	0.731	NA	0	0.501
A3SS	3953	OGT	OGT	chrX	+	71536177	71536358	71536207	71536358	71533061	71533336	36	12	3	2	0	0	3	4	0	0	79	49	1	1	0.918	1	1	1	1	-0.027
A3SS	3962	NFKB1	NFKB1	chr4	+	102533844	102533885	102533847	102533885	102529835	102529914	2	0	0	2	2	2	0	0	0	1	52	49	1	1	0.485	0	0	NA	0	0.162
A3SS	3975	ELF2	ELF2	chr4	-	139071865	139072039	139071865	139072003	139073453	139073567	16	8	6	34	11	7	0	4	0	0	85	49	1	1	0.213	0.295	0.331	NA	1	-0.72
A3SS	3980	KIAA0922	KIAA0922	chr4	+	153584834	153584931	153584837	153584931	153583563	153583672	15	0	2	6	1	0	0	2	0	3	52	49	1	1	0.702	0	1	NA	0.386	0.181

A3SS	3989	RWDD4	RWDD4	chr4	-	183651217	183651327	183651217	183651323	183655880	183655961	0	0	0	0	2	0	0	1	0	0	53	49	1	1	NA	0	NA	NA	1	-1
A3SS	3994	CMTM3	CMTM3	chr16	+	66609758	66610003	66609882	66610003	66609434	66609530	0	0	0	6	0	2	0	1	0	1	98	49	1	1	0	NA	0	NA	0.333	-0.333
A3SS	4007	ATL2	ATL2	chr2	-	38293956	38296590	38293956	38296113	38298143	38298575	2	0	0	8	1	3	0	1	0	0	98	49	1	1	0.111	0	0	NA	1	-0.963
A3SS	4025	RICTOR	RICTOR	chr5	-	38945490	38945724	38945490	38945610	38947263	38947441	90	27	9	0	2	0	1	14	0	0	98	49	1	1	1	0.871	1	1	1	-0.043
A3SS	4038	SON	SON	chr21	+	33576364	33577514	33576417	33577514	33575777	33575893	12	4	1	7	1	1	0	5	0	0	98	49	1	1	0.462	0.667	0.333	NA	1	-0.513
A3SS	4039	GAPT	GAPT	chr5	+	58493618	58493838	58493710	58493838	58491434	58491541	3	0	0	2	0	0	0	1	0	0	98	49	1	1	0.429	NA	NA	NA	1	-0.571
A3SS	4040	GAPT	GAPT	chr5	+	58494147	58496358	58494246	58496358	58493710	58493838	8	5	2	7	1	0	0	1	0	2	98	49	1	1	0.364	0.714	1	NA	0.2	0.493
A3SS	4047	ZFAND5	ZFAND5	chr9	-	72360627	72360787	72360627	72360784	72363469	72363606	13	4	1	1	0	0	0	3	0	0	52	49	1	1	0.925	1	1	NA	1	-0.025
A3SS	4049	AGTPBP1	AGTPBP1	chr9	-	85655142	85655320	85655142	85655200	85657434	85657643	47	28	17	25	13	0	0	20	0	4	98	49	1	1	0.485	0.519	1	NA	0.714	-0.046
A3SS	4054	GLRX	GLRX	chr5	-	95813848	95814807	95813848	95814389	95816506	95816626	1	0	0	10	3	1	0	0	0	3	98	49	1	1	0.048	0	0	NA	0	0.016
A3SS	4055	GLRX	GLRX	chr5	-	95813848	95814957	95813848	95814389	95816506	95816626	3	0	0	10	3	1	0	0	0	3	98	49	1	1	0.13	0	0	NA	0	0.043
A3SS	4058	CAST	CAST	chr5	+	96765220	96765325	96765223	96765325	96762273	96762372	19	3	1	2	0	0	0	8	0	3	52	49	1	1	0.9	1	1	NA	0.715	0.252
A3SS	4059	CUX1	CUX1	chr7	+	102178468	102178657	102178474	102178657	102170445	102170550	9	7	2	2	0	0	0	5	0	0	55	49	1	1	0.8	1	1	NA	1	-0.067
A3SS	4078	CNBP	CNBP	chr3	-	129171078	129171283	129171078	129171280	129171445	129171538	0	0	2	12	7	6	0	0	0	2	52	49	1	1	0	0	0.239	NA	0	0.08
A3SS	4080	H2AFY	H2AFY	chr5	-	135360496	135360607	135360496	135360604	135369405	135369603	18	4	7	14	5	10	0	3	0	5	52	49	1	1	0.548	0.43	0.397	NA	0.361	0.097
A3SS	4091	SRA1	SRA1	chr5	-	140557146	140557332	140557146	140557272	140557427	140558093	9	2	0	0	0	0	0	1	0	1	98	49	1	1	1	1	NA	NA	0.333	0.667
A3SS	4093	HARS2	HARS2	chr5	+	140693490	140693665	140693590	140693665	140691425	140691756	4	5	0	9	0	0	0	2	0	0	98	49	1	1	0.182	1	NA	NA	1	-0.409
A3SS	4096	SCARF1	SCARF1	chr17	-	1636708	1636855	1636708	1636818	1636940	1637062	13	19	3	2	0	0	0	2	0	0	86	49	1	1	0.787	1	1	NA	1	-0.071
A3SS	4097	SCARF1	SCARF1	chr17	-	1639911	1640040	1639911	1639970	1640447	1640666	4	10	0	0	1	0	0	2	0	0	98	49	1	1	1	0.833	NA	NA	1	-0.084
A3SS	4104	RNF111	RNF111	chr15	+	59092512	59092640	59092536	59092640	59091058	59091154	3	0	0	7	0	2	0	2	0	0	73	49	1	1	0.223	NA	0	NA	1	-0.888
A3SS	4118	PHYKPL	PHYKPL	chr5	-	178222354	178222580	178222354	178222492	178222851	178222934	9	0	0	3	0	0	0	5	0	0	98	49	1	1	0.6	NA	NA	NA	1	-0.4
A5SS	5	DNASE1L1	DNASE1L1	chrX	-	154408557	154409278	154409111	154409278	154405433	154405655	0	0	0	3	0	0	0	1	0	0	98	49	1	1	0	NA	NA	NA	1	-1
A5SS	24	PSMD10	PSMD10	chrX	-	108087685	108087852	108087764	108087852	108084204	108085127	4	0	0	0	0	0	0	0	0	2	98	49	1	1	1	NA	NA	NA	0	1
A5SS	40	TAF1	TAF1	chrX	+	71423116	71423264	71423116	71423239	71423973	71424066	0	1	0	0	0	0	0	0	0	2	74	49	1	1	NA	1	NA	NA	0	1
A5SS	64	OTUD5	OTUD5	chrX	-	48934695	48934867	48934710	48934867	48934463	48934612	1	0	0	11	7	8	0	3	0	2	64	49	1	1	0.065	0	0	NA	0.535	-0.513
A5SS	66	USP9X	USP9X	chrX	+	41229566	41229827	41229566	41229779	41230500	41230596	13	1	1	17	2	0	0	0	0	1	97	49	1	1	0.279	0.202	1	NA	0	0.494
A5SS	73	TRAPPC2	TRAPPC2	chrX	-	13734043	13734185	13734049	13734185	13719870	13719982	2	3	1	0	2	0	0	2	0	0	55	49	1	1	1	0.572	1	NA	1	-0.143
A5SS	95	ODF2	ODF2	chr9	+	128460941	128461067	128460941	128461010	128469182	128469353	3	0	0	1	0	0	0	0	1	0	98	49	1	1	0.6	NA	NA	0	NA	0.6
A5SS	97	SLC25A25	SLC25A25	chr9	+	128098481	128098768	128098481	128098725	128101095	128101222	0	0	0	2	0	0	0	1	0	0	92	49	1	1	0	NA	NA	NA	1	-1
A5SS	115	CTSL	CTSL	chr9	+	87726058	87726308	87726058	87726253	87727593	87727729	1	0	0	0	0	0	0	0	0	2	98	49	1	1	1	NA	NA	NA	0	1
A5SS	133	ZCCHC6	ZCCHC6	chr9	-	86354270	86354487	86354380	86354487	86352679	86353230	90	62	15	0	2	0	3	11	0	0	98	49	1	1	1	0.939	1	1	1	-0.02
A5SS	151	GRINA	GRINA	chr8	+	143990057	143990311	143990057	143990199	143991199	143991602	1	1	7	3	0	0	1	0	0	0	98	49	1	1	0.143	1	1	1	NA	-0.286
A5SS	156	FAM49B	FAM49B	chr8	-	129896671	129896854	129896748	129896854	129879388	129879471	23	11	0	0	1	2	0	9	0	0	98	49	1	1	1	0.846	0	NA	1	-0.385
A5SS	173	CHCHD7	CHCHD7	chr8	+	56212761	56212909	56212761	56212889	56214597	56214667	2	0	0	0	0	0	0	0	0	1	69	49	1	1	1	NA	NA	NA	0	1
A5SS	191	NUB1	NUB1	chr7	+	151374096	151374243	151374096	151374201	151375847	151375943	3	4	3	5	1	0	0	3	0	1	91	49	1	1	0.244	0.683	1	NA	0.618	0.024
A5SS	192	FASTK	FASTK	chr7	-	151078841	151079021	151078922	151079021	151078561	151078701	3	0	1	1	0	0	0	1	0	0	98	49	1	1	0.6	NA	1	NA	1	-0.2
A5SS	204	TRIM24	TRIM24	chr7	+	138554697	138554966	138554697	138554864	138567480	138567654	9	1	1	1	0	0	0	2	0	0	98	49	1	1	0.818	1	1	NA	1	-0.061
A5SS	207	C7orf49	C7orf49	chr7	-	135168748	135169060	135168785	135169060	135167731	135167807	18	11	3	0	1	0	0	0	0	1	86	49	1	1	1	0.862	1	NA	0	0.954
A5SS	236	AKAP9	AKAP9	chr7	+	92070029	92070206	92070029	92070182	92070904	92071009	4	0	0	2	0	0	2	0	0	0	73	49	1	1	0.573	NA	NA	1	NA	-0.427
A5SS	247	IKZF1	IKZF1	chr7	+	50391728	50391863	50391728	50391833	50399917	50405100	3	3	0	2	0	2	0	2	0	3	79	49	1	1	0.482	1	0	NA	0.293	0.201
A5SS	262	WIPI2	WIPI2	chr7	+	5193117	5193345	5193117	5193171	5199575	5199658	7	3	0	0	1	0	0	0	0	2	98	49	1	1	1	0.6	NA	NA	0	0.8
A5SS	263	WIPI2	WIPI2	chr7	+	5229607	5229738	5229607	5229705	5230834	5233855	12	1	2	3	2	3	0	1	0	0	82	49	1	1	0.705	0.23	0.285	NA	1	-0.593
A5SS	272	PCMT1	PCMT1	chr6	+	149802199	149802416	149802199	149802369	149810615	149811421	4	5	10	1	0	0	0	1	0	0	96	49	1	1	0.671	1	1	NA	1	-0.11
A5SS	282	TNFAIP3	TNFAIP3	chr6	+	137867187	137867518	137867187	137867494	137871212	137871522	10	13	0	2	2	0	3	1	2	0	73	49	1	1	0.77	0.814	NA	0.502	1	0.041
A5SS	283	TNFAIP3	TNFAIP3	chr6	+	137867187	137867542	137867187	137867494	137871212	137871522	10	18	0	2	2	0	3	6	2	0	97	49	1	1	0.716	0.82	NA	0.431	1	0.052
A5SS	286	VNN2	VNN2	chr6	-	132752460	132752749	132752672	132752749	132751144	132751518	402	191	203	1	1	0	5	36	0	0	98	49	1	1	0.995	0.99	1	1	1	-0.005
A5SS	303	SYTL3	SYTL3	chr6	+	158662749	158663378	158662749	158662812	158665394	158665613	13	7	1	1	1	0	1	2	0	1	98	49	1	1	0.867	0.778	1	1	0.5	0.132
A5SS	304	HMGN3	HMGN3	chr6	-	79202275	79202389	79202316	79202389	79201244	79201726	5	0	0	1	0	0	0	1	0	0	90	49	1	1	0.731	NA	NA	NA	1	-0.269
A5SS	305	COX7A2	COX7A2	chr6	-	75241122	75241265	75241175	75241265	75240300	75240385	4	1	1	6	1	7	0	2	0	0	98	49	1	1	0.25	0.333	0.067	NA	1	-0.783
A5SS	322	TJAP1	TJAP1	chr6	+	43498977	43499136	43498977	43499100	43500743	43500772	2	0	0	2	0	0	0	0	1	0	85	49	1	1	0.366	NA	NA	0	NA	0.366
A5SS	324	CCND3	CCND3	chr6	-	41940951	41941848	41941451	41941848	41940369	41940585	2	3	0	4	0	0	0	0	0	5	98	49	1	1	0.2	1	NA	NA	0	0.6
A5SS	330	HMGA1	HMGA1	chr6	+	34240736	34240915	34240736	34240882	34242711	34242795	6	4	0	5	0	0	1	4	0	3	82	49	1	1	0.418	1	NA	1	0.443	-0.013
A5SS	352	VPS52	VPS52	chr6	-	33271136	33271965	33271585	33271965	33270198	33270283	2	0	2	3	1	0	0	0	0	1	98	49	1	1	0.25	0	1	NA	0	0.417
A5SS	423	DDAH2	DDAH2	chr6	-	31728864	31730265	31730050	31730265	31728645	31728745	13	5	0	0	0	1	0	5	0	0	98	49	1	1	1	1	0	NA	1	-0.333
A5SS	430	APOM	APOM	chr6	+	31656471	31656633	31656471	31656626	31657224	31657298	0	1	0	0	0	0	0	0	0	1	56	49	1	1	NA	1	NA	NA	0	1
A5SS	437	BAG6	BAG6	chr6	-	31652399	31652700	31652423	31652700	31651655	31651776	8	3	0	3	0	0	0	0	0	1	73	49	1	1	0.642	1	NA	NA	0	0.821
A5SS	459	NRM	NRM	chr6	-	30690024	30690243	30690046	30690243	30689275	30689452	2	2	0	1	0	0	1	0	0	0	71	49	1	1	0.58	1	NA	1	NA	-0.21
A5SS	491	ARRB2	ARRB2	chr17	+	4716411	4716671	4716411	4716608	4717216	4717276	2	1	0	6	4	1	0	0	0	1	98	49	1	1	0.143	0.111	0	NA	0	0.085
A5SS	499	CRK	CRK	chr17	-	1436619	1437155	1436789	1437155	1421352	1423650	4	5	4	3	4	1	0	6	0	1	98	49	1	1	0.4	0.385	0.667	NA	0.75	-0.266
A5SS	503	CHMP1A	CHMP1A	chr16	-	89647034	89647331	89647202	89647331	89646526	89646714	4	0	2	4	4	3	0	1	0	0	98	49	1	1	0.333	0	0.25	NA	1	-0.806
A5SS	504	C12orf57	C12orf57	chr12	+	6944379	6944652	6944379	6944565	6945770	6946003	12	0	1	2	0	0	2	1	0	1	98	49	1	1	0.75	NA	1	1	0.333	0.209
A5SS	508	TSPAN17	TSPAN17	chr5	+	176656894	176656956	176656894	176656924	176657517	176659058	2	0	0	2	0	0	0	1	0	0	81	49	1	1	0.377	NA	NA	NA	1	-0.623
A5SS	513	ATRAID	ATRAID	chr2	+	27212350	27212607	27212350	27212467	27213176	27213298	1	0	0	1	1	0	1	3	0	0	98	49	1	1	0.333	0	NA	1	1	-0.834
A5SS	518	SH2B1	SH2B1	chr16	+	28872533	28872758	28872533	28872705	28873169	28873269	0	1	0	2	0	0	0	0	0	1	98	49	1	1	0	1	NA	NA	0	0.5
A5SS	521	PRKD2	PRKD2	chr19	-	46716814	46717127	46716950	46717127	46716130	46716535	3	1	0	0	0	0	0	2	0	1	98	49	1	1	1	1	NA	NA	0.5	0.5
A5SS	525	SLC35A2	SLC35A2	chrX	-	48911506	48911958	48911545	48911958	48909813	48909996	3	3	0	0	0	0	0	0	0	3	88	49	1	1	1	1	NA	NA	0	1
A5SS	531	NMRAL1	NMRAL1	chr16	-	4474409	4474650	4474422	4474650	4474092	4474166	0	1	0	1	0	0	0	1	0	0	62	49	1	1	0	1	NA	NA	1	-0.5
A5SS	536	TTC31	TTC31	chr2	+	74491480	74491672	74491480	74491666	74492003	74492055	1	0	0	2	2	0	0	1	3	0	55	49	1	1	0.308	0	NA	0	1	-0.346
A5SS	539	UNKL	UNKL	chr16	-	1369933	1370357	1370129	1370357	1367655	1367858	0	0	0	0	1	2	1	0	0	1	98	49	1	1	NA	0	0	1	0	-0.5
A5SS	580	TRAPPC5	TRAPPC5	chr19	+	7680820	7681026	7680820	7680878	7682241	7682862	2	0	0	0	0	0	0	0	0	1	98	49	1	1	1	NA	NA	NA	0	1
A5SS	581	TRAPPC5	TRAPPC5	chr19	+	7680820	7681049	7680820	7680878	7682241	7682862	2	0	0	0	0	0	0	0	0	1	98	49	1	1	1	NA	NA	NA	0	1
A5SS	585	ISG20	ISG20	chr15	+	88638742	88639178	88638742	88639076	88639342	88639594	0	2	0	12	6	1	0	1	0	1	98	49	1	1	0	0.143	0	NA	0.333	-0.285
A5SS	597	STX16	STX16	chr20	+	58651252	58652138	58651252	58652087	58659622	58659634	13	10	3	0	0	0	4	7	0	1	98	49	1	1	1	1	1	1	0.778	0.111
A5SS	608	PML	PML	chr15	+	74033155	74033422	74033155	74033414	74034477	74034530	5	1	1	3	0	0	0	1	0	2	57	49	1	1	0.589	1	1	NA	0.301	0.562
A5SS	616	USP8	USP8	chr15	+	50424376	50424654	50424376	50424514	50439008	50439177	6	3	0	3	0	0	0	1	0	0	98	49	1	1	0.5	1	NA	NA	1	-0.25
A5SS	621	PIGG	PIGG	chr4	+	499199	499725	499199	499489	500395	500601	5	0	0	7	0	0	0	1	0	3	98	49	1	1	0.263	NA	NA	NA	0.143	0.12
A5SS	622	PIGG	PIGG	chr4	+	499199	500047	499199	499489	500395	500601	4	0	0	7	0	0	0	1	0	3	98	49	1	1	0.222	NA	NA	NA	0.143	0.079
A5SS	647	ANAPC11	ANAPC11	chr17	+	81894466	81894624	81894466	81894586	81899919	81900533	0	2	0	10	1	3	0	0	1	0	87	49	1	1	0	0.53	0	0	NA	0.177
A5SS	672	ZNF302	ZNF302	chr19	+	34677638	34677856	34677638	34677833	34678736	34678813	0	0	0	1	0	0	0	1	0	0	72	49	1	1	0	NA	NA	NA	1	-1
A5SS	673	ZNF302	ZNF302	chr19	+	34677638	34677862	34677638	34677833	34678736	34678813	0	0	0	1	0	0	0	1	0	0	78	49	1	1	0	NA	NA	NA	1	-1
A5SS	674	ZNF302	ZNF302	chr19	+	34677638	34678103	34677638	34677833	34678736	34678813	2	0	0	1	0	0	0	1	0	0	98	49	1	1	0.5	NA	NA	NA	1	-0.5
A5SS	689	NIPA2	NIPA2	chr15	+	22838640	22838947	22838640	22838921	22845145	22845267	0	0	1	0	1	0	2	1	0	0	75	49	1	1	NA	0	1	1	1	-0.5
A5SS	725	ARL5A	ARL5A	chr2	-	151828130	151828495	151828388	151828495	151815138	151815199	1	0	0	1	1	0	0	1	0	0	98	49	1	1	0.333	0	NA	NA	1	-0.834
A5SS	731	UQCR10	UQCR10	chr22	+	29767368	29767607	29767368	29767548	29769677	29770413	1	0	0	0	1	4	0	1	0	3	98	49	1	1	1	0	0	NA	0.143	0.19
A5SS	738	TRRAP	TRRAP	chr7	+	98983263	98983492	98983263	98983459	98984092	98984358	1	0	0	1	5	0	0	3	0	1	82	49	1	1	0.374	0	NA	NA	0.642	-0.455
A5SS	741	PSME1	PSME1	chr14	+	24138473	24138568	24138473	24138560	24138735	24138967	3	5	2	37	24	15	0	1	0	11	57	49	1	1	0.065	0.152	0.103	NA	0.072	0.035
A5SS	746	ARHGEF40	ARHGEF40	chr14	+	21073931	21075180	21073931	21074991	21075331	21075499	23	4	0	1	0	0	0	3	0	0	98	49	1	1	0.92	1	NA	NA	1	-0.04
A5SS	748	FANCA	FANCA	chr16	-	89739128	89739289	89739132	89739289	89738881	89738974	5	0	1	1	0	0	0	0	0	1	53	49	1	1	0.822	NA	1	NA	0	0.911
A5SS	763	PLEKHB2	PLEKHB2	chr2	+	131132901	131133015	131132901	131132991	131140169	131140275	2	0	0	2	0	0	0	2	0	0	73	49	1	1	0.402	NA	NA	NA	1	-0.598
A5SS	764	AFF2	AFF2	chrX	+	148651998	148652131	148651998	148652119	148661907	148662768	1	0	4	6	7	3	0	0	0	1	61	49	1	1	0.118	0	0.517	NA	0	0.212
A5SS	774	PICK1	PICK1	chr22	+	38057254	38057587	38057254	38057489	38057752	38057850	0	0	0	1	0	0	1	0	0	0	98	49	1	1	0	NA	NA	1	NA	-1
A5SS	793	AGTRAP	AGTRAP	chr1	+	11748414	11748610	11748414	11748589	11750076	11750771	12	8	7	4	0	3	0	4	0	1	70	49	1	1	0.677	1	0.62	NA	0.737	0.029
A5SS	801	PSEN1	PSEN1	chr14	+	73147966	73148106	73147966	73148094	73170796	73171047	10	6	2	25	3	0	0	2	0	2	61	49	1	1	0.243	0.616	1	NA	0.445	0.175
A5SS	827	ARL6IP4	ARL6IP4	chr12	+	122981128	122981299	122981128	122981266	122981594	122981879	11	2	3	5	0	0	0	3	0	1	82	49	1	1	0.568	1	1	NA	0.642	0.214
A5SS	835	PPP4R3A	PPP4R3A	chr14	-	91475810	91475966	91475849	91475966	91473238	91473370	15	12	4	1	0	0	0	3	0	2	88	49	1	1	0.893	1	1	NA	0.455	0.509
A5SS	838	TRAPPC3	TRAPPC3	chr1	-	36149091	36149514	36149336	36149514	36140068	36140166	1	2	0	3	1	0	0	0	4	0	98	49	1	1	0.143	0.5	NA	0	NA	0.322
A5SS	844	NLRP1	NLRP1	chr17	-	5533291	5533384	5533303	5533384	5532821	5532984	6	4	1	30	9	7	0	1	0	7	61	49	1	1	0.138	0.263	0.103	NA	0.103	0.065
A5SS	846	MAPKAPK5	MAPKAPK5	chr12	+	111888884	111889006	111888884	111889000	111890039	111890144	0	1	1	2	0	0	0	2	0	2	55	49	1	1	0	1	1	NA	0.471	0.196
A5SS	847	GIT2	GIT2	chr12	-	109951166	109951316	109951211	109951316	109948061	109948846	39	29	1	2	0	0	0	5	0	0	94	49	1	1	0.91	1	1	NA	1	-0.03
A5SS	876	ITFG1	ITFG1	chr16	-	47460837	47461274	47461114	47461274	47459102	47459175	3	4	0	0	2	0	0	2	0	0	98	49	1	1	1	0.5	NA	NA	1	-0.25
A5SS	877	IL32	IL32	chr16	+	3067376	3067613	3067376	3067415	3067983	3068010	16	3	0	3	1	0	0	1	0	0	98	49	1	1	0.727	0.6	NA	NA	1	-0.337
A5SS	880	KLF7	KLF7	chr2	-	207123773	207124404	207124343	207124404	207088457	207088581	26	13	1	2	0	0	4	5	0	0	98	49	1	1	0.867	1	1	1	1	-0.044
A5SS	881	KLF7	KLF7	chr2	-	207123975	207124404	207124343	207124404	207088457	207088581	24	12	0	2	0	0	4	4	0	0	98	49	1	1	0.857	1	NA	1	1	-0.072
A5SS	889	STARD3	STARD3	chr17	+	39657774	39657852	39657774	39657839	39657972	39658026	4	11	1	3	0	0	0	4	0	0	62	49	1	1	0.513	1	1	NA	1	-0.162
A5SS	892	PTGES3	PTGES3	chr12	-	56665631	56666266	56666203	56666266	56664775	56664800	0	2	0	6	13	1	0	0	0	5	98	49	1	1	0	0.071	0	NA	0	0.024
A5SS	952	NDUFB11	NDUFB11	chrX	-	47142583	47142744	47142613	47142744	47142215	47142440	1	0	0	7	1	0	0	0	0	3	79	49	1	1	0.081	0	NA	NA	0	0.041
A5SS	954	CLEC2D	CLEC2D	chr12	+	9687901	9688161	9687901	9688086	9692827	9692931	11	0	0	18	3	0	0	1	0	0	98	49	1	1	0.234	0	NA	NA	1	-0.883
A5SS	976	KIAA1033	KIAA1033	chr12	+	105133769	105133899	105133769	105133896	105137885	105138011	1	2	0	29	17	4	0	0	0	6	52	49	1	1	0.031	0.1	0	NA	0	0.044
A5SS	982	WNK1	WNK1	chr12	+	890452	890513	890452	890510	894561	894635	7	4	0	4	0	0	0	1	0	0	52	49	1	1	0.623	1	NA	NA	1	-0.189
A5SS	1006	NLRX1	NLRX1	chr11	+	119168712	119169302	119168712	119168894	119171355	119171473	0	0	0	1	0	0	1	0	0	0	98	49	1	1	0	NA	NA	1	NA	-1
A5SS	1012	SLC12A9	SLC12A9	chr7	+	100852718	100852839	100852718	100852835	100854155	100854378	4	2	1	3	2	1	0	0	0	2	53	49	1	1	0.552	0.48	0.48	NA	0	0.504
A5SS	1016	POLG	POLG	chr15	-	89334672	89334795	89334684	89334795	89333095	89333913	3	0	0	1	0	0	0	0	0	3	61	49	1	1	0.707	NA	NA	NA	0	0.707
A5SS	1019	SNX19	SNX19	chr11	-	130914265	130916487	130916279	130916487	130911632	130911771	7	8	0	1	0	0	0	3	0	1	98	49	1	1	0.778	1	NA	NA	0.6	0.289
A5SS	1051	PTPN7	PTPN7	chr1	-	202160544	202161588	202161386	202161588	202159280	202159454	0	0	0	1	0	0	0	1	0	0	98	49	1	1	0	NA	NA	NA	1	-1
A5SS	1067	LOC374443	LOC374443	chr12	+	9648046	9648933	9648046	9648328	9653682	9653808	13	0	0	0	0	3	0	2	0	0	98	49	1	1	1	NA	0	NA	1	-0.5
A5SS	1075	IRF2BP2	IRF2BP2	chr1	-	234608446	234609525	234608494	234609525	234604268	234607852	16	2	0	9	1	0	0	5	1	5	97	49	1	1	0.473	0.503	NA	0	0.336	0.32
A5SS	1114	TMEM55B	TMEM55B	chr14	-	20461162	20461612	20461183	20461612	20460654	20460845	0	2	0	0	1	0	0	2	0	0	70	49	1	1	NA	0.583	NA	NA	1	-0.417
A5SS	1133	WDR26	WDR26	chr1	-	224431476	224431581	224431524	224431581	224419517	224419615	33	17	3	1	1	0	0	10	0	0	97	49	1	1	0.943	0.896	1	NA	1	-0.054
A5SS	1134	WDR26	WDR26	chr1	-	224431476	224431581	224431524	224431581	224424517	224424654	40	25	3	10	11	1	0	11	0	4	97	49	1	1	0.669	0.534	0.602	NA	0.581	0.021
A5SS	1160	PKM	PKM	chr15	-	72230895	72231386	72231115	72231386	72218943	72219110	1	0	0	6	0	0	0	0	0	1	98	49	1	1	0.077	NA	NA	NA	0	0.077
A5SS	1192	CNOT1	CNOT1	chr16	-	58556846	58556993	58556861	58556993	58555783	58555908	9	1	0	3	0	0	0	1	0	1	64	49	1	1	0.697	1	NA	NA	0.434	0.415
A5SS	1203	CANX	CANX	chr5	+	179698928	179699102	179698928	179699049	179705678	179705852	3	0	0	0	0	0	0	0	0	1	98	49	1	1	1	NA	NA	NA	0	1
A5SS	1239	CHST11	CHST11	chr12	+	104456913	104457529	104456913	104457514	104601905	104601991	11	14	8	5	8	0	0	7	0	2	64	49	1	1	0.627	0.573	1	NA	0.728	0.005
A5SS	1248	APAF1	APAF1	chr12	+	98648625	98648815	98648625	98648782	98649486	98649684	58	11	6	1	0	0	0	9	0	0	82	49	1	1	0.972	1	1	NA	1	-0.009
A5SS	1249	TRERF1	TRERF1	chr6	-	42263259	42263568	42263319	42263568	42259338	42259723	1	0	0	0	0	0	0	0	0	1	98	49	1	1	1	NA	NA	NA	0	1
A5SS	1255	NBPF11	NBPF11	chr1	-	148110377	148110541	148110457	148110541	148109283	148109335	0	1	0	2	0	0	1	0	0	0	98	49	1	1	0	1	NA	1	NA	-0.5
A5SS	1274	TUG1	TUG1	chr22	+	30972854	30973601	30972854	30973587	30975169	30979394	9	4	0	4	2	0	1	4	0	0	63	49	1	1	0.636	0.609	NA	1	1	-0.377
A5SS	1279	TRIM5	TRIM5	chr11	-	5684867	5685109	5685015	5685109	5679760	5680238	3	2	0	0	2	0	0	1	0	0	98	49	1	1	1	0.333	NA	NA	1	-0.334
A5SS	1303	DAP3	DAP3	chr1	+	155689093	155689243	155689093	155689174	155709772	155709824	2	2	0	0	0	0	0	0	0	1	98	49	1	1	1	1	NA	NA	0	1
A5SS	1308	AP1G2	AP1G2	chr14	-	23565815	23566160	23566063	23566160	23565605	23565701	1	0	0	0	0	0	0	3	0	1	98	49	1	1	1	NA	NA	NA	0.6	0.4
A5SS	1310	SIRPB2	SIRPB2	chr20	-	1479699	1480065	1479993	1480065	1478265	1478607	21	3	5	5	2	2	0	8	0	2	98	49	1	1	0.677	0.429	0.556	NA	0.667	-0.113
A5SS	1323	THBS3	THBS3	chr1	-	155206192	155206406	155206199	155206406	155205059	155205316	0	0	0	2	0	0	0	1	0	0	56	49	1	1	0	NA	NA	NA	1	-1
A5SS	1330	AP3B1	AP3B1	chr5	-	78294439	78294755	78294451	78294755	78267519	78267595	2	1	0	13	5	3	0	0	0	2	61	49	1	1	0.11	0.138	0	NA	0	0.083
A5SS	1334	PAPD4	PAPD4	chr5	+	79612419	79612769	79612419	79612735	79613010	79613075	7	8	0	0	0	0	2	1	0	1	83	49	1	1	1	1	NA	1	0.371	0.315
A5SS	1343	TRAFD1	TRAFD1	chr12	+	112125544	112126149	112125544	112125618	112130510	112130569	0	0	0	2	0	0	0	1	0	0	98	49	1	1	0	NA	NA	NA	1	-1
A5SS	1349	STX10	STX10	chr19	-	13144763	13144870	13144767	13144870	13144576	13144671	38	11	6	0	0	1	4	7	0	0	53	49	1	1	1	1	0.847	1	1	-0.051
A5SS	1359	MIER1	MIER1	chr1	+	66984571	66984652	66984571	66984592	66986402	66988619	30	2	5	2	2	0	0	2	0	0	98	49	1	1	0.882	0.333	1	NA	1	-0.262
A5SS	1366	TSEN15	TSEN15	chr1	+	184072156	184072310	184072156	184072298	184072826	184074210	1	0	0	0	0	0	0	0	0	1	61	49	1	1	1	NA	NA	NA	0	1
A5SS	1375	RAP1B	RAP1B	chr12	+	68610838	68611052	68610838	68611043	68648698	68648781	1	1	0	8	3	0	0	0	0	1	58	49	1	1	0.096	0.22	NA	NA	0	0.158
A5SS	1382	ARID4A	ARID4A	chr14	+	58366023	58366230	58366023	58366068	58366882	58367029	22	7	2	0	0	1	0	4	0	0	98	49	1	1	1	1	0.5	NA	1	-0.167
A5SS	1384	MDM2	MDM2	chr12	+	68835828	68835984	68835828	68835843	68836671	68836749	57	22	17	3	0	0	0	9	0	0	98	49	1	1	0.905	1	1	NA	1	-0.032
A5SS	1394	MKL1	MKL1	chr22	-	40416975	40417046	40416985	40417046	40410280	40411907	1	0	0	3	5	0	0	0	4	0	59	49	1	1	0.217	0	NA	0	NA	0.108
A5SS	1397	FLYWCH2	FLYWCH2	chr16	+	2883194	2883366	2883194	2883272	2895219	2895320	0	3	0	2	0	0	0	2	0	0	98	49	1	1	0	1	NA	NA	1	-0.5
A5SS	1406	UFM1	UFM1	chr13	+	38349998	38350227	38349998	38350055	38354238	38354296	2	0	0	1	1	0	0	0	0	2	98	49	1	1	0.5	0	NA	NA	0	0.25
A5SS	1418	TMEM170A	TMEM170A	chr16	-	75451668	75451839	75451737	75451839	75443243	75447688	2	1	3	1	1	0	0	1	0	0	98	49	1	1	0.5	0.333	1	NA	1	-0.389
A5SS	1424	UBE2J2	UBE2J2	chr1	-	1267732	1267992	1267861	1267992	1263345	1263386	1	0	0	5	1	0	0	1	0	0	98	49	1	1	0.091	0	NA	NA	1	-0.955
A5SS	1427	RBMS1	RBMS1	chr2	-	160286968	160287084	160286977	160287084	160284994	160285044	15	4	2	13	6	1	0	2	0	2	58	49	1	1	0.494	0.36	0.628	NA	0.458	0.036
A5SS	1445	RBBP5	RBBP5	chr1	-	205094872	205095064	205094986	205095064	205086141	205088815	5	5	0	0	7	0	0	1	0	0	98	49	1	1	1	0.263	NA	NA	1	-0.369
A5SS	1453	TAF12	TAF12	chr1	-	28642654	28643092	28642991	28643092	28621913	28622165	0	0	0	1	0	1	0	1	0	0	98	49	1	1	0	NA	0	NA	1	-1
A5SS	1469	KHDRBS1	KHDRBS1	chr1	+	32037834	32038119	32037834	32038036	32038551	32038619	2	1	0	4	2	1	0	0	0	2	98	49	1	1	0.2	0.2	0	NA	0	0.133
A5SS	1476	IFI6	IFI6	chr1	-	27669220	27669346	27669232	27669346	27668457	27668535	1	0	0	0	0	0	0	4	0	1	61	49	1	1	1	NA	NA	NA	0.763	0.237
A5SS	1489	NPEPL1	NPEPL1	chr20	+	58691723	58691830	58691723	58691780	58693736	58693922	13	4	0	1	0	0	0	5	0	1	98	49	1	1	0.867	1	NA	NA	0.714	0.22
A5SS	1490	DEDD2	DEDD2	chr19	-	42201988	42202072	42202050	42202072	42198592	42199829	10	3	0	3	1	0	4	0	0	1	98	49	1	1	0.625	0.6	NA	1	0	0.113
A5SS	1495	ADD1	ADD1	chr4	+	2904763	2905108	2904763	2905015	2907742	2907844	10	0	0	2	2	0	1	2	0	0	98	49	1	1	0.714	0	NA	1	1	-0.643
A5SS	1500	CAP1	CAP1	chr1	+	40040582	40040801	40040582	40040783	40059336	40059458	5	2	2	1	0	0	0	1	0	0	67	49	1	1	0.785	1	1	NA	1	-0.072

A5SS	1503	ACAA1	ACAA1	chr3	-	38126161	38126700	38126509	38126700	38125825	38125881	7	5	5	2	0	0	0	3	0	0	98	49	1	1	0.636	1	1	NA	1	-0.121
A5SS	1520	BTF3	BTF3	chr5	+	73498424	73498799	73498424	73498558	73499133	73499202	0	1	0	12	0	0	0	2	0	2	98	49	1	1	0	1	NA	NA	0.333	0.167
A5SS	1534	TTLL3	TTLL3	chr3	+	9826996	9827240	9826996	9827072	9828959	9829395	12	12	4	1	0	0	2	5	0	0	98	49	1	1	0.857	1	1	1	1	-0.048
A5SS	1536	CASP8	CASP8	chr2	+	201276826	201277104	201276826	201276968	201284815	201285317	190	74	9	20	6	10	0	9	0	3	98	49	1	1	0.826	0.86	0.31	NA	0.6	0.065
A5SS	1537	EIF4B	EIF4B	chr12	+	53034611	53034724	53034611	53034709	53037408	53037622	8	2	0	51	13	14	0	0	0	3	64	49	1	1	0.107	0.105	0	NA	0	0.071
A5SS	1546	NR3C1	NR3C1	chr5	-	143313998	143314168	143314061	143314168	143310096	143310213	8	1	0	16	5	3	0	0	0	4	52	49	1	1	0.32	0.159	0	NA	0	0.16
A5SS	1553	NAT9	NAT9	chr17	-	74775598	74775707	74775621	74775707	74773575	74773685	1	0	0	2	0	0	0	1	0	0	72	49	1	1	0.254	NA	NA	NA	1	-0.746
A5SS	1576	C12orf43	C12orf43	chr12	-	121011010	121011146	121011103	121011146	121010827	121010926	0	0	0	0	4	0	0	1	0	0	98	49	1	1	NA	0	NA	NA	1	-1
A5SS	1597	RPA2	RPA2	chr1	-	27913911	27914169	27914062	27914169	27907180	27907282	7	5	0	4	0	0	0	0	0	2	98	49	1	1	0.467	1	NA	NA	0	0.734
A5SS	1614	CPEB2	CPEB2	chr4	+	15007304	15007595	15007304	15007586	15017187	15017278	2	3	0	3	1	0	0	1	0	2	58	49	1	1	0.36	0.717	NA	NA	0.297	0.241
A5SS	1619	PLCB2	PLCB2	chr15	-	40296745	40296908	40296757	40296908	40296521	40296634	16	7	1	3	6	0	0	7	0	3	61	49	1	1	0.811	0.484	1	NA	0.652	0.113
A5SS	1621	RBM10	RBM10	chrX	+	47179879	47180040	47179879	47180037	47180211	47180309	0	0	0	3	3	0	0	3	0	0	52	49	1	1	0	0	NA	NA	1	-1
A5SS	1636	DENND5A	DENND5A	chr11	-	9141939	9142108	9141973	9142108	9138825	9139854	41	25	22	0	1	1	1	6	0	0	83	49	1	1	1	0.937	0.929	1	1	-0.045
A5SS	1643	CDC42EP3	CDC42EP3	chr2	-	37671425	37672199	37672084	37672199	37641881	37646822	22	3	4	1	0	0	4	6	0	0	98	49	1	1	0.917	1	1	1	1	-0.028
A5SS	1644	CDC42EP3	CDC42EP3	chr2	-	37672060	37672199	37672084	37672199	37641881	37646822	3	0	3	1	0	0	0	1	0	0	73	49	1	1	0.668	NA	1	NA	1	-0.166
A5SS	1659	CELF2	CELF2	chr10	+	11314138	11314270	11314138	11314258	11321206	11321386	9	11	3	6	13	8	0	1	0	3	61	49	1	1	0.546	0.405	0.231	NA	0.211	0.183
A5SS	1677	BPTF	BPTF	chr17	+	67945408	67946325	67945408	67945896	67947725	67947808	2	2	0	7	1	0	0	0	1	1	98	49	1	1	0.125	0.5	NA	0	0	0.312
A5SS	1680	CTNND1	CTNND1	chr11	+	57761761	57762119	57761761	57762046	57794009	57794081	0	2	0	0	0	0	0	1	0	1	98	49	1	1	NA	1	NA	NA	0.333	0.667
A5SS	1697	UBAP2L	UBAP2L	chr1	+	154234590	154234792	154234590	154234759	154235195	154235291	4	0	1	4	0	1	0	1	0	0	82	49	1	1	0.374	NA	0.374	NA	1	-0.626
A5SS	1705	RCHY1	RCHY1	chr4	-	75491582	75491637	75491610	75491637	75490580	75490701	0	1	0	4	2	0	0	0	1	0	77	49	1	1	0	0.241	NA	0	NA	0.12
A5SS	1706	RCHY1	RCHY1	chr4	-	75508819	75508935	75508823	75508935	75494100	75494179	3	0	0	0	0	0	0	0	0	2	53	49	1	1	1	NA	NA	NA	0	1
A5SS	1711	AMICA1	AMICA1	chr11	-	118197967	118198091	118197997	118198091	118196734	118196821	130	46	31	45	39	38	0	14	0	6	79	49	1	1	0.642	0.422	0.336	NA	0.591	-0.124
A5SS	1720	ZNF33A	ZNF33A	chr10	+	38016870	38017020	38016870	38017015	38017290	38017386	3	2	0	13	6	4	0	0	0	3	54	49	1	1	0.173	0.232	0	NA	0	0.135
A5SS	1729	GMPR2	GMPR2	chr14	+	24232421	24232847	24232421	24232788	24233218	24233340	21	11	1	9	4	3	0	2	0	4	98	49	1	1	0.538	0.579	0.143	NA	0.2	0.22
A5SS	1730	GMPR2	GMPR2	chr14	+	24233478	24233598	24233478	24233593	24235736	24235820	9	8	7	2	0	0	0	1	0	0	54	49	1	1	0.803	1	1	NA	1	-0.066
A5SS	1732	UBE3B	UBE3B	chr12	+	109477622	109478109	109477622	109477764	109481636	109481742	5	0	0	1	1	0	0	1	0	0	98	49	1	1	0.714	0	NA	NA	1	-0.643
A5SS	1770	CPEB3	CPEB3	chr10	-	92192476	92192636	92192545	92192636	92180962	92181019	2	0	0	0	0	0	0	0	0	1	98	49	1	1	1	NA	NA	NA	0	1
A5SS	1771	ARFIP1	ARFIP1	chr4	+	152779936	152780226	152779936	152780091	152790758	152790875	3	3	0	2	0	0	0	3	0	0	98	49	1	1	0.429	1	NA	NA	1	-0.285
A5SS	1772	ARFIP1	ARFIP1	chr4	+	152779936	152780226	152779936	152780091	152829624	152829726	11	3	0	6	1	0	0	1	0	1	98	49	1	1	0.478	0.6	NA	NA	0.333	0.206
A5SS	1778	ZNF518A	ZNF518A	chr10	+	96155325	96155398	96155325	96155376	96155923	96156047	0	1	0	3	0	0	0	0	0	3	71	49	1	1	0	1	NA	NA	0	0.5
A5SS	1783	PIGBOS1	PIGBOS1	chr15	-	55318863	55319194	55318930	55319194	55317183	55317867	14	1	0	0	4	2	1	0	0	0	98	49	1	1	1	0.111	0	1	NA	-0.63
A5SS	1784	PIGBOS1	PIGBOS1	chr15	-	55318906	55319194	55318930	55319194	55317183	55317867	13	0	0	0	4	2	1	0	0	0	73	49	1	1	1	0	0	1	NA	-0.667
A5SS	1788	PDCD6	PDCD6	chr5	+	306601	306760	306601	306754	311292	311402	11	2	4	1	0	0	0	1	0	1	55	49	1	1	0.907	1	1	NA	0.471	0.498
A5SS	1800	TAZ	TAZ	chrX	+	154411517	154412006	154411517	154411952	154412085	154412214	0	1	0	1	1	0	0	0	2	1	98	49	1	1	0	0.333	NA	0	0	0.167
A5SS	1801	TAZ	TAZ	chrX	+	154413206	154413358	154413206	154413252	154413481	154413567	2	5	0	4	1	0	0	4	2	1	98	49	1	1	0.2	0.714	NA	0	0.667	0.123
A5SS	1802	GBF1	GBF1	chr10	+	102376934	102377140	102376934	102377128	102379283	102379434	1	0	0	2	0	0	0	2	0	2	61	49	1	1	0.287	NA	NA	NA	0.445	-0.158
A5SS	1810	NAA60	NAA60	chr16	+	3484698	3485083	3484698	3485061	3485466	3486963	3	3	0	1	0	0	0	0	0	1	71	49	1	1	0.674	1	NA	NA	0	0.837
A5SS	1813	RNASEK	RNASEK	chr17	+	7012416	7012761	7012416	7012757	7013665	7013742	20	8	4	2	1	2	0	4	0	2	53	49	1	1	0.902	0.881	0.649	NA	0.649	0.162
A5SS	1819	CAMK2G	CAMK2G	chr10	-	73825278	73825347	73825320	73825347	73824039	73824084	26	12	10	0	1	0	0	2	0	1	91	49	1	1	1	0.866	1	NA	0.519	0.436
A5SS	1823	PABPC4	PABPC4	chr1	-	39564422	39564542	39564470	39564542	39563613	39563741	10	2	0	2	0	0	0	1	0	0	97	49	1	1	0.716	1	NA	NA	1	-0.142
A5SS	1832	METTL10	METTL10	chr10	-	124789038	124789157	124789042	124789157	124774674	124774782	0	3	0	0	0	0	0	0	0	1	53	49	1	1	NA	1	NA	NA	0	1
A5SS	1835	UBE4A	UBE4A	chr11	+	118372506	118372687	118372506	118372666	118373085	118373288	2	1	0	14	1	0	0	0	0	1	70	49	1	1	0.091	0.412	NA	NA	0	0.252
A5SS	1856	NUP98	NUP98	chr11	-	3760487	3760626	3760538	3760626	3753315	3753408	40	18	0	24	18	4	0	4	0	2	98	49	1	1	0.455	0.333	0	NA	0.5	-0.237
A5SS	1859	ZSWIM8	ZSWIM8	chr10	+	73800010	73800194	73800010	73800170	73800295	73800472	7	3	4	6	0	3	0	1	0	0	73	49	1	1	0.439	1	0.472	NA	1	-0.363
A5SS	1864	KLF6	KLF6	chr10	-	3781640	3782214	3781766	3782214	3780105	3780229	132	44	18	2	0	0	3	35	0	0	98	49	1	1	0.971	1	1	1	1	-0.01
A5SS	1890	SF1	SF1	chr11	-	64767566	64767844	64767570	64767844	64767191	64767251	0	3	0	8	0	1	0	0	0	3	53	49	1	1	0	1	0	NA	0	0.333
A5SS	1901	SUPT7L	SUPT7L	chr2	-	27663323	27663840	27663328	27663840	27662178	27662281	1	0	0	2	0	0	0	0	0	3	54	49	1	1	0.312	NA	NA	NA	0	0.312
A5SS	1907	P3H1	P3H1	chr1	-	42747252	42747412	42747271	42747412	42746334	42746852	0	0	0	1	0	0	0	1	0	0	68	49	1	1	0	NA	NA	NA	1	-1
A5SS	1909	HDAC9	HDAC9	chr7	+	18591515	18591642	18591515	18591636	18593907	18594029	6	0	0	0	0	0	0	2	0	1	55	49	1	1	1	NA	NA	NA	0.641	0.359
A5SS	1910	MICAL2	MICAL2	chr11	+	12138389	12138464	12138389	12138460	12162078	12162419	7	2	0	2	5	0	0	1	0	0	53	49	1	1	0.764	0.27	NA	NA	1	-0.483
A5SS	1929	NPL	NPL	chr1	+	182806144	182806531	182806144	182806232	182812155	182812213	12	8	0	37	9	6	0	10	0	0	98	49	1	1	0.14	0.308	0	NA	1	-0.851
A5SS	1936	MADD	MADD	chr11	+	47284940	47285194	47284940	47285065	47285450	47285590	7	0	0	6	0	0	2	5	0	0	98	49	1	1	0.368	NA	NA	1	1	-0.632
A5SS	1950	ABI3	ABI3	chr17	+	49216530	49216698	49216530	49216680	49217738	49217915	6	2	1	3	0	0	0	1	0	0	67	49	1	1	0.594	1	1	NA	1	-0.135
A5SS	1952	JMJD4	JMJD4	chr1	-	227732880	227733027	227732928	227733027	227731188	227732676	1	0	0	1	0	0	0	1	0	0	97	49	1	1	0.336	NA	NA	NA	1	-0.664
A5SS	2000	THTPA	THTPA	chr14	+	23555981	23557304	23555981	23557034	23558694	23559581	1	0	0	0	0	0	0	0	0	1	98	49	1	1	1	NA	NA	NA	0	1
A5SS	2008	ENTPD4	ENTPD4	chr8	-	23441568	23441723	23441592	23441723	23439748	23439915	5	2	0	4	0	1	0	2	0	0	73	49	1	1	0.456	1	0	NA	1	-0.515
A5SS	2014	ANAPC15	ANAPC15	chr11	-	72111411	72111496	72111441	72111496	72111156	72111286	2	0	2	6	0	0	0	2	0	0	79	49	1	1	0.171	NA	1	NA	1	-0.414
A5SS	2035	HPS5	HPS5	chr11	-	18321945	18322174	18322132	18322174	18317750	18317907	2	1	0	0	0	1	0	1	0	0	98	49	1	1	1	1	0	NA	1	-0.333
A5SS	2040	ZNF276	ZNF276	chr16	+	89727278	89727357	89727278	89727325	89729234	89729318	11	3	1	4	2	0	0	8	0	2	81	49	1	1	0.625	0.476	1	NA	0.708	-0.008
A5SS	2055	NDRG1	NDRG1	chr8	-	133296736	133297304	133297133	133297304	133284248	133284329	2	0	0	3	0	0	0	0	0	2	98	49	1	1	0.25	NA	NA	NA	0	0.25
A5SS	2058	TTC19	TTC19	chr17	+	16000117	16000382	16000117	16000245	16001914	16002025	0	3	0	0	0	0	0	0	0	1	98	49	1	1	NA	1	NA	NA	0	1
A5SS	2073	GOSR1	GOSR1	chr17	+	30481142	30481263	30481142	30481257	30484213	30484301	3	1	0	5	0	2	0	1	0	0	55	49	1	1	0.348	1	0	NA	1	-0.551
A5SS	2086	STAT3	STAT3	chr17	-	42322281	42322494	42322284	42322494	42317181	42317224	61	32	30	16	10	10	0	19	0	4	52	49	1	1	0.782	0.751	0.739	NA	0.817	-0.06
A5SS	2095	AOC2	AOC2	chr17	+	42849085	42849371	42849085	42849290	42849600	42849730	12	2	0	0	1	0	1	0	0	1	98	49	1	1	1	0.5	NA	1	0	0.25
A5SS	2101	TMEM230	TMEM230	chr20	-	5112729	5113087	5112960	5113087	5109331	5109445	0	2	0	1	0	0	0	0	0	2	98	49	1	1	0	1	NA	NA	0	0.5
A5SS	2102	TMEM230	TMEM230	chr20	-	5112742	5113087	5112960	5113087	5109331	5109445	2	2	0	1	0	0	0	0	0	2	98	49	1	1	0.5	1	NA	NA	0	0.75
A5SS	2104	KDM2A	KDM2A	chr11	+	67250085	67250798	67250085	67250346	67252693	67252857	30	10	3	1	0	0	2	7	0	0	98	49	1	1	0.937	1	1	1	1	-0.021
A5SS	2117	BRPF1	BRPF1	chr3	+	9742024	9742171	9742024	9742153	9742943	9743253	6	0	1	1	3	0	0	1	0	2	67	49	1	1	0.814	0	1	NA	0.268	0.337
A5SS	2120	SUPT4H1	SUPT4H1	chr17	-	58352064	58352238	58352066	58352238	58351401	58351508	5	1	0	12	8	1	0	5	0	0	51	49	1	1	0.286	0.107	0	NA	1	-0.869
A5SS	2126	CLTC	CLTC	chr17	+	59644275	59644495	59644275	59644483	59647397	59647666	2	0	0	12	3	3	0	0	0	1	61	49	1	1	0.118	0	0	NA	0	0.039
A5SS	2135	LYN	LYN	chr8	+	55941854	55941991	55941854	55941928	55946447	55946493	101	55	27	28	24	5	0	16	0	3	98	49	1	1	0.643	0.534	0.73	NA	0.727	-0.091
A5SS	2138	SP140	SP140	chr2	+	230244987	230245080	230244987	230245071	230245862	230245940	3	6	0	3	0	0	2	3	0	0	58	49	1	1	0.458	1	NA	1	1	-0.271
A5SS	2147	PRKAR1A	PRKAR1A	chr17	+	68512378	68512579	68512378	68512548	68515393	68515576	1	0	1	26	8	2	0	0	0	1	80	49	1	1	0.023	0	0.234	NA	0	0.086
A5SS	2151	CTAGE5	CTAGE5	chr14	+	39276933	39277065	39276933	39276955	39279336	39279358	43	21	4	2	0	1	1	6	0	0	98	49	1	1	0.915	1	0.667	1	1	-0.139
A5SS	2152	CTAGE5	CTAGE5	chr14	+	39291018	39291111	39291018	39291096	39293270	39293381	1	1	0	2	5	0	0	0	0	3	64	49	1	1	0.277	0.133	NA	NA	0	0.205
A5SS	2190	ARHGDIA	ARHGDIA	chr17	-	81869329	81869625	81869541	81869625	81869172	81869236	21	15	3	1	0	0	3	16	0	1	98	49	1	1	0.913	1	1	1	0.889	0.027
A5SS	2225	DDX17	DDX17	chr22	-	38487872	38488115	38487878	38488115	38483437	38486440	8	1	2	27	5	0	0	0	0	2	55	49	1	1	0.209	0.151	1	NA	0	0.453
A5SS	2247	DYRK1A	DYRK1A	chr21	+	37506098	37506354	37506098	37506223	37511910	37515376	1	4	0	10	2	0	0	3	0	0	98	49	1	1	0.048	0.5	NA	NA	1	-0.726
A5SS	2270	ADGRE1	ADGRE1	chr19	+	6928144	6928310	6928144	6928211	6934986	6935078	1	1	0	2	6	0	0	0	0	4	98	49	1	1	0.2	0.077	NA	NA	0	0.139
A5SS	2278	CREM	CREM	chr10	+	35195901	35196142	35195901	35196099	35206894	35207051	14	3	0	1	0	0	3	1	0	0	92	49	1	1	0.882	1	NA	1	1	-0.059
A5SS	2284	TMEM205	TMEM205	chr19	-	11346007	11346305	11346126	11346305	11345519	11345628	1	0	2	3	0	0	0	2	0	1	98	49	1	1	0.143	NA	1	NA	0.5	0.072
A5SS	2311	CYP4F3	CYP4F3	chr19	+	15640896	15640945	15640896	15640929	15641414	15641613	1	1	1	0	4	0	4	0	0	0	65	49	1	1	1	0.159	1	1	NA	-0.28
A5SS	2312	EPS15L1	EPS15L1	chr19	-	16440861	16440909	16440884	16440909	16437769	16437865	8	7	0	1	0	0	0	2	0	0	72	49	1	1	0.845	1	NA	NA	1	-0.078
A5SS	2314	MYO9B	MYO9B	chr19	+	17211646	17211786	17211646	17211774	17211894	17213295	5	3	1	7	7	0	0	2	0	2	61	49	1	1	0.365	0.256	1	NA	0.445	0.095
A5SS	2327	CDV3	CDV3	chr3	+	133574433	133574756	133574433	133574592	133575038	133575115	10	0	0	2	0	0	0	1	0	0	98	49	1	1	0.714	NA	NA	NA	1	-0.286
A5SS	2334	ING4	ING4	chr12	-	6652935	6653050	6652944	6653050	6652661	6652767	4	3	0	1	0	2	0	2	0	0	58	49	1	1	0.772	1	0	NA	1	-0.409
A5SS	2343	CUTA	CUTA	chr6	-	33417868	33418288	33417958	33418288	33417480	33417695	1	1	0	0	1	0	0	0	0	1	98	49	1	1	1	0.333	NA	NA	0	0.666
A5SS	2365	C19orf47	C19orf47	chr19	-	40341785	40341890	40341838	40341890	40336319	40336407	0	1	0	0	0	0	0	0	0	1	98	49	1	1	NA	1	NA	NA	0	1
A5SS	2383	CLASRP	CLASRP	chr19	+	45068014	45068058	45068014	45068054	45068419	45068480	0	1	0	1	1	0	0	0	0	1	53	49	1	1	0	0.48	NA	NA	0	0.24
A5SS	2389	CNOT8	CNOT8	chr5	+	154858518	154858816	154858518	154858768	154863206	154863395	7	5	0	6	0	0	0	3	0	2	97	49	1	1	0.371	1	NA	NA	0.431	0.255
A5SS	2403	CD37	CD37	chr19	+	49335419	49335609	49335419	49335575	49335713	49335786	41	11	2	2	0	0	3	10	0	0	83	49	1	1	0.924	1	1	1	1	-0.025
A5SS	2509	ZNF341	ZNF341	chr20	+	33757147	33757343	33757147	33757322	33758715	33758806	1	0	0	0	0	0	0	0	1	0	70	49	1	1	1	NA	NA	0	NA	1
A5SS	2533	ATAD2B	ATAD2B	chr2	-	23786026	23786223	23786041	23786223	23782868	23783028	7	2	1	0	3	0	0	0	0	1	64	49	1	1	1	0.338	1	NA	0	0.779
A5SS	2545	ZBTB8OS	ZBTB8OS	chr1	-	32650401	32650590	32650432	32650590	32634767	32634792	1	0	0	6	8	0	0	1	0	0	80	49	1	1	0.093	0	NA	NA	1	-0.954
A5SS	2548	CLIP4	CLIP4	chr2	+	29157203	29157402	29157203	29157347	29160332	29160467	2	0	0	2	0	0	2	0	0	0	98	49	1	1	0.333	NA	NA	1	NA	-0.667
A5SS	2564	SS18L1	SS18L1	chr20	+	62158671	62158942	62158671	62158748	62159876	62159961	3	0	0	0	3	2	0	0	0	1	98	49	1	1	1	0	0	NA	0	0.333
A5SS	2565	VRK2	VRK2	chr2	+	58046641	58046868	58046641	58046821	58048826	58048967	0	2	0	4	0	0	0	2	0	1	96	49	1	1	0	1	NA	NA	0.505	-0.005
A5SS	2585	MAP4K4	MAP4K4	chr2	+	101887087	101887261	101887087	101887237	101887777	101887937	18	17	4	41	11	9	0	2	0	5	73	49	1	1	0.228	0.509	0.23	NA	0.212	0.11
A5SS	2648	GOLGB1	GOLGB1	chr3	-	121669196	121669355	121669211	121669355	121668060	121668158	1	0	0	5	0	0	0	1	0	1	64	49	1	1	0.133	NA	NA	NA	0.434	-0.301
A5SS	2649	GOLGB1	GOLGB1	chr3	-	121730847	121730973	121730875	121730973	121729864	121730017	3	3	0	10	5	0	0	2	0	0	77	49	1	1	0.16	0.276	NA	NA	1	-0.782
A5SS	2650	SP100	SP100	chr2	+	230467119	230467215	230467119	230467206	230469042	230469096	26	33	4	4	0	0	3	13	0	1	58	49	1	1	0.846	1	1	1	0.917	-0.01
A5SS	2658	CCDC66	CCDC66	chr3	+	56566593	56566759	56566593	56566740	56566949	56567053	6	0	7	13	4	0	0	1	0	0	68	49	1	1	0.25	0	1	NA	1	-0.583
A5SS	2663	ATG4B	ATG4B	chr2	+	241671311	241671425	241671311	241671405	241672190	241673856	2	3	0	0	2	3	0	2	0	0	69	49	1	1	1	0.516	0	NA	1	-0.495
A5SS	2710	HM13	HM13	chr20	+	31568077	31568280	31568077	31568224	31569119	31569567	3	2	0	0	3	0	0	1	0	0	98	49	1	1	1	0.25	NA	NA	1	-0.375
A5SS	2715	POLD4	POLD4	chr11	-	67353293	67353596	67353302	67353596	67352987	67353077	2	0	0	3	3	0	0	0	0	1	58	49	1	1	0.36	0	NA	NA	0	0.18
A5SS	2722	CPNE1	CPNE1	chr20	-	35664759	35664956	35664801	35664956	35632794	35632923	7	5	0	3	2	0	0	2	0	0	91	49	1	1	0.557	0.574	NA	NA	1	-0.434
A5SS	2725	C20orf24	C20orf24	chr20	+	36609600	36609758	36609600	36609730	36612009	36612557	7	4	2	20	30	28	0	1	0	4	77	49	1	1	0.182	0.078	0.043	NA	0.137	-0.036
A5SS	2737	NCOA3	NCOA3	chr20	+	47649004	47649109	47649004	47649097	47650981	47651276	6	3	0	0	2	0	0	2	0	0	61	49	1	1	1	0.546	NA	NA	1	-0.227
A5SS	2749	ZGPAT	ZGPAT	chr20	+	63708027	63708188	63708027	63708118	63708552	63709164	1	0	0	2	1	0	0	2	0	0	98	49	1	1	0.2	0	NA	NA	1	-0.9
A5SS	2787	SUMO3	SUMO3	chr21	-	44813861	44814104	44813975	44814104	44809046	44809118	1	0	0	3	0	1	0	0	0	1	98	49	1	1	0.143	NA	0	NA	0	0.071
A5SS	2797	TANGO2	TANGO2	chr22	+	20036759	20037094	20036759	20036854	20043354	20043443	3	5	0	4	2	3	0	2	0	7	98	49	1	1	0.273	0.556	0	NA	0.125	0.151
A5SS	2798	TANGO2	TANGO2	chr22	+	20061529	20061683	20061529	20061681	20063337	20063442	0	6	2	1	0	0	0	2	0	0	51	49	1	1	0	1	1	NA	1	-0.333
A5SS	2810	SMARCB1	SMARCB1	chr22	+	23791755	23791894	23791755	23791867	23793558	23793688	1	0	0	1	1	0	0	1	0	1	76	49	1	1	0.392	0	NA	NA	0.392	-0.196
A5SS	2815	ADORA2A	ADORA2A	chr22	+	24427561	24427746	24427561	24427726	24433130	24433736	3	1	0	2	0	0	3	0	0	0	69	49	1	1	0.516	1	NA	1	NA	-0.242
A5SS	2816	ADORA2A	ADORA2A	chr22	+	24427561	24427746	24427561	24427726	24440582	24442361	2	2	0	0	2	0	3	0	0	0	69	49	1	1	1	0.415	NA	1	NA	-0.292
A5SS	2828	FAM193A	FAM193A	chr4	+	2694945	2695129	2694945	2695082	2696362	2696593	7	3	2	1	0	0	2	2	0	0	96	49	1	1	0.781	1	1	1	1	-0.073
A5SS	2829	FAM193A	FAM193A	chr4	+	2694945	2695142	2694945	2695082	2696362	2696593	5	2	2	1	0	0	0	2	0	0	98	49	1	1	0.714	1	1	NA	1	-0.095
A5SS	2831	TOM1	TOM1	chr22	+	35346929	35346972	35346929	35346969	35347054	35347994	3	0	2	2	0	3	0	0	0	5	52	49	1	1	0.586	NA	0.386	NA	0	0.486
A5SS	2835	MAFF	MAFF	chr22	+	38201931	38202238	38201931	38202212	38213822	38213889	5	1	0	3	4	0	0	0	0	2	75	49	1	1	0.521	0.14	NA	NA	0	0.331
A5SS	2847	TLE3	TLE3	chr15	-	70058149	70058291	70058185	70058291	70057458	70057658	20	2	3	5	2	1	0	2	0	1	85	49	1	1	0.698	0.366	0.634	NA	0.536	0.03
A5SS	2849	TLE3	TLE3	chr15	-	70074502	70074607	70074532	70074607	70066013	70066218	21	12	3	27	10	5	0	7	0	4	79	49	1	1	0.325	0.427	0.271	NA	0.52	-0.179
A5SS	2855	ARSA	ARSA	chr22	-	50627555	50628173	50627945	50628173	50627165	50627406	8	5	2	1	0	0	1	0	0	0	98	49	1	1	0.8	1	1	1	NA	-0.067
A5SS	2860	ITPR1	ITPR1	chr3	+	4675067	4675248	4675067	4675221	4676613	4676801	1	3	0	4	1	0	0	1	0	3	76	49	1	1	0.139	0.659	NA	NA	0.177	0.222
A5SS	2869	CASP8AP2	CASP8AP2	chr6	+	89829899	89830106	89829899	89830067	89846561	89846644	1	2	0	1	0	0	3	0	0	0	88	49	1	1	0.358	1	NA	1	NA	-0.321
A5SS	2873	FGD5-AS1	FGD5-AS1	chr3	-	14947086	14947505	14947113	14947505	14942778	14946154	3	2	1	1	1	0	0	1	0	0	76	49	1	1	0.659	0.563	1	NA	1	-0.259
A5SS	2877	MS4A6A	MS4A6A	chr11	-	60175401	60175611	60175505	60175611	60173027	60173129	21	1	18	5	0	0	0	27	0	0	98	49	1	1	0.677	1	1	NA	1	-0.108
A5SS	2883	GORASP1	GORASP1	chr3	-	39100746	39100877	39100787	39100877	39100304	39100503	2	0	1	2	0	0	1	0	0	0	90	49	1	1	0.353	NA	1	1	NA	-0.324
A5SS	2889	LPPR2	LPPR2	chr19	+	11363712	11363835	11363712	11363819	11364160	11364212	8	4	6	9	0	2	0	2	0	1	65	49	1	1	0.401	1	0.693	NA	0.601	0.097
A5SS	2911	ENY2	ENY2	chr8	+	109334322	109334474	109334322	109334402	109336127	109336204	1	1	3	0	0	0	0	1	0	2	98	49	1	1	1	1	1	NA	0.2	0.8
A5SS	2921	ERBB2IP	ERBB2IP	chr5	+	66044136	66044310	66044136	66044298	66046352	66046538	31	16	9	3	2	0	0	8	0	0	61	49	1	1	0.892	0.865	1	NA	1	-0.081
A5SS	2940	PARP9	PARP9	chr3	-	122558328	122558467	122558433	122558467	122555285	122556121	8	7	0	20	9	2	0	10	0	6	98	49	1	1	0.167	0.28	0	NA	0.455	-0.306
A5SS	2941	ZXDC	ZXDC	chr3	-	126461269	126462220	126461534	126462220	126459652	126459737	6	3	0	4	0	0	6	4	0	0	98	49	1	1	0.429	1	NA	1	1	-0.285
A5SS	2942	ACAD9	ACAD9	chr3	+	128879489	128880017	128879489	128879841	128884652	128884746	1	2	0	1	1	0	0	4	0	2	98	49	1	1	0.333	0.5	NA	NA	0.5	-0.084
A5SS	2944	MBD4	MBD4	chr3	-	129436442	129437308	129436460	129437308	129434061	129434136	4	0	0	24	4	2	0	0	0	1	67	49	1	1	0.109	0	0	NA	0	0.036
A5SS	2945	MBD4	MBD4	chr3	-	129437719	129437950	129437807	129437950	129436460	129437308	24	13	3	1	0	1	1	2	0	0	98	49	1	1	0.923	1	0.6	1	1	-0.159
A5SS	2963	MYNN	MYNN	chr3	+	169774264	169774561	169774264	169774431	169778767	169779561	16	4	1	1	0	0	0	1	0	0	98	49	1	1	0.889	1	1	NA	1	-0.037
A5SS	2981	SMARCA2	SMARCA2	chr9	+	2086828	2087071	2086828	2086897	2088499	2088613	14	3	2	2	0	0	0	1	0	0	98	49	1	1	0.778	1	1	NA	1	-0.074
A5SS	2984	TMEM175	TMEM175	chr4	+	948115	948615	948115	948154	950420	950518	4	1	2	0	0	0	0	0	0	1	98	49	1	1	1	1	1	NA	0	1
A5SS	2998	EXOC1	EXOC1	chr4	+	55853649	55853953	55853649	55853797	55858313	55858447	5	2	2	4	4	0	0	1	0	0	98	49	1	1	0.385	0.2	1	NA	1	-0.472
A5SS	3003	CNOT6L	CNOT6L	chr4	-	77819232	77819390	77819303	77819390	77776270	77776392	13	12	0	1	0	0	0	1	0	0	98	49	1	1	0.867	1	NA	NA	1	-0.067
A5SS	3006	SNX10	SNX10	chr7	+	26291894	26292133	26291894	26292086	26346419	26346466	7	1	1	9	1	2	0	0	0	2	96	49	1	1	0.284	0.338	0.203	NA	0	0.275
A5SS	3007	ANTXR2	ANTXR2	chr4	-	80072408	80073323	80072708	80073323	80071582	80071654	46	15	16	1	0	0	10	10	0	0	98	49	1	1	0.958	1	1	1	1	-0.014
A5SS	3019	CNN2	CNN2	chr19	+	1032558	1032696	1032558	1032663	1036129	1036246	117	73	37	0	3	0	8	20	0	0	82	49	1	1	1	0.936	1	1	1	-0.021
A5SS	3056	PAIP1	PAIP1	chr5	-	43556581	43557093	43556818	43557093	43555829	43555999	2	0	0	1	1	0	1	1	0	0	98	49	1	1	0.5	0	NA	1	1	-0.75
A5SS	3059	GAPT	GAPT	chr5	+	58493618	58493838	58493618	58493683	58494246	58496358	7	1	0	4	1	0	0	2	0	2	98	49	1	1	0.467	0.333	NA	NA	0.333	0.067
A5SS	3065	C14orf159	C14orf159	chr14	+	91173288	91173439	91173288	91173403	91175933	91176056	66	21	10	8	4	0	0	12	0	2	85	49	1	1	0.826	0.752	1	NA	0.776	0.083
A5SS	3078	GLRX	GLRX	chr5	-	95816495	95816626	95816506	95816626	95813848	95814389	0	3	0	10	3	1	0	0	0	3	60	49	1	1	0	0.45	0	NA	0	0.15
A5SS	3089	HINT1	HINT1	chr5	-	131165059	131165348	131165094	131165348	131162571	131162676	3	0	0	23	5	0	0	0	0	5	84	49	1	1	0.071	0	NA	NA	0	0.035
A5SS	3090	CNBP	CNBP	chr3	-	129171633	129171771	129171654	129171771	129171445	129171538	8	7	10	24	12	13	0	4	0	5	70	49	1	1	0.189	0.29	0.35	NA	0.359	-0.083
A5SS	3100	ETF1	ETF1	chr5	-	138542633	138542936	138542832	138542936	138518691	138518867	36	7	0	20	18	0	0	2	2	4	98	49	1	1	0.474	0.163	NA	0	0.2	0.218
A5SS	3106	SCARF1	SCARF1	chr17	-	1645559	1645789	1645596	1645789	1645177	1645239	2	3	1	5	0	0	0	0	0	1	86	49	1	1	0.186	1	1	NA	0	0.729
A5SS	3113	ARHGAP26	ARHGAP26	chr5	+	143207197	143207473	143207197	143207308	143213996	143214088	8	0	2	6	4	0	0	0	0	1	98	49	1	1	0.4	0	1	NA	0	0.467
A5SS	3114	ZNF438	ZNF438	chr10	-	30848530	30850367	30850221	30850367	30844635	30845573	12	4	2	1	0	0	1	7	0	0	98	49	1	1	0.857	1	1	1	1	-0.048
A5SS	3115	CSNK1A1	CSNK1A1	chr5	-	149505446	149505595	149505482	149505595	149495893	149496860	35	10	0	10	3	0	0	11	0	1	85	49	1	1	0.669	0.658	NA	NA	0.864	-0.201
A5SS	3122	FAM193B	FAM193B	chr5	-	177553167	177554547	177554248	177554547	177538904	177539147	16	4	0	1	0	0	0	4	0	0	98	49	1	1	0.889	1	NA	NA	1	-0.055

A5SS	3123	PHYKPL	PHYKPL	chr5	-	178231668	178232822	178232491	178232822	178231404	178231523	1	0	0	0	0	0	0	3	2	0	98	49	1	1	1	NA	NA		0	1	0.5
A5SS	3129	MCMBP	MCMBP	chr10	-	119843247	119843426	119843253	119843426	119842471	119842595	0	2	7	2	1	2	0	0	0	3	55	49	1	1	0	0.641	0.757	NA		0	0.466
SE	20	VAMP7	VAMP7	chrX	+	155900496	155900587	155898111	155898249	155919812	155919880	12	7	3	1	0	0	0	2	0	0	98	49	1	1	0.857	1	1	NA		1	-0.048
SE	30	FLNA	FLNA	chrX	-	154352552	154352675	154352180	154352447	154352771	154352924	85	28	9	1	0	0	9	44	0	1	98	49	1	1	0.977	1	1		1	0.957	0.014
SE	31	FLNA	FLNA	chrX	-	154353553	154353727	154353295	154353457	154353914	154354043	72	9	10	1	1	0	2	35	0	1	98	49	1	1	0.973	0.818	1		1	0.946	-0.043
SE	32	FLNA	FLNA	chrX	-	154354615	154354711	154354380	154354483	154354824	154355072	79	21	7	0	0	0	2	25	0	1	98	49	1	1	1	1	1		1	0.926	0.037
SE	34	FLNA	FLNA	chrX	-	154359989	154360587	154359731	154359905	154361307	154361570	88	21	6	2	0	0	8	61	0	0	98	49	1	1	0.957	1	1		1	1	-0.014
SE	35	FLNA	FLNA	chrX	-	154361669	154361787	154361307	154361570	154361978	154362148	49	27	7	2	0	0	0	22	0	0	98	49	1	1	0.925	1	1	NA		1	-0.025
SE	38	IDH3G	IDH3G	chrX	-	153786800	153786947	153786354	153786449	153787050	153787153	8	0	3	1	0	3	2	2	0	0	98	49	1	1	0.8	NA	0.333		1	1	-0.433
SE	49	IDS	IDS	chrX	-	149491537	149491667	149490313	149490440	149496345	149496516	0	0	1	60	26	19	0	1	0	7	98	49	1	1	0	0	0.026	NA		0.067	-0.058
SE	50	IDS	IDS	chrX	-	149498106	149498307	149496345	149496516	149500948	149501037	102	48	33	0	1	2	0	9	0	0	98	49	1	1	1	0.96	0.892	NA		1	-0.049
SE	53	IDS	IDS	chrX	-	149504156	149504293	149500948	149501037	149505034	149505354	42	12	17	1	1	0	1	14	0	1	98	49	1	1	0.955	0.857	1		1	0.875	0
SE	54	ZNF75D	ZNF75D	chrX	-	135293729	135294258	135291008	135291135	135295767	135296039	5	0	0	0	0	0	0	0	0	1	98	49	1	1	1	NA	NA	NA		0	1
SE	55	MOSPD1	MOSPD1	chrX	-	134891478	134891640	134887625	134889192	134896816	134897034	1	1	0	0	0	0	0	0	0	1	98	49	1	1	1	1	NA	NA		0	1
SE	62	ELF4	ELF4	chrX	-	130081255	130081539	130074580	130074752	130110324	130110500	9	9	2	2	0	0	2	5	1	0	98	49	1	1	0.692	1	1		0.5	1	0.147
SE	69	STAG2	STAG2	chrX	+	124021366	124021431	123960559	123960866	124022530	124022671	23	8	2	4	0	0	0	2	0	0	98	49	1	1	0.742	1	1	NA		1	-0.086
SE	70	STAG2	STAG2	chrX	+	124021366	124021431	123961705	123961856	124022530	124022671	24	7	4	13	2	3	0	2	0	3	98	49	1	1	0.48	0.636	0.4	NA		0.25	0.255
SE	71	STAG2	STAG2	chrX	+	124090853	124090964	124090574	124090764	124094017	124094144	7	2	2	43	16	4	0	0	0	3	98	49	1	1	0.075	0.059	0.2	NA		0	0.111
SE	72	XIAP	XIAP	chrX	+	123885630	123886539	123860166	123860293	123888618	123888718	6	0	2	1	0	0	0	1	0	0	98	49	1	1	0.75	NA	1	NA		1	-0.125
SE	83	LONRF3	LONRF3	chrX	+	119013038	119013201	119011814	119011973	119014206	119014356	0	0	0	1	0	0	0	1	0	0	98	49	1	1	0	NA	NA	NA		1	-1
SE	84	DOCK11	DOCK11	chrX	+	118590209	118590302	118588412	118588478	118593213	118593337	77	26	27	7	3	0	0	15	0	1	98	49	1	1	0.846	0.812	1	NA		0.882	0.004
SE	85	DOCK11	DOCK11	chrX	+	118639434	118639577	118638079	118638127	118641189	118641305	46	22	1	2	0	0	0	4	0	0	98	49	1	1	0.92	1	1	NA		1	-0.027
SE	86	DOCK11	DOCK11	chrX	+	118643456	118643594	118641189	118641305	118648944	118649127	42	22	10	0	1	0	0	10	0	0	98	49	1	1	1	0.917	1	NA		1	-0.028
SE	87	DOCK11	DOCK11	chrX	+	118681693	118681794	118681057	118681248	118683078	118683217	40	19	8	1	0	0	1	6	0	0	98	49	1	1	0.952	1	1		1	1	-0.016
SE	94	ACSL4	ACSL4	chrX	-	109661530	109661645	109659353	109659511	109663210	109663402	24	14	10	1	0	0	1	2	0	0	98	49	1	1	0.923	1	1		1	1	-0.026
SE	123	DIAPH2	DIAPH2	chrX	+	96735757	96735790	96684662	96685190	96738585	96738762	2	1	2	1	1	0	0	1	0	0	82	49	1	1	0.544	0.374	1	NA		1	-0.361
SE	136	PGK1	PGK1	chrX	+	78113743	78113899	78109866	78109917	78114015	78114160	33	16	18	1	0	0	0	6	0	0	98	49	1	1	0.943	1	1	NA		1	-0.019
SE	139	ATRX	ATRX	chrX	-	77600433	77600564	77599731	77599820	77616612	77616730	13	7	0	0	0	0	0	2	0	2	98	49	1	1	1	1	NA	NA		0.333	0.667
SE	140	ATRX	ATRX	chrX	-	77693823	77693937	77688817	77688927	77696576	77696704	15	1	5	10	0	0	0	3	0	0	98	49	1	1	0.429	1	1	NA		1	-0.19
SE	153	ABCB7	ABCB7	chrX	-	75114753	75114834	75112885	75112972	75156104	75156340	1	1	1	1	0	0	0	1	0	0	98	49	1	1	0.333	1	1	NA		1	-0.222
SE	154	RLIM	RLIM	chrX	-	74612581	74612643	74595808	74596000	74614421	74614626	6	3	5	2	1	1	0	0	0	1	98	49	1	1	0.6	0.6	0.714	NA		0	0.638
SE	159	NHSL2	NHSL2	chrX	+	72137093	72137225	72134508	72134704	72138440	72140771	9	3	0	3	1	0	0	2	0	2	98	49	1	1	0.6	0.6	NA	NA		0.333	0.267
SE	160	TAF1	TAF1	chrX	+	71388236	71388378	71387260	71387461	71388737	71388868	9	0	2	0	0	0	0	3	0	1	98	49	1	1	1	NA	1	NA		0.6	0.4
SE	165	NLGN3	NLGN3	chrX	+	71153476	71153536	71147549	71148206	71155213	71155363	1	0	0	0	3	0	1	0	0	0	98	49	1	1	1	0	NA		1	NA	-0.5
SE	167	MED12	MED12	chrX	+	71125350	71125495	71124975	71125146	71125662	71125713	11	4	7	2	0	0	0	3	0	0	98	49	1	1	0.733	1	1	NA		1	-0.089
SE	168	MED12	MED12	chrX	+	71132844	71132956	71132376	71132538	71133122	71133212	5	9	1	1	0	0	0	6	0	0	98	49	1	1	0.714	1	1	NA		1	-0.095
SE	170	IL2RG	IL2RG	chrX	-	71110155	71110295	71109227	71109390	71110503	71110688	52	18	18	5	2	0	0	7	0	0	98	49	1	1	0.839	0.818	1	NA		1	-0.114
SE	171	IL2RG	IL2RG	chrX	-	71110896	71111050	71110503	71110688	71111424	71111631	58	14	18	0	1	0	1	12	0	0	98	49	1	1	1	0.875	1		1	1	-0.042
SE	172	SNX12	SNX12	chrX	-	71061842	71061967	71059246	71061118	71062853	71062949	7	1	0	0	1	0	0	3	2	0	98	49	1	1	1	0.333	NA		0	1	0.166
SE	177	IGBP1	IGBP1	chrX	+	70146632	70146828	70134522	70134816	70148760	70148840	21	12	5	0	1	0	2	1	0	0	98	49	1	1	1	0.857	1		1	1	-0.048
SE	189	TSR2	TSR2	chrX	+	54440689	54440780	54440419	54440502	54443399	54443491	0	0	0	0	2	0	0	1	0	0	98	49	1	1	NA	0	NA	NA		1	-1
SE	190	PHF8	PHF8	chrX	-	53993600	53993903	53992735	53992839	53995692	53995782	8	2	1	1	0	0	0	0	0	1	98	49	1	1	0.8	1	1	NA		0	0.933
SE	191	HUWE1	HUWE1	chrX	-	53544559	53544762	53543840	53543968	53545028	53545161	5	2	0	0	2	0	1	5	0	0	98	49	1	1	1	0.333	NA		1	1	-0.334
SE	192	HUWE1	HUWE1	chrX	-	53603367	53603501	53602563	53602658	53604588	53604834	12	2	0	0	0	0	0	1	0	2	98	49	1	1	1	1	NA	NA		0.2	0.8
SE	205	WDR45	WDR45	chrX	-	49075135	49075281	49074432	49074912	49075363	49075465	8	4	10	1	0	0	0	5	0	0	98	49	1	1	0.8	1	1	NA		1	-0.067
SE	208	PIM2	PIM2	chrX	-	48918535	48918645	48917780	48917831	48918773	48919136	22	13	11	1	0	0	0	2	0	0	98	49	1	1	0.917	1	1	NA		1	-0.028
SE	209	TIMM17B	TIMM17B	chrX	-	48895351	48895501	48895037	48895101	48896758	48896858	2	0	0	5	3	2	0	0	0	2	98	49	1	1	0.167	0	0	NA		0	0.056
SE	210	TBC1D25	TBC1D25	chrX	+	48544868	48545023	48541332	48541442	48558896	48559024	3	4	3	0	1	0	1	1	0	0	98	49	1	1	1	0.667	1		1	1	-0.111
SE	225	CDK16	CDK16	chrX	+	47225771	47225866	47224987	47225102	47225964	47226027	15	8	1	2	0	0	1	4	0	0	98	49	1	1	0.789	1	1		1	1	-0.07
SE	227	UBA1	UBA1	chrX	+	47199208	47199377	47199047	47199106	47199479	47199614	18	5	6	1	0	0	0	5	0	0	98	49	1	1	0.9	1	1	NA		1	-0.033
SE	228	UBA1	UBA1	chrX	+	47211035	47211225	47210841	47210916	47212423	47212512	19	7	9	1	0	0	0	10	0	0	98	49	1	1	0.905	1	1	NA		1	-0.032
SE	241	DDX3X	DDX3X	chrX	+	41346858	41347012	41346504	41346622	41347311	41347451	172	69	39	6	3	0	0	35	0	0	98	49	1	1	0.935	0.92	1	NA		1	-0.048
SE	242	DDX3X	DDX3X	chrX	+	41346858	41347012	41346504	41346622	41347314	41347451	146	54	29	0	2	0	0	33	0	0	98	49	1	1	1	0.931	1	NA		1	-0.023
SE	243	DDX3X	DDX3X	chrX	+	41346858	41347012	41346504	41346622	41347639	41347915	108	47	22	1	2	0	0	27	0	0	98	49	1	1	0.982	0.922	1	NA		1	-0.032
SE	244	DDX3X	DDX3X	chrX	+	41347311	41347451	41346504	41346622	41347639	41347915	108	30	29	1	2	0	1	11	0	0	98	49	1	1	0.982	0.882	1		1	1	-0.045
SE	248	USP9X	USP9X	chrX	+	41136803	41137022	41134724	41134837	41140655	41140771	53	19	6	0	1	0	1	4	0	0	98	49	1	1	1	0.905	1		1	1	-0.032
SE	249	USP9X	USP9X	chrX	+	41140655	41140771	41136803	41137022	41140965	41141217	41	25	3	1	0	0	1	4	0	1	98	49	1	1	0.953	1	1		1	0.667	0.151
SE	250	USP9X	USP9X	chrX	+	41150920	41151057	41148368	41148575	41152947	41153081	29	11	2	4	0	0	0	10	0	0	98	49	1	1	0.784	1	1	NA		1	-0.072
SE	251	USP9X	USP9X	chrX	+	41169994	41170235	41168006	41168218	41170469	41170619	37	15	1	0	1	0	0	5	0	0	98	49	1	1	1	0.882	1	NA		1	-0.039
SE	252	USP9X	USP9X	chrX	+	41214567	41214709	41210508	41210682	41215898	41216652	53	12	2	1	0	0	0	2	0	0	98	49	1	1	0.964	1	1	NA		1	-0.012
SE	253	USP9X	USP9X	chrX	+	41219101	41219231	41218371	41218597	41223216	41223402	32	6	7	1	0	0	0	1	0	0	98	49	1	1	0.941	1	1	NA		1	-0.02
SE	254	ATP6AP2	ATP6AP2	chrX	+	40597248	40597344	40591233	40591365	40597526	40597664	35	4	3	1	0	0	4	11	0	0	98	49	1	1	0.946	1	1		1	1	-0.018
SE	267	GK	GK	chrX	+	30668011	30668118	30665510	30665584	30677374	30677452	29	11	6	6	9	3	0	4	0	0	98	49	1	1	0.707	0.379	0.5	NA		1	-0.471
SE	270	GK	GK	chrX	+	30694399	30694537	30677374	30677452	30696041	30696151	13	4	10	0	1	0	0	1	0	0	98	49	1	1	1	0.667	1	NA		1	-0.111
SE	271	GK	GK	chrX	+	30694399	30694537	30691122	30691199	30696041	30696151	13	4	8	9	2	0	0	1	0	0	98	49	1	1	0.419	0.5	1	NA		1	-0.36
SE	272	GK	GK	chrX	+	30697731	30697749	30696616	30696683	30700413	30700449	0	0	9	15	10	4	0	1	0	2	67	49	1	1	0	0	0.622	NA		0.268	-0.061
SE	274	GK	GK	chrX	+	30727465	30727552	30724100	30724181	30728731	30731460	3	0	0	33	25	9	0	0	2	3	98	49	1	1	0.043	0	0		0	0	0.014
SE	275	ACOT9	ACOT9	chrX	-	23713234	23713208	23707876	23707944	23721880	23721984	18	11	2	6	0	0	0	9	0	0	98	49	1	1	0.6	1	1	NA		1	-0.133
SE	281	SH3KBP1	SH3KBP1	chrX	-	19545921	19546050	19541924	19542193	19549973	19550083	21	6	4	0	1	0	0	2	0	0	98	49	1	1	1	0.75	1	NA		1	-0.083
SE	283	SH3KBP1	SH3KBP1	chrX	-	19706880	19706984	19695611	19695741	19746317	19746441	54	27	15	1	0	0	0	3	0	0	98	49	1	1	0.964	1	1	NA		1	-0.012
SE	293	PHKA2	PHKA2	chrX	-	18899172	18899226	18897162	18897333	18900669	18900699	3	1	0	4	0	3	0	1	0	0	98	49	1	1	0.273	1	0	NA		1	-0.576
SE	294	PHKA2	PHKA2	chrX	-	18900669	18900699	18897162	18897333	18901484	18901603	8	0	3	3	0	0	1	2	0	0	79	49	1	1	0.623	NA	1		1	1	-0.189
SE	295	PHKA2	PHKA2	chrX	-	18943709	18943808	18941528	18941675	18945077	18945158	6	1	0	1	0	0	2	4	0	0	98	49	1	1	0.75	1	NA		1	1	-0.125
SE	300	REPS2	REPS2	chrX	+	17022122	17022271	17006220	17006344	17029525	17029623	12	4	5	1	0	0	0	0	0	1	98	49	1	1	0.857	1	1	NA		0	0.952
SE	301	REPS2	REPS2	chrX	+	17025058	17025185	17022122	17022271	17029525	17029623	12	5	3	1	1	4	0	2	0	0	98	49	1	1	0.857	0.714	0.273	NA		1	-0.385
SE	302	REPS2	REPS2	chrX	+	17025061	17025185	17022122	17022271	17029525	17029623	20	7	6	1	1	4	0	1	0	0	98	49	1	1	0.909	0.778	0.429	NA		1	-0.295
SE	303	SYAP1	SYAP1	chrX	+	16754944	16755093	16743700	16743840	16756662	16756721	13	9	0	2	0	0	1	19	0	0	98	49	1	1	0.765	1	NA		1	1	-0.117
SE	305	MOSPD2	MOSPD2	chrX	+	14915667	14915764	14914502	14914599	14916196	14916326	12	3	2	0	2	0	0	1	0	0	98	49	1	1	1	0.429	1	NA		1	-0.19
SE	317	WWC3	WWC3	chrX	+	10125023	10125110	10124264	10124339	10126113	10126306	26	7	2	0	1	0	0	3	0	0	98	49	1	1	1	0.778	1	NA		1	-0.074
SE	318	WWC3	WWC3	chrX	+	10126113	10126306	10125023	10125110	10127987	10128161	20	4	8	1	0	0	0	3	0	0	98	49	1	1	0.909	1	1	NA		1	-0.03
SE	319	WWC3	WWC3	chrX	+	10127987	10128161	10126113	10126306	10128556	10128708	23	8	16	0	1	0	0	2	0	0	98	49	1	1	1	0.8	1	NA		1	-0.067
SE	321	TBL1X	TBL1X	chrX	+	9653544	9653689	9640272	9640360	9654214	9654322	6	6	9	4	3	1	0	1	0	3	98	49	1	1	0.429	0.5	0.818	NA		0.143	0.439
SE	322	TBL1X	TBL1X	chrX	+	9684042	9684188	9654214	9654322	9688016	9688275	39	9	7	0	2	0	0	4	0	0	98	49	1	1	1	0.692	1	NA		1	-0.103
SE	330	DHRSX	DHRSX	chrX	-	2243022	2243230	2219513	2221229	2266739	2266947	4	0	2	0	1	0	0	2	0	0	98	49	1	1	1	0	1	NA		1	-0.333
SE	347	CCDC183-AS1	CCDC183-AS1	chr9	-	136806049	136806128	136803926	136805584	136806384	136806537	0	0	0	1	0	0	0	1	0	0	98	49	1	1	0	NA	NA	NA		1	-1
SE	348	SNHG7	SNHG7	chr9	-	136726310	136726416	136724593	136725110	136727045	136727086	1	0	0	0	1	0	0	2	0	0	98	49	1	1	1	0	NA	NA		1	-0.5
SE	351	NOTCH1	NOTCH1	chr9	-	136511151	136511271	136510652	136510805	136513020	136513134	138	61	22	0	1	0	9	22	0	0	98	49	1	1	1	0.968	1		1	1	-0.011
SE	353	NOTCH1	NOTCH1	chr9	-	136518136	136518292	136517751	136517937	136518590	136518824	89	54	24	1	0	0	7	26	0	0	98	49	1	1	0.978	1	1		1	1	-0.007
SE	354	NOTCH1	NOTCH1	chr9	-	136544023	136544102	136519442	136519565	136545725	136545786	60	27	10	0	1	0	6	16	0	0	98	49	1	1	1	0.931	1		1	1	-0.023
SE	356	SEC16A	SEC16A	chr9	-	136445644	136445719	136443822	136443900	136446854	136446949	6	3	2	0	1	0	0	2	0	0	98	49	1	1	1	0.6	1	NA		1	-0.133
SE	357	SEC16A	SEC16A	chr9	-	136468414	136468512	136466956	136467083	136471974	136472111	6	1	0	0	0	0	1	1	0	0	98	49	1	1	1	1	NA		0	1	0.5
SE	359	PMPCA	PMPCA	chr9	+	136414552	136414647	136412809	136412892	136416290	136416391	3	0	0	0	0	0	0	0	0	1	98	49	1	1	1	NA	NA	NA		0	1
SE	360	PMPCA	PMPCA	chr9	+	136414554	136414647	136412809	136412892	136416290	136416391	1	0	0	0	0	0	0	0	0	1	98	49	1	1	1	NA	NA	NA		0	1
SE	369	BRD3	BRD3	chr9	-	134036031	134036324	134034700	134034829	134041759	134041951	2	1	0	0	1	0	0	1	0	0	98	49	1	1	1	0.333	NA	NA		1	-0.334
SE	380	SURF6	SURF6	chr9	-	133333717	133333806	133332547	133332760	133334391	133334601	0	0	2	0	0	0	0	1	0	1	98	49	1	1	NA	NA	1	NA		0.333	0.667
SE	383	RALGDS	RALGDS	chr9	-	133107084	133107300	133106644	133106748	133107987	133108406	1	0	0	0	0	0	2	1	0	1	98	49	1	1	1	NA	NA		1	0.333	0.334
SE	388	RAPGEF1	RAPGEF1	chr9	-	131630235	131630324	131629101	131629254	131638634	131638791	25	12	3	2	1	0	0	6	0	2	98	49	1	1	0.862	0.857	1	NA		0.6	0.306
SE	391	PRRC2B	PRRC2B	chr9	+	131446400	131446642	131444184	131444328	131447084	131447206	7	3	0	1	0	0	0	2	0	1	98	49	1	1	0.778	1	NA	NA		0.5	0.389
SE	392	PRRC2B	PRRC2B	chr9	+	131474453	131476535	131473507	131473724	131477743	131477949	5	0	2	1	0	0	0	3	0	0	98	49	1	1	0.714	NA	1	NA		1	-0.143
SE	393	PRRC2B	PRRC2B	chr9	+	131494404	131494486	131492168	131492260	131495739	131500188	0	2	0	2	0	0	0	1	0	0	98	49	1	1	0	1	NA	NA		1	-0.5
SE	412	GOLGA2	GOLGA2	chr9	-	128267193	128267274	128266286	128266325	128267457	128267517	6	2	3	0	2	0	0	3	0	0	98	49	1	1	1	0.333	1	NA		1	-0.222
SE	422	LRSAM1	LRSAM1	chr9	+	127497252	127497334	127495963	127496095	127501009	127501143	4	2	0	0	0	0	0	0	0	1	98	49	1	1	1	1	NA	NA		0	1
SE	425	RALGPS1	RALGPS1	chr9	+	127050042	127050132	127034430	127034514	127052846	127052939	8	5	0	0	0	0	0	0	0	1	98	49	1	1	1	1	NA	NA		0	1
SE	430	MAPKAP1	MAPKAP1	chr9	-	125672315	125672643	125669817	125669907	125706970	125707234	8	3	0	3	0	0	0	3	0	2	98	49	1	1	0.571	1	NA	NA		0.429	0.356
SE	435	DENND1A	DENND1A	chr9	-	123450692	123450749	123440359	123440491	123452275	123452347	9	2	1	1	0	0	0	5	0	0	98	49	1	1	0.818	1	1	NA		1	-0.061
SE	436	DENND1A	DENND1A	chr9	-	123769513	123769563	123757702	123757822	123792586	123792630	12	8	1	0	0	0	0	3	0	0	98	49	1	1	0.857	1	1	NA		1	-0.048
SE	442	RBM18	RBM18	chr9	-	122254273	122254367	122251846	122251973	122261379	122261508	0	0	0	2	4	0	0	0	0	4	98	49	1	1	0	0	NA	NA		0.111	-0.111
SE	448	GSN	GSN	chr9	+	121282438	121282550	121281469	121281562	121301962	121302167	1	0	0	5	5	3	0	0	0	4	98	49	1	1	0.091	0	0	NA		0	0.03
SE	449	GSN	GSN	chr9	+	121283318	121283392	121281469	121281562	121301962	121302167	0	0	1	5	5	3	0	0	0	4	98	49	1	1	0	0	0.143	NA		0	0.048
SE	450	GSN	GSN	chr9	+	121300055	121300126	121281469	121281562	121301962	121302167	3	0	0	5	5	3	0	0	0	4	98	49	1	1	0.231	0	0	NA		0	0.077
SE	451	GSN	GSN	chr9	+	121300064	121300126	121281469	121281562	121301962	121302167	3	0	0	5	5	3	0	0	0	4	98	49	1	1	0.231	0	0	NA		0	0.077
SE	452	CNTRL	CNTRL	chr9	+	121140640	121140786	121138544	121138679	121142090	121142270	6	1	1	1	0	0	0	5	0	0	98	49	1	1	0.75	1	1	NA		1	-0.083
SE	453	CNTRL	CNTRL	chr9	+	121141380	121141588	121140640	121140786	121142090	121142270	3	6	4	4	1	0	0	2	0	0	98	49	1	1	0.273	0.75	1	NA		1	-0.326
SE	462	TLR4	TLR4	chr9	+	117708198	117708318	117704174	117704565	117708562	117708729	93	18	11	13	5	14	0	18	0	5	98	49	1	1	0.782	0.643	0.282	NA		0.643	-0.074
SE	463	TLR4	TLR4	chr9	+	117708198	117708318	117704174	117704565	117712388	117717491	80	13	10	66	14	7	0	14	0	10	98	49	1	1	0.377	0.317	0.417	NA		0.412	-0.042
SE	464	TLR4	TLR4	chr9	+	117708562	117708729	117704174	117704565	117712388	117717491	72	13	36	66	14	7	0	10	0	10	98	49	1	1	0.353	0.317	0.72	NA		0.333	0.13
SE	465	TLR4	TLR4	chr9	+	117708562	117708729	117708198	117708318	117712388	117717491	74	13	23	1	0	0	0	9	0	0	98	49	1	1	0.974	1	1	NA		1	-0.009
SE	470	PTBP3	PTBP3	chr9	-	112224132	112224210	112217714	112223983	112227410	112227627	67	38	24	0	2	0	1	15	0	0	98	49	1	1	1	0.905	1		1	1	-0.032
SE	471	PTBP3	PTBP3	chr9	-	112262434	112262599	112252677	112252788	112268048	112268195	54	21	32	1	1	0	2	17	0	0	98	49	1	1	0.964	0.913	1		1	1	-0.041
SE	473	PTBP3	PTBP3	chr9	-	112330440	112330474	112297831	112297916	112333469	112333664	2	0	0	53	5	6	0	0	0	3	83	49	1	1	0.022	0	0	NA		0	0.007
SE	476	UGCG	UGCG	chr9	+	111931270	111931357	111929499	111929678	111932169	111932359	9	10	2	1	0	0	0	3	0	0	98	49	1	1	0.818	1	1	NA		1	-0.061
SE	478	DNAJC25	DNAJC25	chr9	+	111642856	111643094	111631351	111631743	111647106	111647259	1	1	0	0	1	0	1	1	0	0	98	49	1	1	1	0.333	NA		1	1	-0.334
SE	479	KIAA0368	KIAA0368	chr9	-	111383210	111383332	111378579	111378730	111384521	111384569	6	2	2	1	0	0	0	4	0	0	98	49	1	1	0.75	1	1	NA		1	-0.083
SE	488	ABCA1	ABCA1	chr9	-	104785395	104785639	104781002	104784455	104786297	104786390	33	7	8	1	0	0	0	2	0	0	98	49	1	1	0.943	1	1	NA		1	-0.019
SE	489	ABCA1	ABCA1	chr9	-	104786872	104786976	104786297	104786390	104787919	104788054	9	2	0	8	1	0	0	1	0	1	98	49	1	1	0.36	0.5	NA	NA		0.333	0.097
SE	490	ABCA1	ABCA1	chr9	-	104798420	104798598	104796308	104796424	104799818	104799988	11	10	6	3	0	0	0	2	0	0	98	49	1	1	0.647	1	1	NA		1	-0.118
SE	493	ABCA1	ABCA1	chr9	-	104819585	104819723	104818662	104818883	104819926	104820069	56	29	10	1	0	0	1	17	0	0	98	49	1	1	0.966	1	1		1	1	-0.011
SE	498	RNF20	RNF20	chr9	+	101544766	101544885	101540792	101540975	101546819	101546966	4	2	1	0	1	0	0	1	0	0	98	49	1	1	1	0.5	1	NA		1	-0.167
SE	502	ZNF189	ZNF189	chr9	+	101399394	101399530	101398853	101399189	101399925	101400010	1	0	0	0	0	0	0	0	0	1	98	49	1	1	1	NA	NA	NA		0	1
SE	511	PHF2	PHF2	chr9	+	93645628	93645789	93636410	93636525	93649070	93649212	27	3	5	2	0	0	0	3	0	0	98	49	1	1	0.871	1	1	NA		1	-0.043
SE	512	FAM120A	FAM120A	chr9	+	93558580	93558718	93557826	93558010	93561108	93561250	34	12	14	0	0	0	0	12	0	1	98	49	1	1	1	1	1	NA		0.857	0.143
SE	524	SEMA4D	SEMA4D	chr9	-	89418055	89418118	89405350	89405699	89455887	89455953	17	6	0	4	0	0	0	5	0	0	98	49	1	1	0.68	1	NA	NA		1	-0.16
SE	525	SEMA4D	SEMA4D	chr9	-	89418362	89418437	89418055	89418118	89455887	89455953	8	1	0	4	2	0	0	2	0	0	98	49	1	1	0.5	0.2	NA	NA		1	-0.65
SE	526	SECISBP2	SECISBP2	chr9	+	89357410	89357565	89350631	89350852	89357998	89358191	8	2	1	3	0	0	0	2	0	0	98	49	1	1	0.571	1	1	NA		1	-0.143
SE	527	SPIN1	SPIN1	chr9	+	88462495	88462749	88448940	88448989	88468371	88468605	0	0	0	2	0	0	0	2	0	0	98	49	1	1	0	NA	NA	NA		1	-1
SE	530	DAPK1	DAPK1	chr9	+	87686550	87686739	87681403	87681626	87697006	87697204	6	3	5	0	1	0	0	7	0	0	98	49	1	1	1	0.6	1	NA		1	-0.133
SE	531	DAPK1	DAPK1	chr9	+	87697006	87697204	87686550	87686739	87698655	87698794	8	4	0	1	1	0	0	3	0	0	98	49	1	1	0.8	0.667	NA	NA		1	-0.266
SE	537	UBQLN1	UBQLN1	chr9	-	83666349	83666433	83665029	83665145	83669184	83669327	17	3	10	3	0	0	0	4	0	1	98	49	1	1	0.739	1	1	NA		0.667	0.246
SE	539	GNAQ	GNAQ	chr9	-	77794462	77794592	77728513	77728667	77797519	77797648	36	21	13	0	0	0	0	9	0	1	98	49	1	1	1	1	1	NA		0.818	0.182
SE	545	TRPM6	TRPM6	chr9	-	74738406	74738612	74732684	74732736	74739366	74739449	5	2	6	1	0	0	0	2	0	0	98	49	1	1	0.714	1	1	NA		1	-0.095
SE	546	TRPM6	TRPM6	chr9	-	74761998	74763134	74761695	74761808	74771702	74771835	9	6	5	1	0	1	0	1	0	0	98	49	1	1	0.818	1	0.714	NA		1	-0.156
SE	548	TRPM6	TRPM6	chr9	-	74788613	74788742	74785873	74786125	74792623	74792770	2	6	3	4	2	0	0	0	1	0	98	49	1	1	0.2	0.6	1		0	NA	0.6
SE	550	TRPM6	TRPM6	chr9	-	74821668	74821837	74820303	74820427	74827777	74827949	22	8	7	0	2	0	2	4	0	0	98	49	1	1	1	0.667	1		1	1	-0.111
SE	551	TRPM6	TRPM6	chr9	-	74842165	74842343	74840023	74840237	74855526	74855565	13	9	4	0	2	0	0	4	0	0	98	49	1	1	1	0.692	1	NA		1	-0.103
SE	557	PTAR1	PTAR1	chr9	-	69750780	69750950	69741791	69741858	69759852	69759960	4	4	0	0	1	0	0	1	0	0	98	49	1	1	1	0.667	NA	NA		1	-0.166
SE	560	LINC01506	LINC01506	chr9	-	68545716	68545836	68545500	68545624	68546558	68546602	9	8	17	0	0	1	0	1	0	0	98	49	1	1	1	1	0.895	NA		1	-0.035
SE	565	FRG1HP	FRG1HP	chr9	+	40993252	40993434	40992336	40992775	40994632	40994699	3	0	0	1	0	0	0	1	0	0	98	49	1	1	0.6	NA	NA	NA		1	-0.4
SE	566	SLC25A51	SLC25A51	chr9	-	37903530	37903557	37899828	37899950	37904067	37904353	2	0	2	1	0	0	0	0	0	1	76	49	1	1	0.563	NA	1	NA		0	0.781
SE	567	DCAF10	DCAF10	chr9	+	37842088	37842286	37819287	37819401	37854779	37854982	1	2	0	2	0	0	0	1	0	0	98	49	1	1	0.2	1	NA	NA		1	-0.4
SE	594	RNF38	RNF38	chr9	-	36342324	36342424	36336400	36339814	36344831	36344953	10	4	0	0	0	0	0	1	0	2	98	49	1	1	1	1	NA	NA		0.2	0.8
SE	602	ZCCHC6	ZCCHC6	chr9	-	86309462	86309576	86309211	86309289	86309927	86310017	48	40	12	2	0	0	0	12	0	0	98	49	1	1	0.923	1	1	NA		1	-0.026
SE	603	ZCCHC6	ZCCHC6	chr9	-	86318957	86319058	86317218	86317276	86319583	86319670	49	13	19	26	5	0	0	8	0	0	98	49	1	1	0.485	0.565	1	NA		1	-0.317
SE	605	ZCCHC6	ZCCHC6	chr9	-	86322324	86322475	86319583	86319670	86322872	86323960	66	41	27	1	0	0	0	6	0	0	98	49	1	1	0.971	1	1	NA		1	-0.01
SE	606	ZCCHC6	ZCCHC6	chr9	-	86346298	86346480	86345668	86345785	86352679	86353230	269	107	11	0	1	0	0	27	0	0	98	49	1	1	1	0.982	1	NA		1	-0.006
SE	609	TLN1	TLN1	chr9	-	35699355	35699461	35699031	35699156	35699973	35700081	195	53	21	0	3	0	9	45	0	0	98	49	1	1	1	0.898	1		1	1	-0.034

SE	610	TLN1	TLN1	chr9	-	35699973	35700081	35699355	35699461	35700190	35700376	207	50	28	1	0	0	11	39	0	0	98	49	1	1	0.99	1	1	1	1	-0.003
SE	612	TLN1	TLN1	chr9	-	35712834	35713043	35712004	35712124	35713195	35713298	75	34	30	0	1	0	11	20	0	0	98	49	1	1	1	0.944	1	1	1	-0.019
SE	613	TLN1	TLN1	chr9	-	35716389	35716556	35715058	35715187	35717145	35717440	178	63	47	2	0	0	14	44	0	0	98	49	1	1	0.978	1	1	1	1	-0.007
SE	614	TLN1	TLN1	chr9	-	35717145	35717440	35715058	35715187	35717618	35717786	40	14	13	1	0	0	0	10	0	0	98	49	1	1	0.952	1	1	NA	1	-0.016
SE	616	TLN1	TLN1	chr9	-	35725564	35725727	35725223	35725321	35732074	35732395	80	26	10	0	0	0	1	14	0	1	98	49	1	1	1	1	1	1	0.875	0.062
SE	619	IL11RA	IL11RA	chr9	+	34657034	34657149	34656738	34656908	34657302	34657335	3	1	0	2	0	0	0	1	0	0	98	49	1	1	0.429	1	NA	NA	1	-0.285
SE	627	B4GALT1	B4GALT1	chr9	-	33115990	33116113	33113773	33113878	33120418	33120606	10	8	2	0	3	0	0	1	0	1	98	49	1	1	1	0.571	1	NA	0.333	0.524
SE	628	DNAJA1	DNAJA1	chr9	+	33026812	33026990	33026474	33026616	33029884	33029989	11	14	3	2	0	0	0	6	0	1	98	49	1	1	0.733	1	1	NA	0.75	0.161
SE	664	KDM4C	KDM4C	chr9	+	6849506	6849700	6814630	6814745	6880011	6880061	5	3	9	0	0	0	0	4	0	2	98	49	1	1	1	1	1	NA	0.5	0.5
SE	665	KDM4C	KDM4C	chr9	+	6981841	6982765	6980924	6981118	6984165	6984404	1	0	0	2	2	0	0	0	0	3	98	49	1	1	0.2	0	NA	NA	0	0.1
SE	666	UHRF2	UHRF2	chr9	+	6420911	6421142	6413150	6413643	6433913	6434173	14	1	0	0	0	1	0	4	0	0	98	49	1	1	1	1	0	NA	1	-0.333
SE	667	UHRF2	UHRF2	chr9	+	6433913	6434173	6420911	6421142	6460572	6460791	13	7	0	0	3	0	0	2	0	0	98	49	1	1	1	0.538	NA	NA	1	-0.231
SE	670	RIC1	RIC1	chr9	+	5689958	5690038	5656582	5656690	5713895	5714003	14	0	1	0	0	1	0	9	0	0	98	49	1	1	1	NA	0.333	NA	1	-0.334
SE	671	RIC1	RIC1	chr9	+	5745930	5746083	5743688	5743737	5747301	5747505	8	0	3	1	0	0	0	9	0	0	98	49	1	1	0.8	NA	1	NA	1	-0.1
SE	672	RIC1	RIC1	chr9	+	5753535	5753646	5753199	5753238	5754840	5754930	8	4	0	2	0	0	0	3	0	0	98	49	1	1	0.667	1	NA	NA	1	-0.166
SE	677	RFX3	RFX3	chr9	-	3270370	3270525	3266207	3266305	3271002	3271118	6	0	0	1	0	0	0	0	0	2	98	49	1	1	0.75	NA	NA	NA	0	0.75
SE	678	RFX3	RFX3	chr9	-	3301545	3301620	3293076	3293258	3330258	3330517	0	0	0	3	0	0	0	4	0	0	98	49	1	1	0	NA	NA	NA	1	-1
SE	682	DOCK8	DOCK8	chr9	+	304580	304704	289509	289581	311953	312166	236	104	21	0	2	0	7	41	0	0	98	49	1	1	1	0.963	1	1	1	-0.012
SE	683	DOCK8	DOCK8	chr9	+	379770	379935	376976	377211	382512	382685	81	40	9	1	0	0	2	12	0	0	98	49	1	1	0.976	1	1	1	1	-0.008
SE	685	DOCK8	DOCK8	chr9	+	420948	421078	420400	420583	422047	422135	81	19	24	6	0	0	0	11	0	1	98	49	1	1	0.871	1	1	NA	0.846	0.111
SE	694	GRINA	GRINA	chr8	+	143991877	143992078	143991691	143991804	143992244	143992373	9	2	9	2	0	0	0	5	0	0	98	49	1	1	0.692	1	1	NA	1	-0.103
SE	695	PARP10	PARP10	chr8	-	143983166	143983811	143982931	143983065	143984007	143984104	16	5	2	0	0	1	4	3	0	0	98	49	1	1	1	1	0.5	1	1	-0.167
SE	696	PARP10	PARP10	chr8	-	143985411	143985648	143984543	143985328	143985720	143985975	10	2	0	0	0	0	3	2	0	1	98	49	1	1	1	1	NA	1	0.5	0.25
SE	697	PLEC	PLEC	chr8	-	143922503	143925884	143915146	143922395	143926783	143926882	4	2	0	0	0	2	0	5	0	0	98	49	1	1	1	1	0	NA	1	-0.333
SE	698	NAPRT	NAPRT	chr8	-	143577061	143577177	143576645	143576842	143577268	143577399	8	0	7	3	0	0	2	4	0	0	98	49	1	1	0.571	NA	1	1	1	-0.215
SE	702	DENND3	DENND3	chr8	+	141136557	141136791	141128620	141128809	141150833	141150953	4	2	0	0	3	0	0	1	0	0	98	49	1	1	1	0.25	NA	NA	1	-0.375
SE	703	DENND3	DENND3	chr8	+	141138021	141138137	141136557	141136791	141150833	141150953	64	64	11	1	0	0	3	16	0	0	98	49	1	1	0.97	1	1	1	1	-0.01
SE	704	DENND3	DENND3	chr8	+	141138021	141138137	141136557	141136791	141151618	141151837	53	54	11	1	0	0	3	15	0	0	98	49	1	1	0.964	1	1	1	1	-0.012
SE	705	DENND3	DENND3	chr8	+	141141202	141141324	141138021	141138137	141144147	141144259	87	36	18	0	0	0	0	17	0	1	98	49	1	1	1	1	1	NA	0.895	0.105
SE	706	DENND3	DENND3	chr8	+	141144147	141144259	141138021	141138137	141150833	141150953	22	14	1	11	11	0	0	6	0	2	98	49	1	1	0.5	0.389	1	NA	0.6	0.03
SE	707	DENND3	DENND3	chr8	+	141150833	141150953	141136557	141136791	141151618	141151837	84	39	10	1	0	0	0	20	0	0	98	49	1	1	0.977	1	1	NA	1	-0.008
SE	708	DENND3	DENND3	chr8	+	141150833	141150953	141138021	141138137	141151618	141151837	94	50	10	0	1	0	0	22	0	1	98	49	1	1	1	0.962	1	NA	0.917	0.07
SE	709	DENND3	DENND3	chr8	+	141151618	141151837	141138021	141138137	141155848	141155970	73	51	17	1	0	0	1	31	0	0	98	49	1	1	0.973	1	1	1	1	-0.009
SE	710	DENND3	DENND3	chr8	+	141160631	141160787	141155848	141155970	141163332	141163429	75	32	15	5	0	0	0	19	0	0	98	49	1	1	0.882	1	1	NA	1	-0.039
SE	711	DENND3	DENND3	chr8	+	141160631	141160787	141155848	141155970	141165185	141165289	52	20	10	1	0	0	0	11	0	1	98	49	1	1	0.963	1	1	NA	0.846	0.142
SE	712	DENND3	DENND3	chr8	+	141163332	141163429	141155848	141155970	141165185	141165289	60	17	13	1	0	0	0	8	0	1	98	49	1	1	0.968	1	1	NA	0.8	0.189
SE	713	DENND3	DENND3	chr8	+	141163332	141163429	141160631	141160787	141165185	141165289	85	35	21	7	6	3	0	16	0	0	98	49	1	1	0.859	0.745	0.778	NA	1	-0.206
SE	714	DENND3	DENND3	chr8	+	141168003	141168525	141166189	141166389	141175199	141175459	151	71	16	2	0	0	0	36	0	0	98	49	1	1	0.974	1	1	NA	1	-0.009
SE	716	DENND3	DENND3	chr8	+	141192330	141192449	141190283	141190417	141192525	141192663	58	24	6	1	0	0	3	17	0	0	98	49	1	1	0.967	1	1	1	1	-0.011
SE	719	AGO2	AGO2	chr8	-	140558484	140558572	140557088	140557236	140559394	140559529	20	14	10	0	1	0	0	9	0	0	98	49	1	1	1	0.875	1	NA	1	-0.042
SE	731	SLA	SLA	chr8	-	133044983	133045115	133039997	133040130	133047829	133047933	51	33	34	6	2	1	0	35	0	6	98	49	1	1	0.81	0.892	0.944	NA	0.745	0.137
SE	734	TMEM71	TMEM71	chr8	-	132722039	132722115	132714153	132714215	132727797	132727986	72	42	31	1	0	0	0	14	0	0	98	49	1	1	0.973	1	1	NA	1	-0.009
SE	735	TMEM71	TMEM71	chr8	-	132727797	132727986	132722039	132722115	132746941	132747057	47	25	29	2	2	5	0	9	0	0	98	49	1	1	0.922	0.862	0.744	NA	1	-0.157
SE	736	TMEM71	TMEM71	chr8	-	132757233	132757294	132751784	132751997	132758839	132758915	62	39	30	2	0	0	3	28	0	0	98	49	1	1	0.939	1	1	1	1	-0.02
SE	737	FAM49B	FAM49B	chr8	-	129862228	129862334	129855610	129855747	129871374	129871496	146	109	79	0	1	0	0	28	0	0	98	49	1	1	1	0.982	1	NA	1	-0.006
SE	738	FAM49B	FAM49B	chr8	-	129879388	129879471	129871374	129871496	129903311	129903350	118	67	33	8	10	2	0	14	0	1	98	49	1	1	0.881	0.77	0.892	NA	0.875	-0.027
SE	740	FAM49B	FAM49B	chr8	-	129896671	129896854	129879388	129879471	129903311	129903350	1	2	0	26	14	7	0	0	0	3	98	49	1	1	0.019	0.067	0	NA	0	0.029
SE	741	FAM49B	FAM49B	chr8	-	129896748	129896854	129879388	129879471	129903311	129903350	0	3	2	26	14	7	0	0	0	3	98	49	1	1	0	0.097	0.125	NA	0	0.074
SE	742	FAM49B	FAM49B	chr8	-	129903311	129903350	129879388	129879471	129970942	129970995	52	31	21	0	2	0	0	6	0	0	88	49	1	1	1	0.896	1	NA	1	-0.035
SE	744	FAM49B	FAM49B	chr8	-	129904498	129904585	129903311	129903350	129970942	129970995	45	17	5	27	22	15	0	3	0	3	98	49	1	1	0.455	0.279	0.143	NA	0.333	-0.041
SE	747	CNTNAP3	CNTNAP3	chr9	-	39140518	39140638	39132931	39133135	39144239	39144346	3	2	2	0	0	0	0	1	0	1	98	49	1	1	1	1	1	NA	0.333	0.667
SE	748	KIAA0196	KIAA0196	chr8	-	125074997	125075111	125073152	125073324	125076347	125076500	6	1	0	0	0	0	0	2	0	1	98	49	1	1	1	1	NA	NA	0.5	0.5
SE	751	TMEM65	TMEM65	chr8	-	124327353	124327421	124323320	124323375	124330747	124330792	3	1	4	2	0	0	0	2	0	0	98	49	1	1	0.429	1	1	NA	1	-0.19
SE	752	FAM91A1	FAM91A1	chr8	+	123774079	123774164	123768641	123768774	123775146	123775298	31	18	8	0	1	0	0	6	0	0	98	49	1	1	1	0.9	1	NA	1	-0.033
SE	753	FAM91A1	FAM91A1	chr8	+	123779984	123780075	123778658	123778772	123780479	123780542	28	15	2	1	0	0	0	6	0	0	98	49	1	1	0.933	1	1	NA	1	-0.022
SE	754	FAM91A1	FAM91A1	chr8	+	123786494	123786610	123785628	123785741	123787260	123787373	17	18	8	3	0	0	0	4	0	1	98	49	1	1	0.739	1	1	NA	0.667	0.246
SE	755	FAM91A1	FAM91A1	chr8	+	123789612	123789745	123787663	123787750	123798089	123798238	13	3	3	0	2	0	0	2	0	0	98	49	1	1	1	0.429	1	NA	1	-0.19
SE	756	FAM91A1	FAM91A1	chr8	+	123806079	123806229	123805266	123805339	123808271	123808376	18	13	5	1	0	0	0	4	0	0	98	49	1	1	0.9	1	1	NA	1	-0.033
SE	760	ATAD2	ATAD2	chr8	-	123337624	123337821	123336372	123336532	123339310	123339446	1	0	0	0	0	0	0	0	0	1	98	49	1	1	1	NA	NA	NA	0	1
SE	767	RAD21	RAD21	chr8	-	116854244	116854468	116852548	116852708	116856165	116856288	82	29	36	1	0	0	2	16	0	0	98	49	1	1	0.976	1	1	1	1	-0.008
SE	768	EIF3H	EIF3H	chr8	-	116726015	116726172	116658812	116658980	116755665	116755823	41	6	2	3	0	0	0	12	0	0	98	49	1	1	0.872	1	1	NA	1	-0.043
SE	769	TRPS1	TRPS1	chr8	-	115623600	115623758	115619131	115620060	115668544	115669028	5	1	0	3	0	0	0	2	0	0	98	49	1	1	0.455	1	NA	NA	1	-0.272
SE	770	TRPS1	TRPS1	chr8	-	115623614	115623758	115619131	115620060	115668544	115669028	0	0	0	3	0	0	0	1	0	0	98	49	1	1	0	NA	NA	NA	1	-1
SE	779	OXR1	OXR1	chr8	+	106745788	106745862	106742221	106742317	106750805	106752693	5	2	0	1	0	0	0	5	0	0	98	49	1	1	0.714	1	NA	NA	1	-0.143
SE	783	SLC25A32	SLC25A32	chr8	-	103404775	103404861	103403163	103403324	103407633	103407784	11	0	0	1	0	0	0	2	0	0	98	49	1	1	0.846	NA	NA	NA	1	-0.154
SE	788	UBR5	UBR5	chr8	-	102271133	102271245	102270038	102270183	102272528	102272772	20	4	1	0	2	0	0	2	0	0	98	49	1	1	1	0.5	1	NA	1	-0.167
SE	789	UBR5	UBR5	chr8	-	102278825	102278939	102276971	102277192	102279035	102279208	16	12	5	2	0	0	0	4	0	0	98	49	1	1	0.8	1	1	NA	1	-0.067
SE	790	UBR5	UBR5	chr8	-	102312341	102312485	102312133	102312245	102313803	102313916	17	15	3	0	1	0	1	4	0	0	98	49	1	1	1	0.882	1	1	1	-0.039
SE	791	YWHAZ	YWHAZ	chr8	-	100923954	100924050	100918575	100920752	100924134	100924298	25	16	6	1	0	0	0	5	0	0	98	49	1	1	0.926	1	1	NA	1	-0.025
SE	792	YWHAZ	YWHAZ	chr8	-	100924134	100924298	100918575	100920752	100924915	100925039	110	47	20	0	0	0	0	26	0	1	98	49	1	1	1	1	1	NA	0.929	0.071
SE	794	RNF19A	RNF19A	chr8	-	100261541	100261755	100259853	100259997	100264033	100264195	16	2	5	1	0	0	0	1	0	0	98	49	1	1	0.889	1	1	NA	1	-0.037
SE	795	RNF19A	RNF19A	chr8	-	100264033	100264195	100261541	100261755	100264670	100264785	11	0	6	2	0	0	0	1	0	0	98	49	1	1	0.733	NA	1	NA	1	-0.133
SE	797	RNF19A	RNF19A	chr8	-	100287500	100288267	100269868	100270013	100309866	100310166	9	9	3	1	0	0	0	3	0	0	98	49	1	1	0.818	1	1	NA	1	-0.061
SE	799	SPAG1	SPAG1	chr8	+	100233410	100233537	100231155	100231288	100239239	100239404	0	8	3	0	2	0	0	1	0	0	98	49	1	1	NA	0.667	1	NA	1	-0.166
SE	803	CPQ	CPQ	chr8	+	96834972	96835180	96784863	96785330	96879797	96880005	64	40	16	1	0	0	1	16	0	0	98	49	1	1	0.97	1	1	1	1	-0.01
SE	804	CPQ	CPQ	chr8	+	96879797	96880005	96834972	96835180	96965934	96966046	57	39	14	3	0	0	0	7	0	0	98	49	1	1	0.905	1	1	NA	1	-0.032
SE	817	TP53INP1	TP53INP1	chr8	-	94932069	94932107	94925971	94930728	94939859	94940220	28	17	6	2	4	1	0	4	0	1	87	49	1	1	0.887	0.705	0.772	NA	0.693	0.095
SE	818	INTS8	INTS8	chr8	+	94856776	94856978	94853804	94853915	94859510	94859632	7	1	1	1	0	0	0	2	0	0	98	49	1	1	0.778	1	1	NA	1	-0.074
SE	822	INTS8	INTS8	chr8	+	94868691	94868813	94867275	94867337	94871883	94872002	1	0	0	2	1	1	0	0	0	1	98	49	1	1	0.2	0	0	NA	0	0.067
SE	825	PDP1	PDP1	chr8	+	93917910	93917945	93916933	93917079	93922015	93926068	1	0	0	3	0	0	0	0	0	2	84	49	1	1	0.163	NA	NA	NA	0	0.163
SE	849	C8orf59	C8orf59	chr8	-	85214920	85215037	85214052	85214631	85217385	85217502	21	3	1	0	0	0	2	3	0	1	98	49	1	1	1	1	1	1	0.6	0.2
SE	853	IMPA1	IMPA1	chr8	-	81670938	81671047	81660515	81660667	81673840	81673949	5	2	7	1	0	0	0	2	0	0	98	49	1	1	0.714	1	1	NA	1	-0.095
SE	855	PAG1	PAG1	chr8	-	80993102	80993307	80991478	80991530	81029995	81030089	9	8	1	0	0	0	0	2	0	1	98	49	1	1	1	1	1	NA	0.5	0.5
SE	856	PAG1	PAG1	chr8	-	81070111	81070170	81029995	81030089	81111590	81112068	8	3	1	1	0	0	2	16	0	0	98	49	1	1	0.8	1	1	1	1	-0.067
SE	869	LY96	LY96	chr8	+	74004795	74004885	73991328	73991554	74010000	74010129	45	39	34	4	0	1	0	10	0	0	98	49	1	1	0.849	1	0.944	NA	1	-0.069
SE	872	UBE2W	UBE2W	chr8	-	73825146	73825249	73810473	73810629	73830380	73830472	32	23	8	1	0	0	0	4	0	0	98	49	1	1	0.941	1	1	NA	1	-0.02
SE	878	NCOA2	NCOA2	chr8	-	70144641	70144848	70141183	70141399	70148272	70148483	10	3	3	8	2	0	0	5	0	1	98	49	1	1	0.385	0.429	1	NA	0.714	-0.109
SE	879	NCOA2	NCOA2	chr8	-	70174755	70174859	70170201	70170379	70213902	70214075	55	23	11	0	2	1	0	9	0	0	98	49	1	1	1	0.852	0.846	NA	1	-0.101
SE	880	ARFGEF1	ARFGEF1	chr8	-	67259814	67259926	67258084	67258290	67266005	67266207	21	14	1	0	0	0	0	6	0	1	98	49	1	1	1	1	1	NA	0.75	0.25
SE	886	PDE7A	PDE7A	chr8	-	65724254	65724351	65723540	65723621	65724776	65724921	11	10	3	2	0	0	0	2	0	0	98	49	1	1	0.733	1	1	NA	1	-0.089
SE	888	CHD7	CHD7	chr8	+	60801527	60801593	60800387	60800525	60808216	60808272	15	15	6	0	1	0	0	2	0	0	98	49	1	1	1	0.882	1	NA	1	-0.039
SE	890	GTF3C5	GTF3C5	chr9	+	133054943	133054964	133054711	133054809	133056011	133056094	0	0	0	2	0	0	1	0	0	0	70	49	1	1	0	NA	NA	1	NA	-1
SE	892	RAB2A	RAB2A	chr8	+	60572045	60572113	60558851	60558923	60584207	60584290	20	12	7	5	1	0	1	0	0	0	98	49	1	1	0.667	0.857	1	1	NA	-0.159
SE	893	CYSLTR1	CYSLTR1	chrX	-	78283453	78283540	78271471	78273773	78327304	78327691	17	15	9	1	0	0	0	4	0	0	98	49	1	1	0.895	1	1	NA	1	-0.035
SE	896	CHCHD7	CHCHD7	chr8	+	56212761	56212889	56211755	56211837	56214597	56214667	0	0	0	0	3	0	0	1	0	0	98	49	1	1	NA	0	NA	NA	1	-1
SE	899	RB1CC1	RB1CC1	chr8	-	52628031	52628168	52624716	52624787	52630469	52630528	21	23	1	5	0	0	0	3	0	0	98	49	1	1	0.677	1	1	NA	1	-0.108
SE	900	RB1CC1	RB1CC1	chr8	-	52628040	52628168	52624716	52624787	52630469	52630528	17	19	0	5	0	0	0	3	0	0	98	49	1	1	0.63	1	NA	NA	1	-0.185
SE	901	RB1CC1	RB1CC1	chr8	-	52660595	52660657	52658872	52658976	52660925	52661007	34	13	8	4	9	0	0	3	0	0	98	49	1	1	0.81	0.419	1	NA	1	-0.257
SE	902	RB1CC1	RB1CC1	chr8	-	52686852	52686967	52685398	52685520	52714074	52714466	30	11	3	3	2	0	1	1	0	0	98	49	1	1	0.882	1	1	1	1	-0.039
SE	905	SPIDR	SPIDR	chr8	+	47396375	47396626	47293866	47294030	47407860	47407961	16	4	3	0	3	0	0	2	0	0	98	49	1	1	1	0.4	1	NA	1	-0.2
SE	909	POMK	POMK	chr8	+	43102504	43102600	43097523	43097615	43103527	43103830	8	1	0	0	0	0	0	0	0	2	98	49	1	1	1	1	NA	NA	0	1
SE	912	RNF170	RNF170	chr8	-	42865415	42865489	42861744	42861855	42870003	42870112	0	0	0	0	0	3	0	6	0	0	98	49	1	1	NA	NA	0	NA	1	-1
SE	914	POLB	POLB	chr8	+	42339011	42339069	42338454	42338685	42344952	42345019	13	2	5	4	0	0	0	3	0	2	98	49	1	1	0.619	1	1	NA	0.429	0.444
SE	915	POLB	POLB	chr8	+	42349015	42349090	42344952	42345019	42350006	42350065	16	20	1	1	0	0	0	1	0	0	98	49	1	1	0.889	1	1	NA	1	-0.037
SE	916	POLB	POLB	chr8	+	42350006	42350065	42344952	42345019	42352518	42352568	12	1	0	0	0	0	0	0	0	1	98	49	1	1	1	1	NA	NA	0	1
SE	917	POLB	POLB	chr8	+	42362611	42362698	42361294	42361365	42369270	42369335	7	5	3	4	2	2	0	1	0	0	98	49	1	1	0.467	0.556	0.429	NA	1	-0.516
SE	919	IKBKB	IKBKB	chr8	+	42288633	42288728	42272082	42272205	42290155	42290273	4	1	0	0	1	0	0	3	0	0	98	49	1	1	1	0.333	NA	NA	1	-0.334
SE	924	KAT6A	KAT6A	chr8	-	41974703	41974822	41955295	41955411	41977007	41977327	47	39	14	1	0	0	2	10	0	0	98	49	1	1	0.959	1	1	1	1	-0.014
SE	925	KAT6A	KAT6A	chr8	-	41987454	41987563	41981838	41981954	42048377	42049302	94	32	16	1	0	0	0	17	0	0	98	49	1	1	0.979	1	1	NA	1	-0.007
SE	938	PLEKHA2	PLEKHA2	chr8	+	38943788	38943837	38935993	38936050	38946123	38946221	20	19	9	0	1	0	0	2	0	0	98	49	1	1	1	0.905	1	NA	1	-0.032
SE	939	PLEKHA2	PLEKHA2	chr8	+	38946123	38946221	38935993	38936050	38950849	38950990	5	8	4	0	0	1	0	4	0	0	98	49	1	1	1	1	0.667	NA	1	-0.111
SE	940	PLEKHA2	PLEKHA2	chr8	+	38952165	38952312	38950849	38950990	38952635	38952704	17	14	9	1	0	0	0	4	0	0	98	49	1	1	0.895	1	1	NA	1	-0.035
SE	941	PLEKHA2	PLEKHA2	chr8	+	38957322	38957386	38953296	38953367	38968591	38968669	9	13	3	0	0	0	0	5	0	1	98	49	1	1	1	1	1	NA	0.714	0.286
SE	943	WHSC1L1	WHSC1L1	chr8	-	38299443	38299590	38295795	38295952	38304586	38304757	23	0	2	1	0	0	0	1	0	0	98	49	1	1	0.92	NA	1	NA	1	-0.04
SE	944	WHSC1L1	WHSC1L1	chr8	-	38299443	38299590	38295795	38295952	38314646	38314773	13	0	2	1	0	0	0	1	0	0	98	49	1	1	0.867	NA	1	NA	1	-0.067
SE	945	WHSC1L1	WHSC1L1	chr8	-	38326729	38326856	38321071	38321172	38329377	38329893	21	5	1	1	0	0	0	1	0	0	98	49	1	1	0.913	1	1	NA	1	-0.029
SE	954	INTS9	INTS9	chr8	-	28846746	28846809	28837636	28837776	28850212	28850273	0	1	0	1	0	0	0	1	0	0	98	49	1	1	0	1	NA	NA	1	-0.5
SE	956	EXTL3	EXTL3	chr8	+	28713456	28713550	28701472	28701659	28715584	28716066	4	3	4	1	0	0	0	1	0	0	98	49	1	1	0.667	1	1	NA	1	-0.111
SE	966	PPP2R2A	PPP2R2A	chr8	+	26363720	26363890	26362683	26362848	26366314	26366406	16	3	2	0	2	0	0	2	0	0	98	49	1	1	1	0.429	1	NA	1	-0.19
SE	967	DOCK5	DOCK5	chr8	+	25268844	25268885	25243673	25243757	25275385	25275441	148	62	18	1	0	0	1	18	0	0	90	49	1	1	0.988	1	1	1	1	-0.004
SE	968	DOCK5	DOCK5	chr8	+	25278568	25278665	25275385	25275441	25292023	25292172	229	94	12	0	3	0	5	32	0	0	98	49	1	1	1	0.94	1	1	1	-0.02
SE	969	DOCK5	DOCK5	chr8	+	25296512	25296648	25292023	25292172	25298943	25299101	181	82	14	2	1	0	3	29	0	0	98	49	1	1	0.978	0.976	1	1	1	-0.015
SE	971	DOCK5	DOCK5	chr8	+	25317006	25317131	25310406	25310532	25319577	25319676	132	75	18	1	0	0	2	30	0	0	98	49	1	1	0.985	1	1	1	1	-0.005
SE	972	DOCK5	DOCK5	chr8	+	25334095	25334196	25332602	25332692	25336238	25336373	175	73	14	1	0	0	1	36	0	0	98	49	1	1	0.989	1	1	1	1	-0.004
SE	973	DOCK5	DOCK5	chr8	+	25340876	25340988	25336238	25336373	25341738	25341809	115	49	6	8	7	0	0	19	0	0	98	49	1	1	0.878	0.778	1	NA	1	-0.115
SE	974	DOCK5	DOCK5	chr8	+	25342400	25342507	25341738	25341809	25345474	25345611	107	55	21	1	0	0	0	15	0	0	98	49	1	1	0.982	1	1	NA	1	-0.006
SE	975	DOCK5	DOCK5	chr8	+	25368191	25368250	25366869	25366970	25368570	25368725	86	32	10	3	0	0	0	11	0	5	98	49	1	1	0.935	1	1	NA	0.524	0.454
SE	977	DOCK5	DOCK5	chr8	+	25373617	25373658	25372558	25372718	25374563	25374654	85	38	16	0	0	0	0	8	0	1	90	49	1	1	1	1	1	NA	0.813	0.187
SE	982	TNFRSF10C	TNFRSF10C	chr8	+	23111719	23111825	23102813	23103181	23114656	23114770	340	217	190	33	15	0	0	51	0	4	98	49	1	1	0.837	0.879	1	NA	0.864	0.041
SE	983	TNFRSF10C	TNFRSF10C	chr8	+	23115507	23115616	23114656	23114770	23116640	23117437	394	187	150	0	1	0	3	45	0	0	98	49	1	1	1	0.989	1	1	1	-0.004
SE	984	CCAR2	CCAR2	chr8	+	22618348	22618495	22617695	22617778	22618616	22618728	2	3	0	1	0	0	0	1	0	0	98	49	1	1	0.5	1	NA	NA	1	-0.25
SE	985	PDLIM2	PDLIM2	chr8	+	22589297	22589374	22585320	22585399	22589595	22589741	5	3	7	0	0	1	0	1	0	0	98	49	1	1	1	1	0.778	NA	1	-0.074
SE	986	PDLIM2	PDLIM2	chr8	+	22591550	22591668	22589595	22589741	22593732	22593832	20	11	13	2	0	0	0	6	0	0	98	49	1	1	0.833	1	1	NA	1	-0.056
SE	988	PPP3CC	PPP3CC	chr8	+	22539468	22539498	22532920	22533018	22540614	22541144	3	0	0	1	0	0	0	1	0	0	79	49	1	1	0.65	NA	NA	NA	1	-0.35
SE	994	XPO7	XPO7	chr8	+	21990810	21990919	21990343	21990407	21991867	21991974	3	0	3	1	0	0	0	2	0	0	98	49	1	1	0.6	NA	1	NA	1	-0.2
SE	995	DOK2	DOK2	chr8	-	21911900	21911988	21910672	21910857	21912228	21912510	10	2	1	1	0	0	0	7	0	2	98	49	1	1	0.833	1	1	NA	0.636	0.308
SE	998	ATP6V1B2	ATP6V1B2	chr8	+	20210568	20210646	20210345	20210439	20211176	20211316	141	47	27	1	0	0	4	14	0	0	98	49	1	1	0.986	1	1	1	1	-0.005
SE	999	ATP6V1B2	ATP6V1B2	chr8	+	20214817	20214968	20212781	20212905	20216412	20216495	93	25	30	1	1	0	0	19	0	0	98	49	1	1	0.979	0.926	1	NA	1	-0.032
SE	1000	ATP6V1B2	ATP6V1B2	chr8	+	20216412	20216495	20212781	20212905	20217219	20217324	41	18	22	1	0	0	0	19	0	0	98	49	1	1	0.953	1	1	NA	1	-0.016
SE	1015	ASAH1	ASAH1	chr8	-	18062278	18062423	18061685	18061740	18063184	18063230	84	50	32	1	0	0	0	18	0	0	98	49	1	1	0.977	1	1	NA	1	-0.008
SE	1016	ASAH1	ASAH1	chr8	-	18063184	18063230	18062278	18062423	18064456	18064531	84	32	38	0	0	1	0	20	0	0	95	49	1	1	1	1	0.951	NA	1	-0.016
SE	1018	ASAH1	ASAH1	chr8	-	18073255	18073276	18071299	18071390	18075540	18075587	0	2	0	61	34	21	0	0	0	27	70	49	1	1	0	0.04	0	NA	0	0.013
SE	1019	PCM1	PCM1	chr8	+	17924712	17924780	17922856	17923188	17935588	17935706	10	8	3	1	0	4	0	1	0	0	98	49	1	1	0.833	1	0.273	NA	1	-0.298
SE	1020	PCM1	PCM1	chr8	+	17963100	17963291	17962033	17962174	17964567	17964768	18	2	0	2	1	0	0	10	0	0	98	49	1	1	0.818	0.5	NA	NA	1	-0.341
SE	1021	PCM1	PCM1	chr8	+	17980590	17980755	17972328	17972687	17985446	17985619	4	1	1	2	0	0	0	1	0	0	98	49	1	1	0.5	1	1	NA	1	-0.167
SE	1027	ZDHHC2	ZDHHC2	chr8	+	17207959	17208092	17205654	17205775	17209931	17210058	8	3	9	0	0	2	0	2	0	0	98	49	1	1	1	1	0.692	NA	1	-0.103
SE	1030	CTSB	CTSB	chr8	-	11849045	11849164	11848066	11848152	11850865	11850980	21	9	3	0	0	0	0	32	0	2	98	49	1	1	1	1	1	NA	0.889	0.111
SE	1034	CTSB	CTSB	chr8	-	11864375	11864463	11853328	11853479	11868000	11868137	2	0	0	0	1	0	0	1	2	1	98	49	1	1	1	0	NA	0	0.333	0.334
SE	1041	TNKS	TNKS	chr8	+	9730889	9731035	9726640	9726720	9733278	9733444	4	2	0	0	0	0	0	1	0	1	98	49	1	1	1	1	NA	NA	0.333	0.667
SE	1051	DNAJB6	DNAJB6	chr7	+	157363160	157363270	157358546	157358637	157366501	157366561	26	17	6	0	1	0	5	5	0	0	98	49	1	1	1	0.895	1	1	1	-0.035
SE	1052	RBM33	RBM33	chr7	+	155706859	155707068	155700772	155700944	155711202	155711455	31	9	7	1	0	0	0	4	0	0	98	49	1	1	0.939	1	1	NA	1	-0.02
SE	1053	RBM33	RBM33	chr7	+	155739714	155740026	155738059	155738403	155741818	155742106	8	1	0	2	0	0	0	2	0	0	98	49	1	1	0.667	1	NA	NA	1	-0.166
SE	1054	KMT2C	KMT2C	chr7	-	152146598	152146735	152145152	152145295	152148032	152149152	47	11	8	0	0	0	0	11	0	1	98	49	1	1	1	1	1	NA	0.846	0.154
SE	1055	KMT2C	KMT2C	chr7	-	152182973	152183156	152180710	152182594	152185557	152185631	46	15	5	5	2	2	0	9	0	3	98	49	1	1	0.821	0.789	0.556	NA	0.6	0.122
SE	1056	KMT2C	KMT2C	chr7	-	152195906	152196011	152194439	152194568	152199278	152199459	44	14	2	1	0	0	0	8	0	0	98	49	1	1	0.957	1	1	NA	1	-0.014

SE	1057	KMT2C	KMT2C	chr7	-	152311797	152311946	152309965	152310075	152315137	152315338	99	35	7	1	0	0	0	12	0	0	98	49	1	1	0.98	1	1	NA		1	-0.007
SE	1061	NUB1	NUB1	chr7	+	151355767	151355950	151352811	151352882	151360140	151360247	11	9	4	0	0	0	0	3	1	0	98	49	1	1	1	1	1		0	1	0.5
SE	1062	NUB1	NUB1	chr7	+	151356127	151356222	151355767	151355950	151360140	151360247	32	9	3	0	3	0	0	3	0	0	98	49	1	1	1	0.6	1	NA		1	-0.133
SE	1071	ZNF862	ZNF862	chr7	+	149860382	149862494	149859421	149859526	149864108	149867479	1	0	0	2	0	0	0	1	0	0	98	49	1	1	0.2	NA	NA	NA		1	-0.8
SE	1078	TPK1	TPK1	chr7	-	144835559	144835618	144765879	144765951	144835966	144836053	3	0	0	1	0	0	0	4	0	0	98	49	1	1	0.6	NA	NA	NA		1	-0.4
SE	1094	MGAM	MGAM	chr7	+	142034261	142034379	142032824	142032909	142034669	142034841	154	87	31	2	0	0	2	32	0	0	98	49	1	1	0.975	1	1		1	1	-0.008
SE	1095	MGAM	MGAM	chr7	+	142036168	142036285	142034669	142034841	142036822	142036977	158	41	27	0	2	0	2	23	0	0	98	49	1	1	1	0.911	1		1	1	-0.03
SE	1096	MGAM	MGAM	chr7	+	142036168	142036285	142034669	142034841	142038530	142038615	110	29	10	1	0	0	2	18	0	0	98	49	1	1	0.982	1	1		1	1	-0.006
SE	1097	MGAM	MGAM	chr7	+	142036822	142036977	142034669	142034841	142038530	142038615	97	19	10	1	0	0	0	14	0	0	98	49	1	1	0.98	1	1	NA		1	-0.007
SE	1098	MGAM	MGAM	chr7	+	142036822	142036977	142036168	142036285	142038530	142038615	147	30	27	2	1	0	0	20	0	1	98	49	1	1	0.974	0.937	1	NA		0.909	0.061
SE	1099	MGAM	MGAM	chr7	+	142047784	142047873	142040721	142040846	142050234	142050284	176	46	39	2	0	0	2	16	0	0	98	49	1	1	0.978	1	1		1	1	-0.007
SE	1100	MGAM	MGAM	chr7	+	142047784	142047873	142040721	142040846	142050696	142050864	85	20	17	1	2	0	2	7	0	0	98	49	1	1	0.977	0.833	1		1	1	-0.063
SE	1101	MGAM	MGAM	chr7	+	142050234	142050284	142040721	142040846	142050696	142050864	124	20	11	1	2	0	0	9	0	0	98	49	1	1	0.984	0.833	1	NA		1	-0.061
SE	1102	MGAM	MGAM	chr7	+	142050234	142050284	142047784	142047873	142050696	142050864	214	46	33	1	0	0	0	18	0	0	98	49	1	1	0.991	1	1	NA		1	-0.003
SE	1106	BRWD3	BRWD3	chrX	-	80681996	80682094	80681340	80681499	80682464	80682628	9	10	5	1	0	0	0	6	0	0	98	49	1	1	0.818	1	1	NA		1	-0.061
SE	1110	SLC37A3	SLC37A3	chr7	-	140355667	140355764	140352061	140352146	140364407	140364491	12	7	4	1	0	0	0	2	0	0	98	49	1	1	0.857	1	1	NA		1	-0.048
SE	1111	SLC37A3	SLC37A3	chr7	-	140358639	140358785	140355667	140355764	140364407	140364491	24	13	2	1	3	0	0	12	0	0	98	49	1	1	0.923	0.684	1	NA		1	-0.131
SE	1112	SLC37A3	SLC37A3	chr7	-	140380281	140380390	140369589	140369682	140382437	140382596	14	6	0	3	0	0	1	1	0	0	98	49	1	1	0.7	1	NA		1	1	-0.15
SE	1113	PARP12	PARP12	chr7	-	140027275	140027406	140026196	140026348	140028612	140028688	15	14	5	1	0	0	1	16	0	0	98	49	1	1	0.882	1	1		1	1	-0.039
SE	1115	PARP12	PARP12	chr7	-	140057898	140058034	140056855	140057153	140062521	140063721	20	4	7	0	0	0	1	8	1	0	98	49	1	1	1	1	1		0.333	1	0.334
SE	1117	KIF13B	KIF13B	chr8	-	29186291	29186472	29181918	29182006	29188514	29188607	2	1	0	0	2	0	0	2	0	0	98	49	1	1	1	0.2	NA	NA		1	-0.4
SE	1132	LINC-PINT	LINC-PINT	chr7	-	130912109	130912152	130884316	130884396	130945719	130945835	12	17	6	11	1	0	0	1	0	0	92	49	1	1	0.367	0.901	1	NA		1	-0.244
SE	1133	LINC-PINT	LINC-PINT	chr7	-	130912109	130912152	130884316	130884396	130959868	130959970	7	10	0	1	0	0	0	1	0	0	92	49	1	1	0.789	1	NA	NA		1	-0.105
SE	1134	LINC-PINT	LINC-PINT	chr7	-	130959868	130959970	130944159	130945835	130967388	130967517	22	3	0	18	3	0	0	1	0	3	98	49	1	1	0.379	0.333	NA	NA		0.143	0.213
SE	1137	LINC-PINT	LINC-PINT	chr7	-	130967388	130967517	130959868	130959970	130984052	130984109	22	7	0	3	1	0	0	2	0	0	98	49	1	1	0.786	0.778	NA	NA		1	-0.218
SE	1140	LINC-PINT	LINC-PINT	chr7	-	131052424	131052555	130984052	130984109	131107719	131108803	56	24	8	1	0	0	2	11	0	0	98	49	1	1	0.966	1	1		1	1	-0.011
SE	1150	NRF1	NRF1	chr7	+	129710373	129710571	129709074	129709233	129711474	129711576	4	4	5	1	2	0	0	1	0	0	98	49	1	1	0.667	0.5	1	NA		1	-0.278
SE	1152	AHCYL2	AHCYL2	chr7	+	129400289	129400384	129397221	129397324	129403378	129403485	5	0	0	0	0	0	0	0	0	2	98	49	1	1	1	NA	NA	NA		0	1
SE	1154	TNPO3	TNPO3	chr7	-	128993806	128993914	128991998	128992090	128997388	128997535	12	5	10	0	0	0	0	0	0	2	98	49	1	1	1	1	1	NA		0	1
SE	1157	SND1	SND1	chr7	+	127707556	127707647	127704838	127704945	127721286	127721400	16	3	2	0	1	0	0	1	0	0	98	49	1	1	1	0.6	1	NA		1	-0.133
SE	1163	POT1	POT1	chr7	-	124848539	124848659	124846941	124846998	124851871	124851951	1	0	0	2	0	0	0	0	1	0	98	49	1	1	0.2	NA	NA		0	NA	0.2
SE	1170	ING3	ING3	chr7	+	120964741	120964838	120955558	120955624	120966625	120966697	3	3	9	2	0	0	0	0	0	2	98	49	1	1	0.429	1	1	NA		0	0.81
SE	1171	ING3	ING3	chr7	+	120973204	120973243	120970687	120970880	120974727	120975657	8	4	5	0	0	0	0	3	0	1	88	49	1	1	1	1	1	NA		0.626	0.374
SE	1174	CAPZA2	CAPZA2	chr7	+	116906262	116906342	116904176	116904383	116910232	116910311	96	49	37	0	1	1	0	23	0	3	98	49	1	1	1	0.961	0.949	NA		0.793	0.177
SE	1183	IFRD1	IFRD1	chr7	+	112456913	112457038	112456001	112456086	112458860	112459018	274	84	26	3	0	0	6	52	0	0	98	49	1	1	0.979	1	1		1	1	-0.007
SE	1184	IFRD1	IFRD1	chr7	+	112456913	112457038	112456001	112456086	112461865	112461916	216	63	15	0	2	0	6	38	0	0	98	49	1	1	1	0.94	1		1	1	-0.02
SE	1185	IFRD1	IFRD1	chr7	+	112458860	112459018	112456001	112456086	112461865	112461916	61	44	24	0	2	0	0	28	0	0	98	49	1	1	1	0.917	1	NA		1	-0.028
SE	1186	IFRD1	IFRD1	chr7	+	112458860	112459018	112456913	112457038	112461865	112461916	116	67	35	0	2	0	0	42	0	0	98	49	1	1	1	0.944	1	NA		1	-0.019
SE	1193	PNPLA8	PNPLA8	chr7	-	108521475	108521521	108514435	108515574	108526028	108526318	17	15	0	1	0	0	0	3	0	0	95	49	1	1	0.898	1	NA	NA		1	-0.051
SE	1207	NAMPT	NAMPT	chr7	-	106261587	106261707	106253016	106253151	106263391	106263617	12	6	0	0	1	0	0	4	0	0	98	49	1	1	1	0.75	NA	NA		1	-0.125
SE	1208	NAMPT	NAMPT	chr7	-	106261587	106261707	106254363	106254504	106268463	106268600	3	3	0	1	6	0	0	0	0	1	98	49	1	1	0.6	0.2	NA	NA		0	0.4
SE	1209	NAMPT	NAMPT	chr7	-	106263391	106263617	106253016	106253151	106272529	106272658	0	1	0	0	0	1	0	1	0	0	98	49	1	1	NA	1	0	NA		1	-0.5
SE	1211	NAMPT	NAMPT	chr7	-	106268463	106268600	106263391	106263617	106269153	106269312	678	328	111	0	1	0	9	124	0	0	98	49	1	1	1	0.994	1		1	1	-0.002
SE	1212	NAMPT	NAMPT	chr7	-	106269153	106269312	106263391	106263617	106272529	106272658	0	1	0	0	0	0	0	0	0	1	98	49	1	1	NA	1	NA	NA		0	1
SE	1214	NAMPT	NAMPT	chr7	-	106277022	106277179	106272529	106272658	106284827	106285192	592	331	90	0	3	0	12	69	0	0	98	49	1	1	1	0.982	1		1	1	-0.006
SE	1216	SRPK2	SRPK2	chr7	-	105133003	105133104	105132790	105132898	105142007	105142490	40	17	4	1	0	0	0	9	0	0	98	49	1	1	0.952	1	1	NA		1	-0.016
SE	1217	SRPK2	SRPK2	chr7	-	105268805	105268869	105203627	105203785	105388647	105388702	4	4	1	10	2	5	0	0	0	1	98	49	1	1	0.167	0.5	0.091	NA		0	0.253
SE	1223	PMPCB	PMPCB	chr7	+	103307595	103307708	103304410	103304490	103308951	103309095	3	5	0	1	2	0	0	2	0	0	98	49	1	1	0.6	0.556	NA	NA		1	-0.422
SE	1236	ORAI2	ORAI2	chr7	+	102436201	102436333	102433529	102433661	102438943	102439181	20	10	0	1	0	0	0	4	0	0	98	49	1	1	0.909	1	NA	NA		1	-0.045
SE	1237	ORAI2	ORAI2	chr7	+	102436224	102436333	102433529	102433661	102438943	102439181	20	14	0	1	0	0	0	4	0	0	98	49	1	1	0.909	1	NA	NA		1	-0.045
SE	1238	ORAI2	ORAI2	chr7	+	102438943	102439181	102436201	102436333	102446512	102456821	30	16	5	1	0	0	2	6	0	0	98	49	1	1	0.937	1	1		1	1	-0.021
SE	1247	LRCH4	LRCH4	chr7	-	100577272	100577389	100576901	100577005	100577496	100577558	15	9	1	2	0	0	0	1	0	0	98	49	1	1	0.789	1	1	NA		1	-0.07
SE	1250	STAG3L5P-PVRIG2P-PILRB	STAG3L5P-PVRIG2P-PILRB	chr7	+	100353372	100353483	100353209	100353270	100355142	100355240	4	1	0	0	2	0	1	1	0	0	98	49	1	1	1	0.2	NA		1	1	-0.4
SE	1251	STAG3L5P-PVRIG2P-PILRB	STAG3L5P-PVRIG2P-PILRB	chr7	+	100353372	100354012	100353209	100353270	100355142	100355240	8	2	0	0	2	0	2	1	0	0	98	49	1	1	1	0.333	NA		1	1	-0.334
SE	1268	TECPR1	TECPR1	chr7	-	98241069	98241244	98240850	98240951	98243466	98243592	10	3	0	0	0	0	0	2	0	1	98	49	1	1	1	1	NA	NA		0.5	0.5
SE	1269	TECPR1	TECPR1	chr7	-	98244884	98245067	98244570	98244693	98245921	98246165	11	12	0	2	0	0	0	1	0	0	98	49	1	1	0.733	1	NA	NA		1	-0.133
SE	1283	SAMD9L	SAMD9L	chr7	-	93145908	93146040	93145384	93145532	93148193	93148388	8	1	3	1	1	0	0	1	0	0	98	49	1	1	0.8	0.333	1	NA		1	-0.289
SE	1292	AKAP9	AKAP9	chr7	+	91992157	91992211	91980288	91980333	91992884	91993055	8	0	0	6	0	3	0	3	0	0	98	49	1	1	0.4	NA	0	NA		1	-0.8
SE	1293	AKAP9	AKAP9	chr7	+	92000847	92003235	91995602	91995800	92012428	92012642	16	7	0	0	1	0	3	11	0	0	98	49	1	1	1	0.778	NA		1	1	-0.111
SE	1294	AKAP9	AKAP9	chr7	+	92107292	92107422	92105677	92105763	92108493	92108633	3	2	3	1	0	0	1	1	0	0	98	49	1	1	0.6	1	1		1	1	-0.133
SE	1300	STEAP4	STEAP4	chr7	-	88282640	88283168	88280914	88281079	88283813	88284271	130	54	39	0	2	0	2	17	0	0	98	49	1	1	1	0.931	1		1	1	-0.023
SE	1301	STEAP4	STEAP4	chr7	-	88290915	88291014	88283813	88284271	88306791	88306913	12	7	2	5	8	0	0	4	0	0	98	49	1	1	0.545	0.304	1	NA		1	-0.384
SE	1305	DMTF1	DMTF1	chr7	+	87164933	87165050	87163494	87163617	87166482	87166605	36	14	4	3	2	0	0	2	0	0	98	49	1	1	0.857	0.778	1	NA		1	-0.122
SE	1306	DMTF1	DMTF1	chr7	+	87166482	87166605	87164933	87165050	87170994	87171089	42	16	0	3	0	0	0	1	0	0	98	49	1	1	0.875	1	NA	NA		1	-0.062
SE	1307	DMTF1	DMTF1	chr7	+	87194683	87194828	87193724	87194102	87195030	87196332	13	3	0	4	1	0	0	1	0	2	98	49	1	1	0.619	0.6	NA	NA		0.2	0.409
SE	1314	PTPN12	PTPN12	chr7	+	77585542	77585581	77583554	77583650	77592184	77592256	38	19	22	0	0	0	1	9	0	1	88	49	1	1	1	1	1		1	0.834	0.083
SE	1315	GSAP	GSAP	chr7	-	77311840	77311940	77310750	77311449	77312100	77312202	6	14	0	0	0	0	1	2	0	1	98	49	1	1	1	1	NA		1	0.5	0.25
SE	1316	GSAP	GSAP	chr7	-	77349350	77349404	77330238	77330367	77352943	77353026	5	0	0	1	0	0	0	2	0	0	98	49	1	1	0.714	NA	NA	NA		1	-0.286
SE	1324	DTX2	DTX2	chr7	+	76480420	76480777	76461654	76461836	76482507	76483147	4	1	0	1	0	0	0	6	0	0	98	49	1	1	0.667	1	NA	NA		1	-0.166
SE	1329	TMEM120A	TMEM120A	chr7	-	75987537	75987602	75987359	75987428	75987717	75987810	2	0	1	2	1	0	0	1	0	0	98	49	1	1	0.333	0	1	NA		1	-0.556
SE	1330	RHBDD2	RHBDD2	chr7	+	75881364	75881486	75878998	75879260	75881828	75882236	2	0	1	2	0	1	0	2	1	0	98	49	1	1	0.333	NA	0.333		0	1	-0.167
SE	1334	GTF2I	GTF2I	chr7	+	74718878	74718941	74716893	74716950	74728785	74728896	1	1	0	3	0	0	0	1	0	1	98	49	1	1	0.143	1	NA	NA		0.333	0.238
SE	1337	LAT2	LAT2	chr7	+	74214821	74215010	74209756	74210088	74215946	74216069	22	12	5	0	2	0	1	2	0	0	98	49	1	1	1	0.75	1		1	1	-0.083
SE	1338	LAT2	LAT2	chr7	+	74216824	74216864	74215946	74216069	74219743	74219787	42	29	18	1	0	0	0	13	0	0	89	49	1	1	0.959	1	1	NA		1	-0.014
SE	1339	LAT2	LAT2	chr7	+	74221636	74221692	74220703	74220734	74223723	74223783	55	33	20	0	0	1	1	20	0	0	98	49	1	1	1	1	0.909		1	1	-0.03
SE	1340	LAT2	LAT2	chr7	+	74224638	74224760	74224017	74224197	74228943	74229834	39	22	20	0	0	0	0	7	0	1	98	49	1	1	1	1	1	NA		0.778	0.222
SE	1365	RABGEF1	RABGEF1	chr7	+	66783674	66783841	66775226	66775393	66795510	66795592	9	3	5	0	2	1	0	9	0	0	98	49	1	1	1	0.429	0.714	NA		1	-0.286
SE	1366	RABGEF1	RABGEF1	chr7	+	66795510	66795592	66775226	66775393	66797373	66797506	6	9	14	0	0	0	0	4	0	1	98	49	1	1	1	1	1	NA		0.667	0.333
SE	1367	TPST1	TPST1	chr7	+	66240324	66241270	66205271	66205522	66286510	66286709	19	5	4	0	0	1	0	7	0	0	98	49	1	1	1	1	0.667	NA		1	-0.111
SE	1368	ASL	ASL	chr7	+	66086743	66086821	66086584	66086662	66087333	66087386	2	0	0	0	0	0	0	2	1	0	98	49	1	1	1	NA	NA		0	1	0.5
SE	1385	CHCHD2	CHCHD2	chr7	-	56104225	56104475	56102866	56103011	56106363	56106494	17	1	9	1	0	0	0	9	0	0	98	49	1	1	0.895	1	1	NA		1	-0.035
SE	1391	GRB10	GRB10	chr7	-	50755886	50756056	50703820	50703908	50793223	50793462	0	0	2	1	0	0	0	1	0	0	98	49	1	1	0	NA	1	NA		1	-0.5
SE	1401	IKZF1	IKZF1	chr7	+	50368005	50368065	50327637	50327757	50382539	50382707	10	0	0	12	1	3	0	1	0	2	98	49	1	1	0.294	0	0	NA		0.2	-0.102
SE	1402	IKZF1	IKZF1	chr7	+	50376532	50376793	50327637	50327757	50382539	50382707	10	9	9	12	1	3	0	1	0	2	98	49	1	1	0.294	0.818	0.6	NA		0.2	0.371
SE	1414	IKZF1	IKZF1	chr7	+	50391728	50391833	50387344	50387470	50399917	50405100	7	2	7	1	0	0	0	6	0	0	98	49	1	1	0.778	1	1	NA		1	-0.074
SE	1417	IKZF1	IKZF1	chr7	+	50391728	50391863	50387344	50387470	50399917	50405100	8	4	5	1	0	0	0	3	0	0	98	49	1	1	0.8	1	1	NA		1	-0.067
SE	1418	NCBP1	NCBP1	chr9	+	97662041	97662144	97660945	97661068	97662953	97663047	6	3	0	0	0	0	1	1	0	2	98	49	1	1	1	1	NA		1	0.2	0.4
SE	1419	ABCA13	ABCA13	chr7	+	48372167	48372497	48367793	48367908	48374346	48374416	0	0	0	0	1	0	1	0	0	0	98	49	1	1	NA	0	NA		1	NA	-1
SE	1420	UPP1	UPP1	chr7	+	48099669	48099787	48094762	48094827	48101823	48101982	2	3	1	1	0	0	0	1	0	0	98	49	1	1	0.5	1	1	NA		1	-0.167
SE	1421	UPP1	UPP1	chr7	+	48101823	48101870	48094762	48094827	48106872	48107082	7	0	0	1	0	0	0	2	0	0	96	49	1	1	0.781	NA	NA	NA		1	-0.219
SE	1425	LRRC6	LRRC6	chr8	-	132632739	132632963	132625271	132625454	132637934	132638107	7	2	0	0	1	0	0	1	0	1	98	49	1	1	1	0.5	NA	NA		0.333	0.417
SE	1429	FBXL13	FBXL13	chr7	-	102931880	102931933	102926273	102926374	102963532	102963665	42	16	3	2	0	0	0	8	0	0	98	49	1	1	0.913	1	1	NA		1	-0.029
SE	1430	FBXL13	FBXL13	chr7	-	102963532	102963665	102931880	102931933	102968021	102968117	43	6	1	7	7	0	0	3	0	0	98	49	1	1	0.754	0.3	1	NA		1	-0.315
SE	1433	TBRG4	TBRG4	chr7	-	45104098	45104256	45103332	45103443	45104537	45104709	3	0	0	0	0	0	0	0	0	1	98	49	1	1	1	NA	NA	NA		0	1
SE	1435	CCM2	CCM2	chr7	+	45038252	45038426	45000187	45000363	45063917	45064001	11	2	9	0	1	0	1	0	0	0	98	49	1	1	1	0.5	1		1	NA	-0.167
SE	1436	CCM2	CCM2	chr7	+	45038252	45038426	45027633	45027797	45063917	45064001	12	2	11	2	4	0	1	0	0	0	98	49	1	1	0.75	0.2	1		1	NA	-0.35
SE	1437	CCM2	CCM2	chr7	+	45068442	45068579	45064462	45064646	45069825	45069961	21	13	11	0	3	3	0	7	0	0	98	49	1	1	1	0.684	0.647	NA		1	-0.223
SE	1438	CCM2	CCM2	chr7	+	45068442	45068579	45064462	45064646	45072725	45072783	9	8	6	0	0	2	0	4	0	0	98	49	1	1	1	1	0.6	NA		1	-0.133
SE	1439	CCM2	CCM2	chr7	+	45069825	45069961	45064462	45064646	45072725	45072783	11	11	10	0	0	2	0	1	0	0	98	49	1	1	1	1	0.714	NA		1	-0.095
SE	1440	CCM2	CCM2	chr7	+	45069825	45069961	45068442	45068579	45072725	45072783	24	13	12	1	0	0	0	4	0	0	98	49	1	1	0.923	1	1	NA		1	-0.026
SE	1441	SNHG15	SNHG15	chr7	-	44986020	44986097	44984356	44984426	44986524	44986660	4	1	0	1	0	0	0	1	0	0	98	49	1	1	0.667	1	NA	NA		1	-0.166
SE	1445	PPIA	PPIA	chr7	+	44798747	44798904	44796635	44796793	44799246	44799277	2	0	0	2	0	0	0	1	0	0	98	49	1	1	0.333	NA	NA	NA		1	-0.667
SE	1462	VPS41	VPS41	chr7	-	38817816	38817882	38796744	38796864	38821202	38821265	22	6	2	1	0	0	0	4	0	0	98	49	1	1	0.917	1	1	NA		1	-0.028
SE	1470	NT5C3A	NT5C3A	chr7	-	33024038	33024108	33022052	33022099	33026816	33026915	14	3	3	0	1	0	0	6	0	0	98	49	1	1	1	0.6	1	NA		1	-0.133
SE	1471	NT5C3A	NT5C3A	chr7	-	33029628	33029714	33026816	33026915	33035933	33035988	2	0	0	12	3	0	0	0	0	1	98	49	1	1	0.077	0	NA	NA		0	0.038
SE	1474	KBTBD2	KBTBD2	chr7	-	32879434	32879942	32874991	32875157	32891535	32891856	28	11	2	2	2	0	0	6	0	0	98	49	1	1	0.875	0.733	1	NA		1	-0.131
SE	1479	LOC401320	LOC401320	chr7	-	30552099	30552179	30548356	30550781	30561465	30562128	7	2	0	3	1	0	1	4	0	0	98	49	1	1	0.538	0.5	NA		1	1	-0.481
SE	1496	CPVL	CPVL	chr7	-	29064060	29064234	29030576	29030759	29066022	29066121	29	0	0	1	0	0	0	9	0	0	98	49	1	1	0.935	NA	NA	NA		1	-0.065
SE	1500	SKAP2	SKAP2	chr7	-	26684735	26684848	26670090	26670192	26690284	26690362	60	30	13	0	2	0	0	3	0	0	98	49	1	1	1	0.882	1	NA		1	-0.039
SE	1501	HNRNPA2B1	HNRNPA2B1	chr7	-	26193574	26193694	26193250	26193373	26195846	26195909	30	7	1	5	1	0	0	1	0	0	98	49	1	1	0.75	0.778	1	NA		1	-0.157
SE	1509	CCDC126	CCDC126	chr7	+	23597966	23598051	23597378	23597605	23611170	23611553	2	3	4	2	5	0	0	0	0	1	98	49	1	1	0.333	0.231	1	NA		0	0.521
SE	1510	FAM126A	FAM126A	chr7	-	22978268	22978448	22977340	22977421	22983943	22984045	9	5	6	1	0	0	0	2	0	0	98	49	1	1	0.818	1	1	NA		1	-0.061
SE	1514	SNX13	SNX13	chr7	-	17845594	17845694	17839806	17840000	17868406	17868490	33	12	0	0	0	1	0	2	0	0	98	49	1	1	1	1	0	NA		1	-0.333
SE	1515	SNX13	SNX13	chr7	-	17897333	17897446	17893331	17893434	17940283	17940508	13	2	3	1	0	0	0	2	0	0	98	49	1	1	0.867	1	1	NA		1	-0.044
SE	1521	PHF14	PHF14	chr7	+	11013746	11013906	10990702	10990847	11022867	11022979	1	1	0	1	0	0	0	2	0	0	98	49	1	1	0.333	1	NA	NA		1	-0.334
SE	1524	TDRD7	TDRD7	chr9	+	97470557	97470669	97464841	97465028	97472292	97472495	4	0	1	2	0	0	0	3	0	1	98	49	1	1	0.5	NA	1	NA		0.6	0.15
SE	1525	TDRD7	TDRD7	chr9	+	97475382	97475469	97473491	97473626	97478438	97478573	14	3	0	0	2	0	0	3	0	0	98	49	1	1	1	0.429	NA	NA		1	-0.285
SE	1527	ZDHHC4	ZDHHC4	chr7	+	6580554	6580678	6578500	6578720	6581606	6581680	4	0	2	1	0	0	1	0	0	0	98	49	1	1	0.667	NA	1		1	NA	-0.166
SE	1532	RNF216	RNF216	chr7	-	5721032	5721172	5716715	5716766	5725323	5725438	19	7	0	0	1	0	0	2	0	0	98	49	1	1	1	0.778	NA	NA		1	-0.111
SE	1544	BRAT1	BRAT1	chr7	-	2544908	2545056	2543589	2543962	2547323	2547478	5	8	1	1	0	0	0	2	0	2	98	49	1	1	0.714	1	1	NA		0.333	0.572
SE	1551	CCDC22	CCDC22	chrX	+	49249166	49249262	49248816	49248924	49249508	49249568	1	0	0	0	0	0	0	5	0	1	98	49	1	1	1	NA	NA	NA		0.714	0.286
SE	1561	ERMARD	ERMARD	chr6	+	169766637	169766667	169762428	169762531	169768102	169768171	5	1	1	0	0	0	0	1	0	1	79	49	1	1	1	1	1	NA		0.383	0.617
SE	1578	IGF2R	IGF2R	chr6	+	160027184	160027314	160024571	160024704	160029549	160029655	445	161	69	3	0	0	8	60	0	0	98	49	1	1	0.987	1	1		1	1	-0.004
SE	1580	IGF2R	IGF2R	chr6	+	160034418	160034522	160032941	160033107	160040559	160040724	215	89	53	2	1	0	2	31	0	0	98	49	1	1	0.982	0.978	1		1	1	-0.013
SE	1583	IGF2R	IGF2R	chr6	+	160060546	160060717	160058905	160059098	160061502	160061646	359	123	60	0	1	0	6	48	0	0	98	49	1	1	1	0.984	1		1	1	-0.005
SE	1584	IGF2R	IGF2R	chr6	+	160061502	160061646	160060546	160060717	160062531	160062619	204	65	37	1	0	0	5	19	0	0	98	49	1	1	0.99	1	1		1	1	-0.003
SE	1586	IGF2R	IGF2R	chr6	+	160063414	160063630	160062531	160062619	160064400	160064531	257	90	50	1	0	0	2	31	0	0	98	49	1	1	0.992	1	1		1	1	-0.003
SE	1588	IGF2R	IGF2R	chr6	+	160072764	160072884	160071909	160072036	160073212	160073469	250	74	63	1	0	0	1	16	0	1	98	49	1	1	0.992	1	1		1	0.889	0.053
SE	1590	IGF2R	IGF2R	chr6	+	160084994	160085131	160083949	160084184	160089106	160089253	119	27	21	1	0	0	4	13	0	1	98	49	1	1	0.983	1	1		1	0.867	0.061
SE	1591	IGF2R	IGF2R	chr6	+	160088032	160088147	160084994	160085131	160089106	160089253	111	35	13	1	0	0	0	17	0	0	98	49	1	1	0.982	1	1	NA		1	-0.006
SE	1592	TCP1	TCP1	chr6	-	159780934	159781110	159780442	159780566	159783940	159784067	13	7	3	3	0	0	0	1	0	0	98	49	1	1	0.684	1	1	NA		1	-0.105
SE	1594	SOD2	SOD2	chr6	-	159682473	159682638	159679115	159679298	159684853	159685033	606	399	313	2	2	0	4	86	0	0	98	49	1	1	0.993	0.99	1		1	1	-0.006
SE	1595	SOD2	SOD2	chr6	-	159684853	159685033	159681722	159682638	159688125	159688242	869	535	430	0	1	0	1	107	0	0	98	49	1	1	1	0.996	1		1	1	-0.001
SE	1602	TAGAP	TAGAP	chr6	-	159043977	159044031	159043588	159043655	159044119	159044204	179	63	27	1	0	0	1	15	0	0	98	49	1	1	0.989	1	1		1	1	-0.004
SE	1606	TMEM181	TMEM181	chr6	+	158583953	158584044	158580939	158580995	158585303	158585425	7	5	3	0	0	2	0	4	0	0	98	49	1	1	1	1	0.429	NA		1	-0.19
SE	1610	ARID1B	ARID1B	chr6	+	156871613	156871652	156829226	156829421	156901375	156901525	0	1	0	12	0	0	0	0	1	1	88	49	1	1	0	1	NA		0	0	0.5
SE	1611	ARID1B	ARID1B	chr6	+	157110471	157110561	157084661	157084905	157133027	157133207	0	2	0	0	1	0	0	1	0	0	98	49	1	1	NA	0.5	NA	NA		1	-0.5
SE	1614	ARMT1	ARMT1	chr6	+	151454547	151454653	151452257	151452586	151458327	151458572	2	1	0	0	3	0	0	2	0	0	98	49	1	1	1	0.143	NA	NA		1	-0.428
SE	1616	CSGALNACT1	CSGALNACT1	chr8	-	19457529	19457816	19439829	19439931	19458425	19458642	1	0	0	1	1	0	0	3	1	5	98	49	1	1	0.333	0	NA		0	0.231	0.051
SE	1617	CSGALNACT1	CSGALNACT1	chr8	-	19585165	19585317	19505200	19506130	19601770	19601865	3	0	0	8	2	0	0	0	0	2	98	49	1	1	0.158	0	NA	NA		0	0.079
SE	1618	CSGALNACT1	CSGALNACT1	chr8	-	19591159	19591278	19505200	19506130	19601770	19601865	1	0	0	8	2	0	0	1	0	2	98	49	1	1	0.059	0	NA	NA		0.2	-0.171
SE	1623	GLIPR2	GLIPR2	chr9	+	36148546	36148650	36147785	36147894	36150871	36150949	48	16	21	1	0	0	2	8	0	0	98	49	1	1	0.96	1	1		1	1	-0.013
SE	1629	NUP43	NUP43	chr6	-	149743637	149743715	149742389	149742570	149745939	149746062	3	0	0	0	1	0	0	2	0	0	98	49	1	1	1	0	NA	NA		1	-0.5
SE	1632	STXBP5	STXBP5	chr6	+	147314263	147314331	147313883	147314031	147314595	147314636	51	28	17	3	0	0	0	9	0	0	98	49	1	1	0.895	1	1	NA		1	-0.035
SE	1633	STXBP5	STXBP5	chr6	+	147314595	147314636	147313883	147314031	147315514	147315735	26	3	1	0	1	0	0	4	0	0	90	49	1	1	1	0.62	1	NA		1	-0.127
SE	1634	STXBP5	STXBP5	chr6	+	147339178	147339238	147334156	147334222	147353322	147353373	15	3	0	6	0	0	0	4	0	0	98	49	1	1	0.556	1	NA	NA		1	-0.222
SE	1635	STXBP5	STXBP5	chr6	+	147339336	147339384	147339178	147339238	147353322	147353373	7	10	6	1	0	0	0	1	0	0	97	49	1	1	0.78	1	1	NA		1	-0.073
SE	1639	UTRN	UTRN	chr6	+	144437564	144437746	144435934	144436138	144438744	144438895	57	20	10	3	0	0	0	19	0	1	98	49	1	1	0.905	1	1	NA		0.905	0.063
SE	1640	UTRN	UTRN	chr6	+	144827347	144827386	144820881	144821018	144827610	144827676	10	0	0	13	3	0	0	2	0	2	88	49	1	1	0.3	0	NA	NA		0.358	-0.208
SE	1641	UTRN	UTRN	chr6	+	144839172	144839284	144836300	144836541	144840739	144840832	10	2	4	3	0	0	0	9	0	0	98	49	1	1	0.625	1	1	NA		1	-0.125
SE	1643	PHACTR2	PHACTR2	chr6	+	143765260	143765798	143753753	143753912	143772257	143772457	10	0	0	0	0	0	1	4	0	1	98	49	1	1	1	NA	NA		1	0.667	0.166
SE	1702	TNFAIP3	TNFAIP3	chr6	+	137880070	137880252	137878431	137879351	137881034	137883314	26	16	1	1	0	0	0	12	0	0	98	49	1	1	0.929	1	1	NA		1	-0.024
SE	1705	MAP3K5	MAP3K5	chr6	-	136601801	136601979	136601021	136601042	136605208	136605366	38	6	4	1	0	0	0	2	0	0	98	49	1	1	0.95	1	1	NA		1	-0.017
SE	1706	MAP3K5	MAP3K5	chr6	-	136622847	136622981	136614158	136614286	136637324	136637406	53	13	17	2	2	0	0	8	0	0	98	49	1	1	0.93	0.765	1	NA		1	-0.102
SE	1707	MAP3K5	MAP3K5	chr6	-	136650983	136651091	136642519	136642569	136656306	136656460	74	31	17	2	0	0	0	7	0	0	98	49	1	1	0.949	1	1	NA		1	-0.017

SE	1708	MAP3K5	MAP3K5	chr6	-	136720449	136720589	136705109	136705133	136791709	136792518	43	16	20	1	0	0	1	12	0	1	98	49	1	1	0.956	1	1	1	0.857	0.057
SE	1715	SGK1	SGK1	chr6	-	134171636	134171732	134171022	134171178	134172192	134172316	109	54	11	1	0	0	2	13	0	0	98	49	1	1	0.982	1	1	1	1	-0.006
SE	1716	SGK1	SGK1	chr6	-	134172192	134172316	134172661	134171732	134172661	134172774	100	69	18	0	1	1	0	15	0	0	98	49	1	1	1	0.972	0.9	NA	1	-0.043
SE	1717	SGK1	SGK1	chr6	-	134173022	134173154	134172661	134172774	134173273	134173357	177	96	37	0	1	0	3	25	0	0	98	49	1	1	1	0.98	1	1	1	-0.007
SE	1720	VNN2	VNN2	chr6	-	132751144	132751278	132749694	132749865	132755842	132756035	125	82	68	2	1	0	0	8	0	0	98	49	1	1	0.969	0.976	1	NA	1	-0.018
SE	1721	VNN2	VNN2	chr6	-	132751144	132751518	132749694	132749865	132755842	132756035	130	94	71	2	1	0	0	8	0	0	98	49	1	1	0.97	0.979	1	NA	1	-0.017
SE	1723	VNN2	VNN2	chr6	-	132752460	132752749	132751144	132751518	132755842	132756035	330	178	186	5	12	3	0	32	0	0	98	49	1	1	0.971	0.881	0.969	NA	1	-0.06
SE	1724	VNN2	VNN2	chr6	-	132752672	132752749	132751144	132751518	132753417	132753491	8	8	4	4	0	2	0	3	0	3	98	49	1	1	0.5	1	0.5	NA	0.333	0.334
SE	1725	VNN2	VNN2	chr6	-	132752672	132752749	132751144	132751518	132755842	132756035	115	82	84	5	12	3	0	10	0	0	98	49	1	1	0.92	0.774	0.933	NA	1	-0.124
SE	1726	VNN2	VNN2	chr6	-	132753417	132753491	132751144	132751518	132755842	132756035	34	5	4	5	12	3	0	3	0	0	98	49	1	1	0.773	0.172	0.4	NA	1	-0.552
SE	1727	VNN2	VNN2	chr6	-	132753417	132753491	132752672	132752749	132755842	132756035	37	12	6	114	81	84	0	3	0	10	98	49	1	1	0.14	0.069	0.034	NA	0.13	-0.049
SE	1729	VNN2	VNN2	chr6	-	132755842	132756035	132751144	132751518	132757415	132757546	302	183	114	1	0	0	4	21	0	0	98	49	1	1	0.993	1	1	1	1	-0.002
SE	1730	VNN3	VNN3	chr6	-	132724605	132724979	132723733	132723907	132734252	132734383	38	17	5	1	0	0	0	5	0	0	98	49	1	1	0.95	1	1	NA	1	-0.017
SE	1732	VNN3	VNN3	chr6	-	132726721	132727012	132724605	132724979	132734252	132734383	39	26	1	7	6	0	0	9	0	0	98	49	1	1	0.736	0.684	1	NA	1	-0.193
SE	1734	VNN3	VNN3	chr6	-	132728740	132728933	132726721	132727012	132734252	132734383	70	34	7	1	0	0	0	6	0	0	98	49	1	1	0.972	1	1	NA	1	-0.009
SE	1736	VNN3	VNN3	chr6	-	132728740	132728979	132726721	132727012	132734252	132734383	63	27	9	1	0	0	0	3	0	0	98	49	1	1	0.969	1	1	NA	1	-0.01
SE	1756	ECHDC1	ECHDC1	chr6	-	127316449	127316502	127314815	127314896	127327001	127327144	8	5	0	1	0	0	0	1	0	0	98	49	1	1	0.8	1	NA	NA	1	-0.1
SE	1761	RNF146	RNF146	chr6	+	127286049	127286177	127280230	127280340	127286615	127288560	1	0	1	0	0	6	3	1	0	0	98	49	1	1	1	NA	0.077	1	1	-0.462
SE	1762	HDDC2	HDDC2	chr6	-	125298713	125298816	125292840	125292909	125300537	125300659	2	0	0	1	0	0	0	1	0	0	98	49	1	1	0.5	NA	NA	NA	1	-0.5
SE	1773	MAN1A1	MAN1A1	chr6	-	119204758	119204882	119201253	119201347	119290682	119290763	17	8	2	0	0	0	0	0	0	2	98	49	1	1	1	1	1	NA	0	1
SE	1774	MAN1A1	MAN1A1	chr6	-	119248259	119248354	119204758	119204882	119290682	119290763	20	6	2	0	1	0	0	11	0	0	98	49	1	1	1	0.75	1	NA	1	-0.083
SE	1777	GOPC	GOPC	chr6	-	117570859	117570955	117569571	117569736	117573466	117573632	5	1	0	1	0	0	0	1	0	0	98	49	1	1	0.714	1	NA	NA	1	-0.143
SE	1780	KPNA5	KPNA5	chr6	+	116692292	116692392	116692054	116692156	116698703	116698798	5	6	0	3	0	0	0	2	0	0	98	49	1	1	0.455	1	NA	NA	1	-0.272
SE	1786	HDAC2	HDAC2	chr6	-	113953276	113953418	113949167	113949260	113956012	113956151	2	2	0	1	0	0	0	2	0	0	98	49	1	1	0.5	1	NA	NA	1	-0.25
SE	1791	FGFR1OP	FGFR1OP	chr6	+	167026546	167026615	167024781	167024894	167032602	167032642	14	4	0	0	1	0	0	1	0	0	98	49	1	1	1	0.667	NA	NA	1	-0.166
SE	1794	AMD1	AMD1	chr6	+	110887504	110887591	110874783	110875215	110888856	110888983	55	14	14	0	1	0	0	4	0	0	98	49	1	1	1	0.875	1	NA	1	-0.042
SE	1802	CD164	CD164	chr6	-	109370410	109370467	109366513	109369017	109376073	109376112	45	20	3	3	2	0	0	18	0	1	98	49	1	1	0.882	0.833	1	NA	0.9	0.005
SE	1808	SNX3	SNX3	chr6	-	108214497	108214622	108211216	108212254	108222949	108223045	21	20	18	1	0	1	0	5	0	0	98	49	1	1	0.913	1	0.9	NA	1	-0.062
SE	1810	SEC63	SEC63	chr6	-	107897648	107897731	107893837	107893897	107901369	107901517	6	7	1	1	0	0	0	2	0	0	98	49	1	1	0.75	1	1	NA	1	-0.083
SE	1818	ASCC3	ASCC3	chr6	-	100509931	100510107	100508194	100509533	100512708	100512918	1	0	0	0	0	0	0	0	0	1	98	49	1	1	1	NA	NA	NA	0	1
SE	1825	MAP3K7	MAP3K7	chr6	-	90544551	90544632	90536336	90536401	90547257	90547387	1	0	0	8	4	0	1	0	0	0	98	49	1	1	0.059	0	NA	1	NA	-0.971
SE	1826	UBE2J1	UBE2J1	chr6	-	89333085	89333205	89326624	89329957	89335301	89335431	58	17	8	1	0	0	2	6	0	0	98	49	1	1	0.967	1	1	1	1	-0.011
SE	1828	PM20D2	PM20D2	chr6	+	89154747	89154902	89153042	89153185	89158324	89158460	1	2	0	1	0	0	0	1	0	0	98	49	1	1	0.333	1	NA	NA	1	-0.334
SE	1832	RARS2	RARS2	chr6	-	87548590	87548646	87545615	87545699	87555407	87555505	4	10	2	1	0	0	0	2	0	0	98	49	1	1	0.667	1	1	NA	1	-0.111
SE	1836	SYNCRIP	SYNCRIP	chr6	-	85641291	85641451	85640445	85640564	85642796	85642890	3	2	0	2	0	0	0	1	0	0	98	49	1	1	0.429	1	NA	NA	1	-0.285
SE	1837	SNX14	SNX14	chr6	-	85528261	85528362	85526125	85526237	85530191	85530275	21	17	6	1	0	0	0	8	0	0	98	49	1	1	0.913	1	1	NA	1	-0.029
SE	1838	SNX14	SNX14	chr6	-	85538837	85538864	85536791	85536924	85541984	85542043	2	0	0	6	4	1	0	0	0	2	76	49	1	1	0.177	0	0	NA	0	0.059
SE	1839	SNX14	SNX14	chr6	-	85557975	85558060	85549722	85549879	85565331	85565419	13	6	0	4	0	0	0	4	0	0	98	49	1	1	0.619	1	NA	NA	1	-0.191
SE	1840	SNX14	SNX14	chr6	-	85565331	85565419	85557975	85558060	85567533	85567577	16	5	3	2	0	0	0	5	0	0	98	49	1	1	0.8	1	1	NA	1	-0.067
SE	1841	SNX14	SNX14	chr6	-	85565331	85565419	85557975	85558060	85572136	85572215	13	6	0	2	0	0	0	2	0	0	98	49	1	1	0.765	1	NA	NA	1	-0.117
SE	1843	SNX14	SNX14	chr6	-	85567533	85567577	85565331	85565419	85572136	85572215	8	1	3	2	2	0	0	2	0	0	93	49	1	1	0.678	0.209	1	NA	1	-0.371
SE	1851	SYTL3	SYTL3	chr6	+	158663204	158663378	158662749	158662812	158665394	158665613	9	2	1	1	1	0	1	1	0	1	98	49	1	1	0.818	0.5	1	1	0.333	0.106
SE	1852	HMGN3	HMGN3	chr6	-	79202062	79202155	79201244	79201726	79202275	79202389	1	0	0	0	0	0	0	0	0	1	98	49	1	1	1	NA	NA	NA	0	1
SE	1853	PHIP	PHIP	chr6	-	78947622	78947775	78946710	78946874	78954813	78954963	28	16	0	1	1	0	0	6	0	0	98	49	1	1	0.933	0.889	NA	NA	1	-0.089
SE	1854	PHIP	PHIP	chr6	-	78965944	78966056	78965702	78965764	78969834	78969917	33	14	8	2	0	0	0	8	0	0	98	49	1	1	0.892	1	1	NA	1	-0.036
SE	1855	PHIP	PHIP	chr6	-	78970048	78970173	78969834	78969917	78970780	78970888	30	8	3	1	0	0	0	4	0	0	98	49	1	1	0.937	1	1	NA	1	-0.021
SE	1856	PHIP	PHIP	chr6	-	79042842	79043003	79025942	79026164	79060477	79060576	47	25	9	0	2	0	6	12	0	0	98	49	1	1	1	0.862	1	1	1	-0.046
SE	1859	SENP6	SENP6	chr6	+	75640683	75640704	75634706	75634811	75647730	75647801	6	4	0	2	0	0	0	1	0	1	70	49	1	1	0.677	1	NA	NA	0.412	0.427
SE	1860	SENP6	SENP6	chr6	+	75647730	75647801	75634706	75634811	75659261	75659407	16	12	10	2	0	0	0	10	0	0	98	49	1	1	0.8	1	1	NA	1	-0.067
SE	1861	SENP6	SENP6	chr6	+	75702644	75703072	75697424	75697517	75709526	75709630	25	1	4	2	1	0	0	7	0	0	98	49	1	1	0.862	0.333	1	NA	1	-0.268
SE	1865	SLC17A5	SLC17A5	chr6	-	73638411	73638499	73636620	73636707	73641690	73641924	6	1	2	0	0	0	0	0	0	1	98	49	1	1	1	1	1	NA	0	1
SE	1869	SMAP1	SMAP1	chr6	+	70791688	70791769	70773349	70773425	70798656	70798737	1	0	2	3	2	0	0	1	0	0	98	49	1	1	0.143	0	1	NA	1	-0.619
SE	1875	ZNF451	ZNF451	chr6	+	57090810	57090894	57090009	57090274	57099060	57099141	4	1	0	0	0	0	0	0	0	3	98	49	1	1	1	1	NA	NA	0	1
SE	1881	EEF1D	EEF1D	chr8	-	143590607	143590738	143588990	143590081	143592646	143592660	2	0	0	1	2	0	2	0	0	0	98	49	1	1	0.5	0	NA	1	NA	-0.75
SE	1900	CDC5L	CDC5L	chr6	+	44426481	44426724	44426102	44426183	44429712	44429910	14	2	6	0	2	0	0	1	0	0	98	49	1	1	1	0.333	1	NA	1	-0.222
SE	1916	VEGFA	VEGFA	chr6	+	43781955	43782087	43778888	43778918	43784540	43786486	0	0	0	1	0	0	0	2	0	0	98	49	1	1	0	NA	NA	NA	1	-1
SE	1921	XPO5	XPO5	chr6	-	43533906	43534007	43531478	43531575	43546570	43546752	3	0	1	0	0	0	0	0	0	1	98	49	1	1	1	NA	1	NA	0	1
SE	1922	YIPF3	YIPF3	chr6	-	43515594	43515701	43513587	43513633	43515888	43516095	33	23	14	0	0	0	0	5	0	1	98	49	1	1	1	1	1	NA	0.714	0.286
SE	1924	TJAP1	TJAP1	chr6	+	43478658	43478718	43478086	43478232	43497880	43497977	1	0	0	1	0	0	0	1	0	0	98	49	1	1	0.333	NA	NA	NA	1	-0.667
SE	1934	KLHDC3	KLHDC3	chr6	+	43017518	43017695	43017133	43017346	43017852	43017968	13	8	4	2	0	0	0	4	0	0	98	49	1	1	0.765	1	1	NA	1	-0.078
SE	1936	KLHDC3	KLHDC3	chr6	+	43018144	43018216	43017852	43017968	43018342	43018556	3	6	6	1	0	0	0	2	0	0	98	49	1	1	0.6	1	1	NA	1	-0.133
SE	1937	PPP2R5D	PPP2R5D	chr6	+	43006910	43007110	43006445	43006679	43007195	43007306	4	4	0	0	1	0	0	1	0	0	98	49	1	1	1	0.667	NA	NA	1	-0.166
SE	1940	UBR2	UBR2	chr6	+	42605720	42605859	42603587	42603718	42606588	42606651	232	113	25	1	0	0	0	23	0	1	98	49	1	1	0.991	1	1	NA	0.92	0.077
SE	1941	UBR2	UBR2	chr6	+	42665408	42665512	42663257	42663419	42666166	42666245	92	43	15	0	0	0	0	12	0	1	98	49	1	1	1	1	1	NA	0.857	0.143
SE	1942	UBR2	UBR2	chr6	+	42673790	42673887	42670659	42670715	42674125	42674193	149	52	20	1	0	0	1	13	0	0	98	49	1	1	0.987	1	1	1	1	-0.004
SE	1943	UBR2	UBR2	chr6	+	42676055	42676191	42674125	42674193	42676782	42676873	67	40	8	1	0	0	0	20	0	1	98	49	1	1	0.971	1	1	NA	0.909	0.081
SE	1944	UBR2	UBR2	chr6	+	42683054	42683111	42679723	42679832	42684793	42684871	88	57	29	2	0	0	0	7	0	0	98	49	1	1	0.957	1	1	NA	1	-0.014
SE	1946	CCND3	CCND3	chr6	-	41940369	41940585	41937234	41937394	41941451	41941848	17	14	5	2	0	0	0	22	0	0	98	49	1	1	0.81	1	1	NA	1	-0.063
SE	1948	CCND3	CCND3	chr6	-	42030008	42030190	41937234	41937394	42048500	42048894	0	0	0	3	0	1	0	1	0	1	98	49	1	1	0	NA	0	NA	0.333	-0.333
SE	1955	TREML1	TREML1	chr6	-	41150260	41150313	41149260	41149918	41150818	41150907	1	0	0	6	0	0	0	0	3	0	98	49	1	1	0.077	NA	NA	0	NA	0.077
SE	1957	ADCY10P1	ADCY10P1	chr6	+	41118923	41119107	41118128	41118184	41120522	41120657	0	0	0	1	0	0	0	1	0	0	98	49	1	1	0	NA	NA	NA	1	-1
SE	1960	ADCY10P1	ADCY10P1	chr6	+	41127544	41127608	41124484	41124820	41132293	41132513	5	0	1	2	0	0	0	1	0	0	98	49	1	1	0.556	NA	1	NA	1	-0.222
SE	1961	NFYA	NFYA	chr6	+	41080810	41080897	41079028	41079164	41084045	41084192	0	0	0	2	2	0	0	1	0	1	98	49	1	1	0	0	NA	NA	0.333	-0.333
SE	1962	C9orf91	C9orf91	chr9	+	114624349	114624459	114617117	114617301	114626898	114627006	4	0	0	0	0	0	0	1	0	2	98	49	1	1	1	NA	NA	NA	0.2	0.8
SE	1966	BTBD9	BTBD9	chr6	-	38592575	38592840	38580217	38580437	38593963	38594327	3	4	0	0	0	0	0	4	0	1	98	49	1	1	1	1	NA	NA	0.667	0.333
SE	1969	MTCH1	MTCH1	chr6	-	36977633	36977691	36977198	36977250	36978077	36978155	65	27	13	1	0	0	2	12	0	0	98	49	1	1	0.97	1	1	1	1	-0.01
SE	1970	MTCH1	MTCH1	chr6	-	36977633	36977784	36977198	36977250	36978077	36978155	47	22	9	1	0	0	2	9	0	0	98	49	1	1	0.959	1	1	1	1	-0.014
SE	1971	MTCH1	MTCH1	chr6	-	36981587	36981672	36978504	36978611	36985852	36986551	33	15	9	0	1	0	2	15	0	0	98	49	1	1	1	0.882	1	1	1	-0.039
SE	1974	STK38	STK38	chr6	-	36498362	36498486	36497779	36497875	36506582	36506644	39	8	2	0	1	0	0	3	0	0	98	49	1	1	1	0.8	1	NA	1	-0.067
SE	1975	STK38	STK38	chr6	-	36499872	36499990	36498362	36498486	36506582	36506644	18	2	3	1	0	0	0	3	0	0	98	49	1	1	0.9	1	1	NA	1	-0.033
SE	1977	KCTD20	KCTD20	chr6	+	36470051	36470257	36442766	36443111	36479120	36479223	6	0	0	0	1	0	0	5	0	0	98	49	1	1	1	0	NA	NA	1	-0.5
SE	1979	KCTD20	KCTD20	chr6	+	36474788	36475062	36470051	36470257	36479120	36479223	12	2	1	1	0	0	0	2	0	4	98	49	1	1	0.857	1	1	NA	0.2	0.752
SE	1981	KCTD20	KCTD20	chr6	+	36479120	36479223	36470051	36470257	36479590	36479711	5	0	0	0	3	0	0	6	0	0	98	49	1	1	1	0	NA	NA	1	-0.5
SE	1985	BRPF3	BRPF3	chr6	+	36222167	36222265	36217916	36218010	36225266	36225364	5	4	0	1	0	0	0	1	0	0	98	49	1	1	0.714	1	NA	NA	1	-0.143
SE	1986	SRPK1	SRPK1	chr6	-	35869009	35869110	35857260	35857368	35869481	35869901	49	15	8	2	1	0	0	5	0	0	98	49	1	1	0.925	0.882	1	NA	1	-0.064
SE	1988	FKBP5	FKBP5	chr6	-	35587033	35587117	35580035	35580221	35591129	35591220	9	4	2	0	0	0	0	12	0	1	98	49	1	1	1	1	1	NA	0.857	0.143
SE	1990	FKBP5	FKBP5	chr6	-	35620131	35620274	35619095	35619210	35637013	35637158	11	2	5	2	0	0	0	13	0	0	98	49	1	1	0.733	1	1	NA	1	-0.089
SE	1991	FKBP5	FKBP5	chr6	-	35642719	35642843	35637013	35637158	35688803	35688942	7	5	4	0	0	2	0	3	0	0	98	49	1	1	1	1	0.5	NA	1	-0.167
SE	2000	TAF11	TAF11	chr6	-	34879966	34880063	34877777	34878720	34880288	34880376	2	0	0	1	0	0	0	0	0	2	98	49	1	1	0.5	NA	NA	NA	0	0.5
SE	2001	YKT6	YKT6	chr7	+	44211022	44211124	44208132	44208198	44212246	44214294	7	4	4	0	1	0	0	1	0	0	98	49	1	1	1	0.667	1	NA	1	-0.111
SE	2025	TAPBP	TAPBP	chr6	-	33313216	33313477	33304988	33305387	33313693	33313864	50	15	8	1	0	0	9	3	0	0	98	49	1	1	0.962	1	1	1	1	-0.013
SE	2026	TAPBP	TAPBP	chr6	-	33313693	33313864	33313216	33313477	33314004	33314387	27	9	4	0	0	0	4	1	0	1	98	49	1	1	1	1	1	1	0.333	0.334
SE	2033	TAP1	TAP1	chr6	-	32847918	32848092	32847512	32847675	32848651	32848840	69	36	37	1	0	0	4	21	0	0	98	49	1	1	0.972	1	1	1	1	-0.009
SE	2062	HLA-DQB1	HLA-DQB1	chr6	-	32661346	32661457	32659463	32660249	32661966	32662248	9	0	0	0	0	0	0	4	0	2	98	49	1	1	1	NA	NA	NA	0.5	0.5
SE	2063	HLA-DQB1	HLA-DQB1	chr6	-	32664797	32665067	32661966	32662248	32666498	32666689	28	2	0	3	0	0	0	9	0	0	98	49	1	1	0.824	1	NA	NA	1	-0.088
SE	2089	PBX2	PBX2	chr6	-	32186812	32186901	32186603	32186690	32187241	32187395	88	43	26	1	1	0	1	11	0	0	98	49	1	1	0.978	0.956	1	1	1	-0.022
SE	2133	C6orf48	C6orf48	chr6	+	31836423	31836517	31835410	31835451	31837235	31837435	1	2	0	7	4	0	0	0	0	1	98	49	1	1	0.067	0.2	NA	NA	0	0.134
SE	2135	DDAH2	DDAH2	chr6	-	31728864	31729225	31728645	31728745	31730050	31730265	13	6	0	0	0	1	0	8	0	0	98	49	1	1	1	1	0	NA	1	-0.333
SE	2169	BAG6	BAG6	chr6	-	31639499	31639646	31639027	31639226	31640198	31640306	12	1	3	11	2	3	0	4	0	2	98	49	1	1	0.353	0.2	0.333	NA	0.5	-0.205
SE	2170	BAG6	BAG6	chr6	-	31640384	31640528	31640198	31640306	31640644	31640704	37	7	9	0	0	2	1	16	0	0	98	49	1	1	1	1	0.692	1	1	-0.103
SE	2171	BAG6	BAG6	chr6	-	31640644	31640704	31640198	31640306	31640791	31640938	17	2	2	0	1	0	0	1	0	0	98	49	1	1	1	0.5	1	NA	1	-0.167
SE	2172	BAG6	BAG6	chr6	-	31643889	31643974	31643100	31643115	31644081	31644194	11	1	3	0	0	0	1	2	0	1	98	49	1	1	1	1	1	1	0.5	0.25
SE	2173	BAG6	BAG6	chr6	-	31643889	31643977	31642828	31643115	31644081	31644194	12	1	4	0	0	0	1	3	0	1	98	49	1	1	1	1	1	1	0.6	0.2
SE	2176	PRRC2A	PRRC2A	chr6	+	31622689	31622901	31620672	31620858	31624260	31624360	2	1	0	0	0	0	0	1	2	0	98	49	1	1	1	1	NA	0	1	0.5
SE	2178	PRRC2A	PRRC2A	chr6	+	31623731	31623909	31622689	31622901	31624260	31624360	34	10	6	0	1	0	1	6	0	0	98	49	1	1	1	0.833	1	1	1	-0.056
SE	2181	PRRC2A	PRRC2A	chr6	+	31633378	31633647	31631138	31632992	31633858	31633989	9	5	1	1	0	0	0	4	0	0	98	49	1	1	0.818	1	1	NA	1	-0.061
SE	2183	AIF1	AIF1	chr6	+	31615669	31615736	31615520	31615582	31616343	31616506	105	37	22	1	0	0	2	21	0	0	98	49	1	1	0.981	1	1	1	1	-0.006
SE	2184	AIF1	AIF1	chr6	+	31616103	31616145	31615669	31615736	31616343	31616506	111	101	88	1	0	1	0	44	0	0	91	49	1	1	0.984	1	0.979	NA	1	-0.012
SE	2185	TNF	TNF	chr6	+	31576533	31575579	31575566	31575927	31576766	31576814	5	1	0	0	0	0	0	2	0	1	95	49	1	1	1	1	NA	NA	0.508	0.492
SE	2186	DDX39B	DDX39B	chr6	-	31538762	31538855	31536499	31536683	31540321	31540534	15	10	0	2	0	0	0	1	0	0	98	49	1	1	0.789	1	NA	NA	1	-0.105
SE	2187	DDX39B	DDX39B	chr6	-	31539146	31539274	31538762	31538855	31540321	31540534	60	21	10	1	0	0	2	20	0	0	98	49	1	1	0.968	1	1	1	1	-0.011
SE	2212	FLOT1	FLOT1	chr6	-	30730021	30730186	30727733	30728145	30730427	30730565	37	19	26	0	5	0	0	6	0	0	98	49	1	1	1	0.655	1	NA	1	-0.115
SE	2213	FLOT1	FLOT1	chr6	-	30741189	30741333	30740678	30740798	30741613	30741704	50	24	20	1	0	0	0	12	0	0	98	49	1	1	0.962	1	1	NA	1	-0.013
SE	2214	FLOT1	FLOT1	chr6	-	30741189	30741333	30740678	30740798	30742146	30742203	39	12	17	0	1	1	0	12	0	0	98	49	1	1	1	0.857	0.895	NA	1	-0.083
SE	2215	FLOT1	FLOT1	chr6	-	30741613	30741704	30741189	30741333	30741791	30741867	34	26	16	0	2	0	1	6	0	1	98	49	1	1	1	0.867	1	1	0.75	0.081
SE	2216	FLOT1	FLOT1	chr6	-	30741791	30741867	30741189	30741333	30742146	30742203	24	7	4	0	0	0	0	2	0	2	98	49	1	1	1	1	1	NA	0.333	0.667
SE	2294	HLA-E	HLA-E	chr6	+	30491536	30491653	30491136	30491412	30492403	30492436	747	497	484	1	1	0	33	293	0	1	98	49	1	1	0.997	0.996	1	1	0.993	0.001
SE	2295	HLA-E	HLA-E	chr6	+	30492403	30492436	30491536	30491653	30492540	30492583	584	384	406	0	4	0	26	172	0	1	82	49	1	1	1	0.983	1	1	0.99	-0.001
SE	2296	HLA-E	HLA-E	chr6	+	30492540	30492583	30491536	30491653	30492748	30494205	221	145	117	0	0	0	3	55	1	0	92	49	1	1	1	1	1	0.615	1	0.193
SE	2307	HLA-H	HLA-H	chr6	+	29889706	29889823	29889327	29889604	29890263	29890296	613	40	312	0	0	1	18	52	0	0	98	49	1	1	1	1	0.994	1	1	-0.002
SE	2314	HLA-F	HLA-F	chr6	+	29725030	29725306	29724172	29724448	29725446	29725563	316	96	64	2	0	2	4	52	0	0	98	49	1	1	0.987	1	0.941	1	1	-0.024
SE	2315	HLA-F	HLA-F	chr6	+	29725446	29725563	29725030	29725306	29726402	29726526	197	46	33	1	1	0	4	28	0	0	98	49	1	1	0.99	0.958	1	1	1	-0.017
SE	2316	HLA-F	HLA-F	chr6	+	29726010	29726043	29725446	29725563	29726402	29726526	148	28	38	0	0	1	4	18	0	0	82	49	1	1	1	1	0.958	1	1	-0.014
SE	2317	HLA-F	HLA-F	chr6	+	29726010	29726043	29725446	29725563	29726882	29727296	74	16	14	0	0	1	3	16	0	0	82	49	1	1	1	1	0.893	1	1	-0.036
SE	2365	BTN2A1	BTN2A1	chr6	+	26458606	26458718	26457903	26458142	26459480	26459828	5	0	2	3	0	0	0	1	0	1	98	49	1	1	0.455	NA	1	NA	0.333	0.395
SE	2376	FAM65B	FAM65B	chr6	-	24832255	24832391	24830508	24830670	24839090	24839272	18	16	12	0	0	0	1	4	0	1	98	49	1	1	1	1	1	1	0.667	0.166
SE	2377	FAM65B	FAM65B	chr6	-	24835702	24835871	24832255	24832391	24839090	24839272	113	65	53	1	0	0	0	36	0	0	98	49	1	1	0.983	1	1	NA	1	-0.006
SE	2378	FAM65B	FAM65B	chr6	-	24835702	24835871	24832255	24832391	24842861	24843554	61	28	18	1	1	0	0	21	0	0	98	49	1	1	0.968	0.933	0.9	NA	1	-0.066
SE	2379	FAM65B	FAM65B	chr6	-	24839090	24839272	24832255	24832391	24842861	24843554	81	33	16	1	1	1	0	4	0	0	98	49	1	1	0.976	0.943	0.889	NA	1	-0.064
SE	2380	FAM65B	FAM65B	chr6	-	24839090	24839272	24835702	24835871	24842861	24843554	131	71	51	0	1	0	0	19	0	0	98	49	1	1	1	0.973	1	NA	1	-0.009
SE	2383	FAM65B	FAM65B	chr6	-	24870865	24870889	24869093	24869147	24872880	24872959	224	146	94	2	0	0	4	57	0	0	73	49	1	1	0.987	1	1	1	1	-0.004
SE	2384	KIAA0319	KIAA0319	chr6	-	24554540	24554631	24551433	24551525	24559012	24559155	2	1	0	1	0	0	0	1	0	0	98	49	1	1	0.5	1	NA	NA	1	-0.25
SE	2386	KIAA0319	KIAA0319	chr6	-	24572574	24572659	24569902	24570035	24576367	24576596	0	1	0	1	0	0	0	2	0	0	98	49	1	1	0	1	NA	NA	1	-0.5
SE	2387	KIAA0319	KIAA0319	chr6	-	24572574	24572698	24569902	24570035	24576367	24576596	1	1	2	1	0	0	0	2	0	0	98	49	1	1	0.333	1	1	NA	1	-0.222
SE	2393	TMEM164	TMEM164	chrX	+	110003500	110004164	110002634	110002772	110067346	110067396	35	6	0	3	0	0	2	5	0	0	98	49	1	1	0.854	1	NA	1	1	-0.073
SE	2395	RNF144B	RNF144B	chr6	+	18427580	18427685	18399498	18399699	18439683	18439744	18	9	13	0	4	3	0	10	0	0	98	49	1	1	1	0.529	0.684	NA	1	-0.262
SE	2396	RNF144B	RNF144B	chr6	+	18457154	18457359	18439683	18439744	18459606	18459751	15	9	10	2	0	0	0	9	0	3	98	49	1	1	0.789	1	1	NA	0.6	0.33
SE	2398	KDM1B	KDM1B	chr6	+	18162834	18162924	18161326	18161454	18166266	18166378	22	10	4	0	0	1	2	3	0	1	98	49	1	1	1	1	0.667	1	0.6	0.089
SE	2399	KDM1B	KDM1B	chr6	+	18185771	18185810	18171362	18171479	18197056	18197233	3	6	3	1	0	0	0	5	0	0	88	49	1	1	0.626	1	1	NA	1	-0.125
SE	2400	NUP153	NUP153	chr6	-	17632649	17632844	17628654	17629539	17637152	17637770	23	10	12	1	0	0	0	6	0	0	98	49	1	1	0.92	1	1	NA	1	-0.027
SE	2402	NUP153	NUP153	chr6	-	17651833	17651926	17649162	17649300	17661652	17661779	0	2	3	10	1	0	0	0	0	6	98	49	1	1	0	0.5	0.6	NA	0	0.367
SE	2403	NUP153	NUP153	chr6	-	17661652	17661779	17649162	17649300	17662017	17662070	27	3	3	1	0	0	0	7	0	0	98	49	1	1	0.931	1	1	NA	1	-0.023
SE	2404	NUP153	NUP153	chr6	-	17668974	17669028	17665238	17665385	17669292	17669337	48	20	4	0	0	0	3	7	0	1	98	49	1	1	1	1	1	1	0.778	0.111
SE	2405	ATXN1	ATXN1	chr6	-	16753232	16753347	16657775	16657901	16761297	16761490	12	5	2	1	0	0	0	1	0	0	98	49	1	1	0.857	1	1	NA	1	-0.048
SE	2414	RANBP9	RANBP9	chr6	-	13642478	13642591	13641198	13641307	13644544	13644729	22	9	4	0	2	0	0	5	0	0	98	49	1	1	1	0.692	1	NA	1	-0.103
SE	2415	SIRT5	SIRT5	chr6	+	13588330	13588464	13584075	13584225	13591668	13591894	4	0	0	2	0	0	0	2	0	0	98	49	1	1	0.5	NA	NA	NA	1	-0.5
SE	2418	TBC1D7	TBC1D7	chr6	-	13325093	13325174	13320907	13321095	13326786	13326906	4	0	0	1	5	0	0	1	0	0	98	49	1	1	0.667	0	NA	NA	1	-0.666
SE	2419	HIVEP1	HIVEP1	chr6	+	12129758	12129892	12119889	12125870	12130766	12130942	21	1	2	0	1	0	0	3	0	1	98	49	1	1	1	0.333	1	NA	0.6	0.178
SE	2423	NEDD9	NEDD9	chr6	-	11213280	11213727	11193590	11193692	11305991	11306155	24	11	15	1	0	0	3	2	0	0	98	49	1	1	0.923	1	1	1	1	-0.026
SE	2424	NEDD9	NEDD9	chr6	-	11305065	11305159	11213280	11213727	11305991	11306155	13	11	4	13	5	8	0	1	0	1	98	49	1	1	0.333	0.524	0.2	NA	0.333	0.019
SE	2425	MAK	MAK	chr6	-	10770110	10770230	10762722	10764606	10775327	10775459	3	2	2	1	0	0	0	3	0	0	98	49	1	1	0.6	1	1	NA	1	-0.133
SE	2426	MAK	MAK	chr6	-	10773033	10773108	10770110	10770230	10775327	10775459	0	1	0	0	0	1	0	0	0	3	98	49	1	1	NA	1	0	NA	0	0.5
SE	2427	MAK	MAK	chr6	-	10791674	10791847	10784423	10784572	10795997	10796309	2	4	1	3	0	0	0	4	0	0	98	49	1	1	0.25	1	1	NA	1	-0.25
SE	2428	MCU	MCU	chr10	+	72715110	72715226	72692130	72692301	72715853	72715902	15	7	3	0	2	0	0	1	0	0	98	49	1	1	1	0.636	1	NA	1	-0.121
SE	2435	TNFSF13	TNFSF13	chr17	+	7559845	7559893	7559623	7559702	7560048	7560167	18	3	1	0	0	0	0	11	0	1	97	49	1	1	1	1	1	NA	0.847	0.153

SE	2446 XAF1	XAF1	chr17	+	6759661	6759718	6758088	6758224	6760405	6760601	14	20	1	0	2	0	0	19	0	0	98	49	1	1	1	0.833	1	NA			1	-0.056
SE	2450 XAF1	XAF1	chr17	+	6760405	6760601	6759661	6759718	6762154	6762240	6	11	1	0	1	0	0	16	0	0	98	49	1	1	1	0.846	1	NA			1	-0.051
SE	2451 XAF1	XAF1	chr17	+	6762154	6762240	6758088	6758224	6770642	6770984	5	5	0	1	0	0	0	11	0	0	98	49	1	1	0.714	1	NA	NA			1	-0.143
SE	2459 DERL2	DERL2	chr17	-	5481295	5481389	5480386	5480582	5482808	5482882	4	1	4	0	0	5	0	1	0	0	98	49	1	1	1	1	0.286	NA			1	-0.238
SE	2461 EML3	EML3	chr11	-	62605641	62605773	62605112	62605180	62605854	62605980	13	5	1	1	0	0	0	1	0	0	98	49	1	1	0.867	1	1	NA			1	-0.044
SE	2465 CAMTA2	CAMTA2	chr17	-	4973584	4973769	4973174	4973253	4974384	4974500	6	8	7	2	0	0	0	3	0	0	98	49	1	1	0.6	1	1	NA			1	-0.133
SE	2466 CAMTA2	CAMTA2	chr17	-	4982088	4982160	4981677	4981831	4982756	4982892	21	1	3	1	0	0	0	2	0	0	98	49	1	1	0.913	1	1	NA			1	-0.029
SE	2481 ARRB2	ARRB2	chr17	+	4715012	4715043	4710493	4710744	4715972	4716033	32	34	5	7	1	0	0	7	0	0	80	49	1	1	0.737	0.954	1	NA			1	-0.103
SE	2483 ARRB2	ARRB2	chr17	+	4716411	4716608	4715972	4716033	4717216	4717276	26	10	1	0	0	0	0	2	0	3	98	49	1	1	1	1	1	NA			0.25	0.75
SE	2486 DDX49	DDX49	chr19	+	18921662	18921748	18920579	18920703	18921842	18921964	5	3	0	0	3	0	0	7	0	0	98	49	1	1	1	0.333	NA	NA			1	-0.334
SE	2490 PRPF8	PRPF8	chr17	-	1661610	1661790	1661270	1661406	1661905	1662153	19	10	9	1	0	0	0	5	0	0	98	49	1	1	0.905	1	1	NA			1	-0.032
SE	2493 INPP5K	INPP5K	chr17	-	1509682	1509799	1509177	1509353	1513871	1513979	1	0	2	2	0	0	0	1	0	0	98	49	1	1	0.2	NA	1	NA			1	-0.4
SE	2494 INPP5K	INPP5K	chr17	-	1513452	1513561	1509682	1509799	1513871	1513979	7	5	8	0	0	0	0	2	0	1	98	49	1	1	1	1	1	NA			0.5	0.5
SE	2496 INPP5K	INPP5K	chr17	-	1515426	1515585	1513871	1513979	1516455	1516888	0	1	0	1	1	3	1	0	0	1	98	49	1	1	0	0.333	0		1		0	-0.389
SE	2500 YWHAE	YWHAE	chr17	-	1361901	1362008	1361091	1361298	1364858	1365058	27	3	5	1	0	0	0	5	0	0	98	49	1	1	0.931	1	1	NA			1	-0.023
SE	2502 BIVM-ERCC5	BIVM-ERCC5	chr13	+	102865666	102865911	102862029	102863103	102866261	102866381	8	5	2	2	0	0	3	4	0	0	98	49	1	1	0.667	1	1		1		1	-0.111
SE	2503 CCDC130	CCDC130	chr19	+	13754288	13754342	13751607	13751811	13756196	13756279	16	5	2	2	0	0	0	2	0	0	98	49	1	1	0.8	1	1	NA			1	-0.067
SE	2504 GLOD4	GLOD4	chr17	-	775774	775919	771324	771461	776867	776988	7	0	0	0	0	0	0	0	0	2	98	49	1	1	1	NA	NA	NA			0	1
SE	2506 GNB4	GNB4	chr3	-	179419398	179419505	179416492	179416556	179420888	179420927	13	5	4	1	0	0	0	8	0	0	98	49	1	1	0.867	1	1	NA			1	-0.044
SE	2508 CHMP1A	CHMP1A	chr16	-	89649350	89649497	89647034	89647331	89651568	89651646	27	11	13	0	0	1	0	4	0	0	98	49	1	1	1	1	0.867	NA			1	-0.044
SE	2514 SLC4A7	SLC4A7	chr3	-	27421621	27421779	27420699	27420787	27424036	27424152	4	0	2	0	3	0	0	1	0	0	98	49	1	1	1	0	1	NA			1	-0.333
SE	2517 BSDC1	BSDC1	chr1	-	32369229	32369338	32368446	32368550	32376261	32376741	0	0	3	2	3	0	0	0	0	1	98	49	1	1	0	0	1	NA			0	0.333
SE	2518 BSDC1	BSDC1	chr1	-	32383829	32383997	32381213	32381268	32386778	32386895	16	9	15	0	0	0	0	1	0	1	98	49	1	1	1	1	1	NA			0.333	0.667
SE	2519 BSDC1	BSDC1	chr1	-	32383829	32384048	32381213	32381268	32386778	32386895	8	5	12	0	0	0	0	0	0	1	98	49	1	1	1	1	1	NA			0	1
SE	2523 ZC3H18	ZC3H18	chr16	+	88587541	88587613	88586599	88586684	88598177	88598326	5	5	0	3	1	0	0	1	0	0	98	49	1	1	0.455	0.714	NA	NA			1	-0.415
SE	2533 ANXA6	ANXA6	chr5	-	151110626	151110644	151109752	151109846	151117126	151117180	17	2	4	1	0	2	0	2	0	0	67	49	1	1	0.926	1	0.594	NA			1	-0.16
SE	2534 FYN	FYN	chr6	-	111699514	111699670	111696276	111696456	111702884	111703034	5	3	0	3	0	0	0	7	0	0	98	49	1	1	0.455	1	NA	NA			1	-0.272
SE	2536 EXOC6	EXOC6	chr10	+	92934143	92934190	92928338	92928422	92934309	92934430	23	12	4	1	0	0	0	5	0	0	96	49	1	1	0.922	1	1	NA			1	-0.026
SE	2537 EXOC6	EXOC6	chr10	+	92934309	92934430	92934143	92934190	92935813	92935885	19	4	5	0	0	0	0	9	1	0	98	49	1	1	1	1	1		0		1	0.5
SE	2538 EXOC6	EXOC6	chr10	+	92935813	92935885	92934309	92934430	92940726	92940824	20	4	4	0	1	0	0	8	0	0	98	49	1	1	1	0.667	1	NA			1	-0.111
SE	2539 EXOC6	EXOC6	chr10	+	92952272	92952382	92948273	92948379	92954629	92954741	20	5	3	0	2	0	0	2	0	0	98	49	1	1	1	0.556	1	NA			1	-0.148
SE	2541 MAP3K1	MAP3K1	chr5	+	56887377	56887520	56885931	56886063	56888225	56888357	34	26	13	2	0	1	0	11	0	0	98	49	1	1	0.895	1	0.867	NA			1	-0.079
SE	2545 PLCG2	PLCG2	chr16	+	81858262	81858356	81854443	81854587	81859115	81859163	19	21	6	1	0	0	0	9	0	0	98	49	1	1	0.905	1	1	NA			1	-0.032
SE	2546 PLCG2	PLCG2	chr16	+	81880909	81880953	81870851	81870935	81889171	81889273	12	6	0	1	0	0	3	0	0	0	93	49	1	1	0.863	1	NA		1	NA		-0.069
SE	2547 PLCG2	PLCG2	chr16	+	81883268	81883341	81880909	81880953	81889171	81889273	9	20	1	0	1	0	0	12	0	0	98	49	1	1	1	0.909	1	NA			1	-0.03
SE	2548 PLCG2	PLCG2	chr16	+	81956694	81956879	81946174	81946263	81957955	81962693	42	15	6	2	0	0	0	7	0	0	98	49	1	1	0.913	1	1	NA			1	-0.029
SE	2553 ANXA3	ANXA3	chr4	+	78586259	78586350	78582176	78582290	78591543	78591623	37	24	18	2	0	0	1	19	0	0	98	49	1	1	0.902	1	1		1		1	-0.033
SE	2555 RTFDC1	RTFDC1	chr20	+	56516934	56516989	56513314	56513428	56517105	56517201	69	49	26	1	0	0	0	7	0	2	98	49	1	1	0.972	1	1	NA			0.636	0.355
SE	2562 ZMYM2	ZMYM2	chr13	+	19970024	19970071	19959957	19960026	19993062	19993919	6	4	0	2	0	0	0	1	0	0	96	49	1	1	0.605	1	NA	NA			1	-0.198
SE	2563 PSME3	PSME3	chr17	+	42838092	42838205	42837648	42837697	42838730	42838799	34	18	12	1	0	0	0	5	0	0	98	49	1	1	0.944	1	1	NA			1	-0.019
SE	2564 HNRNPR	HNRNPR	chr1	-	23318482	23318688	23313552	23313702	23321527	23321663	7	4	3	0	1	0	0	4	0	0	98	49	1	1	1	0.667	1	NA			1	-0.111
SE	2565 HNRNPR	HNRNPR	chr1	-	23318482	23318697	23313552	23313702	23321527	23321663	3	1	3	0	1	0	0	4	0	0	98	49	1	1	1	0.333	1	NA			1	-0.222
SE	2572 NEMF	NEMF	chr14	-	49829053	49829262	49828615	49828807	49829348	49829426	6	15	12	1	0	0	0	4	0	0	98	49	1	1	0.75	1	1	NA			1	-0.083
SE	2573 NEMF	NEMF	chr14	-	49832050	49832126	49831298	49831361	49832206	49832277	6	6	1	1	0	0	0	3	0	0	98	49	1	1	0.75	1	1	NA			1	-0.083
SE	2574 NEMF	NEMF	chr14	-	49833422	49833496	49832206	49832277	49834362	49834449	7	4	7	0	0	1	1	0	0	0	98	49	1	1	1	1	0.778		1	NA		-0.074
SE	2575 NEMF	NEMF	chr14	-	49840717	49840866	49838138	49838206	49846139	49846265	7	11	0	0	2	0	0	2	0	0	98	49	1	1	1	0.733	NA	NA			1	-0.133
SE	2583 RPL27	RPL27	chr17	+	42999932	43000102	42998748	42998831	43002672	43002783	97	25	13	1	0	0	3	13	0	0	98	49	1	1	0.98	1	1		1		1	-0.007
SE	2585 CDC42SE1	CDC42SE1	chr1	-	151054230	151054321	151050970	151053327	151055676	151055993	126	56	40	1	0	0	0	20	0	0	98	49	1	1	0.984	1	1	NA			1	-0.005
SE	2586 CDC42SE1	CDC42SE1	chr1	-	151055015	151055126	151054230	151054321	151055676	151055993	253	113	63	3	0	0	6	25	0	0	98	49	1	1	0.977	1	1		1		1	-0.008
SE	2587 CDC42SE1	CDC42SE1	chr1	-	151055676	151055993	151055015	151055126	151059478	151059649	172	70	37	1	0	0	6	17	0	0	98	49	1	1	0.989	1	1		1		1	-0.004
SE	2589 NFAT5	NFAT5	chr16	+	69568494	69568548	69565965	69566374	69626402	69626528	4	11	0	3	3	0	0	5	0	1	98	49	1	1	0.4	0.647	NA	NA			0.714	-0.19
SE	2590 NFAT5	NFAT5	chr16	+	69646516	69646578	69626402	69626528	69647027	69647586	10	6	0	2	0	0	0	3	0	0	98	49	1	1	0.714	1	NA	NA			1	-0.143
SE	2592 ERCC6-PGBD3	ERCC6-PGBD3	chr10	-	49530719	49530840	49528416	49528525	49532542	49532978	6	2	0	1	0	0	0	3	0	0	98	49	1	1	0.75	1	NA	NA			1	-0.125
SE	2596 MS4A7	MS4A7	chr11	+	60385087	60385222	60383128	60383288	60386716	60386773	7	0	0	4	0	0	0	10	0	6	98	49	1	1	0.467	NA	NA	NA			0.455	0.012
SE	2597 MS4A7	MS4A7	chr11	+	60392684	60392786	60389389	60389596	60393786	60395953	7	0	2	1	0	0	0	13	0	5	98	49	1	1	0.778	NA	1	NA			0.565	0.324
SE	2598 EDC4	EDC4	chr16	+	67882678	67882865	67882428	67882594	67882957	67883177	11	2	1	1	0	0	0	1	0	0	98	49	1	1	0.846	1	1	NA			1	-0.051
SE	2599 NAA20	NAA20	chr20	+	20032507	20032653	20026783	20026919	20033101	20033629	1	0	0	0	0	0	0	3	0	1	98	49	1	1	1	NA	NA	NA			0.6	0.4
SE	2601 SPG21	SPG21	chr15	-	64983506	64983593	64980863	64981025	64989664	64989946	22	7	15	0	0	0	1	6	0	1	98	49	1	1	1	1	1		1		0.75	0.125
SE	2602 CYTH4	CYTH4	chr22	+	37303253	37303402	37300906	37301019	37309211	37309323	107	76	29	0	2	0	2	12	0	0	98	49	1	1	1	0.95	1		1		1	-0.017
SE	2604 CYTH4	CYTH4	chr22	+	37311455	37311527	37310987	37311064	37312019	37312174	146	103	62	1	0	0	2	23	0	0	98	49	1	1	0.986	1	1		1		1	-0.005
SE	2607 ATP6V0D1	ATP6V0D1	chr16	-	67444527	67444706	67443098	67443178	67453543	67453715	40	22	21	2	1	1	0	13	0	0	98	49	1	1	0.909	0.917	0.913	NA			1	-0.087
SE	2616 CKLF	CKLF	chr16	+	66558189	66558348	66552562	66552793	66563121	66563217	82	69	45	17	11	4	0	8	0	2	98	49	1	1	0.707	0.758	0.849	NA			0.667	0.104
SE	2620 CKLF	CKLF	chr16	+	66558189	66558348	66552562	66552793	66565885	66566287	71	53	18	1	0	1	0	9	0	0	98	49	1	1	0.973	1	0.9	NA			1	-0.042
SE	2621 CKLF	CKLF	chr16	+	66563121	66563217	66552562	66552793	66565885	66566287	47	24	20	1	0	1	0	7	0	0	98	49	1	1	0.959	1	0.909	NA			1	-0.044
SE	2622 CKLF	CKLF	chr16	+	66563121	66563217	66558189	66558348	66565885	66566287	43	33	43	2	4	0	0	6	0	2	98	49	1	1	0.915	0.805	1	NA			0.6	0.307
SE	2628 NLRC5	NLRC5	chr16	+	57028071	57028155	57025367	57027018	57028301	57028385	16	6	0	1	0	0	0	2	0	0	98	49	1	1	0.889	1	NA	NA			1	-0.055
SE	2630 NLRC5	NLRC5	chr16	+	57054750	57054840	57051537	57051621	57055031	57055094	52	17	3	2	0	0	3	12	0	1	98	49	1	1	0.929	1	1		1		0.857	0.048
SE	2648 TLR1	TLR1	chr4	-	38800856	38800948	38796254	38798898	38804305	38804382	97	25	13	9	2	0	0	5	0	0	98	49	1	1	0.843	0.862	1	NA			1	-0.098
SE	2653 ASB6	ASB6	chr9	-	129640540	129640722	129639401	129639508	129641886	129642169	6	1	0	0	0	0	0	1	0	1	98	49	1	1	1	1	NA	NA			0.333	0.667
SE	2661 ADCY7	ADCY7	chr16	+	50290456	50290660	50287911	50288350	50292675	50292825	14	5	2	0	0	0	0	6	0	1	98	49	1	1	1	1	1	NA			0.75	0.25
SE	2662 ADCY7	ADCY7	chr16	+	50291735	50291897	50290456	50290660	50292675	50292825	19	13	0	1	0	0	3	6	0	0	98	49	1	1	0.905	1	NA		1		1	-0.047
SE	2663 ADCY7	ADCY7	chr16	+	50293353	50293502	50292675	50292825	50294639	50294751	9	2	5	1	0	0	0	3	0	0	98	49	1	1	0.818	1	1	NA			1	-0.061
SE	2664 ADCY7	ADCY7	chr16	+	50310686	50310880	50309547	50309646	50311692	50311786	23	14	1	2	0	0	0	5	0	0	98	49	1	1	0.852	1	1	NA			1	-0.049
SE	2665 ADCY7	ADCY7	chr16	+	50315013	50315138	50314291	50314406	50315358	50318134	26	12	0	0	1	0	1	9	0	0	98	49	1	1	1	0.857	NA		1		1	-0.072
SE	2669 LONP2	LONP2	chr16	+	48256609	48256741	48252130	48252365	48258617	48258740	11	3	1	0	0	0	0	0	0	1	98	49	1	1	1	1	1	NA			0	1
SE	2670 LONP2	LONP2	chr16	+	48261423	48261587	48258617	48258740	48262777	48262872	5	0	3	0	0	0	0	4	0	1	98	49	1	1	1	NA	1	NA			0.667	0.333
SE	2673 PHKB	PHKB	chr16	+	47547432	47547548	47515520	47515601	47580294	47580358	3	0	0	0	0	0	0	1	0	1	98	49	1	1	1	NA	NA	NA			0.333	0.667
SE	2675 PHKB	PHKB	chr16	+	47665941	47666032	47663676	47663734	47669214	47669417	2	0	0	1	0	0	0	2	0	0	98	49	1	1	0.5	NA	NA	NA			1	-0.5
SE	2676 CEACAM3	CEACAM3	chr19	+	41807110	41807617	41797588	41797948	41808812	41808930	4	1	0	15	4	6	0	2	0	2	98	49	1	1	0.118	0.111	0	NA			0.333	-0.257
SE	2690 STX4	STX4	chr16	+	31039722	31039818	31039540	31039651	31039911	31040167	12	11	1	1	0	0	0	5	0	0	98	49	1	1	0.857	1	1	NA			1	-0.048
SE	2695 TMEM189	TMEM189	chr20	-	50151211	50151315	50143501	50143621	50153516	50153798	3	0	0	2	0	0	0	0	0	2	98	49	1	1	0.429	NA	NA	NA			0	0.429
SE	2698 SLPI	SLPI	chr20	-	45253001	45253151	45252238	45252419	45253574	45253733	14	18	3	1	0	0	1	4	0	0	98	49	1	1	0.875	1	1		1		1	-0.042
SE	2699 NR4A1	NR4A1	chr12	+	52052495	52052645	52051401	52051568	52054326	52055204	2	0	0	1	0	0	0	1	0	2	98	49	1	1	0.5	NA	NA	NA			0.2	0.3
SE	2704 MAZ	MAZ	chr16	+	29809539	29809764	29808569	29808741	29810076	29811183	9	0	9	12	1	0	0	1	0	0	98	49	1	1	0.273	0	1	NA			1	-0.576
SE	2707 LY75-CD302	LY75-CD302	chr2	-	159882123	159882315	159881082	159881240	159885152	159885293	30	13	4	1	0	0	0	2	0	0	98	49	1	1	0.937	1	1	NA			1	-0.021
SE	2708 LY75-CD302	LY75-CD302	chr2	-	159885152	159885293	159881082	159881240	159886419	159886530	23	4	6	0	2	0	0	1	0	0	98	49	1	1	1	0.5	1	NA			1	-0.167
SE	2709 PIP4K2B	PIP4K2B	chr17	-	38784242	38784339	38780451	38780604	38786822	38786920	1	0	0	1	0	0	0	2	0	0	98	49	1	1	0.333	NA	NA	NA			1	-0.667
SE	2711 SH2B1	SH2B1	chr16	+	28866011	28867033	28863667	28863754	28867330	28867432	2	0	0	0	0	0	0	0	0	1	98	49	1	1	1	NA	NA	NA			0	1
SE	2713 TUFM	TUFM	chr16	-	28845313	28845480	28844950	28845055	28845911	28846106	5	0	1	1	0	0	0	1	0	0	98	49	1	1	0.714	NA	1	NA			1	-0.143
SE	2714 ATXN2L	ATXN2L	chr16	+	28826861	28826986	28826239	28826390	28829400	28829492	11	8	0	1	0	0	0	1	0	0	98	49	1	1	0.846	1	NA	NA			1	-0.077
SE	2715 ATXN2L	ATXN2L	chr16	+	28832816	28832887	28832495	28832567	28833058	28833354	12	2	0	1	0	0	0	3	0	0	98	49	1	1	0.857	1	NA	NA			1	-0.072
SE	2718 CD33	CD33	chr19	+	51226308	51226356	51225802	51226081	51235156	51235253	3	0	0	0	0	0	0	7	1	0	97	49	1	1	1	NA	NA		0		1	0.5
SE	2722 PRKCB	PRKCB	chr16	+	23837374	23837406	23835978	23836348	23988507	23988590	40	36	7	0	1	0	7	15	0	0	81	49	1	1	1	0.956	1		1		1	-0.015
SE	2726 EIF2S2	EIF2S2	chr20	-	34093674	34093731	34090516	34090602	34096656	34096805	6	0	0	0	3	0	0	4	0	0	98	49	1	1	1	0	NA	NA			1	-0.5
SE	2735 RHOT1	RHOT1	chr17	+	32193986	32194107	32193135	32193244	32198946	32199031	16	15	6	1	0	0	0	7	0	0	98	49	1	1	0.889	1	1	NA			1	-0.037
SE	2736 RHOT1	RHOT1	chr17	+	32209349	32209445	32208106	32208309	32211115	32211238	4	5	1	2	0	0	0	2	0	0	98	49	1	1	0.5	1	1	NA			1	-0.167
SE	2737 RHOT1	RHOT1	chr17	+	32209349	32209445	32208106	32208309	32224615	32225771	3	0	0	4	1	0	0	1	0	2	98	49	1	1	0.273	0	NA	NA			0.2	-0.064
SE	2738 RHOT1	RHOT1	chr17	+	32211115	32211238	32208106	32208309	32224615	32225771	4	0	5	4	1	0	0	2	0	2	98	49	1	1	0.333	0	1	NA			0.333	0.111
SE	2740 RHOT1	RHOT1	chr17	+	32221048	32221186	32211115	32211238	32224615	32225771	1	0	0	2	0	5	0	2	0	2	98	49	1	1	0.2	NA	0	NA			0.333	-0.233
SE	2746 MTHFD2	MTHFD2	chr2	+	74205704	74205889	74198562	74198742	74207703	74207826	7	1	0	1	0	0	0	2	0	0	98	49	1	1	0.778	1	NA	NA			1	-0.111
SE	2747 MTHFD2	MTHFD2	chr2	+	74205704	74205889	74201381	74201479	74207703	74207826	7	0	0	0	2	0	0	2	0	0	98	49	1	1	1	0.2	NA	NA			1	-0.4
SE	2753 WDTC1	WDTC1	chr1	+	27294021	27294116	27292214	27292397	27294513	27294629	19	9	7	1	2	0	0	7	0	0	98	49	1	1	0.905	0.692	1	NA			1	-0.134
SE	2754 SMG1	SMG1	chr16	-	18838013	18838232	18837252	18837443	18838356	18838466	18	2	5	1	1	0	4	5	0	0	98	49	1	1	0.9	0.5	1		1		1	-0.2
SE	2755 SMG1	SMG1	chr16	-	18879494	18879719	18877130	18877232	18882164	18882338	6	7	0	0	0	0	0	3	0	2	98	49	1	1	1	1	NA	NA			0.429	0.571
SE	2756 SMG1	SMG1	chr16	-	18889371	18889585	18885540	18885666	18890862	18890921	13	8	4	0	0	0	0	4	0	3	98	49	1	1	1	1	1	NA			0.4	0.6
SE	2758 ARL6IP1	ARL6IP1	chr16	-	18797924	18798044	18795463	18795581	18798700	18798834	29	5	22	0	1	0	1	3	0	0	98	49	1	1	1	0.714	1		1		1	-0.095
SE	2760 PMM2	PMM2	chr16	+	8811078	8811178	8806315	8806407	8811637	8811713	1	0	0	1	0	0	0	3	0	0	98	49	1	1	0.333	NA	NA	NA			1	-0.667
SE	2765 PCBP2	PCBP2	chr12	+	53465931	53465973	53464759	53464852	53467220	53467293	92	28	6	3	0	1	0	24	0	0	91	49	1	1	0.943	1	0.764	NA			1	-0.098
SE	2768 GSPT1	GSPT1	chr16	-	11887569	11887750	11886776	11886931	11891061	11891139	8	0	4	1	0	0	0	1	0	0	98	49	1	1	0.8	NA	1	NA			1	-0.1
SE	2775 EBPL	EBPL	chr13	-	49663056	49663195	49660675	49661208	49691253	49691487	4	0	4	0	1	0	0	1	0	0	98	49	1	1	1	0	1	NA			1	-0.333
SE	2784 ARHGAP27	ARHGAP27	chr17	-	45396486	45396585	45396206	45396284	45396667	45396790	56	30	10	0	0	0	4	14	0	1	98	49	1	1	1	1	1		1		0.875	0.062
SE	2785 ARHGAP27	ARHGAP27	chr17	-	45396667	45396790	45396206	45396284	45397948	45398047	0	1	1	0	2	0	0	1	0	0	98	49	1	1	NA	0.2	1	NA			1	-0.4
SE	2786 ARHGAP27	ARHGAP27	chr17	-	45396915	45397024	45396667	45396790	45397948	45398047	38	16	15	0	1	1	1	12	0	0	98	49	1	1	1	0.889	0.882		1		1	-0.076
SE	2788 ARHGAP27	ARHGAP27	chr17	-	45432215	45432349	45431620	45431752	45432751	45432916	12	6	3	0	1	0	0	4	0	0	98	49	1	1	1	0.75	1	NA			1	-0.083
SE	2789 USP25	USP25	chr21	+	15777903	15778027	15765996	15766141	15791501	15791664	36	14	8	0	0	0	0	1	0	1	98	49	1	1	1	1	1	NA			0.333	0.667
SE	2790 USP25	USP25	chr21	+	15808808	15808885	15805120	15805258	15811136	15811210	34	9	1	0	7	0	0	6	0	6	98	49	1	1	1	0.391	1	NA			0.333	0.464
SE	2791 USP25	USP25	chr21	+	15811136	15811210	15805120	15805258	15818697	15818846	21	13	2	0	1	0	0	11	0	0	98	49	1	1	1	0.867	1	NA			1	-0.044
SE	2793 USP25	USP25	chr21	+	15833347	15833548	15831400	15831629	15842397	15842540	39	4	6	1	0	0	0	11	0	0	98	49	1	1	0.951	1	1	NA			1	-0.016
SE	2797 USP25	USP25	chr21	+	15864267	15864446	15830530	15830601	15866265	15866344	8	6	0	1	0	0	0	3	0	0	98	49	1	1	0.8	1	NA	NA			1	-0.1
SE	2798 USP25	USP25	chr21	+	15864267	15864446	15842397	15842540	15866265	15866344	8	8	0	1	0	0	0	6	0	0	98	49	1	1	0.8	1	NA	NA			1	-0.1
SE	2799 USP25	USP25	chr21	+	15864267	15864446	15849776	15849872	15866265	15866344	7	6	0	1	0	0	0	3	0	0	98	49	1	1	0.778	1	NA	NA			1	-0.111
SE	2801 IFI44L	IFI44L	chr1	+	78627905	78628393	78620381	78620571	78628950	78628999	13	0	0	1	0	0	1	91	0	0	98	49	1	1	0.867	NA	NA		1		1	-0.133
SE	2803 UBN1	UBN1	chr16	+	4857989	4858076	4852878	4853166	4858567	4858663	61	20	13	2	0	0	2	11	0	1	98	49	1	1	0.938	1	1		1		0.846	0.056
SE	2805 UBN1	UBN1	chr16	+	4870843	4870972	4870515	4870634	4872883	4872934	23	14	1	3	0	0	0	8	0	0	98	49	1	1	0.793	1	1	NA			1	-0.069
SE	2807 UBN1	UBN1	chr16	+	4872883	4872934	4870515	4870634	4873030	4873073	49	24	12	0	0	0	1	8	0	1	98	49	1	1	1	1	1		1		0.8	0.1
SE	2808 UBN1	UBN1	chr16	+	4876870	4877111	4874210	4875434	4880082	4882401	5	6	0	1	0	0	0	3	0	1	98	49	1	1	1	0.75	NA	NA			0.6	0.275
SE	2809 UBN1	UBN1	chr16	+	4877384	4877474	4876870	4877111	4880082	4882401	13	16	5	0	0	0	0	5	0	1	98	49	1	1	1	1	1	NA			0.333	0.667
SE	2811 SLC15A4	SLC15A4	chr12	-	128808787	128808956	128800853	128801009	128809395	128809473	65	33	40	1	0	0	2	5	0	0	98	49	1	1	0.97	1	1		1		1	-0.01
SE	2812 SLC15A4	SLC15A4	chr12	-	128809942	128810111	128809395	128809473	128814774	128815070	33	35	17	7	6	3	0	4	0	3	98	49	1	1	0.702	0.745	0.739	NA			0.4	0.329
SE	2813 CORO7	CORO7	chr16	-	4361984	4362160	4361360	4361469	4362611	4362738	19	8	2	1	0	0	1	6	0	0	98	49	1	1	0.905	1	1		1		1	-0.032
SE	2816 USB1	USB1	chr16	+	58017333	58017439	58014272	58014326	58018971	58019055	12	3	3	0	1	0	0	5	0	0	98	49	1	1	1	0.6	1	NA			1	-0.133
SE	2817 AGO4	AGO4	chr1	+	35822861	35822982	35816881	35817047	35825312	35825494	88	35	16	1	0	0	0	10	0	0	98	49	1	1	0.978	1	1	NA			1	-0.007
SE	2818 AGO4	AGO4	chr1	+	35831426	35831574	35826747	35826835	35831811	35831931	84	26	20	0	1	2	0	12	0	0	98	49	1	1	1	0.929	0.833	NA			1	-0.079
SE	2819 AGO4	AGO4	chr1	+	35832436	35832570	35832056	35832185	35833989	35834174	49	20	20	1	0	0	2	14	0	1	98	49	1	1	0.961	1	1		1		0.875	0.049
SE	2820 AGO4	AGO4	chr1	+	35833989	35834174	35832436	35832570	35835833	35835993	45	32	14	0	0	0	2	1	0	1	98	49	1	1	1	1	1		1		0.333	0.334
SE	2821 MEFV	MEFV	chr16	-	3244472	3244588	3244253	3244286	3246524	3246547	49	38	44	1	1	0	0	19	0	0	98	49	1	1	0.961	0.95	1	NA			1	-0.03
SE	2822 MEFV	MEFV	chr16	-	3247015	3247246	3246524	3246547	3248908	3249004	131	74	74	0	0	2	3	43	0	0	98	49	1	1	1	1	0.949		1		1	-0.017
SE	2823 MEFV	MEFV	chr16	-	3248908	3249004	3247015	3247246	3249430	3249780	70	28	45	0	2	0	3	23	0	0	98	49	1	1	1	0.875	1		1		1	-0.042
SE	2826 MEFV	MEFV	chr16	-	3249430	3249780	3248908	3249004	3254157	3254790	69	30	28	1	0	0	1	16	0	0	98	49	1	1	0.972	1	1		1		1	-0.009
SE	2827 MEFV	MEFV	chr16	-	3254157	3254790	3249430	3249780	3256310	3256627	126	46	48	0	0	1	0	28	0	0	98	49	1	1	1	1	0.96	NA			1	-0.013
SE	2830 STK38L	STK38L	chr12	+	27312548	27312672	27309113	27309197	27314503	27314658	32	14	6	1	0	0	0	3	0	0	98	49	1	1	0.941	1	1	NA			1	-0.02
SE	2831 STK38L	STK38L	chr12	+	27315014	27315117	27314503	27314658	27315288	27315350	7	4	1	3	0	0	0	2	0	3	98	49	1	1	0.538	1	1	NA			0.25	0.596
SE	2833 STK38L	STK38L	chr12	+	27322142	27322234	27319327	27319423	27322327	27325957	46	19	4	1	0	0	0	10	0	0	98	49	1	1	0.958	1	1	NA			1	-0.014
SE	2836 MMP25	MMP25	chr16	+	3050253	3050546	3050008	3050144	3057032	3057209	153	63	44	1	0	0	7	19	0	1	98	49	1	1	0.987	1	1		1		0.905	0.043
SE	2837 MMP25	MMP25	chr16	+	3057309	3057394	3057032	3057209	3057530	3057613	162	58	58	0	0	0	1	33	0	1	98	49	1	1	1	1	1		1		0.943	0.028
SE	2838 MMP25	MMP25	chr16	+	3057530	3057613	3057032	3057209	3058180	3058333	41	12	11	1	0	0	0	8	0	0	98	49	1	1	0.953	1	1	NA			1	-0.016
SE	2839 ZC3H14	ZC3H14	chr14	+	88563650	88563693	88562908	88563169	88568038	88568153	1	0	0	1	0	0	1	0	0	0	92	49	1	1	0.348	NA	NA		1	NA		-0.652
SE	2849 NBAS	NBAS	chr2	-	15353552	15353710	15351991	15352081	15356302	15356416	1	0	0	1	0	0	0	0	0	1	98	49	1	1	0.333	NA	NA	NA			0	0.333
SE	2850 NBAS	NBAS	chr2	-	15488893	15489022	15478225	15478289	15504144	15504213	9	1	2	0	1	0	0	1	0	0	98	49	1	1	1	0.333	1	NA			1	-0.222
SE	2856 DENND4C	DENND4C	chr9	+	19357964	19358160	19356971	19357154	19360243	19360489	5	0	0	0	0	0	0	3	0	2	98	49	1	1	1	NA	NA	NA			0.429	0.571
SE	2859 TTC31	TTC31	chr2	+	74490024	74490127	74483321	74483410	74490243	74490473	1	0	0	1	0	0	0	1	0	0	98	49	1	1	0.333	NA	NA	NA			1	-0.667
SE	2869 NLRP12	NLRP12	chr19	-	53798242	53798413	53795858	53796029	53801226	53801397	14	9	7	1	1	0	0	2	0	0	98	49	1	1	0.875	0.818	1	NA			1	-0.102
SE	2872 NLRP12	NLRP12	chr19	-	53809586	53811288	53807494	53807665	53814907	53814988	46	17	10	0	0	0	1	12	0	1	98	49	1	1	1	1	1		1		0.857	0.072
SE	2875 AXIN1	AXIN1	chr16	-	291189	291297	289439	289607	293487	293718	2	1	0	1	2	0	1	1	0	0	98	49	1	1	0.5	0.2	NA		1		1	-0.65
SE	2882 WAC	WAC	chr10	+	28595732	28596041	28590719	28590832	28608185	28608431	38	18	6	5	0	0	0	6	0	0	98	49	1	1	0.792	1	1	NA			1	-0.069

SE	2883	WAC	WAC	chr10	+	28608185	28608431	28595732	28596041	28610698	28610821	34	14	13	1	0	0	0	4	0	0	98	49	1	1	0.944	1	1	NA	1	-0.019
SE	2884	TM2D3	TM2D3	chr15	-	101650003	101650161	101646724	101646899	101652270	101652391	10	2	0	1	0	0	4	4	0	0	98	49	1	1	0.833	1	NA	1	1	-0.084
SE	2890	SLC7A6	SLC7A6	chr16	+	68273817	68273904	68266592	68266721	68274690	68275249	3	0	0	2	0	0	0	1	0	0	98	49	1	1	0.429	NA	NA	NA	1	-0.571
SE	2893	MAGOH	MAGOH	chr1	-	53233541	53233652	53228871	53228954	53235576	53235635	9	11	1	2	2	0	0	1	0	0	98	49	1	1	0.692	0.733	1	NA	1	-0.192
SE	2939	FCAR	FCAR	chr19	+	54875329	54875365	54874092	54874323	54885234	54885525	17	5	2	0	1	0	1	6	0	0	85	49	1	1	1	0.742	1	1	1	-0.086
SE	2942	FCAR	FCAR	chr19	+	54888006	54888228	54885234	54885525	54889648	54891420	37	7	5	4	9	0	3	17	0	1	98	49	1	1	0.822	0.28	1	1	0.895	-0.247
SE	2946	USP3	USP3	chr15	+	63529013	63529166	63504510	63504830	63532646	63532707	5	1	0	11	4	1	0	0	0	2	98	49	1	1	0.185	0.111	0	NA	0	0.099
SE	2947	USP3	USP3	chr15	+	63533802	63533918	63532646	63532707	63537024	63537156	26	13	13	12	9	8	0	6	0	6	98	49	1	1	0.52	0.419	0.448	NA	0.333	0.129
SE	2949	USP3	USP3	chr15	+	63558105	63558188	63556666	63556748	63559856	63559970	54	17	19	2	0	0	0	22	0	0	98	49	1	1	0.931	1	1	NA	1	-0.023
SE	2950	MAT2B	MAT2B	chr5	+	163513554	163513669	163512001	163512196	163513841	163513994	34	22	17	0	2	0	0	6	0	0	98	49	1	1	1	0.846	1	NA	1	-0.051
SE	2952	IQGAP1	IQGAP1	chr15	+	90441505	90441684	90440501	90440615	90443393	90443478	166	76	39	0	1	0	2	36	0	0	98	49	1	1	1	0.974	1	1	1	-0.009
SE	2953	IQGAP1	IQGAP1	chr15	+	90466268	90466436	90466000	90466091	90467449	90467592	172	74	60	0	2	0	0	20	0	0	98	49	1	1	1	0.949	1	NA	1	-0.017
SE	2954	IQGAP1	IQGAP1	chr15	+	90467449	90467592	90466000	90466091	90472839	90473010	71	42	17	1	0	0	0	16	0	0	98	49	1	1	0.973	1	1	NA	1	-0.009
SE	2957	ANPEP	ANPEP	chr15	-	89790952	89791093	89790459	89790541	89792159	89792327	113	49	24	0	1	1	5	13	0	0	98	49	1	1	1	0.961	0.923	1	1	-0.039
SE	2958	ANPEP	ANPEP	chr15	-	89793034	89793126	89792451	89792562	89799259	89799315	42	26	17	0	2	0	0	8	0	0	98	49	1	1	1	0.867	1	NA	1	-0.044
SE	2959	ANPEP	ANPEP	chr15	-	89797574	89797722	89793034	89793126	89799259	89799315	131	65	23	1	0	0	0	50	0	0	98	49	1	1	0.985	1	1	NA	1	-0.005
SE	2960	ANPEP	ANPEP	chr15	-	89805077	89805217	89804490	89804617	89805320	89805463	109	37	13	2	0	0	3	14	0	1	98	49	1	1	0.965	1	1	1	0.875	0.051
SE	2962	ABHD2	ABHD2	chr15	+	89104181	89104270	89103902	89104044	89113724	89113824	3	4	1	0	3	0	0	0	0	2	98	49	1	1	1	0.4	1	NA	0	0.8
SE	2963	ABHD2	ABHD2	chr15	+	89151676	89151852	89116321	89116521	89155366	89155534	77	56	17	0	1	0	4	15	0	0	98	49	1	1	1	0.966	1	1	1	-0.011
SE	2967	RAB8B	RAB8B	chr15	+	63255506	63255584	63249644	63249705	63256504	63256594	37	14	24	2	0	0	0	9	0	0	98	49	1	1	0.902	1	1	NA	1	-0.033
SE	2978	SEC11A	SEC11A	chr15	-	84691534	84691644	84687624	84687774	84716024	84716460	20	6	20	0	2	0	2	2	0	0	98	49	1	1	1	0.6	1	1	1	-0.133
SE	2984	TM6SF1	TM6SF1	chr15	+	83115844	83115942	83112796	83112900	83119577	83119681	18	2	0	1	0	0	0	1	0	0	98	49	1	1	0.9	1	NA	NA	1	-0.05
SE	2985	TM6SF1	TM6SF1	chr15	+	83124671	83124776	83119577	83119681	83126754	83126847	13	2	6	0	2	0	0	1	0	0	98	49	1	1	1	0.333	1	NA	1	-0.222
SE	2987	BTBD1	BTBD1	chr15	-	83018706	83018853	83016428	83018225	83020674	83020762	6	2	1	2	0	0	0	6	0	0	98	49	1	1	0.6	1	1	NA	1	-0.133
SE	2994	ZFAND6	ZFAND6	chr15	+	80098415	80098578	80059567	80059809	80120327	80120498	8	5	1	5	1	2	0	3	0	0	98	49	1	1	0.444	0.714	0.2	NA	1	-0.547
SE	3018	ERAP2	ERAP2	chr5	+	96886654	96886789	96883791	96883930	96889184	96889305	9	0	0	0	0	0	0	2	2	0	98	49	1	1	1	NA	NA	0	1	0.5
SE	3022	XPO6	XPO6	chr16	-	28121669	28121762	28117317	28117462	28125688	28125848	334	215	81	0	4	0	6	79	0	0	98	49	1	1	1	0.964	1	1	1	-0.012
SE	3023	XPO6	XPO6	chr16	-	28125688	28125848	28121669	28121762	28132333	28132403	311	194	61	1	0	0	9	69	0	0	98	49	1	1	0.994	1	1	1	1	-0.002
SE	3025	XPO6	XPO6	chr16	-	28133840	28133933	28132333	28132403	28146093	28146203	134	92	25	2	1	0	2	26	0	0	98	49	1	1	0.971	0.979	1	1	1	-0.017
SE	3028	XPO6	XPO6	chr16	-	28146093	28146203	28135215	28135324	28152658	28152785	247	115	41	2	1	0	2	47	0	0	98	49	1	1	0.984	0.983	1	1	1	-0.011
SE	3029	XPO6	XPO6	chr16	-	28152658	28152785	28135215	28135324	28156073	28156527	51	33	19	1	1	0	1	13	0	0	98	49	1	1	0.962	0.943	1	1	1	-0.032
SE	3030	XPO6	XPO6	chr16	-	28177219	28177332	28175897	28176095	28180940	28181031	306	142	58	2	2	1	3	53	0	1	98	49	1	1	0.987	0.973	0.967	1	0.964	-0.006
SE	3031	XPO6	XPO6	chr16	-	28177219	28177332	28175897	28176095	28211365	28211918	18	34	6	0	0	2	0	15	0	0	98	49	1	1	1	1	0.6	NA	1	-0.133
SE	3032	XPO6	XPO6	chr16	-	28180940	28181031	28175897	28176095	28211365	28211918	50	24	9	0	0	2	0	18	0	0	98	49	1	1	1	1	0.692	NA	1	-0.103
SE	3033	XPO6	XPO6	chr16	-	28180940	28181031	28177219	28177332	28208986	28209122	292	121	54	2	0	0	3	39	0	0	98	49	1	1	0.986	1	1	1	1	-0.005
SE	3035	XPO6	XPO6	chr16	-	28208986	28209122	28177219	28177332	28211365	28211918	8	6	1	2	9	0	1	0	0	0	98	49	1	1	0.667	0.25	1	1	NA	-0.361
SE	3041	STX16	STX16	chr20	+	58659622	58659634	58651252	58652087	58667489	58667597	0	0	0	3	2	2	0	1	0	3	61	49	1	1	0	0	0	NA	0.211	-0.211
SE	3043	STX16	STX16	chr20	+	58669290	58669453	58667986	58668127	58670511	58670603	45	7	4	26	14	3	0	9	0	3	98	49	1	1	0.464	0.2	0.4	NA	0.6	-0.245
SE	3045	CSK	CSK	chr15	+	74798614	74798728	74798232	74798312	74799271	74799491	12	7	2	2	0	0	0	5	0	0	98	49	1	1	0.75	1	1	NA	1	-0.083
SE	3046	CSK	CSK	chr15	+	74798825	74798938	74798614	74798728	74799271	74799491	38	13	16	0	1	0	2	10	0	0	98	49	1	1	1	0.867	1	1	1	-0.044
SE	3048	EDC3	EDC3	chr15	-	74640465	74640619	74635408	74635626	74655732	74656068	2	2	0	1	0	0	3	1	0	0	98	49	1	1	0.5	1	NA	1	1	-0.25
SE	3062	PML	PML	chr15	+	74033155	74033414	74024856	74024927	74034477	74034530	3	0	0	2	2	0	0	2	0	0	98	49	1	1	0.429	0	NA	NA	1	-0.785
SE	3063	DDX5	DDX5	chr17	-	64500548	64500773	64498255	64500326	64502009	64502069	189	84	35	2	0	0	3	45	0	0	98	49	1	1	0.979	1	1	1	1	-0.007
SE	3069	CELF6	CELF6	chr15	-	72315844	72315927	72304745	72304794	72319612	72319946	1	0	0	0	0	0	0	0	0	1	98	49	1	1	1	NA	NA	NA	0	1
SE	3073	XPC	XPC	chr3	-	14156334	14156495	14152334	14152416	14158010	14158892	8	6	3	2	0	0	0	3	0	0	98	49	1	1	0.667	1	1	NA	1	-0.111
SE	3084	RNASEH1	RNASEH1	chr2	-	3556788	3556904	3552143	3552308	3558132	3558371	5	2	0	0	0	0	0	0	0	2	98	49	1	1	1	1	NA	NA	0	1
SE	3085	PAFAH1B1	PAFAH1B1	chr17	+	2665371	2665456	2638098	2638320	2666015	2666090	35	10	8	2	0	0	0	3	0	0	98	49	1	1	0.897	1	1	NA	1	-0.034
SE	3096	HERC1	HERC1	chr15	-	63623724	63623890	63622814	63622891	63624157	63624327	16	0	2	5	0	3	0	2	0	2	98	49	1	1	0.615	NA	0.25	NA	0.333	0.099
SE	3097	HERC1	HERC1	chr15	-	63662983	63663204	63661752	63662021	63664469	63664594	17	3	0	0	0	0	0	1	0	1	98	49	1	1	1	1	NA	NA	0.333	0.667
SE	3098	HERC1	HERC1	chr15	-	63694773	63694894	63694311	63694549	63696123	63696339	30	8	5	0	4	0	0	2	0	0	98	49	1	1	1	0.5	1	NA	1	-0.167
SE	3100	HERC1	HERC1	chr15	-	63758174	63758369	63756436	63756748	63764095	63764191	19	11	3	0	1	0	0	2	0	0	98	49	1	1	1	0.846	1	NA	1	-0.051
SE	3103	15-Sep	15-Sep	chr1	-	86868052	86868102	86862444	86863605	86880661	86880725	58	21	16	0	0	0	0	12	1	0	98	49	1	1	1	1	1	0	1	0.5
SE	3104	ADAM10	ADAM10	chr15	-	58611807	58611991	58610998	58611107	58621470	58621621	52	25	8	0	0	0	0	11	0	1	98	49	1	1	1	1	1	NA	0.846	0.154
SE	3105	ADAM10	ADAM10	chr15	-	58643885	58643978	58640776	58640960	58646054	58646204	67	50	23	1	0	0	0	18	0	0	98	49	1	1	0.971	1	1	NA	1	-0.01
SE	3109	COMMD9	COMMD9	chr11	-	36277088	36277123	36276136	36276240	36278476	36278616	4	3	0	1	0	0	0	1	0	0	84	49	1	1	0.7	1	NA	NA	1	-0.15
SE	3116	SIN3B	SIN3B	chr19	+	16862883	16862979	16862351	16862559	16863679	16863796	2	0	0	1	2	0	0	4	0	0	98	49	1	1	0.5	0	NA	NA	1	-0.75
SE	3120	MYO5A	MYO5A	chr15	-	52348817	52348826	52346360	52346461	52351253	52351481	1	2	1	0	0	0	0	0	0	1	58	49	1	1	1	1	1	NA	0	1
SE	3121	MYO5A	MYO5A	chr15	-	52407291	52407399	52405286	52405393	52408058	52408140	26	8	2	3	0	0	0	5	0	0	98	49	1	1	0.812	1	1	NA	1	-0.063
SE	3123	MYO5A	MYO5A	chr15	-	52416144	52416301	52410332	52410476	52425829	52425974	54	15	6	1	0	0	0	7	0	0	98	49	1	1	0.964	1	1	NA	1	-0.012
SE	3124	UFC1	UFC1	chr1	+	161158120	161158211	161157616	161157693	161158411	161158856	7	1	1	0	0	0	0	3	0	1	98	49	1	1	1	1	1	NA	0.6	0.4
SE	3126	ZNF207	ZNF207	chr17	+	32366664	32366757	32365329	32365487	32367771	32368014	23	9	1	4	2	1	0	0	0	2	98	49	1	1	0.742	0.692	0.333	NA	0	0.589
SE	3128	CMIP	CMIP	chr16	+	81699684	81699801	81696559	81696667	81701659	81701800	24	11	1	1	0	0	6	14	0	0	98	49	1	1	0.923	1	1	1	1	-0.026
SE	3131	TRPM7	TRPM7	chr15	-	50628148	50628249	50624165	50624300	50631416	50631489	9	12	0	0	0	0	0	4	0	1	98	49	1	1	1	1	NA	NA	0.667	0.333
SE	3133	TRPM7	TRPM7	chr15	-	50662966	50663046	50657780	50657819	50686530	50686815	20	3	0	1	0	0	2	1	0	0	98	49	1	1	0.909	1	NA	1	1	-0.045
SE	3134	USP8	USP8	chr15	+	50481480	50482065	50477275	50477499	50484274	50484361	10	8	0	1	0	0	0	5	0	0	98	49	1	1	0.833	1	NA	NA	1	-0.084
SE	3135	USP8	USP8	chr15	+	50484274	50484361	50481480	50482065	50489800	50489881	8	5	2	8	1	1	0	2	0	0	98	49	1	1	0.333	0.714	0.5	NA	1	-0.484
SE	3136	ATP8B4	ATP8B4	chr15	-	49901091	49901239	49898067	49898251	49916933	49917039	5	0	0	0	1	0	3	4	0	0	98	49	1	1	1	0	NA	1	1	-0.5
SE	3138	ATP8B4	ATP8B4	chr15	-	49996676	49996759	49987390	49987549	50002152	50002223	5	9	0	0	0	0	1	2	0	1	98	49	1	1	1	1	NA	1	0.5	0.25
SE	3139	ATP8B4	ATP8B4	chr15	-	50002152	50002223	49987390	49987549	50010844	50010917	7	8	3	2	0	0	0	2	0	2	98	49	1	1	0.636	1	1	NA	0.333	0.546
SE	3140	ATP8B4	ATP8B4	chr15	-	50018888	50018976	50010844	50010917	50038767	50038829	4	8	1	9	5	8	0	0	0	5	98	49	1	1	0.182	0.444	0.059	NA	0	0.228
SE	3142	DERL1	DERL1	chr8	-	123030604	123030716	123024985	123025050	123041969	123042423	8	8	5	0	1	1	0	2	0	0	98	49	1	1	1	0.8	0.714	NA	1	-0.162
SE	3147	PIGG	PIGG	chr4	+	521659	521941	521055	521273	523458	523913	1	2	0	2	0	0	0	1	0	0	98	49	1	1	0.2	1	NA	NA	1	-0.4
SE	3148	SQRDL	SQRDL	chr15	+	45676100	45676310	45673606	45673801	45682477	45682661	37	20	14	0	2	0	0	14	0	0	98	49	1	1	1	0.833	1	NA	1	-0.056
SE	3149	B4GALT5	B4GALT5	chr20	-	49639677	49639800	49637340	49637442	49640477	49640665	47	17	9	0	2	0	5	13	0	1	98	49	1	1	1	0.81	1	1	0.867	0.003
SE	3158	PDXDC1	PDXDC1	chr16	+	15026642	15026706	15022703	15022754	15028877	15028966	2	0	0	0	0	0	2	0	0	2	98	49	1	1	1	NA	NA	1	0	0.5
SE	3161	PATL2	PATL2	chr15	-	44667105	44667203	44666391	44666541	44668341	44668482	3	1	0	1	1	0	0	1	0	0	98	49	1	1	0.6	0.333	NA	NA	1	-0.533
SE	3183	PLAGL1	PLAGL1	chr6	-	143985134	143985174	143968906	143968978	144064467	144064599	0	1	0	1	0	0	0	1	0	0	89	49	1	1	0	1	NA	NA	1	-0.5
SE	3194	CCNDBP1	CCNDBP1	chr15	+	43185819	43185879	43185267	43185607	43189198	43189280	27	19	15	0	1	0	1	4	0	0	98	49	1	1	1	0.905	1	1	1	-0.032
SE	3195	CCNDBP1	CCNDBP1	chr15	+	43186153	43186233	43185819	43185879	43189198	43189280	48	23	24	4	5	0	0	10	0	0	98	49	1	1	0.857	0.697	1	NA	1	-0.149
SE	3196	CCNDBP1	CCNDBP1	chr15	+	43190054	43190151	43189198	43189280	43190324	43190398	34	10	15	20	2	3	0	7	0	5	98	49	1	1	0.459	0.714	0.714	NA	0.412	0.217
SE	3199	ABCD4	ABCD4	chr14	-	74288206	74288259	74287809	74287886	74288715	74288765	7	0	0	0	0	0	0	1	0	1	98	49	1	1	1	NA	NA	NA	0.333	0.667
SE	3200	ABCD4	ABCD4	chr14	-	74293153	74293248	74292747	74292869	74295147	74295198	3	0	0	1	0	0	0	1	0	0	98	49	1	1	0.6	NA	NA	NA	1	-0.4
SE	3207	NFATC3	NFATC3	chr16	+	68214328	68214432	68190767	68191775	68226349	68229259	2	0	0	5	2	0	0	0	0	1	98	49	1	1	0.167	0	NA	NA	0	0.084
SE	3208	NFATC3	NFATC3	chr16	+	68221195	68221284	68190767	68191775	68226349	68229259	1	0	0	5	2	0	0	0	0	1	98	49	1	1	0.091	0	NA	NA	0	0.045
SE	3211	SEMA7A	SEMA7A	chr15	-	74414837	74414946	74414546	74414745	74415800	74415985	4	5	0	1	0	0	0	1	0	0	98	49	1	1	0.667	1	NA	NA	1	-0.166
SE	3246	MTF2	MTF2	chr1	+	93129277	93129448	93127231	93127299	93133702	93133808	6	0	0	3	0	0	0	1	0	0	98	49	1	1	0.5	NA	NA	NA	1	-0.5
SE	3248	YTHDF3	YTHDF3	chr8	+	63172652	63172798	63169386	63169411	63175330	63175416	1	0	0	3	6	0	0	0	0	3	98	49	1	1	0.143	0	NA	NA	0	0.071
SE	3250	YTHDF3	YTHDF3	chr8	+	63175330	63175416	63169386	63169411	63186146	63187745	9	12	3	3	0	0	0	5	0	0	98	49	1	1	0.6	1	1	NA	1	-0.133
SE	3252	GANC	GANC	chr15	+	42297610	42297656	42292734	42292917	42306545	42306612	3	0	2	0	0	0	0	2	0	1	95	49	1	1	1	NA	1	NA	0.508	0.492
SE	3258	IRAK4	IRAK4	chr12	+	43771219	43771365	43768102	43768272	43772179	43772362	13	6	4	13	0	0	0	4	0	0	98	49	1	1	0.333	1	1	NA	1	-0.222
SE	3259	IRAK4	IRAK4	chr12	+	43782306	43782490	43778192	43778302	43783661	43783724	17	5	2	1	0	0	0	6	0	0	98	49	1	1	0.895	1	1	NA	1	-0.035
SE	3260	FCER1G	FCER1G	chr1	+	161217985	161218077	161215296	161215370	161218240	161218276	34	19	6	1	0	0	7	28	0	0	98	49	1	1	0.944	1	1	1	1	-0.019
SE	3261	LPCAT4	LPCAT4	chr15	-	34361399	34361532	34360110	34360209	34362195	34362321	1	2	0	1	3	0	0	1	0	0	98	49	1	1	0.333	0.25	NA	NA	1	-0.709
SE	3269	MTMR10	MTMR10	chr15	-	30942889	30943072	30938940	30942072	30947129	30947300	15	16	8	0	0	0	0	0	0	1	98	49	1	1	1	1	1	NA	0	1
SE	3270	MTMR10	MTMR10	chr15	-	30967919	30968010	30960880	30961073	30974313	30974456	43	18	12	1	2	0	0	8	0	0	98	49	1	1	0.956	0.818	1	NA	1	-0.075
SE	3271	MTMR10	MTMR10	chr15	-	30976818	30976955	30974930	30975003	30990776	30990837	49	8	3	7	0	0	0	4	0	2	98	49	1	1	0.778	1	1	NA	0.5	0.426
SE	3272	USP4	USP4	chr3	-	49278813	49278902	49277143	49278451	49280743	49280847	27	17	9	3	3	0	0	9	0	0	98	49	1	1	0.818	0.739	1	NA	1	-0.148
SE	3273	USP4	USP4	chr3	-	49283986	49284136	49280743	49280847	49284465	49284584	50	18	16	0	2	0	0	17	0	0	98	49	1	1	1	0.818	1	NA	1	-0.061
SE	3275	USP4	USP4	chr3	-	49300466	49300691	49298551	49298635	49302383	49302542	22	9	2	2	4	0	1	0	0	0	98	49	1	1	0.846	0.529	1	1	NA	-0.208
SE	3276	USP4	USP4	chr3	-	49310619	49310737	49305714	49305888	49324701	49324763	31	21	9	0	0	0	0	3	0	1	98	49	1	1	1	1	1	NA	0.6	0.4
SE	3277	USP4	USP4	chr3	-	49311513	49311654	49310619	49310737	49324701	49324763	5	12	2	8	10	1	0	1	0	1	98	49	1	1	0.238	0.375	0.5	NA	0.333	0.038
SE	3278	USP4	USP4	chr3	-	49324893	49325039	49324701	49324763	49327685	49327816	29	19	4	2	0	0	1	4	0	0	98	49	1	1	0.879	1	1	1	1	-0.04
SE	3279	USP4	USP4	chr3	-	49325718	49325845	49324893	49325039	49327685	49327816	78	61	12	2	0	0	0	17	0	0	98	49	1	1	0.951	1	1	NA	1	-0.016
SE	3282	PTTG1IP	PTTG1IP	chr21	-	44865394	44865447	44861162	44861271	44873501	44873903	13	3	9	1	0	0	2	4	0	0	98	49	1	1	0.867	1	1	1	1	-0.044
SE	3283	CNOT2	CNOT2	chr12	+	70294070	70294294	70278131	70278274	70310894	70311017	2	1	1	4	2	0	0	0	0	1	98	49	1	1	0.2	0.2	1	NA	0	0.467
SE	3286	RNASET2	RNASET2	chr6	-	166938894	166939008	166934090	166934136	166943018	166943089	273	108	110	1	0	0	4	64	0	0	98	49	1	1	0.993	1	1	1	1	-0.002
SE	3290	RAB37	RAB37	chr17	+	74745007	74745084	74744872	74744929	74745305	74747335	19	9	4	1	0	0	0	1	0	0	98	49	1	1	0.905	1	1	NA	1	-0.032
SE	3301	GABPB1	GABPB1	chr15	-	50346371	50346395	50309690	50309798	50354984	50355408	1	2	4	2	1	0	0	0	0	1	73	49	1	1	0.251	0.573	1	NA	0	0.608
SE	3306	CDK17	CDK17	chr12	-	96311051	96311177	96300303	96300360	96313320	96313454	14	7	4	0	1	0	0	5	0	0	98	49	1	1	1	0.778	1	NA	1	-0.074
SE	3308	GNL3L	GNL3L	chrX	+	54554564	54554692	54552291	54552428	54558435	54558655	28	6	1	1	0	0	0	6	0	0	98	49	1	1	0.933	1	1	NA	1	-0.022
SE	3311	VAPA	VAPA	chr18	+	9944923	9945058	9936985	9937066	9950394	9950568	4	0	0	16	0	4	0	1	0	3	98	49	1	1	0.111	NA	0	NA	0.143	-0.087
SE	3312	VAPA	VAPA	chr18	+	9950394	9950568	9936985	9937066	9954052	9960021	47	19	25	1	0	0	0	10	0	0	98	49	1	1	0.959	1	1	NA	1	-0.014
SE	3316	RAB1A	RAB1A	chr2	-	65090982	65091078	65088938	65089070	65097970	65098066	27	16	8	3	1	0	0	8	0	0	98	49	1	1	0.818	0.889	1	NA	1	-0.098
SE	3317	PYGL	PYGL	chr14	-	50912155	50912303	50911977	50912036	50913028	50913130	125	85	57	1	0	0	1	14	0	1	98	49	1	1	0.984	1	1	1	0.875	0.057
SE	3318	PYGL	PYGL	chr14	-	50913028	50913130	50911977	50912036	50915335	50915499	1	0	1	1	0	0	0	1	0	0	98	49	1	1	0.333	NA	1	NA	1	-0.334
SE	3319	PYGL	PYGL	chr14	-	50914700	50914815	50913028	50913130	50915335	50915499	119	57	34	0	0	1	1	20	0	0	98	49	1	1	1	1	0.944	1	1	-0.019
SE	3320	PYGL	PYGL	chr14	-	50915335	50915499	50914700	50914815	50915824	50915971	97	79	29	0	1	0	1	25	0	0	98	49	1	1	1	0.975	1	1	1	-0.008
SE	3323	NIPA2	NIPA2	chr15	+	22839654	22839790	22838640	22838921	22845145	22845267	0	0	0	0	1	0	0	1	0	0	98	49	1	1	NA	0	NA	NA	1	-1
SE	3324	NIPA2	NIPA2	chr15	+	22839654	22839790	22838640	22838921	22851638	22851870	0	0	0	2	4	0	0	1	0	0	98	49	1	1	0	0	NA	NA	1	-1
SE	3330	WDR11	WDR11	chr10	+	120860108	120860282	120858642	120858796	120862734	120862921	6	0	4	0	0	0	0	2	0	1	98	49	1	1	1	NA	1	NA	0.5	0.5
SE	3331	WDR11	WDR11	chr10	+	120867065	120867169	120866568	120866764	120871169	120871346	4	5	2	0	0	0	0	3	0	1	98	49	1	1	1	1	1	NA	0.6	0.4
SE	3341	UQCRH	UQCRH	chr1	+	46309896	46310044	46309100	46309127	46310154	46310316	8	0	0	4	2	0	0	1	2	0	98	49	1	1	0.5	0	NA	0	1	-0.25
SE	3343	RABAC1	RABAC1	chr19	-	41957017	41957119	41956680	41956934	41958285	41958383	19	5	13	0	1	0	0	5	0	0	98	49	1	1	1	0.714	1	NA	1	-0.095
SE	3356	CHST15	CHST15	chr10	-	124044579	124044919	124042300	124042447	124045666	124046724	136	60	56	1	0	0	0	34	0	0	98	49	1	1	0.986	1	1	NA	1	-0.005
SE	3357	TNFAIP2	TNFAIP2	chr14	+	103127004	103127629	103126326	103126692	103129739	103129854	227	74	53	0	1	0	2	36	0	0	98	49	1	1	1	0.974	1	1	1	-0.009
SE	3358	TNFAIP2	TNFAIP2	chr14	+	103129739	103129854	103126326	103126692	103130001	103130124	139	78	24	0	0	1	4	22	0	0	98	49	1	1	1	1	0.923	1	1	-0.026
SE	3359	TNFAIP2	TNFAIP2	chr14	+	103130314	103130415	103130001	103130124	103131051	103131150	222	105	48	1	0	0	9	41	0	0	98	49	1	1	0.991	1	1	1	1	-0.003
SE	3360	TNFAIP2	TNFAIP2	chr14	+	103133361	103133517	103132749	103132872	103133681	103133803	266	119	54	0	2	0	7	23	0	0	98	49	1	1	1	0.967	1	1	1	-0.011
SE	3389	KIAA0430	KIAA0430	chr16	-	15600640	15600701	15600427	15600553	15601990	15602203	21	10	8	3	3	0	0	2	0	0	98	49	1	1	0.778	0.625	1	NA	1	-0.199
SE	3390	KIAA0430	KIAA0430	chr16	-	15625371	15625797	15625015	15625173	15630331	15630504	22	11	7	1	0	0	0	5	0	0	98	49	1	1	0.917	1	1	NA	1	-0.028
SE	3391	KIAA0430	KIAA0430	chr16	-	15625371	15625800	15625015	15625173	15630331	15630504	33	16	5	1	0	0	0	7	0	0	98	49	1	1	0.943	1	1	NA	1	-0.019
SE	3394	SNHG6	SNHG6	chr8	-	66922323	66922392	66921929	66922114	66922613	66922725	22	6	1	0	0	0	0	2	0	1	98	49	1	1	1	1	1	NA	0.5	0.5
SE	3395	TECPR2	TECPR2	chr14	+	102424978	102425291	102414635	102414793	102434234	102435211	36	8	2	0	0	0	0	1	0	3	98	49	1	1	1	1	1	NA	0.143	0.857
SE	3396	TECPR2	TECPR2	chr14	+	102431795	102432128	102414635	102414793	102434234	102435211	19	5	3	0	0	0	0	1	0	3	98	49	1	1	1	1	1	NA	0.143	0.857
SE	3397	TECPR2	TECPR2	chr14	+	102452393	102452627	102450559	102450649	102465140	102465289	26	4	8	1	0	0	0	6	0	0	98	49	1	1	0.929	1	1	NA	1	-0.024
SE	3400	ZZEF1	ZZEF1	chr17	-	4056215	4056345	4054056	4054195	4057993	4058155	18	7	5	0	0	0	0	2	0	1	98	49	1	1	1	1	1	NA	0.5	0.5
SE	3401	ZZEF1	ZZEF1	chr17	-	4072607	4072756	4070683	4070924	4074149	4074351	15	2	1	0	5	0	0	2	0	1	98	49	1	1	1	0.167	1	NA	0.5	0.222
SE	3402	ZZEF1	ZZEF1	chr17	-	4109652	4109863	4105692	4105809	4112608	4112808	30	11	3	1	3	0	0	2	0	2	98	49	1	1	0.937	0.647	1	NA	0.333	0.528
SE	3405	RSU1	RSU1	chr10	-	16816972	16817084	16782033	16782084	16817314	16817454	23	0	0	0	0	1	1	0	0	0	98	49	1	1	1	NA	0	1	NA	-0.5
SE	3410	NME8	NME8	chr7	+	37850377	37850435	37850259	37850299	37850628	37850735	5	0	0	0	4	0	0	7	0	0	98	49	1	1	1	0	NA	NA	1	-0.5
SE	3453	CIITA	CIITA	chr16	+	10922166	10922250	10918439	10918526	10922406	10922490	9	0	0	1	0	0	0	1	0	0	98	49	1	1	0.818	NA	NA	NA	1	-0.182
SE	3456	SLC15A3	SLC15A3	chr11	-	60942034	60942145	60939479	60939638	60943688	60943836	32	13	10	1	0	0	2	6	0	0	98	49	1	1	0.941	1	1	1	1	-0.02
SE	3457	PAPOLA	PAPOLA	chr14	+	96555846	96555947	96552479	96552622	96556174	96556413	34	8	15	2	0	0	0	7	0	0	98	49	1	1	0.895	1	1	NA	1	-0.035
SE	3458	PAPOLA	PAPOLA	chr14	+	96555849	96555947	96552479	96552622	96556174	96556413	15	0	5	2	0	0	0	5	0	0	98	49	1	1	0.789	NA	1	NA	1	-0.105
SE	3459	GPR107	GPR107	chr9	+	130104419	130104550	130101105	130101223	130107495	130107539	5	6	8	0	2	0	0	1	0	0	98	49	1	1	1	0.6	1	NA	1	-0.133
SE	3462	CEP162	CEP162	chr6	-	84169327	84169433	84163143	84163270	84171605	84171718	2	1	0	2	0	0	0	1	0	0	98	49	1	1	0.333	1	NA	NA	1	-0.334
SE	3468	HIATL2	HIATL2	chr9	-	96972852	96972948	96958834	96958897	97013258	97013580	0	0	0	1	0	0	0	1	0	0	98	49	1	1	0	NA	NA	NA	1	-1
SE	3475	SUGP2	SUGP2	chr19	-	19001612	19001674	18995143	18995280	19004167	19004646	4	2	0	1	0	0	0	2	0	0	98	49	1	1	0.667	1	NA	NA	1	-0.166
SE	3477	CTSS	CTSS	chr1	-	150750005	150750171	150747776	150747879	150751780	150752008	370	220	161	0	0	3	1	129	0	1	98	49	1	1	1	1	0.964	1	0.985	-0.004
SE	3478	CTSS	CTSS	chr1	-	150750005	150750171	150747776	150747879	150755000	150755150	104	71	67	2	0	0	0	58	0	0	98	49	1	1	0.963	1	1	NA	1	-0.012
SE	3479	CTSS	CTSS	chr1	-	150751780	150752008	150747776	150747879	150755000	150755150	261	144	134	2	0	0	2	110	0	0	98	49	1	1	0.985	1	1	1	1	-0.005
SE	3480	CTSS	CTSS	chr1	-	150751780	150752008	150750005	150750171	150755000	150755150	527	293	226	0	0	1	3	181	0	1	98	49	1	1	1	1	0.991	1	0.989	0.003
SE	3481	CTSS	CTSS	chr1	-	150755000	150755150	150750005	150750171	150764637	150764764	1	0	1	0	0	2	0	1	0	0	98	49	1	1	1	NA	0.2	NA	1	-0.4
SE	3482	CTSS	CTSS	chr1	-	150755000	150755150	150751780	150752008	150757857	150757980	673	276	253	2	0	2	4	234	0	1	98	49	1	1	0.994	1	0.984	1	0.992	-0.003
SE	3483	CTSS	CTSS	chr1	-	150757857	150757980	150755000	150755150	150764637	150764764	820	292	228	1	0	0	3	247	0	0	98	49	1	1	0.998	1	1	1	1	-0.001

SE	3488 PHC2	PHC2	chr1	-	33331347	33331462	33330070	33330212	33332274	33332404	50	47	51	0	3	0	0	12	0	0	98	49	1	1	1	0.887	1 NA		1	-0.038
SE	3489 HIPK1	HIPK1	chr1	+	113952765	113952889	113940381	113941459	113955562	113955649	22	12	8	0	2	0	0	5	0	0	98	49	1	1	1	0.75	1 NA		1	-0.083
SE	3490 HIPK1	HIPK1	chr1	+	113954650	113954770	113952765	113952889	113955562	113955649	18	24	5	2	4	0	0	6	0	0	98	49	1	1	0.818	0.75	1 NA		1	-0.144
SE	3491 HIPK1	HIPK1	chr1	+	113956626	113956811	113955562	113955649	113958065	113958291	25	9	1	0	1	0	0	8	0	0	98	49	1	1	1	0.818	1 NA		1	-0.061
SE	3492 HIPK1	HIPK1	chr1	+	113957123	113957286	113956626	113956811	113958065	113958291	23	9	4	1	0	0	0	7	0	0	98	49	1	1	0.92	1	1 NA		1	-0.027
SE	3499 MMS19	MMS19	chr10	-	97461495	97461622	97460906	97461007	97461827	97461896	4	1	0	1	0	0	0	3	0	0	98	49	1	1	0.667	1 NA		1	1	-0.166
SE	3509 ABCC1	ABCC1	chr16	+	16124788	16124915	16121974	16122174	16125809	16125911	3	6	5	1	0	0	0	1	0	0	98	49	1	1	0.6	1	1 NA		1	-0.133
SE	3511 VIPAS39	VIPAS39	chr14	-	77429680	77429767	77429005	77429095	77433841	77433931	3	2	0	0	0	1	0	1	0	0	98	49	1	1	1	1	0 NA		1	-0.333
SE	3514 LOC283922	LOC283922	chr16	-	74334891	74334986	74332405	74332656	74336593	74336706	4	0	0	1	0	0	0	1	0	0	98	49	1	1	0.667 NA	NA	NA		1	-0.333
SE	3518 MTMR14	MTMR14	chr3	+	9677983	9678058	9677316	9677387	9683177	9683244	32	19	18	10	2	0	0	2	0	1	98	49	1	1	0.615	0.826	1 NA		0.5	0.314
SE	3519 MTMR14	MTMR14	chr3	+	9687820	9687891	9685210	9685247	9688695	9688754	15	17	6	0	1	0	1	10	0	0	98	49	1	1	1	0.895	1	1	1	-0.035
SE	3521 MTMR14	MTMR14	chr3	+	9697710	9697866	9689963	9690143	9701789	9702394	8	5	2	1	5	0	0	2	0	1	98	49	1	1	0.8	0.333	1 NA		0.5	0.211
SE	3527 CFL1	CFL1	chr11	-	65855653	65855730	65854813	65855448	65855934	65856242	296	100	133	2	0	0	9	66	0	0	98	49	1	1	0.987	1	1	1	1	-0.004
SE	3528 NAA25	NAA25	chr12	-	112054387	112054568	112053557	112053657	112060269	112060359	1	0	0	0	0	0	0	0	0	1	98	49	1	1	1 NA	NA	NA		0	1
SE	3531 DLST	DLST	chr14	+	74891055	74891167	74889896	74889952	74892833	74892986	2	2	0	2	0	0	0	1	0	0	98	49	1	1	0.333	1 NA	NA		1	-0.334
SE	3532 DLST	DLST	chr14	+	74894311	74894409	74893347	74893424	74898368	74898499	7	3	0	0	3	0	0	2	0	0	98	49	1	1	1	0.333 NA	NA		1	-0.334
SE	3533 NPC2	NPC2	chr14	-	74484414	74484587	74480701	74480779	74486328	74486436	58	22	23	0	0	1	2	26	0	0	98	49	1	1	1	1	0.92	1	1	-0.027
SE	3534 EIF2AK2	EIF2AK2	chr2	-	37122505	37122664	37119958	37120139	37126288	37126411	18	18	8	1	0	0	0	18	0	0	98	49	1	1	0.9	1	1 NA		1	-0.033
SE	3539 ZNF410	ZNF410	chr14	+	73894358	73894409	73893796	73893932	73896315	73896534	1	2	0	1	3	0	0	0	0	1	98	49	1	1	0.333	0.25 NA	NA		0	0.291
SE	3542 ZNF410	ZNF410	chr14	+	73922065	73922206	73920979	73921105	73923394	73923522	2	0	7	3	3	0	0	2	0	4	98	49	1	1	0.25	0	1 NA		0.2	0.217
SE	3546 PCNX	PCNX	chr14	+	70995740	70995925	70976941	70978648	71009633	71009724	5	4	3	5	2	0	0	2	0	0	98	49	1	1	0.333	0.5	1 NA		1	-0.389
SE	3547 PCNX	PCNX	chr14	+	71011491	71011549	71009633	71009724	71012999	71013202	38	22	2	0	0	0	0	10	0	1	98	49	1	1	1	1	1 NA		0.833	0.167
SE	3548 PCNX	PCNX	chr14	+	71012984	71013202	71011491	71011549	71019008	71019162	107	31	11	0	1	0	2	12	0	0	98	49	1	1	1	0.939	1	1	1	-0.02
SE	3549 PCNX	PCNX	chr14	+	71012999	71013202	71011491	71011549	71019008	71019162	61	19	7	0	1	0	2	7	0	0	98	49	1	1	1	0.905	1	1	1	-0.032
SE	3561 SUSD6	SUSD6	chr14	+	69708676	69709104	69704603	69704742	69710953	69715144	54	23	15	2	0	0	0	7	0	0	98	49	1	1	0.931	1	1 NA		1	-0.023
SE	3563 SLC39A9	SLC39A9	chr14	+	69453240	69453309	69442068	69442266	69454811	69454897	3	0	4	0	0	0	0	0	0	1	98	49	1	1	1 NA		1 NA		0	1
SE	3568 DIP2B	DIP2B	chr12	+	50671185	50671398	50660193	50660319	50674473	50674629	44	16	14	1	0	0	2	5	0	0	98	49	1	1	0.957	1	1	1	1	-0.014
SE	3569 DIP2B	DIP2B	chr12	+	50678678	50678876	50675328	50675448	50680671	50680763	68	34	8	2	0	0	1	8	0	0	98	49	1	1	0.944	1	1	1	1	-0.019
SE	3571 DIP2B	DIP2B	chr12	+	50723201	50723323	50721272	50721396	50724774	50724886	54	30	17	0	1	0	0	12	0	0	98	49	1	1	1	0.937	1 NA		1	-0.021
SE	3572 DIP2B	DIP2B	chr12	+	50731368	50731537	50728547	50728678	50732365	50732536	27	15	2	0	1	0	0	4	0	0	98	49	1	1	1	0.882	1 NA		1	-0.039
SE	3573 ACTN1	ACTN1	chr14	-	68878457	68878523	68877081	68877240	68878988	68879069	0	1	0	44	25	15	0	0	1	9	98	49	1	1	0	0.02	0	0	0	0.007
SE	3575 ACTN1	ACTN1	chr14	-	68882872	68883055	68882457	68882592	68884167	68884308	68	39	22	2	0	0	0	14	0	0	98	49	1	1	0.944	1	1 NA		1	-0.019
SE	3577 MIDN	MIDN	chr19	+	1251561	1251649	1249889	1250529	1251838	1251901	60	24	6	0	0	0	6	6	0	1	98	49	1	1	1	1	1	1	0.75	0.125
SE	3581 ERN1	ERN1	chr17	-	64066670	64066932	64065208	64065287	64068189	64068291	14	3	3	0	1	0	0	3	0	0	98	49	1	1	1	0.6	1 NA		1	-0.133
SE	3582 ERN1	ERN1	chr17	-	64068189	64068291	64065208	64065287	64071980	64072103	15	3	0	2	0	1	0	3	0	0	98	49	1	1	0.789	1	0 NA		1	-0.404
SE	3583 RDH11	RDH11	chr14	-	67685014	67685204	67676800	67678423	67690211	67690421	5	0	0	2	0	0	0	1	0	0	98	49	1	1	0.556 NA	NA	NA		1	-0.444
SE	3585 RDH11	RDH11	chr14	-	67692437	67692593	67691139	67691244	67692933	67693052	8	0	0	1	0	0	0	2	0	0	98	49	1	1	0.8 NA	NA	NA		1	-0.2
SE	3593 HDAC5	HDAC5	chr17	-	44085021	44085155	44084554	44084675	44086571	44086737	34	15	11	2	0	1	0	4	0	0	98	49	1	1	0.895	1	0.846 NA		1	-0.086
SE	3595 HDAC5	HDAC5	chr17	-	44117493	44117704	44110728	44110800	44123503	44123646	1	5	2	0	3	0	0	1	0	0	98	49	1	1	1	0.455	1 NA		1	-0.182
SE	3596 HDAC5	HDAC5	chr17	-	44117493	44117704	44110728	44110803	44123503	44123646	4	6	1	1	0	0	0	1	0	0	98	49	1	1	0.667	1	1 NA		1	-0.111
SE	3603 CHURC1	CHURC1	chr14	+	64926006	64926080	64923990	64924126	64932137	64935366	13	13	5	0	2	4	0	2	0	2	98	49	1	1	1	0.765	0.385 NA		0.333	0.384
SE	3604 CHURC1	CHURC1	chr14	+	64926009	64926080	64923990	64924126	64932137	64935366	25	24	10	0	2	4	0	2	0	2	98	49	1	1	1	0.857	0.556 NA		0.333	0.471
SE	3620 PPM1A	PPM1A	chr14	+	60250379	60250481	60249247	60249677	60282683	60283537	4	0	1	2	3	4	0	0	0	1	98	49	1	1	0.5	0	0.111 NA		0	0.204
SE	3621 PPM1A	PPM1A	chr14	+	60282683	60283537	60249247	60249677	60285623	60285741	20	9	9	0	1	0	0	4	0	0	98	49	1	1	1	0.818	1 NA		1	-0.061
SE	3623 MIR22HG	MIR22HG	chr17	-	1714370	1714453	1713702	1714014	1716129	1716272	11	11	0	3	7	0	0	1	0	0	98	49	1	1	0.647	0.44 NA	NA		1	-0.457
SE	3627 AP5M1	AP5M1	chr14	+	57274243	57274889	57268887	57269388	57280194	57280422	9	5	1	0	0	0	0	3	0	1	98	49	1	1	1	1	1 NA		0.6	0.4
SE	3633 SFT2D1	SFT2D1	chr6	-	166329506	166329589	166328275	166328357	166330160	166330247	15	11	3	1	0	0	1	4	0	0	98	49	1	1	0.882	1	1	1	1	-0.039
SE	3635 SFT2D1	SFT2D1	chr6	-	166331384	166331407	166330160	166330247	166342418	166342606	0	0	0	4	1	2	0	1	0	1	72	49	1	1	0	0	0 NA		0.405	-0.405
SE	3659 SOS2	SOS2	chr14	-	50145169	50145332	50139941	50140059	50145476	50145596	81	33	4	1	0	0	1	9	0	1	98	49	1	1	0.976	1	1	1	0.818	0.083
SE	3661 SOS2	SOS2	chr14	-	50174453	50174552	50161481	50161609	50180571	50180682	91	37	15	2	0	0	1	8	0	0	98	49	1	1	0.958	1	1	1	1	-0.014
SE	3662 SOS2	SOS2	chr14	-	50180571	50180682	50174453	50174552	50182462	50182606	123	54	17	1	0	0	3	10	0	0	98	49	1	1	0.984	1	1	1	1	-0.005
SE	3663 SOS2	SOS2	chr14	-	50188496	50188700	50182462	50182606	50199690	50199855	72	25	16	0	1	0	1	13	0	0	98	49	1	1	1	0.926	1	1	1	-0.025
SE	3664 VCPKMT	VCPKMT	chr14	-	50112614	50112719	50108631	50109713	50114284	50114404	21	10	0	1	0	0	0	3	0	0	98	49	1	1	0.913	1 NA	NA		1	-0.043
SE	3673 MIS18BP1	MIS18BP1	chr14	-	45206082	45206170	45204398	45204453	45210379	45210528	6	1	0	0	0	0	0	5	0	2	98	49	1	1	1	1 NA	NA		0.556	0.444
SE	3686 TPP2	TPP2	chr13	+	102635586	102635702	102633949	102634098	102636223	102636392	8	3	0	2	0	0	0	1	0	0	98	49	1	1	0.667	1 NA	NA		1	-0.166
SE	3690 G2E3	G2E3	chr14	+	30605504	30605812	30601998	30602131	30607887	30608069	1	2	7	1	0	0	0	2	0	0	98	49	1	1	0.333	1	1 NA		1	-0.222
SE	3691 TRRAP	TRRAP	chr7	+	98899678	98899767	98899421	98899499	98900623	98900720	1	1	0	1	0	0	0	3	0	0	98	49	1	1	0.333	1 NA	NA		1	-0.334
SE	3692 TRRAP	TRRAP	chr7	+	98945746	98945800	98942948	98943017	98948220	98948340	2	0	0	0	0	0	0	0	0	3	98	49	1	1	1 NA	NA	NA		0	1
SE	3693 YY1	YY1	chr14	+	100276489	100276648	100274697	100274758	100277417	100279034	25	16	5	0	0	0	0	2	0	1	98	49	1	1	1	1	1 NA		0.5	0.5
SE	3701 TCP11L2	TCP11L2	chr12	+	106335638	106335826	106323509	106323646	106336031	106336213	37	37	7	1	0	0	0	7	0	0	98	49	1	1	0.949	1	1 NA		1	-0.017
SE	3704 CDK9	CDK9	chr9	+	127787946	127788113	127787517	127787608	127788213	127788385	25	13	1	2	0	0	3	8	0	0	98	49	1	1	0.862	1	1	1	1	-0.046
SE	3707 IRF9	IRF9	chr14	+	24163377	24163508	24162965	24163149	24164062	24164134	55	65	7	1	1	0	3	26	0	0	98	49	1	1	0.965	0.97	1	1	1	-0.022
SE	3710 RNF31	RNF31	chr14	+	24159863	24159960	24158141	24158199	24160238	24160407	3	0	1	1	0	0	0	1	0	0	98	49	1	1	0.6 NA		1 NA		1	-0.2
SE	3712 TNIP1	TNIP1	chr5	-	151033607	151033799	151032286	151032383	151035001	151035067	18	14	8	0	0	1	1	5	0	0	98	49	1	1	1	1	0.8	1	1	-0.067
SE	3713 TNIP1	TNIP1	chr5	-	151045860	151045950	151042895	151042961	151049823	151049947	67	21	27	2	0	0	0	6	0	0	98	49	1	1	0.944	1	1 NA		1	-0.019
SE	3716 DCAF11	DCAF11	chr14	+	24116916	24117044	24115381	24115749	24117265	24117393	9	13	2	1	6	0	0	3	0	1	98	49	1	1	0.818	0.52	1 NA		0.6	0.179
SE	3717 DCAF11	DCAF11	chr14	+	24116971	24117044	24115282	24115749	24117265	24117393	6	7	2	1	6	0	0	3	0	1	98	49	1	1	0.75	0.368	1 NA		0.6	0.106
SE	3718 DCAF11	DCAF11	chr14	+	24117667	24117732	24117265	24117393	24118054	24118155	15	5	5	1	0	0	0	2	0	1	98	49	1	1	0.882	1	1 NA		0.5	0.461
SE	3719 DCAF11	DCAF11	chr14	+	24120837	24120991	24119710	24119896	24121364	24121517	4	9	0	0	0	0	0	4	1	0	98	49	1	1	1	1 NA		0	1	0.5
SE	3724 ADGRG3	ADGRG3	chr16	+	57679176	57679311	57678169	57678316	57679815	57679855	63	48	24	0	0	1	0	15	1	0	98	49	1	1	1	1	0.923	0	1	0.474
SE	3725 ADGRG3	ADGRG3	chr16	+	57683931	57684212	57680504	57680617	57684389	57684483	41	4	9	6	0	3	0	1	0	1	98	49	1	1	0.774	1	0.6 NA		0.333	0.458
SE	3727 ADGRG3	ADGRG3	chr16	+	57685642	57685926	57680504	57680617	57688351	57689378	41	13	13	1	0	0	4	8	0	0	98	49	1	1	0.953	1	1	1	1	-0.016
SE	3731 NDFIP1	NDFIP1	chr5	+	142137733	142137858	142135729	142135817	142140562	142140629	24	7	9	1	0	0	0	4	0	1	98	49	1	1	0.923	1	1 NA		0.667	0.307
SE	3732 HAUS4	HAUS4	chr14	-	22951554	22951689	22950313	22950410	22952327	22952459	25	12	10	7	1	2	0	16	0	1	98	49	1	1	0.641	0.857	0.714 NA		0.889	-0.152
SE	3733 HAUS4	HAUS4	chr14	-	22952540	22952683	22952327	22952459	22955099	22955176	32	20	16	0	4	0	0	8	0	0	98	49	1	1	1	0.714	1 NA		1	-0.095
SE	3734 DDX58	DDX58	chr9	-	32466289	32466441	32459370	32459514	32467761	32467932	40	17	10	1	0	0	0	23	0	0	98	49	1	1	0.952	1	1 NA		1	-0.016
SE	3735 DDX58	DDX58	chr9	-	32467761	32467932	32459370	32459514	32472974	32473065	17	13	3	2	0	0	0	7	0	0	98	49	1	1	0.81	1	1 NA		1	-0.063
SE	3736 DDX58	DDX58	chr9	-	32488733	32488887	32487944	32488203	32489343	32489451	40	34	7	1	0	0	0	14	0	0	98	49	1	1	0.952	1	1 NA		1	-0.016
SE	3737 DDX58	DDX58	chr9	-	32492390	32492538	32491300	32491420	32493760	32493942	41	26	2	6	4	0	0	8	0	1	98	49	1	1	0.774	0.765	1 NA		0.8	0.046
SE	3739 RPGRIP1	RPGRIP1	chr14	+	21343035	21343228	21334604	21334705	21345112	21345197	19	7	0	1	0	0	0	2	0	0	98	49	1	1	0.905	1 NA	NA		1	-0.047
SE	3741 ARHGEF40	ARHGEF40	chr14	+	21087319	21087463	21087000	21087105	21087967	21088098	13	8	5	0	0	1	0	4	0	1	98	49	1	1	1	1	0.714 NA		0.667	0.238
SE	3750 FANCA	FANCA	chr16	-	89752137	89752222	89749729	89749902	89758576	89758705	0	2	1	0	0	1	0	1	0	0	98	49	1	1 NA		1	0.333 NA		1	-0.334
SE	3751 KDM5B	KDM5B	chr1	-	202746141	202746323	202745857	202745982	202748944	202749139	8	5	0	2	0	0	0	1	0	0	98	49	1	1	0.667	1 NA	NA		1	-0.166
SE	3760 TINF2	TINF2	chr14	-	24241889	24241994	24241674	24241776	24242140	24242674	10	13	0	7	4	0	1	0	0	0	98	49	1	1	0.417	0.619 NA		1 NA		-0.482
SE	3765 CD47	CD47	chr3	-	108050577	108050602	108043093	108047292	108051938	108051970	3	6	3	3	6	1	0	1	0	1	74	49	1	1	0.398	0.398	0.665 NA		0.398	0.089
SE	3769 PSME2	PSME2	chr14	-	24143974	24144029	24143584	24143671	24144191	24144259	20	6	2	1	0	0	1	5	0	0	98	49	1	1	0.909	1	1	1	1	-0.03
SE	3772 PIEZO1	PIEZO1	chr16	-	88725008	88725080	88723870	88723971	88725415	88725519	3	0	0	1	0	0	0	1	0	0	98	49	1	1	0.6 NA	NA	NA		1	-0.4
SE	3777 CARS2	CARS2	chr13	-	110651033	110651100	110647100	110647239	110663450	110663518	21	15	14	2	0	0	0	5	0	0	98	49	1	1	0.84	1	1 NA		1	-0.053
SE	3782 PLEKHB2	PLEKHB2	chr2	+	131144407	131144449	131140166	131140275	131146636	131149852	3	1	0	10	1	5	0	0	0	3	91	49	1	1	0.139	0.35	0 NA		0	0.163
SE	3785 RGS14	RGS14	chr5	+	177367713	177367825	177367413	177367557	177368156	177368266	23	14	6	0	1	0	1	12	0	0	98	49	1	1	1	0.875	1	1	1	-0.042
SE	3801 THBS1	THBS1	chr15	+	39588525	39588699	39588041	39588218	39588958	39589086	18	0	1	0	0	0	2	8	0	4	98	49	1	1	1 NA		1	1	0.5	0.25
SE	3804 LINC00963	LINC00963	chr9	+	129493477	129493595	129488659	129489299	129503322	129503672	9	1	2	1	1	0	0	2	0	0	98	49	1	1	0.818	0.333	1 NA		1	-0.283
SE	3805 LINC00963	LINC00963	chr9	+	129502396	129503139	129493477	129493595	129503322	129503672	1	2	0	9	1	2	0	0	0	2	98	49	1	1	0.053	0.5	0 NA		0	0.184
SE	3807 SLC25A1	SLC25A1	chr22	-	19177941	19178041	19177119	19177204	19178132	19178240	1	0	0	2	0	0	0	1	0	0	98	49	1	1	0.2 NA	NA	NA		1	-0.8
SE	3808 BIN2	BIN2	chr12	-	51295795	51295878	51291590	51292344	51297088	51297164	175	64	47	1	0	0	3	28	0	0	98	49	1	1	0.989	1	1	1	1	-0.004
SE	3810 BIN2	BIN2	chr12	-	51313822	51313903	51303086	51303141	51324021	51324198	60	24	22	1	0	0	0	5	0	0	98	49	1	1	0.968	1	1 NA		1	-0.011
SE	3812 MYO18A	MYO18A	chr17	-	29086437	29086577	29082315	29082438	29086935	29087121	0	2	0	0	2	0	0	3	0	0	98	49	1	1 NA		0.333 NA	NA		1	-0.667
SE	3823 ANP32A	ANP32A	chr15	-	68782955	68783053	68780409	68780473	68784396	68784595	42	11	11	0	1	0	2	5	0	0	98	49	1	1	1	0.846	1	1	1	-0.051
SE	3825 PTPRE	PTPRE	chr10	+	128066074	128066194	128063082	128063180	128068122	128068286	182	99	13	1	2	0	4	51	0	0	98	49	1	1	0.989	0.961	1	1	1	-0.017
SE	3826 PTPRE	PTPRE	chr10	+	128076602	128076728	128073336	128073471	128077616	128077783	147	62	23	0	1	0	3	39	0	0	98	49	1	1	1	0.969	1	1	1	-0.01
SE	3827 PTPRE	PTPRE	chr10	+	128079559	128079695	128077616	128077783	128082831	128085900	77	47	14	1	0	0	2	19	0	0	98	49	1	1	0.975	1	1	1	1	-0.008
SE	3846 C17orf85	C17orf85	chr17	-	3814321	3814483	3806750	3813279	3816115	3816270	9	3	1	0	0	0	1	1	0	1	98	49	1	1	1	1	1	1	0.333	0.334
SE	3852 MYCBP2	MYCBP2	chr13	-	77095357	77095602	77093164	77093332	77096311	77096481	11	2	0	0	0	0	0	1	0	1	98	49	1	1	1	1 NA	NA		0.333	0.667
SE	3853 MYCBP2	MYCBP2	chr13	-	77126317	77126542	77125335	77125468	77139195	77139336	5	1	0	2	2	0	0	2	1	0	98	49	1	1	0.556	0.2 NA		0	1	-0.122
SE	3854 MYCBP2	MYCBP2	chr13	-	77233155	77233263	77225434	77225554	77243058	77243160	20	6	3	1	0	0	0	2	0	0	98	49	1	1	0.909	1	1 NA		1	-0.03
SE	3856 ITCH	ITCH	chr20	+	34393790	34393881	34369393	34369470	34408650	34408792	31	16	4	1	0	0	0	6	0	0	98	49	1	1	0.939	1	1 NA		1	-0.02
SE	3857 ITCH	ITCH	chr20	+	34408650	34408792	34393790	34393881	34412514	34412639	49	38	20	0	0	0	0	5	0	1	98	49	1	1	1	1	1 NA		0.714	0.286
SE	3858 ITCH	ITCH	chr20	+	34417116	34417239	34413741	34413879	34424479	34424525	1	0	0	30	36	4	0	3	0	3	98	49	1	1	0.016	0	0 NA		0.333	-0.328
SE	3859 ITCH	ITCH	chr20	+	34424479	34424525	34413741	34413879	34438473	34438631	67	44	15	4	0	0	0	5	0	0	95	49	1	1	0.896	1	1 NA		1	-0.035
SE	3860 ITCH	ITCH	chr20	+	34457389	34457474	34449410	34449480	34462092	34462221	33	28	9	0	0	0	0	3	0	1	98	49	1	1	1	1	1 NA		0.6	0.4
SE	3861 COMMD6	COMMD6	chr13	-	75527812	75527851	75525213	75526639	75530113	75530266	0	2	0	13	2	2	0	0	1	2	88	49	1	1	0	0.358	0	0	0	0.119
SE	3865 SYNJ1	SYNJ1	chr21	-	32699837	32700105	32695056	32695282	32701960	32702047	9	2	1	3	0	0	0	1	0	1	98	49	1	1	0.6	1	1 NA		0.333	0.534
SE	3866 PSAP	PSAP	chr10	-	71819030	71819111	71818616	71818724	71819464	71819622	436	98	134	1	0	0	15	181	0	0	98	49	1	1	0.995	1	1	1	1	-0.002
SE	3867 PSAP	PSAP	chr10	-	71825836	71825893	71821875	71822007	71828013	71828157	520	148	225	0	0	1	10	255	0	0	98	49	1	1	1	1	0.991	1	1	-0.003
SE	3876 PPP1R16B	PPP1R16B	chr20	+	38900580	38900684	38895564	38895710	38902667	38902792	1	0	0	2	0	0	0	2	0	0	98	49	1	1	0.2 NA	NA	NA		1	-0.8
SE	3878 PSMB9	PSMB9	chr6	+	32857262	32857394	32856137	32856205	32858004	32858134	37	41	13	1	0	0	0	23	0	0	98	49	1	1	0.949	1	1 NA		1	-0.017
SE	3879 PSMB9	PSMB9	chr6	+	32858363	32858505	32858004	32858134	32859404	32859851	36	36	49	0	3	0	0	16	0	0	98	49	1	1	1	0.857	1 NA		1	-0.048
SE	3889 ST3GAL1	ST3GAL1	chr8	-	133475718	133476600	133465893	133466090	133499134	133499189	11	7	0	0	0	0	1	1	0	3	98	49	1	1	1	1 NA		1	0.143	0.428
SE	3893 MCM9	MCM9	chr6	-	118931419	118931738	118923810	118924127	118934890	118935162	4	4	0	1	0	0	3	1	0	0	98	49	1	1	0.667	1 NA		1	1	-0.166
SE	3895 INTS6	INTS6	chr13	-	51421111	51421334	51395299	51395483	51423029	51423090	3	1	0	0	0	0	0	0	0	2	98	49	1	1	1	1 NA	NA		0	1
SE	3911 ATF6	ATF6	chr1	+	161791407	161791537	161783989	161784096	161792123	161792327	97	30	3	2	0	0	0	16	0	0	98	49	1	1	0.96	1	1 NA		1	-0.013
SE	3912 ATF6	ATF6	chr1	+	161802051	161802272	161792123	161792327	161819632	161819818	47	7	7	2	0	0	0	8	0	0	98	49	1	1	0.922	1	1 NA		1	-0.026
SE	3913 ATF6	ATF6	chr1	+	161860206	161860277	161853223	161853323	161863197	161863312	53	15	4	2	0	0	0	17	0	0	98	49	1	1	0.93	1	1 NA		1	-0.023
SE	3916 EPG5	EPG5	chr18	-	45879012	45879214	45878375	45878448	45880074	45880223	5	1	8	0	1	0	0	3	0	0	98	49	1	1	1	0.333	1 NA		1	-0.222
SE	3917 EPG5	EPG5	chr18	-	45880074	45880223	45879012	45879214	45882273	45882487	2	4	3	0	0	1	0	4	0	0	98	49	1	1	1	1	0.6 NA		1	-0.133
SE	3919 EPG5	EPG5	chr18	-	45889797	45889940	45887750	45887907	45899403	45899566	10	3	0	1	0	0	0	1	0	0	98	49	1	1	0.833	1 NA	NA		1	-0.084
SE	3920 EPG5	EPG5	chr18	-	45916008	45916206	45915510	45915621	45916437	45916582	4	2	0	1	0	0	0	1	0	0	98	49	1	1	0.667	1 NA	NA		1	-0.166
SE	3922 SPG11	SPG11	chr15	-	44572682	44572820	44570524	44570658	44583813	44584558	16	0	2	0	1	0	0	10	0	0	98	49	1	1	1	0	1 NA		1	-0.333
SE	3923 SPG11	SPG11	chr15	-	44589251	44589414	44585635	44585850	44592330	44592438	17	4	7	1	0	0	0	2	0	0	98	49	1	1	0.895	1	1 NA		1	-0.035
SE	3924 SPG11	SPG11	chr15	-	44598630	44598836	44598264	44598373	44600466	44600632	16	3	0	0	1	0	0	1	0	0	98	49	1	1	1	0.6 NA	NA		1	-0.2
SE	3925 SPG11	SPG11	chr15	-	44606024	44606091	44600466	44600632	44608443	44608605	26	14	1	6	0	1	0	3	0	2	98	49	1	1	0.684	1	0.333 NA		0.429	0.243
SE	3926 SPG11	SPG11	chr15	-	44613429	44613536	44610839	44610985	44615362	44615566	36	11	5	3	2	0	0	13	0	0	98	49	1	1	0.857	0.733	1 NA		1	-0.137
SE	3932 DAPP1	DAPP1	chr4	+	99863769	99863855	99863009	99863072	99866033	99866121	52	18	16	1	0	0	0	14	0	0	98	49	1	1	0.963	1	1 NA		1	-0.012
SE	3933 DAPP1	DAPP1	chr4	+	99866556	99866618	99866033	99866121	99868116	99870189	6	0	0	31	6	4	0	3	0	6	98	49	1	1	0.088	0	0 NA		0.2	-0.171
SE	3935 TPT1	TPT1	chr13	-	45340711	45340785	45339993	45340184	45341041	45341284	288	116	51	1	2	0	1	67	0	0	98	49	1	1	0.993	0.967	1	1	1	-0.013
SE	3938 DECR1	DECR1	chr8	+	90036840	90036940	90020908	90021056	90042727	90042800	36	16	9	1	0	0	0	6	0	0	98	49	1	1	0.947	1	1 NA		1	-0.018
SE	3942 SREBF1	SREBF1	chr17	-	17814614	17814747	17814244	17814410	17814834	17814944	3	1	0	2	0	0	0	0	2	0	98	49	1	1	0.429	1 NA		0 NA		0.715
SE	3943 SREBF1	SREBF1	chr17	-	17817255	17817457	17816957	17817136	17817695	17817916	7	1	1	0	1	0	0	1	0	0	98	49	1	1	1	0.333	1 NA		1	-0.222
SE	3944 SREBF1	SREBF1	chr17	-	17819012	17819234	17818259	17818374	17819319	17819454	6	2	2	0	0	0	0	0	2	0	98	49	1	1	1	1	1	0 NA		1
SE	3965 ARMC2	ARMC2	chr6	+	108868823	108868995	108858198	108858271	108876142	108876350	5	6	0	0	0	0	0	1	0	1	98	49	1	1	1	1 NA	NA		0.333	0.667
SE	3975 SUPT20H	SUPT20H	chr13	-	37033448	37033588	37031738	37031895	37040404	37040458	20	3	13	1	0	0	0	4	0	0	98	49	1	1	0.909	1	1 NA		1	-0.03
SE	3976 SUPT20H	SUPT20H	chr13	-	37048563	37048599	37047877	37047936	37051487	37051583	19	2	10	4	0	1	0	0	0	1	85	49	1	1	0.732	1	0.852 NA		0	0.861
SE	3993 PARVG	PARVG	chr22	+	44183317	44183408	44181741	44181917	44185807	44185872	97	32	18	0	2	0	7	24	0	0	98	49	1	1	1	0.889	1	1	1	-0.037
SE	3995 CIC	CIC	chr19	+	42289847	42289951	42289180	42289406	42290232	42291466	13	7	2	0	1	0	3	3	0	0	98	49	1	1	1	0.778	1	1	1	-0.074
SE	3996 CIC	CIC	chr19	+	42291557	42291745	42290232	42291466	42292085	42292207	4	0	1	1	0	0	0	1	0	2	98	49	1	1	0.667 NA		1 NA		0.2	0.633
SE	3997 HMGB1	HMGB1	chr13	-	30463206	30463352	30462537	30462712	30463530	30463694	0	0	0	0	0	1	1	0	0	0	98	49	1	1 NA	NA		0	1 NA		-1
SE	4000 RPL24	RPL24	chr3	-	101682428	101682492	101681089	101681215	101682770	101682907	46	7	10	4	0	0	0	9	0	0	98	49	1	1	0.852	1	1 NA		1	-0.049
SE	4001 PAN3	PAN3	chr13	+	28177864	28177935	28176492	28176559	28220230	28220378	38	26	9	0	0	0	0	12	0	1	98	49	1	1	1	1	1 NA		0.857	0.143
SE	4002 PAN3	PAN3	chr13	+	28197184	28197346	28177864	28177935	28220230	28220378	19	10	0	12	1	0	0	1	0	5	98	49	1	1	0.442	0.833 NA	NA		0.091	0.546
SE	4003 PAN3	PAN3	chr13	+	28220230	28220378	28177864	28177935	28256291	28256539	26	1	1	1	0	0	0	6	0	0	98	49	1	1	0.929	1	1 NA		1	-0.024
SE	4004 PAN3	PAN3	chr13	+	28271980	28272071	28270700	28270866	28277236	28277376	12	5	8	0	0	0	0	0	0	1	98	49	1	1	1	1	1 NA		0	1
SE	4008 NUPL1	NUPL1	chr13	+	25312882	25313032	25309246	25309282	25313613	25313751	19	8	4	0	0	0	0	3	0	1	98	49	1	1	1	1	1 NA		0.6	0.4
SE	4016 SPATA13	SPATA13	chr13	+	24278613	24278821	24270686	24270912	24284134	24284271	3	0	0	4	4	0	0	1	1	3	98	49	1	1	0.273	0 NA		0	0.143	0.065
SE	4017 SPATA13	SPATA13	chr13	+	24286764	24286950	24286213	24286393	24288998	24289178	9	5	6	1	0	1	0	5	0	2	98	49	1	1	0.818	1	0.75 NA		0.556	0.3
SE	4035 CNOT7	CNOT7	chr8	-	17234715	17234860	17231512	17232537	17237211	17237373	17	19	3	1	0	0	2	1	0	0	98	49	1	1	0.895	1	1	1	1	-0.035

SE	4053	FBRSL1	FBRSL1	chr12	+	132581740	132581824	132581438	132581516	132582061	132582266	1	0	1	1	0	0	1	0	0	0	98	49	1	1	0.333	NA	1	1	NA	-0.334
SE	4070	RRP12	RRP12	chr10	-	97367040	97367132	97366741	97366909	97369424	97369582	14	6	4	0	3	0	0	1	0	0	98	49	1	1	1	0.5	1	NA	1	-0.167
SE	4071	RRP12	RRP12	chr10	-	97372735	97372803	97372072	97372166	97373045	97373200	15	11	2	1	1	0	0	4	0	2	98	49	1	1	0.882	0.846	1	NA	0.5	0.409
SE	4072	RRP12	RRP12	chr10	-	97379627	97379770	97379292	97379414	97380798	97380913	37	8	1	1	0	0	6	8	0	0	98	49	1	1	0.949	1	1	1	1	-0.017
SE	4073	RRP12	RRP12	chr10	-	97381714	97381826	97381385	97381483	97385165	97385257	26	8	0	2	0	0	0	7	0	0	98	49	1	1	0.867	1	NA	NA	1	-0.067
SE	4074	RRP12	RRP12	chr10	-	97390422	97390539	97388488	97388624	97390738	97390844	53	20	5	0	0	0	7	7	0	1	98	49	1	1	1	1	1	1	0.778	0.111
SE	4076	RRP12	RRP12	chr10	-	97390738	97390844	97390422	97390539	97393683	97393760	86	26	3	1	0	0	8	9	0	0	98	49	1	1	0.977	1	1	1	1	-0.008
SE	4082	NCOR2	NCOR2	chr12	-	124374412	124374463	124372021	124372610	124378236	124378384	3	0	0	1	0	1	0	2	0	0	98	49	1	1	0.6	NA	0	NA	1	-0.7
SE	4083	NCOR2	NCOR2	chr12	-	124385744	124385887	124378236	124378384	124398118	124398181	1	2	3	1	0	0	0	2	0	0	98	49	1	1	0.333	1	1	NA	1	-0.222
SE	4084	NCOR2	NCOR2	chr12	-	124457105	124457162	124449814	124449867	124466172	124466286	6	2	0	0	4	0	0	0	0	2	98	49	1	1	1	0.2	NA	NA	0	0.6
SE	4085	NCOR2	NCOR2	chr12	-	124535564	124535611	124495146	124495368	124567307	124567464	3	2	0	1	0	0	0	3	0	0	96	49	1	1	0.605	1	NA	NA	1	-0.198
SE	4094	OGFOD2	OGFOD2	chr12	+	122976653	122976767	122975810	122975867	122976870	122976970	0	0	0	0	0	1	0	1	0	0	98	49	1	1	NA	NA	0	NA	1	-1
SE	4101	ZCCHC8	ZCCHC8	chr12	-	122482634	122482695	122481944	122482087	122489385	122489463	2	5	2	1	0	0	0	1	0	0	98	49	1	1	0.5	1	1	NA	1	-0.167
SE	4108	MLXIP	MLXIP	chr12	+	122130843	122130933	122129940	122130112	122132291	122132383	10	4	2	0	0	0	0	1	0	1	98	49	1	1	1	1	1	NA	0.333	0.667
SE	4112	FAM219B	FAM219B	chr15	-	74906277	74906365	74905153	74905231	74906586	74907121	1	0	0	1	0	0	0	0	0	1	98	49	1	1	0.333	NA	NA	NA	0	0.333
SE	4116	CAMKK2	CAMKK2	chr12	-	121248605	121248734	121245139	121245240	121249786	121249874	8	2	0	5	11	0	0	2	1	0	98	49	1	1	0.444	0.083	NA	0	1	-0.236
SE	4117	CAMKK2	CAMKK2	chr12	-	121249960	121250034	121249786	121249874	121252660	121252714	10	16	5	1	0	0	1	14	0	0	98	49	1	1	0.833	1	1	1	1	-0.056
SE	4120	P2RX4	P2RX4	chr12	+	121222729	121222767	121222093	121222166	121222946	121223043	1	0	0	3	0	0	0	0	0	5	87	49	1	1	0.158	NA	NA	NA	0	0.158
SE	4121	CAMK2D	CAMK2D	chr4	-	113455725	113455821	113451031	113454515	113457334	113457563	2	0	0	2	0	0	0	1	0	0	98	49	1	1	0.333	NA	NA	NA	1	-0.667
SE	4130	HIRA	HIRA	chr22	-	19361726	19361931	19361236	19361341	19375630	19375792	14	8	0	0	1	0	0	4	0	0	98	49	1	1	1	0.8	NA	NA	1	-0.1
SE	4131	HIRA	HIRA	chr22	-	19387710	19387816	19385520	19385736	19388483	19388554	15	6	2	0	0	0	0	8	0	2	98	49	1	1	1	1	1	NA	0.667	0.333
SE	4132	HIRA	HIRA	chr22	-	19388483	19388554	19385520	19385736	19392100	19392214	18	12	2	0	0	0	0	5	0	1	98	49	1	1	1	1	1	NA	0.714	0.286
SE	4138	ZNRF2	ZNRF2	chr7	+	30323641	30323737	30284306	30285826	30355727	30355833	17	6	3	0	1	0	0	2	0	0	98	49	1	1	1	0.75	1	NA	1	-0.083
SE	4139	MED13L	MED13L	chr12	-	115968939	115969097	115966081	115966243	115970593	115970770	40	20	11	0	0	0	6	8	0	1	98	49	1	1	1	1	1	1	0.8	0.1
SE	4140	MED13L	MED13L	chr12	-	115986265	115986489	115984179	115984372	115987108	115987288	20	15	8	0	3	0	0	4	0	0	98	49	1	1	1	0.714	1	NA	1	-0.095
SE	4141	MED13L	MED13L	chr12	-	116096668	116096752	116022455	116022601	116111427	116111512	100	56	21	0	1	0	0	23	0	0	98	49	1	1	1	0.966	1	NA	1	-0.011
SE	4142	MED13L	MED13L	chr12	-	116111427	116111512	116096668	116096752	116237467	116237705	130	65	32	2	1	0	0	17	0	0	98	49	1	1	0.97	0.97	1	NA	1	-0.02
SE	4143	MED13L	MED13L	chr12	-	116237467	116237705	116111427	116111512	116277059	116277186	138	53	19	1	0	0	0	18	0	0	98	49	1	1	0.986	1	1	NA	1	-0.005
SE	4150	PSTPIP1	PSTPIP1	chr15	+	77030501	77030581	77029528	77029574	77031179	77031278	41	18	9	0	3	0	0	3	0	0	98	49	1	1	1	0.75	1	NA	1	-0.083
SE	4152	PSTPIP1	PSTPIP1	chr15	+	77032861	77032952	77032297	77032394	77035507	77035563	26	15	9	0	1	0	4	6	0	0	98	49	1	1	1	0.882	1	1	1	-0.039
SE	4153	OAS2	OAS2	chr12	+	112987037	112987308	112978468	112978785	112995295	112995474	5	1	0	0	0	0	6	42	0	3	98	49	1	1	1	1	NA	1	0.875	0.062
SE	4154	OAS2	OAS2	chr12	+	113004933	113005222	113002931	113003102	113006412	113006600	10	1	0	0	0	0	2	43	0	1	98	49	1	1	1	1	NA	1	0.956	0.022
SE	4155	OAS3	OAS3	chr12	+	112941569	112941852	112938432	112938707	112944475	112944651	3	5	0	0	0	0	7	34	0	1	98	49	1	1	1	1	NA	1	0.944	0.028
SE	4156	NLRP1	NLRP1	chr17	-	5515472	5515517	5501398	5501872	5517745	5517887	14	10	6	19	9	3	0	7	0	6	94	49	1	1	0.278	0.367	0.51	NA	0.378	0.007
SE	4157	NLRP1	NLRP1	chr17	-	5517745	5517887	5515472	5515517	5530480	5530704	13	10	3	1	0	0	0	7	1	0	98	49	1	1	0.867	1	1	0	1	0.456
SE	4159	NLRP1	NLRP1	chr17	-	5521523	5521786	5517745	5517887	5530480	5530704	22	22	0	1	0	0	0	9	0	0	98	49	1	1	0.917	1	NA	NA	1	-0.041
SE	4161	NLRP1	NLRP1	chr17	-	5533303	5533384	5532821	5532984	5536850	5536940	31	9	7	5	2	0	0	7	0	0	98	49	1	1	0.756	0.692	1	NA	1	-0.184
SE	4162	NLRP1	NLRP1	chr17	-	5533896	5533988	5533291	5533384	5536850	5536940	85	31	17	1	0	0	2	27	0	0	98	49	1	1	0.977	1	1	1	1	-0.008
SE	4165	OAS1	OAS1	chr12	+	112908535	112908824	112906933	112907219	112911050	112911235	7	5	0	0	1	0	1	25	0	0	98	49	1	1	1	0.714	NA	1	1	-0.143
SE	4166	OAS1	OAS1	chr12	+	112911050	112911235	112906933	112907219	112916508	112916738	7	5	0	0	0	0	0	15	0	2	98	49	1	1	1	1	NA	NA	0.789	0.211
SE	4167	OAS1	OAS1	chr12	+	112917546	112917700	112916508	112916738	112919486	112919907	4	4	0	0	0	0	0	12	0	1	98	49	1	1	1	1	NA	NA	0.857	0.143
SE	4170	POLDIP3	POLDIP3	chr22	-	42591954	42592084	42587505	42587572	42595536	42595603	9	6	7	0	0	0	0	0	0	2	98	49	1	1	1	1	1	NA	0	1
SE	4179	GIT2	GIT2	chr12	-	109945259	109945349	109939164	109939247	109947255	109947504	2	1	0	1	0	1	0	0	0	1	98	49	1	1	0.5	1	0	NA	0	0.5
SE	4180	GIT2	GIT2	chr12	-	109947255	109947504	109939164	109939247	109953091	109953234	1	3	1	0	0	3	0	2	0	0	98	49	1	1	1	1	0.143	NA	1	-0.286
SE	4186	GIT2	GIT2	chr12	-	109980951	109981046	109967457	109967503	109983372	109983503	81	22	28	0	1	0	2	20	0	0	98	49	1	1	1	0.917	1	1	1	-0.028
SE	4187	GIT2	GIT2	chr12	-	109991626	109991760	109989689	109989802	109996172	109996389	30	24	5	0	0	1	1	5	0	0	98	49	1	1	1	1	0.714	1	1	-0.095
SE	4191	DEPDC5	DEPDC5	chr22	+	31810520	31810641	31809610	31809647	31814991	31815212	4	0	0	0	0	0	0	1	0	1	98	49	1	1	1	NA	NA	NA	0.333	0.667
SE	4196	YIPF1	YIPF1	chr1	-	53860055	53860153	53855000	53855051	53866199	53866382	11	5	6	0	0	0	0	1	0	1	98	49	1	1	1	1	1	NA	0.333	0.667
SE	4197	YIPF1	YIPF1	chr1	-	53866199	53866382	53860055	53860153	53866757	53866924	19	4	8	1	0	0	0	1	0	0	98	49	1	1	0.905	1	1	NA	1	-0.032
SE	4198	YIPF1	YIPF1	chr1	-	53866199	53866382	53860055	53860153	53871371	53871488	9	2	3	2	3	0	0	2	0	0	98	49	1	1	0.692	0.25	1	NA	1	-0.353
SE	4199	YIPF1	YIPF1	chr1	-	53866757	53866924	53860055	53860153	53871371	53871488	13	2	7	2	3	0	0	4	0	0	98	49	1	1	0.765	0.25	1	NA	1	-0.328
SE	4200	YIPF1	YIPF1	chr1	-	53866757	53866924	53866199	53866382	53871371	53871488	22	4	12	0	0	0	0	4	0	1	98	49	1	1	1	1	1	NA	0.667	0.333
SE	4201	YIPF1	YIPF1	chr1	-	53888906	53888986	53883112	53883276	53889712	53889814	1	6	0	1	0	0	1	1	0	0	98	49	1	1	0.333	1	NA	1	1	-0.334
SE	4210	SART3	SART3	chr12	-	108543027	108543152	108538933	108539089	108544426	108544478	5	0	8	2	0	0	0	1	0	0	98	49	1	1	0.556	NA	1	NA	1	-0.222
SE	4211	SART3	SART3	chr12	-	108544426	108544478	108543027	108543152	108545138	108545323	6	6	1	4	0	0	1	0	0	0	98	49	1	1	0.429	1	1	1	NA	-0.19
SE	4212	EIF3L	EIF3L	chr22	+	37858678	37858740	37855564	37855644	37862968	37863038	20	6	3	0	1	0	0	4	0	0	98	49	1	1	1	0.75	1	NA	1	-0.083
SE	4213	EIF3L	EIF3L	chr22	+	37886764	37886845	37877673	37878171	37888425	37888782	39	4	4	1	0	0	0	6	0	0	98	49	1	1	0.951	1	1	NA	1	-0.016
SE	4220	DEF8	DEF8	chr16	+	89961736	89961864	89960930	89961095	89963362	89963443	28	16	1	1	0	0	0	2	0	0	98	49	1	1	0.933	1	1	NA	1	-0.022
SE	4226	MARK3	MARK3	chr14	+	103448918	103448967	103428386	103428440	103451917	103451983	31	17	4	3	0	0	0	9	0	0	98	49	1	1	0.838	1	1	NA	1	-0.054
SE	4227	MARK3	MARK3	chr14	+	103465556	103465793	103462404	103462461	103465971	103466091	24	11	4	1	1	0	0	4	0	0	98	49	1	1	0.923	0.846	1	NA	1	-0.077
SE	4228	MARK3	MARK3	chr14	+	103465971	103466091	103465556	103465793	103466342	103466442	26	6	3	0	0	0	0	1	0	1	98	49	1	1	1	1	1	NA	0.333	0.667
SE	4229	MARK3	MARK3	chr14	+	103474992	103475210	103468032	103468186	103480386	103480490	25	5	5	1	0	0	0	7	0	0	98	49	1	1	0.926	1	1	NA	1	-0.025
SE	4230	MARK3	MARK3	chr14	+	103491776	103492034	103480386	103480490	103502881	103503829	12	11	5	0	0	0	0	0	0	1	98	49	1	1	1	1	1	NA	0	1
SE	4235	UHRF1BP1L	UHRF1BP1L	chr12	-	100058028	100059533	100057570	100057746	100059849	100060043	31	15	9	2	0	0	2	4	0	0	98	49	1	1	0.886	1	1	1	1	-0.038
SE	4236	UHRF1BP1L	UHRF1BP1L	chr12	-	100084475	100084658	100083015	100083134	100086274	100086358	37	13	1	6	1	0	0	4	0	0	98	49	1	1	0.755	0.867	1	NA	1	-0.126
SE	4237	UHRF1BP1L	UHRF1BP1L	chr12	-	100088914	100089095	100086274	100086358	100095666	100095837	48	20	9	0	0	0	0	11	0	3	98	49	1	1	1	1	1	NA	0.647	0.353
SE	4239	C9orf89	C9orf89	chr9	+	93111878	93111938	93110567	93110721	93112217	93112289	8	5	5	3	1	2	0	6	0	0	98	49	1	1	0.571	0.714	0.556	NA	1	-0.386
SE	4240	TMEM86B	TMEM86B	chr19	-	55228190	55228437	55226633	55227563	55228690	55229264	0	0	2	0	0	0	0	1	1	0	98	49	1	1	NA	NA	1	0	1	0.5
SE	4261	KATNBL1	KATNBL1	chr15	-	34146760	34146850	34145397	34145491	34147199	34147289	13	1	5	0	2	0	0	2	0	0	98	49	1	1	1	0.2	1	NA	1	-0.267
SE	4264	MTMR1	MTMR1	chrX	+	150762564	150762737	150755688	150755865	150764511	150765103	4	4	0	0	0	0	0	1	1	0	98	49	1	1	1	1	NA	0	1	0.5
SE	4268	LTA4H	LTA4H	chr12	-	96021084	96021137	96019167	96019240	96022146	96022251	60	15	8	1	0	0	1	16	0	0	98	49	1	1	0.968	1	1	1	1	-0.011
SE	4271	RSL24D1	RSL24D1	chr15	-	55190974	55191047	55185361	55185425	55192719	55192833	22	10	2	0	2	0	0	5	0	0	98	49	1	1	1	0.714	1	NA	1	-0.095
SE	4273	TOP3A	TOP3A	chr17	-	18290841	18291027	18290556	18290686	18294702	18294785	5	0	0	0	1	0	0	4	0	0	98	49	1	1	1	0	NA	NA	1	-0.5
SE	4274	TOP3A	TOP3A	chr17	-	18292644	18292852	18290841	18291027	18294702	18294785	19	6	2	1	0	0	0	1	0	1	98	49	1	1	0.905	1	1	NA	0.333	0.635
SE	4277	IFI35	IFI35	chr17	+	43013775	43013882	43013475	43013662	43014107	43014459	10	6	0	0	1	0	0	5	0	0	98	49	1	1	1	0.75	NA	NA	1	-0.125
SE	4282	PLXNC1	PLXNC1	chr12	+	94181445	94181580	94169152	94169293	94186372	94186473	118	70	70	0	2	0	1	22	0	0	98	49	1	1	1	0.946	1	1	1	-0.018
SE	4284	PLXNC1	PLXNC1	chr12	+	94259336	94259375	94255192	94255296	94259609	94259734	103	64	33	2	2	0	0	26	0	3	88	49	1	1	0.966	0.947	1	NA	0.828	0.143
SE	4286	PLXNC1	PLXNC1	chr12	+	94298631	94298795	94297315	94297423	94303755	94303896	31	26	12	2	0	0	0	14	0	0	98	49	1	1	0.886	1	1	NA	1	-0.038
SE	4287	PLXNC1	PLXNC1	chr12	+	94300909	94301057	94297315	94297423	94303755	94303896	68	30	19	2	0	0	0	11	0	0	98	49	1	1	0.944	1	1	NA	1	-0.019
SE	4295	CEP135	CEP135	chr4	+	56019352	56019555	56017647	56017857	56020675	56020780	6	0	0	0	0	0	0	2	0	1	98	49	1	1	1	NA	NA	NA	0.5	0.5
SE	4296	PCID2	PCID2	chr13	-	113190871	113190975	113185484	113185560	113195070	113195125	5	1	0	1	0	0	0	2	0	0	98	49	1	1	0.714	1	NA	NA	1	-0.143
SE	4298	PACSIN2	PACSIN2	chr22	-	42882183	42882304	42879047	42879169	42884385	42884561	70	39	24	0	2	0	6	13	0	0	98	49	1	1	1	0.907	1	1	1	-0.031
SE	4299	PACSIN2	PACSIN2	chr22	-	42890946	42891182	42888642	42888798	42893456	42893613	59	47	13	1	0	0	1	8	0	0	98	49	1	1	0.967	1	1	1	1	-0.011
SE	4303	PFDN5	PFDN5	chr12	+	53296243	53296275	53295838	53295941	53297849	53297924	43	7	8	2	0	0	0	8	0	0	81	49	1	1	0.929	1	1	NA	1	-0.024
SE	4313	ZNF512	ZNF512	chr2	+	27600690	27600815	27599969	27600053	27601355	27601442	9	2	0	0	0	0	0	0	0	1	98	49	1	1	1	1	NA	NA	0	1
SE	4322	GTPBP1	GTPBP1	chr22	+	38708844	38708956	38705801	38706147	38715906	38716087	31	8	3	4	7	1	0	8	0	1	98	49	1	1	0.795	0.364	0.6	NA	0.8	-0.214
SE	4323	GTPBP1	GTPBP1	chr22	+	38721741	38721865	38716651	38717000	38724296	38724411	32	15	1	0	1	0	0	4	0	0	98	49	1	1	1	0.882	1	NA	1	-0.039
SE	4324	GTPBP1	GTPBP1	chr22	+	38726257	38726440	38726005	38726150	38727982	38728161	22	19	5	0	0	0	0	12	0	1	98	49	1	1	1	1	1	NA	0.857	0.143
SE	4326	GTPBP1	GTPBP1	chr22	+	38727982	38728161	38726005	38726150	38729461	38729662	24	12	0	1	0	0	0	8	0	0	98	49	1	1	0.923	1	NA	NA	1	-0.038
SE	4327	GTPBP1	GTPBP1	chr22	+	38727982	38728161	38727212	38727348	38729461	38729662	53	27	5	1	0	0	0	11	0	0	98	49	1	1	0.964	1	1	NA	1	-0.012
SE	4352	SLC8A1	SLC8A1	chr2	-	40170280	40170349	40164853	40164984	40174824	40174842	1	0	0	10	4	5	0	0	0	2	98	49	1	1	0.048	0	0	NA	0	0.016
SE	4354	SLC8A1	SLC8A1	chr2	-	40177751	40177855	40174824	40174842	40428472	40430304	17	2	6	0	0	1	0	2	0	0	98	49	1	1	1	1	0.75	NA	1	-0.083
SE	4357	TBC1D15	TBC1D15	chr12	+	71880468	71880607	71872928	71873003	71884810	71885021	14	4	0	2	2	0	0	3	0	0	98	49	1	1	0.778	0.5	NA	NA	1	-0.361
SE	4358	TBC1D15	TBC1D15	chr12	+	71884810	71885021	71880468	71880607	71893221	71893324	10	7	0	1	0	0	0	3	0	0	98	49	1	1	0.833	1	NA	NA	1	-0.084
SE	4359	TBC1D15	TBC1D15	chr12	+	71894324	71894375	71893221	71893324	71894685	71894883	2	0	2	4	4	3	0	0	0	1	98	49	1	1	0.2	0	0.25	NA	0	0.15
SE	4364	2-Sep	2-Sep	chr2	+	241335321	241335351	241335125	241335212	241335974	241336098	4	1	0	7	2	0	0	0	0	1	79	49	1	1	0.262	0.237	NA	NA	0	0.249
SE	4365	2-Sep	2-Sep	chr2	+	241335974	241336098	241335125	241335212	241337381	241337516	17	3	0	0	1	0	0	4	0	0	98	49	1	1	1	0.6	NA	NA	1	-0.2
SE	4373	SNRK	SNRK	chr3	+	43332168	43332310	43303097	43303792	43340286	43340499	50	33	15	2	0	0	0	7	0	0	98	49	1	1	0.926	1	1	NA	1	-0.025
SE	4376	IL32	IL32	chr16	+	3067553	3067613	3067376	3067415	3067983	3068010	29	15	2	3	1	0	0	4	0	0	98	49	1	1	0.829	0.882	1	NA	1	-0.096
SE	4379	LYZ	LYZ	chr12	+	69352219	69352298	69350107	69350272	69353152	69354233	1515	182	52	5	0	0	40	456	0	1	98	49	1	1	0.993	1	1	1	0.996	0
SE	4385	SIK3	SIK3	chr11	-	116867945	116868089	116863667	116863818	116870330	116870401	11	3	1	3	0	0	0	4	0	0	98	49	1	1	0.647	1	1	NA	1	-0.118
SE	4386	SIK3	SIK3	chr11	-	116875373	116875451	116875157	116875267	116876252	116876363	13	5	0	0	0	0	0	3	0	1	98	49	1	1	1	1	NA	NA	0.6	0.4
SE	4387	SIK3	SIK3	chr11	-	116875865	116876009	116875373	116875451	116876252	116876363	7	1	5	1	4	0	0	1	0	2	98	49	1	1	0.778	0.111	1	NA	0.2	0.43
SE	4388	SIK3	SIK3	chr11	-	116876923	116877042	116876252	116876363	116896252	116896376	20	10	10	1	1	0	0	8	0	0	98	49	1	1	0.909	0.833	1	NA	1	-0.086
SE	4393	ZNF692	ZNF692	chr1	-	248850681	248850781	248850003	248850516	248853936	248854051	12	3	0	0	0	0	0	0	0	3	98	49	1	1	1	1	NA	NA	0	1
SE	4407	TOR2A	TOR2A	chr9	-	127732563	127732691	127731523	127732278	127733384	127733557	2	1	1	1	0	0	0	2	0	0	98	49	1	1	0.5	1	1	NA	1	-0.167
SE	4410	AP1G1	AP1G1	chr16	-	71748250	71748378	71746587	71746692	71749893	71749983	19	7	0	0	0	0	1	4	0	1	98	49	1	1	1	1	NA	1	0.667	0.166
SE	4411	AP1G1	AP1G1	chr16	-	71756018	71756159	71753832	71753887	71758807	71758921	37	15	4	2	3	0	0	7	0	0	98	49	1	1	0.902	0.714	1	NA	1	-0.128
SE	4412	AP1G1	AP1G1	chr16	-	71771155	71771252	71769622	71769699	71773220	71773362	19	8	1	1	0	0	0	11	0	0	98	49	1	1	0.905	1	1	NA	1	-0.032
SE	4413	AP1G1	AP1G1	chr16	-	71789278	71789482	71774467	71774592	71808762	71809073	26	27	10	0	2	0	1	7	0	0	98	49	1	1	1	0.871	1	1	1	-0.043
SE	4416	ARHGAP9	ARHGAP9	chr12	-	57473602	57473708	57472254	57472688	57474041	57474176	289	109	70	30	11	8	0	32	0	3	98	49	1	1	0.828	0.832	0.814	NA	0.842	-0.017
SE	4417	ARHGAP9	ARHGAP9	chr12	-	57474041	57474176	57473602	57473708	57474422	57474476	362	134	91	5	0	0	0	40	0	0	98	49	1	1	0.973	1	1	NA	1	-0.009
SE	4418	ARHGAP9	ARHGAP9	chr12	-	57474874	57474973	57474625	57474703	57475290	57475398	204	55	68	1	1	0	3	37	0	0	98	49	1	1	0.99	0.965	1	1	1	-0.015
SE	4423	MPZL1	MPZL1	chr1	+	167772274	167772488	167765582	167765749	167773235	167773368	13	0	19	1	0	0	0	1	0	0	98	49	1	1	0.867	NA	1	NA	1	-0.067
SE	4424	MPZL1	MPZL1	chr1	+	167773235	167773368	167765582	167765749	167787819	167791919	10	5	10	0	0	3	0	1	0	0	98	49	1	1	1	1	0.625	NA	1	-0.125
SE	4425	MPZL1	MPZL1	chr1	+	167776063	167776166	167773235	167773368	167787819	167791919	5	0	0	9	5	10	0	1	0	1	98	49	1	1	0.217	0	0	NA	0.333	-0.261
SE	4427	STARD3	STARD3	chr17	+	39657972	39658026	39657774	39657852	39658404	39658522	8	15	2	0	2	0	0	4	0	0	98	49	1	1	1	0.789	1	NA	1	-0.07
SE	4429	LRP1	LRP1	chr12	+	57180666	57180807	57180329	57180479	57181156	57181291	7	0	0	2	0	0	0	9	0	0	98	49	1	1	0.636	NA	NA	NA	1	-0.364
SE	4432	MON2	MON2	chr12	+	62566013	62566031	62565236	62565380	62566321	62566450	5	1	0	1	0	0	0	0	0	1	67	49	1	1	0.785	1	NA	NA	0	0.893
SE	4438	PTGES3	PTGES3	chr12	-	56665631	56666266	56664775	56664800	56670274	56670364	17	6	14	1	0	0	1	2	0	0	98	49	1	1	0.895	1	1	1	1	-0.035
SE	4439	PTGES3	PTGES3	chr12	-	56666203	56666266	56664775	56664800	56670274	56670364	23	19	15	1	0	0	1	7	0	0	98	49	1	1	0.92	1	1	1	1	-0.027
SE	4445	DYNC1H1	DYNC1H1	chr14	+	101991526	101991673	101988702	101988852	101994183	101994324	16	4	1	2	0	0	1	6	0	0	98	49	1	1	0.8	1	1	1	1	-0.067
SE	4455	ATP5B	ATP5B	chr12	-	56645170	56645353	56644780	56644955	56645836	56646068	35	9	6	2	0	1	0	21	0	0	98	49	1	1	0.897	1	0.75	NA	1	-0.118
SE	4463	ANKRD52	ANKRD52	chr12	-	56243784	56243876	56237806	56243392	56244050	56244133	6	1	0	0	0	0	0	0	0	1	98	49	1	1	1	1	NA	NA	0	1
SE	4473	ANAPC4	ANAPC4	chr4	+	25413644	25413742	25409697	25409791	25414323	25414385	2	1	0	1	0	0	0	6	0	0	98	49	1	1	0.5	1	NA	NA	1	-0.25
SE	4484	USP15	USP15	chr12	+	62349220	62349307	62325871	62325933	62355330	62355475	170	68	40	5	5	1	0	15	0	0	98	49	1	1	0.944	0.872	0.952	NA	1	-0.077
SE	4485	USP15	USP15	chr12	+	62389604	62389699	62389430	62389514	62389796	62389988	194	66	36	1	0	0	4	22	0	0	98	49	1	1	0.99	1	1	1	1	-0.003
SE	4486	USP15	USP15	chr12	+	62396294	62396398	62393052	62393202	62401186	62401275	126	35	33	5	6	0	0	13	0	1	98	49	1	1	0.926	0.745	1	NA	0.867	0.023
SE	4488	R3HDM2	R3HDM2	chr12	-	57288856	57288910	57283823	57284056	57289008	57289040	0	2	0	5	0	4	0	0	0	3	98	49	1	1	0	1	0	NA	0	0.333
SE	4489	R3HDM2	R3HDM2	chr12	-	57289008	57289040	57283823	57284056	57295398	57295507	5	3	4	1	0	0	0	4	0	0	81	49	1	1	0.752	1	1	NA	1	-0.083
SE	4490	NCKAP1L	NCKAP1L	chr12	+	54523824	54523956	54523393	54523539	54526527	54526746	35	36	3	0	1	0	3	12	0	0	98	49	1	1	1	0.947	1	1	1	-0.018
SE	4495	NFE2	NFE2	chr12	-	54300146	54300276	54295134	54295304	54300800	54301037	8	1	7	9	9	1	0	0	0	2	98	49	1	1	0.308	0.053	0.778	NA	0	0.38
SE	4500	ICAM1	ICAM1	chr19	+	10284782	10285028	10284402	10284657	10285114	10286615	10	13	6	0	1	0	0	7	0	0	98	49	1	1	1	0.867	1	NA	1	-0.044
SE	4501	MARS	MARS	chr12	+	57489895	57489971	57489423	57489558	57490206	57490379	15	4	0	5	1	0	0	5	0	0	98	49	1	1	0.6	0.667	NA	NA	1	-0.367
SE	4502	MARS	MARS	chr12	+	57498156	57498273	57490537	57490644	57498419	57498623	15	10	2	0	2	0	1	6	0	0	98	49	1	1	1	0.714	1	1	1	-0.095
SE	4503	MARS	MARS	chr12	+	57500320	57500522	57498419	57498623	57504224	57504299	34	20	8	1	0	0	1	6	0	0	98	49	1	1	0.944	1	1	1	1	-0.019
SE	4508	FIG4	FIG4	chr6	+	109727108	109727265	109716444	109716568	109732636	109732687	3	4	0	1	0	0	1	3	0	0	98	49	1	1	0.6	1	NA	1	1	-0.2
SE	4510	APPL2	APPL2	chr12	-	105203702	105203785	105199372	105199531	105207060	105207207	10	4	5	2	0	0	0	1	0	0	98	49	1	1	0.714	1	1	NA	1	-0.095
SE	4511	CALCOCO1	CALCOCO1	chr12	-	53715792	53716047	53715199	53715325	53719738	53719829	16	1	2	2	0	0	0	1	0	0	98	49	1	1	0.8	1	1	NA	1	-0.067
SE	4516	VTI1B	VTI1B	chr14	-	67662476	67662535	67659730	67659922	67674374	67674885	14	2	1	1	0	0	1	2	0	0	98	49	1	1	0.875	1	1	1	1	-0.042
SE	4517	TEP1	TEP1	chr14	-	20372732	20372857	20371488	20371632	20373010	20373147	12	0	0	4	3	0	0	3	0	0	98	49	1	1	0.6	0	NA	NA	1	-0.7
SE	4518	TEP1	TEP1	chr14	-	20378753	20378853	20378379	20378535	20378980	20379105	8	0	0	2	2	0	0	1	0	0	98	49	1	1	0.667	0	NA	NA	1	-0.666
SE	4519	TEP1	TEP1	chr14	-	20381552	20381686	20381312	20381401	20381912	20382063	2	1	0	1	0	0	0	1	0	0	98	49	1	1	0.5	1	NA	NA	1	-0.25
SE	4533	CTBP1	CTBP1	chr4	-	1241324	1241519	1238182	1238337	1248915	1249120	7	1	1	2	1	0	0	6	0	0	98	49	1	1	0.636	0.333	1	NA	1	-0.344
SE	4537	SPRYD3	SPRYD3	chr12	-	53074648	53074784	53073285	53073471	53075094	53075219	2	6	4	0	0	0	0	0	0	2	98	49	1	1	1	1	1	NA	0	1
SE	4539	MDM1	MDM1	chr12	-	68316580	68316610	68316077	68316253	68321346	68321446	0	1	0	1	0	0	0	2	0	0	79	49	1	1	0	1	NA	NA	1	-0.5
SE	4541	C16orf93	C16orf93	chr16	-	30759011	30759101	30757422	30757654	30759177	30759247	0	0	0	1	1	0	1	0	0	0	98	49	1	1	0	0	NA	1	NA	-1
SE	4542	VCL	VCL	chr10	+	74111908	74112112	74108970	74109156	74114183	74114387	1	0	0	5	0	0	0	0	1	0	98	49	1	1	0.091	NA	NA	0	NA	0.091
SE	4549	UBE2Q2	UBE2Q2	chr15	+	75859877	75859982	75854385	75854487	75868950	75869010	10	3	6	0	0	0	0	5	0	1	98	49	1	1	1	1	1	NA	0.714	0.286
SE	4550	NDUFA11	NDUFA11	chr19	-	5901334	5901454	5896904	5896997	5903611	5904013	1	1	0	14	3	2	0	0	0	2	98	49	1	1	0.034	0.143	0	NA	0	0.059
SE	4553	TFCP2	TFCP2	chr12	-	51098775	51098918	51095988	51096040	51099654	51099779	3	1	0	0	0	1	0	3	0	0	98	49	1	1	1	1	0	NA	1	-0.333
SE	4554	TFCP2	TFCP2	chr12	-	51109120	51109273	51107235	51107346	51110876	51110983	2	0	2	2	0	0	0	1	0	0	98	49	1	1	0.333	NA	1	NA	1	-0.334
SE	4555	RASSF2	RASSF2	chr20	-	4795814	4795966	4792538	4792627	4798009	4798085	136	98	66	0	0	0	4	29	0	1	98	49	1	1	1	1	1	1	0.935	0.032
SE	4556	RASSF2	RASSF2	chr20	-	4822328	4822403	4800971	4801062	4823556	4823645	51	31	16	3	0	0	0	4	0	0	98	49	1	1	0.895	1	1	NA	1	-0.035
SE	4557	INPP5A	INPP5A	chr10	+	132645867	132645968	132607914	132607956	132650417	132650505	19	13	0	1	0	0	1	3	0	0	98	49	1	1	0.905	1	NA	1	1	-0.047

SE	4558	INPP5A	INPP5A	chr10	+	132690391	132690455	132650417	132650505	132697815	132697919	17	11	5	1	0	0	0	7	0	0	98	49	1	1	0.895	1	1	NA	1	-0.035
SE	4563	LRRC28	LRRC28	chr15	+	99333922	99334129	99287813	99287951	99352368	99352471	1	0	0	2	0	0	0	1	0	0	98	49	1	1	0.2	NA	NA	NA	1	-0.8
SE	4564	LRRC28	LRRC28	chr15	+	99363105	99363265	99361335	99361511	99386029	99386309	2	0	4	1	0	0	0	3	0	0	98	49	1	1	0.5	NA	1	NA	1	-0.25
SE	4565	HEXDC	HEXDC	chr17	+	82439630	82439713	82437167	82437363	82440996	82441266	2	3	0	0	0	0	0	1	0	1	98	49	1	1	1	1	NA	NA	0.333	0.667
SE	4577	PI4KB	PI4KB	chr1	-	151324744	151324917	151315572	151316509	151326171	151326373	0	1	0	2	0	0	0	0	0	2	98	49	1	1	0	1	NA	NA	0	0.5
SE	4579	PI4KB	PI4KB	chr1	-	151326171	151326373	151315572	151316509	151327270	151327657	3	1	0	1	3	0	0	4	0	0	98	49	1	1	0.6	0.143	NA	NA	1	-0.629
SE	4580	PI4KB	PI4KB	chr1	-	151326171	151326373	151324744	151324917	151327270	151327657	1	1	0	1	1	0	0	2	0	0	98	49	1	1	0.333	0.333	NA	NA	1	-0.667
SE	4581	TRIP12	TRIP12	chr2	-	229860405	229860531	229858771	229859574	229879981	229880128	14	5	0	9	3	0	0	3	0	2	98	49	1	1	0.437	0.455	NA	NA	0.429	0.017
SE	4583	PSMA3	PSMA3	chr14	+	58252118	58252242	58247749	58247832	58257744	58257846	14	8	0	0	0	1	0	3	0	0	98	49	1	1	1	1	0	NA	1	-0.333
SE	4584	PSMA3	PSMA3	chr14	+	58270933	58270998	58270417	58270485	58271850	58272009	12	6	3	0	0	0	0	6	0	1	98	49	1	1	1	1	1	NA	0.75	0.25
SE	4589	PARP8	PARP8	chr5	+	50759642	50759732	50750150	50750188	50761820	50761898	26	32	18	0	1	0	0	5	0	0	98	49	1	1	1	0.941	1	NA	1	-0.02
SE	4590	PARP8	PARP8	chr5	+	50760291	50760362	50759642	50759732	50761820	50761898	111	49	38	3	4	0	0	17	0	0	98	49	1	1	0.949	0.86	1	NA	1	-0.064
SE	4591	PARP8	PARP8	chr5	+	50763147	50763242	50761820	50761898	50778068	50778129	89	35	10	2	0	0	3	15	0	1	98	49	1	1	0.957	1	1	1	0.882	0.045
SE	4592	PARP8	PARP8	chr5	+	50794852	50795417	50794206	50794332	50796981	50797032	152	56	11	6	2	0	0	30	0	0	98	49	1	1	0.927	0.933	1	NA	1	-0.047
SE	4594	PARP8	PARP8	chr5	+	50824907	50824975	50822334	50822400	50826754	50826803	109	45	14	0	0	0	0	14	0	1	98	49	1	1	1	1	1	NA	0.875	0.125
SE	4609	YWHAB	YWHAB	chr20	+	44887647	44887742	44885598	44885886	44901530	44901833	5	0	0	9	5	4	0	0	0	1	98	49	1	1	0.217	0	0	NA	0	0.072
SE	4610	P2RX7	P2RX7	chr12	+	121154784	121154953	121132827	121133095	121156078	121156147	5	0	0	2	0	0	0	1	0	0	98	49	1	1	0.556	NA	NA	NA	1	-0.444
SE	4617	KIF13A	KIF13A	chr6	-	17779591	17779684	17778946	17779099	17780729	17780906	13	3	4	2	0	0	1	2	0	0	98	49	1	1	0.765	1	1	1	1	-0.078
SE	4619	KIF13A	KIF13A	chr6	-	17794248	17794395	17787775	17787875	17794571	17794704	6	5	7	2	0	0	2	2	0	0	98	49	1	1	0.6	1	1	1	1	-0.133
SE	4620	KIF13A	KIF13A	chr6	-	17799951	17800113	17799265	17799439	17804360	17804510	36	15	2	1	0	0	0	2	0	0	98	49	1	1	0.947	1	1	NA	1	-0.018
SE	4621	KIF13A	KIF13A	chr6	-	17817019	17817233	17808767	17808930	17825767	17825934	21	7	4	0	0	0	0	5	0	1	98	49	1	1	1	1	1	NA	0.714	0.286
SE	4622	KIF13A	KIF13A	chr6	-	17855436	17855617	17851954	17852042	17856029	17856122	30	20	3	0	1	0	0	9	0	1	98	49	1	1	1	0.909	1	NA	0.818	0.152
SE	4623	KIF13A	KIF13A	chr6	-	17898167	17898180	17873376	17873437	17987053	17987144	16	14	2	0	0	0	0	6	0	1	62	49	1	1	1	1	1	NA	0.826	0.174
SE	4625	AKAP11	AKAP11	chr13	+	42317527	42317688	42313893	42313940	42319087	42323267	7	0	0	0	0	0	0	1	0	1	98	49	1	1	1	NA	NA	NA	0.333	0.667
SE	4626	INPP4A	INPP4A	chr2	+	98545968	98546073	98543876	98544007	98552785	98552969	18	2	0	0	0	0	3	0	0	1	98	49	1	1	1	1	NA	1	0	0.5
SE	4627	INPP4A	INPP4A	chr2	+	98546585	98546694	98545968	98546073	98552785	98552969	14	11	1	1	0	0	0	1	0	0	98	49	1	1	0.875	1	1	NA	1	-0.042
SE	4630	HADHA	HADHA	chr2	-	26193576	26193772	26192309	26192424	26194569	26194638	25	3	4	1	0	0	1	11	0	1	98	49	1	1	0.926	1	1	1	0.846	0.052
SE	4631	HADHA	HADHA	chr2	-	26204061	26204196	26201148	26201320	26209779	26209889	22	3	2	1	0	0	0	2	0	0	98	49	1	1	0.917	1	1	NA	1	-0.028
SE	4633	MS4A4A	MS4A4A	chr11	+	60301000	60301057	60297196	60297325	60306099	60306201	1	0	0	1	0	0	0	1	0	0	98	49	1	1	0.333	NA	NA	NA	1	-0.667
SE	4637	RPAP3	RPAP3	chr12	-	47679492	47679594	47670106	47670345	47679703	47679774	8	2	1	1	0	0	0	2	0	0	98	49	1	1	0.8	1	1	NA	1	-0.067
SE	4646	ANO6	ANO6	chr12	+	45416698	45416904	45409356	45409487	45421070	45421273	9	0	0	1	0	0	0	1	0	0	98	49	1	1	0.818	NA	NA	NA	1	-0.182
SE	4647	MACF1	MACF1	chr1	+	39378460	39378523	39372478	39372596	39379202	39379444	2	1	0	7	6	0	0	0	0	1	98	49	1	1	0.125	0.077	NA	NA	0	0.101
SE	4648	MACF1	MACF1	chr1	+	39381952	39382152	39380243	39380373	39385433	39385929	11	3	0	1	0	0	0	1	0	0	98	49	1	1	0.846	1	NA	NA	1	-0.077
SE	4651	UBR1	UBR1	chr15	-	42970519	42970607	42966152	42966286	42976716	42976867	12	3	0	1	0	0	0	1	0	0	98	49	1	1	0.857	1	NA	NA	1	-0.072
SE	4664	ADGRE3	ADGRE3	chr19	-	14641418	14641616	14638104	14638340	14644107	14644275	87	42	15	0	2	0	3	9	0	0	98	49	1	1	1	0.913	1	1	1	-0.029
SE	4665	ADGRE3	ADGRE3	chr19	-	14654981	14655165	14651084	14651204	14658512	14658550	144	56	36	4	2	0	0	16	0	0	98	49	1	1	0.947	0.933	1	NA	1	-0.04
SE	4667	ADGRE3	ADGRE3	chr19	-	14658512	14658550	14654981	14655165	14661962	14662118	162	46	27	1	0	0	0	13	0	1	87	49	1	1	0.989	1	1	NA	0.88	0.116
SE	4668	ADGRE3	ADGRE3	chr19	-	14661962	14662118	14658512	14658550	14663417	14663540	172	64	29	3	0	0	0	12	0	0	98	49	1	1	0.966	1	1	NA	1	-0.011
SE	4669	ADGRE3	ADGRE3	chr19	-	14663417	14663540	14661962	14662118	14668801	14668852	164	56	21	1	0	0	0	9	0	0	98	49	1	1	0.988	1	1	NA	1	-0.004
SE	4670	ADGRE3	ADGRE3	chr19	-	14668801	14668852	14663417	14663540	14674745	14674918	103	30	4	1	1	0	0	6	0	0	98	49	1	1	0.981	0.937	1	NA	1	-0.027
SE	4675	DNM1L	DNM1L	chr12	+	32705824	32705863	32701414	32701562	32707366	32707413	0	1	0	1	0	0	0	0	0	1	88	49	1	1	0	1	NA	NA	0	0.5
SE	4680	FGD4	FGD4	chr12	+	32576265	32576449	32564136	32564289	32581959	32582467	6	1	0	0	1	0	0	5	0	0	98	49	1	1	1	0.333	NA	NA	1	-0.334
SE	4681	FGD4	FGD4	chr12	+	32581959	32582467	32564136	32564289	32598496	32598586	3	7	1	0	1	0	0	2	0	0	98	49	1	1	1	0.778	1	NA	1	-0.074
SE	4682	FGD4	FGD4	chr12	+	32600409	32600509	32598496	32598586	32601277	32601423	3	0	0	21	9	5	0	0	0	5	98	49	1	1	0.067	0	0	NA	0	0.022
SE	4683	FGD4	FGD4	chr12	+	32607937	32608095	32602160	32602317	32610775	32610834	33	19	22	0	0	1	0	10	0	0	98	49	1	1	1	1	0.917	NA	1	-0.028
SE	4684	FGD4	FGD4	chr12	+	32607956	32608095	32602160	32602317	32610775	32610834	56	25	37	0	0	1	0	14	0	0	98	49	1	1	1	1	0.949	NA	1	-0.017
SE	4685	ZNF267	ZNF267	chr16	+	31875151	31875302	31873757	31873969	31884497	31884624	1	0	0	22	15	2	0	0	0	2	98	49	1	1	0.022	0	0	NA	0	0.007
SE	4686	ZNF267	ZNF267	chr16	+	31885160	31885256	31884497	31884624	31914475	31917308	72	26	8	0	1	0	1	12	0	0	98	49	1	1	1	0.929	1	1	1	-0.024
SE	4695	SERPINB9	SERPINB9	chr6	-	2893410	2893553	2891832	2891988	2895390	2895508	7	1	0	0	0	0	0	3	0	1	98	49	1	1	1	1	NA	NA	0.6	0.4
SE	4696	PHF3	PHF3	chr6	+	63646526	63646795	63635801	63636150	63679999	63680161	2	0	5	6	1	0	0	0	0	1	98	49	1	1	0.143	0	1	NA	0	0.381
SE	4698	PHF3	PHF3	chr6	+	63679999	63680161	63635801	63636150	63684128	63685911	24	3	0	1	1	2	0	7	0	0	98	49	1	1	0.923	0.6	0	NA	1	-0.492
SE	4700	IPO8	IPO8	chr12	-	30662326	30662487	30661140	30661266	30663488	30663654	11	0	2	1	0	0	0	2	0	0	98	49	1	1	0.846	NA	1	NA	1	-0.077
SE	4701	LCP1	LCP1	chr13	-	46130813	46130938	46125922	46127723	46134126	46134250	450	237	209	0	0	1	5	131	0	0	98	49	1	1	1	1	0.991	1	1	-0.003
SE	4702	LCP1	LCP1	chr13	-	46146907	46147103	46144441	46144520	46148351	46148447	618	315	228	1	1	0	9	167	0	1	98	49	1	1	0.997	0.994	1	1	0.988	0.003
SE	4703	LCP1	LCP1	chr13	-	46148351	46148447	46144441	46144520	46150935	46151078	82	45	35	0	0	0	0	37	0	3	98	49	1	1	1	1	1	NA	0.86	0.14
SE	4704	LCP1	LCP1	chr13	-	46158521	46158651	46156437	46156570	46158825	46158989	663	268	190	5	0	0	1	128	0	0	98	49	1	1	0.985	1	1	1	1	-0.005
SE	4706	VPS13C	VPS13C	chr15	-	61920497	61920647	61920066	61920331	61921946	61922033	14	2	3	1	0	0	0	5	0	0	98	49	1	1	0.875	1	1	NA	1	-0.042
SE	4709	FGFR1OP2	FGFR1OP2	chr12	+	26960514	26960628	26957600	26957743	26963341	26963455	4	0	1	3	7	1	0	0	0	1	98	49	1	1	0.4	0	0.333	NA	0	0.244
SE	4710	ASUN	ASUN	chr12	-	26914407	26914578	26913973	26914128	26916001	26916180	1	0	0	1	0	0	0	1	0	0	98	49	1	1	0.333	NA	NA	NA	1	-0.667
SE	4716	LPCAT2	LPCAT2	chr16	+	55534442	55534477	55532823	55532882	55537577	55537632	41	32	16	1	0	0	0	7	0	0	84	49	1	1	0.96	1	1	NA	1	-0.013
SE	4717	KRAS	KRAS	chr12	-	25215436	25215560	25204788	25209911	25225613	25225773	0	1	0	6	5	0	0	0	0	1	98	49	1	1	0	0.091	NA	NA	0	0.045
SE	4718	KRAS	KRAS	chr12	-	25227233	25227412	25225613	25225773	25245273	25245395	10	4	1	0	1	0	0	1	0	0	98	49	1	1	1	0.667	1	NA	1	-0.111
SE	4722	LRMP	LRMP	chr12	+	25062819	25062900	25052246	25052956	25063719	25063816	52	35	13	8	0	2	0	10	0	0	98	49	1	1	0.765	1	0.765	NA	1	-0.157
SE	4723	LRMP	LRMP	chr12	+	25066364	25066512	25063719	25063816	25069349	25069431	121	46	10	5	1	0	0	17	0	0	98	49	1	1	0.924	0.958	1	NA	1	-0.039
SE	4727	HOOK3	HOOK3	chr8	+	42968012	42968214	42966472	42966613	42973288	42973399	5	1	0	0	0	0	0	1	0	3	98	49	1	1	1	1	NA	NA	0.143	0.857
SE	4735	RECQL	RECQL	chr12	-	21477802	21477969	21476910	21476992	21483375	21483574	8	2	0	0	4	0	0	2	0	0	98	49	1	1	1	0.2	NA	NA	1	-0.4
SE	4736	CSAD	CSAD	chr12	-	53161126	53161191	53160762	53160844	53161272	53161389	8	3	6	4	5	0	0	2	0	4	98	49	1	1	0.5	0.231	1	NA	0.2	0.377
SE	4737	PYROXD1	PYROXD1	chr12	+	21452080	21452154	21449562	21449691	21455131	21455292	1	0	3	1	0	0	0	1	0	0	98	49	1	1	0.333	NA	1	NA	1	-0.334
SE	4744	ARID2	ARID2	chr12	+	45836740	45836991	45836588	45836655	45837320	45837417	15	6	0	2	0	0	0	2	0	1	98	49	1	1	0.789	1	NA	NA	0.5	0.395
SE	4754	SMARCC2	SMARCC2	chr12	-	56172936	56173029	56172584	56172704	56173695	56173849	1	3	0	3	7	0	0	0	0	1	98	49	1	1	0.143	0.176	NA	NA	0	0.159
SE	4755	SMARCC2	SMARCC2	chr12	-	56178403	56178534	56178021	56178093	56178809	56178847	9	2	2	0	1	0	0	3	0	0	98	49	1	1	1	0.5	1	NA	1	-0.167
SE	4756	SMARCC2	SMARCC2	chr12	-	56183860	56183930	56182003	56182079	56184174	56184244	17	6	0	0	2	0	0	2	0	0	98	49	1	1	1	0.6	NA	NA	1	-0.2
SE	4758	SPG7	SPG7	chr16	+	89529476	89529579	89526328	89526468	89530682	89530808	7	6	2	1	2	0	0	2	0	0	98	49	1	1	0.778	0.6	1	NA	1	-0.207
SE	4762	ATP2C1	ATP2C1	chr3	+	130979248	130979419	130975331	130975488	130980581	130980679	1	0	0	2	0	0	1	2	0	0	98	49	1	1	0.2	NA	NA	1	1	-0.8
SE	4773	NDST2	NDST2	chr10	-	73805586	73805769	73804772	73804869	73805899	73806028	10	3	0	1	0	0	0	5	0	0	98	49	1	1	0.833	1	NA	NA	1	-0.084
SE	4774	NDST2	NDST2	chr10	-	73806288	73806474	73805899	73806028	73806656	73806811	8	3	6	1	0	0	0	8	0	0	98	49	1	1	0.8	1	1	NA	1	-0.067
SE	4783	CARD8	CARD8	chr19	-	48232452	48232493	48231659	48231810	48234402	48234543	23	9	0	6	5	1	0	3	0	0	90	49	1	1	0.676	0.495	0	NA	1	-0.61
SE	4784	CARD8	CARD8	chr19	-	48238382	48238532	48234402	48234543	48240961	48241063	57	18	20	18	6	7	0	7	0	1	98	49	1	1	0.613	0.6	0.588	NA	0.778	-0.178
SE	4785	CARD8	CARD8	chr19	-	48249522	48249633	48240961	48241063	48249750	48249847	30	12	5	3	0	0	0	1	0	1	98	49	1	1	0.833	1	1	NA	0.333	0.611
SE	4790	HNRNPH3	HNRNPH3	chr10	+	68337857	68337996	68337198	68337333	68338502	68338642	8	5	0	7	0	0	0	1	0	0	98	49	1	1	0.364	1	NA	NA	1	-0.318
SE	4795	HNRNPK	HNRNPK	chr9	-	83975461	83975505	83974516	83974589	83976994	83977051	100	74	44	5	4	2	0	23	0	0	93	49	1	1	0.913	0.907	0.921	NA	1	-0.086
SE	4796	PADI2	PADI2	chr1	-	17071405	17071491	17070087	17070216	17074855	17074949	29	22	20	1	0	0	0	13	0	0	98	49	1	1	0.935	1	1	NA	1	-0.022
SE	4797	PADI2	PADI2	chr1	-	17079263	17079415	17075678	17075823	17082544	17082652	30	16	16	2	1	0	0	12	0	1	98	49	1	1	0.882	0.889	1	NA	0.857	0.067
SE	4798	PADI2	PADI2	chr1	-	17086520	17086699	17084598	17084702	17092407	17092533	35	39	36	1	0	0	1	22	0	0	98	49	1	1	0.946	1	1	1	1	-0.018
SE	4803	YBX3	YBX3	chr12	-	10709907	10710114	10704050	10704148	10713210	10713333	8	4	1	3	1	0	0	5	0	0	98	49	1	1	0.571	0.667	1	NA	1	-0.254
SE	4804	KDM6A	KDM6A	chrX	+	45020609	45020730	45010960	45011019	45034930	45034985	27	6	0	0	0	0	0	7	0	3	98	49	1	1	1	1	NA	NA	0.538	0.462
SE	4805	KDM6A	KDM6A	chrX	+	45059005	45059104	45053828	45053955	45059246	45059466	36	9	0	1	0	0	4	4	0	1	98	49	1	1	0.947	1	NA	1	0.667	0.14
SE	4806	KDM6A	KDM6A	chrX	+	45060021	45060156	45059246	45059466	45060608	45060764	6	0	0	7	0	0	0	0	0	3	98	49	1	1	0.3	NA	NA	NA	0	0.3
SE	4807	KDM6A	KDM6A	chrX	+	45060021	45060156	45059246	45059466	45061323	45061419	7	0	0	1	0	0	0	0	0	3	98	49	1	1	0.778	NA	NA	NA	0	0.778
SE	4808	KDM6A	KDM6A	chrX	+	45060608	45060764	45059246	45059466	45061323	45061419	11	8	2	1	0	0	0	4	0	3	98	49	1	1	0.846	1	1	NA	0.4	0.549
SE	4809	KDM6A	KDM6A	chrX	+	45060608	45060764	45060021	45060156	45061323	45061419	4	8	2	1	0	0	0	1	0	0	98	49	1	1	0.667	1	1	NA	1	-0.111
SE	4810	KDM6A	KDM6A	chrX	+	45062646	45062748	45061323	45061419	45063421	45063817	20	2	1	1	1	1	0	6	0	0	98	49	1	1	0.909	0.5	0.333	NA	1	-0.419
SE	4811	KDM6A	KDM6A	chrX	+	45110078	45110249	45107409	45107536	45111381	45112612	26	8	2	1	0	0	1	7	0	0	98	49	1	1	0.929	1	1	1	1	-0.024
SE	4815	FOXP1	FOXP1	chr3	-	71359149	71359244	71299819	71299880	71493425	71493555	16	5	2	0	0	0	0	4	0	1	98	49	1	1	1	1	1	NA	0.667	0.333
SE	4816	QKI	QKI	chr6	+	163563419	163563719	163561981	163562069	163566720	163566795	39	13	15	0	2	0	0	4	0	0	98	49	1	1	1	0.765	1	NA	1	-0.078
SE	4817	QKI	QKI	chr6	+	163563443	163563719	163561981	163562069	163566720	163566795	13	4	5	0	2	0	0	1	0	0	98	49	1	1	1	0.5	1	NA	1	-0.167
SE	4818	QKI	QKI	chr6	+	163566720	163566795	163563419	163563719	163570693	163578596	22	4	10	1	0	0	0	4	0	0	98	49	1	1	0.917	1	1	NA	1	-0.028
SE	4826	FRYL	FRYL	chr4	-	48539970	48540068	48535656	48535827	48540352	48540960	11	0	0	0	1	0	0	2	0	0	98	49	1	1	1	0	NA	NA	1	-0.5
SE	4827	FRYL	FRYL	chr4	-	48609743	48609823	48608986	48609067	48619273	48619370	19	7	0	0	0	0	0	3	0	1	98	49	1	1	1	1	NA	NA	0.6	0.4
SE	4828	FRYL	FRYL	chr4	-	48710518	48710698	48684672	48684795	48780077	48780299	5	9	0	3	0	1	0	1	0	0	98	49	1	1	0.455	1	0	NA	1	-0.515
SE	4831	CLEC7A	CLEC7A	chr12	-	10123244	10123363	10116780	10118590	10125296	10125448	141	62	38	12	2	1	0	38	0	8	98	49	1	1	0.855	0.939	0.95	NA	0.704	0.211
SE	4834	CLEC7A	CLEC7A	chr12	-	10125296	10125448	10123244	10123363	10127746	10127845	86	43	27	1	0	0	0	19	0	0	98	49	1	1	0.977	1	1	NA	1	-0.008
SE	4835	CLEC7A	CLEC7A	chr12	-	10126570	10126708	10125296	10125448	10127746	10127845	63	57	12	36	17	14	0	18	0	11	98	49	1	1	0.467	0.626	0.3	NA	0.45	0.014
SE	4836	CLEC7A	CLEC7A	chr12	-	10127746	10127845	10125296	10125448	10129979	10130269	234	91	50	2	0	0	6	55	0	0	98	49	1	1	0.983	1	1	1	1	-0.006
SE	4837	CLEC12A	CLEC12A	chr12	+	9978965	9979064	9971408	9971687	9979335	9979524	13	42	0	0	0	0	0	25	0	3	98	49	1	1	1	1	NA	NA	0.806	0.194
SE	4839	COMMD2	COMMD2	chr3	-	149750677	149750851	149738469	149741718	149751402	149751485	14	0	0	0	1	0	0	3	0	0	98	49	1	1	1	0	NA	NA	1	-0.5
SE	4843	CLEC2D	CLEC2D	chr12	+	9693033	9693115	9692827	9692931	9694759	9699555	11	1	1	8	4	2	0	1	0	2	98	49	1	1	0.407	0.111	0.2	NA	0.2	0.039
SE	4845	SH3BP1	SH3BP1	chr22	+	37641125	37641168	37639676	37639846	37641373	37641478	9	0	3	1	0	0	0	2	0	0	92	49	1	1	0.827	NA	1	NA	1	-0.087
SE	4846	SH3BP1	SH3BP1	chr22	+	37641373	37641478	37639676	37639846	37642538	37642615	11	6	0	0	1	0	0	4	0	0	98	49	1	1	1	0.75	NA	NA	1	-0.125
SE	4847	M6PR	M6PR	chr12	-	8943800	8943910	8943404	8943535	8945417	8945584	18	2	0	1	0	0	1	7	0	0	98	49	1	1	0.9	1	NA	1	1	-0.05
SE	4852	CLEC4E	CLEC4E	chr12	-	8536089	8536205	8533304	8534809	8539216	8539306	51	35	31	1	0	1	0	35	0	1	98	49	1	1	0.962	1	0.939	NA	0.946	0.021
SE	4853	CLEC4E	CLEC4E	chr12	-	8537114	8537266	8536089	8536205	8539216	8539306	31	39	26	2	0	0	1	35	0	0	98	49	1	1	0.886	1	1	1	1	-0.038
SE	4864	NECAP1	NECAP1	chr12	+	8091768	8091850	8090194	8090299	8092675	8092784	8	3	3	5	0	0	0	2	0	0	98	49	1	1	0.444	1	1	NA	1	-0.185
SE	4869	SLC2A3	SLC2A3	chr12	-	7922820	7923024	7919227	7921631	7924409	7924511	143	75	22	0	1	0	2	34	0	0	98	49	1	1	1	0.974	1	1	1	-0.009
SE	4870	SLC2A3	SLC2A3	chr12	-	7931244	7931485	7930479	7930642	7932986	7933147	217	146	65	0	0	1	12	78	0	0	98	49	1	1	1	1	0.97	1	1	-0.01
SE	4871	SLC2A3	SLC2A3	chr12	-	7933809	7933902	7932986	7933147	7936019	7936296	419	206	58	2	0	0	21	107	0	0	98	49	1	1	0.991	1	1	1	1	-0.003
SE	4876	ANKFY1	ANKFY1	chr17	-	4173353	4173444	4172555	4172680	4173908	4174056	2	0	3	2	4	0	0	2	0	0	98	49	1	1	0.333	0	1	NA	1	-0.556
SE	4877	ANKFY1	ANKFY1	chr17	-	4189381	4189479	4184817	4185046	4194977	4195177	11	7	0	0	0	0	0	0	0	1	98	49	1	1	1	1	NA	NA	0	1
SE	4878	ANKFY1	ANKFY1	chr17	-	4235771	4235890	4216982	4217118	4242255	4242448	13	6	6	2	3	0	0	6	0	0	98	49	1	1	0.765	0.5	1	NA	1	-0.245
SE	4882	FCHO2	FCHO2	chr5	+	73006444	73006549	72990711	72990864	73015625	73015724	19	7	1	1	0	0	0	6	0	0	98	49	1	1	0.905	1	1	NA	1	-0.032
SE	4884	FCHO2	FCHO2	chr5	+	73081782	73081982	73078179	73078312	73082760	73082825	15	9	11	0	1	0	2	0	0	0	98	49	1	1	1	0.818	1	1	NA	-0.061
SE	4887	C1RL-AS1	C1RL-AS1	chr12	+	7115385	7118677	7115129	7115219	7118898	7119026	2	5	0	3	0	0	0	1	0	0	98	49	1	1	0.25	1	NA	NA	1	-0.375
SE	4889	C1RL	C1RL	chr12	-	7108250	7108479	7101605	7102087	7109109	7109278	22	15	7	1	1	0	0	11	0	0	98	49	1	1	0.917	0.882	1	NA	1	-0.067
SE	4908	MLF2	MLF2	chr12	-	6751640	6751676	6750712	6750766	6751924	6752054	15	23	15	1	0	0	4	7	0	0	85	49	1	1	0.896	1	1	1	1	-0.035
SE	4909	BCR	BCR	chr22	+	23290338	23290413	23289516	23289621	23292540	23292638	7	0	3	0	1	0	0	3	0	0	98	49	1	1	1	0	1	NA	1	-0.333
SE	4912	ASAP1	ASAP1	chr8	-	130091972	130092143	130079901	130079971	130115627	130115735	5	5	2	1	0	0	1	0	0	0	98	49	1	1	0.714	1	1	1	NA	-0.095
SE	4913	ASAP1	ASAP1	chr8	-	130112093	130112322	130079901	130079971	130115627	130115735	37	7	0	1	0	0	0	12	0	0	98	49	1	1	0.949	1	NA	NA	1	-0.026
SE	4914	ASAP1	ASAP1	chr8	-	130112093	130112322	130091972	130092143	130115627	130115735	38	9	1	0	3	0	0	11	0	0	98	49	1	1	1	0.6	1	NA	1	-0.133
SE	4921	KTN1	KTN1	chr14	+	55663954	55664041	55661521	55661612	55667240	55667330	3	0	0	4	3	1	0	1	0	0	98	49	1	1	0.273	0	0	NA	1	-0.909
SE	4923	KTN1	KTN1	chr14	+	55680685	55680741	55679564	55679685	55684098	55684584	4	0	0	3	5	0	0	1	0	3	98	49	1	1	0.4	0	NA	NA	0.143	0.057
SE	4925	TLE4	TLE4	chr9	+	79612655	79612718	79576132	79576177	79627373	79627448	31	9	5	1	0	0	0	4	0	0	98	49	1	1	0.939	1	1	NA	1	-0.02
SE	4926	TLE4	TLE4	chr9	+	79627373	79627448	79612655	79612718	79652589	79652794	12	5	2	1	0	0	0	4	0	0	98	49	1	1	0.857	1	1	NA	1	-0.048
SE	4928	TLE4	TLE4	chr9	+	79671208	79671338	79654058	79654075	79704782	79704902	1	1	0	14	9	4	0	0	0	7	98	49	1	1	0.034	0.053	0	NA	0	0.029
SE	4930	TLE4	TLE4	chr9	+	79706746	79706899	79705888	79705942	79708117	79708250	41	9	2	3	0	0	0	9	0	0	98	49	1	1	0.872	1	1	NA	1	-0.043
SE	4931	TLE4	TLE4	chr9	+	79707122	79707218	79706746	79706899	79708117	79708250	0	0	2	18	5	1	0	0	0	2	98	49	1	1	0	0	0.5	NA	0	0.167
SE	4934	TNFRSF1A	TNFRSF1A	chr12	-	6330852	6330926	6330597	6330711	6333068	6333147	104	35	58	0	0	0	0	21	0	1	98	49	1	1	1	1	1	NA	0.913	0.087
SE	4935	TNFRSF1A	TNFRSF1A	chr12	-	6333736	6333865	6333366	6333516	6334090	6334244	131	58	49	1	0	0	0	16	0	0	98	49	1	1	0.985	1	1	NA	1	-0.005
SE	4938	UBE2D1	UBE2D1	chr10	+	58363608	58363686	58361494	58361526	58364770	58364876	42	8	17	1	0	0	1	16	0	0	98	49	1	1	0.955	1	1	1	1	-0.015
SE	4940	KIAA1033	KIAA1033	chr12	+	105126003	105126127	105122117	105122238	105127128	105127289	35	5	9	0	6	0	0	4	0	0	98	49	1	1	1	0.294	1	NA	1	-0.235
SE	4941	KIAA1033	KIAA1033	chr12	+	105126234	105126362	105126003	105126127	105127128	105127289	58	33	2	1	0	0	0	12	0	1	98	49	1	1	0.967	1	1	NA	0.857	0.132
SE	4943	KIAA1033	KIAA1033	chr12	+	105142452	105142558	105141166	105141246	105143126	105143243	36	8	10	1	4	0	0	10	0	0	98	49	1	1	0.947	0.5	1	NA	1	-0.184
SE	4945	KIAA1033	KIAA1033	chr12	+	105144286	105144455	105143126	105143243	105144717	105144872	69	24	10	0	3	0	0	26	0	0	98	49	1	1	1	0.8	1	NA	1	-0.067
SE	4946	KIAA1033	KIAA1033	chr12	+	105156725	105156792	105152342	105152451	105157235	105157322	44	32	4	3	0	0	0	5	0	0	98	49	1	1	0.88	1	1	NA	1	-0.04
SE	4947	KIAA1033	KIAA1033	chr12	+	105160000	105160148	105157235	105157322	105162748	105162845	69	19	4	2	0	0	1	14	0	0	98	49	1	1	0.945	1	1	1	1	-0.018
SE	4948	KIAA1033	KIAA1033	chr12	+	105162748	105162845	105160000	105160148	105164110	105164307	31	20	5	0	0	0	1	7	1	0	98	49	1	1	1	1	1	0.333	1	0.334
SE	4949	KIAA1033	KIAA1033	chr12	+	105164110	105164307	105162748	105162845	105164640	105164740	25	17	9	0	1	2	0	4	0	0	98	49	1	1	1	0.895	0.692	NA	1	-0.138
SE	4952	PARP11	PARP11	chr12	-	3863835	3863957	3829889	3830018	3873211	3873448	3	1	0	0	0	0	0	0	0	1	98	49	1	1	1	1	NA	NA	0	1
SE	4953	HERPUD1	HERPUD1	chr16	+	56935400	56935475	56935234	56935312	56936686	56936817	12	5	2	1	0	0	1	5	0	0	98	49	1	1	0.857	1	1	1	1	-0.048
SE	4954	HERPUD1	HERPUD1	chr16	+	56935403	56935475	56935234	56935312	56936686	56936817	9	1	1	1	0	0	1	1	0	0	98	49	1	1	0.818	1	1	1	1	-0.061
SE	4955	HERPUD1	HERPUD1	chr16	+	56939236	56939359	56936686	56936817	56939894	56940088	19	12	8	0	1	0	0	3	0	0	98	49	1	1	1	0.857	1	NA	1	-0.048
SE	4956	SLTM	SLTM	chr15	-	58890280	58890461	58889429	58889554	58892896	58893060	7	0	0	1	2	0	0	2	0	1	98	49	1	1	0.778	0	NA	NA	0.5	-0.111
SE	4957	SLTM	SLTM	chr15	-	58899468	58899883	58898802	58898852	58901259	58901287	7	1	0	4	0	0	1	0	0	0	98	49	1	1	0.467	1	NA	1	NA	-0.266
SE	4958	SLTM	SLTM	chr15	-	58899468	58899937	58898802	58898852	58901259	58901287	7	2	0	4	0	0	1	0	0	0	98	49	1	1	0.467	1	NA	1	NA	-0.266
SE	4959	SLTM	SLTM	chr15	-	58913498	58913696	58912562	58912610	58916934	58916999	51	28	23	1	0	0	0	6	0	0	98	49	1	1	0.962	1	1	NA	1	-0.013
SE	4972	FNIP1	FNIP1	chr5	-	131651801	131651999	131647089	131647205	131670462	131670631	49	19	9	0	2	0	1	3	0	0	98	49	1	1	1	0.826	1	1	1	-0.058

SE	4973	FNIP1	FNIP1	chr5	-	131677702	131677872	131671504	131672924	131679028	131679175	80	41	7	1	0	0	0	15	0	0	98	49	1	1	0.976	1	1	NA	1	-0.008
SE	4974	FNIP1	FNIP1	chr5	-	131679028	131679175	131677702	131677872	131698916	131699002	62	28	7	1	0	0	0	10	0	0	98	49	1	1	0.969	1	1	NA	1	-0.01
SE	4979	ERC1	ERC1	chr12	+	1371832	1371977	1289851	1290012	1408148	1408247	2	1	2	2	0	0	0	1	0	0	98	49	1	1	0.333	1	1	NA	1	-0.222
SE	4986	WNK1	WNK1	chr12	+	871264	871348	862082	862270	878211	878361	17	4	0	6	0	0	2	4	0	1	98	49	1	1	0.586	1	NA	1	0.667	-0.041
SE	4987	WNK1	WNK1	chr12	+	871264	871348	862082	862270	878214	878361	12	4	0	1	1	0	0	5	0	2	98	49	1	1	0.857	0.667	NA	NA	0.556	0.206
SE	5004	BAZ1A	BAZ1A	chr14	-	34785776	34786001	34783761	34783927	34786125	34786221	42	19	5	1	0	0	1	4	0	0	98	49	1	1	0.955	1	1	1	1	-0.015
SE	5005	BAZ1A	BAZ1A	chr14	-	34786125	34786221	34785776	34786001	34792774	34792921	16	9	6	0	0	3	0	4	0	2	98	49	1	1	1	1	0.5	NA	0.5	0.333
SE	5006	BAZ1A	BAZ1A	chr14	-	34795669	34795765	34794748	34794887	34800223	34800390	60	25	14	1	0	0	0	9	0	0	98	49	1	1	0.968	1	1	NA	1	-0.011
SE	5007	BAZ1A	BAZ1A	chr14	-	34826012	34826156	34810934	34811036	34874491	34874662	38	17	14	0	0	0	0	13	1	0	98	49	1	1	1	1	1	0	1	0.5
SE	5008	BAZ1A	BAZ1A	chr14	-	34862043	34862322	34826012	34826156	34874491	34874662	94	34	12	2	0	0	0	20	0	0	98	49	1	1	0.959	1	1	NA	1	-0.014
SE	5010	ACAD8	ACAD8	chr11	+	134257087	134257257	134256547	134256648	134258514	134258624	1	1	2	0	1	0	0	1	0	0	98	49	1	1	1	0.333	1	NA	1	-0.222
SE	5011	ACAD8	ACAD8	chr11	+	134261737	134261890	134261274	134261372	134262519	134262622	1	0	2	1	0	0	0	1	0	0	98	49	1	1	0.333	NA	1	NA	1	-0.334
SE	5015	KANSL3	KANSL3	chr2	-	96611063	96611138	96610725	96610883	96612281	96612353	1	6	0	1	0	0	0	0	0	1	98	49	1	1	0.333	1	NA	NA	0	0.666
SE	5018	NCAPD3	NCAPD3	chr11	-	134209144	134209206	134208863	134208951	134209312	134209477	4	1	0	0	0	0	0	0	1	0	98	49	1	1	1	1	NA	0	NA	1
SE	5023	AHCTF1	AHCTF1	chr1	-	246843794	246843928	246842693	246842776	246849614	246851442	12	1	5	3	1	1	0	0	0	2	98	49	1	1	0.667	0.333	0.714	NA	0	0.571
SE	5024	AHCTF1	AHCTF1	chr1	-	246857690	246857814	246855729	246855827	246860898	246861295	27	2	5	1	1	0	0	6	0	0	98	49	1	1	0.931	0.5	1	NA	1	-0.19
SE	5025	AHCTF1	AHCTF1	chr1	-	246863923	246864116	246861958	246862153	246867243	246867351	26	16	4	1	0	0	0	1	0	0	98	49	1	1	0.929	1	1	NA	1	-0.024
SE	5028	AHCTF1	AHCTF1	chr1	-	246916141	246916395	246913231	246913412	246918249	246918377	12	12	2	5	1	1	0	4	0	1	98	49	1	1	0.545	0.857	0.5	NA	0.667	-0.033
SE	5029	SPOP	SPOP	chr17	-	49636758	49636809	49622732	49622876	49668026	49668078	2	3	0	2	1	0	0	0	0	2	98	49	1	1	0.333	0.6	NA	NA	0	0.467
SE	5032	SPOP	SPOP	chr17	-	49668026	49668078	49622732	49622876	49677932	49678163	2	1	0	1	2	0	0	2	0	0	98	49	1	1	0.5	0.2	NA	NA	1	-0.65
SE	5039	APLP2	APLP2	chr11	+	130123611	130123779	130122304	130122513	130126699	130126830	64	27	9	18	8	3	0	33	0	3	98	49	1	1	0.64	0.628	0.6	NA	0.846	-0.223
SE	5048	ETS1	ETS1	chr11	-	128480190	128480451	128463508	128463627	128484822	128485071	27	8	5	0	0	1	2	2	0	0	98	49	1	1	1	1	0.714	1	1	-0.095
SE	5050	UNC45A	UNC45A	chr15	+	90942436	90942605	90940305	90940473	90942911	90943082	3	3	5	0	0	0	0	0	0	2	98	49	1	1	1	1	1	NA	0	1
SE	5054	ARAP1	ARAP1	chr11	-	72686041	72686191	72685068	72685681	72687438	72687502	120	66	39	2	0	0	2	18	0	0	98	49	1	1	0.968	1	1	1	1	-0.011
SE	5055	ARAP1	ARAP1	chr11	-	72686041	72686191	72685068	72685681	72687687	72687738	65	28	16	1	0	0	0	13	0	0	98	49	1	1	0.97	1	1	NA	1	-0.01
SE	5056	ARAP1	ARAP1	chr11	-	72687438	72687502	72685068	72685681	72687687	72687738	29	18	25	1	0	0	1	5	0	0	98	49	1	1	0.935	1	1	1	1	-0.022
SE	5057	ARAP1	ARAP1	chr11	-	72687438	72687502	72686041	72686191	72687687	72687738	83	56	48	2	0	0	3	10	0	0	98	49	1	1	0.954	1	1	1	1	-0.015
SE	5060	ARAP1	ARAP1	chr11	-	72697322	72697486	72696982	72697195	72697597	72697649	141	72	50	1	2	0	2	24	1	0	98	49	1	1	0.986	0.947	1	0.5	1	0.228
SE	5061	ARAP1	ARAP1	chr11	-	72697910	72698106	72697597	72697649	72699004	72699107	92	69	31	0	1	0	7	25	0	0	98	49	1	1	1	0.972	1	1	1	-0.009
SE	5063	ARAP1	ARAP1	chr11	-	72704151	72704334	72702904	72703079	72705804	72705890	79	69	42	6	2	3	0	25	0	0	98	49	1	1	0.868	0.945	0.875	NA	1	-0.104
SE	5065	ARAP1	ARAP1	chr11	-	72732514	72732597	72726619	72727172	72752327	72752389	18	20	1	1	0	0	2	8	0	0	98	49	1	1	0.9	1	1	1	1	-0.033
SE	5071	CYFIP1	CYFIP1	chr15	-	22873490	22873729	22872824	22872972	22874549	22874644	6	1	0	1	0	0	0	3	0	0	98	49	1	1	0.75	1	NA	NA	1	-0.125
SE	5074	PPP4R3B	PPP4R3B	chr2	-	55573618	55573777	55568193	55568363	55577314	55577356	19	2	7	2	0	0	0	10	0	0	98	49	1	1	0.826	1	1	NA	1	-0.058
SE	5075	PPP4R3B	PPP4R3B	chr2	-	55578246	55578342	55577314	55577356	55579678	55579781	3	0	1	6	0	0	0	0	1	1	98	49	1	1	0.2	NA	1	0	0	0.6
SE	5076	PPP4R3B	PPP4R3B	chr2	-	55586617	55586734	55585050	55585167	55588878	55588956	22	5	4	3	0	0	0	4	0	0	98	49	1	1	0.786	1	1	NA	1	-0.071
SE	5078	LOC100288778	LOC100288778	chr12	-	18372	18484	18036	18183	26800	26954	0	2	0	0	0	0	0	0	0	1	98	49	1	1	NA	1	NA	NA	0	1
SE	5089	ITGB7	ITGB7	chr12	-	53192690	53192910	53192329	53192538	53193139	53193363	4	1	1	0	0	0	0	0	0	1	98	49	1	1	1	1	1	NA	0	1
SE	5092	TMEM218	TMEM218	chr11	-	125102131	125102302	125101200	125101303	125102733	125102809	1	0	0	0	1	0	0	1	0	0	98	49	1	1	1	0	NA	NA	1	-0.5
SE	5094	TMEM218	TMEM218	chr11	-	125102131	125102317	125101200	125101303	125102733	125102809	1	0	0	0	1	0	0	1	0	0	98	49	1	1	1	0	NA	NA	1	-0.5
SE	5096	TMEM218	TMEM218	chr11	-	125102131	125102351	125101200	125101303	125102733	125102809	1	0	0	0	1	0	0	1	0	0	98	49	1	1	1	0	NA	NA	1	-0.5
SE	5102	GALC	GALC	chr14	-	87945552	87945733	87941394	87941558	87947727	87947878	7	2	1	0	0	0	0	3	0	1	98	49	1	1	1	1	1	NA	0.6	0.4
SE	5103	GALC	GALC	chr14	-	87988454	87988523	87988143	87988207	87992969	87993271	11	4	0	0	2	0	1	5	1	0	98	49	1	1	1	0.5	NA	0.333	1	0.084
SE	5113	FLI1	FLI1	chr11	+	128807179	128807239	128805365	128805431	128809156	128809204	38	15	23	0	0	0	1	11	0	1	98	49	1	1	1	1	1	1	0.846	0.077
SE	5114	N4BP1	N4BP1	chr16	-	48546146	48546254	48538725	48543261	48548006	48548114	24	3	5	0	0	0	0	2	0	1	98	49	1	1	1	1	1	NA	0.5	0.5
SE	5116	N4BP1	N4BP1	chr16	-	48560753	48562444	48553538	48553669	48609774	48610209	16	14	5	1	0	0	2	1	0	0	98	49	1	1	0.889	1	1	1	1	-0.037
SE	5122	HSPA8	HSPA8	chr11	-	123060592	123060798	123060115	123060268	123061119	123061329	3	0	5	2	0	0	0	1	0	0	98	49	1	1	0.429	NA	1	NA	1	-0.285
SE	5133	SNX17	SNX17	chr2	+	27373039	27373113	27372598	27372740	27373246	27373311	1	0	0	2	0	0	0	0	0	1	98	49	1	1	0.2	NA	NA	NA	0	0.2
SE	5134	SNX17	SNX17	chr2	+	27374688	27374758	27374345	27374433	27375060	27375153	23	4	3	0	3	0	0	6	0	0	98	49	1	1	1	0.4	1	NA	1	-0.2
SE	5135	MYL6	MYL6	chr12	+	56159586	56159730	56158683	56158711	56159974	56160148	149	85	76	1	0	0	7	24	0	1	98	49	1	1	0.987	1	1	1	0.923	0.034
SE	5138	VPS35	VPS35	chr16	-	46668929	46669052	46662982	46663162	46671704	46671860	18	10	11	0	0	1	3	6	0	0	98	49	1	1	1	1	0.846	1	1	-0.051
SE	5157	NLRX1	NLRX1	chr11	+	119172355	119172425	119171355	119171473	119172900	119172989	2	0	0	0	3	0	0	1	0	2	98	49	1	1	0.25	NA	NA	NA	0.2	0.05
SE	5166	RBM42	RBM42	chr19	+	35633686	35634019	35633076	35633252	35634255	35634373	5	2	2	0	0	0	2	0	1	0	98	49	1	1	1	1	1	0.5	NA	0.5
SE	5167	DDX6	DDX6	chr11	-	118758773	118758902	118757170	118757287	118759921	118760044	64	44	17	3	3	0	0	9	0	0	98	49	1	1	0.914	0.88	1	NA	1	-0.069
SE	5168	DDX6	DDX6	chr11	-	118765208	118765355	118763211	118763306	118768222	118768352	40	25	7	2	0	0	0	12	0	0	98	49	1	1	0.909	1	1	NA	1	-0.03
SE	5169	ARCN1	ARCN1	chr11	+	118581245	118581509	118572386	118572550	118583178	118583358	13	4	4	4	0	0	0	1	0	0	98	49	1	1	0.619	1	1	NA	1	-0.127
SE	5180	TSPAN32	TSPAN32	chr11	+	2308735	2308810	2304106	2304204	2313653	2313755	11	4	0	2	0	0	2	3	0	0	98	49	1	1	0.733	1	NA	1	1	-0.133
SE	5181	GPATCH8	GPATCH8	chr17	-	44464471	44464544	44436477	44436545	44474828	44474903	5	3	0	1	0	0	0	1	0	0	98	49	1	1	0.714	1	NA	NA	1	-0.143
SE	5184	MPZL3	MPZL3	chr11	-	118235423	118235589	118233459	118233523	118240210	118240377	36	6	6	0	1	1	0	1	0	0	98	49	1	1	1	0.75	0.75	NA	1	-0.167
SE	5185	MPZL3	MPZL3	chr11	-	118237049	118237260	118235423	118235589	118240210	118240341	13	9	14	4	1	1	0	2	0	0	98	49	1	1	0.619	0.818	0.875	NA	1	-0.229
SE	5189	OSGEP	OSGEP	chr14	-	20447903	20447994	20447614	20447690	20448105	20448171	10	0	1	4	0	0	0	2	0	0	98	49	1	1	0.556	NA	1	NA	1	-0.222
SE	5200	ARNTL	ARNTL	chr11	+	13349944	13350070	13309986	13310059	13354300	13354448	26	10	0	0	1	0	0	4	0	0	98	49	1	1	1	0.833	NA	NA	1	-0.084
SE	5201	ARNTL	ARNTL	chr11	+	13354300	13354448	13349944	13350070	13356738	13356777	28	6	1	0	0	5	0	9	0	0	98	49	1	1	1	1	0.091	NA	1	-0.303
SE	5202	ARNTL	ARNTL	chr11	+	13355225	13355296	13354300	13354448	13356738	13356777	1	0	0	10	1	1	0	1	0	5	98	49	1	1	0.048	0	0	NA	0.091	-0.075
SE	5203	ARNTL	ARNTL	chr11	+	13355232	13355296	13354300	13354448	13356738	13356777	0	0	0	10	1	1	0	1	0	5	98	49	1	1	0	0	0	NA	0.091	-0.091
SE	5204	ARNTL	ARNTL	chr11	+	13365499	13365592	13360344	13360424	13366664	13366782	16	12	3	0	1	0	0	1	0	2	98	49	1	1	1	0.857	1	NA	0.2	0.752
SE	5208	PTPRJ	PTPRJ	chr11	+	48144999	48145124	48144674	48144885	48146875	48146963	45	8	15	0	0	0	11	9	0	1	98	49	1	1	1	1	1	1	0.818	0.091
SE	5210	PTPRJ	PTPRJ	chr11	+	48164379	48164515	48163457	48163618	48167203	48170842	48	34	30	1	0	0	1	10	0	1	98	49	1	1	0.96	1	1	1	0.833	0.07
SE	5211	ETV6	ETV6	chr12	+	11752449	11752579	11649853	11650160	11839139	11839304	22	6	0	0	0	1	3	1	0	0	98	49	1	1	1	1	0	1	1	-0.333
SE	5216	LPGAT1	LPGAT1	chr1	-	211787631	211787727	211783228	211783502	211793071	211793190	60	46	19	0	1	1	0	15	0	0	98	49	1	1	1	0.958	0.905	NA	1	-0.046
SE	5217	PLEKHM2	PLEKHM2	chr1	+	15716706	15716816	15716236	15716343	15717892	15717992	5	4	3	2	0	0	0	3	0	0	98	49	1	1	0.556	1	1	NA	1	-0.148
SE	5218	PLEKHM2	PLEKHM2	chr1	+	15719733	15719920	15718537	15718625	15721328	15721388	12	9	5	0	2	0	1	2	0	0	98	49	1	1	1	0.692	1	1	1	-0.103
SE	5228	POLG	POLG	chr15	-	89322741	89322902	89321961	89322015	89323403	89323511	4	0	2	0	2	0	0	3	0	0	98	49	1	1	1	0	1	NA	1	-0.333
SE	5229	POLG	POLG	chr15	-	89323403	89323511	89322741	89322902	89323814	89323901	10	1	2	1	0	0	0	4	0	0	98	49	1	1	0.833	1	1	NA	1	-0.056
SE	5230	AKIP1	AKIP1	chr11	+	8912452	8912533	8911191	8911671	8914825	8914930	2	0	0	0	0	0	0	0	0	2	98	49	1	1	1	NA	NA	NA	0	1
SE	5232	SNX19	SNX19	chr11	-	130907952	130908083	130906624	130906721	130910017	130910137	4	4	1	1	0	0	0	1	0	0	98	49	1	1	0.667	1	1	NA	1	-0.111
SE	5240	TRAF3IP3	TRAF3IP3	chr1	+	209780469	209780606	209779314	209779374	209781344	209781458	10	3	0	3	0	0	0	3	0	0	98	49	1	1	0.625	1	NA	NA	1	-0.188
SE	5242	CHI3L1	CHI3L1	chr1	-	203183640	203183791	203182730	203182852	203184575	203184632	111	43	38	0	1	0	1	4	0	0	98	49	1	1	1	0.956	1	1	1	-0.015
SE	5244	PFKFB3	PFKFB3	chr10	+	6217134	6217191	6216705	6216780	6219568	6219693	70	45	12	0	0	0	2	31	0	1	98	49	1	1	1	1	1	1	0.939	0.03
SE	5245	PFKFB3	PFKFB3	chr10	+	6221640	6221745	6221380	6221527	6222854	6222984	73	49	6	2	0	0	16	35	0	0	98	49	1	1	0.948	1	1	1	1	-0.017
SE	5246	PFKFB3	PFKFB3	chr10	+	6221640	6221745	6221380	6221527	6223957	6224020	56	41	5	0	0	0	11	20	0	1	98	49	1	1	1	1	1	1	0.909	0.045
SE	5247	PFKFB3	PFKFB3	chr10	+	6222854	6222984	6221380	6221527	6223957	6224020	69	41	7	0	0	0	3	22	0	1	98	49	1	1	1	1	1	1	0.917	0.041
SE	5248	PFKFB3	PFKFB3	chr10	+	6222854	6222984	6221640	6221745	6223957	6224020	85	49	8	1	0	0	8	37	0	0	98	49	1	1	0.977	1	1	1	1	-0.008
SE	5249	PFKFB3	PFKFB3	chr10	+	6224148	6224213	6223957	6224020	6226191	6226365	78	53	4	2	0	0	10	43	0	0	98	49	1	1	0.951	1	1	1	1	-0.016
SE	5252	PTPRC	PTPRC	chr1	+	198696711	198696909	198692346	198692373	198699563	198699704	12	10	0	43	17	16	0	1	0	12	98	49	1	1	0.122	0.227	0	NA	0.04	0.076
SE	5255	PTPRC	PTPRC	chr1	+	198709686	198709824	198708132	198708261	198712952	198713072	276	128	85	1	0	0	0	36	0	0	98	49	1	1	0.993	1	1	NA	1	-0.002
SE	5256	PTPRC	PTPRC	chr1	+	198741868	198742026	198735126	198735252	198742231	198742367	553	282	135	3	0	0	20	106	0	0	98	49	1	1	0.989	1	1	1	1	-0.004
SE	5257	PTPRC	PTPRC	chr1	+	198742231	198742367	198741868	198742026	198744053	198744203	562	258	160	1	0	0	17	98	0	0	98	49	1	1	0.996	1	1	1	1	-0.001
SE	5258	PTPRC	PTPRC	chr1	+	198749415	198749549	198748108	198748199	198750491	198750626	477	276	181	2	3	0	1	72	0	1	98	49	1	1	0.992	0.979	1	1	0.973	0.004
SE	5266	ARID3B	ARID3B	chr15	+	74573131	74573204	74572861	74572933	74589819	74590003	0	0	0	1	0	0	0	1	0	0	98	49	1	1	0	NA	NA	NA	1	-1
SE	5268	TATDN3	TATDN3	chr1	+	212802700	212802763	212797111	212797196	212804319	212804429	4	2	5	0	1	0	0	2	0	1	98	49	1	1	1	0.5	1	NA	0.5	0.333
SE	5269	MARK2	MARK2	chr11	+	63902600	63902782	63902197	63902330	63903060	63903158	13	1	3	2	0	0	0	3	0	0	98	49	1	1	0.765	1	1	NA	1	-0.078
SE	5270	MARK2	MARK2	chr11	+	63902603	63902782	63902197	63902330	63903060	63903158	37	5	11	2	0	0	0	9	0	0	98	49	1	1	0.902	1	1	NA	1	-0.033
SE	5271	MARK2	MARK2	chr11	+	63903985	63904147	63903060	63903158	63904785	63905043	8	5	2	10	6	0	0	1	0	0	98	49	1	1	0.286	0.294	1	NA	1	-0.473
SE	5272	MARK2	MARK2	chr11	+	63906087	63906114	63904785	63905043	63908259	63908304	2	0	0	18	2	1	0	0	0	1	76	49	1	1	0.067	0	0	NA	0	0.022
SE	5284	ZNRD1	ZNRD1	chr6	+	30061916	30062017	30061239	30061669	30062223	30062333	7	2	2	1	0	3	0	2	0	0	98	49	1	1	0.778	1	0.25	NA	1	-0.324
SE	5286	CALML4	CALML4	chr15	-	68199640	68199681	68197439	68197628	68205120	68205151	3	0	0	2	0	0	0	2	0	0	98	49	1	1	0.429	NA	NA	NA	1	-0.571
SE	5290	ZRANB1	ZRANB1	chr10	+	124974199	124974398	124973644	124973716	124981708	124981829	14	1	3	0	0	0	0	1	0	1	98	49	1	1	1	1	1	NA	0	1
SE	5291	ZRANB1	ZRANB1	chr10	+	124981708	124981829	124974199	124974398	124983174	124983304	18	11	2	1	0	0	1	1	0	1	98	49	1	1	0.9	1	1	1	0.333	0.3
SE	5292	ZRANB1	ZRANB1	chr10	+	124983174	124983304	124974199	124974398	124984773	124987436	3	0	0	0	1	0	0	2	0	0	98	49	1	1	1	0	NA	NA	1	-0.5
SE	5293	ZRANB1	ZRANB1	chr10	+	124983458	124983688	124983174	124983304	124984773	124987436	10	12	6	2	0	0	0	0	0	1	98	49	1	1	0.714	1	1	NA	0	0.905
SE	5294	TGFB1	TGFB1	chr19	-	41341882	41342030	41332127	41332281	41342169	41342247	75	17	8	1	0	0	1	23	0	0	98	49	1	1	0.974	1	1	1	1	-0.009
SE	5304	PTPN7	PTPN7	chr1	-	202153724	202153835	202152541	202152699	202155532	202155609	7	2	2	0	0	0	0	1	0	2	98	49	1	1	1	1	1	NA	0.2	0.8
SE	5305	PTPN7	PTPN7	chr1	-	202154185	202154323	202153724	202153835	202155532	202155609	9	2	6	1	0	0	1	0	0	0	98	49	1	1	0.818	1	1	1	NA	-0.061
SE	5310	PPFIA1	PPFIA1	chr11	+	70348188	70348420	70343668	70343892	70354300	70354452	21	2	4	1	0	0	0	3	0	0	98	49	1	1	0.913	1	1	NA	1	-0.029
SE	5312	PPFIA1	PPFIA1	chr11	+	70382087	70382158	70378029	70378195	70383002	70384501	18	2	1	3	6	1	0	4	0	0	98	49	1	1	0.75	0.143	0.333	NA	1	-0.591
SE	5313	NDUFV1	NDUFV1	chr11	+	67611896	67611978	67611402	67611569	67612119	67612265	11	2	1	1	0	0	0	5	0	0	98	49	1	1	0.846	1	1	NA	1	-0.051
SE	5317	GATAD2A	GATAD2A	chr19	+	19501968	19502043	19501117	19501416	19502330	19502526	14	3	4	0	4	0	0	3	0	0	98	49	1	1	1	0.273	1	NA	1	-0.242
SE	5325	BAHD1	BAHD1	chr15	+	40461911	40462294	40458450	40459896	40463860	40464020	1	0	0	0	1	0	0	1	0	0	98	49	1	1	1	0	NA	NA	1	-0.5
SE	5343	SF3B1	SF3B1	chr2	-	197409769	197410007	197408368	197408581	197416740	197416911	141	48	38	0	1	0	2	16	0	0	98	49	1	1	1	0.96	1	1	1	-0.013
SE	5353	NBPF10	NBPF10	chr1	-	146133522	146133728	146132185	146132237	146134192	146134265	15	0	0	2	0	0	0	4	0	0	98	49	1	1	0.789	NA	NA	NA	1	-0.211
SE	5355	TAB2	TAB2	chr6	+	149399103	149399184	149397968	149398062	149409576	149411613	59	22	4	1	0	0	1	9	0	0	98	49	1	1	0.967	1	1	1	1	-0.011
SE	5356	CXCL8	CXCL8	chr4	+	73741541	73741677	73740505	73740722	73741948	73742032	1882	3505	450	5	7	0	26	133	1	0	98	49	1	1	0.995	0.996	1	0.929	1	0.032
SE	5362	TNRC18	TNRC18	chr7	-	5361593	5361722	5359397	5359569	5361896	5362033	24	5	1	0	3	3	0	4	0	2	98	49	1	1	1	0.455	0.143	NA	0.5	0.033
SE	5364	TROVE2	TROVE2	chr1	+	193069033	193069634	193059421	193059776	193075819	193076040	2	0	0	3	2	0	0	4	0	1	98	49	1	1	0.25	0	NA	NA	0.667	-0.542
SE	5372	RGS2	RGS2	chr1	+	192810165	192810267	192809038	192809181	192810369	192810431	1491	624	334	1	0	0	11	186	0	0	98	49	1	1	0.999	1	1	1	1	0
SE	5374	RGS2	RGS2	chr1	+	192810980	192811147	192810369	192810431	192811401	192812277	1435	717	501	0	1	1	8	199	0	0	98	49	1	1	1	0.997	0.996	1	1	-0.002
SE	5377	SLC9A8	SLC9A8	chr20	+	49839540	49839599	49823060	49823141	49845035	49845119	31	5	3	0	0	0	0	8	0	1	98	49	1	1	1	1	1	NA	0.8	0.2
SE	5379	SLC9A8	SLC9A8	chr20	+	49849578	49849680	49845035	49845119	49855437	49855581	32	13	0	3	0	0	0	10	0	2	98	49	1	1	0.842	1	NA	NA	0.714	0.207
SE	5380	SLC9A8	SLC9A8	chr20	+	49850761	49850844	49849578	49849680	49855437	49855581	7	4	0	14	9	0	0	4	0	4	98	49	1	1	0.2	0.182	NA	NA	0.333	-0.142
SE	5381	SLC9A8	SLC9A8	chr20	+	49850809	49850844	49849578	49849680	49855437	49855581	7	6	0	14	9	0	0	4	0	4	84	49	1	1	0.226	0.28	NA	NA	0.368	-0.115
SE	5382	SLC9A8	SLC9A8	chr20	+	49874704	49874821	49864727	49864844	49877980	49878063	23	10	8	2	0	0	0	4	0	0	98	49	1	1	0.852	1	1	NA	1	-0.049
SE	5388	RRM2B	RRM2B	chr8	-	102224884	102225018	102224045	102224140	102225917	102226034	32	21	15	0	2	0	0	4	0	2	98	49	1	1	1	0.84	1	NA	0.5	0.447
SE	5389	RRM2B	RRM2B	chr8	-	102232148	102232304	102225917	102226034	102238826	102239118	24	7	1	0	0	2	0	1	0	1	98	49	1	1	1	1	0.2	NA	1	-0.267
SE	5392	TMEM126B	TMEM126B	chr11	+	85631144	85631316	85628572	85628688	85631686	85631808	2	3	0	1	0	0	0	2	0	0	98	49	1	1	0.5	1	NA	NA	1	-0.25
SE	5405	RASGRP1	RASGRP1	chr15	-	38501142	38501287	38500102	38500139	38502311	38502421	5	5	4	1	0	0	0	1	0	0	98	49	1	1	0.714	1	1	NA	1	-0.095
SE	5406	RASGRP1	RASGRP1	chr15	-	38503271	38503376	38502311	38502421	38505839	38505920	9	1	3	0	1	0	0	2	0	1	98	49	1	1	1	0.333	1	NA	1	-0.222
SE	5408	NCF2	NCF2	chr1	-	183565703	183565779	183564004	183564030	183566919	183566988	308	136	154	1	0	0	16	51	0	0	98	49	1	1	0.994	1	1	1	1	-0.002
SE	5409	NCF2	NCF2	chr1	-	183566919	183566988	183565703	183565779	183567203	183567345	228	117	141	1	0	0	12	47	0	0	98	49	1	1	0.991	1	1	1	1	-0.003
SE	5413	NCF2	NCF2	chr1	-	183569141	183569185	183565703	183565779	183570779	183570839	247	138	71	2	0	0	6	30	0	0	93	49	1	1	0.985	1	1	1	1	-0.005
SE	5414	NCF2	NCF2	chr1	-	183573184	183573292	183570779	183570839	183574486	183574621	417	212	150	0	0	0	5	63	0	1	98	49	1	1	1	1	1	1	0.969	0.016
SE	5416	NCF2	NCF2	chr1	-	183574486	183574621	183570779	183570839	183577598	183577707	118	56	89	3	2	0	0	30	0	1	98	49	1	1	0.952	0.933	1	NA	0.937	0.025
SE	5417	NCF2	NCF2	chr1	-	183574486	183574621	183573184	183573292	183577598	183577707	255	137	159	24	16	0	0	51	0	12	98	49	1	1	0.842	0.811	1	NA	0.68	0.204
SE	5418	NCF2	NCF2	chr1	-	183577598	183577707	183573184	183573292	183586894	183586977	182	104	98	0	1	0	3	42	0	0	98	49	1	1	1	0.981	1	1	1	-0.006
SE	5419	NCF2	NCF2	chr1	-	183586894	183586977	183577598	183577707	183590155	183590359	468	224	221	1	0	0	9	109	0	0	98	49	1	1	0.996	1	1	1	1	-0.001
SE	5426	HBEGF	HBEGF	chr5	-	140345910	140346084	140342634	140342812	140346282	140346603	4	0	0	0	0	0	0	5	0	1	98	49	1	1	1	NA	NA	NA	0.714	0.286
SE	5427	PFKFB4	PFKFB4	chr3	-	48539253	48539310	48538497	48538619	48539696	48539771	20	7	2	1	1	0	0	7	0	1	98	49	1	1	0.909	0.778	1	NA	0.778	0.118
SE	5431	SMU1	SMU1	chr9	-	33053122	33053290	33048105	33048258	33056112	33056239	13	2	0	1	0	0	0	1	0	0	98	49	1	1	0.867	1	NA	NA	1	-0.067
SE	5432	SMU1	SMU1	chr9	-	33071739	33071892	33068823	33068934	33073595	33073806	3	2	0	0	0	0	0	1	0	2	98	49	1	1	1	1	NA	NA	0.2	0.8
SE	5433	GLIPR1	GLIPR1	chr12	+	75490405	75490518	75481833	75482079	75495576	75495662	60	35	15	0	0	0	0	15	0	1	98	49	1	1	1	1	1	NA	0.882	0.118
SE	5435	FAR2	FAR2	chr12	+	29293299	29293475	29270411	29270638	29297020	29297200	20	6	11	1	0	0	0	1	0	0	98	49	1	1	0.909	1	1	NA	1	-0.03
SE	5436	FAR2	FAR2	chr12	+	29297020	29297200	29293299	29293475	29307657	29307835	29	16	12	0	0	0	0	1	0	1	98	49	1	1	1	1	1	NA	0.333	0.667
SE	5437	FAR2	FAR2	chr12	+	29316840	29317012	29311882	29311950	29321794	29321924	9	0	1	0	0	0	0	1	0	1	98	49	1	1	1	NA	1	NA	0.333	0.667
SE	5438	TIMM8B	TIMM8B	chr11	-	112085977	112086310	112084810	112085462	112086639	112086798	0	0	0	0	0	3	0	1	0	0	98	49	1	1	NA	NA	0	NA	1	-1
SE	5439	RASGRP4	RASGRP4	chr19	-	38414847	38414916	38413393	38413474	38417051	38417168	17	10	3	1	0	0	0	3	0	0	98	49	1	1	0.895	1	1	NA	1	-0.035
SE	5440	RASGRP4	RASGRP4	chr19	-	38414847	38415123	38413393	38413474	38417051	38417168	56	20	10	1	0	0	2	16	0	0	98	49	1	1	0.966	1	1	1	1	-0.011
SE	5446	BCL6	BCL6	chr3	-	187728359	187728544	187726730	187726898	187729049	187730021	81	30	25	0	0	0	2	24	0	1	98	49	1	1	1	1	1	1	0.923	0.038
SE	5447	RALGPS2	RALGPS2	chr1	+	178821611	178821704	178811314	178811404	178833423	178833550	8	1	5	1	0	0	0	1	0	0	98	49	1	1	0.8	1	1	NA	1	-0.067
SE	5448	RALGPS2	RALGPS2	chr1	+	178892229	178892307	178889641	178889696	178893918	178894024	6	0	5	1	1	0	0	0	0	1	98	49	1	1	0.75	0	1	NA	0	0.583
SE	5449	RALGPS2	RALGPS2	chr1	+	178906775	178906867	178902105	178902211	178916329	178921842	8	6	0	1	0	0	0	1	0	0	98	49	1	1	0.8	1	NA	NA	1	-0.1
SE	5460	SAMD8	SAMD8	chr10	+	75168540	75168658	75164644	75164740	75176065	75176216	11	10	2	1	0	0	0	2	0	1	98	49	1	1	0.846	1	1	NA	0.5	0.449
SE	5461	LOC374443	LOC374443	chr12	+	9653682	9653808	9648046	9648933	9655872	9655979	16	0	0	0	3	0	0	5	0	0	98	49	1	1	1	0	NA	NA	1	-0.5
SE	5464	LOC374443	LOC374443	chr12	+	9656567	9656649	9655872	9655979	9656920	9658414	8	0	1	7	0	2	0	0	0	3	98	49	1	1	0.364	NA	0.2	NA	0	0.282
SE	5465	LOC374443	LOC374443	chr12	+	9656567	9656697	9655872	9655979	9656920	9658414	7	1	2	7	0	2	0	0	0	3	98	49	1	1	0.333	1	0.333	NA	0	0.555
SE	5466	RC3H1	RC3H1	chr1	-	173943441	173943588	173941264	173941380	173946475	173946608	6	6	6	2	3	0	0	2	0	3	98	49	1	1	0.6	0.5	1	NA	0.25	0.45
SE	5467	RC3H1	RC3H1	chr1	-	173943441	173943615	173941264	173941380	173946475	173946608	11	3	2	2	3	0	0	2	0	3	98	49	1	1	0.733	0.333	1	NA	0.25	0.439

SE	5468	RC3H1	RC3H1	chr1	-	173951985	173952138	173947368	173947582	173961076	173961244	35	12	1	0	0	0	0	9	0	1	98	49	1	1	1	1	1	NA		0.818	0.182
SE	5469	RC3H1	RC3H1	chr1	-	173964838	173965120	173963972	173964187	173970504	173970617	27	19	2	0	0	0	0	5	0	1	98	49	1	1	1	1	1	NA		0.714	0.286
SE	5470	RC3H1	RC3H1	chr1	-	173972508	173972627	173970504	173970617	173978487	173978620	28	12	2	1	0	0	0	14	0	0	98	49	1	1	0.933	1	1	NA		1	-0.022
SE	5471	RC3H1	RC3H1	chr1	-	173983417	173983657	173982726	173982902	173984498	173984619	71	13	14	0	0	0	1	7	0	1	98	49	1	1	1	1	1		1	0.778	0.111
SE	5480	DNAJB12	DNAJB12	chr10	-	72338201	72338311	72336523	72336696	72340788	72340868	11	7	3	0	0	0	0	2	0	1	98	49	1	1	1	1	1	NA		0.5	0.5
SE	5485	TCIRG1	TCIRG1	chr11	+	68043370	68043497	68042945	68043031	68043570	68043653	76	29	21	1	0	0	2	17	0	0	98	49	1	1	0.974	1	1		1	1	-0.009
SE	5487	ZHX1-C8orf76	ZHX1-C8orf76	chr8	-	123231299	123231757	123226188	123226632	123237797	123237941	4	0	0	0	1	1	0	3	0	0	98	49	1	1	1	0	0	NA		1	-0.667
SE	5491	YWHAQ	YWHAQ	chr2	-	9588164	9588328	9587413	9587509	9591391	9591515	25	2	4	0	2	0	0	10	0	0	98	49	1	1	1	0.333	1	NA		1	-0.222
SE	5492	OGFRL1	OGFRL1	chr6	+	71296316	71296395	71293532	71293611	71296494	71296561	170	67	42	1	0	0	0	15	0	0	98	49	1	1	0.988	1	1	NA		1	-0.004
SE	5493	OGFRL1	OGFRL1	chr6	+	71296494	71296561	71293532	71293611	71296671	71296817	81	39	29	1	0	0	0	17	0	0	98	49	1	1	0.976	1	1	NA		1	-0.008
SE	5495	SYNE1	SYNE1	chr6	-	152145486	152145555	152143622	152143765	152148044	152148378	5	0	2	1	0	0	2	2	0	0	98	49	1	1	0.714	NA	1		1	1	-0.143
SE	5498	SYNE1	SYNE1	chr6	-	152325957	152326102	152325083	152325302	152326295	152326633	10	7	2	1	0	0	0	5	0	0	98	49	1	1	0.833	1	1	NA		1	-0.056
SE	5500	SYNE1	SYNE1	chr6	-	152352026	152352353	152350617	152350770	152353262	152353433	9	12	0	0	1	0	0	2	0	0	98	49	1	1	1	0.857	NA	NA		1	-0.072
SE	5502	SYNE1	SYNE1	chr6	-	152396774	152396980	152395515	152395671	152398618	152398731	12	5	1	1	0	0	0	2	0	0	98	49	1	1	0.857	1	1	NA		1	-0.048
SE	5512	RPAIN	RPAIN	chr17	+	5425970	5426082	5422768	5422829	5432541	5433020	0	2	0	0	1	0	0	2	0	1	98	49	1	1	NA	0.5	NA	NA		0.5	0
SE	5517	RPAIN	RPAIN	chr17	+	5428070	5428211	5422768	5422829	5432541	5433020	2	0	0	0	1	0	0	0	0	1	98	49	1	1	1	0	NA	NA		0	0.5
SE	5527	AMBRA1	AMBRA1	chr11	-	46512726	46512813	46508190	46508370	46541944	46542981	1	1	2	1	0	0	0	1	0	1	98	49	1	1	0.333	1	1	NA		0.333	0.445
SE	5529	MICALL1	MICALL1	chr22	+	37927410	37927826	37925660	37926043	37931798	37931933	1	2	0	0	0	0	0	0	0	1	98	49	1	1	1	1	NA	NA		0	1
SE	5537	PDE4B	PDE4B	chr1	+	66361614	66361793	66355526	66355620	66363167	66363266	55	43	14	1	0	0	0	8	0	0	98	49	1	1	0.965	1	1	NA		1	-0.012
SE	5538	PADI4	PADI4	chr1	+	17354532	17354687	17347940	17348048	17355982	17356127	50	18	47	1	0	0	4	8	0	0	98	49	1	1	0.962	1	1		1	1	-0.013
SE	5539	PADI4	PADI4	chr1	+	17355982	17356127	17347940	17348048	17356356	17356459	24	5	8	1	1	0	0	6	0	0	98	49	1	1	0.923	0.714	1	NA		1	-0.121
SE	5548	CRLF3	CRLF3	chr17	-	30793449	30793672	30792439	30792572	30796159	30796337	57	14	18	0	1	0	0	4	0	0	98	49	1	1	1	0.875	1	NA		1	-0.042
SE	5554	NAAA	NAAA	chr4	-	75918760	75918789	75914867	75914985	75919908	75919975	2	0	0	0	0	0	0	0	0	2	78	49	1	1	1	NA	NA	NA		0	1
SE	5555	FCGR1B	FCGR1B	chr1	+	121094673	121094958	121092995	121093250	121095871	121097161	0	0	0	10	3	6	0	2	0	0	98	49	1	1	0	0	0	NA		1	-1
SE	5556	TAF2	TAF2	chr8	-	119793365	119793451	119791323	119791459	119795531	119795631	10	3	1	0	0	0	0	1	0	2	98	49	1	1	1	1	1	NA		0.2	0.8
SE	5559	SDHAF2	SDHAF2	chr11	+	61437624	61437848	61430124	61430182	61438003	61438113	6	4	0	1	0	0	0	4	0	0	98	49	1	1	0.75	1	NA	NA		1	-0.125
SE	5560	SDHAF2	SDHAF2	chr11	+	61438003	61438113	61430124	61430182	61445940	61446767	3	1	6	1	1	1	0	2	0	0	98	49	1	1	0.6	0.333	0.75	NA		1	-0.439
SE	5561	KIAA0141	KIAA0141	chr5	+	141937197	141937357	141934493	141934586	141938520	141938760	10	4	0	1	0	0	0	2	0	0	98	49	1	1	0.833	1	NA	NA		1	-0.084
SE	5567	CERS2	CERS2	chr1	-	150968082	150968201	150967819	150967877	150968394	150968512	15	14	8	1	2	0	0	16	0	1	98	49	1	1	0.882	0.778	1	NA		0.889	-0.002
SE	5581	NSUN2	NSUN2	chr5	-	6622015	6622100	6620105	6620298	6623213	6623285	9	2	1	3	0	0	0	1	0	2	98	49	1	1	0.6	1	1	NA		0.2	0.667
SE	5583	ROCK2	ROCK2	chr2	-	11222082	11222174	11221197	11221357	11224321	11224460	12	3	2	0	0	4	0	10	0	0	98	49	1	1	1	1	0.2	NA		1	-0.267
SE	5592	COPA	COPA	chr1	-	160294483	160294593	160293385	160293463	160294767	160294857	14	5	0	0	0	0	0	3	0	2	98	49	1	1	1	1	NA	NA		0.429	0.571
SE	5593	COPA	COPA	chr1	-	160307162	160307245	160306353	160306493	160309100	160309176	16	5	7	2	0	0	0	6	0	0	98	49	1	1	0.8	1	1	NA		1	-0.067
SE	5594	COPA	COPA	chr1	-	160333602	160333679	160332447	160332557	160335241	160335322	17	6	3	0	2	0	0	4	0	0	98	49	1	1	1	0.6	1	NA		1	-0.133
SE	5600	DCAF8	DCAF8	chr1	-	160243959	160244034	160239221	160240370	160261284	160261325	8	2	0	1	0	0	0	3	0	0	98	49	1	1	0.8	1	NA	NA		1	-0.1
SE	5601	DCAF8	DCAF8	chr1	-	160243959	160244034	160239221	160240370	160262448	160262560	4	2	0	1	0	0	0	3	0	0	98	49	1	1	0.667	1	NA	NA		1	-0.166
SE	5611	KCNJ15	KCNJ15	chr21	+	38291605	38291747	38229914	38230023	38296925	38297023	35	2	4	8	0	0	0	2	0	0	98	49	1	1	0.686	1	1	NA		1	-0.105
SE	5612	KCNJ15	KCNJ15	chr21	+	38291605	38291747	38257045	38257185	38296925	38297023	40	2	3	8	2	2	0	2	0	0	98	49	1	1	0.714	0.333	0.429	NA		1	-0.508
SE	5613	KCNJ15	KCNJ15	chr21	+	38298143	38298282	38296925	38297023	38299243	38301824	15	1	0	39	7	14	0	1	0	7	98	49	1	1	0.161	0.067	0	NA		0.067	0.009
SE	5619	VTI1A	VTI1A	chr10	+	112527086	112527164	112464546	112464657	112538245	112538330	21	7	1	4	0	0	0	13	0	0	98	49	1	1	0.724	1	1	NA		1	-0.092
SE	5622	AIM1	AIM1	chr6	+	106525132	106525180	106519130	106521453	106525267	106525386	23	8	4	0	1	0	0	11	0	0	97	49	1	1	1	0.802	1	NA		1	-0.066
SE	5627	CD74	CD74	chr5	-	150406880	150406960	150406258	150406321	150407151	150407324	145	8	14	1	1	0	7	68	0	1	98	49	1	1	0.986	0.8	1		1	0.971	-0.057
SE	5628	MORF4L1	MORF4L1	chr15	+	78884966	78885083	78880511	78880579	78886140	78886227	3	5	0	17	10	0	0	0	0	5	98	49	1	1	0.081	0.2	NA	NA		0	0.141
SE	5629	MORF4L1	MORF4L1	chr15	+	78892211	78892313	78891483	78891572	78893538	78893627	18	8	2	1	0	0	1	4	0	0	98	49	1	1	0.9	1	1		1	1	-0.033
SE	5630	MORF4L1	MORF4L1	chr15	+	78894057	78894230	78893538	78893627	78894819	78894904	27	7	4	1	3	0	0	7	0	3	98	49	1	1	0.931	0.538	1	NA		0.538	0.285
SE	5636	MNDA	MNDA	chr1	+	158843954	158844122	158843278	158843415	158845586	158846003	605	393	302	0	0	3	8	82	0	0	98	49	1	1	1	1	0.981		1	1	-0.006
SE	5637	MNDA	MNDA	chr1	+	158845586	158846003	158843954	158844122	158847727	158847916	679	552	400	4	1	4	6	105	0	0	98	49	1	1	0.988	0.996	0.98		1	1	-0.012
SE	5642	METTL9	METTL9	chr16	+	21612644	21612835	21599475	21599898	21617864	21618074	40	22	15	1	0	0	0	16	0	0	98	49	1	1	0.952	1	1	NA		1	-0.016
SE	5644	XPR1	XPR1	chr1	+	180803387	180803611	180787752	180787854	180806061	180806211	2	2	3	0	0	2	0	1	0	0	98	49	1	1	1	1	0.429	NA		1	-0.19
SE	5647	INO80	INO80	chr15	-	41074369	41074565	41073427	41073495	41079700	41079904	6	3	0	1	0	0	0	1	0	0	98	49	1	1	0.75	1	NA	NA		1	-0.125
SE	5655	WDFY3	WDFY3	chr4	-	84702352	84702506	84696731	84696823	84704337	84704444	35	12	9	1	0	0	0	21	0	0	98	49	1	1	0.946	1	1	NA		1	-0.018
SE	5656	WDFY3	WDFY3	chr4	-	84705393	84705511	84704337	84704444	84708908	84709028	53	17	2	2	1	0	0	11	0	1	98	49	1	1	0.93	0.895	1	NA		0.846	0.096
SE	5659	WDFY3	WDFY3	chr4	-	84765809	84766027	84756925	84757161	84766251	84766372	51	16	10	0	1	0	0	8	0	0	98	49	1	1	1	0.889	1	NA		1	-0.037
SE	5660	WDFY3	WDFY3	chr4	-	84796520	84796752	84794878	84794979	84797995	84798108	75	23	16	1	0	0	1	13	0	0	98	49	1	1	0.974	1	1		1	1	-0.009
SE	5661	WDFY3	WDFY3	chr4	-	84808333	84808417	84803289	84803467	84809886	84810344	0	3	0	30	7	0	0	0	0	5	98	49	1	1	0	0.176	NA	NA		0	0.088
SE	5662	WDFY3	WDFY3	chr4	-	84817391	84817585	84809886	84810344	84820084	84820186	100	28	9	2	0	0	1	19	1	0	98	49	1	1	0.962	1	1		0.333	1	0.321
SE	5669	PPP2R5A	PPP2R5A	chr1	+	212329134	212329331	212285536	212286291	212333496	212333598	62	24	18	2	0	0	0	17	0	0	98	49	1	1	0.939	1	1	NA		1	-0.02
SE	5680	SELP	SELP	chr1	-	169593604	169593724	169591425	169591456	169594691	169594877	4	0	0	0	0	0	1	0	0	1	98	49	1	1	NA		NA		1	0	0.5
SE	5683	PPP1R12A	PPP1R12A	chr12	-	79786373	79786478	79781814	79781862	79788647	79788783	15	8	11	0	1	0	0	2	0	0	98	49	1	1	1	0.8	1	NA		1	-0.067
SE	5684	PPP1R12A	PPP1R12A	chr12	-	79805591	79805768	79798493	79798584	79806165	79806333	14	2	0	17	6	2	0	2	0	4	98	49	1	1	0.292	0.143	0	NA		0.2	-0.055
SE	5685	PPP1R12A	PPP1R12A	chr12	-	79806165	79806333	79805591	79805768	79807225	79807330	12	6	1	1	0	0	0	4	0	0	98	49	1	1	0.857	1	1	NA		1	-0.048
SE	5686	PPP1R12A	PPP1R12A	chr12	-	79822115	79822190	79821077	79821166	79828319	79828464	40	19	8	2	0	0	0	13	0	0	98	49	1	1	0.909	1	1	NA		1	-0.03
SE	5696	SERPINA1	SERPINA1	chr14	-	94379463	94379611	94376746	94378640	94390456	94390692	142	65	104	2	0	0	6	51	0	0	98	49	1	1	0.973	1	1		1	1	-0.009
SE	5697	SERPINA1	SERPINA1	chr14	-	94382591	94383241	94376746	94378640	94390456	94390692	108	35	38	2	0	0	2	20	0	0	98	49	1	1	0.964	1	1		1	1	-0.012
SE	5698	SERPINA1	SERPINA1	chr14	-	94388559	94388660	94379463	94379611	94390456	94390692	34	6	10	1	0	0	1	3	0	0	98	49	1	1	0.944	1	1		1	1	-0.019
SE	5700	SERPINA1	SERPINA1	chr14	-	94388559	94388663	94379463	94379611	94390456	94390692	29	19	5	1	0	0	0	4	0	0	98	49	1	1	0.935	1	1	NA		1	-0.022
SE	5701	SERPINA1	SERPINA1	chr14	-	94388559	94388663	94382591	94383241	94390419	94390692	69	15	37	3	0	0	0	13	0	0	98	49	1	1	0.92	1	1	NA		1	-0.027
SE	5703	SERPINA1	SERPINA1	chr14	-	94388800	94389010	94376746	94378640	94390456	94390692	47	13	5	2	0	0	1	15	0	0	98	49	1	1	0.922	1	1		1	1	-0.026
SE	5717	ARPC3	ARPC3	chr12	-	110436556	110436683	110436109	110436204	110437083	110437152	164	84	70	0	1	0	3	30	0	0	98	49	1	1	1	0.977	1		1	1	-0.008
SE	5719	KIAA1324	KIAA1324	chr1	+	109173687	109173837	109173473	109173579	109185044	109185133	20	2	12	1	2	0	0	1	0	0	98	49	1	1	0.909	0.333	1	NA		1	-0.253
SE	5720	KIAA1324	KIAA1324	chr1	+	109185044	109185133	109173687	109173837	109188176	109188354	23	12	1	1	0	1	0	1	0	0	98	49	1	1	0.92	1	0.333	NA		1	-0.249
SE	5721	KIAA1324	KIAA1324	chr1	+	109197978	109198075	109197473	109197654	109198572	109198674	26	8	24	0	2	0	0	1	0	0	98	49	1	1	1	0.667	1	NA		1	-0.111
SE	5723	LILRB3	LILRB3	chr19	-	54222746	54222782	54222277	54222562	54222942	54223087	28	15	3	1	1	0	4	6	0	0	85	49	1	1	0.942	0.896	1		1	1	-0.054
SE	5726	WARS	WARS	chr14	-	100360553	100360662	100354446	100354566	100361707	100361921	16	5	3	0	1	0	0	7	0	0	98	49	1	1	1	0.714	1	NA		1	-0.095
SE	5739	WDR7	WDR7	chr18	+	56717963	56718159	56696241	56696462	56731382	56731597	25	5	1	2	0	0	0	2	0	0	98	49	1	1	0.862	1	1	NA		1	-0.046
SE	5741	EYA3	EYA3	chr1	-	27998283	27998351	27997319	27997378	27999959	28000049	5	3	0	0	0	0	0	0	0	1	98	49	1	1	1	1	NA	NA		0	1
SE	5749	TMEM50A	TMEM50A	chr1	+	25342960	25343073	25340473	25340579	25351625	25351693	39	35	20	0	1	0	0	3	0	0	98	49	1	1	1	0.946	1	NA		1	-0.018
SE	5752	TPR	TPR	chr1	-	186317481	186317600	186314628	186314724	186318446	186318603	24	14	7	0	2	0	0	4	0	0	98	49	1	1	1	0.778	1	NA		1	-0.074
SE	5753	TPR	TPR	chr1	-	186335067	186335129	186334324	186334533	186335337	186335543	68	24	15	1	0	0	1	10	0	0	98	49	1	1	0.971	1	1		1	1	-0.01
SE	5754	TPR	TPR	chr1	-	186339641	186339772	186338032	186338243	186341027	186341159	58	25	7	1	0	0	0	1	0	0	98	49	1	1	0.967	1	1	NA		1	-0.011
SE	5755	TPR	TPR	chr1	-	186353687	186353850	186351975	186352110	186355409	186355558	72	36	3	0	2	0	0	5	0	0	98	49	1	1	1	0.9	1	NA		1	-0.033
SE	5756	TPR	TPR	chr1	-	186361621	186361709	186360764	186360905	186361788	186361869	28	15	2	0	2	0	0	5	0	0	98	49	1	1	1	0.789	1	NA		1	-0.07
SE	5757	TPR	TPR	chr1	-	186367885	186367982	186363341	186363445	186370969	186371043	53	11	7	1	0	0	0	3	0	0	98	49	1	1	0.964	1	1	NA		1	-0.012
SE	5767	SCAMP3	SCAMP3	chr1	-	155261656	155261734	155260536	155260659	155262085	155262385	0	1	3	2	0	0	0	1	0	0	98	49	1	1	0	1	1	NA		1	-0.333
SE	5778	TBC1D2B	TBC1D2B	chr15	-	78017846	78017957	78016545	78016739	78024155	78024539	5	3	4	1	0	0	0	2	0	0	98	49	1	1	0.714	1	1	NA		1	-0.095
SE	5786	SRSF10	SRSF10	chr1	-	23971337	23971439	23964346	23967723	23971572	23971626	4	1	0	4	0	2	0	1	0	0	98	49	1	1	0.333	1	0	NA		1	-0.556
SE	5793	RARA	RARA	chr17	+	40330856	40331396	40318220	40318448	40348315	40348464	34	14	3	1	0	0	1	16	0	0	98	49	1	1	0.944	1	1		1	1	-0.019
SE	5794	KIAA1109	KIAA1109	chr4	+	122173035	122173152	122170602	122170744	122174573	122174652	28	23	5	2	0	0	1	7	0	0	98	49	1	1	0.875	1	1		1	1	-0.042
SE	5795	KIAA1109	KIAA1109	chr4	+	122224497	122224677	122220332	122220470	122229120	122229240	13	13	4	0	1	0	0	2	0	0	98	49	1	1	1	0.867	1	NA		1	-0.044
SE	5796	KIAA1109	KIAA1109	chr4	+	122226706	122226835	122224497	122224677	122229120	122229240	32	18	2	7	3	0	0	1	0	1	98	49	1	1	0.696	0.75	1	NA		0.333	0.482
SE	5797	KIAA1109	KIAA1109	chr4	+	122234772	122235023	122229930	122230211	122238091	122238350	41	11	2	3	1	0	0	9	0	0	98	49	1	1	0.872	0.846	1	NA		1	-0.094
SE	5798	KIAA1109	KIAA1109	chr4	+	122275971	122276022	122274342	122274436	122279774	122279981	2	7	0	6	3	3	0	4	0	0	98	49	1	1	0.143	0.538	0	NA		1	-0.773
SE	5799	KIAA1109	KIAA1109	chr4	+	122356620	122356755	122355795	122355990	122359556	122359722	43	19	10	0	0	0	0	14	0	1	98	49	1	1	1	1	1	NA		0.875	0.125
SE	5800	WDR26	WDR26	chr1	-	224393827	224394013	224385142	224389860	224398096	224398226	44	22	8	3	2	0	0	9	0	0	98	49	1	1	0.88	0.846	1	NA		1	-0.091
SE	5801	WDR26	WDR26	chr1	-	224398096	224398226	224385142	224389860	224398514	224398593	13	14	3	1	0	0	0	4	0	0	98	49	1	1	0.867	1	1	NA		1	-0.044
SE	5802	WDR26	WDR26	chr1	-	224398888	224399034	224398514	224398593	224400949	224401069	49	24	15	1	0	0	0	5	0	1	98	49	1	1	0.961	1	1	NA		0.714	0.273
SE	5803	WDR26	WDR26	chr1	-	224418259	224418416	224411426	224411565	224419517	224419615	50	26	15	1	6	0	0	16	0	0	98	49	1	1	0.962	0.684	1	NA		1	-0.118
SE	5804	WDR26	WDR26	chr1	-	224424517	224424654	224419517	224419615	224431476	224431581	23	25	1	0	1	0	2	12	0	0	98	49	1	1	1	0.926	1		1	1	-0.025
SE	5805	WDR26	WDR26	chr1	-	224424517	224424654	224419517	224419615	224431524	224431581	26	27	2	1	1	0	2	15	0	0	98	49	1	1	0.929	0.931	1		1	1	-0.047
SE	5806	WDR26	WDR26	chr1	-	224431681	224431781	224431476	224431581	224433683	224434299	44	51	5	2	0	0	1	6	0	1	98	49	1	1	0.917	1	1		1	0.75	0.097
SE	5807	CCDC9	CCDC9	chr19	+	47270406	47270453	47266610	47266792	47270552	47270688	5	14	0	2	0	0	0	3	0	0	96	49	1	1	0.561	1	NA	NA		1	-0.22
SE	5813	CHTOP	CHTOP	chr1	+	153643226	153643364	153642242	153642429	153645063	153646306	2	3	2	0	0	1	0	1	0	0	98	49	1	1	1	1	0.5	NA		1	-0.167
SE	5814	EDRF1	EDRF1	chr10	+	125729002	125729104	125725681	125725838	125729357	125729479	2	0	0	0	0	2	0	0	0	1	98	49	1	1	1	NA	0	NA		0	0.5
SE	5823	WAPL	WAPL	chr10	-	86472611	86472764	86472207	86472344	86473877	86473973	25	14	10	5	1	0	0	8	0	0	98	49	1	1	0.714	0.875	1	NA		1	-0.137
SE	5824	WAPL	WAPL	chr10	-	86497200	86497319	86473877	86473973	86499717	86500743	20	6	4	2	0	0	0	1	0	0	98	49	1	1	0.833	1	1	NA		1	-0.056
SE	5825	WAPL	WAPL	chr10	-	86517570	86518091	86499717	86500743	86521364	86521784	23	19	8	1	0	0	0	2	0	0	98	49	1	1	0.92	1	1	NA		1	-0.027
SE	5834	ITGB1	ITGB1	chr10	-	32932514	32932600	32929821	32930044	32935491	32935558	11	0	2	0	0	0	1	2	0	1	98	49	1	1	1	NA	1		1	0.5	0.25
SE	5835	STX3	STX3	chr11	+	59787036	59787136	59773210	59773294	59788872	59788947	101	67	28	6	1	0	0	14	0	0	98	49	1	1	0.894	0.971	1	NA		1	-0.045
SE	5836	STX3	STX3	chr11	+	59787036	59787136	59773210	59773294	59790518	59790586	38	20	10	1	0	0	0	6	0	0	98	49	1	1	0.95	1	1	NA		1	-0.017
SE	5837	STX3	STX3	chr11	+	59788872	59788947	59773210	59773294	59790518	59790586	57	26	14	1	0	0	1	6	0	0	98	49	1	1	0.966	1	1		1	1	-0.011
SE	5838	STX3	STX3	chr11	+	59788872	59788947	59787036	59787136	59790518	59790586	114	73	32	0	1	0	1	14	0	0	98	49	1	1	1	0.973	1		1	1	-0.009
SE	5839	STX3	STX3	chr11	+	59795371	59795482	59793379	59793514	59797282	59797396	46	23	13	1	0	0	0	8	0	0	98	49	1	1	0.958	1	1	NA		1	-0.014
SE	5840	STX3	STX3	chr11	+	59797282	59797396	59795371	59795482	59800854	59805882	45	25	21	3	3	2	0	8	0	0	98	49	1	1	0.882	0.806	0.84	NA		1	-0.157
SE	5854	SNX27	SNX27	chr1	+	151662165	151662270	151660797	151660862	151665932	151666011	41	18	10	3	3	1	0	9	0	0	98	49	1	1	0.872	0.75	0.833	NA		1	-0.182
SE	5855	SNX27	SNX27	chr1	+	151665932	151666011	151662165	151662270	151668471	151668635	44	26	6	2	0	0	0	9	0	0	98	49	1	1	0.917	1	1	NA		1	-0.028
SE	5862	CTNNA1	CTNNA1	chr5	+	138783176	138783372	138781922	138782029	138810037	138810204	15	3	0	0	1	0	0	12	0	0	98	49	1	1	1	0.6	NA	NA		1	-0.2
SE	5866	PIP5K1A	PIP5K1A	chr1	+	151242122	151242269	151239954	151240039	151242437	151242567	1	0	0	0	0	0	0	0	0	1	98	49	1	1	1	NA	NA	NA		0	1
SE	5869	PLEKHJ1	PLEKHJ1	chr19	-	2234149	2234240	2233997	2234061	2235761	2235828	6	6	2	0	0	0	0	0	0	1	98	49	1	1	1	1	1	NA		0	1
SE	5870	ZDHHC13	ZDHHC13	chr11	+	19142977	19143123	19117144	19117276	19146180	19146303	2	0	0	1	0	0	0	2	0	0	98	49	1	1	0.5	NA	NA	NA		1	-0.5
SE	5871	ZDHHC13	ZDHHC13	chr11	+	19166301	19166385	19165051	19165145	19170410	19170568	2	2	0	2	0	0	0	2	0	0	98	49	1	1	0.333	1	NA	NA		1	-0.334
SE	5887	ATXN3	ATXN3	chr14	-	92093745	92093831	92093251	92093318	92096092	92096137	10	2	0	3	1	0	0	1	0	0	98	49	1	1	0.625	0.5	NA	NA		1	-0.438
SE	5901	FAM63A	FAM63A	chr1	-	150998081	150998273	150997623	150997779	150999368	150999511	27	10	8	2	0	0	0	1	0	0	98	49	1	1	0.871	1	1	NA		1	-0.043
SE	5902	FAM63A	FAM63A	chr1	-	151000456	151000615	150999861	150999964	151001249	151001314	10	8	14	2	0	0	0	4	0	0	98	49	1	1	0.714	1	1	NA		1	-0.095
SE	5904	RPN2	RPN2	chr20	+	37232295	37232391	37229972	37230059	37234019	37234095	19	0	5	0	0	0	0	2	0	1	98	49	1	1	1	NA	1	NA		0.5	0.5
SE	5910	TP53BP2	TP53BP2	chr1	-	223818205	223818337	223814239	223814353	223821219	223821367	0	3	0	2	1	2	0	1	0	3	98	49	1	1	0	0.6	0	NA		0.143	0.057
SE	5924	CDIPT	CDIPT	chr16	-	29859441	29859523	29858355	29859334	29860580	29860662	14	7	8	1	0	2	0	5	0	0	98	49	1	1	0.875	1	0.667	NA		1	-0.153
SE	5925	CDIPT	CDIPT	chr16	-	29862585	29862635	29861105	29861259	29862814	29863288	12	5	3	1	0	0	0	2	0	0	98	49	1	1	0.857	1	1	NA		1	-0.048
SE	5926	CDIPT	CDIPT	chr16	-	29862585	29862720	29861105	29861259	29862814	29863288	19	5	8	1	0	0	0	2	0	0	98	49	1	1	0.905	1	1	NA		1	-0.032
SE	5930	GDI2	GDI2	chr10	-	5794884	5795019	5785851	5786050	5796762	5796862	32	5	12	3	0	0	0	7	0	0	98	49	1	1	0.842	1	1	NA		1	-0.053
SE	5932	CREBBP	CREBBP	chr16	-	3738558	3738672	3736649	3736815	3739577	3739724	32	5	6	1	0	0	0	4	0	0	98	49	1	1	0.941	1	1	NA		1	-0.02
SE	5933	CREBBP	CREBBP	chr16	-	3739577	3739724	3736649	3736815	3740398	3740549	31	3	4	0	1	0	0	3	0	3	98	49	1	1	1	0.6	1	NA		0.333	0.534
SE	5934	CREBBP	CREBBP	chr16	-	3777612	3777657	3774568	3774693	3778010	3778182	55	33	6	1	0	0	1	13	0	0	94	49	1	1	0.966	1	1		1	1	-0.011
SE	5935	CREBBP	CREBBP	chr16	-	3778699	3778817	3778010	3778182	3780731	3780878	61	17	8	0	1	0	1	10	0	0	98	49	1	1	1	0.895	1		1	1	-0.035
SE	5936	CREBBP	CREBBP	chr16	-	3780731	3780878	3778699	3778817	3781203	3781306	72	14	3	0	0	0	1	9	0	1	98	49	1	1	1	1	1		1	0.818	0.091
SE	5937	CREBBP	CREBBP	chr16	-	3791980	3792094	3782683	3782926	3793385	3793626	57	18	12	1	0	0	1	9	0	0	98	49	1	1	0.966	1	1		1	1	-0.011
SE	5940	C11orf30	C11orf30	chr11	+	76459932	76460085	76458182	76458358	76463820	76464080	5	0	0	0	1	0	0	1	0	0	98	49	1	1	1	0	NA	NA		1	-0.5
SE	5941	C11orf30	C11orf30	chr11	+	76459935	76460085	76458182	76458358	76463820	76464080	4	0	0	0	1	0	0	1	0	0	98	49	1	1	1	0	NA	NA		1	-0.5
SE	5949	SORL1	SORL1	chr11	+	121478117	121478243	121470006	121470123	121488031	121488193	344	191	88	1	1	0	3	54	0	0	98	49	1	1	0.994	0.99	1		1	1	-0.005
SE	5950	SORL1	SORL1	chr11	+	121488031	121488193	121478117	121478243	121490042	121490110	458	227	186	3	2	0	0	79	0	0	98	49	1	1	0.987	0.983	1	NA		1	-0.01
SE	5951	SORL1	SORL1	chr11	+	121490042	121490110	121478117	121478243	121496868	121497049	317	113	79	1	0	0	5	41	0	0	98	49	1	1	0.994	1	1		1	1	-0.002
SE	5952	SORL1	SORL1	chr11	+	121513002	121513104	121496868	121497049	121514151	121514321	463	284	111	0	1	0	3	110	0	0	98	49	1	1	1	0.993	1		1	1	-0.002
SE	5953	SORL1	SORL1	chr11	+	121543547	121543726	121532463	121532552	121545242	121545429	373	144	88	1	0	0	4	58	0	0	98	49	1	1	0.995	1	1		1	1	-0.002
SE	5954	SORL1	SORL1	chr11	+	121545242	121545429	121543547	121543726	121549959	121550088	389	148	91	0	0	0	6	54	0	1	98	49	1	1	1	1	1		1	0.964	0.018
SE	5955	SORL1	SORL1	chr11	+	121549959	121550088	121545242	121545429	121550584	121550670	332	120	72	1	3	0	2	33	0	0	98	49	1	1	0.994	0.952	1		1	1	-0.018
SE	5956	SORL1	SORL1	chr11	+	121550584	121550670	121545242	121545429	121553936	121554109	163	138	47	1	0	0	0	27	0	0	98	49	1	1	0.988	1	1	NA		1	-0.004
SE	5957	SORL1	SORL1	chr11	+	121553936	121554109	121545242	121545429	121555186	121555318	291	134	57	2	0	0	10	39	0	0	98	49	1	1	0.986	1	1		1	1	-0.005
SE	5958	SORL1	SORL1	chr11	+	121555186	121555318	121545242	121545429	121558590	121558837	17	4	3	1	0	0	0	2	0	0	98	49	1	1	0.895	1	1	NA		1	-0.035
SE	5961	SORL1	SORL1	chr11	+	121570156	121570270	121566939	121567113	121583457	121583583	170	45	28	3	0	0	0	32	0	0	98	49	1	1	0.966	1	1	NA		1	-0.011
SE	5963	SORL1	SORL1	chr11	+	121574240	121574363	121570156	121570270	121583457	121583583	349	131	68	1	0	0	0	27	0	0	98	49	1	1	0.994	1	1	NA		1	-0.002
SE	5965	SORL1	SORL1	chr11	+	121605401	121605571	121605112	121605239	121606844	121606957	524	184	145	2	4	0	11	57	0	1	98	49	1	1	0.992	0.958	1		1	0.966	0
SE	5966	SORL1	SORL1	chr11	+	121608103	121608176	121607185	121607290	121611075	121611158	260	99	77	1	2	0	0	31	0	1	98	49	1	1	0.992	0.961	1	NA		0.939	0.045
SE	5967	SORL1	SORL1	chr11	+	121611075	121611158	121608103	121608176	121612735	121612832	425	149	106	0	1	0	0	40	0	0	98	49	1	1	1	0.987	1	NA		1	-0.004
SE	5968	SORL1	SORL1	chr11	+	121625084	121625277	121622161	121622268	121627554	121627767	236	96	58	0	1	0	1	33	0	0	98	49	1	1	1	0.98	1		1	1	-0.007
SE	5974	CDK11B	CDK11B	chr1	-	1637080	1637202	1636896	1637004	1637407	1637513	12	0	4	0	0	0	1	0	2	0	98	49	1	1	NA		1		0.2	NA	0.8
SE	5979	FLII	FLII	chr17	-	18246307	18246462	18246161	18246222	18246593	18246828	50	23	10	0	1	0	2	0	0	0	98	49	1	1	1	0.92	1		1	NA	-0.027

SE	5980	FLII	FLII	chr17	-	18253543	18253719	18253300	18253458	18254078	18254182	9	3	0	3	0	0	3	1	0	0	98	49	1	1	0.6	1	NA	1	1	-0.2
SE	5982	FLII	FLII	chr17	-	18256525	18256597	18255182	18255263	18256908	18257019	44	20	6	2	0	0	7	15	0	0	98	49	1	1	0.917	1	1	1	1	-0.028
SE	5990	RPS6KB2	RPS6KB2	chr11	+	67431367	67431515	67429526	67429595	67432599	67432657	10	2	2	2	0	0	0	4	0	0	98	49	1	1	0.714	1	1	NA	1	-0.095
SE	5992	RPS6KB2	RPS6KB2	chr11	+	67433125	67433216	67432951	67433042	67433339	67433447	7	0	2	0	0	0	0	0	0	1	98	49	1	1	1	NA	1	NA	0	1
SE	5993	DPP9	DPP9	chr19	-	4695377	4695555	4694660	4694823	4697550	4697651	4	0	0	1	0	0	0	4	0	0	98	49	1	1	0.667	NA	NA	NA	1	-0.333
SE	5994	DPP9	DPP9	chr19	-	4702602	4702716	4702026	4702155	4703885	4704054	1	0	0	1	0	0	2	0	0	0	98	49	1	1	0.333	NA	NA	1	NA	-0.667
SE	5999	CHD1L	CHD1L	chr1	+	147270931	147271005	147268781	147268878	147272170	147272281	0	2	0	0	0	0	0	1	0	1	98	49	1	1	NA	1	NA	NA	0.333	0.667
SE	6001	NIN	NIN	chr14	-	50756491	50758630	50754741	50754867	50759856	50760359	17	9	0	4	2	1	0	2	0	0	98	49	1	1	0.68	0.692	0	NA	1	-0.543
SE	6002	NIN	NIN	chr14	-	50759856	50760359	50754741	50754867	50761789	50761911	21	18	9	5	1	1	0	2	0	2	98	49	1	1	0.677	0.9	0.818	NA	0.333	0.465
SE	6003	NIN	NIN	chr14	-	50766306	50766396	50763825	50763964	50766779	50766890	59	27	16	5	3	1	0	14	0	1	98	49	1	1	0.855	0.818	0.889	NA	0.875	-0.021
SE	6004	NIN	NIN	chr14	-	50772300	50772468	50771331	50771468	50772948	50773095	68	22	15	1	1	0	1	12	0	0	98	49	1	1	0.971	0.917	1	1	1	-0.037
SE	6006	NIN	NIN	chr14	-	50830463	50830517	50821873	50822077	50831005	50831121	29	13	2	0	0	0	3	3	0	1	98	49	1	1	1	1	1	1	0.6	0.2
SE	6008	FOS	FOS	chr14	+	75279876	75280128	75278777	75279123	75280559	75280667	1366	319	192	0	0	0	18	153	0	1	98	49	1	1	1	1	1	1	0.987	0.006
SE	6009	FOS	FOS	chr14	+	75280559	75280667	75279876	75280128	75280782	75282234	1634	373	175	5	1	2	25	189	1	0	98	49	1	1	0.994	0.995	0.978	0.926	1	0.026
SE	6019	KSR1	KSR1	chr17	+	27601359	27601401	27597267	27597436	27603833	27603888	10	0	0	2	0	0	0	2	0	0	91	49	1	1	0.729	NA	NA	NA	1	-0.271
SE	6026	ZNF185	ZNF185	chrX	+	152922719	152922809	152922172	152922256	152928574	152928661	5	1	2	4	1	0	1	0	0	0	98	49	1	1	0.385	0.333	1	1	NA	-0.427
SE	6027	ZNF185	ZNF185	chrX	+	152922719	152922809	152922172	152922256	152931671	152931776	5	3	0	1	0	0	1	1	0	0	98	49	1	1	0.714	1	NA	1	1	-0.143
SE	6029	ZNF185	ZNF185	chrX	+	152928574	152928661	152922719	152922809	152931671	152931776	7	2	2	0	3	0	0	0	0	1	98	49	1	1	1	0.25	1	NA	0	0.75
SE	6040	USPL1	USPL1	chr13	+	30653147	30653305	30646931	30647057	30657473	30659549	5	5	2	1	0	0	0	1	0	0	98	49	1	1	0.714	1	1	NA	1	-0.095
SE	6052	NUDT5	NUDT5	chr10	-	12170899	12170908	12170716	12170770	12172764	12172866	0	1	2	0	1	0	0	1	0	0	58	49	1	1	NA	0.458	1	NA	1	-0.271
SE	6054	ALPK1	ALPK1	chr4	+	112423943	112424003	112411826	112412025	112425664	112425751	24	15	8	2	2	0	0	6	0	0	98	49	1	1	0.857	0.789	1	NA	1	-0.118
SE	6055	ALPK1	ALPK1	chr4	+	112430447	112432581	112429148	112429253	112435147	112435301	29	4	8	1	0	0	1	3	0	0	98	49	1	1	0.935	1	1	1	1	-0.022
SE	6058	TBC1D10B	TBC1D10B	chr16	-	30359171	30359361	30358662	30358817	30359537	30359603	10	2	2	4	2	0	0	2	0	0	98	49	1	1	0.556	0.333	1	NA	1	-0.37
SE	6060	THRAP3	THRAP3	chr1	+	36296582	36296770	36293850	36293935	36300885	36301084	26	18	7	0	1	0	0	6	0	0	98	49	1	1	1	0.9	1	NA	1	-0.033
SE	6061	THRAP3	THRAP3	chr1	+	36301552	36301696	36300885	36301084	36303795	36305356	27	24	11	0	0	0	0	3	0	2	98	49	1	1	1	1	1	NA	0.429	0.571
SE	6062	HLA-DMB	HLA-DMB	chr6	-	32935535	32935652	32935341	32935377	32937171	32937456	9	0	0	0	0	0	0	4	0	1	98	49	1	1	1	NA	NA	NA	0.667	0.333
SE	6064	NOTCH2	NOTCH2	chr1	-	119922635	119922778	119922235	119922446	119923636	119923984	68	17	10	0	0	0	2	5	0	1	98	49	1	1	1	1	1	1	0.714	0.143
SE	6065	NOTCH2	NOTCH2	chr1	-	119923636	119923984	119922235	119922446	119925304	119925810	38	11	1	0	2	0	0	6	0	0	98	49	1	1	1	0.733	1	NA	1	-0.089
SE	6066	NOTCH2	NOTCH2	chr1	-	119925304	119925810	119923636	119923984	119926498	119926611	77	31	7	1	0	0	0	20	0	0	98	49	1	1	0.975	1	1	NA	1	-0.008
SE	6067	NOTCH2	NOTCH2	chr1	-	119959391	119959502	119955039	119955232	119963573	119963807	70	12	14	7	1	0	0	4	0	1	98	49	1	1	0.833	0.857	1	NA	0.667	0.23
SE	6068	NOTCH2	NOTCH2	chr1	-	119968076	119968232	119967432	119967621	119969510	119969744	106	47	22	1	1	0	1	16	0	0	98	49	1	1	0.981	0.959	1	1	1	-0.02
SE	6069	NOTCH2	NOTCH2	chr1	-	120029905	120029987	120005328	120005588	120069333	120069703	50	22	2	0	1	0	0	9	0	0	98	49	1	1	1	0.917	1	NA	1	-0.028
SE	6070	FAM13A	FAM13A	chr4	-	88737471	88737555	88732001	88732198	88739029	88739125	2	0	0	0	0	0	0	0	0	2	98	49	1	1	1	NA	NA	NA	0	1
SE	6074	CLIP1	CLIP1	chr12	-	122347374	122347479	122340752	122341697	122352725	122352786	2	2	0	4	3	0	0	1	0	0	98	49	1	1	0.2	0.25	NA	NA	1	-0.775
SE	6076	CLIP1	CLIP1	chr12	-	122355114	122355312	122354452	122354556	122360958	122361181	19	15	0	0	2	0	0	7	0	0	98	49	1	1	1	0.789	NA	NA	1	-0.105
SE	6091	C1orf162	C1orf162	chr1	+	111477650	111477775	111477333	111477428	111477982	111478512	3	1	0	0	0	0	1	9	0	1	98	49	1	1	1	1	NA	1	0.818	0.091
SE	6092	C1orf162	C1orf162	chr1	+	111477725	111477775	111477333	111477428	111477982	111478512	12	1	0	0	0	0	1	9	0	1	98	49	1	1	1	1	NA	1	0.818	0.091
SE	6098	UBE2K	UBE2K	chr4	+	39755656	39755739	39745751	39745810	39774833	39774933	12	8	4	0	0	0	0	2	0	1	98	49	1	1	1	1	1	NA	0.5	0.5
SE	6106	ITPR2	ITPR2	chr12	-	26659112	26659285	26658010	26658130	26663684	26663846	16	6	1	0	2	0	0	6	0	0	98	49	1	1	1	0.6	1	NA	1	-0.133
SE	6109	ECT2	ECT2	chr3	+	172755482	172755575	172755294	172755374	172756982	172757165	1	0	0	0	0	0	0	0	1	0	98	49	1	1	1	NA	NA	0	NA	1
SE	6110	GAB2	GAB2	chr11	-	78220318	78220444	78215289	78219415	78221676	78221779	23	15	18	2	0	0	1	4	0	0	98	49	1	1	0.852	1	1	1	1	-0.049
SE	6111	GAB2	GAB2	chr11	-	78250156	78250400	78226464	78227051	78280600	78280901	52	32	17	2	0	0	4	13	0	0	98	49	1	1	0.929	1	1	1	1	-0.024
SE	6118	CUTC	CUTC	chr10	+	99750368	99750396	99747256	99747390	99754528	99754634	10	6	0	1	0	0	0	0	0	3	77	49	1	1	0.864	1	NA	NA	0	0.932
SE	6119	SENP1	SENP1	chr12	-	48074689	48074793	48074323	48074607	48083590	48083762	7	1	0	0	1	0	0	2	0	0	98	49	1	1	1	0.333	NA	NA	1	-0.334
SE	6120	SENP1	SENP1	chr12	-	48089162	48089293	48088800	48088960	48096342	48096427	0	1	0	2	0	0	0	0	0	1	98	49	1	1	0	1	NA	NA	0	0.5
SE	6121	TIPRL	TIPRL	chr1	+	168191368	168191500	168184778	168184878	168196546	168196642	11	8	6	0	1	0	0	2	0	0	98	49	1	1	1	0.8	1	NA	1	-0.067
SE	6123	NUMB	NUMB	chr14	-	73279280	73279424	73275209	73277293	73282358	73282505	0	0	0	24	8	2	0	2	0	10	98	49	1	1	0	0	0	NA	0.091	-0.091
SE	6124	NUMB	NUMB	chr14	-	73284080	73284374	73282358	73282505	73287109	73287314	45	12	18	5	0	0	0	6	0	0	98	49	1	1	0.818	1	1	NA	1	-0.061
SE	6128	KDSR	KDSR	chr18	-	63335256	63335358	63327737	63331901	63338799	63338883	6	0	0	2	0	0	0	1	0	0	98	49	1	1	0.6	NA	NA	NA	1	-0.4
SE	6134	CD55	CD55	chr1	+	207324558	207324750	207322381	207322567	207325621	207325721	431	170	63	2	1	0	6	79	0	0	98	49	1	1	0.991	0.988	1	1	1	-0.007
SE	6135	CD55	CD55	chr1	+	207331107	207331296	207326751	207326837	207336692	207336818	181	53	39	0	3	0	2	28	0	0	98	49	1	1	1	0.898	1	1	1	-0.034
SE	6136	CD55	CD55	chr1	+	207340390	207340508	207339396	207339417	207359545	207360966	5	0	9	27	5	14	0	0	0	5	98	49	1	1	0.085	0	0.243	NA	0	0.109
SE	6137	CD55	CD55	chr1	+	207347446	207347561	207339396	207339417	207359545	207360966	1	0	0	27	5	14	0	0	0	5	98	49	1	1	0.018	0	0	NA	0	0.006
SE	6139	CD55	CD55	chr1	+	207354006	207354099	207339396	207339417	207359545	207360966	1	0	0	27	5	14	0	0	0	5	98	49	1	1	0.018	0	0	NA	0	0.006
SE	6143	SRSF5	SRSF5	chr14	+	69768797	69768896	69768603	69768674	69769181	69769251	179	77	40	1	0	0	0	20	0	0	98	49	1	1	0.989	1	1	NA	1	-0.004
SE	6146	CNOT1	CNOT1	chr16	-	58547565	58547682	58547185	58547296	58549718	58549898	51	11	0	2	0	1	0	13	0	0	98	49	1	1	0.927	1	0	NA	1	-0.358
SE	6147	CNOT1	CNOT1	chr16	-	58555250	58555537	58553781	58553860	58555783	58555908	35	10	0	1	0	0	0	2	0	0	98	49	1	1	0.946	1	NA	NA	1	-0.027
SE	6149	CNOT1	CNOT1	chr16	-	58575006	58575129	58574608	58574760	58576462	58576582	23	11	7	1	0	0	2	6	0	0	98	49	1	1	0.92	1	1	1	1	-0.027
SE	6150	CNOT1	CNOT1	chr16	-	58583055	58583182	58582792	58582903	58585337	58585506	35	8	1	0	2	0	0	6	0	0	98	49	1	1	1	0.667	1	NA	1	-0.111
SE	6151	CNOT1	CNOT1	chr16	-	58599235	58599511	58588798	58588906	58629727	58629886	12	4	0	1	0	0	1	0	0	0	98	49	1	1	0.857	1	NA	1	NA	-0.072
SE	6157	LIMK2	LIMK2	chr22	+	31265945	31266132	31262594	31262791	31266983	31267070	68	29	16	0	1	0	2	21	0	0	98	49	1	1	1	0.935	1	1	1	-0.022
SE	6158	LIMK2	LIMK2	chr22	+	31266983	31267070	31265945	31266132	31267775	31267907	112	56	23	0	0	0	0	20	0	1	98	49	1	1	1	1	1	NA	0.909	0.091
SE	6159	LIMK2	LIMK2	chr22	+	31268143	31268200	31267775	31267907	31271135	31271201	71	30	30	1	0	0	1	20	0	0	98	49	1	1	0.973	1	1	1	1	-0.009
SE	6160	LIMK2	LIMK2	chr22	+	31273451	31273507	31272529	31272704	31275150	31275308	201	114	21	1	0	0	13	40	0	0	98	49	1	1	0.99	1	1	1	1	-0.003
SE	6165	NBPF14	NBPF14	chr1	-	148593597	148593700	148592551	148592766	148595542	148595717	5	1	3	2	2	0	0	1	0	1	98	49	1	1	0.556	0.2	1	NA	0.333	0.252
SE	6166	TCF25	TCF25	chr16	+	89893727	89893858	89892192	89892275	89895037	89895137	9	8	5	1	0	0	0	6	0	0	98	49	1	1	0.818	1	1	NA	1	-0.061
SE	6174	MAP2K7	MAP2K7	chr19	+	7905807	7905855	7903779	7904068	7909754	7909896	0	3	0	2	3	0	0	0	1	0	97	49	1	1	0	0.336	NA	0	NA	0.168
SE	6175	PTGS2	PTGS2	chr1	-	186677648	186677830	186676832	186676916	186678260	186678404	526	106	24	2	0	0	1	45	0	0	98	49	1	1	0.992	1	1	1	1	-0.003
SE	6185	DDX60	DDX60	chr4	-	168272042	168272138	168268853	168268969	168273278	168273398	5	2	1	0	1	0	1	7	1	0	98	49	1	1	1	0.5	1	0.333	1	0.167
SE	6186	ARHGEF2	ARHGEF2	chr1	-	155954901	155954969	155952627	155952828	155957712	155957882	53	26	10	2	0	0	0	12	0	0	98	49	1	1	0.93	1	1	NA	1	-0.023
SE	6187	ARHGEF2	ARHGEF2	chr1	-	155957712	155957882	155954901	155954969	155958319	155958396	35	29	20	0	2	0	0	10	0	0	98	49	1	1	1	0.879	1	NA	1	-0.04
SE	6188	ARHGEF2	ARHGEF2	chr1	-	155965630	155965760	155965302	155965412	155966415	155966479	37	4	9	6	2	0	0	8	0	0	98	49	1	1	0.755	0.5	1	NA	1	-0.248
SE	6189	ARHGEF2	ARHGEF2	chr1	-	155966819	155966887	155966415	155966479	155969155	155969300	45	23	7	1	1	0	0	4	0	0	98	49	1	1	0.957	0.92	1	NA	1	-0.041
SE	6192	TXNDC17	TXNDC17	chr17	+	6641752	6641834	6640901	6641227	6642248	6642324	1	1	5	0	0	0	0	1	0	2	98	49	1	1	1	1	1	NA	0.2	0.8
SE	6200	CS	CS	chr12	-	56283791	56283857	56282859	56282991	56285915	56286023	9	6	0	1	0	0	0	7	0	0	98	49	1	1	0.818	1	NA	NA	1	-0.091
SE	6208	ADAT1	ADAT1	chr16	-	75618585	75618640	75617141	75617272	75620265	75620334	6	1	3	0	1	0	1	0	0	0	98	49	1	1	1	0.333	1	1	NA	-0.222
SE	6210	TRIM33	TRIM33	chr1	-	114397939	114397990	114392776	114397860	114399456	114399609	17	8	5	6	3	0	0	7	0	2	98	49	1	1	0.586	0.571	1	NA	0.636	0.083
SE	6217	RIF1	RIF1	chr2	+	151462883	151466120	151462411	151462466	151467999	151468146	8	7	4	0	0	3	0	4	0	0	98	49	1	1	1	1	0.4	NA	1	-0.2
SE	6238	EHBP1L1	EHBP1L1	chr11	+	65589935	65589991	65589750	65589820	65590086	65590210	52	21	9	0	1	0	1	10	0	0	98	49	1	1	1	0.913	1	1	1	-0.029
SE	6240	APBB1IP	APBB1IP	chr10	+	26503196	26503274	26500818	26501111	26511746	26511906	165	44	23	3	0	0	0	28	0	0	98	49	1	1	0.965	1	1	NA	1	-0.012
SE	6241	APBB1IP	APBB1IP	chr10	+	26513538	26513660	26511746	26511906	26533438	26533525	80	38	24	1	1	0	0	20	0	0	98	49	1	1	0.976	0.95	1	NA	1	-0.025
SE	6244	PSMA5	PSMA5	chr1	-	109409927	109410014	109399030	109402090	109411010	109411113	11	8	5	1	0	0	0	4	0	0	98	49	1	1	0.846	1	1	NA	1	-0.051
SE	6248	STK24	STK24	chr13	-	98457167	98457304	98450198	98453209	98460371	98460440	30	9	4	0	0	0	0	5	0	1	98	49	1	1	1	1	1	NA	0.714	0.286
SE	6249	STK24	STK24	chr13	-	98482264	98482321	98475249	98475358	98519242	98519473	42	8	6	0	2	0	0	2	0	0	98	49	1	1	1	0.667	1	NA	1	-0.111
SE	6251	SEC24B	SEC24B	chr4	+	109481676	109481781	109473003	109473186	109491326	109491407	4	1	0	5	0	0	0	2	0	0	98	49	1	1	0.286	1	NA	NA	1	-0.357
SE	6252	MAP2K1	MAP2K1	chr15	+	66484989	66485191	66481754	66481879	66487227	66487292	8	0	2	2	0	0	0	5	0	0	98	49	1	1	0.667	NA	1	NA	1	-0.166
SE	6262	CLASP1	CLASP1	chr2	-	121409023	121409047	121407470	121407715	121410865	121410969	2	0	1	1	1	0	0	0	0	2	73	49	1	1	0.573	0	1	NA	0	0.524
SE	6266	CLASP1	CLASP1	chr2	-	121445448	121445496	121430072	121430177	121447336	121447507	5	0	0	6	2	0	0	0	0	1	97	49	1	1	0.296	0	NA	NA	0	0.148
SE	6267	CLASP1	CLASP1	chr2	-	121459979	121460125	121458839	121458975	121461100	121461193	6	4	0	2	0	0	0	2	0	0	98	49	1	1	0.6	1	NA	NA	1	-0.2
SE	6268	CLASP1	CLASP1	chr2	-	121515664	121515762	121503166	121503234	121525844	121525920	20	11	2	0	0	0	0	1	0	2	98	49	1	1	1	1	1	NA	0.2	0.8
SE	6269	CLASP1	CLASP1	chr2	-	121527798	121527890	121525844	121525920	121528676	121528780	41	15	5	1	0	0	0	5	0	0	98	49	1	1	0.953	1	1	NA	1	-0.016
SE	6270	CLASP1	CLASP1	chr2	-	121530246	121530325	121528676	121528780	121605700	121606180	34	9	1	3	0	0	0	2	0	0	98	49	1	1	0.85	1	1	NA	1	-0.05
SE	6271	CANX	CANX	chr5	+	179708977	179709059	179708238	179708380	179709872	179710065	32	5	2	1	0	0	3	6	0	0	98	49	1	1	0.941	1	1	1	1	-0.02
SE	6272	HIAT1	HIAT1	chr1	+	100068472	100068586	100067976	100068195	100069613	100069685	21	2	6	2	0	0	0	2	0	0	98	49	1	1	0.84	1	1	NA	1	-0.053
SE	6287	CBFB	CBFB	chr16	+	67036638	67036755	67029726	67029813	67066681	67066798	12	10	2	1	0	0	0	3	0	0	98	49	1	1	0.857	1	1	NA	1	-0.048
SE	6293	FXR1	FXR1	chr3	+	180970157	180970358	180968050	180968254	180976121	180982751	12	4	1	2	0	0	0	2	0	0	98	49	1	1	0.75	1	1	NA	1	-0.083
SE	6296	TMEM87A	TMEM87A	chr15	-	42272062	42272123	42267946	42268032	42273254	42273584	13	3	3	1	0	0	0	1	0	0	98	49	1	1	0.867	1	1	NA	1	-0.044
SE	6297	SOAT1	SOAT1	chr1	+	179302676	179302802	179293713	179293936	179323436	179323495	9	3	3	1	0	0	0	6	0	0	98	49	1	1	0.818	1	1	NA	1	-0.061
SE	6301	ADCK3	ADCK3	chr1	+	226965670	226965737	226964999	226965410	226977448	226977523	4	6	8	1	0	0	0	3	0	0	98	49	1	1	0.667	1	1	NA	1	-0.111
SE	6318	PTBP2	PTBP2	chr1	+	96813028	96813106	96812711	96812928	96813272	96815049	4	0	0	0	0	0	0	0	0	2	98	49	1	1	1	NA	NA	NA	0	1
SE	6326	SHISA5	SHISA5	chr3	-	48503094	48503193	48501136	48501293	48504018	48504249	0	2	0	9	3	0	0	0	0	1	98	49	1	1	0	0.25	NA	NA	0	0.125
SE	6327	SHISA5	SHISA5	chr3	-	48503094	48503193	48501136	48501293	48504422	48504814	0	0	0	1	5	0	0	2	0	0	98	49	1	1	0	0	NA	NA	1	-1
SE	6335	VWA8	VWA8	chr13	-	41949935	41950013	41912037	41912168	41960852	41961085	2	0	0	0	0	0	0	0	0	1	98	49	1	1	1	NA	NA	NA	0	1
SE	6336	LINC01578	LINC01578	chr15	+	92883584	92883778	92882842	92883186	92885514	92885584	8	4	0	8	3	0	0	2	0	0	98	49	1	1	0.333	0.4	NA	NA	1	-0.633
SE	6339	ZFAND4	ZFAND4	chr10	-	45652983	45653059	45651965	45652033	45663541	45663842	4	0	0	0	0	0	0	0	0	2	98	49	1	1	1	NA	NA	NA	0	1
SE	6346	CDC14A	CDC14A	chr1	+	100455404	100455492	100442933	100442996	100462650	100462881	12	10	2	2	0	0	0	1	0	1	98	49	1	1	0.75	1	1	NA	0.333	0.584
SE	6351	EVI5	EVI5	chr1	-	92607580	92607727	92605306	92605402	92624175	92624334	5	0	0	0	1	0	0	6	0	0	98	49	1	1	1	0	NA	NA	1	-0.5
SE	6353	EVI5	EVI5	chr1	-	92693801	92693899	92677157	92677218	92694298	92694388	18	6	0	0	0	0	0	4	0	1	98	49	1	1	1	1	NA	NA	0.667	0.333
SE	6365	DARS2	DARS2	chr1	+	173828332	173828399	173826686	173826786	173830659	173830761	0	0	0	2	0	0	0	1	0	0	98	49	1	1	0	NA	NA	NA	1	-1
SE	6368	ALDOA	ALDOA	chr16	+	30066884	30067038	30065672	30065927	30067233	30067366	0	0	0	44	14	9	0	1	0	7	98	49	1	1	0	0	0	NA	0.067	-0.067
SE	6369	ALDOA	ALDOA	chr16	+	30068645	30068700	30067449	30067661	30068817	30068978	149	91	77	0	0	0	3	41	0	1	98	49	1	1	1	1	1	1	0.953	0.024
SE	6384	KMT2D	KMT2D	chr12	-	49028827	49028958	49028008	49028141	49029060	49029236	12	7	0	2	0	0	4	7	3	0	98	49	1	1	0.75	1	NA	0.4	1	0.175
SE	6385	KMT2D	KMT2D	chr12	-	49030892	49031033	49030600	49030768	49031174	49033964	21	19	1	1	0	0	1	3	0	0	98	49	1	1	0.913	1	1	1	1	-0.029
SE	6386	KMT2D	KMT2D	chr12	-	49054899	49055026	49053977	49054140	49055275	49055324	11	21	0	0	1	0	0	3	0	0	98	49	1	1	1	0.913	NA	NA	1	-0.043
SE	6387	KMT2D	KMT2D	chr12	-	49054899	49055026	49054527	49054751	49055275	49055324	13	23	4	0	3	0	0	3	0	0	98	49	1	1	1	0.793	1	NA	1	-0.069
SE	6400	TGFBR3	TGFBR3	chr1	-	91800958	91801055	91797286	91797471	91861470	91861644	0	4	0	1	0	0	0	0	0	3	98	49	1	1	0	1	NA	NA	0	0.5
SE	6405	ARID1A	ARID1A	chr1	+	26729650	26729863	26696030	26697540	26731151	26731604	12	5	0	1	0	0	0	3	0	0	98	49	1	1	0.857	1	NA	NA	1	-0.072
SE	6408	POU2F1	POU2F1	chr1	+	167396285	167396427	167389587	167389761	167397993	167398133	2	2	4	0	0	0	0	3	0	1	98	49	1	1	1	1	1	NA	0.6	0.4
SE	6412	DGKA	DGKA	chr12	+	55941509	55941584	55941251	55941325	55941997	55942083	1	0	0	1	0	0	0	1	0	0	98	49	1	1	0.333	NA	NA	NA	1	-0.667
SE	6416	ECD	ECD	chr10	-	73154255	73154448	73152292	73152421	73156274	73156453	3	0	1	0	0	0	0	2	0	1	98	49	1	1	1	NA	1	NA	0.5	0.5
SE	6417	ECD	ECD	chr10	-	73163732	73163950	73160433	73160551	73167865	73168095	9	3	4	1	0	0	0	1	0	0	98	49	1	1	0.818	1	1	NA	1	-0.061
SE	6418	ZRANB2	ZRANB2	chr1	-	71065677	71065752	71063290	71065137	71066775	71066934	6	2	0	12	0	0	0	1	0	0	98	49	1	1	0.2	1	NA	NA	1	-0.4
SE	6420	RALGAPA2	RALGAPA2	chr20	-	20396694	20396734	20389627	20393253	20412026	20412148	11	2	6	0	0	3	0	3	0	0	89	49	1	1	1	1	0.524	NA	1	-0.159
SE	6421	RALGAPA2	RALGAPA2	chr20	-	20524405	20524543	20520916	20521100	20524829	20524898	12	9	2	0	3	0	0	2	0	1	98	49	1	1	1	0.6	1	NA	0.5	0.367
SE	6422	RALGAPA2	RALGAPA2	chr20	-	20611314	20611426	20605174	20605412	20616042	20616191	33	4	0	0	0	0	0	6	0	1	98	49	1	1	1	1	NA	NA	0.75	0.25
SE	6423	RALGAPA2	RALGAPA2	chr20	-	20629362	20629590	20620462	20620630	20635417	20635617	22	21	4	1	0	0	0	6	0	0	98	49	1	1	0.917	1	1	NA	1	-0.028
SE	6424	RALGAPA2	RALGAPA2	chr20	-	20637362	20637501	20635417	20635617	20639784	20639900	37	17	18	1	4	0	0	13	0	2	98	49	1	1	0.949	0.68	1	NA	0.765	0.111
SE	6425	RALGAPA2	RALGAPA2	chr20	-	20676235	20676288	20653529	20653587	20680690	20680801	45	24	6	0	1	0	0	13	0	2	98	49	1	1	1	0.923	1	NA	0.765	0.209
SE	6427	PRRG4	PRRG4	chr11	+	32840106	32840239	32838881	32838930	32853295	32858123	14	2	0	1	2	1	0	1	0	0	98	49	1	1	0.875	0.333	0	NA	1	-0.597
SE	6428	NPTN	NPTN	chr15	-	73597021	73597369	73591965	73592137	73633124	73633412	1	0	0	10	6	1	0	0	0	1	98	49	1	1	0.048	0	0	NA	0	0.016
SE	6429	LMBR1L	LMBR1L	chr12	-	49102886	49102951	49102467	49102540	49103090	49103159	7	3	1	0	1	0	1	4	0	0	98	49	1	1	1	0.6	1	1	1	-0.133
SE	6434	SLK	SLK	chr10	+	104010815	104010908	104008176	104008356	104018159	104018289	0	1	0	7	14	4	0	0	0	4	98	49	1	1	0	0.034	0	NA	0	0.011
SE	6441	NDUFB6	NDUFB6	chr9	-	32558909	32558954	32553525	32553944	32572880	32573184	6	15	12	0	0	0	0	5	0	3	94	49	1	1	1	1	1	NA	0.465	0.535
SE	6442	NDUFB6	NDUFB6	chr9	-	32570959	32571052	32553525	32553944	32572880	32573184	2	2	2	0	0	0	0	0	0	3	98	49	1	1	1	1	1	NA	0	1
SE	6443	NDUFB6	NDUFB6	chr9	-	32570959	32571052	32558909	32558954	32572880	32573184	7	11	4	0	4	2	1	0	0	0	98	49	1	1	1	0.579	0.5	1	NA	-0.307
SE	6444	IRAK3	IRAK3	chr12	+	66203710	66203893	66189197	66189432	66209455	66209520	24	8	5	1	1	0	0	13	0	0	98	49	1	1	0.923	0.8	1	NA	1	-0.092
SE	6449	ILF3	ILF3	chr19	+	10680345	10680443	10679815	10679927	10681018	10681130	5	1	1	0	0	2	0	7	0	0	98	49	1	1	1	1	0.2	NA	1	-0.267
SE	6451	ZMIZ1	ZMIZ1	chr10	+	79289774	79289889	79277180	79277325	79290958	79291176	10	3	1	1	0	0	0	5	0	0	98	49	1	1	0.833	1	1	NA	1	-0.056
SE	6455	NCF1	NCF1	chr7	+	74779081	74779157	74777266	74777347	74779256	74779422	17	13	5	2	1	0	0	4	0	0	98	49	1	1	0.81	0.867	1	NA	1	-0.108
SE	6462	DPH3	DPH3	chr3	-	16264154	16264229	16257060	16260829	16264768	16264989	8	2	1	3	1	7	0	1	0	0	98	49	1	1	0.571	0.5	0.067	NA	1	-0.621
SE	6467	C1orf43	C1orf43	chr1	-	154213892	154213946	154212457	154212624	154214456	154214574	12	6	10	2	1	0	0	3	0	1	98	49	1	1	0.75	0.75	1	NA	0.6	0.233
SE	6468	C1orf43	C1orf43	chr1	-	154214456	154214574	154213892	154213946	154220341	154220797	8	1	3	1	0	0	1	3	0	0	98	49	1	1	0.8	1	1	1	1	-0.067
SE	6474	GBP5	GBP5	chr1	-	89262219	89262401	89258950	89260817	89263735	89263948	65	9	9	0	2	0	0	10	0	0	98	49	1	1	1	0.692	1	NA	1	-0.103
SE	6475	GBP5	GBP5	chr1	-	89262682	89262785	89262219	89262401	89263735	89263948	85	28	13	2	0	0	3	3	0	0	98	49	1	1	0.955	1	1	1	1	-0.015
SE	6476	GBP5	GBP5	chr1	-	89266956	89267153	89266345	89266588	89268728	89268856	62	19	18	1	0	0	0	7	0	0	98	49	1	1	0.969	1	1	NA	1	-0.01
SE	6477	GBP5	GBP5	chr1	-	89267416	89267526	89266956	89267153	89268728	89268856	136	36	23	1	0	0	2	14	0	0	98	49	1	1	0.986	1	1	1	1	-0.005
SE	6478	GBP5	GBP5	chr1	-	89268728	89268856	89267416	89267526	89269365	89269574	72	16	9	1	1	0	1	7	0	0	98	49	1	1	0.973	0.889	1	1	1	-0.046
SE	6479	GBP5	GBP5	chr1	-	89268728	89268856	89267416	89267526	89272451	89272861	48	11	5	1	1	0	1	3	0	0	98	49	1	1	0.96	0.846	1	1	1	-0.065
SE	6481	GBP5	GBP5	chr1	-	89269365	89269574	89268728	89268856	89272451	89272861	30	9	5	2	1	0	0	4	0	0	98	49	1	1	0.882	0.818	1	NA	1	-0.1
SE	6482	GBP5	GBP5	chr1	-	89270754	89270862	89269365	89269574	89272451	89272861	52	16	3	4	3	1	0	7	0	0	98	49	1	1	0.867	0.727	0.6	NA	1	-0.269
SE	6501	ARHGEF11	ARHGEF11	chr1	-	156947768	156947956	156947303	156947450	156948180	156948228	18	10	9	0	0	0	0	13	0	1	98	49	1	1	1	1	1	NA	0.867	0.133
SE	6508	ADAM15	ADAM15	chr1	+	155061414	155061489	155060762	155060832	155062244	155062369	0	0	0	2	0	0	0	1	0	0	98	49	1	1	0	NA	NA	NA	1	-1
SE	6517	ACAP2	ACAP2	chr3	-	195289120	195289231	195285795	195285857	195291705	195291815	60	38	19	2	0	0	0	11	0	0	98	49	1	1	0.937	1	1	NA	1	-0.021
SE	6518	ACAP2	ACAP2	chr3	-	195291705	195291815	195289120	195289231	195292264	195292452	48	38	13	1	0	1	0	12	0	2	98	49	1	1	0.96	1	0.867	NA	0.75	0.192
SE	6520	KLHL12	KLHL12	chr1	-	202893238	202893425	202891095	202892659	202894183	202894282	4	0	3	0	0	0	0	2	0	1	98	49	1	1	1	NA	1	NA	0.5	0.5

SE	6523	LYST	LYST	chr1	-	235677479	235677619	235677090	235677188	235686948	235687047	90	32	16	1	0	0	0	11	0	0	98	49	1	1	0.978	1	1	NA			1	-0.007
SE	6525	LYST	LYST	chr1	-	235733502	235733691	235731031	235731177	235733829	235733906	121	59	17	0	3	0	0	3	0	0	98	49	1	1	1	0.908	1	NA			1	-0.031
SE	6526	LYST	LYST	chr1	-	235757280	235757458	235755477	235755647	235758971	235759599	59	61	8	4	0	0	0	13	0	0	98	49	1	1	0.881	1	1	NA			1	-0.04
SE	6527	LYST	LYST	chr1	-	235758971	235759599	235755477	235755647	235762719	235762851	61	30	8	0	1	0	0	6	0	0	98	49	1	1	1	0.937	1	NA			1	-0.021
SE	6528	LYST	LYST	chr1	-	235777062	235777308	235774912	235775086	235780864	235781055	124	53	18	1	0	0	2	15	0	0	98	49	1	1	0.984	1	1		1		1	-0.005
SE	6531	LYST	LYST	chr1	-	235812970	235813061	235808454	235810534	235830225	235830424	181	54	20	1	0	0	3	35	0	0	98	49	1	1	0.989	1	1		1		1	-0.004
SE	6537	RBM39	RBM39	chr20	-	35740823	35740887	35738967	35739017	35741940	35742336	73	28	8	1	0	0	2	10	0	0	98	49	1	1	0.973	1	1		1		1	-0.009
SE	6538	UBR4	UBR4	chr1	-	19077975	19078066	19076739	19076902	19081348	19081573	13	1	0	1	0	0	1	2	0	0	98	49	1	1	0.867	1	NA		1		1	-0.067
SE	6539	UBR4	UBR4	chr1	-	19092818	19092918	19088758	19088977	19093312	19093486	24	3	0	1	0	0	0	1	0	0	98	49	1	1	0.923	1	NA	NA			1	-0.038
SE	6540	UBR4	UBR4	chr1	-	19094905	19095025	19093948	19094139	19095544	19095652	4	1	1	0	0	0	0	3	0	1	98	49	1	1	1	1	1	NA			0.6	0.4
SE	6541	UBR4	UBR4	chr1	-	19097192	19097280	19096522	19096650	19099596	19099677	8	3	0	0	1	0	0	2	0	0	98	49	1	1	1	0.6	NA	NA			1	-0.2
SE	6542	UBR4	UBR4	chr1	-	19099596	19099677	19096522	19096650	19100375	19100573	8	3	1	1	0	0	0	1	0	0	98	49	1	1	0.8	1	1	NA			1	-0.067
SE	6543	UBR4	UBR4	chr1	-	19148562	19148626	19147992	19148127	19150576	19150793	10	6	3	0	1	0	0	2	0	1	98	49	1	1	1	0.75	1	NA			0.5	0.417
SE	6544	UBR4	UBR4	chr1	-	19150576	19150793	19148562	19148626	19151642	19151859	10	9	0	3	0	0	0	3	0	0	98	49	1	1	0.625	1	NA	NA			1	-0.188
SE	6545	UBR4	UBR4	chr1	-	19153767	19153939	19153300	19153502	19154917	19155075	20	0	0	0	0	0	0	1	0	1	98	49	1	1	1	NA	NA	NA			0.333	0.667
SE	6547	UBR4	UBR4	chr1	-	19165655	19165757	19165248	19165349	19167021	19167231	20	9	0	1	0	0	0	4	0	1	98	49	1	1	0.909	1	NA	NA			0.667	0.287
SE	6548	UBR4	UBR4	chr1	-	19168026	19168184	19167021	19167231	19169434	19169532	47	6	6	0	1	0	0	11	0	0	98	49	1	1	1	0.75	1	NA			1	-0.083
SE	6549	UBR4	UBR4	chr1	-	19174318	19174447	19173438	19173621	19174953	19175033	17	3	5	1	0	0	0	3	0	0	98	49	1	1	0.895	1	1	NA			1	-0.035
SE	6550	UBR4	UBR4	chr1	-	19185098	19185286	19184015	19184175	19186539	19186657	8	9	0	1	0	0	0	2	0	0	98	49	1	1	0.8	1	NA	NA			1	-0.1
SE	6553	APAF1	APAF1	chr12	+	98665552	98665791	98662674	98662806	98666189	98666357	17	0	10	7	0	0	0	2	0	1	98	49	1	1	0.548	NA	1	NA			0.5	0.274
SE	6554	APAF1	APAF1	chr12	+	98677424	98677551	98671534	98671719	98680276	98680402	69	15	8	4	0	0	0	7	0	1	98	49	1	1	0.896	1	1	NA			0.778	0.187
SE	6555	APAF1	APAF1	chr12	+	98703370	98703499	98699407	98699569	98706484	98706610	18	12	11	21	0	2	0	5	0	7	98	49	1	1	0.3	1	0.733	NA			0.263	0.415
SE	6556	APAF1	APAF1	chr12	+	98706484	98706610	98699407	98699569	98708584	98708704	52	13	6	0	0	0	0	7	0	1	98	49	1	1	1	1	1	NA			0.778	0.222
SE	6557	APAF1	APAF1	chr12	+	98725414	98725540	98723638	98723764	98727172	98727316	37	11	9	1	0	0	0	5	0	0	98	49	1	1	0.949	1	1	NA			1	-0.017
SE	6558	APAF1	APAF1	chr12	+	98727172	98727316	98725414	98725540	98732419	98735433	53	18	8	0	1	0	0	7	0	0	98	49	1	1	1	0.9	1	NA			1	-0.033
SE	6561	TRERF1	TRERF1	chr6	-	42263319	42263568	42259338	42259723	42264703	42264854	0	0	0	10	4	0	0	1	1	0	98	49	1	1	0	0	NA		0		1	-0.5
SE	6570	PSRC1	PSRC1	chr1	-	109280395	109280489	109279553	109280156	109280776	109280916	3	0	4	0	0	0	0	1	3	0	98	49	1	1	1	NA	1		0		1	0.5
SE	6577	NOD2	NOD2	chr16	+	50719924	50720008	50716890	50716974	50722621	50722705	5	1	2	0	0	0	0	3	0	1	98	49	1	1	1	1	1	NA			0.6	0.4
SE	6586	RGL4	RGL4	chr22	+	23697170	23697245	23696613	23696688	23697837	23697861	24	12	0	1	0	0	1	6	0	1	98	49	1	1	0.923	1	NA		1		1	-0.038
SE	6587	RGL4	RGL4	chr22	+	23697837	23697861	23696613	23696688	23698211	23698333	4	0	0	0	0	0	2	0	0	1	73	49	1	1	1	NA	NA		1		0	0.5
SE	6588	SETD2	SETD2	chr3	-	47083719	47084382	47067069	47067118	47086194	47086314	15	5	0	0	1	0	1	1	0	0	98	49	1	1	1	0.714	NA		1		1	-0.143
SE	6590	PRKAA1	PRKAA1	chr5	-	40774927	40774972	40771718	40771863	40775409	40775503	4	3	0	6	0	2	0	0	0	1	94	49	1	1	0.258	1	0	NA			0	0.419
SE	6591	CHPF2	CHPF2	chr7	+	151235047	151235612	151232488	151234274	151237373	151238827	4	3	4	0	1	0	0	2	0	0	98	49	1	1	1	0.6	1	NA			1	-0.133
SE	6592	CHPF2	CHPF2	chr7	+	151236407	151236590	151235047	151235612	151237373	151238827	10	2	3	2	1	0	0	4	0	0	98	49	1	1	0.714	0.5	1	NA			1	-0.262
SE	6593	KANSL2	KANSL2	chr12	-	48679035	48679150	48671798	48671962	48679654	48679833	5	1	0	3	3	1	0	6	0	2	98	49	1	1	0.455	0.143	0	NA			0.6	-0.401
SE	6606	NDUFB5	NDUFB5	chr3	+	179616982	179617044	179615982	179616049	179618414	179618521	11	2	3	0	0	0	0	7	0	1	98	49	1	1	1	1	1	NA			0.778	0.222
SE	6611	AKAP13	AKAP13	chr15	+	85662387	85662453	85655416	85655787	85664562	85664755	3	1	0	3	5	0	0	0	0	1	98	49	1	1	0.333	0.091	NA	NA			0	0.212
SE	6613	AKAP13	AKAP13	chr15	+	85715787	85715923	85710578	85710645	85717289	85717402	43	10	2	1	0	0	0	8	0	0	98	49	1	1	0.956	1	1	NA			1	-0.015
SE	6614	PPP1R10	PPP1R10	chr6	-	30604993	30605094	30604587	30604735	30605922	30606035	14	3	1	5	0	0	0	1	0	0	98	49	1	1	0.583	1	1	NA			1	-0.139
SE	6619	ILK	ILK	chr11	+	6608045	6608211	6603809	6604360	6608393	6608489	9	6	7	2	0	0	1	3	0	0	98	49	1	1	0.692	1	1		1		1	-0.103
SE	6631	PRUNE	PRUNE	chr1	+	151018466	151018669	151017811	151017904	151024610	151024795	2	0	6	4	0	0	0	1	0	0	98	49	1	1	0.2	NA	1	NA			1	-0.4
SE	6637	CSF3R	CSF3R	chr1	-	36469651	36469840	36469155	36469257	36471432	36471646	963	336	202	1	0	0	37	229	0	0	98	49	1	1	0.998	1	1		1		1	-0.001
SE	6638	CSF3R	CSF3R	chr1	-	36472065	36472139	36471432	36471646	36472237	36472391	930	461	214	3	0	0	94	256	0	1	98	49	1	1	0.994	1	1		1		0.992	0.002
SE	6640	CSF3R	CSF3R	chr1	-	36473763	36473887	36472516	36472686	36479432	36479516	33	13	3	1	0	0	1	3	0	0	98	49	1	1	0.943	1	1		1		1	-0.019
SE	6641	CSF3R	CSF3R	chr1	-	36475376	36475673	36473763	36473887	36479432	36479516	826	284	151	2	0	0	19	114	0	0	98	49	1	1	0.995	1	1		1		1	-0.002
SE	6642	RPE	RPE	chr2	+	210003437	210003468	210002564	210002783	210009656	210009736	1	0	0	0	1	0	0	0	0	1	80	49	1	1	0.38	NA	NA	NA			0	0.38
SE	6664	ERP29	ERP29	chr12	+	112019755	112019894	112013347	112013609	112022149	112023220	5	0	2	1	0	0	0	0	0	1	98	49	1	1	0.714	NA	1	NA			0	0.857
SE	6672	ARHGAP4	ARHGAP4	chrX	-	153910921	153910999	153910699	153910834	153911128	153911189	56	22	15	1	1	0	4	9	0	0	98	49	1	1	0.966	0.917	1		1		1	-0.039
SE	6673	ARHGAP4	ARHGAP4	chrX	-	153913217	153913302	153913023	153913051	153913408	153913600	39	25	7	1	0	0	0	4	0	0	98	49	1	1	0.951	1	1	NA			1	-0.016
SE	6676	ARHGAP4	ARHGAP4	chrX	-	153921096	153921159	153920625	153920808	153921364	153921527	34	22	6	2	0	0	3	6	0	0	98	49	1	1	0.895	1	1		1		1	-0.035
SE	6679	PKP4	PKP4	chr2	+	158676738	158676867	158673882	158674000	158678580	158678654	2	0	0	1	1	0	0	0	0	2	98	49	1	1	0.5	0	NA	NA			0	0.25
SE	6680	NUDT22	NUDT22	chr11	+	64227567	64227666	64226634	64227132	64229246	64229344	3	1	0	0	0	2	0	2	0	1	98	49	1	1	1	1	0	NA			1	-0.333
SE	6698	GATAD2B	GATAD2B	chr1	-	153818039	153818171	153817371	153817542	153818790	153818922	16	7	3	0	0	0	0	2	0	1	98	49	1	1	1	1	1	NA			0.5	0.5
SE	6701	LAPTM5	LAPTM5	chr1	-	30741639	30741716	30739808	30739937	30742455	30742549	209	62	86	0	0	0	4	37	0	1	98	49	1	1	1	1	1		1		0.949	0.026
SE	6702	LAPTM5	LAPTM5	chr1	-	30741639	30741716	30739808	30739937	30757658	30757836	131	34	43	1	0	0	1	20	0	0	98	49	1	1	0.985	1	1		1		1	-0.005
SE	6703	LAPTM5	LAPTM5	chr1	-	30742455	30742549	30739808	30739937	30757658	30757836	11	7	3	1	0	0	0	4	0	0	98	49	1	1	0.846	1	1	NA			1	-0.051
SE	6705	STX12	STX12	chr1	+	27817850	27817923	27812162	27812268	27819649	27819732	5	6	1	0	0	0	0	5	0	1	98	49	1	1	1	1	1	NA			0.714	0.286
SE	6707	MTMR3	MTMR3	chr22	+	29970975	29971062	29957035	29957088	29978441	29978531	31	37	4	3	3	0	0	9	0	1	98	49	1	1	0.838	0.86	1	NA			0.818	0.081
SE	6708	MTMR3	MTMR3	chr22	+	30007113	30007319	30002879	30002993	30007900	30008032	48	15	5	0	1	0	0	11	0	0	98	49	1	1	1	0.882	1	NA			1	-0.039
SE	6709	MTMR3	MTMR3	chr22	+	30007900	30008032	30002879	30002993	30009017	30009129	33	31	13	1	1	0	1	14	0	0	98	49	1	1	0.943	0.939	1		1		1	-0.039
SE	6710	MTMR3	MTMR3	chr22	+	30012367	30012563	30009017	30009129	30013355	30013541	32	20	11	0	0	0	0	2	0	4	98	49	1	1	1	0.909	1	NA			0.2	0.77
SE	6711	MTMR3	MTMR3	chr22	+	30013355	30013541	30009017	30009129	30016527	30016698	25	20	5	0	1	0	1	8	0	0	98	49	1	1	1	0.909	1		1		1	-0.03
SE	6712	MTMR3	MTMR3	chr22	+	30022028	30022139	30019479	30020884	30022608	30022697	31	7	12	3	5	0	0	10	0	0	98	49	1	1	0.838	0.412	1	NA			1	-0.25
SE	6713	MTMR3	MTMR3	chr22	+	30023456	30023483	30022608	30022697	30025629	30030868	10	6	3	12	4	4	0	5	0	2	76	49	1	1	0.35	0.492	0.326	NA			0.617	-0.228
SE	6714	TK2	TK2	chr16	-	66536963	66537017	66531379	66531469	66541878	66541953	30	8	0	1	0	0	0	3	0	0	98	49	1	1	0.937	1	NA	NA			1	-0.031
SE	6716	TK2	TK2	chr16	-	66547970	66548065	66541878	66541953	66548977	66549009	2	0	0	4	0	0	0	0	0	2	98	49	1	1	0.2	NA	NA	NA			0	0.2
SE	6719	TRIM5	TRIM5	chr11	-	5665980	5666081	5663194	5665682	5667688	5667711	5	2	3	1	0	0	0	1	0	1	98	49	1	1	0.714	1	1	NA			0.333	0.572
SE	6720	TRIM5	TRIM5	chr11	-	5679073	5679169	5678203	5678434	5679760	5680238	10	7	2	1	0	0	2	4	0	0	98	49	1	1	0.833	1	1		1		1	-0.056
SE	6721	C20orf24	C20orf24	chr20	+	36609600	36609730	36607714	36607818	36612009	36612557	29	41	54	1	0	0	0	8	0	0	98	49	1	1	0.935	1	0.964	NA			1	-0.034
SE	6723	TNFRSF1B	TNFRSF1B	chr1	+	12193954	12194032	12192862	12193098	12194583	12194618	112	87	55	1	0	0	8	48	0	0	98	49	1	1	0.982	1	1		1		1	-0.006
SE	6724	TNFRSF1B	TNFRSF1B	chr1	+	12201966	12202171	12194583	12194618	12206739	12209220	77	78	38	1	0	0	5	27	0	0	98	49	1	1	0.975	1	1		1		1	-0.008
SE	6726	DSCR3	DSCR3	chr21	-	37233361	37233442	37232376	37232451	37238459	37238609	7	12	2	1	0	0	0	3	0	0	98	49	1	1	0.778	1	1	NA			1	-0.074
SE	6748	DHRS12	DHRS12	chr13	-	51777059	51777121	51773929	51774034	51790010	51790092	3	7	3	1	0	7	0	1	0	0	98	49	1	1	0.6	1	0.176	NA			1	-0.408
SE	6749	DHRS12	DHRS12	chr13	-	51797825	51797908	51791164	51791257	51799533	51799667	1	0	0	6	1	0	0	2	0	6	98	49	1	1	0.077	0	NA	NA			0.143	-0.104
SE	6754	KMT2E	KMT2E	chr7	+	105038125	105038199	105014189	105014535	105040838	105041023	25	23	8	9	4	3	0	7	0	2	98	49	1	1	0.581	0.742	0.571	NA			0.636	-0.005
SE	6755	KMT2E	KMT2E	chr7	+	105062163	105062278	105040838	105041023	105063350	105063580	58	34	8	1	0	0	1	9	0	0	98	49	1	1	0.967	1	1		1		1	-0.011
SE	6757	SCCPDH	SCCPDH	chr1	+	246735974	246736055	246726891	246727004	246740171	246740301	0	3	2	1	0	0	0	5	0	0	98	49	1	1	0	1	1	NA			1	-0.333
SE	6763	OPA1	OPA1	chr3	+	193617177	193617285	193615673	193615770	193618868	193618936	11	0	1	4	0	0	0	2	0	0	98	49	1	1	0.579	NA	1	NA			1	-0.211
SE	6765	OPA1	OPA1	chr3	+	193617783	193617837	193617177	193617285	193618868	193618936	4	0	0	7	0	1	0	1	0	0	98	49	1	1	0.222	NA	0	NA			1	-0.889
SE	6766	OPA1	OPA1	chr3	+	193626091	193626202	193618868	193618936	193631611	193631665	2	0	0	3	0	0	0	1	0	0	98	49	1	1	0.25	NA	NA	NA			1	-0.75
SE	6771	PRPF19	PRPF19	chr11	-	60898861	60898931	60898540	60898626	60899148	60899304	1	3	0	2	0	0	0	1	0	0	98	49	1	1	0.2	1	NA	NA			1	-0.4
SE	6775	ZNF384	ZNF384	chr12	-	6672349	6672532	6670759	6670838	6673215	6673440	2	0	5	6	1	3	0	0	0	1	98	49	1	1	0.143	0	0.455	NA			0	0.199
SE	6776	ZNF384	ZNF384	chr12	-	6673215	6673440	6670759	6670838	6678126	6678343	7	3	3	2	0	0	0	1	0	1	98	49	1	1	0.636	1	1	NA			0.333	0.546
SE	6777	CSF1	CSF1	chr1	+	109914258	109914381	109910610	109911062	109915633	109915696	0	3	0	1	0	0	1	1	3	0	98	49	1	1	0	1	NA		0.143		1	-0.072
SE	6778	CSF1	CSF1	chr1	+	109915633	109915696	109910610	109911062	109917292	109917463	3	4	0	0	1	0	3	0	0	0	98	49	1	1	1	0.667	NA		1	NA	1	-0.166
SE	6783	JMJD1C	JMJD1C	chr10	-	63189167	63189446	63186214	63186383	63190893	63191108	189	51	23	0	1	0	3	24	0	0	98	49	1	1	1	0.962	1		1		1	-0.013
SE	6784	JMJD1C	JMJD1C	chr10	-	63198512	63198727	63197410	63197563	63206594	63208801	79	32	3	0	2	0	0	10	0	0	98	49	1	1	1	0.889	1	NA			1	-0.037
SE	6785	JMJD1C	JMJD1C	chr10	-	63200475	63200677	63198512	63198727	63206594	63208801	40	16	20	5	5	0	0	13	0	1	98	49	1	1	0.8	0.615	1	NA			0.867	-0.062
SE	6786	JMJD1C	JMJD1C	chr10	-	63215552	63215696	63215262	63215455	63217206	63217331	74	29	14	5	1	0	0	21	0	1	98	49	1	1	0.881	0.935	1	NA			0.913	0.026
SE	6787	JMJD1C	JMJD1C	chr10	-	63217206	63217331	63215262	63215455	63264650	63264764	25	7	4	1	0	0	0	3	0	0	98	49	1	1	0.926	1	1	NA			1	-0.025
SE	6788	JMJD1C	JMJD1C	chr10	-	63219877	63219983	63217206	63217331	63264650	63264764	74	21	8	20	6	4	0	14	0	2	98	49	1	1	0.649	0.636	0.5	NA			0.778	-0.183
SE	6789	JMJD1C	JMJD1C	chr10	-	63219877	63219983	63217206	63217331	63268662	63269223	28	10	1	6	1	0	0	2	0	0	98	49	1	1	0.7	0.833	1	NA			1	-0.156
SE	6791	JMJD1C	JMJD1C	chr10	-	63264650	63264764	63219877	63219983	63268662	63269223	179	39	25	1	0	0	1	25	0	0	98	49	1	1	0.989	1	1		1		1	-0.004
SE	6792	JMJD1C	JMJD1C	chr10	-	63264650	63264764	63219877	63219983	63380317	63380482	48	11	8	2	0	0	1	12	0	0	98	49	1	1	0.923	1	1		1		1	-0.026
SE	6793	ZNF592	ZNF592	chr15	+	84778182	84778312	84764706	84764815	84782656	84784895	11	2	3	1	0	0	0	2	0	2	98	49	1	1	0.846	1	1	NA			0.333	0.616
SE	6794	FAM96A	FAM96A	chr15	-	64081101	64081151	64075491	64075537	64088686	64088851	3	1	5	1	0	0	0	5	0	0	98	49	1	1	0.6	1	1	NA			1	-0.133
SE	6796	RRAGD	RRAGD	chr6	-	89380167	89380367	89379223	89379338	89387294	89387590	10	6	10	0	0	0	0	4	0	1	98	49	1	1	1	1	1	NA			0.667	0.333
SE	6799	UBE2G1	UBE2G1	chr17	-	4282797	4282921	4269216	4272516	4289229	4289408	35	32	9	0	2	0	0	11	0	0	98	49	1	1	1	0.889	1	NA			1	-0.037
SE	6800	GAS5	GAS5	chr1	-	173864483	173864506	173864256	173864304	173864674	173864704	20	1	6	1	0	0	0	3	0	0	72	49	1	1	0.932	1	1	NA			1	-0.023
SE	6807	TMEM63A	TMEM63A	chr1	-	225860859	225860997	225859195	225859349	225862217	225862351	11	0	2	0	1	0	0	1	0	0	98	49	1	1	1	0	1	NA			1	-0.333
SE	6809	DMXL2	DMXL2	chr15	-	51451644	51451697	51450128	51450346	51453549	51453641	40	1	10	4	0	0	0	8	0	0	98	49	1	1	0.833	1	1	NA			1	-0.056
SE	6810	DMXL2	DMXL2	chr15	-	51457327	51457466	51456308	51456369	51458505	51458627	62	8	4	1	0	0	1	12	0	0	98	49	1	1	0.969	1	1		1		1	-0.01
SE	6811	DMXL2	DMXL2	chr15	-	51464674	51464876	51463378	51463496	51465565	51465651	46	7	3	4	3	0	0	15	0	1	98	49	1	1	0.852	0.538	1	NA			0.882	-0.085
SE	6812	DMXL2	DMXL2	chr15	-	51465565	51465651	51464674	51464876	51466183	51466311	52	3	3	2	0	0	0	15	0	0	98	49	1	1	0.929	1	1	NA			1	-0.024
SE	6813	DMXL2	DMXL2	chr15	-	51495023	51495134	51491577	51491747	51498551	51500231	43	7	9	1	0	0	1	12	0	0	98	49	1	1	0.956	1	1		1		1	-0.015
SE	6814	DMXL2	DMXL2	chr15	-	51565087	51565166	51564124	51564260	51568486	51568558	34	10	3	1	0	0	0	6	0	0	98	49	1	1	0.944	1	1	NA			1	-0.019
SE	6819	PRMT3	PRMT3	chr11	+	20397576	20397721	20395802	20395962	20402918	20402984	8	1	0	0	0	0	3	0	0	1	98	49	1	1	1	1	NA		1		0	0.5
SE	6820	UBE3A	UBE3A	chr15	-	25354527	25354683	25354352	25354426	25355891	25356056	7	1	0	1	0	0	0	1	0	0	98	49	1	1	0.778	1	NA	NA			1	-0.111
SE	6821	UBE3A	UBE3A	chr15	-	25356690	25356896	25355891	25356056	25360382	25360527	3	0	0	1	0	0	0	1	0	0	98	49	1	1	0.6	NA	NA	NA			1	-0.4
SE	6826	GRIPAP1	GRIPAP1	chrX	-	48990925	48991110	48990684	48990732	48993427	48993578	15	7	0	0	2	0	0	6	0	0	98	49	1	1	1	0.636	NA	NA			1	-0.182
SE	6827	LOC102724552	LOC102724552	chr1	+	8428481	8428588	8424644	8424701	8429826	8429972	1	0	0	0	0	0	0	0	0	1	98	49	1	1	1	NA	NA	NA			0	1
SE	6837	GAK	GAK	chr4	-	913606	913668	904636	904779	932042	932386	9	2	0	1	1	0	3	2	0	0	98	49	1	1	0.818	0.5	NA		1		1	-0.341
SE	6839	BBIP1	BBIP1	chr10	-	110907679	110907836	110901537	110901612	110918120	110918213	1	0	0	6	2	0	0	1	0	0	98	49	1	1	0.077	0	NA	NA			1	-0.962
SE	6843	NAA16	NAA16	chr13	+	41331273	41331369	41328723	41328843	41355143	41355216	2	0	0	0	1	0	0	1	0	0	98	49	1	1	1	0	NA	NA			1	-0.5
SE	6845	TMCO3	TMCO3	chr13	+	113503495	113503588	113501751	113501880	113510237	113510424	11	1	4	0	0	0	0	1	0	2	98	49	1	1	1	1	1	NA			0.2	0.8
SE	6846	TMCO3	TMCO3	chr13	+	113510237	113510424	113503495	113503588	113520615	113520733	7	2	5	0	2	0	0	1	0	0	98	49	1	1	1	0.333	1	NA			1	-0.222
SE	6847	TMCO3	TMCO3	chr13	+	113534044	113534240	113520615	113520733	113539356	113539507	2	2	1	1	0	0	0	2	0	0	98	49	1	1	0.5	1	1	NA			1	-0.167
SE	6849	AMPD2	AMPD2	chr1	+	109620913	109621266	109619812	109620278	109625661	109625792	4	3	0	11	6	2	0	0	0	3	98	49	1	1	0.154	0.2	0	NA			0	0.118
SE	6850	AMPD2	AMPD2	chr1	+	109625302	109625433	109619812	109620278	109625661	109625792	26	6	5	11	6	2	0	2	0	3	98	49	1	1	0.542	0.333	0.556	NA			0.25	0.227
SE	6851	AMPD2	AMPD2	chr1	+	109625302	109625433	109620580	109621266	109625661	109625792	24	5	2	4	3	0	0	2	0	0	98	49	1	1	0.75	0.455	1	NA			1	-0.265
SE	6852	AMPD2	AMPD2	chr1	+	109625661	109625792	109619812	109620278	109626159	109626228	33	38	5	0	0	0	2	10	0	1	98	49	1	1	1	1	1		1		0.833	0.084
SE	6854	AMPD2	AMPD2	chr1	+	109629326	109629490	109629108	109629235	109629795	109629916	36	18	5	1	0	0	2	5	0	0	98	49	1	1	0.947	1	1		1		1	-0.018
SE	6856	AMPD2	AMPD2	chr1	+	109630682	109630793	109630232	109630406	109630942	109632055	93	61	25	3	3	1	0	9	0	0	98	49	1	1	0.939	0.91	0.926	NA			1	-0.075
SE	6867	TXNIP	TXNIP	chr1	-	145994543	145994800	145994280	145994437	145994928	145995031	796	229	218	0	0	0	17	121	0	1	98	49	1	1	1	1	1		1		0.984	0.008
SE	6869	C11orf21	C11orf21	chr11	-	2300519	2300613	2299427	2299707	2300683	2300768	5	0	0	2	0	0	0	1	0	0	98	49	1	1	0.556	NA	NA	NA			1	-0.444
SE	6871	RNF4	RNF4	chr4	+	2470986	2471109	2469067	2469258	2490336	2490502	1	3	0	5	0	4	0	0	0	2	98	49	1	1	0.091	1	0	NA			0	0.364
SE	6872	RNF4	RNF4	chr4	+	2511955	2511965	2500658	2500738	2512437	2512597	14	2	0	2	0	0	2	3	0	0	59	49	1	1	0.853	1	NA		1		1	-0.074
SE	6882	ZNF655	ZNF655	chr7	+	99571687	99571792	99560532	99560695	99572244	99576456	4	3	0	1	1	0	0	1	0	0	98	49	1	1	0.667	0.6	NA	NA			1	-0.367
SE	6883	ZNF655	ZNF655	chr7	+	99571687	99571792	99562367	99562494	99572244	99576456	5	4	0	2	0	0	0	4	0	0	98	49	1	1	0.714	1	0	NA			1	-0.429
SE	6890	MFSD10	MFSD10	chr4	-	2931554	2931663	2931374	2931465	2931824	2931936	2	2	0	2	0	0	0	2	0	0	98	49	1	1	0.333	1	NA	NA			1	-0.334
SE	6896	SLC43A2	SLC43A2	chr17	-	1591268	1591471	1590801	1590948	1591565	1591699	123	35	27	0	0	0	5	19	0	1	98	49	1	1	1	1	1		1		0.905	0.047
SE	6897	SLC43A2	SLC43A2	chr17	-	1616561	1616769	1614978	1615034	1627714	1627920	74	25	23	7	1	5	0	9	0	1	98	49	1	1	0.841	0.926	0.697	NA			0.818	0.003
SE	6901	CASP1	CASP1	chr11	-	105026225	105026356	105025509	105025600	105026841	105026951	97	20	29	1	2	0	2	29	0	0	98	49	1	1	0.98	0.833	1		1		1	-0.062
SE	6903	CASP1	CASP1	chr11	-	105033063	105033126	105031164	105031280	105034207	105034474	92	40	27	11	0	3	0	28	0	6	98	49	1	1	0.807	1	0.818	NA			0.7	0.175
SE	6904	PPTC7	PPTC7	chr12	-	110538143	110538273	110534431	110537095	110539821	110539945	11	5	6	2	0	0	0	1	0	0	98	49	1	1	0.733	1	1	NA			1	-0.089
SE	6917	TMEM41B	TMEM41B	chr11	-	9286454	9286593	9280654	9283593	9287701	9287806	6	10	0	1	0	0	0	1	0	0	98	49	1	1	0.75	1	NA	NA			1	-0.125
SE	6921	DAP3	DAP3	chr1	+	155729041	155729122	155727607	155727738	155729207	155729366	11	3	1	0	2	0	0	4	0	0	98	49	1	1	1	0.429	1	NA			1	-0.19
SE	6922	KIF21B	KIF21B	chr1	-	200983055	200983094	200980959	200981096	200984858	200984972	2	0	0	3	0	0	0	1	0	0	88	49	1	1	0.271	NA	NA	NA			1	-0.729
SE	6923	KIF21B	KIF21B	chr1	-	201004765	201004933	201004339	201004455	201005307	201005442	25	8	7	1	0	1	2	3	0	0	98	49	1	1	0.926	1	0.778		1		1	-0.099
SE	6927	SNHG12	SNHG12	chr1	-	28581110	28581229	28580559	28580646	28581589	28581854	0	1	0	1	0	0	0	1	0	0	98	49	1	1	0	1	NA	NA			1	-0.5
SE	6931	IL17RA	IL17RA	chr22	+	17100354	17100481	17098774	17098887	17101995	17102043	64	25	25	8	1	0	0	15	0	0	98	49	1	1	0.8	0.926	1	NA			1	-0.091
SE	6932	IL17RA	IL17RA	chr22	+	17103493	17103577	17102138	17102302	17104725	17104810	73	13	14	1	0	0	0	8	0	0	98	49	1	1	0.973	1	1	NA			1	-0.009
SE	6943	CDA	CDA	chr1	+	20604927	20605039	20588950	20589283	20613841	20613899	26	20	25	0	1	0	0	15	0	0	98	49	1	1	1	0.909	1	NA			1	-0.03
SE	6944	CDA	CDA	chr1	+	20604927	20605039	20588950	20589283	20618451	20618907	15	12	15	0	0	0	0	14	0	1	98	49	1	1	1	1	1	NA			0.875	0.125
SE	6945	CDA	CDA	chr1	+	20613841	20613899	20588950	20589283	20618451	20618907	26	7	10	0	0	0	0	3	0	1	98	49	1	1	1	1	1	NA			0.6	0.4
SE	6946	CDA	CDA	chr1	+	20613841	20613899	20604927	20605039	20618451	20618907	37	14	20	0	0	0	0	6	0	2	98	49	1	1	1	1	1	NA			0.6	0.4
SE	6948	SDHA	SDHA	chr5	+	230875	231000	228184	228333	233476	233645	4	3	2	1	0	0	1	1	0	0	98	49	1	1	0.667	1	1		1		1	-0.111
SE	6950	OSBPL9	OSBPL9	chr1	+	51669433	51669512	51651990	51652041	51714002	51714079	24	12	4	2	0	0	0	10	0	0	98	49	1	1	0.857	1	1	NA			1	-0.048
SE	6951	OSBPL9	OSBPL9	chr1	+	51740142	51740211	51714002	51714079	51745535	51745631	0	1	3	10	1	0	0	0	0	2	98	49	1	1	0	0.333	1	NA			0	0.444
SE	6966	HCLS1	HCLS1	chr3	-	121647318	121647448	121644817	121644928	121657278	121657352	202	87	88	3	0	0	1	33	0	1	98	49	1	1	0.971	1	1		1		0.943	0.019
SE	6967	AP1G2	AP1G2	chr14	-	23565815	23565892	23565605	23565701	23566063	23566160	0	3	0	0	0	0	0	2	0	1	98	49	1	1	NA	1	NA	NA			0.5	0.5
SE	6968	AP1G2	AP1G2	chr14	-	23566561	23566686	23566277	23566419	23567110	23567319	10	4	5	3	1	2	0	4	0	0	98	49	1	1	0.625	0.667	0.556	NA			1	-0.384
SE	6969	EIF4G3	EIF4G3	chr1	-	20827616	20827698	20825099	20825198	20829146	20829272	6	1	2	1	0	0	0	1	0	0	98	49	1	1	0.75	1	1	NA			1	-0.083
SE	6970	EIF4G3	EIF4G3	chr1	-	20892618	20892729	20886200	20886371	20893516	20893636	6	1	0	2	2	0	0	0	0	1	98	49	1	1	0.6	0.2	NA	NA			0	0.4
SE	6976	DSC2	DSC2	chr18	-	31068298	31068344	31065973	31068212	31068893	31069151	0	1	2	1	2	0	0	0	0	1	95	49	1	1	0	0.205	1	NA			0	0.402

SE	6986 MVP	MVP	chr16	+	29841914	29842112	29841595	29841840	29844492	29844879	74	15	23	1	0	0	8	14	0	0	98	49	1	1	0.974	1	1	1	1	-0.009
SE	6987 MVP	MVP	chr16	+	29844492	29844879	29841914	29842112	29845862	29845979	73	23	34	1	0	0	5	10	0	0	98	49	1	1	0.973	1	1	1	1	-0.009
SE	6990 MPDU1	MPDU1	chr17	+	7586898	7587017	7586691	7586777	7587160	7587271	3	0	0	1	0	0	0	1	0	1	98	49	1	1	0.6	NA	NA	NA	0.333	0.267
SE	6996 SAMD9	SAMD9	chr7	-	93114794	93114895	93099512	93106105	93117862	93118023	48	33	1	3	0	0	0	9	0	0	98	49	1	1	0.889	1	1	NA	1	-0.037
SE	6997 PUM2	PUM2	chr2	-	20263192	20263460	20260336	20260466	20282946	20283231	20	4	1	1	0	0	0	2	0	0	98	49	1	1	0.909	1	1	NA	1	-0.03
SE	6999 PUM2	PUM2	chr2	-	20290651	20290790	20283342	20283486	20294375	20294518	24	12	5	1	1	0	0	1	0	0	98	49	1	1	0.923	0.857	1	NA	1	-0.073
SE	7000 PUM2	PUM2	chr2	-	20294375	20294518	20283342	20283486	20297552	20297678	13	9	1	2	0	0	3	2	0	0	98	49	1	1	0.765	1	1	1	1	-0.078
SE	7004 THBS3	THBS3	chr1	-	155200450	155200610	155199994	155200113	155200896	155201004	7	5	0	0	1	0	0	1	0	0	98	49	1	1	1	0.714	NA	NA	1	-0.143
SE	7008 TACC1	TACC1	chr8	+	38827167	38827375	38825307	38825368	38831124	38831177	20	5	8	1	0	0	0	1	0	0	98	49	1	1	0.909	1	1	NA	1	-0.03
SE	7009 SFI1	SFI1	chr22	+	31528689	31528863	31508254	31508376	31546860	31546971	6	13	3	1	3	0	0	0	0	1	98	49	1	1	0.75	0.684	1	NA	0	0.811
SE	7011 SFI1	SFI1	chr22	+	31546860	31546971	31528689	31528863	31550253	31550348	7	13	2	1	0	0	0	1	0	0	98	49	1	1	0.778	1	1	NA	1	-0.074
SE	7012 SFI1	SFI1	chr22	+	31561289	31561392	31556941	31557059	31573057	31573214	22	16	3	1	0	0	0	4	0	0	98	49	1	1	0.917	1	1	NA	1	-0.028
SE	7014 SFI1	SFI1	chr22	+	31613601	31613855	31613353	31613530	31614788	31614860	9	5	7	1	0	0	2	0	0	0	98	49	1	1	0.818	1	1	1	NA	-0.061
SE	7024 ELOVL5	ELOVL5	chr6	-	53270592	53270727	53267397	53269270	53273219	53273344	79	34	58	0	0	0	0	15	0	2	98	49	1	1	1	1	1	NA	0.789	0.211
SE	7025 ELOVL5	ELOVL5	chr6	-	53273219	53273344	53270592	53270727	53275089	53275261	107	42	48	1	0	0	1	21	0	0	98	49	1	1	0.982	1	1	1	1	-0.006
SE	7038 MPHOSPH9	MPHOSPH9	chr12	-	123203249	123203375	123202623	123203084	123210055	123210162	2	0	0	0	0	0	0	0	0	1	98	49	1	1	1	NA	NA	NA	0	1
SE	7039 MPHOSPH9	MPHOSPH9	chr12	-	123214743	123214834	123210055	123210162	123218375	123218499	3	0	0	0	0	0	0	0	0	3	98	49	1	1	1	NA	NA	NA	0	1
SE	7043 RHOT2	RHOT2	chr16	+	668487	668569	668352	668411	668655	668699	13	2	0	2	0	0	3	3	0	0	98	49	1	1	0.765	1	NA	1	1	-0.117
SE	7044 RHOT2	RHOT2	chr16	+	669552	669606	668655	668699	670122	670175	5	2	0	0	0	0	0	1	2	0	98	49	1	1	1	1	NA	0	1	0.5
SE	7045 RHOT2	RHOT2	chr16	+	670674	670773	670455	670557	670891	671000	12	1	0	1	0	0	0	3	0	0	98	49	1	1	0.857	1	NA	NA	1	-0.072
SE	7047 SLC25A44	SLC25A44	chr1	+	156196012	156196173	156193931	156194247	156199834	156200472	0	0	1	5	0	0	0	0	0	1	98	49	1	1	0	NA	1	NA	0	0.5
SE	7058 CHD1	CHD1	chr5	-	98858179	98858390	98855203	98856725	98858963	98859015	24	5	3	2	0	0	0	2	0	1	98	49	1	1	0.857	1	1	NA	0.5	0.452
SE	7059 CHD1	CHD1	chr5	-	98876397	98876558	98875071	98875113	98879551	98879728	17	7	0	4	5	0	1	2	0	0	98	49	1	1	0.68	0.412	NA	1	1	-0.454
SE	7061 CHD1	CHD1	chr5	-	98896225	98896442	98894596	98894686	98897192	98897320	51	8	1	0	1	0	1	8	0	0	98	49	1	1	1	0.8	1	1	1	-0.067
SE	7066 GPBP1	GPBP1	chr5	+	57221375	57221396	57214073	57214193	57230845	57230969	9	3	0	25	2	4	0	3	0	5	70	49	1	1	0.201	0.512	0	NA	0.296	-0.058
SE	7067 GPBP1	GPBP1	chr5	+	57250953	57251141	57249408	57249576	57261179	57261282	52	20	21	0	1	0	0	10	0	0	98	49	1	1	1	0.909	1	NA	1	-0.03
SE	7069 PAPD4	PAPD4	chr5	+	79641104	79641196	79640850	79640965	79642843	79642910	13	6	2	0	1	0	0	1	0	0	98	49	1	1	1	0.75	1	NA	1	-0.083
SE	7070 PAPD4	PAPD4	chr5	+	79642831	79642910	79641104	79641196	79645122	79645192	8	4	5	0	0	0	0	4	0	1	98	49	1	1	1	1	1	NA	0.667	0.333
SE	7071 PAPD4	PAPD4	chr5	+	79642843	79642910	79641104	79641196	79645122	79645192	9	7	6	0	0	0	0	1	0	1	98	49	1	1	1	1	1	NA	0.333	0.667
SE	7072 PAPD4	PAPD4	chr5	+	79649061	79649190	79648616	79648693	79656957	79657001	24	5	2	4	0	0	0	0	0	1	98	49	1	1	0.75	1	1	NA	0	0.917
SE	7073 PAPD4	PAPD4	chr5	+	79656957	79657001	79649061	79649190	79668891	79669028	16	4	2	1	0	0	0	3	0	0	93	49	1	1	0.894	1	1	NA	1	-0.035
SE	7083 PPP3CB	PPP3CB	chr10	-	73439871	73439901	73436427	73438420	73444724	73444822	11	1	0	2	0	0	0	1	0	2	79	49	1	1	0.773	1	NA	NA	0.237	0.65
SE	7087 GPR108	GPR108	chr19	-	6731890	6731934	6731472	6731522	6732024	6732155	17	0	4	1	0	0	0	5	0	0	93	49	1	1	0.9	NA	1	NA	1	-0.05
SE	7088 GPR108	GPR108	chr19	-	6732986	6733062	6732475	6732549	6733167	6733301	17	11	15	2	0	0	0	7	0	0	98	49	1	1	0.81	1	1	NA	1	-0.063
SE	7090 MEGF6	MEGF6	chr1	-	3498697	3498826	3498370	3498499	3499587	3499716	9	2	0	1	0	1	0	0	3	0	98	49	1	1	0.818	1	0	0	NA	0.606
SE	7091 MEGF6	MEGF6	chr1	-	3499137	3499266	3498697	3498826	3499587	3499716	15	6	2	1	0	0	1	3	0	0	98	49	1	1	0.882	1	1	1	1	-0.039
SE	7092 MEGF6	MEGF6	chr1	-	3511549	3511687	3510782	3510902	3512005	3512128	14	13	3	1	0	0	0	1	0	0	98	49	1	1	0.875	1	1	NA	1	-0.042
SE	7095 ABCC5	ABCC5	chr3	-	183987769	183987917	183985303	183985378	183988571	183988727	9	8	1	1	0	0	2	1	0	0	98	49	1	1	0.818	1	1	1	1	-0.061
SE	7096 DPP8	DPP8	chr15	-	65450998	65451110	65445659	65447006	65451959	65452102	3	0	1	1	0	0	0	1	0	0	98	49	1	1	0.6	NA	1	NA	1	-0.2
SE	7100 DPP8	DPP8	chr15	-	65500605	65500779	65497863	65498032	65507242	65507355	7	0	0	4	0	0	0	3	0	0	98	49	1	1	0.467	NA	NA	NA	1	-0.533
SE	7103 ANKRD12	ANKRD12	chr18	+	9208656	9208803	9195550	9195698	9211583	9211784	52	32	9	1	0	0	1	5	0	0	98	49	1	1	0.963	1	1	1	1	-0.012
SE	7108 USP47	USP47	chr11	+	11891967	11892106	11884466	11884580	11897596	11897693	11	2	0	0	0	0	0	1	0	2	98	49	1	1	1	1	NA	NA	0.2	0.8
SE	7109 USP47	USP47	chr11	+	11905398	11905548	11903262	11903342	11920155	11920251	11	7	1	0	0	0	0	0	0	1	98	49	1	1	1	1	1	NA	0	1
SE	7115 GNB1	GNB1	chr1	-	1804418	1804581	1793244	1793311	1806474	1806538	21	15	6	1	1	0	0	1	0	0	98	49	1	1	0.913	0.882	1	NA	1	-0.068
SE	7116 GNB1	GNB1	chr1	-	1839189	1839238	1825396	1825499	1890819	1891117	27	19	12	1	0	0	2	5	1	0	98	49	1	1	0.931	1	1	0.5	1	0.227
SE	7119 PRDM2	PRDM2	chr1	+	13715540	13715614	13700239	13700300	13730999	13731117	2	4	1	0	0	0	0	0	0	2	98	49	1	1	1	1	1	NA	0	1
SE	7124 LAP3	LAP3	chr4	+	17597045	17597134	17595409	17595534	17598455	17598558	10	3	0	0	1	0	0	13	0	0	98	49	1	1	1	0.6	NA	NA	1	-0.2
SE	7125 AMDHD2	AMDHD2	chr16	+	2527560	2527615	2520983	2521123	2527772	2527985	8	8	5	1	0	0	1	0	0	0	98	49	1	1	0.8	1	1	1	NA	-0.067
SE	7129 MBD6	MBD6	chr12	+	57527847	57528017	57527506	57527660	57528146	57528560	22	7	1	1	0	0	1	4	0	0	98	49	1	1	0.917	1	1	1	1	-0.028
SE	7131 SIVA1	SIVA1	chr14	+	104755629	104755824	104753132	104753319	104756603	104756760	7	1	0	1	0	0	0	1	0	0	98	49	1	1	0.778	1	NA	NA	1	-0.111
SE	7135 PPM1M	PPM1M	chr3	+	52246973	52247228	52246694	52246812	52247681	52247794	18	3	5	2	0	0	2	4	0	0	98	49	1	1	0.818	1	1	1	1	-0.061
SE	7136 PPM1M	PPM1M	chr3	+	52247681	52247794	52246694	52246812	52248152	52248237	13	2	9	1	1	0	0	0	0	1	98	49	1	1	0.867	0.5	1	NA	0	0.789
SE	7139 FANCM	FANCM	chr14	+	45140631	45140709	45137068	45137241	45148836	45148995	0	2	0	0	0	3	0	0	0	1	98	49	1	1	NA	1	0	NA	0	0.5
SE	7144 ISCU	ISCU	chr12	+	108567657	108567743	108567189	108567268	108568830	108569384	0	1	0	28	21	15	0	0	2	8	98	49	1	1	0	0.023	0	0	0	0.008
SE	7147 CDC73	CDC73	chr1	+	193236255	193236356	193232992	193233154	193249729	193249871	4	2	3	0	2	0	0	5	0	0	98	49	1	1	1	0.333	1	NA	1	-0.222
SE	7152 FHL3	FHL3	chr1	-	37997962	37998132	37997683	37997870	37998973	37999148	7	4	4	1	0	0	0	7	0	0	98	49	1	1	0.778	1	1	NA	1	-0.074
SE	7154 UROD	UROD	chr1	+	45013908	45014070	45013593	45013791	45014438	45014576	0	1	0	0	0	0	0	1	0	2	98	49	1	1	NA	1	NA	NA	0.2	0.8
SE	7155 FOXN3	FOXN3	chr14	-	89190383	89190449	89180700	89180806	89280949	89281014	3	6	0	3	1	0	1	1	0	1	98	49	1	1	0.333	0.75	NA	1	0.333	-0.125
SE	7168 PSMA4	PSMA4	chr15	+	78542482	78542645	78542176	78542219	78544189	78544267	16	4	9	0	1	0	1	4	0	1	98	49	1	1	1	0.667	1	1	0.667	0.055
SE	7177 RGS12	RGS12	chr4	+	3428557	3428711	3428089	3428169	3430406	3430955	2	0	0	2	0	0	0	0	0	1	98	49	1	1	0.333	NA	NA	NA	0	0.333
SE	7178 CSNK1G1	CSNK1G1	chr15	-	64259200	64259241	64251511	64251581	64300318	64300723	3	6	0	2	0	0	0	2	0	1	90	49	1	1	0.45	1	NA	NA	0.521	0.204
SE	7181 TRPT1	TRPT1	chr11	-	64224099	64224210	64223798	64223967	64224284	64224341	2	1	0	0	0	0	0	0	0	1	98	49	1	1	1	1	NA	NA	0	1
SE	7182 TRPT1	TRPT1	chr11	-	64224099	64224213	64223798	64223967	64224284	64224341	2	0	0	0	0	0	0	0	0	1	98	49	1	1	1	NA	NA	NA	0	1
SE	7184 AFG3L2	AFG3L2	chr18	-	12367275	12367382	12366964	12367117	12370848	12370926	5	7	0	0	0	0	0	0	0	1	98	49	1	1	1	1	NA	NA	0	1
SE	7187 NEK4	NEK4	chr3	-	52749691	52749829	52746733	52746904	52751931	52752336	0	0	0	2	0	0	0	2	0	0	98	49	1	1	0	NA	NA	NA	1	-1
SE	7189 SREK1	SREK1	chr5	+	66153462	66153596	66144217	66144537	66159218	66159334	6	2	0	0	1	0	0	1	0	0	98	49	1	1	1	0.5	NA	NA	1	-0.25
SE	7190 SREK1	SREK1	chr5	+	66158808	66158932	66153462	66153596	66159218	66159334	1	0	1	4	0	0	0	1	0	0	98	49	1	1	0.111	NA	1	NA	1	-0.445
SE	7191 SREK1	SREK1	chr5	+	66177513	66177658	66174945	66175041	66178718	66183616	18	8	0	0	3	0	0	2	0	0	98	49	1	1	1	0.571	NA	NA	1	-0.215
SE	7192 SREK1	SREK1	chr5	+	66177516	66177658	66174945	66175041	66178718	66183616	14	3	0	0	3	0	0	1	0	0	98	49	1	1	1	0.333	NA	NA	1	-0.334
SE	7194 RERE	RERE	chr1	-	8362682	8362844	8361762	8361876	8364055	8364255	36	14	6	0	0	0	2	6	0	1	98	49	1	1	1	1	1	1	0.75	0.125
SE	7201 NBEAL2	NBEAL2	chr3	+	46992474	46992555	46991839	46991946	46993936	46994020	32	12	3	29	2	9	0	10	0	9	98	49	1	1	0.356	0.75	0.143	NA	0.357	0.059
SE	7202 NBEAL2	NBEAL2	chr3	+	46996750	46996833	46996270	46996592	46996953	46997046	43	31	5	2	0	0	7	12	0	0	98	49	1	1	0.915	1	1	1	1	-0.028
SE	7203 NBEAL2	NBEAL2	chr3	+	46998066	46998226	46997560	46997694	46998462	46998565	95	41	7	1	0	0	5	29	0	0	98	49	1	1	0.979	1	1	1	1	-0.007
SE	7204 NBEAL2	NBEAL2	chr3	+	47001278	47001438	47001000	47001179	47001688	47001826	117	46	6	0	1	0	9	30	0	0	98	49	1	1	1	0.958	1	1	1	-0.014
SE	7205 NBEAL2	NBEAL2	chr3	+	47001919	47002288	47001688	47001826	47002370	47002520	90	39	9	0	1	0	2	25	0	0	98	49	1	1	1	0.951	1	1	1	-0.016
SE	7207 NBEAL2	NBEAL2	chr3	+	47003815	47003976	47003173	47003309	47004076	47004393	88	20	7	2	0	0	11	24	0	0	98	49	1	1	0.957	1	1	1	1	-0.014
SE	7208 NBEAL2	NBEAL2	chr3	+	47005945	47006063	47005737	47005847	47006164	47006262	111	38	17	0	2	0	6	28	0	0	98	49	1	1	1	0.905	1	1	1	-0.032
SE	7212 NBEAL2	NBEAL2	chr3	+	47008282	47008441	47007815	47007910	47008519	47008668	84	31	11	1	0	0	5	33	0	0	98	49	1	1	0.977	1	1	1	1	-0.008
SE	7225 UPF2	UPF2	chr10	-	11979056	11979165	11967340	11967454	11997671	11997757	19	14	5	0	1	0	0	5	0	0	98	49	1	1	1	0.875	1	NA	1	-0.042
SE	7227 UBE2Q1	UBE2Q1	chr1	-	154551396	154551492	154550937	154551004	154551770	154551819	9	6	6	1	0	0	0	1	0	0	98	49	1	1	0.818	1	1	NA	1	-0.061
SE	7228 UBE2Q1	UBE2Q1	chr1	-	154552735	154552820	154552403	154552464	154553031	154553172	12	6	2	0	1	0	0	2	0	0	98	49	1	1	1	0.75	1	NA	1	-0.083
SE	7234 PPP3CA	PPP3CA	chr4	-	101029165	101029195	101023429	101026061	101032266	101032364	3	0	0	22	10	3	0	0	0	2	79	49	1	1	0.078	0	0	NA	0	0.026
SE	7235 PPP3CA	PPP3CA	chr4	-	101063231	101063357	101061086	101061161	101080531	101080626	15	8	2	0	0	0	0	15	0	3	98	49	1	1	1	1	1	NA	0.714	0.286
SE	7240 MTR	MTR	chr1	+	236831965	236832078	236829188	236829268	236835546	236835687	6	4	0	5	0	0	0	2	0	0	98	49	1	1	0.375	1	NA	NA	1	-0.312
SE	7241 MTR	MTR	chr1	+	236852520	236852637	236850343	236850523	236852947	236853088	4	0	0	1	0	0	0	3	0	0	98	49	1	1	0.667	NA	NA	NA	1	-0.333
SE	7242 MTR	MTR	chr1	+	236861124	236861277	236859832	236859922	236862235	236862343	5	0	1	1	0	0	0	3	0	0	98	49	1	1	0.714	NA	1	NA	1	-0.143
SE	7243 SSH1	SSH1	chr12	-	108798999	108799200	108791918	108792829	108800779	108800926	3	4	1	1	0	0	0	1	0	0	98	49	1	1	0.6	1	1	NA	1	-0.133
SE	7244 SSH1	SSH1	chr12	-	108802321	108802368	108800779	108800926	108805055	108805184	7	4	0	1	2	1	0	1	0	0	96	49	1	1	0.781	0.505	0	NA	1	-0.571
SE	7245 USP48	USP48	chr1	-	21721649	21721764	21721035	21721166	21723897	21724095	14	7	0	1	2	0	0	3	0	0	98	49	1	1	0.875	0.636	NA	NA	1	-0.244
SE	7246 USP48	USP48	chr1	-	21736445	21736625	21729703	21729832	21747066	21747149	7	1	2	1	0	0	1	4	0	0	98	49	1	1	0.778	1	1	1	1	-0.074
SE	7255 PANK4	PANK4	chr1	-	2512887	2513039	2511627	2511683	2514001	2514089	19	7	0	6	0	0	0	4	0	0	98	49	1	1	0.613	1	NA	NA	1	-0.194
SE	7256 PANK4	PANK4	chr1	-	2515561	2515717	2514353	2514466	2518163	2518264	9	11	1	0	0	0	0	0	1	0	98	49	1	1	1	1	1	0	NA	1
SE	7268 HEXA	HEXA	chr15	-	72349078	72349259	72348047	72348134	72350517	72350650	10	3	0	0	0	0	0	3	0	2	98	49	1	1	1	1	NA	NA	0.429	0.571
SE	7270 SESN2	SESN2	chr1	+	28273357	28273508	28272580	28272793	28274039	28274158	0	2	2	0	4	0	0	1	0	0	98	49	1	1	NA	0.2	1	NA	1	-0.4
SE	7271 STK40	STK40	chr1	-	36344119	36344264	36343859	36343979	36348699	36348815	32	23	29	0	2	0	3	6	0	0	98	49	1	1	1	0.852	1	1	1	-0.049
SE	7272 STK40	STK40	chr1	-	36358736	36358822	36358238	36358382	36361220	36361340	40	24	21	0	1	0	1	4	0	0	98	49	1	1	1	0.923	1	1	1	-0.026
SE	7273 STK40	STK40	chr1	-	36358736	36358837	36358238	36358382	36361220	36361340	29	18	9	0	1	0	1	3	0	0	98	49	1	1	1	0.9	1	1	1	-0.033
SE	7274 STK40	STK40	chr1	-	36361220	36361340	36358736	36358822	36385722	36385927	30	13	15	1	0	0	0	1	0	0	98	49	1	1	0.937	1	1	NA	1	-0.021
SE	7277 MFN2	MFN2	chr1	+	11989164	11989343	11981969	11982114	11992554	11992690	66	13	5	1	0	0	0	10	0	0	98	49	1	1	0.971	1	1	NA	1	-0.01
SE	7278 MFN2	MFN2	chr1	+	12007052	12007249	12006537	12006693	12009591	12009726	25	16	10	2	0	0	0	5	1	0	98	49	1	1	0.862	1	1	0	1	0.454
SE	7279 SHC1	SHC1	chr1	-	154965542	154965781	154962297	154963931	154965945	154966077	6	1	0	0	2	0	0	1	0	0	98	49	1	1	1	0.2	NA	NA	1	-0.4
SE	7280 SHC1	SHC1	chr1	-	154968203	154968257	154967979	154968031	154968494	154968614	7	1	2	0	2	0	0	3	0	0	98	49	1	1	1	0.2	1	NA	1	-0.267
SE	7291 IL6R	IL6R	chr1	+	154435968	154436110	154434989	154435156	154448124	154448171	67	21	27	4	6	1	0	7	0	0	98	49	1	1	0.893	0.636	0.931	NA	1	-0.18
SE	7292 IL6R	IL6R	chr1	+	154449910	154449980	154448124	154448171	154465133	154469450	25	9	9	0	0	6	0	5	0	0	98	49	1	1	1	1	0.429	NA	1	-0.19
SE	7293 IL6R	IL6R	chr1	+	154454487	154454581	154449910	154449980	154465133	154469450	30	17	4	0	1	0	0	4	0	2	98	49	1	1	1	0.895	1	NA	0.5	0.465
SE	7296 IST1	IST1	chr16	+	71924768	71924817	71923287	71923380	71927613	71930637	4	10	5	1	0	0	0	1	0	0	98	49	1	1	0.667	1	1	NA	1	-0.111
SE	7297 IST1	IST1	chr16	+	71924772	71924817	71923287	71923380	71927613	71930637	4	5	3	1	0	0	0	1	0	0	94	49	1	1	0.676	1	1	NA	1	-0.108
SE	7298 KIAA0040	KIAA0040	chr1	-	175177610	175177684	175166561	175166737	175192639	175192716	46	29	2	2	0	0	2	7	0	0	98	49	1	1	0.92	1	1	1	1	-0.027
SE	7307 MAX	MAX	chr14	-	65077327	65077428	65075123	65076663	65077912	65078036	16	3	9	15	4	2	0	0	0	1	98	49	1	1	0.348	0.273	0.692	NA	0	0.438
SE	7311 ANKDD1A	ANKDD1A	chr15	+	64942466	64942565	64934135	64934234	64943483	64943582	0	5	0	0	2	0	0	2	0	0	98	49	1	1	NA	0.556	NA	NA	1	-0.444
SE	7316 MIER1	MIER1	chr1	+	66963087	66963160	66959678	66959743	66970807	66970959	38	31	20	0	3	0	0	11	0	0	98	49	1	1	1	0.838	1	NA	1	-0.054
SE	7317 MSH5-SAPCD1	MSH5-SAPCD1	chr6	+	31758547	31758620	31758164	31758293	31758765	31758875	1	0	0	1	0	0	0	1	0	0	98	49	1	1	0.333	NA	NA	NA	1	-0.667
SE	7318 VPS11	VPS11	chr11	+	119071595	119071843	119070233	119070397	119073197	119073399	10	0	0	0	0	0	0	0	0	2	98	49	1	1	1	NA	NA	NA	0	1
SE	7325 KIAA0513	KIAA0513	chr16	+	85078414	85078455	85077424	85077632	85078924	85079003	39	11	2	0	0	0	0	9	1	0	90	49	1	1	1	1	1	0	1	0.5
SE	7326 KIAA0513	KIAA0513	chr16	+	85082563	85082593	85081314	85081392	85086643	85086724	1	0	0	18	4	11	0	0	2	3	79	49	1	1	0.033	0	0	0	0	0.011
SE	7328 PI4KA	PI4KA	chr22	-	20727773	20727864	20727229	20727397	20729312	20729506	4	4	0	3	0	0	0	2	0	0	98	49	1	1	0.4	1	NA	NA	1	-0.3
SE	7329 PI4KA	PI4KA	chr22	-	20744627	20744720	20742607	20742764	20747582	20747702	13	5	0	0	1	0	0	2	0	0	98	49	1	1	1	0.714	NA	NA	1	-0.143
SE	7330 PI4KA	PI4KA	chr22	-	20764816	20764950	20761303	20761386	20765099	20765236	18	9	0	0	1	0	0	1	0	0	98	49	1	1	1	0.818	NA	NA	1	-0.091
SE	7331 PI4KA	PI4KA	chr22	-	20796145	20796314	20793192	20793243	20798583	20798687	11	1	0	0	0	0	0	0	0	1	98	49	1	1	1	1	NA	NA	0	1
SE	7332 PI4KA	PI4KA	chr22	-	20818482	20818549	20813357	20813506	20819640	20819900	22	6	0	2	0	0	2	10	0	0	98	49	1	1	0.846	1	NA	1	1	-0.077
SE	7336 TSEN15	TSEN15	chr1	+	184072156	184072298	184054727	184054863	184072826	184074210	0	0	0	1	1	0	0	1	0	0	98	49	1	1	0	0	NA	NA	1	-1
SE	7342 ADA	ADA	chr20	-	44623006	44623078	44622828	44622930	44624201	44624329	1	0	0	3	0	0	0	4	0	0	98	49	1	1	0.143	NA	NA	NA	1	-0.857
SE	7350 RASA3	RASA3	chr13	-	114040999	114041094	114029810	114029887	114052051	114052155	29	14	0	0	1	0	0	4	0	0	98	49	1	1	1	0.875	NA	NA	1	-0.062
SE	7367 ZNF324	ZNF324	chr19	+	58469179	58469306	58467044	58467183	58469727	58469844	3	1	0	0	1	0	0	1	0	0	98	49	1	1	1	0.333	NA	NA	1	-0.334
SE	7370 SMAP2	SMAP2	chr1	+	40406735	40406869	40373705	40374223	40408652	40408738	139	72	82	0	2	0	0	53	0	0	98	49	1	1	1	0.947	1	NA	1	-0.018
SE	7377 ATP11A	ATP11A	chr13	+	112816082	112816211	112810618	112810726	112819303	112819407	43	25	0	1	0	0	0	8	0	0	98	49	1	1	0.956	1	NA	NA	1	-0.022
SE	7378 ATP11A	ATP11A	chr13	+	112824343	112824425	112823344	112823409	112826693	112826891	26	9	1	0	1	0	0	3	0	0	98	49	1	1	1	0.818	1	NA	1	-0.061
SE	7379 ATP11A	ATP11A	chr13	+	112825429	112825580	112824343	112824425	112826693	112826891	16	10	3	1	1	0	4	5	0	0	98	49	1	1	0.889	0.833	1	1	1	-0.093
SE	7380 ATP11A	ATP11A	chr13	+	112851036	112851218	112842275	112842379	112854278	112854530	13	9	11	0	1	0	0	8	0	0	98	49	1	1	1	0.818	1	NA	1	-0.061
SE	7381 ATP11A	ATP11A	chr13	+	112855910	112856085	112854278	112854530	112857817	112857920	7	8	3	2	0	0	0	4	0	0	98	49	1	1	0.636	1	1	NA	1	-0.121
SE	7382 ATP11A	ATP11A	chr13	+	112858144	112858290	112857817	112857920	112859392	112859452	20	5	0	1	1	0	1	1	0	1	98	49	1	1	0.909	0.714	NA	1	0.333	0.145
SE	7383 ATP11A	ATP11A	chr13	+	112878216	112878303	112875775	112875941	112881875	112887168	2	2	3	0	3	0	0	0	0	1	98	49	1	1	1	0.25	1	NA	0	0.75
SE	7384 TRIM21	TRIM21	chr11	-	4388299	4388530	4386967	4386990	4389653	4389749	21	15	17	0	5	0	0	6	0	0	98	49	1	1	1	0.6	1	NA	1	-0.133
SE	7387 CXorf38	CXorf38	chrX	-	40636532	40636712	40630613	40630773	40637006	40637156	11	7	8	0	1	0	0	1	0	0	98	49	1	1	1	0.778	1	NA	1	-0.074
SE	7388 CXorf38	CXorf38	chrX	-	40647006	40647141	40639008	40639128	40647304	40647567	4	3	4	5	0	0	0	1	0	1	98	49	1	1	0.286	1	1	NA	0.333	0.429
SE	7391 DAZAP1	DAZAP1	chr19	+	1429966	1429996	1428841	1428995	1430221	1430362	5	1	0	0	0	1	0	4	0	0	79	49	1	1	1	1	0	NA	1	-0.333
SE	7397 LOC100131564	LOC100131564	chr1	-	93326601	93326662	93325768	93325895	93337376	93339274	0	0	0	4	0	0	0	1	0	0	98	49	1	1	0	NA	NA	NA	1	-1
SE	7404 LOC100289473	LOC100289473	chr20	-	1774510	1774665	1773364	1773542	1778519	1778848	5	2	2	1	0	0	0	1	0	0	98	49	1	1	0.714	1	1	NA	1	-0.095
SE	7405 ZDHHC7	ZDHHC7	chr16	-	84988762	84988873	84981869	84981994	84990303	84990635	1	0	2	8	3	4	0	3	0	6	98	49	1	1	0.059	0	0.2	NA	0.2	-0.114
SE	7406 KIN	KIN	chr10	-	7778837	7779019	7775750	7775799	7780055	7780178	21	4	0	1	0	0	0	2	0	0	98	49	1	1	0.913	1	NA	NA	1	-0.043
SE	7408 HECTD1	HECTD1	chr14	-	31113876	31114059	31113276	31113484	31114285	31114340	6	1	1	0	1	0	0	0	0	1	98	49	1	1	1	0.333	1	NA	0	0.778
SE	7415 NARF	NARF	chr17	+	82483715	82483779	82481081	82481211	82484812	82484950	11	5	12	0	1	0	0	2	0	0	98	49	1	1	1	0.714	1	NA	1	-0.095
SE	7421 TPM3	TPM3	chr1	-	154169304	154169383	154155303	154157721	154170399	154170469	9	2	0	75	46	36	0	4	0	20	98	49	1	1	0.057	0.021	0	NA	0.091	-0.065
SE	7422 TPM3	TPM3	chr1	-	154169304	154169383	154161812	154167940	154170399	154170469	7	2	0	2	0	0	0	3	0	0	98	49	1	1	0.636	1	NA	NA	1	-0.182
SE	7431 C9orf72	C9orf72	chr9	-	27560226	27560299	27558490	27558607	27561584	27561649	20	6	9	1	0	0	0	1	0	0	98	49	1	1	0.909	1	1	NA	1	-0.03
SE	7435 ZNF100	ZNF100	chr19	-	21744940	21745067	21744016	21744115	21765693	21765786	4	2	0	2	0	0	0	1	0	0	98	49	1	1	0.5	1	NA	NA	1	-0.25
SE	7439 TBC1D32	TBC1D32	chr6	-	121170404	121170527	121160947	121161056	121205074	121205163	0	0	0	1	0	0	0	1	0	0	98	49	1	1	0	NA	NA	NA	1	-1
SE	7447 STX16-NPEPL1	STX16-NPEPL1	chr20	+	58673630	58673711	58671153	58671297	58691723	58691780	24	19	3	0	0	0	0	10	0	2	98	49	1	1	1	1	1	NA	0.714	0.286
SE	7448 STX16-NPEPL1	STX16-NPEPL1	chr20	+	58705497	58705569	58701015	58701158	58707122	58707200	0	6	1	11	1	0	0	0	0	1	98	49	1	1	0	0.75	1	NA	0	0.583
SE	7458 KIF1B	KIF1B	chr1	+	10337370	10337533	10337073	10337203	10339768	10339859	9	7	6	2	1	0	0	4	0	0	98	49	1	1	0.692	0.778	1	NA	1	-0.177
SE	7459 KIF1B	KIF1B	chr1	+	10347760	10347827	10345844	10345953	10348648	10348733	8	9	5	0	1	0	0	1	0	0	98	49	1	1	1	0.818	1	NA	1	-0.061
SE	7461 ARID4A	ARID4A	chr14	+	58301579	58301690	58299797	58299860	58304943	58305009	10	12	3	0	1	0	0	3	0	0	98	49	1	1	1	0.857	1	NA	1	-0.048
SE	7463 ARID4A	ARID4A	chr14	+	58366023	58366230	58365517	58365622	58366882	58367029	13	5	2	3	0	0	0	1	0	0	98	49	1	1	0.684	1	1	NA	1	-0.105
SE	7464 AATF	AATF	chr17	+	36986616	36986731	36953769	36953907	36988518	36988720	10	8	13	0	0	0	0	2	0	3	98	49	1	1	1	1	1	NA	0.25	0.75
SE	7467 VPS13D	VPS13D	chr1	+	12319496	12319630	12318071	12318337	12321808	12321964	8	2	1	0	0	1	0	2	0	0	98	49	1	1	1	1	0.333	NA	1	-0.222

SE	7469	VPS13D	VPS13D	chr1	+	12349163	12349374	12348822	12348973	12353973	12354221	3	2	0	0	0	0	0	3	0	1	98	49	1	1	1	1	NA	NA	0.6	0.4
SE	7475	NRBP1	NRBP1	chr2	+	27434470	27434560	27433988	27434090	27434721	27434762	39	15	10	0	0	0	0	5	0	1	98	49	1	1	1	1	1	NA	0.714	0.286
SE	7476	MDM2	MDM2	chr12	+	68816811	68816945	68813553	68813628	68820324	68820374	23	12	9	0	0	0	0	12	0	1	98	49	1	1	1	1	1	NA	0.857	0.143
SE	7477	MDM2	MDM2	chr12	+	68828770	68828931	68824554	68824651	68835828	68835843	70	21	3	1	0	0	0	8	0	0	98	49	1	1	0.972	1	1	NA	1	-0.009
SE	7493	FAM126B	FAM126B	chr2	-	200992886	200993003	200992280	200992368	200997444	200997540	85	21	12	1	0	0	0	15	0	2	98	49	1	1	0.977	1	1	NA	0.789	0.203
SE	7494	FAM126B	FAM126B	chr2	-	200992886	200993003	200992280	200992368	201008972	201009088	39	11	3	1	0	0	0	10	0	0	98	49	1	1	0.951	1	1	NA	1	-0.016
SE	7495	FAM126B	FAM126B	chr2	-	200997444	200997540	200992280	200992368	201008972	201009088	39	9	6	1	0	0	0	6	0	0	98	49	1	1	0.951	1	1	NA	1	-0.016
SE	7496	FAM126B	FAM126B	chr2	-	200997444	200997540	200992886	200993003	201008972	201009088	85	19	15	1	0	0	0	9	0	0	98	49	1	1	0.977	1	1	NA	1	-0.008
SE	7497	FAM126B	FAM126B	chr2	-	201016990	201017170	201011391	201011472	201022830	201022932	47	21	6	1	0	0	1	15	0	0	98	49	1	1	0.959	1	1	1	1	-0.014
SE	7498	SLC12A6	SLC12A6	chr15	-	34238964	34239160	34238231	34238401	34240660	34240829	71	12	3	0	1	0	1	11	0	0	98	49	1	1	1	0.857	1	1	1	-0.048
SE	7499	SLC12A6	SLC12A6	chr15	-	34242101	34242221	34241232	34241337	34243973	34244072	67	19	8	7	5	0	0	7	0	1	98	49	1	1	0.827	0.655	1	NA	0.778	0.049
SE	7500	SLC12A6	SLC12A6	chr15	-	34245284	34245403	34243973	34244072	34245692	34245867	82	40	22	2	0	0	0	16	0	0	98	49	1	1	0.953	1	1	NA	1	-0.016
SE	7506	HAL	HAL	chr12	-	95990392	95990532	95988192	95988240	95992679	95992805	69	12	16	2	0	0	0	8	0	0	98	49	1	1	0.945	1	1	NA	1	-0.018
SE	7507	HAL	HAL	chr12	-	95993771	95993838	95993450	95993488	95993925	95993998	28	14	17	9	0	5	0	3	0	0	98	49	1	1	0.609	1	0.63	NA	1	-0.254
SE	7508	TMEM167B	TMEM167B	chr1	+	109092889	109093021	109090780	109090882	109094416	109096932	13	9	3	1	0	0	0	2	0	0	98	49	1	1	0.867	1	1	NA	1	-0.044
SE	7512	IFFO2	IFFO2	chr1	-	18918361	18918502	18916902	18917042	18919677	18919773	1	0	0	1	0	0	0	2	0	0	98	49	1	1	0.333	NA	NA	NA	1	-0.667
SE	7513	ZEB2	ZEB2	chr2	-	144398300	144400270	144396411	144396592	144401198	144401307	14	3	2	2	1	0	0	8	0	1	98	49	1	1	0.778	0.6	1	NA	0.8	-0.007
SE	7514	ZEB2	ZEB2	chr2	-	144404835	144405024	144403915	144404130	144429768	144430026	18	6	0	0	0	0	1	3	0	1	98	49	1	1	1	1	NA	1	0.6	0.2
SE	7515	ZEB2	ZEB2	chr2	-	144424795	144424867	144404835	144405024	144429768	144430026	15	14	0	1	0	0	0	6	0	1	98	49	1	1	0.882	1	NA	NA	0.75	0.191
SE	7517	MKL1	MKL1	chr22	-	40423535	40423685	40420846	40421100	40429605	40429767	5	6	0	0	1	0	0	1	0	0	98	49	1	1	1	0.75	NA	NA	1	-0.125
SE	7518	MKL1	MKL1	chr22	-	40424205	40424381	40420846	40421100	40429605	40429767	10	3	1	0	1	0	2	1	0	0	98	49	1	1	1	0.6	1	1	1	-0.133
SE	7520	MKL1	MKL1	chr22	-	40594673	40594735	40552105	40552367	40636477	40636719	7	4	0	0	0	0	0	4	0	2	98	49	1	1	1	1	NA	NA	0.5	0.5
SE	7521	EDEM3	EDEM3	chr1	-	184723750	184723856	184721288	184721386	184726254	184726389	22	3	0	3	0	0	0	2	0	0	98	49	1	1	0.786	1	NA	NA	1	-0.107
SE	7522	FAM53B	FAM53B	chr10	-	124696157	124696212	124681606	124682379	124706635	124706887	17	17	5	2	0	0	1	12	0	0	98	49	1	1	0.81	1	1	1	1	-0.063
SE	7524	FAM53B	FAM53B	chr10	-	124706635	124706887	124681606	124682379	124744012	124744361	12	3	0	0	0	3	0	4	0	0	98	49	1	1	1	1	0	NA	1	-0.333
SE	7525	FAM53B	FAM53B	chr10	-	124706635	124706887	124696157	124696212	124744012	124744361	24	14	2	1	0	0	1	16	0	0	98	49	1	1	0.923	1	1	1	1	-0.026
SE	7532	6-Mar	6-Mar	chr5	+	10381799	10381943	10378758	10378832	10386993	10387066	13	6	0	1	0	0	0	5	0	0	98	49	1	1	0.867	1	NA	NA	1	-0.067
SE	7533	6-Mar	6-Mar	chr5	+	10386993	10387066	10378758	10378832	10390331	10390500	2	4	0	1	0	0	0	2	0	0	98	49	1	1	0.5	1	NA	NA	1	-0.25
SE	7534	6-Mar	6-Mar	chr5	+	10390331	10390500	10386993	10387066	10391541	10391731	8	5	0	0	0	0	0	6	0	2	98	49	1	1	1	1	NA	NA	0.6	0.4
SE	7535	6-Mar	6-Mar	chr5	+	10407101	10407202	10405557	10405677	10410138	10410276	38	22	2	0	1	0	0	15	0	0	98	49	1	1	1	0.917	1	NA	1	-0.028
SE	7536	6-Mar	6-Mar	chr5	+	10426389	10426522	10423734	10423824	10429892	10430028	31	5	1	4	1	0	0	0	0	1	98	49	1	1	0.795	0.714	1	NA	0	0.836
SE	7537	SSFA2	SSFA2	chr2	+	181896029	181896079	181892614	181892660	181896907	181896964	14	27	8	1	0	0	0	5	0	0	98	49	1	1	0.875	1	1	NA	1	-0.042
SE	7538	SSFA2	SSFA2	chr2	+	181920596	181920662	181919295	181919446	181921947	181922412	12	6	10	6	7	0	0	3	0	0	98	49	1	1	0.5	0.3	1	NA	1	-0.4
SE	7542	SFXN4	SFXN4	chr10	-	119155061	119155177	119147774	119147860	119156677	119156756	3	0	0	0	0	0	0	0	0	1	98	49	1	1	1	NA	NA	NA	0	1
SE	7545	HEATR5A	HEATR5A	chr14	-	31304904	31305177	31302294	31302519	31306731	31306879	10	6	2	0	1	0	0	1	0	0	98	49	1	1	1	0.75	1	NA	1	-0.083
SE	7547	HEATR5A	HEATR5A	chr14	-	31364188	31364298	31358893	31359057	31371809	31371909	20	4	9	2	0	0	0	2	0	0	98	49	1	1	0.833	1	1	NA	1	-0.056
SE	7549	HEATR5A	HEATR5A	chr14	-	31400300	31400512	31398672	31398781	31402849	31403049	29	16	3	1	1	0	0	2	0	0	98	49	1	1	0.935	0.889	1	NA	1	-0.059
SE	7550	RFWD2	RFWD2	chr1	-	176135009	176135086	176116623	176116681	176149005	176149074	147	61	9	2	0	0	1	21	0	1	98	49	1	1	0.974	1	1	1	0.913	0.035
SE	7552	RFWD2	RFWD2	chr1	-	176149005	176149074	176135009	176135086	176175909	176176007	30	7	0	0	2	0	0	4	0	0	98	49	1	1	1	0.636	NA	NA	1	-0.182
SE	7553	RFWD2	RFWD2	chr1	-	176149005	176149074	176136487	176136547	176184632	176184692	68	13	10	1	0	0	0	6	0	0	98	49	1	1	0.971	1	1	NA	1	-0.01
SE	7554	RFWD2	RFWD2	chr1	-	176162868	176162988	176149005	176149074	176163826	176163891	120	61	15	0	2	0	3	17	0	0	98	49	1	1	1	0.938	1	1	1	-0.021
SE	7555	RFWD2	RFWD2	chr1	-	176163826	176163891	176149005	176149074	176175909	176176007	74	35	11	1	1	0	2	13	0	0	98	49	1	1	0.974	0.946	1	1	1	-0.027
SE	7559	CDC16	CDC16	chr13	+	114257077	114257230	114250548	114250674	114259334	114259398	3	0	0	0	1	0	0	2	0	2	98	49	1	1	1	0	NA	NA	0.333	0.167
SE	7573	KCNAB2	KCNAB2	chr1	+	6041830	6041872	6040516	6040645	6072754	6072798	5	5	0	3	1	0	0	1	0	0	91	49	1	1	0.473	0.729	NA	NA	1	-0.399
SE	7584	KIAA1551	KIAA1551	chr12	+	31960776	31960871	31959418	31959491	31970188	31970356	85	65	41	1	0	0	0	22	0	0	98	49	1	1	0.977	1	1	NA	1	-0.008
SE	7585	KIAA1551	KIAA1551	chr12	+	31960776	31960871	31959418	31959491	31980877	31985957	45	20	11	0	1	0	0	5	0	0	98	49	1	1	1	0.909	1	NA	1	-0.03
SE	7586	KIAA1551	KIAA1551	chr12	+	31970188	31970356	31959418	31959491	31980877	31985957	204	96	57	0	1	0	0	41	0	0	98	49	1	1	1	0.98	1	NA	1	-0.007
SE	7587	KIAA1551	KIAA1551	chr12	+	31970188	31970356	31960776	31960871	31980877	31985957	265	145	96	23	4	9	0	59	0	1	98	49	1	1	0.852	0.948	0.842	NA	0.967	-0.086
SE	7588	KIAA1551	KIAA1551	chr12	+	31980877	31985957	31970188	31970356	31987238	31987322	275	135	73	3	3	2	0	49	0	0	98	49	1	1	0.979	0.957	0.948	NA	1	-0.039
SE	7589	KIAA1551	KIAA1551	chr12	+	31980877	31985957	31970188	31970356	31992377	31993107	203	97	57	1	0	0	0	41	0	0	98	49	1	1	0.99	1	1	NA	1	-0.003
SE	7590	KIAA1551	KIAA1551	chr12	+	31987238	31987322	31970188	31970356	31992377	31993107	69	25	16	1	0	0	0	6	0	0	98	49	1	1	0.972	1	1	NA	1	-0.009
SE	7591	KIAA1551	KIAA1551	chr12	+	31987238	31987322	31980877	31985957	31992377	31993107	138	61	30	0	1	0	0	14	0	0	98	49	1	1	1	0.968	1	NA	1	-0.011
SE	7592	CSNK2A2	CSNK2A2	chr16	-	58167206	58167308	58166583	58166684	58167684	58167795	1	0	0	0	0	0	1	0	0	1	98	49	1	1	1	NA	NA	1	0	0.5
SE	7605	CPSF2	CPSF2	chr14	+	92134010	92134170	92130950	92131133	92134249	92134355	12	1	1	0	0	0	0	2	0	3	98	49	1	1	1	1	1	NA	0.25	0.75
SE	7617	NDUFA10	NDUFA10	chr2	-	240022171	240022340	240021196	240021412	240025226	240025402	4	3	3	0	1	0	0	3	0	0	98	49	1	1	1	0.6	1	NA	1	-0.133
SE	7619	DRAM2	DRAM2	chr1	-	111124741	111124881	111120515	111120693	111126226	111126294	5	1	2	1	0	0	0	6	0	0	98	49	1	1	0.714	1	1	NA	1	-0.095
SE	7626	CSDE1	CSDE1	chr1	-	114719578	114719742	114718612	114718745	114720538	114720717	55	29	5	0	1	0	0	2	0	0	98	49	1	1	1	0.935	1	NA	1	-0.022
SE	7627	CSDE1	CSDE1	chr1	-	114725220	114725333	114723882	114724002	114726210	114726386	83	40	13	3	1	0	0	22	0	0	98	49	1	1	0.933	0.952	1	NA	1	-0.038
SE	7628	CSDE1	CSDE1	chr1	-	114730257	114730422	114726982	114727090	114730507	114730648	73	35	18	0	0	0	0	21	0	1	98	49	1	1	1	1	1	NA	0.913	0.087
SE	7629	CSDE1	CSDE1	chr1	-	114730507	114730648	114726982	114727090	114732603	114732816	43	32	14	2	0	0	0	6	0	0	98	49	1	1	0.915	1	1	NA	1	-0.028
SE	7630	CSDE1	CSDE1	chr1	-	114737470	114737563	114736757	114736855	114737962	114738072	9	2	0	60	36	2	0	0	0	18	98	49	1	1	0.07	0.027	0	NA	0	0.032
SE	7632	CSDE1	CSDE1	chr1	-	114741526	114741673	114739691	114739890	114749820	114750207	2	0	0	71	15	15	0	0	2	17	98	49	1	1	0.014	0	0	0	0	0.005
SE	7633	CSDE1	CSDE1	chr1	-	114749820	114750207	114739691	114739890	114757924	114758050	71	18	15	1	0	0	2	17	0	0	98	49	1	1	0.973	1	1	1	1	-0.009
SE	7635	CNNM3	CNNM3	chr2	+	96828098	96828195	96827730	96827900	96828566	96828700	3	0	0	1	0	0	0	1	0	0	98	49	1	1	0.6	NA	NA	NA	1	-0.4
SE	7636	PTEN	PTEN	chr10	+	87925512	87925557	87894024	87894109	87931045	87931089	77	45	51	1	0	0	0	17	0	0	94	49	1	1	0.976	1	1	NA	1	-0.008
SE	7638	PTEN	PTEN	chr10	+	87952117	87952259	87933012	87933251	87957852	87958019	16	9	10	3	0	2	0	4	0	1	98	49	1	1	0.727	1	0.714	NA	0.667	0.147
SE	7654	CPSF3L	CPSF3L	chr1	-	1314300	1314365	1313731	1313921	1314823	1314962	7	2	1	0	1	0	0	1	0	0	98	49	1	1	1	0.5	1	NA	1	-0.167
SE	7663	PAPSS1	PAPSS1	chr4	-	107693770	107694006	107687038	107687177	107701170	107701285	33	4	10	1	0	0	0	3	0	0	98	49	1	1	0.943	1	1	NA	1	-0.019
SE	7664	TRAM2	TRAM2	chr6	-	52535782	52535846	52516627	52516737	52576795	52577064	1	0	0	1	0	0	0	1	0	0	98	49	1	1	0.333	NA	NA	NA	1	-0.667
SE	7671	PPP2R3C	PPP2R3C	chr14	-	35091069	35091207	35087950	35088010	35095047	35095184	13	10	4	1	1	0	0	5	0	0	98	49	1	1	0.867	0.833	1	NA	1	-0.1
SE	7672	PPP2R3C	PPP2R3C	chr14	-	35095047	35095184	35091069	35091207	35096557	35096633	9	5	1	3	0	0	0	6	0	0	98	49	1	1	0.6	1	1	NA	1	-0.133
SE	7673	PPP2R3C	PPP2R3C	chr14	-	35096708	35096764	35096557	35096633	35099251	35099384	24	17	6	1	0	0	0	6	0	0	98	49	1	1	0.923	1	1	NA	1	-0.026
SE	7674	PPP2R3C	PPP2R3C	chr14	-	35107303	35107374	35099251	35099384	35108138	35108236	33	15	8	1	0	0	0	6	0	0	98	49	1	1	0.943	1	1	NA	1	-0.019
SE	7675	PPP2R3C	PPP2R3C	chr14	-	35116609	35116737	35110524	35110629	35121901	35122542	15	6	2	0	0	0	1	4	1	0	98	49	1	1	1	1	1	0.333	1	0.334
SE	7678	FNDC3B	FNDC3B	chr3	+	172247532	172247776	172226870	172226947	172251259	172251541	35	29	22	1	1	0	0	2	0	0	98	49	1	1	0.946	0.935	1	NA	1	-0.04
SE	7679	FNDC3B	FNDC3B	chr3	+	172247532	172247776	172226870	172226947	172285925	172285984	20	6	8	3	2	0	0	1	0	1	98	49	1	1	0.769	0.6	1	NA	0.333	0.457
SE	7680	FNDC3B	FNDC3B	chr3	+	172251259	172251541	172226870	172226947	172285925	172285984	18	4	7	3	2	0	0	5	0	1	98	49	1	1	0.75	0.5	1	NA	0.714	0.036
SE	7681	FNDC3B	FNDC3B	chr3	+	172251259	172251541	172247532	172247776	172285925	172285984	33	26	21	1	0	0	0	6	0	0	98	49	1	1	0.943	1	1	NA	1	-0.019
SE	7682	FNDC3B	FNDC3B	chr3	+	172328951	172329076	172310827	172310881	172330540	172330715	44	27	6	0	1	0	0	12	0	7	98	49	1	1	1	0.931	1	NA	0.462	0.515
SE	7683	FNDC3B	FNDC3B	chr3	+	172333088	172333175	172330540	172330715	172334943	172335082	82	35	12	0	1	0	2	17	0	0	98	49	1	1	1	0.946	1	1	1	-0.018
SE	7684	FNDC3B	FNDC3B	chr3	+	172343010	172343116	172341112	172341231	172344085	172344258	16	5	6	1	1	0	0	10	0	0	98	49	1	1	0.889	0.714	1	NA	1	-0.132
SE	7685	FNDC3B	FNDC3B	chr3	+	172346326	172346440	172344085	172344258	172347211	172347361	36	16	5	0	1	0	1	6	0	0	98	49	1	1	1	0.889	1	1	1	-0.037
SE	7686	FNDC3B	FNDC3B	chr3	+	172347211	172347361	172344085	172344258	172352802	172353083	11	10	4	0	1	0	0	6	0	0	98	49	1	1	1	0.833	1	NA	1	-0.056
SE	7687	FNDC3B	FNDC3B	chr3	+	172352802	172353083	172347211	172347361	172362632	172362845	27	13	14	1	0	0	0	13	0	0	98	49	1	1	0.931	1	1	NA	1	-0.023
SE	7696	TREM1	TREM1	chr6	-	41280960	41281153	41275260	41276230	41282394	41282751	176	96	73	10	10	0	0	37	0	1	98	49	1	1	0.898	0.828	1	NA	0.949	-0.04
SE	7700	IKBIP	IKBIP	chr12	-	98634295	98634413	98613403	98614340	98644522	98645051	25	9	3	1	2	1	0	1	0	3	98	49	1	1	0.926	0.692	0.6	NA	0.143	0.596
SE	7704	TBXAS1	TBXAS1	chr7	+	139911224	139911321	139875584	139875637	139936190	139936307	93	77	31	0	2	0	2	22	0	0	98	49	1	1	1	0.951	1	1	1	-0.016
SE	7707	TBXAS1	TBXAS1	chr7	+	139955458	139955607	139953367	139953456	139957633	139957764	65	38	25	1	0	0	1	15	0	0	98	49	1	1	0.97	1	1	1	1	-0.01
SE	7708	TBXAS1	TBXAS1	chr7	+	139957633	139957764	139955458	139955607	139961918	139962233	75	29	32	0	0	0	3	22	1	0	98	49	1	1	1	1	1	0.6	1	0.2
SE	7709	TBXAS1	TBXAS1	chr7	+	140017670	140017833	140015722	140015860	140020024	140020325	18	34	7	0	2	0	0	14	0	1	98	49	1	1	1	0.895	1	NA	0.875	0.09
SE	7712	MDM4	MDM4	chr1	+	204537458	204537497	204532190	204532246	204538208	204538308	11	9	1	2	2	0	0	1	0	0	88	49	1	1	0.754	0.715	1	NA	1	-0.177
SE	7714	MDM4	MDM4	chr1	+	204542783	204542944	204538208	204538308	204546796	204546877	13	11	1	2	0	0	0	2	0	0	98	49	1	1	0.765	1	1	NA	1	-0.078
SE	7715	MDM4	MDM4	chr1	+	204544534	204544684	204542783	204542944	204546796	204546877	35	27	1	0	0	0	0	3	0	1	98	49	1	1	1	1	1	NA	0.6	0.4
SE	7717	CCDC88B	CCDC88B	chr11	+	64343996	64345157	64343777	64343914	64349330	64349458	35	12	6	1	0	0	4	4	0	0	98	49	1	1	0.946	1	1	1	1	-0.018
SE	7718	CCDC88B	CCDC88B	chr11	+	64351159	64351255	64349550	64349668	64351475	64351616	12	3	4	2	0	0	0	0	0	1	98	49	1	1	0.75	1	1	NA	0	0.917
SE	7724	IFI16	IFI16	chr1	+	159053532	159053724	159051678	159052098	159054820	159055155	48	18	17	8	3	0	0	18	0	0	98	49	1	1	0.75	0.75	1	NA	1	-0.167
SE	7749	ACAP1	ACAP1	chr17	+	7349944	7350054	7348994	7349167	7350126	7350237	21	14	12	1	0	0	5	14	0	0	98	49	1	1	0.913	1	1	1	1	-0.029
SE	7761	PLCB3	PLCB3	chr11	+	64264020	64264112	64263690	64263795	64264950	64265104	0	0	0	1	0	0	0	2	0	0	98	49	1	1	0	NA	NA	NA	1	-1
SE	7773	APH1A	APH1A	chr1	-	150267956	150267991	150267715	150267789	150268697	150269209	7	6	1	2	0	0	8	4	0	0	84	49	1	1	0.671	1	1	1	1	-0.11
SE	7775	APH1A	APH1A	chr1	-	150267956	150268127	150267715	150267789	150268697	150269209	14	6	0	2	0	0	8	7	0	0	98	49	1	1	0.778	1	NA	1	1	-0.111
SE	7779	CYLD	CYLD	chr16	+	50744729	50744866	50742761	50742841	50749575	50750202	3	3	0	15	2	0	0	0	2	1	98	49	1	1	0.091	0.429	NA	0	0	0.26
SE	7780	CYLD	CYLD	chr16	+	50776178	50776277	50754318	50754424	50777824	50777941	117	44	15	1	0	0	1	7	0	0	98	49	1	1	0.983	1	1	1	1	-0.006
SE	7783	RANBP2	RANBP2	chr2	+	108762100	108762195	108758412	108758548	108763236	108768388	2	0	0	0	2	0	0	1	0	0	98	49	1	1	1	0	NA	NA	1	-0.5
SE	7784	RANBP2	RANBP2	chr2	+	108775731	108775936	108772867	108773046	108777129	108777231	15	2	0	1	0	0	0	1	0	0	98	49	1	1	0.882	1	NA	NA	1	-0.059
SE	7786	WWP2	WWP2	chr16	+	69842023	69842120	69840125	69840263	69871803	69871931	58	20	9	0	2	0	1	4	0	0	98	49	1	1	1	0.833	1	1	1	-0.056
SE	7787	WWP2	WWP2	chr16	+	69917708	69917883	69908760	69908850	69925429	69925484	28	11	3	1	0	0	0	1	0	0	98	49	1	1	0.933	1	1	NA	1	-0.022
SE	7789	CC2D1B	CC2D1B	chr1	-	52359704	52359883	52359458	52359534	52360073	52360233	4	0	0	0	0	0	0	1	0	1	98	49	1	1	1	NA	NA	NA	0.333	0.667
SE	7794	UBE2L3	UBE2L3	chr22	+	21614556	21614665	21610856	21611043	21621514	21624034	4	3	0	0	0	2	0	1	0	0	98	49	1	1	1	1	0	NA	1	-0.333
SE	7799	SLC22A15	SLC22A15	chr1	+	116026892	116027022	116020720	116020885	116031365	116031581	9	3	1	2	0	0	0	3	0	0	98	49	1	1	0.692	1	1	NA	1	-0.103
SE	7800	GS1-124K5.11	GS1-124K5.11	chr7	-	66560578	66560675	66553467	66554837	66573384	66573550	0	1	0	5	0	0	0	1	1	0	98	49	1	1	0	1	NA	0	1	0
SE	7806	UBE4B	UBE4B	chr1	+	10106196	10106583	10105515	10105744	10117458	10117600	1	0	0	4	1	3	0	0	0	2	98	49	1	1	0.111	0	0	NA	0	0.037
SE	7807	UBE4B	UBE4B	chr1	+	10121961	10122076	10119512	10119613	10126793	10126877	17	7	4	0	1	0	1	9	0	0	98	49	1	1	1	0.778	1	1	1	-0.074
SE	7808	UBE4B	UBE4B	chr1	+	10130499	10130616	10129391	10129448	10130714	10130813	24	10	4	1	0	0	0	2	0	0	98	49	1	1	0.923	1	1	NA	1	-0.026
SE	7809	UBE4B	UBE4B	chr1	+	10137066	10137205	10134987	10135186	10144939	10145039	20	7	2	1	0	0	0	7	0	0	98	49	1	1	0.909	1	1	NA	1	-0.03
SE	7810	UBE4B	UBE4B	chr1	+	10158355	10158482	10151325	10151561	10161141	10161286	24	3	3	0	0	0	0	0	0	1	98	49	1	1	1	1	1	NA	0	1
SE	7811	NDUFS2	NDUFS2	chr1	+	161210110	161210188	161209856	161209931	161210303	161210389	22	1	4	0	1	0	3	9	0	0	98	49	1	1	1	0.333	1	1	1	-0.222
SE	7818	SELL	SELL	chr1	-	169696534	169696553	169690664	169691802	169701559	169701688	275	185	116	1	2	0	0	82	0	0	68	49	1	1	0.995	0.985	1	NA	1	-0.007
SE	7819	SELL	SELL	chr1	-	169696534	169696553	169690664	169691802	169703254	169703440	38	26	7	1	0	0	1	13	0	0	68	49	1	1	0.965	1	1	1	1	-0.012
SE	7820	SELL	SELL	chr1	-	169701559	169701688	169690664	169691802	169703254	169703440	124	55	47	1	0	0	2	26	0	0	98	49	1	1	0.984	1	1	1	1	-0.005
SE	7821	SELL	SELL	chr1	-	169701559	169701688	169696534	169696553	169703254	169703440	366	212	157	5	0	0	2	95	1	0	98	49	1	1	0.973	1	1	0.5	1	0.241
SE	7823	CD58	CD58	chr1	-	116519230	116519267	116514533	116514822	116521905	116521983	20	16	3	2	0	0	0	1	0	0	86	49	1	1	0.851	1	1	NA	1	-0.05
SE	7826	ZNF687	ZNF687	chr1	+	151288527	151288706	151286274	151288406	151289094	151289271	7	0	4	0	0	2	0	1	0	0	98	49	1	1	1	NA	0.5	NA	1	-0.25
SE	7828	ZNF687	ZNF687	chr1	+	151289094	151289271	151286274	151288406	151289377	151289540	6	0	2	1	0	0	0	2	0	0	98	49	1	1	0.75	NA	1	NA	1	-0.125
SE	7829	ZNF687	ZNF687	chr1	+	151289094	151289271	151288527	151288706	151289377	151289540	9	0	0	0	1	1	0	3	0	0	98	49	1	1	1	0	0	NA	1	-0.667
SE	7834	CD44	CD44	chr11	+	35209964	35210054	35208104	35208206	35211245	35211449	4	1	0	1	0	0	0	4	0	0	98	49	1	1	0.667	1	NA	NA	1	-0.166
SE	7835	CD44	CD44	chr11	+	35211245	35211449	35189834	35190065	35214851	35214914	5	0	0	20	3	6	0	1	0	3	98	49	1	1	0.111	0	0	NA	0.143	-0.106
SE	7836	CD44	CD44	chr11	+	35211245	35211449	35209964	35210054	35214851	35214914	6	1	0	2	0	0	0	1	0	0	98	49	1	1	0.6	1	NA	NA	1	-0.2
SE	7841	LRRK2	LRRK2	chr12	+	40240482	40240617	40237968	40238103	40243549	40243681	732	253	82	0	1	0	5	60	0	0	98	49	1	1	1	0.992	1	1	1	-0.003
SE	7843	LRRK2	LRRK2	chr12	+	40257247	40257377	40252909	40253016	40259479	40259604	496	207	63	5	0	0	7	46	0	0	98	49	1	1	0.98	1	1	1	1	-0.007
SE	7844	LRRK2	LRRK2	chr12	+	40257247	40257377	40252909	40253016	40263788	40263901	175	69	34	2	0	0	2	19	0	1	98	49	1	1	0.978	1	1	1	0.905	0.04
SE	7845	LRRK2	LRRK2	chr12	+	40259479	40259604	40252909	40253016	40263788	40263901	372	93	25	2	0	0	2	27	0	1	98	49	1	1	0.989	1	1	1	0.931	0.031
SE	7846	LRRK2	LRRK2	chr12	+	40259479	40259604	40257247	40257377	40263788	40263901	689	231	54	1	0	0	7	54	0	0	98	49	1	1	0.997	1	1	1	1	-0.001
SE	7847	LRRK2	LRRK2	chr12	+	40278090	40278261	40277887	40278016	40283874	40284133	367	182	77	0	1	0	0	39	0	0	98	49	1	1	1	0.989	1	NA	1	-0.004
SE	7848	LRRK2	LRRK2	chr12	+	40283874	40284133	40278090	40278261	40287350	40287539	445	186	61	3	2	0	0	46	0	0	98	49	1	1	0.987	0.979	1	NA	1	-0.011
SE	7850	LRRK2	LRRK2	chr12	+	40295426	40295644	40294844	40294914	40298242	40298493	504	133	63	0	0	1	8	74	0	0	98	49	1	1	1	1	0.969	1	1	-0.01
SE	7852	LRRK2	LRRK2	chr12	+	40298242	40298493	40295426	40295644	40302788	40302882	192	59	33	2	2	0	0	32	0	1	98	49	1	1	0.98	0.937	1	NA	0.941	0.031
SE	7853	LRRK2	LRRK2	chr12	+	40299108	40299257	40295426	40295644	40302788	40302882	390	124	39	2	2	0	2	64	0	1	98	49	1	1	0.99	0.969	1	1	0.97	0.001
SE	7854	LRRK2	LRRK2	chr12	+	40299108	40299257	40298242	40298493	40302788	40302882	598	167	57	0	3	0	5	93	0	0	98	49	1	1	1	0.965	1	1	1	-0.012
SE	7855	LRRK2	LRRK2	chr12	+	40303947	40304134	40302788	40302882	40305784	40305966	405	135	63	1	0	1	2	47	0	0	98	49	1	1	0.995	1	0.969	1	1	-0.012
SE	7856	LRRK2	LRRK2	chr12	+	40303947	40304134	40302788	40302882	40308466	40308696	208	58	33	1	0	0	1	29	0	0	98	49	1	1	0.99	1	1	1	1	-0.003
SE	7857	LRRK2	LRRK2	chr12	+	40305784	40305966	40302788	40302882	40308466	40308696	91	86	28	1	0	0	1	14	0	0	98	49	1	1	0.978	1	1	1	1	-0.007
SE	7859	LRRK2	LRRK2	chr12	+	40308466	40308696	40305784	40305966	40309105	40309233	365	203	48	3	0	0	1	48	0	0	98	49	1	1	0.984	1	1	1	1	-0.005
SE	7860	LRRK2	LRRK2	chr12	+	40310430	40310649	40309105	40309233	40315211	40315300	161	70	29	3	0	0	0	24	0	0	98	49	1	1	0.964	1	1	NA	1	-0.012
SE	7864	LRRK2	LRRK2	chr12	+	40322318	40322510	40321033	40321188	40323159	40323306	237	64	26	0	0	1	6	37	0	0	98	49	1	1	1	1	0.929	1	1	-0.024
SE	7865	LRRK2	LRRK2	chr12	+	40340293	40340454	40334966	40335157	40346752	40346923	352	103	38	3	0	1	5	39	0	0	98	49	1	1	0.983	1	0.95	1	1	-0.022
SE	7866	LRRK2	LRRK2	chr12	+	40346752	40346923	40334966	40335157	40348408	40348509	146	58	53	1	0	0	1	17	0	0	98	49	1	1	0.986	1	1	1	1	-0.005
SE	7867	LRRK2	LRRK2	chr12	+	40348408	40348509	40346752	40346923	40351538	40351733	306	120	73	2	1	0	4	30	0	0	98	49	1	1	0.987	0.984	1	1	1	-0.01
SE	7868	LRRK2	LRRK2	chr12	+	40351538	40351733	40348408	40348509	40354298	40354492	244	99	31	1	0	0	3	25	0	0	98	49	1	1	0.992	1	1	1	1	-0.003
SE	7870	LRRK2	LRRK2	chr12	+	40367005	40367077	40364841	40365050	40367643	40369284	176	97	43	15	4	0	0	13	0	3	98	49	1	1	0.854	0.924	1	NA	0.684	0.242
SE	7875	PAK1	PAK1	chr11	-	77332729	77332867	77322014	77323360	77336085	77336282	68	38	15	0	0	0	2	22	1	0	98	49	1	1	1	1	1	0.5	1	0.25
SE	7876	PAK1	PAK1	chr11	-	77337323	77337423	77336085	77336282	77340645	77340763	47	32	22	1	1	0	0	12	0	0	98	49	1	1	0.959	0.941	1	NA	1	-0.033
SE	7877	PAK1	PAK1	chr11	-	77379240	77379388	77374327	77374365	77379893	77379994	55	23	22	0	1	0	0	15	0	0	98	49	1	1	1	0.92	1	NA	1	-0.027
SE	7888	BIRC2	BIRC2	chr11	+	102350843	102350943	102348597	102350749	102362895	102362974	17	8	4	1	0	0	0	4	0	0	98	49	1	1	0.895	1	1	NA	1	-0.035
SE	7890	CUBN	CUBN	chr10	-	16836234	16836382	16835013	16835195	16840329	16840535	0	0	1	0	0	0	0	0	0	1	98	49	1	1	NA	NA	1	NA	0	1
SE	7904	FOXJ3	FOXJ3	chr1	-	42199101	42199230	42194889	42195064	42205761	42205863	9	0	0	0	0	0	0	0	0	1	98	49	1	1	1	NA	NA	NA	0	1

SE	7906 GLG1	GLG1	chr16	-	74468945	74469063	74467755	74467848	74469984	74470073	20	1	4	1	0	0	0	5	0	0	98	49	1	1	0.909	1	1	NA		1	-0.03
SE	7918 EPB41	EPB41	chr1	+	28989355	28989435	28987430	28987905	28993329	28993542	3	3	0	45	17	5	0	0	0	2	98	49	1	1	0.032	0.081	0	NA		0	0.038
SE	7919 EPB41	EPB41	chr1	+	28993329	28993542	28987430	28987905	28997214	28997319	90	37	10	0	1	0	0	3	0	0	98	49	1	1	1	0.949	1	NA		1	-0.017
SE	7920 EPB41	EPB41	chr1	+	28993329	28993542	28987430	28987905	29011864	29011907	46	19	5	6	2	0	0	2	0	0	98	49	1	1	0.793	0.826	1	NA		1	-0.127
SE	7922 EPB41	EPB41	chr1	+	28997214	28997319	28993329	28993542	29011864	29011907	72	26	12	1	2	0	0	1	0	0	98	49	1	1	0.973	0.867	1	NA		1	-0.053
SE	7924 EPB41	EPB41	chr1	+	29058588	29058645	29053103	29053312	29064981	29065158	0	2	0	21	11	11	0	1	2	10	98	49	1	1	0	0.083	0		0	0.048	0.004
SE	7928 EPB41	EPB41	chr1	+	29097806	29097935	29064981	29065158	29109335	29109437	64	23	19	2	0	0	0	16	0	0	98	49	1	1	0.941	1	1	NA		1	-0.02
SE	7944 DHRS7	DHRS7	chr14	-	60150064	60150187	60149352	60149568	60152938	60153178	70	52	39	2	0	0	2	13	0	0	98	49	1	1	0.946	1	1		1	1	-0.018
SE	7946 CUL4A	CUL4A	chr13	+	113239432	113239551	113236822	113236890	113242967	113243160	7	2	0	0	0	0	0	5	0	1	98	49	1	1	1	1	NA	NA		0.714	0.286
SE	7951 RSRC2	RSRC2	chr12	-	122520503	122520581	122518838	122519029	122521384	122521428	3	2	0	3	0	0	1	3	0	1	98	49	1	1	0.333	1	NA	NA	1	0.6	-0.134
SE	7961 TNRC6B	TNRC6B	chr22	+	40270121	40270280	40264687	40267036	40273424	40273600	7	5	2	5	4	0	0	1	0	0	98	49	1	1	0.412	0.385	1	NA		1	-0.401
SE	7971 LCOR	LCOR	chr10	+	96833405	96833479	96832259	96832399	96907264	96907330	10	1	0	2	0	0	1	1	0	0	98	49	1	1	0.714	1	NA		1	1	-0.143
SE	7975 KHDRBS1	KHDRBS1	chr1	+	32031523	32031640	32030297	32030422	32033187	32033334	8	3	0	3	1	0	0	4	0	0	98	49	1	1	0.571	0.6	NA	NA		1	-0.415
SE	7977 TXLNA	TXLNA	chr1	+	32184524	32184616	32181241	32181577	32187953	32188124	2	0	0	0	0	0	0	1	0	1	98	49	1	1	1	NA	NA	NA		0.333	0.667
SE	7978 MAN2A1	MAN2A1	chr5	+	109729341	109729513	109716119	109716264	109755328	109755456	17	2	0	1	1	0	0	3	0	0	98	49	1	1	0.895	0.5	NA	NA		1	-0.302
SE	7979 MAN2A1	MAN2A1	chr5	+	109817272	109817438	109789459	109789527	109819668	109819887	7	1	1	2	0	0	0	3	0	0	98	49	1	1	0.636	1	1	NA		1	-0.121
SE	7980 MAN2A1	MAN2A1	chr5	+	109820219	109820342	109819668	109819887	109823722	109823837	10	7	0	1	0	0	0	5	0	0	98	49	1	1	0.833	1	NA	NA		1	-0.084
SE	7981 MAN2A1	MAN2A1	chr5	+	109845864	109846006	109842327	109842461	109847656	109847790	3	2	0	0	3	0	0	4	0	0	98	49	1	1	1	0.25	NA	NA		1	-0.375
SE	7984 PTPN18	PTPN18	chr2	+	130372872	130372947	130372256	130372483	130373156	130375409	0	0	0	1	0	0	0	2	0	0	98	49	1	1	0	NA	NA	NA		1	-1
SE	7985 PAFAH1B2	PAFAH1B2	chr11	+	117152440	117152528	117144283	117144418	117159933	117160023	20	23	3	2	1	0	0	4	0	0	98	49	1	1	0.833	0.92	1	NA		1	-0.082
SE	7987 PAFAH1B2	PAFAH1B2	chr11	+	117161144	117161261	117159933	117160023	117163769	117163874	32	32	6	0	0	0	0	6	0	2	98	49	1	1	1	1	1	NA		0.6	0.4
SE	7998 PMF1	PMF1	chr1	+	156225556	156225668	156212987	156213176	156232319	156232425	3	1	0	3	0	0	0	0	0	1	98	49	1	1	0.333	1	NA	NA		0	0.666
SE	8001 USF1	USF1	chr1	-	161041650	161041846	161041323	161041411	161042115	161042217	24	9	4	2	1	0	0	5	0	0	98	49	1	1	0.857	0.818	1	NA		1	-0.108
SE	8006 ANO10	ANO10	chr3	-	43565652	43565727	43561219	43561402	43574808	43574864	12	6	2	1	0	0	2	1	0	0	98	49	1	1	0.857	1	1		1	1	-0.048
SE	8009 ANO10	ANO10	chr3	-	43600383	43600581	43598531	43598666	43605713	43605863	5	9	4	0	2	0	0	1	0	0	98	49	1	1	1	0.692	1	NA		1	-0.103
SE	8014 RNF19B	RNF19B	chr1	-	32938396	32938528	32936445	32937259	32942251	32942459	79	34	18	1	0	0	1	11	0	0	98	49	1	1	0.975	1	1		1	1	-0.008
SE	8015 RNF19B	RNF19B	chr1	-	32942251	32942459	32938396	32938528	32944018	32944159	91	49	45	1	1	0	2	25	0	0	98	49	1	1	0.978	0.961	1		1	1	-0.02
SE	8016 RNF19B	RNF19B	chr1	-	32945513	32945628	32944018	32944159	32946401	32946564	110	64	21	1	0	0	1	24	0	0	98	49	1	1	0.982	1	1		1	1	-0.006
SE	8017 RNF19B	RNF19B	chr1	-	32946401	32946564	32945513	32945628	32948221	32948363	124	54	35	1	0	0	1	19	0	0	98	49	1	1	0.984	1	1		1	1	-0.005
SE	8019 TBC1D9B	TBC1D9B	chr5	-	179867444	179867495	179865837	179865888	179867777	179867849	0	1	2	3	0	0	0	0	0	4	98	49	1	1	0	1	1	NA		0	0.667
SE	8020 TBC1D9B	TBC1D9B	chr5	-	179875919	179876037	179874901	179875187	179878308	179878523	8	2	0	0	0	0	0	3	0	1	98	49	1	1	1	1	NA	NA		0.6	0.4
SE	8021 SLC35D1	SLC35D1	chr1	-	67049781	67049850	67047264	67047367	67050432	67050504	1	2	0	0	0	0	0	2	0	1	98	49	1	1	1	1	NA	NA		0.5	0.5
SE	8037 ARHGEF7	ARHGEF7	chr13	+	111205288	111205373	111153713	111153991	111209871	111210002	3	3	0	1	0	0	2	1	0	0	98	49	1	1	0.6	1	NA	NA	1	1	-0.2
SE	8038 ARHGEF7	ARHGEF7	chr13	+	111209871	111210002	111205288	111205373	111217678	111217880	5	2	1	1	0	0	1	0	0	0	98	49	1	1	0.714	1	1	NA		1	-0.095
SE	8041 SRCAP	SRCAP	chr16	+	30712678	30712815	30712261	30712439	30713207	30713377	6	3	1	0	0	0	1	5	0	1	98	49	1	1	1	1	1		1	0.714	0.143
SE	8043 AK2	AK2	chr1	-	33031543	33031661	33024441	33024567	33036735	33036911	1	0	0	4	1	0	0	1	0	0	98	49	1	1	0.111	0	NA	NA		1	-0.945
SE	8050 ADAM8	ADAM8	chr10	-	133268747	133268862	133267928	133268118	133269444	133269529	134	56	50	1	0	0	2	19	0	0	98	49	1	1	0.985	1	1		1	1	-0.005
SE	8052 ADAM8	ADAM8	chr10	-	133269896	133269974	133269444	133269529	133270359	133270510	208	103	61	2	0	0	7	30	0	0	98	49	1	1	0.981	1	1		1	1	-0.006
SE	8053 ADAM8	ADAM8	chr10	-	133270880	133271070	133270735	133270805	133271199	133271289	192	136	64	0	1	0	20	40	0	0	98	49	1	1	1	0.986	1		1	1	-0.005
SE	8054 ADAM8	ADAM8	chr10	-	133274158	133274235	133273950	133274029	133275483	133275587	116	70	41	1	0	0	14	29	0	0	98	49	1	1	0.983	1	1		1	1	-0.006
SE	8066 EIF5	EIF5	chr14	+	103336035	103336117	103335652	103335932	103336676	103336849	48	27	14	1	2	0	0	7	0	1	98	49	1	1	0.96	0.871	1	NA		0.778	0.166
SE	8067 EIF5	EIF5	chr14	+	103339638	103339803	103339171	103339333	103340426	103340561	98	50	5	0	1	0	1	11	0	0	98	49	1	1	1	0.962	1		1	1	-0.013
SE	8068 TALDO1	TALDO1	chr11	+	758949	759057	755878	756002	760121	760253	161	136	132	0	1	0	1	25	0	0	98	49	1	1	1	0.986	1		1	1	-0.005
SE	8069 TALDO1	TALDO1	chr11	+	763343	763519	760121	760253	763746	763944	219	205	155	2	0	0	5	46	0	0	98	49	1	1	0.982	1	1		1	1	-0.006
SE	8088 HPS1	HPS1	chr10	-	98429790	98429889	98429145	98429642	98430570	98430670	14	1	2	0	0	3	0	1	0	1	98	49	1	1	1	1	0.25	NA		0.333	0.417
SE	8089 HPS1	HPS1	chr10	-	98431130	98431291	98430570	98430670	98433982	98434091	15	0	3	2	0	0	0	6	0	0	98	49	1	1	0.789	NA	1	NA		1	-0.105
SE	8090 HPS1	HPS1	chr10	-	98435271	98435414	98433982	98434091	98435634	98435772	15	1	2	7	0	0	0	7	0	0	98	49	1	1	0.517	1	1	NA		1	-0.161
SE	8091 PANK2	PANK2	chr20	+	3912457	3912634	3910576	3910830	3916926	3917050	27	8	9	1	2	0	0	6	0	0	98	49	1	1	0.931	0.667	1	NA		1	-0.134
SE	8092 PANK2	PANK2	chr20	+	3918670	3918796	3916926	3917050	3923243	3923855	10	4	2	0	3	0	0	2	0	0	98	49	1	1	1	0.4	1	NA		1	-0.2
SE	8096 PARGP1	PARGP1	chr10	-	45939360	45939447	45916078	45916171	45946836	45946911	0	0	0	1	0	0	0	2	0	0	98	49	1	1	0	NA	NA	NA		1	-1
SE	8097 TMEM62	TMEM62	chr15	+	43178606	43178711	43169592	43169677	43181180	43181299	1	0	0	0	0	0	0	0	0	1	98	49	1	1	1	NA	NA	NA		0	1
SE	8100 DEDD2	DEDD2	chr19	-	42204461	42204559	42198592	42199829	42209699	42209840	0	0	0	16	25	11	1	0	3	5	98	49	1	1	0	0	0		0.143	0	-0.071
SE	8101 DEDD2	DEDD2	chr19	-	42209699	42209840	42198592	42199829	42215132	42215252	32	36	19	2	0	0	3	17	0	0	98	49	1	1	0.889	1	1		1	1	-0.037
SE	8105 TAOK1	TAOK1	chr17	+	29498317	29498521	29495559	29495727	29502588	29502723	33	15	3	0	1	0	0	5	0	0	98	49	1	1	1	0.882	1	NA		1	-0.039
SE	8114 FCGR3B	FCGR3B	chr1	-	161626144	161626402	161623197	161624639	161629777	161630032	212	147	132	6	3	3	0	59	0	1	98	49	1	1	0.946	0.961	0.957	NA		0.967	-0.012
SE	8115 TMEM183A	TMEM183A	chr1	+	203007773	203007863	203007405	203007574	203008642	203008810	4	0	0	0	0	0	0	0	1	0	98	49	1	1	1	NA	NA		0	NA	1
SE	8121 U2SURP	U2SURP	chr3	+	143023974	143024018	143022852	143023064	143027148	143027253	10	2	0	0	2	0	0	1	0	0	93	49	1	1	1	0.345	NA	NA		1	-0.328
SE	8122 U2SURP	U2SURP	chr3	+	143054942	143055119	143053675	143053794	143056311	143060725	12	3	1	1	0	0	0	1	0	0	98	49	1	1	0.857	1	1	NA		1	-0.048
SE	8123 ADD1	ADD1	chr4	+	2909338	2909431	2908514	2908604	2914883	2915040	2	0	0	10	2	5	0	0	0	2	98	49	1	1	0.091	0	0	NA		0	0.03
SE	8124 ADD1	ADD1	chr4	+	2926641	2926675	2926013	2926112	2928170	2930075	1	0	0	21	8	3	0	0	2	3	83	49	1	1	0.027	0	0		0	0	0.009
SE	8125 RCOR1	RCOR1	chr14	+	102714422	102714617	102710934	102711013	102721006	102721084	33	18	7	2	0	0	0	5	0	0	98	49	1	1	0.892	1	1	NA		1	-0.036
SE	8126 RCOR1	RCOR1	chr14	+	102721319	102721377	102721006	102721084	102722186	102722416	11	10	4	0	0	0	0	7	0	2	98	49	1	1	1	1	1	NA		0.636	0.364
SE	8130 CNEP1R1	CNEP1R1	chr16	+	50029724	50029798	50026395	50026467	50033396	50033506	13	7	4	0	3	0	0	6	0	0	98	49	1	1	1	0.538	1	NA		1	-0.154
SE	8133 ATP2B4	ATP2B4	chr1	+	203707008	203707223	203703651	203703813	203707861	203708104	15	12	1	1	0	0	0	2	0	0	98	49	1	1	0.882	1	1	NA		1	-0.039
SE	8134 ATP2B4	ATP2B4	chr1	+	203721196	203721410	203720548	203720740	203722477	203722689	16	7	4	2	0	0	0	2	0	0	98	49	1	1	0.8	1	1	NA		1	-0.067
SE	8136 FAM160B1	FAM160B1	chr10	+	114848646	114848737	114847089	114847233	114855196	114855340	22	10	1	1	0	0	0	3	0	0	98	49	1	1	0.917	1	1	NA		1	-0.028
SE	8137 GNL2	GNL2	chr1	-	37574664	37574823	37574342	37574456	37575594	37575699	5	0	0	0	0	0	0	1	0	1	98	49	1	1	1	NA	NA	NA		0.333	0.667
SE	8143 CD48	CD48	chr1	-	160684886	160685189	160680421	160681468	160711681	160711851	82	7	6	0	3	0	0	22	0	1	98	49	1	1	1	0.538	1	NA		0.917	-0.071
SE	8149 AKIRIN1	AKIRIN1	chr1	+	39000971	39001106	38998170	38998311	39003346	39003418	8	8	6	1	0	0	0	1	0	0	98	49	1	1	0.8	1	1	NA		1	-0.067
SE	8150 TFEC	TFEC	chr7	-	115974169	115974256	115956678	115956793	115984261	115984513	2	0	2	1	0	0	0	2	0	0	98	49	1	1	0.5	NA	1	NA		1	-0.25
SE	8153 CAP1	CAP1	chr1	+	40064226	40064370	40061734	40061812	40064473	40064559	175	135	62	1	0	0	2	29	0	0	98	49	1	1	0.989	1	1		1	1	-0.004
SE	8157 RBPJ	RBPJ	chr4	+	26420550	26420725	26415474	26415640	26424341	26424479	41	15	3	0	1	0	0	8	0	0	98	49	1	1	1	0.882	1	NA		1	-0.039
SE	8158 RBPJ	RBPJ	chr4	+	26429897	26430053	26428719	26428860	26430418	26430522	44	13	7	2	0	0	0	3	0	0	98	49	1	1	0.917	1	1	NA		1	-0.028
SE	8160 ACAA1	ACAA1	chr3	-	38126161	38126341	38125825	38125881	38126509	38126700	5	3	5	2	0	0	0	1	0	0	98	49	1	1	0.556	1	1	NA		1	-0.148
SE	8161 ACAA1	ACAA1	chr3	-	38129289	38129388	38127785	38127866	38131595	38131638	17	5	2	0	0	3	0	2	0	0	98	49	1	1	1	1	0.25	NA		1	-0.25
SE	8163 MAP3K11	MAP3K11	chr11	-	65607921	65608070	65607640	65607816	65608267	65608448	36	19	21	1	0	0	1	13	0	0	98	49	1	1	0.947	1	1		1	1	-0.018
SE	8164 MAP3K11	MAP3K11	chr11	-	65608267	65608448	65607921	65608070	65613017	65614249	44	27	21	1	0	0	1	10	0	0	98	49	1	1	0.957	1	1		1	1	-0.014
SE	8166 ATP5C1	ATP5C1	chr10	+	7802757	7802854	7802269	7802425	7806973	7807010	26	7	3	0	1	0	0	6	0	1	98	49	1	1	1	0.778	1	NA		0.75	0.176
SE	8168 PECAM1	PECAM1	chr17	-	64329699	64329722	64319415	64323845	64341633	64341690	158	84	42	1	0	0	1	23	0	0	72	49	1	1	0.991	1	1		1	1	-0.003
SE	8169 PECAM1	PECAM1	chr17	-	64341633	64341690	64329699	64329722	64348259	64348322	124	89	59	0	0	1	0	17	0	0	98	49	1	1	1	1	0.967	NA		1	-0.011
SE	8170 PECAM1	PECAM1	chr17	-	64348259	64348322	64341633	64341690	64350379	64350433	62	64	67	0	5	0	0	15	0	0	98	49	1	1	1	0.865	1	NA		1	-0.045
SE	8172 PECAM1	PECAM1	chr17	-	64356110	64356398	64353490	64353518	64360139	64360415	127	62	42	0	2	0	1	25	0	0	98	49	1	1	1	0.939	1		1	1	-0.02
SE	8173 PECAM1	PECAM1	chr17	-	64360139	64360415	64356110	64356398	64363148	64363397	212	112	75	1	2	0	2	31	0	0	98	49	1	1	0.991	0.966	1		1	1	-0.014
SE	8175 NRIP1	NRIP1	chr21	-	15014343	15014466	14961234	14968526	15043494	15043574	3	1	0	1	0	0	0	1	0	0	98	49	1	1	0.6	1	NA	NA		1	-0.2
SE	8187 TMEM165	TMEM165	chr4	+	55403235	55403338	55395912	55396396	55411613	55411839	1	0	0	3	0	1	0	0	0	1	98	49	1	1	0.143	NA	0	NA		0	0.071
SE	8198 DCLRE1C	DCLRE1C	chr10	-	14947524	14947609	14945104	14945189	14949035	14949087	3	0	0	0	2	2	0	1	0	3	98	49	1	1	1	0	0	NA		0.143	0.19
SE	8208 SULF2	SULF2	chr20	-	47659398	47659452	47657405	47658392	47659696	47659721	48	27	15	1	0	4	0	9	0	0	98	49	1	1	0.96	1	0.652	NA		1	-0.129
SE	8209 SULF2	SULF2	chr20	-	47666259	47666488	47665856	47665953	47672197	47672393	28	16	11	0	0	0	1	5	0	1	98	49	1	1	1	1	1		1	0.714	0.143
SE	8210 SULF2	SULF2	chr20	-	47702518	47702670	47690125	47690295	47736702	47736942	13	11	5	1	0	0	1	3	0	1	98	49	1	1	0.867	1	1		1	0.6	0.156
SE	8211 SULF2	SULF2	chr20	-	47736702	47736942	47702518	47702670	47757188	47757463	38	13	6	1	0	0	7	14	0	0	98	49	1	1	0.95	1	1		1	1	-0.017
SE	8213 EFCAB14	EFCAB14	chr1	-	46688352	46688544	46686783	46686870	46689586	46689691	1	0	0	5	1	3	0	0	1	0	98	49	1	1	0.091	0	0		0	NA	0.03
SE	8214 EFCAB14	EFCAB14	chr1	-	46716294	46716443	46707905	46708051	46717902	46719064	15	2	4	1	0	0	0	5	0	0	98	49	1	1	0.882	1	1	NA		1	-0.039
SE	8216 ACADVL	ACADVL	chr17	+	7220923	7221058	7220765	7220830	7221537	7221682	59	17	4	0	0	0	0	10	0	1	98	49	1	1	1	1	1	NA		0.833	0.167
SE	8217 ACADVL	ACADVL	chr17	+	7224145	7224243	7223967	7224069	7224320	7224393	34	16	6	5	2	0	0	5	0	0	98	49	1	1	0.773	0.8	1	NA		1	-0.142
SE	8220 HELQ	HELQ	chr4	-	83446834	83447035	83446013	83446086	83448782	83448961	2	2	0	0	0	0	0	0	0	1	98	49	1	1	1	1	NA	NA		0	1
SE	8222 EFHD2	EFHD2	chr1	+	15427149	15427284	15425870	15426018	15428592	15430343	150	117	81	1	0	0	8	39	0	0	98	49	1	1	0.987	1	1		1	1	-0.004
SE	8228 SYNRG	SYNRG	chr17	-	37529782	37529851	37520537	37520648	37535978	37536127	1	0	0	5	0	0	0	0	0	2	98	49	1	1	0.091	NA	NA	NA		0	0.091
SE	8234 ELOVL1	ELOVL1	chr1	-	43365185	43365376	43364927	43365008	43365563	43365623	12	4	1	4	2	0	0	1	0	0	98	49	1	1	0.6	0.5	1	NA		1	-0.3
SE	8235 ZFC3H1	ZFC3H1	chr12	-	71619314	71619409	71615205	71615316	71619925	71620124	12	2	3	1	0	0	0	2	0	0	98	49	1	1	0.857	1	1	NA		1	-0.048
SE	8236 ZFC3H1	ZFC3H1	chr12	-	71624103	71624292	71623359	71623597	71626259	71626446	8	0	1	0	0	0	0	0	0	2	98	49	1	1	1	NA	1	NA		0	1
SE	8237 ZFC3H1	ZFC3H1	chr12	-	71636849	71637059	71636489	71636654	71638417	71638515	27	17	1	0	0	0	0	3	0	1	98	49	1	1	1	1	1	NA		0.6	0.4
SE	8247 SNX29P2	SNX29P2	chr16	+	29320927	29321095	29312349	29312589	29326346	29326483	1	2	0	0	4	0	1	0	0	0	98	49	1	1	1	0.2	NA		1	NA	-0.4
SE	8249 EWSR1	EWSR1	chr22	+	29282389	29282557	29278029	29278216	29286922	29287134	37	8	16	0	0	0	0	11	0	1	98	49	1	1	1	1	1	NA		0.846	0.154
SE	8250 PPT1	PPT1	chr1	-	40076841	40076913	40072709	40074183	40078559	40078658	75	38	21	6	1	2	0	9	0	5	98	49	1	1	0.862	0.95	0.84	NA		0.474	0.41
SE	8254 PKN2	PKN2	chr1	+	88771420	88771566	88770351	88770469	88771662	88771879	101	33	9	1	2	0	0	19	0	0	98	49	1	1	0.981	0.892	1	NA		1	-0.042
SE	8255 PKN2	PKN2	chr1	+	88804390	88804534	88786103	88786213	88804845	88804921	26	14	0	11	6	7	0	4	0	0	98	49	1	1	0.542	0.538	0	NA		1	-0.64
SE	8262 NLRP3	NLRP3	chr1	+	247429584	247429755	247423846	247425599	247434102	247434273	9	0	0	1	0	0	0	5	0	0	98	49	1	1	0.818	NA	NA	NA		1	-0.182
SE	8263 NLRP3	NLRP3	chr1	+	247434102	247434273	247429584	247429755	247443971	247444142	3	0	0	1	0	0	0	3	0	0	98	49	1	1	0.6	NA	NA	NA		1	-0.4
SE	8264 NLRP3	NLRP3	chr1	+	247435969	247436140	247434102	247434273	247443971	247444142	6	2	2	1	0	0	0	1	0	2	98	49	1	1	0.75	1	1	NA		0.2	0.717
SE	8266 COL4A3BP	COL4A3BP	chr5	-	75459064	75459181	75426370	75426478	75505981	75506116	9	2	2	2	0	0	0	2	0	0	98	49	1	1	0.692	1	1	NA		1	-0.103
SE	8282 MLLT10	MLLT10	chr10	+	21617111	21617207	21614830	21614924	21651672	21651768	6	1	0	1	0	0	0	1	0	0	98	49	1	1	0.75	1	NA	NA		1	-0.125
SE	8285 PLK3	PLK3	chr1	+	44803930	44804024	44803599	44803691	44804338	44804501	10	0	2	2	0	0	0	2	0	0	98	49	1	1	0.714	NA	1	NA		1	-0.143
SE	8286 PLK3	PLK3	chr1	+	44804162	44804246	44803930	44804024	44804338	44804501	36	14	8	1	0	0	6	3	0	0	98	49	1	1	0.947	1	1		1	1	-0.018
SE	8287 PLK3	PLK3	chr1	+	44804649	44804779	44804338	44804501	44805265	44805379	9	6	1	20	9	2	0	2	0	3	98	49	1	1	0.184	0.25	0.2	NA		0.25	-0.039
SE	8294 TMED5	TMED5	chr1	-	93158814	93158885	93156299	93156483	93160128	93160226	3	0	0	8	0	0	0	1	0	0	98	49	1	1	0.158	NA	NA	NA		1	-0.842
SE	8298 ZNF821	ZNF821	chr16	-	71861775	71861942	71859679	71860672	71864137	71864242	1	0	0	0	0	0	0	0	0	1	98	49	1	1	1	NA	NA	NA		0	1
SE	8301 IL10RA	IL10RA	chr11	+	117986683	117986813	117986390	117986534	117988381	117988502	4	2	2	1	2	1	0	0	0	2	98	49	1	1	0.667	0.333	0.5	NA		0	0.5
SE	8302 IL10RA	IL10RA	chr11	+	117988381	117988502	117986390	117986534	117993240	117993410	1	2	1	0	0	0	0	3	0	1	98	49	1	1	1	1	1	NA		0.6	0.4
SE	8303 IL10RA	IL10RA	chr11	+	117989441	117989620	117988381	117988502	117993240	117993410	18	0	0	0	0	0	2	14	0	1	98	49	1	1	1	1	NA	NA	1	0.875	0.062
SE	8310 ZBTB7B	ZBTB7B	chr1	+	155014014	155014093	155010915	155011015	155014654	155015814	0	4	1	0	1	0	0	1	0	0	98	49	1	1	NA	0.667	1	NA		1	-0.166
SE	8314 RNF10	RNF10	chr12	+	120552498	120552698	120546404	120546601	120554717	120554808	32	18	10	0	0	0	1	5	0	1	98	49	1	1	1	1	1		1	0.714	0.143
SE	8315 RNF10	RNF10	chr12	+	120560725	120560886	120557545	120557682	120562944	120563070	56	34	6	1	0	0	0	12	0	0	98	49	1	1	0.966	1	1	NA		1	-0.011
SE	8316 RNF10	RNF10	chr12	+	120563346	120563623	120562944	120563070	120563809	120563943	38	19	16	0	1	0	1	4	0	0	98	49	1	1	1	0.905	1			1	-0.032
SE	8317 RNF10	RNF10	chr12	+	120565071	120565189	120563809	120563943	120565427	120565529	68	29	6	1	0	0	1	10	0	0	98	49	1	1	0.971	1	1		1	1	-0.01
SE	8318 RNF10	RNF10	chr12	+	120566824	120566980	120565427	120565529	120571190	120571291	37	26	14	1	0	0	1	8	0	0	98	49	1	1	0.949	1	1		1	1	-0.017
SE	8319 RNF10	RNF10	chr12	+	120566824	120566980	120565427	120565529	120575630	120575688	19	19	11	1	0	0	0	6	0	0	98	49	1	1	0.905	1	1	NA		1	-0.032
SE	8320 RNF10	RNF10	chr12	+	120571190	120571291	120565427	120565529	120575630	120575688	14	16	5	1	0	0	2	3	0	0	98	49	1	1	0.875	1	1		1	1	-0.042
SE	8325 COPS7A	COPS7A	chr12	+	6727925	6728001	6724282	6724818	6728222	6728311	11	8	5	4	2	0	0	0	0	1	98	49	1	1	0.579	0.667	1	NA		0	0.749
SE	8332 RAB3D	RAB3D	chr19	-	11335664	11335783	11335446	11335571	11337171	11337460	54	28	7	1	0	0	1	17	0	0	98	49	1	1	0.964	1	1		1	1	-0.012
SE	8335 DHTKD1	DHTKD1	chr10	+	12106245	12106396	12101041	12101181	12107908	12108015	10	5	8	0	1	0	0	1	0	0	98	49	1	1	1	0.714	1	NA		1	-0.095
SE	8336 TTLL3	TTLL3	chr3	+	9816073	9816202	9813247	9813345	9817644	9817759	6	4	0	4	2	1	0	1	0	0	98	49	1	1	0.429	0.5	0	NA		1	-0.69
SE	8337 TTLL3	TTLL3	chr3	+	9833103	9833245	9828959	9829395	9834680	9834907	4	3	4	1	3	3	0	2	0	0	98	49	1	1	0.667	0.333	0.4	NA		1	-0.533
SE	8340 CASP8	CASP8	chr2	+	201271515	201271621	201266460	201266791	201272637	201272776	142	47	33	7	10	0	0	9	0	1	98	49	1	1	0.91	0.701	1	NA		0.818	0.052
SE	8342 CASP8	CASP8	chr2	+	201272897	201272942	201272637	201272776	201276826	201276968	106	49	12	4	2	0	0	6	0	1	94	49	1	1	0.932	0.927	1	NA		0.758	0.195
SE	8343 CASP8	CASP8	chr2	+	201274888	201274953	201272637	201272776	201276826	201276968	89	36	17	4	2	0	0	3	0	1	98	49	1	1	0.918	0.9	1	NA		0.6	0.339
SE	8346 EIF4B	EIF4B	chr12	+	53039237	53039343	53038355	53038411	53039629	53039702	16	4	0	1	0	0	0	1	0	0	98	49	1	1	0.889	1	NA	NA		1	-0.055
SE	8350 IL4R	IL4R	chr16	+	27330065	27330198	27313908	27314020	27340185	27340273	13	11	3	1	0	2	0	1	0	0	98	49	1	1	0.867	1	0.429	NA		1	-0.235
SE	8351 IL4R	IL4R	chr16	+	27340185	27340273	27330065	27330198	27342120	27342259	66	31	10	0	0	1	2	6	0	0	98	49	1	1	1	1	0.833		1	1	-0.056
SE	8352 IL4R	IL4R	chr16	+	27341069	27341313	27340185	27340273	27342120	27342259	2	0	1	53	21	10	0	0	2	5	98	49	1	1	0.019	0	0.048		0	0	0.022
SE	8353 IL4R	IL4R	chr16	+	27346466	27346618	27344868	27345020	27352539	27352696	36	13	22	0	0	0	0	8	0	1	98	49	1	1	1	1	1	NA		0.8	0.2
SE	8354 IL4R	IL4R	chr16	+	27355807	27355907	27352539	27352696	27358915	27358994	34	12	14	2	0	0	0	7	0	0	98	49	1	1	0.895	1	1	NA		1	-0.035
SE	8355 IL4R	IL4R	chr16	+	27360765	27360815	27358915	27358994	27362251	27364778	14	13	6	0	0	0	0	4	0	2	98	49	1	1	1	1	1	NA		0.5	0.5
SE	8358 HNRNPDL	HNRNPDL	chr4	-	82425562	82425667	82422563	82424883	82426036	82426129	0	4	0	16	11	5	0	0	0	3	98	49	1	1	0	0.154	0	NA		0	0.051
SE	8359 HNRNPDL	HNRNPDL	chr4	-	82426462	82426633	82426036	82426129	82427189	82427304	28	8	4	3	0	0	0	1	0	0	98	49	1	1	0.824	1	1	NA		1	-0.059
SE	8360 ASPH	ASPH	chr8	-	61553030	61553120	61548070	61548208	61555923	61556022	5	8	1	0	0	0	2	4	0	1	98	49	1	1	1	1	1		1	0.667	0.166
SE	8361 ASPH	ASPH	chr8	-	61633682	61633727	61618977	61619019	61637946	61638003	9	10	6	1	0	0	0	1	0	0	94	49	1	1	0.824	1	1	NA		1	-0.059
SE	8362 ASPH	ASPH	chr8	-	61638321	61638360	61637946	61638003	61642887	61642920	11	12	3	1	1	0	1	6	0	0	88	49	1	1	0.86	0.87	1		1	1	-0.09
SE	8363 ASPH	ASPH	chr8	-	61638321	61638363	61637946	61638003	61642887	61642920	11	14	4	1	1	0	1	9	0	0	91	49	1	1	0.856	0.883	1		1	1	-0.087
SE	8364 ASPH	ASPH	chr8	-	61643944	61644001	61643385	61643433	61644599	61644632	12	19	2	1	0	0	0	2	0	0	98	49	1	1	0.857	1	1	NA		1	-0.048
SE	8365 ASPH	ASPH	chr8	-	61646749	61646878	61644599	61644632	61651049	61651124	21	6	0	0	1	1	1	8	0	0	98	49	1	1	1	0.75	0		1	1	-0.417
SE	8366 ASPH	ASPH	chr8	-	61682438	61682483	61680967	61681036	61684038	61684188	9	7	8	8	10	0	0	0	0	3	94	49	1	1	0.37	0.267	1	NA		0	0.546
SE	8378 BCAS3	BCAS3	chr17	+	61026865	61026910	61015750	61015901	61034665	61034790	1	0	0	10	0	0	0	0	0	4	94	49	1	1	0.05	NA	NA	NA		0	0.05
SE	8388 FRY	FRY	chr13	+	32131671	32131840	32124794	32124875	32134903	32134996	104	22	7	0	1	0	0	9	0	0	98	49	1	1	1	0.917	1	NA		1	-0.028
SE	8389 FRY	FRY	chr13	+	32147838	32147947	32147281	32147385	32149747	32149834	52	23	7	2	0	0	2	8	0	0	98	49	1	1	0.929	1	1		1	1	-0.024
SE	8391 FRY	FRY	chr13	+	32178176	32178436	32175545	32175632	32178843	32179033	65	17	5	2	0	0	3	7	0	0	98	49	1	1	0.97	1	1		1	1	-0.01

SE	8392 FRY	FRY	chr13	+	32194142	32194297	32187545	32187656	32201940	32202040	15	7	1	3	0	0	0	2	0	0	98	49	1	1	0.714	1	1	NA	1	-0.095
SE	8397 ATP2A3	ATP2A3	chr17	-	3951580	3951685	3951250	3951389	3953346	3953429	44	33	19	0	1	0	9	2	0	0	98	49	1	1	1	0.943	1	1	1	-0.019
SE	8399 ATP2A3	ATP2A3	chr17	-	3953692	3953710	3953346	3953429	3964173	3964464	22	10	13	0	1	0	2	5	0	0	67	49	1	1	1	0.88	1	1	1	-0.04
SE	8411 FCHO1	FCHO1	chr19	+	17755117	17755191	17754316	17754361	17762761	17762853	9	3	0	1	0	0	1	1	0	0	98	49	1	1	0.818	1	NA	1	1	-0.091
SE	8412 FCHO1	FCHO1	chr19	+	17781230	17781343	17778608	17778884	17781451	17781539	8	6	0	0	0	0	1	3	0	3	98	49	1	1	1	1	NA	1	0.333	0.334
SE	8413 OS9	OS9	chr12	+	57694749	57694926	57693954	57694323	57695779	57695843	52	34	24	0	1	0	3	9	0	0	98	49	1	1	1	0.944	1	1	1	-0.019
SE	8415 OS9	OS9	chr12	+	57695961	57696038	57695779	57695843	57715759	57715970	22	16	12	0	1	0	0	1	0	0	98	49	1	1	1	0.889	1	NA	1	-0.037
SE	8417 OS9	OS9	chr12	+	57716088	57716193	57715759	57715970	57716411	57716512	27	17	4	1	0	0	0	12	0	0	98	49	1	1	0.931	1	1	NA	1	-0.023
SE	8418 OS9	OS9	chr12	+	57716091	57716193	57715759	57715970	57716411	57716512	26	17	4	1	0	0	0	11	0	0	98	49	1	1	0.929	1	1	NA	1	-0.024
SE	8419 OS9	OS9	chr12	+	57720098	57720263	57718992	57719182	57720405	57720518	24	14	22	25	13	21	0	2	0	3	98	49	1	1	0.324	0.35	0.344	NA	0.25	0.089
SE	8420 NUCB2	NUCB2	chr11	+	17315385	17315475	17312027	17312120	17330126	17330297	1	0	0	0	0	0	0	0	1	0	98	49	1	1	1	NA	NA	0	NA	1
SE	8423 MTDH	MTDH	chr8	+	97699753	97699852	97690951	97691188	97706625	97706750	10	3	1	2	0	0	0	8	0	0	98	49	1	1	0.714	1	1	NA	1	-0.095
SE	8431 AGO1	AGO1	chr1	+	35893673	35893810	35893096	35893278	35894036	35894171	12	0	4	1	1	0	0	4	0	0	98	49	1	1	0.857	0	1	NA	1	-0.381
SE	8432 AGO1	AGO1	chr1	+	35906934	35907119	35902203	35902337	35913841	35914001	1	2	1	0	0	0	0	2	0	1	98	49	1	1	1	1	1	NA	0.5	0.5
SE	8442 CHD8	CHD8	chr14	-	21391462	21391642	21390946	21391063	21391832	21391946	18	7	4	2	1	0	0	1	0	0	98	49	1	1	0.818	0.778	1	NA	1	-0.135
SE	8443 CHD8	CHD8	chr14	-	21393105	21393254	21392506	21392809	21393475	21394195	25	1	2	0	0	0	2	2	0	1	98	49	1	1	1	1	1	1	0.5	0.25
SE	8444 CHD8	CHD8	chr14	-	21408311	21408555	21406855	21407032	21408703	21408825	19	11	4	1	1	0	0	1	0	0	98	49	1	1	0.905	0.846	1	NA	1	-0.083
SE	8445 AEBP2	AEBP2	chr12	+	19514670	19514784	19512397	19512465	19518086	19518113	6	0	0	1	0	0	0	6	0	0	98	49	1	1	0.75	NA	NA	NA	1	-0.25
SE	8446 KDM3B	KDM3B	chr5	+	138379583	138379708	138377719	138377825	138381515	138381590	21	16	1	2	2	0	0	8	0	0	98	49	1	1	0.84	0.8	1	NA	1	-0.12
SE	8447 KDM3B	KDM3B	chr5	+	138386021	138386621	138381515	138381590	138391012	138392261	8	8	5	0	0	0	0	2	0	2	98	49	1	1	1	1	1	NA	0.333	0.667
SE	8448 KDM3B	KDM3B	chr5	+	138391012	138392261	138381515	138381590	138393170	138393372	14	9	1	0	3	0	0	6	0	0	98	49	1	1	1	0.6	1	NA	1	-0.133
SE	8453 NRD1	NRD1	chr1	-	51790532	51790649	51789567	51789657	51790899	51790990	74	20	12	1	0	0	0	17	0	0	98	49	1	1	0.974	1	1	NA	1	-0.009
SE	8454 NRD1	NRD1	chr1	-	51791577	51791661	51790899	51790990	51792045	51792098	42	26	7	1	0	0	0	13	0	0	98	49	1	1	0.955	1	1	NA	1	-0.015
SE	8455 NRD1	NRD1	chr1	-	51800555	51800683	51798248	51798411	51803813	51803964	56	22	7	0	0	0	0	18	0	1	98	49	1	1	1	1	1	NA	0.9	0.1
SE	8456 NRD1	NRD1	chr1	-	51819799	51819873	51818065	51818135	51821497	51821555	62	28	13	3	0	0	0	24	0	0	98	49	1	1	0.912	1	1	NA	1	-0.029
SE	8464 MAST3	MAST3	chr19	+	18128399	18128458	18124264	18124366	18128865	18128951	36	23	1	0	1	0	1	4	0	0	98	49	1	1	1	0.92	1	1	1	-0.027
SE	8465 MAST3	MAST3	chr19	+	18134816	18134982	18134578	18134711	18135739	18135841	57	17	9	2	3	0	0	12	0	0	98	49	1	1	0.934	0.739	1	NA	1	-0.109
SE	8470 ST8SIA4	ST8SIA4	chr5	-	100856102	100856396	100806934	100812129	100886342	100886600	82	44	51	5	2	1	0	12	0	0	98	49	1	1	0.891	0.917	0.962	NA	1	-0.077
SE	8471 ST8SIA4	ST8SIA4	chr5	-	100895653	100895785	100885310	100886600	100902842	100903285	87	58	27	0	2	0	4	14	0	0	98	49	1	1	1	0.935	1	1	1	-0.022
SE	8473 CCNH	CCNH	chr5	-	87404843	87405007	87401701	87401772	87407975	87408186	43	11	1	2	0	0	0	11	0	0	98	49	1	1	0.915	1	1	NA	1	-0.028
SE	8481 GABPB2	GABPB2	chr1	+	151103561	151103675	151097851	151098002	151107036	151107222	1	0	0	0	4	0	0	1	0	0	98	49	1	1	1	0	NA	NA	1	-0.5
SE	8482 GABPB2	GABPB2	chr1	+	151117391	151117516	151107036	151107222	151117956	151118531	0	0	0	1	0	0	0	2	0	0	98	49	1	1	0	NA	NA	NA	1	-1
SE	8485 SCYL3	SCYL3	chr1	-	169855795	169855957	169854269	169854964	169859040	169859212	1	0	0	0	0	1	0	0	0	1	98	49	1	1	1	NA	0	NA	0	0.5
SE	8500 DPF2	DPF2	chr11	+	65343990	65344069	65343744	65343837	65345665	65345803	9	0	5	0	3	0	0	3	0	0	98	49	1	1	1	0	1	NA	1	-0.333
SE	8504 ABI1	ABI1	chr10	-	26755654	26755741	26751597	26751783	26759061	26759235	4	0	0	8	10	1	0	1	0	10	98	49	1	1	0.2	0	0	NA	0.048	0.019
SE	8505 ABI1	ABI1	chr10	-	26755654	26755741	26751597	26751783	26765217	26765318	0	0	0	8	8	3	0	1	0	0	98	49	1	1	0	0	0	NA	1	-1
SE	8506 ABI1	ABI1	chr10	-	26759061	26759235	26751597	26751783	26765217	26765318	9	13	1	8	8	3	0	12	0	0	98	49	1	1	0.36	0.448	0.143	NA	1	-0.683
SE	8508 ABI1	ABI1	chr10	-	26759061	26759238	26751597	26751783	26765217	26765318	12	12	2	8	8	3	0	11	0	0	98	49	1	1	0.429	0.429	0.25	NA	1	-0.631
SE	8510 ABI1	ABI1	chr10	-	26763879	26763960	26759061	26759238	26765217	26765318	1	0	0	4	2	1	0	0	0	1	98	49	1	1	0.111	0	0	NA	0	0.037
SE	8511 ABI1	ABI1	chr10	-	26771074	26771089	26770244	26770345	26777064	26777241	7	4	4	13	1	0	0	0	0	1	64	49	1	1	0.292	0.754	1	NA	0	0.682
SE	8519 SELK	SELK	chr3	-	53885825	53885912	53885198	53885561	53886850	53886934	39	34	16	0	1	0	2	9	0	0	98	49	1	1	1	0.944	1	1	1	-0.019
SE	8522 RFC2	RFC2	chr7	-	74249011	74249118	74246661	74246763	74249738	74249780	5	0	4	3	0	0	3	0	0	0	98	49	1	1	0.455	NA	1	1	NA	-0.272
SE	8523 RFC2	RFC2	chr7	-	74249011	74249220	74246661	74246763	74249738	74249780	6	0	4	3	0	0	3	0	0	0	98	49	1	1	0.5	NA	1	1	NA	-0.25
SE	8526 NFKBIZ	NFKBIZ	chr3	+	101857072	101857183	101855732	101855902	101857291	101857459	56	36	6	2	2	5	0	19	0	3	98	49	1	1	0.933	0.9	0.375	NA	0.76	-0.024
SE	8527 KIAA0556	KIAA0556	chr16	+	27699533	27699599	27698327	27698500	27701588	27701695	14	3	0	2	0	0	2	3	0	0	98	49	1	1	0.778	1	NA	1	1	-0.111
SE	8528 KIAA0556	KIAA0556	chr16	+	27708704	27708920	27703895	27703998	27721557	27721695	11	1	0	1	0	0	0	1	0	0	98	49	1	1	0.846	1	NA	NA	1	-0.077
SE	8529 KIAA0556	KIAA0556	chr16	+	27754172	27754251	27751718	27751924	27761412	27761590	5	4	2	0	1	0	0	4	0	0	98	49	1	1	1	0.667	1	NA	1	-0.111
SE	8530 KIAA0556	KIAA0556	chr16	+	27777609	27777770	27776927	27777029	27777880	27777969	8	2	1	1	0	0	2	2	0	0	98	49	1	1	0.8	1	1	1	1	-0.067
SE	8532 ITGAM	ITGAM	chr16	+	31261691	31261797	31259966	31260092	31265394	31265498	29	18	4	0	2	0	0	9	0	0	98	49	1	1	1	0.818	1	NA	1	-0.061
SE	8533 ITGAM	ITGAM	chr16	+	31277966	31278109	31276919	31277049	31297513	31297654	53	36	11	1	1	0	0	11	0	0	98	49	1	1	0.964	0.947	1	NA	1	-0.03
SE	8534 ITGAM	ITGAM	chr16	+	31297741	31297954	31297513	31297654	31321240	31321371	30	7	3	2	0	0	3	14	0	0	98	49	1	1	0.882	1	1	1	1	-0.039
SE	8535 ITGAM	ITGAM	chr16	+	31297744	31297954	31297513	31297654	31321240	31321371	38	15	4	2	0	0	2	19	0	0	98	49	1	1	0.905	1	1	1	1	-0.032
SE	8536 ITGAM	ITGAM	chr16	+	31321463	31321627	31321240	31321371	31324398	31324553	52	25	23	0	0	0	0	12	0	1	98	49	1	1	1	1	1	NA	0.857	0.143
SE	8537 ATG3	ATG3	chr3	-	112553279	112553329	112550191	112550262	112558375	112558417	31	9	9	1	3	0	0	4	0	3	98	49	1	1	0.939	0.6	1	NA	0.4	0.446
SE	8538 CPSF6	CPSF6	chr12	+	69257731	69257905	69256696	69256842	69258589	69259094	7	5	0	0	0	1	0	2	0	0	98	49	1	1	1	1	0	NA	1	-0.333
SE	8547 TMEM180	TMEM180	chr10	+	102470664	102471117	102469992	102470075	102471272	102471396	0	0	0	1	2	0	0	1	0	0	98	49	1	1	0	0	NA	NA	1	-1
SE	8551 UBR3	UBR3	chr2	+	169877493	169877637	169875790	169875949	169878524	169878574	27	11	0	1	0	0	0	3	0	0	98	49	1	1	0.931	1	NA	NA	1	-0.034
SE	8577 MTHFR	MTHFR	chr1	-	11801160	11801399	11800211	11800322	11802880	11803129	7	2	0	1	0	0	0	1	0	0	98	49	1	1	0.778	1	NA	NA	1	-0.111
SE	8582 RASSF3	RASSF3	chr12	+	64684786	64684894	64610512	64610743	64691469	64691579	51	35	26	1	0	0	0	16	0	0	98	49	1	1	0.962	1	1	NA	1	-0.013
SE	8595 MINOS1	MINOS1	chr1	+	19625391	19625632	19623473	19623583	19626386	19629821	1	0	0	0	3	0	0	0	0	1	98	49	1	1	1	0	NA	NA	0	0.5
SE	8603 MAPKAPK2	MAPKAPK2	chr1	+	206730687	206730763	206729971	206730098	206731137	206731262	32	21	5	0	1	0	1	6	0	0	98	49	1	1	1	0.913	1	1	1	-0.029
SE	8607 TKT	TKT	chr3	-	53240250	53240348	53234982	53235174	53241131	53241245	159	86	68	1	0	0	8	59	0	0	98	49	1	1	0.988	1	1	1	1	-0.004
SE	8624 EPS15	EPS15	chr1	-	51448045	51448135	51446959	51447105	51461090	51461150	16	6	5	1	0	0	0	8	0	0	98	49	1	1	0.889	1	1	NA	1	-0.037
SE	8625 CTR9	CTR9	chr11	+	10755677	10755795	10754957	10755197	10756748	10756838	2	3	4	0	0	0	0	2	0	1	98	49	1	1	1	1	1	NA	0.5	0.5
SE	8627 ANKRD13C	ANKRD13C	chr1	-	70306223	70306290	70300763	70300908	70313744	70313790	8	0	0	0	0	1	0	1	0	0	98	49	1	1	1	NA	0	NA	1	-0.5
SE	8633 REC8	REC8	chr14	+	24175542	24175624	24173290	24173411	24176821	24176901	14	13	0	1	1	0	3	10	0	0	98	49	1	1	0.875	0.867	NA	1	1	-0.129
SE	8634 REC8	REC8	chr14	+	24179084	24179133	24178776	24178916	24179396	24179463	4	2	0	1	1	0	0	2	0	0	98	49	1	1	0.667	0.5	NA	NA	1	-0.416
SE	8635 OSBPL11	OSBPL11	chr3	-	125552180	125552679	125547405	125547592	125560378	125560519	11	4	4	2	0	2	0	5	0	1	98	49	1	1	0.733	1	0.5	NA	0.714	0.03
SE	8644 ZFAND1	ZFAND1	chr8	-	81717248	81717288	81714986	81715114	81718181	81718224	2	0	0	2	0	0	0	2	0	1	89	49	1	1	0.355	NA	NA	NA	0.524	-0.169
SE	8646 SAMSN1	SAMSN1	chr21	-	14510309	14510461	14500528	14500735	14512443	14512573	34	13	0	4	0	0	0	29	0	0	98	49	1	1	0.81	1	NA	NA	1	-0.095
SE	8649 RABGGTB	RABGGTB	chr1	+	75792180	75792306	75791460	75791571	75794083	75794233	3	8	0	0	1	0	0	3	0	0	98	49	1	1	1	0.8	NA	NA	1	-0.1
SE	8650 DDX21	DDX21	chr10	+	68963290	68963469	68962081	68962157	68965376	68965494	36	20	1	1	0	0	1	5	0	0	98	49	1	1	0.947	1	1	1	1	-0.018
SE	8651 DDX21	DDX21	chr10	+	68970200	68970350	68968975	68969121	68973544	68973664	18	10	0	0	1	0	0	9	0	0	98	49	1	1	1	0.833	NA	NA	1	-0.084
SE	8652 DDX21	DDX21	chr10	+	68971890	68972052	68970200	68970350	68973544	68973664	6	4	0	2	0	0	0	3	0	0	98	49	1	1	0.6	1	NA	NA	1	-0.2
SE	8665 RNASE4	RNASE4	chr14	+	20693546	20693650	20684212	20684758	20699354	20700602	2	0	0	0	0	0	0	0	0	1	98	49	1	1	1	NA	NA	NA	0	1
SE	8680 FGR	FGR	chr1	-	27614429	27614583	27613218	27613350	27614849	27614926	151	51	50	1	0	0	25	47	0	0	98	49	1	1	0.987	1	1	1	1	-0.004
SE	8681 FGR	FGR	chr1	-	27614849	27614926	27614429	27614583	27615433	27615613	131	57	34	1	0	0	9	55	0	0	98	49	1	1	0.985	1	1	1	1	-0.005
SE	8682 FGR	FGR	chr1	-	27617192	27617296	27616856	27617006	27621558	27621657	145	63	78	3	1	0	3	47	0	1	98	49	1	1	0.96	0.969	1	1	0.959	-0.003
SE	8683 KIAA0195	KIAA0195	chr17	+	75497112	75497198	75496729	75496807	75497780	75497862	0	0	2	0	2	0	0	1	0	0	98	49	1	1	NA	0	1	NA	1	-0.5
SE	8692 POGZ	POGZ	chr1	-	151424954	151425061	151423948	151424286	151430665	151430841	4	2	0	1	0	0	0	2	0	0	98	49	1	1	0.667	1	NA	NA	1	-0.166
SE	8695 POGZ	POGZ	chr1	-	151428122	151428413	151424954	151425061	151430665	151430841	2	0	0	0	0	0	0	0	0	2	98	49	1	1	1	NA	NA	NA	0	1
SE	8696 POGZ	POGZ	chr1	-	151429602	151429711	151428122	151428413	151430665	151430841	1	1	0	1	0	0	0	3	0	0	98	49	1	1	0.333	1	NA	NA	1	-0.334
SE	8699 NAGK	NAGK	chr2	+	71071685	71071827	71070740	71070839	71072640	71072751	28	24	6	1	0	0	2	11	0	0	98	49	1	1	0.933	1	1	1	1	-0.022
SE	8705 PCSK7	PCSK7	chr11	-	117208896	117209053	117207969	117208064	117218465	117218568	8	8	0	2	0	0	0	0	0	3	98	49	1	1	0.667	1	NA	NA	0	0.834
SE	8708 IFT88	IFT88	chr13	+	20615792	20615879	20605034	20605105	20625749	20625849	1	1	4	2	0	0	0	2	0	0	98	49	1	1	0.2	1	1	NA	1	-0.267
SE	8718 IFI44	IFI44	chr1	+	78660553	78660654	78659311	78659483	78662703	78662878	7	2	0	2	0	0	0	36	0	6	98	49	1	1	0.636	1	NA	NA	0.75	0.068
SE	8719 IFI44	IFI44	chr1	+	78660553	78660654	78659311	78659483	78663764	78664078	5	1	0	1	0	0	0	31	1	1	98	49	1	1	0.714	1	NA	0	0.939	0.388
SE	8720 IFI44	IFI44	chr1	+	78662703	78662878	78659311	78659483	78663764	78664078	12	6	0	1	0	0	6	51	1	1	98	49	1	1	0.857	1	NA	0.75	0.962	0.073
SE	8721 IFI44	IFI44	chr1	+	78662703	78662878	78660553	78660654	78663764	78664078	12	7	0	0	0	0	6	52	0	2	98	49	1	1	1	1	NA	1	0.929	0.035
SE	8722 CTSZ	CTSZ	chr20	-	58996638	58996801	58995186	58995759	58997602	58997753	62	27	18	0	0	1	0	22	0	0	98	49	1	1	1	1	0.9	NA	1	-0.033
SE	8725 RABGAP1L	RABGAP1L	chr1	+	174988640	174988838	174982833	174982905	174989848	174995308	21	9	10	0	5	0	0	4	0	0	98	49	1	1	1	0.474	1	NA	1	-0.175
SE	8728 TBC1D10C	TBC1D10C	chr11	+	67405398	67405506	67405084	67405184	67405594	67405701	48	38	14	0	0	1	0	7	0	0	98	49	1	1	1	1	0.875	NA	1	-0.042
SE	8729 TBC1D10C	TBC1D10C	chr11	+	67406626	67406692	67405902	67406017	67408978	67409133	12	14	9	0	0	1	0	5	0	0	98	49	1	1	1	1	0.818	NA	1	-0.061
SE	8735 PTBP1	PTBP1	chr19	+	805491	805569	805012	805187	806407	806556	7	0	0	2	0	0	0	1	0	0	98	49	1	1	0.636	NA	NA	NA	1	-0.364
SE	8736 PTBP1	PTBP1	chr19	+	805512	805569	805012	805187	806407	806556	1	0	0	2	0	0	0	1	0	0	98	49	1	1	0.2	NA	NA	NA	1	-0.8
SE	8737 PTBP1	PTBP1	chr19	+	807868	807902	806407	806556	808359	808452	4	0	0	0	0	0	0	3	0	1	83	49	1	1	1	NA	NA	NA	0.639	0.361
SE	8757 ZNF106	ZNF106	chr15	-	42417804	42417951	42412436	42417360	42421060	42421132	50	5	5	4	4	0	0	5	0	0	98	49	1	1	0.862	0.385	1	NA	1	-0.251
SE	8758 ZNF106	ZNF106	chr15	-	42428017	42428134	42424833	42425025	42435383	42435518	53	15	17	0	0	0	0	7	0	1	98	49	1	1	1	1	1	NA	0.778	0.222
SE	8759 ZNF106	ZNF106	chr15	-	42449770	42451954	42448071	42448705	42466052	42466114	19	7	4	2	0	0	0	3	0	0	98	49	1	1	0.826	1	1	NA	1	-0.058
SE	8763 RAB3GAP2	RAB3GAP2	chr1	-	220170891	220171120	220167501	220167675	220171888	220172049	5	0	0	1	0	0	0	0	1	0	98	49	1	1	0.714	NA	NA	0	NA	0.714
SE	8770 ZCCHC2	ZCCHC2	chr18	+	62556202	62556297	62550347	62550460	62558686	62558770	28	33	4	0	0	0	0	20	0	2	98	49	1	1	1	1	1	NA	0.833	0.167
SE	8771 ZCCHC2	ZCCHC2	chr18	+	62558686	62558770	62556202	62556297	62560586	62560644	17	12	4	0	3	0	0	13	0	2	98	49	1	1	1	0.667	1	NA	0.765	0.124
SE	8780 SNW1	SNW1	chr14	-	77723180	77723277	77720710	77720828	77730987	77731129	12	8	5	1	1	0	2	0	0	0	98	49	1	1	0.857	0.8	1	1	NA	-0.114
SE	8781 SNW1	SNW1	chr14	-	77735936	77736006	77734946	77735012	77736970	77737075	16	16	11	1	0	0	0	5	0	0	98	49	1	1	0.889	1	1	NA	1	-0.037
SE	8786 ZKSCAN5	ZKSCAN5	chr7	+	99520168	99520304	99519826	99519909	99525812	99526418	0	5	0	0	3	0	0	0	0	1	98	49	1	1	NA	0.455	NA	NA	0	0.455
SE	8789 CD46	CD46	chr1	+	207767606	207767651	207767012	207767195	207767778	207767823	16	7	1	68	16	11	0	7	0	0	94	49	1	1	0.109	0.186	0.045	NA	1	-0.887
SE	8790 CD46	CD46	chr1	+	207767778	207767823	207767012	207767195	207770320	207770362	140	36	38	11	2	9	0	12	0	0	94	49	1	1	0.869	0.904	0.688	NA	1	-0.18
SE	8793 CD46	CD46	chr1	+	207790252	207790345	207785618	207785682	207793518	207795516	88	36	23	89	37	22	0	12	0	6	98	49	1	1	0.331	0.327	0.343	NA	0.5	-0.166
SE	8802 CELF1	CELF1	chr11	-	47483452	47483532	47482694	47482856	47484388	47484520	31	1	5	0	1	0	0	4	0	0	98	49	1	1	1	0.333	1	NA	1	-0.222
SE	8803 CELF1	CELF1	chr11	-	47484388	47484520	47482694	47482856	47486749	47486798	0	1	0	0	0	0	0	0	0	1	98	49	1	1	NA	1	NA	NA	0	1
SE	8804 CELF1	CELF1	chr11	-	47484388	47484523	47482694	47482856	47486749	47486798	6	3	0	0	0	0	0	2	0	1	98	49	1	1	1	1	NA	NA	0.5	0.5
SE	8805 ASH1L	ASH1L	chr1	-	155415743	155415923	155395458	155395553	155438326	155439068	13	1	0	0	2	0	0	2	0	0	98	49	1	1	1	0.2	NA	NA	1	-0.4
SE	8806 TLR5	TLR5	chr1	-	223137177	223137263	223134681	223134864	223141647	223141763	4	0	0	2	0	1	0	7	0	0	98	49	1	1	0.5	NA	0	NA	1	-0.75
SE	8807 TLR5	TLR5	chr1	-	223141647	223141763	223137177	223137263	223143195	223143282	2	1	0	0	0	1	0	3	0	0	98	49	1	1	1	1	0	NA	1	-0.333
SE	8808 C1orf52	C1orf52	chr1	-	85258934	85259061	85258523	85258722	85259357	85259672	3	8	4	0	0	1	0	1	0	0	98	49	1	1	1	1	0.667	NA	1	-0.111
SE	8820 LBR	LBR	chr1	-	225410290	225410416	225406663	225406832	225411336	225411440	78	21	18	5	3	6	0	18	0	3	98	49	1	1	0.886	0.778	0.6	NA	0.75	0.005
SE	8821 LBR	LBR	chr1	-	225411336	225411440	225406663	225406832	225412453	225412645	48	39	33	0	0	1	0	6	0	0	98	49	1	1	1	1	0.943	NA	1	-0.019
SE	8822 AQP9	AQP9	chr15	+	58173067	58173205	58166672	58166799	58174917	58175036	290	177	171	2	0	0	2	58	0	0	98	49	1	1	0.986	1	1	1	1	-0.005
SE	8823 AQP9	AQP9	chr15	+	58174917	58175036	58173067	58173205	58179127	58179345	289	151	147	0	0	0	1	43	0	1	98	49	1	1	1	1	1	1	0.956	0.022
SE	8826 DOCK2	DOCK2	chr5	+	169670541	169670597	169669287	169669328	169671077	169671174	96	41	16	1	0	0	0	22	0	0	98	49	1	1	0.98	1	1	NA	1	-0.007
SE	8827 DOCK2	DOCK2	chr5	+	169671077	169671174	169670541	169670597	169674296	169674445	205	65	16	0	1	0	2	36	0	0	98	49	1	1	1	0.97	1	1	1	-0.01
SE	8829 DOCK2	DOCK2	chr5	+	169689251	169689333	169684195	169684350	169695802	169695938	176	92	10	2	0	0	5	49	0	0	98	49	1	1	0.978	1	1	1	1	-0.007
SE	8830 DOCK2	DOCK2	chr5	+	169712119	169712223	169711934	169712007	169714027	169714211	141	46	6	0	1	0	4	19	0	0	98	49	1	1	1	0.958	1	1	1	-0.014
SE	8833 DOCK2	DOCK2	chr5	+	169803057	169803206	169761518	169761625	169840756	169840852	143	72	20	0	2	0	2	21	0	0	98	49	1	1	1	0.947	1	1	1	-0.018
SE	8834 DOCK2	DOCK2	chr5	+	169983067	169983166	169840756	169840852	169985827	169985922	45	22	7	0	0	0	0	12	0	1	98	49	1	1	1	1	1	NA	0.857	0.143
SE	8835 DOCK2	DOCK2	chr5	+	170008687	170008746	170008496	170008597	170018959	170019108	74	29	11	0	1	0	2	21	0	0	98	49	1	1	1	0.935	1	1	1	-0.022
SE	8836 DOCK2	DOCK2	chr5	+	170034398	170034555	170027862	170027948	170036514	170036555	61	8	18	1	0	0	0	16	0	0	98	49	1	1	0.968	1	1	NA	1	-0.011
SE	8837 DOCK2	DOCK2	chr5	+	170045815	170045905	170042012	170042132	170047509	170047614	49	19	5	0	1	0	1	14	0	0	98	49	1	1	1	0.905	1	1	1	-0.032
SE	8841 RPLP2	RPLP2	chr11	+	810233	810357	809935	810039	811596	811645	104	26	3	2	0	0	1	24	0	0	98	49	1	1	0.963	1	1	1	1	-0.012
SE	8849 CORO1A	CORO1A	chr16	+	30188360	30188576	30188191	30188249	30188859	30189076	248	133	94	0	1	0	2	46	0	0	98	49	1	1	1	0.985	1	1	1	-0.005
SE	8852 NCOA6	NCOA6	chr20	-	34727258	34727407	34714773	34715365	34732558	34732595	9	11	4	0	0	0	0	1	0	2	98	49	1	1	1	1	1	NA	0.2	0.8
SE	8857 SEPSECS	SEPSECS	chr4	-	25145003	25145133	25144773	25144865	25151959	25152062	0	0	2	2	0	0	0	1	0	0	98	49	1	1	0	NA	1	NA	1	-0.5
SE	8859 GUK1	GUK1	chr1	+	228145510	228145624	228140227	228140363	228146025	228146067	26	8	4	2	0	0	0	3	0	0	98	49	1	1	0.867	1	1	NA	1	-0.044
SE	8861 IFNAR1	IFNAR1	chr21	+	33343267	33343422	33340998	33341174	33343534	33343676	51	46	6	0	0	0	0	6	0	1	98	49	1	1	1	1	1	NA	0.75	0.25
SE	8863 ZC3HAV1	ZC3HAV1	chr7	-	139061035	139061138	139055204	139055295	139064878	139064999	22	5	2	0	1	0	1	2	0	0	98	49	1	1	1	0.714	1	1	1	-0.095
SE	8864 ZC3HAV1	ZC3HAV1	chr7	-	139076285	139076409	139073855	139074030	139078551	139078653	30	7	2	5	4	1	0	12	0	0	98	49	1	1	0.75	0.467	0.5	NA	1	-0.428
SE	8866 SYF2	SYF2	chr1	-	25228997	25229123	25228117	25228235	25232103	25232211	15	21	19	1	0	0	0	1	0	0	98	49	1	1	0.882	1	1	NA	1	-0.039
SE	8869 SRF	SRF	chr6	+	43175705	43175967	43173846	43174113	43176547	43176667	8	1	7	1	0	0	0	3	0	0	98	49	1	1	0.8	1	1	NA	1	-0.067
SE	8872 CLMN	CLMN	chr14	-	95196497	95196694	95194535	95194596	95202837	95204463	2	0	0	1	0	0	0	2	0	0	98	49	1	1	0.5	NA	NA	NA	1	-0.5
SE	8874 TRIM8	TRIM8	chr10	+	102654652	102654748	102644494	102645187	102655079	102655313	26	7	7	0	1	0	0	8	0	1	98	49	1	1	1	0.778	1	NA	0.8	0.126
SE	8878 CPEB2	CPEB2	chr4	+	15007304	15007586	15002673	15004335	15017187	15017278	8	1	1	0	0	0	0	4	0	1	98	49	1	1	1	1	1	NA	0.667	0.333
SE	8879 CPEB2	CPEB2	chr4	+	15007304	15007595	15002673	15004335	15017187	15017278	6	3	1	0	0	0	0	3	0	1	98	49	1	1	1	1	1	NA	0.6	0.4
SE	8880 CPEB2	CPEB2	chr4	+	15008337	15008427	15007304	15007586	15017187	15017278	1	0	0	3	1	0	0	0	0	2	98	49	1	1	0.143	0	NA	NA	0	0.071
SE	8883 DENND1B	DENND1B	chr1	-	197647054	197647114	197645689	197645743	197652234	197652315	8	1	2	7	0	4	1	0	0	0	98	49	1	1	0.364	1	0.2	1	NA	-0.479
SE	8884 DENND1B	DENND1B	chr1	-	197715030	197715074	197674119	197674169	197772867	197772932	4	0	2	1	0	0	2	1	0	0	93	49	1	1	0.678	NA	1	1	1	-0.161
SE	8891 PTPRA	PTPRA	chr20	+	2975214	2975241	2964860	2965202	2986764	2986849	1	0	0	5	2	0	0	0	0	3	76	49	1	1	0.114	0	NA	NA	0	0.057
SE	8892 PTPRA	PTPRA	chr20	+	3022053	3022220	3021308	3021428	3022688	3022824	8	1	2	0	1	0	0	6	0	0	98	49	1	1	1	0.333	1	NA	1	-0.222
SE	8896 CASP5	CASP5	chr11	-	105007082	105007334	105003273	105003383	105008806	105008980	13	2	0	1	0	0	0	2	0	0	98	49	1	1	0.867	1	NA	NA	1	-0.067
SE	8917 PITHD1	PITHD1	chr1	+	23779437	23779481	23778385	23778713	23779863	23779941	0	1	2	1	0	0	1	0	0	0	93	49	1	1	0	1	1	1	NA	-0.333
SE	8923 DISC1	DISC1	chr1	+	231720847	231720924	231701954	231702024	231749925	231750619	1	0	0	4	7	3	0	0	0	3	98	49	1	1	0.111	0	0	NA	0	0.037
SE	8932 PLCB2	PLCB2	chr15	-	40290024	40290082	40289271	40289358	40290576	40290672	63	24	12	0	3	0	0	8	0	0	98	49	1	1	1	0.8	1	NA	1	-0.067
SE	8933 PLCB2	PLCB2	chr15	-	40291605	40291650	40291264	40291487	40291848	40291924	10	5	0	10	3	1	0	1	0	2	94	49	1	1	0.343	0.465	0	NA	0.207	0.062
SE	8934 PLCB2	PLCB2	chr15	-	40297876	40297959	40297520	40297605	40298222	40298380	59	15	5	1	0	0	1	6	0	0	98	49	1	1	0.967	1	1	1	1	-0.011
SE	8940 RBM10	RBM10	chrX	+	47147356	47147498	47145217	47145485	47169314	47169498	14	2	2	0	1	0	2	3	0	0	98	49	1	1	1	0.5	1	1	1	-0.167
SE	8942 RBM10	RBM10	chrX	+	47175018	47175092	47173127	47173197	47176499	47176586	2	4	0	3	0	0	0	1	0	0	98	49	1	1	0.25	1	NA	NA	1	-0.375

SE	8967 HEATR3	HEATR3	chr16	+	50072604	50072714	50070177	50070290	50075570	50075711	7	0	0	2	0	0	0	0	0	2	98	49	1	1	0.636 NA	NA	NA		0	0.636
SE	8971 FCGR2A	FCGR2A	chr1	+	161506333	161506591	161505986	161506007	161509819	161510074	225	98	85	1	1	0	2	42	0	0	98	49	1	1	0.991	0.98	1	1	1	-0.01
SE	8972 FCGR2A	FCGR2A	chr1	+	161506336	161506591	161505986	161506007	161509819	161510074	241	93	87	1	1	0	1	45	0	0	98	49	1	1	0.992	0.979	1	1	1	-0.01
SE	8974 SGPL1	SGPL1	chr10	+	70854707	70854855	70851142	70851210	70857613	70857690	6	0	1	1	0	0	0	2	0	0	98	49	1	1	0.75 NA		1 NA		1	-0.125
SE	8975 SDCCAG8	SDCCAG8	chr1	+	243293090	243293219	243286271	243286397	243304712	243304777	5	1	0	0	0	0	0	0	0	1	98	49	1	1	1	1 NA	NA		0	1
SE	8977 SAT1	SAT1	chrX	+	23783799	23783883	23783657	23783709	23785327	23785429	590	359	141	2	3	0	20	265	1	1	98	49	1	1	0.993	0.984	1	0.909	0.993	0.041
SE	8982 GTF2F1	GTF2F1	chr19	-	6383310	6383495	6381696	6381850	6387388	6387559	5	3	0	0	0	0	0	1	0	1	98	49	1	1	1	1 NA	NA		0.333	0.667
SE	8983 RUNX2	RUNX2	chr6	+	45491940	45492114	45437946	45438051	45512245	45512407	5	1	5	1	0	0	0	1	0	2	98	49	1	1	0.714	1	1 NA		0.2	0.705
SE	8989 GLYR1	GLYR1	chr16	-	4821379	4821453	4817597	4817697	4822874	4822931	13	4	2	1	0	0	3	6	0	0	98	49	1	1	0.867	1	1	1	1	-0.044
SE	8991 GLYR1	GLYR1	chr16	-	4831978	4832221	4823820	4823907	4832773	4832912	12	13	1	2	2	0	0	1	0	0	98	49	1	1	0.75	0.765	1 NA		1	-0.162
SE	9001 ZDHHC20	ZDHHC20	chr13	-	21378660	21378738	21372570	21376655	21381433	21381549	3	0	2	7	2	0	0	3	0	2	98	49	1	1	0.176	0	1 NA		0.429	-0.037
SE	9002 ZDHHC20	ZDHHC20	chr13	-	21381900	21381945	21381433	21381546	21382919	21383009	1	1	6	1	0	0	0	2	0	3	94	49	1	1	0.343	1	1 NA		0.258	0.523
SE	9003 ZDHHC20	ZDHHC20	chr13	-	21381900	21381945	21381433	21381549	21382919	21383009	1	1	6	7	0	0	0	3	0	5	94	49	1	1	0.069	1	1 NA		0.238	0.452
SE	9004 ZDHHC20	ZDHHC20	chr13	-	21400372	21400493	21391721	21391854	21401652	21401685	11	5	0	0	4	0	0	1	0	0	98	49	1	1	1	0.385 NA	NA		1	-0.307
SE	9005 ZDHHC20	ZDHHC20	chr13	-	21404224	21404342	21402796	21402866	21413651	21413772	0	0	0	6	0	0	0	1	0	3	98	49	1	1	0 NA	NA	NA		0.143	-0.143
SE	9006 ZDHHC20	ZDHHC20	chr13	-	21421060	21421164	21413651	21413772	21459053	21459370	7	4	2	0	1	0	0	3	0	0	98	49	1	1	1	0.667	1 NA		1	-0.111
SE	9008 HLA-B	HLA-B	chr6	-	31354632	31354665	31354478	31354526	31355106	31355223	1036	480	341	0	0	0	16	132	0	1	82	49	1	1	1	1	1	1	0.987	0.006
SE	9010 HLA-B	HLA-B	chr6	-	31356166	31356442	31355316	31355592	31356687	31356957	1295	658	422	0	1	0	23	208	0	0	98	49	1	1	1	0.997	1	1	1	-0.001
SE	9017 HSD17B11	HSD17B11	chr4	-	87374698	87374830	87372708	87372815	87382254	87382362	170	78	84	1	0	0	3	26	0	1	98	49	1	1	0.988	1	1	1	0.929	0.031
SE	9023 CDC123	CDC123	chr10	+	12249580	12249718	12246148	12246277	12250310	12250590	22	15	11	5	0	0	0	3	0	0	98	49	1	1	0.687	1	1 NA		1	-0.104
SE	9031 DENND5A	DENND5A	chr11	-	9142721	9142845	9141939	9142108	9143402	9143485	80	32	21	0	2	0	0	11	0	0	98	49	1	1	1	0.889	1 NA		1	-0.037
SE	9032 DENND5A	DENND5A	chr11	-	9142721	9142845	9141939	9142108	9144096	9144278	65	20	10	1	6	0	0	11	0	0	98	49	1	1	0.97	0.625	1 NA		1	-0.135
SE	9033 DENND5A	DENND5A	chr11	-	9143402	9143485	9141939	9142108	9144096	9144278	36	15	7	1	6	0	0	7	0	0	98	49	1	1	0.947	0.556	1 NA		1	-0.166
SE	9034 DENND5A	DENND5A	chr11	-	9143402	9143485	9142721	9142845	9144096	9144278	58	26	18	7	1	0	0	7	0	0	98	49	1	1	0.806	0.929	1 NA		1	-0.088
SE	9037 DENND5A	DENND5A	chr11	-	9150679	9150764	9150080	9150209	9152357	9152442	63	40	27	1	2	0	0	16	0	0	98	49	1	1	0.969	0.909	1 NA		1	-0.041
SE	9043 CCAR1	CCAR1	chr10	+	68756272	68756483	68755369	68755536	68757293	68757377	9	0	0	4	0	0	0	2	0	0	98	49	1	1	0.529 NA	NA	NA		1	-0.471
SE	9045 CEP57	CEP57	chr11	+	95795515	95795559	95790460	95790743	95799231	95799388	1	1	0	2	2	2	0	0	0	1	93	49	1	1	0.209	0.209	0 NA		0	0.139
SE	9046 CEP57	CEP57	chr11	+	95822498	95822576	95821870	95821978	95827785	95828027	1	2	0	0	4	1	0	2	0	0	98	49	1	1	1	0.2	0 NA		1	-0.6
SE	9047 STAT2	STAT2	chr12	-	56346818	56346955	56346441	56346624	56348528	56348623	12	8	0	0	2	0	2	22	0	0	98	49	1	1	1	0.667 NA		1	1	-0.166
SE	9049 STAT2	STAT2	chr12	-	56350096	56350190	56349588	56349636	56350411	56350432	26	14	3	2	0	0	0	18	0	0	98	49	1	1	0.867	1	1 NA		1	-0.044
SE	9054 CPNE2	CPNE2	chr16	+	57137148	57137282	57134774	57134826	57146084	57146321	11	4	2	1	0	0	1	5	0	1	98	49	1	1	0.846	1	1	1	0.714	0.092
SE	9055 AGAP2	AGAP2	chr12	-	57727680	57727771	57727359	57727582	57727936	57728085	15	2	1	4	0	0	0	3	0	0	98	49	1	1	0.652	1	1 NA		1	-0.116
SE	9056 AGAP2	AGAP2	chr12	-	57727936	57728085	57727359	57727582	57729638	57729767	7	1	0	0	0	0	0	0	1	0	98	49	1	1	1	1 NA		0 NA		1
SE	9058 AGAP2	AGAP2	chr12	-	57729638	57729767	57727359	57727582	57730494	57730614	6	2	2	1	0	0	2	0	0	0	98	49	1	1	0.75	1	1	1 NA		-0.083
SE	9059 AGAP2	AGAP2	chr12	-	57730494	57730614	57729638	57729767	57730790	57730953	13	2	3	1	0	0	2	0	0	0	98	49	1	1	0.867	1	1	1 NA		-0.044
SE	9060 AGAP2	AGAP2	chr12	-	57731808	57731967	57731555	57731642	57732402	57732512	17	7	0	3	0	0	1	1	0	0	98	49	1	1	0.739	1 NA		1	1	-0.131
SE	9061 AGAP2	AGAP2	chr12	-	57732844	57732979	57732402	57732512	57734025	57734173	13	3	0	1	0	0	0	2	0	0	98	49	1	1	0.867	1 NA	NA		1	-0.067
SE	9062 SIPA1	SIPA1	chr11	+	65642249	65642377	65640800	65641600	65642462	65642639	40	21	14	4	1	0	0	14	0	0	98	49	1	1	0.833	0.913	1 NA		1	-0.085
SE	9064 SIPA1	SIPA1	chr11	+	65645853	65645957	65644954	65645129	65646220	65646378	49	27	20	0	0	0	0	19	0	2	98	49	1	1	1	1	1 NA		0.826	0.174
SE	9066 SIPA1	SIPA1	chr11	+	65650400	65650479	65650137	65650192	65650568	65650920	54	31	18	0	0	0	0	9	1	0	98	49	1	1	1	1	1	0	1	0.5
SE	9071 ESRRA	ESRRA	chr11	+	64307167	64307504	64305527	64305736	64313950	64314067	5	3	6	0	0	0	0	3	0	1	98	49	1	1	1	1	1 NA		0.6	0.4
SE	9074 PRPF3	PRPF3	chr1	+	150334934	150335241	150332978	150333199	150338159	150338326	22	7	0	2	0	0	0	6	0	0	98	49	1	1	0.846	1 NA	NA		1	-0.077
SE	9075 PRPF3	PRPF3	chr1	+	150340397	150340477	150338159	150338326	150343308	150343452	9	8	1	1	0	0	0	4	0	0	98	49	1	1	0.818	1	1 NA		1	-0.061
SE	9076 PRPF3	PRPF3	chr1	+	150344433	150344547	150344161	150344261	150346017	150346136	11	1	5	2	0	0	0	3	0	0	98	49	1	1	0.733	1	1 NA		1	-0.089
SE	9078 BBX	BBX	chr3	+	107789786	107789876	107778370	107778519	107791239	107791299	6	0	2	1	0	0	0	0	0	2	98	49	1	1	0.75 NA		1 NA		0	0.875
SE	9087 FBXL3	FBXL3	chr13	-	77021512	77021861	77018599	77018722	77026826	77027196	14	20	1	1	0	0	0	1	0	0	98	49	1	1	0.875	1	1 NA		1	-0.042
SE	9103 GAPVD1	GAPVD1	chr9	+	125295457	125295574	125268935	125268984	125298889	125299106	2	0	0	1	0	0	1	1	0	0	98	49	1	1	0.5 NA	NA		1	1	-0.5
SE	9104 GAPVD1	GAPVD1	chr9	+	125301982	125302826	125298889	125299106	125305062	125305149	28	8	3	1	0	0	0	11	0	0	98	49	1	1	0.933	1	1 NA		1	-0.022
SE	9106 GAPVD1	GAPVD1	chr9	+	125360527	125360725	125359419	125359492	125362605	125365011	13	4	1	0	2	0	0	5	0	2	98	49	1	1	1	0.5	1 NA		0.556	0.277
SE	9107 MAN2C1	MAN2C1	chr15	-	75362641	75362748	75362342	75362453	75364487	75364665	4	2	0	0	0	0	0	4	0	1	98	49	1	1	1	1 NA	NA		0.667	0.333
SE	9110 TPCN1	TPCN1	chr12	+	113284717	113284771	113284580	113284637	113285888	113285961	1	0	0	1	0	0	0	2	0	0	98	49	1	1	0.333 NA	NA	NA		1	-0.667
SE	9114 WDR37	WDR37	chr10	+	1077906	1078003	1072115	1072293	1080010	1080106	10	4	7	0	2	0	0	3	0	0	98	49	1	1	1	0.5	1 NA		1	-0.167
SE	9115 WDR37	WDR37	chr10	+	1103601	1103836	1096169	1096246	1124217	1124352	2	0	0	0	0	0	0	0	0	1	98	49	1	1	1 NA	NA	NA		0	1
SE	9116 WDR37	WDR37	chr10	+	1105125	1105267	1103601	1103836	1124217	1124352	3	1	0	1	0	0	0	4	0	0	98	49	1	1	0.6	1 NA	NA		1	-0.2
SE	9130 DPM1	DPM1	chr20	-	50940864	50940933	50936147	50936262	50942030	50942126	18	4	3	0	0	1	0	3	0	0	98	49	1	1	1	1	0.6 NA		1	-0.133
SE	9131 DPM1	DPM1	chr20	-	50945736	50945762	50942030	50942126	50945846	50945923	3	7	5	0	1	0	0	1	0	0	75	49	1	1	1	0.821	1 NA		1	-0.06
SE	9137 HERC4	HERC4	chr10	-	67990210	67990400	67988662	67988835	67990903	67991015	8	4	3	1	0	0	0	1	0	2	98	49	1	1	0.8	1	1 NA		0.2	0.733
SE	9139 HERC4	HERC4	chr10	-	68044403	68044563	68038092	68038169	68072882	68073186	20	10	0	1	0	0	0	1	0	0	98	49	1	1	0.909	1 NA	NA		1	-0.045
SE	9141 TLR10	TLR10	chr4	-	38775774	38775963	38772238	38775652	38776109	38776426	1	0	0	0	0	3	0	2	0	0	98	49	1	1	1 NA		0 NA		1	-0.5
SE	9150 FBXO18	FBXO18	chr10	+	5903019	5903175	5890202	5890346	5906036	5906632	2	3	0	0	1	0	0	3	0	0	98	49	1	1	1	0.6 NA	NA		1	-0.2
SE	9151 FBXO18	FBXO18	chr10	+	5918341	5918478	5917589	5917676	5921257	5921357	4	6	2	2	0	0	0	1	0	0	98	49	1	1	0.5	1	1 NA		1	-0.167
SE	9161 CHD4	CHD4	chr12	-	6582136	6582281	6581648	6581814	6582614	6582748	33	7	10	1	1	0	0	4	0	1	98	49	1	1	0.943	0.778	1 NA		0.667	0.24
SE	9162 CHD4	CHD4	chr12	-	6583197	6583378	6583026	6583113	6587383	6587559	12	5	5	0	1	0	0	5	0	0	98	49	1	1	1	0.714	1 NA		1	-0.095
SE	9163 CHD4	CHD4	chr12	-	6600925	6601053	6600533	6600669	6601288	6601530	24	21	1	2	0	0	0	7	0	0	98	49	1	1	0.857	1	1 NA		1	-0.048
SE	9166 CELF2	CELF2	chr10	+	11266597	11266677	11257737	11257872	11270665	11270824	30	21	7	13	13	6	0	15	0	5	98	49	1	1	0.536	0.447	0.368 NA		0.6	-0.15
SE	9167 CELF2	CELF2	chr10	+	11266597	11266677	11257737	11257872	11275056	11275120	20	6	1	3	6	0	0	9	0	0	98	49	1	1	0.769	0.333	1 NA		1	-0.299
SE	9168 CELF2	CELF2	chr10	+	11270665	11270824	11257737	11257872	11275056	11275120	104	45	14	3	6	0	0	11	0	0	98	49	1	1	0.945	0.789	1 NA		1	-0.089
SE	9169 CELF2	CELF2	chr10	+	11270665	11270824	11266597	11266677	11275056	11275120	102	47	14	1	0	0	0	12	0	0	98	49	1	1	0.981	1	1 NA		1	-0.006
SE	9170 CELF2	CELF2	chr10	+	11288417	11288552	11275056	11275120	11314138	11314258	120	37	13	1	0	0	2	18	0	0	98	49	1	1	0.984	1	1	1	1	-0.005
SE	9171 CELF2	CELF2	chr10	+	11325835	11325979	11321188	11321386	11328925	11329097	46	30	19	0	1	0	0	9	0	0	98	49	1	1	1	0.937	1 NA		1	-0.021
SE	9172 TPP1	TPP1	chr11	-	6616004	6616074	6615441	6615562	6616314	6616503	16	3	4	0	0	0	0	13	0	1	98	49	1	1	1	1	1 NA		0.867	0.133
SE	9184 TNNT1	TNNT1	chr19	-	55146433	55146466	55145543	55145565	55146680	55146707	1	1	0	0	0	0	0	0	0	1	82	49	1	1	1	1 NA	NA		0	1
SE	9187 NCSTN	NCSTN	chr1	+	160351221	160351372	160350104	160350250	160351695	160351805	41	9	3	1	0	0	0	9	0	0	98	49	1	1	0.953	1	1 NA		1	-0.016
SE	9188 NCSTN	NCSTN	chr1	+	160355654	160355757	160354117	160354290	160355862	160355958	8	4	7	0	0	0	1	4	0	1	98	49	1	1	1	1	1	1	0.667	0.166
SE	9189 NCSTN	NCSTN	chr1	+	160355862	160355958	160354117	160354290	160356259	160356347	17	2	2	1	0	0	0	9	0	1	98	49	1	1	0.895	1	1 NA		0.818	0.147
SE	9190 NCSTN	NCSTN	chr1	+	160356259	160356347	160354117	160354290	160356599	160356754	13	3	5	0	0	0	0	4	0	1	98	49	1	1	1	1	1 NA		0.667	0.333
SE	9191 C2CD3	C2CD3	chr11	-	74122987	74123135	74118227	74118382	74132843	74132972	7	0	0	1	0	0	0	5	0	0	98	49	1	1	0.778 NA	NA	NA		1	-0.222
SE	9192 C2CD3	C2CD3	chr11	-	74161398	74161556	74139604	74139828	74168343	74168613	8	2	0	0	0	0	0	2	0	1	98	49	1	1	1	1 NA	NA		0.5	0.5
SE	9200 ACSL1	ACSL1	chr4	-	184770398	184770476	184768315	184768450	184773080	184773154	230	105	146	1	2	0	1	55	0	1	98	49	1	1	0.991	0.963	1	1	0.965	0.002
SE	9201 ACSL1	ACSL1	chr4	-	184770398	184770476	184768315	184768450	184773662	184773714	85	32	78	1	0	0	1	16	0	1	98	49	1	1	0.977	1	1	1	0.889	0.048
SE	9202 ACSL1	ACSL1	chr4	-	184773080	184773154	184768315	184768450	184773662	184773714	163	95	53	1	0	0	2	38	0	1	98	49	1	1	0.988	1	1	1	0.95	0.021
SE	9204 ACSL1	ACSL1	chr4	-	184773842	184773875	184773662	184773714	184776483	184776662	231	70	108	1	0	0	2	51	0	0	82	49	1	1	0.993	1	1	1	1	-0.002
SE	9206 ACSL1	ACSL1	chr4	-	184780331	184780433	184776883	184776983	184783926	184783991	374	132	114	1	1	0	5	72	0	0	98	49	1	1	0.995	0.985	1	1	1	-0.007
SE	9207 ACSL1	ACSL1	chr4	-	184788378	184788513	184783926	184783991	184788616	184788731	1	3	1	158	76	30	0	0	1	55	98	49	1	1	0.003	0.019	0.016	0	0	0.013
SE	9212 DIS3	DIS3	chr13	-	72781288	72781356	72780845	72781003	72781604	72782128	1	5	1	0	2	0	0	0	0	1	98	49	1	1	1	0.556	1 NA		0	0.852
SE	9214 BPTF	BPTF	chr17	+	67875555	67875744	67874816	67875020	67886127	67886316	4	3	2	4	0	0	0	3	0	0	98	49	1	1	0.333	1	1 NA		1	-0.222
SE	9215 BPTF	BPTF	chr17	+	67875555	67875744	67874816	67875020	67891843	67892034	6	1	0	0	2	0	0	1	0	0	98	49	1	1	1	0.2 NA	NA		1	-0.4
SE	9216 BPTF	BPTF	chr17	+	67886127	67886316	67874816	67875020	67891843	67892034	10	0	0	0	2	0	0	3	0	0	98	49	1	1	1	0 NA	NA		1	-0.5
SE	9217 BPTF	BPTF	chr17	+	67886127	67886316	67875555	67875744	67891843	67892034	6	2	2	2	0	0	0	6	0	1	98	49	1	1	0.6	1	1 NA		0.75	0.117
SE	9218 BPTF	BPTF	chr17	+	67966571	67966656	67964211	67964404	67975771	67975958	14	2	4	0	0	0	1	3	0	1	98	49	1	1	1	1	1	1	0.6	0.2
SE	9221 CTNND1	CTNND1	chr11	+	57791384	57791673	57761761	57762046	57794009	57794081	1	0	0	0	0	0	0	0	0	1	98	49	1	1	1 NA	NA	NA		0	1
SE	9222 CTNND1	CTNND1	chr11	+	57791493	57791673	57761761	57762046	57794009	57794081	1	0	0	0	0	0	0	0	0	1	98	49	1	1	1 NA	NA	NA		0	1
SE	9234 FAM107B	FAM107B	chr10	-	14553321	14553387	14530331	14530515	14604239	14604418	1	4	3	1	0	0	1	0	0	0	98	49	1	1	0.333	1	1	1 NA		-0.222
SE	9237 SPEN	SPEN	chr1	+	15876201	15876678	15872815	15873136	15909320	15909481	17	2	1	0	0	2	0	3	0	0	98	49	1	1	1	1	0.2 NA		1	-0.267
SE	9239 SVIL	SVIL	chr10	-	29512734	29512861	29499115	29499263	29522409	29522635	34	9	16	2	2	0	0	3	0	0	98	49	1	1	0.895	0.692	1 NA		1	-0.138
SE	9248 CDC42SE2	CDC42SE2	chr5	+	131315975	131316144	131264008	131264166	131359208	131359547	17	3	6	0	1	0	0	1	0	0	98	49	1	1	1	0.6	1 NA		1	-0.133
SE	9249 CDC42SE2	CDC42SE2	chr5	+	131359208	131359547	131315975	131316144	131385542	131385644	59	14	16	1	0	0	1	10	0	0	98	49	1	1	0.967	1	1	1	1	-0.011
SE	9252 SEL1L	SEL1L	chr14	-	81492479	81492548	81490387	81490465	81495080	81495137	20	16	10	1	0	0	0	1	0	0	98	49	1	1	0.909	1	1 NA		1	-0.03
SE	9262 SLC38A9	SLC38A9	chr5	-	55649206	55649314	55645788	55645895	55652528	55652723	2	2	0	1	0	0	0	2	0	0	98	49	1	1	0.5	1 NA	NA		1	-0.25
SE	9268 OGDH	OGDH	chr7	+	44707224	44707388	44701542	44701615	44707581	44707736	6	1	4	0	4	0	0	1	0	0	98	49	1	1	1	0.111	1 NA		1	-0.296
SE	9270 PCF11	PCF11	chr11	+	83161326	83161452	83157094	83157631	83163678	83163867	14	20	0	0	0	1	0	3	0	0	98	49	1	1	1	1	0 NA		1	-0.333
SE	9271 PCF11	PCF11	chr11	+	83171817	83171914	83168427	83169995	83177084	83177204	48	9	5	0	0	1	0	9	0	0	98	49	1	1	1	1	0.714 NA		1	-0.095
SE	9275 VIM	VIM	chr10	+	17235845	17235889	17235168	17235389	17236293	17236379	301	80	44	4	0	0	12	171	0	0	93	49	1	1	0.975	1	1	1	1	-0.008
SE	9276 VIM	VIM	chr10	+	17235845	17235889	17235168	17235389	17237229	17237593	146	40	21	1	0	0	0	65	0	0	93	49	1	1	0.987	1	1 NA		1	-0.004
SE	9277 VIM	VIM	chr10	+	17236293	17236379	17235168	17235389	17237229	17237593	218	59	45	1	0	0	7	135	0	0	98	49	1	1	0.991	1	1	1	1	-0.003
SE	9278 VIM	VIM	chr10	+	17236293	17236379	17235845	17235889	17237229	17237593	384	100	70	3	0	0	19	241	0	0	98	49	1	1	0.985	1	1	1	1	-0.005
SE	9280 MGRN1	MGRN1	chr16	+	4677462	4677572	4673497	4673657	4681549	4681776	4	8	0	0	2	0	0	1	0	0	98	49	1	1	1	0.667 NA	NA		1	-0.166
SE	9281 MGRN1	MGRN1	chr16	+	4680031	4680097	4677462	4677572	4681549	4681776	18	8	1	1	3	0	0	3	0	0	98	49	1	1	0.9	0.571	1 NA		1	-0.176
SE	9283 RIPK2	RIPK2	chr8	+	89762828	89762982	89757746	89758233	89765340	89765496	3	1	0	6	2	0	0	1	0	0	98	49	1	1	0.2	0.2 NA	NA		1	-0.8
SE	9284 MBNL1	MBNL1	chr3	+	152299404	152300367	152268039	152269092	152414940	152415111	53	20	2	8	4	0	0	6	0	1	98	49	1	1	0.768	0.714	1 NA		0.75	0.077
SE	9285 MBNL1	MBNL1	chr3	+	152432716	152432920	152414940	152415111	152445281	152445539	101	30	7	2	0	0	1	24	0	1	98	49	1	1	0.962	1	1	1	0.923	0.026
SE	9286 MBNL1	MBNL1	chr3	+	152446703	152446757	152445281	152445539	152447619	152447773	3	0	0	51	23	13	0	0	0	10	98	49	1	1	0.029	0	0 NA		0	0.01
SE	9290 MBNL1	MBNL1	chr3	+	152458129	152458193	152456266	152456361	152459270	152459345	1	0	1	35	7	9	0	0	0	5	98	49	1	1	0.014	0	0.053 NA		0	0.022
SE	9291 SETD3	SETD3	chr14	-	99404224	99404310	99400098	99400259	99405204	99405371	2	3	0	1	0	2	0	2	0	0	98	49	1	1	0.5	1	0 NA		1	-0.5
SE	9292 SETD3	SETD3	chr14	-	99461191	99461340	99459112	99459185	99463485	99463578	21	8	0	2	0	0	0	4	0	1	98	49	1	1	0.84	1 NA	NA		0.667	0.253
SE	9293 WLS	WLS	chr1	-	68153516	68153653	68150187	68150356	68155098	68155260	54	15	2	1	0	0	0	9	0	0	98	49	1	1	0.964	1	1 NA		1	-0.012
SE	9299 BEST1	BEST1	chr11	+	61959891	61960043	61959497	61959578	61962254	61962893	44	13	20	1	0	0	1	3	0	1	98	49	1	1	0.957	1	1	1	0.6	0.186
SE	9300 PIK3CD	PIK3CD	chr1	+	9691466	9691571	9651731	9651802	9710423	9710596	11	6	1	0	3	2	0	2	0	0	98	49	1	1	1	0.5	0.2 NA		1	-0.433
SE	9301 PIK3CD	PIK3CD	chr1	+	9717536	9717626	9716958	9717108	9718693	9718915	93	40	40	1	0	0	1	8	0	0	98	49	1	1	0.979	1	1	1	1	-0.007
SE	9304 PIK3CD	PIK3CD	chr1	+	9723968	9724092	9723124	9723292	9724803	9724936	34	17	14	1	0	0	0	3	0	0	98	49	1	1	0.944	1	1 NA		1	-0.019
SE	9305 PIK3CD	PIK3CD	chr1	+	9724275	9724421	9723124	9723292	9724803	9724936	43	22	17	1	0	0	0	6	0	0	98	49	1	1	0.956	1	1 NA		1	-0.015
SE	9306 PIK3CD	PIK3CD	chr1	+	9724275	9724421	9723968	9724092	9724803	9724936	75	52	22	1	0	0	3	15	0	0	98	49	1	1	0.974	1	1	1	1	-0.009
SE	9309 PLXDC2	PLXDC2	chr10	+	20068169	20068239	20046868	20047015	20143294	20143417	73	48	13	0	0	0	0	21	0	1	98	49	1	1	1	1	1 NA		0.913	0.087
SE	9314 UBAP2L	UBAP2L	chr1	+	154234590	154234738	154228614	154228725	154235195	154235291	3	3	0	4	4	0	0	1	0	0	98	49	1	1	0.273	0.273 NA	NA		1	-0.727
SE	9315 UBAP2L	UBAP2L	chr1	+	154234590	154234759	154228614	154228725	154235195	154235291	7	3	1	4	4	0	0	1	0	0	98	49	1	1	0.467	0.273	1 NA		1	-0.42
SE	9316 UBAP2L	UBAP2L	chr1	+	154234590	154234792	154228614	154228725	154235195	154235291	4	3	1	4	4	0	0	2	0	0	98	49	1	1	0.333	0.273	1 NA		1	-0.465
SE	9318 PIP4K2A	PIP4K2A	chr10	-	22591628	22591781	22573310	22573457	22607926	22608023	20	11	1	0	0	0	3	3	0	1	98	49	1	1	1	1	1	1	0.6	0.2
SE	9339 ENKUR	ENKUR	chr10	-	24999400	24999546	24995645	24995869	25015859	25016156	0	0	0	1	0	0	0	1	0	0	98	49	1	1	0 NA	NA	NA		1	-1
SE	9340 FAM32A	FAM32A	chr19	+	16190519	16190573	16185623	16185765	16190886	16192046	13	4	6	5	0	1	0	2	0	0	98	49	1	1	0.565	1	0.75 NA		1	-0.228
SE	9342 RBM47	RBM47	chr4	-	40436440	40436647	40432650	40432862	40437770	40438924	31	16	8	3	2	0	0	6	0	0	98	49	1	1	0.838	0.8	1 NA		1	-0.121
SE	9343 RBM47	RBM47	chr4	-	40437770	40438924	40432650	40432862	40466576	40466699	29	10	8	0	0	0	2	9	0	1	98	49	1	1	1	1	1	1	0.818	0.091
SE	9347 YME1L1	YME1L1	chr10	-	27147497	27147668	27145427	27145590	27148905	27149040	3	0	0	7	1	2	0	0	0	2	98	49	1	1	0.176	0	0 NA		0	0.059
SE	9351 LGALS8	LGALS8	chr1	+	236542914	236543040	236542760	236542787	236543559	236543648	7	7	0	2	1	0	0	0	0	1	98	49	1	1	0.636	0.778 NA	NA		0	0.707
SE	9354 LMTK2	LMTK2	chr7	+	98137314	98137442	98106884	98107280	98141396	98141541	18	29	15	1	0	0	1	0	0	0	98	49	1	1	0.9	1	1	1 NA		-0.033
SE	9355 LMTK2	LMTK2	chr7	+	98185050	98185135	98171540	98171674	98186876	98186998	8	4	4	0	2	0	0	5	0	0	98	49	1	1	1	0.5	1 NA		1	-0.167
SE	9356 LMTK2	LMTK2	chr7	+	98186876	98186998	98171540	98171674	98190727	98190877	5	4	0	0	2	0	0	2	0	0	98	49	1	1	1	0.5 NA	NA		1	-0.25
SE	9357 LMTK2	LMTK2	chr7	+	98203943	98204186	98203573	98203706	98205463	98209632	13	8	7	0	1	0	0	2	0	0	98	49	1	1	1	0.8	1 NA		1	-0.067
SE	9360 PPP4R1L	PPP4R1L	chr20	-	58248114	58248273	58247321	58247506	58250886	58250952	12	4	0	1	0	0	0	3	0	0	98	49	1	1	0.857	1 NA	NA		1	-0.072
SE	9361 PPP4R1L	PPP4R1L	chr20	-	58250886	58250952	58247321	58247506	58251756	58251864	11	2	0	1	0	0	0	4	0	0	98	49	1	1	0.846	1 NA	NA		1	-0.077
SE	9372 NFYC	NFYC	chr1	+	40766917	40766974	40766595	40766703	40769355	40769415	0	0	1	6	2	0	0	0	0	1	98	49	1	1	0	0	1 NA		0	0.333
SE	9377 MAP3K8	MAP3K8	chr10	+	30437175	30437406	30434020	30434378	30438915	30439274	11	5	0	0	4	0	0	6	0	1	98	49	1	1	1	0.385 NA	NA		0.75	-0.057
SE	9383 EPC1	EPC1	chr10	-	32292495	32292644	32291162	32291322	32292987	32293194	23	11	6	2	0	0	0	3	0	0	98	49	1	1	0.852	1	1 NA		1	-0.049
SE	9385 AMICA1	AMICA1	chr11	-	118212406	118212561	118210486	118210712	118224940	118225094	168	114	70	1	0	0	4	27	0	0	98	49	1	1	0.988	1	1	1	1	-0.004
SE	9395 RTN3	RTN3	chr11	+	63718980	63721032	63681449	63681778	63749990	63750198	0	0	1	12	13	15	0	0	0	3	98	49	1	1	0	0	0.032 NA		0	0.011
SE	9401 TXNRD1	TXNRD1	chr12	+	104321090	104321316	104319469	104319585	104325336	104325429	21	24	5	2	0	0	3	2	0	0	98	49	1	1	0.84	1	1	1	1	-0.053
SE	9402 TXNRD1	TXNRD1	chr12	+	104326346	104326423	104325336	104325429	104327514	104327671	37	22	11	2	0	0	0	5	0	0	98	49	1	1	0.902	1	1 NA		1	-0.033
SE	9404 KIAA0232	KIAA0232	chr4	+	6804522	6804606	6782731	6782841	6824184	6824684	1	0	0	8	4	0	0	0	0	1	98	49	1	1	0.059	0 NA	NA		0	0.029
SE	9407 LDLR	LDLR	chr19	+	11105219	11105600	11102663	11102786	11106564	11106687	1	1	0	0	0	0	0	0	0	1	98	49	1	1	1	1 NA	NA		0	1
SE	9410 CCNY	CCNY	chr10	+	35394815	35394878	35336873	35337207	35483403	35483478	1	0	0	13	1	8	0	0	2	1	98	49	1	1	0.037	0	0	0	0	0.012
SE	9412 CCNY	CCNY	chr10	+	35529972	35530030	35525963	35525999	35530123	35530243	41	24	19	0	0	2	0	8	0	0	98	49	1	1	1	1	0.826 NA		1	-0.058
SE	9416 ZNF33A	ZNF33A	chr10	+	38025347	38025411	38017290	38017386	38054374	38060067	1	0	1	9	5	3	0	0	0	1	98	49	1	1	0.053	0	0.143 NA		0	0.065
SE	9417 ZNF33A	ZNF33A	chr10	+	38034277	38034400	38017290	38017386	38054374	38060067	0	0	1	9	5	3	0	0	0	1	98	49	1	1	0	0	0.143 NA		0	0.048
SE	9419 ZNF33A	ZNF33A	chr10	+	38034325	38034400	38017290	38017386	38054374	38060067	0	1	1	9	5	3	0	0	0	1	98	49	1	1	0	0.091	0.143 NA		0	0.078
SE	9420 ZNF33A	ZNF33A	chr10	+	38039412	38039492	38017290	38017386	38054374	38060067	0	0	0	9	5	3	0	2	0	1	98	49	1	1	0	0	0 NA		0.5	-0.5
SE	9430 SCYL1	SCYL1	chr11	+	65526123	65526350	65525920	65526043	65526782	65526873	14	4	6	0	0	1	1	1	0	0	98	49	1	1	1	1	0.75	1	1	-0.083

SE	9432	TATDN2	TATDN2	chr3	+	10249194	10249614	10248492	10249067	10260136	10260670	8	2	2	0	0	0	3	6	0	1	98	49	1	1	1	1	1	1	0.75	0.125
SE	9433	CUL5	CUL5	chr11	+	108049889	108050066	108046269	108046369	108052659	108052801	3	0	0	0	4	0	0	1	0	0	98	49	1	1	1	0	NA	NA	1	-0.5
SE	9435	ACBD5	ACBD5	chr10	-	27235091	27235212	27231747	27231820	27240318	27240484	15	3	4	1	0	0	0	2	0	0	98	49	1	1	0.882	1	1	NA	1	-0.039
SE	9436	TFRC	TFRC	chr3	-	196071395	196071498	196069454	196069568	196072002	196072152	9	4	0	0	2	0	0	3	0	0	98	49	1	1	1	0.5	NA	NA	1	-0.25
SE	9446	RAB1B	RAB1B	chr11	+	66272364	66272460	66272156	66272252	66275803	66275935	34	25	22	1	0	0	2	11	0	0	98	49	1	1	0.944	1	1	1	1	-0.019
SE	9449	ANKRD27	ANKRD27	chr19	-	32602014	32602126	32599971	32600050	32604262	32604424	2	0	0	0	0	2	0	1	0	0	98	49	1	1	1	NA	0	NA	1	-0.5
SE	9450	ANKRD27	ANKRD27	chr19	-	32646458	32646615	32644324	32644479	32649681	32649792	4	3	0	0	0	0	0	1	0	1	98	49	1	1	1	1	NA	NA	0.333	0.667
SE	9451	ANKRD27	ANKRD27	chr19	-	32649681	32649792	32644324	32644479	32658913	32659045	3	1	0	0	0	0	0	2	0	2	98	49	1	1	1	1	NA	NA	0.333	0.667
SE	9454	ZNF467	ZNF467	chr7	-	149769089	149769200	149764363	149766239	149770439	149770556	19	3	14	1	0	0	1	2	0	0	98	49	1	1	0.905	1	1	1	1	-0.032
SE	9455	ZNF467	ZNF467	chr7	-	149769089	149769200	149764363	149766239	149770998	149771074	9	3	5	1	0	0	2	1	0	0	98	49	1	1	0.818	1	1	1	1	-0.061
SE	9459	NCOA4	NCOA4	chr10	-	46009123	46009212	46005087	46006597	46009410	46009551	1	0	0	162	77	49	0	0	2	9	98	49	1	1	0.003	0	0	0	0	0.001
SE	9460	NCOA4	NCOA4	chr10	-	46016539	46016694	46015125	46015266	46030525	46030714	102	33	29	2	1	0	0	5	0	0	98	49	1	1	0.962	0.943	1	NA	1	-0.032
SE	9464	LITAF	LITAF	chr16	-	11551706	11551804	11547721	11549745	11553532	11553689	3	0	2	92	51	66	0	0	2	12	98	49	1	1	0.016	0	0.015	0	0	0.01
SE	9467	FAM131A	FAM131A	chr3	+	184337310	184337371	184335928	184336123	184341723	184341817	0	2	1	0	0	0	0	1	0	1	98	49	1	1	NA	1	1	NA	0.333	0.667
SE	9487	PDCD4	PDCD4	chr10	+	110876669	110876753	110875965	110876070	110881232	110881535	5	3	1	2	0	1	0	0	0	1	98	49	1	1	0.556	1	0.333	NA	0	0.63
SE	9488	PDCD4	PDCD4	chr10	+	110887664	110887886	110885252	110885366	110889532	110889630	48	22	9	0	1	0	1	4	0	0	98	49	1	1	1	0.917	1	1	1	-0.028
SE	9489	PDCD4	PDCD4	chr10	+	110890555	110890670	110889532	110889630	110894090	110894198	20	21	6	0	1	0	0	4	0	0	98	49	1	1	1	0.913	1	NA	1	-0.029
SE	9490	PDCD4	PDCD4	chr10	+	110894411	110894522	110894090	110894198	110895947	110896087	33	14	7	3	0	0	0	6	0	0	98	49	1	1	0.846	1	1	NA	1	-0.051
SE	9494	EMC4	EMC4	chr15	+	34225535	34225650	34224996	34225200	34227692	34227846	8	5	6	1	0	0	0	4	0	0	98	49	1	1	0.8	1	1	NA	1	-0.067
SE	9496	WDFY4	WDFY4	chr10	+	48727469	48727659	48725880	48726070	48729431	48729589	37	6	0	1	0	0	0	5	0	0	98	49	1	1	0.949	1	NA	NA	1	-0.026
SE	9497	WDFY4	WDFY4	chr10	+	48760346	48760440	48742967	48743548	48774457	48774672	28	13	13	0	0	0	0	11	0	1	98	49	1	1	1	1	1	NA	0.846	0.154
SE	9498	WDFY4	WDFY4	chr10	+	48817244	48817409	48813956	48814082	48820233	48820437	21	1	5	1	0	0	1	7	0	0	98	49	1	1	0.913	1	1	1	1	-0.029
SE	9499	WDFY4	WDFY4	chr10	+	48826670	48826909	48822379	48822537	48828777	48828896	16	1	1	4	0	0	0	5	0	0	98	49	1	1	0.667	1	1	NA	1	-0.111
SE	9503	C10orf128	C10orf128	chr10	-	49165765	49165796	49161598	49161622	49166868	49167009	0	7	7	0	0	1	0	1	0	1	80	49	1	1	NA	1	0.811	NA	0.38	0.525
SE	9508	BNIP2	BNIP2	chr15	-	59668891	59668990	59662862	59664120	59669275	59669362	72	49	20	1	0	0	1	13	0	0	98	49	1	1	0.973	1	1	1	1	-0.009
SE	9509	BNIP2	BNIP2	chr15	-	59669275	59669362	59668891	59668990	59671182	59671314	55	35	13	1	2	0	0	14	0	0	98	49	1	1	0.965	0.897	1	NA	1	-0.046
SE	9510	BNIP2	BNIP2	chr15	-	59679591	59679768	59677910	59678087	59680240	59680308	44	20	17	2	0	0	0	15	0	0	98	49	1	1	0.917	1	1	NA	1	-0.028
SE	9515	TBRG1	TBRG1	chr11	+	124626472	124626609	124625670	124625903	124626903	124627050	5	0	0	0	0	1	0	1	0	0	98	49	1	1	1	NA	0	NA	1	-0.5
SE	9519	XRN1	XRN1	chr3	-	142412543	142412663	142404906	142405076	142414134	142414291	51	17	2	1	0	0	0	14	0	0	98	49	1	1	0.962	1	1	NA	1	-0.013
SE	9520	XRN1	XRN1	chr3	-	142414134	142414291	142412543	142412663	142417139	142417229	55	17	3	0	3	0	0	11	0	0	98	49	1	1	1	0.739	1	NA	1	-0.087
SE	9521	XRN1	XRN1	chr3	-	142426743	142426841	142425428	142425538	142432660	142432893	36	11	1	0	2	0	0	8	0	1	98	49	1	1	1	0.733	1	NA	0.8	0.111
SE	9526	LAMTOR2	LAMTOR2	chr1	+	156057977	156058067	156055262	156055425	156058314	156058510	6	1	5	0	0	1	0	3	0	0	98	49	1	1	1	1	0.714	NA	1	-0.095
SE	9537	GMPR2	GMPR2	chr14	+	24233218	24233340	24232421	24232847	24233478	24233593	22	9	4	0	5	0	0	4	0	0	98	49	1	1	1	0.474	1	NA	1	-0.175
SE	9539	GMPR2	GMPR2	chr14	+	24233478	24233598	24232421	24232847	24235736	24235820	8	9	2	0	1	0	0	1	0	0	98	49	1	1	1	0.818	1	NA	1	-0.061
SE	9540	GMPR2	GMPR2	chr14	+	24234106	24234241	24233478	24233598	24235736	24235820	1	0	0	8	4	2	0	0	0	1	98	49	1	1	0.059	0	0	NA	0	0.02
SE	9541	GMPR2	GMPR2	chr14	+	24235736	24235820	24233478	24233598	24235966	24236140	13	11	5	1	0	0	0	1	0	0	98	49	1	1	0.867	1	1	NA	1	-0.044
SE	9542	GMPR2	GMPR2	chr14	+	24237041	24237152	24235966	24236140	24237244	24237351	6	3	3	0	2	0	0	3	0	0	98	49	1	1	1	0.429	1	NA	1	-0.19
SE	9543	GMPR2	GMPR2	chr14	+	24237070	24237152	24235966	24236140	24237244	24237351	19	8	11	0	2	0	0	4	0	0	98	49	1	1	1	0.667	1	NA	1	-0.111
SE	9545	FURIN	FURIN	chr15	+	90877526	90877615	90877134	90877211	90878131	90878304	29	17	18	0	0	0	1	6	0	1	98	49	1	1	1	1	1	1	0.75	0.125
SE	9546	UBE3B	UBE3B	chr12	+	109530546	109530658	109529889	109530072	109533465	109533558	5	1	0	1	0	0	0	1	0	2	98	49	1	1	0.714	1	NA	NA	0.2	0.657
SE	9550	ELP2	ELP2	chr18	+	36139418	36139613	36138794	36138872	36141136	36141201	0	6	0	3	0	0	0	0	0	2	98	49	1	1	0	1	NA	NA	0	0.5
SE	9553	RBSN	RBSN	chr3	-	15082366	15082608	15080731	15080802	15084734	15084896	2	0	0	0	1	0	0	3	0	0	98	49	1	1	1	0	NA	NA	1	-0.5
SE	9555	HNRNPU	HNRNPU	chr1	-	244856727	244856856	244856456	244856625	244857597	244857717	116	47	31	1	3	0	0	23	0	0	98	49	1	1	0.983	0.887	1	NA	1	-0.043
SE	9582	SH3GLB1	SH3GLB1	chr1	+	86715723	86715865	86704569	86704971	86719506	86719635	55	24	4	1	1	0	0	15	0	0	98	49	1	1	0.965	0.923	1	NA	1	-0.037
SE	9583	SH3GLB1	SH3GLB1	chr1	+	86728402	86728489	86724312	86724405	86734601	86734691	1	1	4	14	9	9	0	1	0	7	98	49	1	1	0.034	0.053	0.182	NA	0.067	0.023
SE	9593	ADPGK	ADPGK	chr15	-	72756250	72756447	72755555	72755654	72760406	72760527	17	4	10	5	0	0	0	5	0	0	98	49	1	1	0.63	1	1	NA	1	-0.123
SE	9594	ADPGK	ADPGK	chr15	-	72774871	72775097	72771782	72771845	72783245	72783785	37	7	6	2	2	1	0	5	0	2	98	49	1	1	0.902	0.636	0.75	NA	0.556	0.207
SE	9606	GLT1D1	GLT1D1	chr12	+	128888638	128888744	128875913	128876062	128899235	128899287	76	42	18	2	0	0	6	18	0	1	98	49	1	1	0.95	1	1	1	0.9	0.033
SE	9607	GLT1D1	GLT1D1	chr12	+	128899235	128899287	128888638	128888744	128945325	128945369	48	28	7	0	1	0	5	8	0	0	98	49	1	1	1	0.933	1	1	1	-0.022
SE	9609	SUPV3L1	SUPV3L1	chr10	+	69189266	69189435	69187641	69187756	69191654	69191766	3	1	0	0	0	0	0	0	1	0	98	49	1	1	1	1	NA	0	NA	1
SE	9611	CTNNBL1	CTNNBL1	chr20	+	37842338	37842419	37840101	37840199	37859898	37860036	3	0	0	0	0	0	0	2	0	1	98	49	1	1	1	NA	NA	NA	0.5	0.5
SE	9614	RMND5A	RMND5A	chr2	+	86771557	86771712	86770022	86770125	86773347	86778041	39	30	22	0	0	1	0	1	0	0	98	49	1	1	1	1	0.917	NA	1	-0.028
SE	9617	TMUB2	TMUB2	chr17	+	44189021	44189588	44186985	44187109	44190500	44191731	10	0	1	0	0	0	0	5	0	1	98	49	1	1	1	NA	1	NA	0.714	0.286
SE	9619	GSK3B	GSK3B	chr3	-	119866589	119866628	119863418	119863605	119876412	119876508	0	5	0	10	3	1	0	0	0	2	88	49	1	1	0	0.481	0	NA	0	0.16
SE	9628	CCBL2	CCBL2	chr1	-	88988251	88988351	88969408	88969467	88992584	88992960	7	5	1	5	1	0	0	1	0	0	98	49	1	1	0.412	0.714	1	NA	1	-0.291
SE	9634	ARIH1	ARIH1	chr15	+	72563393	72563500	72561482	72561549	72566562	72566605	19	11	1	2	2	0	0	1	0	0	98	49	1	1	0.826	0.733	1	NA	1	-0.147
SE	9636	ITGA5	ITGA5	chr12	-	54401584	54401665	54401372	54401478	54401775	54401855	28	26	11	1	0	0	0	14	0	0	98	49	1	1	0.933	1	1	NA	1	-0.022
SE	9637	ITGA5	ITGA5	chr12	-	54403624	54403779	54403186	54403324	54403910	54403966	30	11	14	1	0	0	0	12	0	0	98	49	1	1	0.937	1	1	NA	1	-0.021
SE	9638	ITGA5	ITGA5	chr12	-	54404144	54404246	54403910	54403966	54404429	54404475	51	22	7	0	1	0	7	15	0	0	98	49	1	1	1	0.917	1	1	1	-0.028
SE	9639	ITGA5	ITGA5	chr12	-	54409484	54409597	54409231	54409352	54411833	54411964	54	32	16	0	1	0	2	8	0	0	98	49	1	1	1	0.941	1	1	1	-0.02
SE	9640	ITGA5	ITGA5	chr12	-	54411833	54411964	54409484	54409597	54418980	54419266	40	22	10	0	1	0	3	6	0	0	98	49	1	1	1	0.917	1	1	1	-0.028
SE	9641	C10orf54	C10orf54	chr10	-	71755358	71755466	71752974	71753002	71760867	71760924	126	101	78	0	0	0	3	48	0	1	98	49	1	1	1	1	1	1	0.96	0.02
SE	9642	C10orf54	C10orf54	chr10	-	71761597	71762026	71760867	71760924	71773357	71773580	131	70	76	1	0	0	4	72	0	0	98	49	1	1	0.985	1	1	1	1	-0.005
SE	9643	MCTP1	MCTP1	chr5	-	94799012	94799132	94779109	94779163	94868332	94868452	16	7	5	2	0	0	0	1	0	1	98	49	1	1	0.8	1	1	NA	0.333	0.6
SE	9644	MCTP1	MCTP1	chr5	-	94870871	94870973	94870416	94870491	94871314	94871417	12	13	1	1	0	0	0	2	0	0	98	49	1	1	0.857	1	1	NA	1	-0.048
SE	9646	MCTP1	MCTP1	chr5	-	94917895	94917973	94912805	94912976	94940083	94940195	8	15	2	0	1	0	0	3	0	2	98	49	1	1	1	0.882	1	NA	0.429	0.532
SE	9647	MCTP1	MCTP1	chr5	-	94923961	94924021	94912805	94912976	94931952	94931991	18	7	5	1	0	3	0	3	0	0	98	49	1	1	0.9	1	0.455	NA	1	-0.215
SE	9648	MCTP1	MCTP1	chr5	-	94923961	94924021	94912805	94912976	94940083	94940195	13	3	1	0	1	0	0	1	0	2	98	49	1	1	1	0.6	1	NA	0.2	0.667
SE	9649	MCTP1	MCTP1	chr5	-	94923961	94924021	94917895	94917973	94940083	94940195	9	13	13	0	1	0	2	1	0	0	98	49	1	1	1	0.867	1	1	1	-0.044
SE	9650	MCTP1	MCTP1	chr5	-	94931952	94931991	94912805	94912976	94940083	94940195	25	9	6	0	1	0	0	6	0	2	88	49	1	1	1	0.834	1	NA	0.626	0.319
SE	9651	MCTP1	MCTP1	chr5	-	94931952	94931991	94923961	94924021	94940083	94940195	29	13	7	0	0	0	0	9	0	1	88	49	1	1	1	1	1	NA	0.834	0.166
SE	9679	SLC3A2	SLC3A2	chr11	+	62883988	62884112	62882907	62882999	62884456	62884525	2	0	0	11	4	6	0	1	0	4	98	49	1	1	0.083	0	0	NA	0.111	-0.083
SE	9689	MTHFS	MTHFS	chr15	-	79889092	79889354	79843546	79845442	79896871	79897049	13	8	2	1	0	0	0	3	0	0	98	49	1	1	0.867	1	1	NA	1	-0.044
SE	9690	MTHFS	MTHFS	chr15	-	79889092	79889354	79843546	79845442	79897160	79897285	9	7	0	1	0	0	0	2	0	0	98	49	1	1	0.818	1	NA	NA	1	-0.091
SE	9691	PSMG4	PSMG4	chr6	+	3264208	3264325	3263683	3263759	3267590	3268066	0	2	0	1	0	0	0	0	0	1	98	49	1	1	0	1	NA	NA	0	0.5
SE	9703	XPNPEP1	XPNPEP1	chr10	-	109893011	109893075	109891721	109891826	109907690	109907815	4	0	0	1	0	0	0	1	0	0	98	49	1	1	0.667	NA	NA	NA	1	-0.333
SE	9705	TSPAN14	TSPAN14	chr10	+	80509300	80509471	80507227	80507374	80512143	80512269	20	10	13	1	0	0	0	2	0	0	98	49	1	1	0.909	1	1	NA	1	-0.03
SE	9713	TRPC4AP	TRPC4AP	chr20	-	35013007	35013066	35010186	35010288	35016007	35016139	40	11	5	0	1	0	0	10	0	0	98	49	1	1	1	0.846	1	NA	1	-0.051
SE	9714	TRPC4AP	TRPC4AP	chr20	-	35021189	35021332	35016007	35016139	35035122	35035308	19	13	4	4	1	0	0	4	0	0	98	49	1	1	0.704	0.867	1	NA	1	-0.143
SE	9715	TRPC4AP	TRPC4AP	chr20	-	35021189	35021356	35016007	35016139	35035122	35035308	22	11	3	4	1	0	0	4	0	0	98	49	1	1	0.733	0.846	1	NA	1	-0.14
SE	9716	TRPC4AP	TRPC4AP	chr20	-	35069295	35069412	35057513	35057571	35078045	35078174	52	29	4	1	1	0	1	10	0	0	98	49	1	1	0.963	0.935	1	1	1	-0.034
SE	9717	NXF1	NXF1	chr11	-	62796286	62796345	62796065	62796181	62796459	62796567	20	6	6	0	2	0	0	1	0	0	98	49	1	1	1	0.6	1	NA	1	-0.133
SE	9718	NXF1	NXF1	chr11	-	62801093	62801201	62798538	62800486	62801328	62801417	48	17	8	1	0	0	4	11	0	0	98	49	1	1	0.96	1	1	1	1	-0.013
SE	9719	NXF1	NXF1	chr11	-	62803418	62803572	62802176	62802260	62803791	62803978	25	23	4	0	2	0	0	9	0	0	98	49	1	1	1	0.852	1	NA	1	-0.049
SE	9722	PNPLA6	PNPLA6	chr19	+	7536190	7536273	7535702	7536020	7536448	7536546	7	9	0	1	0	0	4	6	0	0	98	49	1	1	0.778	1	NA	1	1	-0.111
SE	9724	PNPLA6	PNPLA6	chr19	+	7555248	7555367	7554892	7555075	7555606	7555763	6	2	0	0	3	0	0	1	0	0	98	49	1	1	1	0.25	NA	NA	1	-0.375
SE	9725	PNPLA6	PNPLA6	chr19	+	7561013	7561110	7560647	7560764	7561207	7561317	14	6	6	0	1	0	0	3	0	0	98	49	1	1	1	0.75	1	NA	1	-0.083
SE	9728	DPYD	DPYD	chr1	-	97549559	97549744	97515725	97515941	97573759	97573970	166	35	16	3	0	0	4	25	0	1	98	49	1	1	0.965	1	1	1	0.926	0.025
SE	9729	DPYD	DPYD	chr1	-	97593217	97593387	97573759	97573970	97595058	97595166	125	17	22	1	0	0	0	31	0	0	98	49	1	1	0.984	1	1	NA	1	-0.005
SE	9730	DPYD	DPYD	chr1	-	97828113	97828196	97740391	97740479	97883263	97883374	260	75	28	1	0	0	9	88	0	0	98	49	1	1	0.992	1	1	1	1	-0.003
SE	9731	CLEC12B	CLEC12B	chr12	+	10015251	10015406	10014522	10014741	10015611	10015727	0	0	0	0	2	0	0	2	0	0	98	49	1	1	NA	0	NA	NA	1	-1
SE	9732	AHCYL1	AHCYL1	chr1	+	110020730	110020851	110019547	110019626	110021673	110023742	1	0	1	1	1	0	0	4	0	0	98	49	1	1	0.333	0	1	NA	1	-0.556
SE	9737	CRBN	CRBN	chr3	-	3153959	3154075	3153423	3153488	3154746	3154831	9	5	5	1	0	0	0	7	0	0	98	49	1	1	0.818	1	1	NA	1	-0.061
SE	9738	CRBN	CRBN	chr3	-	3167633	3167793	3156218	3156281	3172775	3172925	15	3	7	0	1	0	0	1	0	0	98	49	1	1	1	0.6	1	NA	1	-0.133
SE	9739	VPS53	VPS53	chr17	-	586269	586364	562502	562745	601794	601896	9	1	0	2	0	0	0	1	0	0	98	49	1	1	0.692	1	NA	NA	1	-0.154
SE	9743	IGHMBP2	IGHMBP2	chr11	+	68935298	68935422	68934463	68934558	68936236	68937091	3	0	0	0	2	0	1	1	0	0	98	49	1	1	1	0	NA	1	1	-0.5
SE	9745	PRKACA	PRKACA	chr19	-	14093627	14093792	14091694	14093237	14097360	14097483	30	3	3	3	2	0	0	11	0	0	98	49	1	1	0.833	0.429	1	NA	1	-0.246
SE	9751	OSM	OSM	chr22	-	30265001	30265144	30262829	30264464	30266765	30266840	27	17	12	0	1	0	0	6	0	0	98	49	1	1	1	0.895	1	NA	1	-0.035
SE	9753	RELT	RELT	chr11	+	73391143	73391223	73390754	73390921	73392210	73392468	20	6	14	1	3	0	0	2	0	0	98	49	1	1	0.909	0.5	1	NA	1	-0.197
SE	9754	RELT	RELT	chr11	+	73391143	73391223	73390754	73390921	73393836	73393917	9	6	8	2	0	0	0	3	0	0	98	49	1	1	0.692	1	1	NA	1	-0.103
SE	9755	RELT	RELT	chr11	+	73392210	73392468	73390754	73390921	73393836	73393917	23	10	6	2	0	0	0	3	0	0	98	49	1	1	0.852	1	1	NA	1	-0.049
SE	9756	RELT	RELT	chr11	+	73392210	73392468	73391143	73391223	73393836	73393917	33	7	12	0	0	0	0	3	0	1	98	49	1	1	1	1	1	NA	0.6	0.4
SE	9757	FAS	FAS	chr10	+	89007699	89007837	89003028	89003194	89010538	89010600	15	8	7	0	0	0	0	1	0	2	98	49	1	1	1	1	1	NA	0.2	0.8
SE	9758	FAS	FAS	chr10	+	89008888	89008997	89007699	89007837	89010538	89010600	34	16	6	1	0	0	1	6	0	0	98	49	1	1	0.944	1	1	1	1	-0.019
SE	9759	FAS	FAS	chr10	+	89010752	89010815	89010538	89010600	89011998	89012081	20	13	3	8	15	1	0	2	0	2	98	49	1	1	0.556	0.302	0.6	NA	0.333	0.153
SE	9763	IFIT1	IFIT1	chr10	+	89399889	89400003	89392545	89392717	89402280	89406487	5	2	0	1	6	0	0	11	0	5	98	49	1	1	0.714	0.143	NA	NA	0.524	-0.096
SE	9764	IFIT1	IFIT1	chr10	+	89399894	89400003	89392545	89392717	89402280	89406487	3	2	0	1	6	0	1	12	0	5	98	49	1	1	0.6	0.143	NA	1	0.545	-0.401
SE	9773	E2F4	E2F4	chr16	+	67194397	67194459	67192760	67192870	67194685	67194980	8	4	0	0	0	1	0	2	0	0	98	49	1	1	1	1	0	NA	1	-0.333
SE	9775	NADK2	NADK2	chr5	-	36219595	36219679	36217747	36217884	36225541	36225623	3	4	0	0	2	0	0	1	0	0	98	49	1	1	1	0.5	NA	NA	1	-0.25
SE	9776	PISD	PISD	chr22	-	31620552	31620713	31618490	31619836	31620995	31621142	26	34	9	1	0	0	0	8	0	0	98	49	1	1	0.929	1	1	NA	1	-0.024
SE	9777	PISD	PISD	chr22	-	31621333	31621472	31620995	31621142	31621648	31621885	50	25	20	0	2	0	0	12	0	0	98	49	1	1	1	0.862	1	NA	1	-0.046
SE	9778	PISD	PISD	chr22	-	31623683	31623849	31621648	31621885	31625762	31626016	23	7	12	1	0	0	0	6	0	0	98	49	1	1	0.92	1	1	NA	1	-0.027
SE	9779	ACSL5	ACSL5	chr10	+	112411455	112411529	112410583	112410635	112411901	112411979	5	1	0	1	0	0	0	1	0	0	98	49	1	1	0.714	1	NA	NA	1	-0.143
SE	9781	TNKS2	TNKS2	chr10	+	91827016	91827203	91822295	91822362	91828284	91828406	24	12	5	2	0	0	1	6	0	0	98	49	1	1	0.857	1	1	1	1	-0.048
SE	9782	BTAF1	BTAF1	chr10	+	91953736	91953873	91951402	91951566	91956527	91956657	30	3	0	1	0	0	1	5	0	1	98	49	1	1	0.937	1	NA	1	0.714	0.112
SE	9783	BTAF1	BTAF1	chr10	+	91989153	91989580	91984200	91984404	91992118	91992309	19	2	2	0	1	0	0	2	0	0	98	49	1	1	1	0.5	1	NA	1	-0.167
SE	9784	BTAF1	BTAF1	chr10	+	92027129	92027300	92026591	92026751	92028789	92030323	19	1	0	0	0	0	0	4	0	3	98	49	1	1	1	1	NA	NA	0.4	0.6
SE	9787	PIAS1	PIAS1	chr15	+	68145815	68145906	68142289	68142337	68146565	68146700	52	36	14	2	0	0	0	8	0	0	98	49	1	1	0.929	1	1	NA	1	-0.024
SE	9788	PIAS1	PIAS1	chr15	+	68173731	68173892	68164730	68164804	68175636	68175767	16	9	12	0	2	0	0	7	0	0	98	49	1	1	1	0.692	1	NA	1	-0.103
SE	9789	ARFIP1	ARFIP1	chr4	+	152790758	152790875	152779936	152780091	152829624	152829726	3	0	0	6	1	0	0	0	0	1	98	49	1	1	0.2	0	NA	NA	0	0.1
SE	9790	ARFIP1	ARFIP1	chr4	+	152790758	152790875	152779936	152780226	152829624	152829726	3	0	0	10	0	0	0	2	0	0	98	49	1	1	0.13	NA	NA	NA	1	-0.87
SE	9791	ARFIP1	ARFIP1	chr4	+	152870752	152870848	152863605	152863714	152872451	152872564	22	7	2	9	4	1	0	3	0	0	98	49	1	1	0.55	0.467	0.5	NA	1	-0.494
SE	9792	ARFIP1	ARFIP1	chr4	+	152882722	152882880	152880962	152881184	152888132	152888307	10	4	5	1	0	0	0	1	0	0	98	49	1	1	0.833	1	1	NA	1	-0.056
SE	9798	RNH1	RNH1	chr11	-	504823	504996	502061	502181	507112	507283	1	2	0	0	1	0	0	2	0	0	98	49	1	1	1	0.5	NA	NA	1	-0.25
SE	9830	ARL8A	ARL8A	chr1	-	202135458	202135526	202135149	202135220	202135706	202135800	18	18	25	0	0	0	0	11	0	1	98	49	1	1	1	1	1	NA	0.846	0.154
SE	9836	UBL3	UBL3	chr13	-	29777154	29777263	29772111	29772198	29849511	29850683	22	18	2	1	0	0	0	6	0	0	98	49	1	1	0.917	1	1	NA	1	-0.028
SE	9837	ZCCHC11	ZCCHC11	chr1	-	52458335	52458449	52446589	52446667	52461133	52461223	5	0	0	2	1	0	0	0	0	1	98	49	1	1	0.556	0	NA	NA	0	0.278
SE	9849	MYH9	MYH9	chr22	-	36303994	36304155	36302567	36302676	36305032	36305102	184	98	47	0	1	0	6	33	0	0	98	49	1	1	1	0.98	1	1	1	-0.007
SE	9854	NADK	NADK	chr1	-	1753566	1753649	1751231	1753060	1754050	1754208	75	38	62	1	0	0	9	28	0	0	98	49	1	1	0.974	1	1	1	1	-0.009
SE	9856	NADK	NADK	chr1	-	1754543	1754698	1754283	1754383	1756257	1756343	98	57	38	0	2	0	4	24	0	0	98	49	1	1	1	0.934	1	1	1	-0.022
SE	9857	NADK	NADK	chr1	-	1755373	1755476	1754543	1754698	1756257	1756343	59	31	34	6	4	2	0	12	0	3	98	49	1	1	0.831	0.795	0.895	NA	0.667	0.173
SE	9859	NADK	NADK	chr1	-	1765227	1765446	1761951	1762035	1779904	1780069	137	67	39	0	1	0	7	28	0	0	98	49	1	1	1	0.971	1	1	1	-0.01
SE	9860	STOM	STOM	chr9	-	121366115	121366245	121356052	121356156	121370126	121370304	0	0	0	6	2	0	2	0	6	0	98	49	1	1	0	0	NA	0.143	NA	-0.143
SE	9862	RNF40	RNF40	chr16	+	30766162	30766282	30765424	30765499	30766378	30766558	15	10	1	0	2	0	0	3	0	0	98	49	1	1	1	0.714	1	NA	1	-0.095
SE	9866	ZFYVE27	ZFYVE27	chr10	+	97743093	97743164	97738476	97738674	97744728	97744915	10	2	2	0	1	0	0	2	0	0	98	49	1	1	1	0.5	1	NA	1	-0.167
SE	9873	RCBTB2	RCBTB2	chr13	-	48522307	48522403	48521897	48521962	48524658	48524757	6	3	0	1	0	0	0	5	0	0	98	49	1	1	0.75	1	NA	NA	1	-0.125
SE	9874	VPS33A	VPS33A	chr12	-	122239877	122239945	122238586	122238724	122242381	122242508	1	3	3	2	0	0	0	2	0	0	98	49	1	1	0.2	1	1	NA	1	-0.267
SE	9875	VPS33A	VPS33A	chr12	-	122244568	122244762	122242381	122242508	122249870	122250045	12	2	0	0	0	0	0	1	2	0	98	49	1	1	1	1	NA	0	1	0.5
SE	9876	BAZ2B	BAZ2B	chr2	-	159336941	159337077	159332539	159332686	159337566	159337772	10	6	1	6	2	3	0	4	0	0	98	49	1	1	0.455	0.6	0.143	NA	1	-0.601
SE	9877	BAZ2B	BAZ2B	chr2	-	159337566	159337772	159332539	159332686	159347485	159347646	44	21	6	0	0	0	1	9	0	1	98	49	1	1	1	1	1	1	0.818	0.091
SE	9878	BAZ2B	BAZ2B	chr2	-	159389344	159389485	159386352	159386607	159395768	159395834	85	42	3	1	0	0	1	9	0	0	98	49	1	1	0.977	1	1	1	1	-0.008
SE	9879	BAZ2B	BAZ2B	chr2	-	159427940	159428042	159412334	159412545	159428310	159428419	57	12	2	19	1	0	0	5	0	0	98	49	1	1	0.6	0.857	1	NA	1	-0.181
SE	9880	BAZ2B	BAZ2B	chr2	-	159430862	159431156	159429199	159429260	159432756	159433363	82	34	2	5	8	0	0	18	0	3	98	49	1	1	0.891	0.68	1	NA	0.75	0.107
SE	9882	BAZ2B	BAZ2B	chr2	-	159446781	159446975	159439008	159439212	159478574	159478721	77	22	2	1	0	0	0	16	0	0	98	49	1	1	0.975	1	1	NA	1	-0.008
SE	9883	BAZ2B	BAZ2B	chr2	-	159448241	159448403	159446781	159446975	159453612	159453801	41	23	5	1	0	0	0	9	0	0	98	49	1	1	0.953	1	1	NA	1	-0.016
SE	9884	BAZ2B	BAZ2B	chr2	-	159448241	159448403	159446781	159446975	159478574	159478721	37	22	5	5	3	0	0	9	0	0	98	49	1	1	0.787	0.786	1	NA	1	-0.142
SE	9885	BAZ2B	BAZ2B	chr2	-	159448241	159448409	159446781	159446975	159453612	159453801	78	30	5	1	0	0	0	14	0	0	98	49	1	1	0.975	1	1	NA	1	-0.008
SE	9886	BAZ2B	BAZ2B	chr2	-	159448241	159448409	159446781	159446975	159478574	159478721	43	28	5	5	3	0	0	14	0	0	98	49	1	1	0.811	0.824	1	NA	1	-0.122
SE	9887	BAZ2B	BAZ2B	chr2	-	159453612	159453801	159446781	159446975	159478574	159478721	36	15	2	5	3	0	0	6	0	0	98	49	1	1	0.783	0.714	1	NA	1	-0.168
SE	9888	BAZ2B	BAZ2B	chr2	-	159453612	159453801	159448241	159448403	159478574	159478721	41	16	2	2	0	0	0	6	0	0	98	49	1	1	0.911	1	1	NA	1	-0.03
SE	9889	BAZ2B	BAZ2B	chr2	-	159453612	159453801	159448241	159448409	159478574	159478721	78	23	2	8	6	0	0	11	0	5	98	49	1	1	0.83	0.657	1	NA	0.524	0.305
SE	9890	MLKL	MLKL	chr16	-	74675354	74675404	74674959	74675100	74675612	74675764	19	5	2	1	0	0	5	6	0	0	98	49	1	1	0.905	1	1	1	1	-0.032
SE	9891	MLKL	MLKL	chr16	-	74678898	74678980	74675612	74675764	74682650	74682786	20	3	1	1	0	0	0	7	0	0	98	49	1	1	0.909	1	1	NA	1	-0.03
SE	9892	MLKL	MLKL	chr16	-	74678898	74678980	74675612	74675764	74685485	74685583	6	1	8	2	0	0	0	0	0	1	98	49	1	1	0.6	1	1	NA	0	0.867
SE	9893	MLKL	MLKL	chr16	-	74682650	74682786	74675612	74675764	74685485	74685583	17	2	0	2	0	0	0	3	0	1	98	49	1	1	0.81	1	NA	NA	0.6	0.305

SE	9894	MLKL	MLKL	chr16	-	74682650	74682786	74678898	74678980	74685485	74685583	32	4	1	2	0	8	0	10	0	0	98	49	1	1	0.889	1	0.059	NA	1	-0.351
SE	9898	ICAM3	ICAM3	chr19	-	10338681	10338948	10335670	10335976	10339538	10339669	126	79	79	1	0	1	0	15	0	0	98	49	1	1	0.984	1	0.975	NA	1	-0.014
SE	9900	SLC30A6	SLC30A6	chr2	+	32175318	32175361	32174062	32174147	32184272	32184338	10	5	1	0	2	0	0	3	0	0	92	49	1	1	1	0.571	1	NA	1	-0.143
SE	9901	SLC30A6	SLC30A6	chr2	+	32177476	32177596	32175318	32175361	32184272	32184338	1	0	0	6	1	1	0	0	0	1	98	49	1	1	0.077	0	0	NA	0	0.026
SE	9909	PTP4A2	PTP4A2	chr1	-	31910037	31910112	31906420	31908960	31911695	31911826	61	20	8	2	0	0	0	12	0	0	98	49	1	1	0.938	1	1	NA	1	-0.021
SE	9912	PRKCQ	PRKCQ	chr10	-	6441892	6442081	6430809	6430938	6456673	6456812	5	0	0	2	0	0	0	2	0	0	98	49	1	1	0.556	NA	NA	NA	1	-0.444
SE	9914	MFNG	MFNG	chr22	-	37472442	37472528	37469062	37470029	37474511	37474677	14	2	4	1	0	0	0	1	0	0	98	49	1	1	0.875	1	1	NA	1	-0.042
SE	9918	DBNL	DBNL	chr7	+	44051829	44051942	44050224	44050280	44052866	44052941	88	25	26	1	0	0	5	6	0	0	98	49	1	1	0.978	1	1	1	1	-0.007
SE	9920	DBNL	DBNL	chr7	+	44059353	44059449	44058877	44058983	44059542	44059658	79	28	18	0	1	0	5	13	0	0	98	49	1	1	1	0.933	1	1	1	-0.022
SE	9921	ERLIN1	ERLIN1	chr10	-	100167347	100167406	100164003	100164095	100174207	100174281	2	3	0	1	0	0	0	3	0	0	98	49	1	1	0.5	1	NA	NA	1	-0.25
SE	9922	CHUK	CHUK	chr10	-	100194424	100194521	100193983	100194131	100199970	100200020	5	2	9	0	0	0	0	2	0	2	98	49	1	1	1	1	1	NA	0.333	0.667
SE	9923	CHUK	CHUK	chr10	-	100219007	100219132	100218717	100218825	100219269	100219359	6	4	3	1	0	0	0	2	0	1	98	49	1	1	0.75	1	1	NA	0.5	0.417
SE	9932	MKNK1	MKNK1	chr1	-	46571416	46571539	46568442	46568498	46572062	46572167	1	0	0	28	5	0	0	0	2	4	98	49	1	1	0.018	0	NA	0	0	0.009
SE	9934	MKNK1	MKNK1	chr1	-	46580529	46580627	46576574	46576654	46583227	46583329	20	6	12	1	0	0	0	3	0	0	98	49	1	1	0.909	1	1	NA	1	-0.03
SE	9935	MKNK1	MKNK1	chr1	-	46585873	46585974	46583227	46583329	46594112	46594280	1	1	1	2	0	2	0	1	0	0	98	49	1	1	0.2	1	0.2	NA	1	-0.533
SE	9937	TRIM38	TRIM38	chr6	+	25973053	25973076	25971868	25972099	25973172	25973285	18	11	3	0	0	0	0	5	0	1	72	49	1	1	1	1	1	NA	0.773	0.227
SE	9939	SART1	SART1	chr11	+	65964073	65964131	65961688	65962093	65964514	65964570	3	10	3	0	1	0	0	2	0	0	98	49	1	1	1	0.833	1	NA	1	-0.056
SE	9942	TMEM2	TMEM2	chr9	-	71697984	71698204	71694508	71694607	71700641	71700824	19	4	0	1	0	0	0	1	0	0	98	49	1	1	0.905	1	NA	NA	1	-0.047
SE	9943	TMEM2	TMEM2	chr9	-	71734805	71734994	71732350	71732520	71740062	71740232	35	3	3	6	0	2	0	6	0	0	98	49	1	1	0.745	1	0.429	NA	1	-0.275
SE	9947	MATR3	MATR3	chr5	+	139307238	139308327	139293643	139293805	139314674	139314736	12	7	5	0	0	0	0	0	1	0	98	49	1	1	1	1	1	0	NA	1
SE	9949	MATR3	MATR3	chr5	+	139318907	139319033	139317595	139317721	139319333	139319501	16	8	0	0	1	0	1	0	0	0	98	49	1	1	1	0.8	NA	1	NA	-0.1
SE	9951	SEC14L1	SEC14L1	chr17	+	77193420	77193549	77191180	77191312	77194676	77194911	298	137	75	0	0	0	5	49	0	1	98	49	1	1	1	1	1	1	0.961	0.02
SE	9953	SEC14L1	SEC14L1	chr17	+	77213313	77213492	77211949	77212201	77213917	77214019	89	66	57	1	1	0	5	14	0	0	98	49	1	1	0.978	0.971	1	1	1	-0.017
SE	9956	CLEC16A	CLEC16A	chr16	+	10977224	10977399	10972931	10973061	10979328	10979382	10	5	5	0	0	0	0	0	1	0	98	49	1	1	1	1	1	0	NA	1
SE	9957	ASCC1	ASCC1	chr10	-	72204421	72204505	72203426	72203524	72210731	72210831	0	1	0	0	0	0	0	0	0	1	98	49	1	1	NA	1	NA	NA	0	1
SE	9958	ASCC1	ASCC1	chr10	-	72210731	72210831	72203426	72203524	72213186	72213331	0	0	0	1	0	0	0	1	0	0	98	49	1	1	0	NA	NA	NA	1	-1
SE	9960	HDAC7	HDAC7	chr12	-	47791258	47791308	47789812	47789920	47791585	47791706	9	17	3	2	0	0	0	2	0	0	98	49	1	1	0.692	1	1	NA	1	-0.103
SE	9961	HDAC7	HDAC7	chr12	-	47795905	47796016	47795586	47795767	47796206	47796298	5	2	0	5	1	0	0	2	0	1	98	49	1	1	0.333	0.5	NA	NA	0.5	-0.084
SE	9965	TAZ	TAZ	chrX	+	154414100	154414190	154413481	154413567	154419542	154419623	0	2	0	1	1	0	0	0	0	1	98	49	1	1	0	0.5	NA	NA	0	0.25
SE	9967	TAZ	TAZ	chrX	+	154420211	154420264	154420031	154420094	154420657	154420735	9	4	2	0	0	0	0	2	0	1	98	49	1	1	1	1	1	NA	0.5	0.5
SE	9968	DHX9	DHX9	chr1	+	182855576	182855713	182854029	182854178	182856531	182856578	19	5	0	13	2	0	2	1	0	5	98	49	1	1	0.422	0.556	NA	1	0.091	-0.056
SE	9969	DHX9	DHX9	chr1	+	182856531	182856578	182855576	182855713	182858103	182858240	25	10	3	0	1	0	0	3	0	0	96	49	1	1	1	0.836	1	NA	1	-0.055
SE	9971	DUSP1	DUSP1	chr5	-	172769574	172769794	172768089	172769132	172770585	172771200	438	200	178	0	2	0	6	110	1	0	98	49	1	1	1	0.98	1	0.75	1	0.118
SE	9972	DUSP1	DUSP1	chr5	-	172770160	172770306	172768089	172769132	172770585	172771200	561	303	269	0	2	0	93	267	1	0	98	49	1	1	1	0.987	1	0.979	1	0.006
SE	9975	GBF1	GBF1	chr10	+	102359266	102359435	102358505	102358729	102360183	102360395	2	0	1	1	1	0	0	1	0	0	98	49	1	1	0.5	0	1	NA	1	-0.5
SE	9976	GBF1	GBF1	chr10	+	102359269	102359435	102358505	102358729	102360183	102360395	5	0	1	1	1	0	0	2	0	0	98	49	1	1	0.714	0	1	NA	1	-0.429
SE	9977	GBF1	GBF1	chr10	+	102363255	102363396	102362474	102362664	102363709	102363798	8	2	0	0	2	0	1	0	0	0	98	49	1	1	1	0.333	NA	1	NA	-0.334
SE	9978	GBF1	GBF1	chr10	+	102368217	102368454	102367477	102367560	102368738	102368832	12	3	4	3	0	0	0	4	0	0	98	49	1	1	0.667	1	1	NA	1	-0.111
SE	9979	GBF1	GBF1	chr10	+	102368738	102368832	102368217	102368454	102369210	102369387	13	5	5	1	0	0	0	6	0	0	98	49	1	1	0.867	1	1	NA	1	-0.044
SE	9980	GBF1	GBF1	chr10	+	102381126	102381255	102380505	102380686	102382055	102382899	6	1	6	1	0	0	0	1	0	0	98	49	1	1	0.75	1	1	NA	1	-0.083
SE	9984	ACTR1A	ACTR1A	chr10	-	102484159	102484376	102483010	102483103	102485608	102485733	26	8	5	0	1	0	0	6	0	0	98	49	1	1	1	0.8	1	NA	1	-0.067
SE	10002	TXNL1	TXNL1	chr18	-	56626161	56626266	56624287	56624461	56626360	56626457	3	0	0	6	0	3	0	3	0	1	98	49	1	1	0.2	NA	0	NA	0.6	-0.5
SE	10007	NT5C2	NT5C2	chr10	-	103090610	103090787	103088016	103089908	103090935	103090996	71	22	11	8	4	2	0	14	0	5	98	49	1	1	0.816	0.733	0.733	NA	0.583	0.178
SE	10009	NT5C2	NT5C2	chr10	-	103139405	103139479	103106588	103106706	103174857	103174982	52	36	9	0	2	0	1	7	0	0	98	49	1	1	1	0.9	1	1	1	-0.033
SE	10013	BSG	BSG	chr19	+	577773	578121	572453	572701	579499	579656	0	0	4	10	2	5	0	0	0	1	98	49	1	1	0	0	0.286	NA	0	0.095
SE	10015	SBF2	SBF2	chr11	-	9795830	9795957	9790555	9790683	9807999	9808185	5	1	1	0	3	0	0	1	0	1	98	49	1	1	1	0.143	1	NA	0.333	0.381
SE	10016	SBF2	SBF2	chr11	-	9853539	9853712	9852675	9852749	9856457	9856720	32	7	0	1	0	0	0	3	0	2	98	49	1	1	0.941	1	NA	NA	0.429	0.541
SE	10017	SBF2	SBF2	chr11	-	9992414	9992543	9989496	9989595	9992989	9993103	37	10	2	5	5	0	0	5	0	5	98	49	1	1	0.787	0.5	1	NA	0.333	0.429
SE	10023	DAPK2	DAPK2	chr15	-	63924815	63924861	63912107	63912197	63925940	63926093	24	18	8	1	0	0	0	6	0	0	95	49	1	1	0.925	1	1	NA	1	-0.025
SE	10024	DAPK2	DAPK2	chr15	-	63939231	63939361	63930406	63930455	63971422	63971561	37	15	3	2	0	0	0	4	0	0	98	49	1	1	0.902	1	1	NA	1	-0.033
SE	10028	ANKRD11	ANKRD11	chr16	-	89291680	89291777	89291012	89291183	89305205	89305344	2	3	0	9	4	2	0	0	0	4	98	49	1	1	0.1	0.273	0	NA	0	0.124
SE	10030	ANKRD11	ANKRD11	chr16	-	89431255	89431326	89418283	89418368	89490244	89490561	0	2	0	0	2	0	0	0	0	1	98	49	1	1	NA	0.333	NA	NA	0	0.333
SE	10035	PRKCD	PRKCD	chr3	+	53184873	53184974	53183451	53183581	53185603	53185700	74	31	6	1	1	0	1	23	0	0	98	49	1	1	0.974	0.939	1	1	1	-0.029
SE	10038	TESK2	TESK2	chr1	-	45346978	45347062	45346692	45346779	45347608	45347693	0	1	0	0	0	0	3	3	1	0	98	49	1	1	NA	1	NA	0.6	1	0.2
SE	10039	TESK2	TESK2	chr1	-	45347917	45348000	45347608	45347693	45355302	45355449	7	3	0	0	1	0	0	1	0	0	98	49	1	1	1	0.6	NA	NA	1	-0.2
SE	10046	SAFB2	SAFB2	chr19	-	5616421	5616486	5616131	5616335	5621308	5621396	21	15	1	2	0	0	0	1	0	0	98	49	1	1	0.84	1	1	NA	1	-0.053
SE	10047	ADD3	ADD3	chr10	+	110116258	110116410	110112776	110112915	110117341	110117422	37	17	16	4	0	0	0	7	0	1	98	49	1	1	0.822	1	1	NA	0.778	0.163
SE	10049	ADD3	ADD3	chr10	+	110132304	110132400	110130362	110130486	110133325	110135565	22	3	8	0	0	0	0	9	0	2	98	49	1	1	1	1	1	NA	0.692	0.308
SE	10059	NRBF2	NRBF2	chr10	+	63146208	63146293	63133246	63133500	63152149	63152190	22	9	3	0	0	1	1	2	0	0	98	49	1	1	1	1	0.6	1	1	-0.133
SE	10069	CD53	CD53	chr1	+	110892344	110892533	110891391	110891471	110894326	110894401	240	104	119	1	0	0	3	41	0	0	98	49	1	1	0.992	1	1	1	1	-0.003
SE	10070	CD53	CD53	chr1	+	110892344	110892533	110891391	110891471	110899123	110899936	141	54	53	3	0	0	3	18	0	0	98	49	1	1	0.959	1	1	1	1	-0.014
SE	10071	CD53	CD53	chr1	+	110896652	110896733	110891391	110891471	110897808	110897892	45	26	57	0	1	0	4	12	0	0	98	49	1	1	1	0.929	1	1	1	-0.024
SE	10072	CD53	CD53	chr1	+	110896652	110896733	110891391	110891471	110899123	110899936	1	0	0	3	0	0	0	3	0	0	98	49	1	1	0.143	NA	NA	NA	1	-0.857
SE	10073	CD53	CD53	chr1	+	110896652	110896733	110894959	110895055	110897808	110897892	77	55	92	1	0	0	4	21	0	0	98	49	1	1	0.975	1	1	1	1	-0.008
SE	10075	CD53	CD53	chr1	+	110897808	110897892	110896652	110896733	110899123	110899936	143	75	113	1	0	0	6	27	0	0	98	49	1	1	0.986	1	1	1	1	-0.005
SE	10076	CAMK2G	CAMK2G	chr10	-	73819531	73819645	73817478	73817554	73821681	73821730	6	3	2	12	5	4	0	2	0	0	98	49	1	1	0.2	0.231	0.2	NA	1	-0.79
SE	10077	CAMK2G	CAMK2G	chr10	-	73825278	73825347	73824039	73824084	73837467	73837511	17	25	6	1	0	0	0	1	0	0	98	49	1	1	0.895	1	1	NA	1	-0.035
SE	10078	CAMK2G	CAMK2G	chr10	-	73825320	73825347	73824039	73824084	73837467	73837511	7	15	2	1	0	0	0	1	0	0	76	49	1	1	0.819	1	1	NA	1	-0.06
SE	10081	CAMK2G	CAMK2G	chr10	-	73847224	73847347	73842457	73842541	73847987	73848082	22	16	3	1	0	0	0	3	0	0	98	49	1	1	0.917	1	1	NA	1	-0.028
SE	10082	CAMK2G	CAMK2G	chr10	-	73847224	73847347	73842457	73842541	73848525	73848609	15	1	2	1	0	0	0	2	0	0	98	49	1	1	0.882	1	1	NA	1	-0.039
SE	10084	CAMK2G	CAMK2G	chr10	-	73847987	73848082	73847224	73847347	73848525	73848609	20	23	6	1	0	0	0	1	0	0	98	49	1	1	0.909	1	1	NA	1	-0.03
SE	10085	CAMK2G	CAMK2G	chr10	-	73848525	73848609	73847987	73848082	73849012	73849115	34	24	22	5	0	0	0	2	0	2	98	49	1	1	0.773	1	1	NA	0.333	0.591
SE	10086	CAMK2G	CAMK2G	chr10	-	73853191	73853246	73852253	73852319	73860829	73860889	30	29	9	1	1	0	3	2	0	0	98	49	1	1	0.937	0.935	1	1	1	-0.043
SE	10090	PHTF1	PHTF1	chr1	-	113706593	113706722	113705889	113706162	113710253	113710475	1	3	3	1	0	0	0	1	0	0	98	49	1	1	0.333	1	1	NA	1	-0.222
SE	10096	PICALM	PICALM	chr11	-	85974707	85974812	85957170	85959060	85981128	85981228	54	19	8	0	0	1	0	13	0	0	98	49	1	1	1	1	0.8	NA	1	-0.067
SE	10097	PICALM	PICALM	chr11	-	85976622	85976682	85974707	85974812	85981128	85981228	131	73	41	1	0	0	2	30	0	0	98	49	1	1	0.985	1	1	1	1	-0.005
SE	10101	PICALM	PICALM	chr11	-	86001034	86001158	86000642	86000779	86003365	86003451	424	170	81	1	0	0	6	50	0	0	98	49	1	1	0.995	1	1	1	1	-0.002
SE	10102	PICALM	PICALM	chr11	-	86022366	86022469	86014869	86014963	86026291	86026367	309	142	70	7	5	0	0	36	0	1	98	49	1	1	0.957	0.934	1	NA	0.947	0.017
SE	10103	PICALM	PICALM	chr11	-	86022366	86022469	86014869	86014963	86031468	86031611	188	89	37	4	0	1	0	16	0	0	98	49	1	1	0.959	1	0.949	NA	1	-0.031
SE	10105	PICALM	PICALM	chr11	-	86026291	86026367	86022366	86022469	86031468	86031611	260	114	89	2	0	0	1	33	0	0	98	49	1	1	0.985	1	1	1	1	-0.005
SE	10106	PICALM	PICALM	chr11	-	86031468	86031611	86014869	86014963	86068650	86069097	92	24	16	1	1	0	1	7	0	0	98	49	1	1	0.979	0.923	1	1	1	-0.033
SE	10109	SUV420H1	SUV420H1	chr11	-	68179469	68179537	68175017	68175183	68180131	68180200	1	2	0	1	3	6	0	0	0	6	98	49	1	1	0.333	0.25	0	NA	0	0.194
SE	10125	PDZD8	PDZD8	chr10	-	117340979	117341102	117318871	117318974	117374355	117375467	8	8	1	2	1	0	0	4	0	0	98	49	1	1	0.667	0.8	1	NA	1	-0.178
SE	10127	KMT2B	KMT2B	chr19	+	35726235	35726353	35725722	35725818	35727155	35727269	11	9	1	0	1	0	0	2	0	0	98	49	1	1	1	0.818	1	NA	1	-0.061
SE	10131	PIK3C2A	PIK3C2A	chr11	-	17101277	17101434	17099859	17099969	17102661	17102831	7	2	1	2	0	0	0	1	0	0	98	49	1	1	0.636	1	1	NA	1	-0.121
SE	10132	ABHD16A	ABHD16A	chr6	-	31701273	31701340	31700941	31701028	31703149	31703360	7	1	1	4	2	3	0	0	0	3	98	49	1	1	0.467	0.2	0.143	NA	0	0.27
SE	10133	ABHD16A	ABHD16A	chr6	-	31702073	31702130	31701273	31701340	31703149	31703360	11	1	9	1	0	0	0	3	0	0	98	49	1	1	0.846	1	1	NA	1	-0.051
SE	10135	FNDC3A	FNDC3A	chr13	+	49191014	49191120	49188514	49188633	49191208	49191384	3	3	0	1	0	0	0	1	0	0	98	49	1	1	0.6	1	NA	NA	1	-0.2
SE	10136	EIF3A	EIF3A	chr10	-	119061223	119061328	119060745	119060844	119065398	119065570	7	2	0	0	1	0	0	3	0	0	98	49	1	1	1	0.5	NA	NA	1	-0.25
SE	10146	RGS10	RGS10	chr10	-	119515508	119515652	119499826	119500259	119526031	119526118	22	3	0	2	0	0	0	6	0	0	98	49	1	1	0.846	1	NA	NA	1	-0.077
SE	10149	SEC23IP	SEC23IP	chr10	+	119929606	119929762	119926035	119926227	119930328	119930431	6	0	0	2	0	0	0	1	0	0	98	49	1	1	0.6	NA	NA	NA	1	-0.4
SE	10161	DNM2	DNM2	chr19	+	10793719	10793855	10786563	10786706	10795371	10795439	55	19	14	2	0	0	1	15	0	0	98	49	1	1	0.932	1	1	1	1	-0.023
SE	10162	DNM2	DNM2	chr19	+	10796060	10796199	10795371	10795439	10798485	10798572	12	5	1	2	0	0	0	2	0	0	98	49	1	1	0.75	1	1	NA	1	-0.083
SE	10163	DNM2	DNM2	chr19	+	10797379	10797518	10795371	10795439	10798485	10798572	10	6	8	2	0	0	0	4	0	0	98	49	1	1	0.714	1	1	NA	1	-0.095
SE	10164	DNM2	DNM2	chr19	+	10798485	10798572	10795371	10795439	10802287	10802358	38	14	3	2	0	0	1	10	0	0	98	49	1	1	0.905	1	1	1	1	-0.032
SE	10165	DNM2	DNM2	chr19	+	10808568	10808580	10805915	10805967	10812263	10812377	23	13	2	0	0	0	0	4	0	1	61	49	1	1	1	1	1	NA	0.763	0.237
SE	10168	RIT1	RIT1	chr1	-	155910655	155910804	155910449	155910506	155911242	155911402	27	20	4	0	2	1	0	3	0	0	98	49	1	1	1	0.833	0.667	NA	1	-0.167
SE	10177	CYFIP2	CYFIP2	chr5	+	157327972	157328049	157326170	157326267	157330741	157330850	21	7	2	1	0	0	0	3	0	0	98	49	1	1	0.913	1	1	NA	1	-0.029
SE	10182	KIAA1468	KIAA1468	chr18	+	62252663	62252754	62244763	62244876	62255406	62255478	24	3	0	1	1	0	0	7	0	0	98	49	1	1	0.923	0.6	NA	NA	1	-0.238
SE	10183	KIAA1468	KIAA1468	chr18	+	62280359	62280442	62275373	62275473	62280645	62280709	7	2	3	9	1	0	0	1	0	1	98	49	1	1	0.28	0.5	1	NA	0.333	0.26
SE	10186	CASP9	CASP9	chr1	-	15493891	15494001	15491400	15493035	15495272	15495452	1	1	1	0	0	3	0	3	0	0	98	49	1	1	1	1	0.143	NA	1	-0.286
SE	10188	LGALS2	LGALS2	chr22	-	37571848	37571931	37570575	37570735	37579899	37580017	9	0	0	2	0	0	0	8	0	1	98	49	1	1	0.692	NA	NA	NA	0.8	-0.108
SE	10239	LST1	LST1	chr6	+	31587640	31587733	31587199	31587318	31587943	31587966	63	29	32	11	10	2	0	1	0	2	98	49	1	1	0.741	0.592	0.889	NA	0.2	0.541
SE	10240	LST1	LST1	chr6	+	31587640	31587733	31587199	31587318	31588496	31588909	39	24	22	3	2	0	0	0	0	3	98	49	1	1	0.867	0.857	1	NA	0	0.908
SE	10241	LST1	LST1	chr6	+	31587640	31587733	31587199	31587318	31588517	31588909	42	28	26	46	21	20	0	0	0	4	98	49	1	1	0.313	0.4	0.394	NA	0	0.369
SE	10244	LST1	LST1	chr6	+	31587943	31587966	31587199	31587318	31588517	31588909	17	17	14	46	21	20	0	7	0	4	72	49	1	1	0.201	0.355	0.323	NA	0.544	-0.251
SE	10247	LST1	LST1	chr6	+	31587943	31587966	31587640	31587733	31588517	31588909	31	14	19	3	4	4	0	6	0	0	72	49	1	1	0.876	0.704	0.764	NA	1	-0.219
SE	10248	LST1	LST1	chr6	+	31587943	31587966	31587640	31587733	31588562	31588909	20	6	3	10	5	2	0	1	0	2	72	49	1	1	0.576	0.45	0.505	NA	0.254	0.256
SE	10252	OXA1L	OXA1L	chr14	+	22769790	22769934	22767957	22768171	22770192	22770278	12	7	1	0	1	0	0	5	0	0	98	49	1	1	1	0.778	1	NA	1	-0.074
SE	10259	AP3D1	AP3D1	chr19	-	2108686	2108766	2100987	2102268	2109085	2109207	20	7	1	0	0	0	2	2	0	1	98	49	1	1	1	1	1	1	0.5	0.25
SE	10260	AP3D1	AP3D1	chr19	-	2114747	2114821	2114124	2114302	2115218	2115418	15	6	1	1	0	0	0	2	0	0	98	49	1	1	0.882	1	1	NA	1	-0.039
SE	10261	AP3D1	AP3D1	chr19	-	2127151	2127201	2123829	2123879	2129089	2129163	13	5	0	1	0	0	0	7	0	0	98	49	1	1	0.867	1	NA	NA	1	-0.067
SE	10267	NAP1L4	NAP1L4	chr11	-	2949226	2949264	2944429	2945646	2951258	2951315	3	4	0	2	0	0	0	0	0	1	87	49	1	1	0.458	1	NA	NA	0	0.729
SE	10274	TRAPPC6B	TRAPPC6B	chr14	-	39154210	39154294	39151745	39151839	39158284	39158402	18	6	1	1	0	0	0	2	0	0	98	49	1	1	0.9	1	1	NA	1	-0.033
SE	10280	EIF3M	EIF3M	chr11	+	32587011	32587144	32583766	32583929	32588593	32588732	13	1	0	0	0	0	0	0	0	1	98	49	1	1	1	1	NA	NA	0	1
SE	10285	PPP2R2D	PPP2R2D	chr10	+	131911561	131912598	131901237	131901330	131934457	131934555	1	0	0	6	6	0	0	0	0	3	98	49	1	1	0.077	0	NA	NA	0	0.038
SE	10286	PPP2R2D	PPP2R2D	chr10	+	131934457	131934555	131901237	131901330	131940030	131940196	15	9	0	1	2	0	0	6	0	0	98	49	1	1	0.882	0.692	NA	NA	1	-0.213
SE	10287	PPP2R2D	PPP2R2D	chr10	+	131934893	131935006	131934457	131934555	131940030	131940196	4	7	1	9	3	0	0	1	0	3	98	49	1	1	0.182	0.538	1	NA	0.143	0.43
SE	10292	DOK1	DOK1	chr2	+	74555574	74555668	74555153	74555453	74556307	74557551	7	0	5	0	0	0	1	4	0	1	98	49	1	1	1	NA	1	1	0.667	0.166
SE	10293	DOK1	DOK1	chr2	+	74555893	74556078	74555574	74555668	74556307	74557551	7	0	3	0	0	4	1	1	0	0	98	49	1	1	1	NA	0.273	1	1	-0.363
SE	10299	NLRP6	NLRP6	chr11	+	279833	279872	279326	279607	282704	282797	5	7	4	0	1	2	1	0	0	0	88	49	1	1	1	0.796	0.527	1	NA	-0.226
SE	10300	NLRP6	NLRP6	chr11	+	280083	281839	279833	279872	282701	282797	4	3	5	1	1	0	0	1	0	0	98	49	1	1	0.667	0.6	1	NA	1	-0.244
SE	10305	MCTP2	MCTP2	chr15	+	94345128	94345164	94340812	94340924	94356136	94356301	41	15	10	1	2	0	0	2	0	0	85	49	1	1	0.959	0.812	1	NA	1	-0.076
SE	10306	MCTP2	MCTP2	chr15	+	94398960	94399062	94385422	94385525	94399920	94399995	44	27	3	1	0	0	2	16	0	0	98	49	1	1	0.957	1	1	1	1	-0.014
SE	10307	MCTP2	MCTP2	chr15	+	94440175	94440298	94401899	94402019	94442918	94442960	39	18	15	2	2	0	0	8	0	0	98	49	1	1	0.907	0.818	1	NA	1	-0.092
SE	10308	MCTP2	MCTP2	chr15	+	94442918	94442960	94401899	94402019	94458136	94458246	27	16	5	2	0	0	0	4	0	1	91	49	1	1	0.879	1	1	NA	0.683	0.277
SE	10311	JAK1	JAK1	chr1	-	64839602	64839795	64838464	64838589	64841244	64841339	86	36	33	0	0	0	2	17	0	1	98	49	1	1	1	1	1	1	0.895	0.052
SE	10312	JAK1	JAK1	chr1	-	64860104	64860262	64857655	64857779	64864786	64864972	100	30	21	1	0	0	3	12	0	0	98	49	1	1	0.98	1	1	1	1	-0.007
SE	10315	FRS2	FRS2	chr12	+	69480298	69480359	69470348	69470530	69530864	69530960	2	1	0	5	0	1	0	0	0	1	98	49	1	1	0.167	1	0	NA	0	0.389
SE	10316	FRS2	FRS2	chr12	+	69486201	69486297	69470348	69470530	69530864	69530960	1	0	2	5	0	1	0	0	0	1	98	49	1	1	0.091	NA	0.5	NA	0	0.295
SE	10322	IRF7	IRF7	chr11	-	613950	614037	613784	613865	614173	614399	6	4	0	0	1	0	2	6	0	0	98	49	1	1	1	0.667	NA	1	1	-0.166
SE	10323	IRF7	IRF7	chr11	-	613950	614037	613784	613865	614475	614534	13	11	0	2	2	0	8	14	8	3	98	49	1	1	0.765	0.733	NA	0.333	0.7	0.233
SE	10329	RASSF5	RASSF5	chr1	+	206585179	206585295	206584386	206584684	206586825	206589284	52	36	23	1	4	3	0	10	0	2	98	49	1	1	0.963	0.818	0.793	NA	0.714	0.144
SE	10330	ANKRD13D	ANKRD13D	chr11	+	67289828	67289965	67289344	67289550	67290077	67290213	6	4	2	7	3	7	0	3	0	4	98	49	1	1	0.3	0.4	0.125	NA	0.273	0.002
SE	10331	ANKRD13D	ANKRD13D	chr11	+	67299057	67299124	67291990	67292180	67299529	67299611	21	14	19	0	0	1	0	3	0	1	98	49	1	1	1	1	0.905	NA	0.6	0.368
SE	10332	ANKRD13D	ANKRD13D	chr11	+	67301731	67301823	67301487	67301651	67302118	67302484	32	23	10	2	0	0	1	8	0	0	98	49	1	1	0.889	1	1	1	1	-0.037
SE	10337	FIP1L1	FIP1L1	chr4	+	53379072	53379117	53377652	53377923	53379224	53379264	9	8	0	5	2	3	0	0	0	3	94	49	1	1	0.484	0.676	0	NA	0	0.387
SE	10338	FIP1L1	FIP1L1	chr4	+	53391429	53391498	53391008	53391139	53399729	53399839	4	5	3	2	0	0	0	1	0	0	98	49	1	1	0.5	1	1	NA	1	-0.167
SE	10350	DYNC1LI2	DYNC1LI2	chr16	-	66749196	66749313	66742437	66742668	66751272	66751346	25	12	0	0	0	0	1	6	0	1	98	49	1	1	1	1	NA	1	0.75	0.125
SE	10351	TUBA4A	TUBA4A	chr2	-	219251564	219251713	219249710	219251323	219252007	219252230	48	21	26	0	0	1	6	4	0	0	98	49	1	1	1	1	0.929	1	1	-0.024
SE	10357	GALNS	GALNS	chr16	-	88831997	88832101	88826701	88826838	88835212	88835352	16	5	6	1	0	0	1	3	0	0	98	49	1	1	0.889	1	1	1	1	-0.037
SE	10358	GALNS	GALNS	chr16	-	88837621	88837765	88836200	88836267	88840991	88841094	4	3	0	1	0	0	0	1	0	2	98	49	1	1	0.667	1	NA	NA	0.2	0.633
SE	10359	GALNS	GALNS	chr16	-	88842705	88842829	88841896	88841971	88856757	88856966	2	2	3	4	1	1	0	0	0	1	98	49	1	1	0.2	0.5	0.6	NA	0	0.433
SE	10364	IL13RA1	IL13RA1	chrX	+	118746953	118747092	118741016	118741156	118749657	118749778	58	17	13	0	1	0	0	11	0	0	98	49	1	1	1	0.895	1	NA	1	-0.035
SE	10366	AKNA	AKNA	chr9	-	114376465	114377532	114374092	114374167	114381059	114381446	128	42	26	0	1	0	3	25	0	0	98	49	1	1	1	0.955	1	1	1	-0.015
SE	10370	NUP98	NUP98	chr11	-	3731390	3731578	3725102	3725219	3735190	3735324	51	20	5	1	0	0	0	11	0	0	98	49	1	1	0.962	1	1	NA	1	-0.013
SE	10371	NUP98	NUP98	chr11	-	3744508	3744649	3735190	3735324	3753315	3753408	28	28	13	3	2	0	0	13	0	0	98	49	1	1	0.824	0.875	1	NA	1	-0.1
SE	10372	NUP98	NUP98	chr11	-	3782041	3782145	3779155	3779257	3797399	3797792	55	29	7	1	0	0	2	7	0	0	98	49	1	1	0.965	1	1	1	1	-0.012
SE	10373	STIM1	STIM1	chr11	+	3967551	3967682	3855702	3856409	4023872	4023987	35	15	13	0	2	0	7	10	0	0	98	49	1	1	1	0.789	1	1	1	-0.07
SE	10384	PIK3R5	PIK3R5	chr17	-	8890737	8890912	8889972	8890126	8893585	8893655	47	18	19	0	1	0	4	14	0	0	98	49	1	1	1	0.9	1	1	1	-0.033
SE	10389	ZSWIM8	ZSWIM8	chr10	+	73791323	73791499	73790974	73791176	73791858	73792852	9	5	6	1	0	0	0	6	1	0	98	49	1	1	0.818	1	1	0	1	0.439
SE	10390	IP6K1	IP6K1	chr3	-	49738211	49738422	49732790	49732972	49786353	49786540	9	2	1	0	2	0	0	3	0	0	98	49	1	1	1	0.333	1	NA	1	-0.222
SE	10393	C18orf25	C18orf25	chr18	+	46240004	46240187	46215810	46216595	46253696	46253823	2	0	0	1	0	0	0	2	0	0	98	49	1	1	0.5	NA	NA	NA	1	-0.5
SE	10396	GCH1	GCH1	chr14	-	54844000	54844143	54842005	54843054	54845767	54845852	1	0	0	0	0	0	0	1	0	2	98	49	1	1	1	NA	NA	NA	0.2	0.8
SE	10419	ANKRD10	ANKRD10	chr13	-	110894108	110894202	110892691	110893263	110906032	110906124	3	0	0	1	1	1	0	0	0	4	98	49	1	1	0.6	0	0	NA	0	0.2

SE	10526	TMEM33	TMEM33	chr4	+	41939195	41939383	41938601	41938696	41943746	41943814	13	6	3	6	0	0	0	4	0	0	98	49	1	1	0.52	1	1	NA		1	-0.16
SE	10529	SF1	SF1	chr11	-	64765950	64766155	64764603	64765518	64766899	64767079	1	1	0	4	0	0	0	1	0	0	98	49	1	1	0.111	1	NA	NA		1	-0.445
SE	10530	SF1	SF1	chr11	-	64773429	64773505	64770255	64770408	64776497	64776626	22	9	1	2	0	0	1	6	0	0	98	49	1	1	0.846	1	1		1	1	-0.051
SE	10532	PPP1CA	PPP1CA	chr11	-	67401067	67401199	67400688	67400919	67401727	67401905	53	18	11	1	1	0	1	10	0	0	98	49	1	1	0.964	0.9	1		1	1	-0.045
SE	10533	PPP1CA	PPP1CA	chr11	-	67401067	67401232	67400688	67400919	67401727	67401905	35	9	7	1	1	0	0	6	0	0	98	49	1	1	0.946	0.818	1	NA		1	-0.079
SE	10542	ATF7IP	ATF7IP	chr12	+	14446987	14447053	14438129	14438267	14456560	14456634	43	7	2	1	0	0	4	17	0	0	98	49	1	1	0.956	1	1		1	1	-0.015
SE	10544	RAD9A	RAD9A	chr11	+	67395715	67395825	67393690	67393790	67395911	67396021	1	0	2	0	0	0	0	0	0	2	98	49	1	1	1	NA	1	NA		0	1
SE	10551	FLAD1	FLAD1	chr1	+	154990338	154990528	154990158	154990257	154992712	154992786	2	0	0	0	2	0	1	7	0	0	98	49	1	1	1	0	NA		1	1	-0.5
SE	10552	TMEM222	TMEM222	chr1	+	27330984	27331137	27330719	27330804	27332069	27332101	1	0	0	3	0	5	0	0	0	1	98	49	1	1	0.143	NA	0	NA		0	0.071
SE	10557	PBRM1	PBRM1	chr3	-	52615350	52615456	52609312	52609955	52617261	52617538	6	0	1	1	0	0	0	1	0	1	98	49	1	1	0.75	NA	1	NA		0.333	0.542
SE	10558	PBRM1	PBRM1	chr3	-	52643247	52643343	52641953	52642045	52644703	52644789	11	1	0	1	0	0	0	4	0	0	98	49	1	1	0.846	1	NA	NA		1	-0.077
SE	10574	NFKBIA	NFKBIA	chr14	-	35403149	35403360	35402770	35402859	35403689	35403798	122	334	51	0	2	0	2	41	0	0	98	49	1	1	1	0.988	1		1	1	-0.004
SE	10583	GZMH	GZMH	chr14	-	24607148	24607406	24606479	24606746	24608264	24608412	7	8	4	0	2	0	0	3	0	0	98	49	1	1	1	0.667	1	NA		1	-0.111
SE	10585	GZMH	GZMH	chr14	-	24607340	24607406	24606479	24606746	24608264	24608412	0	0	0	0	2	0	0	3	0	0	98	49	1	1	NA	0	NA	NA		1	-1
SE	10586	GZMH	GZMH	chr14	-	24607611	24607747	24606479	24606746	24608264	24608412	8	9	0	0	2	0	1	0	0	0	98	49	1	1	1	0.692	NA		1	NA	-0.154
SE	10587	GZMH	GZMH	chr14	-	24607611	24607747	24607148	24607406	24608264	24608412	13	5	0	0	0	0	1	0	0	3	98	49	1	1	1	1	NA		1	0	0.5
SE	10604	HDAC9	HDAC9	chr7	+	18629349	18629481	18593907	18594029	18634626	18634742	2	0	2	2	0	0	0	1	0	1	98	49	1	1	0.333	NA	1	NA		0.333	0.333
SE	10606	MICAL2	MICAL2	chr11	+	12138389	12138460	12110575	12110726	12162078	12162419	14	10	1	2	0	0	0	2	0	1	98	49	1	1	0.778	1	1	NA		0.5	0.426
SE	10607	MICAL2	MICAL2	chr11	+	12138389	12138464	12110575	12110726	12162078	12162419	13	6	1	2	0	0	0	3	0	1	98	49	1	1	0.765	1	1	NA		0.6	0.322
SE	10609	MICAL2	MICAL2	chr11	+	12220200	12220458	12216218	12216319	12221643	12221759	9	6	0	0	0	0	0	3	0	1	98	49	1	1	1	1	NA	NA		0.6	0.4
SE	10610	MICAL2	MICAL2	chr11	+	12249183	12249246	12239435	12239585	12256784	12256971	10	6	2	7	13	0	0	1	0	0	98	49	1	1	0.417	0.187	1	NA		1	-0.465
SE	10613	ZFAND3	ZFAND3	chr6	+	37929958	37929999	37819530	37820016	38061592	38061775	4	4	7	0	0	0	0	1	0	1	90	49	1	1	1	1	1	NA		0.353	0.647
SE	10614	CSNK1D	CSNK1D	chr17	-	82245975	82246039	82242660	82244831	82248874	82249014	2	0	0	1	2	0	0	2	0	1	98	49	1	1	0.5	0	NA	NA		0.5	-0.25
SE	10615	CSNK1D	CSNK1D	chr17	-	82253015	82253244	82252433	82252604	82255428	82255577	24	9	7	0	0	0	2	3	1	0	98	49	1	1	1	1	1		0.5	1	0.25
SE	10616	CSNK1D	CSNK1D	chr17	-	82255428	82255577	82253015	82253244	82265685	82265796	20	10	7	0	0	0	0	3	0	2	98	49	1	1	1	1	1	NA		0.429	0.571
SE	10617	ATP5F1	ATP5F1	chr1	+	111454210	111454356	111449836	111449873	111456085	111456249	33	2	2	0	2	0	0	8	0	1	98	49	1	1	1	0.333	1	NA		0.8	-0.022
SE	10625	ADAR	ADAR	chr1	-	154586180	154586363	154585752	154585865	154588124	154588258	119	57	37	8	3	1	0	38	0	2	98	49	1	1	0.881	0.905	0.949	NA		0.905	0.007
SE	10626	ADAR	ADAR	chr1	-	154589368	154589462	154588550	154588673	154589756	154589928	104	53	29	2	0	0	4	39	0	0	98	49	1	1	0.963	1	1		1	1	-0.012
SE	10632	KATNB1	KATNB1	chr16	+	57750826	57750927	57744393	57744511	57751260	57751302	4	1	1	0	1	0	0	2	0	1	98	49	1	1	1	0.333	1	NA		0.5	0.278
SE	10638	HAGH	HAGH	chr16	-	1826506	1826602	1822864	1823037	1826711	1827194	1	0	0	1	1	0	0	0	1	0	98	49	1	1	0.333	0	NA		0	NA	0.167
SE	10639	EED	EED	chr11	+	86266082	86266216	86264171	86264263	86276979	86277138	4	1	0	0	1	0	0	2	0	0	98	49	1	1	1	0.333	NA	NA		1	-0.334
SE	10648	TBC1D14	TBC1D14	chr4	+	6923372	6924111	6909767	6909951	6967303	6967424	52	35	23	1	0	0	2	5	0	0	98	49	1	1	0.963	1	1		1	1	-0.012
SE	10649	TBC1D14	TBC1D14	chr4	+	6996324	6996407	6994183	6994302	6999084	6999202	83	50	52	1	0	0	0	7	0	0	98	49	1	1	0.976	1	1	NA		1	-0.008
SE	10650	TBC1D14	TBC1D14	chr4	+	7004843	7004924	7001144	7001251	7006631	7006726	55	35	8	0	2	0	0	11	0	0	98	49	1	1	1	0.897	1	NA		1	-0.034
SE	10651	TBC1D14	TBC1D14	chr4	+	7004843	7004924	7001144	7001251	7010652	7010781	33	21	4	0	0	0	0	3	0	3	98	49	1	1	1	1	1	NA		0.333	0.667
SE	10652	TBC1D14	TBC1D14	chr4	+	7006631	7006726	7001144	7001251	7010652	7010781	7	3	2	0	0	0	0	0	0	3	98	49	1	1	1	1	1	NA		0	1
SE	10653	TBC1D14	TBC1D14	chr4	+	7006631	7006726	7004843	7004924	7010652	7010781	29	15	6	0	0	0	0	9	0	1	98	49	1	1	1	1	1	NA		0.818	0.182
SE	10654	TBC1D14	TBC1D14	chr4	+	7009876	7009948	7006631	7006726	7010652	7010781	27	13	12	7	1	2	0	6	0	0	98	49	1	1	0.659	0.867	0.75	NA		1	-0.241
SE	10656	MAPK3	MAPK3	chr16	-	30118046	30118163	30117669	30117784	30118348	30118538	10	2	9	0	0	0	0	0	0	2	98	49	1	1	1	1	1	NA		0	1
SE	10666	LILRB2	LILRB2	chr19	-	54276380	54276456	54276263	54276301	54276806	54276929	8	7	3	3	0	0	0	5	0	1	98	49	1	1	0.571	1	1	NA		0.714	0.143
SE	10667	LILRB2	LILRB2	chr19	-	54280263	54280299	54279790	54280075	54280462	54280544	41	17	10	1	0	0	0	8	0	0	85	49	1	1	0.959	1	1	NA		1	-0.014
SE	10668	LILRB2	LILRB2	chr19	-	54280462	54280544	54279790	54280075	54280960	54281178	1	0	0	0	1	0	0	0	0	1	98	49	1	1	1	0	NA	NA		0	0.5
SE	10671	IFFO1	IFFO1	chr12	-	6549475	6549484	6548667	6548825	6549755	6549896	5	0	1	2	0	0	0	1	0	0	58	49	1	1	0.679	NA	1	NA		1	-0.16
SE	10686	DICER1	DICER1	chr14	-	95129471	95129632	95126579	95126748	95130057	95130192	57	22	7	1	0	0	3	18	0	0	98	49	1	1	0.966	1	1		1	1	-0.011
SE	10687	DICER1	DICER1	chr14	-	95131508	95131639	95130057	95130192	95132514	95132677	20	13	9	5	2	0	0	8	0	0	98	49	1	1	0.667	0.765	1	NA		1	-0.189
SE	10688	THAP5	THAP5	chr7	-	108565829	108566022	108562143	108565105	108569489	108569768	2	0	0	0	0	0	0	0	0	2	98	49	1	1	1	NA	NA	NA		0	1
SE	10689	THAP5	THAP5	chr7	-	108565951	108566022	108562143	108565105	108569489	108569768	2	0	0	0	0	0	0	0	0	2	98	49	1	1	1	NA	NA	NA		0	1
SE	10691	NPL	NPL	chr1	+	182803697	182803771	182794355	182794439	182812155	182812213	6	2	0	0	3	0	0	1	0	0	98	49	1	1	1	0.25	NA	NA		1	-0.375
SE	10692	NPL	NPL	chr1	+	182806144	182806232	182803697	182803771	182812155	182812213	58	17	23	0	2	0	0	2	0	0	98	49	1	1	1	0.81	1	NA		1	-0.063
SE	10693	NPL	NPL	chr1	+	182806144	182806531	182803697	182803771	182812155	182812213	24	10	17	0	2	0	0	8	0	0	98	49	1	1	1	0.714	1	NA		1	-0.095
SE	10699	PHF12	PHF12	chr17	-	28907589	28907672	28906855	28906994	28910225	28910369	13	8	0	0	1	0	0	4	0	0	98	49	1	1	1	0.8	NA	NA		1	-0.1
SE	10700	PHF12	PHF12	chr17	-	28908782	28908881	28907589	28907672	28910225	28910369	26	11	3	2	1	0	0	5	0	1	98	49	1	1	0.867	0.846	1	NA		0.714	0.19
SE	10701	PHF12	PHF12	chr17	-	28919142	28919275	28917284	28917449	28921687	28921808	39	13	7	4	2	0	0	4	0	0	98	49	1	1	0.83	0.765	1	NA		1	-0.135
SE	10702	PHF12	PHF12	chr17	-	28950064	28950246	28926990	28927063	28950894	28951490	10	3	6	0	3	0	0	3	0	0	98	49	1	1	1	0.333	1	NA		1	-0.222
SE	10705	KLRD1	KLRD1	chr12	+	10309387	10309480	10307817	10308084	10309625	10309688	9	3	1	0	3	0	0	2	0	0	98	49	1	1	1	0.333	1	NA		1	-0.222
SE	10706	PRR5L	PRR5L	chr11	+	36446299	36446440	36437384	36437476	36451208	36451335	8	5	1	1	0	0	0	1	0	0	98	49	1	1	0.8	1	1	NA		1	-0.067
SE	10710	RASGRP2	RASGRP2	chr11	-	64727295	64727360	64726910	64727131	64728862	64729042	36	5	10	0	0	0	0	7	1	0	98	49	1	1	1	1	1		0	1	0.5
SE	10714	SRSF6	SRSF6	chr20	+	43459152	43459420	43458360	43458509	43459770	43459895	2	1	0	3	2	0	0	0	0	1	98	49	1	1	0.25	0.2	NA	NA		0	0.225
SE	10721	GAS7	GAS7	chr17	-	9940125	9940200	9934165	9934244	9943120	9943236	24	6	2	0	0	0	0	15	0	1	98	49	1	1	1	1	1	NA		0.882	0.118
SE	10722	GAS7	GAS7	chr17	-	9969676	9969762	9959201	9959255	9981803	9981884	18	4	2	1	0	0	1	9	1	0	98	49	1	1	0.9	1	1		0.333	1	0.3
SE	10730	ANXA5	ANXA5	chr4	-	121678414	121678494	121677893	121677950	121681670	121681761	35	25	3	0	0	0	0	22	0	1	98	49	1	1	1	1	1	NA		0.917	0.083
SE	10731	ANXA5	ANXA5	chr4	-	121684676	121684771	121683363	121683477	121686287	121686372	23	12	9	0	1	0	3	20	0	1	98	49	1	1	1	0.857	1		1	0.909	-0.002
SE	10732	NUCB1	NUCB1	chr19	+	48921826	48921932	48921153	48921324	48922317	48923283	26	5	8	0	1	0	1	8	0	0	98	49	1	1	1	0.714	1		1	1	-0.095
SE	10734	MYO1F	MYO1F	chr19	-	8527337	8527483	8526788	8526935	8530195	8530365	341	289	82	0	1	0	16	86	0	0	98	49	1	1	1	0.993	1		1	1	-0.002
SE	10736	MYO1F	MYO1F	chr19	-	8536251	8536396	8530458	8530573	8536498	8536597	241	169	83	0	2	0	6	64	0	0	98	49	1	1	1	0.977	1		1	1	-0.008
SE	10739	MYO1F	MYO1F	chr19	-	8544296	8544464	8539946	8540028	8548035	8548122	3	12	0	2	0	0	0	3	0	0	98	49	1	1	0.429	1	NA	NA		1	-0.285
SE	10740	MYO1F	MYO1F	chr19	-	8545649	8545736	8544296	8544464	8548035	8548122	339	179	105	1	3	0	11	71	0	0	98	49	1	1	0.994	0.968	1		1	1	-0.013
SE	10742	MYO1F	MYO1F	chr19	-	8550159	8550356	8548236	8548317	8550561	8550694	248	136	68	1	0	0	11	62	0	0	98	49	1	1	0.992	1	1		1	1	-0.003
SE	10743	MYO1F	MYO1F	chr19	-	8552032	8552164	8551739	8551874	8553138	8553228	238	75	63	1	0	0	1	36	0	0	98	49	1	1	0.992	1	1		1	1	-0.003
SE	10746	ATG13	ATG13	chr11	+	46618324	46618375	46617275	46617890	46630044	46630100	2	2	2	1	1	0	0	1	0	1	98	49	1	1	0.5	0.5	1	NA		0.333	0.334
SE	10747	ATG13	ATG13	chr11	+	46618324	46618375	46617275	46617890	46644278	46644360	0	0	0	1	0	0	0	2	0	0	98	49	1	1	0	NA	NA	NA		1	-1
SE	10748	ATG13	ATG13	chr11	+	46630044	46630100	46617275	46617890	46644278	46644360	6	2	1	1	0	0	0	2	0	0	98	49	1	1	0.75	1	1	NA		1	-0.083
SE	10749	ATG13	ATG13	chr11	+	46630044	46630100	46618324	46618375	46644278	46644360	7	3	3	0	0	0	0	1	0	1	98	49	1	1	1	1	1	NA		0.333	0.667
SE	10753	DOCK4	DOCK4	chr7	-	111735053	111735167	111732225	111732287	111736916	111736989	10	2	0	3	0	0	3	1	0	0	98	49	1	1	0.625	1	NA		1	1	-0.188
SE	10754	DOCK4	DOCK4	chr7	-	111877029	111877186	111872466	111872564	111895611	111895718	20	5	1	1	0	0	0	10	0	0	98	49	1	1	0.909	1	1	NA		1	-0.03
SE	10755	DOCK4	DOCK4	chr7	-	111945716	111945798	111944810	111944871	111977131	111977283	35	1	0	2	0	0	4	8	0	0	98	49	1	1	0.897	1	NA		1	1	-0.051
SE	10759	TNFSF13	TNFSF13	chr17	+	7559623	7559702	7558291	7559297	7559845	7559893	13	3	0	1	0	0	0	6	0	0	98	49	1	1	0.867	1	NA	NA		1	-0.067
SE	10769	NFXL1	NFXL1	chr4	-	47885497	47885657	47884345	47884437	47885878	47885999	30	4	2	1	0	0	0	2	0	0	98	49	1	1	0.937	1	1	NA		1	-0.021
SE	10773	PTK2B	PTK2B	chr8	+	27436250	27436348	27435742	27435793	27437121	27437206	38	21	12	2	0	0	1	13	0	0	98	49	1	1	0.905	1	1		1	1	-0.032
SE	10774	PTK2B	PTK2B	chr8	+	27437121	27437206	27435742	27435793	27437395	27437496	50	15	10	0	3	0	0	9	0	0	98	49	1	1	1	0.714	1	NA		1	-0.095
SE	10776	PTK2B	PTK2B	chr8	+	27454530	27454611	27454153	27454291	27458293	27459391	87	62	19	1	0	0	7	21	0	0	98	49	1	1	0.978	1	1		1	1	-0.007
SE	10779	TTC7A	TTC7A	chr2	+	47021861	47021979	47011330	47011435	47023407	47023465	9	0	0	2	0	0	0	8	0	0	98	49	1	1	0.692	NA	NA	NA		1	-0.308
SE	10780	TTC7A	TTC7A	chr2	+	47047265	47047337	47046314	47046431	47049948	47050046	0	0	0	0	2	0	0	1	0	2	98	49	1	1	NA	0	NA	NA		0.2	-0.2
SE	10781	FNBP4	FNBP4	chr11	-	47743952	47744163	47736615	47736740	47746055	47746394	17	4	0	1	0	0	0	1	0	0	98	49	1	1	0.895	1	NA	NA		1	-0.052
SE	10784	BIN1	BIN1	chr2	-	127051153	127051243	127050801	127050912	127059010	127059155	1	0	0	0	3	0	0	2	0	0	98	49	1	1	1	0	NA	NA		1	-0.5
SE	10792	PHF21A	PHF21A	chr11	-	45938156	45938312	45936493	45936569	45945839	45946003	27	2	1	0	1	0	0	6	0	3	98	49	1	1	1	0.5	1	NA		0.5	0.333
SE	10793	PHF21A	PHF21A	chr11	-	45945839	45946003	45938156	45938312	45948885	45948946	16	2	1	2	0	0	0	4	0	0	98	49	1	1	0.8	1	1	NA		1	-0.067
SE	10794	PHF21A	PHF21A	chr11	-	45946075	45946098	45938156	45938312	45948885	45948946	7	6	0	2	0	0	1	0	0	0	72	49	1	1	0.704	1	NA		1	NA	-0.148
SE	10796	PHF21A	PHF21A	chr11	-	45971115	45971367	45969814	45969904	45979759	45979966	18	4	7	1	0	0	0	2	0	0	98	49	1	1	0.9	1	1	NA		1	-0.033
SE	10797	MAN2A2	MAN2A2	chr15	+	90906739	90906913	90906369	90906497	90907308	90907495	28	10	11	0	1	0	1	19	0	0	98	49	1	1	1	0.833	1		1	1	-0.056
SE	10798	MAN2A2	MAN2A2	chr15	+	90910089	90910292	90909326	90909504	90910500	90910683	16	10	11	2	0	3	0	8	0	1	98	49	1	1	0.8	1	0.647	NA		0.8	0.016
SE	10799	MAN2A2	MAN2A2	chr15	+	90912541	90912664	90912042	90912279	90912876	90912991	21	12	4	0	0	0	0	2	0	1	98	49	1	1	1	1	1	NA		0.5	0.5
SE	10803	ERAP1	ERAP1	chr5	-	96788530	96788685	96786469	96786549	96790295	96790367	19	2	3	1	0	0	0	5	0	0	98	49	1	1	0.905	1	1	NA		1	-0.032
SE	10811	LPCAT3	LPCAT3	chr12	-	6977597	6977745	6977366	6977525	6978340	6978507	8	3	2	1	0	0	0	2	0	0	98	49	1	1	0.8	1	1	NA		1	-0.067
SE	10813	JMJD4	JMJD4	chr1	-	227734650	227734816	227733906	227734032	227735011	227735411	1	0	0	1	0	0	0	1	0	0	98	49	1	1	0.333	NA	NA	NA		1	-0.667
SE	10819	HIF1A	HIF1A	chr14	+	61721738	61721823	61721508	61721654	61726705	61726818	142	125	35	2	0	0	1	43	0	0	98	49	1	1	0.973	1	1		1	1	-0.009
SE	10820	HIF1A	HIF1A	chr14	+	61727452	61727655	61726705	61726818	61732417	61732524	264	166	27	0	1	0	5	67	0	0	98	49	1	1	1	0.988	1		1	1	-0.004
SE	10821	HIF1A	HIF1A	chr14	+	61738086	61738373	61736888	61737109	61740504	61740627	62	29	14	0	1	0	1	10	0	1	98	49	1	1	1	0.935	1		1	0.833	0.062
SE	10822	HIF1A	HIF1A	chr14	+	61740504	61740627	61738086	61738373	61740754	61741188	64	37	8	0	0	0	0	14	1	0	98	49	1	1	1	1	1		0	1	0.5
SE	10824	HIF1A	HIF1A	chr14	+	61745690	61745817	61744704	61744813	61746933	61748259	124	77	15	0	1	1	4	49	0	0	98	49	1	1	1	0.975	0.882		1	1	-0.048
SE	10825	SLC43A3	SLC43A3	chr11	-	57409934	57410121	57409174	57409298	57414614	57414731	3	0	0	1	0	0	0	1	0	0	98	49	1	1	0.6	NA	NA	NA		1	-0.4
SE	10826	SLC43A3	SLC43A3	chr11	-	57425540	57425670	57423981	57424028	57425988	57426352	5	0	0	0	0	0	0	2	0	1	98	49	1	1	1	NA	NA	NA		0.5	0.5
SE	10827	SLC43A3	SLC43A3	chr11	-	57425540	57425709	57423981	57424028	57425988	57426389	1	0	0	0	0	0	0	0	0	1	98	49	1	1	1	NA	NA	NA		0	1
SE	10834	ZDHHC5	ZDHHC5	chr11	+	57695919	57696043	57693782	57693915	57696760	57696873	17	5	8	0	0	0	0	0	0	1	98	49	1	1	1	1	1	NA		0	1
SE	10836	PEX16	PEX16	chr11	-	45915467	45915568	45914603	45914684	45915702	45915836	19	14	1	1	0	0	0	1	0	0	98	49	1	1	0.905	1	1	NA		1	-0.032
SE	10841	ITGAX	ITGAX	chr16	+	31355892	31355998	31355133	31355291	31356624	31356728	246	143	17	0	1	0	17	42	0	0	98	49	1	1	1	0.986	1		1	1	-0.005
SE	10842	ITGAX	ITGAX	chr16	+	31356624	31356728	31355892	31355998	31357030	31357101	346	206	30	0	1	0	26	76	0	0	98	49	1	1	1	0.99	1		1	1	-0.003
SE	10843	ITGAX	ITGAX	chr16	+	31357252	31357364	31357030	31357101	31359699	31359830	117	72	28	1	1	0	10	34	0	0	98	49	1	1	0.983	0.973	1		1	1	-0.015
SE	10844	ITGAX	ITGAX	chr16	+	31371083	31371214	31363164	31363374	31371629	31371784	51	35	10	1	0	0	4	8	0	0	98	49	1	1	0.962	1	1		1	1	-0.013
SE	10850	SRP54	SRP54	chr14	+	35000935	35001020	34999557	34999649	35007282	35007387	13	3	5	1	0	0	0	3	0	0	98	49	1	1	0.867	1	1	NA		1	-0.044
SE	10851	SRP54	SRP54	chr14	+	35013345	35013494	35011508	35011659	35013801	35013902	8	2	4	1	0	0	0	2	0	1	98	49	1	1	0.8	1	1	NA		0.5	0.433
SE	10853	STAT6	STAT6	chr12	-	57099293	57099440	57099014	57099078	57099995	57100090	37	24	7	1	0	0	0	8	0	0	98	49	1	1	0.949	1	1	NA		1	-0.017
SE	10854	STAT6	STAT6	chr12	-	57099766	57099903	57099293	57099440	57099995	57100090	74	45	23	3	0	0	0	13	0	0	98	49	1	1	0.925	1	1	NA		1	-0.025
SE	10855	STAT6	STAT6	chr12	-	57104725	57104813	57104463	57104586	57105150	57105339	123	56	34	2	0	0	3	18	0	0	98	49	1	1	0.969	1	1		1	1	-0.01
SE	10867	FAM111A	FAM111A	chr11	+	59145826	59145856	59143082	59143304	59148835	59148953	6	2	0	1	0	0	1	2	0	0	79	49	1	1	0.788	1	NA		1	1	-0.106
SE	10873	PRDX5	PRDX5	chr11	+	64320660	64320792	64319733	64319868	64320864	64320903	14	31	17	1	0	0	0	4	0	1	98	49	1	1	0.875	1	1	NA		0.667	0.291
SE	10874	COPB2	COPB2	chr3	-	139373665	139373808	139373212	139373412	139374488	139374588	15	3	4	10	0	0	0	1	0	0	98	49	1	1	0.429	1	1	NA		1	-0.19
SE	10875	INPP5B	INPP5B	chr1	-	37940687	37940798	37931912	37932053	37943639	37943669	3	0	0	0	0	2	0	2	0	0	98	49	1	1	1	NA	0	NA		1	-0.5
SE	10877	PSMB4	PSMB4	chr1	+	151401238	151401355	151400763	151400845	151401541	151401630	54	27	17	1	0	0	0	11	0	0	98	49	1	1	0.964	1	1	NA		1	-0.012
SE	10890	ADGRE2	ADGRE2	chr19	-	14754953	14755127	14752328	14752526	14756237	14756345	20	5	0	0	0	0	0	3	1	1	98	49	1	1	1	1	NA		0	0.6	0.7
SE	10892	ADGRE2	ADGRE2	chr19	-	14755653	14755877	14754953	14755127	14756237	14756345	24	6	12	2	0	0	0	4	0	0	98	49	1	1	0.857	1	1	NA		1	-0.048
SE	10893	ADGRE2	ADGRE2	chr19	-	14764432	14764610	14756237	14756345	14765319	14765397	20	8	0	1	0	0	0	4	0	0	98	49	1	1	0.909	1	NA	NA		1	-0.045
SE	10894	ADGRE2	ADGRE2	chr19	-	14765657	14765804	14765523	14765570	14772341	14772497	8	1	0	0	0	0	0	0	0	1	98	49	1	1	1	1	NA	NA		0	1
SE	10895	ADGRE2	ADGRE2	chr19	-	14766234	14766381	14765657	14765804	14766977	14767109	16	1	0	2	0	0	0	1	0	0	98	49	1	1	0.8	1	NA	NA		1	-0.1
SE	10896	ADGRE2	ADGRE2	chr19	-	14766977	14767109	14765657	14765804	14772341	14772497	17	0	1	8	1	0	0	1	0	0	98	49	1	1	0.515	0	1	NA		1	-0.495
SE	10897	PTS	PTS	chr11	+	112230207	112230230	112228593	112228673	112230625	112230682	1	0	0	10	6	0	0	0	0	4	72	49	1	1	0.064	0	NA	NA		0	0.032
SE	10898	PTS	PTS	chr11	+	112230625	112230682	112228593	112228673	112233162	112233233	23	8	0	0	0	0	0	5	0	1	98	49	1	1	1	1	NA	NA		0.714	0.286
SE	10899	KPNB1	KPNB1	chr17	+	47663088	47663178	47661118	47661178	47664158	47664269	58	19	19	2	0	0	0	10	0	0	98	49	1	1	0.935	1	1	NA		1	-0.022
SE	10900	ZNF805	ZNF805	chr19	+	57243922	57244049	57240684	57240921	57248604	57248700	0	2	3	0	0	0	0	0	1	0	98	49	1	1	NA	1	1		0	NA	1
SE	10901	PACS1	PACS1	chr11	+	66221153	66221247	66220630	66220791	66227503	66227584	42	22	9	0	3	0	0	9	0	0	98	49	1	1	1	0.786	1	NA		1	-0.071
SE	10902	PACS1	PACS1	chr11	+	66242911	66243031	66241426	66241653	66243164	66244747	33	5	4	1	2	1	0	8	0	0	98	49	1	1	0.943	0.556	0.667	NA		1	-0.278
SE	10909	CTBS	CTBS	chr1	-	84569930	84570139	84565840	84566012	84570581	84570720	20	11	11	9	0	11	0	4	0	0	98	49	1	1	0.526	1	0.333	NA		1	-0.38
SE	10912	RBL2	RBL2	chr16	+	53469915	53470185	53467057	53467169	53470382	53470663	38	16	11	0	2	0	0	3	0	0	98	49	1	1	1	0.8	1	NA		1	-0.067
SE	10913	PLBD1	PLBD1	chr12	-	14553192	14553412	14542207	14542291	14567581	14567857	67	19	15	3	0	0	2	33	0	1	98	49	1	1	0.918	1	1		1	0.943	0.001
SE	10916	SPOPL	SPOPL	chr2	+	138550482	138550604	138550156	138550294	138550902	138551054	34	7	3	1	0	0	0	2	0	0	98	49	1	1	0.944	1	1	NA		1	-0.019
SE	10921	GANAB	GANAB	chr11	-	62632564	62632745	62631029	62631183	62633004	62633101	14	1	0	0	0	0	1	3	0	1	98	49	1	1	1	1	NA		1	0.6	0.2
SE	10922	GANAB	GANAB	chr11	-	62634309	62634375	62633444	62633514	62634820	62635000	4	0	0	4	1	0	0	0	0	1	98	49	1	1	0.333	0	NA	NA		0	0.167
SE	10925	ZNF292	ZNF292	chr6	+	87218595	87218731	87216298	87216377	87233324	87233527	25	9	1	0	2	0	0	1	0	0	98	49	1	1	1	0.692	1	NA		1	-0.103
SE	10926	ZNF292	ZNF292	chr6	+	87245502	87245644	87243474	87243611	87254649	87263688	13	13	3	0	1	0	0	7	0	0	98	49	1	1	1	0.867	1	NA		1	-0.044
SE	10931	PRRC2C	PRRC2C	chr1	+	171512031	171512200	171485526	171485735	171513000	171513172	25	3	1	0	0	0	1	4	1	0	98	49	1	1	1	1	1		0.333	1	0.334
SE	10932	PRRC2C	PRRC2C	chr1	+	171545478	171545687	171539970	171542229	171550085	171550240	22	7	0	0	0	0	0	0	0	1	98	49	1	1	1	1	NA	NA		0	1
SE	10936	SIGIRR	SIGIRR	chr11	-	408694	408893	408072	408206	409867	410027	2	0	0	2	0	0	0	2	0	0	98	49	1	1	0.333	NA	NA	NA		1	-0.667
SE	10937	TNPO1	TNPO1	chr5	+	72855773	72855923	72851243	72851319	72861807	72861914	18	6	6	1	0	0	0	1	0	0	98	49	1	1	0.9	1	1	NA		1	-0.033
SE	10939	TNPO1	TNPO1	chr5	+	72900005	72900081	72897055	72897151	72900973	72901073	8	3	3	0	0	0	0	0	0	1	98	49	1	1	1	1	1	NA		0	1
SE	10944	TM9SF2	TM9SF2	chr13	+	99539445	99539557	99537738	99537863	99540713	99540793	64	22	27	0	0	0	0	11	0	1	98	49	1	1	1	1	1	NA		0.846	0.154
SE	10945	TM9SF2	TM9SF2	chr13	+	99546984	99547104	99543862	99543995	99549164	99549222	97	46	25	0	1	0	0	9	0	0	98	49	1	1	1	0.958	1	NA		1	-0.014
SE	10946	TM9SF2	TM9SF2	chr13	+	99549164	99549222	99543862	99543995	99552166	99552326	34	17	13	1	0	0	0	19	0	0	98	49	1	1	0.944	1	1	NA		1	-0.019
SE	10949	CERS5	CERS5	chr12	-	50144768	50144969	50143951	50144057	50167100	50167533	0	1	0	0	0	0	0	0	0	2	98	49	1	1	NA	1	NA	NA		0	1
SE	10953	ZC3H11A	ZC3H11A	chr1	+	203837965	203838064	203833790	203833853	203840305	203840374	35	25	8	1	0	0	2	2	0	0	98	49	1	1	0.946	1	1		1	1	-0.018
SE	10957	CR1	CR1	chr1	+	207587385	207587565	207584648	207584876	207588674	207588774	158	63	21	1	0	0	2	47	0	0	98	49	1	1	0.987	1	1		1	1	-0.004
SE	10959	CR1	CR1	chr1	+	207622992	207623068	207621972	207621996	207630516	207630621	49	24	31	0	2	0	0	11	0	0	98	49	1	1	1	0.857	1	NA		1	-0.048
SE	10960	CR1	CR1	chr1	+	207630516	207630621	207622992	207623068	207639396	207641765	76	44	28	2	0	0	1	9	0	0	98	49	1	1	0.95	1	1		1	1	-0.017
SE	10967	PPP6R3	PPP6R3	chr11	+	68551120	68551186	68548066	68548204	68554144	68554257	35	26	12	0	0	0	0	2	0	2	98	49	1	1	1	1	1	NA		0.333	0.667
SE	10968	PPP6R3	PPP6R3	chr11	+	68567013	68567166	68564302	68564432	68569747	68569897	17	8	6	0	3	0	0	6	0	0	98	49	1	1	1	0.571	1	NA		1	-0.143
SE	10969	PPP6R3	PPP6R3	chr11	+	68583042	68583129	68575957	68576043	68587926	68588024	25	11	1	1	0	0	0	5	0	0	98	49	1	1	0.926	1	1	NA		1	-0.025

SE	10970	PPP6R3	PPP6R3	chr11	+	68583042	68583129	68575957	68576043	68587944	68588024	20	5	1	0	0	0	0	2	0	1	98	49	1	1	1	1	1	NA	0.5	0.5
SE	10971	PPP6R3	PPP6R3	chr11	+	68587926	68588024	68575957	68576043	68590659	68590714	19	12	5	0	1	0	1	3	0	0	98	49	1	1	1	0.857	1	1	1	-0.048
SE	10972	PPP6R3	PPP6R3	chr11	+	68587944	68588024	68575957	68576043	68590659	68590714	18	12	5	0	1	0	1	4	0	0	98	49	1	1	1	0.857	1	1	1	-0.048
SE	10976	FRMD8	FRMD8	chr11	+	65393572	65393674	65389360	65389528	65394040	65394099	6	7	6	1	1	0	1	3	0	0	98	49	1	1	0.75	0.778	1	1	1	-0.157
SE	10979	PCNXL3	PCNXL3	chr11	+	65617469	65617509	65617249	65617349	65617939	65619067	4	3	0	0	2	0	0	1	0	0	89	49	1	1	1	0.452	NA	NA	1	-0.274
SE	10980	PCNXL3	PCNXL3	chr11	+	65617610	65617706	65617469	65617509	65617939	65619067	5	6	0	2	0	0	0	2	0	0	98	49	1	1	0.556	1	NA	NA	1	-0.222
SE	10982	PCNXL3	PCNXL3	chr11	+	65625651	65625744	65625404	65625510	65625903	65626054	4	1	1	1	0	0	0	1	0	0	98	49	1	1	0.667	1	1	NA	1	-0.111
SE	11002	TFAM	TFAM	chr10	+	58390764	58390860	58388669	58388819	58394357	58394414	4	7	6	0	4	0	0	1	0	0	98	49	1	1	1	0.467	1	NA	1	-0.178
SE	11009	MSH6	MSH6	chr2	+	47790926	47791123	47783081	47783493	47795893	47796063	5	0	0	1	0	0	0	1	0	0	98	49	1	1	0.714	NA	NA	NA	1	-0.286
SE	11011	ANKRD13A	ANKRD13A	chr12	+	110016387	110016433	110013124	110013249	110019138	110019328	36	20	9	1	0	0	0	9	0	0	95	49	1	1	0.949	1	1	NA	1	-0.017
SE	11012	ANKRD13A	ANKRD13A	chr12	+	110018344	110018488	110016387	110016433	110019138	110019328	55	14	25	2	2	0	0	18	0	1	98	49	1	1	0.932	0.778	1	NA	0.9	0.003
SE	11013	ANKRD13A	ANKRD13A	chr12	+	110027704	110027766	110025741	110025823	110028511	110028642	71	33	35	1	0	0	0	8	0	0	98	49	1	1	0.973	1	1	NA	1	-0.009
SE	11014	ANKRD13A	ANKRD13A	chr12	+	110033796	110033957	110030644	110030758	110036260	110036328	38	19	14	1	0	0	2	4	0	1	98	49	1	1	0.95	1	1	1	0.667	0.15
SE	11015	IFIT3	IFIT3	chr10	+	89337420	89337542	89332478	89332627	89338660	89340968	0	1	0	2	10	0	0	0	1	1	98	49	1	1	0	0.048	NA	0	0	0.024
SE	11016	IFIT3	IFIT3	chr10	+	89337424	89337542	89327818	89328078	89338660	89340968	0	1	0	5	5	0	0	0	0	1	98	49	1	1	0	0.091	NA	NA	0	0.045
SE	11017	IFIT3	IFIT3	chr10	+	89337424	89337542	89332478	89332627	89338660	89340968	0	2	0	2	10	0	0	0	1	1	98	49	1	1	0	0.091	NA	0	0	0.045
SE	11020	RBM4	RBM4	chr11	+	66643449	66644140	66639699	66640123	66646026	66646473	13	5	2	1	0	1	0	2	0	0	98	49	1	1	0.867	1	0.5	NA	1	-0.211
SE	11027	KANSL1	KANSL1	chr17	-	46170854	46172232	46094559	46094701	46192822	46192906	38	12	0	0	1	0	0	7	0	0	98	49	1	1	1	0.857	NA	NA	1	-0.072
SE	11032	SIRT6	SIRT6	chr19	-	4180781	4180909	4179103	4179286	4182473	4182599	2	1	0	0	1	0	0	1	0	0	98	49	1	1	1	0.333	NA	NA	1	-0.334
SE	11036	DNAH1	DNAH1	chr3	+	52349988	52350108	52349194	52349420	52350507	52350590	3	2	0	0	1	0	0	3	0	0	98	49	1	1	1	0.5	NA	NA	1	-0.25
SE	11038	DNAH1	DNAH1	chr3	+	52398031	52398162	52397706	52397877	52398849	52399201	5	0	0	1	1	0	0	0	0	1	98	49	1	1	0.714	0	NA	NA	0	0.357
SE	11045	PNISR	PNISR	chr6	-	99403828	99403882	99402539	99402710	99404602	99404702	23	7	3	0	4	1	0	4	0	0	98	49	1	1	1	0.467	0.6	NA	1	-0.311
SE	11049	THEMIS2	THEMIS2	chr1	+	27876587	27876728	27872542	27872665	27879643	27879963	181	71	24	1	0	0	13	31	0	1	98	49	1	1	0.989	1	1	1	0.939	0.027
SE	11050	THEMIS2	THEMIS2	chr1	+	27876587	27876728	27872542	27872665	27885294	27885451	75	50	16	0	0	0	10	8	1	0	98	49	1	1	1	1	1	0.833	1	0.084
SE	11051	THEMIS2	THEMIS2	chr1	+	27879643	27880054	27872542	27872665	27885294	27885451	3	0	1	0	0	0	0	2	1	0	98	49	1	1	1	NA	1	0	1	0.5
SE	11054	THEMIS2	THEMIS2	chr1	+	27881970	27882125	27879643	27880054	27882512	27883043	46	9	18	1	0	0	1	18	0	0	98	49	1	1	0.958	1	1	1	1	-0.014
SE	11057	THEMIS2	THEMIS2	chr1	+	27882512	27883043	27879643	27880054	27885294	27885451	26	9	1	2	0	1	0	1	0	1	98	49	1	1	0.867	1	0.333	NA	0.333	0.4
SE	11059	CNOT10	CNOT10	chr3	+	32713226	32713369	32708669	32708820	32716224	32716311	15	4	0	2	0	0	0	3	0	0	98	49	1	1	0.789	1	NA	NA	1	-0.105
SE	11060	CNOT10	CNOT10	chr3	+	32717153	32717237	32716224	32716311	32720113	32720231	16	2	5	0	1	0	0	4	0	0	98	49	1	1	1	0.5	1	NA	1	-0.167
SE	11089	FCHSD2	FCHSD2	chr11	-	73140030	73140128	73083694	73083740	73141856	73142098	9	9	2	1	1	0	0	1	0	0	98	49	1	1	0.818	0.818	1	NA	1	-0.121
SE	11091	PCMTD1	PCMTD1	chr8	-	51845660	51845763	51833517	51833689	51860844	51861246	18	13	22	0	0	1	0	8	0	0	98	49	1	1	1	1	0.917	NA	1	-0.028
SE	11092	PCMTD1	PCMTD1	chr8	-	51845660	51845763	51833517	51833689	51898929	51899186	9	7	13	0	0	0	0	4	0	1	98	49	1	1	1	1	1	NA	0.667	0.333
SE	11093	PCMTD1	PCMTD1	chr8	-	51860844	51861246	51833517	51833689	51898929	51899186	12	12	2	0	0	0	1	1	0	1	98	49	1	1	1	1	1	1	0.333	0.334
SE	11095	ARHGAP15	ARHGAP15	chr2	+	143202133	143202202	143155476	143155655	143216383	143216445	323	56	40	1	0	0	3	32	0	0	98	49	1	1	0.994	1	1	1	1	-0.002
SE	11097	ARHGAP15	ARHGAP15	chr2	+	143487372	143487495	143436912	143437042	143519265	143519364	193	53	16	3	0	0	0	19	0	0	98	49	1	1	0.97	1	1	NA	1	-0.01
SE	11098	ARHGAP15	ARHGAP15	chr2	+	143519265	143519364	143487372	143487495	143556407	143556485	160	33	14	0	2	0	1	15	0	0	98	49	1	1	1	0.892	1	1	1	-0.036
SE	11105	SF3A3	SF3A3	chr1	-	37978719	37978827	37976883	37976953	37978987	37979055	9	8	0	1	0	0	0	3	0	0	98	49	1	1	0.818	1	NA	NA	1	-0.091
SE	11106	CYB5R4	CYB5R4	chr6	+	83909008	83909090	83893521	83893622	83918004	83918065	12	15	18	0	2	0	0	3	0	0	98	49	1	1	1	0.789	1	NA	1	-0.07
SE	11107	CYB5R4	CYB5R4	chr6	+	83914415	83914448	83909008	83909090	83918004	83918065	113	38	42	0	0	1	0	17	0	0	82	49	1	1	1	1	0.962	NA	1	-0.013
SE	11108	CYB5R4	CYB5R4	chr6	+	83918004	83918065	83914415	83914448	83919396	83919454	125	40	36	1	0	0	0	16	0	0	98	49	1	1	0.984	1	1	NA	1	-0.005
SE	11121	RAVER1	RAVER1	chr19	-	10321047	10321259	10320654	10320951	10321530	10321618	7	2	0	0	0	1	0	1	0	0	98	49	1	1	1	1	0	NA	1	-0.333
SE	11126	CCT4	CCT4	chr2	-	61872457	61872588	61872081	61872316	61873001	61873112	6	0	1	0	0	0	0	0	0	1	98	49	1	1	1	NA	1	NA	0	1
SE	11139	PER1	PER1	chr17	-	8145957	8146137	8144750	8144993	8146371	8146502	3	0	2	1	1	0	0	3	0	1	98	49	1	1	0.6	0	1	NA	0.6	-0.067
SE	11140	PER1	PER1	chr17	-	8146896	8147002	8146593	8146765	8147249	8147381	3	0	1	0	0	0	0	5	0	2	98	49	1	1	1	NA	1	NA	0.556	0.444
SE	11141	ENTPD4	ENTPD4	chr8	-	23442006	23442066	23441568	23441723	23443849	23443953	19	10	4	3	4	0	0	2	0	1	98	49	1	1	0.76	0.556	1	NA	0.5	0.272
SE	11142	SRGN	SRGN	chr10	+	69097083	69097231	69088071	69088236	69103870	69104811	1921	1250	628	5	6	1	28	397	0	0	98	49	1	1	0.995	0.99	0.997	1	1	-0.006
SE	11143	ZYX	ZYX	chr7	+	143382592	143382685	143382247	143382447	143382800	143383322	102	20	25	0	1	0	10	23	0	0	98	49	1	1	1	0.909	1	1	1	-0.03
SE	11144	ZYX	ZYX	chr7	+	143388218	143388339	143382800	143383322	143388488	143388658	89	33	35	1	0	0	5	34	0	0	98	49	1	1	0.978	1	1	1	1	-0.007
SE	11145	THADA	THADA	chr2	-	43556344	43556555	43552067	43552339	43560233	43560385	14	7	0	1	0	0	0	4	0	0	98	49	1	1	0.875	1	NA	NA	1	-0.062
SE	11151	FCRL6	FCRL6	chr1	+	159808960	159809245	159808177	159808444	159809401	159809683	2	0	0	3	0	0	0	0	0	1	98	49	1	1	0.25	NA	NA	NA	0	0.25
SE	11154	RNF34	RNF34	chr12	+	121402741	121402807	121400117	121400218	121416158	121416377	1	0	0	1	0	1	0	0	0	1	98	49	1	1	0.333	NA	0	NA	0	0.167
SE	11155	RNF34	RNF34	chr12	+	121420576	121420778	121420241	121420334	121423385	121424352	6	1	6	0	1	0	0	3	0	0	98	49	1	1	1	0.333	1	NA	1	-0.222
SE	11161	CBL	CBL	chr11	+	119232447	119232695	119206275	119206612	119271734	119271881	18	6	8	1	0	0	0	3	0	0	98	49	1	1	0.9	1	1	NA	1	-0.033
SE	11162	PHB2	PHB2	chr12	-	6967170	6967238	6966419	6966500	6967891	6968021	27	4	1	0	2	0	0	4	0	0	98	49	1	1	1	0.5	1	NA	1	-0.167
SE	11167	LARP4B	LARP4B	chr10	-	825064	825316	820799	820845	825763	825870	10	6	2	0	2	0	0	4	0	0	98	49	1	1	1	0.6	1	NA	1	-0.133
SE	11177	POLR2A	POLR2A	chr17	+	7496209	7496346	7495940	7496073	7496439	7496613	41	20	2	1	0	0	1	9	0	0	98	49	1	1	0.953	1	1	1	1	-0.016
SE	11184	CD300LF	CD300LF	chr17	-	74696194	74696217	74695724	74695859	74698368	74698481	12	2	2	2	1	0	0	2	0	0	72	49	1	1	0.803	0.576	1	NA	1	-0.207
SE	11187	CD300LF	CD300LF	chr17	-	74698368	74698481	74696194	74696217	74703034	74703098	24	8	10	0	1	0	0	1	0	0	98	49	1	1	1	0.8	1	NA	1	-0.067
SE	11193	MRE11A	MRE11A	chr11	-	94470470	94470642	94467812	94467893	94471573	94471759	6	0	0	1	0	0	0	2	0	0	98	49	1	1	0.75	NA	NA	NA	1	-0.25
SE	11208	PIP5K1C	PIP5K1C	chr19	-	3642906	3642939	3641704	3641809	3643242	3643381	2	0	0	0	0	0	0	2	0	2	82	49	1	1	1	NA	NA	NA	0.374	0.626
SE	11209	SIPA1L1	SIPA1L1	chr14	+	71698980	71699127	71685361	71685631	71702380	71702505	42	17	0	1	0	0	0	6	0	0	98	49	1	1	0.955	1	NA	NA	1	-0.022
SE	11211	SIPA1L1	SIPA1L1	chr14	+	71730054	71730301	71724669	71724835	71733665	71733812	16	7	3	2	0	0	0	3	0	0	98	49	1	1	0.8	1	1	NA	1	-0.067
SE	11212	SIPA1L1	SIPA1L1	chr14	+	71735276	71735391	71733665	71733812	71738240	71738325	22	1	2	1	0	0	1	1	0	0	98	49	1	1	0.917	1	1	1	1	-0.028
SE	11220	HPS5	HPS5	chr11	-	18317750	18317907	18311913	18312024	18321945	18322174	2	1	0	0	1	0	0	1	0	0	98	49	1	1	1	0.333	NA	NA	1	-0.334
SE	11223	CASP4	CASP4	chr11	-	104951895	104952005	104950924	104951098	104954746	104955001	94	48	44	7	0	2	0	13	0	3	98	49	1	1	0.87	1	0.917	NA	0.684	0.245
SE	11232	DPY19L3	DPY19L3	chr19	+	32437193	32437339	32436445	32436567	32439111	32439235	7	2	6	1	0	0	0	3	0	0	98	49	1	1	0.778	1	1	NA	1	-0.074
SE	11237	ATM	ATM	chr11	+	108235669	108235834	108229177	108229323	108243952	108244118	30	16	6	0	2	0	0	9	0	0	98	49	1	1	1	0.8	1	NA	1	-0.067
SE	11238	ATM	ATM	chr11	+	108244787	108245026	108243952	108244118	108246963	108247127	26	10	5	1	0	0	1	4	0	0	98	49	1	1	0.929	1	1	1	1	-0.024
SE	11239	ATM	ATM	chr11	+	108268409	108268609	108267170	108267342	108271063	108271146	31	6	10	0	0	0	0	9	0	1	98	49	1	1	1	1	1	NA	0.818	0.182
SE	11243	MAP4K2	MAP4K2	chr11	-	64800899	64801031	64800763	64800826	64801110	64801183	7	7	0	1	0	0	1	0	0	0	98	49	1	1	0.778	1	NA	1	NA	-0.111
SE	11244	MTOR	MTOR	chr1	-	11233397	11233487	11232435	11232528	11234142	11234265	8	0	4	0	1	0	0	5	0	0	98	49	1	1	1	0	1	NA	1	-0.333
SE	11245	MTOR	MTOR	chr1	-	11256932	11257165	11255991	11256192	11258484	11258593	5	3	0	0	1	0	2	4	0	0	98	49	1	1	1	0.6	NA	1	1	-0.2
SE	11246	PROK2	PROK2	chr3	-	71774444	71774507	71771654	71772828	71781466	71781592	105	34	27	27	7	5	0	2	0	4	98	49	1	1	0.66	0.708	0.73	NA	0.2	0.499
SE	11253	ABTB1	ABTB1	chr3	+	127674544	127674600	127674390	127674453	127675969	127676114	85	66	21	1	1	0	1	14	0	0	98	49	1	1	0.977	0.971	1	1	1	-0.017
SE	11254	ABTB1	ABTB1	chr3	+	127675969	127676114	127674544	127674600	127676535	127676581	61	43	14	1	0	0	1	12	0	0	98	49	1	1	0.968	1	1	1	1	-0.011
SE	11255	ABTB1	ABTB1	chr3	+	127676271	127676431	127675969	127676114	127676535	127676581	168	72	42	1	0	0	3	20	0	0	98	49	1	1	0.988	1	1	1	1	-0.004
SE	11256	ABTB1	ABTB1	chr3	+	127676966	127677083	127676535	127676581	127677167	127677286	96	44	25	0	0	2	1	20	0	0	98	49	1	1	1	1	0.862	1	1	-0.046
SE	11260	DLEU2	DLEU2	chr13	-	50029277	50029492	50027206	50027406	50044650	50044768	11	4	17	3	3	2	0	2	0	0	98	49	1	1	0.647	0.4	0.81	NA	1	-0.381
SE	11261	DLEU2	DLEU2	chr13	-	50044650	50044768	50027206	50027406	50049583	50049662	13	3	2	0	1	1	0	1	0	0	98	49	1	1	1	0.6	0.5	NA	1	-0.3
SE	11263	DLEU2	DLEU2	chr13	-	50075558	50075653	50068096	50068163	50125301	50125541	31	2	3	1	0	0	0	1	0	0	98	49	1	1	0.939	1	1	NA	1	-0.02
SE	11264	STXBP3	STXBP3	chr1	+	108800219	108800305	108798144	108798237	108807400	108807549	28	18	6	2	0	0	0	5	0	0	98	49	1	1	0.875	1	1	NA	1	-0.042
SE	11270	WBP11	WBP11	chr12	-	14794970	14795104	14794536	14794736	14796806	14797003	11	0	0	0	1	0	0	2	0	0	98	49	1	1	1	0	NA	NA	1	-0.5
SE	11278	FXR2	FXR2	chr17	-	7594237	7594347	7593917	7594004	7594678	7594757	4	0	5	1	1	0	0	1	0	0	98	49	1	1	0.667	0	1	NA	1	-0.444
SE	11279	SAT2	SAT2	chr17	-	7626942	7627044	7626752	7626793	7627142	7627226	0	0	0	4	0	0	0	2	0	1	98	49	1	1	0	NA	NA	NA	0.5	-0.5
SE	11290	TRIM39	TRIM39	chr6	+	30340271	30340361	30339907	30339930	30340504	30340620	0	0	1	6	4	4	0	0	1	0	98	49	1	1	0	0	0.111	0	NA	0.037
SE	11295	MBD1	MBD1	chr18	-	50274185	50274353	50273563	50273863	50274976	50275045	0	7	1	1	0	0	0	1	0	0	98	49	1	1	0	1	1	NA	1	-0.333
SE	11299	KDM6B	KDM6B	chr17	+	7844900	7845020	7839916	7840024	7845313	7845456	88	56	22	1	0	0	3	14	0	1	98	49	1	1	0.978	1	1	1	0.875	0.055
SE	11301	KDM6B	KDM6B	chr17	+	7851647	7851796	7851477	7851549	7851950	7852065	67	20	19	0	1	0	1	28	0	0	98	49	1	1	1	0.909	1	1	1	-0.03
SE	11306	YEATS2	YEATS2	chr3	+	183803255	183803335	183801454	183801528	183803986	183804188	2	0	0	0	0	0	0	0	0	1	98	49	1	1	1	NA	NA	NA	0	1
SE	11308	CTC1	CTC1	chr17	-	8228726	8228892	8228502	8228629	8229301	8229446	0	0	0	4	0	0	0	1	0	0	98	49	1	1	0	NA	NA	NA	1	-1
SE	11310	CTC1	CTC1	chr17	-	8231275	8231469	8230562	8230651	8231725	8231815	5	7	0	2	0	0	0	2	0	0	98	49	1	1	0.556	1	NA	NA	1	-0.222
SE	11311	CTC1	CTC1	chr17	-	8238030	8238242	8237374	8237519	8238391	8238629	3	4	2	3	0	1	0	1	0	0	98	49	1	1	0.333	1	0.5	NA	1	-0.389
SE	11315	NDEL1	NDEL1	chr17	+	8445710	8445864	8444259	8444357	8446753	8446902	95	27	15	5	2	0	0	20	0	0	98	49	1	1	0.905	0.871	1	NA	1	-0.075
SE	11316	NDEL1	NDEL1	chr17	+	8446753	8446902	8445710	8445864	8448549	8448686	132	51	28	1	0	0	0	20	0	0	98	49	1	1	0.985	1	1	NA	1	-0.005
SE	11317	NDEL1	NDEL1	chr17	+	8454795	8454887	8450779	8450953	8460008	8460160	142	40	9	2	0	0	6	23	0	0	98	49	1	1	0.973	1	1	1	1	-0.009
SE	11326	PAM	PAM	chr5	+	103025130	103025334	103019789	103019843	103028886	103031104	2	3	0	0	0	1	0	1	0	0	98	49	1	1	1	1	0	NA	1	-0.333
SE	11329	PAM	PAM	chr5	+	103028181	103028238	103025130	103025334	103028886	103031104	2	2	0	1	0	0	0	1	0	0	98	49	1	1	0.5	1	NA	NA	1	-0.25
SE	11335	PABPC1	PABPC1	chr8	-	100718086	100718280	100717772	100717888	100721390	100722087	50	8	3	0	0	0	0	29	1	0	98	49	1	1	1	1	1	0	1	0.5
SE	11343	AMPD3	AMPD3	chr11	+	10478525	10478730	10461514	10461740	10482062	10482225	9	2	0	3	0	0	0	1	0	0	98	49	1	1	0.6	1	NA	NA	1	-0.2
SE	11344	AMPD3	AMPD3	chr11	+	10482062	10482225	10478525	10478730	10484819	10485039	23	7	0	2	0	0	0	7	0	0	98	49	1	1	0.852	1	NA	NA	1	-0.074
SE	11345	AMPD3	AMPD3	chr11	+	10495569	10495733	10494898	10495030	10496811	10496938	18	10	1	0	3	0	0	4	0	0	98	49	1	1	1	0.625	1	NA	1	-0.125
SE	11346	AMPD3	AMPD3	chr11	+	10504548	10504659	10502720	10502894	10505707	10507579	27	7	4	1	0	0	0	3	0	0	98	49	1	1	0.931	1	1	NA	1	-0.023
SE	11354	NDRG1	NDRG1	chr8	-	133258365	133258426	133256776	133256863	133259167	133259230	9	2	5	0	0	0	0	6	0	1	98	49	1	1	1	1	1	NA	0.75	0.25
SE	11355	NDRG1	NDRG1	chr8	-	133264546	133264652	133262046	133262167	133280231	133280267	26	7	9	1	0	0	0	9	0	0	98	49	1	1	0.929	1	1	NA	1	-0.024
SE	11357	ZNFX1	ZNFX1	chr20	-	49252719	49252830	49251526	49251622	49253665	49253811	61	18	9	1	0	0	0	12	0	0	98	49	1	1	0.968	1	1	NA	1	-0.011
SE	11364	SCAF11	SCAF11	chr12	-	45922935	45923154	45922462	45922582	45924727	45925074	12	6	2	2	0	0	0	5	0	0	98	49	1	1	0.75	1	1	NA	1	-0.083
SE	11368	TRPV2	TRPV2	chr17	+	16433573	16433698	16431965	16432300	16434889	16434969	5	1	0	0	0	4	0	1	0	0	98	49	1	1	1	1	0	NA	1	-0.333
SE	11371	LRRC75A-AS1	LRRC75A-AS1	chr17	+	16439527	16439703	16438986	16439414	16440184	16440253	2	2	0	1	1	0	0	0	0	1	98	49	1	1	0.5	0.5	NA	NA	0	0.5
SE	11372	LRRC75A-AS1	LRRC75A-AS1	chr17	+	16439580	16439703	16438986	16439414	16440184	16440253	2	2	0	1	1	0	0	0	0	1	98	49	1	1	0.5	0.5	NA	NA	0	0.5
SE	11374	LRRC75A-AS1	LRRC75A-AS1	chr17	+	16439659	16439703	16438986	16439414	16440184	16440253	3	2	0	1	1	0	0	0	0	1	93	49	1	1	0.613	0.513	NA	NA	0	0.563
SE	11393	COPS3	COPS3	chr17	-	17247479	17247560	17246623	17247151	17248925	17249039	6	4	7	0	3	0	0	1	0	2	98	49	1	1	1	0.4	1	NA	0.2	0.6
SE	11398	TOM1L2	TOM1L2	chr17	-	17882704	17882863	17879626	17879743	17893660	17893810	4	1	1	1	0	0	0	0	0	1	98	49	1	1	0.667	1	1	NA	0	0.889
SE	11399	TOM1L2	TOM1L2	chr17	-	17884633	17884768	17879626	17879743	17893660	17893810	4	0	1	1	0	0	0	3	0	1	98	49	1	1	0.667	NA	1	NA	0.6	0.234
SE	11407	RPS24	RPS24	chr10	+	78037961	78037982	78037193	78037304	78040203	78040225	0	1	0	99	14	7	0	0	0	10	70	49	1	1	0	0.048	0	NA	0	0.016
SE	11409	RPS24	RPS24	chr10	+	78040200	78040225	78037193	78037304	78040614	78040715	62	12	6	72	17	11	0	1	0	26	74	49	1	1	0.363	0.319	0.265	NA	0.025	0.291
SE	11410	RPS24	RPS24	chr10	+	78040203	78040225	78037193	78037304	78040614	78040715	161	26	13	72	17	11	0	11	0	26	71	49	1	1	0.607	0.514	0.449	NA	0.226	0.297
SE	11413	LCMT1	LCMT1	chr16	+	25128474	25128566	25111725	25111996	25132401	25132523	1	0	0	0	0	0	0	0	0	1	98	49	1	1	1	NA	NA	NA	0	1
SE	11415	PRPSAP2	PRPSAP2	chr17	+	18867281	18867334	18865801	18865952	18872582	18872649	7	0	1	3	0	0	0	1	0	0	98	49	1	1	0.538	NA	1	NA	1	-0.231
SE	11423	ANKHD1	ANKHD1	chr5	+	140485120	140485248	140482579	140482667	140485588	140485732	16	15	0	2	0	0	0	3	0	0	98	49	1	1	0.8	1	NA	NA	1	-0.1
SE	11425	ANKHD1	ANKHD1	chr5	+	140507784	140507998	140506834	140506977	140509636	140509812	44	9	8	1	1	0	1	11	0	0	98	49	1	1	0.957	0.818	1	1	1	-0.075
SE	11426	ANKHD1	ANKHD1	chr5	+	140538085	140538261	140537388	140537589	140538918	140539083	17	3	0	1	0	0	0	3	0	0	98	49	1	1	0.895	1	NA	NA	1	-0.052
SE	11427	QSOX1	QSOX1	chr1	+	180194212	180194392	180190432	180190580	180196261	180196599	6	3	8	0	0	0	1	3	0	1	98	49	1	1	1	1	1	1	0.6	0.2
SE	11430	MX1	MX1	chr21	+	41439693	41439848	41437014	41437152	41440886	41441025	77	58	9	1	0	0	6	103	1	0	98	49	1	1	0.975	1	1	0.75	1	0.117
SE	11432	MX1	MX1	chr21	+	41449136	41449295	41445999	41446141	41451166	41451243	24	19	4	1	3	0	0	30	0	0	98	49	1	1	0.923	0.76	1	NA	1	-0.106
SE	11436	AKAP10	AKAP10	chr17	-	19940886	19941010	19939712	19939849	19941825	19941910	12	4	0	2	6	0	0	4	0	4	98	49	1	1	0.75	0.25	NA	NA	0.333	0.167
SE	11437	SPECC1	SPECC1	chr17	+	20204332	20205912	20110426	20110562	20227412	20227620	3	4	0	1	0	0	0	1	0	0	98	49	1	1	0.6	1	NA	NA	1	-0.2
SE	11438	DHX8	DHX8	chr17	+	43492680	43493040	43492182	43492292	43493444	43493589	25	12	0	0	2	0	0	4	0	0	98	49	1	1	1	0.75	NA	NA	1	-0.125
SE	11439	DHX8	DHX8	chr17	+	43493444	43493589	43492680	43493040	43493682	43493886	21	12	4	2	0	0	0	5	0	1	98	49	1	1	0.84	1	1	NA	0.714	0.233
SE	11440	DHX8	DHX8	chr17	+	43508338	43508520	43507808	43508019	43513361	43513502	8	8	8	1	0	0	1	1	0	0	98	49	1	1	0.8	1	1	1	1	-0.067
SE	11441	DHX8	DHX8	chr17	+	43520750	43520879	43520129	43520267	43521368	43521565	15	5	16	0	1	0	0	1	0	0	98	49	1	1	1	0.714	1	NA	1	-0.095
SE	11445	MAP2K3	MAP2K3	chr17	+	21303182	21303234	21302142	21302259	21304425	21304553	57	30	11	0	2	0	0	14	0	0	98	49	1	1	1	0.882	1	NA	1	-0.039
SE	11446	MAP2K3	MAP2K3	chr17	+	21312141	21312281	21305050	21305128	21313491	21313537	31	9	2	1	0	0	1	10	0	0	98	49	1	1	0.939	1	1	1	1	-0.02
SE	11453	PIGS	PIGS	chr17	-	28561421	28561629	28560048	28560191	28563430	28563522	11	4	2	0	1	0	1	4	0	0	98	49	1	1	1	0.667	1	1	1	-0.111
SE	11454	NCK2	NCK2	chr2	+	105881327	105882049	105855047	105855289	105892981	105894274	11	4	2	1	1	0	0	1	0	0	98	49	1	1	0.846	0.667	1	NA	1	-0.162
SE	11456	KIAA0100	KIAA0100	chr17	-	28615663	28615798	28614439	28615246	28616095	28616197	13	1	4	1	0	0	0	5	0	0	98	49	1	1	0.867	1	1	NA	1	-0.044
SE	11457	KIAA0100	KIAA0100	chr17	-	28616888	28616999	28616617	28616753	28617231	28617318	23	7	4	0	0	0	0	7	0	1	98	49	1	1	1	1	1	NA	0.778	0.222
SE	11458	KIAA0100	KIAA0100	chr17	-	28620508	28620630	28619859	28620015	28620987	28621170	3	7	1	1	0	0	0	3	0	0	98	49	1	1	0.6	1	1	NA	1	-0.133
SE	11459	KIAA0100	KIAA0100	chr17	-	28628264	28628559	28624232	28624390	28631478	28631681	20	5	0	0	1	1	0	7	0	1	98	49	1	1	1	0.714	0	NA	0.778	-0.207
SE	11460	KIAA0100	KIAA0100	chr17	-	28633282	28633401	28632941	28633162	28633548	28633751	5	1	0	2	0	0	0	4	0	0	98	49	1	1	0.556	1	NA	NA	1	-0.222
SE	11461	KIAA0100	KIAA0100	chr17	-	28641888	28642078	28640559	28640683	28642274	28642335	11	3	0	1	4	0	0	4	0	0	98	49	1	1	0.846	0.273	NA	NA	1	-0.441
SE	11462	KIAA0100	KIAA0100	chr17	-	28642274	28642335	28641888	28642078	28642868	28642987	14	3	0	1	0	0	0	5	0	0	98	49	1	1	0.875	1	NA	NA	1	-0.062
SE	11479	NFX1	NFX1	chr9	+	33351559	33351790	33347037	33347117	33352645	33352719	2	3	0	0	0	0	0	1	0	1	98	49	1	1	1	1	NA	NA	0.333	0.667
SE	11482	BRD8	BRD8	chr5	-	138166517	138166610	138165827	138166108	138167933	138168078	2	0	2	1	0	0	0	1	0	0	98	49	1	1	0.5	NA	1	NA	1	-0.25
SE	11483	BRD8	BRD8	chr5	-	138166517	138166727	138165827	138166108	138167933	138168078	2	0	2	1	0	0	0	1	0	0	98	49	1	1	0.5	NA	1	NA	1	-0.25
SE	11486	EGLN1	EGLN1	chr1	-	231367568	231367636	231363750	231366475	231370561	231370698	57	42	33	6	4	0	0	7	0	1	98	49	1	1	0.826	0.84	1	NA	0.778	0.111
SE	11488	FLOT2	FLOT2	chr17	-	28884224	28884315	28883107	28883231	28888944	28889026	57	43	33	6	6	2	0	13	0	2	98	49	1	1	0.826	0.782	0.892	NA	0.765	0.068
SE	11489	FLOT2	FLOT2	chr17	-	28884224	28884315	28883107	28883231	28897525	28897697	33	28	26	1	0	0	0	10	0	0	98	49	1	1	0.943	1	1	NA	1	-0.019
SE	11490	FLOT2	FLOT2	chr17	-	28888944	28889026	28883107	28883231	28897525	28897697	26	7	14	1	0	0	1	4	0	0	98	49	1	1	0.929	1	1	1	1	-0.024
SE	11491	FLOT2	FLOT2	chr17	-	28888944	28889026	28884224	28884315	28897525	28897697	44	17	19	0	1	0	1	5	0	0	98	49	1	1	1	0.895	1	1	1	-0.035
SE	11494	DHRS13	DHRS13	chr17	-	28901492	28901616	28900989	28901301	28902582	28902701	8	9	17	1	4	0	0	5	0	3	98	49	1	1	0.8	0.529	1	NA	0.455	0.321
SE	11496	SSH2	SSH2	chr17	-	29635967	29636802	29625938	29632931	29648143	29648344	140	48	23	2	0	0	2	21	0	0	98	49	1	1	0.972	1	1	1	1	-0.009
SE	11497	SSH2	SSH2	chr17	-	29648143	29648344	29625938	29632931	29650653	29650800	30	39	5	1	0	0	0	19	0	0	98	49	1	1	0.937	1	1	NA	1	-0.021
SE	11499	SSH2	SSH2	chr17	-	29695458	29695523	29684562	29684684	29702958	29703062	114	62	32	1	1	0	0	20	0	0	98	49	1	1	0.983	0.969	1	NA	1	-0.016
SE	11500	SSH2	SSH2	chr17	-	29702958	29703062	29695458	29695523	29761149	29761432	106	48	34	0	0	1	1	17	0	0	98	49	1	1	1	1	0.944	1	1	-0.019
SE	11507	CPD	CPD	chr17	+	30443801	30443971	30442307	30442450	30445690	30446020	63	33	11	0	0	1	0	1	0	0	98	49	1	1	1	1	0.846	NA	1	-0.051

SE	11508 CPD	CPD	chr17	+	30449552	30449748	30445690	30446020	30451710	30451846	50	26	28	1	0	0	0	4	0	0	98	49	1	1	0.962	1	1	NA		1	-0.013
SE	11509 CEP164	CEP164	chr11	+	117338565	117338668	117335604	117335680	117344165	117344277	4	3	1	1	0	0	0	3	0	0	98	49	1	1	0.667	1	1	NA		1	-0.111
SE	11512 SBNO2	SBNO2	chr19	-	1111018	1111093	1110744	1110888	1111505	1111614	39	25	10	0	0	0	1	6	0	1	98	49	1	1	1	1	1		1	0.75	0.125
SE	11514 SBNO2	SBNO2	chr19	-	1113534	1113704	1112817	1112949	1114230	1114422	92	52	19	1	0	0	9	21	0	1	98	49	1	1	0.979	1	1		1	0.913	0.036
SE	11516 RNF135	RNF135	chr17	+	30987943	30988106	30984616	30984760	30997241	30997331	14	3	3	2	0	0	0	5	0	0	98	49	1	1	0.778	1	1	NA		1	-0.074
SE	11518 NF1	NF1	chr17	+	31225094	31225250	31223443	31223567	31226434	31226684	14	2	0	1	0	0	0	1	0	0	98	49	1	1	0.875	1	NA	NA		1	-0.062
SE	11519 NF1	NF1	chr17	+	31232699	31232881	31232072	31232189	31233001	31233213	5	4	1	1	0	0	0	2	0	0	98	49	1	1	0.714	1	1	NA		1	-0.095
SE	11520 NF1	NF1	chr17	+	31252937	31253000	31248983	31249119	31258343	31258502	0	0	2	4	2	0	0	1	0	3	98	49	1	1	0	0	1	NA		0.143	0.19
SE	11521 CHFR	CHFR	chr12	-	132851617	132851734	132848640	132848724	132853430	132853573	9	3	4	1	0	0	0	3	0	0	98	49	1	1	0.818	1	1	NA		1	-0.061
SE	11522 CHFR	CHFR	chr12	-	132851617	132851737	132848640	132848724	132853430	132853573	8	1	0	1	0	0	0	3	0	0	98	49	1	1	0.8	1	NA	NA		1	-0.1
SE	11524 CHFR	CHFR	chr12	-	132869618	132869798	132861466	132861634	132870723	132870783	6	6	1	1	0	0	0	0	0	2	98	49	1	1	0.75	1	1	NA		0	0.917
SE	11525 CHFR	CHFR	chr12	-	132869618	132869834	132861466	132861634	132870723	132870783	4	4	1	1	0	0	0	0	0	2	98	49	1	1	0.667	1	1	NA		0	0.889
SE	11527 TCF7L2	TCF7L2	chr10	+	112964555	112964624	112951482	112951607	113040024	113040126	1	1	0	7	1	0	0	0	0	2	98	49	1	1	0.067	0.333	NA	NA		0	0.2
SE	11538 EVI2A	EVI2A	chr17	-	31320372	31320513	31316409	31319023	31321494	31321749	2	1	0	46	21	7	0	0	0	4	98	49	1	1	0.021	0.023	0	NA		0	0.015
SE	11548 TRNAU1AP	TRNAU1AP	chr1	+	28561112	28561260	28560632	28560732	28561345	28561398	2	1	0	1	2	0	0	1	0	1	98	49	1	1	0.5	0.2	NA	NA		0.333	0.017
SE	11549 NATD1	NATD1	chr17	-	21244105	21244224	21238870	21243429	21253158	21253266	7	2	0	2	0	0	0	1	0	0	98	49	1	1	0.636	1	NA	NA		1	-0.182
SE	11550 CCDC69	CCDC69	chr5	-	151198996	151199084	151187385	151187459	151201581	151201688	27	17	1	0	0	1	0	5	0	0	98	49	1	1	1	1	0.333	NA		1	-0.222
SE	11551 RFFL	RFFL	chr17	-	35017522	35017606	35016369	35016580	35021370	35021781	12	8	6	1	0	0	0	3	0	0	98	49	1	1	0.857	1	1	NA		1	-0.048
SE	11552 RFFL	RFFL	chr17	-	35021370	35021781	35017522	35017606	35026373	35026561	10	5	2	5	0	1	0	6	0	1	98	49	1	1	0.5	1	0.5	NA		0.75	-0.083
SE	11554 WDFY2	WDFY2	chr13	+	51660595	51660663	51584347	51584824	51675169	51675243	9	6	2	0	2	0	0	2	0	0	98	49	1	1	1	0.6	1	NA		1	-0.133
SE	11555 WDFY2	WDFY2	chr13	+	51719197	51719348	51703595	51703650	51727677	51727790	20	3	3	0	0	0	0	3	0	1	98	49	1	1	1	1	1	NA		0.6	0.4
SE	11556 WDFY2	WDFY2	chr13	+	51739048	51739175	51727677	51727790	51751309	51751415	7	7	2	1	0	0	0	1	0	0	98	49	1	1	0.778	1	1	NA		1	-0.074
SE	11568 PLB1	PLB1	chr2	+	28605852	28605948	28604654	28604759	28606495	28606567	13	2	2	2	0	0	0	1	0	0	98	49	1	1	0.765	1	1	NA		1	-0.078
SE	11569 PLB1	PLB1	chr2	+	28620599	28620643	28620264	28620332	28620878	28620978	17	7	6	0	0	0	2	1	0	1	93	49	1	1	1	1	1		1	0.345	0.328
SE	11570 PLB1	PLB1	chr2	+	28640926	28641001	28632943	28633039	28642857	28643787	11	2	3	0	1	0	0	5	0	0	98	49	1	1	1	0.5	1	NA		1	-0.167
SE	11579 LUCAT1	LUCAT1	chr5	-	91313077	91313229	91303872	91303971	91313637	91313777	6	1	5	1	0	0	2	3	0	1	98	49	1	1	0.75	1	1		1	0.6	0.117
SE	11580 LUCAT1	LUCAT1	chr5	-	91313077	91313229	91310913	91311032	91313637	91313777	7	1	5	0	1	0	2	4	0	0	98	49	1	1	1	0.333	1		1	1	-0.222
SE	11582 PRDM8	PRDM8	chr4	+	80196962	80197216	80196351	80196601	80197624	80197763	2	0	1	0	2	0	0	2	0	0	98	49	1	1	1	0	1	NA		1	-0.333
SE	11601 GGNBP2	GGNBP2	chr17	+	36545618	36545817	36544887	36545097	36554819	36554900	9	4	0	2	0	0	4	2	0	0	98	49	1	1	0.692	1	NA		1	1	-0.154
SE	11606 P4HA1	P4HA1	chr10	-	73051052	73051249	73046924	73047101	73053350	73053590	6	2	0	0	1	0	0	1	0	0	98	49	1	1	1	0.5	NA	NA		1	-0.25
SE	11607 P4HA1	P4HA1	chr10	-	73073730	73073827	73072028	73072180	73074807	73074915	5	0	4	1	0	0	0	1	0	0	98	49	1	1	0.714	NA	1	NA		1	-0.143
SE	11614 ARHGAP12	ARHGAP12	chr10	-	31839304	31839319	31831738	31831800	31839636	31839711	1	0	0	0	2	0	0	2	0	0	64	49	1	1	1	0	NA	NA		1	-0.5
SE	11619 MLLT6	MLLT6	chr17	+	38716366	38716981	38715611	38715828	38717431	38717613	16	1	6	1	0	0	2	4	0	0	98	49	1	1	0.889	1	1		1	1	-0.037
SE	11625 CWC25	CWC25	chr17	-	38815624	38815741	38814860	38815097	38820900	38821073	0	3	0	18	20	0	0	0	1	0	98	49	1	1	0	0.07	NA		0	NA	0.035
SE	11627 LASP1	LASP1	chr17	+	38896901	38897071	38890419	38890504	38898411	38898519	3	0	0	67	19	16	0	0	0	7	98	49	1	1	0.022	0	0	NA		0	0.007
SE	11628 LASP1	LASP1	chr17	+	38915042	38915146	38914324	38914475	38918604	38921770	62	20	16	1	1	0	2	5	0	0	98	49	1	1	0.969	0.909	1		1	1	-0.041
SE	11629 FKBP8	FKBP8	chr19	-	18533269	18533347	18532663	18532795	18537600	18537773	69	44	50	0	3	0	0	16	0	0	98	49	1	1	1	0.88	1	NA		1	-0.04
SE	11630 FKBP8	FKBP8	chr19	-	18538215	18538433	18537600	18537773	18539370	18539459	110	48	32	2	0	0	4	20	0	0	98	49	1	1	0.965	1	1		1	1	-0.012
SE	11631 FKBP8	FKBP8	chr19	-	18538215	18538436	18537600	18537773	18539370	18539459	111	64	47	2	0	0	6	17	0	0	98	49	1	1	0.965	1	1		1	1	-0.012
SE	11632 FKBP8	FKBP8	chr19	-	18539370	18539459	18538215	18538436	18541678	18541995	42	29	29	0	1	0	2	2	0	0	98	49	1	1	1	0.935	1		1	1	-0.022
SE	11636 FBXL20	FBXL20	chr17	-	39285477	39285573	39282728	39282855	39297126	39297195	20	3	3	0	1	0	0	1	0	0	98	49	1	1	1	0.6	1	NA		1	-0.133
SE	11637 FBXL20	FBXL20	chr17	-	39301000	39301075	39298989	39299084	39303584	39303639	17	8	7	1	0	0	0	1	0	0	98	49	1	1	0.895	1	1	NA		1	-0.035
SE	11669 PSMD3	PSMD3	chr17	+	39996182	39996338	39995423	39995527	39997329	39997380	10	1	2	1	0	0	0	5	0	0	98	49	1	1	0.833	1	1	NA		1	-0.056
SE	11670 MSL1	MSL1	chr17	+	40131536	40131584	40129244	40129627	40132033	40132098	73	53	21	3	2	0	0	7	0	2	97	49	1	1	0.925	0.93	1	NA		0.639	0.313
SE	11671 UBXN1	UBXN1	chr11	-	62676812	62677005	62676497	62676639	62677517	62677634	8	6	10	1	0	0	0	11	0	0	98	49	1	1	0.8	1	1	NA		1	-0.067
SE	11672 ARNT	ARNT	chr1	-	150842423	150842468	150839440	150839654	150846262	150846307	2	2	0	0	0	2	0	0	0	1	94	49	1	1	1	1	0	NA		0	0.667
SE	11680 TMEM70	TMEM70	chr8	+	73979078	73979171	73978755	73978861	73981154	73982783	0	4	0	2	1	2	0	1	0	1	98	49	1	1	0	0.667	0	NA		0.333	-0.111
SE	11687 ZDHHC3	ZDHHC3	chr3	-	44929305	44929436	44915260	44926847	44932906	44932990	7	2	2	1	0	0	0	3	0	0	98	49	1	1	0.778	1	1	NA		1	-0.074
SE	11688 ZDHHC3	ZDHHC3	chr3	-	44932906	44932990	44929305	44929436	44933117	44933199	2	1	1	10	1	0	0	2	0	0	98	49	1	1	0.091	0.333	1	NA		1	-0.525
SE	11690 DNAL1	DNAL1	chr14	+	73661986	73662042	73658846	73658956	73671541	73671597	1	0	0	1	0	0	0	1	0	0	98	49	1	1	0.333	NA	NA	NA		1	-0.667
SE	11691 SMARCA5	SMARCA5	chr4	+	143527867	143528023	143526280	143526460	143528582	143528714	29	8	4	1	0	0	0	4	0	0	98	49	1	1	0.935	1	1	NA		1	-0.022
SE	11692 SMARCA5	SMARCA5	chr4	+	143540362	143540495	143538785	143538938	143543508	143543657	15	1	1	1	0	0	0	1	0	1	98	49	1	1	0.882	1	1	NA		0.333	0.628
SE	11693 SMARCA5	SMARCA5	chr4	+	143545924	143546047	143545469	143545583	143546775	143546908	9	3	2	0	1	0	0	3	0	0	98	49	1	1	1	0.6	1	NA		1	-0.133
SE	11698 DNAJC7	DNAJC7	chr17	-	41982254	41982401	41981854	41982007	41983562	41983636	4	2	2	0	2	0	0	1	0	0	98	49	1	1	1	0.333	1	NA		1	-0.222
SE	11699 DNAJC7	DNAJC7	chr17	-	41989403	41989557	41988731	41988896	41990263	41990382	16	7	0	0	1	0	0	1	0	0	98	49	1	1	1	0.778	NA	NA		1	-0.111
SE	11702 STAT5B	STAT5B	chr17	-	42218722	42218878	42218150	42218330	42219311	42219463	45	21	22	1	0	0	1	4	0	0	98	49	1	1	0.957	1	1		1	1	-0.014
SE	11705 STAT3	STAT3	chr17	-	42324710	42324846	42323572	42323625	42324962	42325061	97	43	40	1	0	0	1	28	0	0	98	49	1	1	0.98	1	1		1	1	-0.007
SE	11706 STAT3	STAT3	chr17	-	42324710	42324846	42323572	42323625	42326115	42326199	49	25	19	0	0	0	1	17	0	1	98	49	1	1	1	1	1		1	0.895	0.052
SE	11707 STAT3	STAT3	chr17	-	42324962	42325061	42323572	42323625	42326115	42326199	27	18	26	0	0	0	0	11	0	1	98	49	1	1	1	1	1	NA		0.846	0.154
SE	11708 STAT3	STAT3	chr17	-	42324962	42325061	42324710	42324846	42326115	42326199	75	36	47	1	0	0	0	22	0	0	98	49	1	1	0.974	1	1	NA		1	-0.009
SE	11709 STAT3	STAT3	chr17	-	42348388	42348517	42346568	42346713	42388278	42388495	68	44	19	0	0	2	1	28	0	0	98	49	1	1	1	1	0.826		1	1	-0.058
SE	11710 STAT3	STAT3	chr17	-	42348388	42348539	42346568	42346713	42388278	42388387	62	40	21	0	0	2	2	29	0	0	98	49	1	1	1	1	0.84		1	1	-0.053
SE	11711 ATP6V0A1	ATP6V0A1	chr17	+	42468009	42468107	42466428	42466507	42470089	42470218	19	13	2	1	0	0	0	12	0	0	98	49	1	1	0.905	1	1	NA		1	-0.032
SE	11715 COASY	COASY	chr17	+	42563960	42564175	42562073	42563322	42564445	42564577	4	6	3	0	1	0	0	2	0	0	98	49	1	1	1	0.75	1	NA		1	-0.083
SE	11716 MLX	MLX	chr17	+	42568469	42568559	42567618	42567655	42568836	42568943	2	1	0	2	2	0	1	0	0	0	98	49	1	1	0.333	0.2	NA		1	NA	-0.734
SE	11721 EZH1	EZH1	chr17	-	42713208	42713389	42712288	42712485	42717975	42718067	27	4	1	0	0	1	0	4	0	0	98	49	1	1	1	1	0.333	NA		1	-0.222
SE	11722 EZH1	EZH1	chr17	-	42724304	42724424	42722794	42722915	42727634	42727763	33	7	3	2	3	0	0	11	0	1	98	49	1	1	0.892	0.538	1	NA		0.846	-0.036
SE	11724 BECN1	BECN1	chr17	-	42811654	42811797	42810131	42810928	42813947	42814008	13	3	6	0	0	2	0	5	0	0	98	49	1	1	1	1	0.6	NA		1	-0.133
SE	11725 BECN1	BECN1	chr17	-	42813947	42814008	42810131	42810928	42814523	42814673	5	4	7	3	1	0	0	1	0	0	98	49	1	1	0.455	0.667	1	NA		1	-0.293
SE	11733 NBR1	NBR1	chr17	+	43189587	43189802	43189041	43189119	43190608	43190776	24	7	14	0	2	0	0	9	0	0	98	49	1	1	1	0.636	1	NA		1	-0.121
SE	11734 NBR1	NBR1	chr17	+	43191371	43191581	43190608	43190776	43193093	43193253	28	8	9	1	0	0	1	8	0	0	98	49	1	1	0.933	1	1		1	1	-0.022
SE	11735 NBR1	NBR1	chr17	+	43196941	43197106	43196480	43196591	43200166	43200608	45	20	8	0	1	0	0	3	0	1	98	49	1	1	1	0.909	1	NA		0.6	0.37
SE	11736 NBR1	NBR1	chr17	+	43201685	43201780	43200166	43200608	43202654	43202712	28	13	10	0	1	0	0	10	0	0	98	49	1	1	1	0.867	1	NA		1	-0.044
SE	11747 KDM2A	KDM2A	chr11	+	67121233	67121358	67119268	67120049	67180078	67180217	57	13	19	1	0	0	0	11	0	0	98	49	1	1	0.966	1	1	NA		1	-0.011
SE	11748 KDM2A	KDM2A	chr11	+	67181319	67181398	67180078	67180217	67181845	67181892	39	17	7	3	3	0	0	7	0	1	98	49	1	1	0.867	0.739	1	NA		0.778	0.091
SE	11749 KDM2A	KDM2A	chr11	+	67215855	67215949	67215339	67215446	67217730	67217884	45	18	9	2	6	0	0	5	0	1	98	49	1	1	0.918	0.6	1	NA		0.714	0.125
SE	11750 KDM2A	KDM2A	chr11	+	67250085	67250346	67248280	67248370	67252693	67252857	27	12	2	0	1	0	1	5	0	0	98	49	1	1	1	0.857	1		1	1	-0.048
SE	11751 KDM2A	KDM2A	chr11	+	67250085	67250798	67248280	67248370	67252693	67252857	41	18	4	0	1	0	1	11	0	0	98	49	1	1	1	0.9	1		1	1	-0.033
SE	11756 GRN	GRN	chr17	+	44351549	44351795	44351362	44351460	44352014	44352248	119	44	41	1	0	0	7	58	0	0	98	49	1	1	0.983	1	1		1	1	-0.006
SE	11761 EFTUD2	EFTUD2	chr17	-	44868286	44868350	44867806	44867897	44872445	44872570	10	2	0	1	0	0	1	4	0	0	98	49	1	1	0.833	1	NA		1	1	-0.084
SE	11776 FMNL1	FMNL1	chr17	+	45245252	45245416	45244978	45245108	45245877	45245973	31	11	19	2	0	0	3	24	0	0	98	49	1	1	0.886	1	1		1	1	-0.038
SE	11786 PPP6R1	PPP6R1	chr19	-	55232074	55232211	55231801	55231982	55236642	55236821	23	18	3	2	0	0	2	8	0	0	98	49	1	1	0.852	1	1		1	1	-0.049
SE	11811 ANKRD17	ANKRD17	chr4	-	73147240	73147432	73146763	73146873	73148812	73149050	16	4	2	1	0	0	0	10	0	0	98	49	1	1	0.889	1	1	NA		1	-0.037
SE	11812 PTPN6	PTPN6	chr12	+	6954804	6954994	6951982	6952177	6955371	6955485	49	28	32	1	3	0	0	15	0	0	98	49	1	1	0.961	0.824	1	NA		1	-0.072
SE	11813 PTPN6	PTPN6	chr12	+	6955150	6955267	6954804	6954994	6955371	6955485	94	59	30	1	0	0	2	28	0	1	98	49	1	1	0.979	1	1		1	0.933	0.026
SE	11814 PTPN6	PTPN6	chr12	+	6956418	6956568	6956141	6956221	6959926	6959994	176	99	46	1	0	0	6	29	0	0	98	49	1	1	0.989	1	1		1	1	-0.004
SE	11815 PTPN6	PTPN6	chr12	+	6957653	6957785	6956418	6956568	6959926	6959994	118	62	31	1	0	0	8	22	0	0	98	49	1	1	0.983	1	1		1	1	-0.006
SE	11817 PTPN6	PTPN6	chr12	+	6960343	6960435	6960087	6960239	6960805	6960945	171	77	46	1	0	0	3	32	0	0	98	49	1	1	0.988	1	1		1	1	-0.004
SE	11818 PTPN6	PTPN6	chr12	+	6960662	6960945	6960343	6960435	6961125	6961316	104	37	13	8	3	0	0	11	0	1	98	49	1	1	0.867	0.86	1	NA		0.846	0.063
SE	11820 NPEPPS	NPEPPS	chr17	+	47586147	47586244	47585499	47585700	47586364	47586398	16	13	1	1	0	0	0	6	0	1	98	49	1	1	0.889	1	1	NA		0.75	0.213
SE	11828 UTP15	UTP15	chr5	+	73572488	73572624	73570585	73570711	73576951	73577036	2	0	0	1	0	0	0	1	0	0	98	49	1	1	0.5	NA	NA	NA		1	-0.5
SE	11831 HK3	HK3	chr5	-	176883769	176883869	176881943	176882127	176884038	176884134	26	20	11	2	0	0	1	12	0	0	98	49	1	1	0.867	1	1		1	1	-0.044
SE	11832 HK3	HK3	chr5	-	176887200	176887337	176887001	176887121	176887450	176887746	34	31	3	0	0	0	10	17	1	0	98	49	1	1	1	1	1		0.833	1	0.084
SE	11843 ST3GAL6	ST3GAL6	chr3	+	98791840	98791993	98773919	98773983	98793674	98795845	10	0	0	3	0	0	0	2	0	0	98	49	1	1	0.625	NA	NA	NA		1	-0.375
SE	11846 CALCOCO2	CALCOCO2	chr17	+	48846442	48846514	48841697	48841887	48848063	48848166	0	0	0	10	8	0	0	1	1	5	98	49	1	1	0	0	NA		0	0.091	-0.045
SE	11848 CALCOCO2	CALCOCO2	chr17	+	48851088	48851177	48849251	48849377	48851558	48851628	33	14	3	0	3	0	0	2	0	0	98	49	1	1	1	0.7	1	NA		1	-0.1
SE	11849 CALCOCO2	CALCOCO2	chr17	+	48852505	48852628	48851558	48851628	48852925	48853012	32	16	10	1	1	0	0	3	0	0	98	49	1	1	0.941	0.889	1	NA		1	-0.057
SE	11850 HMBOX1	HMBOX1	chr8	+	28980070	28980156	28970042	28970519	29009071	29009182	11	3	0	2	0	0	0	2	0	0	98	49	1	1	0.733	1	NA	NA		1	-0.133
SE	11863 KAT7	KAT7	chr17	+	49815802	49815913	49811475	49811574	49817819	49818011	17	11	1	1	0	0	0	1	0	0	98	49	1	1	0.895	1	1	NA		1	-0.035
SE	11864 KAT7	KAT7	chr17	+	49826692	49826799	49825999	49826146	49827400	49829096	7	6	0	1	0	0	2	1	0	0	98	49	1	1	0.778	1	NA		1	1	-0.111
SE	11879 SPAG9	SPAG9	chr17	-	50975862	50975901	50974770	50974947	50977107	50977221	4	0	0	8	1	1	0	0	0	2	88	49	1	1	0.218	0	0	NA		0	0.073
SE	11880 SPAG9	SPAG9	chr17	-	50984922	50984990	50982523	50982672	50985697	50985778	68	29	6	1	1	0	1	8	0	1	98	49	1	1	0.971	0.935	1		1	0.8	0.069
SE	11881 SPAG9	SPAG9	chr17	-	50984922	50984990	50982523	50982672	50987111	50987237	27	19	5	0	0	0	1	3	0	1	98	49	1	1	1	1	1		1	0.6	0.2
SE	11882 SPAG9	SPAG9	chr17	-	50985697	50985778	50982523	50982672	50987111	50987237	21	10	4	0	0	0	0	7	0	1	98	49	1	1	1	1	1	NA		0.778	0.222
SE	11883 SPAG9	SPAG9	chr17	-	50985697	50985778	50984922	50984990	50987111	50987237	62	19	5	1	0	0	0	11	0	0	98	49	1	1	0.969	1	1	NA		1	-0.01
SE	11884 SPAG9	SPAG9	chr17	-	50996564	50996694	50995443	50995533	50998443	50998617	25	10	7	0	0	4	0	8	0	0	98	49	1	1	1	1	0.467	NA		1	-0.178
SE	11886 SPAG9	SPAG9	chr17	-	51014231	51014353	51007268	51007326	51020158	51020258	111	61	27	0	0	0	0	8	1	0	98	49	1	1	1	1	1		0	1	0.5
SE	11887 SPAG9	SPAG9	chr17	-	51031680	51031722	51021157	51021365	51041500	51041651	17	6	3	17	15	7	0	0	0	8	91	49	1	1	0.35	0.177	0.188	NA		0	0.238
SE	11888 KIAA0319L	KIAA0319L	chr1	-	35442245	35442336	35441046	35441138	35442905	35443028	10	2	6	3	1	1	0	3	0	3	98	49	1	1	0.625	0.5	0.75	NA		0.333	0.292
SE	11889 KIAA0319L	KIAA0319L	chr1	-	35451627	35451776	35450357	35450509	35453556	35453689	7	1	3	0	0	0	0	0	0	1	98	49	1	1	1	1	1	NA		0	1
SE	11890 KIAA0319L	KIAA0319L	chr1	-	35456012	35456241	35454361	35454485	35460304	35460437	14	2	0	1	0	0	0	2	0	0	98	49	1	1	0.875	1	NA	NA		1	-0.062
SE	11891 KIAA0319L	KIAA0319L	chr1	-	35506611	35507135	35478965	35479212	35554349	35554519	10	3	0	1	0	0	0	1	0	0	98	49	1	1	0.833	1	NA	NA		1	-0.084
SE	11895 ME2	ME2	chr18	+	50916167	50916243	50912800	50912950	50917346	50917508	7	5	9	1	0	0	0	2	0	0	98	49	1	1	0.778	1	1	NA		1	-0.074
SE	11897 TRIM25	TRIM25	chr17	-	56895520	56895604	56895342	56895441	56895925	56895952	56	7	12	0	0	0	0	13	0	1	98	49	1	1	1	1	1	NA		0.867	0.133
SE	11899 DYNLL2	DYNLL2	chr17	+	58087081	58087222	58083418	58083683	58089141	58090257	18	11	9	2	0	0	1	4	0	0	98	49	1	1	0.818	1	1		1	1	-0.061
SE	11909 SUPT4H1	SUPT4H1	chr17	-	58347528	58347584	58347187	58347241	58351401	58351508	19	26	25	1	0	0	0	7	0	0	98	49	1	1	0.905	1	1	NA		1	-0.032
SE	11913 GAA	GAA	chr17	+	80112875	80113027	80112577	80112711	80113217	80113366	16	0	3	0	1	0	0	11	0	0	98	49	1	1	1	0	1	NA		1	-0.333
SE	11916 DHX40	DHX40	chr17	+	59566626	59566794	59565524	59565783	59570517	59570663	20	3	4	0	0	0	0	1	0	1	98	49	1	1	1	1	1	NA		0.333	0.667
SE	11920 VMP1	VMP1	chr17	+	59735337	59735473	59731420	59731522	59737452	59737543	112	95	120	1	0	0	2	24	0	0	98	49	1	1	0.982	1	1		1	1	-0.006
SE	11922 VMP1	VMP1	chr17	+	59738836	59738947	59735337	59735473	59764970	59765138	142	54	63	0	2	2	0	29	0	1	98	49	1	1	1	0.931	0.94	NA		0.935	0.022
SE	11923 VMP1	VMP1	chr17	+	59764970	59765138	59738836	59738947	59773753	59773885	299	134	131	0	2	0	0	47	0	0	98	49	1	1	1	0.971	1	NA		1	-0.01
SE	11924 VMP1	VMP1	chr17	+	59773753	59773885	59738836	59738947	59808795	59808876	237	109	110	0	1	0	2	43	0	0	98	49	1	1	1	0.982	1		1	1	-0.006
SE	11925 VMP1	VMP1	chr17	+	59808795	59808876	59773753	59773885	59811669	59811786	367	190	180	2	0	0	2	76	0	0	98	49	1	1	0.989	1	1		1	1	-0.004
SE	11926 VMP1	VMP1	chr17	+	59811669	59811786	59773753	59773885	59817711	59817773	90	72	55	0	0	0	1	26	0	1	98	49	1	1	1	1	1		1	0.929	0.035
SE	11927 VMP1	VMP1	chr17	+	59817711	59817773	59811669	59811786	59838294	59838397	187	135	114	0	2	0	1	36	0	0	98	49	1	1	1	0.971	1		1	1	-0.01
SE	11934 PPM1D	PPM1D	chr17	+	60656598	60656841	60647891	60648082	60662994	60666279	0	1	1	0	3	0	0	1	0	0	98	49	1	1	NA	0.143	1	NA		1	-0.428
SE	11936 KIAA1257	KIAA1257	chr3	-	128988727	128988918	128987615	128987829	128993042	128993336	4	0	0	0	1	0	0	1	0	0	98	49	1	1	1	0	NA	NA		1	-0.5
SE	11937 MED13	MED13	chr17	-	61955679	61955838	61955381	61955567	61956338	61956481	9	5	1	1	0	0	0	2	0	1	98	49	1	1	0.818	1	1	NA		0.5	0.439
SE	11939 MAP7D1	MAP7D1	chr1	+	36176198	36176581	36173363	36173478	36176696	36176842	29	7	6	0	1	0	8	13	0	0	98	49	1	1	1	0.778	1		1	1	-0.074
SE	11951 MAP3K3	MAP3K3	chr17	+	63634708	63634801	63632680	63632802	63646033	63646074	44	24	10	11	2	0	0	4	0	2	98	49	1	1	0.667	0.857	1	NA		0.5	0.341
SE	11952 MAP3K3	MAP3K3	chr17	+	63666939	63667060	63657793	63657907	63681765	63681899	39	30	19	1	0	0	0	5	0	0	98	49	1	1	0.951	1	1	NA		1	-0.016
SE	11953 MAP3K3	MAP3K3	chr17	+	63688788	63688881	63688526	63688594	63689543	63689735	57	21	13	1	0	0	0	13	0	0	98	49	1	1	0.966	1	1	NA		1	-0.011
SE	11954 MAP3K3	MAP3K3	chr17	+	63692241	63692419	63691732	63691862	63693548	63696310	53	28	15	1	0	0	0	8	0	0	98	49	1	1	0.964	1	1	NA		1	-0.012
SE	11957 CHD3	CHD3	chr17	+	7907600	7907702	7907352	7907488	7907893	7908019	8	0	2	2	1	1	0	0	0	2	98	49	1	1	0.667	0	0.5	NA		0	0.389
SE	11958 STRADA	STRADA	chr17	-	63707246	63707418	63706634	63706739	63710490	63710614	1	3	1	3	0	0	0	2	0	0	98	49	1	1	0.143	1	1	NA		1	-0.286
SE	11962 SP140	SP140	chr2	+	230238212	230238381	230237082	230237260	230241403	230241487	31	6	0	2	0	0	1	9	0	0	98	49	1	1	0.886	1	NA		1	1	-0.057
SE	11968 SP140	SP140	chr2	+	230292645	230292788	230290459	230290564	230294270	230294318	4	0	4	3	1	0	0	2	0	0	98	49	1	1	0.4	0	1	NA		1	-0.533
SE	11969 SMARCD2	SMARCD2	chr17	-	63833297	63833420	63833068	63833170	63833586	63833722	17	3	7	1	0	0	0	3	0	0	98	49	1	1	0.895	1	1	NA		1	-0.035
SE	11980 FTH1	FTH1	chr11	-	61964986	61965112	61964284	61964891	61965368	61965515	399	143	55	0	1	0	8	114	0	1	98	49	1	1	1	0.986	1		1	0.983	0.004
SE	11995 CEP95	CEP95	chr17	+	64516722	64516828	64514247	64514358	64519320	64519436	0	0	1	0	0	0	0	0	0	1	98	49	1	1	NA	NA	1	NA		0	1
SE	11996 PLEKHM1P	PLEKHM1P	chr17	-	64804829	64805100	64799999	64800917	64821766	64822052	18	11	6	1	0	0	0	3	0	0	98	49	1	1	0.9	1	1	NA		1	-0.033
SE	11998 LRRC37A3	LRRC37A3	chr17	-	64889680	64889752	64869094	64869166	64892528	64892600	1	0	0	0	0	0	0	0	0	1	98	49	1	1	1	NA	NA	NA		0	1
SE	12002 ITGB2-AS1	ITGB2-AS1	chr21	+	44922912	44922983	44921034	44921327	44926867	44927004	2	0	4	2	0	0	0	2	0	0	98	49	1	1	0.333	NA	1	NA		1	-0.334
SE	12003 ITGB2-AS1	ITGB2-AS1	chr21	+	44927624	44927663	44926867	44927004	44927853	44929680	0	0	1	0	1	0	0	0	0	1	88	49	1	1	NA	0	1	NA		0	0.5
SE	12004 ITGB2-AS1	ITGB2-AS1	chr21	+	44927624	44927667	44926867	44927004	44927853	44929680	2	0	1	0	1	0	0	0	0	1	92	49	1	1	1	0	1	NA		0	0.667
SE	12006 UMAD1	UMAD1	chr7	+	7676101	7676208	7673308	7673453	7801669	7801743	1	1	1	0	0	0	0	0	0	1	98	49	1	1	1	1	1	NA		0	1
SE	12007 HELZ	HELZ	chr17	-	67135969	67136198	67128650	67128855	67137930	67138114	12	1	0	2	0	0	0	4	0	0	98	49	1	1	0.75	1	NA	NA		1	-0.125
SE	12008 HELZ	HELZ	chr17	-	67149866	67149985	67148568	67148714	67151045	67151224	13	7	0	0	2	0	0	5	0	0	98	49	1	1	1	0.636	NA	NA		1	-0.182
SE	12009 HELZ	HELZ	chr17	-	67167462	67167796	67166477	67166608	67178658	67178926	16	7	5	0	1	0	0	2	0	0	98	49	1	1	1	0.778	1	NA		1	-0.074
SE	12010 SYS1	SYS1	chr20	+	45363528	45363693	45363099	45363315	45365618	45365686	1	0	0	1	0	0	1	0	0	0	98	49	1	1	0.333	NA	NA		1	NA	-0.667
SE	12019 KLHL2	KLHL2	chr4	+	165228806	165228913	165219933	165220059	165238777	165238899	8	2	8	3	4	3	0	4	0	0	98	49	1	1	0.571	0.2	0.571	NA		1	-0.553
SE	12023 PRKAR1A	PRKAR1A	chr17	+	68514986	68515119	68512378	68512548	68515393	68515576	2	0	0	26	8	2	0	3	0	1	98	49	1	1	0.037	0	0	NA		0.6	-0.588
SE	12024 PRKAR1A	PRKAR1A	chr17	+	68525753	68525912	68524911	68524958	68527839	68527900	98	42	39	0	0	4	0	5	0	0	98	49	1	1	1	1	0.83	NA		1	-0.057
SE	12025 WIPI1	WIPI1	chr17	-	68434555	68434626	68433467	68433575	68435619	68435712	15	3	0	0	2	0	0	4	0	0	98	49	1	1	1	0.429	NA	NA		1	-0.285
SE	12026 WIPI1	WIPI1	chr17	-	68452909	68452992	68450727	68450897	68457341	68457512	18	7	3	0	0	1	0	1	0	0	98	49	1	1	1	1	0.6	NA		1	-0.133

SE	12047	FAM104A	FAM104A	chr17	-	73227164	73227264	73207352	73209768	73232085	73232394	5	0	0	1	0	0	0	1	1	0	98	49	1	1	0.714	NA		NA			0		1	0.214
SE	12048	ANXA7	ANXA7	chr10	-	73379878	73379954	73378910	73379023	73380030	73380201	32	7	16	1	0	0	0	9	0	0	98	49	1	1	0.941		1		1	NA			1	-0.02
SE	12049	ANXA7	ANXA7	chr10	-	73396043	73396109	73388311	73388414	73396518	73396583	0	4	0	5	3	3	0	0	0	1	98	49	1	1	0		0.4		0	NA			0	0.133
SE	12050	CTAGE5	CTAGE5	chr14	+	39291018	39291096	39279448	39279537	39293270	39293381	6	8	2	0	0	1	0	5	0	0	98	49	1	1	1		1		0.5	NA			1	-0.167
SE	12051	CTAGE5	CTAGE5	chr14	+	39291018	39291111	39279448	39279537	39293270	39293381	5	3	2	0	0	1	0	2	0	0	98	49	1	1	1		1		0.5	NA			1	-0.167
SE	12052	CTAGE5	CTAGE5	chr14	+	39308448	39308587	39304290	39304381	39313339	39313441	14	4	3	1	0	0	0	4	0	0	98	49	1	1	0.875		1		1	NA			1	-0.042
SE	12076	ZNF200	ZNF200	chr16	-	3233505	3233836	3232832	3232921	3234986	3235456	4	3	0	2	0	0	0	1	0	0	98	49	1	1	0.5		1	NA		NA			1	-0.25
SE	12093	IQSEC1	IQSEC1	chr3	-	12902772	12902822	12897042	12901522	12908348	12908525	4	2	3	14	2	5	0	2	0	3	98	49	1	1	0.125		0.333		0.231	NA			0.25	-0.02
SE	12094	IQSEC1	IQSEC1	chr3	-	12920429	12920596	12915593	12915733	12922119	12922242	72	30	16	1	0	0	3	24	0	0	98	49	1	1	0.973		1		1		1		1	-0.009
SE	12098	SAP30BP	SAP30BP	chr17	+	75693439	75693482	75671815	75671863	75699782	75699871	32	13	6	0	2	0	1	5	0	0	92	49	1	1	1		0.776		1		1		1	-0.075
SE	12101	WBP2	WBP2	chr17	-	75851567	75851676	75849603	75849739	75855238	75855420	33	21	8	0	2	0	2	6	0	0	98	49	1	1	1		0.84		1		1		1	-0.053
SE	12106	HSPA4	HSPA4	chr5	+	133091192	133091374	133089561	133089695	133092699	133092789	12	1	0	1	0	0	0	3	0	0	98	49	1	1	0.857		1	NA		NA			1	-0.072
SE	12110	EXOC7	EXOC7	chr17	-	76090328	76090397	76089174	76089320	76094413	76094581	1	0	0	4	0	1	0	2	0	0	98	49	1	1	0.111	NA			0	NA			1	-0.945
SE	12123	PATL1	PATL1	chr11	-	59651543	59651641	59650753	59650813	59652463	59652587	23	5	4	1	0	0	0	7	0	0	98	49	1	1	0.92		1		1	NA			1	-0.027
SE	12124	PATL1	PATL1	chr11	-	59655955	59656045	59655522	59655740	59656498	59656600	5	1	2	0	3	2	0	3	0	0	98	49	1	1	1		0.143		0.333	NA			1	-0.508
SE	12125	JMJD6	JMJD6	chr17	-	76723771	76724058	76721797	76721933	76725466	76725855	25	9	1	0	1	0	0	4	0	1	98	49	1	1	1		0.818		1	NA			0.667	0.272
SE	12137	EPB41L3	EPB41L3	chr18	-	5433902	5434121	5433468	5433556	5438034	5438110	3	0	0	0	0	0	0	2	0	1	98	49	1	1	1	NA		NA		NA			0.5	0.5
SE	12141	PGS1	PGS1	chr17	+	78392475	78392665	78378617	78378808	78396307	78396385	31	24	7	1	0	0	0	4	0	0	98	49	1	1	0.939		1		1	NA			1	-0.02
SE	12142	PGS1	PGS1	chr17	+	78396307	78396385	78392475	78392665	78398251	78398351	31	34	8	2	5	1	0	5	0	1	98	49	1	1	0.886		0.773		0.8	NA			0.714	0.106
SE	12143	PGS1	PGS1	chr17	+	78400676	78400855	78399347	78399537	78403567	78404089	48	30	19	1	0	0	0	13	0	0	98	49	1	1	0.96		1		1	NA			1	-0.013
SE	12144	PGS1	PGS1	chr17	+	78400734	78400855	78399347	78399537	78403567	78404089	30	17	5	1	0	0	0	9	0	0	98	49	1	1	0.937		1		1	NA			1	-0.021
SE	12147	DCP2	DCP2	chr5	+	113003941	113004077	113001566	113001674	113007937	113008042	22	10	10	5	1	1	0	10	0	0	98	49	1	1	0.687		0.833		0.833	NA			1	-0.216
SE	12149	ATG7	ATG7	chr3	+	11299361	11299416	11298685	11298855	11306942	11307060	67	30	7	0	0	0	0	7	1	0	98	49	1	1	1		1		1		0		1	0.5
SE	12151	ATG7	ATG7	chr3	+	11332971	11333093	11331339	11331428	11340644	11340735	11	10	1	1	0	0	1	6	0	0	98	49	1	1	0.846		1		1		1		1	-0.051
SE	12152	ATG7	ATG7	chr3	+	11342134	11342279	11340644	11340735	11347876	11348035	13	12	7	0	1	0	0	16	0	1	98	49	1	1	1		0.857		1	NA			0.889	0.063
SE	12153	ATG7	ATG7	chr3	+	11347876	11348035	11340644	11340735	11358417	11358612	8	8	1	0	1	0	0	5	0	0	98	49	1	1	1		1		0.333	NA			1	-0.222
SE	12157	CYTH1	CYTH1	chr17	-	78692416	78692493	78680970	78681042	78698268	78698380	28	5	3	0	1	0	0	7	0	0	98	49	1	1	1		0.714		1	NA			1	-0.095
SE	12159	TYMP	TYMP	chr22	-	50526000	50526141	50525752	50525918	50526245	50526476	33	36	25	0	1	0	8	30	1	0	98	49	1	1	1		0.947		1		0.8		1	0.082
SE	12160	TYMP	TYMP	chr22	-	50526000	50526156	50525752	50525918	50526245	50526476	22	22	12	0	1	0	3	17	1	0	98	49	1	1	1		0.917		1		0.6		1	0.172
SE	12167	EIF4A3	EIF4A3	chr17	-	80141318	80141381	80140007	80140140	80141781	80141848	19	2	1	0	2	0	0	3	0	0	98	49	1	1	1		0.333		1	NA			1	-0.222
SE	12170	RNF213	RNF213	chr17	+	80339200	80340356	80337832	80337997	80343131	80343325	67	45	9	0	1	0	4	50	0	0	98	49	1	1	1		0.957		1		1		1	-0.014
SE	12171	RNF213	RNF213	chr17	+	80349769	80349906	80344677	80348286	80350300	80350396	91	49	5	0	2	0	3	93	0	0	98	49	1	1	1		0.925		1		1		1	-0.025
SE	12172	RNF213	RNF213	chr17	+	80352939	80353059	80351684	80351803	80353511	80353666	37	34	4	1	1	0	1	35	0	0	98	49	1	1	0.949		1		1		1		1	-0.017
SE	12173	RNF213	RNF213	chr17	+	80360060	80360206	80358287	80358479	80361733	80361888	73	31	11	0	1	0	1	46	0	0	98	49	1	1	1		0.939		1		1		1	-0.02
SE	12174	RNF213	RNF213	chr17	+	80361733	80361888	80360060	80360206	80363101	80363314	74	53	13	0	1	0	2	52	0	0	98	49	1	1	1		0.964		1		1		1	-0.012
SE	12175	RNF213	RNF213	chr17	+	80363101	80363314	80361733	80361888	80363608	80363790	98	69	11	1	0	0	15	88	0	0	98	49	1	1	0.98		1		1		1		1	-0.007
SE	12177	RNF213	RNF213	chr17	+	80379619	80379714	80377761	80377796	80380830	80380987	78	29	10	0	0	0	3	53	0	1	98	49	1	1	1		1		1		1		0.964	0.018
SE	12178	RNF213	RNF213	chr17	+	80385038	80385171	80383676	80383928	80385537	80385621	58	14	11	3	2	0	0	23	0	2	98	49	1	1	0.906		0.778		1	NA			0.852	0.043
SE	12180	RNF213	RNF213	chr17	+	80390011	80390196	80389827	80389917	80393344	80398781	120	54	11	0	0	0	0	79	1	1	98	49	1	1	1		1		1		0		0.975	0.512
SE	12183	AATK	AATK	chr17	-	81131060	81131205	81128469	81128549	81134367	81134501	20	20	6	2	0	0	0	4	0	0	98	49	1	1	0.833		1		1	NA			1	-0.056
SE	12186	CD36	CD36	chr7	+	80662989	80663169	80661062	80661210	80664405	80664497	28	0	0	2	0	0	1	28	0	2	98	49	1	1	0.875	NA		NA			1		0.875	-0.062
SE	12188	NPLOC4	NPLOC4	chr17	-	81610209	81610258	81608727	81608822	81613317	81613494	13	9	0	0	0	3	0	4	0	0	98	49	1	1	1		1		0	NA			1	-0.333
SE	12191	SP110	SP110	chr2	-	230170620	230170761	230168925	230169237	230172065	230172174	12	0	1	0	2	0	0	1	0	2	98	49	1	1	1		0		1	NA			0.2	0.467
SE	12193	SP110	SP110	chr2	-	230177537	230177680	230176472	230176724	230178156	230178255	23	18	11	0	0	0	0	5	0	2	98	49	1	1	1		1		1	NA			0.556	0.444
SE	12199	ARAP3	ARAP3	chr5	-	141656503	141656637	141656193	141656276	141656717	141656846	14	16	4	0	0	0	0	2	0	1	98	49	1	1	1		1		1	NA			0.5	0.5
SE	12201	ARAP3	ARAP3	chr5	-	141669921	141670063	141669708	141669811	141670511	141670628	6	8	3	1	0	0	2	0	0	0	98	49	1	1	0.75		1		1		1	NA		-0.083
SE	12202	ARAP3	ARAP3	chr5	-	141671894	141671980	141671569	141671752	141672551	141672658	11	4	1	1	0	0	0	4	0	0	98	49	1	1	0.846		1		1	NA			1	-0.051
SE	12203	ARAP3	ARAP3	chr5	-	141672101	141672301	141671894	141671980	141672551	141672658	15	11	2	0	0	0	0	1	0	1	98	49	1	1	1		1		1	NA			0.333	0.667
SE	12205	ARHGDIA	ARHGDIA	chr17	-	81869329	81869406	81869172	81869236	81869541	81869625	29	11	4	1	0	0	0	21	0	1	98	49	1	1	0.935		1		1	NA			0.913	0.065
SE	12208	PCYT2	PCYT2	chr17	-	81908875	81909035	81908567	81908634	81909513	81909602	6	0	0	1	0	0	0	2	0	0	98	49	1	1	0.75	NA		NA		NA			1	-0.25
SE	12209	PCYT2	PCYT2	chr17	-	81908875	81909037	81908567	81908634	81909513	81909602	7	0	0	1	0	0	0	2	0	0	98	49	1	1	0.778	NA		NA		NA			1	-0.222
SE	12210	PCYT2	PCYT2	chr17	-	81908875	81909321	81908567	81908634	81909513	81909602	6	0	2	1	0	0	0	2	0	0	98	49	1	1	0.75	NA			1	NA			1	-0.125
SE	12213	GPR155	GPR155	chr2	-	174482872	174482918	174481496	174481987	174486872	174487088	3	2	3	2	0	0	0	0	0	3	95	49	1	1	0.436		1		1	NA			0	0.812
SE	12214	GPR155	GPR155	chr2	-	174483009	174483158	174481496	174481987	174486872	174487088	3	0	0	2	0	0	0	0	0	3	98	49	1	1	0.429	NA		NA		NA			0	0.429
SE	12215	IL1R1	IL1R1	chr2	+	102157718	102157785	102153940	102154017	102164773	102165008	5	2	2	2	0	0	0	1	0	0	98	49	1	1	0.556		1		1	NA			1	-0.148
SE	12216	IL1R1	IL1R1	chr2	+	102166112	102166281	102165114	102165304	102168597	102168663	9	4	0	1	3	0	0	2	0	0	98	49	1	1	0.818		0.4	NA		NA			1	-0.391
SE	12219	HSDL1	HSDL1	chr16	-	84130336	84130431	84129985	84130171	84131101	84131327	0	1	0	0	1	0	0	1	0	0	98	49	1	1	NA		0.333	NA		NA			1	-0.667
SE	12233	NR4A2	NR4A2	chr2	-	156328403	156328533	156327850	156328014	156329322	156330188	4	0	0	0	0	0	0	1	0	1	98	49	1	1	1	NA		NA		NA			0.333	0.667
SE	12235	SMCHD1	SMCHD1	chr18	+	2673280	2673363	2666869	2667031	2688393	2688508	216	145	39	0	2	0	5	46	0	0	98	49	1	1	1		0.973		1		1		1	-0.009
SE	12239	SMCHD1	SMCHD1	chr18	+	2707562	2707645	2706363	2706470	2707806	2707920	430	242	69	0	1	0	2	82	0	0	98	49	1	1	1		0.992		1		1		1	-0.003
SE	12242	SMCHD1	SMCHD1	chr18	+	2722518	2722663	2718314	2718434	2726451	2726524	103	62	32	2	0	0	0	26	0	0	98	49	1	1	0.963		1		1	NA			1	-0.012
SE	12243	SMCHD1	SMCHD1	chr18	+	2724898	2724995	2718314	2718434	2726451	2726524	217	108	24	2	0	0	1	40	0	0	98	49	1	1	0.982		1		1		1		1	-0.006
SE	12244	SMCHD1	SMCHD1	chr18	+	2724898	2724995	2722518	2722663	2726451	2726524	282	123	55	1	1	0	2	53	0	1	98	49	1	1	0.993		0.984		1		1		0.964	0.01
SE	12245	SMCHD1	SMCHD1	chr18	+	2732264	2732492	2729274	2729409	2738396	2738545	279	95	47	0	1	0	1	52	0	1	98	49	1	1	1		0.979		1		1		0.963	0.011
SE	12246	SMCHD1	SMCHD1	chr18	+	2740702	2740821	2739431	2739520	2743760	2743928	427	196	66	1	0	0	3	91	0	0	98	49	1	1	0.995		1		1		1		1	-0.002
SE	12249	SMCHD1	SMCHD1	chr18	+	2760651	2760739	2752487	2752552	2762104	2762236	286	142	51	1	0	0	2	50	0	0	98	49	1	1	0.993		1		1		1		1	-0.002
SE	12251	SMCHD1	SMCHD1	chr18	+	2775733	2775924	2772249	2772372	2777805	2777915	335	159	66	3	0	0	1	70	0	0	98	49	1	1	0.982		1		1		1		1	-0.006
SE	12253	LPIN2	LPIN2	chr18	-	2923774	2923861	2922046	2922199	2924397	2924546	12	8	4	1	0	0	0	7	0	0	98	49	1	1	0.857		1		1	NA			1	-0.048
SE	12254	LPIN2	LPIN2	chr18	-	2925223	2925368	2924397	2924546	2926722	2926805	16	7	3	1	0	0	0	2	0	0	98	49	1	1	0.889		1		1	NA			1	-0.037
SE	12255	FBXO38	FBXO38	chr5	+	148427212	148427722	148425521	148425701	148433423	148433524	23	2	0	0	0	0	3	2	0	2	98	49	1	1	1		1	NA			1		0.333	0.334
SE	12256	FBXO38	FBXO38	chr5	+	148439646	148439792	148438331	148438498	148440423	148440527	24	3	2	2	3	0	0	1	0	0	98	49	1	1	0.857		0.333		1	NA			1	-0.27
SE	12264	JARID2	JARID2	chr6	+	15452005	15452175	15410223	15410365	15468541	15468718	39	22	8	1	0	0	0	10	0	0	98	49	1	1	0.951		1		1	NA			1	-0.016
SE	12265	JARID2	JARID2	chr6	+	15496131	15497170	15487306	15487542	15500906	15501409	10	10	5	1	0	0	0	5	0	0	98	49	1	1	0.833		1		1	NA			1	-0.056
SE	12272	PPP4R1	PPP4R1	chr18	-	9553321	9553422	9550277	9550398	9557220	9557382	19	16	27	1	0	0	0	12	0	0	98	49	1	1	0.905		1		1	NA			1	-0.032
SE	12273	PPP4R1	PPP4R1	chr18	-	9562921	9563046	9561979	9562075	9563377	9563550	15	19	5	16	14	10	0	4	0	5	98	49	1	1	0.319		0.404		0.2	NA			0.286	0.022
SE	12274	PPP4R1	PPP4R1	chr18	-	9593767	9593874	9588710	9588853	9595017	9595102	77	43	31	1	3	2	0	10	0	0	98	49	1	1	0.975		0.878		0.886	NA			1	-0.087
SE	12279	MPPE1	MPPE1	chr18	-	11884878	11885043	11883470	11884627	11885675	11885816	20	3	3	26	17	5	0	3	0	2	98	49	1	1	0.278		0.081		0.231	NA			0.429	-0.232
SE	12280	MPPE1	MPPE1	chr18	-	11885675	11885816	11883470	11884627	11886916	11887025	26	18	7	2	0	0	0	2	0	0	98	49	1	1	0.867		1		1	NA			1	-0.044
SE	12281	MPPE1	MPPE1	chr18	-	11886498	11886621	11885675	11885816	11886916	11887025	14	4	4	0	1	2	0	1	0	0	98	49	1	1	1		0.667		0.5	NA			1	-0.278
SE	12282	MPPE1	MPPE1	chr18	-	11886712	11886778	11886498	11886621	11886916	11887025	10	8	2	2	0	1	0	1	0	1	98	49	1	1	0.714		1		0.5	NA			0.333	0.405
SE	12283	MPPE1	MPPE1	chr18	-	11886712	11886781	11886498	11886621	11886916	11887025	7	11	3	2	0	1	0	1	0	1	98	49	1	1	0.636		1		0.6	NA			0.333	0.412
SE	12285	MPPE1	MPPE1	chr18	-	11906202	11906309	11896983	11897356	11908200	11908797	17	11	8	0	1	0	1	1	0	0	98	49	1	1	1		0.846		1		1		1	-0.051
SE	12286	SLC11A1	SLC11A1	chr2	+	218382959	218383102	218382028	218382375	218384242	218384365	55	28	4	9	3	3	0	13	0	0	98	49	1	1	0.753		0.824		0.4	NA			1	-0.341
SE	12287	SLC11A1	SLC11A1	chr2	+	218382959	218383102	218382028	218382375	218385146	218385266	27	17	0	1	0	0	0	2	0	0	98	49	1	1	0.931		1	NA		NA			1	-0.034
SE	12288	SLC11A1	SLC11A1	chr2	+	218384242	218384365	218382028	218382375	218385146	218385266	106	58	23	1	0	0	2	27	0	0	98	49	1	1	0.981		1		1		1		1	-0.006
SE	12289	SLC11A1	SLC11A1	chr2	+	218384242	218384365	218382959	218383102	218385146	218385266	125	67	24	0	1	0	2	38	0	0	98	49	1	1	1		0.971		1		1		1	-0.01
SE	12294	PSMG2	PSMG2	chr18	+	12706549	12706721	12702987	12703164	12712701	12712760	3	2	4	0	1	0	0	1	0	0	98	49	1	1	1		0.5		1	NA			1	-0.167
SE	12304	ESCO1	ESCO1	chr18	-	21567979	21568094	21566145	21566206	21573313	21575430	14	4	7	1	0	0	0	2	0	0	98	49	1	1	0.875		1		1	NA			1	-0.042
SE	12306	TRAPPC11	TRAPPC11	chr4	+	183682731	183682825	183680119	183680267	183683974	183684054	11	3	0	2	0	0	0	1	0	0	98	49	1	1	0.733		1	NA		NA			1	-0.133
SE	12307	TRAPPC11	TRAPPC11	chr4	+	183686617	183686748	183685270	183685403	183691315	183691471	12	2	3	1	0	0	0	3	0	0	98	49	1	1	0.857		1		1	NA			1	-0.048
SE	12308	ABHD3	ABHD3	chr18	-	21656860	21657026	21650896	21651763	21657103	21657152	57	33	17	1	0	0	0	13	0	0	98	49	1	1	0.966		1		1	NA			1	-0.011
SE	12309	ABHD3	ABHD3	chr18	-	21664117	21664230	21659169	21659343	21702315	21702498	13	3	2	2	1	0	0	2	0	1	98	49	1	1	0.765		0.6		1	NA			0.5	0.288
SE	12310	ABHD3	ABHD3	chr18	-	21683919	21683965	21664117	21664230	21702315	21702498	38	13	8	4	0	1	0	6	0	0	95	49	1	1	0.831		1		0.805	NA			1	-0.121
SE	12319	TTC39C	TTC39C	chr18	+	24069156	24069271	24066011	24066140	24080584	24080939	20	6	2	1	0	0	1	2	0	0	98	49	1	1	0.909		1		1		1		1	-0.03
SE	12321	CARS	CARS	chr11	-	3017856	3017954	3017105	3017295	3018407	3018511	11	4	0	1	0	0	0	0	0	1	98	49	1	1	0.846		1	NA		NA			0	0.923
SE	12322	CARS	CARS	chr11	-	3026675	3026797	3020219	3020332	3028995	3029084	20	13	9	2	0	0	0	7	0	0	98	49	1	1	0.833		1		1	NA			1	-0.056
SE	12323	CARS	CARS	chr11	-	3028995	3029084	3026675	3026797	3029302	3029443	35	15	9	2	0	0	0	10	0	0	98	49	1	1	0.897		1		1	NA			1	-0.034
SE	12324	CARS	CARS	chr11	-	3040895	3040984	3039834	3039931	3042164	3042256	11	6	0	0	1	0	0	3	0	0	98	49	1	1	1		0.75	NA		NA			1	-0.125
SE	12338	MAPK1	MAPK1	chr22	-	21788693	21788808	21788256	21788388	21799011	21799128	17	3	28	0	0	0	0	7	0	1	98	49	1	1	1		1		1	NA			0.778	0.222
SE	12342	TRAPPC8	TRAPPC8	chr18	-	31913368	31913522	31909666	31909760	31916271	31916446	29	9	11	2	1	0	0	3	0	0	98	49	1	1	0.879		0.818		1	NA			1	-0.101
SE	12347	IQCB1	IQCB1	chr3	-	121788283	121788432	121781742	121781874	121790072	121790215	2	0	0	0	1	0	0	3	0	0	98	49	1	1	1		0	NA		NA			1	-0.5
SE	12354	RPRD1A	RPRD1A	chr18	-	36040806	36040865	36033707	36033837	36067253	36067594	4	4	0	1	4	2	0	2	0	0	98	49	1	1	0.667		0.333		0	NA			1	-0.667
SE	12360	PIK3C3	PIK3C3	chr18	+	41957569	41957758	41955197	41955359	41962488	41962632	34	11	1	0	0	4	0	2	0	0	98	49	1	1	1		1		0.111	NA			1	-0.296
SE	12361	PIK3C3	PIK3C3	chr18	+	41987811	41987898	41970326	41970456	41990458	41990554	26	17	0	1	0	0	0	2	0	0	98	49	1	1	0.929		1	NA		NA			1	-0.035
SE	12367	MAN2B2	MAN2B2	chr4	+	6598197	6598354	6597112	6597303	6600622	6600756	2	1	2	0	0	0	0	0	0	1	98	49	1	1	1		1		1	NA			0	1
SE	12372	GPCPD1	GPCPD1	chr20	-	5559939	5560076	5558683	5558819	5561464	5561530	106	59	12	1	0	0	0	16	0	0	98	49	1	1	0.981		1		1	NA			1	-0.006
SE	12373	GPCPD1	GPCPD1	chr20	-	5570146	5570239	5567482	5567560	5573914	5573969	77	21	12	0	1	0	0	14	0	1	98	49	1	1	1		0.913		1	NA			0.875	0.096
SE	12374	GPCPD1	GPCPD1	chr20	-	5580007	5580131	5578379	5578611	5584280	5584322	123	47	12	0	0	0	0	17	0	1	98	49	1	1	1		1		1	NA			0.895	0.105
SE	12375	GPCPD1	GPCPD1	chr20	-	5586193	5586269	5584280	5584322	5593326	5593411	32	19	2	29	13	6	0	11	0	2	98	49	1	1	0.356		0.422		0.143	NA			0.733	-0.426
SE	12376	GPCPD1	GPCPD1	chr20	-	5593326	5593411	5584280	5584322	5598724	5598821	191	66	20	0	0	0	1	27	0	1	98	49	1	1	1		1		1		1		0.931	0.034
SE	12381	PIAS2	PIAS2	chr18	-	46844733	46844839	46844053	46844127	46846706	46846841	0	0	0	1	0	0	0	2	0	0	98	49	1	1	0	NA		NA		NA			1	-1
SE	12383	ATG2A	ATG2A	chr11	-	64896461	64896616	64895289	64895442	64896747	64896869	23	19	1	1	0	0	0	5	0	0	98	49	1	1	0.92		1		1	NA			1	-0.027
SE	12384	ATG2A	ATG2A	chr11	-	64900883	64901092	64900493	64900629	64901961	64902176	16	9	0	0	0	0	0	0	0	1	98	49	1	1	1		1	NA		NA			0	1
SE	12385	ATG2A	ATG2A	chr11	-	64902515	64902680	64902259	64902386	64903287	64903364	5	4	0	0	1	0	1	0	0	0	98	49	1	1	1		0.667	NA			1	NA		-0.166
SE	12386	ATG2A	ATG2A	chr11	-	64906112	64906193	64905741	64905848	64906333	64906533	23	20	4	0	1	0	1	7	0	0	98	49	1	1	1		0.909		1		1		1	-0.03
SE	12387	ATG2A	ATG2A	chr11	-	64906664	64906815	64906333	64906533	64907254	64907439	20	14	4	1	0	0	2	5	0	0	98	49	1	1	0.909		1		1		1		1	-0.03
SE	12388	ATG2A	ATG2A	chr11	-	64908990	64909150	64907747	64907890	64909270	64909367	24	18	9	0	5	0	0	2	0	0	98	49	1	1	1		0.643		1	NA			1	-0.119
SE	12389	ATG2A	ATG2A	chr11	-	64909270	64909367	64907747	64907890	64909680	64909924	11	10	3	0	1	0	0	6	0	0	98	49	1	1	1		0.833		1	NA			1	-0.056
SE	12390	ATG2A	ATG2A	chr11	-	64910615	64910708	64910039	64910195	64910806	64910954	43	46	13	0	1	0	8	7	0	0	98	49	1	1	1		0.958		1		1		1	-0.014
SE	12391	ATG2A	ATG2A	chr11	-	64912326	64912423	64912084	64912249	64913037	64913136	36	27	6	0	0	0	2	5	0	1	98	49	1	1	1		1		1		1		0.714	0.143
SE	12393	DYM	DYM	chr18	-	49391592	49391645	49379664	49379758	49430254	49430447	16	11	1	1	0	0	3	1	0	0	98	49	1	1	0.889		1		1		1		1	-0.037
SE	12404	CLPTM1L	CLPTM1L	chr5	-	1330279	1330383	1325750	1325816	1331798	1331883	2	10	3	1	0	0	0	1	0	0	98	49	1	1	0.5		1		1	NA			1	-0.167
SE	12405	POLI	POLI	chr18	+	54280666	54280903	54277702	54277855	54282836	54283015	9	0	3	1	0	0	0	3	0	0	98	49	1	1	0.818	NA			1	NA			1	-0.091
SE	12406	RAB27B	RAB27B	chr18	+	54877566	54877738	54828476	54828700	54879368	54879454	10	0	0	1	0	0	1	0	0	0	98	49	1	1	0.833	NA		NA			1	NA		-0.167
SE	12411	ALAS1	ALAS1	chr3	+	52205838	52206023	52204692	52204915	52206571	52206751	2	3	9	1	0	0	0	2	0	0	98	49	1	1	0.5		1		1	NA			1	-0.167
SE	12414	WSB1	WSB1	chr17	+	27301787	27301956	27294079	27294435	27303366	27303635	47	23	0	7	7	0	0	12	0	1	98	49	1	1	0.77		0.622	NA		NA			0.857	-0.161
SE	12416	WSB1	WSB1	chr17	+	27310060	27310174	27309099	27309272	27311508	27311616	54	17	7	1	0	0	0	17	0	0	98	49	1	1	0.964		1		1	NA			1	-0.012
SE	12434	DYRK1A	DYRK1A	chr21	+	37420298	37420384	37367556	37367628	37472683	37472880	35	14	4	3	0	2	0	2	0	2	98	49	1	1	0.854		1		0.5	NA			0.333	0.452
SE	12448	CFAP36	CFAP36	chr2	+	55522101	55522166	55519594	55519916	55523720	55523822	4	0	0	0	1	0	3	0	0	0	98	49	1	1	1		0	NA			1	NA		-0.5
SE	12450	NECAB2	NECAB2	chr16	+	83997215	83997269	83994608	83994688	83998204	83998317	2	2	4	0	1	0	0	3	0	0	98	49	1	1	1		0.5		1	NA			1	-0.167
SE	12452	RSBN1	RSBN1	chr1	-	113796185	113796303	113777670	113777808	113797362	113798036	1	0	0	4	4	0	0	0	0	1	98	49	1	1	0.111		0	NA		NA			0	0.056
SE	12458	TTN-AS1	TTN-AS1	chr2	+	178538697	178539890	178537360	178537691	178540106	178540169	11	4	3	1	0	0	2	1	0	0	98	49	1	1	0.846		1		1		1		1	-0.051
SE	12462	ZNF516	ZNF516	chr18	-	76378854	76380303	76371466	76371571	76441244	76443211	20	9	2	1	0	0	3	4	0	0	98	49	1	1	0.909		1		1		1		1	-0.03
SE	12463	ZNF516	ZNF516	chr18	-	76463027	76463141	76441244	76443211	76495143	76495190	1	0	0	0	0	0	0	0	1	0	98	49	1	1	1	NA		NA			0	NA		1
SE	12466	MBP	MBP	chr18	-	77066297	77066385	77012782	77017268	77105210	77105286	42	23	27	0	1	0	0	7	0	0	98	49	1	1	1		0.92		1	NA			1	-0.027
SE	12471	PANK3	PANK3	chr5	-	168563888	168564065	168561392	168561516	168566012	168566266	8	0	0	1	0	0	0	4	0	0	98	49	1	1	0.8	NA		NA		NA			1	-0.2
SE	12475	ATP9B	ATP9B	chr18	+	79348131	79348196	79347769	79347925	79359353	79359462	0	0	2	1	0	0	0	1	0	0	98	49	1	1	0	NA	0.5		1	NA			1	-0.5
SE	12479	TXNL4A	TXNL4A	chr18	-	79976717	79976787	79972866	79973856	79977597	79977701	0	0	0	8	0	0	0	1	0	0	98	49	1	1	0	NA		NA		NA			1	-1
SE	12488	RAB28	RAB28	chr4	-	13369870	13369965	13367722	13368650	13376544	13376622	0	0	0	1	2	0	0	2	0	0	98	49	1	1	0		0	NA		NA			1	-1
SE	12491	CLEC4A	CLEC4A	chr12	+	8125560	8125677	8123631	8123960	8129263	8129362	8	6	14	0	0	0	0	2	0	1	98	49	1	1	1		1		1	NA			0.5	0.5
SE	12495	CLEC4A	CLEC4A	chr12	+	8135584	8135736	8129263	8129362	8136787	8136903	12	2	3	0	0	9	0	4	0	0	98	49	1	1	1		1		0.143	NA			1	-0.286
SE	12497	ARID3A	ARID3A	chr19	+	932417	932742	929261	929896	960091	960164	12	4	9	1	0	0	1	2	0	0	98	49	1	1	0.857		1		1		1		1	-0.048
SE	12498	ARID3A	ARID3A	chr19	+	964832	965080	964247	964431	966571	966868	12	6	3	2	0	0	2	4	0	0	98	49	1	1	0.75		1		1		1		1	-0.083
SE	12499	ARID3A	ARID3A	chr19	+	964832	965080	964247	964431	968404	968503	8	3	3	0	0	0	1	1	0	1	98	49	1	1	1		1		1		1		0.333	0.334
SE	12500	ARID3A	ARID3A	chr19	+	966571	966868	964247	964431	968404	968503	17	4	3	0	0	0	0	0	0	1	98	49	1	1	1		1		1	NA			0	1
SE	12501	ARID3A	ARID3A	chr19	+	966571	966868	964832	965080	968404	968503	19	7	3	0	0	0	1	4	0	1	98	49	1	1	1		1		1		1		0.667	0.166
SE	12513	GPX4	GPX4	chr19	+	1106241	1106266	1105657	1105809	1106399	1106459	20	10	1	0	1	0	0	2	0	0	74	49	1	1	1		0.869		1	NA			1	-0.044
SE	12526	MKNK2	MKNK2	chr19	-	2043123	2043197	2042765	2042870	2043502	2043582	125	73	21	0	1	0	1	17	0	0	98	49	1	1	1		0.973		1		1		1	-0.009
SE	12527	MKNK2	MKNK2	chr19	-	2046185	2046283	2043502	2043582	2046366	2046468	123	61	23	0	2	0	4	17	0	0	98	49	1	1	1		0.938		1		1		1	-0.021
SE	12528	MOB3A	MOB3A	chr19	-	2085174	2085328	2078139	2078679	2096225	2096270	88	41	6	0	1	0	2	14	0	0	98	49	1	1	1		0.953		1		1		1	-0.016
SE	12530	GADD45B	GADD45B	chr19	+	2476528	2476630	2476124	2476402	2477028	2477251	32	45	18	0	2	0	7	19	0	0	98	49	1	1	1		0.918		1		1		1	-0.027
SE	12531	GADD45B	GADD45B	chr19	+	2477028	2477251	2476528	2476630	2477487	2478259	25	39	18	0	0	0	1	6	0	1	98	49	1	1	1		1		1		1		0.75	0.125
SE	12544	FBXL4	FBXL4	chr6	-	98917373	98917719	98905425	98905670	98926476	98927060	1	0	0	0	0	0	0	0	0	1	98	49	1	1	1	NA		NA		NA			0	1
SE	12546	PLIN3	PLIN3	chr19	-	4859825	4860024	4859589	4859672	4861328	4861411	18	17	13	0	1	0	0	10	0	0	98	49	1	1	1		0.895		1	NA			1	-0.035
SE	12548	KDM4B	KDM4B	chr19	+	5071009	5071059	5047475	5047669	5077366	5077470	32	20	10	0	2	0	0	3	0	0	98	49	1	1	1		0.833		1	NA			1	-0.056

SE	12549	KDM4B	KDM4B	chr19	+	5119652	5119852	5110621	5110818	5131075	5131545	17	23	1	0	1	0	0	3	0	0	98	49	1	1	1	0.92	1	NA	1	-0.027
SE	12551	SAFB	SAFB	chr19	+	5641739	5641946	5641593	5641658	5645336	5645399	24	17	4	1	0	0	0	3	0	0	98	49	1	1	0.923	1	1	NA	1	-0.026
SE	12552	SAFB	SAFB	chr19	+	5648988	5649499	5648015	5648043	5649925	5649975	13	2	0	0	0	0	0	1	0	3	98	49	1	1	1	1	NA	NA	0.143	0.857
SE	12559	RNF24	RNF24	chr20	-	3974331	3974400	3963874	3964024	4015436	4015569	41	23	21	11	3	0	0	6	0	1	98	49	1	1	0.651	0.793	1	NA	0.75	0.065
SE	12567	RFX2	RFX2	chr19	-	6008105	6008224	6007689	6007802	6010135	6010251	2	0	4	1	0	0	0	2	0	0	98	49	1	1	0.5	NA	1	NA	1	-0.25
SE	12569	OMA1	OMA1	chr1	-	58530600	58530729	58527260	58527335	58533952	58534060	13	5	8	3	0	0	0	1	0	0	98	49	1	1	0.684	1	1	NA	1	-0.105
SE	12572	VAV1	VAV1	chr19	+	6821790	6821859	6821621	6821680	6822220	6822329	84	32	11	0	1	0	4	4	0	0	98	49	1	1	1	0.941	1	1	1	-0.02
SE	12574	VAV1	VAV1	chr19	+	6826611	6826711	6825302	6825406	6828075	6828171	63	15	19	1	0	0	0	8	0	0	98	49	1	1	0.969	1	1	NA	1	-0.01
SE	12577	ADGRE1	ADGRE1	chr19	+	6897148	6897304	6896397	6896541	6897427	6897451	19	7	4	0	4	0	0	2	0	0	98	49	1	1	1	0.467	1	NA	1	-0.178
SE	12578	ADGRE1	ADGRE1	chr19	+	6924677	6924872	6921712	6921883	6926365	6926601	5	3	0	1	2	0	0	3	0	0	98	49	1	1	0.714	0.429	NA	NA	1	-0.428
SE	12580	KIAA0226L	KIAA0226L	chr13	-	46361440	46361573	46359485	46359631	46362537	46362598	99	18	15	2	0	0	0	6	0	0	98	49	1	1	0.961	1	1	NA	1	-0.013
SE	12581	KIAA0226L	KIAA0226L	chr13	-	46361440	46361573	46359485	46359631	46363114	46363213	64	9	9	2	1	0	0	6	0	1	98	49	1	1	0.941	0.818	1	NA	0.75	0.17
SE	12582	KIAA0226L	KIAA0226L	chr13	-	46362537	46362598	46359485	46359631	46363114	46363213	42	11	10	2	1	0	0	3	0	1	98	49	1	1	0.913	0.846	1	NA	0.6	0.32
SE	12583	KIAA0226L	KIAA0226L	chr13	-	46362537	46362598	46361440	46361573	46363114	46363213	83	20	16	8	0	0	0	3	0	0	98	49	1	1	0.838	1	1	NA	1	-0.054
SE	12590	KIAA0226L	KIAA0226L	chr13	-	46377889	46378005	46371940	46372597	46387133	46387500	10	3	4	5	3	0	0	1	0	0	98	49	1	1	0.5	0.333	1	NA	1	-0.389
SE	12591	PET100	PET100	chr19	+	7630572	7630659	7629784	7629860	7630822	7630846	34	9	13	0	1	0	0	9	0	0	98	49	1	1	1	0.818	1	NA	1	-0.061
SE	12592	PET100	PET100	chr19	+	7631231	7631321	7630822	7630846	7631472	7631624	2	0	1	32	10	5	0	0	0	8	98	49	1	1	0.03	0	0.091	NA	0	0.04
SE	12593	STXBP2	STXBP2	chr19	+	7640697	7640809	7639730	7639807	7640899	7641003	22	12	10	2	1	0	0	6	0	0	98	49	1	1	0.846	0.857	1	NA	1	-0.099
SE	12595	STXBP2	STXBP2	chr19	+	7640739	7640809	7639730	7639807	7640899	7641003	22	12	10	2	1	0	0	6	0	0	98	49	1	1	0.846	0.857	1	NA	1	-0.099
SE	12596	STXBP2	STXBP2	chr19	+	7641704	7641853	7640899	7641003	7642033	7642118	55	38	17	0	2	0	2	13	0	0	98	49	1	1	1	0.905	1	1	1	-0.032
SE	12598	STXBP2	STXBP2	chr19	+	7644878	7644934	7644613	7644752	7645196	7645306	2	0	0	42	50	6	0	0	3	13	98	49	1	1	0.023	0	0	0	0	0.008
SE	12599	STXBP2	STXBP2	chr19	+	7647353	7647511	7647161	7647247	7647724	7647874	16	11	10	1	1	0	0	14	0	0	98	49	1	1	0.889	0.846	1	NA	1	-0.088
SE	12629	DNMT1	DNMT1	chr19	-	10180186	10180234	10177291	10177367	10180349	10180569	4	0	0	2	0	0	0	0	1	0	97	49	1	1	0.503	NA	NA	0	NA	0.503
SE	12632	ALOX5	ALOX5	chr10	+	45395854	45395936	45382482	45382681	45412190	45412313	225	79	37	0	2	0	5	67	0	1	98	49	1	1	1	0.952	1	1	0.971	-0.002
SE	12633	ALOX5	ALOX5	chr10	+	45428617	45428764	45424959	45425132	45440429	45440633	148	49	46	1	0	0	9	46	0	1	98	49	1	1	0.987	1	1	1	0.958	0.017
SE	12637	TYK2	TYK2	chr19	-	10351051	10351162	10350527	10350968	10352433	10352551	15	9	7	0	2	0	2	4	0	0	98	49	1	1	1	0.692	1	1	1	-0.103
SE	12638	TYK2	TYK2	chr19	-	10362263	10362456	10362077	10362181	10362548	10362657	40	32	6	1	0	0	4	13	0	0	98	49	1	1	0.952	1	1	1	1	-0.016
SE	12640	COPG2	COPG2	chr7	-	130554480	130554724	130552354	130552430	130555036	130555132	5	0	0	0	4	0	0	4	0	0	98	49	1	1	1	0	NA	NA	1	-0.5
SE	12648	SLC44A2	SLC44A2	chr19	+	10631625	10631749	10631474	10631535	10631867	10631951	122	50	40	1	0	0	3	17	0	0	98	49	1	1	0.984	1	1	1	1	-0.005
SE	12649	SLC44A2	SLC44A2	chr19	+	10634973	10635073	10634755	10634887	10636322	10636585	30	16	14	2	0	0	3	8	0	1	98	49	1	1	0.882	1	1	1	0.8	0.061
SE	12650	SLC44A2	SLC44A2	chr19	+	10636322	10636585	10634973	10635073	10636661	10636756	53	30	11	1	0	0	8	8	0	0	98	49	1	1	0.964	1	1	1	1	-0.012
SE	12651	SLC44A2	SLC44A2	chr19	+	10638022	10638093	10637855	10637929	10638226	10638315	157	45	30	1	0	0	4	16	0	0	98	49	1	1	0.987	1	1	1	1	-0.004
SE	12653	RNF123	RNF123	chr3	+	49712478	49712656	49706790	49706898	49713512	49713587	4	0	1	1	0	0	0	1	0	0	98	49	1	1	0.667	NA	1	NA	1	-0.166
SE	12656	SMARCA4	SMARCA4	chr19	+	11033766	11033865	11033289	11033517	11034122	11034200	0	1	0	4	0	0	0	0	0	1	98	49	1	1	0	1	NA	NA	0	0.5
SE	12658	KDM7A	KDM7A	chr7	-	140113490	140113582	140111094	140111184	140119112	140119219	68	37	8	1	0	0	0	11	0	0	98	49	1	1	0.971	1	1	NA	1	-0.01
SE	12666	B3GNTL1	B3GNTL1	chr17	-	82965650	82965746	82961056	82961181	83005114	83005210	27	8	1	0	3	0	0	5	0	0	98	49	1	1	1	0.571	1	NA	1	-0.143
SE	12677	GABARAP	GABARAP	chr17	-	7241600	7241679	7241341	7241460	7242240	7242434	29	15	24	0	0	2	0	15	0	0	98	49	1	1	1	1	0.857	NA	1	-0.048
SE	12681	ZNF136	ZNF136	chr19	+	12185784	12185911	12163056	12163206	12186113	12186174	3	9	0	0	0	0	0	0	2	0	98	49	1	1	1	1	NA	0	NA	1
SE	12684	SLC38A2	SLC38A2	chr12	-	46363020	46363145	46362493	46362638	46363725	46363826	11	14	5	1	2	0	0	6	0	0	98	49	1	1	0.846	0.778	1	NA	1	-0.125
SE	12685	SLC38A2	SLC38A2	chr12	-	46365106	46365189	46364643	46364702	46366863	46366945	19	10	2	1	0	0	0	5	0	0	98	49	1	1	0.905	1	1	NA	1	-0.032
SE	12689	ZNF564	ZNF564	chr19	-	12528303	12528364	12525369	12527916	12528569	12528696	14	4	1	0	1	0	0	1	0	0	98	49	1	1	1	0.667	1	NA	1	-0.111
SE	12690	KCNQ1	KCNQ1	chr11	+	2571324	2571403	2570627	2570754	2572012	2572109	6	3	2	0	2	0	0	3	0	0	98	49	1	1	1	0.429	1	NA	1	-0.19
SE	12691	KCNQ1	KCNQ1	chr11	+	2585211	2585307	2583434	2583545	2587569	2587692	11	3	1	0	0	0	0	4	1	0	98	49	1	1	1	1	1	0	1	0.5
SE	12692	MAN2B1	MAN2B1	chr19	-	12658427	12658507	12658223	12658344	12661259	12661376	6	0	2	2	0	0	0	8	0	1	98	49	1	1	0.6	NA	1	NA	0.8	0
SE	12693	MAN2B1	MAN2B1	chr19	-	12658427	12658510	12658223	12658344	12661259	12661376	20	5	2	2	0	0	0	10	0	1	98	49	1	1	0.833	1	1	NA	0.833	0.111
SE	12694	MAN2B1	MAN2B1	chr19	-	12663316	12663462	12661259	12661376	12663702	12663835	32	8	2	0	2	0	2	11	0	0	98	49	1	1	1	0.667	1	1	1	-0.111
SE	12696	C19orf43	C19orf43	chr19	-	12731343	12731421	12730639	12731068	12734312	12734715	35	27	25	0	0	0	0	5	0	1	98	49	1	1	1	1	1	NA	0.714	0.286
SE	12698	GLUL	GLUL	chr1	-	182385356	182385556	182378092	182384723	182385759	182385887	1476	554	212	6	0	0	32	197	0	0	98	49	1	1	0.992	1	1	1	1	-0.003
SE	12699	GLUL	GLUL	chr1	-	182385759	182385887	182385356	182385556	182386255	182386402	833	304	145	0	1	0	16	127	0	0	98	49	1	1	1	0.993	1	1	1	-0.002
SE	12700	GLUL	GLUL	chr1	-	182386255	182386402	182385759	182385887	182388571	182388750	659	195	81	0	2	0	10	80	0	0	98	49	1	1	1	0.98	1	1	1	-0.007
SE	12706	CLOCK	CLOCK	chr4	-	55478814	55478963	55475962	55476054	55479639	55479699	8	0	0	1	0	0	0	1	0	0	98	49	1	1	0.8	NA	NA	NA	1	-0.2
SE	12711	RNF145	RNF145	chr5	-	159181959	159182051	159176631	159176867	159194715	159194824	15	9	4	0	0	3	0	4	0	0	98	49	1	1	1	1	0.4	NA	1	-0.2
SE	12728	ADGRE5	ADGRE5	chr19	+	14402596	14402862	14401652	14401760	14404382	14404562	206	95	53	0	1	0	5	56	0	0	98	49	1	1	1	0.979	1	1	1	-0.007
SE	12729	ADGRE5	ADGRE5	chr19	+	14406330	14406557	14405747	14405939	14406699	14406766	132	72	50	1	0	0	4	63	0	0	98	49	1	1	0.985	1	1	1	1	-0.005
SE	12730	ADGRE5	ADGRE5	chr19	+	14407060	14407229	14406868	14406960	14407907	14408009	170	88	107	0	1	0	1	40	0	0	98	49	1	1	1	0.978	1	1	1	-0.007
SE	12731	ADGRE5	ADGRE5	chr19	+	14407907	14408009	14407060	14407229	14408091	14408725	184	68	100	0	0	2	0	44	0	0	98	49	1	1	1	1	0.962	NA	1	-0.013
SE	12733	PKN1	PKN1	chr19	+	14463827	14463969	14463666	14463742	14464059	14464153	22	7	6	0	1	0	0	5	0	0	98	49	1	1	1	0.778	1	NA	1	-0.074
SE	12734	PKN1	PKN1	chr19	+	14469751	14469814	14469340	14469517	14469911	14469988	24	28	4	2	0	0	1	5	0	1	98	49	1	1	0.857	1	1	1	0.714	0.095
SE	12742	QARS	QARS	chr3	-	49101619	49101705	49101354	49101441	49101827	49101899	5	0	7	0	1	0	0	0	0	1	98	49	1	1	1	0	1	NA	0	0.667
SE	12743	BRD4	BRD4	chr19	-	15267415	15267551	15265353	15265643	15268904	15269042	16	14	8	0	2	0	0	1	0	0	98	49	1	1	1	0.778	1	NA	1	-0.074
SE	12744	AKAP8L	AKAP8L	chr19	-	15398629	15398766	15397713	15397855	15399301	15399410	1	1	0	4	5	1	0	0	0	4	98	49	1	1	0.111	0.091	0	NA	0	0.067
SE	12746	RASAL3	RASAL3	chr19	-	15452044	15452108	15451626	15451938	15452657	15452815	13	2	2	3	0	0	1	0	0	0	98	49	1	1	0.684	1	1	1	NA	-0.105
SE	12747	LRRK1	LRRK1	chr15	+	100973803	100973967	100924510	100924729	100983527	100983699	8	0	0	1	0	0	2	0	0	0	98	49	1	1	0.8	NA	NA	1	NA	-0.2
SE	12749	CYP4F3	CYP4F3	chr19	+	15643906	15644051	15641414	15641613	15647051	15647105	2	3	5	5	2	0	0	1	0	0	98	49	1	1	0.167	0.429	1	NA	1	-0.468
SE	12760	KLF2	KLF2	chr19	+	16325215	16326032	16324839	16324998	16326855	16327528	71	29	9	1	1	0	4	7	0	0	98	49	1	1	0.973	0.935	1	1	1	-0.031
SE	12762	EPS15L1	EPS15L1	chr19	-	16377121	16377254	16361577	16361984	16385128	16385211	6	4	0	0	0	3	0	1	0	0	98	49	1	1	1	1	0	NA	1	-0.333
SE	12770	CCDC125	CCDC125	chr5	-	69303846	69303929	69300011	69300127	69306816	69306902	10	3	3	0	0	3	0	4	0	0	98	49	1	1	1	1	0.333	NA	1	-0.222
SE	12771	CCDC125	CCDC125	chr5	-	69313984	69314043	69311117	69311204	69320236	69320345	8	9	6	1	0	0	0	3	0	0	98	49	1	1	0.8	1	1	NA	1	-0.067
SE	12772	CCDC125	CCDC125	chr5	-	69313984	69314046	69311117	69311204	69320236	69320580	11	6	5	1	0	0	0	3	0	0	98	49	1	1	0.846	1	1	NA	1	-0.051
SE	12773	CCDC125	CCDC125	chr5	-	69320236	69320345	69311117	69311204	69320464	69320580	6	1	1	1	0	0	0	1	0	0	98	49	1	1	0.75	1	1	NA	1	-0.083
SE	12780	CSNK1G3	CSNK1G3	chr5	+	123575728	123575963	123573392	123573541	123588067	123588153	9	8	0	1	0	0	0	3	0	0	98	49	1	1	0.818	1	NA	NA	1	-0.091
SE	12785	MYO9B	MYO9B	chr19	+	17159394	17159484	17156908	17157038	17162349	17162466	96	72	14	1	0	0	4	24	0	0	98	49	1	1	0.98	1	1	1	1	-0.007
SE	12786	MYO9B	MYO9B	chr19	+	17210332	17210380	17209585	17209709	17210714	17210848	1	1	0	22	7	2	0	0	0	3	97	49	1	1	0.022	0.067	0	NA	0	0.03
SE	12787	PCGF5	PCGF5	chr10	+	91240483	91240580	91222688	91222983	91248504	91248560	12	6	1	1	0	0	0	2	0	0	98	49	1	1	0.857	1	1	NA	1	-0.048
SE	12788	PCGF5	PCGF5	chr10	+	91271637	91271697	91264430	91264520	91278268	91284331	9	0	2	1	0	0	0	7	0	0	98	49	1	1	0.818	NA	1	NA	1	-0.091
SE	12794	JAK3	JAK3	chr19	-	17843776	17843900	17843379	17843491	17844233	17844430	71	11	6	1	1	0	0	14	0	0	98	49	1	1	0.973	0.846	1	NA	1	-0.06
SE	12798	ARRDC2	ARRDC2	chr19	+	18009591	18009694	18008970	18009118	18009782	18010039	10	4	7	2	0	0	0	1	0	0	98	49	1	1	0.714	1	1	NA	1	-0.095
SE	12799	EDEM2	EDEM2	chr20	-	35118597	35118719	35115356	35115933	35123889	35124034	7	4	3	2	0	0	0	6	0	1	98	49	1	1	0.636	1	1	NA	0.75	0.129
SE	12800	EDEM2	EDEM2	chr20	-	35131641	35131783	35126250	35126375	35134737	35134949	5	3	2	1	0	0	0	2	0	0	98	49	1	1	0.714	1	1	NA	1	-0.095
SE	12801	EDEM2	EDEM2	chr20	-	35144978	35145018	35142372	35142478	35146824	35146935	4	8	5	1	0	0	0	2	0	0	89	49	1	1	0.688	1	1	NA	1	-0.104
SE	12804	SSBP4	SSBP4	chr19	+	18431352	18431418	18430840	18430930	18431646	18431706	2	0	0	1	0	0	0	0	0	1	98	49	1	1	0.5	NA	NA	NA	0	0.5
SE	12811	COPE	COPE	chr19	-	18906959	18907112	18905575	18905629	18910970	18911071	29	11	7	2	0	0	1	15	0	0	98	49	1	1	0.879	1	1	1	1	-0.04
SE	12818	ARMC6	ARMC6	chr19	+	19034130	19034238	19033577	19033930	19042710	19042877	0	0	0	1	1	0	0	2	0	0	98	49	1	1	0	0	NA	NA	1	-1
SE	12823	GMIP	GMIP	chr19	-	19635414	19635569	19635024	19635213	19636706	19636787	51	48	6	0	1	0	5	9	0	0	98	49	1	1	1	0.96	1	1	1	-0.013
SE	12827	ZNF101	ZNF101	chr19	+	19678725	19678786	19677863	19677990	19679180	19683506	8	1	0	0	0	0	0	1	0	1	98	49	1	1	1	1	NA	NA	0.333	0.667
SE	12839	ING4	ING4	chr12	-	6653229	6653396	6652935	6653050	6656726	6656798	11	4	2	1	4	0	0	1	0	0	98	49	1	1	0.846	0.333	1	NA	1	-0.274
SE	12848	ANXA11	ANXA11	chr10	-	80168968	80169358	80167225	80167313	80170799	80170915	35	26	22	0	0	0	0	6	0	2	98	49	1	1	1	1	1	NA	0.6	0.4
SE	12850	ANXA11	ANXA11	chr10	-	80176106	80176155	80172806	80172869	80205342	80205444	35	25	21	3	3	0	0	4	0	2	98	49	1	1	0.854	0.806	1	NA	0.5	0.387
SE	12858	TLK2	TLK2	chr17	+	62524235	62524331	62523133	62523177	62536169	62536337	11	2	0	1	1	1	0	1	0	0	98	49	1	1	0.846	0.5	0	NA	1	-0.551
SE	12861	TLK2	TLK2	chr17	+	62574344	62574410	62573214	62573367	62576708	62576775	2	0	0	11	2	5	0	0	0	2	98	49	1	1	0.083	0	0	NA	0	0.028
SE	12862	TLK2	TLK2	chr17	+	62602041	62602180	62600650	62600820	62606129	62606241	3	4	0	0	1	0	0	1	0	0	98	49	1	1	1	0.667	NA	NA	1	-0.166
SE	12880	GPI	GPI	chr19	+	34377502	34377586	34368582	34368702	34377734	34377881	13	7	1	0	0	0	0	3	0	1	98	49	1	1	1	1	1	NA	0.6	0.4
SE	12882	GRAMD1A	GRAMD1A	chr19	+	35009422	35009443	35009118	35009329	35009887	35009972	24	7	9	1	0	0	1	5	0	0	70	49	1	1	0.944	1	1	1	1	-0.019
SE	12885	GRAMD1A	GRAMD1A	chr19	+	35023426	35023547	35023235	35023343	35026048	35026469	23	0	13	0	1	0	0	3	0	0	98	49	1	1	1	0	1	NA	1	-0.333
SE	12909	MMP8	MMP8	chr11	-	102714555	102714709	102713753	102713857	102715303	102715437	1	1	0	0	0	0	4	2	0	3	98	49	1	1	1	1	NA	1	0.25	0.375
SE	12910	MMP8	MMP8	chr11	-	102722990	102723078	102722428	102722673	102724753	102724954	0	1	0	0	1	0	3	3	0	1	98	49	1	1	NA	0.333	NA	1	0.6	-0.467
SE	12911	MMP8	MMP8	chr11	-	102722990	102723081	102722428	102722673	102724753	102724954	0	1	0	0	1	0	3	3	0	1	98	49	1	1	NA	0.333	NA	1	0.6	-0.467
SE	12912	TREML3P	TREML3P	chr6	-	41217126	41217177	41215761	41215819	41217652	41217947	7	3	1	0	1	0	1	0	0	0	98	49	1	1	1	0.6	1	1	NA	-0.133
SE	12917	U2AF1L4	U2AF1L4	chr19	-	35744625	35744738	35744069	35744421	35745124	35745187	1	0	0	0	2	0	0	2	0	0	98	49	1	1	1	0	NA	NA	1	-0.5
SE	12920	TYROBP	TYROBP	chr19	-	35907217	35907261	35904400	35904634	35907445	35907580	126	73	76	0	0	0	7	39	0	1	93	49	1	1	1	1	1	1	0.954	0.023
SE	12921	TYROBP	TYROBP	chr19	-	35907217	35907264	35904400	35904634	35907445	35907580	242	131	137	0	0	0	18	77	0	1	96	49	1	1	1	1	1	1	0.975	0.012
SE	12926	INO80E	INO80E	chr16	+	30001211	30001530	30000928	30001040	30005220	30005790	4	1	5	0	1	0	0	2	0	0	98	49	1	1	1	0.333	1	NA	1	-0.222
SE	12927	INO80E	INO80E	chr16	+	30001413	30001530	30000928	30001040	30005220	30005790	5	0	5	0	1	0	0	2	0	0	98	49	1	1	1	0	1	NA	1	-0.333
SE	12928	INO80E	INO80E	chr16	+	30004567	30004657	30001211	30001530	30005220	30005790	1	0	0	0	0	5	0	0	0	2	98	49	1	1	1	NA	0	NA	0	0.5
SE	12934	KDM1A	KDM1A	chr1	+	23044426	23044486	23030468	23030634	23050386	23050520	2	6	0	4	0	0	0	2	0	0	98	49	1	1	0.2	1	NA	NA	1	-0.4
SE	12948	PQBP1	PQBP1	chrX	+	48902232	48902517	48901929	48902042	48902731	48902795	4	0	6	1	0	0	0	2	0	0	98	49	1	1	0.667	NA	1	NA	1	-0.166
SE	12961	DDX60L	DDX60L	chr4	-	168379725	168379830	168379362	168379504	168384611	168384812	28	10	8	0	0	0	1	18	0	1	98	49	1	1	1	1	1	1	0.9	0.05
SE	12964	DDX60L	DDX60L	chr4	-	168415407	168415517	168406601	168406706	168415656	168415799	69	58	11	0	1	0	1	57	0	0	98	49	1	1	1	0.967	1	1	1	-0.011
SE	12973	GMFG	GMFG	chr19	-	39329543	39329626	39328999	39329073	39333076	39333126	288	205	223	2	1	0	0	53	0	0	98	49	1	1	0.986	0.99	1	NA	1	-0.008
SE	12974	GMFG	GMFG	chr19	-	39333076	39333126	39328999	39329073	39335434	39335531	6	1	0	1	0	0	0	1	0	0	98	49	1	1	0.75	1	NA	NA	1	-0.125
SE	12975	GMFG	GMFG	chr19	-	39333076	39333126	39329543	39329626	39335260	39335310	200	151	145	0	1	0	1	30	0	0	98	49	1	1	1	0.987	1	1	1	-0.004
SE	12977	GMFG	GMFG	chr19	-	39335260	39335310	39333076	39333126	39335973	39336086	97	63	46	1	0	0	1	12	0	0	98	49	1	1	0.98	1	1	1	1	-0.007
SE	12978	GMFG	GMFG	chr19	-	39335434	39335531	39333076	39333126	39335973	39336086	5	0	0	1	0	0	0	1	0	0	98	49	1	1	0.714	NA	NA	NA	1	-0.286
SE	12981	IFI30	IFI30	chr19	+	18177139	18177292	18175604	18175697	18177710	18177764	252	82	46	1	0	0	13	127	0	0	98	49	1	1	0.992	1	1	1	1	-0.003
SE	12989	CDK19	CDK19	chr6	-	110623289	110623362	110622814	110622912	110626775	110626845	23	2	0	3	0	0	3	1	0	0	98	49	1	1	0.793	1	NA	1	1	-0.103
SE	12990	CDK19	CDK19	chr6	-	110632029	110632161	110627001	110627145	110638648	110638706	11	4	4	1	0	0	0	4	0	0	98	49	1	1	0.846	1	1	NA	1	-0.051
SE	12991	CDK19	CDK19	chr6	-	110773875	110773937	110746125	110746201	110815008	110815316	5	2	0	4	1	1	0	0	0	1	98	49	1	1	0.385	0.5	0	NA	0	0.295
SE	12993	ADCK4	ADCK4	chr19	-	40703714	40703855	40703540	40703622	40705095	40705181	2	1	0	0	1	0	0	3	0	0	98	49	1	1	1	0.333	NA	NA	1	-0.334
SE	12995	SASH3	SASH3	chrX	+	129787974	129788070	129779915	129780154	129790939	129791081	41	14	8	2	0	0	0	8	0	0	98	49	1	1	0.911	1	1	NA	1	-0.03
SE	12996	SASH3	SASH3	chrX	+	129788430	129788577	129787974	129788070	129790939	129791081	52	17	17	1	2	0	0	8	0	1	98	49	1	1	0.963	0.81	1	NA	0.8	0.124
SE	12998	SASH3	SASH3	chrX	+	129792626	129792836	129792327	129792476	129792988	129793139	41	25	8	2	0	0	0	20	0	0	98	49	1	1	0.911	1	1	NA	1	-0.03
SE	13001	CEACAM4	CEACAM4	chr19	-	41619669	41619711	41618970	41619395	41620210	41620242	29	10	30	1	0	0	1	10	0	0	91	49	1	1	0.94	1	1	1	1	-0.02
SE	13002	CEACAM4	CEACAM4	chr19	-	41621650	41621768	41620574	41620627	41625600	41625960	36	26	10	0	1	0	5	18	0	0	98	49	1	1	1	0.929	1	1	1	-0.024
SE	13005	RPS19	RPS19	chr19	+	41869030	41869214	41861111	41861212	41869698	41869753	205	40	27	0	0	0	6	58	0	1	98	49	1	1	1	1	1	1	0.967	0.016
SE	13006	ARHGEF1	ARHGEF1	chr19	+	41888751	41888865	41888191	41888278	41892330	41892373	19	16	3	0	1	0	2	6	0	0	98	49	1	1	1	0.889	1	1	1	-0.037
SE	13008	ARHGEF1	ARHGEF1	chr19	+	41904948	41905036	41904215	41904383	41905174	41905261	38	13	4	0	1	0	3	4	0	0	98	49	1	1	1	0.867	1	1	1	-0.044
SE	13010	ARHGEF1	ARHGEF1	chr19	+	41906456	41906620	41905938	41906025	41906702	41906803	52	33	5	4	0	0	0	18	0	2	98	49	1	1	0.867	1	1	NA	0.818	0.138
SE	13028	DGCR2	DGCR2	chr22	-	19063201	19063278	19056985	19057162	19064847	19065058	17	23	13	1	0	0	0	14	0	0	98	49	1	1	0.895	1	1	NA	1	-0.035
SE	13043	JTB	JTB	chr1	-	153976692	153976775	153975825	153975905	153976975	153977013	46	9	42	0	0	2	0	5	0	0	98	49	1	1	1	1	0.913	NA	1	-0.029
SE	13046	TRAPPC6A	TRAPPC6A	chr19	-	45165126	45165194	45164852	45164970	45178134	45178243	2	0	0	1	0	0	0	2	0	0	98	49	1	1	0.5	NA	NA	NA	1	-0.5
SE	13048	CHD2	CHD2	chr15	+	92924320	92924552	92901166	92901299	92927243	92927330	47	19	9	1	0	0	5	6	0	0	98	49	1	1	0.959	1	1	1	1	-0.014
SE	13050	CHD2	CHD2	chr15	+	92944414	92944515	92942842	92943068	92945820	92945865	45	12	4	0	2	0	0	5	0	0	98	49	1	1	1	0.75	1	NA	1	-0.083
SE	13051	CHD2	CHD2	chr15	+	92945820	92945865	92944414	92944515	92946037	92946216	67	20	12	1	0	0	2	13	0	0	94	49	1	1	0.972	1	1	1	1	-0.009
SE	13052	CHD2	CHD2	chr15	+	92956458	92956649	92955422	92955512	92967324	92967513	66	24	4	6	3	0	0	13	0	1	98	49	1	1	0.846	0.8	1	NA	0.867	0.015
SE	13053	CHD2	CHD2	chr15	+	92978233	92978383	92974878	92974950	92979134	92979283	19	16	7	0	1	0	0	3	0	0	98	49	1	1	1	0.889	1	NA	1	-0.037
SE	13054	CHD2	CHD2	chr15	+	92992858	92992998	92991475	92991517	92996956	92997095	13	24	2	0	1	0	1	5	0	0	98	49	1	1	1	0.923	1	1	1	-0.026
SE	13055	CHD2	CHD2	chr15	+	93004616	93004751	93002176	93002317	93009144	93009323	21	13	0	1	0	0	0	6	0	0	98	49	1	1	0.913	1	NA	NA	1	-0.043
SE	13056	WAS	WAS	chrX	+	48685733	48685836	48685546	48685633	48685945	48685987	112	59	30	0	0	2	5	20	0	0	98	49	1	1	1	1	0.882	1	1	-0.039
SE	13057	WAS	WAS	chrX	+	48688659	48689066	48688299	48688453	48689319	48689434	64	13	22	2	0	0	1	15	0	0	98	49	1	1	0.941	1	1	1	1	-0.02
SE	13059	ERCC1	ERCC1	chr19	-	45413962	45414034	45413433	45413745	45414860	45414960	7	0	0	1	0	0	0	2	0	0	98	49	1	1	0.778	NA	NA	NA	1	-0.222
SE	13060	DOK3	DOK3	chr5	-	177508236	177508542	177505010	177505110	177509474	177509575	79	29	16	0	0	0	7	32	0	1	98	49	1	1	1	1	1	1	0.941	0.03
SE	13070	CMTR1	CMTR1	chr6	+	37461969	37462102	37461548	37461645	37462828	37463008	6	2	0	0	0	0	0	3	0	1	98	49	1	1	1	1	NA	NA	0.6	0.4
SE	13071	CMTR1	CMTR1	chr6	+	37462828	37463008	37461548	37461645	37471020	37471077	8	0	0	0	0	0	0	3	1	0	98	49	1	1	1	NA	NA	0	1	0.5
SE	13073	CNOT8	CNOT8	chr5	+	154863206	154863395	154858518	154858768	154865191	154865385	12	3	4	2	0	1	0	2	0	0	98	49	1	1	0.75	1	0.667	NA	1	-0.194
SE	13074	CNOT8	CNOT8	chr5	+	154863206	154863395	154858518	154858768	154870660	154870822	6	0	0	0	0	0	0	2	0	1	98	49	1	1	1	NA	NA	NA	0.5	0.5
SE	13075	CNOT8	CNOT8	chr5	+	154865191	154865385	154858518	154858768	154870660	154870822	6	2	6	0	0	0	0	0	0	1	98	49	1	1	1	1	1	NA	0	1
SE	13080	CNOT8	CNOT8	chr5	+	154870660	154870822	154867691	154867792	154871729	154871874	10	3	2	0	0	1	0	3	0	0	98	49	1	1	1	1	0.5	NA	1	-0.167
SE	13085	STT3B	STT3B	chr3	+	31624913	31625085	31623673	31623861	31625953	31626127	15	7	1	2	0	0	0	3	0	0	98	49	1	1	0.789	1	1	NA	1	-0.07
SE	13087	TSC1	TSC1	chr9	-	132903650	132903817	132902604	132902787	132904410	132904454	1	0	3	1	0	0	0	1	0	0	98	49	1	1	0.333	NA	1	NA	1	-0.334
SE	13093	USP33	USP33	chr1	-	77701368	77701471	77697862	77697931	77711746	77711855	9	2	2	1	2	0	0	5	0	2	98	49	1	1	0.818	0.333	1	NA	0.556	0.161
SE	13094	USP33	USP33	chr1	-	77745420	77745599	77741616	77741748	77759642	77759879	2	2	0	8	3	0	0	1	0	0	98	49	1	1	0.111	0.25	NA	NA	1	-0.82
SE	13098	DHX34	DHX34	chr19	+	47357865	47358120	47355038	47355350	47359967	47360070	58	21	2	0	0	0	2	24	0	1	98	49	1	1	1	1	1	1	0.923	0.038
SE	13099	DHX34	DHX34	chr19	+	47373598	47373700	47372729	47372923	47375465	47375708	21	21	7	1	0	0	1	14	0	0	98	49	1	1	0.913	1	1	1	1	-0.029
SE	13107	KIDINS220	KIDINS220	chr2	-	8793809	8793987	8791059	8791224	8796770	8796869	18	7	4	0	1	0	0	2	0	0	98	49	1	1	1	0.778	1	NA	1	-0.074
SE	13108	KIDINS220	KIDINS220	chr2	-	8826985	8827129	8818694	8818793	8837479	8837625	14	8	3	0	1	0	0	3	0	0	98	49	1	1	1	0.8	1	NA	1	-0.067

SE	13109	GBP3	GBP3	chr1	-	89020531	89020743	89015286	89015414	89022683	89022866	8	1	0	1	0	0	1	0	0	0	98	49	1	1	0.8	1	NA	1	NA	-0.1
SE	13113	LRRC42	LRRC42	chr1	+	53962033	53962122	53960355	53960474	53962295	53962409	0	0	0	0	1	0	0	1	0	0	98	49	1	1	NA	0	NA	NA	1	-1
SE	13120	PRPF18	PRPF18	chr10	+	13613740	13613881	13611614	13611683	13614014	13614086	9	7	1	3	0	0	0	3	0	0	98	49	1	1	0.6	1	1	NA	1	-0.133
SE	13123	EHMT1	EHMT1	chr9	+	137752330	137752408	137743901	137744090	137754170	137754291	9	0	0	2	0	0	0	4	0	0	98	49	1	1	0.692	NA	NA	NA	1	-0.308
SE	13126	CEP350	CEP350	chr1	+	179990506	179990621	179987239	179987286	179992061	179992221	17	4	0	1	0	2	0	5	0	0	98	49	1	1	0.895	1	0	NA	1	-0.368
SE	13127	CEP350	CEP350	chr1	+	180036925	180037089	180033861	180034082	180041137	180041248	5	7	4	0	0	0	0	1	0	1	98	49	1	1	1	1	1	NA	0.333	0.667
SE	13128	CEP350	CEP350	chr1	+	180043055	180043192	180041661	180041802	180044050	180044173	13	3	0	2	0	0	2	5	0	0	98	49	1	1	0.765	1	NA	1	1	-0.117
SE	13137	RPS6KA3	RPS6KA3	chrX	-	20167588	20167747	20164898	20165060	20169401	20169491	26	19	12	0	4	0	0	11	0	0	98	49	1	1	1	0.704	1	NA	1	-0.099
SE	13141	CD37	CD37	chr19	+	49339329	49339413	49338699	49338936	49340250	49340604	29	17	24	2	0	0	1	21	0	0	98	49	1	1	0.879	1	1	1	1	-0.04
SE	13146	IL1RN	IL1RN	chr2	+	113131044	113131157	113129575	113129664	113132655	113134016	37	37	14	0	1	0	0	12	0	0	98	49	1	1	1	0.949	1	NA	1	-0.017
SE	13150	FCGRT	FCGRT	chr19	+	49524506	49524776	49514210	49514486	49526009	49526428	73	30	27	1	0	0	3	16	0	0	98	49	1	1	0.973	1	1	1	1	-0.009
SE	13151	FCGRT	FCGRT	chr19	+	49525456	49525573	49524506	49524776	49526009	49526428	66	64	45	2	0	0	6	18	0	0	98	49	1	1	0.943	1	1	1	1	-0.019
SE	13160	AP2A1	AP2A1	chr19	+	49800960	49801058	49799967	49800150	49801389	49801621	20	9	5	0	0	0	1	3	1	0	98	49	1	1	1	1	1	0.333	1	0.334
SE	13163	SMG7	SMG7	chr1	+	183512836	183512868	183472370	183472649	183515873	183515991	3	2	1	0	1	0	0	2	0	0	81	49	1	1	1	0.547	1	NA	1	-0.151
SE	13166	GPATCH2L	GPATCH2L	chr14	+	76178207	76178322	76177987	76178042	76180763	76180849	0	3	0	6	6	0	0	0	0	2	98	49	1	1	0	0.2	NA	NA	0	0.1
SE	13167	GPATCH2L	GPATCH2L	chr14	+	76180763	76180849	76177987	76178042	76195877	76195972	16	7	0	2	0	0	1	4	0	0	98	49	1	1	0.8	1	NA	1	1	-0.1
SE	13168	PNKP	PNKP	chr19	-	49861607	49861695	49861448	49861510	49861771	49861881	3	0	6	0	1	0	1	2	0	0	98	49	1	1	1	0	1	1	1	-0.333
SE	13172	VRK3	VRK3	chr19	-	49997503	49997570	49995190	49995275	50000789	50000854	20	14	8	1	1	0	0	5	0	0	98	49	1	1	0.909	0.875	1	NA	1	-0.072
SE	13173	VRK3	VRK3	chr19	-	50009235	50009385	50007568	50007826	50016023	50016163	25	3	2	2	3	1	0	4	0	0	98	49	1	1	0.862	0.333	0.5	NA	1	-0.435
SE	13183	EMC10	EMC10	chr19	+	50481860	50481947	50480883	50480977	50482148	50483526	2	0	0	3	0	1	0	1	0	2	98	49	1	1	0.25	NA	0	NA	0.2	-0.075
SE	13194	LONRF1	LONRF1	chr8	-	12725726	12725879	12721896	12723254	12728900	12729063	2	1	0	0	1	0	0	1	0	0	98	49	1	1	1	0.333	NA	NA	1	-0.334
SE	13204	ARHGAP30	ARHGAP30	chr1	-	161051313	161051715	161049423	161049689	161052285	161052363	62	18	14	1	0	0	0	6	0	0	98	49	1	1	0.969	1	1	NA	1	-0.01
SE	13205	ARHGAP30	ARHGAP30	chr1	-	161056387	161056532	161054622	161054705	161059613	161059716	92	62	21	5	3	2	0	26	0	3	98	49	1	1	0.902	0.912	0.84	NA	0.812	0.073
SE	13206	ARHGAP30	ARHGAP30	chr1	-	161056387	161056532	161054622	161054705	161069527	161069970	54	33	7	1	1	0	0	18	0	2	98	49	1	1	0.964	0.943	1	NA	0.818	0.151
SE	13207	ARHGAP30	ARHGAP30	chr1	-	161059613	161059716	161054622	161054705	161069527	161069970	40	18	10	1	1	0	1	18	0	2	98	49	1	1	0.952	0.9	1	1	0.818	0.042
SE	13208	ARHGAP30	ARHGAP30	chr1	-	161059613	161059716	161056387	161056532	161069527	161069970	75	44	22	2	0	0	1	23	0	0	98	49	1	1	0.949	1	1	1	1	-0.017
SE	13213	FES	FES	chr15	+	90885411	90885585	90885036	90885258	90886960	90887057	23	13	6	1	0	0	1	3	0	1	98	49	1	1	0.92	1	1	1	0.6	0.173
SE	13215	FES	FES	chr15	+	90890981	90891191	90890400	90890484	90891553	90891676	10	9	5	1	0	0	0	7	0	0	98	49	1	1	0.833	1	1	NA	1	-0.056
SE	13216	FES	FES	chr15	+	90891553	90891676	90890400	90890484	90892057	90892111	13	6	4	1	0	0	0	8	0	0	98	49	1	1	0.867	1	1	NA	1	-0.044
SE	13217	SIGLEC5	SIGLEC5	chr19	-	51626031	51626113	51611502	51612422	51627148	51627248	23	18	26	1	0	1	0	13	0	1	98	49	1	1	0.92	1	0.929	NA	0.867	0.083
SE	13219	ALG13	ALG13	chrX	+	111686136	111686174	111684964	111685103	111687886	111690395	4	0	0	4	0	0	0	2	0	0	87	49	1	1	0.36	NA	NA	NA	1	-0.64
SE	13225	AFF4	AFF4	chr5	-	132893029	132893118	132892163	132892404	132896322	132897240	7	10	0	0	1	0	0	2	0	0	98	49	1	1	1	0.833	NA	NA	1	-0.084
SE	13226	AFF4	AFF4	chr5	-	132898229	132898392	132896322	132897240	132899103	132899141	15	7	7	1	0	0	0	2	0	0	98	49	1	1	0.882	1	1	NA	1	-0.039
SE	13230	PPP2R1A	PPP2R1A	chr19	+	52220188	52220249	52219690	52219864	52220978	52221133	5	0	4	0	0	0	0	6	0	1	98	49	1	1	1	NA	1	NA	0.75	0.25
SE	13232	RBM23	RBM23	chr14	-	22908332	22908380	22906194	22906368	22909482	22909595	25	12	2	7	6	8	0	2	0	0	97	49	1	1	0.643	0.503	0.112	NA	1	-0.581
SE	13236	RBM23	RBM23	chr14	-	22911327	22911403	22908332	22908380	22918998	22919187	9	16	3	0	1	0	0	2	0	0	98	49	1	1	1	0.889	1	NA	1	-0.037
SE	13244	RELL1	RELL1	chr4	-	37638446	37638504	37634886	37635123	37647367	37647439	56	55	36	2	0	0	0	7	0	0	98	49	1	1	0.933	1	1	NA	1	-0.022
SE	13245	RELL1	RELL1	chr4	-	37649275	37649500	37647367	37647439	37686199	37686377	26	15	12	0	0	2	1	7	0	0	98	49	1	1	1	1	0.75	1	1	-0.083
SE	13249	TSR3	TSR3	chr16	-	1349888	1349952	1349239	1349608	1350057	1350234	12	9	5	0	0	0	0	0	0	1	98	49	1	1	1	1	1	NA	0	1
SE	13252	ENTPD1	ENTPD1	chr10	+	95842343	95842494	95839690	95839808	95844475	95844635	38	26	11	3	4	1	0	10	0	0	98	49	1	1	0.864	0.765	0.846	NA	1	-0.175
SE	13253	ENTPD1	ENTPD1	chr10	+	95845356	95845596	95844475	95844635	95847445	95847706	49	20	10	1	1	0	1	12	0	0	98	49	1	1	0.961	0.909	1	1	1	-0.043
SE	13266	PSMF1	PSMF1	chr20	+	1127425	1127508	1125497	1125650	1135120	1135306	16	6	5	0	2	0	1	1	0	0	98	49	1	1	1	0.6	1	1	1	-0.133
SE	13268	CYP20A1	CYP20A1	chr2	+	203246754	203246921	203245845	203245895	203251966	203252109	1	0	0	0	0	0	0	0	1	0	98	49	1	1	1	NA	NA	0	NA	1
SE	13269	CYP20A1	CYP20A1	chr2	+	203272669	203272748	203266513	203266681	203278572	203278688	2	0	0	3	0	0	0	1	0	0	98	49	1	1	0.25	NA	NA	NA	1	-0.75
SE	13270	CYP20A1	CYP20A1	chr2	+	203278572	203278688	203266513	203266681	203280058	203280113	4	1	0	0	1	0	0	1	0	0	98	49	1	1	1	0.333	NA	NA	1	-0.334
SE	13271	BCKDK	BCKDK	chr16	+	31110687	31110761	31110400	31110499	31111090	31111219	11	3	7	1	2	0	0	1	0	1	98	49	1	1	0.846	0.429	1	NA	0.333	0.425
SE	13281	RBM5	RBM5	chr3	+	50100531	50100605	50099981	50100051	50103082	50103166	8	1	5	14	6	1	0	0	0	2	98	49	1	1	0.222	0.077	0.714	NA	0	0.338
SE	13282	RBM5	RBM5	chr3	+	50104247	50104308	50103082	50103166	50105076	50105142	49	24	14	1	0	0	2	6	0	1	98	49	1	1	0.961	1	1	1	0.75	0.112
SE	13283	RBM5	RBM5	chr3	+	50105548	50105709	50105076	50105142	50106766	50106864	70	35	32	0	1	0	1	9	0	0	98	49	1	1	1	0.946	1	1	1	-0.018
SE	13284	RBM5	RBM5	chr3	+	50110378	50110463	50109602	50109688	50110678	50110770	37	6	17	25	5	2	0	3	0	0	98	49	1	1	0.425	0.375	0.81	NA	1	-0.463
SE	13285	RBM5	RBM5	chr3	+	50115427	50115607	50114179	50114251	50115905	50115980	93	20	12	0	1	0	6	16	0	0	98	49	1	1	1	0.909	1	1	1	-0.03
SE	13286	RBM5	RBM5	chr3	+	50117249	50117379	50117073	50117171	50118330	50118964	49	8	21	0	1	0	2	10	0	0	98	49	1	1	1	0.8	1	1	1	-0.067
SE	13287	TARM1	TARM1	chr19	-	54075882	54075918	54074823	54075114	54081306	54081365	0	0	0	2	0	0	0	1	0	0	85	49	1	1	0	NA	NA	NA	1	-1
SE	13294	MBOAT7	MBOAT7	chr19	-	54180772	54181133	54178274	54178941	54183520	54183680	69	32	18	1	1	0	0	9	0	0	98	49	1	1	0.972	0.941	1	NA	1	-0.029
SE	13295	MBOAT7	MBOAT7	chr19	-	54187160	54187287	54183520	54183680	54188216	54188346	115	46	59	1	0	0	10	24	0	1	98	49	1	1	0.983	1	1	1	0.923	0.033
SE	13296	MBOAT7	MBOAT7	chr19	-	54188216	54188346	54187160	54187287	54188432	54188511	68	38	34	1	0	0	3	20	0	0	98	49	1	1	0.971	1	1	1	1	-0.01
SE	13297	MBOAT7	MBOAT7	chr19	-	54188432	54188511	54188216	54188346	54189337	54189882	118	64	47	4	4	0	9	20	0	1	98	49	1	1	0.937	0.889	1	1	0.909	-0.012
SE	13298	TSEN34	TSEN34	chr19	+	54191360	54191607	54190267	54190450	54191720	54191964	18	11	29	0	1	0	2	5	0	0	98	49	1	1	1	0.846	1	1	1	-0.051
SE	13301	LILRA6	LILRA6	chr19	-	54239900	54239951	54238821	54239089	54240273	54240576	3	5	0	0	2	0	1	4	0	0	98	49	1	1	1	0.556	NA	1	1	-0.222
SE	13316	LILRA5	LILRA5	chr19	-	54311863	54312148	54311164	54311716	54312536	54312621	57	36	42	0	0	0	1	18	0	1	98	49	1	1	1	1	1	1	0.9	0.05
SE	13317	LILRA5	LILRA5	chr19	-	54312334	54312370	54311863	54312148	54312536	54312621	39	27	49	6	7	2	0	15	0	2	85	49	1	1	0.789	0.69	0.934	NA	0.812	-0.008
SE	13318	LILRA5	LILRA5	chr19	-	54312536	54312621	54312334	54312370	54313016	54313139	33	20	36	2	0	0	0	14	0	0	98	49	1	1	0.892	1	1	NA	1	-0.036
SE	13319	LENG8	LENG8	chr19	+	54451289	54451382	54449161	54449310	54452092	54452267	17	15	2	0	1	0	1	4	0	0	98	49	1	1	1	0.882	1	1	1	-0.039
SE	13320	LENG8	LENG8	chr19	+	54453545	54453656	54452650	54452752	54454429	54454682	20	5	1	1	0	0	0	5	0	1	98	49	1	1	0.909	1	1	NA	0.714	0.256
SE	13331	LILRB1	LILRB1	chr19	+	54633637	54633688	54633015	54633318	54633970	54634021	5	1	0	0	0	0	0	2	0	1	98	49	1	1	1	1	NA	NA	0.5	0.5
SE	13571	CEP192	CEP192	chr18	+	13038369	13038579	13037236	13037301	13040829	13040956	1	1	0	1	0	0	0	2	0	0	98	49	1	1	0.333	1	NA	NA	1	-0.334
SE	13573	CEP192	CEP192	chr18	+	13059081	13059312	13055779	13056698	13067830	13067956	2	5	0	2	0	0	1	1	0	0	98	49	1	1	0.333	1	NA	1	1	-0.334
SE	13583	PIK3AP1	PIK3AP1	chr10	-	96602279	96602398	96593311	96595634	96603978	96604049	36	19	18	0	1	0	2	27	0	0	98	49	1	1	1	0.905	1	1	1	-0.032
SE	13584	PIK3AP1	PIK3AP1	chr10	-	96603978	96604049	96593311	96595634	96609711	96609867	18	5	6	1	0	0	0	9	0	0	98	49	1	1	0.9	1	1	NA	1	-0.033
SE	13585	PIK3AP1	PIK3AP1	chr10	-	96609711	96609867	96603978	96604049	96616638	96616711	32	26	11	0	1	0	1	11	0	0	98	49	1	1	1	0.929	1	1	1	-0.024
SE	13586	PIK3AP1	PIK3AP1	chr10	-	96652697	96652842	96651508	96651651	96656797	96656934	27	16	3	1	0	0	1	15	0	0	98	49	1	1	0.931	1	1	1	1	-0.023
SE	13590	FAM129A	FAM129A	chr1	-	184805945	184806056	184803584	184803692	184808073	184808235	167	67	46	0	1	0	1	25	0	0	98	49	1	1	1	0.971	1	1	1	-0.01
SE	13591	FAM129A	FAM129A	chr1	-	184818637	184818825	184808073	184808235	184823166	184823329	163	107	53	0	0	1	0	16	0	0	98	49	1	1	1	1	0.964	NA	1	-0.012
SE	13593	FAM129A	FAM129A	chr1	-	184831846	184831962	184823637	184823742	184884632	184884800	227	89	67	0	3	0	0	32	0	0	98	49	1	1	1	0.937	1	NA	1	-0.021
SE	13594	HIATL1	HIATL1	chr9	+	94456239	94456337	94453957	94454070	94458321	94458464	23	5	5	0	0	0	0	10	0	1	98	49	1	1	1	1	1	NA	0.833	0.167
SE	13612	GTF3C2	GTF3C2	chr2	-	27335597	27335706	27333973	27333999	27335916	27336028	6	0	0	0	1	0	0	5	0	0	98	49	1	1	1	0	NA	NA	1	-0.5
SE	13615	ZNF274	ZNF274	chr19	+	58209960	58210073	58206719	58207202	58211559	58211686	1	1	0	0	0	0	1	0	3	0	98	49	1	1	1	1	NA	0.143	NA	0.857
SE	13623	VPS8	VPS8	chr3	+	184866875	184866950	184862896	184863067	184868023	184868059	94	40	4	0	1	0	1	11	0	0	98	49	1	1	1	0.952	1	1	1	-0.016
SE	13624	VPS8	VPS8	chr3	+	184868945	184869036	184868023	184868059	184869481	184869528	93	22	7	2	0	0	0	12	0	0	98	49	1	1	0.959	1	1	NA	1	-0.014
SE	13625	VPS8	VPS8	chr3	+	184914980	184915053	184913518	184913561	184915354	184915474	51	32	11	2	0	0	2	12	0	0	98	49	1	1	0.927	1	1	1	1	-0.024
SE	13628	SH3YL1	SH3YL1	chr2	-	224863	224920	218135	219001	229965	230044	0	0	0	1	0	0	0	3	0	0	98	49	1	1	0	NA	NA	NA	1	-1
SE	13636	CAMLG	CAMLG	chr5	+	134741062	134741523	134738479	134738792	134743986	134744052	8	2	4	0	0	0	0	0	0	1	98	49	1	1	1	1	1	NA	0	1
SE	13638	CAMLG	CAMLG	chr5	+	134743986	134744052	134738479	134738792	134750758	134752160	4	4	1	2	2	0	0	1	0	0	98	49	1	1	0.5	0.5	1	NA	1	-0.333
SE	13640	EPSTI1	EPSTI1	chr13	-	42889210	42889242	42886387	42888502	42895008	42895108	0	1	0	0	0	0	0	8	0	4	81	49	1	1	NA	1	NA	NA	0.547	0.453
SE	13641	EPSTI1	EPSTI1	chr13	-	42895008	42895108	42886387	42888502	42900309	42900383	2	5	0	0	0	0	1	9	0	2	98	49	1	1	1	1	NA	1	0.692	0.154
SE	13643	EPSTI1	EPSTI1	chr13	-	42970611	42970670	42969093	42969177	42991977	42992271	5	5	0	0	0	0	0	25	1	0	98	49	1	1	1	1	NA	0	1	0.5
SE	13644	DOPEY2	DOPEY2	chr21	+	36211552	36211651	36208714	36208904	36211973	36212097	3	1	2	0	0	0	2	1	0	1	98	49	1	1	1	1	1	1	0.333	0.334
SE	13645	DOPEY2	DOPEY2	chr21	+	36214080	36214190	36211973	36212097	36214441	36214556	3	1	0	0	0	0	2	6	0	1	98	49	1	1	1	1	NA	1	0.75	0.125
SE	13660	CLK1	CLK1	chr2	-	200857717	200857884	200856890	200856985	200857972	200858089	45	41	28	3	2	1	0	12	0	0	98	49	1	1	0.882	0.911	0.933	NA	1	-0.091
SE	13661	CLK1	CLK1	chr2	-	200857972	200858089	200856890	200856985	200859679	200859746	53	18	16	1	0	0	1	1	0	0	98	49	1	1	0.964	1	1	1	1	-0.012
SE	13662	CLK1	CLK1	chr2	-	200860124	200860215	200859679	200859746	200861237	200861466	7	1	3	18	9	6	0	0	0	2	98	49	1	1	0.163	0.053	0.2	NA	0	0.139
SE	13669	STYXL1	STYXL1	chr7	-	76013741	76013887	76005258	76005404	76021850	76021992	2	2	0	0	0	3	0	1	0	0	98	49	1	1	1	1	0	NA	1	-0.333
SE	13672	LOC101929567	LOC101929567	chr2	-	8579910	8580047	8578569	8578711	8583716	8583792	0	1	0	0	0	0	0	0	0	2	98	49	1	1	NA	1	NA	NA	0	1
SE	13674	ADAM17	ADAM17	chr2	-	9492897	9492986	9491100	9491151	9493746	9493825	26	3	3	1	0	0	0	7	0	0	98	49	1	1	0.929	1	1	NA	1	-0.024
SE	13675	ADAM17	ADAM17	chr2	-	9518102	9518247	9517900	9517989	9521202	9521316	18	10	3	3	0	0	0	10	0	0	98	49	1	1	0.75	1	1	NA	1	-0.083
SE	13676	ADAM17	ADAM17	chr2	-	9535833	9535922	9527785	9527954	9536697	9536828	17	2	3	0	0	0	0	3	0	1	98	49	1	1	1	1	1	NA	0.6	0.4
SE	13677	ADAM17	ADAM17	chr2	-	9536697	9536828	9527785	9527954	9543152	9543285	5	2	4	2	0	0	0	2	0	0	98	49	1	1	0.556	1	1	NA	1	-0.148
SE	13683	XPO1	XPO1	chr2	-	61485767	61485962	61483936	61484105	61488164	61488271	8	0	2	1	0	0	0	4	0	0	98	49	1	1	0.8	NA	1	NA	1	-0.1
SE	13684	XPO1	XPO1	chr2	-	61488587	61488771	61488164	61488271	61490641	61490776	24	3	6	3	0	0	0	8	0	0	98	49	1	1	0.8	1	1	NA	1	-0.067
SE	13685	XPO1	XPO1	chr2	-	61492914	61493053	61492566	61492748	61493893	61494091	18	16	0	0	0	0	0	4	0	2	98	49	1	1	1	1	NA	NA	0.5	0.5
SE	13686	PQLC3	PQLC3	chr2	+	11171924	11172045	11164203	11164261	11174967	11175009	3	2	3	0	1	0	0	1	0	1	98	49	1	1	1	0.5	1	NA	0.333	0.5
SE	13693	AAED1	AAED1	chr9	-	96645892	96646024	96641250	96641886	96651389	96651495	2	0	0	0	1	0	0	1	0	1	98	49	1	1	1	0	NA	NA	0.333	0.167
SE	13694	FAM49A	FAM49A	chr2	-	16562004	16562141	16561455	16561533	16563988	16564094	24	8	2	0	2	0	3	10	0	0	98	49	1	1	1	0.667	1	1	1	-0.111
SE	13695	FAM49A	FAM49A	chr2	-	16563988	16564094	16561455	16561533	16565645	16565767	9	10	2	0	0	0	0	11	0	2	98	49	1	1	1	1	1	NA	0.733	0.267
SE	13697	FAM49A	FAM49A	chr2	-	16565645	16565767	16561455	16561533	16588049	16588129	16	6	5	1	0	0	0	4	0	0	98	49	1	1	0.889	1	1	NA	1	-0.037
SE	13698	FAM49A	FAM49A	chr2	-	16565645	16565767	16563988	16564094	16588049	16588129	25	14	7	1	0	0	0	13	0	0	98	49	1	1	0.926	1	1	NA	1	-0.025
SE	13700	ARPP19	ARPP19	chr15	-	52564189	52564246	52557099	52557222	52568847	52568902	6	0	0	20	1	0	0	2	0	0	98	49	1	1	0.13	0	NA	NA	1	-0.935
SE	13701	ROCK1	ROCK1	chr18	-	21042564	21042709	21042096	21042235	21044101	21044186	40	18	2	0	1	0	0	4	0	0	98	49	1	1	1	0.9	1	NA	1	-0.033
SE	13706	LAPTM4A	LAPTM4A	chr2	-	20037537	20037614	20037315	20037438	20040890	20041011	30	14	3	0	1	0	0	11	0	0	98	49	1	1	1	0.875	1	NA	1	-0.042
SE	13713	RBM38	RBM38	chr20	+	57393278	57393333	57392653	57392777	57407542	57409330	4	1	1	1	0	0	0	1	0	0	98	49	1	1	0.667	1	1	NA	1	-0.111
SE	13715	FAM228B	FAM228B	chr2	+	24137908	24138100	24135118	24135187	24139369	24139450	6	1	5	1	0	1	0	1	0	0	98	49	1	1	0.75	1	0.714	NA	1	-0.179
SE	13718	TP53I3	TP53I3	chr2	-	24079443	24079640	24077432	24077761	24080818	24081031	0	2	0	1	0	0	0	1	0	0	98	49	1	1	0	1	NA	NA	1	-0.5
SE	13719	ITSN2	ITSN2	chr2	-	24284762	24284843	24275712	24275849	24286211	24286351	2	1	0	29	15	2	0	0	0	3	98	49	1	1	0.033	0.032	0	NA	0	0.022
SE	13720	NCOA1	NCOA1	chr2	+	24705085	24705233	24697657	24697798	24706567	24707888	77	48	17	1	0	0	1	11	0	1	98	49	1	1	0.975	1	1	1	0.846	0.069
SE	13721	NCOA1	NCOA1	chr2	+	24762686	24762776	24757972	24758156	24768078	24768135	47	14	7	1	0	0	0	6	0	0	98	49	1	1	0.959	1	1	NA	1	-0.014
SE	13722	NCOA1	NCOA1	chr2	+	24768078	24768135	24762686	24762776	24768220	24770701	31	14	2	4	4	0	0	8	0	2	98	49	1	1	0.795	0.636	1	NA	0.667	0.143
SE	13723	NCOA1	NCOA1	chr2	+	24768078	24768135	24762686	24762776	24768223	24770701	16	12	2	1	0	0	0	3	0	0	98	49	1	1	0.889	1	1	NA	1	-0.037
SE	13725	RAPGEF2	RAPGEF2	chr4	+	159355852	159356158	159353486	159354046	159358113	159360149	20	14	4	0	1	0	0	2	0	0	98	49	1	1	1	0.875	1	NA	1	-0.042
SE	13729	DNMT3A	DNMT3A	chr2	-	25241561	25241707	25240639	25240730	25243897	25243982	5	6	0	0	0	0	0	0	0	1	98	49	1	1	1	1	NA	NA	0	1
SE	13735	SMG5	SMG5	chr1	-	156250857	156250996	156249223	156250670	156251402	156251477	6	4	0	0	0	2	0	1	0	0	98	49	1	1	1	1	0	NA	1	-0.333
SE	13737	SMG5	SMG5	chr1	-	156273360	156273450	156272319	156272398	156274596	156274686	11	3	2	0	0	0	0	3	0	1	98	49	1	1	1	1	1	NA	0.6	0.4
SE	13740	BIRC6	BIRC6	chr2	+	32435495	32435585	32433643	32433804	32436052	32436184	5	2	0	4	0	0	0	1	0	0	98	49	1	1	0.385	1	NA	NA	1	-0.307
SE	13741	BIRC6	BIRC6	chr2	+	32464508	32464823	32463193	32463381	32465064	32465164	22	23	3	0	0	0	0	1	0	1	98	49	1	1	1	1	1	NA	0.333	0.667
SE	13742	BIRC6	BIRC6	chr2	+	32499546	32500109	32493539	32493667	32501712	32501888	20	14	0	1	0	0	0	13	0	0	98	49	1	1	0.909	1	NA	NA	1	-0.045
SE	13743	NBN	NBN	chr8	-	89946139	89946295	89943252	89943366	89947823	89947892	30	16	13	1	0	0	0	6	0	0	98	49	1	1	0.937	1	1	NA	1	-0.021
SE	13745	NBN	NBN	chr8	-	89980733	89980893	89971172	89971290	89981374	89981523	10	1	1	1	0	0	0	1	0	0	98	49	1	1	0.833	1	1	NA	1	-0.056
SE	13746	NBN	NBN	chr8	-	89981952	89982002	89981374	89981523	89982721	89982855	3	2	2	3	0	1	0	2	0	2	98	49	1	1	0.333	1	0.5	NA	0.333	0.278
SE	13751	ZBTB8OS	ZBTB8OS	chr1	-	32631826	32631879	32627507	32627544	32633644	32633727	6	7	8	0	2	0	0	1	0	0	98	49	1	1	1	0.636	1	NA	1	-0.121
SE	13756	ACIN1	ACIN1	chr14	-	23089981	23090101	23081747	23081836	23090521	23090633	17	4	13	2	0	0	1	5	0	0	98	49	1	1	0.81	1	1	1	1	-0.063
SE	13758	ERGIC1	ERGIC1	chr5	+	172909666	172909761	172897001	172897074	172914713	172914838	92	46	44	0	0	0	1	13	0	1	98	49	1	1	1	1	1	1	0.867	0.067
SE	13759	ERGIC1	ERGIC1	chr5	+	172924004	172924109	172914713	172914838	172926508	172926569	58	26	32	0	0	4	0	24	0	0	98	49	1	1	1	1	0.8	NA	1	-0.067
SE	13760	MRPL33	MRPL33	chr2	+	27774423	27774530	27772673	27772692	27779432	27779741	4	1	0	1	0	0	0	0	0	1	98	49	1	1	0.667	1	NA	NA	0	0.834
SE	13762	GFER	GFER	chr16	+	1984746	1984943	1984148	1984476	1985865	1987749	1	0	0	2	0	0	0	1	0	0	98	49	1	1	0.2	NA	NA	NA	1	-0.8
SE	13763	BRE	BRE	chr2	+	27892382	27892448	27890614	27890842	27894532	27894684	3	0	0	0	0	0	0	1	0	3	98	49	1	1	1	NA	NA	NA	0.143	0.857
SE	13765	BRE	BRE	chr2	+	28327273	28327447	28298337	28298491	28338449	28338900	1	0	0	1	0	0	0	1	0	0	98	49	1	1	0.333	NA	NA	NA	1	-0.667
SE	13767	ZSWIM6	ZSWIM6	chr5	+	61490785	61490934	61472680	61473037	61494259	61494410	17	6	1	1	0	0	0	4	0	0	98	49	1	1	0.895	1	1	NA	1	-0.035
SE	13778	MTRR	MTRR	chr5	+	7886614	7886703	7885700	7885854	7889094	7889275	3	1	0	0	0	0	0	0	0	1	98	49	1	1	1	1	NA	NA	0	1
SE	13779	GALNT14	GALNT14	chr2	-	30944857	30944942	30942200	30942304	30945782	30945870	1	1	1	0	1	0	0	1	0	0	98	49	1	1	1	0.333	1	NA	1	-0.222
SE	13781	GALNT14	GALNT14	chr2	-	30992837	30993007	30966203	30966302	31137957	31138726	3	2	0	0	1	0	1	3	0	0	98	49	1	1	1	0.5	NA	1	1	-0.25
SE	13785	SPAST	SPAST	chr2	+	32098795	32098891	32089521	32089605	32114637	32114825	25	10	5	2	0	0	0	2	0	0	98	49	1	1	0.862	1	1	NA	1	-0.046
SE	13804	PARP14	PARP14	chr3	+	122703741	122703978	122699389	122701635	122704526	122704748	66	23	3	0	0	0	6	21	1	0	98	49	1	1	1	1	1	0.75	1	0.125
SE	13805	PARP14	PARP14	chr3	+	122704526	122704748	122699389	122701635	122708189	122708268	18	2	0	1	1	0	2	3	0	1	98	49	1	1	0.9	0.5	NA	1	0.6	-0.1
SE	13809	HNRNPLL	HNRNPLL	chr2	-	38569803	38569925	38569132	38569334	38573209	38573427	4	6	1	1	0	0	0	2	0	0	98	49	1	1	0.667	1	1	NA	1	-0.111
SE	13811	APEH	APEH	chr3	+	49676776	49676817	49676608	49676700	49676902	49677024	5	10	4	0	0	0	0	4	0	1	90	49	1	1	1	1	1	NA	0.685	0.315
SE	13812	APEH	APEH	chr3	+	49679592	49679644	49678851	49678949	49680540	49680629	10	0	6	0	0	1	0	4	0	0	98	49	1	1	1	NA	0.75	NA	1	-0.125
SE	13813	APEH	APEH	chr3	+	49681721	49681805	49681100	49681239	49681886	49681967	6	1	2	2	0	0	0	2	0	0	98	49	1	1	0.6	1	1	NA	1	-0.133
SE	13814	MAP4K3	MAP4K3	chr2	-	39307942	39308005	39299742	39299801	39309460	39309519	1	0	0	0	0	1	0	3	0	0	98	49	1	1	1	NA	0	NA	1	-0.5
SE	13815	MEF2A	MEF2A	chr15	+	99598429	99598511	99565927	99566104	99632977	99633173	4	0	0	1	0	0	0	2	0	0	98	49	1	1	0.667	NA	NA	NA	1	-0.333
SE	13818	MEF2A	MEF2A	chr15	+	99690240	99690428	99675398	99675458	99710633	99710760	13	1	7	0	0	0	0	7	0	1	98	49	1	1	1	1	1	NA	0.778	0.222
SE	13820	MEF2A	MEF2A	chr15	+	99706728	99706855	99690240	99690428	99710633	99710760	9	0	0	0	0	0	0	6	0	1	98	49	1	1	1	NA	NA	NA	0.75	0.25
SE	13829	EML4	EML4	chr2	+	42301240	42301392	42295380	42295516	42303103	42303229	17	11	2	1	0	0	0	6	0	0	98	49	1	1	0.895	1	1	NA	1	-0.035
SE	13830	SYK	SYK	chr9	+	90867130	90867199	90865047	90865097	90874203	90874291	31	22	12	1	0	0	0	3	0	0	98	49	1	1	0.939	1	1	NA	1	-0.02
SE	13844	SRBD1	SRBD1	chr2	-	45476992	45477075	45419787	45419894	45488239	45488331	11	4	1	0	1	0	0	2	0	0	98	49	1	1	1	0.667	1	NA	1	-0.111
SE	13845	SRBD1	SRBD1	chr2	-	45547521	45547612	45546731	45546839	45551124	45551282	15	9	8	0	1	0	0	3	0	0	98	49	1	1	1	0.818	1	NA	1	-0.061
SE	13846	RHOQ	RHOQ	chr2	+	46543753	46543812	46542727	46543188	46576086	46576251	13	7	2	0	0	0	0	0	0	1	98	49	1	1	1	1	1	NA	0	1

SE	13847	MYLIP	MYLIP	chr6	+	16130556	16130747	16129085	16129409	16141624	16141810	15	10	9	0	2	0	0	3	0	0	98	49	1	1	1	0.714	1	NA	1	-0.095
SE	13856	GNAS	GNAS	chr20	+	58909683	58909804	58909520	58909579	58909950	58910081	83	39	33	1	0	0	8	35	0	0	98	49	1	1	0.976	1	1	1	1	-0.008
SE	13857	CALM2	CALM2	chr2	-	47172413	47172459	47170733	47170764	47176440	47176623	1	0	0	15	11	3	0	0	0	1	95	49	1	1	0.033	0	0	NA	0	0.011
SE	13861	TBCK	TBCK	chr4	-	106254946	106255047	106251865	106252007	106260436	106260510	0	0	0	1	0	0	0	3	0	0	98	49	1	1	0	NA	NA	NA	1	-1
SE	13862	PPP1R21	PPP1R21	chr2	+	48471086	48471188	48465492	48465642	48479923	48480016	8	4	3	1	0	0	0	2	0	0	98	49	1	1	0.8	1	1	NA	1	-0.067
SE	13874	EIF4E3	EIF4E3	chr3	-	71696459	71696520	71693874	71693941	71699613	71699708	60	32	41	1	0	0	0	13	0	1	98	49	1	1	0.968	1	1	NA	0.867	0.122
SE	13875	EIF4E3	EIF4E3	chr3	-	71696459	71696520	71693874	71693941	71710411	71710484	29	15	15	2	2	0	0	3	0	0	98	49	1	1	0.879	0.789	1	NA	1	-0.111
SE	13876	EIF4E3	EIF4E3	chr3	-	71699613	71699708	71693874	71693941	71710411	71710484	11	12	8	2	2	0	0	2	0	0	98	49	1	1	0.733	0.75	1	NA	1	-0.172
SE	13877	EIF4E3	EIF4E3	chr3	-	71699613	71699708	71696459	71696520	71710411	71710484	44	29	34	3	0	0	0	11	0	0	98	49	1	1	0.88	1	1	NA	1	-0.04
SE	13878	EIF4E3	EIF4E3	chr3	-	71728475	71728623	71710411	71710484	71754214	71754383	12	7	2	0	0	0	0	10	0	1	98	49	1	1	1	1	1	NA	0.833	0.167
SE	13879	PSME4	PSME4	chr2	-	53899880	53900017	53898300	53898354	53901349	53901559	8	1	2	1	2	0	0	2	0	0	98	49	1	1	0.8	0.2	1	NA	1	-0.333
SE	13880	PPIP5K2	PPIP5K2	chr5	+	103176865	103176910	103173857	103173972	103177666	103177774	4	1	0	4	1	0	0	2	0	1	94	49	1	1	0.343	0.343	NA	NA	0.51	-0.167
SE	13881	PPIP5K2	PPIP5K2	chr5	+	103183233	103183407	103180020	103180188	103184671	103184744	2	0	0	6	4	0	0	0	1	0	98	49	1	1	0.143	0	NA	0	NA	0.071
SE	13882	PPIP5K2	PPIP5K2	chr5	+	103186319	103186439	103184671	103184744	103190841	103190982	1	0	0	3	1	0	0	1	0	0	98	49	1	1	0.143	0	NA	NA	1	-0.928
SE	13883	PPIP5K2	PPIP5K2	chr5	+	103187313	103187376	103186319	103186439	103190841	103190982	0	6	1	0	0	0	0	0	0	1	98	49	1	1	NA	1	1	NA	0	1
SE	13886	CCDC88A	CCDC88A	chr2	-	55312433	55312579	55309161	55309254	55315927	55316114	0	0	0	2	0	0	0	6	0	0	98	49	1	1	0	NA	NA	NA	1	-1
SE	13891	VRK2	VRK2	chr2	+	58154753	58154802	58146315	58146474	58159348	58159920	0	1	0	1	1	0	0	2	0	1	98	49	1	1	0	0.333	NA	NA	0.5	-0.334
SE	13892	GPBP1L1	GPBP1L1	chr1	-	45634095	45634236	45633488	45633647	45640209	45640403	28	8	1	0	0	2	0	13	0	0	98	49	1	1	1	1	0.2	NA	1	-0.267
SE	13893	GPBP1L1	GPBP1L1	chr1	-	45685575	45685620	45660183	45661225	45686411	45686630	17	14	0	1	1	0	0	4	0	0	94	49	1	1	0.899	0.879	NA	NA	1	-0.111
SE	13896	USP34	USP34	chr2	-	61192900	61192980	61190517	61190658	61203139	61203263	32	4	3	0	0	0	0	3	0	1	98	49	1	1	1	1	1	NA	0.6	0.4
SE	13897	USP34	USP34	chr2	-	61204255	61204380	61203139	61203263	61204496	61204601	12	7	9	1	0	0	0	4	0	0	98	49	1	1	0.857	1	1	NA	1	-0.048
SE	13898	USP34	USP34	chr2	-	61214059	61214694	61211771	61211929	61220309	61220457	16	2	3	3	0	0	0	2	0	0	98	49	1	1	0.727	1	1	NA	1	-0.091
SE	13900	USP34	USP34	chr2	-	61265396	61265557	61259710	61259776	61265983	61266167	16	19	4	1	0	0	0	3	0	0	98	49	1	1	0.889	1	1	NA	1	-0.037
SE	13909	ATP8A1	ATP8A1	chr4	-	42485495	42485668	42464892	42465076	42503449	42503514	4	6	1	1	0	0	0	1	0	1	98	49	1	1	0.667	1	1	NA	0.333	0.556
SE	13912	ATP8A1	ATP8A1	chr4	-	42594288	42594362	42588259	42588329	42600477	42600518	8	5	0	2	1	1	0	1	0	0	98	49	1	1	0.667	0.714	0	NA	1	-0.54
SE	13913	AFTPH	AFTPH	chr2	+	64569091	64569218	64567561	64567713	64569622	64569679	32	6	14	2	0	0	0	3	0	0	98	49	1	1	0.889	1	1	NA	1	-0.037
SE	13921	NEURL4	NEURL4	chr17	-	7327164	7327230	7326710	7327009	7327439	7327884	1	0	3	1	1	0	0	1	0	0	98	49	1	1	0.333	0	1	NA	1	-0.556
SE	13929	G3BP2	G3BP2	chr4	-	75653982	75654081	75648638	75648741	75655065	75655246	3	0	0	17	7	2	0	1	0	3	98	49	1	1	0.081	0	0	NA	0.143	-0.116
SE	13930	G3BP2	G3BP2	chr4	-	75656923	75657014	75655767	75655870	75657556	75657730	20	5	0	1	0	0	0	6	0	0	98	49	1	1	0.909	1	NA	NA	1	-0.045
SE	13933	TRAPPC9	TRAPPC9	chr8	-	140426614	140426641	140405576	140405698	140435111	140435240	5	4	0	2	0	0	0	4	0	0	76	49	1	1	0.617	1	NA	NA	1	-0.192
SE	13935	AAK1	AAK1	chr2	-	69532040	69532162	69530624	69530706	69542522	69542665	10	1	2	1	0	0	0	1	0	0	98	49	1	1	0.833	1	1	NA	1	-0.056
SE	13939	ATP2B1	ATP2B1	chr12	-	89603711	89603925	89603042	89603254	89604154	89604346	11	11	2	1	0	0	0	6	0	0	98	49	1	1	0.846	1	1	NA	1	-0.051
SE	13940	ATP2B1	ATP2B1	chr12	-	89616801	89617039	89611192	89611372	89619998	89620240	39	13	12	3	0	0	0	11	0	0	98	49	1	1	0.867	1	1	NA	1	-0.044
SE	13945	ZNF638	ZNF638	chr2	+	71408121	71408247	71406127	71406262	71418601	71418639	12	3	4	1	0	0	0	3	0	0	98	49	1	1	0.857	1	1	NA	1	-0.048
SE	13946	DYSF	DYSF	chr2	+	71511806	71511921	71503213	71503319	71513715	71513921	43	15	11	0	0	0	0	5	0	1	98	49	1	1	1	1	1	NA	0.714	0.286
SE	13948	DYSF	DYSF	chr2	+	71520177	71520208	71516988	71517039	71520788	71520904	73	27	16	0	0	0	1	12	1	0	80	49	1	1	1	1	1	0.38	1	0.31
SE	13950	DYSF	DYSF	chr2	+	71570228	71570334	71569819	71569934	71570598	71570741	107	69	18	0	1	0	5	39	0	0	98	49	1	1	1	0.972	1	1	1	-0.009
SE	13951	DYSF	DYSF	chr2	+	71574197	71574371	71570598	71570741	71589592	71589686	85	27	20	0	2	0	5	17	0	0	98	49	1	1	1	0.871	1	1	1	-0.043
SE	13952	DYSF	DYSF	chr2	+	71620546	71620609	71613333	71613410	71643964	71644063	19	13	4	9	13	8	0	13	0	3	98	49	1	1	0.514	0.333	0.2	NA	0.684	-0.335
SE	13954	DYSF	DYSF	chr2	+	71668753	71668842	71667375	71667515	71669604	71669746	9	3	1	1	0	0	0	8	0	0	98	49	1	1	0.818	1	1	NA	1	-0.061
SE	13955	DYSF	DYSF	chr2	+	71669111	71669207	71668753	71668842	71669604	71669746	54	16	13	0	0	0	0	17	0	3	98	49	1	1	1	1	1	NA	0.739	0.261
SE	13956	DYSF	DYSF	chr2	+	71674196	71674296	71669604	71669746	71679056	71679235	94	46	36	0	1	0	3	26	0	0	98	49	1	1	1	0.958	1	1	1	-0.014
SE	13964	CCT7	CCT7	chr2	+	73243996	73244049	73243003	73243129	73244543	73244715	6	2	12	0	0	0	0	0	1	0	98	49	1	1	1	1	1	0	NA	1
SE	13965	CCT7	CCT7	chr2	+	73249818	73249916	73248990	73249179	73250305	73250438	5	2	3	2	0	0	1	0	0	0	98	49	1	1	0.556	1	1	1	NA	-0.148
SE	13966	CCT7	CCT7	chr2	+	73251225	73251432	73250305	73250438	73252639	73253022	10	1	1	0	1	0	0	1	0	0	98	49	1	1	1	0.333	1	NA	1	-0.222
SE	13969	MSN	MSN	chrX	+	65731837	65731984	65731106	65731190	65733183	65733280	186	98	81	0	1	0	1	29	0	0	98	49	1	1	1	0.98	1	1	1	-0.007
SE	13970	MSN	MSN	chrX	+	65738524	65738617	65737177	65737338	65738969	65739194	138	48	47	0	0	0	3	22	0	1	98	49	1	1	1	1	1	1	0.917	0.041
SE	13973	PSMA3-AS1	PSMA3-AS1	chr14	-	58285590	58285756	58273978	58274010	58291634	58291707	6	3	0	1	3	1	0	1	0	2	98	49	1	1	0.75	0.333	0	NA	0.2	0.161
SE	13974	PSMA3-AS1	PSMA3-AS1	chr14	-	58285590	58285756	58273978	58274135	58291634	58291707	13	5	4	6	1	4	0	1	0	0	98	49	1	1	0.52	0.714	0.333	NA	1	-0.478
SE	13975	INTS3	INTS3	chr1	+	153763232	153763362	153762727	153762847	153763831	153763886	29	4	1	1	0	0	0	4	0	0	98	49	1	1	0.935	1	1	NA	1	-0.022
SE	13976	STAMBP	STAMBP	chr2	+	73847386	73847753	73845166	73845262	73849362	73849487	8	0	7	0	2	0	0	2	0	0	98	49	1	1	1	0	1	NA	1	-0.333
SE	13981	TET3	TET3	chr2	+	74046277	74048411	74003109	74003166	74073548	74073639	17	4	6	0	1	0	0	4	0	0	98	49	1	1	1	0.667	1	NA	1	-0.111
SE	13989	APMAP	APMAP	chr20	-	24968891	24969084	24962943	24964022	24969525	24969660	47	18	23	0	0	1	2	2	0	0	98	49	1	1	1	1	0.92	1	1	-0.027
SE	13990	APMAP	APMAP	chr20	-	24978766	24978882	24973644	24973737	24983902	24984019	52	17	38	0	0	0	1	5	0	1	98	49	1	1	1	1	1	1	0.714	0.143
SE	13991	APMAP	APMAP	chr20	-	24983902	24984019	24973644	24973737	24992593	24992789	13	9	2	1	0	0	0	3	0	0	98	49	1	1	0.867	1	1	NA	1	-0.044
SE	13993	VPS13B	VPS13B	chr8	+	99134631	99134727	99121176	99121445	99135014	99135137	94	29	11	0	2	0	2	12	0	0	98	49	1	1	1	0.879	1	1	1	-0.04
SE	13994	VPS13B	VPS13B	chr8	+	99156548	99156743	99147840	99148010	99170038	99170163	90	46	2	0	1	0	0	24	0	0	98	49	1	1	1	0.958	1	NA	1	-0.014
SE	13995	VPS13B	VPS13B	chr8	+	99507136	99507203	99502835	99502950	99507769	99507911	26	5	0	1	0	0	0	3	0	0	98	49	1	1	0.929	1	NA	NA	1	-0.035
SE	13996	VPS13B	VPS13B	chr8	+	99507136	99507203	99502835	99502950	99511103	99511512	38	11	0	1	0	0	0	5	0	0	98	49	1	1	0.95	1	NA	NA	1	-0.025
SE	13997	VPS13B	VPS13B	chr8	+	99507769	99507911	99502835	99502950	99511103	99511512	4	1	0	1	0	0	0	2	0	0	98	49	1	1	0.667	1	NA	NA	1	-0.166
SE	13998	VPS13B	VPS13B	chr8	+	99507769	99507911	99507136	99507203	99511103	99511512	4	1	0	13	6	0	0	2	0	2	98	49	1	1	0.133	0.077	NA	NA	0.333	-0.228
SE	14004	HK2	HK2	chr2	+	74882119	74882239	74881710	74881859	74885493	74885589	4	10	1	1	0	0	0	3	0	0	98	49	1	1	0.667	1	1	NA	1	-0.111
SE	14011	ELMOD3	ELMOD3	chr2	+	85355554	85355598	85355075	85355170	85356966	85357252	1	0	0	0	0	0	0	1	0	1	93	49	1	1	1	NA	NA	NA	0.345	0.655
SE	14015	RNF181	RNF181	chr2	+	85596821	85596931	85596518	85596649	85597103	85597178	12	22	10	0	0	5	0	1	0	0	98	49	1	1	1	1	0.5	NA	1	-0.167
SE	14016	RNF181	RNF181	chr2	+	85597103	85597178	85596518	85596649	85597444	85597708	21	4	11	0	1	0	1	4	0	0	98	49	1	1	1	0.667	1	1	1	-0.111
SE	14021	GADD45A	GADD45A	chr1	+	67686024	67686126	67685176	67685538	67687660	67688338	4	0	2	0	0	0	0	0	0	1	98	49	1	1	1	NA	1	NA	0	1
SE	14022	GADD45A	GADD45A	chr1	+	67686349	67686587	67685176	67685538	67687660	67688338	3	4	1	0	0	0	0	0	0	1	98	49	1	1	1	1	1	NA	0	1
SE	14026	PTCD3	PTCD3	chr2	+	86125454	86125515	86124994	86125082	86125794	86125880	11	5	1	1	0	0	0	2	0	0	98	49	1	1	0.846	1	1	NA	1	-0.051
SE	14028	TPCN2	TPCN2	chr11	+	69054032	69054097	69048881	69049106	69062883	69062990	6	12	1	1	0	0	2	2	0	1	98	49	1	1	0.75	1	1	1	0.5	0.167
SE	14029	TPCN2	TPCN2	chr11	+	69054720	69054797	69054032	69054097	69055174	69055352	0	3	3	0	0	0	0	2	0	1	98	49	1	1	NA	1	1	NA	0.5	0.5
SE	14030	TPCN2	TPCN2	chr11	+	69055174	69055352	69054032	69054097	69057577	69057694	3	0	0	0	1	0	0	3	0	1	98	49	1	1	1	0	NA	NA	0.6	-0.1
SE	14031	TPCN2	TPCN2	chr11	+	69057577	69057694	69054032	69054097	69062883	69062990	0	3	1	0	4	0	0	2	0	0	98	49	1	1	NA	0.273	1	NA	1	-0.363
SE	14032	TPCN2	TPCN2	chr11	+	69072626	69072708	69071922	69072023	69072914	69073001	7	5	0	0	0	0	2	0	0	1	98	49	1	1	1	1	NA	1	0	0.5
SE	14033	TPCN2	TPCN2	chr11	+	69072914	69073001	69071922	69072023	69078481	69078601	5	2	0	0	1	0	0	2	0	2	98	49	1	1	1	0.5	NA	NA	0.333	0.417
SE	14034	TPCN2	TPCN2	chr11	+	69079833	69079883	69078891	69079020	69081399	69081499	7	2	2	0	1	0	0	2	0	0	98	49	1	1	1	0.5	1	NA	1	-0.167
SE	14037	CHMP3	CHMP3	chr2	-	86542251	86542312	86529217	86529397	86563303	86563497	18	5	8	2	0	0	0	1	0	0	98	49	1	1	0.818	1	1	NA	1	-0.061
SE	14042	HNRNPC	HNRNPC	chr14	-	21211405	21211566	21209135	21211306	21211809	21211923	69	43	28	0	0	0	0	6	0	2	98	49	1	1	1	1	1	NA	0.6	0.4
SE	14045	IRAK2	IRAK2	chr3	+	10238747	10239039	10234458	10234659	10242115	10243743	1	0	0	1	0	0	0	2	0	0	98	49	1	1	0.333	NA	NA	NA	1	-0.667
SE	14054	TMEM131	TMEM131	chr2	-	97759651	97759749	97758892	97759053	97760592	97760689	5	0	0	1	0	0	0	2	0	0	98	49	1	1	0.714	NA	NA	NA	1	-0.286
SE	14055	TMEM131	TMEM131	chr2	-	97794929	97795115	97793394	97793553	97796217	97796404	5	0	0	0	0	0	0	2	0	2	98	49	1	1	1	NA	NA	NA	0.333	0.667
SE	14056	TMEM131	TMEM131	chr2	-	97796217	97796404	97793394	97793553	97796843	97796986	4	3	0	0	0	0	0	3	0	2	98	49	1	1	1	1	NA	NA	0.429	0.571
SE	14057	TMEM131	TMEM131	chr2	-	97797364	97797516	97796843	97796986	97801894	97801961	16	5	0	1	0	0	0	1	0	0	98	49	1	1	0.889	1	NA	NA	1	-0.055
SE	14058	TMEM131	TMEM131	chr2	-	97844144	97844261	97841814	97841937	97859303	97859427	5	2	1	1	0	0	0	1	0	0	98	49	1	1	0.714	1	1	NA	1	-0.095
SE	14059	PAXBP1	PAXBP1	chr21	-	32750916	32751032	32748498	32748698	32751118	32751218	6	0	0	1	0	0	0	3	0	0	98	49	1	1	0.75	NA	NA	NA	1	-0.25
SE	14070	TBC1D8	TBC1D8	chr2	-	101036017	101036168	101033543	101033758	101037531	101037708	7	0	3	1	0	0	0	2	0	0	98	49	1	1	0.778	NA	1	NA	1	-0.111
SE	14072	RNF149	RNF149	chr2	-	101286080	101286177	101281858	101282057	101288972	101289055	112	84	53	8	6	0	0	25	0	0	98	49	1	1	0.875	0.875	1	NA	1	-0.083
SE	14078	MAP4K4	MAP4K4	chr2	+	101831720	101831851	101829503	101829594	101834408	101834463	54	39	9	1	0	0	2	6	0	0	98	49	1	1	0.964	1	1	1	1	-0.012
SE	14079	MAP4K4	MAP4K4	chr2	+	101855976	101856138	101844100	101844311	101858995	101859082	28	13	4	3	0	0	0	1	0	0	98	49	1	1	0.824	1	1	NA	1	-0.059
SE	14081	MAP4K4	MAP4K4	chr2	+	101860824	101860986	101859642	101859864	101864929	101865036	8	3	2	10	3	1	0	1	0	2	98	49	1	1	0.286	0.333	0.5	NA	0.2	0.173
SE	14083	MAP4K4	MAP4K4	chr2	+	101863820	101864051	101860824	101860986	101864929	101865036	1	0	0	4	0	2	0	0	0	1	98	49	1	1	0.111	NA	0	NA	0	0.056
SE	14084	MAP4K4	MAP4K4	chr2	+	101868028	101868037	101867211	101867309	101869621	101869797	4	1	0	17	16	9	0	0	0	5	58	49	1	1	0.166	0.05	0	NA	0	0.072
SE	14086	MAP4K4	MAP4K4	chr2	+	101874081	101874252	101873646	101873764	101877002	101877146	28	24	5	1	0	0	0	14	0	0	98	49	1	1	0.933	1	1	NA	1	-0.022
SE	14087	MAP4K4	MAP4K4	chr2	+	101887087	101887237	101885186	101885287	101887777	101887937	63	23	22	2	0	0	0	10	0	0	98	49	1	1	0.94	1	1	NA	1	-0.02
SE	14088	MAP4K4	MAP4K4	chr2	+	101887087	101887261	101885186	101885287	101887777	101887937	30	19	17	2	0	0	0	6	0	0	98	49	1	1	0.882	1	1	NA	1	-0.039
SE	14089	MAP4K4	MAP4K4	chr2	+	101888795	101888935	101887777	101887937	101891165	101894690	60	19	26	1	0	0	0	8	0	0	98	49	1	1	0.968	1	1	NA	1	-0.011
SE	14092	IL18R1	IL18R1	chr2	+	102373936	102373993	102371952	102372118	102375906	102376063	2	0	0	1	0	0	0	2	0	4	98	49	1	1	0.5	NA	NA	NA	0.2	0.3
SE	14093	IL18RAP	IL18RAP	chr2	+	102423810	102424135	102423177	102423347	102424230	102424414	9	9	2	2	0	8	1	0	0	0	98	49	1	1	0.692	1	0.111	1	NA	-0.399
SE	14094	IL18RAP	IL18RAP	chr2	+	102443199	102443323	102441311	102441377	102445188	102445340	5	7	14	1	0	0	0	1	0	0	98	49	1	1	0.714	1	1	NA	1	-0.095
SE	14095	IL18RAP	IL18RAP	chr2	+	102450847	102451021	102447069	102447207	102451765	102452564	20	12	22	0	4	0	0	1	0	0	98	49	1	1	1	0.6	1	NA	1	-0.133
SE	14104	HADHB	HADHB	chr2	+	26260980	26261063	26254429	26254474	26263379	26263479	1	1	0	10	1	3	0	1	0	2	98	49	1	1	0.048	0.333	0	NA	0.2	-0.073
SE	14106	HADHB	HADHB	chr2	+	26285406	26285571	26284882	26284957	26289917	26290465	10	7	1	1	1	0	2	11	0	0	98	49	1	1	0.833	0.778	1	1	1	-0.13
SE	14109	TPM4	TPM4	chr19	+	16093535	16093598	16089044	16089120	16093683	16093753	80	7	14	1	0	0	1	10	0	0	98	49	1	1	0.976	1	1	1	1	-0.008
SE	14122	BCL2L11	BCL2L11	chr2	+	111123732	111123869	111120913	111121188	111150043	111150147	3	1	0	2	0	0	0	1	0	1	98	49	1	1	0.429	1	NA	NA	0.333	0.382
SE	14123	BCL2L11	BCL2L11	chr2	+	111123732	111124139	111120913	111121188	111150043	111150147	9	0	1	2	0	0	0	1	0	1	98	49	1	1	0.692	NA	1	NA	0.333	0.513
SE	14136	TMEM87B	TMEM87B	chr2	+	112077191	112077282	112074911	112074962	112081056	112081118	9	0	0	1	0	0	0	1	0	0	98	49	1	1	0.818	NA	NA	NA	1	-0.182
SE	14137	TMEM87B	TMEM87B	chr2	+	112081334	112081518	112081056	112081118	112086004	112086104	3	0	0	1	0	0	0	1	0	0	98	49	1	1	0.6	NA	NA	NA	1	-0.4
SE	14142	CHCHD5	CHCHD5	chr2	+	112585973	112586114	112584436	112584649	112586199	112586365	2	1	0	1	0	0	0	1	0	0	98	49	1	1	0.5	1	NA	NA	1	-0.25
SE	14143	SLC20A1	SLC20A1	chr2	+	112647323	112647464	112646562	112647162	112647652	112647738	3	3	2	0	2	0	2	0	0	0	98	49	1	1	1	0.429	1	1	NA	-0.19
SE	14144	SLC20A1	SLC20A1	chr2	+	112660386	112660572	112659203	112659762	112661141	112661226	27	12	8	0	1	0	1	2	0	0	98	49	1	1	1	0.857	1	1	1	-0.048
SE	14145	IL1B	IL1B	chr2	-	112835565	112835617	112833373	112833575	112836182	112836244	72	61	3	2	0	0	0	3	0	0	98	49	1	1	0.947	1	1	NA	1	-0.018
SE	14146	PSD4	PSD4	chr2	+	113192379	113192589	113185876	113186255	113193047	113193128	31	16	13	0	3	0	0	11	0	0	98	49	1	1	1	0.727	1	NA	1	-0.091
SE	14147	PSD4	PSD4	chr2	+	113195726	113195770	113193858	113193948	113196146	113196307	24	29	14	1	1	0	2	12	0	0	93	49	1	1	0.927	0.939	1	1	1	-0.045
SE	14152	SLC35F5	SLC35F5	chr2	-	113717752	113717850	113712791	113715195	113719153	113719308	8	1	3	0	2	0	0	1	0	0	98	49	1	1	1	0.2	1	NA	1	-0.267
SE	14154	EMB	EMB	chr5	-	50410876	50410965	50405724	50405852	50411196	50411383	84	43	30	2	0	0	1	22	0	1	98	49	1	1	0.955	1	1	1	0.917	0.026
SE	14155	F5	F5	chr1	-	169523800	169523904	169523196	169523352	169524836	169524908	7	9	0	1	0	0	0	2	0	0	98	49	1	1	0.778	1	NA	NA	1	-0.111
SE	14165	ACSF3	ACSF3	chr16	+	89100661	89101347	89093808	89093996	89102603	89102759	4	0	0	0	2	0	0	1	0	0	98	49	1	1	1	0	NA	NA	1	-0.5
SE	14167	FAM188A	FAM188A	chr10	-	15838227	15838279	15837203	15837318	15841425	15841599	1	0	1	3	3	7	0	0	0	1	98	49	1	1	0.143	0	0.067	NA	0	0.07
SE	14177	MAP3K2	MAP3K2	chr2	-	127325727	127325807	127324173	127324241	127326686	127326817	78	26	17	4	5	0	0	17	0	0	98	49	1	1	0.907	0.722	1	NA	1	-0.124
SE	14178	MAP3K2	MAP3K2	chr2	-	127330391	127330505	127329920	127330008	127335869	127335969	56	19	17	1	0	0	0	7	0	0	98	49	1	1	0.966	1	1	NA	1	-0.011
SE	14186	UGGT1	UGGT1	chr2	+	128180889	128181072	128179785	128179870	128182129	128182290	13	4	6	2	0	0	0	3	0	0	98	49	1	1	0.765	1	1	NA	1	-0.078
SE	14189	ACOX1	ACOX1	chr17	-	75973624	75973785	75957458	75957566	75978533	75978693	8	3	1	9	4	0	0	3	0	0	98	49	1	1	0.308	0.273	1	NA	1	-0.473
SE	14193	CNST	CNST	chr1	+	246591511	246591941	246566336	246566663	246621428	246621634	7	2	0	1	0	0	0	1	0	0	98	49	1	1	0.778	1	NA	NA	1	-0.111
SE	14201	INSR	INSR	chr19	-	7166153	7166404	7163031	7163199	7167967	7168094	2	2	0	1	0	0	0	1	0	0	98	49	1	1	0.5	1	NA	NA	1	-0.25
SE	14215	MGAT5	MGAT5	chr2	+	134341589	134341759	134338258	134338420	134344929	134345064	20	1	2	1	0	0	1	2	0	0	98	49	1	1	0.909	1	1	1	1	-0.03
SE	14217	RRBP1	RRBP1	chr20	-	17616731	17616839	17615925	17616009	17618595	17618679	13	3	0	0	0	0	1	0	0	1	98	49	1	1	1	1	NA	1	0	0.5
SE	14218	RRBP1	RRBP1	chr20	-	17679998	17680075	17659979	17660528	17682027	17682283	7	2	0	2	1	0	0	0	1	0	98	49	1	1	0.636	0.5	NA	0	NA	0.568
SE	14222	RAB3GAP1	RAB3GAP1	chr2	+	135164593	135164696	135162985	135163101	135168544	135170709	15	9	5	0	0	1	0	2	0	0	98	49	1	1	1	1	0.714	NA	1	-0.095
SE	14237	ACVR2A	ACVR2A	chr2	+	147922972	147923111	147920229	147920344	147926030	147926161	1	0	0	1	0	0	0	2	0	0	98	49	1	1	0.333	NA	NA	NA	1	-0.667
SE	14239	GALK2	GALK2	chr15	+	49281986	49282085	49239220	49239367	49283565	49283718	4	1	0	0	1	0	0	2	0	0	98	49	1	1	1	0.333	NA	NA	1	-0.334
SE	14241	ORC4	ORC4	chr2	-	147981927	147982081	147975901	147975975	148020632	148020747	3	0	0	2	2	0	0	2	0	0	98	49	1	1	0.429	0	NA	NA	1	-0.785
SE	14242	ITGAL	ITGAL	chr16	+	30475305	30475400	30474195	30474298	30479090	30479208	40	17	2	0	1	0	0	12	0	0	98	49	1	1	1	0.895	1	NA	1	-0.035
SE	14243	ITGAL	ITGAL	chr16	+	30475512	30475580	30475305	30475400	30479090	30479208	44	17	15	1	0	0	0	17	0	0	98	49	1	1	0.957	1	1	NA	1	-0.014
SE	14244	ITGAL	ITGAL	chr16	+	30479090	30479208	30475512	30475580	30481438	30481584	42	10	11	4	0	0	0	18	0	1	98	49	1	1	0.84	1	1	NA	0.9	0.047
SE	14245	ITGAL	ITGAL	chr16	+	30479330	30479461	30479090	30479208	30481438	30481584	64	37	10	11	1	0	0	11	0	4	98	49	1	1	0.744	0.949	1	NA	0.579	0.319
SE	14247	ITGAL	ITGAL	chr16	+	30510880	30510960	30510360	30510471	30511049	30511136	65	20	9	1	0	0	2	13	0	1	98	49	1	1	0.97	1	1	1	0.867	0.056
SE	14251	SLC2A9	SLC2A9	chr4	-	9941912	9942045	9920384	9920572	9980591	9980737	1	0	0	0	0	0	0	0	0	2	98	49	1	1	1	NA	NA	NA	0	1
SE	14252	SLC2A9	SLC2A9	chr4	-	9985668	9985793	9980591	9980737	9996780	9996941	1	0	0	1	0	0	0	2	0	0	98	49	1	1	0.333	NA	NA	NA	1	-0.667
SE	14259	SH2D3C	SH2D3C	chr9	-	127741787	127741959	127740257	127740369	127742848	127742964	31	24	3	0	0	0	2	7	1	0	98	49	1	1	1	1	1	0.5	1	0.25
SE	14260	SH2D3C	SH2D3C	chr9	-	127761610	127761650	127751171	127751300	127773989	127774467	12	14	8	1	1	1	0	1	0	1	89	49	1	1	0.869	0.885	0.815	NA	0.355	0.501
SE	14262	SH2D3C	SH2D3C	chr9	-	127766900	127767271	127761610	127761650	127773989	127774467	2	3	0	3	13	6	0	0	0	1	98	49	1	1	0.25	0.103	0	NA	0	0.118
SE	14263	CYTIP	CYTIP	chr2	-	157418522	157418589	157414618	157416143	157427350	157427420	156	108	46	0	0	0	5	36	0	1	98	49	1	1	1	1	1	1	0.947	0.026
SE	14264	CYTIP	CYTIP	chr2	-	157430859	157430962	157430558	157430652	157434369	157434424	437	195	67	0	2	0	11	97	0	0	98	49	1	1	1	0.98	1	1	1	-0.007
SE	14265	CYTIP	CYTIP	chr2	-	157434369	157434424	157430859	157430962	157434697	157434747	436	196	66	2	0	0	9	93	0	0	98	49	1	1	0.991	1	1	1	1	-0.003
SE	14281	HDAC10	HDAC10	chr22	-	50247691	50247776	50246874	50246966	50247889	50248145	5	2	1	0	0	0	0	0	0	1	98	49	1	1	1	1	1	NA	0	1
SE	14287	MAP2K4	MAP2K4	chr17	+	12032265	12032298	12020817	12021001	12054888	12054991	0	1	0	2	0	0	1	1	0	0	82	49	1	1	0	1	NA	1	1	-0.5
SE	14289	MAP2K4	MAP2K4	chr17	+	12081355	12081530	12054888	12054991	12095574	12095694	10	5	5	0	5	0	0	6	0	0	98	49	1	1	1	0.333	1	NA	1	-0.222
SE	14291	MAP2K4	MAP2K4	chr17	+	12129138	12129287	12125293	12125371	12139838	12139884	13	8	7	1	0	0	0	1	0	0	98	49	1	1	0.867	1	1	NA	1	-0.044
SE	14298	ARHGDIB	ARHGDIB	chr12	-	14949801	14949885	14947872	14947949	14950531	14950724	464	197	202	0	0	0	13	90	0	1	98	49	1	1	1	1	1	1	0.978	0.011
SE	14311	UBE2F	UBE2F	chr2	+	237973648	237973747	237973091	237973225	237987962	237987992	1	0	0	4	0	0	0	0	0	1	98	49	1	1	0.111	NA	NA	NA	0	0.111
SE	14312	UBE2F	UBE2F	chr2	+	238016565	238016633	237994743	237994809	238025341	238025412	6	2	0	2	0	0	5	1	0	0	98	49	1	1	0.6	1	NA	1	1	-0.2
SE	14313	UBE2F	UBE2F	chr2	+	238035877	238035940	238032221	238032254	238041287	238042782	9	9	0	1	0	0	0	3	0	0	98	49	1	1	0.818	1	NA	NA	1	-0.091
SE	14317	EIF6	EIF6	chr20	-	35280653	35280829	35279941	35280118	35284175	35284261	5	2	0	1	1	0	0	5	0	0	98	49	1	1	0.714	0.5	NA	NA	1	-0.393
SE	14319	PDK1	PDK1	chr2	+	172562740	172562800	172562219	172562291	172564502	172564687	4	3	1	4	0	0	0	1	0	0	98	49	1	1	0.333	1	1	NA	1	-0.222
SE	14320	PDK1	PDK1	chr2	+	172562740	172562800	172562219	172562291	172564977	172565073	3	3	0	1	2	0	1	0	0	1	98	49	1	1	0.6	0.429	NA	1	0	0.014
SE	14321	PDK1	PDK1	chr2	+	172564502	172564687	172562219	172562291	172564977	172565073	8	0	2	1	2	0	0	0	0	1	98	49	1	1	0.8	0	1	NA	0	0.6

SE	14322	PDK1	PDK1	chr2	+	172564502	172564687	172562740	172562800	172564977	172565073	6	0	3	1	0	0	0	1	1	0	98	49	1	1	0.75	NA		1		0	1	0.375
SE	14323	PDK1	PDK1	chr2	+	172568740	172568817	172566855	172566933	172570725	172570824	9	5	1	1	1	0	0	2	0	0	98	49	1	1	0.818		0.714	1	NA		1	-0.156
SE	14326	SP3	SP3	chr2	-	173918592	173918785	173913069	173913266	173954872	173956232	72	44	13	0	0	0	0	19	0	1	98	49	1	1	1		1	1	NA		0.905	0.095
SE	14334	ATF2	ATF2	chr2	-	175121443	175121540	175118250	175118369	175130137	175130207	16	6	4	2	3	0	0	4	0	1	98	49	1	1	0.8		0.5	1	NA		0.667	0.1
SE	14336	ATF2	ATF2	chr2	-	175147945	175147980	175136411	175136486	175151059	175151158	2	0	0	8	2	0	0	0	0	2	84	49	1	1	0.127		0	NA	NA		0	0.064
SE	14345	SETX	SETX	chr9	-	132288235	132288351	132286422	132286494	132288549	132288651	76	23	10	0	1	0	0	7	0	0	98	49	1	1	1		0.92	1	NA		1	-0.027
SE	14346	SETX	SETX	chr9	-	132298079	132298312	132296886	132297054	132300629	132300803	103	13	3	0	0	0	0	16	0	1	98	49	1	1	1		1	1	NA		0.889	0.111
SE	14347	SETX	SETX	chr9	-	132326323	132330499	132311756	132311856	132331051	132331139	110	34	10	1	0	0	0	18	0	0	98	49	1	1	0.982		1	1	NA		1	-0.006
SE	14348	SETX	SETX	chr9	-	132334607	132334727	132331276	132331448	132336295	132336515	171	39	6	0	3	0	0	17	0	0	98	49	1	1	1		0.867	1	NA		1	-0.044
SE	14349	SETX	SETX	chr9	-	132334607	132334727	132331276	132331448	132342689	132342799	80	20	2	1	0	0	0	8	0	0	98	49	1	1	0.976		1	1	NA		1	-0.008
SE	14350	SETX	SETX	chr9	-	132336295	132336515	132331276	132331448	132342689	132342799	116	29	8	1	0	0	0	9	0	0	98	49	1	1	0.983		1	1	NA		1	-0.006
SE	14351	SETX	SETX	chr9	-	132336295	132336515	132334607	132334727	132342689	132342799	207	45	12	1	0	0	0	18	0	0	98	49	1	1	0.99		1	1	NA		1	-0.003
SE	14352	SETX	SETX	chr9	-	132342689	132342799	132336295	132336515	132346260	132346471	187	43	10	0	1	0	1	22	0	0	98	49	1	1	1		0.956	1		1	1	-0.015
SE	14355	SESTD1	SESTD1	chr2	-	179116667	179116790	179115064	179115256	179117531	179117613	3	0	0	0	0	0	0	5	0	1	98	49	1	1	1	NA		NA	NA		0.714	0.286
SE	14365	ITGA4	ITGA4	chr2	+	181475158	181475288	181474959	181475066	181478756	181478824	13	8	7	1	1	0	0	2	0	0	98	49	1	1	0.867		0.8	1	NA		1	-0.111
SE	14366	ITGA4	ITGA4	chr2	+	181530523	181530649	181529540	181529648	181531656	181531776	26	11	0	0	0	0	0	7	0	2	98	49	1	1	1		1	NA	NA		0.636	0.364
SE	14375	PUM1	PUM1	chr1	-	30941997	30942123	30941150	30941272	30945345	30945483	7	0	5	1	0	0	0	2	0	2	98	49	1	1	0.778	NA		1	NA		0.333	0.556
SE	14376	PUM1	PUM1	chr1	-	30964673	30964910	30953713	30953981	30965981	30966278	5	0	2	0	0	0	0	0	0	1	98	49	1	1	1	NA		1	NA		0	1
SE	14377	PUM1	PUM1	chr1	-	30980061	30980163	30974650	30974802	30981311	30981405	6	0	2	2	1	0	0	2	0	0	98	49	1	1	0.6		0	1	NA		1	-0.467
SE	14383	DNAJC10	DNAJC10	chr2	+	182752548	182752623	182752071	182752188	182755002	182755104	1	0	0	7	2	0	0	0	0	3	98	49	1	1	0.067		0	NA	NA		0	0.034
SE	14385	SMPDL3A	SMPDL3A	chr6	+	122795676	122795890	122789048	122789458	122796823	122796968	2	0	0	0	0	0	0	1	0	1	98	49	1	1	1	NA		NA	NA		0.333	0.667
SE	14386	ABHD14B	ABHD14B	chr3	-	51971459	51971698	51969942	51970184	51973964	51974630	2	0	0	0	0	0	0	0	0	1	98	49	1	1	1	NA		NA	NA		0	1
SE	14387	ABHD14B	ABHD14B	chr3	-	51971459	51971892	51969942	51970184	51973964	51974630	2	0	0	0	0	0	0	0	0	1	98	49	1	1	1	NA		NA	NA		0	1
SE	14391	HIBCH	HIBCH	chr2	-	190294545	190294630	190290404	190290485	190296812	190296953	3	0	0	1	0	0	0	1	0	0	98	49	1	1	0.6	NA		NA	NA		1	-0.4
SE	14399	STAT1	STAT1	chr2	-	190998216	190998308	190997855	190998007	190999625	190999704	112	56	20	3	5	0	0	33	0	2	98	49	1	1	0.949		0.848	1	NA		0.892	0.04
SE	14400	STAT1	STAT1	chr2	-	191001073	191001163	190999625	190999704	191007562	191007661	162	84	22	1	6	0	0	49	0	1	98	49	1	1	0.988		0.875	1	NA		0.961	-0.007
SE	14407	HSDL2	HSDL2	chr9	+	112416840	112416944	112408906	112409021	112418859	112418958	7	10	5	1	0	0	0	1	0	0	98	49	1	1	0.778		1	1	NA		1	-0.074
SE	14410	RPL32P3	RPL32P3	chr3	-	129397039	129397140	129393634	129393795	129399007	129399439	0	0	2	0	0	1	0	0	0	1	98	49	1	1	NA	NA		0.5	NA		0	0.5
SE	14411	ANKRD44	ANKRD44	chr2	-	196998906	196999052	196998336	196998419	197000418	197000502	57	21	15	2	2	0	0	9	0	5	98	49	1	1	0.934		0.84	1	NA		0.474	0.451
SE	14412	ANKRD44	ANKRD44	chr2	-	197000418	197000502	196998906	196999052	197001752	197001840	77	25	8	0	2	0	0	8	0	0	98	49	1	1	1		0.862	1	NA		1	-0.046
SE	14413	ANKRD44	ANKRD44	chr2	-	197013510	197013712	197008943	197009031	197025195	197025267	83	36	15	1	3	0	0	9	0	0	98	49	1	1	0.976		0.857	1	NA		1	-0.056
SE	14414	ANKRD44	ANKRD44	chr2	-	197078702	197078814	197025195	197025267	197081644	197081725	114	32	21	2	0	0	0	14	0	0	98	49	1	1	0.966		1	1	NA		1	-0.011
SE	14415	ANKRD44	ANKRD44	chr2	-	197086679	197086748	197083368	197083509	197088710	197088774	58	38	25	1	1	0	2	9	0	0	98	49	1	1	0.967		0.95	1		1	1	-0.028
SE	14423	SLC10A7	SLC10A7	chr4	-	146256825	146256936	146253980	146256520	146258691	146258837	0	0	0	2	0	0	0	2	0	0	98	49	1	1	0	NA		NA	NA		1	-1
SE	14427	ORC2	ORC2	chr2	-	200925835	200925932	200920992	200921139	200931338	200931448	4	0	0	0	0	0	0	0	0	1	98	49	1	1	1	NA		NA	NA		0	1
SE	14430	CFLAR	CFLAR	chr2	+	201145377	201145432	201140274	201140439	201149002	201149052	277	153	108	2	0	0	0	64	0	0	98	49	1	1	0.986		1	1	NA		1	-0.005
SE	14441	INO80D	INO80D	chr2	-	206063150	206063244	206062798	206063045	206085900	206086182	6	0	0	1	0	0	0	2	0	0	98	49	1	1	0.75	NA		NA	NA		1	-0.25
SE	14445	DCTN4	DCTN4	chr5	-	150742113	150742157	150733367	150733475	150753478	150753657	12	4	5	2	4	0	0	6	0	0	93	49	1	1	0.76		0.345	1	NA		1	-0.298
SE	14446	CCNYL1	CCNYL1	chr2	+	207733946	207734047	207726841	207726876	207737410	207737446	8	2	2	3	1	0	0	1	0	0	98	49	1	1	0.571		0.5	1	NA		1	-0.31
SE	14448	PIKFYVE	PIKFYVE	chr2	+	208273583	208273733	208271510	208271691	208285725	208285933	11	0	3	1	0	0	0	2	0	0	98	49	1	1	0.846	NA		1	NA		1	-0.077
SE	14450	PIKFYVE	PIKFYVE	chr2	+	208277536	208277708	208276711	208276830	208285725	208285933	10	4	3	2	0	0	0	5	0	0	98	49	1	1	0.714		1	1	NA		1	-0.095
SE	14452	PIKFYVE	PIKFYVE	chr2	+	208285725	208285933	208276711	208276830	208288728	208288818	13	9	0	1	0	0	0	1	0	0	98	49	1	1	0.867		1	NA	NA		1	-0.067
SE	14453	PIKFYVE	PIKFYVE	chr2	+	208285725	208285933	208277536	208277708	208288728	208288818	18	11	0	5	0	0	0	6	0	0	98	49	1	1	0.643		1	NA	NA		1	-0.178
SE	14454	PIKFYVE	PIKFYVE	chr2	+	208304845	208305013	208304170	208304318	208312235	208312295	4	1	2	2	7	0	0	1	0	4	98	49	1	1	0.5		0.067	1	NA		0.111	0.411
SE	14455	PIKFYVE	PIKFYVE	chr2	+	208333314	208333493	208330522	208330694	208335305	208335419	3	0	2	0	2	0	0	1	0	0	98	49	1	1	1		0	1	NA		1	-0.333
SE	14456	PIKFYVE	PIKFYVE	chr2	+	208347858	208348023	208346049	208346147	208350023	208350083	9	2	0	0	0	0	0	2	0	2	98	49	1	1	1		1	NA	NA		0.333	0.667
SE	14457	PIKFYVE	PIKFYVE	chr2	+	208351351	208351455	208350770	208350947	208352653	208352782	10	6	0	0	0	0	0	3	0	1	98	49	1	1	1		1	NA	NA		0.6	0.4
SE	14464	BARD1	BARD1	chr2	-	214792296	214792445	214780559	214781509	214809411	214809711	0	0	0	0	1	0	0	2	0	0	98	49	1	1	NA		0	NA	NA		1	-1
SE	14466	BARD1	BARD1	chr2	-	214797060	214797117	214780559	214781509	214809411	214809711	0	0	0	0	1	0	0	1	0	0	98	49	1	1	NA		0	NA	NA		1	-1
SE	14472	UBXN7	UBXN7	chr3	-	196369420	196369511	196368027	196368155	196371895	196372042	9	14	3	0	3	0	0	8	0	0	98	49	1	1	1		0.7	1	NA		1	-0.1
SE	14473	RELA	RELA	chr11	-	65658286	65658499	65655854	65655935	65658717	65658822	12	7	1	0	0	1	0	4	0	0	98	49	1	1	1		1	0.333	NA		1	-0.222
SE	14474	RELA	RELA	chr11	-	65658717	65658822	65655854	65655935	65659665	65659788	28	2	4	8	1	0	0	2	0	0	98	49	1	1	0.636		0.5	1	NA		1	-0.288
SE	14479	BAZ2A	BAZ2A	chr12	-	56604584	56604799	56604216	56604291	56605072	56605327	31	14	0	1	2	0	0	3	0	0	98	49	1	1	0.939		0.778	NA	NA		1	-0.141
SE	14481	BAZ2A	BAZ2A	chr12	-	56610408	56610517	56610113	56610215	56611572	56611633	34	13	4	0	2	0	1	2	0	0	98	49	1	1	1		0.765	1		1	1	-0.078
SE	14482	ATP5L	ATP5L	chr11	+	118405656	118405709	118401388	118401717	118406936	118407097	0	0	0	9	4	0	0	2	0	0	98	49	1	1	0		0	NA	NA		1	-1
SE	14486	ARPC2	ARPC2	chr2	+	218234351	218234397	218228737	218228850	218238663	218238850	168	112	91	1	0	0	4	32	0	0	95	49	1	1	0.989		1	1		1	1	-0.004
SE	14487	ARPC2	ARPC2	chr2	+	218238663	218238850	218228737	218228850	218245419	218245546	2	0	0	1	1	0	0	1	0	1	98	49	1	1	0.5		0	NA	NA		0.333	-0.083
SE	14488	ARPC2	ARPC2	chr2	+	218239390	218239484	218238663	218238850	218245419	218245546	338	115	89	1	0	0	0	56	0	1	98	49	1	1	0.994		1	1	NA		0.966	0.032
SE	14492	FRMD4B	FRMD4B	chr3	-	69249225	69249248	69224606	69224690	69250042	69250099	1	0	0	1	0	0	0	1	0	0	72	49	1	1	0.405	NA		NA	NA		1	-0.595
SE	14500	ZFAND2B	ZFAND2B	chr2	+	219207647	219207779	219207326	219207421	219207886	219208038	11	2	3	0	2	0	0	2	0	0	98	49	1	1	1		0.333	1	NA		1	-0.222
SE	14501	ZFAND2B	ZFAND2B	chr2	+	219208976	219209049	219208571	219208639	219209261	219209651	9	5	4	2	0	0	0	5	0	0	98	49	1	1	0.692		1	1	NA		1	-0.103
SE	14502	ATG9A	ATG9A	chr2	-	219227921	219228053	219227769	219227813	219228433	219228485	8	3	0	1	0	0	0	1	0	0	98	49	1	1	0.8		1	NA	NA		1	-0.1
SE	14506	GOLGB1	GOLGB1	chr3	-	121699811	121699885	121693740	121698929	121702480	121702595	12	2	0	1	0	0	0	2	0	1	98	49	1	1	0.857		1	NA	NA		0.5	0.428
SE	14507	GOLGB1	GOLGB1	chr3	-	121702480	121702595	121693740	121698929	121714860	121714976	5	8	1	1	0	0	0	5	0	0	98	49	1	1	0.714		1	1	NA		1	-0.095
SE	14508	GOLGB1	GOLGB1	chr3	-	121719645	121719753	121718387	121718501	121722261	121722378	1	0	0	3	0	2	0	0	0	2	98	49	1	1	0.143	NA		0	NA		0	0.071
SE	14509	GOLGB1	GOLGB1	chr3	-	121719645	121719768	121718387	121718501	121722261	121722378	1	0	0	3	0	2	0	0	0	2	98	49	1	1	0.143	NA		0	NA		0	0.071
SE	14512	ACSL3	ACSL3	chr2	+	222900673	222900780	222887829	222887888	222908732	222909150	3	7	0	1	0	0	0	6	0	0	98	49	1	1	0.6		1	NA	NA		1	-0.2
SE	14513	ACSL3	ACSL3	chr2	+	222921279	222921430	222919063	222919202	222922707	222922831	4	4	0	0	2	0	0	9	0	0	98	49	1	1	1		0.5	NA	NA		1	-0.25
SE	14517	CCNJL	CCNJL	chr5	-	160254128	160254335	160251651	160253798	160255548	160255708	1	0	0	14	7	4	0	0	0	4	98	49	1	1	0.034		0	0	NA		0	0.011
SE	14521	MFF	MFF	chr2	+	227328677	227328789	227325150	227325427	227330625	227330846	1	0	0	1	0	0	1	0	0	0	98	49	1	1	0.333	NA		NA		1	NA	-0.667
SE	14523	MFF	MFF	chr2	+	227330625	227330846	227325150	227325427	227332418	227332588	6	0	0	1	1	0	0	0	0	1	98	49	1	1	0.75		0	NA	NA		0	0.375
SE	14535	SP100	SP100	chr2	+	230440475	230440557	230417590	230417665	230442936	230443099	0	1	0	26	36	1	0	1	1	3	98	49	1	1	0		0.014	0		0	0.143	-0.067
SE	14536	SP100	SP100	chr2	+	230449560	230449710	230449087	230449150	230450171	230450255	86	64	10	1	0	0	7	33	0	0	98	49	1	1	0.977		1	1		1	1	-0.008
SE	14537	SP100	SP100	chr2	+	230450171	230450255	230449087	230449150	230461261	230461414	52	32	10	1	1	0	2	23	1	0	98	49	1	1	0.963		0.941	1		0.5	1	0.218
SE	14538	SP100	SP100	chr2	+	230464066	230464150	230462434	230462518	230466300	230466354	71	39	23	5	8	0	0	25	0	2	98	49	1	1	0.877		0.709	1	NA		0.862	0
SE	14539	SP100	SP100	chr2	+	230498460	230498535	230494415	230494460	230503065	230503110	42	29	5	1	0	0	0	11	0	0	98	49	1	1	0.955		1	1	NA		1	-0.015
SE	14540	SP100	SP100	chr2	+	230507992	230508031	230506302	230506445	230511124	230511166	25	15	4	1	0	0	0	4	0	0	88	49	1	1	0.933		1	1	NA		1	-0.022
SE	14546	EIF4G2	EIF4G2	chr11	-	10803479	10803590	10803210	10803294	10803898	10804050	152	46	33	0	1	0	1	22	0	0	98	49	1	1	1		0.958	1		1	1	-0.014
SE	14547	EIF4G2	EIF4G2	chr11	-	10804912	10805015	10804286	10804418	10805906	10806047	114	18	23	3	0	0	0	14	0	0	98	49	1	1	0.95		1	1	NA		1	-0.017
SE	14553	GIGYF2	GIGYF2	chr2	+	232734416	232734486	232703423	232703489	232735154	232735238	0	2	3	1	0	0	0	3	0	0	98	49	1	1	0		1	1	NA		1	-0.333
SE	14559	CLK3	CLK3	chr15	+	74627351	74627446	74624901	74625018	74627538	74627668	18	5	4	1	0	0	0	5	0	0	98	49	1	1	0.9		1	1	NA		1	-0.033
SE	14560	CLK3	CLK3	chr15	+	74627969	74628052	74627538	74627668	74628603	74628683	24	3	13	0	0	0	0	3	0	1	98	49	1	1	1		1	1	NA		0.6	0.4
SE	14562	SEC23A	SEC23A	chr14	-	39074414	39074530	39067172	39067296	39075934	39076093	18	3	2	1	0	0	2	0	0	0	98	49	1	1	0.9		1	1		1	NA	-0.033
SE	14563	DGKD	DGKD	chr2	+	233434768	233434901	233434379	233434484	233435817	233435924	21	5	8	1	0	0	0	13	0	0	98	49	1	1	0.913		1	1	NA		1	-0.029
SE	14564	DGKD	DGKD	chr2	+	233435817	233435924	233434768	233434901	233436315	233436441	12	9	12	0	0	0	0	12	0	1	98	49	1	1	1		1	1	NA		0.857	0.143
SE	14572	SERPINB1	SERPINB1	chr6	-	2839334	2839466	2838548	2838686	2840418	2840594	0	0	0	11	11	9	0	2	0	7	98	49	1	1	0		0	0	NA		0.125	-0.125
SE	14574	LRRFIP1	LRRFIP1	chr2	+	237708543	237708630	237692163	237692521	237748363	237748399	15	14	1	0	0	0	0	2	0	2	98	49	1	1	1		1	1	NA		0.333	0.667
SE	14576	LRRFIP1	LRRFIP1	chr2	+	237717761	237717809	237708543	237708630	237719522	237719567	2	2	2	1	0	0	0	1	0	0	97	49	1	1	0.503		1	1	NA		1	-0.166
SE	14579	UNC13D	UNC13D	chr17	-	75835646	75835777	75835408	75835529	75835854	75835906	47	14	10	2	0	0	4	11	0	0	98	49	1	1	0.922		1	1		1	1	-0.026
SE	14580	UNC13D	UNC13D	chr17	-	75840506	75840576	75840224	75840329	75840761	75840830	39	19	9	0	2	0	3	7	0	0	98	49	1	1	1		0.826	1		1	1	-0.058
SE	14584	HDAC4	HDAC4	chr2	-	239053459	239053601	239048167	239053136	239054748	239054833	6	1	1	1	0	0	0	6	0	0	98	49	1	1	0.75		1	1	NA		1	-0.083
SE	14586	HDAC4	HDAC4	chr2	-	239144582	239144714	239139683	239139796	239156651	239156773	12	3	3	0	0	0	0	3	0	2	98	49	1	1	1		1	1	NA		0.429	0.571
SE	14587	CAPN10	CAPN10	chr2	+	240596318	240596521	240595023	240595304	240597887	240598087	1	2	1	0	0	0	0	2	0	1	98	49	1	1	1		1	1	NA		0.5	0.5
SE	14591	RFC1	RFC1	chr4	-	39306591	39306701	39304813	39304928	39308635	39309032	8	2	0	1	0	0	0	2	0	0	98	49	1	1	0.8		1	NA	NA		1	-0.1
SE	14594	GOLPH3	GOLPH3	chr5	-	32143748	32143880	32135571	32135686	32173809	32174319	17	14	9	0	0	2	0	3	0	0	98	49	1	1	1		1	0.692	NA		1	-0.103
SE	14595	CCDC66	CCDC66	chr3	+	56559568	56559594	56558845	56558910	56563683	56564125	2	0	0	1	0	0	0	1	0	0	75	49	1	1	0.566	NA		NA	NA		1	-0.434
SE	14612	ATG4B	ATG4B	chr2	+	241655270	241655343	241654545	241654647	241659107	241659187	3	5	1	3	0	0	0	1	0	0	98	49	1	1	0.333		1	1	NA		1	-0.222
SE	14619	USP32	USP32	chr17	-	60288522	60288682	60271349	60271481	60294682	60294801	75	33	12	1	0	0	0	12	0	0	98	49	1	1	0.974		1	1	NA		1	-0.009
SE	14622	RBCK1	RBCK1	chr20	+	409880	410025	408064	408779	417525	417619	25	15	16	1	0	0	0	5	0	0	98	49	1	1	0.926		1	1	NA		1	-0.025
SE	14623	RBCK1	RBCK1	chr20	+	420870	421031	419557	419731	422126	422238	7	8	4	0	1	0	2	3	0	1	98	49	1	1	1		0.8	1		1	0.6	0.133
SE	14627	TBC1D20	TBC1D20	chr20	-	447888	448074	445049	445130	462335	462553	8	4	0	2	0	0	1	6	0	0	98	49	1	1	0.667		1	NA		1	1	-0.166
SE	14637	SNRPB	SNRPB	chr20	-	2465707	2465819	2463746	2463899	2467606	2467758	25	14	8	0	0	0	1	10	0	1	98	49	1	1	1		1	1		1	0.833	0.084
SE	14656	NUMA1	NUMA1	chr11	-	72012400	72012442	72010785	72010854	72012894	72016260	10	0	0	4	2	1	0	0	0	1	91	49	1	1	0.574		0	0	NA		0	0.191
SE	14668	CSF1R	CSF1R	chr5	-	150059669	150059860	150057503	150057592	150060861	150060972	20	1	3	1	0	0	0	7	0	0	98	49	1	1	0.909		1	1	NA		1	-0.03
SE	14673	LRRFIP2	LRRFIP2	chr3	-	37091466	37091538	37083635	37083806	37094791	37094908	4	1	1	5	0	0	0	2	0	0	98	49	1	1	0.286		1	1	NA		1	-0.238
SE	14678	PCIF1	PCIF1	chr20	+	45943339	45943423	45943096	45943244	45943665	45943765	6	4	4	0	0	0	0	6	0	1	98	49	1	1	1		1	1	NA		0.75	0.25
SE	14679	PCIF1	PCIF1	chr20	+	45943665	45943765	45943096	45943244	45944867	45945030	5	0	4	0	3	0	0	2	0	0	98	49	1	1	1		0	1	NA		1	-0.333
SE	14680	PCIF1	PCIF1	chr20	+	45945710	45945883	45944867	45945030	45946028	45946115	3	1	0	1	0	3	0	1	0	1	98	49	1	1	0.6		1	0	NA		0.333	0.2
SE	14681	TIAM1	TIAM1	chr21	-	31202907	31203012	31195223	31195305	31210044	31210215	2	0	0	1	0	0	0	4	0	0	98	49	1	1	0.5	NA		NA	NA		1	-0.5
SE	14696	NAPB	NAPB	chr20	-	23395138	23395185	23394921	23394999	23397071	23397188	0	0	0	1	0	0	0	2	0	0	96	49	1	1	0	NA		NA	NA		1	-1
SE	14701	STK10	STK10	chr5	-	172093411	172093960	172090231	172090362	172105655	172105737	42	3	6	2	0	0	1	8	0	0	98	49	1	1	0.913		1	1		1	1	-0.029
SE	14702	STK10	STK10	chr5	-	172096425	172096560	172093411	172093960	172105655	172105737	59	25	13	1	0	0	2	8	0	0	98	49	1	1	0.967		1	1		1	1	-0.011
SE	14703	STK10	STK10	chr5	-	172107779	172107852	172106619	172106814	172117480	172117630	107	56	14	0	1	0	6	14	0	0	98	49	1	1	1		0.966	1		1	1	-0.011
SE	14710	PLEKHG3	PLEKHG3	chr14	+	64728995	64729093	64727592	64727982	64730641	64730688	21	11	5	2	0	0	1	2	0	0	98	49	1	1	0.84		1	1		1	1	-0.053
SE	14711	PLEKHG3	PLEKHG3	chr14	+	64730242	64730312	64728995	64729093	64730641	64730688	37	27	18	5	6	0	0	4	0	0	98	49	1	1	0.787		0.692	1	NA		1	-0.174
SE	14712	PLEKHG3	PLEKHG3	chr14	+	64731360	64731543	64731037	64731169	64731713	64731806	35	20	25	2	0	0	0	6	0	0	98	49	1	1	0.897		1	1	NA		1	-0.034
SE	14727	HM13	HM13	chr20	+	31568077	31568224	31566209	31566295	31569119	31569567	0	3	0	4	0	0	0	0	0	1	98	49	1	1	0		1	NA	NA		0	0.5
SE	14734	HCK	HCK	chr20	+	32069687	32069751	32052187	32052486	32071661	32071782	2	0	0	135	59	44	0	0	9	33	98	49	1	1	0.007		0	0		0	0	0.002
SE	14735	HCK	HCK	chr20	+	32073318	32073361	32071661	32071782	32073715	32073818	146	80	82	3	3	0	0	24	0	0	92	49	1	1	0.963		0.934	1	NA		1	-0.034
SE	14736	HCK	HCK	chr20	+	32073318	32073361	32071661	32071782	32073718	32073818	114	49	55	2	0	0	0	19	0	0	92	49	1	1	0.968		1	1	NA		1	-0.011
SE	14737	HCK	HCK	chr20	+	32079773	32079877	32074622	32074721	32083893	32084043	186	83	65	1	0	0	0	38	0	0	98	49	1	1	0.989		1	1	NA		1	-0.004
SE	14739	TM9SF4	TM9SF4	chr20	+	32157793	32157969	32155102	32155186	32158450	32158514	6	0	0	1	0	0	1	3	0	0	98	49	1	1	0.75	NA		NA		1	1	-0.25
SE	14740	TM9SF4	TM9SF4	chr20	+	32159991	32160111	32158450	32158514	32161275	32161365	9	1	0	1	0	0	2	4	1	0	98	49	1	1	1		1	NA		0.5	1	0.25
SE	14741	GPR132	GPR132	chr14	-	105055386	105056166	105049388	105052102	105057166	105057280	3	0	0	5	2	0	0	0	0	2	98	49	1	1	0.231		0	NA	NA		0	0.116
SE	14744	IDUA	IDUA	chr4	+	1000881	1000989	1000611	1000697	1001467	1001563	1	0	0	0	0	0	0	0	0	2	98	49	1	1	1	NA		NA	NA		0	1
SE	14750	ASXL1	ASXL1	chr20	+	32429900	32430053	32429337	32429431	32431320	32431484	6	2	0	1	3	0	0	1	0	0	98	49	1	1	0.75		0.25	NA	NA		1	-0.5
SE	14753	RALY	RALY	chr20	+	34031521	34031604	33993651	33994131	34072065	34072330	9	10	6	1	0	0	3	10	0	0	98	49	1	1	0.818		1	1		1	1	-0.061
SE	14754	RALY	RALY	chr20	+	34073818	34073866	34073562	34073635	34075873	34076040	11	3	7	1	0	0	0	8	0	0	97	49	1	1	0.847		1	1	NA		1	-0.051
SE	14761	MAP1LC3A	MAP1LC3A	chr20	+	34559207	34559263	34558696	34558908	34559346	34559453	4	2	2	1	0	0	0	1	0	0	98	49	1	1	0.667		1	1	NA		1	-0.111
SE	14770	CPNE1	CPNE1	chr20	-	35630740	35630795	35630438	35630490	35630900	35631034	31	7	24	1	0	2	0	4	0	0	98	49	1	1	0.939		1	0.857	NA		1	-0.068
SE	14771	CPNE1	CPNE1	chr20	-	35632162	35632234	35631944	35632025	35632310	35632385	16	11	4	5	1	2	4	0	0	0	98	49	1	1	0.615		0.846	0.5		1	NA	-0.346
SE	14772	CPNE1	CPNE1	chr20	-	35655201	35655344	35632794	35632923	35658929	35658982	12	5	0	0	3	0	0	0	0	2	98	49	1	1	1		0.455	NA	NA		0	0.728
SE	14773	CPNE1	CPNE1	chr20	-	35658929	35658982	35632794	35632923	35664759	35664937	3	4	0	3	1	0	0	2	0	0	98	49	1	1	0.333		0.667	NA	NA		1	-0.5
SE	14774	CPNE1	CPNE1	chr20	-	35658929	35659014	35632794	35632923	35664759	35664956	3	5	1	3	1	0	0	2	0	0	98	49	1	1	0.333		0.714	1	NA		1	-0.318
SE	14782	PHF20	PHF20	chr20	+	35863012	35863400	35858301	35858381	35869437	35869551	19	8	4	3	0	0	0	2	0	0	98	49	1	1	0.76		1	1	NA		1	-0.08
SE	14783	PHF20	PHF20	chr20	+	35870954	35871134	35869437	35869551	35871649	35871829	14	2	4	1	0	0	0	1	0	0	98	49	1	1	0.875		1	1	NA		1	-0.042
SE	14784	PHF20	PHF20	chr20	+	35927779	35927879	35917483	35917662	35931248	35931444	14	4	3	0	1	0	0	1	0	0	98	49	1	1	1		0.667	1	NA		1	-0.111
SE	14785	DHX36	DHX36	chr3	-	154280762	154280862	154280578	154280669	154283187	154283271	7	4	0	2	0	0	0	3	0	0	98	49	1	1	0.636		1	NA	NA		1	-0.182
SE	14786	DHX36	DHX36	chr3	-	154306215	154306295	154305093	154305168	154309652	154309823	5	2	0	0	0	0	0	1	0	1	98	49	1	1	1		1	NA	NA		0.333	0.667
SE	14787	MYL9	MYL9	chr20	+	36548031	36548193	36544858	36545068	36549076	36549823	7	0	2	1	0	0	4	0	0	0	98	49	1	1	0.778	NA		1		1	NA	-0.111
SE	14789	PHF1	PHF1	chr6	+	33412507	33412589	33412247	33412422	33413195	33413296	2	1	0	2	0	0	2	2	0	0	98	49	1	1	0.333		1	NA		1	1	-0.334
SE	14790	PHF1	PHF1	chr6	+	33412697	33412793	33412507	33412589	33413195	33413296	10	8	1	1	0	0	0	4	0	0	98	49	1	1	0.833		1	1	NA		1	-0.056
SE	14795	C20orf24	C20orf24	chr20	+	36607889	36608000	36607714	36607818	36609600	36609730	14	8	3	9	11	26	0	3	0	4	98	49	1	1	0.437		0.267	0.055	NA		0.273	-0.02
SE	14796	C20orf24	C20orf24	chr20	+	36607889	36608000	36607714	36607818	36612009	36612557	3	3	1	1	0	1	0	1	0	0	98	49	1	1	0.6		1	0.333	NA		1	-0.356
SE	14797	C20orf24	C20orf24	chr20	+	36609600	36609730	36607889	36608000	36612009	36612557	32	36	30	1	1	0	0	6	0	0	98	49	1	1	0.941		0.947	1	NA		1	-0.037
SE	14798	C20orf24	C20orf24	chr20	+	36609600	36609758	36607714	36607818	36612009	36612557	9	11	26	1	0	1	0	4	0	0	98	49	1	1	0.818		1	0.929	NA		1	-0.084
SE	14806	SAMHD1	SAMHD1	chr20	-	36898439	36898544	36897821	36897959	36904156	36904249	35	12	6	0	1	0	0	25	0	1	98	49	1	1	1		0.857	1	NA		0.926	0.026
SE	14807	SAMHD1	SAMHD1	chr20	-	36935028	36935189	36930759	36930875	36941038	36941111	27	1	2	0	0	0	0	21	0	1	98	49	1	1	1		1	1	NA		0.913	0.087
SE	14809	HNRNPH1	HNRNPH1	chr5	-	179615545	179615595	179614177	179614959	179616125	179616218	83	9	12	4	2	0	0	4	0	0	98	49	1	1	0.912		0.692	1	NA		1	-0.132
SE	14810	HNRNPH1	HNRNPH1	chr5	-	179619268	179619407	179618144	179618323	179620891	179621035	23	5	0	12	4	0	0	3	0	0	98	49	1	1	0.489		0.385	NA	NA		1	-0.563
SE	14811	HNRNPH1	HNRNPH1	chr5	-	179623036	179623164	179621241	179621397	179623594	179623710	43	5	1	0	2	0	0	3	0	0	98	49	1	1	1		0.556	1	NA		1	-0.148
SE	14819	RALGAPB	RALGAPB	chr20	+	38548688	38548795	38546242	38546430	38551070	38551223	18	11	3	0	1	0	0	4	0	3	98	49	1	1	1		0.846	1	NA		0.4	0.549
SE	14820	RALGAPB	RALGAPB	chr20	+	38565355	38565478	38562531	38562697	38567095	38567232	8	4	6	0	0	0	0	0	0	4	98	49	1	1	1		1	1	NA		0	1
SE	14821	RALGAPB	RALGAPB	chr20	+	38565358	38565478	38562531	38562697	38567095	38567232	7	2	5	0	0	0	0	2	0	4	98	49	1	1	1		1	1	NA		0.2	0.8
SE	14827	TOP1	TOP1	chr20	+	41076170	41076294	41061393	41061490	41077581	41077637	9	6	0	0	1	0	1	3	0	0	98	49	1	1	1		0.75	NA		1	1	-0.125

SE	14828 MBD5	MBD5	chr2	+	148233244	148233395	148178699	148178793	148342213	148342336	13	1	0	1	0	0	0	1	0	0	98	49	1	1	0.867	1	NA	NA	1	-0.067
SE	14838 SERINC3	SERINC3	chr20	-	44511288	44511368	44509890	44510028	44512800	44512994	46	6	28	2	0	0	0	13	0	0	98	49	1	1	0.92	1	1	NA	1	-0.027
SE	14842 STK4	STK4	chr20	+	45001166	45001353	45000391	45000520	45024972	45025130	116	59	21	0	1	0	0	11	0	0	98	49	1	1	1	0.967	1	NA	1	-0.011
SE	14874 ZMYND8	ZMYND8	chr20	-	47212641	47212725	47209213	47210897	47220257	47220324	2	0	0	3	0	0	0	0	0	1	98	49	1	1	0.25	NA	NA	NA	0	0.25
SE	14876 ZMYND8	ZMYND8	chr20	-	47236325	47236516	47229725	47229806	47246007	47246517	10	3	4	1	0	0	0	3	0	0	98	49	1	1	0.833	1	1	NA	1	-0.056
SE	14880 ZMYND8	ZMYND8	chr20	-	47276313	47276795	47262287	47262428	47282101	47282217	18	11	1	0	1	0	0	1	0	0	98	49	1	1	1	0.846	1	NA	1	-0.051
SE	14881 ZMYND8	ZMYND8	chr20	-	47282101	47282217	47262287	47262428	47283570	47283648	4	5	2	0	1	0	0	3	0	0	98	49	1	1	1	0.714	1	NA	1	-0.095
SE	14885 PREX1	PREX1	chr20	-	48627547	48627615	48624254	48625927	48627860	48627963	156	84	50	2	0	0	6	30	0	0	98	49	1	1	0.975	1	1	1	1	-0.008
SE	14890 PREX1	PREX1	chr20	-	48676192	48676268	48666282	48666355	48679359	48679409	252	177	111	1	0	0	11	96	0	0	98	49	1	1	0.992	1	1	1	1	-0.003
SE	14891 PREX1	PREX1	chr20	-	48679359	48679409	48676192	48676268	48679650	48679754	183	119	92	1	0	0	9	74	0	0	98	49	1	1	0.989	1	1	1	1	-0.004
SE	14892 PREX1	PREX1	chr20	-	48690946	48691096	48688656	48688804	48692671	48692790	182	122	60	0	1	0	5	38	0	0	98	49	1	1	1	0.984	1	1	1	-0.005
SE	14893 PREX1	PREX1	chr20	-	48726289	48726391	48708259	48708421	48734545	48734650	330	204	94	0	1	0	23	82	0	0	98	49	1	1	1	0.99	1	1	1	-0.003
SE	14894 PREX1	PREX1	chr20	-	48734545	48734650	48708259	48708421	48745024	48745147	112	69	46	0	1	0	7	26	0	0	98	49	1	1	1	0.972	1	1	1	-0.009
SE	14895 PREX1	PREX1	chr20	-	48747808	48747880	48745024	48745147	48827641	48827883	97	107	30	1	0	1	20	45	0	1	98	49	1	1	0.98	1	0.937	1	0.957	-0.006
SE	14897 DOPEY1	DOPEY1	chr6	+	83155950	83156103	83154179	83154241	83157181	83157318	1	0	0	1	0	0	0	1	0	0	98	49	1	1	0.333	NA	NA	NA	1	-0.667
SE	14901 CSE1L	CSE1L	chr20	+	49085282	49085386	49084025	49084162	49088008	49088106	5	2	0	2	0	0	0	2	0	0	98	49	1	1	0.556	1	NA	NA	1	-0.222
SE	14905 TMEM8A	TMEM8A	chr16	-	375132	375256	374755	374892	375344	375435	6	9	8	0	0	1	1	5	0	0	98	49	1	1	1	1	0.8	1	1	-0.067
SE	14906 TMEM8A	TMEM8A	chr16	-	376543	376812	376135	376455	377036	377164	16	9	5	0	1	0	2	7	0	0	98	49	1	1	1	0.818	1	1	1	-0.061
SE	14907 DCP1A	DCP1A	chr3	-	53304176	53304290	53292068	53292827	53312240	53312379	2	1	0	1	0	0	0	2	0	0	98	49	1	1	0.5	1	NA	NA	1	-0.25
SE	14919 ZNF217	ZNF217	chr20	-	53571720	53571853	53567070	53569264	53575726	53577280	19	15	4	3	0	1	0	4	0	1	98	49	1	1	0.76	1	0.667	NA	0.667	0.142
SE	14921 UBA3	UBA3	chr3	-	69057255	69057309	69056778	69056815	69061813	69061927	9	2	8	2	0	0	0	7	0	0	98	49	1	1	0.692	1	1	NA	1	-0.103
SE	14922 UBA3	UBA3	chr3	-	69071534	69071617	69067927	69068008	69075429	69075510	16	6	14	4	0	0	0	3	0	0	98	49	1	1	0.667	1	1	NA	1	-0.111
SE	14926 ATP6V1D	ATP6V1D	chr14	-	67349036	67349104	67347408	67347453	67350610	67350690	21	18	1	2	1	0	0	2	0	0	98	49	1	1	0.84	0.9	1	NA	1	-0.087
SE	14930 SYCP2	SYCP2	chr20	-	59877384	59877555	59875270	59875469	59878007	59878045	2	0	0	1	0	0	0	1	0	0	98	49	1	1	0.5	NA	NA	NA	1	-0.5
SE	14934 OSBPL2	OSBPL2	chr20	+	62260016	62260125	62256056	62256221	62263615	62263691	27	6	5	2	1	0	0	3	0	0	98	49	1	1	0.871	0.75	1	NA	1	-0.126
SE	14935 OSBPL2	OSBPL2	chr20	+	62264628	62264718	62263615	62263691	62272124	62272259	13	5	0	17	28	8	0	2	0	3	98	49	1	1	0.277	0.082	0	NA	0.25	-0.13
SE	14936 OSBPL2	OSBPL2	chr20	+	62273308	62273406	62272124	62272259	62279156	62279339	13	12	6	0	2	0	0	1	0	0	98	49	1	1	1	0.75	1	NA	1	-0.083
SE	14937 OSBPL2	OSBPL2	chr20	+	62289206	62289330	62286582	62286711	62291702	62291793	24	19	7	1	0	2	0	7	0	0	98	49	1	1	0.923	1	0.636	NA	1	-0.147
SE	14938 ADRM1	ADRM1	chr20	+	62304460	62304577	62303567	62303781	62306196	62306320	6	3	0	3	0	0	0	5	0	0	98	49	1	1	0.5	1	NA	NA	1	-0.25
SE	14939 ADRM1	ADRM1	chr20	+	62308367	62308470	62308020	62308178	62308654	62308862	2	2	2	0	2	1	0	4	0	1	98	49	1	1	1	0.333	0.5	NA	0.667	-0.056
SE	14940 ERCC6L2	ERCC6L2	chr9	+	95907077	95907271	95897848	95897971	95915667	95915829	8	1	0	4	0	0	0	0	0	1	98	49	1	1	0.5	1	NA	NA	0	0.75
SE	14942 EIF3K	EIF3K	chr19	+	38620336	38620435	38619073	38619327	38624076	38624197	31	7	4	0	0	0	0	8	0	1	98	49	1	1	1	1	1	NA	0.8	0.2
SE	14943 EIF3K	EIF3K	chr19	+	38632600	38632678	38632429	38632496	38634992	38635118	30	9	10	0	0	0	0	5	0	1	98	49	1	1	1	1	1	NA	0.714	0.286
SE	14951 OGFR	OGFR	chr20	+	62809584	62809663	62808246	62808325	62810498	62810565	31	15	8	0	2	0	2	5	0	0	98	49	1	1	1	0.789	1	1	1	-0.07
SE	14952 CGGBP1	CGGBP1	chr3	-	88057190	88057292	88051949	88055999	88058018	88058223	2	6	0	15	1	4	0	1	0	1	98	49	1	1	0.062	0.75	0	NA	0.333	-0.062
SE	14954 ARFGAP1	ARFGAP1	chr20	+	63277204	63277305	63276479	63276651	63278116	63278203	5	3	4	1	0	0	0	1	0	0	98	49	1	1	0.714	1	1	NA	1	-0.095
SE	14955 ARFGAP1	ARFGAP1	chr20	+	63282818	63282851	63281290	63281347	63284865	63284922	3	1	1	2	0	0	0	1	0	0	82	49	1	1	0.473	1	1	NA	1	-0.176
SE	14964 HELZ2	HELZ2	chr20	-	63561600	63561745	63561349	63561466	63561822	63561984	22	11	8	1	0	0	2	25	0	0	98	49	1	1	0.917	1	1	1	1	-0.028
SE	14965 HELZ2	HELZ2	chr20	-	63562071	63562203	63561822	63561984	63562287	63562382	8	12	1	0	0	3	0	11	0	0	98	49	1	1	1	1	0.143	NA	1	-0.286
SE	14971 TPD52L2	TPD52L2	chr20	+	63875815	63875875	63873667	63873816	63882718	63882820	39	21	14	2	0	1	0	17	0	0	98	49	1	1	0.907	1	0.875	NA	1	-0.073
SE	14974 TPD52L2	TPD52L2	chr20	+	63887563	63887605	63882718	63882820	63889189	63889238	0	0	0	13	7	4	0	1	0	8	91	49	1	1	0	0	0	NA	0.063	-0.063
SE	14977 RGS19	RGS19	chr20	-	64076856	64076954	64076524	64076646	64079293	64079492	18	4	3	5	1	0	0	7	0	2	98	49	1	1	0.643	0.667	1	NA	0.636	0.134
SE	14979 OPRL1	OPRL1	chr20	+	64092687	64092953	64080117	64080352	64097801	64098157	0	1	0	1	0	0	0	1	0	0	98	49	1	1	0	1	NA	NA	1	-0.5
SE	14980 PCMTD2	PCMTD2	chr20	+	64265257	64265429	64264428	64264531	64267886	64268010	10	1	6	0	1	0	0	2	0	0	98	49	1	1	1	0.333	1	NA	1	-0.222
SE	14988 RPS3	RPS3	chr11	+	75404019	75404207	75402303	75402446	75404671	75404868	77	16	16	1	0	0	1	7	0	0	98	49	1	1	0.975	1	1	1	1	-0.008
SE	14997 MGAM2	MGAM2	chr7	+	142154728	142154845	142154017	142154189	142157936	142158091	1	2	1	1	0	0	0	1	0	0	98	49	1	1	0.333	1	1	NA	1	-0.222
SE	14998 MGAM2	MGAM2	chr7	+	142172097	142172194	142171271	142171440	142172651	142172764	3	2	0	1	0	0	0	2	0	0	98	49	1	1	0.6	1	NA	NA	1	-0.2
SE	15008 UBP1	UBP1	chr3	-	33408689	33408797	33402800	33402904	33409235	33409346	6	0	0	3	1	1	0	2	0	0	98	49	1	1	0.5	0	0	NA	1	-0.833
SE	15011 APP	APP	chr21	-	25905023	25905077	25897572	25897673	25911740	25911962	11	0	1	2	1	0	0	6	0	1	98	49	1	1	0.733	0	1	NA	0.75	-0.172
SE	15012 APP	APP	chr21	-	25997359	25997416	25982343	25982477	26000014	26000182	1	0	0	2	0	0	0	1	0	3	98	49	1	1	0.2	NA	NA	NA	0.143	0.057
SE	15015 N6AMT1	N6AMT1	chr21	-	28879869	28879953	28878191	28878333	28882159	28882250	0	0	0	0	2	0	1	0	0	0	98	49	1	1	NA	0	NA	1	NA	-1
SE	15016 FBRS	FBRS	chr16	+	30661179	30661215	30660262	30660442	30662419	30662468	6	5	0	0	1	0	0	4	0	0	85	49	1	1	1	0.742	NA	NA	1	-0.129
SE	15017 FBRS	FBRS	chr16	+	30661303	30661333	30661179	30661215	30662419	30662468	10	7	0	0	1	0	0	1	0	1	79	49	1	1	1	0.813	NA	NA	0.383	0.523
SE	15018 FBRS	FBRS	chr16	+	30666511	30666541	30665637	30665706	30666918	30666990	14	10	0	0	1	0	0	4	0	0	79	49	1	1	1	0.861	NA	1	NA	-0.07
SE	15019 FBRS	FBRS	chr16	+	30667319	30667437	30666918	30666990	30667541	30667622	30	14	11	1	0	0	5	5	0	0	98	49	1	1	0.937	1	1	1	1	-0.021
SE	15026 BACH1	BACH1	chr21	+	29305203	29305322	29299415	29299598	29321220	29321514	14	3	0	6	5	0	0	3	0	2	98	49	1	1	0.538	0.231	NA	NA	0.429	-0.044
SE	15027 BACH1	BACH1	chr21	+	29351633	29351813	29329486	29329693	29361551	29361896	14	3	0	1	0	0	0	3	0	0	98	49	1	1	0.875	1	NA	NA	1	-0.062
SE	15029 BACH1	BACH1	chr21	+	29361030	29361243	29352578	29352663	29361551	29361896	0	1	0	2	0	0	0	2	0	0	98	49	1	1	0	1	NA	NA	1	-0.5
SE	15030 ATP5A1	ATP5A1	chr18	-	46087340	46087492	46087007	46087232	46088108	46088257	14	3	3	1	0	0	0	16	0	0	98	49	1	1	0.875	1	1	NA	1	-0.042
SE	15038 HERC3	HERC3	chr4	+	88605794	88606049	88595556	88595614	88649839	88649999	76	39	3	4	0	0	0	14	0	0	98	49	1	1	0.905	1	1	NA	1	-0.032
SE	15039 HERC3	HERC3	chr4	+	88667891	88668081	88667376	88667488	88669859	88670023	91	34	3	3	3	0	0	20	0	0	98	49	1	1	0.938	0.85	1	NA	1	-0.071
SE	15040 HERC3	HERC3	chr4	+	88676217	88676241	88670138	88670252	88676333	88676423	26	21	4	2	2	0	0	6	0	3	73	49	1	1	0.897	0.876	1	NA	0.573	0.351
SE	15041 HERC3	HERC3	chr4	+	88677963	88678134	88676333	88676423	88680092	88680236	35	13	2	2	0	0	0	10	0	0	98	49	1	1	0.897	1	1	NA	1	-0.034
SE	15042 HERC3	HERC3	chr4	+	88681158	88681325	88680092	88680236	88686735	88686802	75	16	16	4	9	0	0	12	0	2	98	49	1	1	0.904	0.471	1	NA	0.75	0.042
SE	15048 IFNAR2	IFNAR2	chr21	+	33248708	33248854	33246717	33246890	33252661	33252830	56	12	3	0	0	0	1	7	0	1	98	49	1	1	1	1	1	1	0.778	0.111
SE	15049 IFNAR2	IFNAR2	chr21	+	33260596	33260727	33252661	33252830	33262560	33262757	10	12	0	0	5	0	0	7	0	0	98	49	1	1	1	0.545	NA	NA	1	-0.228
SE	15050 IFNAR2	IFNAR2	chr21	+	33260596	33260727	33252661	33252830	33262792	33264513	14	13	1	1	0	0	0	7	0	0	98	49	1	1	0.875	1	1	NA	1	-0.042
SE	15053 IFNGR2	IFNGR2	chr21	+	33432176	33432336	33426883	33427032	33432713	33432871	80	42	45	0	0	0	0	16	0	1	98	49	1	1	1	1	1	NA	0.889	0.111
SE	15059 RER1	RER1	chr1	+	2399414	2399514	2397115	2397220	2400856	2400935	25	16	13	1	0	0	0	3	0	3	98	49	1	1	0.926	1	1	NA	0.333	0.642
SE	15062 MORC3	MORC3	chr21	+	36356619	36356724	36349310	36349408	36359954	36360077	18	6	11	1	0	0	0	2	0	0	98	49	1	1	0.9	1	1	NA	1	-0.033
SE	15070 ETS2	ETS2	chr21	+	38818424	38818646	38817007	38817091	38819502	38819766	21	8	2	1	0	0	3	18	0	0	98	49	1	1	0.913	1	1	1	1	-0.029
SE	15071 ETS2	ETS2	chr21	+	38821585	38821704	38819502	38819766	38822673	38824954	37	4	16	0	0	0	0	8	0	3	98	49	1	1	1	1	1	NA	0.571	0.429
SE	15072 HMHA1	HMHA1	chr19	+	1068413	1068744	1067165	1067495	1073148	1073292	65	43	18	1	0	0	8	14	0	1	98	49	1	1	0.97	1	1	1	0.875	0.052
SE	15073 HMHA1	HMHA1	chr19	+	1073673	1073746	1073505	1073590	1073947	1074014	99	51	38	1	0	3	0	23	0	1	98	49	1	1	0.98	1	0.864	NA	0.92	0.028
SE	15074 HMHA1	HMHA1	chr19	+	1083142	1083353	1082839	1083066	1084237	1084346	152	62	42	0	1	0	10	40	0	0	98	49	1	1	1	0.969	1	1	1	-0.01
SE	15075 BRWD1	BRWD1	chr21	-	39218151	39218272	39215236	39215362	39218504	39218660	17	3	2	0	1	0	0	2	0	0	98	49	1	1	1	0.6	1	NA	1	-0.133
SE	15090 C2CD2	C2CD2	chr21	-	41921971	41922085	41918855	41918960	41942146	41942245	0	1	0	0	0	0	0	0	0	3	98	49	1	1	NA	1	NA	NA	0	1
SE	15094 TRAPPC10	TRAPPC10	chr21	+	44037791	44037927	44032090	44032172	44052279	44052476	14	13	12	0	3	0	0	5	0	0	98	49	1	1	1	0.684	1	NA	1	-0.105
SE	15095 TRAPPC10	TRAPPC10	chr21	+	44074323	44074470	44063537	44063785	44075038	44075153	16	12	2	0	2	0	0	4	0	0	98	49	1	1	1	0.75	1	NA	1	-0.083
SE	15118 ITGB2	ITGB2	chr21	-	44889275	44889495	44888692	44888895	44889977	44890222	214	174	89	0	3	0	4	78	0	0	98	49	1	1	1	0.967	1	1	1	-0.011
SE	15123 POFUT2	POFUT2	chr21	-	45266021	45266227	45263927	45265635	45267589	45267713	0	0	1	5	3	1	0	0	1	0	98	49	1	1	0	0	0.333	0	NA	0.111
SE	15124 POFUT2	POFUT2	chr21	-	45267589	45267713	45263927	45265635	45269838	45270019	10	4	1	1	0	0	1	0	0	0	98	49	1	1	0.833	1	1	1	NA	-0.056
SE	15125 POFUT2	POFUT2	chr21	-	45283382	45283527	45282348	45282459	45285677	45285928	2	5	0	1	0	0	0	1	0	0	98	49	1	1	0.5	1	NA	NA	1	-0.25
SE	15132 DIP2A	DIP2A	chr21	+	46532126	46532237	46529091	46529183	46533523	46533647	5	9	0	1	0	0	0	5	0	0	98	49	1	1	0.714	1	NA	NA	1	-0.143
SE	15133 PRMT2	PRMT2	chr21	+	46636438	46636547	46635594	46635763	46636895	46636990	4	3	0	0	2	1	0	1	0	0	98	49	1	1	1	0.429	0	NA	1	-0.524
SE	15134 PRMT2	PRMT2	chr21	+	46658744	46658920	46649574	46649739	46664294	46665124	9	2	5	0	0	0	0	2	0	1	98	49	1	1	1	1	1	NA	0.5	0.5
SE	15135 PRMT2	PRMT2	chr21	+	46661799	46661936	46649574	46649739	46663382	46663554	9	4	3	1	5	0	0	3	0	0	98	49	1	1	0.818	0.286	1	NA	1	-0.299
SE	15136 PRMT2	PRMT2	chr21	+	46663382	46663554	46649574	46649739	46664294	46665124	4	9	2	0	0	0	0	1	0	1	98	49	1	1	1	1	1	NA	0.333	0.667
SE	15137 BID	BID	chr22	-	17743802	17744013	17739348	17739488	17750104	17750174	28	26	19	1	0	0	4	10	0	0	98	49	1	1	0.933	1	1	1	1	-0.022
SE	15145 PARP4	PARP4	chr13	-	24459971	24460136	24459263	24459310	24469023	24469110	27	18	9	0	1	0	1	15	0	0	98	49	1	1	1	0.9	1	1	1	-0.033
SE	15154 TANGO2	TANGO2	chr22	+	20053436	20053551	20052464	20052584	20055942	20056013	5	10	8	2	3	1	0	4	0	0	98	49	1	1	0.556	0.625	0.8	NA	1	-0.34
SE	15157 TANGO2	TANGO2	chr22	+	20055942	20056013	20052464	20052584	20063337	20063442	2	3	1	2	0	0	0	1	0	0	98	49	1	1	0.333	1	1	NA	1	-0.222
SE	15158 TANGO2	TANGO2	chr22	+	20055942	20056013	20053436	20053551	20063337	20063442	4	6	1	0	2	0	0	5	0	0	98	49	1	1	1	0.6	1	NA	1	-0.133
SE	15159 TANGO2	TANGO2	chr22	+	20061529	20061681	20055942	20056013	20063337	20063442	2	1	2	0	0	0	0	1	0	1	98	49	1	1	1	1	1	NA	0	1
SE	15160 TANGO2	TANGO2	chr22	+	20061529	20061683	20052464	20052584	20063337	20063442	0	3	2	2	0	0	0	2	0	0	98	49	1	1	0	1	1	NA	1	-0.333
SE	15161 TANGO2	TANGO2	chr22	+	20061529	20061683	20055942	20056013	20063337	20063442	1	4	4	0	0	0	0	2	0	1	98	49	1	1	1	1	1	NA	0.5	0.5
SE	15162 TANGO2	TANGO2	chr22	+	20063337	20063442	20055942	20056013	20064541	20065926	5	7	5	0	1	0	0	4	0	0	98	49	1	1	1	0.778	1	NA	1	-0.074
SE	15165 LZTR1	LZTR1	chr22	+	20989624	20989682	20988788	20988872	20990385	20990525	3	0	0	0	3	0	0	4	0	0	98	49	1	1	1	0	NA	NA	1	-0.5
SE	15172 SHKBP1	SHKBP1	chr19	+	40583397	40583485	40582350	40582466	40583600	40583717	151	50	36	2	1	0	1	9	0	0	98	49	1	1	0.974	0.962	1	1	1	-0.021
SE	15174 SHKBP1	SHKBP1	chr19	+	40590243	40590422	40589081	40589178	40590729	40590853	51	5	15	2	0	0	0	6	0	0	98	49	1	1	0.927	1	1	NA	1	-0.024
SE	15181 NME6	NME6	chr3	-	48296118	48296158	48295074	48295235	48296726	48296829	2	1	0	1	0	0	0	6	0	0	89	49	1	1	0.524	1	NA	NA	1	-0.238
SE	15188 GUSBP11	GUSBP11	chr22	-	23700725	23700845	23694118	23695517	23705428	23705541	5	6	2	5	3	0	0	6	0	0	98	49	1	1	0.333	0.5	1	NA	1	-0.389
SE	15189 GUSBP11	GUSBP11	chr22	-	23714186	23714342	23709785	23709867	23715008	23715194	46	24	3	0	2	0	0	4	0	0	98	49	1	1	1	0.857	1	NA	1	-0.048
SE	15199 CABIN1	CABIN1	chr22	+	24168446	24168521	24166638	24167313	24171712	24171995	14	15	1	1	0	0	0	4	0	0	98	49	1	1	0.875	1	1	NA	1	-0.042
SE	15207 ADORA2A	ADORA2A	chr22	+	24433130	24433736	24427561	24427746	24440582	24442361	5	0	1	0	1	0	1	1	0	0	98	49	1	1	1	0	1	1	1	-0.333
SE	15212 GUCD1	GUCD1	chr22	-	24547907	24548073	24546913	24547005	24548916	24549001	13	22	4	1	0	0	0	1	0	0	98	49	1	1	0.867	1	1	NA	1	-0.044
SE	15213 GUCD1	GUCD1	chr22	-	24547907	24548073	24546913	24547005	24554948	24555307	13	7	0	0	0	0	0	1	0	1	98	49	1	1	1	1	NA	NA	0.333	0.667
SE	15214 GUCD1	GUCD1	chr22	-	24548916	24549001	24546913	24547005	24554948	24555307	5	4	0	0	0	0	2	0	0	0	98	49	1	1	1	1	NA	1	0	0.5
SE	15215 GUCD1	GUCD1	chr22	-	24548916	24549001	24547907	24548073	24554948	24555307	7	19	4	3	0	0	2	0	0	0	98	49	1	1	0.538	1	1	1	NA	-0.154
SE	15224 CRYBB2P1	CRYBB2P1	chr22	+	25453315	25453434	25448086	25448284	25455711	25455840	3	0	3	1	0	0	0	1	0	0	98	49	1	1	0.6	NA	1	NA	1	-0.2
SE	15225 ADAM19	ADAM19	chr5	-	157502802	157502980	157499572	157499662	157505668	157505808	11	0	2	0	1	3	0	1	0	0	98	49	1	1	1	0	0.25	NA	1	-0.583
SE	15226 DIAPH1	DIAPH1	chr5	-	141529602	141529697	141529171	141529273	141534334	141534433	20	17	0	2	2	0	0	8	0	0	98	49	1	1	0.833	0.81	NA	NA	1	-0.178
SE	15227 DIAPH1	DIAPH1	chr5	-	141583484	141583615	141583205	141583292	141584123	141584225	41	10	5	2	0	0	3	26	0	0	98	49	1	1	0.911	1	1	1	1	-0.03
SE	15228 DIAPH1	DIAPH1	chr5	-	141588223	141588250	141587041	141587197	141618797	141619055	0	1	0	16	10	3	0	0	0	7	76	49	1	1	0	0.061	0	NA	0	0.02
SE	15229 XBP1	XBP1	chr22	-	28796046	28796192	28794559	28795732	28797076	28797205	15	0	0	2	0	0	0	6	1	0	98	49	1	1	1	NA	1	0	1	0.5
SE	15230 AP1B1	AP1B1	chr22	-	29329711	29329720	29327679	29328895	29330377	29330532	1	0	0	2	0	0	0	1	0	0	58	49	1	1	0.297	NA	NA	NA	1	-0.703
SE	15231 AP1B1	AP1B1	chr22	-	29339753	29339774	29338989	29339133	29340655	29340857	0	0	0	1	0	0	0	3	0	3	70	49	1	1	0	NA	NA	NA	0.412	-0.412
SE	15232 AP1B1	AP1B1	chr22	-	29356425	29356616	29354649	29354871	29358725	29358971	2	1	0	1	0	0	0	3	0	0	98	49	1	1	0.5	1	NA	NA	1	-0.25
SE	15238 THOC5	THOC5	chr22	-	29550727	29550843	29549051	29549158	29553670	29553747	5	1	2	2	0	0	0	1	0	0	98	49	1	1	0.556	1	1	NA	1	-0.148
SE	15239 GIGYF1	GIGYF1	chr7	-	100683549	100683632	100683303	100683444	100683817	100683918	21	5	1	0	0	0	0	0	1	0	98	49	1	1	1	1	1	0	NA	1
SE	15241 ZMAT5	ZMAT5	chr22	-	29754748	29754863	29748417	29748571	29766871	29766980	2	0	0	0	0	1	0	1	0	0	98	49	1	1	1	NA	0	NA	1	-0.5
SE	15246 DUSP18	DUSP18	chr22	-	30664954	30665121	30661457	30664080	30667461	30667890	0	0	0	4	0	0	0	1	0	2	98	49	1	1	0	NA	NA	NA	0.2	-0.2
SE	15266 SIGLEC10	SIGLEC10	chr19	-	51414823	51415108	51414421	51414515	51415180	51415438	13	19	1	10	7	0	0	4	0	0	98	49	1	1	0.394	0.576	1	NA	1	-0.343
SE	15267 SIGLEC10	SIGLEC10	chr19	-	51415897	51416167	51415567	51415615	51416309	51416357	8	5	6	2	0	1	0	1	0	0	98	49	1	1	0.667	1	0.75	NA	1	-0.194
SE	15269 PRR14L	PRR14L	chr22	-	31738386	31738911	31725537	31725610	31749992	31750134	4	3	0	2	0	0	0	1	0	0	98	49	1	1	0.5	1	NA	NA	1	-0.25
SE	15270 FAM193A	FAM193A	chr4	+	2655057	2655129	2646684	2646832	2657802	2657880	3	2	0	4	0	0	0	1	0	2	98	49	1	1	0.273	1	NA	NA	0.2	0.437
SE	15276 RTCB	RTCB	chr22	-	32397940	32398100	32396073	32396249	32399602	32399759	3	1	0	0	0	0	0	4	0	1	98	49	1	1	1	1	NA	NA	0.667	0.333
SE	15290 CSF2RB	CSF2RB	chr22	+	36925986	36926177	36923243	36923367	36929401	36929559	203	99	92	3	0	0	1	39	0	1	98	49	1	1	0.971	1	1	1	0.951	0.015
SE	15291 CSF2RB	CSF2RB	chr22	+	36929401	36929559	36925986	36926177	36929638	36929807	249	107	99	1	0	0	6	42	0	0	98	49	1	1	0.992	1	1	1	1	-0.003
SE	15292 CSF2RB	CSF2RB	chr22	+	36930374	36930510	36929638	36929807	36930672	36930830	68	27	46	1	1	0	2	8	0	0	98	49	1	1	0.971	0.931	1	1	1	-0.033
SE	15297 TRIOBP	TRIOBP	chr22	+	37769027	37769187	37768073	37768176	37769261	37769375	25	20	20	1	0	0	0	6	0	0	98	49	1	1	0.926	1	1	NA	1	-0.025
SE	15308 TSTD1	TSTD1	chr1	-	161038550	161038673	161037631	161038075	161038879	161038984	1	0	0	1	0	0	0	1	0	0	98	49	1	1	0.333	NA	NA	NA	1	-0.667
SE	15314 EP300	EP300	chr22	+	41135812	41135906	41131387	41131633	41137652	41137790	46	21	15	0	0	0	2	14	0	1	98	49	1	1	1	1	1	1	0.875	0.062
SE	15315 EP300	EP300	chr22	+	41146738	41146816	41141047	41141222	41147836	41147946	56	5	2	0	0	1	0	8	0	0	98	49	1	1	1	1	0.5	NA	1	-0.167
SE	15320 ADAMTSL4	ADAMTSL4	chr1	+	150556620	150556793	150556161	150556366	150556938	150556976	5	0	0	4	0	0	0	5	0	0	98	49	1	1	0.385	NA	NA	NA	1	-0.615
SE	15321 UTP11L	UTP11L	chr1	+	38016358	38016420	38012711	38012865	38017667	38017770	4	0	0	1	0	0	0	1	0	0	98	49	1	1	0.667	NA	NA	NA	1	-0.333
SE	15323 SCAF8	SCAF8	chr6	+	154787860	154788022	154778000	154778045	154792822	154792976	15	10	4	0	0	3	0	3	0	0	98	49	1	1	1	1	0.4	NA	1	-0.2
SE	15324 SCAF8	SCAF8	chr6	+	154822275	154822409	154820176	154820333	154824233	154824378	47	8	9	1	0	0	0	3	0	0	98	49	1	1	0.959	1	1	NA	1	-0.014
SE	15325 SCAF8	SCAF8	chr6	+	154824233	154824378	154822275	154822409	154827171	154827240	44	4	1	1	0	0	0	2	0	0	98	49	1	1	0.957	1	1	NA	1	-0.014
SE	15327 C16orf72	C16orf72	chr16	+	9092920	9093001	9091679	9092270	9102983	9103259	102	57	22	0	1	0	1	22	1	1	98	49	1	1	1	0.966	1	0.333	0.917	0.364
SE	15334 ARFGAP3	ARFGAP3	chr22	-	42810812	42810944	42808766	42808890	42817141	42817264	14	4	1	0	3	0	0	3	0	1	98	49	1	1	1	0.667	1	NA	0.6	0.289
SE	15335 ARFGAP3	ARFGAP3	chr22	-	42834241	42834325	42831548	42831636	42835361	42835493	28	19	6	0	0	0	0	8	1	0	98	49	1	1	1	1	1	0	1	0.5
SE	15337 TLE3	TLE3	chr15	-	70054437	70054685	70053226	70053374	70055048	70055298	92	29	26	1	1	0	4	15	0	4	98	49	1	1	0.979	0.935	1	1	0.652	0.145
SE	15338 TLE3	TLE3	chr15	-	70056297	70056374	70055048	70055298	70057473	70057658	43	8	21	2	0	0	0	3	0	0	98	49	1	1	0.915	1	1	NA	1	-0.028
SE	15340 TLE3	TLE3	chr15	-	70064453	70064470	70060529	70060649	70066013	70066218	72	39	17	0	0	0	3	13	0	1	66	49	1	1	1	1	1	1	0.906	0.047
SE	15341 TLE3	TLE3	chr15	-	70066013	70066218	70060529	70060649	70074532	70074607	27	10	5	3	0	0	0	5	0	0	98	49	1	1	0.818	1	1	NA	1	-0.061
SE	15342 TLE3	TLE3	chr15	-	70074502	70074607	70066013	70066218	70076095	70076158	71	30	16	1	1	0	0	15	0	0	98	49	1	1	0.973	0.937	1	NA	1	-0.03
SE	15343 TLE3	TLE3	chr15	-	70074532	70074607	70066013	70066218	70076095	70076158	92	36	18	1	1	0	0	15	0	0	98	49	1	1	0.979	0.947	1	NA	1	-0.025
SE	15344 TLE3	TLE3	chr15	-	70095577	70095641	70094531	70094576	70096160	70096261	106	18	28	2	0	0	0	2	0	1	98	49	1	1	0.964	1	1	NA	0.5	0.488
SE	15345 SZT2	SZT2	chr1	+	43415959	43416101	43415081	43415213	43416534	43416641	6	4	0	2	0	0	1	0	0	0	98	49	1	1	0.6	1	NA	1	NA	-0.2
SE	15346 SZT2	SZT2	chr1	+	43443596	43443796	43443351	43443477	43445893	43445984	4	0	0	3	0	0	0	1	0	0	98	49	1	1	0.4	NA	NA	NA	1	-0.6
SE	15351 TAX1BP1	TAX1BP1	chr7	+	27765833	27766021	27758030	27758133	27769675	27769834	60	42	24	1	0	0	0	11	0	0	98	49	1	1	0.968	1	1	NA	1	-0.011
SE	15352 NUP50	NUP50	chr22	+	45170987	45171177	45168167	45168246	45171599	45171683	7	3	2	6	9	1	0	2	0	0	98	49	1	1	0.368	0.143	0.5	NA	1	-0.663
SE	15356 NUP50	NUP50	chr22	+	45178237	45178900	45171599	45171683	45181285	45181367	12	12	3	1	1	0	0	1	0	0	98	49	1	1	0.857	0.857	1	NA	1	-0.095
SE	15357 NUP50	NUP50	chr22	+	45178237	45178900	45175893	45176080	45181285	45181367	20	16	3	0	0	1	0	1	0	0	98	49	1	1	1	1	0.6	NA	1	-0.133

SE	15362	CERK	CERK	chr22	-	46689991	46690200	46684409	46687206	46691571	46691777	26	10	0	2	0	0	0	2	0	0	98	49	1	1	0.867	1	NA	NA	1	-0.067
SE	15366	TBC1D22A	TBC1D22A	chr22	+	46878652	46878723	46797443	46797620	46891265	46891394	25	15	0	1	0	0	3	5	0	0	98	49	1	1	0.926	1	NA	1	1	-0.037
SE	15368	TBC1D22A	TBC1D22A	chr22	+	46974289	46974399	46912073	46912188	46997633	46997709	16	5	10	1	0	0	0	3	0	0	98	49	1	1	0.889	1	1	NA	1	-0.037
SE	15369	TBC1D22A	TBC1D22A	chr22	+	46997633	46997709	46974289	46974399	47037070	47037198	18	11	14	1	0	0	0	3	0	0	98	49	1	1	0.9	1	1	NA	1	-0.033
SE	15377	PIM3	PIM3	chr22	+	49961124	49961234	49960494	49961032	49961317	49961368	39	20	17	1	0	0	8	15	1	0	98	49	1	1	0.951	1	1	0.8	1	0.084
SE	15383	PLXNB2	PLXNB2	chr22	-	50294718	50294778	50289516	50290597	50307552	50307572	0	0	0	1	0	0	0	1	0	0	98	49	1	1	0	NA	NA	NA	1	-1
SE	15384	FBXW11	FBXW11	chr5	-	171872871	171872990	171870747	171870858	171876284	171876534	13	4	1	0	2	0	0	2	0	0	98	49	1	1	1	0.5	1	NA	1	-0.167
SE	15387	PPP6R2	PPP6R2	chr22	+	50436987	50437068	50436366	50436452	50437505	50437603	2	4	1	2	1	0	0	2	0	0	98	49	1	1	0.333	0.667	1	NA	1	-0.333
SE	15388	SBF1	SBF1	chr22	-	50457033	50457111	50456491	50456673	50459254	50459392	4	1	0	2	0	0	0	4	0	0	98	49	1	1	0.5	1	NA	NA	1	-0.25
SE	15389	LMF2	LMF2	chr22	-	50505056	50505153	50504801	50504984	50505228	50505334	10	4	8	1	0	0	1	12	0	0	98	49	1	1	0.833	1	1	1	1	-0.056
SE	15391	JOSD2	JOSD2	chr19	-	50507573	50507699	50506377	50506572	50510285	50510445	1	0	0	0	0	0	0	2	0	1	98	49	1	1	1	NA	NA	NA	0.5	0.5
SE	15393	ZNF346	ZNF346	chr5	+	177023159	177023234	177022679	177022913	177041125	177041229	0	0	0	4	0	0	1	0	0	0	98	49	1	1	0	NA	NA	1	NA	-1
SE	15418	BHLHE40	BHLHE40	chr3	+	4981391	4981515	4980300	4980408	4982835	4985180	11	10	12	0	0	0	3	3	0	1	98	49	1	1	1	1	1	1	0.6	0.2
SE	15423	SETD5	SETD5	chr3	+	9436849	9436888	9435727	9435906	9440455	9440698	2	5	0	0	0	0	0	0	0	3	88	49	1	1	1	1	NA	NA	0	1
SE	15428	ARPC4	ARPC4	chr3	+	9801660	9801756	9800184	9800296	9803842	9804013	132	94	52	0	0	0	3	21	0	1	98	49	1	1	1	1	1	1	0.913	0.043
SE	15437	RPRD2	RPRD2	chr1	+	150441880	150441958	150440922	150441023	150443230	150443283	5	4	0	1	0	0	2	0	0	0	98	49	1	1	0.714	1	NA	1	NA	-0.143
SE	15448	SEC13	SEC13	chr3	-	10312578	10312730	10311576	10312098	10315320	10315436	18	2	6	1	0	0	0	4	0	0	98	49	1	1	0.9	1	1	NA	1	-0.033
SE	15459	RAF1	RAF1	chr3	-	12600151	12600279	12599690	12599808	12600387	12600415	162	48	33	2	1	0	2	19	0	0	98	49	1	1	0.976	0.96	1	1	1	-0.021
SE	15460	RAF1	RAF1	chr3	-	12606200	12606299	12604135	12604289	12608765	12608923	154	59	46	39	3	3	0	20	0	5	98	49	1	1	0.664	0.908	0.885	NA	0.667	0.152
SE	15470	SLC6A6	SLC6A6	chr3	+	14416411	14416453	14402575	14402847	14443623	14443863	175	62	37	1	0	0	3	30	0	0	91	49	1	1	0.989	1	1	1	1	-0.004
SE	15471	SLC6A6	SLC6A6	chr3	+	14443623	14443863	14416411	14416453	14445716	14445851	155	60	57	0	0	1	5	30	0	1	98	49	1	1	1	1	0.966	1	0.937	0.02
SE	15472	SLC6A6	SLC6A6	chr3	+	14443627	14443863	14416411	14416453	14445716	14445851	80	39	28	0	0	1	5	14	0	1	98	49	1	1	1	1	0.933	1	0.875	0.04
SE	15473	SLC6A6	SLC6A6	chr3	+	14457949	14458082	14447581	14447816	14466515	14466650	191	46	45	1	1	0	3	36	0	0	98	49	1	1	0.99	0.958	1	1	1	-0.017
SE	15474	SLC6A6	SLC6A6	chr3	+	14468087	14468212	14467852	14467956	14472204	14472317	40	15	13	5	4	2	0	3	0	1	98	49	1	1	0.8	0.652	0.765	NA	0.6	0.139
SE	15475	SLC6A6	SLC6A6	chr3	+	14472204	14472317	14467852	14467956	14477204	14477342	41	22	15	2	0	0	0	5	0	0	98	49	1	1	0.911	1	1	NA	1	-0.03
SE	15476	SLC6A6	SLC6A6	chr3	+	14472204	14472317	14467852	14467956	14481670	14481841	5	5	2	1	0	0	0	1	0	0	98	49	1	1	0.714	1	1	NA	1	-0.095
SE	15477	SLC6A6	SLC6A6	chr3	+	14478465	14478568	14477204	14477342	14479084	14479185	88	33	20	1	0	0	2	14	0	0	98	49	1	1	0.978	1	1	1	1	-0.007
SE	15479	TRANK1	TRANK1	chr3	-	36838371	36838504	36834761	36834907	36838612	36838716	31	9	2	2	7	0	0	3	0	0	98	49	1	1	0.886	0.391	1	NA	1	-0.241
SE	15480	TRANK1	TRANK1	chr3	-	36842621	36842710	36838612	36838716	36846247	36846404	47	26	9	1	0	0	0	3	0	0	98	49	1	1	0.959	1	1	NA	1	-0.014
SE	15481	TRANK1	TRANK1	chr3	-	36847199	36847346	36846247	36846404	36851718	36851856	66	11	15	1	0	0	0	12	0	0	98	49	1	1	0.971	1	1	NA	1	-0.01
SE	15482	TRANK1	TRANK1	chr3	-	36855172	36858049	36852145	36852345	36858717	36858894	27	12	5	0	0	0	0	15	0	2	98	49	1	1	1	1	1	NA	0.789	0.211
SE	15483	TRANK1	TRANK1	chr3	-	36874125	36874296	36864318	36864480	36889828	36889960	63	26	5	1	0	0	3	13	0	1	98	49	1	1	0.969	1	1	1	0.867	0.056
SE	15484	TRANK1	TRANK1	chr3	-	36892201	36892340	36889828	36889960	36892900	36892984	42	16	6	0	2	0	0	20	0	0	98	49	1	1	1	0.8	1	NA	1	-0.067
SE	15485	TRANK1	TRANK1	chr3	-	36892900	36892984	36892201	36892340	36899108	36899259	27	10	5	0	0	0	0	15	0	2	98	49	1	1	1	1	1	NA	0.789	0.211
SE	15486	TRANK1	TRANK1	chr3	-	36895639	36895758	36892900	36892984	36899108	36899259	58	12	1	1	0	0	0	8	0	3	98	49	1	1	0.967	1	1	NA	0.571	0.418
SE	15496	NR2C2	NR2C2	chr3	+	15004556	15004613	15003875	15003986	15013588	15013789	1	0	0	1	0	0	0	2	0	0	98	49	1	1	0.333	NA	NA	NA	1	-0.667
SE	15497	EAF1	EAF1	chr3	+	15432086	15432223	15429912	15430007	15434347	15434538	13	4	1	2	0	2	0	3	0	0	98	49	1	1	0.765	1	0.2	NA	1	-0.345
SE	15501	HACL1	HACL1	chr3	-	15585247	15585342	15582876	15582989	15586524	15586602	1	0	0	1	0	0	0	1	0	0	98	49	1	1	0.333	NA	NA	NA	1	-0.667
SE	15508	STAB1	STAB1	chr3	+	52502631	52502727	52502158	52502228	52502998	52503109	8	1	0	1	0	0	0	4	0	0	98	49	1	1	0.8	1	NA	NA	1	-0.1
SE	15509	STAB1	STAB1	chr3	+	52512340	52512436	52511649	52511745	52512595	52512643	9	0	0	0	0	0	0	13	0	2	98	49	1	1	1	NA	NA	NA	0.765	0.235
SE	15514	KAT2B	KAT2B	chr3	+	20125904	20126113	20122667	20122804	20136941	20137052	12	3	0	0	0	0	0	2	0	1	98	49	1	1	1	1	NA	NA	0.5	0.5
SE	15515	KAT2B	KAT2B	chr3	+	20127422	20127549	20125904	20126113	20136941	20137052	27	11	8	0	2	0	0	12	0	0	98	49	1	1	1	0.733	1	NA	1	-0.089
SE	15519	TOP2B	TOP2B	chr3	-	25604759	25604870	25601099	25601225	25606042	25606122	24	5	5	2	1	0	0	10	0	1	98	49	1	1	0.857	0.714	1	NA	0.833	0.024
SE	15520	TOP2B	TOP2B	chr3	-	25618417	25618509	25615430	25615586	25618653	25618849	17	5	0	1	0	0	0	1	0	0	98	49	1	1	0.895	1	NA	NA	1	-0.052
SE	15521	TOP2B	TOP2B	chr3	-	25618653	25618849	25615430	25615586	25619861	25620062	14	2	0	0	0	0	0	2	0	2	98	49	1	1	1	1	NA	NA	0.333	0.667
SE	15524	NGLY1	NGLY1	chr3	-	25732318	25732483	25729132	25729318	25733871	25733982	6	0	2	1	0	4	0	1	0	0	98	49	1	1	0.75	NA	0.2	NA	1	-0.525
SE	15525	NGLY1	NGLY1	chr3	-	25737333	25737455	25736308	25736400	25739576	25739799	2	0	0	0	0	0	0	0	0	1	98	49	1	1	1	NA	NA	NA	0	1
SE	15527	TGFBR2	TGFBR2	chr3	+	30623198	30623273	30606501	30606977	30644746	30644915	24	5	3	9	3	4	0	3	0	2	98	49	1	1	0.571	0.455	0.273	NA	0.429	0.004
SE	15528	MS4A6A	MS4A6A	chr11	-	60179830	60179965	60178259	60178316	60181580	60181741	27	2	6	3	1	0	0	20	0	4	98	49	1	1	0.818	0.5	1	NA	0.714	0.059
SE	15529	CMTM7	CMTM7	chr3	+	32449453	32449552	32441839	32442013	32452391	32452473	16	0	0	0	0	2	0	2	0	0	98	49	1	1	1	NA	0	NA	1	-0.5
SE	15530	DYNC1LI1	DYNC1LI1	chr3	-	32530283	32530328	32529539	32529660	32530460	32530520	49	39	10	1	0	0	1	7	0	0	94	49	1	1	0.962	1	1	1	1	-0.013
SE	15531	DYNC1LI1	DYNC1LI1	chr3	-	32544875	32545106	32541036	32541206	32545848	32545965	22	4	10	2	0	0	0	4	0	0	98	49	1	1	0.846	1	1	NA	1	-0.051
SE	15532	DYNC1LI1	DYNC1LI1	chr3	-	32545848	32545965	32541036	32541206	32570345	32570419	15	9	6	2	0	0	0	2	0	0	98	49	1	1	0.789	1	1	NA	1	-0.07
SE	15534	GLB1	GLB1	chr3	-	33065462	33065557	33058088	33058269	33068229	33068290	19	15	11	0	0	0	0	3	0	1	98	49	1	1	1	1	1	NA	0.6	0.4
SE	15544	ATG16L2	ATG16L2	chr11	+	72824733	72824842	72824059	72824122	72825301	72825407	355	162	46	0	1	0	13	68	0	0	98	49	1	1	1	0.988	1	1	1	-0.004
SE	15546	ATG16L2	ATG16L2	chr11	+	72826702	72826823	72826172	72826243	72827187	72827293	55	44	20	1	0	0	1	24	0	0	98	49	1	1	0.965	1	1	1	1	-0.012
SE	15548	PDCD6IP	PDCD6IP	chr3	+	33812071	33812126	33798570	33798937	33813558	33813628	20	10	4	1	0	0	1	6	0	0	98	49	1	1	0.909	1	1	1	1	-0.03
SE	15549	PDCD6IP	PDCD6IP	chr3	+	33825186	33825340	33821954	33822082	33826479	33826580	21	4	10	1	0	0	0	8	0	0	98	49	1	1	0.913	1	1	NA	1	-0.029
SE	15550	PDCD6IP	PDCD6IP	chr3	+	33828837	33828969	33826479	33826580	33836043	33836266	15	6	3	0	0	0	0	1	0	1	98	49	1	1	1	1	1	NA	0.333	0.667
SE	15551	PDCD6IP	PDCD6IP	chr3	+	33828852	33828969	33826479	33826580	33836043	33836266	16	5	3	0	0	0	0	1	0	1	98	49	1	1	1	1	1	NA	0.333	0.667
SE	15559	MLH1	MLH1	chr3	+	37017505	37017599	37014431	37014544	37020309	37020463	2	3	0	2	0	0	0	1	0	0	98	49	1	1	0.333	1	NA	NA	1	-0.334
SE	15564	KLHL6	KLHL6	chr3	-	183499589	183499827	183494078	183494281	183508058	183508508	2	4	0	0	1	0	0	1	0	0	98	49	1	1	1	0.667	NA	NA	1	-0.166
SE	15566	GOLGA4	GOLGA4	chr3	+	37337142	37337163	37335052	37335166	37337665	37337734	4	3	0	2	0	0	0	3	0	0	70	49	1	1	0.583	1	NA	NA	1	-0.209
SE	15567	GOLGA4	GOLGA4	chr3	+	37347192	37347296	37340123	37340199	37355100	37355187	2	3	0	1	0	0	0	1	0	0	98	49	1	1	0.5	1	NA	NA	1	-0.25
SE	15575	MTHFSD	MTHFSD	chr16	-	86548034	86548111	86546558	86546649	86548463	86548577	0	0	1	1	0	0	0	1	0	0	98	49	1	1	0	NA	1	NA	1	-0.5
SE	15576	SUN2	SUN2	chr22	-	38738872	38738988	38738586	38738754	38739341	38739426	45	18	11	0	3	0	0	6	0	4	98	49	1	1	1	0.75	1	NA	0.429	0.488
SE	15589	TRAK1	TRAK1	chr3	+	42176813	42176890	42125419	42125614	42184684	42184801	9	4	6	1	0	0	0	3	0	0	98	49	1	1	0.818	1	1	NA	1	-0.061
SE	15595	ABHD5	ABHD5	chr3	+	43702214	43702587	43699275	43699361	43711708	43711863	85	16	18	1	0	0	1	13	0	0	98	49	1	1	0.977	1	1	1	1	-0.008
SE	15596	ABHD5	ABHD5	chr3	+	43714946	43715058	43711708	43711863	43717670	43717857	89	26	26	1	0	0	0	18	0	0	98	49	1	1	0.978	1	1	NA	1	-0.007
SE	15597	ABHD5	ABHD5	chr3	+	43717670	43717857	43714946	43715058	43718442	43722725	74	17	10	7	1	0	0	17	0	3	98	49	1	1	0.841	0.895	1	NA	0.739	0.173
SE	15598	LARS2	LARS2	chr3	+	45491516	45491800	45488696	45488812	45496274	45496373	2	0	3	1	0	0	0	1	0	0	98	49	1	1	0.5	NA	1	NA	1	-0.25
SE	15599	SACM1L	SACM1L	chr3	+	45735234	45735373	45732052	45732151	45737582	45737652	16	5	0	1	0	0	0	2	0	0	98	49	1	1	0.889	1	NA	NA	1	-0.055
SE	15605	YDJC	YDJC	chr22	-	21629009	21629187	21628089	21628787	21629307	21629407	3	0	0	1	5	0	0	0	0	2	98	49	1	1	0.6	0	NA	NA	0	0.3
SE	15606	HIPK3	HIPK3	chr11	+	33337074	33337194	33328509	33328633	33338756	33338843	93	20	18	2	0	0	1	27	0	0	98	49	1	1	0.959	1	1	1	1	-0.014
SE	15608	HIPK3	HIPK3	chr11	+	33348165	33348228	33347851	33348013	33349146	33349287	14	4	2	2	1	0	0	6	0	0	98	49	1	1	0.778	0.667	1	NA	1	-0.185
SE	15610	HIPK3	HIPK3	chr11	+	33348521	33348818	33348165	33348228	33349146	33349287	52	23	5	3	2	0	0	4	0	0	98	49	1	1	0.897	0.852	1	NA	1	-0.084
SE	15611	HIPK3	HIPK3	chr11	+	33349146	33349287	33348521	33348818	33351607	33351843	68	32	7	1	0	0	0	9	0	0	98	49	1	1	0.971	1	1	NA	1	-0.01
SE	15612	SCAP	SCAP	chr3	-	47417303	47417826	47417121	47417207	47418133	47418249	5	2	4	1	0	0	1	0	0	0	98	49	1	1	0.714	1	1	1	NA	-0.095
SE	15617	SMARCC1	SMARCC1	chr3	-	47662333	47662592	47661293	47661455	47670657	47670717	22	3	1	1	0	0	1	1	0	0	98	49	1	1	0.917	1	1	1	1	-0.028
SE	15620	SMARCC1	SMARCC1	chr3	-	47729024	47729094	47720665	47720735	47736033	47736126	24	11	4	0	2	0	0	2	0	0	98	49	1	1	1	0.733	1	NA	1	-0.089
SE	15621	MAP4	MAP4	chr3	-	47918718	47918841	47915950	47917174	47921764	47921878	0	1	2	0	0	0	0	0	0	1	98	49	1	1	NA	1	1	NA	0	1
SE	15630	UQCRC1	UQCRC1	chr3	-	48604232	48604431	48603563	48603643	48604650	48604780	15	2	9	0	1	0	0	2	0	0	98	49	1	1	1	0.5	1	NA	1	-0.167
SE	15631	UQCRC1	UQCRC1	chr3	-	48605769	48605856	48604650	48604780	48609161	48609302	7	0	1	2	0	0	0	6	0	0	98	49	1	1	0.636	NA	1	NA	1	-0.182
SE	15632	NCOR1	NCOR1	chr17	-	16039432	16039654	16034764	16034944	16040440	16040494	32	0	2	1	0	0	0	3	0	0	98	49	1	1	0.941	NA	1	NA	1	-0.03
SE	15633	NCOR1	NCOR1	chr17	-	16048844	16048988	16046950	16047093	16057513	16057737	12	4	3	0	0	0	0	3	0	2	98	49	1	1	1	1	1	NA	0.429	0.571
SE	15634	NCOR1	NCOR1	chr17	-	16067893	16068121	16065484	16065694	16070164	16070525	15	6	4	1	1	0	1	2	0	1	98	49	1	1	0.882	0.75	1	1	0.5	0.127
SE	15635	NCOR1	NCOR1	chr17	-	16072144	16072228	16071408	16071665	16073428	16073569	14	9	4	1	0	0	0	4	0	0	98	49	1	1	0.875	1	1	NA	1	-0.042
SE	15636	NCOR1	NCOR1	chr17	-	16080407	16080509	16079963	16080064	16080606	16080727	29	9	1	2	0	0	0	8	0	0	98	49	1	1	0.879	1	1	NA	1	-0.04
SE	15637	NCOR1	NCOR1	chr17	-	16126081	16126206	16121051	16121269	16137310	16137412	14	9	2	0	2	0	0	1	0	0	98	49	1	1	1	0.692	1	NA	1	-0.103
SE	15643	ARIH2	ARIH2	chr3	+	48922747	48922811	48918847	48918998	48927461	48927813	4	4	0	1	0	0	0	1	0	0	98	49	1	1	0.667	1	NA	NA	1	-0.166
SE	15645	NMT2	NMT2	chr10	-	15130141	15130312	15128349	15128458	15132816	15132933	4	0	2	1	0	0	0	1	0	0	98	49	1	1	0.667	NA	1	NA	1	-0.166
SE	15647	QRICH1	QRICH1	chr3	-	49044389	49044504	49033119	49033228	49046424	49046579	8	0	0	0	0	1	0	5	0	0	98	49	1	1	1	NA	0	NA	1	-0.5
SE	15648	QRICH1	QRICH1	chr3	-	49092396	49092456	49076708	49077038	49093911	49094071	1	2	0	4	5	0	0	0	1	0	98	49	1	1	0.111	0.167	NA	0	NA	0.139
SE	15667	UBA7	UBA7	chr3	-	49809389	49809464	49808975	49809159	49809541	49809725	7	3	0	1	0	0	0	2	0	1	98	49	1	1	0.778	1	NA	NA	0.5	0.389
SE	15671	RBM6	RBM6	chr3	+	49967469	49968748	49962575	49962685	49972058	49972148	14	1	6	2	0	0	0	4	0	0	98	49	1	1	0.778	1	1	NA	1	-0.074
SE	15672	RBM6	RBM6	chr3	+	49999439	49999513	49975322	49975392	50048244	50048319	13	7	0	3	0	0	0	1	0	0	98	49	1	1	0.684	1	NA	NA	1	-0.158
SE	15673	RBM6	RBM6	chr3	+	50059648	50059746	50058401	50058562	50060955	50060998	10	9	1	0	0	0	0	2	0	2	98	49	1	1	1	1	1	NA	0.333	0.667
SE	15677	HEXB	HEXB	chr5	+	74713505	74713635	74705218	74705320	74715509	74715690	12	10	5	0	0	0	0	12	0	2	98	49	1	1	1	1	1	NA	0.75	0.25
SE	15685	MAPKAPK3	MAPKAPK3	chr3	+	50644408	50644532	50642252	50642332	50645709	50645785	34	23	12	2	5	0	0	11	0	5	98	49	1	1	0.895	0.697	1	NA	0.524	0.34
SE	15686	MAPKAPK3	MAPKAPK3	chr3	+	50645709	50645785	50644408	50644532	50646139	50646264	18	16	7	0	1	0	1	3	0	0	98	49	1	1	1	0.889	1	1	1	-0.037
SE	15691	DSE	DSE	chr6	+	116433342	116433550	116430953	116431193	116435586	116438281	8	1	3	2	0	0	0	1	0	0	98	49	1	1	0.667	1	1	NA	1	-0.111
SE	15693	DENND6A	DENND6A	chr3	-	57630420	57630523	57628810	57628885	57630714	57630824	9	18	5	2	0	0	0	8	0	0	98	49	1	1	0.692	1	1	NA	1	-0.103
SE	15695	WDR82	WDR82	chr3	-	52270711	52270809	52266951	52267018	52278200	52278643	20	13	5	0	0	1	0	5	0	1	98	49	1	1	1	1	0.714	NA	0.714	0.191
SE	15696	BAP1	BAP1	chr3	-	52405109	52405294	52404452	52404586	52405764	52405912	5	0	2	0	2	0	0	3	0	0	98	49	1	1	1	0	1	NA	1	-0.333
SE	15698	BAP1	BAP1	chr3	-	52408473	52408606	52407957	52408077	52409553	52409608	7	3	1	0	1	0	1	1	0	0	98	49	1	1	1	0.6	1	1	1	-0.133
SE	15703	C4orf29	C4orf29	chr4	+	127989720	127989821	127984338	127984423	128008919	128008998	4	0	2	1	1	0	0	3	0	0	98	49	1	1	0.667	0	1	NA	1	-0.444
SE	15709	FAM208A	FAM208A	chr3	-	56663540	56663572	56662384	56662490	56668396	56668558	2	0	0	0	0	0	0	0	0	1	81	49	1	1	1	NA	NA	NA	0	1
SE	15710	FAM208A	FAM208A	chr3	-	56666259	56666384	56663540	56663572	56668396	56668558	12	0	2	1	0	0	0	1	0	0	98	49	1	1	0.857	NA	1	NA	1	-0.072
SE	15729	ATXN7	ATXN7	chr3	+	63952378	63952483	63913156	63913225	63979914	63980167	35	5	5	1	0	0	0	5	0	0	98	49	1	1	0.946	1	1	NA	1	-0.018
SE	15730	ATXN7	ATXN7	chr3	+	63982185	63982445	63979914	63980167	63982938	63983021	21	12	0	1	0	0	0	9	0	0	98	49	1	1	0.913	1	NA	NA	1	-0.043
SE	15732	ATXN7	ATXN7	chr3	+	63990175	63990374	63988058	63988324	63990737	63990859	13	5	0	3	0	0	0	2	0	0	98	49	1	1	0.684	1	NA	NA	1	-0.158
SE	15733	ATXN7	ATXN7	chr3	+	63997620	63997687	63995504	63996483	63999449	64003460	6	6	0	1	0	1	0	2	0	2	98	49	1	1	0.75	1	0	NA	0.333	0.25
SE	15740	ARL6IP5	ARL6IP5	chr3	+	69101838	69102056	69084938	69085223	69104463	69106088	25	12	7	0	1	0	0	12	0	0	98	49	1	1	1	0.857	1	NA	1	-0.048
SE	15741	ERBB2IP	ERBB2IP	chr5	+	66028273	66028343	66026301	66026417	66038382	66038482	60	18	13	1	0	0	0	7	0	0	98	49	1	1	0.968	1	1	NA	1	-0.011
SE	15742	ERBB2IP	ERBB2IP	chr5	+	66068876	66069020	66053405	66054951	66072168	66072291	2	2	0	5	2	0	0	0	0	2	98	49	1	1	0.167	0.333	NA	NA	0	0.25
SE	15743	ERBB2IP	ERBB2IP	chr5	+	66068876	66069020	66053405	66054951	66075023	66075230	2	0	0	9	5	5	0	0	0	1	98	49	1	1	0.1	0	0	NA	0	0.033
SE	15744	ERBB2IP	ERBB2IP	chr5	+	66072168	66072291	66053405	66054951	66075023	66075230	6	9	0	9	5	5	0	2	0	1	98	49	1	1	0.25	0.474	0	NA	0.5	-0.259
SE	15748	COQ2	COQ2	chr4	-	83269859	83269993	83267585	83267774	83272086	83272172	8	2	0	1	0	0	0	4	0	0	98	49	1	1	0.8	1	NA	NA	1	-0.1
SE	15749	PPP4R2	PPP4R2	chr3	+	73047185	73047356	72998076	72998158	73059036	73059130	24	10	12	1	0	0	0	13	0	0	98	49	1	1	0.923	1	1	NA	1	-0.026
SE	15751	GBE1	GBE1	chr3	-	81646391	81646482	81642780	81642990	81648855	81648991	8	2	3	1	0	0	0	0	0	1	98	49	1	1	0.8	1	1	NA	0	0.933
SE	15752	GBE1	GBE1	chr3	-	81705443	81705613	81670837	81670953	81761374	81761799	5	6	1	0	1	0	1	1	0	0	98	49	1	1	1	0.75	1	1	1	-0.083
SE	15761	LSP1	LSP1	chr11	+	1883418	1883560	1880086	1880224	1883931	1884024	182	100	71	0	1	1	4	21	0	0	98	49	1	1	1	0.98	0.973	1	1	-0.016
SE	15762	LSP1	LSP1	chr11	+	1884279	1884323	1883931	1884024	1884499	1884581	322	196	157	2	0	0	5	71	0	1	93	49	1	1	0.988	1	1	1	0.974	0.009
SE	15763	LSP1	LSP1	chr11	+	1887236	1887314	1886731	1886866	1887473	1887576	130	85	44	1	0	0	6	18	0	1	98	49	1	1	0.985	1	1	1	0.9	0.045
SE	15769	CRYBG3	CRYBG3	chr3	+	97892859	97892993	97889354	97889390	97895958	97896085	1	0	0	0	0	1	0	3	0	0	98	49	1	1	1	NA	0	NA	1	-0.5
SE	15771	UBTF	UBTF	chr17	-	44212343	44212454	44211872	44212006	44212818	44212939	4	2	1	9	1	1	0	1	0	1	98	49	1	1	0.182	0.5	0.333	NA	0.333	0.005
SE	15773	CWF19L1	CWF19L1	chr10	-	100256261	100256476	100253420	100253539	100260217	100260319	7	9	1	1	1	0	0	4	0	0	98	49	1	1	0.778	0.818	1	NA	1	-0.135
SE	15774	CWF19L1	CWF19L1	chr10	-	100260217	100260319	100256261	100256476	100260965	100261028	14	8	1	1	1	0	0	3	0	0	98	49	1	1	0.875	0.8	1	NA	1	-0.108
SE	15778	TBC1D23	TBC1D23	chr3	+	100310402	100310542	100306436	100306543	100316098	100316187	6	2	2	0	0	0	0	0	0	1	98	49	1	1	1	1	1	NA	0	1
SE	15783	SENP7	SENP7	chr3	-	101417592	101417790	101398860	101399055	101458954	101459052	11	14	2	1	0	0	0	0	0	1	98	49	1	1	0.846	1	1	NA	0	0.949
SE	15785	HSP90AA1	HSP90AA1	chr14	-	102082110	102082444	102080737	102081821	102083033	102083302	29	17	4	0	0	0	0	5	0	1	98	49	1	1	1	1	1	NA	0.714	0.286
SE	15786	HSP90AA1	HSP90AA1	chr14	-	102085757	102086124	102085297	102085431	102086216	102086378	20	14	7	0	2	0	0	10	0	0	98	49	1	1	1	0.778	1	NA	1	-0.074
SE	15787	TRA2B	TRA2B	chr3	-	185923795	185923984	185922010	185922126	185925463	185925626	19	6	8	1	0	0	0	5	0	0	98	49	1	1	0.905	1	1	NA	1	-0.032
SE	15799	FKBP15	FKBP15	chr9	-	113196931	113197078	113194026	113194169	113198854	113198923	16	8	6	1	0	0	0	4	0	0	98	49	1	1	0.889	1	1	NA	1	-0.037
SE	15813	MRVI1	MRVI1	chr11	-	10580454	10580589	10573090	10576575	10581866	10581986	13	12	6	1	0	0	0	6	0	0	98	49	1	1	0.867	1	1	NA	1	-0.044
SE	15815	MRVI1	MRVI1	chr11	-	10600917	10601059	10594145	10594195	10603119	10603251	47	14	16	1	2	0	0	4	0	1	98	49	1	1	0.959	0.778	1	NA	0.667	0.245
SE	15833	AOAH	AOAH	chr7	-	36514471	36514590	36512940	36513380	36522038	36522115	5	0	0	13	2	0	0	1	0	6	98	49	1	1	0.161	0	NA	NA	0.077	0.004
SE	15834	AOAH	AOAH	chr7	-	36548611	36548686	36540318	36540491	36549438	36549475	87	24	15	10	0	2	0	16	0	1	98	49	1	1	0.813	1	0.789	NA	0.889	-0.022
SE	15836	AOAH	AOAH	chr7	-	36637850	36637910	36632035	36632106	36659165	36659265	60	21	5	0	2	0	6	12	0	0	98	49	1	1	1	0.84	1	1	1	-0.053
SE	15837	AOAH	AOAH	chr7	-	36659165	36659265	36637850	36637910	36673942	36674009	48	12	1	2	2	0	0	10	0	1	98	49	1	1	0.923	0.75	1	NA	0.833	0.058
SE	15842	GON4L	GON4L	chr1	-	155777621	155777820	155776394	155776481	155783985	155784089	8	2	1	0	0	0	1	5	0	1	98	49	1	1	1	1	1	1	0.714	0.143
SE	15846	ATP6V0E1	ATP6V0E1	chr5	+	172994774	172994822	172983759	172983964	173020237	173020367	33	29	33	0	1	0	4	18	0	0	97	49	1	1	1	0.936	1	1	1	-0.021
SE	15847	ARHGAP25	ARHGAP25	chr2	+	68787839	68787956	68782232	68782320	68807272	68807480	59	31	30	3	1	2	0	6	0	0	98	49	1	1	0.908	0.939	0.882	NA	1	-0.09
SE	15850	SYNE2	SYNE2	chr14	+	64009965	64010116	64007042	64007222	64016472	64016631	28	22	16	0	0	0	0	3	0	2	98	49	1	1	1	1	1	NA	0.429	0.571
SE	15852	SYNE2	SYNE2	chr14	+	64075944	64076100	64073967	64074136	64078465	64078606	39	10	5	0	2	0	0	3	0	0	98	49	1	1	1	0.714	1	NA	1	-0.095
SE	15853	SYNE2	SYNE2	chr14	+	64163401	64163581	64162071	64162276	64165284	64165410	20	14	8	0	0	0	0	1	0	1	98	49	1	1	1	1	1	NA	0.333	0.667
SE	15854	SYNE2	SYNE2	chr14	+	64215285	64215354	64214140	64214470	64216247	64216387	3	0	0	1	1	0	0	0	0	4	98	49	1	1	0.6	0	NA	NA	0	0.3
SE	15855	SYNE2	SYNE2	chr14	+	64221533	64221704	64220436	64220637	64223188	64223380	10	3	0	1	0	0	0	2	0	0	98	49	1	1	0.833	1	NA	NA	1	-0.084
SE	15856	SYNE2	SYNE2	chr14	+	64221575	64221704	64220436	64220637	64223188	64223380	14	3	0	1	0	0	0	2	0	0	98	49	1	1	0.875	1	NA	NA	1	-0.062
SE	15857	EAF2	EAF2	chr3	+	121854686	121854823	121844452	121844547	121857010	121857156	3	1	2	1	0	0	0	1	0	0	98	49	1	1	0.6	1	1	NA	1	-0.133
SE	15872	SMURF2	SMURF2	chr17	-	64591083	64591149	64586085	64586170	64593439	64593573	17	3	0	1	0	0	0	1	0	0	98	49	1	1	0.895	1	NA	NA	1	-0.052
SE	15879	MR1	MR1	chr1	+	181053572	181053677	181050010	181050286	181055224	181061938	6	0	3	1	0	0	0	1	0	0	98	49	1	1	0.75	NA	1	NA	1	-0.125
SE	15883	ZXDC	ZXDC	chr3	-	126466154	126466325	126458900	126462220	126470894	126471025	20	5	1	2	0	0	0	5	0	0	98	49	1	1	0.833	1	1	NA	1	-0.056
SE	15884	HDAC1	HDAC1	chr1	+	32330577	32330686	32329067	32329160	32330767	32330908	14	6	0	1	0	0	0	3	0	0	98	49	1	1	0.875	1	NA	NA	1	-0.062
SE	15885	HDAC1	HDAC1	chr1	+	32332700	32332749	32332089	32332242	32333016	32333623	9	3	0	0	1	0	0	4	0	0	98	49	1	1	1	0.6	NA	NA	1	-0.2

SE	15889	ULK4	ULK4	chr3	-	41715446	41715568	41715236	41715293	41717727	41717861	0	0	2	1	0	0	0	1	0	0	98	49	1	1	0	NA	1	NA	1	-0.5
SE	15890	ULK4	ULK4	chr3	-	41915976	41916052	41912806	41912899	41918456	41918540	1	0	0	4	5	0	0	0	0	1	98	49	1	1	0.111	0	NA	NA	0	0.056
SE	15891	DGKZ	DGKZ	chr11	+	46369940	46370009	46369493	46369550	46371312	46371384	17	5	2	4	2	0	0	5	0	0	98	49	1	1	0.68	0.556	1	NA	1	-0.255
SE	15906	TOPBP1	TOPBP1	chr3	-	133624051	133624175	133623310	133623457	133628361	133628471	5	4	2	0	0	0	0	2	0	2	98	49	1	1	1	1	1	NA	0.333	0.667
SE	15908	CEP63	CEP63	chr3	+	134532777	134532900	134531844	134531940	134537154	134537268	27	15	1	2	0	0	1	1	0	0	98	49	1	1	0.871	1	1	1	1	-0.043
SE	15909	CEP63	CEP63	chr3	+	134547334	134547472	134546148	134546288	134549061	134549176	13	10	1	13	4	6	0	4	0	6	98	49	1	1	0.333	0.556	0.077	NA	0.25	0.072
SE	15910	CEP63	CEP63	chr3	+	134558141	134558347	134551925	134552012	134559149	134559429	20	16	2	0	2	0	2	0	0	0	98	49	1	1	1	0.8	1	1	NA	-0.067
SE	15912	CEP63	CEP63	chr3	+	134559149	134559429	134551925	134552012	134561376	134565028	5	2	3	2	4	3	0	0	0	4	98	49	1	1	0.556	0.2	0.333	NA	0	0.363
SE	15914	FMR1	FMR1	chrX	+	147938098	147938161	147937465	147937600	147940575	147940662	8	1	0	2	2	0	0	0	0	1	98	49	1	1	0.667	0.2	NA	NA	0	0.433
SE	15919	STAG1	STAG1	chr3	-	136568764	136568861	136542118	136542195	136604308	136604473	15	7	3	2	0	0	0	4	0	0	98	49	1	1	0.789	1	1	NA	1	-0.07
SE	15920	RPS6KA1	RPS6KA1	chr1	+	26546866	26546983	26545851	26546035	26547188	26547270	60	36	24	1	5	1	3	19	0	1	98	49	1	1	0.968	0.783	0.923	1	0.905	-0.061
SE	15921	RPS6KA1	RPS6KA1	chr1	+	26551396	26551477	26547188	26547270	26551643	26551723	88	46	31	1	0	0	1	19	0	0	98	49	1	1	0.978	1	1	1	1	-0.007
SE	15925	RPS6KA1	RPS6KA1	chr1	+	26561504	26561663	26561044	26561134	26571448	26571610	41	38	38	0	0	0	0	12	0	1	98	49	1	1	1	1	1	NA	0.857	0.143
SE	15926	RPS6KA1	RPS6KA1	chr1	+	26572175	26572293	26571848	26571925	26573223	26573361	49	24	31	1	0	0	0	13	0	0	98	49	1	1	0.961	1	1	NA	1	-0.013
SE	15927	ARMC8	ARMC8	chr3	+	138188400	138188530	138187247	138187599	138209816	138209893	2	0	0	0	2	0	0	2	0	0	98	49	1	1	1	0	NA	NA	1	-0.5
SE	15929	ARMC8	ARMC8	chr3	+	138188454	138188530	138187247	138187599	138209816	138209893	2	0	0	0	2	0	0	4	0	0	98	49	1	1	1	0	NA	NA	1	-0.5
SE	15932	ARMC8	ARMC8	chr3	+	138209816	138209893	138188400	138188530	138221925	138221997	12	0	2	0	3	0	0	4	0	0	98	49	1	1	1	0	1	NA	1	-0.333
SE	15933	ARMC8	ARMC8	chr3	+	138223388	138223531	138221925	138221997	138223635	138223733	13	5	0	0	1	0	0	2	0	0	98	49	1	1	1	0.714	NA	NA	1	-0.143
SE	15935	ARMC8	ARMC8	chr3	+	138237308	138237384	138235033	138235114	138237481	138237572	20	3	1	0	0	0	0	1	0	2	98	49	1	1	1	1	1	NA	0.2	0.8
SE	15941	CSF2RA	CSF2RA	chrX	+	1300490	1300626	1295426	1295456	1303922	1304019	19	15	15	0	0	0	0	19	0	4	98	49	1	1	1	1	1	NA	0.704	0.296
SE	15942	CSF2RA	CSF2RA	chrX	+	1303260	1303362	1300490	1300626	1303922	1304019	4	1	0	12	4	7	0	1	0	5	98	49	1	1	0.143	0.111	0	NA	0.091	-0.006
SE	15944	CSF2RA	CSF2RA	chrX	+	1305445	1305527	1300490	1300626	1309401	1309935	25	8	4	0	1	0	0	1	0	0	98	49	1	1	1	0.8	1	NA	1	-0.067
SE	15950	RASA2	RASA2	chr3	+	141573943	141574067	141573146	141573221	141576999	141577106	11	4	1	1	0	0	0	1	0	0	98	49	1	1	0.846	1	1	NA	1	-0.051
SE	15952	RASA2	RASA2	chr3	+	141609876	141610066	141609419	141609523	141612282	141615363	14	8	9	0	3	0	0	2	0	0	98	49	1	1	1	0.571	1	NA	1	-0.143
SE	15956	ATR	ATR	chr3	-	142542664	142542757	142540903	142541034	142547724	142547910	4	3	0	1	0	0	0	2	0	0	98	49	1	1	0.667	1	NA	NA	1	-0.166
SE	15958	NEK9	NEK9	chr14	-	75121118	75121174	75120509	75120580	75124045	75124223	6	2	0	0	1	0	0	2	0	0	98	49	1	1	1	0.5	NA	NA	1	-0.25
SE	15959	AREL1	AREL1	chr14	-	74667464	74667594	74667318	74667377	74669648	74669774	23	6	1	1	0	0	0	3	0	0	98	49	1	1	0.92	1	1	NA	1	-0.027
SE	15960	AREL1	AREL1	chr14	-	74669946	74670126	74669648	74669774	74670761	74670871	21	12	4	1	1	0	2	2	0	3	98	49	1	1	0.913	0.857	1	1	0.25	0.298
SE	15961	AREL1	AREL1	chr14	-	74674033	74674111	74673076	74673218	74675698	74675946	23	18	4	8	3	0	0	2	0	0	98	49	1	1	0.59	0.75	1	NA	1	-0.22
SE	15962	AREL1	AREL1	chr14	-	74684453	74684680	74683295	74683533	74685599	74685660	18	14	2	2	0	2	0	4	0	0	98	49	1	1	0.818	1	0.333	NA	1	-0.283
SE	15964	DPEP2	DPEP2	chr16	-	67987851	67987987	67987389	67987760	67989322	67989398	61	31	21	6	0	2	0	18	0	0	98	49	1	1	0.836	1	0.84	NA	1	-0.108
SE	15966	DPEP2	DPEP2	chr16	-	67991837	67991979	67991114	67991184	67992063	67992193	21	13	7	7	0	0	0	4	0	0	98	49	1	1	0.6	1	1	NA	1	-0.133
SE	15968	PLSCR1	PLSCR1	chr3	-	146528613	146528831	146525604	146525647	146533469	146533550	8	4	7	0	2	0	0	8	0	2	98	49	1	1	1	0.5	1	NA	0.667	0.166
SE	15976	7-Mar	7-Mar	chr2	+	159745769	159745937	159743060	159743253	159747804	159748903	63	39	21	0	2	0	0	4	0	0	98	49	1	1	1	0.907	1	NA	1	-0.031
SE	15977	7-Mar	7-Mar	chr2	+	159762879	159762993	159759225	159759335	159764625	159764674	56	31	17	1	3	0	0	8	0	0	98	49	1	1	0.966	0.838	1	NA	1	-0.065
SE	15982	FUBP3	FUBP3	chr9	+	130634666	130634738	130632201	130632278	130635998	130636126	3	0	0	0	1	0	0	1	0	0	98	49	1	1	1	0	NA	NA	1	-0.5
SE	15988	NSUN4	NSUN4	chr1	+	46352867	46353028	46344800	46345144	46360703	46360828	1	0	0	1	0	0	0	1	0	0	98	49	1	1	0.333	NA	NA	NA	1	-0.667
SE	15989	TSC22D2	TSC22D2	chr3	+	150423048	150423120	150408334	150411308	150457075	150457127	1	0	0	0	3	0	0	4	0	1	98	49	1	1	1	0	NA	NA	0.667	-0.167
SE	15991	SERP1	SERP1	chr3	-	150545702	150545778	150541992	150544498	150546051	150546641	58	20	6	1	1	0	1	14	0	0	98	49	1	1	0.967	0.909	1	1	1	-0.041
SE	15992	EIF2A	EIF2A	chr3	+	150571957	150572529	150568175	150568292	150575648	150575762	14	0	2	1	0	0	0	6	0	0	98	49	1	1	0.875	NA	1	NA	1	-0.062
SE	15993	EIF2A	EIF2A	chr3	+	150575648	150575762	150571957	150572529	150581617	150581746	14	1	0	1	0	0	0	8	0	0	98	49	1	1	0.875	1	NA	NA	1	-0.062
SE	15998	MME	MME	chr3	+	155114993	155115155	155085058	155085094	155116478	155116559	236	111	57	2	0	0	0	30	0	0	98	49	1	1	0.983	1	1	NA	1	-0.006
SE	15999	MME	MME	chr3	+	155141990	155142127	155140190	155140292	155142236	155142330	84	27	32	0	1	0	0	12	0	0	98	49	1	1	1	0.931	1	NA	1	-0.023
SE	16000	MME	MME	chr3	+	155141990	155142127	155140190	155140292	155143442	155143571	67	18	23	3	1	2	0	9	0	0	98	49	1	1	0.918	0.9	0.852	NA	1	-0.11
SE	16001	MME	MME	chr3	+	155142236	155142330	155140190	155140292	155143442	155143571	95	36	23	3	1	2	0	7	0	0	98	49	1	1	0.941	0.947	0.852	NA	1	-0.087
SE	16002	MME	MME	chr3	+	155142236	155142330	155141990	155142127	155143442	155143571	117	46	32	5	2	0	0	10	0	0	98	49	1	1	0.921	0.92	1	NA	1	-0.053
SE	16003	MME	MME	chr3	+	155168491	155168625	155166901	155167021	155168731	155168797	177	57	55	10	0	0	0	10	0	3	98	49	1	1	0.898	1	1	NA	0.625	0.341
SE	16004	MME	MME	chr3	+	155172116	155172212	155168731	155168797	155172535	155172612	117	74	37	1	0	1	0	17	0	0	98	49	1	1	0.983	1	0.949	NA	1	-0.023
SE	16010	SLC33A1	SLC33A1	chr3	-	155833856	155834041	155833467	155833585	155842431	155842619	3	1	0	1	0	0	0	1	0	0	98	49	1	1	0.6	1	NA	NA	1	-0.2
SE	16017	MFSD1	MFSD1	chr3	+	158805361	158805474	158804318	158804371	158807039	158807082	41	13	11	3	1	2	0	10	0	0	98	49	1	1	0.872	0.867	0.733	NA	1	-0.176
SE	16018	MFSD1	MFSD1	chr3	+	158821983	158822140	158821596	158821653	158823427	158823525	38	4	7	1	0	0	2	16	0	0	98	49	1	1	0.95	1	1	1	1	-0.017
SE	16024	NMD3	NMD3	chr3	+	161246335	161246448	161242507	161242653	161247257	161247330	7	0	0	1	3	0	3	5	0	0	98	49	1	1	0.778	0	NA	1	1	-0.611
SE	16027	ATP11C	ATP11C	chrX	-	139731650	139731755	139726345	139728962	139737915	139738069	0	0	0	2	0	0	0	2	0	0	98	49	1	1	0	NA	NA	NA	1	-1
SE	16028	RNPEPL1	RNPEPL1	chr2	+	240574112	240574348	240573774	240573891	240574514	240574628	18	9	3	3	0	0	0	6	0	1	98	49	1	1	0.75	1	1	NA	0.75	0.167
SE	16035	PDCD10	PDCD10	chr3	-	167725400	167725473	167720061	167720273	167734805	167734863	5	1	0	4	0	0	0	5	0	1	98	49	1	1	0.385	1	NA	NA	0.714	-0.021
SE	16036	YTHDC2	YTHDC2	chr5	+	113540967	113541116	113539073	113539181	113542367	113542503	9	6	0	0	1	0	0	1	0	0	98	49	1	1	1	0.75	NA	NA	1	-0.125
SE	16038	RASGEF1B	RASGEF1B	chr4	-	81447503	81447578	81445742	81445838	81448068	81448281	5	0	0	1	0	0	0	5	0	0	98	49	1	1	0.714	NA	NA	NA	1	-0.286
SE	16039	RASGEF1B	RASGEF1B	chr4	-	81457498	81457621	81456650	81456788	81459331	81459514	16	0	0	4	0	0	0	27	0	0	98	49	1	1	0.667	NA	NA	NA	1	-0.333
SE	16041	MYNN	MYNN	chr3	+	169782464	169782643	169780589	169780749	169783476	169783560	3	1	2	0	4	0	0	4	0	0	98	49	1	1	1	0.111	1	NA	1	-0.296
SE	16044	ECE1	ECE1	chr1	-	21221746	21221842	21217246	21220131	21227158	21227226	29	9	10	1	0	0	3	9	0	0	98	49	1	1	0.935	1	1	1	1	-0.022
SE	16045	ECE1	ECE1	chr1	-	21225249	21225440	21221746	21221842	21227158	21227226	41	24	13	0	3	0	0	8	0	1	98	49	1	1	1	0.8	1	NA	0.8	0.133
SE	16047	ECE1	ECE1	chr1	-	21279190	21279332	21272698	21272911	21290069	21290156	46	22	11	2	0	0	2	12	0	0	98	49	1	1	0.92	1	1	1	1	-0.027
SE	16048	PHC3	PHC3	chr3	-	170145422	170145521	170136418	170136665	170171372	170171450	11	8	14	1	1	0	0	1	0	0	98	49	1	1	0.846	0.8	1	NA	1	-0.118
SE	16049	PHC3	PHC3	chr3	-	170149085	170149244	170145422	170145521	170171372	170171450	14	12	13	1	0	0	0	3	0	0	98	49	1	1	0.875	1	1	NA	1	-0.042
SE	16051	ZMYM5	ZMYM5	chr13	-	19851354	19851448	19838699	19838985	19851688	19852190	9	2	3	1	0	0	0	1	0	0	98	49	1	1	0.818	1	1	NA	1	-0.061
SE	16052	ZMYM5	ZMYM5	chr13	-	19862398	19862466	19851688	19852190	19863449	19863636	19	4	2	1	0	0	0	2	0	0	98	49	1	1	0.905	1	1	NA	1	-0.032
SE	16056	PIK3CA	PIK3CA	chr3	+	179220985	179221157	179219948	179220052	179224080	179224187	20	17	6	0	0	0	0	4	0	1	98	49	1	1	1	1	1	NA	0.667	0.333
SE	16068	ATP11B	ATP11B	chr3	+	182885950	182886010	182884752	182884898	182887585	182887713	43	22	24	1	0	0	0	3	0	0	98	49	1	1	0.956	1	1	NA	1	-0.015
SE	16074	SIPA1L2	SIPA1L2	chr1	-	232404124	232404178	232403447	232403571	232415493	232415625	1	0	0	11	0	0	0	0	0	2	98	49	1	1	0.043	NA	NA	NA	0	0.043
SE	16075	SIPA1L2	SIPA1L2	chr1	-	232441767	232441868	232441290	232441394	232443601	232443685	9	3	2	3	0	0	0	2	0	0	98	49	1	1	0.6	1	1	NA	1	-0.133
SE	16076	SIPA1L2	SIPA1L2	chr1	-	232471370	232471528	232464839	232465416	232479626	232479730	29	1	4	1	0	0	0	3	0	0	98	49	1	1	0.935	1	1	NA	1	-0.022
SE	16081	UNC119	UNC119	chr17	-	28547998	28548101	28546706	28547849	28548591	28548705	38	16	3	1	0	0	0	8	0	0	98	49	1	1	0.95	1	1	NA	1	-0.017
SE	16085	EIF4G1	EIF4G1	chr3	+	184315488	184315545	184314494	184314674	184315762	184315856	4	0	0	1	1	0	0	2	0	1	98	49	1	1	0.667	0	NA	NA	0.5	-0.166
SE	16101	XRN2	XRN2	chr20	+	21328558	21328670	21326489	21326601	21330480	21330539	60	20	9	2	0	0	0	13	0	0	98	49	1	1	0.937	1	1	NA	1	-0.021
SE	16102	XRN2	XRN2	chr20	+	21356585	21356665	21356079	21356177	21357735	21357792	16	9	10	0	0	0	0	12	0	2	98	49	1	1	1	1	1	NA	0.75	0.25
SE	16103	XRN2	XRN2	chr20	+	21357735	21357792	21356585	21356665	21365420	21365489	30	9	7	0	0	0	0	9	0	1	98	49	1	1	1	1	1	NA	0.818	0.182
SE	16106	STK17B	STK17B	chr2	-	196139619	196139799	196133582	196137729	196141248	196141297	171	95	23	0	2	0	1	70	0	0	98	49	1	1	1	0.96	1	1	1	-0.013
SE	16107	STK17B	STK17B	chr2	-	196163261	196163427	196156438	196156651	196171332	196171612	408	196	43	0	1	0	21	136	0	0	98	49	1	1	1	0.99	1	1	1	-0.003
SE	16109	HSPA9	HSPA9	chr5	-	138566625	138566718	138561579	138561789	138567000	138567163	7	6	0	2	0	0	0	2	0	0	98	49	1	1	0.636	1	NA	NA	1	-0.182
SE	16113	GGA3	GGA3	chr17	-	75244618	75244717	75243446	75243570	75246508	75246584	9	0	10	4	0	1	0	0	0	1	98	49	1	1	0.529	NA	0.833	NA	0	0.681
SE	16116	SIRPA	SIRPA	chr20	+	1922312	1922645	1921394	1921712	1924763	1924877	48	17	9	2	0	0	3	7	0	0	98	49	1	1	0.923	1	1	1	1	-0.026
SE	16118	LPP	LPP	chr3	+	188341662	188341719	188225404	188225527	188406111	188406313	21	4	0	0	0	0	0	0	0	1	98	49	1	1	1	1	NA	NA	0	1
SE	16133	IL1RAP	IL1RAP	chr3	+	190550341	190550505	190514050	190514219	190556129	190556216	3	0	0	11	8	0	0	0	3	3	98	49	1	1	0.12	0	NA	0	0	0.06
SE	16134	IL1RAP	IL1RAP	chr3	+	190550341	190550505	190514050	190514219	190564288	190564353	0	1	0	5	1	0	0	0	0	1	98	49	1	1	0	0.333	NA	NA	0	0.167
SE	16136	IL1RAP	IL1RAP	chr3	+	190556129	190556216	190550341	190550505	190564288	190564353	14	12	1	0	1	0	0	4	0	0	98	49	1	1	1	0.857	1	NA	1	-0.048
SE	16137	IL1RAP	IL1RAP	chr3	+	190564288	190564353	190514050	190514219	190604127	190604413	39	17	10	1	0	0	2	14	0	0	98	49	1	1	0.951	1	1	1	1	-0.016
SE	16138	IL1RAP	IL1RAP	chr3	+	190608994	190609181	190604127	190604413	190620274	190620440	40	14	22	0	0	2	0	8	0	0	98	49	1	1	1	1	0.846	NA	1	-0.051
SE	16139	IL1RAP	IL1RAP	chr3	+	190620274	190620440	190604127	190604413	190623343	190623415	16	23	21	1	0	0	0	4	0	0	98	49	1	1	0.889	1	1	NA	1	-0.037
SE	16141	KDM5C	KDM5C	chrX	-	53198489	53198637	53197770	53197876	53198763	53198888	15	7	5	1	0	0	1	3	0	0	98	49	1	1	0.882	1	1	1	1	-0.039
SE	16142	KDM5C	KDM5C	chrX	-	53210675	53210857	53210413	53210576	53211496	53211655	34	10	2	1	1	0	0	10	0	0	98	49	1	1	0.944	0.833	1	NA	1	-0.074
SE	16149	TNK2	TNK2	chr3	-	195882050	195882328	195879048	195879175	195883156	195883309	12	9	2	2	0	0	0	3	0	1	98	49	1	1	0.75	1	1	NA	0.6	0.317
SE	16152	PCYT1A	PCYT1A	chr3	-	196283474	196283538	196270414	196270541	196287614	196287713	1	0	0	1	0	0	0	1	0	1	98	49	1	1	0.333	NA	NA	NA	0.333	0
SE	16154	CEP19	CEP19	chr3	-	196708527	196708727	196706276	196707912	196711928	196712294	7	2	8	1	0	0	0	2	0	0	98	49	1	1	0.778	1	1	NA	1	-0.074
SE	16155	SMARCA2	SMARCA2	chr9	+	2115821	2116049	2110253	2110417	2119457	2119535	26	4	4	0	1	0	0	3	0	0	98	49	1	1	1	0.667	1	NA	1	-0.111
SE	16156	SMARCA2	SMARCA2	chr9	+	2170418	2170472	2161685	2161903	2181570	2181676	6	2	1	6	1	4	0	2	0	0	98	49	1	1	0.333	0.5	0.111	NA	1	-0.685
SE	16165	KIAA0226	KIAA0226	chr3	-	197699186	197699231	197696953	197697049	197700612	197701146	1	0	0	2	0	0	0	1	0	2	94	49	1	1	0.207	NA	NA	NA	0.207	0
SE	16166	KIAA0226	KIAA0226	chr3	-	197717976	197718130	197705091	197705175	197736654	197736926	3	0	3	1	0	0	0	2	1	0	98	49	1	1	0.6	NA	1	0	1	0.3
SE	16167	LRCH3	LRCH3	chr3	+	197858833	197858905	197854391	197854445	197865422	197865471	3	1	0	1	0	0	0	3	0	1	98	49	1	1	0.6	1	NA	NA	0.6	0.2
SE	16168	AFF1	AFF1	chr4	+	87046165	87046286	87007000	87007424	87046694	87047594	28	8	7	1	1	0	2	9	0	0	98	49	1	1	0.933	0.8	1	1	1	-0.089
SE	16169	AFF1	AFF1	chr4	+	87089983	87090070	87084119	87084164	87091792	87091829	23	7	2	0	0	0	1	12	0	1	98	49	1	1	1	1	1	1	0.857	0.072
SE	16170	AFF1	AFF1	chr4	+	87132270	87132408	87131792	87131864	87134470	87134694	5	9	1	0	5	0	0	3	0	0	98	49	1	1	1	0.474	1	NA	1	-0.175
SE	16174	HLA-C	HLA-C	chr6	-	31269965	31270085	31269492	31269525	31270209	31270485	734	697	301	0	1	0	48	112	0	0	98	49	1	1	1	0.997	1	1	1	-0.001
SE	16177	TMEM175	TMEM175	chr4	+	948410	948615	948115	948154	950420	950518	4	0	2	0	0	0	0	0	0	1	98	49	1	1	1	NA	1	NA	0	1
SE	16181	DUSP22	DUSP22	chr6	+	335113	335163	311879	311962	345853	345928	11	7	1	0	0	0	0	2	0	1	98	49	1	1	1	1	1	NA	0.5	0.5
SE	16185	SLC25A13	SLC25A13	chr7	-	96189580	96189674	96189293	96189378	96191108	96191247	6	2	0	0	0	0	0	1	0	1	98	49	1	1	1	1	NA	NA	0.333	0.667
SE	16190	HTT	HTT	chr4	+	3140509	3140656	3136225	3136326	3142765	3142886	8	0	0	1	0	0	0	4	0	0	98	49	1	1	0.8	NA	NA	NA	1	-0.2
SE	16200	SH3TC1	SH3TC1	chr4	+	8216956	8217167	8216110	8216257	8218270	8218347	3	0	0	0	0	0	0	1	0	5	98	49	1	1	1	NA	NA	NA	0.091	0.909
SE	16201	SH3TC1	SH3TC1	chr4	+	8236277	8236428	8235432	8235555	8237473	8237670	2	0	0	3	0	0	0	1	0	0	98	49	1	1	0.25	NA	NA	NA	1	-0.75
SE	16203	DMXL1	DMXL1	chr5	+	119110150	119110283	119105179	119105258	119114474	119114541	10	1	2	2	0	0	0	1	0	0	98	49	1	1	0.714	1	1	NA	1	-0.095
SE	16204	DMXL1	DMXL1	chr5	+	119110154	119110283	119105179	119105258	119114474	119114541	6	1	2	2	0	0	0	1	0	0	98	49	1	1	0.6	1	1	NA	1	-0.133
SE	16206	DMXL1	DMXL1	chr5	+	119238988	119239080	119237321	119237414	119240418	119240471	3	0	0	0	0	2	0	1	0	0	98	49	1	1	1	NA	0	NA	1	-0.5
SE	16207	NIPBL	NIPBL	chr5	+	37000372	37000570	36995621	36995804	37000816	37000888	26	13	1	1	0	0	0	1	0	1	98	49	1	1	0.929	1	1	NA	0.333	0.643
SE	16209	NIPBL	NIPBL	chr5	+	37060843	37061018	37052365	37052566	37063789	37063978	4	2	1	0	1	0	0	1	0	0	98	49	1	1	1	0.5	1	NA	1	-0.167
SE	16210	ATG2B	ATG2B	chr14	-	96322111	96322254	96317697	96317855	96322539	96322735	3	5	2	0	1	0	0	0	1	0	98	49	1	1	1	0.714	1	0	NA	0.905
SE	16212	NSD1	NSD1	chr5	+	177269601	177269807	177267561	177267718	177273671	177273784	14	4	1	1	0	0	0	1	0	0	98	49	1	1	0.875	1	1	NA	1	-0.042
SE	16213	NSD1	NSD1	chr5	+	177282464	177282581	177280564	177280834	177283786	177283928	9	0	5	1	0	0	0	1	0	0	98	49	1	1	0.818	NA	1	NA	1	-0.091
SE	16215	WDR1	WDR1	chr4	-	10077752	10077926	10077303	10077448	10078890	10079001	83	27	26	2	0	0	12	27	0	0	98	49	1	1	0.954	1	1	1	1	-0.015
SE	16217	WDR1	WDR1	chr4	-	10088663	10088741	10088292	10088373	10097710	10097891	55	15	17	2	0	0	3	4	0	0	98	49	1	1	0.932	1	1	1	1	-0.023
SE	16218	WDR1	WDR1	chr4	-	10097710	10097891	10088663	10088741	10116112	10116234	37	7	7	2	0	0	2	3	0	0	98	49	1	1	0.902	1	1	1	1	-0.033
SE	16219	WDR1	WDR1	chr4	-	10098991	10099139	10097710	10097891	10116112	10116234	35	23	17	2	0	0	1	6	0	0	98	49	1	1	0.897	1	1	1	1	-0.034
SE	16220	WDR1	WDR1	chr4	-	10103895	10103986	10088663	10088741	10116112	10116234	36	16	13	2	0	0	4	6	0	0	98	49	1	1	0.9	1	1	1	1	-0.033
SE	16221	WDR1	WDR1	chr4	-	10103895	10103986	10098991	10099139	10116112	10116234	64	30	25	0	0	0	5	16	0	1	98	49	1	1	1	1	1	1	0.889	0.055
SE	16222	SHTN1	SHTN1	chr10	-	116929848	116930002	116927791	116927891	116940465	116940612	6	0	0	1	0	0	0	4	0	0	98	49	1	1	0.75	NA	NA	NA	1	-0.25
SE	16228	BOD1L1	BOD1L1	chr4	-	13595859	13595944	13591922	13591966	13597103	13597168	43	7	2	0	2	0	1	12	0	0	98	49	1	1	1	0.636	1	1	1	-0.121
SE	16229	BOD1L1	BOD1L1	chr4	-	13610933	13611100	13609294	13609406	13613511	13613661	86	25	8	1	0	0	2	9	0	0	98	49	1	1	0.977	1	1	1	1	-0.008
SE	16232	FBXL5	FBXL5	chr4	-	15638507	15638694	15636493	15636676	15640787	15640883	366	142	81	3	0	0	3	71	0	0	98	49	1	1	0.984	1	1	1	1	-0.005
SE	16233	FBXL5	FBXL5	chr4	-	15641523	15641627	15640787	15640883	15644492	15644708	5	5	2	96	46	54	0	1	0	31	98	49	1	1	0.025	0.052	0.018	NA	0.016	0.016
SE	16234	FBXL5	FBXL5	chr4	-	15644492	15644657	15640787	15640883	15655203	15655412	97	48	54	0	2	0	0	31	0	0	98	49	1	1	1	0.923	1	NA	1	-0.026
SE	16235	FBXL5	FBXL5	chr4	-	15644492	15644708	15640787	15640883	15655203	15655412	108	49	63	0	2	0	1	42	0	0	98	49	1	1	1	0.925	1	1	1	-0.025
SE	16236	HLA-DRB1	HLA-DRB1	chr6	-	32581556	32581838	32580745	32580856	32584108	32584170	88	0	4	0	2	0	0	14	0	0	98	49	1	1	1	0	1	NA	1	-0.333
SE	16238	BST1	BST1	chr4	+	15715706	15715799	15715284	15715361	15718906	15718993	55	35	29	0	2	0	2	21	0	0	98	49	1	1	1	0.897	1	1	1	-0.034
SE	16244	TAPT1	TAPT1	chr4	-	16213767	16213898	16191360	16191523	16226258	16226538	2	2	0	1	0	0	0	1	0	0	98	49	1	1	0.5	1	NA	NA	1	-0.25
SE	16249	DHX15	DHX15	chr4	-	24576242	24576678	24570653	24570847	24584322	24584561	10	5	8	0	1	0	0	1	0	0	98	49	1	1	1	0.714	1	NA	1	-0.095
SE	16251	PI4K2B	PI4K2B	chr4	+	25252320	25252475	25234030	25234431	25255064	25255265	2	0	0	1	0	0	0	1	0	0	98	49	1	1	0.5	NA	NA	NA	1	-0.5
SE	16260	ARAP2	ARAP2	chr4	-	36177826	36178005	36166931	36167047	36187450	36187571	10	3	1	1	0	0	1	2	0	0	98	49	1	1	0.833	1	1	1	1	-0.056
SE	16261	ARAP2	ARAP2	chr4	-	36212395	36212487	36210389	36210743	36213242	36213319	16	6	15	0	0	0	0	3	0	2	98	49	1	1	1	1	1	NA	0.429	0.571
SE	16262	PGM2	PGM2	chr4	+	37844363	37844553	37840065	37840259	37845632	37845730	14	3	1	1	4	0	0	6	0	0	98	49	1	1	0.875	0.273	1	NA	1	-0.284
SE	16263	PGM2	PGM2	chr4	+	37848521	37848651	37847201	37847295	37850183	37850373	12	21	7	1	0	0	2	3	0	0	98	49	1	1	0.857	1	1	1	1	-0.048
SE	16264	PGM2	PGM2	chr4	+	37850183	37850373	37848521	37848651	37855607	37855741	28	13	6	1	0	0	2	6	0	0	98	49	1	1	0.933	1	1	1	1	-0.022
SE	16265	TBC1D1	TBC1D1	chr4	+	38118032	38118192	38115709	38115954	38124961	38125131	18	6	4	0	0	1	4	10	0	0	98	49	1	1	1	1	0.667	1	1	-0.111
SE	16275	RIPK1	RIPK1	chr6	+	3085258	3085408	3083084	3083313	3089580	3089657	15	5	9	0	0	0	0	0	0	1	98	49	1	1	1	1	1	NA	0	1
SE	16283	SPTAN1	SPTAN1	chr9	+	128609650	128609665	128609121	128609284	128611713	128611845	2	2	0	4	1	0	0	0	0	2	64	49	1	1	0.277	0.605	NA	NA	0	0.441
SE	16292	DDX46	DDX46	chr5	+	134782944	134783065	134781920	134782086	134784365	134784541	11	3	3	1	0	0	2	3	0	0	98	49	1	1	0.846	1	1	1	1	-0.051
SE	16298	EXOC1	EXOC1	chr4	+	55871848	55871958	55871100	55871233	55877916	55878066	8	3	4	0	0	0	0	2	0	2	98	49	1	1	1	1	1	NA	0.333	0.667
SE	16300	EXOC1	EXOC1	chr4	+	55891314	55891422	55890222	55890386	55892634	55892711	16	5	2	1	0	0	0	3	0	0	98	49	1	1	0.889	1	1	NA	1	-0.037
SE	16301	EXOC1	EXOC1	chr4	+	55893551	55893780	55892634	55892711	55896716	55896900	9	4	3	0	0	0	0	7	0	1	98	49	1	1	1	1	1	NA	0.778	0.222
SE	16311	YTHDC1	YTHDC1	chr4	-	68330201	68330310	68330001	68330119	68332102	68332197	27	18	4	0	1	0	0	1	0	0	98	49	1	1	1	0.9	1	NA	1	-0.033
SE	16312	YTHDC1	YTHDC1	chr4	-	68332793	68332847	68332102	68332197	68333307	68333397	39	26	3	3	0	0	0	2	0	0	98	49	1	1	0.867	1	1	NA	1	-0.044
SE	16314	PARN	PARN	chr16	-	14627105	14627187	14617589	14617650	14628171	14628251	8	4	0	3	0	0	0	1	0	0	98	49	1	1	0.571	1	NA	NA	1	-0.215
SE	16315	PARN	PARN	chr16	-	14627268	14627336	14627105	14627187	14628171	14628251	8	9	2	0	0	0	0	4	0	1	98	49	1	1	1	1	1	NA	0.667	0.333
SE	16333	SCARB2	SCARB2	chr4	-	76163224	76163383	76158738	76161751	76166249	76166301	3	0	0	1	0	0	0	2	0	0	98	49	1	1	0.6	NA	NA	NA	1	-0.4
SE	16334	SCARB2	SCARB2	chr4	-	76179516	76179705	76176436	76176528	76180953	76181101	1	0	0	0	1	0	0	8	0	0	98	49	1	1	1	0	NA	NA	1	-0.5
SE	16341	BCAP31	BCAP31	chrX	-	153702934	153703058	153702006	153702107	153703958	153704094	37	21	11	0	0	0	0	11	0	1	98	49	1	1	1	1	1	NA	0.846	0.154
SE	16343	BMP2K	BMP2K	chr4	+	78847187	78847269	78844927	78845049	78850923	78851056	15	5	5	1	0	0	0	8	0	0	98	49	1	1	0.882	1	1	NA	1	-0.039
SE	16344	BMP2K	BMP2K	chr4	+	78872613	78872798	78871849	78871948	78878733	78878891	19	3	4	1	0	0	0	5	0	0	98	49	1	1	0.905	1	1	NA	1	-0.032

SE	16345 BMP2K	BMP2K	chr4	+	78887173	78887284	78878733	78878891	78910609	78912187	8	0	1	1	0	0	0	1	0	0	98	49	1	1	0.8	NA	1	NA		1	-0.1
SE	16346 SNX10	SNX10	chr7	+	26357006	26357127	26346419	26346466	26360974	26361061	2	0	0	9	3	5	0	3	0	5	98	49	1	1	0.1	0	0	NA		0.231	-0.198
SE	16347 TRABD	TRABD	chr22	+	50197240	50197351	50194899	50195040	50197448	50197588	39	12	10	0	1	0	0	3	0	0	98	49	1	1	1	0.857	1	NA		1	-0.048
SE	16348 ANTXR2	ANTXR2	chr4	-	80033471	80033570	80031622	80031692	80054271	80054352	46	13	12	0	0	0	0	4	0	1	98	49	1	1	1	1	1	NA		0.667	0.333
SE	16349 ANTXR2	ANTXR2	chr4	-	80035971	80036032	80033471	80033570	80054271	80054352	65	9	11	1	0	0	0	6	0	0	98	49	1	1	0.97	1	1	NA		1	-0.01
SE	16351 HNRNPD	HNRNPD	chr4	-	82356536	82356683	82355303	82355401	82356795	82356895	10	11	0	6	6	2	0	1	0	0	98	49	1	1	0.455	0.478	0	NA		1	-0.689
SE	16356 SEC31A	SEC31A	chr4	-	82848803	82848977	82844385	82844509	82851430	82851604	14	3	2	0	0	0	0	3	0	1	98	49	1	1	1	1	1	NA		0.6	0.4
SE	16361 THAP9-AS1	THAP9-AS1	chr4	-	82899712	82899737	82897988	82898062	82900076	82900223	11	7	0	3	0	0	0	3	0	0	74	49	1	1	0.708	1	NA			1	-0.146
SE	16362 EDEM1	EDEM1	chr3	+	5205066	5205241	5202965	5203149	5207152	5207273	11	11	0	1	0	0	0	2	0	0	98	49	1	1	0.846	1	NA	NA		1	-0.077
SE	16363 EDEM1	EDEM1	chr3	+	5210174	5210248	5208092	5208263	5211119	5211216	10	14	0	4	0	0	0	3	0	0	98	49	1	1	0.556	1	NA	NA		1	-0.222
SE	16364 OGT	OGT	chrX	+	71544566	71544635	71537828	71538072	71547906	71548023	8	4	3	0	0	4	0	3	0	0	98	49	1	1	1	1	0.273	NA		1	-0.242
SE	16365 OGT	OGT	chrX	+	71555189	71555385	71554512	71554592	71555953	71556094	32	7	4	8	7	0	0	2	0	0	98	49	1	1	0.667	0.333	1	NA		1	-0.333
SE	16374 PDLIM7	PDLIM7	chr5	-	177489392	177489627	177488067	177488248	177489770	177489832	37	17	12	0	0	0	3	11	0	1	98	49	1	1	1	1	1	1		0.846	0.077
SE	16375 PDLIM7	PDLIM7	chr5	-	177491403	177491420	177491009	177491146	177491806	177491925	3	0	0	8	5	6	0	0	0	4	66	49	1	1	0.218	0	0	NA		0	0.073
SE	16390 HERC5	HERC5	chr4	+	88463854	88463985	88463531	88463623	88467058	88467204	18	20	0	1	0	0	2	27	0	1	98	49	1	1	0.9	1	NA	1		0.931	-0.016
SE	16391 HERC5	HERC5	chr4	+	88470613	88470673	88469156	88469260	88472408	88472502	13	26	2	0	2	0	2	23	0	1	98	49	1	1	1	0.867	1	1		0.92	-0.004
SE	16392 HERC5	HERC5	chr4	+	88472408	88472502	88470613	88470673	88475840	88476030	11	17	2	1	0	0	2	29	0	0	98	49	1	1	0.846	1	1	1		1	-0.051
SE	16393 HERC5	HERC5	chr4	+	88486114	88486228	88479352	88479507	88489165	88489336	6	21	0	1	0	0	0	8	0	1	98	49	1	1	0.75	1	NA	NA		0.8	0.075
SE	16394 HERC5	HERC5	chr4	+	88487068	88487179	88486114	88486228	88489165	88489336	3	17	1	0	0	0	1	19	0	1	98	49	1	1	1	1	1	1		0.905	0.047
SE	16395 HERC5	HERC5	chr4	+	88489165	88489336	88479352	88479507	88493011	88493155	14	15	1	0	1	0	1	15	0	0	98	49	1	1	1	0.882	1	1		1	-0.039
SE	16396 HERC5	HERC5	chr4	+	88499925	88499992	88494164	88494331	88500914	88500985	15	27	0	0	0	0	3	48	1	1	98	49	1	1	1	1	NA	0.6		0.96	0.22
SE	16422 NFKB1	NFKB1	chr4	+	102578880	102579039	102576875	102577039	102580534	102580639	15	2	5	1	0	0	0	4	0	0	98	49	1	1	0.882	1	1	NA		1	-0.039
SE	16423 MANBA	MANBA	chr4	-	102635864	102636007	102634787	102635045	102639712	102639857	12	7	1	1	0	0	1	2	0	0	98	49	1	1	0.857	1	1	1		1	-0.048
SE	16424 MANBA	MANBA	chr4	-	102650536	102650701	102639712	102639857	102657681	102657900	17	2	6	0	0	0	0	4	0	1	98	49	1	1	1	1	1	NA		0.667	0.333
SE	16427 PELI1	PELI1	chr2	-	64100397	64100499	64096412	64096610	64108239	64108379	129	27	17	0	1	0	2	12	0	0	98	49	1	1	1	0.931	1	1		1	-0.023
SE	16428 PELI1	PELI1	chr2	-	64104700	64104830	64100397	64100499	64108239	64108379	77	46	44	0	2	0	0	13	0	0	98	49	1	1	1	0.92	1	NA		1	-0.027
SE	16433 TET2	TET2	chr4	+	105190359	105190505	105146684	105146979	105233896	105237351	65	21	15	5	2	0	0	6	0	0	98	49	1	1	0.867	0.84	1	NA		1	-0.098
SE	16434 TET2	TET2	chr4	+	105241338	105241429	105233896	105237351	105242833	105242927	32	8	0	0	0	0	0	6	0	2	98	49	1	1	1	1	NA	NA		0.6	0.4
SE	16435 TET2	TET2	chr4	+	105243569	105243778	105242833	105242927	105259618	105259769	26	17	12	7	0	0	0	3	0	0	98	49	1	1	0.65	1	1	NA		1	-0.117
SE	16436 RAP1GAP2	RAP1GAP2	chr17	+	2962669	2962714	2957758	2957794	2963429	2963462	8	8	1	10	11	3	0	3	0	6	94	49	1	1	0.294	0.275	0.148	NA		0.207	0.032
SE	16437 ZFR	ZFR	chr5	-	32380074	32380172	32379114	32379210	32385507	32385649	17	2	1	0	0	0	0	4	0	2	98	49	1	1	1	1	1	NA		0.5	0.5
SE	16443 CNN2	CNN2	chr19	+	1032558	1032696	1032391	1032458	1036415	1036562	72	35	36	2	0	0	1	17	0	0	98	49	1	1	0.947	1	1	1		1	-0.018
SE	16444 CNN2	CNN2	chr19	+	1036066	1036246	1032558	1032696	1036415	1036562	55	42	41	3	3	1	0	15	0	1	98	49	1	1	0.902	0.875	0.953	NA		0.882	0.028
SE	16454 KDM5A	KDM5A	chr12	-	297040	297200	295572	295793	306945	307089	40	16	9	1	0	0	0	4	0	0	98	49	1	1	0.952	1	1	NA		1	-0.016
SE	16455 KDM5A	KDM5A	chr12	-	306945	307089	297040	297200	307453	308005	43	15	10	0	0	0	0	1	0	1	98	49	1	1	1	1	1	NA		0.333	0.667
SE	16456 KDM5A	KDM5A	chr12	-	333486	333649	331818	331938	334240	334422	46	23	6	0	1	0	0	8	0	0	98	49	1	1	1	0.92	1	NA		1	-0.027
SE	16457 KDM5A	KDM5A	chr12	-	354075	354234	352204	352324	355157	355249	41	12	13	1	0	0	3	7	0	0	98	49	1	1	0.953	1	1	1		1	-0.016
SE	16462 LOC645513	LOC645513	chr4	+	119497810	119497903	119494432	119494523	119499560	119499592	9	0	6	2	0	0	0	0	0	0	98	49	1	1	0.692	NA	1	NA		1	-0.154
SE	16470 JADE1	JADE1	chr4	+	128867855	128867973	128861703	128862225	128871354	128875224	5	0	8	1	0	0	0	2	0	0	98	49	1	1	0.714	NA	1	NA		1	-0.143
SE	16471 SCLT1	SCLT1	chr4	-	128942995	128943188	128936654	128936851	128946006	128946152	11	9	3	4	1	0	0	0	0	2	98	49	1	1	0.579	0.818	1	NA		0	0.799
SE	16472 SCLT1	SCLT1	chr4	-	128965226	128965318	128959599	128959777	128970377	128970468	20	15	6	3	3	3	0	3	0	1	98	49	1	1	0.769	0.714	0.5	NA		0.6	0.061
SE	16476 ELF2	ELF2	chr4	-	139061864	139062057	139060323	139060674	139067683	139067770	52	21	3	2	0	0	0	7	0	0	98	49	1	1	0.929	1	1	NA		1	-0.024
SE	16478 ELF2	ELF2	chr4	-	139137629	139137867	139125163	139125329	139139412	139139476	63	35	7	3	0	0	0	10	0	0	98	49	1	1	0.913	1	1	NA		1	-0.029
SE	16482 IGF1R	IGF1R	chr15	+	98891324	98891637	98707561	98708107	98896756	98896905	32	7	1	1	0	0	0	1	0	0	98	49	1	1	0.941	1	1	NA		1	-0.02
SE	16484 IGF1R	IGF1R	chr15	+	98934823	98935053	98930234	98930305	98935315	98935426	25	11	3	1	1	0	0	2	0	0	98	49	1	1	0.926	0.846	1	NA		1	-0.076
SE	16492 GAB1	GAB1	chr4	+	143457679	143457769	143440078	143440382	143459384	143459478	1	0	0	2	0	0	0	0	0	3	98	49	1	1	0.2	NA	NA	NA		0	0.2
SE	16509 FBXW7	FBXW7	chr4	-	152412479	152412529	152411159	152411872	152534940	152535033	17	8	0	3	0	0	0	0	2	0	98	49	1	1	0.739	1	NA	0	NA		0.869
SE	16510 CIDEB	CIDEB	chr14	-	24305946	24306137	24305186	24305765	24306373	24306523	4	0	2	0	0	0	0	3	0	2	98	49	1	1	1	NA	1	NA		0.429	0.571
SE	16515 KIAA0922	KIAA0922	chr4	+	153587741	153587811	153586208	153586379	153588889	153589007	22	4	3	0	2	0	0	3	0	2	98	49	1	1	1	0.5	1	NA		0.429	0.404
SE	16516 KIAA0922	KIAA0922	chr4	+	153588889	153589007	153586208	153586379	153593798	153593871	2	2	0	1	0	0	0	2	0	0	98	49	1	1	0.5	1	NA	NA		1	-0.25
SE	16517 KIAA0922	KIAA0922	chr4	+	153591052	153591194	153588889	153589007	153592474	153592584	30	15	2	1	2	0	0	6	0	0	98	49	1	1	0.937	0.789	1	NA		1	-0.091
SE	16518 KIAA0922	KIAA0922	chr4	+	153591052	153591194	153588889	153589007	153593798	153593871	19	14	1	2	0	0	0	3	0	0	98	49	1	1	0.826	1	1	NA		1	-0.058
SE	16519 KIAA0922	KIAA0922	chr4	+	153592474	153592584	153588889	153589007	153593798	153593871	9	13	0	2	0	0	0	4	0	0	98	49	1	1	0.692	1	NA	NA		1	-0.154
SE	16520 KIAA0922	KIAA0922	chr4	+	153592474	153592584	153591052	153591194	153593798	153593871	23	12	1	4	0	0	0	7	0	0	98	49	1	1	0.742	1	1	NA		1	-0.086
SE	16521 KIAA0922	KIAA0922	chr4	+	153632717	153632838	153627604	153627687	153634191	153634280	11	3	8	1	0	0	0	1	0	0	98	49	1	1	0.846	1	1	NA		1	-0.051
SE	16522 PLRG1	PLRG1	chr4	-	154537285	154537479	154534996	154536744	154537968	154538108	5	4	0	1	0	0	0	2	0	0	98	49	1	1	0.714	1	NA	NA		1	-0.143
SE	16574 LILRA2	LILRA2	chr19	+	54574075	54574111	54573697	54573912	54574300	54574582	29	5	8	6	0	0	0	5	0	1	85	49	1	1	0.736	1	1	NA		0.742	0.17
SE	16575 LILRA2	LILRA2	chr19	+	54574300	54574582	54573830	54573912	54574730	54575033	54	25	20	1	0	0	1	22	0	0	98	49	1	1	0.964	1	1	1		1	-0.012
SE	16576 LILRA2	LILRA2	chr19	+	54575255	54575552	54574730	54575033	54575806	54576109	33	16	16	1	0	0	0	9	0	0	98	49	1	1	0.943	1	1	NA		1	-0.019
SE	16577 LILRA2	LILRA2	chr19	+	54577464	54577723	54575806	54576109	54587009	54587060	1	1	3	5	0	2	0	4	0	0	98	49	1	1	0.091	1	0.429	NA		1	-0.493
SE	16578 LILRA2	LILRA2	chr19	+	54577464	54577723	54575806	54576109	54587200	54587561	5	4	4	12	9	9	0	3	0	3	98	49	1	1	0.172	0.182	0.182	NA		0.333	-0.154
SE	16579 LILRA2	LILRA2	chr19	+	54587009	54587060	54575806	54576109	54587200	54587561	11	0	6	12	9	9	0	1	0	3	98	49	1	1	0.314	0	0.25	NA		0.143	0.045
SE	16580 LILRA2	LILRA2	chr19	+	54587009	54587060	54577464	54577723	54587200	54587561	6	0	4	4	3	1	0	2	0	0	98	49	1	1	0.429	0	0.667	NA		1	-0.635
SE	16583 IRF2	IRF2	chr4	-	184408157	184408275	184398914	184399079	184418166	184418213	47	15	19	1	0	0	0	8	0	0	98	49	1	1	0.959	1	1	NA		1	-0.014
SE	16584 IRF2	IRF2	chr4	-	184418166	184418213	184398914	184399079	184418531	184418708	71	9	27	1	0	0	2	7	0	0	96	49	1	1	0.973	1	1	1		1	-0.009
SE	16585 IRF2	IRF2	chr4	-	184419468	184419568	184418531	184418708	184428977	184429070	48	21	42	1	0	0	0	12	0	0	98	49	1	1	0.96	1	1	NA		1	-0.013
SE	16586 GPSM3	GPSM3	chr6	-	32191708	32191908	32190765	32191503	32192147	32192250	95	38	58	1	0	0	6	21	0	0	98	49	1	1	0.979	1	1	1		1	-0.007
SE	16587 GPSM3	GPSM3	chr6	-	32192147	32192250	32191708	32191908	32192471	32192530	64	29	41	0	1	0	4	8	0	0	98	49	1	1	1	0.935	1	1		1	-0.022
SE	16608 EXOC3	EXOC3	chr5	+	456888	457006	453369	454051	457899	458025	12	5	4	1	0	0	0	7	0	0	98	49	1	1	0.857	1	1	NA		1	-0.048
SE	16612 CPEB4	CPEB4	chr5	+	173932449	173932500	173910522	173910604	173944966	173945140	11	2	9	2	0	1	0	5	0	0	98	49	1	1	0.733	1	0.818	NA		1	-0.15
SE	16615 CPEB4	CPEB4	chr5	+	173944966	173945140	173932449	173932500	173949507	173949597	29	12	11	0	1	0	1	18	0	0	98	49	1	1	1	0.857	1	1		1	-0.048
SE	16619 IMPDH1	IMPDH1	chr7	-	128405766	128405865	128403705	128403754	128409755	128409982	18	10	5	2	0	0	0	5	0	0	98	49	1	1	0.818	1	1	NA		1	-0.061
SE	16620 IMPDH1	IMPDH1	chr7	-	128409288	128409366	128405766	128405865	128409755	128409982	2	3	0	12	6	0	0	0	0	4	98	49	1	1	0.077	0.2	NA	NA		0	0.139
SE	16625 RTN4	RTN4	chr2	-	54973820	54973867	54973562	54973621	54974694	54974764	38	8	8	0	3	0	0	9	0	0	96	49	1	1	1	0.576	1	NA		1	-0.141
SE	16629 MPP1	MPP1	chrX	-	154784027	154784108	154783426	154783507	154785050	154785157	32	10	10	1	0	0	0	10	0	0	98	49	1	1	0.941	1	1	NA		1	-0.02
SE	16630 MPP1	MPP1	chrX	-	154799744	154799893	154792141	154792285	154805271	154805527	0	0	0	10	5	5	0	1	0	6	98	49	1	1	0	0	0	NA		0.077	-0.077
SE	16632 HNRNPM	HNRNPM	chr19	+	8483157	8483211	8474166	8474244	8485602	8486405	9	3	0	2	0	0	0	2	0	0	98	49	1	1	0.692	1	NA	NA		1	-0.154
SE	16644 ITM2A	ITM2A	chrX	-	79363422	79363554	79362941	79363139	79367104	79367552	6	0	0	0	1	0	0	2	0	0	98	49	1	1	1	0	NA	NA		1	-0.5
SE	16650 FAM105A	FAM105A	chr5	+	14601350	14601442	14601212	14601244	14602182	14602332	18	4	0	1	0	0	2	3	0	2	98	49	1	1	0.9	1	NA	1		0.429	0.235
SE	16651 OTULIN	OTULIN	chr5	+	14673641	14673718	14664673	14664977	14678680	14678775	16	5	1	1	0	0	1	8	0	0	98	49	1	1	0.889	1	1	1		1	-0.037
SE	16665 PCBP1-AS1	PCBP1-AS1	chr2	-	70083537	70083687	70059642	70059680	70085546	70085624	6	0	2	2	3	0	0	1	0	0	98	49	1	1	0.6	0	1	NA		1	-0.467
SE	16669 ZNF335	ZNF335	chr20	-	45969450	45969691	45968284	45968362	45971209	45971460	9	2	1	0	0	0	0	0	0	1	98	49	1	1	1	1	1	NA		0	1
SE	16685 NUP155	NUP155	chr5	-	37307296	37307432	37305056	37305210	37309128	37309267	4	0	0	0	1	0	0	1	0	1	98	49	1	1	1	0	NA	NA		0.333	0.167
SE	16687 CFAP54	CFAP54	chr12	+	96535514	96535600	96534061	96534227	96538383	96538518	2	1	0	1	0	0	0	4	0	0	98	49	1	1	0.5	1	NA	NA		1	-0.25
SE	16689 RICTOR	RICTOR	chr5	-	38944445	38944569	38942832	38942971	38944912	38945068	105	34	10	1	1	0	0	15	0	0	98	49	1	1	0.981	0.944	1	NA		1	-0.025
SE	16690 RICTOR	RICTOR	chr5	-	38946467	38946552	38945490	38945610	38947263	38947441	70	29	10	0	2	0	0	9	0	0	98	49	1	1	1	0.879	1	NA		1	-0.04
SE	16691 RICTOR	RICTOR	chr5	-	38946467	38946552	38945490	38945724	38947263	38947441	71	26	13	3	0	0	0	9	0	0	98	49	1	1	0.922	1	1	NA		1	-0.026
SE	16692 RICTOR	RICTOR	chr5	-	38949355	38949427	38947263	38947441	38949711	38950720	18	20	2	37	23	1	0	0	0	11	98	49	1	1	0.196	0.303	0.5	NA		0	0.333
SE	16693 RICTOR	RICTOR	chr5	-	38975536	38975604	38971876	38971959	38978582	38978648	133	60	7	0	0	0	0	12	0	1	98	49	1	1	1	1	1	NA		0.857	0.143
SE	16694 RICTOR	RICTOR	chr5	-	38978582	38978648	38975536	38975604	38981866	38982036	100	32	6	0	1	0	0	7	0	0	98	49	1	1	1	0.941	1	NA		1	-0.02
SE	16695 RICTOR	RICTOR	chr5	-	38978582	38978650	38975536	38975604	38981866	38982036	155	57	25	0	1	0	0	18	0	0	98	49	1	1	1	0.966	1	NA		1	-0.011
SE	16696 RICTOR	RICTOR	chr5	-	39074110	39074158	39021038	39021136	39074328	39074408	21	14	0	1	0	0	1	0	0	0	97	49	1	1	0.914	1	NA	1	NA	1	-0.043
SE	16697 FYB	FYB	chr5	-	39125997	39126135	39124252	39124278	39127740	39127807	145	61	23	11	12	8	0	17	0	9	98	49	1	1	0.868	0.718	0.59	NA		0.486	0.239
SE	16698 FYB	FYB	chr5	-	39127740	39127807	39125997	39126135	39130589	39130612	167	59	24	1	0	0	0	25	0	0	98	49	1	1	0.988	1	1	NA		1	-0.004
SE	16699 FYB	FYB	chr5	-	39137599	39137720	39134854	39135014	39138656	39138691	105	57	24	2	0	0	0	20	0	0	98	49	1	1	0.963	1	1	NA		1	-0.012
SE	16700 FYB	FYB	chr5	-	39153447	39153604	39141094	39141141	39201825	39202987	189	65	25	1	0	0	7	35	0	0	98	49	1	1	0.99	1	1	1		1	-0.003
SE	16701 FYB	FYB	chr5	-	39201825	39202987	39153447	39153604	39219442	39219578	151	41	15	1	2	0	8	22	0	0	98	49	1	1	0.987	0.911	1	1		1	-0.034
SE	16702 MICAL1	MICAL1	chr6	-	109445196	109445290	109444895	109444995	109445415	109445529	30	16	9	2	0	0	1	9	0	0	98	49	1	1	0.882	1	1	1		1	-0.039
SE	16703 MICAL1	MICAL1	chr6	-	109447680	109447722	109447356	109447440	109448202	109448393	5	4	3	1	0	0	2	2	0	0	91	49	1	1	0.729	1	1	1		1	-0.09
SE	16705 MICAL1	MICAL1	chr6	-	109450299	109450557	109449969	109450085	109451599	109451700	6	9	1	1	0	0	2	4	0	1	98	49	1	1	0.75	1	1	1		0.667	0.083
SE	16716 HMGCS1	HMGCS1	chr5	-	43298008	43298134	43297001	43297166	43298517	43298975	3	0	2	0	1	0	0	2	0	0	98	49	1	1	1	0	1	NA		1	-0.333
SE	16725 SKIV2L	SKIV2L	chr6	+	31960431	31960546	31960219	31960337	31960665	31960740	17	9	2	1	0	2	0	1	0	0	98	49	1	1	0.895	1	0.333	NA		1	-0.257
SE	16726 SKIV2L	SKIV2L	chr6	+	31961195	31961386	31961040	31961094	31961546	31961675	15	7	5	0	3	0	0	0	0	1	98	49	1	1	1	0.538	1	NA		0.333	0.513
SE	16727 SKIV2L	SKIV2L	chr6	+	31961908	31962054	31961546	31961675	31962438	31962585	16	9	0	0	0	0	1	3	0	0	98	49	1	1	1	0.818	NA	1		1	-0.091
SE	16728 SKIV2L	SKIV2L	chr6	+	31967003	31967141	31966708	31966846	31967272	31967377	22	4	1	1	0	0	0	4	0	0	98	49	1	1	0.917	1	1	NA		1	-0.028
SE	16730 FNBP1	FNBP1	chr9	-	129923843	129924026	129908889	129908999	129924959	129925157	1	1	0	18	1	0	0	0	0	1	98	49	1	1	0.027	0.333	NA	NA		0	0.18
SE	16731 FNBP1	FNBP1	chr9	-	129958450	129958553	129957359	129957464	129978464	129978612	73	11	13	2	0	0	1	8	0	0	98	49	1	1	0.948	1	1	1		1	-0.017
SE	16734 IL6ST	IL6ST	chr5	-	55959625	55959671	55957208	55957291	55960401	55960561	2	2	0	0	0	1	0	0	0	5	95	49	1	1	1	1	0	NA		0	0.667
SE	16736 SON	SON	chr21	+	33572550	33572630	33568970	33569087	33573307	33573455	5	0	0	8	0	5	0	0	0	1	98	49	1	1	0.238	NA	0	NA		0	0.119
SE	16740 C14orf159	C14orf159	chr14	+	91145220	91145313	91114012	91114283	91147780	91147858	26	4	0	1	0	0	0	1	0	0	98	49	1	1	0.929	1	NA	NA		1	-0.035
SE	16741 C14orf159	C14orf159	chr14	+	91145220	91145313	91114012	91114283	91157638	91157690	16	1	2	1	1	0	0	2	0	1	98	49	1	1	0.889	0.333	1	NA		0.5	0.241
SE	16743 C14orf159	C14orf159	chr14	+	91145220	91145313	91114634	91114994	91157638	91157690	35	8	5	22	12	0	0	4	0	5	98	49	1	1	0.443	0.25	1	NA		0.286	0.278
SE	16744 C14orf159	C14orf159	chr14	+	91147780	91147858	91114012	91114283	91157638	91157690	29	9	0	1	1	0	0	6	0	1	98	49	1	1	0.935	0.818	NA	NA		0.75	0.127
SE	16745 C14orf159	C14orf159	chr14	+	91147780	91147858	91145220	91145313	91157638	91157690	54	13	0	16	1	2	0	6	0	1	98	49	1	1	0.628	0.867	0	NA		0.75	-0.252
SE	16747 C14orf159	C14orf159	chr14	+	91170002	91170186	91167224	91167378	91173288	91173403	13	7	6	3	6	1	0	4	0	1	98	49	1	1	0.684	0.368	0.75	NA		0.667	-0.066
SE	16748 C14orf159	C14orf159	chr14	+	91170002	91170201	91167224	91167378	91173288	91173439	31	17	9	3	6	1	0	5	0	1	98	49	1	1	0.838	0.586	0.818	NA		0.714	0.033
SE	16749 C14orf159	C14orf159	chr14	+	91204705	91204825	91199756	91199905	91215404	91215556	94	17	19	8	11	0	0	17	0	0	98	49	1	1	0.855	0.436	1	NA		1	-0.236
SE	16761 SLC30A5	SLC30A5	chr5	+	69128003	69128132	69123198	69123425	69129446	69131072	6	0	2	1	0	0	0	1	0	0	98	49	1	1	0.75	NA	1	NA		1	-0.125
SE	16778 PYGM	PYGM	chr11	-	64751596	64751655	64751324	64751466	64751923	64752071	3	1	6	1	1	0	0	2	0	0	98	49	1	1	0.6	0.333	1	NA		1	-0.356
SE	16779 PYGM	PYGM	chr11	-	64758245	64758349	64757778	64757910	64758436	64758515	2	0	0	0	0	0	0	1	0	2	98	49	1	1	1	NA	NA	NA		0.2	0.8
SE	16787 MCCC2	MCCC2	chr5	+	71649096	71649253	71646210	71646277	71650068	71650183	0	2	0	0	1	0	0	3	0	0	98	49	1	1	NA	0.5	NA	NA		1	-0.5
SE	16793 ANKRA2	ANKRA2	chr5	-	73554860	73554986	73554321	73554388	73555487	73555585	5	3	0	0	1	0	0	0	0	3	98	49	1	1	1	0.6	NA	NA		0	0.8
SE	16795 GFM2	GFM2	chr5	-	74730259	74730398	74725940	74726126	74733021	74733098	0	0	0	4	0	0	0	1	0	0	98	49	1	1	0	NA	NA	NA		1	-1
SE	16797 GFM2	GFM2	chr5	-	74750578	74750667	74747691	74747780	74751367	74751493	9	0	2	1	0	0	0	1	0	0	98	49	1	1	0.818	NA	1	NA		1	-0.091
SE	16801 ZFAND5	ZFAND5	chr9	-	72359417	72359521	72356930	72357056	72360109	72360221	61	15	4	2	0	0	0	17	0	0	98	49	1	1	0.938	1	1	NA		1	-0.021
SE	16802 ZFAND5	ZFAND5	chr9	-	72360109	72360221	72359417	72359521	72360627	72360784	20	10	0	1	1	0	0	9	0	0	98	49	1	1	0.909	0.833	NA	NA		1	-0.129
SE	16806 POLK	POLK	chr5	+	75593877	75594049	75590343	75590440	75596221	75597178	5	0	0	9	0	0	0	2	0	0	98	49	1	1	0.217	NA	NA	NA		1	-0.783
SE	16809 IQGAP2	IQGAP2	chr5	+	76683775	76683917	76683114	76683217	76693354	76693442	40	9	8	1	2	0	0	6	0	0	98	49	1	1	0.952	0.692	1	NA		1	-0.119
SE	16812 ZFYVE16	ZFYVE16	chr5	+	80427491	80427545	80408012	80408153	80434108	80434217	3	5	0	4	0	0	0	2	0	0	98	49	1	1	0.273	1	NA	NA		1	-0.363
SE	16813 ZFYVE16	ZFYVE16	chr5	+	80448025	80448404	80445262	80445405	80449590	80449713	23	13	1	12	9	5	0	8	0	0	98	49	1	1	0.489	0.419	0.091	NA		1	-0.667
SE	16814 ZFYVE16	ZFYVE16	chr5	+	80472760	80472923	80459413	80459494	80473753	80473859	29	9	12	4	1	0	0	2	0	1	98	49	1	1	0.784	0.818	1	NA		0.5	0.367
SE	16815 AGTPBP1	AGTPBP1	chr9	-	85588297	85588478	85586830	85586960	85589527	85589681	55	32	22	4	2	0	0	12	0	1	98	49	1	1	0.873	0.889	1	NA		0.857	0.064
SE	16817 AGTPBP1	AGTPBP1	chr9	-	85692688	85692813	85681267	85681335	85741774	85742029	88	35	10	0	0	0	3	9	0	1	98	49	1	1	1	1	1	1		0.818	0.091
SE	16818 AGTPBP1	AGTPBP1	chr9	-	85712501	85712566	85692688	85692813	85741774	85742029	56	29	17	2	0	0	2	7	0	0	98	49	1	1	0.933	1	1	1		1	-0.022
SE	16824 SSBP2	SSBP2	chr5	-	81489249	81489309	81474495	81474562	81513627	81513717	9	2	3	3	0	0	0	0	0	1	98	49	1	1	0.6	1	1	NA		0	0.867
SE	16825 SSBP2	SSBP2	chr5	-	81513627	81513717	81489249	81489309	81615472	81615557	10	7	0	0	0	0	0	4	0	3	98	49	1	1	1	1	NA	NA		0.4	0.6
SE	16826 TMEM167A	TMEM167A	chr5	-	83061876	83061911	83052845	83057154	83077320	83077453	5	5	0	1	0	0	0	3	0	1	84	49	1	1	1	1	1	NA		0.636	0.364
SE	16827 TMEM167A	TMEM167A	chr5	-	83065007	83065117	83061876	83061911	83077320	83077453	7	5	0	1	0	0	0	3	0	0	98	49	1	1	0.778	1	NA	NA		1	-0.111
SE	16828 VCAN	VCAN	chr5	+	83519348	83522309	83512102	83512396	83537006	83542268	1	0	0	14	0	0	0	1	0	19	98	49	1	1	0.034	NA	NA	NA		0.026	0.008
SE	16829 VCAN	VCAN	chr5	+	83519348	83522309	83512102	83512396	83545536	83545650	1	0	0	1	0	0	0	1	0	1	98	49	1	1	0.333	NA	NA	NA		0.333	0
SE	16830 VCAN	VCAN	chr5	+	83537006	83542268	83512102	83512396	83545536	83545650	56	0	0	1	0	0	2	51	0	1	98	49	1	1	0.966	NA	NA	1		0.962	-0.015
SE	16832 DNAJC13	DNAJC13	chr3	+	132461049	132461205	132460249	132460357	132462466	132462523	9	2	0	2	0	0	1	2	0	0	98	49	1	1	0.692	1	NA	1		1	-0.154
SE	16841 FAM172A	FAM172A	chr5	-	93958776	93958968	93881469	93881688	93964457	93964523	17	8	0	0	0	0	0	5	0	2	98	49	1	1	1	1	NA	NA		0.556	0.444
SE	16842 FAM172A	FAM172A	chr5	-	94050730	94050831	93964457	93964523	94053125	94053227	11	6	1	2	0	0	0	3	0	1	98	49	1	1	0.733	1	1	NA		0.6	0.311
SE	16843 FAM172A	FAM172A	chr5	-	94074645	94074769	94053125	94053227	94111510	94111699	2	0	0	4	1	0	0	2	0	1	98	49	1	1	0.2	0	NA	NA		0.5	-0.4
SE	16844 KIAA0825	KIAA0825	chr5	-	94582432	94582583	94536995	94537127	94618499	94618604	9	3	2	1	0	0	0	1	0	0	98	49	1	1	0.818	1	1	NA		1	-0.061
SE	16845 SLF1	SLF1	chr5	+	94643272	94643435	94630502	94630743	94649453	94649597	0	2	1	0	2	0	0	1	0	0	98	49	1	1	NA	0.333	1	NA		1	-0.334
SE	16846 SLF1	SLF1	chr5	+	94653271	94653421	94651701	94651845	94654629	94654752	8	0	6	0	0	0	0	5	0	1	98	49	1	1	1	NA	1	NA		0.714	0.286
SE	16855 CAST	CAST	chr5	+	96730779	96730860	96729611	96729725	96736171	96736240	16	2	3	2	0	0	0	8	0	1	98	49	1	1	0.8	1	1	NA		0.8	0.133
SE	16857 CAST	CAST	chr5	+	96740744	96740783	96740037	96740118	96741265	96741358	14	3	0	10	3	3	0	8	0	0	88	49	1	1	0.438	0.358	0	NA		1	-0.735
SE	16858 CAST	CAST	chr5	+	96746341	96746425	96742654	96742756	96747344	96747392	25	2	4	2	0	0	0	11	0	0	98	49	1	1	0.862	1	1	NA		1	-0.046
SE	16868 ANKZF1	ANKZF1	chr2	+	219231927	219232040	219230227	219230405	219232259	219232362	10	5	5	1	0	2	1	0	0	0	98	49	1	1	0.833	1	0.556	1	NA		-0.204
SE	16872 APC	APC	chr5	+	112818965	112819344	112815494	112815593	112821895	112821991	14	14	7	0	2	0	0	4	0	0	98	49	1	1	1	0.778	1	NA		1	-0.074
SE	16873 APC	APC	chr5	+	112834950	112835165	112828855	112828972	112837552	112846239	17	13	4	1	0	2	0	1	0	0	98	49	1	1	0.895	1	0.5	NA		1	-0.202
SE	16879 ALPL	ALPL	chr1	+	21560625	21560745	21553977	21554142	21561096	21561212	95	52	20	1	0	0	3	11	0	0	98	49	1	1	0.979	1	1	1		1	-0.007

SE	16880	ALPL	ALPL	chr1	+	21560625	21560745	21553977	21554142	21563109	21563284	43	31	5	1	0	0	0	8	0	0	98	49	1	1	0.956	1	1	NA	1	-0.015
SE	16881	ALPL	ALPL	chr1	+	21561096	21561212	21553977	21554142	21563109	21563284	70	41	31	1	0	0	0	18	0	0	98	49	1	1	0.972	1	1	NA	1	-0.009
SE	16883	ALPL	ALPL	chr1	+	21573664	21573799	21570304	21570374	21575732	21575924	155	52	67	1	0	0	0	18	0	1	98	49	1	1	0.987	1	1	NA	0.9	0.096
SE	16890	HSD17B4	HSD17B4	chr5	+	119531265	119531404	119529893	119529980	119536422	119536550	2	0	0	1	0	0	0	3	0	0	98	49	1	1	0.5	NA	NA	NA	1	-0.5
SE	16891	ITPKC	ITPKC	chr19	+	40733159	40733364	40729201	40729415	40736985	40737087	1	0	0	2	0	0	0	1	0	0	98	49	1	1	0.2	NA	NA	NA	1	-0.8
SE	16894	VASP	VASP	chr19	+	45517928	45518094	45517662	45517834	45521321	45521406	200	80	69	1	0	0	7	36	0	0	98	49	1	1	0.99	1	1	1	1	-0.003
SE	16896	VASP	VASP	chr19	+	45522339	45522581	45517662	45517834	45522717	45522818	17	6	7	1	0	0	2	6	0	0	98	49	1	1	0.895	1	1	1	1	-0.035
SE	16906	LMNB1	LMNB1	chr5	+	126822780	126822885	126820909	126821135	126825987	126826107	126	35	30	0	2	0	2	18	0	0	98	49	1	1	1	0.897	1	1	1	-0.034
SE	16918	RAPGEF6	RAPGEF6	chr5	-	131561977	131562047	131548046	131548190	131592382	131592466	11	8	1	2	2	0	0	1	0	0	98	49	1	1	0.733	0.667	1	NA	1	-0.2
SE	16921	SLC22A4	SLC22A4	chr5	+	132312160	132312264	132294451	132295009	132313613	132313768	15	5	2	0	0	0	0	4	1	0	98	49	1	1	1	1	1	0	1	0.5
SE	16922	SLC22A4	SLC22A4	chr5	+	132313613	132313768	132294451	132295009	132327276	132327403	2	1	2	0	1	0	1	0	0	0	98	49	1	1	1	0.333	1	1	NA	-0.222
SE	16923	SLC22A4	SLC22A4	chr5	+	132322183	132322355	132313613	132313768	132327276	132327403	9	5	4	2	1	2	0	2	0	0	98	49	1	1	0.692	0.714	0.5	NA	1	-0.365
SE	16926	IRF1	IRF1	chr5	-	132484361	132484497	132481608	132484075	132485666	132485716	105	52	26	3	2	0	0	18	0	0	98	49	1	1	0.946	0.929	1	NA	1	-0.042
SE	16927	IRF1	IRF1	chr5	-	132484361	132484497	132481608	132484075	132486250	132486373	43	18	10	2	3	0	0	8	0	0	98	49	1	1	0.915	0.75	1	NA	1	-0.112
SE	16928	IRF1	IRF1	chr5	-	132484361	132484497	132481608	132484075	132486556	132486686	44	24	13	0	0	0	0	8	0	1	98	49	1	1	1	1	1	NA	0.8	0.2
SE	16929	IRF1	IRF1	chr5	-	132485666	132485716	132481608	132484075	132486250	132486373	53	34	26	2	3	0	0	24	0	0	98	49	1	1	0.93	0.85	1	NA	1	-0.073
SE	16930	IRF1	IRF1	chr5	-	132485666	132485716	132481608	132484075	132486556	132486686	11	4	0	0	0	0	0	1	0	1	98	49	1	1	1	1	NA	NA	0.333	0.667
SE	16931	IRF1	IRF1	chr5	-	132485666	132485716	132484361	132484497	132486250	132486373	114	66	42	2	0	0	0	34	0	0	98	49	1	1	0.966	1	1	NA	1	-0.011
SE	16932	IRF1	IRF1	chr5	-	132485666	132485716	132484361	132484497	132486556	132486686	72	36	16	3	6	3	0	11	0	0	98	49	1	1	0.923	0.75	0.727	NA	1	-0.2
SE	16933	IRF1	IRF1	chr5	-	132486250	132486373	132481608	132484075	132486556	132486686	65	21	29	0	0	0	2	12	0	1	98	49	1	1	1	1	1	1	0.857	0.072
SE	16937	IRF1	IRF1	chr5	-	132487925	132488025	132486953	132487130	132489391	132489483	85	62	26	0	3	0	7	14	0	1	98	49	1	1	1	0.912	1	1	0.875	0.033
SE	16939	IRF1	IRF1	chr5	-	132489391	132489483	132486953	132487130	132490544	132490773	43	25	13	18	5	5	0	6	0	2	98	49	1	1	0.544	0.714	0.565	NA	0.6	0.008
SE	16940	IRF1	IRF1	chr5	-	132489391	132489483	132487925	132488025	132490544	132490773	55	28	18	2	4	1	0	8	0	0	98	49	1	1	0.932	0.778	0.9	NA	1	-0.13
SE	16949	PPP2CA	PPP2CA	chr5	-	134200334	134200496	134199085	134199204	134200984	134201074	18	11	8	1	2	0	0	4	0	3	98	49	1	1	0.9	0.733	1	NA	0.4	0.478
SE	16956	H2AFY	H2AFY	chr5	-	135350822	135350913	135345967	135346057	135360496	135360607	5	3	3	3	0	1	0	3	0	0	98	49	1	1	0.455	1	0.6	NA	1	-0.315
SE	16963	FAM13B	FAM13B	chr5	-	137946227	137946311	137945901	137945997	137948954	137949184	9	7	4	1	4	0	0	5	0	0	98	49	1	1	0.818	0.467	1	NA	1	-0.238
SE	16964	FAM13B	FAM13B	chr5	-	137956476	137956542	137954165	137954376	137959615	137959763	0	0	0	6	0	0	0	1	0	0	98	49	1	1	0	NA	NA	NA	1	-1
SE	16965	FAM13B	FAM13B	chr5	-	138018954	138019146	138018301	138018514	138021030	138021197	4	5	0	2	1	0	0	1	0	0	98	49	1	1	0.5	0.714	NA	NA	1	-0.393
SE	16971	ETF1	ETF1	chr5	-	138529554	138529678	138518691	138518867	138542832	138542936	0	1	0	20	18	0	0	0	2	4	98	49	1	1	0	0.027	NA	0	0	0.013
SE	16972	SPI1	SPI1	chr11	-	47359852	47360040	47358843	47359006	47375632	47375729	283	126	83	0	0	2	14	70	0	0	98	49	1	1	1	1	0.954	1	1	-0.015
SE	16976	IK	IK	chr5	+	140655828	140655992	140654680	140654727	140657553	140657662	30	7	6	3	0	0	0	2	0	2	98	49	1	1	0.833	1	1	NA	0.333	0.611
SE	16979	CASC4	CASC4	chr15	+	44402886	44403054	44380805	44380976	44413335	44415761	23	5	14	8	1	2	0	1	0	0	98	49	1	1	0.59	0.714	0.778	NA	1	-0.306
SE	16982	HARS2	HARS2	chr5	+	140696102	140696201	140695737	140695845	140696520	140696614	7	2	1	1	0	0	0	1	0	0	98	49	1	1	0.778	1	1	NA	1	-0.074
SE	16986	HDAC3	HDAC3	chr5	-	141626012	141626071	141625684	141625764	141626193	141626283	16	1	2	0	0	0	0	6	0	2	98	49	1	1	1	1	1	NA	0.6	0.4
SE	16987	FCHSD1	FCHSD1	chr5	-	141643000	141643088	141641701	141641757	141644217	141644438	7	7	5	2	0	0	3	5	0	0	98	49	1	1	0.636	1	1	1	1	-0.121
SE	16988	FCHSD1	FCHSD1	chr5	-	141646602	141646722	141646086	141646191	141647134	141647230	6	4	3	0	0	0	0	1	0	1	98	49	1	1	1	1	1	NA	0.333	0.667
SE	16989	FCHSD1	FCHSD1	chr5	-	141649394	141649536	141649171	141649308	141649886	141649954	4	1	2	1	0	0	0	2	0	0	98	49	1	1	0.667	1	1	NA	1	-0.111
SE	16993	ARHGAP26	ARHGAP26	chr5	+	142873399	142873495	142770726	142770915	142879373	142879445	51	28	8	0	0	0	0	11	0	1	98	49	1	1	1	1	1	NA	0.846	0.154
SE	16994	ARHGAP26	ARHGAP26	chr5	+	142875109	142875171	142873399	142873495	142879373	142879445	232	139	55	2	0	0	5	59	0	0	98	49	1	1	0.983	1	1	1	1	-0.006
SE	16995	ARHGAP26	ARHGAP26	chr5	+	142894237	142894348	142885297	142885399	142903539	142903669	104	70	17	0	1	0	0	26	0	0	98	49	1	1	1	0.972	1	NA	1	-0.009
SE	16996	ARHGAP26	ARHGAP26	chr5	+	142901934	142902039	142894237	142894348	142903539	142903669	282	160	34	3	1	0	2	101	0	0	98	49	1	1	0.979	0.988	1	1	1	-0.011
SE	16997	ARHGAP26	ARHGAP26	chr5	+	143014079	143014116	142932046	142932125	143037195	143037261	231	77	21	1	0	0	1	47	0	0	86	49	1	1	0.992	1	1	1	1	-0.003
SE	16998	ARHGAP26	ARHGAP26	chr5	+	143037195	143037261	143014079	143014116	143041815	143041890	150	75	13	3	1	0	0	32	0	0	98	49	1	1	0.962	0.974	1	NA	1	-0.021
SE	16999	ARHGAP26	ARHGAP26	chr5	+	143207197	143207308	143147230	143147381	143213996	143214088	23	14	0	1	1	1	0	4	0	0	98	49	1	1	0.92	0.875	0	NA	1	-0.402
SE	17000	ARHGAP26	ARHGAP26	chr5	+	143207197	143207473	143147230	143147381	143213996	143214088	17	10	0	1	1	1	0	3	0	0	98	49	1	1	0.895	0.833	0	NA	1	-0.424
SE	17003	WDSUB1	WDSUB1	chr2	-	159248371	159248512	159235792	159236190	159256195	159256375	1	0	0	1	0	0	0	0	0	2	98	49	1	1	0.333	NA	NA	NA	0	0.333
SE	17008	ZNF438	ZNF438	chr10	-	30857679	30857778	30848530	30850367	30876997	30877065	2	5	0	6	3	0	0	0	0	2	98	49	1	1	0.143	0.455	NA	NA	0	0.299
SE	17011	ZNF438	ZNF438	chr10	-	30910359	30910440	30908932	30909015	30941574	30941651	1	1	0	4	4	0	0	0	0	1	98	49	1	1	0.111	0.111	NA	NA	0	0.111
SE	17012	ZNF438	ZNF438	chr10	-	30923286	30923415	30908932	30909015	30941574	30941651	5	4	0	4	4	0	0	1	0	1	98	49	1	1	0.385	0.333	NA	NA	0.333	0.026
SE	17013	ZNF438	ZNF438	chr10	-	30923286	30923415	30910359	30910440	30941574	30941651	1	4	0	1	0	0	0	1	0	0	98	49	1	1	0.333	1	NA	NA	1	-0.334
SE	17014	ZNF438	ZNF438	chr10	-	30941574	30941651	30923286	30923415	30984368	30984426	8	9	1	1	0	0	0	3	0	0	98	49	1	1	0.8	1	1	NA	1	-0.067
SE	17015	ZNF438	ZNF438	chr10	-	30984368	30984426	30941574	30941651	31031832	31031937	7	6	1	0	0	1	0	2	0	0	98	49	1	1	1	1	0.333	NA	1	-0.222
SE	17017	CSNK1A1	CSNK1A1	chr5	-	149517793	149517877	149513069	149513209	149520289	149520388	3	0	0	14	6	2	0	0	0	5	98	49	1	1	0.097	0	0	NA	0	0.032
SE	17023	ARRB1	ARRB1	chr11	-	75276838	75276911	75274073	75274211	75277363	75277448	10	2	0	1	0	0	0	2	0	0	98	49	1	1	0.833	1	NA	NA	1	-0.084
SE	17029	FAM114A2	FAM114A2	chr5	-	154029488	154029580	154028148	154028283	154033790	154033883	2	0	1	0	0	0	1	0	0	2	98	49	1	1	1	NA	1	1	0	0.5
SE	17030	GALNT10	GALNT10	chr5	+	154329571	154329738	154297940	154298079	154376276	154376462	8	3	2	2	0	0	0	1	0	0	98	49	1	1	0.667	1	1	NA	1	-0.111
SE	17033	SAP30L	SAP30L	chr5	+	154452455	154452499	154451090	154451213	154453401	154453500	10	1	0	2	0	0	0	1	0	0	93	49	1	1	0.725	1	NA	NA	1	-0.137
SE	17039	RENBP	RENBP	chrX	-	153941949	153942031	153941477	153941653	153942854	153943079	8	5	2	0	1	0	0	3	0	0	98	49	1	1	1	0.714	1	NA	1	-0.095
SE	17040	ITK	ITK	chr5	+	157214190	157214319	157211286	157211368	157217866	157217907	10	0	0	1	0	0	0	1	0	0	98	49	1	1	0.833	NA	NA	NA	1	-0.167
SE	17054	LCP2	LCP2	chr5	-	170275319	170275351	170274300	170274338	170275794	170275860	63	36	5	3	0	0	0	23	0	0	81	49	1	1	0.927	1	1	NA	1	-0.024
SE	17058	CIR1	CIR1	chr2	-	174378922	174379040	174351618	174351725	174380201	174380250	45	30	9	0	0	1	0	8	0	0	98	49	1	1	1	1	0.818	NA	1	-0.061
SE	17073	GRK6	GRK6	chr5	+	177432705	177432806	177432232	177432310	177433146	177433239	81	54	35	1	0	0	9	23	0	0	98	49	1	1	0.976	1	1	1	1	-0.008
SE	17078	PRKDC	PRKDC	chr8	-	47782161	47782254	47779003	47779093	47782377	47782598	11	3	6	2	2	2	0	4	0	0	98	49	1	1	0.733	0.429	0.6	NA	1	-0.413
SE	17079	PRKDC	PRKDC	chr8	-	47826655	47826861	47823857	47823996	47828167	47828347	13	4	7	2	0	0	0	7	0	0	98	49	1	1	0.765	1	1	NA	1	-0.078
SE	17080	PRKDC	PRKDC	chr8	-	47836337	47836527	47834195	47834396	47837211	47837419	15	6	12	0	0	0	0	11	0	2	98	49	1	1	1	1	1	NA	0.733	0.267
SE	17081	PRKDC	PRKDC	chr8	-	47839147	47839246	47837211	47837419	47840015	47840189	16	5	3	1	0	0	3	15	0	0	98	49	1	1	0.889	1	1	1	1	-0.037
SE	17087	PHYKPL	PHYKPL	chr5	-	178212972	178213103	178211889	178211970	178214795	178214885	11	5	1	3	0	0	0	0	0	1	98	49	1	1	0.647	1	1	NA	0	0.882
SE	17092	CLK4	CLK4	chr5	-	178617343	178617434	178616881	178616948	178618555	178618778	5	0	2	12	3	9	0	0	0	1	98	49	1	1	0.172	0	0.1	NA	0	0.091
SE	17096	CASP10	CASP10	chr2	+	201203729	201203766	201195841	201195948	201208074	201208183	2	0	0	0	0	0	0	1	0	1	86	49	1	1	1	NA	NA	NA	0.363	0.637
SE	17099	NEK7	NEK7	chr1	+	198277960	198278069	198264124	198264235	198278953	198279061	40	12	15	0	2	0	0	4	0	0	98	49	1	1	1	0.75	1	NA	1	-0.083
SE	17102	NQO2	NQO2	chr6	+	3002049	3002286	2999815	3000085	3003668	3003789	7	0	0	1	1	0	0	3	0	0	98	49	1	1	0.778	0	NA	NA	1	-0.611
SE	17105	NQO2	NQO2	chr6	+	3003668	3003789	3002049	3002286	3004494	3004651	11	0	0	1	1	0	0	3	0	0	98	49	1	1	0.846	0	NA	NA	1	-0.577
SE	17107	NQO2	NQO2	chr6	+	3015529	3015643	3012543	3012674	3016883	3016985	31	12	6	4	0	0	0	4	0	1	98	49	1	1	0.795	1	1	NA	0.667	0.265
SE	17108	ECI2	ECI2	chr6	-	4117307	4117451	4115692	4116029	4119185	4119275	2	0	0	0	0	0	0	2	0	1	98	49	1	1	1	NA	NA	NA	0.5	0.5
SE	17117	MCMBP	MCMBP	chr10	-	119836895	119837029	119835539	119835704	119838534	119838700	17	9	27	1	0	0	0	12	0	0	98	49	1	1	0.895	1	1	NA	1	-0.035
SE	17118	MCMBP	MCMBP	chr10	-	119842471	119842595	119840842	119840960	119843247	119843426	2	5	14	0	0	0	0	0	1	0	98	49	1	1	1	1	1	0	NA	1
RI	0	FAM3A	FAM3A	chrX	-	154515759	154516242	154515759	154515812	154515909	154516242	0	1	0	0	0	0	0	0	0	1	98	49	1	1	NA	1	NA	NA	0	1
RI	2	IDH3G	IDH3G	chrX	-	153785765	153786272	153785765	153785973	153786211	153786272	3	0	0	3	0	0	1	0	0	0	98	49	1	1	0.333	NA	NA	1	NA	-0.667
RI	56	MCM7	MCM7	chr7	-	100099022	100099403	100099022	100099203	100099278	100099403	2	2	0	0	2	0	0	1	0	0	98	49	1	1	1	0.333	NA	NA	1	-0.334
RI	58	SAMD9L	SAMD9L	chr7	-	93145384	93146040	93145384	93145532	93145908	93146040	15	3	5	1	0	0	3	10	0	0	98	49	1	1	0.882	1	1	1	1	-0.039
RI	70	TMED4	TMED4	chr7	-	44581722	44582295	44581722	44581823	44582046	44582295	10	1	0	7	0	2	0	1	0	0	98	49	1	1	0.417	1	0	NA	1	-0.528
RI	79	CD164	CD164	chr6	-	109366513	109369017	109366513	109368325	109368921	109369017	36	34	6	0	1	0	1	31	0	1	98	49	1	1	1	0.944	1	1	0.939	0.012
RI	81	SYTL3	SYTL3	chr6	+	158662749	158663378	158662749	158662812	158663204	158663378	17	13	3	2	0	0	2	1	0	0	98	49	1	1	0.81	1	1	1	1	-0.063
RI	82	HMGN3	HMGN3	chr6	-	79201244	79202389	79201244	79201726	79202275	79202389	7	4	0	0	0	0	0	2	0	1	98	49	1	1	1	1	NA	NA	0.5	0.5
RI	83	HMGN3	HMGN3	chr6	-	79201244	79202389	79201244	79201726	79202316	79202389	9	4	0	1	0	0	0	2	0	0	98	49	1	1	0.818	1	NA	NA	1	-0.091
RI	93	SLC39A7	SLC39A7	chr6	+	33200825	33201656	33200825	33200938	33201241	33201656	1	2	0	2	0	0	0	1	0	0	98	49	1	1	0.2	1	NA	NA	1	-0.4
RI	133	POU5F1	POU5F1	chr6	-	31165570	31167170	31165570	31165701	31165926	31167170	0	0	0	2	0	0	0	1	0	0	98	49	1	1	0	NA	NA	NA	1	-1
RI	164	BSDC1	BSDC1	chr1	-	32364632	32368550	32364632	32366654	32368446	32368550	1	4	1	5	1	3	3	0	0	0	98	49	1	1	0.091	0.667	0.143	1	NA	-0.7
RI	170	TAOK2	TAOK2	chr16	+	29986264	29988405	29986264	29986504	29986843	29988405	2	1	0	1	0	0	0	1	0	0	98	49	1	1	0.5	1	NA	NA	1	-0.25
RI	180	PRKD2	PRKD2	chr19	-	46716130	46717127	46716130	46716535	46716814	46717127	11	3	0	2	0	0	0	3	0	0	98	49	1	1	0.733	1	NA	NA	1	-0.133
RI	181	PRKD2	PRKD2	chr19	-	46716130	46717127	46716130	46716535	46716950	46717127	7	4	0	0	0	0	0	4	0	1	98	49	1	1	1	1	NA	NA	0.667	0.333
RI	189	SERF2	SERF2	chr15	+	43793709	43796089	43793709	43793936	43794373	43796089	52	25	17	0	1	0	1	27	0	0	98	49	1	1	1	0.926	1	1	1	-0.025
RI	199	KAT8	KAT8	chr16	+	31130745	31131393	31130745	31130900	31131194	31131393	6	5	4	6	4	0	0	0	1	0	98	49	1	1	0.333	0.385	1	0	NA	0.573
RI	205	PML	PML	chr15	+	74034477	74036394	74034477	74034530	74035171	74036394	14	7	2	1	0	0	1	7	0	0	98	49	1	1	0.875	1	1	1	1	-0.042
RI	206	CCPG1	CCPG1	chr15	-	55355222	55360944	55355222	55356409	55359538	55360944	15	17	7	9	4	1	0	1	0	1	98	49	1	1	0.455	0.68	0.778	NA	0.333	0.305
RI	219	CDK17	CDK17	chr12	-	96278260	96280885	96278260	96280279	96280807	96280885	9	1	6	2	2	0	0	13	0	0	98	49	1	1	0.692	0.2	1	NA	1	-0.369
RI	244	PSME1	PSME1	chr14	+	24138473	24138967	24138473	24138560	24138735	24138967	9	7	5	37	24	15	0	2	0	11	98	49	1	1	0.108	0.127	0.143	NA	0.083	0.043
RI	254	BIN2	BIN2	chr12	-	51302019	51302780	51302019	51302115	51302685	51302780	26	11	2	23	19	17	0	1	4	5	98	49	1	1	0.361	0.224	0.056	0	0.091	0.168
RI	255	LUC7L3	LUC7L3	chr17	+	50750500	50752711	50750500	50750694	50752199	50752711	23	2	1	2	0	0	0	2	0	2	98	49	1	1	0.852	1	1	NA	0.333	0.618
RI	273	ARL6IP4	ARL6IP4	chr12	+	122982468	122982913	122982468	122982538	122982619	122982913	2	0	2	5	1	0	0	0	0	1	98	49	1	1	0.167	0	1	NA	0	0.389
RI	275	ATPIF1	ATPIF1	chr1	+	28236360	28238105	28236360	28236452	28237836	28238105	2	0	0	4	5	2	0	5	0	1	98	49	1	1	0.2	0	0	NA	0.714	-0.647
RI	277	PPP1CC	PPP1CC	chr12	-	110719807	110721165	110719807	110720204	110721104	110721165	28	7	24	0	0	2	1	4	0	0	98	49	1	1	1	1	0.857	1	1	-0.048
RI	288	IL32	IL32	chr16	+	3067376	3067613	3067376	3067415	3067553	3067613	3	4	0	14	13	2	0	1	0	3	98	49	1	1	0.097	0.133	0	NA	0.143	-0.066
RI	306	P2RX7	P2RX7	chr12	+	121177146	121177446	121177146	121177212	121177296	121177446	0	1	0	3	0	0	0	2	0	0	98	49	1	1	0	1	NA	NA	1	-0.5
RI	311	C2CD5	C2CD5	chr12	-	22544060	22544546	22544060	22544179	22544319	22544546	1	0	0	9	2	0	0	2	0	0	98	49	1	1	0.053	0	NA	NA	1	-0.974
RI	312	RECQL	RECQL	chr12	-	21501169	21501669	21501169	21501215	21501412	21501669	4	1	6	2	0	0	0	0	0	1	98	49	1	1	0.5	1	1	NA	0	0.833
RI	314	RPL28	RPL28	chr19	+	55387929	55392083	55387929	55388048	55388242	55392083	6	1	0	17	9	2	0	1	0	13	98	49	1	1	0.15	0.053	0	NA	0.037	0.031
RI	320	QKI	QKI	chr6	+	163563419	163578596	163563419	163563719	163564666	163578596	15	4	5	0	0	2	0	2	0	0	98	49	1	1	1	1	0.556	NA	1	-0.148
RI	321	QKI	QKI	chr6	+	163563419	163578596	163563419	163563719	163565945	163578596	23	7	4	8	0	0	0	4	0	0	98	49	1	1	0.59	1	1	NA	1	-0.137
RI	322	QKI	QKI	chr6	+	163563419	163578596	163563419	163563719	163570693	163578596	17	3	3	1	0	0	0	8	0	0	98	49	1	1	0.895	1	1	NA	1	-0.035
RI	354	ZNRD1	ZNRD1	chr6	+	30061239	30061669	30061239	30061389	30061514	30061669	2	0	0	0	0	2	3	0	0	0	98	49	1	1	1	NA	0	1	NA	-0.5
RI	391	CCL4L2	CCL4L2	chr17	+	36211775	36212878	36211775	36211890	36212496	36212878	11	14	6	0	0	0	0	0	0	1	98	49	1	1	1	1	1	NA	0	1
RI	394	SAMD8	SAMD8	chr10	+	75176065	75182123	75176065	75176216	75176387	75182123	18	6	0	10	2	0	0	0	0	4	98	49	1	1	0.474	0.6	NA	NA	0	0.537
RI	395	DNAJB12	DNAJB12	chr10	-	72332829	72335931	72332829	72334617	72335779	72335931	10	7	1	4	0	4	0	1	0	3	98	49	1	1	0.556	1	0.111	NA	0.143	0.413
RI	400	CDK5RAP3	CDK5RAP3	chr17	+	47974399	47975337	47974399	47974448	47975158	47975337	66	15	4	0	1	0	7	10	0	1	98	49	1	1	1	0.882	1	1	0.833	0.044
RI	411	DNAJB2	DNAJB2	chr2	+	219284631	219286900	219284631	219284835	219285986	219286900	3	0	0	0	0	3	1	0	0	0	98	49	1	1	1	NA	0	1	NA	-0.5
RI	413	CDKN1C	CDKN1C	chr11	-	2884669	2885765	2884669	2885466	2885633	2885765	1	3	0	0	0	0	1	2	0	2	98	49	1	1	1	1	NA	1	0.333	0.334
RI	416	OTUB1	OTUB1	chr11	+	63996529	63996941	63996529	63996648	63996856	63996941	7	4	2	5	0	1	0	0	0	1	98	49	1	1	0.412	1	0.5	NA	0	0.637
RI	417	SPRTN	SPRTN	chr1	+	231351303	231355027	231351303	231351571	231352609	231355027	8	3	4	0	0	0	0	1	0	2	98	49	1	1	1	1	1	NA	0.2	0.8
RI	419	SLC46A3	SLC46A3	chr13	-	28700080	28701581	28700080	28700956	28701500	28701581	2	0	0	3	0	0	0	1	0	0	98	49	1	1	0.25	NA	NA	NA	1	-0.75
RI	433	VAMP4	VAMP4	chr1	-	171700155	171704534	171700155	171702084	171704386	171704534	5	0	0	1	0	0	2	0	0	0	98	49	1	1	0.714	NA	NA	1	NA	-0.286
RI	442	SNRNP27	SNRNP27	chr2	+	69904253	69905236	69904253	69904425	69904511	69905236	2	0	1	0	0	2	0	3	0	0	98	49	1	1	1	NA	0.2	NA	1	-0.4
RI	452	ATP5D	ATP5D	chr19	+	1244314	1244825	1244314	1244440	1244736	1244825	1	0	0	5	0	1	0	3	0	0	98	49	1	1	0.091	NA	0	NA	1	-0.955
RI	462	TK2	TK2	chr16	-	66548977	66550412	66548977	66549009	66549937	66550412	2	0	0	5	0	0	0	0	0	1	98	49	1	1	0.167	NA	NA	NA	0	0.167
RI	463	TRIM5	TRIM5	chr11	-	5663194	5665682	5663194	5664173	5665655	5665682	3	0	0	1	0	0	0	1	0	0	98	49	1	1	0.6	NA	NA	NA	1	-0.4
RI	464	TRIM5	TRIM5	chr11	-	5663194	5665682	5663194	5665395	5665655	5665682	2	0	0	6	0	0	0	2	0	1	98	49	1	1	0.143	NA	NA	NA	0.5	-0.357
RI	466	DHRS12	DHRS12	chr13	-	51767992	51769293	51767992	51768296	51769155	51769293	4	8	3	1	3	0	0	6	0	0	98	49	1	1	0.667	0.571	1	NA	1	-0.254
RI	474	TMEM41B	TMEM41B	chr11	-	9280654	9283593	9280654	9281041	9283370	9283593	8	2	0	1	1	0	0	2	0	0	98	49	1	1	0.8	0.5	NA	NA	1	-0.35
RI	475	AP1G2	AP1G2	chr14	-	23565815	23566160	23565815	23565892	23566063	23566160	6	0	0	0	3	0	0	4	0	2	98	49	1	1	1	0	NA	NA	0.5	0
RI	483	PAPD4	PAPD4	chr5	+	79612419	79613075	79612419	79612735	79613010	79613075	43	22	7	0	0	0	3	7	0	1	98	49	1	1	1	1	1	1	0.778	0.111
RI	490	MIER1	MIER1	chr1	+	66984571	66988619	66984571	66984592	66986402	66988619	48	10	7	2	2	0	0	6	0	0	98	49	1	1	0.923	0.714	1	NA	1	-0.121
RI	496	THUMPD1	THUMPD1	chr16	-	20733665	20737286	20733665	20736358	20736859	20737286	12	3	1	2	0	0	0	2	0	0	98	49	1	1	0.75	1	1	NA	1	-0.083
RI	498	CSTF3	CSTF3	chr11	-	33141430	33141986	33141430	33141762	33141884	33141986	3	0	0	0	3	0	0	0	0	1	98	49	1	1	1	0	NA	NA	0	0.5
RI	503	BICD2	BICD2	chr9	-	92711362	92715463	92711362	92713461	92715252	92715463	9	4	2	11	0	0	0	0	0	2	98	49	1	1	0.29	1	1	NA	0	0.763
RI	507	CDC16	CDC16	chr13	+	114272183	114272675	114272183	114272481	114272563	114272675	3	2	2	0	0	0	0	7	0	2	98	49	1	1	1	1	1	NA	0.636	0.364
RI	531	MYCL	MYCL	chr1	-	39900938	39902015	39900938	39901443	39901807	39902015	1	0	0	1	0	0	0	1	0	0	98	49	1	1	0.333	NA	NA	NA	1	-0.667
RI	533	EGR2	EGR2	chr10	-	62815860	62816366	62815860	62815969	62816069	62816366	85	8	1	1	0	0	0	10	0	0	98	49	1	1	0.977	1	1	NA	1	-0.008
RI	537	NDUFS2	NDUFS2	chr1	+	161213863	161214394	161213863	161213921	161214155	161214394	2	0	0	1	1	0	0	2	0	0	98	49	1	1	0.5	0	NA	NA	1	-0.75
RI	552	ACAA1	ACAA1	chr3	-	38126161	38126700	38126161	38126341	38126509	38126700	9	6	0	2	0	0	0	9	0	1	98	49	1	1	0.692	1	NA	NA	0.818	0.028
RI	554	ACADVL	ACADVL	chr17	+	7219830	7220197	7219830	7220046	7220121	7220197	6	5	0	5	12	0	2	1	2	3	98	49	1	1	0.375	0.172	NA	0.333	0.143	0.035
RI	555	VPS28	VPS28	chr8	-	144423600	144424132	144423600	144423922	144424040	144424132	7	5	0	2	7	7	0	0	0	3	98	49	1	1	0.636	0.263	0	NA	0	0.3
RI	574	HCFC1R1	HCFC1R1	chr16	-	3023232	3023530	3023232	3023361	3023473	3023530	1	0	0	1	0	0	0	1	0	0	98	49	1	1	0.333	NA	NA	NA	1	-0.667
RI	578	NAP1L1	NAP1L1	chr12	-	76045382	76048464	76045382	76047718	76048392	76048464	3	0	0	27	1	5	0	0	0	1	98	49	1	1	0.053	0	0	NA	0	0.018
RI	583	REC8	REC8	chr14	+	24172024	24172608	24172024	24172365	24172466	24172608	2	8	0	0	0	1	1	7	0	0	98	49	1	1	1	1	0	1	1	-0.333
RI	611	NFKB2	NFKB2	chr10	+	102395595	102395980	102395595	102395796	102395887	102395980	11	1	3	0	4	0	0	0	0	1	98	49	1	1	1	0.111	1	NA	0	0.704
RI	614	CELF2	CELF2	chr10	+	11328925	11336673	11328925	11329097	11330455	11336673	106	44	20	9	1	3	0	29	0	1	98	49	1	1	0.855	0.957	0.769	NA	0.935	-0.075
RI	618	TRIM52-AS1	TRIM52-AS1	chr5	+	181263627	181264566	181263627	181263766	181264051	181264566	1	2	3	2	0	0	0	2	0	0	98	49	1	1	0.2	1	1	NA	1	-0.267
RI	630	GMPR2	GMPR2	chr14	+	24238245	24239241	24238245	24238405	24238588	24239241	23	11	3	13	4	5	0	7	0	1	98	49	1	1	0.469	0.579	0.231	NA	0.778	-0.352
RI	648	SEC14L1	SEC14L1	chr17	+	77213917	77217099	77213917	77214019	77216548	77217099	197	72	25	1	0	1	13	22	0	0	98	49	1	1	0.99	1	0.926	1	1	-0.028
RI	663	LRTOMT	LRTOMT	chr11	+	72094947	72096892	72094947	72095096	72095378	72096892	2	0	0	0	1	0	0	1	0	0	98	49	1	1	1	0	NA	NA	1	-0.5
RI	673	KCNK7	KCNK7	chr11	-	65592854	65593874	65592854	65593210	65593475	65593874	12	10	4	3	0	0	0	5	0	0	98	49	1	1	0.667	1	1	NA	1	-0.111
RI	677	P3H1	P3H1	chr1	-	42746334	42747412	42746334	42746852	42747271	42747412	0	0	0	1	0	0	0	1	0	0	98	49	1	1	0	NA	NA	NA	1	-1
RI	712	CREBZF	CREBZF	chr11	-	85657563	85665138	85657563	85662457	85663523	85665138	9	12	0	4	1	1	0	4	0	2	98	49	1	1	0.529	0.857	0	NA	0.5	-0.038
RI	713	CREBZF	CREBZF	chr11	-	85663523	85665138	85663523	85663601	85663814	85665138	7	3	0	3	0	0	0	2	0	0	98	49	1	1	0.538	1	NA	NA	1	-0.231
RI	716	BET1L	BET1L	chr11	-	202923	205667	202923	205469	205610	205667	17	1	0	1	2	0	0	7	0	0	98	49	1	1	0.895	0.2	NA	NA	1	-0.453

RI	728	PTPMT1	PTPMT1	chr11	+	47565429	47565986	47565429	47565796	47565905	47565986	2	1	0	1	0	0	0	2	1	0	98	49	1	1	0.5	1	NA	0	1	0.25
RI	730	WRAP53	WRAP53	chr17	+	7688348	7689079	7688348	7688561	7688647	7689079	3	0	0	0	0	0	0	0	0	2	98	49	1	1	1	NA	NA	NA	0	1
RI	731	RANGRF	RANGRF	chr17	+	8289257	8290091	8289257	8289414	8289502	8290091	1	0	0	2	0	0	0	1	0	0	98	49	1	1	0.2	NA	NA	NA	1	-0.8
RI	741	QSOX1	QSOX1	chr1	+	180196261	180198034	180196261	180196599	180197332	180198034	12	5	11	0	0	0	0	11	0	3	98	49	1	1	1	1	1	NA	0.647	0.353
RI	743	ZNF248	ZNF248	chr10	-	37828764	37833116	37828764	37831312	37833024	37833116	0	0	0	1	0	0	0	1	0	0	98	49	1	1	0	NA	NA	NA	1	-1
RI	754	UBXN1	UBXN1	chr11	-	62676497	62677005	62676497	62676639	62676812	62677005	7	7	0	1	2	4	0	0	0	7	98	49	1	1	0.778	0.636	0	NA	0	0.471
RI	765	SRSF1	SRSF1	chr17	-	58000918	58005973	58000918	58004001	58004922	58005973	31	20	14	3	1	0	0	6	0	0	98	49	1	1	0.838	0.909	1	NA	1	-0.084
RI	766	SRSF1	SRSF1	chr17	-	58000918	58005973	58000918	58005600	58005800	58005973	10	3	5	12	2	1	0	1	0	5	98	49	1	1	0.294	0.429	0.714	NA	0.091	0.388
RI	767	GLYCTK	GLYCTK	chr3	+	52292259	52293192	52292259	52292569	52293045	52293192	4	0	2	0	0	1	2	1	0	0	98	49	1	1	1	NA	0.5	1	1	-0.25
RI	775	DCAF7	DCAF7	chr17	+	63588999	63594281	63588999	63589237	63594202	63594281	3	11	1	1	1	0	0	5	0	2	98	49	1	1	0.6	0.846	1	NA	0.556	0.259
RI	788	TYMP	TYMP	chr22	-	50529495	50530085	50529495	50529731	50529903	50530085	70	48	6	2	0	0	7	17	0	1	98	49	1	1	0.946	1	1	1	0.895	0.034
RI	790	CANT1	CANT1	chr17	-	78996991	78997963	78996991	78997644	78997839	78997963	24	16	4	6	1	0	0	8	0	0	98	49	1	1	0.667	0.889	1	NA	1	-0.148
RI	794	OXLD1	OXLD1	chr17	-	81665035	81666637	81665035	81665584	81666154	81666637	2	0	0	0	0	0	0	1	0	2	98	49	1	1	1	NA	NA	NA	0.2	0.8
RI	797	OXLD1	OXLD1	chr17	-	81665035	81666637	81665035	81665584	81666517	81666637	1	0	0	0	0	0	0	1	0	1	98	49	1	1	1	NA	NA	NA	0.333	0.667
RI	798	FAM195B	FAM195B	chr17	-	81822360	81823311	81822360	81822907	81823208	81823311	3	2	0	2	0	0	0	3	0	0	98	49	1	1	0.429	1	NA	NA	1	-0.285
RI	833	TMEM205	TMEM205	chr19	-	11345519	11346305	11345519	11345628	11346126	11346305	1	1	2	3	0	0	0	4	0	1	98	49	1	1	0.143	1	1	NA	0.667	0.047
RI	840	AKAP8L	AKAP8L	chr19	-	15403474	15403715	15403474	15403555	15403657	15403715	31	5	6	0	2	5	0	4	0	1	98	49	1	1	1	0.556	0.375	NA	0.667	-0.023
RI	843	AUP1	AUP1	chr2	-	74528750	74529282	74528750	74528935	74529131	74529282	6	0	0	0	0	1	1	0	0	2	98	49	1	1	1	NA	0	1	0	0
RI	845	CCDC125	CCDC125	chr5	-	69320236	69320580	69320236	69320345	69320464	69320580	33	7	0	5	1	1	0	0	0	1	98	49	1	1	0.767	0.778	0	NA	0	0.515
RI	847	EDEM2	EDEM2	chr20	-	35146824	35147358	35146824	35146935	35147151	35147358	7	0	0	4	6	0	0	0	0	1	98	49	1	1	0.467	0	NA	NA	0	0.234
RI	849	MCL1	MCL1	chr1	-	150578842	150579738	150578842	150578963	150579422	150579738	1364	513	216	3	0	0	41	264	0	2	98	49	1	1	0.996	1	1	1	0.985	0.006
RI	856	CUTA	CUTA	chr6	-	33417480	33418288	33417480	33417695	33417958	33418288	4	2	0	0	1	0	1	1	0	1	98	49	1	1	1	0.5	NA	1	0.333	0.084
RI	865	U2AF1L4	U2AF1L4	chr19	-	35744069	35744421	35744069	35744145	35744322	35744421	5	13	0	2	1	4	0	1	0	1	98	49	1	1	0.556	0.867	0	NA	0.333	0.141
RI	871	NFKBIB	NFKBIB	chr19	+	38907398	38908894	38907398	38907659	38908730	38908894	9	3	1	2	0	0	0	0	0	1	98	49	1	1	0.692	1	1	NA	0	0.897
RI	881	NAPA	NAPA	chr19	-	47487633	47488389	47487633	47487890	47488234	47488389	16	17	11	1	0	0	0	17	0	0	98	49	1	1	0.889	1	1	NA	1	-0.037
RI	919	PTPRO	PTPRO	chr12	+	15594936	15598331	15594936	15595057	15596089	15598331	1	0	0	0	0	0	0	0	0	1	98	49	1	1	1	NA	NA	NA	0	1
RI	925	CLK1	CLK1	chr2	-	200859679	200861466	200859679	200859746	200861237	200861466	27	16	2	18	9	6	0	5	0	2	98	49	1	1	0.429	0.471	0.143	NA	0.556	-0.208
RI	944	SUMO1	SUMO1	chr2	-	202206179	202207321	202206179	202206803	202207252	202207321	23	13	9	0	0	0	1	10	0	1	98	49	1	1	1	1	1	1	0.833	0.084
RI	945	RPL31	RPL31	chr2	+	101005958	101006423	101005958	101006071	101006349	101006423	6	1	3	53	13	6	0	4	0	5	98	49	1	1	0.054	0.037	0.2	NA	0.286	-0.189
RI	947	C2orf49	C2orf49	chr2	+	105342847	105343223	105342847	105342974	105343100	105343223	5	3	6	1	0	0	0	2	0	0	98	49	1	1	0.714	1	1	NA	1	-0.095
RI	953	EIF6	EIF6	chr20	-	35284175	35284492	35284175	35284261	35284380	35284492	4	0	0	7	1	0	0	1	0	0	98	49	1	1	0.222	0	NA	NA	1	-0.889
RI	956	ATP5G3	ATP5G3	chr2	-	175176257	175179250	175176257	175178402	175179056	175179250	1	2	0	2	0	0	0	0	0	1	98	49	1	1	0.2	1	NA	NA	0	0.6
RI	974	SMOX	SMOX	chr20	+	4182088	4182848	4182088	4182323	4182482	4182848	3	0	0	0	1	0	1	0	1	0	98	49	1	1	1	0	NA	0.333	NA	0.167
RI	991	DCTN1	DCTN1	chr2	-	74366243	74366620	74366243	74366375	74366458	74366620	4	0	0	8	0	1	0	1	1	1	98	49	1	1	0.2	NA	0	0	0.333	-0.067
RI	996	PAQR6	PAQR6	chr1	-	156245781	156246250	156245781	156245987	156246122	156246250	0	0	1	0	0	6	2	0	0	0	98	49	1	1	NA	NA	0.077	1	NA	-0.923
RI	1007	DKC1	DKC1	chrX	+	154774601	154775273	154774601	154774705	154775194	154775273	5	4	0	2	3	0	0	1	0	0	98	49	1	1	0.556	0.4	NA	NA	1	-0.522
RI	1010	TAF1C	TAF1C	chr16	-	84181327	84181663	84181327	84181463	84181591	84181663	8	1	0	0	0	0	0	3	0	1	98	49	1	1	1	1	NA	NA	0.6	0.4
RI	1030	XBP1	XBP1	chr22	-	28796046	28796192	28796046	28796123	28796149	28796192	11	3	0	2	0	0	0	4	0	0	75	49	1	1	0.782	1	NA	NA	1	-0.109
RI	1036	TSTD1	TSTD1	chr1	-	161037631	161038075	161037631	161037826	161037912	161038075	2	0	0	4	7	2	0	0	0	1	98	49	1	1	0.2	0	0	NA	0	0.067
RI	1052	IL17RC	IL17RC	chr3	+	9923880	9924291	9923880	9924020	9924231	9924291	0	0	0	2	0	0	1	0	0	1	98	49	1	1	0	NA	NA	1	0	-0.5
RI	1053	RPRD2	RPRD2	chr1	+	150470560	150476565	150470560	150471742	150471882	150476565	0	0	0	1	0	0	1	0	0	0	98	49	1	1	0	NA	NA	1	NA	-1
RI	1068	CTNNB1	CTNNB1	chr3	+	41239133	41240448	41239133	41239354	41239659	41240448	19	13	0	7	0	6	0	6	0	3	98	49	1	1	0.576	1	0	NA	0.5	0.025
RI	1069	CTNNB1	CTNNB1	chr3	+	41239133	41240448	41239133	41239354	41239818	41240448	19	13	0	1	0	1	0	13	0	0	98	49	1	1	0.905	1	0	NA	1	-0.365
RI	1080	NDUFB4	NDUFB4	chr3	+	120601110	120602411	120601110	120601257	120602207	120602411	1	1	0	1	0	3	0	0	0	1	98	49	1	1	0.333	1	0	NA	0	0.444
RI	1089	ROPN1L	ROPN1L	chr5	+	10441861	10442298	10441861	10442003	10442126	10442298	5	3	1	2	0	0	0	4	0	0	98	49	1	1	0.556	1	1	NA	1	-0.148
RI	1090	NIPBL	NIPBL	chr5	+	37063789	37065819	37063789	37063978	37064526	37065819	15	5	0	6	1	0	0	4	0	0	98	49	1	1	0.556	0.714	NA	NA	1	-0.365
RI	1091	NSD1	NSD1	chr5	+	177135086	177136030	177135086	177135200	177135940	177136030	11	5	1	17	1	0	0	1	0	0	98	49	1	1	0.244	0.714	1	NA	1	-0.347
RI	1096	CIRBP	CIRBP	chr19	+	1271980	1273172	1271980	1272051	1272426	1273172	32	2	2	22	6	3	0	3	0	1	98	49	1	1	0.421	0.143	0.25	NA	0.6	-0.329
RI	1110	HNRNPM	HNRNPM	chr19	+	8465323	8465515	8465323	8465362	8465479	8465515	13	6	1	5	2	3	0	8	0	1	98	49	1	1	0.565	0.6	0.143	NA	0.8	-0.364
RI	1123	SKP1	SKP1	chr5	-	134156390	134158595	134156390	134157768	134158454	134158595	18	3	3	2	0	0	0	1	0	0	98	49	1	1	0.818	1	1	NA	1	-0.061
RI	1131	NDUFA2	NDUFA2	chr5	-	140647255	140647785	140647255	140647362	140647482	140647785	8	2	5	8	5	1	0	0	0	2	98	49	1	1	0.333	0.167	0.714	NA	0	0.405
RI	1133	RELL2	RELL2	chr5	+	141636949	141638409	141636949	141637741	141638169	141638409	6	2	0	2	0	0	2	0	0	0	98	49	1	1	0.6	1	NA	1	NA	-0.2
RI	1134	ABLIM3	ABLIM3	chr5	+	149141482	149142108	149141482	149141654	149142008	149142108	1	0	0	0	0	0	0	1	1	0	98	49	1	1	1	NA	NA	0	1	0.5
RI	1135	ZNF438	ZNF438	chr10	-	30848530	30850367	30848530	30849443	30850221	30850367	10	8	4	1	0	0	1	12	0	0	98	49	1	1	0.833	1	1	1	1	-0.056

U12_4_filtered

												Inclusion counts			*Skipping counts*			*Inclusion counts*		*Skipping counts*						*Inclusion_level(normalized)*					
Splice_type	*ID*	*Gene_ID*	*Gene_Symbol*	*Chromosome*	*Strand*	*LongExonStart_0base*	*LongExonEnd*	*ShortES*	*ShortEE*	*FlankingES*	*FlankingEE*	*control_s3*	*control_s4*	*control_s10*	*control_s3*	*control_s4*	*control_s10*	*Idg_14*	*Ildg_s16*	*Idg_s14*	*Idg_s16*	*IncFormLen*	*SkipFormLen*	*p-value*	*adjust_p*	*IncLevel_s3*	*IncLevel_s4*	*IncLevel_s10*	*IncLevel14*	*IncLevel16*	*Level_diff*
SE	8677	MAEA	MAEA	chr4	+	1322380	1322503	1315396	1315600	1332756	1332865	11	5	2	0	4	0	0	0	1	1	98	49	5.28102E-08	0.000152595	1	0.385	1	0	0	0.795
RI	488	STX10	STX10	chr19	-	13144057	13144671	13144057	13144486	13144576	13144671	15	16	0	9	10	13	1	6	0	1	98	49	0.000313634	0.0129374	0.455	0.444	0	1	0.75	-0.575
SE	6756	SETD1B	SETD1B	chr12	+	121819403	121819895	121817798	121817904	121822489	121823749	8	9	3	0	0	0	3	1	0	3	98	49	0.014021703	0.578795883	1	1	1	1	0.143	0.428
SE	5176	SLC12A9	SLC12A9	chr7	+	100861137	100861262	100860149	100860232	100861391	100861584	62	24	16	1	2	3	4	10	0	0	98	49	0.01640589	0.627878418	0.969	0.857	0.727	1	1	-0.149
SE	389	RAPGEF1	RAPGEF1	chr9	-	131650128	131650242	131643238	131643417	131650809	131650949	18	3	1	4	2	0	2	1	0	1	98	49	0.030356618	0.958638776	0.692	0.429	1	1	0.333	0.04
SE	8832	DOCK2	DOCK2	chr5	+	169759704	169759775	169747395	169747504	169761518	169761625	103	48	17	3	3	0	3	30	0	0	98	49	0.054985887	1	0.945	0.889	1	1	1	-0.055
SE	15461	RAF1	RAF1	chr3	-	12611949	12612062	12609232	12609335	12618514	12618747	159	54	30	3	0	4	3	15	0	0	98	49	0.055208514	1	0.964	1	0.789	1	1	-0.082
SE	6836	GAK	GAK	chr4	-	877614	877809	877089	877207	881906	882040	8	7	5	1	3	0	2	6	0	0	98	49	0.061544568	1	0.8	0.538	1	1	1	-0.221
SE	7502	SLC12A6	SLC12A6	chr15	-	34275344	34275389	34260925	34261020	34336409	34336601	155	85	27	2	2	3	6	36	0	0	94	49	0.072192319	1	0.976	0.957	0.824	1	1	-0.081
SE	1987	SRPK1	SRPK1	chr6	-	35870280	35870494	35869481	35869901	35870933	35870959	19	4	7	8	10	0	0	4	1	5	98	49	0.082834818	1	0.543	0.167	1	0	0.286	0.427
SE	8679	MAEA	MAEA	chr4	+	1327626	1327703	1322380	1322503	1332756	1332865	13	8	8	0	3	1	3	2	0	0	98	49	0.089381693	1	1	0.571	0.8	1	1	-0.21
SE	7319	VPS11	VPS11	chr11	+	119078564	119078704	119078172	119078334	119078794	119078997	6	1	2	0	0	0	0	2	1	0	98	49	0.101331953	1	1	1	1	0	1	0.5
SE	10160	BRMS1	BRMS1	chr11	-	66340120	66340213	66338720	66338785	66340773	66340870	21	4	9	0	0	0	0	2	1	0	98	49	0.101331953	1	1	1	1	0	1	0.5
A3SS	2325	ARPC5	ARPC5	chr1	-	183633081	183633163	183633081	183633154	183635516	183635941	7	1	0	103	77	27	0	0	4	20	58	49	0.153712068	1	0.054	0.011	0	0	0	0.022
SE	6835	GAK	GAK	chr4	-	851749	851974	850935	851084	859605	859722	8	3	6	0	0	0	1	6	2	0	98	49	0.177931592	1	1	1	1	0.2	1	0.4
RI	580	ATG3	ATG3	chr3	-	112532506	112534337	112532506	112532780	112534268	112534337	10	6	4	28	11	3	0	3	3	5	98	49	0.199919419	1	0.152	0.214	0.4	0	0.231	0.14
SE	978	DOCK5	DOCK5	chr8	+	25407982	25408154	25403557	25403724	25408801	25408940	105	24	4	5	5	1	1	23	1	1	98	49	0.239086319	1	0.913	0.706	0.667	0.333	0.92	0.135
SE	976	DOCK5	DOCK5	chr8	+	25368570	25368725	25366869	25366970	25369555	25369641	85	22	4	3	0	0	1	12	0	0	98	49	0.347461072	1	0.934	1	1	1	1	-0.022
SE	11956	CHD3	CHD3	chr17	+	7902936	7903061	7902609	7902727	7903271	7903503	9	7	2	0	0	0	1	3	0	2	98	49	0.39801607	1	1	1	1	1	0.429	0.285
SE	8831	DOCK2	DOCK2	chr5	+	169714359	169714457	169714027	169714211	169716212	169716302	109	62	13	3	0	0	13	24	0	0	98	49	0.477632073	1	0.948	1	1	1	1	-0.017
SE	5177	SLC12A9	SLC12A9	chr7	+	100861736	100861911	100861391	100861584	100862680	100862827	53	23	12	2	1	0	2	16	0	0	98	49	0.480487308	1	0.93	0.92	1	1	1	-0.05
SE	8828	DOCK2	DOCK2	chr5	+	169684195	169684350	169681743	169681879	169689251	169689333	203	80	23	3	1	1	4	41	0	0	98	49	0.555294052	1	0.971	0.976	0.92	1	1	-0.044
SE	6426	RALGAPA2	RALGAPA2	chr20	-	20680690	20680801	20653529	20653587	20712374	20712623	23	10	2	2	1	0	2	7	0	0	98	49	0.574229202	1	0.852	0.833	1	1	1	-0.105
SE	15458	RAF1	RAF1	chr3	-	12590797	12590974	12587590	12587637	12591707	12591792	180	64	32	2	0	0	1	24	0	2	98	49	0.579441471	1	0.978	1	1	1	0.857	0.064
SE	8678	MAEA	MAEA	chr4	+	1327626	1327703	1315396	1315600	1332756	1332865	12	5	7	0	4	0	3	2	1	1	98	49	0.60937349	1	1	0.385	1	0.6	0.5	0.245
SE	6430	LMBR1L	LMBR1L	chr12	-	49104447	49104551	49103686	49103813	49104745	49104885	9	3	2	0	2	0	1	2	0	0	98	49	0.683158228	1	1	0.429	1	1	1	-0.19
SE	970	DOCK5	DOCK5	chr8	+	25300575	25300657	25298943	25299101	25302324	25302454	135	60	13	0	4	0	3	24	0	0	98	49	0.730535785	1	1	0.882	1	1	1	-0.039
A3SS	1384	RASGRP4	RASGRP4	chr19	-	38419859	38420013	38419859	38419971	38420130	38420262	54	25	21	0	0	3	5	20	0	0	91	49	0.75562353	1	1	1	0.79	1	1	-0.07
SE	7501	SLC12A6	SLC12A6	chr15	-	34257641	34257788	34256228	34256283	34258812	34258944	64	22	9	10	11	3	1	11	0	4	98	49	0.771324392	1	0.762	0.5	0.6	1	0.579	-0.169
SE	16463	ZDHHC17	ZDHHC17	chr12	+	76797433	76797537	76764073	76764329	76805316	76805439	20	10	2	2	2	2	1	8	0	3	98	49	0.914990937	1	0.833	0.714	0.333	1	0.571	-0.159
SE	11513	SBNO2	SBNO2	chr19	-	1112188	1112301	1111995	1112067	1112401	1112537	61	21	22	10	4	0	8	15	4	0	98	49	0.956284839	1	0.753	0.724	1	0.5	1	0.076
A3SS	139	NOL8	NOL8	chr9	-	92299889	92300810	92299889	92300016	92301550	92301822	2	0	0	2	1	0	0	1	0	0	98	49	1	1	0.333	0	NA	NA	1	-0.834
A3SS	182	INTS8	INTS8	chr8	+	94838462	94838618	94838493	94838618	94836523	94836631	11	0	1	2	0	0	0	0	0	1	80	49	1	1	0.771	NA	1	NA	0	0.885
A3SS	384	SRPK1	SRPK1	chr6	-	35890894	35891041	35890894	35891013	35920467	35920528	5	2	0	5	2	0	0	0	1	2	77	49	1	1	0.389	0.389	NA	0	0	0.389
A3SS	632	EML3	EML3	chr11	-	62611086	62611347	62611086	62611344	62611424	62611596	1	0	0	4	0	0	0	1	0	0	52	49	1	1	0.191	NA	NA	NA	1	-0.809
A3SS	711	SBNO1	SBNO1	chr12	-	123345257	123345570	123345257	123345567	123348028	123348133	8	1	2	7	4	0	0	0	0	1	52	49	1	1	0.519	0.191	1	NA	0	0.57
A3SS	939	TTC23	TTC23	chr15	-	99139316	99139467	99139316	99139399	99156147	99156297	0	1	0	0	0	0	0	0	0	1	98	49	1	1	NA	1	NA	NA	0	1
A3SS	1382	RASGRP4	RASGRP4	chr19	-	38414847	38415123	38414847	38414916	38417051	38417168	55	18	9	0	1	1	2	16	0	0	98	49	1	1	1	0.9	0.818	1	1	-0.094
A3SS	1493	SRSF10	SRSF10	chr1	-	23964346	23971439	23964346	23967723	23971572	23971623	4	1	0	4	0	2	0	1	0	0	98	49	1	1	0.333	1	0	NA	1	-0.556
A3SS	1494	SRSF10	SRSF10	chr1	-	23964346	23971439	23964346	23967956	23971572	23971623	4	4	0	0	1	0	0	1	0	0	98	49	1	1	1	0.667	NA	NA	1	-0.166
A3SS	1604	CEP170	CEP170	chr1	-	243156220	243156455	243156220	243156347	243164283	243166116	4	2	0	1	0	0	0	1	0	0	98	49	1	1	0.667	1	NA	NA	1	-0.166
A3SS	1651	LMBR1L	LMBR1L	chr12	-	49101249	49101323	49101249	49101263	49101471	49101549	8	3	4	1	1	0	1	4	0	0	98	49	1	1	0.8	0.6	1	1	1	-0.2
A3SS	2082	CNIH4	CNIH4	chr1	+	224371282	224371423	224371329	224371423	224365878	224365991	26	7	6	1	0	0	0	7	0	0	96	49	1	1	0.93	1	1	NA	1	-0.023
A3SS	2225	CHD4	CHD4	chr12	-	6601959	6602175	6601959	6602154	6602375	6602497	18	5	0	4	3	0	0	2	0	1	70	49	1	1	0.759	0.538	NA	NA	0.583	0.066
A3SS	3040	TNPO2	TNPO2	chr19	-	12702071	12702177	12702071	12702147	12702822	12702918	0	0	0	2	0	0	1	0	0	0	79	49	1	1	0	NA	NA	1	NA	-1
A3SS	3096	TMEM150B	TMEM150B	chr19	-	55320968	55321093	55320968	55321044	55322647	55322743	1	0	0	0	0	0	0	0	0	1	98	49	1	1	1	NA	NA	NA	0	1
A3SS	3469	DCTN3	DCTN3	chr9	-	34614041	34614132	34614041	34614101	34614709	34614768	0	5	0	3	4	1	1	0	0	0	80	49	1	1	0	0.434	0	1	NA	-0.855
A3SS	3470	DCTN3	DCTN3	chr9	-	34614041	34614198	34614041	34614101	34614709	34614768	0	5	0	3	4	1	1	0	0	0	98	49	1	1	0	0.385	0	1	NA	-0.872
A3SS	4093	HARS2	HARS2	chr5	+	140693490	140693665	140693590	140693665	140691425	140691756	4	5	0	9	0	0	0	2	0	0	98	49	1	1	0.182	1	NA	NA	1	-0.409
A5SS	73	TRAPPC2	TRAPPC2	chrX	-	13734043	13734185	13734049	13734185	13719870	13719982	2	3	1	0	2	0	0	2	0	0	55	49	1	1	1	0.572	1	NA	1	-0.143
A5SS	352	VPS52	VPS52	chr6	-	33271136	33271965	33271585	33271965	33270198	33270283	2	0	2	3	1	0	0	0	0	1	98	49	1	1	0.25	0	1	NA	0	0.417
A5SS	1012	SLC12A9	SLC12A9	chr7	+	100852718	100852839	100852718	100852835	100854155	100854378	4	2	1	3	2	1	0	0	0	2	53	49	1	1	0.552	0.48	0.48	NA	0	0.504
A5SS	1303	DAP3	DAP3	chr1	+	155689093	155689243	155689093	155689174	155709772	155709824	2	2	0	0	0	0	0	0	0	1	98	49	1	1	1	1	NA	NA	0	1
A5SS	1349	STX10	STX10	chr19	-	13144763	13144870	13144767	13144870	13144576	13144671	38	11	6	0	0	1	4	7	0	0	53	49	1	1	1	1	0.847	1	1	-0.051
A5SS	1619	PLCB2	PLCB2	chr15	-	40296745	40296908	40296757	40296908	40296521	40296634	16	7	1	3	6	0	0	7	0	3	61	49	1	1	0.811	0.484	1	NA	0.652	0.113
A5SS	1810	NAA60	NAA60	chr16	+	3484698	3485083	3484698	3485061	3485466	3486963	3	3	0	1	0	0	0	0	0	1	71	49	1	1	0.674	1	NA	NA	0	0.837
A5SS	2073	GOSR1	GOSR1	chr17	+	30481142	30481263	30481142	30481257	30484213	30484301	3	1	0	5	0	2	0	1	0	0	55	49	1	1	0.348	1	0	NA	1	-0.551
A5SS	3129	MCMBP	MCMBP	chr10	-	119843247	119843426	119843253	119843426	119842471	119842595	0	2	7	2	1	2	0	0	0	3	55	49	1	1	0	0.641	0.757	NA	0	0.466
SE	123	DIAPH2	DIAPH2	chrX	+	96735757	96735790	96684662	96685190	96738585	96738762	2	1	2	1	1	0	0	1	0	0	82	49	1	1	0.544	0.374	1	NA	1	-0.361
SE	388	RAPGEF1	RAPGEF1	chr9	-	131630235	131630324	131629101	131629254	131638634	131638791	25	12	3	2	1	0	0	6	0	2	98	49	1	1	0.862	0.857	1	NA	0.6	0.306
SE	818	INTS8	INTS8	chr8	+	94856776	94856978	94853804	94853915	94859510	94859632	7	1	1	1	0	0	0	2	0	0	98	49	1	1	0.778	1	1	NA	1	-0.074
SE	822	INTS8	INTS8	chr8	+	94868691	94868813	94867275	94867337	94871883	94872002	1	0	0	2	1	1	0	0	0	1	98	49	1	1	0.2	0	0	NA	0	0.067
SE	966	PPP2R2A	PPP2R2A	chr8	+	26363720	26363890	26362683	26362848	26366314	26366406	16	3	2	0	2	0	0	2	0	0	98	49	1	1	1	0.429	1	NA	1	-0.19
SE	967	DOCK5	DOCK5	chr8	+	25268844	25268885	25243673	25243757	25275385	25275441	148	62	18	1	0	0	1	18	0	0	90	49	1	1	0.988	1	1	1	1	-0.004
SE	968	DOCK5	DOCK5	chr8	+	25278568	25278665	25275385	25275441	25292023	25292172	229	94	12	0	3	0	5	32	0	0	98	49	1	1	1	0.94	1	1	1	-0.02
SE	969	DOCK5	DOCK5	chr8	+	25296512	25296648	25292023	25292172	25298943	25299101	181	82	14	2	1	0	3	29	0	0	98	49	1	1	0.978	0.976	1	1	1	-0.015
SE	971	DOCK5	DOCK5	chr8	+	25317006	25317131	25310406	25310532	25319577	25319676	132	75	18	1	0	0	2	30	0	0	98	49	1	1	0.985	1	1	1	1	-0.005
SE	972	DOCK5	DOCK5	chr8	+	25334095	25334196	25332602	25332692	25336238	25336373	175	73	14	1	0	0	1	36	0	0	98	49	1	1	0.989	1	1	1	1	-0.004
SE	973	DOCK5	DOCK5	chr8	+	25340876	25340988	25336238	25336373	25341738	25341809	115	49	6	8	7	0	0	19	0	0	98	49	1	1	0.878	0.778	1	NA	1	-0.115
SE	974	DOCK5	DOCK5	chr8	+	25342400	25342507	25341738	25341809	25345474	25345611	107	55	21	1	0	0	0	15	0	0	98	49	1	1	0.982	1	1	NA	1	-0.006
SE	975	DOCK5	DOCK5	chr8	+	25368191	25368250	25366869	25366970	25368570	25368725	86	32	10	3	0	0	0	11	0	5	98	49	1	1	0.935	1	1	NA	0.524	0.454
SE	977	DOCK5	DOCK5	chr8	+	25373617	25373658	25372558	25372718	25374563	25374654	85	38	16	0	0	0	0	8	0	1	90	49	1	1	1	1	1	NA	0.813	0.187
SE	994	XPO7	XPO7	chr8	+	21990810	21990919	21990343	21990407	21991867	21991974	3	0	3	1	0	0	0	2	0	0	98	49	1	1	0.6	NA	1	NA	1	-0.2
SE	1216	SRPK2	SRPK2	chr7	-	105133003	105133104	105132790	105132898	105142007	105142490	40	17	4	1	0	0	0	9	0	0	98	49	1	1	0.952	1	1	NA	1	-0.016
SE	1217	SRPK2	SRPK2	chr7	-	105268805	105268869	105203627	105203785	105388647	105388702	4	4	1	10	2	5	0	0	0	1	98	49	1	1	0.167	0.5	0.091	NA	0	0.253
SE	1330	RHBDD2	RHBDD2	chr7	+	75881364	75881486	75878998	75879260	75881828	75882236	2	0	1	2	0	1	0	2	1	0	98	49	1	1	0.333	NA	0.333	0	1	-0.167
SE	1418	NCBP1	NCBP1	chr9	+	97662041	97662144	97660945	97661068	97662953	97663047	6	3	0	0	0	0	1	1	0	2	98	49	1	1	1	1	NA	1	0.2	0.4
SE	1592	TCP1	TCP1	chr6	-	159780934	159781110	159780442	159780566	159783940	159784067	13	7	3	3	0	0	0	1	0	0	98	49	1	1	0.684	1	1	NA	1	-0.105
SE	1606	TMEM181	TMEM181	chr6	+	158583953	158584044	158580939	158580995	158585303	158585425	7	5	3	0	0	2	0	4	0	0	98	49	1	1	1	1	0.429	NA	1	-0.19
SE	1632	STXBP5	STXBP5	chr6	+	147314263	147314331	147313883	147314031	147314595	147314636	51	28	17	3	0	0	0	9	0	0	98	49	1	1	0.895	1	1	NA	1	-0.035
SE	1633	STXBP5	STXBP5	chr6	+	147314595	147314636	147313883	147314031	147315514	147315735	26	3	1	0	1	0	0	4	0	0	90	49	1	1	1	0.62	1	NA	1	-0.127
SE	1634	STXBP5	STXBP5	chr6	+	147339178	147339238	147334156	147334222	147353322	147353373	15	3	0	6	0	0	0	4	0	0	98	49	1	1	0.556	1	NA	NA	1	-0.222
SE	1635	STXBP5	STXBP5	chr6	+	147339336	147339384	147339178	147339238	147353322	147353373	7	10	6	1	0	0	0	1	0	0	97	49	1	1	0.78	1	1	NA	1	-0.073
SE	1869	SMAP1	SMAP1	chr6	+	70791688	70791769	70773349	70773425	70798656	70798737	1	0	2	3	2	0	0	1	0	0	98	49	1	1	0.143	0	1	NA	1	-0.619
SE	1921	XPO5	XPO5	chr6	-	43533906	43534007	43531478	43531575	43546570	43546752	3	0	1	0	0	0	0	0	0	1	98	49	1	1	1	NA	1	NA	0	1
SE	1986	SRPK1	SRPK1	chr6	-	35869009	35869110	35857260	35857368	35869481	35869901	49	15	8	2	1	0	0	5	0	0	98	49	1	1	0.925	0.882	1	NA	1	-0.064
SE	2459	DERL2	DERL2	chr17	-	5481295	5481389	5480386	5480582	5482808	5482882	4	1	4	0	0	5	0	1	0	0	98	49	1	1	1	1	0.286	NA	1	-0.238
SE	2461	EML3	EML3	chr11	-	62605641	62605773	62605112	62605180	62605854	62605980	13	5	1	1	0	0	0	1	0	0	98	49	1	1	0.867	1	1	NA	1	-0.044
SE	2598	EDC4	EDC4	chr16	+	67882678	67882865	67882428	67882594	67882957	67883177	11	2	1	1	0	0	0	1	0	0	98	49	1	1	0.846	1	1	NA	1	-0.051
SE	2599	NAA20	NAA20	chr20	+	20032507	20032653	20026783	20026919	20033101	20033629	1	0	0	0	0	0	0	3	0	1	98	49	1	1	1	NA	NA	NA	0.6	0.4
SE	3126	ZNF207	ZNF207	chr17	+	32366664	32366757	32365329	32365487	32367771	32368014	23	9	1	4	2	1	0	0	0	2	98	49	1	1	0.742	0.692	0.333	NA	0	0.589
SE	3142	DERL1	DERL1	chr8	-	123030604	123030716	123024985	123025050	123041969	123042423	8	8	5	0	1	1	0	2	0	0	98	49	1	1	1	0.8	0.714	NA	1	-0.162
SE	3330	WDR11	WDR11	chr10	+	120860108	120860282	120858642	120858796	120862734	120862921	6	0	4	0	0	0	0	2	0	1	98	49	1	1	1	NA	1	NA	0.5	0.5
SE	3331	WDR11	WDR11	chr10	+	120867065	120867169	120866568	120866764	120871169	120871346	4	5	2	0	0	0	0	3	0	1	98	49	1	1	1	1	1	NA	0.6	0.4
SE	3922	SPG11	SPG11	chr15	-	44572682	44572820	44570524	44570658	44583813	44584558	16	0	2	0	1	0	0	10	0	0	98	49	1	1	1	0	1	NA	1	-0.333
SE	3923	SPG11	SPG11	chr15	-	44589251	44589414	44585635	44585850	44592330	44592438	17	4	7	1	0	0	0	2	0	0	98	49	1	1	0.895	1	1	NA	1	-0.035
SE	3924	SPG11	SPG11	chr15	-	44598630	44598836	44598264	44598373	44600466	44600632	16	3	0	0	1	0	0	1	0	0	98	49	1	1	1	0.6	NA	NA	1	-0.2

SE	3925	SPG11	SPG11	chr15	-	44606024	44606091	44600466	44600632	44608443	44608605	26	14	1	6	0	1	0	3	0	2	98	49	1	1	0.684	1	0.333	NA		0.429	0.243
SE	3926	SPG11	SPG11	chr15	-	44613429	44613536	44610839	44610985	44615362	44615566	36	11	5	3	2	0	0	13	0	0	98	49	1	1	0.857	0.733	1	NA		1	-0.137
SE	4101	ZCCHC8	ZCCHC8	chr12	-	122482634	122482695	122481944	122482087	122489385	122489463	2	5	2	1	0	0	0	1	0	0	98	49	1	1	0.5	1	1	NA		1	-0.167
SE	4108	MLXIP	MLXIP	chr12	+	122130843	122130933	122129940	122130112	122132291	122132383	10	4	2	0	0	0	0	1	0	1	98	49	1	1	1	1	1	NA		0.333	0.667
SE	4303	PFDN5	PFDN5	chr12	+	53296243	53296275	53295838	53295941	53297849	53297924	43	7	8	2	0	0	0	8	0	0	81	49	1	1	0.929	1	1	NA		1	-0.024
SE	4508	FIG4	FIG4	chr6	+	109727108	109727265	109716444	109716568	109732636	109732687	3	4	0	1	0	0	1	3	0	0	98	49	1	1	0.6	1	NA		1	1	-0.2
SE	4882	FCHO2	FCHO2	chr5	+	73006444	73006549	72990711	72990864	73015625	73015724	19	7	1	1	0	0	0	6	0	0	98	49	1	1	0.905	1	1	NA		1	-0.032
SE	4884	FCHO2	FCHO2	chr5	+	73081782	73081982	73078179	73078312	73082760	73082825	15	9	11	0	1	0	2	0	0	0	98	49	1	1	1	0.818	1		1	NA	-0.061
SE	5023	AHCTF1	AHCTF1	chr1	-	246843794	246843928	246842693	246842776	246849614	246851442	12	1	5	3	1	1	0	0	0	2	98	49	1	1	0.667	0.333	0.714	NA		0	0.571
SE	5024	AHCTF1	AHCTF1	chr1	-	246857690	246857814	246855729	246855827	246860898	246861295	27	2	5	1	1	0	0	6	0	0	98	49	1	1	0.931	0.5	1	NA		1	-0.19
SE	5025	AHCTF1	AHCTF1	chr1	-	246863923	246864116	246861958	246862153	246867243	246867351	26	16	4	1	0	0	0	1	0	0	98	49	1	1	0.929	1	1	NA		1	-0.024
SE	5028	AHCTF1	AHCTF1	chr1	-	246916141	246916395	246913231	246913412	246918249	246918377	12	12	2	5	1	1	0	4	0	1	98	49	1	1	0.545	0.857	0.5	NA		0.667	-0.033
SE	5138	VPS35	VPS35	chr16	-	46668929	46669052	46662982	46663162	46671704	46671860	18	10	11	0	0	1	3	6	0	0	98	49	1	1	1	1	0.846		1	1	-0.051
SE	5377	SLC9A8	SLC9A8	chr20	+	49839540	49839599	49823060	49823141	49845035	49845119	31	5	3	0	0	0	0	8	0	1	98	49	1	1	1	1	1	NA		0.8	0.2
SE	5379	SLC9A8	SLC9A8	chr20	+	49849578	49849680	49845035	49845119	49855437	49855581	32	13	0	3	0	0	0	10	0	2	98	49	1	1	0.842	1	NA	NA		0.714	0.207
SE	5380	SLC9A8	SLC9A8	chr20	+	49850761	49850844	49849578	49849680	49855437	49855581	7	4	0	14	9	0	0	4	0	4	98	49	1	1	0.2	0.182	NA	NA		0.333	-0.142
SE	5381	SLC9A8	SLC9A8	chr20	+	49850809	49850844	49849578	49849680	49855437	49855581	7	6	0	14	9	0	0	4	0	4	84	49	1	1	0.226	0.28	NA	NA		0.368	-0.115
SE	5382	SLC9A8	SLC9A8	chr20	+	49874704	49874821	49864727	49864844	49877980	49878063	23	10	8	2	0	0	0	4	0	0	98	49	1	1	0.852	1	1	NA		1	-0.049
SE	5405	RASGRP1	RASGRP1	chr15	-	38501142	38501287	38500102	38500139	38502311	38502421	5	5	4	1	0	0	0	1	0	0	98	49	1	1	0.714	1	1	NA		1	-0.095
SE	5406	RASGRP1	RASGRP1	chr15	-	38503271	38503376	38502311	38502421	38505839	38505920	9	1	3	0	1	0	0	2	0	0	98	49	1	1	1	0.333	1	NA		1	-0.222
SE	5439	RASGRP4	RASGRP4	chr19	-	38414847	38414916	38413393	38413474	38417051	38417168	17	10	3	1	0	0	0	3	0	0	98	49	1	1	0.895	1	1	NA		1	-0.035
SE	5440	RASGRP4	RASGRP4	chr19	-	38414847	38415123	38413393	38413474	38417051	38417168	56	20	10	1	0	0	2	16	0	0	98	49	1	1	0.966	1	1		1	1	-0.011
SE	5556	TAF2	TAF2	chr8	-	119793365	119793451	119791323	119791459	119795531	119795631	10	3	1	0	0	0	0	1	0	2	98	49	1	1	1	1	1	NA		0.2	0.8
SE	5786	SRSF10	SRSF10	chr1	-	23971337	23971439	23964346	23967723	23971572	23971626	4	1	0	4	0	2	0	1	0	0	98	49	1	1	0.333	1	0	NA		1	-0.556
SE	5870	ZDHHC13	ZDHHC13	chr11	+	19142977	19143123	19117144	19117276	19146180	19146303	2	0	0	1	0	0	0	2	0	0	98	49	1	1	0.5	NA	NA	NA		1	-0.5
SE	5871	ZDHHC13	ZDHHC13	chr11	+	19166301	19166385	19165051	19165145	19170410	19170568	2	2	0	2	0	0	0	2	0	0	98	49	1	1	0.333	1	NA	NA		1	-0.334
SE	5999	CHD1L	CHD1L	chr1	+	147270931	147271005	147268781	147268878	147272170	147272281	0	2	0	0	0	0	0	1	0	1	98	49	1	1	NA	1	NA	NA		0.333	0.667
SE	6296	TMEM87A	TMEM87A	chr15	-	42272062	42272123	42267946	42268032	42273254	42273584	13	3	3	1	0	0	0	1	0	0	98	49	1	1	0.867	1	1	NA		1	-0.044
SE	6420	RALGAPA2	RALGAPA2	chr20	-	20396694	20396734	20389627	20393253	20412026	20412148	11	2	6	0	0	3	0	3	0	0	89	49	1	1	1	1	0.524	NA		1	-0.159
SE	6421	RALGAPA2	RALGAPA2	chr20	-	20524405	20524543	20520916	20521100	20524829	20524898	12	9	2	0	3	0	0	2	0	1	98	49	1	1	1	0.6	1	NA		0.5	0.367
SE	6422	RALGAPA2	RALGAPA2	chr20	-	20611314	20611426	20605174	20605412	20616042	20616191	33	4	0	0	0	0	0	6	0	1	98	49	1	1	1	1	NA	NA		0.75	0.25
SE	6423	RALGAPA2	RALGAPA2	chr20	-	20629362	20629590	20620462	20620630	20635417	20635617	22	21	4	1	0	0	0	6	0	0	98	49	1	1	0.917	1	1	NA		1	-0.028
SE	6424	RALGAPA2	RALGAPA2	chr20	-	20637362	20637501	20635417	20635617	20639784	20639900	37	17	18	1	4	0	0	13	0	2	98	49	1	1	0.949	0.68	1	NA		0.765	0.111
SE	6425	RALGAPA2	RALGAPA2	chr20	-	20676235	20676288	20653529	20653587	20680690	20680801	45	24	6	0	1	0	0	13	0	2	98	49	1	1	1	0.923	1	NA		0.765	0.209
SE	6429	LMBR1L	LMBR1L	chr12	-	49102886	49102951	49102467	49102540	49103090	49103159	7	3	1	0	1	0	1	4	0	0	98	49	1	1	1	0.6	1		1	1	-0.133
SE	6474	GBP5	GBP5	chr1	-	89262219	89262401	89258950	89260817	89263735	89263948	65	9	9	0	2	0	0	10	0	0	98	49	1	1	1	0.692	1	NA		1	-0.103
SE	6475	GBP5	GBP5	chr1	-	89262682	89262785	89262219	89262401	89263735	89263948	85	28	13	2	0	0	3	3	0	0	98	49	1	1	0.955	1	1		1	1	-0.015
SE	6476	GBP5	GBP5	chr1	-	89266956	89267153	89266345	89266588	89268728	89268856	62	19	18	1	0	0	0	7	0	0	98	49	1	1	0.969	1	1	NA		1	-0.01
SE	6477	GBP5	GBP5	chr1	-	89267416	89267526	89266956	89267153	89268728	89268856	136	36	23	1	0	0	2	14	0	0	98	49	1	1	0.986	1	1		1	1	-0.005
SE	6478	GBP5	GBP5	chr1	-	89268728	89268856	89267416	89267526	89269365	89269574	72	16	9	1	1	0	1	7	0	0	98	49	1	1	0.973	0.889	1		1	1	-0.046
SE	6479	GBP5	GBP5	chr1	-	89268728	89268856	89267416	89267526	89272451	89272861	48	11	5	1	1	0	1	3	0	0	98	49	1	1	0.96	0.846	1		1	1	-0.065
SE	6481	GBP5	GBP5	chr1	-	89269365	89269574	89268728	89268856	89272451	89272861	30	9	5	2	1	0	0	4	0	0	98	49	1	1	0.882	0.818	1	NA		1	-0.1
SE	6482	GBP5	GBP5	chr1	-	89270754	89270862	89269365	89269574	89272451	89272861	52	16	3	4	3	1	0	6	0	0	98	49	1	1	0.867	0.727	0.6	NA		1	-0.269
SE	6593	KANSL2	KANSL2	chr12	-	48679035	48679150	48671798	48671962	48679654	48679833	5	1	0	3	3	1	0	6	0	2	98	49	1	1	0.455	0.143	0	NA		0.6	-0.401
SE	6837	GAK	GAK	chr4	-	913606	913668	904636	904779	932042	932386	9	2	0	1	1	0	3	2	0	0	98	49	1	1	0.818	0.5	NA		1	1	-0.341
SE	6843	NAA16	NAA16	chr13	+	41331273	41331369	41328723	41328843	41355143	41355216	2	0	0	0	1	0	0	1	0	0	98	49	1	1	1	0	NA	NA		1	-0.5
SE	6917	TMEM41B	TMEM41B	chr11	-	9286454	9286593	9280654	9283593	9287701	9287806	6	10	0	1	0	0	0	1	0	0	98	49	1	1	0.75	1	NA	NA		1	-0.125
SE	6921	DAP3	DAP3	chr1	+	155729041	155729122	155727607	155727738	155729207	155729366	11	3	1	0	2	0	0	4	0	0	98	49	1	1	1	0.429	1	NA		1	-0.19
SE	7009	SFI1	SFI1	chr22	+	31528689	31528863	31508254	31508376	31546860	31546971	6	13	3	1	3	0	0	0	0	1	98	49	1	1	0.75	0.684	1	NA		0	0.811
SE	7011	SFI1	SFI1	chr22	+	31546860	31546971	31528689	31528863	31550253	31550348	7	13	2	1	0	0	0	1	0	0	98	49	1	1	0.778	1	1	NA		1	-0.074
SE	7012	SFI1	SFI1	chr22	+	31561289	31561392	31556941	31557059	31573057	31573214	22	16	3	1	0	0	0	4	0	0	98	49	1	1	0.917	1	1	NA		1	-0.028
SE	7014	SFI1	SFI1	chr22	+	31613601	31613855	31613353	31613530	31614788	31614860	9	5	7	1	0	0	2	0	0	0	98	49	1	1	0.818	1	1		1	NA	-0.061
SE	7318	VPS11	VPS11	chr11	+	119071595	119071843	119070233	119070397	119073197	119073399	10	0	0	0	0	0	0	0	0	2	98	49	1	1	1	NA	NA	NA		0	1
SE	7370	SMAP2	SMAP2	chr1	+	40406735	40406869	40373705	40374223	40408652	40408738	139	72	82	0	2	0	0	53	0	0	98	49	1	1	1	0.947	1	NA		1	-0.018
SE	7439	TBC1D32	TBC1D32	chr6	-	121170404	121170527	121160947	121161056	121205074	121205163	0	0	0	1	0	0	0	1	0	0	98	49	1	1	0	NA	NA	NA		1	-1
SE	7498	SLC12A6	SLC12A6	chr15	-	34238964	34239160	34238231	34238401	34240660	34240829	71	12	3	0	1	0	1	11	0	0	98	49	1	1	1	0.857	1		1	1	-0.048
SE	7499	SLC12A6	SLC12A6	chr15	-	34242101	34242221	34241232	34241337	34243973	34244072	67	19	8	7	5	0	0	7	0	1	98	49	1	1	0.827	0.655	1	NA		0.778	0.049
SE	7500	SLC12A6	SLC12A6	chr15	-	34245284	34245403	34243973	34244072	34245692	34245867	82	40	22	2	0	0	0	16	0	0	98	49	1	1	0.953	1	1	NA		1	-0.016
SE	7506	HAL	HAL	chr12	-	95990392	95990532	95988192	95988240	95992679	95992805	69	12	16	2	0	0	0	8	0	0	98	49	1	1	0.945	1	1	NA		1	-0.018
SE	7507	HAL	HAL	chr12	-	95993771	95993838	95993450	95993488	95993925	95993998	28	14	17	9	0	5	0	3	0	0	98	49	1	1	0.609	1	0.63	NA		1	-0.254
SE	7619	DRAM2	DRAM2	chr1	-	111124741	111124881	111120515	111120693	111126226	111126294	5	1	2	1	0	0	0	6	0	0	98	49	1	1	0.714	1	1	NA		1	-0.095
SE	7636	PTEN	PTEN	chr10	+	87925512	87925557	87894024	87894109	87931045	87931089	77	45	51	1	0	0	0	17	0	0	94	49	1	1	0.976	1	1	NA		1	-0.008
SE	7638	PTEN	PTEN	chr10	+	87952117	87952259	87933012	87933251	87957852	87958019	16	9	10	3	0	2	0	4	0	1	98	49	1	1	0.727	1	0.714	NA		0.667	0.147
SE	7761	PLCB3	PLCB3	chr11	+	64264020	64264112	64263690	64263795	64264950	64265104	0	0	0	1	0	0	0	2	0	0	98	49	1	1	0	NA	NA	NA		1	-1
SE	7946	CUL4A	CUL4A	chr13	+	113239432	113239551	113236822	113236890	113242967	113243160	7	2	0	0	0	0	0	5	0	1	98	49	1	1	1	1	NA	NA		0.714	0.286
SE	8088	HPS1	HPS1	chr10	-	98429790	98429889	98429145	98429642	98430570	98430670	14	1	2	0	0	3	0	1	0	1	98	49	1	1	1	1	0.25	NA		0.333	0.417
SE	8089	HPS1	HPS1	chr10	-	98431130	98431291	98430570	98430670	98433982	98434091	15	0	3	2	0	0	0	6	0	0	98	49	1	1	0.789	NA	1	NA		1	-0.105
SE	8090	HPS1	HPS1	chr10	-	98435271	98435414	98433982	98434091	98435634	98435772	15	1	2	7	0	0	0	7	0	0	98	49	1	1	0.517	1	1	NA		1	-0.161
SE	8130	CNEP1R1	CNEP1R1	chr16	+	50029724	50029798	50026395	50026467	50033396	50033506	13	7	4	0	3	0	0	6	0	0	98	49	1	1	1	0.538	1	NA		1	-0.154
SE	8235	ZFC3H1	ZFC3H1	chr12	-	71619314	71619409	71615205	71615316	71619925	71620124	12	2	3	1	0	0	0	2	0	0	98	49	1	1	0.857	1	1	NA		1	-0.048
SE	8236	ZFC3H1	ZFC3H1	chr12	-	71624103	71624292	71623359	71623597	71626259	71626446	8	0	1	0	0	0	0	0	0	2	98	49	1	1	1	NA	1	NA		0	1
SE	8237	ZFC3H1	ZFC3H1	chr12	-	71636849	71637059	71636489	71636654	71638417	71638515	27	17	1	0	0	0	0	3	0	1	98	49	1	1	1	1	1	NA		0.6	0.4
SE	8411	FCHO1	FCHO1	chr19	+	17755117	17755191	17754316	17754361	17762761	17762853	9	3	0	1	0	0	1	1	0	0	98	49	1	1	0.818	1	NA		1	1	-0.091
SE	8412	FCHO1	FCHO1	chr19	+	17781230	17781343	17778608	17778884	17781451	17781539	8	6	0	0	0	0	1	3	0	3	98	49	1	1	1	1	NA		1	0.333	0.334
SE	8527	KIAA0556	KIAA0556	chr16	+	27699533	27699599	27698327	27698500	27701588	27701695	14	3	0	2	0	0	2	3	0	0	98	49	1	1	0.778	1	NA		1	1	-0.111
SE	8528	KIAA0556	KIAA0556	chr16	+	27708704	27708920	27703895	27703998	27721557	27721695	11	1	0	1	0	0	0	1	0	0	98	49	1	1	0.846	1	NA	NA		1	-0.077
SE	8529	KIAA0556	KIAA0556	chr16	+	27754172	27754251	27751718	27751924	27761412	27761590	5	4	2	0	1	0	0	4	0	0	98	49	1	1	1	0.667	1	NA		1	-0.111
SE	8530	KIAA0556	KIAA0556	chr16	+	27777609	27777770	27776927	27777029	27777880	27777969	8	2	1	1	0	0	2	2	0	0	98	49	1	1	0.8	1	1		1	1	-0.067
SE	8537	ATG3	ATG3	chr3	-	112553279	112553329	112550191	112550262	112558375	112558417	31	9	9	1	3	0	0	4	0	3	98	49	1	1	0.939	0.6	1			0.4	0.446
SE	8551	UBR3	UBR3	chr2	+	169877493	169877637	169875790	169875949	169878524	169878574	27	11	0	1	0	0	0	3	0	0	98	49	1	1	0.931	1	NA	NA		1	-0.034
SE	8708	IFT88	IFT88	chr13	+	20615792	20615879	20605034	20605105	20625749	20625849	1	1	4	2	0	0	0	2	0	0	98	49	1	1	0.2	1	1	NA		1	-0.267
SE	8826	DOCK2	DOCK2	chr5	+	169670541	169670597	169669287	169669328	169671077	169671174	96	41	16	1	0	0	0	22	0	0	98	49	1	1	0.98	1	1	NA		1	-0.007
SE	8827	DOCK2	DOCK2	chr5	+	169671077	169671174	169670541	169670597	169674296	169674445	205	65	16	0	1	0	2	36	0	0	98	49	1	1	1	0.97	1		1	1	-0.01
SE	8829	DOCK2	DOCK2	chr5	+	169689251	169689333	169684195	169684350	169695802	169695938	176	92	10	2	0	0	5	49	0	0	98	49	1	1	0.978	1	1		1	1	-0.007
SE	8830	DOCK2	DOCK2	chr5	+	169712119	169712223	169711934	169712007	169714027	169714211	141	46	6	0	1	0	4	19	0	0	98	49	1	1	1	0.958	1		1	1	-0.014
SE	8833	DOCK2	DOCK2	chr5	+	169803057	169803206	169761518	169761625	169840756	169840852	143	72	20	0	2	0	2	21	0	0	98	49	1	1	1	0.947	1		1	1	-0.018
SE	8834	DOCK2	DOCK2	chr5	+	169983067	169983166	169840756	169840852	169985827	169985922	45	22	7	0	0	0	0	12	0	1	98	49	1	1	1	1	1	NA		0.857	0.143
SE	8835	DOCK2	DOCK2	chr5	+	170008687	170008746	170008496	170008597	170018959	170019108	74	29	11	0	1	0	2	21	0	0	98	49	1	1	1	0.935	1		1	1	-0.022
SE	8836	DOCK2	DOCK2	chr5	+	170034398	170034555	170027862	170027948	170036514	170036555	61	8	18	1	0	0	0	16	0	0	98	49	1	1	0.968	1	1	NA		1	-0.011
SE	8837	DOCK2	DOCK2	chr5	+	170045815	170045905	170042012	170042132	170047509	170047614	49	19	5	0	1	0	1	14	0	0	98	49	1	1	1	0.905	1		1	1	-0.032
SE	8923	DISC1	DISC1	chr1	+	231720847	231720924	231701954	231702024	231749925	231750619	1	0	0	4	7	3	0	0	0	3	98	49	1	1	0.111	0	0	NA		0	0.037
SE	8932	PLCB2	PLCB2	chr15	-	40290024	40290082	40289271	40289358	40290576	40290672	63	24	12	0	3	0	0	8	0	0	98	49	1	1	1	0.8	1	NA		1	-0.067
SE	8933	PLCB2	PLCB2	chr15	-	40291605	40291650	40291264	40291487	40291848	40291924	10	5	0	10	3	1	0	1	0	2	94	49	1	1	0.343	0.465	0	NA		0.207	0.062
SE	8934	PLCB2	PLCB2	chr15	-	40297876	40297959	40297520	40297605	40298222	40298380	59	15	5	1	0	0	1	6	0	0	98	49	1	1	0.967	1	1		1	1	-0.011
SE	9161	CHD4	CHD4	chr12	-	6582136	6582281	6581648	6581814	6582614	6582748	33	7	10	1	1	0	0	4	0	1	98	49	1	1	0.943	0.778	1	NA		0.667	0.24
SE	9162	CHD4	CHD4	chr12	-	6583197	6583378	6583026	6583113	6587383	6587559	12	5	5	0	1	0	0	5	0	0	98	49	1	1	1	0.714	1	NA		1	-0.095
SE	9163	CHD4	CHD4	chr12	-	6600925	6601053	6600533	6600669	6601288	6601530	24	21	1	2	0	0	0	7	0	0	98	49	1	1	0.857	1	1	NA		1	-0.048
SE	9191	C2CD3	C2CD3	chr11	-	74122987	74123135	74118227	74118382	74132843	74132972	7	0	0	1	0	0	0	5	0	0	98	49	1	1	0.778	NA	NA	NA		1	-0.222
SE	9192	C2CD3	C2CD3	chr11	-	74161398	74161556	74139604	74139828	74168343	74168613	8	2	0	0	0	0	0	2	0	1	98	49	1	1	1	1	NA	NA		0.5	0.5
SE	9293	WLS	WLS	chr1	-	68153516	68153653	68150187	68150356	68155098	68155260	54	15	2	1	0	0	0	9	0	0	98	49	1	1	0.964	1	1	NA		1	-0.012
SE	9611	CTNNBL1	CTNNBL1	chr20	+	37842338	37842419	37840101	37840199	37859898	37860036	3	0	0	0	0	0	0	2	0	1	98	49	1	1	1	NA	NA	NA		0.5	0.5
SE	9773	E2F4	E2F4	chr16	+	67194397	67194459	67192760	67192870	67194685	67194980	8	4	0	0	0	1	0	2	0	0	98	49	1	1	1	1	0	NA		1	-0.333
SE	9782	BTAF1	BTAF1	chr10	+	91953736	91953873	91951402	91951566	91956527	91956657	30	3	0	1	0	0	1	5	0	1	98	49	1	1	0.937	1	NA		1	0.714	0.112
SE	9783	BTAF1	BTAF1	chr10	+	91989153	91989580	91984200	91984404	91992118	91992309	19	2	2	0	1	0	0	2	0	0	98	49	1	1	1	0.5	1	NA		1	-0.167
SE	9784	BTAF1	BTAF1	chr10	+	92027129	92027300	92026591	92026751	92028789	92030323	19	1	0	0	0	0	0	4	0	3	98	49	1	1	1	1	NA			0.4	0.6
SE	9849	MYH9	MYH9	chr22	-	36303994	36304155	36302567	36302676	36305032	36305102	184	98	47	0	0	0	6	33	0	0	98	49	1	1	1	0.98	1		1	1	-0.007
SE	10285	PPP2R2D	PPP2R2D	chr10	+	131911561	131912598	131901237	131901330	131934457	131934555	1	0	0	6	6	0	0	0	0	3	98	49	1	1	0.077	0	NA	NA		0	0.038
SE	10286	PPP2R2D	PPP2R2D	chr10	+	131934457	131934555	131901237	131901330	131940030	131940196	15	9	0	1	2	0	0	6	0	0	98	49	1	1	0.882	0.692	NA	NA		1	-0.213
SE	10287	PPP2R2D	PPP2R2D	chr10	+	131934893	131935006	131934457	131934555	131940030	131940196	4	7	1	9	3	0	0	1	0	3	98	49	1	1	0.182	0.538	1	NA		0.143	0.43
SE	10350	DYNC1LI2	DYNC1LI2	chr16	-	66749196	66749313	66742437	66742668	66751272	66751346	25	12	0	0	0	0	1	6	0	1	98	49	1	1	1	1	NA		1	0.75	0.125
SE	10390	IP6K1	IP6K1	chr3	-	49738211	49738422	49732790	49732972	49786353	49786540	9	2	1	0	2	0	0	3	0	0	98	49	1	1	1	0.333	1	NA		1	-0.222
SE	10526	TMEM33	TMEM33	chr4	+	41939195	41939383	41938601	41938696	41943746	41943814	13	6	3	6	0	0	0	4	0	0	98	49	1	1	0.52	1	1	NA		1	-0.16
SE	10639	EED	EED	chr11	+	86266082	86266216	86264171	86264263	86276979	86277138	4	1	0	0	1	0	0	2	0	0	98	49	1	1	1	0.333	NA	NA		1	-0.334
SE	10710	RASGRP2	RASGRP2	chr11	-	64727295	64727360	64726910	64727131	64728862	64729042	36	5	10	0	0	0	0	7	1	0	98	49	1	1	1	1	1		0	1	0.5
SE	10834	ZDHHC5	ZDHHC5	chr11	+	57695919	57696043	57693782	57693915	57696760	57696873	17	5	8	0	0	0	0	0	0	1	98	49	1	1	1	1	1	NA		0	1
SE	10836	PEX16	PEX16	chr11	-	45915467	45915568	45914603	45914684	45915702	45915836	19	14	1	1	0	0	0	1	0	0	98	49	1	1	0.905	1	1	NA		1	-0.032
SE	10875	INPP5B	INPP5B	chr1	-	37940687	37940798	37931912	37932053	37943639	37943669	3	0	0	0	0	2	0	2	0	0	98	49	1	1	1	NA	0	NA		1	-0.5
SE	10937	TNPO1	TNPO1	chr5	+	72855773	72855923	72851243	72851319	72861807	72861914	18	6	6	1	0	0	0	1	0	0	98	49	1	1	0.9	1	1	NA		1	-0.033
SE	10939	TNPO1	TNPO1	chr5	+	72900005	72900081	72897055	72897151	72900973	72901073	8	3	3	0	0	0	0	0	0	1	98	49	1	1	1	1	1	NA		0	1
SE	11162	PHB2	PHB2	chr12	-	6967170	6967238	6966419	6966500	6967891	6968021	27	4	1	0	2	0	0	4	0	0	98	49	1	1	1	0.5	1	NA		1	-0.167
SE	11509	CEP164	CEP164	chr11	+	117338565	117338668	117335604	117335680	117344165	117344277	4	3	1	1	0	0	0	3	0	0	98	49	1	1	0.667	1	1	NA		1	-0.111
SE	11512	SBNO2	SBNO2	chr19	-	1111018	1111093	1110744	1110888	1111505	1111614	39	25	10	0	0	0	1	6	0	1	98	49	1	1	1	1	1		1	0.75	0.125
SE	11514	SBNO2	SBNO2	chr19	-	1113534	1113704	1112817	1112949	1114230	1114422	92	52	19	1	0	0	9	21	0	1	98	49	1	1	0.979	1	1		1	0.913	0.036
SE	11554	WDFY2	WDFY2	chr13	+	51660595	51660663	51584347	51584824	51675169	51675243	9	6	2	0	2	0	0	2	0	0	98	49	1	1	1	0.6	1	NA		1	-0.133
SE	11555	WDFY2	WDFY2	chr13	+	51719197	51719348	51703595	51703650	51727677	51727790	20	3	3	0	0	0	0	3	0	1	98	49	1	1	1	1	1	NA		0.6	0.4
SE	11556	WDFY2	WDFY2	chr13	+	51739048	51739175	51727677	51727790	51751309	51751415	7	7	2	1	0	0	0	1	0	0	98	49	1	1	0.778	1	1	NA		1	-0.074
SE	11957	CHD3	CHD3	chr17	+	7907600	7907702	7907352	7907488	7907893	7908019	8	0	2	2	1	1	0	0	0	2	98	49	1	1	0.667	0	0.5	NA		1	0.389
SE	12147	DCP2	DCP2	chr5	+	113003941	113004077	113001566	113001674	113007937	113008042	22	10	10	5	1	1	0	10	0	0	98	49	1	1	0.687	0.833	0.833	NA		1	-0.216
SE	12208	PCYT2	PCYT2	chr17	-	81908875	81909035	81908567	81908634	81909513	81909602	6	0	0	1	0	0	0	4	0	0	98	49	1	1	0.75	NA	NA	NA		1	-0.25
SE	12209	PCYT2	PCYT2	chr17	-	81908875	81909037	81908567	81908634	81909513	81909602	7	0	0	1	0	0	0	2	0	0	98	49	1	1	0.778	NA	NA	NA		1	-0.222
SE	12210	PCYT2	PCYT2	chr17	-	81908875	81909321	81908567	81908634	81909513	81909602	6	0	2	1	0	0	0	2	0	0	98	49	1	1	0.75	NA	1	NA		1	-0.125
SE	12338	MAPK1	MAPK1	chr22	-	21788693	21788808	21788256	21788388	21799011	21799128	17	3	28	0	0	0	0	7	0	1	98	49	1	1	1	1	1	NA		0.778	0.222
SE	12342	TRAPPC8	TRAPPC8	chr18	-	31913368	31913522	31909666	31909760	31916271	31916446	29	9	11	2	1	0	0	3	0	0	98	49	1	1	0.879	0.818	1	NA		1	-0.101
SE	12653	RNF123	RNF123	chr3	+	49712478	49712656	49706790	49706898	49713512	49713587	4	0	1	1	0	0	0	1	0	0	98	49	1	1	0.667	NA	1	NA		1	-0.166
SE	13109	GBP3	GBP3	chr1	-	89020531	89020743	89015286	89015414	89022683	89022866	8	1	0	1	0	0	1	0	0	0	98	49	1	1	0.8	1	NA		1	NA	-0.1
SE	13160	AP2A1	AP2A1	chr19	+	49800960	49801058	49799967	49800150	49801389	49801621	20	9	5	0	0	0	1	3	1	0	98	49	1	1	1	1	1		0.333	1	0.334
SE	13686	PQLC3	PQLC3	chr2	+	11171924	11172045	11164203	11164261	11174967	11175009	3	2	3	0	1	0	0	1	0	1	98	49	1	1	1	0.5	1	NA		0.333	0.5
SE	13809	HNRNPLL	HNRNPLL	chr2	-	38569803	38569925	38569132	38569334	38573209	38573427	4	6	1	1	0	0	0	2	0	0	98	49	1	1	0.667	1	1	NA		1	-0.111
SE	13829	EML4	EML4	chr2	+	42301240	42301392	42295380	42295516	42303103	42303229	17	11	2	1	0	0	0	6	0	0	98	49	1	1	0.895	1	1	NA		1	-0.035
SE	13861	TBCK	TBCK	chr4	-	106254946	106255047	106251865	106252007	106260436	106260510	0	0	0	1	0	0	0	3	0	0	98	49	1	1	0	NA	NA	NA		1	-1
SE	13933	TRAPPC9	TRAPPC9	chr8	-	140426614	140426641	140405576	140405698	140435111	140435240	5	4	0	2	0	0	0	4	0	0	76	49	1	1	0.617	1	NA	NA		1	-0.192
SE	14136	TMEM87B	TMEM87B	chr2	+	112077191	112077282	112074911	112074962	112081056	112081118	9	0	0	1	0	0	0	1	0	0	98	49	1	1	0.818	NA	NA	NA		1	-0.182
SE	14137	TMEM87B	TMEM87B	chr2	+	112081334	112081518	112081056	112081118	112086004	112086104	3	0	0	1	0	0	0	1	0	0	98	49	1	1	0.6	NA	NA	NA		1	-0.4
SE	14193	CNST	CNST	chr1	+	246591511	246591941	246566336	246566663	246621428	246621634	7	2	0	1	0	0	0	1	0	0	98	49	1	1	0.778	1	NA	NA		1	-0.111
SE	14441	INO80D	INO80D	chr2	-	206063150	206063244	206062798	206063045	206085900	206086182	6	0	0	1	0	0	0	2	0	0	98	49	1	1	0.75	NA	NA	NA		1	-0.25
SE	14502	ATG9A	ATG9A	chr2	-	219227921	219228053	219227769	219227813	219228433	219228485	8	3	0	1	0	0	0	1	0	0	98	49	1	1	0.8	1	NA	NA		1	-0.1
SE	14921	UBA3	UBA3	chr3	-	69057255	69057309	69056778	69056815	69061813	69061927	9	2	8	2	0	0	0	7	0	0	98	49	1	1	0.692	1	1	NA		1	-0.103
SE	14922	UBA3	UBA3	chr3	-	69071534	69071617	69067927	69068008	69075429	69075510	16	6	14	4	0	0	0	3	0	0	98	49	1	1	0.667	1	1	NA		1	-0.111
SE	14942	EIF3K	EIF3K	chr19	+	38620336	38620435	38619073	38619327	38624076	38624197	31	7	4	0	0	0	0	8	0	1	98	49	1	1	1	1	1	NA		0.8	0.2
SE	14943	EIF3K	EIF3K	chr19	+	38632600	38632678	38632429	38632496	38634992	38635118	30	9	10	0	0	0	0	5	0	1	98	49	1	1	1	1	1	NA		0.714	0.286
SE	15062	MORC3	MORC3	chr21	+	36356619	36356724	36349310	36349408	36359954	36360077	18	6	11	1	0	0	0	2	0	0	98	49	1	1	0.9	1	1	NA		1	-0.033
SE	15094	TRAPPC10	TRAPPC10	chr21	+	44037791	44037927	44032090	44032172	44052279	44052476	14	13	12	0	3	0	0	5	0	0	98	49	1	1	1	0.684	1	NA		1	-0.105
SE	15095	TRAPPC10	TRAPPC10	chr21	+	44074323	44074470	44063537	44063785	44075038	44075153	16	12	2	0	2	0	0	4	0	0	98	49	1	1	1	0.75	1	NA		1	-0.083
SE	15165	LZTR1	LZTR1	chr22	+	20989624	20989682	20988788	20988872	20990385	20990525	3	0	0	0	3	0	0	4	0	0	98	49	1	1	1	0	NA	NA		1	-0.5
SE	15226	DIAPH1	DIAPH1	chr5	-	141529602	141529697	141529171	141529273	141534334	141534433	20	17	0	2	2	0	0	8	0	0	98	49	1	1	0.833	0.81	NA	NA		1	-0.178

SE	15227	DIAPH1	DIAPH1	chr5	-	141583484	141583615	141583205	141583292	141584123	141584225	41	10	5	2	0	0	3	26	0	0	98	49	1	1	0.911	1	1	1	1	-0.03
SE	15228	DIAPH1	DIAPH1	chr5	-	141588223	141588250	141587041	141587197	141618797	141619055	0	1	0	16	10	3	0	0	0	7	76	49	1	1	0	0.061	0	NA	0	0.02
SE	15459	RAF1	RAF1	chr3	-	12600151	12600279	12599690	12599808	12600387	12600415	162	48	33	2	1	0	2	19	0	0	98	49	1	1	0.976	0.96	1	1	1	-0.021
SE	15460	RAF1	RAF1	chr3	-	12606200	12606299	12604135	12604289	12608765	12608923	154	59	46	39	3	3	0	20	0	5	98	49	1	1	0.664	0.908	0.885	NA	0.667	0.152
SE	15530	DYNC1LI1	DYNC1LI1	chr3	-	32530283	32530328	32529539	32529660	32530460	32530520	49	39	10	1	0	0	1	7	0	0	94	49	1	1	0.962	1	1	1	1	-0.013
SE	15531	DYNC1LI1	DYNC1LI1	chr3	-	32544875	32545106	32541036	32541206	32545848	32545965	22	4	10	2	0	0	0	4	0	0	98	49	1	1	0.846	1	1	NA	1	-0.051
SE	15532	DYNC1LI1	DYNC1LI1	chr3	-	32545848	32545965	32541036	32541206	32570345	32570419	15	9	6	2	0	0	0	2	0	0	98	49	1	1	0.789	1	1	NA	1	-0.07
SE	15599	SACM1L	SACM1L	chr3	+	45735234	45735373	45732052	45732151	45737582	45737652	16	5	0	1	0	0	0	2	0	0	98	49	1	1	0.889	1	NA	NA	1	-0.055
SE	15751	GBE1	GBE1	chr3	-	81646391	81646482	81642780	81642990	81648855	81648991	8	2	3	1	0	0	0	0	0	1	98	49	1	1	0.8	1	1	NA	0	0.933
SE	15752	GBE1	GBE1	chr3	-	81705443	81705613	81670837	81670953	81761374	81761799	5	6	1	0	1	0	1	1	0	0	98	49	1	1	1	0.75	1	1	1	-0.083
SE	16190	HTT	HTT	chr4	+	3140509	3140656	3136225	3136326	3142765	3142886	8	0	0	1	0	0	0	4	0	0	98	49	1	1	0.8	NA	NA	NA	1	-0.2
SE	16215	WDR1	WDR1	chr4	-	10077752	10077926	10077303	10077448	10078890	10079001	83	27	26	2	0	0	12	27	0	0	98	49	1	1	0.954	1	1	1	1	-0.015
SE	16217	WDR1	WDR1	chr4	-	10088663	10088741	10088292	10088373	10097710	10097891	55	15	17	2	0	0	3	4	0	0	98	49	1	1	0.932	1	1	1	1	-0.023
SE	16218	WDR1	WDR1	chr4	-	10097710	10097891	10088663	10088741	10116112	10116234	37	7	7	2	0	0	2	3	0	0	98	49	1	1	0.902	1	1	1	1	-0.033
SE	16219	WDR1	WDR1	chr4	-	10098991	10099139	10097710	10097891	10116112	10116234	35	23	17	2	0	0	1	6	0	0	98	49	1	1	0.897	1	1	1	1	-0.034
SE	16220	WDR1	WDR1	chr4	-	10103895	10103986	10088663	10088741	10116112	10116234	36	16	13	2	0	0	4	6	0	0	98	49	1	1	0.9	1	1	1	1	-0.033
SE	16221	WDR1	WDR1	chr4	-	10103895	10103986	10098991	10099139	10116112	10116234	64	30	25	0	0	0	5	16	0	1	98	49	1	1	1	1	1	1	0.889	0.055
SE	16244	TAPT1	TAPT1	chr4	-	16213767	16213898	16191360	16191523	16226258	16226538	2	2	0	1	0	0	0	1	0	0	98	49	1	1	0.5	1	NA	NA	1	-0.25
SE	16632	HNRNPM	HNRNPM	chr19	+	8483157	8483211	8474166	8474244	8485602	8486405	9	3	0	2	0	0	0	2	0	0	98	49	1	1	0.692	1	NA	NA	1	-0.154
SE	16761	SLC30A5	SLC30A5	chr5	+	69128003	69128132	69123198	69123425	69129446	69131072	6	0	2	1	0	0	0	1	0	0	98	49	1	1	0.75	NA	1	NA	1	-0.125
SE	16976	IK	IK	chr5	+	140655828	140655992	140654680	140654727	140657553	140657662	30	7	6	3	0	0	0	2	0	2	98	49	1	1	0.833	1	1	NA	0.333	0.611
SE	16982	HARS2	HARS2	chr5	+	140696102	140696201	140695737	140695845	140696520	140696614	7	2	1	1	0	0	0	1	0	0	98	49	1	1	0.778	1	1	NA	1	-0.074
SE	17117	MCMBP	MCMBP	chr10	-	119836895	119837029	119835539	119835704	119838534	119838700	17	9	27	1	0	0	0	12	0	0	98	49	1	1	0.895	1	1	NA	1	-0.035
SE	17118	MCMBP	MCMBP	chr10	-	119842471	119842595	119840842	119840960	119843247	119843426	2	5	14	0	0	0	0	0	1	0	98	49	1	1	1	1	1	0	NA	1
RI	474	TMEM41B	TMEM41B	chr11	-	9280654	9283593	9280654	9281041	9283370	9283593	8	2	0	1	1	0	0	2	0	0	98	49	1	1	0.8	0.5	NA	NA	1	-0.35
RI	1010	TAF1C	TAF1C	chr16	-	84181327	84181663	84181327	84181463	84181591	84181663	8	1	0	0	0	0	0	3	0	1	98	49	1	1	1	1	NA	NA	0.6	0.4
RI	1110	HNRNPM	HNRNPM	chr19	+	8465323	8465515	8465323	8465362	8465479	8465515	13	6	1	5	2	3	0	8	0	1	98	49	1	1	0.565	0.6	0.143	NA	0.8	-0.364

MXE_all

													Inclusion counts			Skipping counts			Inclusion counts		Skipping counts						Inclusion_level(normalized)					
ID	Gene_ID	Gene_Symbol	Chromosome	Strand	1stExonStart_0base	1stExonEnd	2ndExonStart_0base	2ndExonEnd	UpstreamES	UpstreamEE	DownstreamES	DownstreamEE	control_s3	control_s4	control_s10	control_s3	control_s4	control_s10	Idg_14	Ildg_s16	Idg_s14	Idg_s16	IncFormLen	SkipFormLen	p-value	adjust_p	IncLevel_s3	IncLevel_s4	IncLevel_s10	IncLevel14	IncLevel16	Level_diff
767	ATP5C1	ATP5C1	chr10	+	7802757	7802854	7806973	7807010	7802269	7802425	7807658	7807799	20	7	2	3	1	3	0	0	1	8	98	86	9.92E-09	6.58E-06	0.854	0.86	0.369	0	0	0.694
69	CPQ	CPQ	chr8	+	96834972	96835180	96879797	96880005	96784863	96785330	96965934	96966046	33	16	7	24	15	5	1	9	0	0	98	98	1.45E-06	0.000481929	0.579	0.516	0.583	1	1	-0.441
973	LST1	LST1	chr6	+	31587640	31587733	31587943	31587966	31587199	31587318	31588496	31588909	39	24	22	9	9	2	0	0	1	1	98	72	1.71E-05	0.00253914	0.761	0.662	0.89	0	0	0.771
700	RFWD2	RFWD2	chr1	-	176136487	176136547	176175909	176176007	176135009	176135086	176184632	176184692	32	16	3	89	32	10	0	0	1	10	98	98	1.93E-05	0.00253914	0.264	0.333	0.231	0	0	0.276
1169	ARID3A	ARID3A	chr19	+	964832	965080	966571	966868	964247	964431	968404	968503	8	3	3	17	4	3	1	1	0	0	98	98	2.60E-05	0.00253914	0.32	0.429	0.5	1	1	-0.584
983	IRF7	IRF7	chr11	-	613950	614037	614173	614399	613784	613865	614475	614534	2	3	7	13	11	0	0	0	8	14	98	98	2.64E-05	0.00253914	0.133	0.214	1	0	0	0.449
814	MAEA	MAEA	chr4	+	1322380	1322503	1327626	1327703	1315396	1315600	1332756	1332865	11	5	2	12	5	7	0	0	3	2	98	98	2.68E-05	0.00253914	0.478	0.5	0.222	0	0	0.4
672	SLBP	SLBP	chr4	-	1703595	1703700	1711873	1711990	1700010	1700070	1712129	1712741	1	0	1	10	6	0	1	2	0	1	98	98	4.66E-05	0.003870084	0.091	0	1	1	0.667	-0.47
933	DUSP1	DUSP1	chr5	-	172769574	172769794	172770160	172770306	172768089	172769132	172770585	172771200	561	303	269	438	200	178	93	267	6	110	98	98	7.32E-05	0.005400629	0.562	0.602	0.602	0.939	0.708	-0.235
677	STK40	STK40	chr1	-	36358736	36358822	36361220	36361340	36358238	36358382	36385722	36385927	19	8	3	30	18	9	0	0	1	3	98	98	9.01E-05	0.005983417	0.388	0.308	0.25	0	0	0.315
1210	CHD2	CHD2	chr15	+	92944414	92944515	92945820	92945865	92942842	92943068	92946037	92946216	23	3	0	44	13	8	0	0	2	8	98	94	0.000158716	0.009580676	0.334	0.181	0	0	0	0.172
887	ZNF467	ZNF467	chr7	-	149769089	149769200	149770439	149770556	149764363	149766239	149770998	149771074	8	1	0	9	3	5	0	0	2	1	98	98	0.000367755	0.020349121	0.471	0.25	0	0	0	0.24
603	C1orf43	C1orf43	chr1	-	154213892	154213946	154214456	154214574	154212457	154212624	154220341	154220797	4	2	0	7	6	7	1	2	0	1	98	98	0.000476501	0.024338219	0.364	0.25	0	1	0.667	-0.629
275	SEC11A	SEC11A	chr15	-	84680712	84680832	84687624	84687774	84670724	84670782	84691534	84691644	15	4	20	3	2	4	2	2	0	0	98	98	0.000719589	0.030060115	0.833	0.667	0.833	1	1	-0.222
1520	ALPL	ALPL	chr1	+	21553977	21554142	21560625	21560745	21509364	21509517	21563109	21563284	25	7	6	5	4	0	2	6	0	0	98	98	0.000721258	0.030060115	0.833	0.636	1	1	1	-0.177
720	APH1A	APH1A	chr1	-	150267715	150267789	150267956	150267991	150267355	150267478	150268697	150269209	2	3	1	11	2	6	0	0	1	1	84	98	0.00072434	0.030060115	0.175	0.636	0.163	0	0	0.325
1140	SAP30BP	SAP30BP	chr17	+	75671815	75671863	75693439	75693482	75668515	75668625	75699782	75699871	25	6	5	15	5	5	1	0	1	4	97	92	0.000838883	0.03276579	0.613	0.532	0.487	0.487	0	0.301
439	KIAA1033	KIAA1033	chr12	+	105160000	105160148	105162748	105162845	105157235	105157322	105164110	105164307	44	7	1	8	8	2	1	8	0	1	98	98	0.001281181	0.047261349	0.846	0.467	0.333	1	0.889	-0.396
347	PLEKHB2	PLEKHB2	chr2	+	131126683	131126786	131132901	131132991	131125752	131125905	131140166	131140275	24	9	2	18	5	2	1	0	1	3	98	98	0.001418793	0.048685792	0.571	0.643	0.5	0.5	0	0.321
272	FCAR	FCAR	chr19	+	54885234	54885525	54888006	54888294	54874092	54874323	54889648	54891420	4	10	0	28	6	2	0	1	4	17	98	98	0.001508121	0.048685792	0.125	0.625	0	0	0.056	0.222
1172	GADD45B	GADD45B	chr19	+	2476528	2476630	2477028	2477251	2476124	2476402	2477487	2478259	29	30	14	22	26	14	7	19	1	5	98	98	0.001539761	0.048685792	0.569	0.536	0.5	0.875	0.792	-0.298
866	CDC42SE2	CDC42SE2	chr5	+	131315975	131316144	131359208	131359547	131264008	131264166	131385542	131385644	9	2	0	50	14	10	0	0	1	9	98	98	0.001681804	0.050759907	0.153	0.125	0	0	0	0.093
605	GBP5	GBP5	chr1	-	89268728	89268856	89269365	89269574	89267416	89267526	89272451	89272861	5	4	1	48	11	5	0	0	1	3	98	98	0.001832725	0.05290996	0.094	0.267	0.167	0	0	0.176
1043	THEMIS2	THEMIS2	chr1	+	27879643	27880054	27881970	27883043	27876587	27876728	27885294	27885451	111	23	15	25	14	1	6	25	0	2	98	98	0.002730415	0.075541495	0.816	0.622	0.937	1	0.926	-0.171
752	ARHGEF7	ARHGEF7	chr13	+	111205288	111205373	111209871	111210002	111153713	111153991	111217678	111217880	4	2	0	6	1	1	2	1	0	1	98	98	0.002961552	0.078658825	0.4	0.667	0	1	0.5	-0.394
924	NADK	NADK	chr1	-	1753566	1753649	1754050	1754208	1751231	1753060	1754283	1754383	60	35	36	54	19	38	2	6	7	15	98	98	0.00311261	0.079491271	0.526	0.648	0.486	0.222	0.286	0.299
1492	GPSM3	GPSM3	chr6	-	32191708	32191908	32192147	32192250	32190765	32191503	32192471	32192530	20	13	8	50	23	25	1	1	3	14	98	98	0.004345138	0.106858211	0.286	0.361	0.242	0.25	0.067	0.138
812	MAEA	MAEA	chr4	+	1315396	1315600	1322380	1322503	1311978	1312161	1327626	1327703	15	13	2	1	4	1	2	1	0	0	98	98	0.005546739	0.13153696	0.937	0.765	0.667	1	1	-0.21
1232	TSEN34	TSEN34	chr19	+	54191360	54191607	54191720	54191964	54190267	54190450	54192115	54192373	3	7	3	44	21	17	0	1	3	12	98	98	0.007192046	0.164673055	0.064	0.25	0.15	0	0.077	0.116
1156	SLC11A1	SLC11A1	chr2	+	218382959	218383102	218384242	218384365	218382028	218382375	218385146	218385266	27	17	0	106	58	23	0	2	2	27	98	98	0.007791047	0.172441837	0.203	0.227	0	0	0.069	0.109
694	MKL1	MKL1	chr22	-	40423535	40423685	40424205	40424381	40420846	40421100	40429605	40429767	10	3	1	5	6	0	2	1	0	1	98	98	0.008293126	0.177633408	0.667	0.333	1	1	0.5	-0.083
995	NFKBIA	NFKBIA	chr14	-	35402770	35402859	35403149	35403360	35402393	35402663	35403689	35403798	77	218	23	78	178	23	0	24	8	46	98	98	0.008601596	0.178483112	0.497	0.551	0.5	0	0.343	0.345
580	LIMK2	LIMK2	chr22	+	31265945	31266132	31266983	31267070	31262594	31262791	31267775	31267907	24	9	2	68	37	9	2	8	0	6	98	98	0.008893756	0.178953152	0.261	0.196	0.182	1	0.571	-0.573
469	PLEKHM2	PLEKHM2	chr1	+	15719733	15719920	15721328	15721388	15718537	15718625	15725316	15725545	10	6	5	2	2	1	1	2	0	0	98	98	0.00958895	0.187266563	0.833	0.75	0.833	1	1	-0.195
478	ZRANB1	ZRANB1	chr10	+	124974199	124974398	124981708	124981829	124973644	124973716	124983174	124983304	9	0	3	12	10	2	0	1	1	2	98	98	0.010790876	0.204718338	0.429	0	0.6	0	0.333	0.176
128	IKZF1	IKZF1	chr7	+	50387344	50387470	50391728	50391863	50382539	50382707	50399917	50405100	25	3	4	6	2	0	2	7	0	1	98	98	0.013093288	0.241498429	0.806	0.6	1	1	0.875	-0.135
428	CLEC7A	CLEC7A	chr12	-	10123244	10123363	10125086	10125173	10116780	10118590	10125296	10125448	26	2	10	141	62	38	3	18	0	38	98	98	0.014111252	0.25323976	0.156	0.031	0.208	1	0.321	-0.529
1058	C18orf8	C18orf8	chr18	+	23504370	23504447	23509192	23509279	23503469	23503720	23515855	23515996	1	7	0	15	5	2	4	3	0	5	98	98	0.026036619	0.454955651	0.062	0.583	0	1	0.375	-0.473
654	MVP	MVP	chr16	+	29841914	29842112	29844492	29844879	29841595	29841840	29845862	29845979	31	8	7	30	16	18	4	10	1	6	98	98	0.027787914	0.473107055	0.508	0.333	0.28	0.8	0.625	-0.339
986	AKNA	AKNA	chr9	-	114376465	114377532	114381059	114381446	114374092	114374167	114394356	114394405	13	9	1	56	16	8	0	2	2	13	98	98	0.030058921	0.498978088	0.188	0.36	0.111	0	0.133	0.153
111	ORAI2	ORAI2	chr7	+	102436224	102436333	102438943	102439181	102433529	102433661	102446512	102456821	1	4	0	11	6	5	0	0	2	2	98	98	0.032538655	0.526967491	0.083	0.4	0	0	0	0.161
1371	SHKBP1	SHKBP1	chr19	+	40582350	40582466	40583397	40583485	40580745	40580936	40583600	40583717	65	23	14	107	42	23	1	10	1	5	98	98	0.038106412	0.600128823	0.378	0.354	0.378	0.5	0.667	-0.214
743	TNRC6B	TNRC6B	chr22	+	40264687	40267036	40270121	40270280	40261831	40262173	40273424	40273600	27	14	0	6	2	1	1	4	0	1	98	98	0.038863764	0.600128823	0.818	0.875	0	1	0.8	-0.336
433	ASAP1	ASAP1	chr8	-	130091972	130092143	130112093	130112322	130079901	130079971	130115627	130115735	37	7	0	5	5	2	0	12	1	0	98	98	0.040307474	0.608276428	0.881	0.583	0	0	1	-0.012
774	SULF2	SULF2	chr20	-	47702518	47702670	47736702	47736942	47690125	47690295	47757188	47757463	32	7	4	7	5	3	6	15	0	3	98	98	0.042284294	0.623928249	0.821	0.583	0.571	1	0.833	-0.258
744	LCOR	LCOR	chr10	+	96944112	96944245	96949007	96949295	96907667	96907747	96952102	96952196	7	2	3	11	4	1	2	0	0	1	98	98	0.043446552	0.627141534	0.389	0.333	0.75	1	0	-0.009
996	GZMH	GZMH	chr14	-	24607148	24607406	24607611	24607747	24606479	24606746	24608264	24608412	8	9	0	7	8	4	1	0	0	3	98	98	0.046773203	0.657258859	0.533	0.529	0	1	0	-0.146
438	UBE2D1	UBE2D1	chr10	+	58361494	58361526	58363608	58363686	58361337	58361401	58364770	58364876	12	0	5	33	4	13	0	2	1	13	81	98	0.047512689	0.657258859	0.306	0	0.318	0	0.157	0.13
382	SART3	SART3	chr12	-	108543027	108543152	108544426	108544478	108538933	108539089	108545138	108545323	5	6	1	6	0	8	1	0	0	1	98	98	0.048879289	0.662364246	0.455	1	0.111	1	0	0.022
788	ATP2A3	ATP2A3	chr17	-	3953346	3953429	3953692	3953710	3951580	3951685	3964173	3964464	6	4	5	19	13	6	1	2	1	1	67	98	0.052742755	0.70042378	0.316	0.31	0.549	0.594	0.745	-0.278
403	USP15	USP15	chr12	+	62325871	62325933	62349220	62349307	62321463	62321609	62355330	62355475	193	56	26	80	33	23	2	22	0	6	98	98	0.055556301	0.713311355	0.707	0.629	0.531	1	0.786	-0.271
430	CLEC7A	CLEC7A	chr12	-	10125296	10125448	10127746	10127845	10123244	10123363	10129979	10130269	199	74	36	52	26	13	6	44	0	8	98	98	0.055861733	0.713311355	0.793	0.74	0.735	1	0.846	-0.167
60	DENND3	DENND3	chr8	+	141190283	141190417	141192330	141192449	141188985	141189146	141192525	141192663	38	11	3	29	8	6	3	9	0	4	98	98	0.056978495	0.71384378	0.567	0.579	0.333	1	0.692	-0.353
1017	PHF21A	PHF21A	chr11	-	45945839	45946003	45946075	45946098	45938156	45938312	45948885	45948946	7	6	0	16	2	1	1	0	0	4	72	98	0.058600631	0.720570725	0.373	0.803	0	1	0	-0.108
1519	ALPL	ALPL	chr1	+	21553977	21554142	21560625	21560745	21509364	21509517	21561096	21561212	25	7	6	57	25	15	2	6	3	3	98	98	0.067066433	0.79747995	0.305	0.219	0.286	0.4	0.667	-0.264
638	JMJD1C	JMJD1C	chr10	-	63219877	63219983	63264650	63264764	63217206	63217331	63268662	63269223	152	34	22	28	10	1	1	15	0	2	98	98	0.067819904	0.79747995	0.844	0.773	0.957	1	0.882	-0.083
1024	ITGAX	ITGAX	chr16	+	31355892	31355998	31356624	31356728	31355133	31355291	31357030	31357101	12	18	3	112	81	16	0	4	9	38	98	98	0.068458369	0.79747995	0.097	0.182	0.158	0	0.095	0.098
251	PCBP2	PCBP2	chr12	+	53464759	53464852	53465931	53465973	53462480	53462567	53467220	53467293	54	17	19	72	31	6	0	13	2	30	98	91	0.073459606	0.821205033	0.411	0.337	0.746	0	0.287	0.355
1149	RNF213	RNF213	chr17	+	80361733	80361888	80363101	80363314	80360060	80360206	80363608	80363790	43	13	9	66	30	7	1	27	14	63	98	98	0.074169176	0.821205033	0.394	0.302	0.562	0.067	0.3	0.236
527	SERPINA1	SERPINA1	chr14	-	94382591	94383241	94388800	94389010	94376746	94378640	94390456	94390692	47	13	5	108	35	38	1	15	2	20	98	98	0.074205274	0.821205033	0.303	0.271	0.116	0.333	0.429	-0.151
241	CYTH4	CYTH4	chr22	+	37310987	37311064	37311455	37311527	37309211	37309323	37312019	37312174	82	23	19	55	41	44	2	12	2	5	98	98	0.076746722	0.835406946	0.599	0.359	0.302	0.5	0.706	-0.183
930	DBNL	DBNL	chr7	+	44050224	44050280	44052866	44052941	44044639	44044820	44056756	44056903	11	3	5	24	6	9	1	1	2	5	98	98	0.083414571	0.893343144	0.314	0.333	0.357	0.333	0.167	0.085
550	SORL1	SORL1	chr11	+	121470006	121470123	121478117	121478243	121452202	121452616	121488031	121488193	174	85	67	166	80	53	6	35	0	23	98	98	0.086250776	0.896644508	0.512	0.515	0.558	1	0.603	-0.273
832	DOCK2	DOCK2	chr5	+	169684195	169684350	169689251	169689333	169681743	169681879	169695802	169695938	109	35	17	83	48	5	2	13	3	21	98	98	0.086423567	0.896644508	0.568	0.422	0.773	0.4	0.382	0.197
330	CTSS	CTSS	chr1	-	150751780	150752008	150755000	150755150	150750005	150750171	150757857	150757980	412	132	123	268	149	97	2	126	1	73	98	98	0.091023357	0.929838596	0.606	0.47	0.559	0.667	0.633	-0.105
1114	CALCOCO2	CALCOCO2	chr17	+	48849251	48849377	48851088	48851177	48848321	48848455	48851558	48851628	15	15	10	11	5	3	2	4	0	2	98	98	0.095466674	0.960452598	0.577	0.75	0.769	1	0.667	-0.135
747	RNF19B	RNF19B	chr1	-	32938396	32938528	32942251	32942459	32936445	32937259	32944018	32944159	55	25	38	43	11	11	1	19	0	5	98	98	0.098734743	0.978505508	0.561	0.694	0.776	1	0.792	-0.219
1187	CYP4F3	CYP4F3	chr19	+	15643906	15644051	15645718	15645863	15641414	15641613	15647051	15647105	2	3	5	161	45	36	0	1	5	21	98	98	0.10473881	1	0.012	0.062	0.122	0	0.045	0.043
213	HLA-E	HLA-E	chr6	+	30491536	30491653	30492403	30492436	30491136	30491412	30492540	30492583	302	247	202	143	135	125	12	185	7	64	98	82	0.10716204	1	0.639	0.605	0.575	0.589	0.707	-0.042
524	SERPINA1	SERPINA1	chr14	-	94379463	94379611	94382591	94383241	94376746	94378640	94388559	94388660	70	15	37	142	66	104	0	13	6	51	98	98	0.113078522	1	0.33	0.185	0.262	0	0.203	0.158
548	MYD88	MYD88	chr3	+	38139863	38139998	38140387	38140568	38138477	38139028	38140756	38140848	50	12	5	12	1	3	6	12	3	7	98	98	0.117799117	1	0.806	0.923	0.625	0.667	0.632	0.135
1008	MYO1F	MYO1F	chr19	-	8545649	8545736	8548035	8548122	8544296	8544464	8548236	8548317	127	112	51	164	100	31	3	27	5	39	98	98	0.118730396	1	0.436	0.528	0.622	0.375	0.409	0.137
556	FOS	FOS	chr14	+	75279876	75280128	75280259	75280667	75278777	75279123	75280782	75282234	619	124	110	881	177	91	6	55	12	92	98	98	0.121335262	1	0.413	0.412	0.547	0.333	0.374	0.104
637	JMJD1C	JMJD1C	chr10	-	63186214	63186383	63189167	63189446	63185562	63185653	63190893	63191108	140	33	16	63	23	6	3	18	2	3	98	98	0.122157015	1	0.69	0.589	0.727	0.6	0.857	-0.06
768	PECAM1	PECAM1	chr17	-	64329699	64329722	64341633	64341690	64319415	64323845	64348259	64348322	21	26	28	56	21	12	0	6	1	12	98	72	0.123750931	1	0.216	0.476	0.632	0	0.269	0.307
847	DENND5A	DENND5A	chr11	-	9145669	9145815	9147029	9147151	9144994	9145113	9150080	9150209	26	15	10	43	12	8	4	5	0	5	98	98	0.125778306	1	0.377	0.556	0.556	1	0.5	-0.254
869	VIM	VIM	chr10	+	17235845	17235889	17236293	17236379	17235168	17235389	17237229	17237593	146	40	21	218	59	45	0	65	7	135	98	93	0.129220765	1	0.414	0.417	0.33	0	0.337	0.218
639	JMJD1C	JMJD1C	chr10	-	63219877	63219983	63264650	63264764	63217206	63217331	63380317	63380482	21	6	5	29	10	1	1	2	0	2	98	98	0.132304353	1	0.42	0.375	0.833	1	0.5	-0.207
303	RNASET2	RNASET2	chr6	-	166934090	166934136	166938894	166939008	166931043	166931118	166943018	166943089	162	68	52	119	30	59	1	31	0	12	98	95	0.132843371	1	0.569	0.687	0.461	1	0.715	-0.285
1522	VASP	VASP	chr19	+	45517928	45518094	45521321	45521406	45517662	45517834	45522167	45522217	121	49	30	98	38	44	7	22	0	20	98	98	0.139102038	1	0.553	0.563	0.405	1	0.524	-0.255
490	NCF2	NCF2	chr1	-	183566919	183566988	183567203	183567345	183565703	183565779	183569141	183569185	111	80	66	180	77	88	1	25	12	35	98	98	0.143527964	1	0.381	0.51	0.429	0.077	0.417	0.193
553	SORL1	SORL1	chr11	+	121545242	121545429	121549959	121550088	121543547	121543726	121550584	121550670	254	80	55	196	49	36	4	41	0	21	98	98	0.154456522	1	0.564	0.62	0.604	1	0.661	-0.235
289	XPO6	XPO6	chr16	-	28180940	28181031	28208986	28209122	28177219	28177332	28211365	28211918	8	6	1	338	139	60	1	0	3	55	98	98	0.159407357	1	0.023	0.041	0.016	0.25	0	-0.098
1521	VASP	VASP	chr19	+	45517662	45517834	45522167	45522217	45507429	45507776	45522339	45522581	125	46	31	50	19	25	6	30	2	9	98	98	0.160587804	1	0.714	0.708	0.554	0.75	0.769	-0.101
144	SOD2	SOD2	chr6	-	159682473	159682638	159684853	159685033	159679115	159679298	159688125	159688242	413	247	217	148	110	100	1	49	4	28	98	98	0.165128506	1	0.736	0.692	0.685	0.2	0.636	0.286
148	GLIPR2	GLIPR2	chr9	+	36148546	36148650	36150871	36150949	36147785	36147894	36162361	36163913	20	9	9	52	13	20	2	3	0	5	98	98	0.167785562	1	0.278	0.409	0.31	1	0.375	-0.355
1528	IRF1	IRF1	chr5	-	132487925	132488025	132489391	132489483	132486953	132487130	132490544	132490773	43	25	13	75	60	22	0	6	7	11	98	98	0.172303606	1	0.364	0.294	0.371	0	0.353	0.166
718	TBXAS1	TBXAS1	chr7	+	139955458	139955607	139957633	139957764	139953367	139953456	139961918	139962233	27	25	5	38	16	12	1	5	2	12	98	98	0.172540394	1	0.415	0.61	0.294	0.333	0.294	0.126
167	VNN2	VNN2	chr6	-	132751144	132751518	132755842	132756035	132749694	132749865	132757415	132757546	299	172	111	126	82	68	4	21	0	8	98	98	0.174346114	1	0.704	0.677	0.62	1	0.724	-0.195
1110	PTPN6	PTPN6	chr12	+	6960343	6960435	6960805	6960945	6960087	6960239	6961125	6961316	84	34	19	105	37	13	2	16	0	11	98	98	0.17819376	1	0.444	0.479	0.594	1	0.593	-0.291
657	ELOVL5	ELOVL5	chr6	-	53270592	53270727	53273219	53273344	53267397	53269270	53275089	53275261	79	38	24	52	30	34	1	16	0	8	98	98	0.187130875	1	0.603	0.559	0.414	1	0.667	-0.308
551	SORL1	SORL1	chr11	+	121478117	121478243	121488031	121488193	121470006	121470123	121490042	121490110	184	118	36	296	153	134	3	32	0	57	98	98	0.193262821	1	0.383	0.435	0.212	1	0.36	-0.337
1131	KLHL2	KLHL2	chr4	+	165228806	165228913	165238777	165238899	165219933	165220059	165263196	165263359	12	5	9	17	4	9	1	6	0	4	98	98	0.193339356	1	0.414	0.556	0.5	1	0.6	-0.31
525	SERPINA1	SERPINA1	chr14	-	94379463	94379611	94382591	94383241	94376746	94378640	94390456	94390692	108	35	38	142	65	104	2	20	6	51	98	98	0.194500005	1	0.432	0.35	0.268	0.25	0.282	0.084
1299	SETX	SETX	chr9	-	132336295	132336515	132342689	132342799	132334607	132334727	132346260	132346471	72	17	2	91	20	4	1	13	0	9	98	98	0.195265172	1	0.442	0.459	0.333	1	0.591	-0.384
283	XPO6	XPO6	chr16	-	28117317	28117462	28121669	28121762	28112903	28113050	28125688	28125848	186	130	42	115	75	26	5	50	0	25	98	98	0.203788993	1	0.618	0.634	0.618	1	0.667	-0.21
237	EXOC6	EXOC6	chr10	+	92934309	92934430	92935813	92935885	92934143	92934190	92940726	92940824	9	4	2	10	3	1	0	2	1	1	98	98	0.213519627	1	0.474	0.571	0.667	0	0.667	0.237
493	NCF2	NCF2	chr1	-	183577598	183577707	183586894	183586977	183573184	183573292	183590155	183590604	310	137	123	25	16	0	6	79	0	12	98	98	0.214252004	1	0.925	0.895	1	1	0.868	0.006
643	AMPD2	AMPD2	chr1	+	109629326	109629490	109629795	109629916	109629108	109629235	109630232	109630406	19	25	0	31	8	12	2	5	1	4	98	98	0.215680739	1	0.38	0.758	0	0.667	0.556	-0.232
474	PTPRC	PTPRC	chr1	+	198741868	198742026	198742231	198742367	198735126	198735252	198744053	198744203	196	110	52	207	86	77	5	34	2	26	98	98	0.216034204	1	0.486	0.561	0.403	0.714	0.567	-0.157
458	ARAP1	ARAP1	chr11	-	72697910	72698106	72699004	72699107	72697597	72697649	72699416	72699552	97	76	23	26	29	15	4	19	3	12	98	98	0.216993421	1	0.789	0.724	0.605	0.571	0.613	0.114
1047	ARHGAP15	ARHGAP15	chr2	+	143436912	143437042	143487372	143487495	143435600	143435699	143519265	143519364	72	17	18	65	21	4	0	10	2	10	98	98	0.223268285	1	0.526	0.447	0.818	0	0.5	0.347
782	IL4R	IL4R	chr16	+	27330065	27330198	27340185	27340273	27313908	27314020	27342120	27342259	0	1	4	54	21	12	0	0	2	5	98	98	0.225979246	1	0	0.045	0.25	0	0	0.098
473	PFKFB3	PFKFB3	chr10	+	6221640	6221745	6222854	6222984	6221380	6221527	6223957	6224020	56	41	5	69	41	7	11	20	3	22	98	98	0.231324144	1	0.448	0.5	0.417	0.786	0.476	-0.176
223	FAM65B	FAM65B	chr6	-	24832255	24832391	24839090	24839272	24830508	24830670	24842861	24843554	80	33	16	18	17	13	0	5	1	4	98	98	0.243139221	1	0.816	0.66	0.552	0	0.556	0.398
1435	AOAH	AOAH	chr7	-	36637850	36637910	36659165	36659265	36632035	36632106	36673942	36674009	18	4	0	32	13	4	0	3	6	6	98	98	0.246598151	1	0.36	0.235	0	0	0.333	0.032
257	MEFV	MEFV	chr16	-	3248908	3249004	3249430	3249780	3247015	3247246	3254157	3254790	46	22	18	48	18	35	0	11	2	18	98	98	0.248207938	1	0.489	0.55	0.34	0	0.379	0.27
1256	PARP14	PARP14	chr3	+	122699389	122701635	122703741	122703978	122695425	122695662	122704526	122704748	46	12	0	34	11	2	7	16	4	9	98	98	0.250710902	1	0.575	0.522	0	0.636	0.64	-0.272
724	SELL	SELL	chr1	-	169696534	169696553	169701559	169701688	169690664	169691802	169703254	169703440	124	55	47	38	26	7	2	26	1	13	98	68	0.257332021	1	0.694	0.595	0.823	0.581	0.581	0.123
143	IGF2R	IGF2R	chr6	+	160027184	160027314	160029549	160029655	160024571	160024704	160032550	160032713	252	84	46	147	63	26	1	40	7	21	98	98	0.25857467	1	0.632	0.571	0.639	0.125	0.656	0.223
998	CSNK1D	CSNK1D	chr17	-	82253015	82253244	82255428	82255577	82252433	82252604	82265685	82265796	14	8	5	18	7	5	1	2	2	4	98	98	0.26440277	1	0.437	0.533	0.5	0.333	0.333	0.157
732	LRRK2	LRRK2	chr12	+	40303947	40304134	40305784	40305966	40302788	40302882	40308466	40308696	208	58	33	91	86	28	1	29	1	14	98	98	0.271535111	1	0.696	0.403	0.541	0.5	0.674	-0.04
858	ACSL1	ACSL1	chr4	-	184770398	184770476	184773080	184773154	184768315	184768450	184773662	184773714	163	95	53	85	32	78	2	38	1	16	98	98	0.274471942	1	0.657	0.748	0.405	0.667	0.704	-0.082
1206	ARHGEF1	ARHGEF1	chr19	+	41904948	41905036	41905174	41905261	41904215	41904383	41905759	41905827	19	9	0	35	10	14	4	1	0	7	98	98	0.276065057	1	0.352	0.474	0	1	0.125	-0.287
815	FGR	FGR	chr1	-	27614429	27614583	27614849	27614926	27613218	27613350	27615433	27615613	67	28	22	87	22	38	1	35	17	27	98	98	0.278358891	1	0.435	0.56	0.367	0.056	0.565	0.144
650	OSBPL9	OSBPL9	chr1	+	51651990	51652041	51669433	51669512	51616873	51617221	51714002	51714079	4	7	0	11	8	3	1	1	0	8	98	98	0.282183756	1	0.267	0.467	0	1	0.111	-0.311
813	MAEA	MAEA	chr4	+	1315396	1315600	1322380	1322503	1311978	1312161	1332756	1332865	15	16	2	0	3	1	3	2	0	0	98	98	0.297185281	1	1	0.842	0.667	1	1	-0.164
1527	IRF1	IRF1	chr5	-	132485666	132485716	132486250	132486373	132484361	132484497	132486556	132486686	65	18	29	72	36	16	2	12	0	11	98	98	0.306476344	1	0.474	0.333	0.644	1	0.522	-0.277
871	BEST1	BEST1	chr11	+	61959497	61959578	61959891	61960043	61958145	61958298	61962254	61962893	7	1	5	26	7	17	0	2	1	3	98	98	0.306596855	1	0.212	0.125	0.227	0	0.4	-0.012
1309	ARPC2	ARPC2	chr2	+	218239390	218239484	218245419	218245546	218238663	218238850	218249363	218249464	144	58	65	163	76	46	0	29	3	31	98	98	0.307941549	1	0.469	0.433	0.586	0	0.483	0.255
754	ADAM8	ADAM8	chr10	-	133274158	133274235	133275483	133275587	133273950	133274029	133276771	133276903	18	15	1	84	46	27	1	3	8	19	98	98	0.314113239	1	0.176	0.246	0.036	0.111	0.136	0.029
829	AQP9	AQP9	chr15	+	58173067	58173205	58174917	58175036	58166672	58166799	58179127	58179345	144	111	100	145	85	76	1	40	0	24	98	98	0.323281869	1	0.498	0.566	0.568	1	0.625	-0.268
400	PTGES3	PTGES3	chr12	-	56670274	56670364	56671748	56671847	56666203	56666266	56672739	56672809	13	2	0	17	6	14	0	2	1	2	98	98	0.333454073	1	0.433	0.25	0	0	0.5	-0.022
1151	C17orf62	C17orf62	chr17	-	82446622	82446696	82447579	82447621	82445863	82445960	82449169	82449292	28	15	1	47	20	15	2	4	2	5	91	98	0.342520084	1	0.391	0.447	0.067	0.519	0.463	-0.189

1397	SLC6A6	SLC6A6	chr3	+	14478465	14478568	14479084	14479185	14477204	14477342	14481670	14481841	59	20	15	97	39	18	1	11	2	9	98	98	0.346752966	1	0.378	0.339	0.455	0.333	0.55	-0.051
557	PKM	PKM	chr15	-	72202453	72202620	72203021	72203188	72200473	72200655	72206727	72206880	3	0	0	36	6	7	0	0	3	15	98	98	0.350414649	1	0.077	0	0	0	0	0.026
165	SGK1	SGK1	chr6	-	134171636	134171732	134172192	134172316	134171022	134171178	134172661	134172774	82	52	12	90	38	6	0	13	2	11	98	98	0.354258391	1	0.477	0.578	0.667	0	0.542	0.303
1497	CPEB4	CPEB4	chr5	+	173932449	173932500	173944966	173945140	173910522	173910604	173949507	173949597	8	3	7	28	12	10	0	5	1	18	98	98	0.363102749	1	0.222	0.2	0.412	0	0.217	0.17
500	BCL6	BCL6	chr3	-	187726730	187726898	187728359	187728544	187725498	187725629	187729049	187730021	47	22	12	23	16	7	2	13	1	13	98	98	0.363508365	1	0.671	0.579	0.632	0.667	0.5	0.044
779	RNF10	RNF10	chr12	+	120566824	120566980	120571190	120571291	120565427	120565529	120575630	120575688	19	19	11	14	16	5	0	6	2	3	98	98	0.367190598	1	0.576	0.543	0.687	0	0.667	0.268
106	IFRD1	IFRD1	chr7	+	112456913	112457038	112458860	112459018	112456001	112456086	112461865	112461916	216	63	15	61	44	24	6	38	0	28	98	98	0.368297315	1	0.78	0.589	0.385	1	0.576	-0.203
57	DENND3	DENND3	chr8	+	141138021	141138137	141150833	141150953	141136557	141136791	141151618	141151837	53	54	11	84	39	10	3	15	0	20	98	98	0.372123371	1	0.387	0.581	0.524	1	0.429	-0.217
1178	ALOX5	ALOX5	chr10	+	45382482	45382681	45395854	45395936	45374175	45374429	45412190	45412313	45	36	5	74	37	11	1	30	0	31	98	98	0.380866482	1	0.378	0.493	0.312	1	0.492	-0.352
1526	IRF1	IRF1	chr5	-	132484361	132484497	132486250	132486373	132481608	132484075	132486556	132486686	65	21	29	44	24	13	2	12	0	8	98	98	0.381480785	1	0.596	0.467	0.69	1	0.6	-0.216
526	SERPINA1	SERPINA1	chr14	-	94379463	94379611	94388800	94389010	94376746	94378640	94390456	94390692	47	13	5	142	65	104	1	15	6	51	98	98	0.381777559	1	0.249	0.167	0.046	0.143	0.227	-0.031
101	MGAM	MGAM	chr7	+	142036168	142036285	142036822	142036977	142034669	142034841	142038530	142038615	110	29	10	97	19	10	2	18	0	14	98	98	0.385226423	1	0.531	0.604	0.5	1	0.562	-0.236
491	NCF2	NCF2	chr1	-	183573184	183573292	183574486	183574621	183570779	183570839	183577598	183577707	118	56	89	304	147	80	0	30	5	53	98	98	0.390483373	1	0.28	0.276	0.527	0	0.361	0.181
623	AKAP13	AKAP13	chr15	+	85658536	85658590	85662387	85662453	85655416	85655787	85664562	85664755	80	39	4	3	1	0	2	13	0	0	98	98	0.394292383	1	0.964	0.975	1	1	1	-0.02
873	PLXDC2	PLXDC2	chr10	+	20046868	20047015	20068169	20068239	20001774	20001986	20143294	20143417	36	9	0	45	27	6	1	6	0	11	98	98	0.394870873	1	0.444	0.25	0	1	0.353	-0.445
1223	ARHGAP30	ARHGAP30	chr1	-	161056387	161056532	161059613	161059716	161054622	161054705	161069527	161069970	40	18	10	54	33	7	1	18	0	18	98	98	0.399292684	1	0.426	0.353	0.588	1	0.5	-0.294
102	MGAM	MGAM	chr7	+	142047784	142047873	142050234	142050284	142040721	142040846	142050696	142050864	85	20	17	124	20	11	2	7	0	9	98	98	0.399781628	1	0.407	0.5	0.607	1	0.437	-0.214
1464	WDR1	WDR1	chr4	-	10097710	10097891	10103895	10103986	10088663	10088741	10116112	10116234	36	16	13	37	7	7	4	6	2	3	98	98	0.401096861	1	0.493	0.696	0.65	0.667	0.667	-0.054
197	TAPBP	TAPBP	chr6	-	33313216	33313477	33313693	33313864	33304988	33305387	33314004	33314387	4	0	0	26	6	4	0	1	5	4	98	98	0.40341675	1	0.133	0	0	0	0.2	-0.056
1007	MYO1F	MYO1F	chr19	-	8539946	8540028	8541905	8541991	8536948	8537055	8544296	8544464	66	33	35	115	78	35	6	17	1	42	98	98	0.414774608	1	0.365	0.297	0.5	0.857	0.288	-0.185
786	FRY	FRY	chr13	+	32175545	32175632	32178176	32178436	32173366	32173549	32178843	32179033	49	12	4	51	15	5	2	3	3	5	98	98	0.42175388	1	0.49	0.444	0.444	0.4	0.375	0.072
1226	RBM23	RBM23	chr14	-	22908332	22908380	22909482	22909595	22906194	22906368	22911327	22911403	67	35	36	7	10	0	2	3	0	2	98	97	0.428534074	1	0.905	0.776	1	1	0.598	0.095
1102	STAT3	STAT3	chr17	-	42324710	42324846	42324962	42325061	42323572	42323625	42326115	42326199	27	18	26	49	25	19	0	11	1	17	98	98	0.432753276	1	0.355	0.419	0.578	0	0.393	0.254
1041	THEMIS2	THEMIS2	chr1	+	27876587	27876728	27879643	27880054	27872542	27872665	27885294	27885451	75	50	16	3	0	1	10	8	0	2	98	98	0.443747177	1	0.962	1	0.941	1	0.8	0.068
1420	MAPKAPK3	MAPKAPK3	chr3	+	50644408	50644532	50645709	50645785	50642252	50642332	50646139	50646264	29	17	9	15	14	4	0	10	1	7	98	98	0.445444298	1	0.659	0.548	0.692	0	0.588	0.339
857	ACSL1	ACSL1	chr4	-	184770254	184770332	184770398	184770476	184768315	184768450	184773080	184773154	230	105	146	18	3	6	1	55	2	3	98	98	0.452276841	1	0.927	0.972	0.961	0.333	0.948	0.313
1122	VMP1	VMP1	chr17	+	59735337	59735473	59737452	59737543	59731420	59731522	59738836	59738947	65	35	48	124	101	62	0	14	2	29	98	98	0.455253454	1	0.344	0.257	0.436	0	0.326	0.183
987	PIK3R5	PIK3R5	chr17	-	8889972	8890126	8890737	8890912	8889138	8889222	8893585	8893655	16	11	14	4	4	1	0	6	1	1	98	98	0.460982421	1	0.8	0.733	0.933	0	0.857	0.394
440	KIAA1033	KIAA1033	chr12	+	105162748	105162845	105164110	105164307	105160000	105160148	105164640	105164740	25	13	5	19	9	7	1	6	1	3	98	98	0.46252304	1	0.568	0.591	0.417	0.5	0.667	-0.058
489	NCF2	NCF2	chr1	-	183565703	183565779	183566919	183566988	183564004	183564030	183567203	183567345	55	44	53	135	63	66	0	14	4	18	98	98	0.46267558	1	0.289	0.411	0.445	0	0.437	0.163
731	LRRK2	LRRK2	chr12	+	40298242	40298493	40299108	40299257	40295426	40295644	40302788	40302882	192	59	33	390	124	39	0	32	2	64	98	98	0.472031432	1	0.33	0.322	0.458	0	0.333	0.204
1529	H2AFY	H2AFY	chr5	-	135350822	135350913	135352945	135353045	135345967	135346057	135360496	135360607	16	12	14	5	3	3	2	10	0	3	98	98	0.472653985	1	0.762	0.8	0.824	1	0.769	-0.089
898	GLT1D1	GLT1D1	chr12	+	128875913	128876062	128888638	128888744	128853535	128853649	128899235	128899287	31	12	3	51	29	7	2	9	5	8	98	98	0.478814744	1	0.378	0.293	0.3	0.286	0.529	-0.084
1440	CSF2RA	CSF2RA	chrX	+	1300490	1300626	1303922	1304019	1295426	1295456	1305445	1305527	15	11	8	8	3	10	0	15	1	10	98	98	0.482127935	1	0.652	0.786	0.444	0	0.6	0.327
1502	FYB	FYB	chr5	-	39153447	39153604	39201825	39202987	39141094	39141141	39219442	39219578	52	17	0	90	43	10	4	8	3	21	98	98	0.490580949	1	0.366	0.283	0	0.571	0.276	-0.207
286	XPO6	XPO6	chr16	-	28135215	28135324	28146093	28146203	28133840	28133933	28152658	28152785	127	43	20	198	116	46	1	24	4	25	98	98	0.502162687	1	0.391	0.27	0.303	0.2	0.49	-0.024
859	ACSL1	ACSL1	chr4	-	184773842	184773875	184776483	184776662	184773662	184773714	184776883	184776983	82	24	41	106	47	33	2	14	1	33	98	82	0.513367948	1	0.393	0.299	0.51	0.626	0.262	-0.043
904	ITGA5	ITGA5	chr12	-	54409484	54409597	54411833	54411964	54409231	54409352	54418980	54419266	6	5	2	20	15	8	1	1	0	3	98	98	0.514001261	1	0.231	0.25	0.2	1	0.25	-0.398
284	XPO6	XPO6	chr16	-	28121669	28121762	28125688	28125848	28117317	28117462	28132333	28132403	129	71	20	153	88	40	4	20	1	30	98	98	0.524444894	1	0.457	0.447	0.333	0.8	0.4	-0.188
1380	CSF2RB	CSF2RB	chr22	+	36925986	36926177	36929401	36929559	36923243	36923367	36929638	36929807	46	34	37	94	42	44	1	13	6	17	98	98	0.526871726	1	0.329	0.447	0.457	0.143	0.433	0.123
302	PTTG1IP	PTTG1IP	chr21	-	44861162	44861271	44865394	44865447	44856192	44856364	44873501	44873903	10	3	6	27	15	4	1	3	0	7	98	98	0.534318244	1	0.27	0.167	0.6	1	0.3	-0.304
1405	HIPK3	HIPK3	chr11	+	33348165	33348228	33348521	33348818	33347851	33348013	33349146	33349287	14	4	2	54	19	5	0	6	1	4	98	98	0.539792793	1	0.206	0.174	0.286	0	0.6	-0.078
1292	CYTIP	CYTIP	chr2	-	157430859	157430962	157434369	157434424	157430558	157430652	157434697	157434747	184	88	26	187	85	27	3	30	5	34	98	98	0.540075756	1	0.496	0.509	0.491	0.375	0.469	0.077
1244	FAM129A	FAM129A	chr1	-	184823637	184823742	184831846	184831962	184823166	184823329	184884632	184884800	136	33	28	122	66	18	0	23	2	19	98	98	0.547893713	1	0.527	0.333	0.609	0	0.548	0.216
1042	THEMIS2	THEMIS2	chr1	+	27879643	27880054	27881970	27882125	27876587	27876728	27882512	27883043	110	23	14	0	5	0	6	24	0	1	98	98	0.548720612	1	1	0.821	1	1	0.96	-0.04
1530	ARHGAP26	ARHGAP26	chr5	+	143014079	143014116	143037195	143037261	142932046	142932125	143041815	143041890	134	37	12	50	33	4	1	28	0	13	86	98	0.550512855	1	0.753	0.561	0.774	1	0.711	-0.159
844	HLA-B	HLA-B	chr6	-	31354632	31354665	31355106	31355223	31354478	31354526	31355316	31355592	1587	500	504	407	232	231	40	238	12	113	98	82	0.551571777	1	0.765	0.643	0.646	0.736	0.638	-0.002
727	LRRK2	LRRK2	chr12	+	40240482	40240617	40243549	40243681	40237968	40238103	40249825	40249945	491	161	45	171	64	23	4	41	1	22	98	98	0.572355298	1	0.742	0.716	0.662	0.8	0.651	-0.019
519	MNDA	MNDA	chr1	+	158843954	158844122	158845586	158846003	158843278	158843415	158847727	158847916	358	171	135	428	329	232	4	45	2	68	98	98	0.576278334	1	0.455	0.342	0.368	0.667	0.398	-0.144
1021	HIF1A	HIF1A	chr14	+	61738086	61738373	61740504	61740627	61736888	61737109	61740754	61741188	45	18	13	47	27	7	2	6	0	11	98	98	0.577069499	1	0.489	0.4	0.65	1	0.353	-0.163
1048	ARHGAP15	ARHGAP15	chr2	+	143487372	143487495	143519265	143519364	143436912	143437042	143556407	143556485	128	34	12	98	12	10	0	9	1	5	98	98	0.577192634	1	0.566	0.739	0.545	0	0.643	0.295
259	MEFV	MEFV	chr16	-	3249430	3249780	3254157	3254790	3248908	3249004	3256310	3256627	81	26	30	23	10	11	0	16	1	5	98	98	0.579281899	1	0.779	0.722	0.732	0	0.762	0.363
1347	PREX1	PREX1	chr20	-	48676192	48676268	48679359	48679409	48666282	48666355	48679650	48679754	40	25	28	109	83	47	1	12	3	34	98	98	0.59053247	1	0.268	0.231	0.373	0.25	0.261	0.035
1026	STAT6	STAT6	chr12	-	57107604	57107743	57108162	57108299	57107230	57107314	57111128	57111413	28	11	3	36	20	11	1	5	1	6	98	98	0.590630237	1	0.437	0.355	0.214	0.5	0.455	-0.142
948	PICALM	PICALM	chr11	-	86022366	86022469	86026291	86026367	86014869	86014963	86031468	86031611	144	66	56	188	89	37	1	14	0	16	98	98	0.593652134	1	0.434	0.426	0.602	1	0.467	-0.246
552	SORL1	SORL1	chr11	+	121543547	121543726	121545242	121545429	121532463	121532552	121549959	121550088	120	67	33	137	71	36	0	18	2	13	98	98	0.596956742	1	0.467	0.486	0.478	0	0.581	0.187
1179	SLC44A2	SLC44A2	chr19	+	10634973	10635073	10636322	10636585	10634755	10634887	10636661	10636756	29	14	12	53	28	9	1	8	6	9	98	98	0.603913668	1	0.354	0.333	0.571	0.143	0.471	0.112
377	GIT2	GIT2	chr12	-	109947255	109947504	109951211	109951316	109939164	109939247	109953091	109953234	17	5	5	1	3	1	1	2	0	2	98	98	0.607057526	1	0.944	0.625	0.833	1	0.5	0.051
1153	SMCHD1	SMCHD1	chr18	+	2722518	2722663	2724898	2724995	2718314	2718434	2726451	2726524	103	62	32	217	108	24	0	26	1	40	98	98	0.620617635	1	0.322	0.365	0.571	0	0.394	0.222
467	PTPRJ	PTPRJ	chr11	+	48112746	48112983	48121002	48121266	48110057	48110076	48123612	48123870	63	43	22	43	18	17	11	21	4	13	98	98	0.623407942	1	0.594	0.705	0.564	0.733	0.618	-0.054
1205	SASH3	SASH3	chrX	+	129792327	129792476	129792626	129792836	129790939	129791081	129792988	129793139	33	18	3	24	12	2	3	5	0	8	98	98	0.625998837	1	0.579	0.6	0.6	1	0.385	-0.1
19	DDX3X	DDX3X	chrX	+	41343215	41343351	41343736	41343822	41342736	41342836	41344029	41344128	147	42	21	166	36	32	2	15	2	21	98	98	0.627654747	1	0.47	0.538	0.396	0.5	0.417	0.009
1075	FLOT2	FLOT2	chr17	-	28884224	28884315	28888944	28889026	28883107	28883231	28897525	28897697	26	7	14	33	28	26	1	4	0	10	98	98	0.633526331	1	0.441	0.2	0.35	1	0.286	-0.313
1385	TBC1D22A	TBC1D22A	chr22	+	46797443	46797620	46878652	46878723	46793500	46793841	46891265	46891394	24	2	2	15	6	0	1	5	3	3	98	98	0.641897603	1	0.615	0.25	1	0.25	0.625	0.184
256	MEFV	MEFV	chr16	-	3247015	3247246	3248908	3249004	3246524	3246547	3249430	3249780	23	10	12	84	58	39	1	5	1	25	98	98	0.643337978	1	0.215	0.147	0.235	0.5	0.167	-0.135
1076	SSH2	SSH2	chr17	-	29695458	29695523	29702958	29703062	29684852	29684684	29761149	29761432	41	20	13	48	33	12	1	4	0	7	98	98	0.656275231	1	0.461	0.377	0.52	1	0.364	-0.229
604	GBP5	GBP5	chr1	-	89267416	89267526	89268728	89268856	89266956	89267153	89269365	89269574	27	6	4	91	27	18	0	4	1	11	98	98	0.659255602	1	0.229	0.182	0.182	0	0.267	0.064
52	TLN1	TLN1	chr9	-	35699355	35699461	35699973	35700081	35699031	35699156	35700190	35700376	85	21	17	74	21	10	2	17	0	23	98	98	0.659351734	1	0.535	0.5	0.63	1	0.425	-0.157
127	IKZF1	IKZF1	chr7	+	50387344	50387470	50391728	50391833	50382539	50382707	50399917	50405100	25	3	4	5	0	2	2	7	0	4	98	98	0.669024756	1	0.833	1	0.667	1	0.636	0.015
831	DOCK2	DOCK2	chr5	+	169670541	169670597	169671077	169671174	169669287	169669328	169674296	169674445	32	11	5	142	34	5	0	8	2	22	98	98	0.672793032	1	0.184	0.244	0.5	0	0.267	0.176
899	GLT1D1	GLT1D1	chr12	+	128888638	128888744	128899235	128899287	128875913	128876062	128945325	128945369	29	15	11	3	0	0	1	10	0	1	98	98	0.676978034	1	0.906	1	1	1	0.909	0.014
1288	ACOX1	ACOX1	chr17	-	75960214	75960375	75973624	75973785	75957458	75957566	75978533	75978693	8	3	1	35	8	22	0	3	1	11	98	98	0.677706498	1	0.186	0.273	0.043	0	0.214	0.06
334	PCNX	PCNX	chr14	+	71011491	71011549	71012999	71013202	71009633	71009724	71019008	71019162	33	15	2	56	11	7	0	6	2	4	98	98	0.689326839	1	0.371	0.577	0.222	0	0.6	0.09
631	LAPTM5	LAPTM5	chr1	-	30741639	30741716	30742455	30742549	30739808	30739937	30757658	30757836	11	7	3	131	34	43	0	4	1	20	98	98	0.695609396	1	0.077	0.171	0.065	0	0.167	0.021
535	STX3	STX3	chr11	+	59787036	59787136	59788872	59788947	59773210	59773294	59790518	59790586	38	20	10	57	26	14	0	6	1	6	98	98	0.713148432	1	0.4	0.435	0.417	0	0.5	0.167
1201	GMFG	GMFG	chr19	-	39329543	39329626	39333076	39333126	39328999	39329073	39335260	39335310	99	64	46	185	118	124	1	11	0	34	98	98	0.716396186	1	0.349	0.352	0.271	1	0.244	-0.298
1406	HIPK3	HIPK3	chr11	+	33348521	33348818	33349146	33349287	33347851	33348013	33351607	33351843	16	2	2	31	16	4	1	1	0	6	98	98	0.724062162	1	0.34	0.111	0.333	1	0.143	-0.31
1148	RNF213	RNF213	chr17	+	80360060	80360206	80361733	80361888	80358287	80358479	80363101	80363314	31	19	2	32	41	4	0	19	1	25	98	98	0.753935402	1	0.492	0.317	0.333	0	0.432	0.165
943	CD53	CD53	chr1	+	110896652	110896733	110897808	110897892	110894959	110895055	110899123	110899936	33	29	35	99	49	56	0	12	2	18	98	98	0.754948443	1	0.25	0.372	0.385	0	0.4	0.136
392	GTPBP1	GTPBP1	chr22	+	38727212	38727348	38727982	38728161	38726257	38726440	38729461	38729662	16	7	5	27	12	0	1	6	0	7	98	98	0.764160975	1	0.372	0.368	1	1	0.462	-0.151
721	APH1A	APH1A	chr1	-	150267715	150267789	150267956	150268127	150267355	150267478	150268697	150269209	9	0	0	11	2	6	0	3	1	1	98	98	0.766857327	1	0.45	0	0	0	0.75	-0.225
328	CTSS	CTSS	chr1	-	150750005	150750171	150751780	150752008	150747776	150747879	150755000	150755150	261	144	134	104	71	67	2	110	0	58	98	98	0.767195433	1	0.715	0.67	0.667	1	0.655	-0.143
669	NBEAL2	NBEAL2	chr3	+	47003173	47003309	47003815	47003976	47002956	47003081	47004076	47004393	47	20	6	13	4	4	3	16	0	11	98	98	0.768824062	1	0.783	0.833	0.6	1	0.593	-0.058
735	LRRK2	LRRK2	chr12	+	40351538	40351733	40354298	40354492	40348408	40348509	40356114	40356187	164	62	21	126	40	21	3	15	2	14	98	98	0.771638748	1	0.566	0.608	0.5	0.6	0.517	-0.001
1189	MYO9B	MYO9B	chr19	+	17156908	17157038	17159394	17159484	17154314	17154415	17162349	17162466	57	25	4	57	54	5	6	9	4	15	98	98	0.775648212	1	0.5	0.316	0.444	0.6	0.375	-0.068
932	DHX9	DHX9	chr1	+	182855576	182855713	182856531	182856578	182854029	182854178	182858103	182858240	9	4	0	28	10	3	2	0	0	7	98	96	0.777970856	1	0.239	0.282	0	1	0	-0.326
1040	THEMIS2	THEMIS2	chr1	+	27876587	27876728	27879643	27880054	27872542	27872665	27881970	27882125	72	53	10	47	9	18	7	8	1	19	98	98	0.786188136	1	0.605	0.855	0.357	0.875	0.296	0.02
733	LRRK2	LRRK2	chr12	+	40305784	40305966	40308466	40308696	40303947	40304134	40309105	40309233	213	78	30	288	118	21	1	18	0	34	98	98	0.79075928	1	0.425	0.398	0.588	1	0.346	-0.203
368	RRP12	RRP12	chr10	-	97390422	97390539	97390738	97390844	97388488	97388624	97393683	97393760	59	13	2	27	7	4	2	7	1	4	98	98	0.791114293	1	0.686	0.65	0.333	0.667	0.636	-0.095
830	AQP9	AQP9	chr15	+	58174917	58175036	58179127	58179345	58173066	58173205	58183690	58185911	150	68	71	86	57	58	1	19	1	12	98	98	0.802867631	1	0.636	0.544	0.55	0.5	0.613	0.02
198	FLOT1	FLOT1	chr6	-	30741189	30741333	30741613	30741704	30740678	30740798	30741791	30741867	24	14	13	39	14	17	1	4	0	11	98	98	0.810040084	1	0.381	0.5	0.433	1	0.267	-0.195
145	SOD2	SOD2	chr6	-	159684853	159685033	159688125	159688242	159682473	159682638	159692660	159692863	370	266	195	468	299	215	6	33	2	58	98	98	0.8101779	1	0.442	0.471	0.476	0.75	0.363	-0.093
787	ATP2A3	ATP2A3	chr17	-	3951580	3951685	3953346	3953429	3951250	3951389	3953692	3953710	18	10	8	27	24	13	1	3	8	1	98	98	0.810828389	1	0.4	0.294	0.381	0.111	0.75	-0.072
457	ARAP1	ARAP1	chr11	-	72686041	72686191	72687438	72687502	72685068	72685681	72687687	72687738	29	18	25	65	28	16	1	5	0	13	98	98	0.813010615	1	0.309	0.391	0.61	1	0.278	-0.202
1346	PREX1	PREX1	chr20	-	48649995	48650206	48650893	48651055	48649299	48649576	48651395	48651583	31	22	13	84	37	35	1	11	6	21	98	98	0.814486416	1	0.27	0.373	0.271	0.143	0.344	0.061
1185	ADGRE5	ADGRE5	chr19	+	14407060	14407229	14407907	14408009	14406868	14406960	14408091	14408725	45	51	44	59	32	34	1	15	0	19	98	98	0.816695615	1	0.433	0.614	0.564	1	0.441	-0.184
374	NLRP1	NLRP1	chr17	-	5532821	5532984	5533896	5533988	5530480	5530704	5536850	5536940	55	19	11	47	39	5	2	20	4	20	98	98	0.823687728	1	0.539	0.328	0.687	0.333	0.5	0.102
730	LRRK2	LRRK2	chr12	+	40295426	40295644	40298242	40298493	40294844	40294914	40299108	40299257	319	82	30	215	48	19	8	42	3	29	98	98	0.825500335	1	0.597	0.631	0.612	0.727	0.592	-0.046
728	LRRK2	LRRK2	chr12	+	40257247	40257377	40259479	40259604	40252909	40253016	40263788	40263901	175	69	34	372	93	25	2	19	2	27	98	98	0.828099365	1	0.32	0.426	0.576	0.5	0.413	-0.016
331	CTSS	CTSS	chr1	-	150755000	150755150	150757857	150757980	150751780	150752008	150764637	150764764	410	160	108	262	144	131	1	123	2	109	98	98	0.829639519	1	0.61	0.526	0.452	0.333	0.53	0.098
236	EXOC6	EXOC6	chr10	+	92934143	92934190	92934309	92934430	92928338	92928422	92935813	92935885	14	9	2	11	1	3	1	3	0	7	96	98	0.832162999	1	0.565	0.902	0.405	1	0.304	-0.028
343	SOS2	SOS2	chr14	-	50174453	50174552	50180571	50180682	50161481	50161609	50182462	50182606	70	30	7	37	13	5	3	9	1	7	98	98	0.834511645	1	0.654	0.698	0.583	0.75	0.562	-0.011
449	BAZ1A	BAZ1A	chr14	-	34785776	34786001	34786125	34786221	34783761	34783927	34792774	34792921	13	6	4	38	16	6	0	4	1	6	98	98	0.836937733	1	0.255	0.273	0.4	0	0.4	0.109
1396	SLC6A6	SLC6A6	chr3	+	14416411	14416453	14443623	14443863	14402575	14402847	14445716	14445851	95	41	9	75	39	28	3	14	5	13	91	98	0.85311396	1	0.577	0.531	0.257	0.393	0.537	-0.01
240	CDC42SE1	CDC42SE1	chr1	-	151055015	151055126	151055676	151055993	151054230	151054321	151059478	151059649	49	16	7	128	59	33	1	4	1	12	98	98	0.858739689	1	0.277	0.213	0.175	0.5	0.25	-0.153
942	CD53	CD53	chr1	+	110892344	110892533	110897808	110897892	110891391	110891471	110899123	110899936	141	54	53	98	50	56	3	18	2	18	98	98	0.865568364	1	0.59	0.519	0.486	0.6	0.5	-0.018
437	TNFRSF1A	TNFRSF1A	chr12	-	6333736	6333865	6334090	6334244	6333366	6333516	6341775	6342117	62	26	20	64	36	11	2	11	3	11	98	98	0.873353503	1	0.492	0.419	0.645	0.4	0.5	0.069
498	RASGRP4	RASGRP4	chr19	-	38417051	38417168	38419859	38420013	38414847	38415123	38420130	38420262	20	18	4	39	11	9	4	7	2	13	98	98	0.882368191	1	0.339	0.621	0.308	0.667	0.35	-0.086
1117	SPAG9	SPAG9	chr17	-	50984922	50984990	50985697	50985778	50982523	50982672	50987111	50987237	21	10	4	27	19	5	0	7	1	3	98	98	0.895684561	1	0.438	0.345	0.444	0	0.7	0.059
1199	DDX60L	DDX60L	chr4	-	168441336	168441492	168448637	168448779	168433009	168433115	168453123	168453282	30	22	3	44	27	0	1	22	2	14	98	98	0.895713753	1	0.405	0.449	1	0.333	0.611	0.146
1006	MYO1F	MYO1F	chr19	-	8530458	8530573	8536251	8536396	8530195	8530365	8536498	8536597	221	148	54	56	48	22	6	53	3	16	98	98	0.899193342	1	0.798	0.755	0.711	0.667	0.768	0.037
58	DENND3	DENND3	chr8	+	141138021	141138137	141151618	141151837	141136557	141136791	141155848	141155970	54	53	11	74	50	17	3	14	1	30	98	98	0.901910799	1	0.422	0.515	0.393	0.75	0.318	-0.091
177	CD164	CD164	chr6	-	109370410	109370467	109376073	109376112	109366513	109369017	109377899	109377971	34	17	3	20	10	0	1	6	0	5	88	98	0.903877373	1	0.654	0.654	1	1	0.572	-0.017
468	PTPRJ	PTPRJ	chr11	+	48144999	48145124	48146875	48146963	48144674	48144885	48149446	48149488	35	12	5	26	4	1	8	8	3	5	98	98	0.904773974	1	0.574	0.75	0.833	0.727	0.615	0.048
699	RFWD2	RFWD2	chr1	-	176136487	176136547	176149005	176149074	176135009	176135086	176184632	176184692	29	7	0	89	32	10	0	4	1	10	98	98	0.907396354	1	0.246	0.179	0	0	0.286	-0.001
1182	GLUL	GLUL	chr1	-	182385356	182385556	182385759	182385887	182378092	182384723	182386255	182386402	662	192	81	1299	443	148	10	77	26	147	98	98	0.913655916	1	0.338	0.302	0.354	0.278	0.344	0.02
734	LRRK2	LRRK2	chr12	+	40348408	40348509	40351538	40351733	40346752	40346923	40354298	40354492	144	58	52	83	38	10	1	17	0	12	98	98	0.915533828	1	0.634	0.604	0.839	1	0.586	-0.101
1118	TRIM25	TRIM25	chr17	-	56895520	56895604	56895925	56895952	56895342	56895441	56899114	56899180	21	14	11	43	9	11	1	4	0	14	76	98	0.930125935	1	0.386	0.667	0.563	1	0.269	-0.096
1209	CHD2	CHD2	chr15	+	92901166	92901299	92924320	92924552	92900320	92900824	92927243	92927330	30	20	8	45	15	9	0	9	1	4	98	98	0.932025488	1	0.4	0.571	0.471	0	0.692	0.135
285	XPO6	XPO6	chr16	-	28133840	28133933	28135215	28135324	28132333	28132403	28146093	28146203	125	73	21	134	92	25	1	25	2	26	98	98	0.937455275	1	0.483	0.442	0.457	0.333	0.49	0.049
1033	CR1	CR1	chr1	+	207622992	207623068	207630516	207630621	207621972	207621996	207639396	207641765	16	5	8	41	25	5	0	6	1	4	98	98	0.939008769	1	0.281	0.167	0.615	0	0.6	0.054
116	PTPN12	PTPN12	chr7	+	77583554	77583650	77585542	77585581	77581426	77581503	77592184	77592256	45	22	13	20	16	11	0	9	1	1	98	88	0.945708507	1	0.669	0.553	0.515	0	0.89	0.134
170	VNN3	VNN3	chr6	-	132726721	132727012	132728740	132728933	132724605	132724979	132734252	132734383	51	21	4	39	26	1	1	5	0	9	98	98	0.94976096	1	0.567	0.447	0.8	1	0.357	-0.074
21	DDX3X	DDX3X	chrX	+	41346858	41347012	41347314	41347451	41346504	41346622	41347639	41350287	108	47	22	102	29	29	0	27	1	11	98	98	0.95277589	1	0.514	0.618	0.431	0	0.711	0.166
889	BNIP2	BNIP2	chr15	-	59668891	59668990	59669275	59669362	59662862	59664120	59671182	59671314	27	15	3	44	31	10	0	8	1	7	98	98	0.963645053	1	0.38	0.326	0.231	0	0.533	0.046
1231	MBOAT7	MBOAT7	chr19	-	54187160	54187287	54188216	54188346	54183520	54183680	54188432	54188511	15	16	8	62	24	33	1	6	8	9	98	98	0.967774437	1	0.195	0.4	0.195	0.111	0.4	0.008
1257	MEF2A	MEF2A	chr15	+	99671322	99671454	99671519	99671657	99645560	99645764	99674392	99674612	20	3	2	6	0	0	1	5	0	3	98	98	0.969117258	1	0.769	1	1	1	0.625	0.111
20	DDX3X	DDX3X	chrX	+	41346858	41347012	41347311	41347451	41346504	41346622	41347639	41347915	108	47	22	108	30	29	0	27	1	11	98	98	0.96964034	1	0.5	0.61	0.431	0	0.711	0.158
171	VNN3	VNN3	chr6	-	132726721	132727012	132728740	132728979	132724605	132724979	132734252	132734383	44	14	6	39	26	1	1	2	0	9	98	98	0.975627308	1	0.53	0.35	0.857	1	0.182	-0.012
492	NCF2	NCF2	chr1	-	183573184	183573292	183577598	183577707	183570779	183570839	183586894	183586977	161	90	98	280	132	80	3	31	5	41	98	98	0.981091398	1	0.365	0.405	0.551	0.375	0.431	0.037
261	NLRP12	NLRP12	chr19	-	53807494	53807665	53809586	53811288	53805279	53805450	53814907	53814988	25	10	4	16	8	11	1	8	1	8	98	98	0.993526134	1	0.61	0.556	0.267	0.5	0.5	-0.022
1383	ARFGAP3	ARFGAP3	chr22	-	42834241	42834325	42835361	42835493	42831548	42831636	42840943	42841016	8	9	6	11	7	5	1	0	0	1	98	98	0.995820004	1	0.421	0.562	0.545	1	0	0.009
2	VAMP7	VAMP7	chrX	+	155900496	155900587	155919812	155919880	155898111	155898249	155939700	155939793	9	2	0	1	3	0	0	2	0	0	98	98	1	1	0.9	0.4	NA	NA	1	-0.35
6	MORF4L2	MORF4L2	chrX	-	103678498	103678600	103684680	103684729	103675497	103677051	103685170	103685260	1	2	0	10	2	3	0	0	0	5	98	98	1	1	0.091	0.5	0	NA	0	0.197
7	MORF4L2	MORF4L2	chrX	-	103678498	103678620	103684680	103684729	103675497	103677051	103685170	103685260	1	2	0	10	2	3	0	0	0	5	98	98	1	1	0.091	0.5	0	NA	0	0.197
8	MORF4L2	MORF4L2	chrX	-	103678498	103678651	103684680	103684729	103675497	103677051	103685170	103685260	1	2	0	12	2	3	0	0	0	5	98	98	1	1	0.077	0.5	0	NA	0	0.192
22	USP9X	USP9X	chrX	+	41136803	41137022	41140655	41140771	41134724	41134837	41140665	41141217	19	2	4	6	9	1	0	4	0	3	98	98	1	1	0.76	0.182	0.8	NA	0.571	0.01
23	GK	GK	chrX	+	30691122	30691199	30694399	30694537	30677374	30677452	30696041	30696151	9	2	0	13	4	10	0	0	0	1	98	98	1	1	0.409	0.333	0	NA	0	0.247
25	WWC3	WWC3	chrX	+	10125023	10125110	10126113	10126306	10124264	10124339	10127987	10128161	16	3	0	9	1	6	0	2	0	2	98	98	1	1	0.64	0.75	0	NA	0.5	-0.037
26	WWC3	WWC3	chrX	+	10126113	10126306	10127987	10128161	10125023	10125110	10128556	10128708	11	5	2	15	8	10	0	1	0	0	98	98	1	1	0.423	0.385	0.167	NA	1	-0.675
43	TLR4	TLR4	chr9	+	117708198	117708318	117708562	117708729	117704174	117704565	117712388	117717491	80	13	10	72	13	36	0	14	0	10	98	98	1	1	0.526	0.5	0.217	NA	0.583	-0.169
44	DAPK1	DAPK1	chr9	+	87686550	87686739	87697006	87697204	87681403	87681626	87698655	87698794	4	4	5	5	5	0	0	4	0	0	98	98	1	1	0.444	0.444	1	NA	1	-0.371
55	UHRF2	UHRF2	chr9	+	6420911	6421142	6433913	6434173	6413150	6413643	6460572	6460791	4	3	0	3	6	1	0	2	0	0	98	98	1	1	0.571	0.333	0	NA	1	-0.699
56	DENND3	DENND3	chr8	+	141136557	141136791	141150833	141150953	141128620	141128809	141151618	141151837	4	2	0	83	42	10	0	1	0	20	98	98	1	1	0.046	0.045	0	NA	0.048	-0.018
59	DENND3	DENND3	chr8	+	141160631	141160787	141163332	141163429	141155848	141155970	141165185	141165289	52	20	10	60	17	13	0	11	0	8	98	98	1	1	0.464	0.541	0.435	NA	0.579	-0.099
64	FAM49B	FAM49B	chr8	-	129879388	129879471	129903311	129903350	129871374	129871496	129970942	129970995	33	27	14	92	55	26	0	4	0	11	88	98	1	1	0.285	0.353	0.375	NA	0.288	0.05
82	RAB2A	RAB2A	chr8	+	60558851	60558923	60572045	60572113	60516909	60517253	60584207	60584290	14	7	0	9	6	4	0	1	0	0	98	98	1	1	0.609	0.538	0	NA	1	-0.618
84	IKBKB	IKBKB	chr8	+	42272082	42272205	42288633	42288728	42271301	42271469	42290155	42290273	0	1	0	1	0	0	0	1	0	2	98	98	1	1	0	1	NA	NA	0.333	0.167
85	ADAM9	ADAM9	chr8	+	39082967	39083073	39090046	39090188	39082640	39082721	39091258	39091346	2	3	0	11	0	0	0	1	1	3	98	98	1	1	0.154	1	NA	0	0.25	0.452
93	ASAH1	ASAH1	chr8	-	18062278	18062423	18063184	18063230	18061685	18061740	18064456	18064531	40	11	20	39	29	15	0	4	0	1	95	98	1	1	0.514	0.281	0.579	NA	0.805	-0.347
104	LINC-PINT	LINC-PINT	chr7	-	130959868	130959970	130967388	130967517	130944159	130945835	130984052	130984109	36	7	0	21	1	0	0	5	0	1	98	98	1	1	0.632	0.875	NA	NA	0.833	-0.079
107	NAMPT	NAMPT	chr7	-	106254363	106254504	106263391	106263617	106253016	106253151	106268463	106268600	0	1	0	1558	790	321	0	0	24	229	98	98	1	1	0	0.001	0	0	0	0
108	NAMPT	NAMPT	chr7	-	106263391	106263617	106268463	106268600	106261587	106261707	106269153	106269312	680	331	111	1	1	1	9	124	0	0	98	98	1	1	0.999	0.997	0.991	1	1	-0.004
115	DMTF1	DMTF1	chr7	+	87164933	87165050	87166482	87166605	87163494	87163617	87170994	87171089	9	7	4	15	11	0	0	1	0	0	98	98	1	1	0.375	0.389	1	NA	1	-0.412
122	IKZF1	IKZF1	chr7	+	50376532	50376793	50391728	50391833	50327637	50327757	50399917	50405100	3	1	5	2	0	2	0	0	0	3	98	98	1	1	0.6	1	0.714	NA	0	0.771

124	IKZF1	IKZF1	chr7	+	50382539	50382707	50387344	50387470	50327637	50327757	50391728	50391833	15	1	3	5	2	5	0	3	0	3	98	98	1	1	0.75	0.333	0.375	NA	0.5	-0.014
125	IKZF1	IKZF1	chr7	+	50387344	50387470	50391728	50391833	50327637	50327757	50399917	50405100	1	0	0	2	0	2	0	0	0	3	98	98	1	1	0.333	NA	0	NA	0	0.167
131	FBXL13	FBXL13	chr7	-	102931880	102931933	102963532	102963665	102926273	102926374	102968021	102968117	26	1	0	30	18	2	0	0	0	5	98	98	1	1	0.464	0.053	0	NA	0	0.172
132	CCM2	CCM2	chr7	+	45068442	45068579	45069825	45069961	45064462	45064646	45072725	45072783	9	8	6	11	11	10	0	4	0	1	98	98	1	1	0.45	0.421	0.375	NA	0.8	-0.385
134	H2AFV	H2AFV	chr7	-	44840898	44841012	44843276	44843354	44835528	44835658	44847968	44848126	4	8	1	7	1	10	0	0	0	3	98	98	1	1	0.364	0.889	0.091	NA	0	0.448
136	GGCT	GGCT	chr7	-	30498802	30498938	30500535	30500681	30496620	30497235	30504568	30504841	2	0	0	2	0	0	0	2	0	0	98	98	1	1	0.5	NA	NA	NA	1	-0.5
147	CSGALNACT1	CSGALNACT1	chr8	-	19585165	19585317	19591159	19591278	19505200	19506130	19601770	19601865	1	0	0	3	0	0	0	1	0	0	98	98	1	1	0.25	NA	NA	NA	1	-0.75
149	NUP43	NUP43	chr6	-	149742389	149742570	149743637	149743715	149738642	149738778	149745939	149746062	2	1	0	0	1	0	0	2	0	0	98	98	1	1	1	0.5	NA	NA	1	-0.25
168	VNN2	VNN2	chr6	-	132752460	132752749	132753417	132753491	132751144	132751518	132755842	132756035	34	5	4	330	178	186	0	3	0	32	98	98	1	1	0.093	0.027	0.021	NA	0.086	-0.039
169	VNN2	VNN2	chr6	-	132752672	132752749	132753417	132753491	132751144	132751518	132755842	132756035	34	5	4	115	82	84	0	3	0	10	98	98	1	1	0.228	0.057	0.045	NA	0.231	-0.121
179	SNX3	SNX3	chr6	-	108214497	108214622	108222949	108223045	108211216	108212254	108260759	108261260	6	5	3	13	14	5	0	0	0	1	98	98	1	1	0.316	0.263	0.375	NA	0	0.318
180	SNX14	SNX14	chr6	-	85557975	85558060	85565331	85565419	85549722	85549879	85567533	85567577	9	1	3	4	2	0	0	3	0	2	98	98	1	1	0.692	0.333	1	NA	0.6	0.075
181	SNX14	SNX14	chr6	-	85565331	85565419	85567533	85567577	85557975	85558060	85572136	85572215	5	0	0	13	6	0	0	0	0	2	93	98	1	1	0.288	0	NA	NA	0	0.144
189	KLHDC3	KLHDC3	chr6	+	43017852	43017968	43018144	43018216	43017518	43017695	43018342	43018556	7	7	0	3	2	0	0	4	0	2	98	98	1	1	0.7	0.778	NA	NA	0.667	0.072
190	CCND3	CCND3	chr6	-	41940369	41940585	42030008	42030190	41937234	41937394	42048500	42048894	0	0	0	13	20	6	0	1	1	23	98	98	1	1	0	0	0	0	0.042	-0.021
192	KCTD20	KCTD20	chr6	+	36470051	36470257	36479120	36479223	36442766	36443111	36479590	36479711	5	3	0	4	1	0	0	1	0	2	98	98	1	1	0.556	0.75	NA	NA	0.333	0.32
193	KCTD20	KCTD20	chr6	+	36470051	36470257	36479120	36479223	36442766	36443111	36481561	36481759	5	0	0	0	1	0	0	1	0	0	98	98	1	1	1	0	NA	NA	1	-0.5
194	KCTD20	KCTD20	chr6	+	36479120	36479223	36479590	36479711	36470051	36470257	36481561	36481759	1	0	0	8	5	2	0	4	0	1	98	98	1	1	0.111	0	0	NA	0.8	-0.763
214	HLA-F	HLA-F	chr6	+	29725030	29725306	29725446	29725563	29724172	29724448	29726402	29726526	120	51	32	2	0	3	0	24	0	0	98	98	1	1	0.984	1	0.914	NA	1	-0.034
224	FAM65B	FAM65B	chr6	-	24835702	24835871	24839090	24839272	24832255	24832391	24842861	24843554	81	33	16	61	28	18	0	4	0	21	98	98	1	1	0.57	0.541	0.471	NA	0.16	0.367
228	XAF1	XAF1	chr17	+	6759661	6759718	6760405	6760601	6758088	6758224	6762154	6762240	11	14	1	3	6	1	0	13	0	10	98	98	1	1	0.786	0.7	0.5	NA	0.565	0.097
229	XAF1	XAF1	chr17	+	6760405	6760601	6762154	6762240	6758088	6758224	6770642	6770984	0	2	0	5	5	0	0	0	0	11	98	98	1	1	0	0.286	NA	NA	0	0.143
230	XAF1	XAF1	chr17	+	6760405	6760601	6762154	6762240	6759661	6759718	6770642	6770984	3	7	0	5	6	0	0	6	0	11	98	98	1	1	0.375	0.538	NA	NA	0.353	0.104
235	FYN	FYN	chr6	-	111699514	111699670	111700103	111700268	111696276	111696456	111702884	111703034	0	0	1	5	3	0	0	0	0	7	98	98	1	1	0	0	1	NA	0	0.333
238	HNRNPR	HNRNPR	chr1	-	23323555	23323732	23333517	23333631	23321527	23321663	23337753	23337861	7	0	0	6	3	0	0	0	0	1	98	98	1	1	0.538	0	NA	NA	0	0.269
243	CKLF-CMTM1	CKLF-CMTM1	chr16	+	66558189	66558348	66563121	66563217	66552562	66552793	66577103	66577202	69	49	18	17	11	4	0	7	0	2	98	98	1	1	0.802	0.817	0.818	NA	0.778	0.034
244	CKLF	CKLF	chr16	+	66558189	66558348	66563121	66563217	66552562	66552793	66565885	66566287	71	53	18	47	24	20	0	9	0	7	98	98	1	1	0.602	0.688	0.474	NA	0.562	0.026
245	ASB6	ASB6	chr9	-	129638572	129638659	129639201	129639310	129634603	129638457	129639401	129639508	2	0	4	4	2	0	0	1	0	1	98	98	1	1	0.333	0	1	NA	0.5	-0.056
246	GUSB	GUSB	chr7	-	65976014	65976202	65979833	65979911	65974918	65975071	65980223	65980409	1	0	0	0	0	1	1	0	0	0	98	98	1	1	1	NA	0	1	NA	-0.5
248	RHOT1	RHOT1	chr17	+	32209349	32209445	32211115	32211238	32208106	32208309	32224615	32225771	3	0	0	4	0	5	0	1	0	2	98	98	1	1	0.429	NA	0	NA	0.333	-0.119
249	MTHFD2	MTHFD2	chr2	+	74201381	74201479	74205704	74205889	74198562	74198742	74207703	74207826	0	2	0	7	1	0	0	0	0	2	98	98	1	1	0	0.667	NA	NA	0	0.334
253	USP25	USP25	chr21	+	15849776	15849872	15864267	15864446	15842397	15842540	15866265	15866344	1	0	0	8	8	0	0	0	0	6	98	98	1	1	0.111	0	NA	NA	0	0.056
254	USB1	USB1	chr16	+	58014272	58014326	58017333	58017439	58009928	58010112	58018971	58019055	14	4	1	6	1	0	0	3	0	4	98	98	1	1	0.7	0.8	1	NA	0.429	0.404
255	AGO4	AGO4	chr1	+	35832436	35832570	35833989	35834174	35832056	35832185	35835833	35835993	21	12	18	18	24	12	0	15	0	2	98	98	1	1	0.538	0.333	0.6	NA	0.882	-0.392
258	MEFV	MEFV	chr16	-	3249430	3249780	3254157	3254790	3247015	3247246	3256310	3256627	81	26	30	0	2	1	4	16	0	0	98	98	1	1	1	0.929	0.968	1	1	-0.034
260	STK38L	STK38L	chr12	+	27315014	27315117	27315288	27315350	27314503	27314658	27317335	27317453	2	0	1	16	5	0	0	2	0	3	98	98	1	1	0.111	0	1	NA	0.4	-0.03
262	WAC	WAC	chr10	+	28595732	28596041	28608185	28608431	28590719	28590832	28610698	28610821	26	13	2	26	9	9	0	3	0	1	98	98	1	1	0.5	0.591	0.182	NA	0.75	-0.326
273	SEC11A	SEC11A	chr15	-	84670724	84670782	84680712	84680832	84669536	84670069	84687624	84687774	0	0	0	6	4	6	0	4	0	5	98	98	1	1	0	0	0	NA	0.444	-0.444
276	SEC11A	SEC11A	chr15	-	84687624	84687774	84691534	84691644	84680712	84680832	84716024	84716460	5	2	0	0	2	0	0	0	0	4	98	98	1	1	1	0.5	NA	NA	0	0.75
277	TM6SF1	TM6SF1	chr15	+	83124671	83124776	83126754	83126847	83119577	83119681	83127357	83127477	3	0	0	11	10	8	0	0	0	1	98	98	1	1	0.214	0	0	NA	0	0.071
278	BTBD1	BTBD1	chr15	-	83018706	83018853	83020674	83020762	83016428	83018225	83030135	83030328	3	4	0	5	2	0	0	0	0	3	98	98	1	1	0.375	0.667	NA	NA	0	0.521
287	XPO6	XPO6	chr16	-	28177219	28177332	28180940	28181031	28175897	28176095	28208986	28209122	4	6	3	18	25	6	0	2	0	15	98	98	1	1	0.182	0.194	0.333	NA	0.118	0.118
288	XPO6	XPO6	chr16	-	28177219	28177332	28180940	28181031	28175897	28176095	28211365	28211918	50	24	9	18	34	6	0	18	0	15	98	98	1	1	0.735	0.414	0.6	NA	0.545	0.038
290	EDC3	EDC3	chr15	-	74635408	74635626	74640465	74640619	74630557	74632946	74655732	74656068	1	2	0	1	1	0	3	1	0	0	98	98	1	1	0.5	0.667	NA	1	1	-0.416
293	USP8	USP8	chr15	+	50481480	50482065	50484274	50484361	50477275	50477499	50489800	50489881	13	6	1	4	2	2	0	5	0	2	98	98	1	1	0.765	0.75	0.333	NA	0.714	-0.098
294	PLAGL1	PLAGL1	chr6	-	143947984	143948460	143960468	143960542	143940299	143942663	143964786	143964818	5	3	0	2	0	0	0	1	0	0	98	98	1	1	0.714	1	NA	NA	1	-0.143
301	YTHDF3	YTHDF3	chr8	+	63175330	63175416	63186146	63187745	63169386	63169411	63209682	63212788	3	6	0	19	3	0	0	3	0	5	98	98	1	1	0.136	0.667	NA	NA	0.375	0.027
306	PYGL	PYGL	chr14	-	50914700	50914815	50915335	50915499	50913028	50913130	50915824	50915971	46	46	19	68	25	23	0	19	0	14	98	98	1	1	0.404	0.648	0.452	NA	0.576	-0.075
307	NIPA2	NIPA2	chr15	+	22839654	22839790	22845145	22845267	22838640	22838921	22851638	22851870	0	0	0	0	2	0	0	1	0	0	98	98	1	1	NA	0	NA	NA	1	-1
311	TECPR2	TECPR2	chr14	+	102424978	102425291	102431795	102432128	102414635	102414793	102434234	102435211	36	8	2	19	5	3	0	1	0	1	98	98	1	1	0.655	0.615	0.4	NA	0.5	0.057
314	NME8	NME8	chr7	+	37864347	37864421	37867701	37867898	37863395	37863462	37876831	37877007	8	9	4	1	1	1	0	4	0	1	98	98	1	1	0.889	0.9	0.8	NA	0.8	0.063
318	FOPNL	FOPNL	chr16	-	15873490	15873627	15879803	15879888	15865718	15867516	15884007	15884205	1	1	0	0	1	0	0	0	0	1	98	98	1	1	1	0.5	NA	NA	0	0.75
329	CTSS	CTSS	chr1	-	150751780	150752008	150755000	150755150	150747776	150747879	150757857	150757980	414	132	122	2	0	5	2	125	0	2	98	98	1	1	0.995	1	0.961	1	0.984	-0.007
332	EIF2AK2	EIF2AK2	chr2	-	37122505	37122664	37126288	37126411	37119958	37120139	37135483	37135546	17	7	0	12	13	2	0	9	0	6	98	98	1	1	0.586	0.35	0	NA	0.6	-0.288
336	ACTN1	ACTN1	chr14	-	68878457	68878523	68878988	68879069	68877081	68877240	68879961	68880108	85	49	27	0	1	0	1	15	0	0	98	98	1	1	1	0.98	1	1	1	-0.007
337	RDH11	RDH11	chr14	-	67685014	67685204	67690211	67690421	67676800	67678423	67691139	67691244	9	0	0	1	0	0	0	0	0	1	98	98	1	1	0.9	NA	NA	NA	0	0.9
349	ITCH	ITCH	chr20	+	34393790	34393881	34408650	34408792	34369393	34369470	34412514	34412639	9	6	0	28	28	16	0	4	0	2	98	98	1	1	0.243	0.176	0	NA	0.667	-0.527
356	EPG5	EPG5	chr18	-	45879012	45879214	45880074	45880223	45878375	45878448	45882273	45882487	1	5	0	4	1	6	0	1	0	0	98	98	1	1	0.2	0.833	0	NA	1	-0.656
357	EPG5	EPG5	chr18	-	45887750	45887907	45889797	45889940	45884616	45884811	45899403	45899566	6	3	0	3	0	6	0	1	0	0	98	98	1	1	0.667	1	0	NA	1	-0.444
359	ARMC2	ARMC2	chr6	+	108858198	108858271	108868823	108868995	108854224	108854485	108876142	108876350	3	0	1	5	3	0	0	3	0	1	98	98	1	1	0.375	0	1	NA	0.75	-0.292
362	PAN3	PAN3	chr13	+	28177864	28177935	28220230	28220378	28176492	28176559	28256291	28256539	27	25	9	14	0	1	0	7	0	2	98	98	1	1	0.659	1	0.9	NA	0.778	0.075
363	NUPL1	NUPL1	chr13	+	25309246	25309282	25312882	25313032	25307805	25307948	25313613	25313751	10	3	3	15	2	2	0	5	0	2	85	98	1	1	0.435	0.634	0.634	NA	0.742	-0.174
365	ZNF268	ZNF268	chr12	+	133187871	133188072	133191488	133191615	133181945	133182030	133191907	133192003	0	0	1	0	2	0	0	0	0	2	98	98	1	1	NA	0	1	NA	0	0.5
369	OGFOD2	OGFOD2	chr12	+	122975810	122975867	122976653	122976767	122974702	122975121	122976870	122976970	0	0	1	0	0	0	0	0	0	1	98	98	1	1	NA	NA	1	NA	0	1
372	MED13L	MED13L	chr12	-	116096668	116096752	116111427	116111512	116022455	116022601	116237467	116237705	84	36	16	56	27	5	0	10	0	16	98	98	1	1	0.6	0.571	0.762	NA	0.385	0.259
373	MED13L	MED13L	chr12	-	116111427	116111512	116237467	116237705	116096668	116096752	116277059	116277186	56	19	3	47	30	16	0	8	0	7	98	98	1	1	0.544	0.388	0.158	NA	0.533	-0.17
375	NLRP1	NLRP1	chr17	-	5533896	5533988	5536850	5536940	5533303	5533384	5539414	5539585	36	25	5	31	12	6	0	10	0	7	98	98	1	1	0.537	0.676	0.455	NA	0.588	-0.032
378	YIPF1	YIPF1	chr1	-	53855000	53855051	53860055	53860153	53851718	53852270	53866199	53866382	9	2	3	4	0	0	0	1	0	2	98	98	1	1	0.692	1	1	NA	0.333	0.564
379	YIPF1	YIPF1	chr1	-	53860055	53860153	53866199	53866382	53855000	53855051	53866757	53866924	10	2	5	3	3	3	0	1	0	0	98	98	1	1	0.769	0.4	0.625	NA	1	-0.402
380	YIPF1	YIPF1	chr1	-	53866199	53866382	53866757	53866924	53860055	53860153	53871371	53871488	13	2	7	9	2	3	0	4	0	2	98	98	1	1	0.591	0.5	0.7	NA	0.667	-0.07
384	MARK3	MARK3	chr14	+	103465556	103465793	103465971	103466091	103462404	103462461	103466342	103466442	10	5	4	15	1	3	0	5	0	1	98	98	1	1	0.4	0.833	0.571	NA	0.833	-0.232
388	NF2	NF2	chr22	+	29636750	29636876	29639089	29639212	29603555	29604112	29642201	29642285	2	0	0	1	0	0	0	1	0	0	98	98	1	1	0.667	NA	NA	NA	1	-0.333
390	PLXNC1	PLXNC1	chr12	+	94298631	94298795	94300909	94301057	94297315	94297423	94303755	94303896	31	26	12	68	30	19	0	14	0	11	98	98	1	1	0.313	0.464	0.387	NA	0.56	-0.172
391	ZNF512	ZNF512	chr2	+	27599969	27600053	27600690	27600815	27599582	27599678	27601355	27601442	6	3	0	4	2	0	0	1	0	0	98	98	1	1	0.6	0.6	NA	NA	1	-0.4
395	IL32	IL32	chr16	+	3067553	3067613	3067983	3068010	3067376	3067415	3068179	3068239	14	13	2	11	2	0	0	3	0	1	98	76	1	1	0.497	0.834	1	NA	0.699	0.078
397	ARHGAP9	ARHGAP9	chr12	-	57473602	57473708	57474041	57474176	57472254	57472688	57474422	57474476	214	71	70	116	35	41	0	27	0	16	98	98	1	1	0.648	0.67	0.631	NA	0.628	0.022
399	PTGES3	PTGES3	chr12	-	56666203	56666266	56670274	56670364	56664775	56664800	56671748	56671847	6	3	0	6	13	1	0	0	0	5	98	98	1	1	0.5	0.187	0	NA	0	0.229
408	PI4KB	PI4KB	chr1	-	151324744	151324917	151326171	151326373	151315572	151316509	151327270	151327657	3	1	0	1	2	0	0	4	0	0	98	98	1	1	0.75	0.333	NA	NA	1	-0.459
409	PSMA3	PSMA3	chr14	+	58247749	58247832	58252118	58252242	58244804	58244941	58257744	58257846	0	1	1	11	4	0	0	2	0	2	98	98	1	1	0	0.2	1	NA	0.5	-0.1
411	P2RX7	P2RX7	chr12	+	121160901	121160974	121162423	121162520	121156078	121156147	121165356	121165437	4	0	0	0	0	0	0	5	0	1	98	98	1	1	1	NA	NA	NA	0.833	0.167
414	ADGRE3	ADGRE3	chr19	-	14654981	14655165	14658512	14658550	14651084	14651204	14661962	14662118	98	26	12	77	34	21	0	7	0	11	87	98	1	1	0.589	0.463	0.392	NA	0.418	0.063
415	ADGRE3	ADGRE3	chr19	-	14658512	14658550	14661962	14662118	14654981	14655165	14663417	14663540	72	31	17	66	13	15	0	5	0	5	98	87	1	1	0.492	0.679	0.502	NA	0.47	0.088
416	ADGRE3	ADGRE3	chr19	-	14661962	14662118	14663417	14663540	14658512	14658550	14668801	14668852	96	25	4	102	33	12	0	5	0	8	98	98	1	1	0.485	0.431	0.25	NA	0.385	0.004
417	ADGRE3	ADGRE3	chr19	-	14663417	14663540	14668801	14668852	14661962	14662118	14674745	14674918	11	5	0	72	32	17	0	1	0	4	98	98	1	1	0.133	0.135	0	NA	0.2	-0.111
419	LCP1	LCP1	chr13	-	46158521	46158651	46158825	46158989	46156437	46156570	46159598	46159686	50	39	15	250	100	86	0	23	0	46	98	98	1	1	0.167	0.281	0.149	NA	0.333	-0.134
421	DLG1	DLG1	chr3	-	197075836	197075870	197076585	197076685	197069218	197069260	197081050	197081117	1	0	0	2	0	0	0	4	0	0	98	83	1	1	0.297	NA	NA	NA	1	-0.703
423	KDM6A	KDM6A	chrX	+	45060021	45060156	45060608	45060764	45059246	45059466	45061323	45061419	7	0	0	11	8	2	0	0	0	4	98	98	1	1	0.389	0	0	NA	0	0.13
426	QKI	QKI	chr6	+	163563419	163563719	163566720	163566795	163561981	163562069	163570693	163578596	28	11	10	10	4	5	0	3	0	3	98	98	1	1	0.737	0.733	0.667	NA	0.5	0.212
427	QKI	QKI	chr6	+	163563443	163563719	163566720	163566795	163561981	163562069	163570693	163578596	2	2	0	10	4	5	0	0	0	3	98	98	1	1	0.167	0.333	0	NA	0	0.167
429	CLEC7A	CLEC7A	chr12	-	10123244	10123363	10125296	10125448	10116780	10118590	10127746	10127845	48	19	15	92	36	25	0	19	0	30	98	98	1	1	0.343	0.345	0.375	NA	0.388	-0.034
432	FCHO2	FCHO2	chr5	+	73006444	73006549	73015625	73015724	72990711	72990864	73017211	73017308	15	3	0	22	9	1	0	6	0	2	98	98	1	1	0.405	0.25	0	NA	0.75	-0.532
434	SYNJ2BP-COX16	SYNJ2BP-COX16	chr14	-	70342657	70342729	70373032	70373131	70329173	70329236	70375675	70375771	8	0	0	0	0	0	0	0	0	2	98	98	1	1	1	NA	NA	NA	0	1
436	TLE4	TLE4	chr9	+	79612655	79612718	79627373	79627448	79576132	79576177	79652592	79652794	22	4	3	7	1	0	0	0	0	2	98	98	1	1	0.759	0.8	1	NA	0	0.853
446	FNIP1	FNIP1	chr5	-	131677702	131677872	131679028	131679175	131671504	131672924	131698916	131699002	16	2	2	34	15	2	0	1	0	6	98	98	1	1	0.32	0.118	0.5	NA	0.143	0.17
450	KANSL3	KANSL3	chr2	-	96602115	96602338	96602752	96602862	96601642	96601776	96604249	96604380	3	1	0	1	0	0	0	0	1	2	98	98	1	1	0.75	1	NA	0	0	0.875
451	AHCTF1	AHCTF1	chr1	-	246863923	246864116	246867243	246867351	246861958	246862153	246867660	246867811	1	0	0	26	16	4	0	0	0	3	98	98	1	1	0.037	0	0	NA	0	0.012
452	SPOP	SPOP	chr17	-	49636758	49636809	49668026	49668078	49622732	49622876	49677932	49678163	2	1	0	2	5	0	0	2	0	0	98	98	1	1	0.5	0.167	NA	NA	1	-0.666
455	APLP2	APLP2	chr11	+	130109428	130109505	130120705	130120818	130069820	130070082	130121613	130121810	21	12	5	53	34	10	0	9	0	20	98	98	1	1	0.284	0.261	0.333	NA	0.31	-0.017
456	MEF2C	MEF2C	chr5	-	88760987	88761125	88761184	88761328	88751856	88752043	88804597	88804801	5	2	0	1	0	0	0	5	0	0	98	98	1	1	0.833	1	NA	NA	1	-0.084
460	SNX17	SNX17	chr2	+	27371268	27371468	27372622	27372740	27370495	27370806	27373246	27373311	0	1	0	2	0	0	0	0	0	1	98	98	1	1	0	1	NA	NA	0	0.5
461	SNX17	SNX17	chr2	+	27372622	27372740	27373039	27373113	27371268	27371343	27373246	27373311	6	1	1	1	0	0	1	4	0	0	98	98	1	1	0.857	1	1	1	1	-0.048
464	GPATCH8	GPATCH8	chr17	-	44464471	44464544	44474828	44474903	44436477	44436545	44503325	44503602	3	0	0	3	0	0	0	0	0	1	98	98	1	1	0.5	NA	NA	NA	0	0.5
466	ARNTL	ARNTL	chr11	+	13349944	13350070	13354300	13354448	13309986	13310059	13356738	13356777	8	5	5	10	2	1	0	0	0	5	98	98	1	1	0.444	0.714	0.833	NA	0	0.664
470	POLG	POLG	chr15	-	89322741	89322902	89323403	89323511	89321961	89322015	89323814	89323901	7	3	0	2	0	0	0	3	0	2	98	98	1	1	0.778	1	NA	NA	0.6	0.289
472	TRAF3IP3	TRAF3IP3	chr1	+	209779314	209779374	209780469	209780606	209778110	209778173	209781344	209781458	23	6	0	8	2	0	0	0	0	3	98	98	1	1	0.742	0.75	NA	NA	0	0.746
485	SLC9A8	SLC9A8	chr20	+	49839540	49839599	49845035	49845119	49823060	49823141	49849578	49849680	21	3	3	18	4	0	0	6	0	7	98	98	1	1	0.538	0.429	1	NA	0.462	0.194
486	SLC9A8	SLC9A8	chr20	+	49845035	49845119	49849578	49849680	49839540	49839599	49855437	49855581	13	2	0	14	9	0	0	4	0	4	98	98	1	1	0.481	0.182	NA	NA	0.5	-0.168
494	NDUFS1	NDUFS1	chr2	-	206149817	206149925	206152418	206152510	206149019	206149096	206153617	206153682	3	4	2	10	6	0	0	1	0	0	98	98	1	1	0.231	0.4	1	NA	1	-0.456
496	FAR2	FAR2	chr12	+	29293299	29293475	29297020	29297200	29270411	29270638	29307657	29307835	2	3	2	12	13	3	0	2	0	1	98	98	1	1	0.143	0.187	0.4	NA	0.667	-0.424
497	RASGRP4	RASGRP4	chr19	-	38417051	38417168	38419859	38420013	38413393	38413474	38420130	38420262	20	18	4	1	0	1	4	7	0	0	98	98	1	1	0.952	1	0.8	1	1	-0.083
501	ELP3	ELP3	chr8	+	28097218	28097318	28099827	28099966	28093066	28093233	28106712	28106783	1	0	0	4	0	0	0	0	0	2	98	98	1	1	0.2	NA	NA	NA	0	0.2
502	RPAIN	RPAIN	chr17	+	5425970	5426082	5426235	5426299	5422768	5422829	5428070	5428211	0	2	0	2	0	2	0	2	0	1	98	98	1	1	0	1	0	NA	0.667	-0.334
504	RPAIN	RPAIN	chr17	+	5425970	5426082	5428070	5428211	5422768	5422829	5432541	5433020	0	2	0	2	0	0	0	2	0	0	98	98	1	1	0	1	NA	NA	1	-0.5
507	SCP2	SCP2	chr1	+	52941795	52941853	52948008	52948080	52927228	52927465	52950754	52950886	0	0	0	1	1	0	0	1	0	0	98	98	1	1	0	0	NA	NA	1	-1
511	NAAA	NAAA	chr4	-	75914867	75914985	75918760	75918789	75913655	75914338	75919908	75919975	1	0	0	3	0	1	0	0	0	2	78	98	1	1	0.295	NA	0	NA	0	0.147
514	DCAF8	DCAF8	chr1	-	160243959	160244034	160261284	160261358	160239696	160240370	160262116	160262528	1	0	0	3	2	0	0	1	0	3	98	98	1	1	0.25	0	NA	NA	0.25	-0.125
515	DCAF8	DCAF8	chr1	-	160243959	160244034	160261284	160261358	160239696	160240370	160262448	160262560	1	2	0	4	2	0	0	0	0	3	98	98	1	1	0.2	0.5	NA	NA	0	0.35
523	PPP1R12A	PPP1R12A	chr12	-	79805591	79805768	79806165	79806333	79798493	79798584	79807225	79807330	22	12	3	8	2	0	0	6	0	0	98	98	1	1	0.733	0.857	1	NA	1	-0.137
533	SLC50A1	SLC50A1	chr1	+	155136827	155136951	155137560	155137722	155136298	155136376	155137978	155138098	2	0	0	0	0	0	0	0	0	1	98	98	1	1	1	NA	NA	NA	0	1
536	STX3	STX3	chr11	+	59795371	59795482	59797282	59797396	59793379	59793514	59800854	59805882	24	12	8	21	11	14	0	5	0	5	98	98	1	1	0.533	0.522	0.364	NA	0.5	-0.027
538	SNX27	SNX27	chr1	+	151662165	151662270	151665932	151666011	151660797	151660862	151668471	151668635	17	0	6	21	11	3	0	5	0	5	98	98	1	1	0.447	0	0.667	NA	0.5	-0.129
541	ATXN3	ATXN3	chr14	-	92093251	92093318	92096092	92096137	92088729	92088817	92096673	92096838	7	0	2	9	1	0	0	1	0	1	94	98	1	1	0.448	0	1	NA	0.51	-0.027
542	ATXN3	ATXN3	chr14	-	92093745	92093831	92096092	92096137	92093251	92093318	92096673	92096838	10	1	2	4	2	0	0	1	0	1	94	98	1	1	0.723	0.343	1	NA	0.51	0.179
543	ATXN3	ATXN3	chr14	-	92093745	92093831	92096092	92096137	92093251	92093318	92106528	92106621	3	1	0	4	2	0	0	0	0	1	94	98	1	1	0.439	0.343	NA	NA	0	0.391
549	CREBBP	CREBBP	chr16	-	3778699	3778817	3780731	3780878	3778010	3778182	3781203	3781306	37	7	1	26	9	6	0	5	0	7	98	98	1	1	0.587	0.437	0.143	NA	0.417	-0.028
554	SORL1	SORL1	chr11	+	121608103	121608176	121611075	121611158	121607185	121607290	121612735	121612832	73	47	30	239	98	59	0	12	0	22	98	98	1	1	0.234	0.324	0.337	NA	0.353	-0.055
555	NIN	NIN	chr14	-	50821873	50822077	50830463	50830517	50806736	50806818	50831005	50831121	5	7	0	34	24	4	0	1	0	6	98	98	1	1	0.128	0.226	0	NA	0.143	-0.025
558	ZNF185	ZNF185	chrX	+	152922719	152922809	152928574	152928661	152922172	152922256	152931671	152931776	5	3	0	11	2	0	1	1	0	0	98	98	1	1	0.312	0.6	NA	1	1	-0.544
559	R3HCC1L	R3HCC1L	chr10	+	98208100	98209899	98231511	98231687	98163292	98163397	98234445	98234516	4	2	0	2	3	0	0	2	0	0	98	98	1	1	0.667	0.4	NA	NA	1	-0.466
568	C1orf162	C1orf162	chr1	+	111477333	111477428	111477650	111477775	111476797	111476867	111477982	111478512	7	2	0	3	1	0	0	5	1	9	98	98	1	1	0.7	0.667	NA	0	0.357	0.505
584	ARHGEF2	ARHGEF2	chr1	-	155954901	155954969	155957712	155957882	155952627	155952828	155958319	155958396	9	15	12	25	14	2	0	5	0	7	98	98	1	1	0.265	0.517	0.857	NA	0.417	0.129
590	SOAT1	SOAT1	chr1	+	179302676	179302802	179323436	179323495	179293713	179293936	179335505	179335657	1	1	0	8	6	0	0	3	0	0	98	98	1	1	0.111	0.143	NA	NA	1	-0.873
598	BLOC1S6	BLOC1S6	chr15	+	45603099	45603187	45605427	45605514	45592134	45592276	45606394	45609716	1	0	1	2	3	0	0	0	0	4	98	98	1	1	0.333	0	1	NA	0	0.444
601	ECD	ECD	chr10	-	73152292	73152421	73154255	73154448	73148275	73148404	73156274	73156453	3	0	0	4	0	0	0	1	0	1	98	98	1	1	0.429	NA	NA	NA	0.5	-0.071
602	NDUFB6	NDUFB6	chr9	-	32558909	32558954	32570959	32571052	32553525	32553944	32572880	32573184	2	2	2	6	15	12	0	0	0	5	98	94	1	1	0.242	0.113	0.138	NA	0	0.164
616	ACAP2	ACAP2	chr3	-	195289120	195289231	195291705	195291815	195285795	195285857	195292264	195292452	20	13	2	31	13	9	0	6	0	7	98	98	1	1	0.392	0.5	0.182	NA	0.462	-0.104
618	UBR4	UBR4	chr1	-	19148562	19148626	19150576	19150793	19147992	19148127	19151642	19151859	5	8	0	8	4	3	0	3	0	1	98	98	1	1	0.385	0.667	0	NA	0.75	-0.399
619	APAF1	APAF1	chr12	+	98725414	98725540	98727172	98727316	98723638	98723764	98732419	98735433	15	4	5	32	10	4	0	2	0	4	98	98	1	1	0.319	0.286	0.556	NA	0.333	0.054
622	NDUFB5	NDUFB5	chr3	+	179616982	179617044	179618414	179618521	179615982	179616049	179623919	179624500	8	0	3	5	0	0	0	5	1	5	98	98	1	1	0.615	NA	1	0	0.5	0.557
626	PRUNE	PRUNE	chr1	+	151017811	151017904	151025514	151025673	151008390	151008671	151027232	151027327	2	0	0	5	3	0	0	0	0	1	98	98	1	1	0.286	0	NA	NA	0	0.143
627	PRUNE	PRUNE	chr1	+	151018466	151018669	151025514	151025673	151017811	151017904	151027232	151027327	0	0	3	5	3	0	0	1	0	1	98	98	1	1	0	0	1	NA	0.5	-0.167
630	SRP19	SRP19	chr5	+	112862507	112862583	112864456	112864528	112861187	112861417	112864620	112864732	1	0	0	2	2	0	0	1	0	0	98	98	1	1	0.333	0	NA	NA	1	-0.834
632	TK2	TK2	chr16	-	66536963	66537017	66541878	66541953	66531379	66531469	66548977	66549009	5	0	0	23	4	0	0	2	0	2	98	98	1	1	0.179	0	NA	NA	0.5	-0.41
634	OPA1	OPA1	chr3	+	193617177	193617285	193617783	193617837	193615673	193615770	193618868	193618936	11	0	1	3	0	0	0	2	0	0	98	98	1	1	0.786	NA	1	NA	1	-0.107
640	DMXL2	DMXL2	chr15	-	51464674	51464876	51465565	51465651	51463378	51463496	51466183	51466311	33	5	3	25	6	3	0	9	0	8	98	98	1	1	0.569	0.455	0.5	NA	0.529	-0.021
642	TMCO3	TMCO3	chr13	+	113503495	113503588	113510237	113510424	113501751	113501880	113520615	113520733	5	3	3	1	2	4	0	1	0	3	98	98	1	1	0.833	0.6	0.429	NA	0.25	0.371
644	C11orf21	C11orf21	chr11	-	2300519	2300613	2300683	2300768	2299427	2299707	2301755	2301913	2	0	0	6	0	0	0	0	0	1	98	98	1	1	0.25	NA	NA	NA	0	0.25
648	DAP3	DAP3	chr1	+	155717005	155717128	155721516	155721618	155709772	155709824	155725381	155725490	6	1	2	5	7	1	0	0	0	3	98	98	1	1	0.545	0.125	0.667	NA	0	0.446
649	CDA	CDA	chr1	+	20604927	20605039	20613841	20613899	20588950	20589283	20618451	20618907	15	12	15	26	7	10	0	14	0	3	98	98	1	1	0.366	0.632	0.6	NA	0.824	-0.291
656	SFI1	SFI1	chr22	+	31528689	31528863	31546860	31546971	31508254	31508376	31550253	31550348	3	5	1	4	8	0	0	0	0	2	98	98	1	1	0.429	0.385	1	NA	0	0.605
658	MPHOSPH9	MPHOSPH9	chr12	-	123202623	123203084	123203113	123203375	123202163	123202319	123210055	123210162	1	0	0	4	0	0	0	0	0	1	98	98	1	1	0.2	NA	NA	NA	0	0.2
660	PAPD4	PAPD4	chr5	+	79641104	79641196	79642843	79642910	79640850	79640965	79645122	79645192	10	3	0	6	5	4	0	2	0	1	98	98	1	1	0.625	0.375	0	NA	0.667	-0.334
661	PAPD4	PAPD4	chr5	+	79649061	79649190	79656957	79657001	79648616	79648693	79668891	79669028	18	4	0	13	3	0	0	0	0	4	98	93	1	1	0.568	0.559	NA	NA	0	0.564
662	ABCC5	ABCC5	chr3	-	183985303	183985378	183987769	183987917	183983752	183984894	183988571	183988727	8	9	0	1	0	0	2	1	0	0	98	98	1	1	0.889	1	NA	1	1	-0.055
663	DPP8	DPP8	chr15	-	65450998	65451110	65451959	65452102	65445659	65447006	65456224	65456371	1	1	0	1	0	1	0	0	0	1	98	98	1	1	0.5	1	0	NA	0	0.5

678	MFN2	MFN2	chr1	+	11981969	11982114	11989164	11989343	11980180	11980484	11992554	11992690	7	3	0	29	10	0	0	1	0	7	98	98	1	1	0.194	0.231	NA	NA		0.125	0.088
679	IST1	IST1	chr16	+	71923287	71923380	71924768	71924817	71922473	71922680	71927613	71930637	20	8	6	4	5	3	0	1	0	1	98	98	1	1	0.833	0.615	0.667	NA		0.5	0.205
690	TPM3	TPM3	chr1	-	154171412	154171488	154172028	154172104	154170648	154170711	154172907	154172978	159	96	73	3	0	0	2	34	0	0	98	98	1	1	0.981	1	1		1	1	-0.006
693	FAM126B	FAM126B	chr2	-	200992886	200993003	200997444	200997540	200992280	200992368	201008972	201009088	39	9	6	39	11	3	0	6	0	10	98	98	1	1	0.5	0.45	0.667	NA		0.375	0.164
695	FAM53B	FAM53B	chr10	-	124696157	124696212	124706635	124706887	124681606	124682379	124744012	124744361	12	3	0	4	6	3	0	4	0	0	98	98	1	1	0.75	0.333	0	NA		1	-0.639
696	6-Mar	6-Mar	chr5	+	10386993	10387066	10390331	10390500	10378758	10378832	10391541	10391731	1	0	0	8	1	0	0	2	0	4	98	98	1	1	0.111	0	NA	NA		0.333	-0.278
698	RFWD2	RFWD2	chr1	-	176135009	176135086	176149005	176149074	176116623	176116681	176175909	176176007	3	1	0	118	57	9	0	1	1	17	98	98	1	1	0.025	0.017	0		0	0.056	-0.014
701	RFWD2	RFWD2	chr1	-	176149005	176149074	176175909	176176007	176135009	176135086	176184632	176184692	32	16	3	29	7	0	0	0	0	4	98	98	1	1	0.525	0.696	1	NA		0	0.74
702	KIAA1551	KIAA1551	chr12	+	31960776	31960871	31970188	31970356	31959418	31959491	31980877	31985957	45	20	11	204	96	57	0	5	0	41	98	98	1	1	0.181	0.172	0.162	NA		0.109	0.063
703	KIAA1551	KIAA1551	chr12	+	31970188	31970356	31980877	31985957	31960776	31960871	31987238	31987322	65	52	41	95	43	25	0	18	0	9	98	98	1	1	0.406	0.547	0.621	NA		0.667	-0.142
704	KIAA1551	KIAA1551	chr12	+	31980877	31985957	31987238	31987322	31970188	31970356	31992377	31993107	203	97	57	69	25	16	0	41	0	6	98	98	1	1	0.746	0.795	0.781	NA		0.872	-0.098
711	CSDE1	CSDE1	chr1	-	114739691	114739890	114749820	114750207	114737962	114738072	114757924	114758050	3	3	0	41	19	5	0	0	0	6	98	98	1	1	0.068	0.136	0	NA		0	0.068
715	PPP2R3C	PPP2R3C	chr14	-	35091069	35091207	35095047	35095184	35087950	35088010	35096557	35096633	3	1	1	9	5	4	0	3	0	2	98	98	1	1	0.25	0.167	0.2	NA		0.6	-0.394
716	FNDC3B	FNDC3B	chr3	+	172247532	172247776	172251259	172251541	172226870	172226947	172285925	172285984	20	6	8	18	4	7	0	1	0	5	98	98	1	1	0.526	0.6	0.533	NA		0.167	0.386
725	ZNF687	ZNF687	chr1	+	151288527	151288706	151289094	151289271	151286274	151288406	151289377	151289540	4	1	5	6	0	2	0	0	0	2	98	98	1	1	0.4	1	0.714	NA		0	0.705
726	CD44	CD44	chr11	+	35209964	35210054	35211245	35211449	35208104	35208206	35214851	35214914	5	0	0	6	0	0	0	4	0	1	98	98	1	1	0.455	NA	NA	NA		0.8	-0.345
729	LRRK2	LRRK2	chr12	+	40278090	40278261	40283874	40284133	40277887	40278016	40287350	40287539	102	91	48	177	94	32	0	22	0	29	98	98	1	1	0.366	0.492	0.6	NA		0.431	0.055
737	FOXJ3	FOXJ3	chr1	-	42199101	42199230	42205761	42205863	42194889	42195064	42227882	42227966	2	1	0	8	0	0	0	1	0	0	98	98	1	1	0.2	1	NA	NA		1	-0.4
738	CYB561D1	CYB561D1	chr1	+	109494519	109494623	109495142	109495180	109494078	109494287	109495755	109500441	1	0	0	1	0	0	0	0	0	1	98	87	1	1	0.47	NA	NA	NA		0	0.47
739	EPB41	EPB41	chr1	+	28993329	28993542	28997214	28997319	28987430	28987905	29011864	29011907	46	19	5	27	7	7	0	2	0	0	98	98	1	1	0.63	0.731	0.417	NA		1	-0.407
740	EPB41	EPB41	chr1	+	29058588	29058645	29060421	29060484	29053103	29053312	29064981	29065158	0	2	0	3	0	0	0	1	0	0	98	98	1	1	0	1	NA	NA		1	-0.5
741	EPB41	EPB41	chr1	+	29097806	29097935	29109335	29109437	29064981	29065158	29112367	29112448	31	14	5	13	12	5	0	8	0	0	98	98	1	1	0.705	0.538	0.5	NA		1	-0.419
748	RNF19B	RNF19B	chr1	-	32945513	32945628	32946401	32946564	32944018	32944159	32948221	32948363	67	25	18	53	35	4	0	7	0	12	98	98	1	1	0.558	0.417	0.818	NA		0.368	0.23
759	DEDD2	DEDD2	chr19	-	42201988	42202072	42204461	42204559	42198592	42199829	42209699	42209840	0	0	0	2	0	1	1	0	1	2	98	98	1	1	0	NA	0		0.5	0	-0.25
760	DEDD2	DEDD2	chr19	-	42202050	42202072	42204461	42204559	42198592	42199829	42209699	42209840	0	0	0	3	0	1	1	0	0	3	98	71	1	1	0	NA	0		1	0	-0.5
763	RAB6A	RAB6A	chr11	-	73718612	73718718	73718784	73718890	73716250	73716362	73720845	73720899	0	0	1	11	3	4	0	0	0	2	98	98	1	1	0	0	0.2	NA		0	0.067
765	ACAA1	ACAA1	chr3	-	38129289	38129388	38131595	38131638	38127785	38127866	38131925	38132005	3	0	3	9	8	2	0	0	0	2	92	98	1	1	0.262	0	0.615	NA		0	0.292
766	MAP3K11	MAP3K11	chr11	-	65607921	65608070	65608267	65608448	65607640	65607816	65613017	65614249	25	18	11	17	10	11	0	4	0	7	98	98	1	1	0.595	0.643	0.5	NA		0.364	0.215
769	PECAM1	PECAM1	chr17	-	64341633	64341690	64348259	64348322	64329699	64329722	64350379	64350433	42	38	40	104	68	31	0	9	0	11	98	98	1	1	0.288	0.358	0.563	NA		0.45	-0.047
770	CD86	CD86	chr3	+	122091600	122091650	122103511	122103847	122055361	122055503	122106197	122106500	3	0	0	2	0	0	0	1	0	0	98	98	1	1	0.6	NA	NA	NA		1	-0.4
772	DCLRE1C	DCLRE1C	chr10	-	14945104	14945189	14949035	14949087	14939809	14939869	14953901	14954432	4	1	0	6	1	0	0	2	0	0	98	98	1	1	0.4	0.5	NA	NA		1	-0.55
775	ELOVL1	ELOVL1	chr1	-	43364927	43365008	43365185	43365376	43364737	43364794	43365563	43365623	0	3	0	6	2	0	0	0	0	5	98	98	1	1	0	0.6	NA	NA		0	0.3
776	NLRP3	NLRP3	chr1	+	247429584	247429755	247434102	247434273	247423846	247425599	247443971	247444142	8	0	0	2	0	0	0	4	0	2	98	98	1	1	0.8	NA	NA	NA		0.667	0.133
780	CASP8	CASP8	chr2	+	201272897	201272942	201274888	201274953	201272637	201272776	201276826	201276968	106	49	12	89	36	17	0	6	0	3	94	98	1	1	0.554	0.587	0.424	NA		0.676	-0.154
796	ABI1	ABI1	chr10	-	26755654	26755741	26759061	26759235	26751597	26751783	26765217	26765318	9	13	1	0	0	0	0	12	0	1	98	98	1	1	1	1	1	NA		0.923	0.077
797	ABI1	ABI1	chr10	-	26755654	26755741	26759061	26759238	26751597	26751783	26765217	26765318	12	12	2	0	0	0	0	11	0	1	98	98	1	1	1	1	1	NA		0.917	0.083
799	RFC2	RFC2	chr7	-	74246661	74246763	74249011	74249220	74243145	74243246	74249738	74249780	1	0	0	5	3	2	0	0	0	1	98	98	1	1	0.167	0	0	NA		0	0.056
805	CNIH4	CNIH4	chr1	+	224360494	224360563	224364108	224364455	224356810	224356993	224365878	224365991	3	2	7	2	1	0	0	2	0	0	98	98	1	1	0.6	0.667	1	NA		1	-0.244
816	POGZ	POGZ	chr1	-	151428122	151428413	151430665	151430841	151424954	151425061	151440927	151441086	0	0	0	1	0	0	0	3	0	0	98	98	1	1	0	NA	NA	NA		1	-1
817	POGZ	POGZ	chr1	-	151430665	151430841	151440927	151441086	151428122	151428413	151442080	151442205	4	0	0	1	0	0	0	2	0	0	98	98	1	1	0.8	NA	NA	NA		1	-0.2
822	IFI44	IFI44	chr1	+	78660553	78660654	78662703	78662878	78659311	78659483	78663764	78664078	5	1	0	12	6	0	0	31	6	51	98	98	1	1	0.294	0.143	NA		0	0.378	0.029
825	ZCCHC2	ZCCHC2	chr18	+	62556202	62556297	62558686	62558770	62550347	62550460	62560586	62560644	23	29	2	12	5	2	0	16	0	9	98	98	1	1	0.657	0.853	0.5	NA		0.64	0.03
828	TLR5	TLR5	chr1	-	223137177	223137263	223141647	223141763	223134681	223134864	223143195	223143282	3	1	1	3	0	1	0	0	0	4	98	98	1	1	0.5	1	0.5	NA		0	0.667
835	CASP5	CASP5	chr11	-	105007082	105007334	105008806	105008980	105003273	105003383	105023129	105023168	1	0	0	10	0	0	0	0	0	2	98	98	1	1	0.091	NA	NA	NA		0	0.091
836	CASP5	CASP5	chr11	-	105007082	105007334	105008806	105009019	105003273	105003383	105023129	105023168	1	0	0	10	0	0	0	0	0	2	98	98	1	1	0.091	NA	NA	NA		0	0.091
846	DENND5A	DENND5A	chr11	-	9142721	9142845	9143402	9143485	9141939	9142108	9144096	9144278	36	15	7	65	20	10	0	7	0	11	98	98	1	1	0.356	0.429	0.412	NA		0.389	0.01
848	WDR37	WDR37	chr10	+	1072115	1072293	1077906	1078003	1056835	1056968	1080010	1080106	2	4	2	9	2	6	0	0	0	3	98	98	1	1	0.182	0.667	0.25	NA		0	0.366
849	CLTA	CLTA	chr9	+	36197550	36197588	36198978	36199096	36190855	36191273	36204067	36204179	11	0	2	3	1	4	0	0	0	4	87	98	1	1	0.805	0	0.36	NA		0	0.388
852	FBXO18	FBXO18	chr10	+	5918341	5918478	5921257	5921357	5917589	5917676	5921447	5921569	4	4	1	6	5	0	0	1	0	0	98	98	1	1	0.4	0.444	1	NA		1	-0.385
853	CELF2	CELF2	chr10	+	11266597	11266677	11270665	11270824	11257737	11257872	11275056	11275120	20	6	1	104	45	14	0	9	0	11	98	98	1	1	0.161	0.118	0.067	NA		0.45	-0.335
860	DIS3	DIS3	chr13	-	72780845	72781003	72781288	72781356	72778186	72778380	72781604	72782128	1	2	1	2	2	0	0	0	0	1	98	98	1	1	0.333	0.5	1	NA		0	0.611
861	BPTF	BPTF	chr17	+	67875555	67875744	67886127	67886316	67874816	67875020	67891843	67892034	6	1	0	10	0	0	0	1	0	3	98	98	1	1	0.375	1	NA	NA		0.25	0.438
868	OGDH	OGDH	chr7	+	44647443	44647534	44647656	44647759	44645326	44645518	44666735	44666851	1	5	0	11	7	4	0	2	0	0	98	98	1	1	0.083	0.417	0	NA		1	-0.833
870	MBNL1	MBNL1	chr3	+	152455541	152455577	152456266	152456361	152447619	152447773	152459270	152459345	17	1	8	35	7	9	0	10	0	5	85	98	1	1	0.359	0.141	0.506	NA		0.698	-0.363
872	PIK3CD	PIK3CD	chr1	+	9723968	9724092	9724275	9724421	9723124	9723292	9724803	9724936	34	17	14	43	22	17	0	3	0	6	98	98	1	1	0.442	0.436	0.452	NA		0.333	0.11
875	FBXO28	FBXO28	chr1	+	224134073	224134212	224153141	224153337	224130471	224130581	224157351	224162047	13	3	5	1	1	0	0	3	0	0	98	98	1	1	0.929	0.75	1	NA		1	-0.107
878	TXNRD1	TXNRD1	chr12	+	104288930	104289040	104311289	104311412	104286681	104287109	104313244	104313317	0	1	0	27	20	2	0	0	0	2	98	98	1	1	0	0.048	0	NA		0	0.016
879	LDLR	LDLR	chr19	+	11102663	11102786	11105219	11105600	11100222	11100345	11106564	11106687	2	0	0	1	0	0	0	2	0	0	98	98	1	1	0.667	NA	NA	NA		1	-0.333
882	ZNF33A	ZNF33A	chr10	+	38025347	38025411	38039412	38039492	38017290	38017386	38054374	38060067	1	0	1	0	0	0	0	0	0	2	98	98	1	1	1	NA	1	NA		0	1
883	ZNF33A	ZNF33A	chr10	+	38034277	38034400	38039412	38039492	38017290	38017386	38054374	38060067	0	0	1	0	0	0	0	0	0	2	98	98	1	1	NA	NA	1	NA		0	1
884	ZNF33A	ZNF33A	chr10	+	38034325	38034400	38039412	38039492	38017290	38017386	38054374	38060067	0	1	1	0	0	0	0	0	0	2	98	98	1	1	NA	1	1	NA		0	1
890	CAPRIN2	CAPRIN2	chr12	-	30719078	30719225	30720810	30720915	30716507	30716676	30723258	30723314	1	0	0	0	3	0	0	1	0	0	98	98	1	1	1	0	NA	NA		1	-0.5
891	XRN1	XRN1	chr3	-	142412543	142412663	142414134	142414291	142404906	142405076	142417139	142417229	21	11	2	16	14	1	0	4	0	7	98	98	1	1	0.568	0.44	0.667	NA		0.364	0.194
892	ELP2	ELP2	chr18	+	36138794	36138872	36139418	36139613	36138269	36138426	36141136	36141201	7	2	0	0	5	0	0	4	0	0	98	98	1	1	1	0.286	NA	NA		1	-0.357
905	MCTP1	MCTP1	chr5	-	94917895	94917973	94931952	94931991	94912805	94912976	94940083	94940195	25	9	6	8	15	2	0	6	0	3	88	98	1	1	0.777	0.401	0.77	NA		0.69	-0.041
912	RELT	RELT	chr11	+	73391143	73391223	73392210	73392468	73390754	73390921	73393836	73393917	9	6	8	23	10	6	0	3	0	3	98	98	1	1	0.281	0.375	0.571	NA		0.5	-0.091
926	RCBTB2	RCBTB2	chr13	-	48521897	48521962	48522307	48522403	48515585	48515741	48524658	48524757	2	1	0	6	3	0	0	0	0	2	98	98	1	1	0.25	0.25	NA	NA		0	0.25
927	BAZ2B	BAZ2B	chr2	-	159448241	159448403	159453612	159453801	159446781	159446975	159478574	159478721	36	15	2	37	22	5	0	6	0	9	98	98	1	1	0.493	0.405	0.286	NA		0.4	-0.005
928	BAZ2B	BAZ2B	chr2	-	159448241	159448409	159453612	159453801	159446781	159446975	159478574	159478721	36	15	2	43	28	5	0	6	0	14	98	98	1	1	0.456	0.349	0.286	NA		0.3	0.064
929	MLKL	MLKL	chr16	-	74678898	74678980	74682650	74682786	74675612	74675764	74685485	74685583	17	2	0	6	1	8	0	3	0	0	98	98	1	1	0.739	0.667	0	NA		1	-0.531
934	GBF1	GBF1	chr10	+	102368217	102368454	102368738	102368832	102367477	102367560	102369210	102369387	7	1	0	10	3	1	0	2	0	4	98	98	1	1	0.412	0.25	0	NA		0.333	-0.112
939	NT5C2	NT5C2	chr10	-	103106588	103106706	103139405	103139479	103105705	103105801	103174857	103174982	36	30	8	43	15	11	0	7	0	7	98	98	1	1	0.456	0.667	0.421	NA		0.5	0.015
940	USMG5	USMG5	chr10	-	103394198	103394394	103395745	103396032	103392370	103392466	103396408	103396513	1	0	0	0	0	0	0	0	0	1	98	98	1	1	1	NA	NA	NA		0	1
946	CAMK2G	CAMK2G	chr10	-	73847224	73847347	73847987	73848082	73842457	73842541	73848525	73848609	13	8	5	15	1	2	0	0	0	2	98	98	1	1	0.464	0.889	0.714	NA		0	0.689
947	CAMK2G	CAMK2G	chr10	-	73847987	73848082	73848525	73848609	73847224	73847347	73849012	73849115	23	16	17	13	15	1	0	2	0	3	98	98	1	1	0.639	0.516	0.944	NA		0.4	0.3
952	ATE1	ATE1	chr10	-	121898840	121898969	121899865	121899994	121870005	121870038	121902390	121902620	4	0	0	3	0	0	0	1	0	0	98	98	1	1	0.571	NA	NA	NA		1	-0.429
953	ATE1	ATE1	chr10	-	121913789	121913893	121922348	121922411	121910905	121911151	121924265	121924329	2	0	0	15	6	0	0	1	0	0	98	98	1	1	0.118	0	NA	NA		1	-0.941
954	DNM2	DNM2	chr19	+	10796060	10796199	10797379	10797518	10795371	10795439	10798485	10798572	12	5	1	10	6	8	0	2	0	4	98	98	1	1	0.545	0.455	0.111	NA		0.333	0.037
955	FAM134C	FAM134C	chr17	-	42586764	42586891	42587833	42587864	42586052	42586137	42592055	42592162	11	2	0	3	0	2	0	1	0	0	80	98	1	1	0.818	1	0	NA		1	-0.394
958	GSR	GSR	chr8	-	30689160	30689319	30692968	30693055	30684087	30684199	30696379	30696479	11	1	1	6	5	7	0	2	0	3	98	98	1	1	0.647	0.167	0.125	NA		0.4	-0.087
974	LST1	LST1	chr6	+	31587640	31587733	31587943	31587966	31587199	31587318	31588517	31588909	42	28	26	17	17	14	0	0	0	7	98	72	1	1	0.645	0.548	0.577	NA		0	0.59
979	MAPK14	MAPK14	chr6	+	36095986	36096066	36100187	36100267	36076536	36076608	36102570	36102649	31	21	14	18	11	4	0	8	0	1	98	98	1	1	0.633	0.656	0.778	NA		0.889	-0.2
985	DYNC1LI2	DYNC1LI2	chr16	-	66742437	66742668	66749196	66749313	66736074	66736244	66751272	66751346	19	2	0	2	5	1	0	2	0	1	98	98	1	1	0.905	0.286	0	NA		0.667	-0.27
997	GZMH	GZMH	chr14	-	24607340	24607406	24607611	24607747	24606479	24606746	24608264	24608412	8	9	0	0	0	0	1	0	0	3	98	98	1	1	1	1	NA		1	0	0.5
1000	SDHD	SDHD	chr11	+	112087856	112087973	112088866	112089011	112086823	112086959	112094804	112095801	1	1	0	2	0	5	0	1	0	0	98	98	1	1	0.333	1	0	NA		1	-0.556
1003	PRR5L	PRR5L	chr11	+	36446299	36446440	36451208	36451335	36437384	36437476	36462341	36465204	4	5	1	1	0	0	0	1	0	0	98	98	1	1	0.8	1	1	NA		1	-0.067
1010	ATG13	ATG13	chr11	+	46618324	46618375	46630044	46630100	46617275	46617890	46644278	46644360	0	0	0	6	2	1	0	2	0	2	98	98	1	1	0	0	0	NA		0.5	-0.5
1012	TNFSF13	TNFSF13	chr17	+	7559623	7559702	7559845	7559893	7558291	7559297	7560048	7560167	6	3	0	12	3	1	0	4	0	8	98	97	1	1	0.331	0.497	0	NA		0.331	-0.055
1013	TNFSF13	TNFSF13	chr17	+	7559623	7559702	7559847	7559893	7558291	7559297	7560048	7560167	6	3	0	11	3	1	0	4	0	8	98	95	1	1	0.346	0.492	0	NA		0.326	-0.047
1014	TNFSF13	TNFSF13	chr17	+	7559623	7559702	7559850	7559893	7558291	7559297	7560048	7560167	6	3	0	13	2	1	0	4	0	9	98	92	1	1	0.302	0.585	0	NA		0.294	0.002
1016	PHF21A	PHF21A	chr11	-	45938156	45938312	45945839	45946003	45936493	45936569	45948885	45948946	8	3	0	21	2	0	0	6	0	5	98	98	1	1	0.276	0.6	NA	NA		0.545	-0.107
1025	SRP54	SRP54	chr14	+	34999557	34999649	35000935	35001020	34996676	34996787	35007282	35007387	6	2	0	10	3	0	2	0	0	2	98	98	1	1	0.375	0.4	NA		1	0	-0.112
1027	ZEB1	ZEB1	chr10	+	31461036	31461237	31495778	31495838	31319171	31319292	31502347	31502509	2	0	0	8	2	0	0	0	0	2	98	98	1	1	0.2	0	NA	NA		0	0.1
1029	ADGRE2	ADGRE2	chr19	-	14754953	14755127	14755653	14755877	14752328	14752526	14756237	14756345	6	6	12	20	5	0	0	2	0	3	98	98	1	1	0.231	0.545	1	NA		0.4	0.192
1034	FRMD8	FRMD8	chr11	+	65389360	65389528	65393572	65393674	65387036	65387121	65394040	65394099	4	3	0	4	3	6	0	0	0	1	98	98	1	1	0.5	0.5	0	NA		0	0.333
1038	TMEM135	TMEM135	chr11	+	87091361	87091395	87157340	87157406	87071522	87071615	87236637	87236684	1	0	0	0	0	0	0	0	1	0	83	98	1	1	1	NA	NA		0	NA	1
1039	CLN3	CLN3	chr16	-	28487661	28487741	28488590	28488662	28487455	28487541	28489289	28489386	4	2	0	8	6	2	0	4	0	1	98	98	1	1	0.333	0.25	0	NA		0.8	-0.606
1049	CYB5R4	CYB5R4	chr6	+	83914415	83914448	83918004	83918065	83909008	83909090	83919396	83919454	65	27	25	68	29	20	0	10	0	6	82	98	1	1	0.533	0.527	0.599	NA		0.666	-0.113
1052	C11orf54	C11orf54	chr11	+	93755209	93755386	93757315	93757465	93753935	93754037	93759741	93759858	7	1	0	11	3	0	0	5	0	0	98	98	1	1	0.389	0.25	NA	NA		1	-0.68
1053	CD300LF	CD300LF	chr17	-	74696194	74696217	74698368	74698481	74695724	74695859	74703034	74703098	15	7	10	1	1	2	0	0	0	1	98	72	1	1	0.917	0.837	0.786	NA		0	0.847
1059	MBD1	MBD1	chr18	-	50274185	50274353	50274976	50275045	50273563	50273863	50275128	50275245	7	0	1	0	4	0	0	1	0	0	98	98	1	1	1	0	1	NA		1	-0.333
1060	NDEL1	NDEL1	chr17	+	8445710	8445864	8446753	8446902	8444259	8444357	8448549	8448686	28	12	1	69	38	14	0	8	0	8	98	98	1	1	0.289	0.24	0.067	NA		0.5	-0.301
1062	PAM	PAM	chr5	+	103025130	103025334	103028184	103028238	103019789	103019843	103028886	103031104	2	3	0	2	2	0	0	1	0	0	98	98	1	1	0.5	0.6	NA	NA		1	-0.45
1064	AMPD3	AMPD3	chr11	+	10478525	10478730	10482062	10482225	10461514	10461740	10484819	10485039	3	0	0	18	5	0	0	0	0	6	98	98	1	1	0.143	0	NA	NA		0	0.071
1065	LRRC75A-AS1	LRRC75A-AS1	chr17	+	16441073	16441130	16460892	16461245	16440184	16440253	16462832	16462941	1	0	3	0	0	0	0	0	0	1	98	98	1	1	1	NA	1	NA		0	1
1066	TOM1L2	TOM1L2	chr17	-	17882704	17882863	17884633	17884768	17879626	17879743	17893660	17893810	4	0	1	4	1	1	0	3	0	0	98	98	1	1	0.5	0	0.5	NA		1	-0.667
1067	TOM1L2	TOM1L2	chr17	-	17884633	17884768	17893660	17893810	17882704	17882863	17898595	17898674	4	0	0	0	0	0	0	1	1	0	98	98	1	1	1	NA	NA		0	1	0.5
1068	GYS1	GYS1	chr19	-	48987193	48987385	48991301	48991483	48985849	48986035	48992994	48993353	0	1	0	2	0	3	0	0	3	2	98	98	1	1	0	1	0		0	0	0.333
1069	DHX8	DHX8	chr17	+	43492680	43493040	43493444	43493589	43492182	43492292	43493682	43493886	21	9	0	15	11	4	0	2	0	2	98	98	1	1	0.583	0.45	0	NA		0.5	-0.156
1070	KIAA0100	KIAA0100	chr17	-	28641888	28642078	28642274	28642335	28640559	28640683	28642868	28642987	9	7	0	6	3	0	0	3	0	2	98	98	1	1	0.6	0.7	NA	NA		0.6	0.05
1077	ANP32E	ANP32E	chr1	-	150229071	150229237	150230570	150230693	150226607	150226795	150231776	150231926	3	3	2	2	0	1	0	1	0	0	98	98	1	1	0.6	1	0.667	NA		1	-0.244
1085	RFFL	RFFL	chr17	-	35017522	35017606	35021370	35021781	35016369	35016580	35026373	35026561	9	1	0	15	4	5	0	3	0	1	98	98	1	1	0.375	0.2	0	NA		0.75	-0.558
1101	ZDHHC3	ZDHHC3	chr3	-	44929305	44929436	44932906	44932990	44915260	44926847	44933117	44933199	1	1	1	15	3	2	0	2	0	3	98	98	1	1	0.062	0.25	0.333	NA		0.4	-0.185
1120	DHX40	DHX40	chr17	+	59566626	59566709	59570517	59570663	59565524	59565783	59573115	59573235	7	3	0	13	6	4	0	1	0	1	98	98	1	1	0.35	0.333	0	NA		0.5	-0.272
1121	DHX40	DHX40	chr17	+	59566626	59566794	59570517	59570663	59565524	59565783	59573115	59573235	8	3	0	13	6	4	0	1	0	1	98	98	1	1	0.381	0.333	0	NA		0.5	-0.262
1128	MILR1	MILR1	chr17	+	64452596	64452866	64457399	64457684	64449313	64449355	64460821	64460932	4	3	0	7	0	0	0	1	0	0	98	98	1	1	0.364	1	NA	NA		1	-0.318
1129	MILR1	MILR1	chr17	+	64457399	64457684	64460821	64460932	64452596	64452866	64465451	64465541	6	2	0	6	0	0	0	6	0	0	98	98	1	1	0.5	1	NA	NA		1	-0.25
1141	EXOC7	EXOC7	chr17	-	76090328	76090397	76091142	76091235	76089174	76089320	76094413	76094581	1	0	0	1	0	0	0	0	0	2	98	98	1	1	0.5	NA	NA	NA		0	0.5
1144	PGS1	PGS1	chr17	+	78392475	78392665	78396307	78396385	78378617	78378808	78398251	78398351	12	14	1	11	19	1	0	4	0	4	98	98	1	1	0.522	0.424	0.5	NA		0.5	-0.018
1145	PGS1	PGS1	chr17	+	78392513	78392665	78396307	78396385	78378617	78378808	78398251	78398351	4	5	1	11	19	1	0	1	0	4	98	98	1	1	0.267	0.208	0.5	NA		0.2	0.125
1146	DCP2	DCP2	chr5	+	113003941	113004077	113007937	113008042	113001566	113001674	113010755	113010807	12	8	7	26	7	2	0	5	0	1	98	98	1	1	0.316	0.533	0.778	NA		0.833	-0.291
1147	ATG7	ATG7	chr3	+	11379971	11380052	11426803	11426926	11364658	11364734	11554810	11557665	16	8	2	3	5	0	0	6	0	2	98	98	1	1	0.842	0.615	1	NA		0.75	0.069
1154	SMCHD1	SMCHD1	chr18	+	2760651	2760739	2762104	2762236	2752487	2752552	2763636	2763789	110	56	32	120	94	33	0	22	0	30	98	98	1	1	0.478	0.373	0.492	NA		0.423	0.025
1158	CARS	CARS	chr11	-	3026675	3026797	3028995	3029084	3020219	3020332	3029302	3029443	27	10	3	12	8	3	0	6	0	3	98	98	1	1	0.692	0.556	0.5	NA		0.667	-0.084
1162	PPCDC	PPCDC	chr15	+	75043440	75043536	75044385	75044514	75028246	75028453	75048552	75048721	0	2	0	16	0	4	0	0	0	2	98	98	1	1	0	1	0	NA		0	0.333
1166	CLEC4A	CLEC4A	chr12	+	8125560	8125677	8129263	8129362	8123631	8123960	8135584	8135736	3	4	1	8	1	0	0	0	0	2	98	98	1	1	0.273	0.8	1	NA		0	0.691
1167	CLEC4A	CLEC4A	chr12	+	8129263	8129362	8135584	8135736	8123631	8123960	8136787	8136903	0	0	9	4	1	3	0	1	0	3	98	98	1	1	0	0	0.75	NA		0.25	0
1168	CLEC4A	CLEC4A	chr12	+	8129263	8129362	8135584	8135736	8125560	8125677	8136787	8136903	5	2	22	4	1	3	0	2	0	3	98	98	1	1	0.556	0.667	0.88	NA		0.4	0.301
1173	KIAA0226L	KIAA0226L	chr13	-	46361440	46361573	46362537	46362598	46359485	46359631	46363114	46363213	42	11	10	64	9	9	0	3	0	6	98	98	1	1	0.396	0.55	0.526	NA		0.333	0.158
1190	EDEM2	EDEM2	chr20	-	35144978	35145018	35146824	35146935	35142372	35142478	35147151	35147358	5	6	0	0	5	5	0	1	0	0	98	89	1	1	1	0.521	0	NA		1	-0.493
1200	GMFG	GMFG	chr19	-	39328999	39329073	39335260	39335310	39328356	39328548	39335434	39335531	60	41	35	85	105	133	0	7	0	24	98	98	1	1	0.414	0.281	0.208	NA		0.226	0.075
1202	GMFG	GMFG	chr19	-	39333076	39333126	39335260	39335310	39329543	39329626	39335434	39335531	60	42	35	107	88	99	0	6	0	20	98	98	1	1	0.359	0.323	0.261	NA		0.231	0.083
1203	GMFG	GMFG	chr19	-	39333076	39333126	39335260	39335310	39329543	39329626	39335973	39336086	0	1	0	104	88	99	0	0	0	19	98	98	1	1	0	0.011	0	NA		0	0.004
1204	GMFG	GMFG	chr19	-	39335260	39335310	39335434	39335531	39333076	39333126	39335973	39336086	5	0	0	97	63	46	0	1	1	12	98	98	1	1	0.049	0	0		0	0.077	-0.022
1211	CNOT8	CNOT8	chr5	+	154865191	154865385	154870660	154870822	154863206	154863395	154871729	154871874	6	3	4	9	2	2	0	0	0	3	98	98	1	1	0.4	0.6	0.667	NA		0	0.556
1212	CNOT8	CNOT8	chr5	+	154867691	154867792	154870660	154870822	154865191	154865385	154871729	154871874	0	0	1	13	4	7	0	0	0	3	98	98	1	1	0	0	0.125	NA		0	0.042
1221	GPATCH2L	GPATCH2L	chr14	+	76177891	76177931	76177987	76178042	76176622	76176690	76180763	76180849	2	0	0	9	13	0	0	0	0	2	89	98	1	1	0.197	0	NA	NA		0	0.099
1225	ALG13	ALG13	chrX	+	111682131	111682235	111682235	111682294	111681169	111681424	111684964	111685103	1	0	0	14	0	2	0	0	3	0	98	98	1	1	0.067	NA	0		0	NA	0.034
1227	RBM23	RBM23	chr14	-	22908332	22908380	22911327	22911403	22906194	22906368	22918998	22919187	7	13	3	5	8	0	0	1	0	1	98	97	1	1	0.581	0.617	1	NA		0.497	0.236
1233	LILRA5	LILRA5	chr19	-	54312334	54312370	54312536	54312621	54311863	54312148	54313016	54313139	21	14	12	25	14	23	0	6	0	5	98	85	1	1	0.421	0.464	0.312	NA		0.51	-0.111
1238	C2	C2	chr6	+	31933866	31933965	31935922	31936061	31933609	31933783	31937318	31937459	0	0	0	1	0	0	0	2	0	0	98	98	1	1	0	NA	NA	NA		1	-1
1241	VPS33B	VPS33B	chr15	-	91016962	91017024	91017804	91017885	91014383	91014433	91022153	91022621	2	0	0	1	0	0	0	2	0	1	98	98	1	1	0.667	NA	NA	NA		0.667	0
1243	FAM129A	FAM129A	chr1	-	184803584	184803692	184805945	184806056	184798078	184798190	184808073	184808235	59	34	14	104	42	32	0	4	0	17	98	98	1	1	0.362	0.447	0.304	NA		0.19	0.181
1246	CAMLG	CAMLG	chr5	+	134741062	134741523	134743986	134744052	134738479	134738792	134750758	134752160	4	0	4	4	4	1	0	0	0	1	98	98	1	1	0.5	0	0.8	NA		0	0.433
1250	PQLC3	PQLC3	chr2	+	11171924	11172045	11174967	11175009	11164203	11164261	11177736	11178872	2	0	3	0	1	0	0	1	0	1	98	91	1	1	1	0	1	NA		0.481	0.186
1251	NCOA1	NCOA1	chr2	+	24762686	24762776	24768078	24768135	24757972	24758156	24768220	24770701	37	9	5	18	5	0	0	5	0	5	98	98	1	1	0.673	0.643	1	NA		0.5	0.272
1253	BRE	BRE	chr2	+	28310081	28310153	28327273	28327447	28298337	28298491	28338449	28338900	1	0	0	1	0	0	0	0	0	1	98	98	1	1	0.5	NA	NA	NA		0	0.5
1259	PPM1B	PPM1B	chr2	+	44201185	44202045	44209209	44209327	44168802	44169274	44217966	44218078	4	7	3	19	7	7	0	2	0	3	98	98	1	1	0.174	0.5	0.3	NA		0.4	-0.075
1260	PREPL	PREPL	chr2	-	44332458	44332656	44338350	44338536	44328936	44329112	44339146	44339363	2	0	1	1	0	0	0	1	0	0	98	98	1	1	0.667	NA	1	NA		1	-0.166
1261	EIF4E3	EIF4E3	chr3	-	71696459	71696520	71699613	71699708	71693874	71693941	71710411	71710484	11	12	8	29	15	15	0	2	0	3	98	98	1	1	0.275	0.444	0.348	NA		0.4	-0.044
1266	PSMA3-AS1	PSMA3-AS1	chr14	-	58273978	58274010	58285590	58285756	58265364	58267393	58291634	58291707	5	3	1	3	6	1	0	1	0	2	98	81	1	1	0.579	0.292	0.453	NA		0.292	0.149
1267	PSMA3-AS1	PSMA3-AS1	chr14	-	58273978	58274135	58285590	58285756	58265364	58267393	58291634	58291707	5	3	1	10	5	4	0	1	0	0	98	98	1	1	0.333	0.375	0.2	NA		1	-0.697
1268	VPS13B	VPS13B	chr8	+	99507136	99507203	99507769	99507911	99502835	99502950	99511103	99511512	38	11	0	4	1	0	0	5	0	2	98	98	1	1	0.905	0.917	NA	NA		0.714	0.197
1271	PTCD3	PTCD3	chr2	+	86125454	86125515	86125794	86125880	86124994	86125082	86127160	86127305	11	3	0	3	0	0	0	2	0	0	98	98	1	1	0.786	1	NA	NA		1	-0.107
1277	BCL2L11	BCL2L11	chr2	+	111123732	111123869	111150043	111150147	111120913	111121188	111164132	111168445	3	0	0	3	2	0	0	1	0	1	98	98	1	1	0.5	0	NA	NA		0.5	-0.25

1278	BCL2L11	BCL2L11	chr2	+	111123732	111124139	111150043	111150147	111120913	111121188	111153779	111153904	3	0	0	5	0	0	0	1	0	1	98	98	1	1	0.375	NA	NA	NA	0.5	-0.125
1279	BCL2L11	BCL2L11	chr2	+	111123732	111124139	111150043	111150147	111120913	111121188	111164132	111168445	3	0	0	3	2	0	0	1	0	1	98	98	1	1	0.5	0	NA	NA	0.5	-0.25
1289	SMPD4	SMPD4	chr2	-	130173278	130173354	130173513	130173656	130172786	130172895	130174913	130175000	0	0	0	0	5	0	0	2	0	0	98	98	1	1	NA	0	NA	NA	1	-1
1295	PDK1	PDK1	chr2	+	172562740	172562800	172564502	172564687	172562219	172562291	172564977	172565073	3	3	0	8	0	2	1	0	0	0	98	98	1	1	0.273	1	0	1	NA	-0.576
1298	SETX	SETX	chr9	-	132334607	132334727	132336295	132336515	132331276	132331448	132342689	132342799	116	29	8	80	20	2	0	9	0	8	98	98	1	1	0.592	0.592	0.8	NA	0.529	0.132
1303	ANKRD44	ANKRD44	chr2	-	196998906	196999052	197000418	197000502	196998336	196998419	197001752	197001840	47	11	6	25	7	13	0	9	0	5	98	98	1	1	0.653	0.611	0.316	NA	0.643	-0.116
1304	PPIL3	PPIL3	chr2	-	200882341	200882435	200885261	200885367	200881420	200881488	200885697	200885772	0	0	0	1	2	0	0	1	0	0	98	98	1	1	0	0	NA	NA	1	-1
1305	PIKFYVE	PIKFYVE	chr2	+	208276711	208276830	208285725	208285933	208273583	208273733	208288728	208288818	10	1	0	12	9	0	0	0	0	1	98	98	1	1	0.455	0.1	NA	NA	0	0.278
1306	PIKFYVE	PIKFYVE	chr2	+	208277536	208277708	208285725	208285933	208276711	208276830	208288728	208288818	8	2	3	13	9	0	0	0	0	1	98	98	1	1	0.381	0.182	1	NA	0	0.521
1307	BARD1	BARD1	chr2	-	214780559	214781509	214792296	214792445	214769231	214769312	214797060	214797117	1	0	0	0	0	0	0	0	0	1	98	98	1	1	1	NA	NA	NA	0	1
1317	DGKD	DGKD	chr2	+	233434768	233434901	233435817	233435924	233434379	233434484	233436315	233436441	11	3	0	3	7	4	0	5	0	3	98	98	1	1	0.786	0.3	0	NA	0.625	-0.263
1319	STK25	STK25	chr2	-	241501477	241501708	241508005	241508135	241500739	241500796	241508442	241508695	0	0	0	1	1	0	0	1	0	1	98	98	1	1	0	0	NA	NA	0.5	-0.5
1324	NAPB	NAPB	chr20	-	23395138	23395185	23397071	23397182	23394921	23394999	23402992	23403072	1	0	0	0	0	0	0	0	0	2	98	96	1	1	1	NA	NA	NA	0	1
1325	NAPB	NAPB	chr20	-	23395138	23395185	23397071	23397188	23394921	23394999	23402992	23403072	1	0	0	0	0	0	0	0	0	2	98	96	1	1	1	NA	NA	NA	0	1
1329	HCK	HCK	chr20	+	32054215	32054281	32069687	32069751	32052187	32052486	32071661	32071782	19	4	8	2	0	0	0	5	0	0	98	98	1	1	0.905	1	1	NA	1	-0.032
1332	C20orf24	C20orf24	chr20	+	36607889	36608000	36609600	36609730	36607714	36607818	36612009	36612557	3	3	1	29	41	54	0	1	0	8	98	98	1	1	0.094	0.068	0.018	NA	0.111	-0.051
1333	C20orf24	C20orf24	chr20	+	36607889	36608000	36609600	36609758	36607714	36607818	36612009	36612557	3	3	1	9	11	26	0	1	0	4	98	98	1	1	0.25	0.214	0.037	NA	0.2	-0.033
1345	ZMYND8	ZMYND8	chr20	-	47262287	47262428	47276313	47276795	47249286	47249439	47282101	47282217	5	7	1	1	3	0	0	1	0	1	98	98	1	1	0.833	0.7	1	NA	0.5	0.344
1365	PRMT2	PRMT2	chr21	+	46658744	46658920	46663382	46663554	46649574	46649739	46664294	46665124	9	2	5	4	9	2	0	2	0	1	98	98	1	1	0.692	0.182	0.714	NA	0.667	-0.138
1366	BID	BID	chr22	-	17743802	17744013	17750104	17750174	17739348	17739488	17774380	17774495	7	0	0	11	15	12	0	0	0	9	98	98	1	1	0.389	0	0	NA	0	0.13
1367	TANGO2	TANGO2	chr22	+	20053436	20053551	20055942	20056013	20052464	20052584	20063337	20063442	1	6	7	2	3	1	0	0	0	1	98	98	1	1	0.333	0.667	0.875	NA	0	0.625
1368	TANGO2	TANGO2	chr22	+	20053436	20053551	20061529	20061683	20052464	20052584	20063337	20063442	1	6	7	0	3	2	0	0	0	2	98	98	1	1	1	0.667	0.778	NA	0	0.815
1369	TANGO2	TANGO2	chr22	+	20055942	20056013	20061529	20061683	20052464	20052584	20063337	20063442	2	3	1	0	3	2	0	1	0	2	98	98	1	1	1	0.5	0.333	NA	0.333	0.278
1370	TANGO2	TANGO2	chr22	+	20055942	20056013	20063337	20063442	20053436	20053551	20064541	20065926	4	7	1	5	9	5	0	4	0	3	98	98	1	1	0.444	0.437	0.167	NA	0.571	-0.222
1372	GUCD1	GUCD1	chr22	-	24547907	24548073	24548916	24549001	24546913	24547005	24554948	24555307	5	4	0	13	7	0	2	0	0	1	98	98	1	1	0.278	0.364	NA	1	0	-0.179
1373	GUCD1	GUCD1	chr22	-	24547907	24548073	24548916	24549001	24546913	24547005	24555614	24555935	2	1	0	11	7	0	0	0	0	1	98	98	1	1	0.154	0.125	NA	NA	0	0.14
1377	UBAP1	UBAP1	chr9	+	34220907	34220948	34234215	34234340	34179004	34179240	34241184	34242108	7	3	4	4	0	4	0	1	0	0	90	98	1	1	0.656	1	0.521	NA	1	-0.274
1382	SCAF8	SCAF8	chr6	+	154822275	154822409	154824233	154824378	154820176	154820333	154827171	154827240	16	4	8	13	0	0	0	1	0	0	98	98	1	1	0.552	1	1	NA	1	-0.149
1384	NUP50	NUP50	chr22	+	45175893	45176080	45178237	45178900	45171599	45171683	45181285	45181367	19	3	3	12	12	3	0	0	0	1	98	98	1	1	0.613	0.2	0.5	NA	0	0.438
1386	TBC1D22A	TBC1D22A	chr22	+	46913333	46913446	46974289	46974399	46912073	46912188	46997633	46997709	0	0	2	16	5	10	0	0	0	3	98	98	1	1	0	0	0.167	NA	0	0.056
1387	TBC1D22A	TBC1D22A	chr22	+	46974289	46974399	46997633	46997709	46912073	46912188	47037070	47037198	11	2	0	13	8	4	0	3	0	3	98	98	1	1	0.458	0.2	0	NA	0.5	-0.281
1391	ZNF346	ZNF346	chr5	+	177041777	177041870	177044391	177044533	177041125	177041229	177050750	177050936	1	1	0	1	0	0	0	1	0	0	98	98	1	1	0.5	1	NA	NA	1	-0.25
1398	HACL1	HACL1	chr3	-	15585247	15585342	15586524	15586602	15582876	15582989	15589539	15589612	1	0	1	0	0	0	0	0	0	1	98	98	1	1	1	NA	1	NA	0	1
1404	ABHD5	ABHD5	chr3	+	43714946	43715058	43717670	43717857	43711708	43711863	43718442	43722725	61	17	23	40	7	7	0	13	0	9	98	98	1	1	0.604	0.708	0.767	NA	0.591	0.102
1407	HIPK3	HIPK3	chr11	+	33348521	33348818	33349146	33349287	33348165	33348228	33351607	33351843	14	6	2	32	17	4	0	1	0	6	98	98	1	1	0.304	0.261	0.333	NA	0.143	0.156
1425	ERBB2IP	ERBB2IP	chr5	+	66068876	66069020	66072168	66072291	66053405	66054951	66075023	66075230	2	0	0	6	9	0	0	0	0	2	98	98	1	1	0.25	0	NA	NA	0	0.125
1427	SENP7	SENP7	chr3	-	101398860	101399055	101417592	101417790	101372007	101372126	101458954	101459052	11	8	2	13	4	0	0	0	0	1	98	98	1	1	0.458	0.667	1	NA	0	0.708
1432	MRVI1	MRVI1	chr11	-	10594145	10594195	10600917	10601059	10593491	10593599	10603119	10603251	27	9	9	10	2	4	0	4	0	1	98	98	1	1	0.73	0.818	0.692	NA	0.8	-0.053
1437	CEP63	CEP63	chr3	+	134558141	134558347	134559149	134559429	134551925	134552012	134561376	134565028	11	12	1	5	2	3	2	0	0	0	98	98	1	1	0.687	0.857	0.25	1	NA	-0.402
1438	ARMC8	ARMC8	chr3	+	138188400	138188530	138209816	138209893	138187247	138187599	138221925	138221997	0	3	0	10	2	2	0	0	0	2	98	98	1	1	0	0.6	0	NA	0	0.2
1439	ARMC8	ARMC8	chr3	+	138188454	138188530	138209816	138209893	138187247	138187599	138221925	138221997	0	3	0	10	2	2	0	2	0	2	98	98	1	1	0	0.6	0	NA	0.5	-0.3
1442	7-Mar	7-Mar	chr2	+	159762879	159762993	159764625	159764674	159759225	159759335	159767283	159768583	16	10	0	26	16	15	0	2	0	5	98	98	1	1	0.381	0.385	0	NA	0.286	-0.031
1443	EIF2A	EIF2A	chr3	+	150571957	150572529	150575648	150575762	150568175	150568292	150581617	150581746	6	0	2	6	1	0	0	0	0	2	98	98	1	1	0.5	0	1	NA	0	0.5
1445	MME	MME	chr3	+	155141990	155142127	155142236	155142330	155140190	155140292	155143442	155143571	67	18	23	95	36	23	0	9	0	7	98	98	1	1	0.414	0.333	0.5	NA	0.562	-0.146
1447	ZMYM5	ZMYM5	chr13	-	19838699	19838985	19851354	19851448	19837452	19837821	19851688	19852190	6	1	0	2	2	0	0	0	0	1	98	98	1	1	0.75	0.333	NA	NA	0	0.541
1450	XRN2	XRN2	chr20	+	21356585	21356665	21357735	21357792	21356079	21356177	21365420	21365489	8	4	4	22	4	1	0	8	0	6	98	98	1	1	0.267	0.5	0.8	NA	0.571	-0.049
1457	IL1RAP	IL1RAP	chr3	+	190550341	190550505	190556129	190556216	190514050	190514219	190564288	190564353	0	1	0	22	20	1	0	0	3	7	98	98	1	1	0	0.048	0	0	0	0.016
1460	DUSP22	DUSP22	chr6	+	311879	311962	335113	335163	304627	304661	345853	345928	5	4	3	7	7	0	0	1	0	1	98	98	1	1	0.417	0.364	1	NA	0.5	0.094
1463	NIPBL	NIPBL	chr5	+	37057185	37057332	37060843	37061018	37052365	37052566	37063789	37063978	16	6	0	4	2	1	0	0	0	1	98	98	1	1	0.8	0.75	0	NA	0	0.517
1465	BST1	BST1	chr4	+	15715284	15715361	15715706	15715799	15711806	15711889	15718906	15718993	30	11	11	37	19	16	0	15	0	3	98	98	1	1	0.448	0.367	0.407	NA	0.833	-0.426
1468	PGM2	PGM2	chr4	+	37848521	37848651	37850183	37850373	37847201	37847295	37855607	37855741	7	18	3	23	10	2	0	0	0	3	98	98	1	1	0.233	0.643	0.6	NA	0	0.492
1476	HERC5	HERC5	chr4	+	88470613	88470673	88472408	88472502	88469156	88469260	88475840	88476030	7	18	1	4	11	1	0	9	0	16	98	98	1	1	0.636	0.621	0.5	NA	0.36	0.226
1480	KDM5A	KDM5A	chr12	-	297040	297200	306945	307089	295572	295793	307453	308005	17	2	3	13	3	2	0	0	0	4	98	98	1	1	0.567	0.4	0.6	NA	0	0.522
1481	ELF2	ELF2	chr4	-	139061864	139062057	139067683	139067770	139060323	139060674	139071865	139072003	33	3	2	49	12	2	0	8	0	5	98	98	1	1	0.402	0.2	0.5	NA	0.615	-0.248
1484	KIAA0922	KIAA0922	chr4	+	153591052	153591194	153592474	153592584	153588889	153589007	153593798	153593871	19	14	1	9	13	0	0	3	0	4	98	98	1	1	0.679	0.519	1	NA	0.429	0.304
1491	LILRA2	LILRA2	chr19	+	54577464	54577723	54587009	54587060	54575806	54576109	54587200	54587561	5	4	4	11	0	6	0	3	0	1	98	98	1	1	0.312	1	0.4	NA	0.75	-0.179
1496	CPEB4	CPEB4	chr5	+	173932449	173932500	173943025	173943049	173910522	173910604	173944966	173945140	11	2	9	1	0	0	0	5	0	0	98	73	1	1	0.891	1	1	NA	1	-0.036
1498	PCBP1-AS1	PCBP1-AS1	chr2	-	70059642	70059680	70083537	70083687	70051202	70051305	70085546	70085624	8	0	0	5	3	0	0	1	0	0	98	87	1	1	0.587	0	NA	NA	1	-0.707
1499	RICTOR	RICTOR	chr5	-	38975536	38975604	38978582	38978648	38971876	38971959	38981866	38982036	0	0	0	33	29	1	0	1	0	5	98	98	1	1	0	0	0	NA	0.167	-0.167
1500	RICTOR	RICTOR	chr5	-	38975536	38975604	38978582	38978650	38971876	38971959	38981866	38982036	55	25	19	33	29	1	0	12	0	5	98	98	1	1	0.625	0.463	0.95	NA	0.706	-0.027
1501	FYB	FYB	chr5	-	39125997	39126135	39127740	39127807	39124252	39124278	39130589	39130612	91	29	17	59	19	8	0	25	0	8	98	98	1	1	0.607	0.604	0.68	NA	0.758	-0.128
1505	GAPT	GAPT	chr5	+	58493618	58493683	58493710	58493838	58491434	58491541	58494246	58496358	4	1	0	9	1	0	0	2	0	2	98	98	1	1	0.308	0.5	NA	NA	0.5	-0.096
1507	RAD17	RAD17	chr5	+	69372033	69372217	69373829	69374087	69371034	69371171	69374627	69374711	4	0	0	1	0	0	0	0	0	1	98	98	1	1	0.8	NA	NA	NA	0	0.8
1508	MRPS18C	MRPS18C	chr4	+	83458345	83458429	83459739	83459797	83456908	83456958	83460972	83461032	3	0	1	2	2	5	0	3	0	5	98	98	1	1	0.6	0	0.167	NA	0.375	-0.119
1510	ZFAND5	ZFAND5	chr9	-	72359417	72359521	72360109	72360221	72356930	72357056	72360627	72360784	2	0	0	42	6	4	0	0	0	8	98	98	1	1	0.045	0	0	NA	0	0.015
1513	SSBP2	SSBP2	chr5	-	81489249	81489309	81513627	81513717	81474495	81474562	81615472	81615557	8	6	0	4	1	3	0	5	0	3	98	98	1	1	0.667	0.857	0	NA	0.625	-0.117
1514	VCAN	VCAN	chr5	+	83519348	83522309	83537006	83542268	83512102	83512396	83545536	83545650	1	0	0	56	0	0	0	1	2	51	98	98	1	1	0.018	NA	NA	0	0.019	0.008
1523	PPP2R4	PPP2R4	chr9	+	129123051	129123138	129128984	129129110	129120512	129120610	129131521	129131639	1	0	0	2	0	0	0	1	2	1	98	98	1	1	0.333	NA	NA	0	0.5	0.083
1524	RAPGEF6	RAPGEF6	chr5	-	131561977	131562047	131592382	131592466	131548046	131548190	131603270	131603327	4	3	0	8	6	1	0	0	0	1	98	98	1	1	0.333	0.333	0	NA	0	0.222
1525	IRF1	IRF1	chr5	-	132484361	132484497	132485666	132485716	132481608	132484075	132486250	132486373	53	34	26	43	18	10	0	24	0	8	98	98	1	1	0.552	0.654	0.722	NA	0.75	-0.107
1533	ZNF438	ZNF438	chr10	-	30910359	30910440	30923286	30923415	30908932	30909015	30941574	30941651	5	4	0	1	1	0	0	1	0	0	98	98	1	1	0.833	0.8	NA	NA	1	-0.183
1534	ZNF438	ZNF438	chr10	-	30923286	30923415	30941574	30941651	30908932	30909015	30984368	30984426	11	10	1	5	1	0	0	3	0	0	98	98	1	1	0.687	0.909	1	NA	1	-0.135
1545	MAPK9	MAPK9	chr5	-	180247438	180247510	180247854	180247926	180242572	180242755	180248972	180249138	0	1	0	3	0	0	0	0	0	1	98	98	1	1	0	1	NA	NA	0	0.5
1546	CASP10	CASP10	chr2	+	201203729	201203766	201208074	201208183	201195841	201195948	201209069	201209562	2	0	0	2	0	0	0	1	0	1	86	98	1	1	0.533	NA	NA	NA	0.533	0
1548	RREB1	RREB1	chr6	+	7240437	7240602	7246423	7247221	7228996	7231907	7248510	7251980	2	0	0	1	0	0	0	0	1	0	98	98	1	1	0.667	NA	NA	0	NA	0.667

U12_MXE_filtered

													Inclusion counts			Skipping counts			Inclusion counts		Skipping counts						Inclusion_level(normalized)					
**ID**	**Gene_ID**	**Gene_Symbol**	**Chromosome**	**Strand**	**1stExonStart_0base**	**1stExonEnd**	**2ndExonStart_0base**	**2ndExonEnd**	**UpstreamES**	**UpstreamEE**	**DownstreamES**	**DownstreamEE**	***control_s3***	***control_s4***	***control_s10***	***control_s3***	***control_s4***	***control_s10***	***ldg_14***	***lldg_s16***	***ldg_s14***	***ldg_s16***	***IncFormLen***	***SkipFormLen***	***p-value***	***adjust_p***	***IncLevel_s3***	***IncLevel_s4***	***IncLevel_s10***	***IncLevel14***	***IncLevel16***	***Level_diff***
814	MAEA	MAEA	chr4	+	1322380	1322503	1327626	1327703	1315396	1315600	1332756	1332865	11	5	2	12	5	7	0	0	3	2	98	98	2.6768E-05	0.00253914	0.478	0.5	0.222	0	0	0.4
605	GBP5	GBP5	chr1	-	89268728	89268856	89269365	89269574	89267416	89267526	89272451	89272861	5	4	1	48	11	5	0	0	1	3	98	98	0.001832725	0.05290996	0.094	0.267	0.167	0	0	0.176
812	MAEA	MAEA	chr4	+	1315396	1315600	1322380	1322503	1311978	1312161	1327626	1327703	15	13	2	1	4	1	2	1	0	0	98	98	0.005546739	0.13153696	0.937	0.765	0.667	1	1	-0.21
832	DOCK2	DOCK2	chr5	+	169684195	169684350	169689251	169689333	169681743	169681879	169695802	169695938	109	35	17	83	48	5	2	13	3	21	98	98	0.086423567	0.896644508	0.568	0.422	0.773	0.4	0.382	0.197
813	MAEA	MAEA	chr4	+	1315396	1315600	1322380	1322503	1311978	1312161	1332756	1332865	15	16	2	0	3	1	3	2	0	0	98	98	0.297185281	1	1	0.842	0.667	1	1	-0.164
1464	WDR1	WDR1	chr4	-	10097710	10097891	10103895	10103986	10088663	10088741	10116112	10116234	36	16	13	37	7	7	4	6	2	3	98	98	0.401096861	1	0.493	0.696	0.65	0.667	0.667	-0.054
604	GBP5	GBP5	chr1	-	89267416	89267526	89268728	89268856	89266956	89267153	89269365	89269574	27	6	4	91	27	18	0	4	1	11	98	98	0.659255602	1	0.229	0.182	0.182	0	0.267	0.064
831	DOCK2	DOCK2	chr5	+	169670541	169670597	169671077	169671174	169669287	169669328	169674296	169674445	32	11	5	142	34	5	0	8	2	22	98	98	0.672793032	1	0.184	0.244	0.5	0	0.267	0.176
498	RASGRP4	RASGRP4	chr19	-	38417051	38417168	38419859	38420013	38414847	38415123	38420130	38420262	20	18	4	39	11	9	4	7	2	13	98	98	0.882368191	1	0.339	0.621	0.308	0.667	0.35	-0.086
432	FCHO2	FCHO2	chr5	+	73006444	73006549	73015625	73015724	72990711	72990864	73017211	73017308	15	3	0	22	9	1	0	6	0	2	98	98	1	1	0.405	0.25	0	NA	0.75	-0.532
451	AHCTF1	AHCTF1	chr1	-	246863923	246864116	246867243	246867351	246861958	246862153	246867660	246867811	1	0	0	26	16	4	0	0	0	3	98	98	1	1	0.037	0	0	NA	0	0.012
485	SLC9A8	SLC9A8	chr20	+	49839540	49839599	49845035	49845119	49823060	49823141	49849578	49849680	21	3	3	18	4	0	0	6	0	7	98	98	1	1	0.538	0.429	1	NA	0.462	0.194
486	SLC9A8	SLC9A8	chr20	+	49845035	49845119	49849578	49849680	49839540	49839599	49855437	49855581	13	2	0	14	9	0	0	4	0	4	98	98	1	1	0.481	0.182	NA	NA	0.5	-0.168
497	RASGRP4	RASGRP4	chr19	-	38417051	38417168	38419859	38420013	38413393	38413474	38420130	38420262	20	18	4	1	0	1	4	7	0	0	98	98	1	1	0.952	1	0.8	1	1	-0.083
648	DAP3	DAP3	chr1	+	155717005	155717128	155721516	155721618	155709772	155709824	155725381	155725490	6	1	2	5	7	1	0	0	0	3	98	98	1	1	0.545	0.125	0.667	NA	0	0.446
656	SFI1	SFI1	chr22	+	31528689	31528863	31546860	31546971	31508254	31508376	31550253	31550348	3	5	1	4	8	0	0	0	0	2	98	98	1	1	0.429	0.385	1	NA	0	0.605
763	RAB6A	RAB6A	chr11	-	73718612	73718718	73718784	73718890	73716250	73716362	73720845	73720899	0	0	1	11	3	4	0	0	0	2	98	98	1	1	0	0	0.2	NA	0	0.067
805	CNIH4	CNIH4	chr1	+	224360494	224360563	224364108	224364455	224356810	224356993	224365878	224365991	3	2	7	2	1	0	0	2	0	0	98	98	1	1	0.6	0.667	1	NA	1	-0.244
979	MAPK14	MAPK14	chr6	+	36095986	36096066	36100187	36100267	36076536	36076608	36102570	36102649	31	21	14	18	11	4	0	8	0	1	98	98	1	1	0.633	0.656	0.778	NA	0.889	-0.2
985	DYNC1LI2	DYNC1LI2	chr16	-	66742437	66742668	66749196	66749313	66736074	66736244	66751272	66751346	19	2	0	2	5	1	0	2	0	1	98	98	1	1	0.905	0.286	0	NA	0.667	-0.27
1146	DCP2	DCP2	chr5	+	113003941	113004077	113007937	113008042	113001566	113001674	113010755	113010807	12	8	7	26	7	2	0	5	0	1	98	98	1	1	0.316	0.533	0.778	NA	0.833	-0.291
1250	PQLC3	PQLC3	chr2	+	11171924	11172045	11174967	11175009	11164203	11164261	11177736	11178872	2	0	3	0	1	0	0	1	0	1	98	91	1	1	1	0	1	NA	0.481	0.186
1289	SMPD4	SMPD4	chr2	-	130173278	130173354	130173513	130173656	130172786	130172895	130174913	130175000	0	0	0	0	5	0	0	2	0	0	98	98	1	1	NA	0	NA	NA	1	-1
1545	MAPK9	MAPK9	chr5	-	180247438	180247510	180247854	180247926	180242572	180242755	180248972	180249138	0	1	0	3	0	0	0	0	0	1	98	98	1	1	0	1	NA	NA	0	0.5

Supplementary Data 2S
4_events_all
SLE LDG versus SLE NDG

											IJC_SAMPLE_1			SJC_SAMPLE_1			IJC_SAMPLE_2			SJC_SAMPLE_2							IncLevel1			IncLevel2			
**Event**	**ID**	**Gene_ID**	**Chromosome**	***Strand***	***LongExonStart_0base***	***LongExonEnd***	***ShortES***	***ShortEE***	***FlankingES***	***FlankingEE***	**ldg_12**	**ldg_14**	**ldg_16**	**ldg_12**	**ldg_14**	**ldg_16**	**pmn_13**	**pmn_15**	**pmn_17**	**pmn_13**	**pmn_15**	**pmn_17**	**IncFormLen**	**SkipFormLen**	**p value**	**FDR**	**ldg_12**	**ldg_14**	**ldg_16**	**pmn_13**	**pmn_15**	**pmn_17**	**IncLevelDifference**
SE	13845	C10orf54	chr10	-	71761597	71762026	71760867	71760924	71773357	71773580	0	4	72	0	0	0	1	16	24	0	1	3	98	49	0.001516497	0.170760496	NA	1	1	1	0.889	0.8	0.104
SE	1661	FAM193B	chr5	-	177531281	177531521	177524184	177525205	177532442	177532641	0	3	7	0	0	0	0	1	2	0	1	2	98	49	0.001603576	0.170760496	NA	1	1	NA	0.333	0.333	0.667
SE	7639	IRF7	chr11	-	614173	614399	613784	613865	614475	614534	0	0	0	0	8	3	0	4	1	0	3	2	98	49	0.001756837	0.170760496	NA	0	0	NA	0.4	0.2	-0.3
SE	6866	ATG2A	chr11	-	64906664	64906815	64906333	64906533	64907254	64907439	0	2	5	0	0	0	0	6	5	0	2	1	98	49	0.001882581	0.170760496	NA	1	1	NA	0.6	0.714	0.343
SE	9706	REC8	chr14	+	24175542	24175624	24173290	24173411	24176821	24176901	0	3	10	0	0	0	1	3	1	0	2	4	98	49	0.002515847	0.170760496	NA	1	1	1	0.429	0.111	0.487
SE	13860	ACSL1	chr4	-	184764852	184764925	184763166	184763255	184765890	184765986	0	2	50	0	0	0	7	51	14	0	2	2	98	49	0.003078802	0.170760496	NA	1	1	1	0.927	0.778	0.098
SE	8555	PIK3AP1	chr10	-	96652697	96652842	96651508	96651651	96656797	96656934	0	1	15	0	0	0	0	4	7	0	2	1	98	49	0.003471069	0.170760496	NA	1	1	NA	0.5	0.778	0.361
SE	11710	SENP3-EIF4A1	chr17	+	7575118	7575258	7574545	7574678	7576523	7576692	0	2	33	0	0	0	3	11	2	0	1	1	98	49	0.004779846	0.170760496	NA	1	1	1	0.846	0.5	0.218
SE	12939	SMCHD1	chr18	+	2666156	2666232	2655886	2656261	2666869	2667031	0	20	98	0	0	0	4	61	8	0	1	1	98	49	0.00491362	0.170760496	NA	1	1	1	0.968	0.8	0.077
A3SS	1863	SRPK1	chr6	-	35890894	35891041	35890894	35891013	35920467	35920528	0	0	0	0	1	2	0	1	1	0	4	1	77	49	0.005762586	0.197717869	NA	0	0	NA	0.137	0.389	-0.263
SE	12824	SEC14L1	chr17	+	77196201	77196311	77194676	77194911	77200483	77200673	0	11	34	0	0	0	0	20	0	0	11	2	98	49	0.005971369	0.170760496	NA	1	1	NA	0.476	0	0.762
A5SS	72	CSF2RA	chrX	+	1274754	1274842	1274754	1274818	1282677	1282779	0	1	3	0	0	0	0	2	0	0	2	1	73	49	0.006411688	0.170414363	NA	1	1	NA	0.402	0	0.799
SE	4425	PREX1	chr20	-	48659918	48660061	48658135	48658228	48666282	48666355	0	1	31	0	0	0	0	16	2	0	1	1	98	49	0.008019007	0.170760496	NA	1	1	NA	0.889	0.5	0.305
SE	11393	ADGRG3	chr16	+	57676199	57676338	57673320	57673468	57678169	57678316	0	2	14	0	0	0	0	25	2	0	3	3	98	49	0.008245186	0.170760496	NA	1	1	NA	0.806	0.25	0.472
SE	9930	PTPRE	chr10	+	128060938	128061015	128056122	128056213	128061678	128061715	0	3	11	0	0	0	0	22	0	0	7	0	98	49	0.009650972	0.170760496	NA	1	1	NA	0.611	NA	0.389
SE	5497	ADAM8	chr10	-	133270880	133271070	133270735	133270805	133271199	133271289	1	20	40	0	0	0	4	55	21	0	1	2	98	49	0.010036062	0.170760496	1	1	1	1	0.965	0.84	0.065
SE	11923	RBM39	chr20	-	35740524	35740597	35738967	35739017	35740823	35740887	0	2	8	0	0	1	0	8	0	0	8	0	98	49	0.010118399	0.170760496	NA	1	0.8	NA	0.333	NA	0.567
SE	14093	NLRP6	chr11	+	280083	281839	279833	279872	282704	282797	0	1	2	0	0	0	0	1	0	0	1	2	98	49	0.010649553	0.170760496	NA	1	1	NA	0.333	0	0.834
SE	10848	IRF1	chr5	-	132486250	132486373	132485666	132485716	132486556	132486686	0	2	36	0	0	1	7	13	0	0	6	0	98	49	0.011205299	0.170760496	NA	1	0.947	1	0.52	NA	0.214
SE	2075	HERC5	chr4	+	88462134	88462356	88460094	88460171	88463531	88463623	0	4	25	0	0	2	0	11	0	0	5	0	98	49	0.011241877	0.170760496	NA	1	0.862	NA	0.524	NA	0.407
SE	8188	EPSTI1	chr13	-	42919299	42919332	42917540	42917624	42926335	42926429	0	0	4	0	4	14	0	6	0	0	10	0	82	49	0.011354222	0.170760496	NA	0	0.146	NA	0.264	NA	-0.191
SE	4426	PREX1	chr20	-	48726289	48726391	48708259	48708421	48734545	48734650	1	23	82	0	0	0	8	65	15	0	2	3	98	49	0.01202775	0.170760496	1	1	1	1	0.942	0.714	0.115
SE	3738	BRD2	chr6	+	32975020	32975112	32974461	32974765	32975383	32975521	0	1	3	0	0	3	0	4	0	0	8	0	98	49	0.012603077	0.170760496	NA	1	0.333	NA	0.2	NA	0.466
SE	3103	MX2	chr21	+	41380016	41380151	41377788	41377981	41382409	41382564	0	4	25	0	0	2	0	16	5	0	11	2	98	49	0.013190421	0.170760496	NA	1	0.862	NA	0.421	0.556	0.443
SE	10602	DMXL2	chr15	-	51495023	51495134	51491577	51491747	51498551	51500231	0	1	12	0	0	0	0	7	0	0	5	0	98	49	0.014099979	0.170760496	NA	1	1	NA	0.412	NA	0.588
SE	3545	OSBPL2	chr20	+	62259980	62260125	62256056	62256221	62263615	62263691	0	2	3	0	0	0	0	5	0	0	4	0	98	49	0.014587636	0.170760496	NA	1	1	NA	0.385	NA	0.615
SE	12803	CYP4F3	chr19	+	15645718	15645863	15641414	15641613	15647051	15647105	0	5	21	0	0	0	7	13	0	0	3	0	98	49	0.014732939	0.170760496	NA	1	1	1	0.684	NA	0.158
SE	1826	MCTP1	chr5	-	94917895	94917973	94912805	94912976	94923961	94924021	0	2	3	0	0	0	0	4	0	0	4	0	98	49	0.014963682	0.170760496	NA	1	1	NA	0.333	NA	0.667
SE	6336	FCAR	chr19	+	54888006	54888294	54885234	54885525	54889648	54891420	0	5	28	0	0	1	0	19	0	0	3	0	98	49	0.01511042	0.170760496	NA	1	0.933	NA	0.76	NA	0.207
SE	2203	CPEB2	chr4	+	15040463	15040487	15033160	15033211	15052413	15052584	0	0	1	0	5	1	0	5	0	0	2	0	73	49	0.015421435	0.170760496	NA	0	0.402	NA	0.627	NA	-0.426
SE	12815	BST2	chr19	-	17404370	17404437	17404128	17404189	17405290	17405648	0	4	23	0	0	0	0	4	0	0	3	0	98	49	0.015554946	0.170760496	NA	1	1	NA	0.4	NA	0.6
SE	11842	RHOT1	chr17	+	32172936	32173060	32171042	32171101	32173830	32173912	0	5	3	0	0	0	0	3	0	0	3	0	98	49	0.015643271	0.170760496	NA	1	1	NA	0.333	NA	0.667
SE	6335	FCAR	chr19	+	54888006	54888228	54885234	54885525	54889648	54891420	0	3	17	0	0	1	0	12	0	0	3	0	98	49	0.015872783	0.170760496	NA	1	0.895	NA	0.667	NA	0.28
SE	5621	ANKRD12	chr18	+	9204475	9204544	9195550	9195698	9208656	9208803	0	1	3	0	0	2	0	5	0	0	4	0	98	49	0.016160337	0.170760496	NA	1	0.429	NA	0.385	NA	0.33
SE	10536	SGK1	chr6	-	134170825	134170915	134169245	134170435	134171022	134171178	0	1	11	0	0	0	1	16	0	0	4	0	98	49	0.016182238	0.170760496	NA	1	1	1	0.667	NA	0.166
SE	6177	ABCA1	chr9	-	104825682	104825887	104824464	104824578	104826947	104827169	0	4	6	0	0	0	0	21	1	0	5	0	98	49	0.016495032	0.170760496	NA	1	1	NA	0.677	1	0.161
SE	799	TBXAS1	chr7	+	139911224	139911321	139875584	139875637	139936190	139936307	0	2	22	0	0	0	0	7	0	0	3	0	98	49	0.016691194	0.170760496	NA	1	1	NA	0.538	NA	0.462
A5SS	1875	N4BP2L2	chr13	-	32527407	32527532	32527457	32527532	32522181	32522270	0	2	17	0	0	2	0	9	0	0	3	0	98	49	0.016742666	0.170414363	NA	1	0.81	NA	0.6	NA	0.305
SE	7593	ATG16L2	chr11	+	72822847	72822961	72822477	72822543	72824059	72824122	0	9	41	0	0	1	4	19	16	0	4	2	98	49	0.017045614	0.170760496	NA	1	0.953	1	0.704	0.8	0.142
A3SS	2805	TPT1	chr13	-	45333470	45337553	45333470	45337388	45338659	45338776	0	0	2	1	3	72	0	4	0	0	47	0	98	49	0.017197054	0.197717869	0	0	0.014	NA	0.041	NA	-0.036
SE	883	SH2B2	chr7	+	102300521	102301279	102285061	102285258	102306720	102306822	0	4	0	0	0	0	0	2	1	0	3	0	98	49	0.017202771	0.170760496	NA	1	NA	NA	0.25	1	0.375
SE	1452	PRRC2A	chr6	+	31625791	31625871	31625459	31625611	31626019	31626162	0	4	4	0	0	1	0	1	0	0	5	0	98	49	0.017880599	0.170760496	NA	1	0.667	NA	0.091	NA	0.743
SE	6626	PCBP2	chr12	+	53464759	53464852	53462480	53462567	53465931	53465973	0	0	23	0	2	16	0	11	0	0	1	0	98	49	0.017987559	0.170760496	NA	0	0.418	NA	0.846	NA	-0.637
SE	6174	ABCA1	chr9	-	104810799	104810924	104809465	104809564	104812573	104812722	0	0	3	0	1	1	0	4	0	2	4	0	98	49	0.018518561	0.170760496	NA	0	0.6	0	0.333	NA	0.133
SE	11731	TAOK3	chr12	-	118212913	118212995	118201295	118201463	118214016	118214110	1	2	4	0	0	1	0	2	0	0	3	0	98	49	0.018766874	0.170760496	1	1	0.667	NA	0.25	NA	0.639
SE	3208	STX16-NPEPL1	chr20	+	58671153	58671297	58670511	58670603	58673630	58673711	0	1	16	0	0	1	0	5	0	0	3	0	98	49	0.018766876	0.170760496	NA	1	0.889	NA	0.455	NA	0.489
SE	1982	DDX60L	chr4	-	168419301	168419397	168416681	168416797	168420260	168420380	0	1	32	0	0	1	0	8	0	0	3	0	98	49	0.018767562	0.170760496	NA	1	0.941	NA	0.571	NA	0.399
SE	6334	FCAR	chr19	+	54888006	54888220	54885234	54885525	54889648	54891420	0	1	11	0	0	1	0	10	0	0	3	0	98	49	0.018937654	0.170760496	NA	1	0.846	NA	0.625	NA	0.298
SE	4057	APBB1IP	chr10	+	26536073	26536217	26533438	26533525	26541581	26541692	0	1	12	0	0	0	3	4	0	0	3	0	98	49	0.018984944	0.170760496	NA	1	1	1	0.4	NA	0.3
SE	3889	MAP7D1	chr1	+	36174897	36175008	36173363	36173478	36176198	36176321	0	0	4	0	2	2	0	8	0	0	1	0	98	49	0.019005986	0.170760496	NA	0	0.5	NA	0.8	NA	-0.55
A3SS	1378	OSBPL8	chr12	-	76450850	76450988	76450850	76450979	76487509	76487618	0	0	3	0	1	1	0	5	0	0	2	0	58	49	0.019031697	0.197717869	NA	0	0.717	NA	0.679	NA	-0.321
SE	5821	MGEA5	chr10	-	101793912	101793998	101792838	101792943	101797979	101798154	0	0	3	0	2	1	0	3	0	0	4	0	98	49	0.019064109	0.170760496	NA	0	0.6	NA	0.273	NA	0.027
SE	5241	PLXNC1	chr12	+	94209589	94209704	94186372	94186473	94220015	94220163	0	4	27	0	0	1	0	5	0	0	2	0	98	49	0.019110193	0.170760496	NA	1	0.931	NA	0.556	NA	0.409
A3SS	3389	RBM39	chr20	-	35709223	35709274	35709223	35709256	35713018	35713096	0	0	7	0	1	1	0	4	0	0	3	0	67	49	0.019156561	0.197717869	NA	0	0.837	NA	0.494	NA	-0.076
SE	10591	RNF213	chr17	+	80383676	80383928	80382978	80383070	80385537	80385621	0	1	30	0	0	0	1	12	0	0	3	0	98	49	0.019182317	0.170760496	NA	1	1	1	0.667	NA	0.166
SE	11022	TAP1	chr6	-	32847918	32848092	32847512	32847675	32848651	32848840	0	4	21	0	0	0	0	7	0	0	2	0	98	49	0.019207741	0.170760496	NA	1	1	NA	0.636	NA	0.364
A5SS	2325	DEDD2	chr19	-	42201988	42202072	42202050	42202072	42198592	42199829	0	4	0	0	0	1	2	3	0	0	2	0	98	49	0.019484918	0.170414363	NA	1	0	1	0.429	NA	-0.215
SE	5270	BID	chr22	-	17750104	17750174	17743802	17744013	17774380	17774495	0	4	1	0	0	0	0	3	0	0	2	0	98	49	0.019484918	0.170760496	NA	1	1	NA	0.429	NA	0.571
SE	7455	FPR1	chr19	-	51750202	51750253	51745769	51747005	51751813	51751897	0	1	2	0	0	1	0	3	0	0	3	0	98	49	0.019485123	0.170760496	NA	1	0.5	NA	0.333	NA	0.417
SE	7415	VAV1	chr19	+	6821790	6821859	6821621	6821680	6822220	6822329	0	4	4	0	0	0	3	11	0	0	2	0	98	49	0.01972669	0.170760496	NA	1	1	1	0.733	NA	0.133
SE	9526	EIF4G2	chr11	-	10801660	10801774	10800961	10801087	10802048	10802209	0	0	6	0	1	1	2	10	0	0	1	0	98	49	0.01979002	0.170760496	NA	0	0.75	1	0.833	NA	-0.541
SE	9353	GBP5	chr1	-	89262682	89262785	89262219	89262401	89263735	89263948	0	3	3	0	0	0	1	6	0	0	2	0	98	49	0.019996576	0.170760496	NA	1	1	1	0.6	NA	0.2
SE	12946	SMCHD1	chr18	+	2750042	2750122	2747521	2747647	2750349	2750507	0	2	54	0	0	8	0	46	0	0	3	0	98	49	0.020053524	0.170760496	NA	1	0.771	NA	0.885	NA	0
SE	496	EEF1D	chr8	-	143586218	143586290	143581228	143581328	143586728	143586849	0	3	12	0	0	0	0	8	0	0	2	0	98	49	0.020218801	0.170760496	NA	1	1	NA	0.667	NA	0.333
SE	4780	TXNRD1	chr12	+	104321090	104321316	104319469	104319585	104325336	104325429	0	3	2	0	0	0	0	8	0	0	2	0	98	49	0.020218801	0.170760496	NA	1	1	NA	0.667	NA	0.333
SE	4967	DDX3X	chrX	+	41347314	41347451	41346858	41347012	41347639	41350287	0	1	17	0	0	0	0	20	0	0	3	0	98	49	0.020293915	0.170760496	NA	1	1	NA	0.769	NA	0.231
SE	1577	USP9X	chrX	+	41123470	41123724	41085634	41086109	41128999	41129145	0	0	4	0	0	0	0	1	2	0	0	2	98	49	0.020294338	0.170760496	NA	NA	1	NA	1	0.333	0.334
A5SS	976	CTAGE5	chr14	+	39276933	39277065	39276933	39276955	39279336	39279358	0	1	6	0	0	0	0	2	0	0	3	0	98	49	0.020627966	0.170414363	NA	1	1	NA	0.25	NA	0.75
SE	9058	FAM107B	chr10	-	14553321	14553387	14530331	14530515	14604239	14604418	0	1	0	0	0	0	0	2	0	0	3	0	98	49	0.020627966	0.170760496	NA	1	NA	NA	0.25	NA	0.75
SE	11109	USP25	chr21	+	15824038	15824166	15818697	15818846	15824965	15825061	0	1	3	0	0	2	0	2	0	0	3	0	98	49	0.020627966	0.170760496	NA	1	0.429	NA	0.25	NA	0.465
SE	7818	DPEP2	chr16	-	67990046	67990131	67989322	67989398	67990820	67990997	0	1	15	0	0	0	0	5	0	0	1	1	98	49	0.020811238	0.170760496	NA	1	1	NA	0.714	0	0.643
SE	3457	CDS2	chr20	+	5186686	5186839	5185757	5185826	5189066	5189186	0	3	8	0	0	0	0	1	0	0	3	0	98	49	0.020882905	0.170760496	NA	1	1	NA	0.143	NA	0.857
A5SS	2345	HN1	chr17	-	75147519	75147653	75147555	75147653	75146665	75146684	2	0	1	0	1	2	0	0	0	4	5	0	85	49	0.020913098	0.170414363	1	0	0.224	0	0	NA	0.408
SE	6630	PCBP2	chr12	+	53467804	53467843	53467220	53467293	53468776	53468832	0	1	9	0	0	7	8	2	0	1	3	0	88	49	0.020946855	0.170760496	NA	1	0.417	0.817	0.271	NA	0.164
SE	1457	ATP6V1G2-DDX39B	chr6	-	31539146	31539274	31538762	31538855	31540321	31540664	0	2	20	0	0	0	0	4	0	0	2	0	98	49	0.021248483	0.170760496	NA	1	1	NA	0.5	NA	0.5
SE	6867	ATG2A	chr11	-	64908990	64909150	64907747	64907890	64909270	64909367	0	0	2	0	0	0	0	10	4	0	1	2	98	49	0.021248483	0.170760496	NA	NA	1	NA	0.833	0.5	0.334
A5SS	2264	MTCH1	chr6	-	36972651	36972796	36972702	36972796	36970646	36970694	0	2	9	0	0	8	0	6	0	0	2	0	98	49	0.021315983	0.170414363	NA	1	0.36	NA	0.6	NA	0.08
SE	12526	GUCD1	chr22	-	24548916	24549001	24547907	24548073	24554948	24555307	0	2	0	0	0	0	0	6	2	0	2	0	98	49	0.021315983	0.170760496	NA	1	NA	NA	0.6	1	0.2
SE	5012	ADRBK1	chr11	+	67281092	67281184	67280731	67280783	67281458	67281558	0	2	32	0	0	0	0	7	0	0	2	0	98	49	0.021459804	0.170760496	NA	1	1	NA	0.636	NA	0.364
SE	2371	MFSD1	chr3	+	158821983	158822140	158821596	158821653	158823427	158823525	0	2	16	0	0	0	0	3	0	0	2	0	98	49	0.021489596	0.170760496	NA	1	1	NA	0.429	NA	0.571
SE	10746	MAN2C1	chr15	-	75363998	75364188	75362641	75362748	75364487	75364665	0	2	0	0	0	2	0	3	0	0	2	0	98	49	0.021489596	0.170760496	NA	1	0	NA	0.429	NA	0.071
SE	13957	DIP2B	chr12	+	50671185	50671398	50660193	50660319	50674473	50674629	0	2	5	0	0	0	0	3	0	0	2	0	98	49	0.021489596	0.170760496	NA	1	1	NA	0.429	NA	0.571
SE	12161	HSH2D	chr19	+	16151446	16151578	16148723	16148875	16152551	16153208	0	0	1	0	4	15	0	2	0	0	9	0	98	49	0.021888757	0.170760496	NA	0	0.032	NA	0.1	NA	-0.084
SE	6970	MFN2	chr1	+	12007052	12007249	12006537	12006693	12009591	12009726	0	0	5	0	1	0	0	6	0	0	1	0	98	49	0.021892715	0.170760496	NA	0	1	NA	0.75	NA	-0.25
A3SS	689	CNBP	chr3	-	129171078	129171280	129171078	129171277	129171445	129171538	0	0	2	0	1	4	0	3	0	0	11	0	52	49	0.021942316	0.197717869	NA	0	0.32	NA	0.204	NA	-0.044
A3SS	490	GRK6	chr5	+	177441734	177442849	177441736	177442849	177440918	177441053	0	1	6	0	0	2	0	0	0	0	2	2	51	49	0.021987328	0.197717869	NA	1	0.742	NA	0	0	0.871
SE	4999	WIPI2	chr7	+	5193117	5193171	5190203	5190493	5199575	5199658	0	1	3	0	0	1	0	0	0	0	2	2	98	49	0.021999349	0.170760496	NA	1	0.6	NA	0	0	0.8
SE	2204	CPEB2	chr4	+	15059186	15059301	15058420	15058539	15062078	15062260	0	1	0	0	1	2	0	3	3	0	0	0	98	49	0.022049079	0.170760496	NA	0.333	0	NA	1	1	-0.834
A5SS	2821	LILRA6	chr19	-	54241575	54241878	54241661	54241878	54240830	54241127	0	0	0	0	2	1	0	4	0	0	1	0	98	49	0.022093415	0.170414363	NA	0	0	NA	0.667	NA	-0.667
SE	9285	VIM	chr10	+	17235845	17235889	17235168	17235389	17236293	17236379	0	12	171	0	0	0	3	36	3	0	3	0	93	49	0.022225644	0.170760496	NA	1	1	1	0.863	1	0.046
SE	13688	BAZ2A	chr12	-	56605829	56606063	56605072	56605327	56606246	56606312	0	2	2	0	0	0	0	2	0	0	2	0	98	49	0.022262485	0.170760496	NA	1	1	NA	0.333	NA	0.667
SE	14090	AREL1	chr14	-	74669946	74670126	74669648	74669774	74670761	74670871	0	2	2	0	0	3	0	2	0	0	2	0	98	49	0.022262485	0.170760496	NA	1	0.25	NA	0.333	NA	0.292
SE	6878	UBN1	chr16	+	4868832	4868903	4860663	4861102	4870211	4870341	0	0	4	0	1	0	0	5	0	0	1	0	98	49	0.022832961	0.170760496	NA	0	1	NA	0.714	NA	-0.214
SE	11349	ADGRE2	chr19	-	14754953	14755127	14752328	14752526	14756237	14756345	0	0	3	0	1	1	0	5	0	0	1	0	98	49	0.022832961	0.170760496	NA	0	0.6	NA	0.714	NA	-0.414
SE	3680	USP15	chr12	+	62325871	62325933	62321463	62321609	62349220	62349307	0	2	32	0	0	1	0	15	0	0	2	0	98	49	0.023010064	0.170760496	NA	1	0.941	NA	0.789	NA	0.181
SE	10210	RFWD2	chr1	-	176175909	176176007	176163814	176163891	176184632	176184692	0	2	13	0	0	0	0	17	0	0	2	0	98	49	0.023408389	0.170760496	NA	1	1	NA	0.81	NA	0.19
SE	558	PABPC1	chr8	-	100713086	100713181	100712651	100712789	100715461	100715601	0	6	63	0	0	0	5	32	2	0	3	0	98	49	0.0234244	0.170760496	NA	1	1	1	0.842	1	0.053
SE	17	FLNA	chrX	-	154353553	154353727	154353295	154353457	154353914	154354043	0	2	35	0	0	1	0	18	0	2	2	0	98	49	0.023592394	0.170760496	NA	1	0.946	0	0.818	NA	0.564
A5SS	93	GAPVD1	chr9	+	125321432	125321562	125321432	125321499	125323797	125323923	0	3	3	0	0	0	0	1	0	0	2	0	98	49	0.023673881	0.170414363	NA	1	1	NA	0.2	NA	0.8
SE	2652	SETD2	chr3	-	47101457	47101555	47097954	47098081	47103345	47103423	0	1	10	0	0	0	0	4	0	0	2	0	98	49	0.023775553	0.170760496	NA	1	1	NA	0.5	NA	0.5
SE	4064	FBXL5	chr4	-	15638507	15638694	15636493	15636676	15640787	15640883	0	3	71	0	0	0	6	32	0	0	2	0	98	49	0.0238404	0.170760496	NA	1	1	1	0.889	NA	0.055
A3SS	681	CDV3	chr3	+	133587158	133590274	133587895	133590274	133586562	133586722	0	1	2	0	0	0	0	5	0	0	2	0	98	49	0.023845162	0.197717869	NA	1	1	NA	0.556	NA	0.444
SE	3215	PDCD6IP	chr3	+	33812071	33812126	33798570	33798937	33813558	33813628	0	1	6	0	0	0	0	1	0	0	3	0	98	49	0.023847213	0.170760496	NA	1	1	NA	0.143	NA	0.857
SE	4847	TINF2	chr14	-	24241889	24241994	24241674	24241776	24242140	24242674	0	1	0	0	0	0	0	1	0	0	3	0	98	49	0.023847213	0.170760496	NA	1	NA	NA	0.143	NA	0.857
SE	2082	HERC6	chr4	+	88393487	88393582	88390651	88390879	88396014	88396142	0	1	12	0	0	0	0	3	0	0	2	0	98	49	0.023922591	0.170760496	NA	1	1	NA	0.429	NA	0.571
SE	7201	MDM4	chr1	+	204537429	204537497	204532190	204532246	204538208	204538308	0	1	6	0	0	0	0	3	0	0	2	0	98	49	0.023922591	0.170760496	NA	1	1	NA	0.429	NA	0.571
SE	10725	PTMA	chr2	+	231711889	231711983	231711347	231711419	231712442	231712516	0	1	3	0	0	0	0	3	0	0	2	0	98	49	0.023922591	0.170760496	NA	1	1	NA	0.429	NA	0.571
SE	6590	UQCRH	chr1	+	46309896	46310044	46309100	46309127	46310154	46310316	0	0	1	0	2	0	0	2	0	3	3	0	98	49	0.024004538	0.170760496	NA	0	1	0	0.25	NA	0.375
SE	4111	ATP2B1	chr12	-	89601325	89601433	89599116	89599299	89603042	89603254	0	1	2	0	0	0	2	6	0	0	2	0	98	49	0.024023827	0.170760496	NA	1	1	1	0.6	NA	0.2
SE	5016	ARHGAP25	chr2	+	68813286	68813419	68807272	68807480	68816288	68816362	0	1	15	0	0	0	0	6	0	0	2	0	98	49	0.024023827	0.170760496	NA	1	1	NA	0.6	NA	0.4
SE	7011	HIPK3	chr11	+	33348521	33348818	33347851	33348013	33349146	33349287	0	1	4	0	0	0	0	6	0	0	2	0	98	49	0.024023827	0.170760496	NA	1	1	NA	0.6	NA	0.4
SE	10363	SOS2	chr14	-	50145169	50145332	50139941	50140059	50145476	50145596	0	1	9	0	0	1	0	6	0	0	2	0	98	49	0.024023827	0.170760496	NA	1	0.818	NA	0.6	NA	0.309
SE	10724	PTMA	chr2	+	231711886	231711983	231711347	231711419	231712442	231712516	0	1	2	0	0	0	0	6	0	0	2	0	98	49	0.024023827	0.170760496	NA	1	1	NA	0.6	NA	0.4
SE	11386	NLRC5	chr16	+	57046551	57046641	57045447	57045492	57047544	57047628	0	1	10	0	0	0	0	6	0	0	2	0	98	49	0.024023827	0.170760496	NA	1	1	NA	0.6	NA	0.4
SE	690	CDK16	chrX	+	47225771	47225866	47224987	47225102	47225964	47226027	0	1	4	0	0	0	0	7	0	0	2	0	98	49	0.02426018	0.170760496	NA	1	1	NA	0.636	NA	0.364
SE	7907	IFIT1	chr10	+	89399894	89400003	89392545	89392717	89402280	89406487	0	1	12	0	0	5	0	7	0	4	2	0	98	49	0.02426018	0.170760496	NA	1	0.545	0	0.636	NA	0.454
SE	8359	ARHGAP30	chr1	-	161059613	161059716	161054622	161054705	161069527	161069970	0	1	18	0	0	2	0	7	0	0	2	0	98	49	0.02426018	0.170760496	NA	1	0.818	NA	0.636	NA	0.273
SE	9209	CELF2	chr10	+	11217424	11217507	11165485	11165682	11249152	11249201	0	1	31	0	0	2	5	7	0	0	2	0	98	49	0.02426018	0.170760496	NA	1	0.886	1	0.636	NA	0.125
SE	8140	ITGAX	chr16	+	31373248	31373390	31372596	31372670	31376798	31376915	0	12	40	0	0	5	6	46	13	0	0	0	98	49	0.024390083	0.170760496	NA	1	0.8	1	1	1	-0.1

SE	12583	SBNO2	chr19	-	1109134	1109211	1108778	1108969	1109291	1109423	0	4	9	0	0	0	0	25	2	0	3	0	98	49	0.024422917	0.170760496	NA	1	1	NA	0.806	1	0.097
SE	13765	ABCG1	chr21	+	42276894	42276945	42273302	42273435	42282273	42282419	0	1	11	0	0	0	0	8	0	0	2	0	98	49	0.024517355	0.170760496	NA	1	1	NA	0.667	NA	0.333
A3SS	683	CDV3	chr3	+	133587161	133590274	133587895	133590274	133586562	133586722	0	1	2	0	0	0	0	2	0	0	2	0	98	49	0.024627853	0.197717869	NA	1	1	NA	0.333	NA	0.667
SE	1480	FLOT1	chr6	-	30741613	30741704	30741189	30741333	30741791	30741867	0	1	6	0	0	1	0	2	0	0	2	0	98	49	0.024627853	0.170760496	NA	1	0.75	NA	0.333	NA	0.542
SE	3632	USP4	chr3	-	49300466	49300691	49298551	49298635	49302383	49302542	0	1	0	0	0	0	0	2	0	0	2	0	98	49	0.024627853	0.170760496	NA	1	NA	NA	0.333	NA	0.667
SE	4078	PHF1	chr6	+	33414954	33415144	33414724	33414829	33415234	33415329	0	1	1	0	0	0	0	2	0	0	2	0	98	49	0.024627853	0.170760496	NA	1	1	NA	0.333	NA	0.667
SE	4079	PHF1	chr6	+	33414965	33415144	33414724	33414829	33415234	33415329	0	1	1	0	0	0	0	2	0	0	2	0	98	49	0.024627853	0.170760496	NA	1	1	NA	0.333	NA	0.667
SE	6133	STXBP3	chr1	+	108779285	108779410	108776332	108776423	108782421	108782517	0	2	7	0	0	0	0	1	0	0	2	0	98	49	0.02492004	0.170760496	NA	1	1	NA	0.2	NA	0.8
SE	7313	WNK1	chr12	+	871264	871348	862082	862270	878211	878361	0	2	4	0	0	1	3	1	0	0	2	0	98	49	0.02492004	0.170760496	NA	1	0.667	1	0.2	NA	0.234
SE	8790	VPS13D	chr1	+	12311453	12311625	12308430	12308641	12311812	12311925	0	2	6	0	0	0	0	1	0	0	2	0	98	49	0.02492004	0.170760496	NA	1	1	NA	0.2	NA	0.8
A5SS	1139	TBC1D10C	chr11	+	67406626	67406738	67406626	67406692	67406826	67407016	0	1	2	0	0	4	0	3	3	0	0	0	95	49	0.024974929	0.170414363	NA	1	0.205	NA	1	1	-0.397
SE	5184	MYO9B	chr19	+	17145396	17145491	17101659	17102557	17152643	17152706	0	3	23	0	0	0	0	19	2	0	4	0	98	49	0.024989397	0.170760496	NA	1	1	NA	0.704	1	0.148
SE	13164	PPP1CB	chr2	+	28788657	28788809	28783906	28783978	28793862	28793997	0	1	6	0	0	0	0	10	0	0	2	0	98	49	0.025074234	0.170760496	NA	1	1	NA	0.714	NA	0.286
SE	2167	FYB	chr5	-	39201825	39202987	39153447	39153604	39219442	39219578	0	8	22	0	0	0	1	10	1	0	2	0	98	49	0.025260336	0.170760496	NA	1	1	1	0.714	1	0.095
SE	2076	HERC5	chr4	+	88463854	88463985	88463531	88463623	88467058	88467204	0	2	27	0	0	1	0	28	0	0	2	0	98	49	0.02534026	0.170760496	NA	1	0.931	NA	0.875	NA	0.091
SE	4015	RPLP0	chr12	-	120198553	120198739	120197321	120197462	120198853	120199000	0	1	3	0	0	0	0	12	0	0	2	0	98	49	0.025646206	0.170760496	NA	1	1	NA	0.75	NA	0.25
SE	5192	PICALM	chr11	-	85976622	85976682	85974707	85974812	85981128	85981228	0	2	30	0	0	0	3	30	0	0	2	0	98	49	0.025657506	0.170760496	NA	1	1	1	0.882	NA	0.059
A3SS	932	CXCL1	chr4	+	73869645	73869792	73869668	73869792	73869391	73869570	0	0	0	0	0	3	3	1	2	0	1	1	72	49	0.025891313	0.197717869	NA	NA	0	1	0.405	0.576	-0.66
SE	7720	THEMIS2	chr1	+	27881970	27883043	27876587	27876728	27885294	27885451	0	0	2	0	3	1	0	2	0	0	1	0	98	49	0.025897097	0.170760496	NA	0	0.5	NA	0.5	NA	-0.25
SE	13194	MBOAT7	chr19	-	54188432	54188511	54188216	54188346	54189337	54189882	0	9	20	0	0	1	1	12	31	0	2	6	98	49	0.025921935	0.170760496	NA	1	0.909	1	0.75	0.721	0.131
SE	6180	ABCA1	chr9	-	104884426	104884568	104883038	104883157	104889101	104889195	0	3	20	0	1	1	0	16	2	0	7	3	98	49	0.026099399	0.170760496	NA	0.6	0.909	NA	0.533	0.25	0.363
SE	2583	LCP2	chr5	-	170270718	170270917	170268384	170268482	170274300	170274338	1	1	19	0	0	0	0	14	0	0	2	0	98	49	0.026208434	0.170760496	1	1	1	NA	0.778	NA	0.222
A5SS	2038	TRAPPC5	chr19	+	7680820	7681026	7680820	7680878	7682241	7682862	0	0	0	0	0	1	0	4	3	0	0	1	98	49	0.026257417	0.170414363	NA	NA	0	NA	1	0.6	-0.8
A5SS	2039	TRAPPC5	chr19	+	7680820	7681049	7680820	7680878	7682241	7682862	0	0	0	0	0	1	0	3	3	0	0	1	98	49	0.026257417	0.170414363	NA	NA	0	NA	1	0.6	-0.8
SE	6627	PCBP2	chr12	+	53464759	53464852	53462480	53462567	53467220	53467293	0	0	13	0	1	3	0	3	0	0	1	0	98	49	0.026257417	0.170760496	NA	0	0.684	NA	0.6	NA	-0.258
SE	7010	HIPK3	chr11	+	33348165	33348228	33347851	33348013	33348521	33348818	0	0	7	0	1	1	0	3	0	0	1	0	98	49	0.026257417	0.170760496	NA	0	0.778	NA	0.6	NA	-0.211
SE	12300	CYTH1	chr17	-	78702537	78702604	78702121	78702240	78708196	78708261	0	0	6	0	1	3	0	3	0	0	1	0	98	49	0.026257417	0.170760496	NA	0	0.5	NA	0.6	NA	-0.35
SE	6175	ABCA1	chr9	-	104812573	104812722	104809465	104809564	104814117	104814231	0	1	3	0	0	0	2	15	0	0	2	0	98	49	0.026481025	0.170760496	NA	1	1	1	0.789	NA	0.105
SE	5194	PICALM	chr11	-	85990249	85990378	85983865	85983973	85996825	85996929	0	1	18	0	0	21	0	16	0	0	2	0	98	49	0.026734839	0.170760496	NA	1	0.3	NA	0.8	NA	-0.15
SE	5975	TAP2	chr6	-	32835153	32835359	32832626	32832824	32835642	32835773	0	0	11	0	0	0	0	11	1	0	0	1	98	49	0.026878049	0.170760496	NA	NA	1	NA	1	0.333	0.334
SE	9687	IFRD1	chr7	+	112456913	112457038	112456001	112456086	112458860	112459018	0	6	52	0	0	0	0	84	4	0	3	0	98	49	0.026897321	0.170760496	NA	1	1	NA	0.933	1	0.033
SE	6887	SRPK1	chr6	-	35870280	35870494	35869481	35869901	35870933	35870959	0	0	4	0	1	5	0	2	0	0	3	0	98	49	0.027065308	0.170760496	NA	0	0.286	NA	0.25	NA	-0.107
A3SS	3741	TMEM91	chr19	+	41383560	41384082	41383862	41384082	41382771	41382921	0	1	3	0	0	3	0	1	0	0	2	0	98	49	0.027259421	0.197717869	NA	1	0.333	NA	0.2	NA	0.466
SE	2726	SATB1	chr3	-	18351991	18352195	18347640	18349682	18378169	18378325	0	1	3	0	0	0	0	1	0	0	2	0	98	49	0.027259421	0.170760496	NA	1	1	NA	0.2	NA	0.8
SE	9126	HGS	chr17	+	81690181	81690234	81688703	81688827	81690673	81690742	0	1	1	0	0	0	0	1	0	0	2	0	98	49	0.027259421	0.170760496	NA	1	1	NA	0.2	NA	0.8
SE	14102	SLC12A4	chr16	-	67951822	67952037	67951139	67951304	67952183	67952425	0	1	0	0	0	0	0	1	0	0	2	0	98	49	0.027259421	0.170760496	NA	1	NA	NA	0.2	NA	0.8
A5SS	1672	PTBP3	chr9	-	112297831	112297916	112297840	112297916	112275843	112276013	0	0	3	0	2	2	0	2	0	0	1	0	58	49	0.027334905	0.170414363	NA	0	0.559	NA	0.628	NA	-0.348
SE	8906	FBXW7	chr4	-	152412479	152412529	152411159	152411872	152534940	152535033	0	0	0	0	2	0	0	2	0	0	1	1	98	49	0.02734801	0.170760496	NA	0	NA	NA	0.5	0	-0.25
SE	3622	ITGAM	chr16	+	31266029	31266147	31265810	31265881	31270953	31271084	0	6	27	0	0	0	0	3	0	0	1	0	98	49	0.027600753	0.170760496	NA	1	1	NA	0.6	NA	0.4
A3SS	405	BAG6	chr6	-	31647590	31647844	31647590	31647826	31648676	31648751	0	0	0	0	1	2	0	2	0	0	2	0	67	49	0.027623127	0.197717869	NA	0	0	NA	0.422	NA	-0.422
SE	3281	RAB11FIP1	chr8	-	37871277	37873179	37870419	37870528	37874514	37875322	0	0	4	0	1	4	0	2	0	0	2	0	98	49	0.027623276	0.170760496	NA	0	0.333	NA	0.333	NA	-0.167
SE	5922	MKNK1	chr1	-	46560233	46560277	46557406	46558800	46561477	46561642	0	0	2	0	1	2	0	2	0	0	2	0	93	49	0.027624169	0.170760496	NA	0	0.345	NA	0.345	NA	-0.172
SE	472	DOCK8	chr9	+	372184	372286	371427	371566	376209	376305	0	10	33	0	0	0	0	9	0	0	1	0	98	49	0.027770234	0.170760496	NA	1	1	NA	0.818	NA	0.182
SE	20	FLNA	chrX	-	154362417	154362578	154362241	154362332	154362660	154362784	0	5	37	0	0	0	2	18	1	0	2	0	98	49	0.027864407	0.170760496	NA	1	1	1	0.818	1	0.061
SE	5181	ANKRD13A	chr12	+	110024045	110024112	110019138	110019328	110025741	110025823	0	6	10	0	0	0	0	4	0	0	1	0	98	49	0.027908407	0.170760496	NA	1	1	NA	0.667	NA	0.333
SE	6868	ATG2A	chr11	-	64909270	64909367	64907747	64907890	64909680	64909924	0	0	6	0	0	0	0	7	4	0	0	1	98	49	0.027908407	0.170760496	NA	NA	1	NA	1	0.667	0.166
SE	4661	AMICA1	chr11	-	118205881	118205991	118203427	118203665	118210486	118210712	0	1	9	0	0	0	0	21	0	0	2	0	98	49	0.028047628	0.170760496	NA	1	1	NA	0.84	NA	0.16
SE	5195	PICALM	chr11	-	85990249	85990399	85983865	85983973	85996825	85996929	0	1	25	0	0	21	0	21	0	0	2	0	98	49	0.028047628	0.170760496	NA	1	0.373	NA	0.84	NA	-0.153
SE	13787	PSD4	chr2	+	113198739	113198884	113197746	113197913	113199082	113199226	0	5	2	0	0	0	0	7	1	0	3	0	98	49	0.028063223	0.170760496	NA	1	1	NA	0.538	1	0.231
SE	16	FLNA	chrX	-	154352552	154352675	154352180	154352447	154352771	154352924	0	9	44	0	0	1	0	9	0	0	1	0	98	49	0.028070044	0.170760496	NA	1	0.957	NA	0.818	NA	0.16
SE	688	PTK2B	chr8	+	27397547	27397788	27325478	27325681	27419894	27420073	0	7	27	0	0	0	0	6	0	0	1	0	98	49	0.028077309	0.170760496	NA	1	1	NA	0.75	NA	0.25
SE	8356	ARHGAP30	chr1	-	161052625	161052797	161052439	161052543	161053257	161053385	0	7	26	0	0	0	0	6	0	0	1	0	98	49	0.028077309	0.170760496	NA	1	1	NA	0.75	NA	0.25
SE	12701	ARFGEF1	chr8	-	67275975	67276109	67271701	67271936	67277281	67277457	0	5	2	0	0	0	7	2	0	0	1	0	98	49	0.028306385	0.170760496	NA	1	1	1	0.5	NA	0.25
SE	4456	CLEC7A	chr12	-	10123244	10123363	10116780	10118590	10125086	10125173	0	0	38	0	2	1	0	15	3	0	0	0	98	49	0.028599911	0.170760496	NA	0	0.95	NA	1	1	-0.525
A5SS	672	STX16	chr20	+	58651252	58652138	58651252	58652087	58667489	58667597	0	4	7	0	0	3	1	2	0	0	1	0	98	49	0.029221508	0.170414363	NA	1	0.538	1	0.5	NA	0.019
SE	5228	NFKBIA	chr14	-	35402770	35402859	35402393	35402663	35403149	35403360	0	10	63	0	0	0	7	167	19	0	4	0	98	49	0.029442582	0.170760496	NA	1	1	1	0.954	1	0.015
SE	5000	WIPI2	chr7	+	5193117	5193345	5190203	5190493	5199575	5199658	0	1	1	0	0	1	0	0	0	0	2	2	98	49	0.029568334	0.170760496	NA	1	0.333	NA	0	0	0.666
SE	4834	ULK3	chr15	-	74838265	74838344	74838151	74838192	74838439	74838504	0	5	0	0	0	0	0	1	0	0	1	0	98	49	0.029576123	0.170760496	NA	1	NA	NA	0.333	NA	0.667
SE	4835	ULK3	chr15	-	74838269	74838344	74838151	74838192	74838439	74838504	0	5	0	0	0	0	0	1	0	0	1	0	98	49	0.029576123	0.170760496	NA	1	NA	NA	0.333	NA	0.667
SE	7841	B4GALT5	chr20	-	49639677	49639800	49637340	49637442	49640477	49640665	0	5	13	0	0	1	0	6	0	0	1	0	98	49	0.029628726	0.170760496	NA	1	0.867	NA	0.75	NA	0.183
SE	11675	ULK1	chr12	+	131915334	131915421	131915082	131915231	131915890	131916159	0	0	5	0	0	0	0	3	6	0	0	1	98	49	0.029628726	0.170760496	NA	NA	1	NA	1	0.75	0.125
SE	6193	FCER1G	chr1	+	161217985	161218077	161215296	161215370	161218240	161218276	0	7	28	0	0	0	4	10	0	0	1	0	98	49	0.029643012	0.170760496	NA	1	1	1	0.833	NA	0.084
A5SS	1841	OS9	chr12	+	57718145	57718421	57718145	57718376	57718992	57719182	0	6	6	0	0	0	3	8	0	0	1	0	94	49	0.029835799	0.170414363	NA	1	1	1	0.807	NA	0.097
SE	11769	FLII	chr17	-	18255182	18255263	18254768	18254854	18256525	18256597	0	6	12	0	0	0	0	9	0	0	1	0	98	49	0.03004548	0.170760496	NA	1	1	NA	0.818	NA	0.182
A3SS	3631	SURF4	chr9	-	133367258	133367543	133367258	133367445	133375921	133376166	0	0	0	0	1	0	1	2	0	0	1	0	98	49	0.030221083	0.197717869	NA	0	NA	1	0.5	NA	-0.75
SE	388	ZFAND5	chr9	-	72363469	72363606	72360627	72360787	72364695	72365247	0	0	4	0	1	0	0	2	0	0	1	0	98	49	0.030221083	0.170760496	NA	0	1	NA	0.5	NA	0
SE	11350	ADGRE2	chr19	-	14755653	14755877	14752328	14752526	14756237	14756345	0	0	2	0	1	1	0	2	0	0	1	0	98	49	0.030221083	0.170760496	NA	0	0.5	NA	0.5	NA	-0.25
SE	5198	PICALM	chr11	-	86026291	86026367	86022366	86022469	86031468	86031611	0	1	33	0	0	0	3	24	1	0	3	0	98	49	0.03042033	0.170760496	NA	1	1	1	0.8	1	0.067
SE	1451	BAG6	chr6	-	31648910	31648964	31648676	31648751	31649198	31649395	0	3	2	0	0	0	0	2	0	0	1	0	98	49	0.030496725	0.170760496	NA	1	1	NA	0.5	NA	0.5
SE	8360	ARHGAP30	chr1	-	161059613	161059716	161056387	161056532	161069527	161069970	0	1	23	0	0	0	0	8	0	0	1	1	98	49	0.030607676	0.170760496	NA	1	1	NA	0.8	0	0.6
SE	143	NONO	chrX	+	71297835	71297938	71297376	71297461	71298468	71298508	0	3	2	0	0	0	0	3	0	0	1	0	98	49	0.030693475	0.170760496	NA	1	1	NA	0.6	NA	0.4
SE	9279	PIK3CD	chr1	+	9723968	9724092	9723124	9723292	9724275	9724421	0	3	12	0	0	0	0	3	0	0	1	0	98	49	0.030693475	0.170760496	NA	1	1	NA	0.6	NA	0.4
SE	9304	SNRNP200	chr2	-	96298283	96298420	96297636	96297720	96298602	96298702	0	3	3	0	0	0	0	3	0	0	1	0	98	49	0.030693475	0.170760496	NA	1	1	NA	0.6	NA	0.4
SE	6670	FGR	chr1	-	27616856	27617006	27615688	27615844	27621558	27621657	0	2	21	0	0	0	0	9	0	0	1	1	98	49	0.030846049	0.170760496	NA	1	1	NA	0.818	0	0.591
A3SS	2290	SF1	chr11	-	64764603	64766155	64764603	64765518	64766899	64767079	0	3	7	0	0	0	0	4	0	0	1	0	98	49	0.03121249	0.197717869	NA	1	1	NA	0.667	NA	0.333
SE	6445	RALGDS	chr9	-	133102475	133102571	133101937	133102139	133102778	133102900	0	3	0	0	0	0	0	4	0	0	1	0	98	49	0.03121249	0.170760496	NA	1	NA	NA	0.667	NA	0.333
SE	54	ELF4	chrX	-	130071039	130071232	130069299	130069677	130071335	130071419	0	3	7	0	0	0	0	1	0	0	1	0	98	49	0.031636388	0.170760496	NA	1	1	NA	0.333	NA	0.667
SE	8271	ADAM9	chr8	+	39025802	39025884	39023155	39023325	39026676	39026810	0	3	4	0	0	0	0	5	0	0	1	0	98	49	0.031777706	0.170760496	NA	1	1	NA	0.714	NA	0.286
SE	11767	KDM5A	chr12	-	354075	354234	352204	352324	355157	355249	0	3	7	0	0	0	1	5	0	0	1	0	98	49	0.031777706	0.170760496	NA	1	1	1	0.714	NA	0.143
SE	12649	TLN1	chr9	-	35722860	35722921	35722118	35722223	35723951	35724079	0	3	26	0	0	0	2	5	0	0	1	0	98	49	0.031777706	0.170760496	NA	1	1	1	0.714	NA	0.143
SE	13848	GNLY	chr2	+	85697505	85697677	85695957	85696056	85698563	85698851	0	3	2	0	0	0	0	5	0	0	1	0	98	49	0.031777706	0.170760496	NA	1	1	NA	0.714	NA	0.286
SE	8338	CHD2	chr15	+	92901166	92901299	92900320	92900824	92924320	92924552	0	4	13	0	0	2	0	8	0	0	1	0	98	49	0.031803979	0.170760496	NA	1	0.765	NA	0.8	NA	0.083
SE	11875	MGAM	chr7	+	142036168	142036285	142034669	142034841	142038530	142038615	0	2	18	0	0	0	2	5	1	0	4	0	98	49	0.032046501	0.170760496	NA	1	1	1	0.385	1	0.205
A5SS	2853	CD37	chr19	+	49335419	49335609	49335419	49335575	49335713	49335786	0	3	10	0	0	0	0	4	0	0	1	0	83	49	0.032094074	0.170414363	NA	1	1	NA	0.703	NA	0.297
SE	2478	GSK3B	chr3	-	119912703	119912810	119905754	119905852	119916043	119916174	0	2	8	0	0	2	0	2	0	0	1	0	98	49	0.032484896	0.170760496	NA	1	0.667	NA	0.5	NA	0.334
SE	6900	PSME3	chr17	+	42838691	42838799	42838092	42838205	42838944	42839012	0	2	1	0	0	0	0	2	0	0	1	0	98	49	0.032484896	0.170760496	NA	1	1	NA	0.5	NA	0.5
SE	9190	GIT2	chr12	-	109980951	109981046	109967457	109967503	109983372	109983503	0	2	20	0	0	0	0	2	0	0	1	0	98	49	0.032484896	0.170760496	NA	1	1	NA	0.5	NA	0.5
SE	13501	HADHB	chr2	+	26285406	26285571	26284882	26284957	26289917	26290465	0	2	11	0	0	0	0	2	0	0	1	0	98	49	0.032484896	0.170760496	NA	1	1	NA	0.5	NA	0.5
SE	13782	SLC20A1	chr2	+	112647323	112647464	112646562	112647162	112647652	112647738	0	2	0	0	0	0	0	2	0	0	1	0	98	49	0.032484896	0.170760496	NA	1	NA	NA	0.5	NA	0.5
SE	4966	DDX3X	chrX	+	41347311	41347451	41346858	41347012	41347639	41347915	0	1	19	0	0	0	0	34	1	0	3	0	98	49	0.032593127	0.170760496	NA	1	1	NA	0.85	1	0.075
SE	5243	PLXNC1	chr12	+	94298631	94298795	94297315	94297423	94300909	94301057	0	4	20	0	0	0	1	10	0	0	1	0	98	49	0.03278556	0.170760496	NA	1	1	1	0.833	NA	0.084
A5SS	1502	MADD	chr11	+	47284940	47285194	47284940	47285065	47285450	47285590	0	2	5	0	0	3	0	3	0	0	1	0	98	49	0.032835937	0.170414363	NA	1	1	1	0.6	NA	0.2
SE	1141	IGF2R	chr6	+	160064803	160064901	160064400	160064531	160068248	160068385	0	2	17	0	0	3	0	3	0	0	1	0	98	49	0.032835937	0.170760496	NA	1	0.739	NA	0.6	NA	0.269
SE	2318	FAM105A	chr5	+	14601350	14601442	14601212	14601244	14602182	14602332	0	2	3	0	0	2	0	3	0	0	1	0	98	49	0.032835937	0.170760496	NA	1	0.429	NA	0.6	NA	0.115
SE	2543	GAK	chr4	-	877614	877809	877089	877207	881906	882040	0	2	6	0	0	0	0	3	0	0	1	0	98	49	0.032835937	0.170760496	NA	1	1	NA	0.6	NA	0.4
SE	11659	NLRP1	chr17	-	5536850	5536940	5533896	5533988	5539414	5539585	0	2	30	0	0	0	0	3	0	0	1	0	98	49	0.032835937	0.170760496	NA	1	1	NA	0.6	NA	0.4
SE	652	KAT6A	chr8	-	41942792	41943000	41940841	41941444	41943747	41943979	0	3	9	0	0	1	0	7	0	0	1	0	98	49	0.032969828	0.170760496	NA	1	0.818	NA	0.778	NA	0.131
SE	1651	CANX	chr5	+	179708977	179709059	179708238	179708380	179709872	179710065	1	3	6	0	0	0	0	7	0	0	1	0	98	49	0.032969828	0.170760496	1	1	1	NA	0.778	NA	0.222
A3SS	3558	TMEM127	chr2	-	96265137	96265512	96265137	96265489	96265868	96266013	0	4	2	0	3	4	0	6	1	0	0	0	72	49	0.033183051	0.197717869	NA	0.476	0.254	NA	1	1	-0.635
SE	13035	VASP	chr19	+	45522339	45522581	45522167	45522217	45522717	45522818	0	4	13	0	0	0	0	11	0	0	1	0	98	49	0.033237301	0.170760496	NA	1	1	NA	0.846	NA	0.154
SE	10971	ARHGAP15	chr2	+	143436912	143437042	143435600	143435699	143487372	143487495	0	0	19	0	2	0	0	21	2	0	0	0	98	49	0.033416613	0.170760496	NA	0	1	NA	1	1	-0.5
SE	2607	RBM5	chr3	+	50104247	50104308	50103082	50103166	50105076	50105142	0	2	6	0	0	1	0	1	0	3	1	0	98	49	0.033473796	0.170760496	NA	1	0.75	0	0.333	NA	0.709
SE	7140	KIF21B	chr1	-	201004765	201004933	201004339	201004455	201005307	201005442	0	2	3	0	0	0	0	1	0	0	1	0	98	49	0.033473796	0.170760496	NA	1	1	NA	0.333	NA	0.667
SE	7425	H2AFY	chr5	-	135352945	135353045	135345967	135346057	135360496	135360604	0	2	10	0	0	0	0	1	0	0	1	0	98	49	0.033473796	0.170760496	NA	1	1	NA	0.333	NA	0.667
SE	7587	DHX9	chr1	+	182855576	182855713	182854029	182854178	182856531	182856578	0	2	1	0	0	5	0	1	0	0	1	0	98	49	0.033473796	0.170760496	NA	1	0.091	NA	0.333	NA	0.212
SE	9970	SCYL3	chr1	-	169870254	169870357	169868927	169869039	169873695	169873752	0	2	1	0	0	0	0	1	0	0	1	0	98	49	0.033473796	0.170760496	NA	1	1	NA	0.333	NA	0.667
SE	12042	TMC8	chr17	+	78137234	78137358	78134869	78135009	78137716	78137814	0	2	4	0	0	0	0	1	0	0	1	0	98	49	0.033473796	0.170760496	NA	1	1	NA	0.333	NA	0.667
SE	12085	DCAF8	chr1	-	160225061	160225119	160224441	160224549	160225590	160225663	0	2	9	0	0	0	2	1	0	0	1	0	98	49	0.033473796	0.170760496	NA	1	1	1	0.333	NA	0.334
SE	12285	ST6GALNAC2	chr17	-	76578755	76578816	76574364	76574539	76585683	76586063	0	2	2	0	0	4	0	1	0	0	1	0	98	49	0.033473796	0.170760496	NA	1	0.2	NA	0.333	NA	0.267
SE	12756	ADGRE5	chr19	+	14397076	14397223	14396341	14396473	14397657	14397804	0	2	8	0	0	0	1	1	0	5	1	0	98	49	0.033473796	0.170760496	NA	1	1	0.091	0.333	NA	0.788
SE	13958	DIP2B	chr12	+	50692948	50693013	50691048	50691151	50695266	50695360	0	2	5	0	0	0	0	1	0	0	1	0	98	49	0.033473796	0.170760496	NA	1	1	NA	0.333	NA	0.667
SE	10454	THBS1	chr15	+	39588525	39588699	39588041	39588218	39588958	39589086	0	2	8	0	0	4	0	4	0	0	1	0	98	49	0.033475981	0.170760496	NA	1	0.5	NA	0.667	NA	0.083
SE	13548	STRN	chr2	-	36855211	36855352	36850999	36851107	36857855	36858023	0	2	0	0	0	0	0	4	0	0	1	0	98	49	0.033475981	0.170760496	NA	1	NA	NA	0.667	NA	0.333
RI	452	BIN2	chr12	-	51302019	51302780	51302019	51302115	51302685	51302780	0	0	1	0	4	5	1	1	0	0	4	0	98	49	0.033604201	0.297702651	NA	0	0.091	1	0.111	NA	-0.51
SE	8340	CHD2	chr15	+	92945820	92945865	92942842	92943068	92946037	92946216	0	2	8	0	0	0	0	4	0	0	1	0	94	49	0.033727313	0.170760496	NA	1	1	NA	0.676	NA	0.324
SE	4559	SLC6A6	chr3	+	14457949	14458082	14447581	14447816	14466515	14466650	0	3	36	0	0	0	0	9	2	0	3	0	98	49	0.033971646	0.170760496	NA	1	1	NA	0.6	1	0.2
SE	2911	PPP6C	chr9	-	125153532	125153742	125146572	125149921	125153905	125153985	0	3	7	0	0	0	0	9	0	0	1	0	98	49	0.034107533	0.170760496	NA	1	1	NA	0.818	NA	0.182
SE	8654	SVIL	chr10	-	29555050	29555108	29554782	29554934	29563200	29563292	0	3	13	0	0	0	0	9	0	0	1	0	98	49	0.034107533	0.170760496	NA	1	1	NA	0.818	NA	0.182
SE	5187	MYO9B	chr19	+	17192745	17193062	17191096	17191219	17194555	17195473	0	2	5	0	0	0	0	5	0	0	1	0	98	49	0.03419934	0.170760496	NA	1	1	NA	0.714	NA	0.286
SE	1137	IGF2R	chr6	+	160058022	160058124	160056423	160056525	160058905	160059098	0	2	35	0	0	0	4	21	1	0	2	0	98	49	0.034257364	0.170760496	NA	1	1	1	0.84	1	0.053
SE	6669	FGR	chr1	-	27614429	27614583	27613218	27613350	27614849	27614926	0	25	47	0	0	0	2	22	2	0	1	0	98	49	0.034275909	0.170760496	NA	1	1	1	0.917	1	0.028
SE	8631	SMG7	chr1	+	183512836	183512868	183472370	183472649	183515873	183515991	0	0	2	0	0	0	0	1	1	0	0	1	81	49	0.034410483	0.170760496	NA	NA	1	NA	1	0.377	0.311
SE	4362	LRRK2	chr12	+	40305784	40305966	40303947	40304134	40308466	40308696	0	2	32	0	0	0	6	23	1	0	2	0	98	49	0.034647673	0.170760496	NA	1	1	1	0.852	1	0.049

SE	691	DOCK5	chr8	+	25300575	25300657	25298943	25299101	25302324	25302454	0	3	24	0	0	0	0	10	0	0	1	0	98	49	0.03465568	0.170760496	NA		1		1	NA		0.833	NA		0.167
A3SS	806	TYMP	chr22	-	50529495	50529754	50529495	50529731	50529903	50530085	0	2	4	0	0	1	3	3	0	0	1	0	72	49	0.034683351	0.197717869	NA		1		0.731		1	0.671	NA		0.03
SE	4962	DDX3X	chrX	+	41339035	41339083	41337407	41337465	41341483	41341616	0	2	15	0	0	0	0	19	3	0	3	0	97	49	0.034732632	0.170760496	NA		1		1	NA		0.762		1	0.119
SE	9028	MYD88	chr3	+	38139863	38139998	38138477	38139028	38140756	38140848	0	6	12	0	0	0	0	11	3	0	2	0	98	49	0.03475926	0.170760496	NA		1		1	NA		0.733		1	0.133
A3SS	2212	GNAS	chr20	+	58903527	58903585	58903530	58903585	58895611	58895684	0	3	4	0	0	2	0	4	0	0	1	0	52	49	0.034903266	0.197717869	NA		1		0.653	NA		0.79	NA		0.036
SE	3591	TRIP12	chr2	-	229778875	229778990	229778432	229778587	229785756	229785855	0	2	11	0	0	0	0	6	0	0	1	0	98	49	0.034934183	0.170760496	NA		1		1	NA		0.75	NA		0.25
SE	863	NAMPT	chr7	-	106268463	106268600	106263391	106263617	106269153	106269312	0	9	124	0	0	0	0	88	7	0	2	0	98	49	0.035105524	0.170760496	NA		1		1	NA		0.957		1	0.022
SE	7287	HDAC5	chr17	-	44078794	44078879	44078499	44078665	44079143	44079277	0	1	2	0	0	0	0	5	1	0	3	0	98	49	0.035115165	0.170760496	NA		1		1	NA		0.455		1	0.272
SE	9359	FERMT3	chr11	+	64219523	64219658	64219250	64219358	64219739	64219789	0	3	13	0	0	0	0	11	0	0	1	0	98	49	0.03520334	0.170760496	NA		1		1	NA		0.846	NA		0.154
SE	7828	SRGN	chr10	+	69097083	69097231	69088071	69088236	69103870	69104811	0	28	397	0	0	0	31	320	22	0	2	0	98	49	0.035427964	0.170760496	NA		1		1		1	0.988		1	0.004
SE	8959	N4BP2L2	chr13	-	32527407	32527532	32522181	32522270	32535768	32537027	0	2	9	0	0	1	0	7	0	0	1	0	98	49	0.0356372	0.170760496	NA		1		0.818	NA		0.778	NA		0.131
A3SS	1851	OAS1	chr12	+	112919388	112919907	112919486	112919907	112917546	112917700	0	3	5	0	0	4	0	15	2	0	0	0	98	49	0.035922391	0.197717869	NA		1		0.385	NA		1		1	-0.307
A5SS	478	UBE2D3	chr4	-	102827426	102827948	102827591	102827948	102826484	102826636	0	2	2	0	0	2	1	9	3	0	0	0	98	49	0.036267739	0.170414363	NA		1		0.333		1	1		1	-0.334
SE	14036	CFLAR	chr2	+	201135971	201136107	201132964	201133134	201140356	201140439	1	6	52	0	0	0	0	29	0	0	1	0	98	49	0.03628875	0.170760496		1	1		1	NA		0.935	NA		0.065
SE	1368	RGL2	chr6	-	33294687	33294762	33294033	33294066	33294976	33295045	0	2	17	0	0	0	2	8	0	0	1	0	98	49	0.036328243	0.170760496	NA		1		1		1	0.8	NA		0.1
SE	4793	CASP1	chr11	-	105026225	105026356	105025509	105025600	105026841	105026951	0	2	29	0	0	0	0	8	0	1	1	0	98	49	0.036328243	0.170760496	NA		1		1		0	0.8	NA		0.6
SE	6901	PSME3	chr17	+	42838730	42838799	42838092	42838205	42838944	42839012	0	2	5	0	0	0	0	8	0	0	1	0	98	49	0.036328243	0.170760496	NA		1		1	NA		0.8	NA		0.2
SE	338	DNAJC25	chr9	+	111642856	111643094	111631351	111631743	111647106	111647259	0	1	1	0	0	0	0	2	0	0	1	0	98	49	0.036515846	0.170760496	NA		1		1	NA		0.5	NA		0.5
SE	2653	CCDC12	chr3	-	46923606	46923668	46923328	46923363	46925455	46925535	0	1	1	0	0	0	0	2	0	0	1	0	98	49	0.036515846	0.170760496	NA		1		1	NA		0.5	NA		0.5
SE	3010	MED15	chr22	+	20584854	20585015	20584358	20584425	20585100	20585267	0	1	1	0	0	0	0	2	0	0	1	0	98	49	0.036515846	0.170760496	NA		1		1	NA		0.5	NA		0.5
SE	6389	SUPT6H	chr17	+	28690920	28691063	28690081	28690229	28693695	28693836	0	1	2	0	0	0	0	2	0	0	1	0	98	49	0.036515846	0.170760496	NA		1		1	NA		0.5	NA		0.5
SE	6793	PLEKHM2	chr1	+	15719733	15719920	15718537	15718625	15721328	15721388	0	1	2	0	0	0	0	2	0	0	1	0	98	49	0.036515846	0.170760496	NA		1		1	NA		0.5	NA		0.5
SE	7794	G6PD	chrX	-	154532941	154533128	154532566	154532802	154533575	154533669	0	1	4	0	0	0	0	2	0	0	1	0	98	49	0.036515846	0.170760496	NA		1		1	NA		0.5	NA		0.5
SE	8149	TTC39C	chr18	+	24069156	24069271	24066011	24066140	24080584	24080939	0	1	2	0	0	0	0	2	0	0	1	0	98	49	0.036515846	0.170760496	NA		1		1	NA		0.5	NA		0.5
SE	8182	LSM14A	chr19	+	34215123	34215300	34208928	34209051	34215595	34215661	0	1	7	0	0	0	0	2	0	0	1	0	98	49	0.036515846	0.170760496	NA		1		1	NA		0.5	NA		0.5
SE	9120	CR1	chr1	+	207609289	207609688	207607250	207607336	207611676	207611853	0	1	20	0	0	0	0	2	0	0	1	0	98	49	0.036515846	0.170760496	NA		1		1	NA		0.5	NA		0.5
SE	9391	ZNF346	chr5	+	177023159	177023234	177022679	177022913	177041125	177041229	0	1	0	0	0	0	0	2	0	0	1	0	98	49	0.036515846	0.170760496	NA		1	NA		NA		0.5	NA		0.5
SE	10262	SNX17	chr2	+	27372622	27372740	27371268	27371343	27373039	27373113	0	1	3	0	0	0	0	2	0	0	1	0	98	49	0.036515846	0.170760496	NA		1		1	NA		0.5	NA		0.5
SE	10380	NFKBIE	chr6	-	44262559	44262662	44261625	44261848	44264981	44265788	0	1	2	0	0	0	0	2	0	0	1	0	98	49	0.036515846	0.170760496	NA		1		1	NA		0.5	NA		0.5
SE	12595	GBF1	chr10	+	102363255	102363396	102362474	102362664	102363709	102363798	0	1	0	0	0	0	0	2	0	0	1	0	98	49	0.036515846	0.170760496	NA		1	NA		NA		0.5	NA		0.5
SE	12839	DENND1B	chr1	-	197647054	197647114	197645689	197645743	197652234	197652315	0	1	0	0	0	0	0	2	0	0	1	0	98	49	0.036515846	0.170760496	NA		1	NA		NA		0.5	NA		0.5
SE	13738	SERPINA1	chr14	-	94388818	94389010	94388559	94388663	94390456	94390692	0	1	17	0	0	4	0	2	0	0	1	0	98	49	0.036515846	0.170760496	NA		1		0.68	NA		0.5	NA		0.34
SE	347	OGT	chrX	+	71561774	71561900	71559587	71559677	71562846	71563017	0	3	24	0	0	0	5	14	0	0	1	0	98	49	0.036639257	0.170760496	NA		1		1		1	0.875	NA		0.062
SE	7594	ATG16L2	chr11	+	72826517	72826589	72826172	72826243	72826702	72826823	0	12	63	0	0	0	11	29	8	0	2	0	98	49	0.036924565	0.170760496	NA		1		1		1	0.879		1	0.04
SE	3590	TRIP12	chr2	-	229771518	229771632	229769230	229769325	229774096	229774261	0	2	2	0	0	0	0	9	0	0	1	0	98	49	0.037002078	0.170760496	NA		1		1	NA		0.818	NA		0.182
SE	8756	ZC3H11A	chr1	+	203848330	203848407	203847183	203847687	203849710	203850026	0	2	10	0	0	0	0	9	0	0	1	0	98	49	0.037002078	0.170760496	NA		1		1	NA		0.818	NA		0.182
SE	279	EXOSC2	chr9	+	130698161	130698251	130697581	130697627	130699328	130699394	0	1	0	0	0	0	0	1	0	0	1	0	98	49	0.037136978	0.170760496	NA		1	NA		NA		0.333	NA		0.667
SE	3510	HDLBP	chr2	-	241255373	241255580	241255050	241255158	241256183	241256399	0	1	1	0	0	0	0	1	0	0	1	0	98	49	0.037136978	0.170760496	NA		1		1	NA		0.333	NA		0.667
SE	4017	AGAP2	chr12	-	57731808	57731967	57731555	57731642	57732402	57732512	0	1	1	0	0	0	4	1	0	0	1	0	98	49	0.037136978	0.170760496	NA		1		1		1	0.333	NA		0.334
SE	4033	TRAFD1	chr12	+	112151800	112152140	112149750	112149871	112152426	112152499	0	1	5	0	0	0	0	1	0	0	1	0	98	49	0.037136978	0.170760496	NA		1		1	NA		0.333	NA		0.667
SE	5121	CCM2	chr7	+	45038252	45038426	45027633	45027797	45063917	45064001	0	1	0	0	0	0	0	1	0	0	1	0	98	49	0.037136978	0.170760496	NA		1	NA		NA		0.333	NA		0.667
SE	6443	ATP11A	chr13	+	112858144	112858290	112857817	112857920	112859392	112859452	0	1	1	0	0	1	0	1	0	0	1	0	98	49	0.037136978	0.170760496	NA		1		0.333	NA		0.333	NA		0.333
SE	6888	SMC3	chr10	+	110590990	110591132	110590411	110590572	110593072	110593223	0	1	3	0	0	0	0	1	0	0	1	0	98	49	0.037136978	0.170760496	NA		1		1	NA		0.333	NA		0.667
SE	10311	KLC1	chr14	+	103657545	103657776	103654563	103654825	103662115	103662194	0	1	8	0	0	0	0	1	0	0	1	0	98	49	0.037136978	0.170760496	NA		1		1	NA		0.333	NA		0.667
SE	11210	GYPC	chr2	+	126690254	126690311	126655934	126656312	126693863	126693947	0	1	0	0	0	0	0	1	0	0	1	0	98	49	0.037136978	0.170760496	NA		1	NA		NA		0.333	NA		0.667
SE	13735	SERPINA1	chr14	-	94388800	94389010	94388559	94388663	94390456	94390692	0	1	17	0	0	4	0	1	0	0	1	0	98	49	0.037136978	0.170760496	NA		1		0.68	NA		0.333	NA		0.507
SE	13855	FBXW11	chr5	-	171914342	171914405	171910571	171910797	172006457	172006873	0	0	1	0	0	0	0	1	1	0	0	1	98	49	0.037136978	0.170760496	NA	NA			1	NA		1		0.333	0.334
SE	1807	ASAP1	chr8	-	130091972	130092143	130079901	130079971	130112093	130112322	0	1	0	0	0	1	0	3	0	0	1	0	98	49	0.03722674	0.170760496	NA		1		0	NA		0.6	NA		-0.1
SE	3240	ZFAS1	chr20	+	49280902	49280964	49280484	49280570	49289044	49289258	0	1	12	0	0	1	0	3	0	0	1	0	98	49	0.03722674	0.170760496	NA		1		0.857	NA		0.6	NA		0.329
SE	5144	KIAA0226L	chr13	-	46371940	46372210	46368732	46368815	46387133	46387500	0	1	1	0	0	0	0	3	0	0	1	0	98	49	0.03722674	0.170760496	NA		1		1	NA		0.6	NA		0.4
SE	5505	SBF2	chr11	-	10031047	10031170	10029764	10029875	10042843	10042981	0	1	12	0	0	0	0	3	0	0	1	0	98	49	0.03722674	0.170760496	NA		1		1	NA		0.6	NA		0.4
SE	6391	KMT2B	chr19	+	35736689	35736827	35733762	35733872	35736911	35736986	0	1	1	0	0	0	0	3	0	0	1	0	98	49	0.03722674	0.170760496	NA		1		1	NA		0.6	NA		0.4
SE	8166	ARL5B	chr10	+	18672621	18672705	18668529	18668677	18673983	18674135	0	1	5	0	0	0	0	3	0	0	1	0	98	49	0.03722674	0.170760496	NA		1		1	NA		0.6	NA		0.4
SE	9178	SH3BP1	chr22	+	37644869	37644960	37644636	37644705	37645364	37645510	0	1	4	0	0	0	2	3	0	0	1	0	98	49	0.03722674	0.170760496	NA		1		1		1	0.6	NA		0.2
SE	10631	VPS13C	chr15	-	61951823	61951980	61950944	61951024	61954420	61954554	0	1	3	0	0	0	0	3	0	0	1	0	98	49	0.03722674	0.170760496	NA		1		1	NA		0.6	NA		0.4
SE	10953	FES	chr15	+	90889516	90889636	90889305	90889443	90889839	90889962	0	1	7	0	0	0	0	3	0	0	1	0	98	49	0.03722674	0.170760496	NA		1		1	NA		0.6	NA		0.4
SE	11935	STK40	chr1	-	36367847	36368084	36361220	36361340	36385722	36385927	0	1	1	0	0	0	0	3	0	0	1	0	98	49	0.03722674	0.170760496	NA		1		1	NA		0.6	NA		0.4
SE	13687	SIRPA	chr20	+	1924763	1924877	1915098	1915455	1927874	1927899	0	1	10	0	0	0	0	3	0	0	1	0	98	49	0.03722674	0.170760496	NA		1		1	NA		0.6	NA		0.4
SE	331	AKNA	chr9	-	114343707	114343803	114342008	114342125	114345862	114346009	0	0	10	0	2	2	0	1	0	0	1	0	98	49	0.037261148	0.170760496	NA		0		0.714	NA		0.333	NA		0.024
SE	6388	SUPT6H	chr17	+	28682941	28683092	28682726	28682856	28683267	28683422	0	0	1	0	2	0	0	1	0	0	1	0	98	49	0.037261148	0.170760496	NA		0		1	NA		0.333	NA		0.167
SE	7325	SREBF1	chr17	-	17814614	17814747	17814244	17814410	17814834	17814944	0	0	0	0	2	0	0	1	0	0	1	0	98	49	0.037261148	0.170760496	NA		0	NA		NA		0.333	NA		-0.333
SE	559	PABPC1	chr8	-	100718086	100718280	100717772	100717888	100721390	100722087	0	0	29	0	1	0	1	19	6	0	0	0	98	49	0.037496585	0.170760496	NA		0		1		1	1		1	-0.5
SE	371	AGTPBP1	chr9	-	85678334	85678398	85677435	85677582	85681267	85681335	0	2	31	0	0	0	0	10	0	0	1	0	98	49	0.037649757	0.170760496	NA		1		1	NA		0.833	NA		0.167
SE	5702	PPP4C	chr16	+	30083654	30083781	30083393	30083567	30084665	30084855	0	2	14	0	0	0	0	10	0	0	1	0	98	49	0.037649757	0.170760496	NA		1		1	NA		0.833	NA		0.167
SE	11023	TAP1	chr6	-	32848989	32849118	32848651	32848840	32850319	32850517	1	2	34	0	0	0	0	10	0	0	1	0	98	49	0.037649757	0.170760496		1	1		1	NA		0.833	NA		0.167
A3SS	1947	SNRPB	chr20	-	2461634	2461939	2461634	2461793	2462635	2462761	0	0	3	0	1	2	7	5	1	0	0	0	98	49	0.037878306	0.197717869	NA		0		0.429		1	1		1	-0.785
SE	370	AGTPBP1	chr9	-	85660935	85660973	85657434	85657643	85669484	85669578	0	1	9	0	0	0	0	3	0	0	1	0	87	49	0.038097905	0.170760496	NA		1		1	NA		0.628	NA		0.372
SE	5887	SIPA1L1	chr14	+	71650334	71650509	71624047	71624236	71658332	71658436	0	1	14	0	0	0	0	4	0	0	1	0	98	49	0.038206571	0.170760496	NA		1		1	NA		0.667	NA		0.333
SE	8614	TM9SF3	chr10	-	96533050	96533190	96530539	96530608	96544075	96544206	0	1	11	0	0	0	0	4	0	0	1	0	98	49	0.038206571	0.170760496	NA		1		1	NA		0.667	NA		0.333
SE	11155	NDE1	chr16	+	15694164	15694256	15691143	15691323	15696708	15696860	0	1	16	0	0	0	0	4	0	0	1	0	98	49	0.038206571	0.170760496	NA		1		1	NA		0.667	NA		0.333
SE	11295	CREBBP	chr16	-	3782683	3782926	3781203	3781306	3791980	3792094	0	1	6	0	0	0	0	4	0	0	1	0	98	49	0.038206571	0.170760496	NA		1		1	NA		0.667	NA		0.333
SE	12432	SPAG9	chr17	-	50974770	50974947	50970706	50970856	50977107	50977221	0	1	3	0	0	0	0	4	0	0	1	0	98	49	0.038206571	0.170760496	NA		1		1	NA		0.667	NA		0.333
SE	3199	TNIP1	chr5	-	151060317	151060395	151056765	151056957	151062126	151062212	0	2	8	0	0	0	0	11	0	0	1	0	98	49	0.038274566	0.170760496	NA		1		1	NA		0.846	NA		0.154
SE	349	NR4A3	chr9	+	99832688	99832818	99828040	99828993	99833281	99833454	0	1	3	0	0	0	0	7	1	0	2	0	98	49	0.038321227	0.170760496	NA		1		1	NA		0.636		1	0.182
SE	11639	ARRB2	chr17	+	4716146	4716191	4715972	4716033	4716411	4716608	0	6	32	0	0	1	0	13	1	0	5	2	94	49	0.038650672	0.170760496	NA		1		0.943	NA		0.575		0.207	0.581
SE	9931	PTPRE	chr10	+	128066074	128066194	128063082	128063180	128068122	128068286	0	4	51	0	0	0	0	47	9	0	3	0	98	49	0.038824113	0.170760496	NA		1		1	NA		0.887		1	0.056
SE	11065	CORO1A	chr16	+	30187941	30188087	30187724	30187829	30188191	30188249	0	18	59	0	0	0	5	24	3	0	1	0	98	49	0.038917622	0.170760496	NA		1		1		1	0.923		1	0.026
A5SS	3018	SERPINA1	chr14	-	94388800	94389010	94388818	94389010	94388559	94388663	0	1	6	0	0	2	0	2	0	0	1	0	67	49	0.039150683	0.170414363	NA		1		0.687	NA		0.594	NA		0.25
A3SS	3771	STRN4	chr19	-	46727451	46727567	46727451	46727546	46727893	46728007	0	1	1	0	0	4	0	1	0	0	1	0	70	49	0.039208323	0.197717869	NA		1		0.149	NA		0.412	NA		0.163
SE	2205	BOD1L1	chr4	-	13595859	13595944	13591922	13591966	13597103	13597168	0	1	12	0	0	0	0	5	0	0	1	0	98	49	0.039248064	0.170760496	NA		1		1	NA		0.714	NA		0.286
SE	2551	ATXN7	chr3	+	63913156	63913225	63912587	63912923	63952378	63952483	0	1	7	0	0	0	0	5	0	0	1	0	98	49	0.039248064	0.170760496	NA		1		1	NA		0.714	NA		0.286
SE	4158	SH3BP2	chr4	+	2831922	2831978	2831570	2831679	2832330	2832412	0	1	12	0	0	1	1	5	0	0	1	0	98	49	0.039248064	0.170760496	NA		1		0.857		1	0.714	NA		0.072
SE	4319	BIRC6	chr2	+	32441328	32441462	32439507	32439686	32442064	32442226	0	1	5	0	0	0	0	5	0	0	1	0	98	49	0.039248064	0.170760496	NA		1		1	NA		0.714	NA		0.286
SE	5146	KIAA0226L	chr13	-	46371940	46372597	46368732	46368815	46387133	46387500	0	1	1	0	0	0	0	5	0	0	1	0	98	49	0.039248064	0.170760496	NA		1		1	NA		0.714	NA		0.286
SE	5222	CLEC4E	chr12	-	8537114	8537266	8536089	8536205	8539216	8539306	0	1	35	0	0	0	0	5	0	0	1	0	98	49	0.039248064	0.170760496	NA		1		1	NA		0.714	NA		0.286
SE	5861	DISC1	chr1	+	231795241	231795296	231770834	231771070	231818328	231819244	0	1	7	0	0	0	0	5	0	0	1	0	98	49	0.039248064	0.170760496	NA		1		1	NA		0.714	NA		0.286
SE	6191	FOXN2	chr2	+	48328560	48328702	48314655	48314814	48346200	48346751	0	1	2	0	0	1	0	5	0	0	1	0	98	49	0.039248064	0.170760496	NA		1		0.5	NA		0.714	NA		0.036
SE	8721	NOD2	chr16	+	50707854	50707960	50699349	50699954	50710557	50712373	0	1	3	0	0	0	0	5	0	0	1	0	98	49	0.039248064	0.170760496	NA		1		1	NA		0.714	NA		0.286
SE	8817	KDM2A	chr11	+	67243008	67243092	67231565	67231960	67245188	67245458	0	1	11	0	0	0	2	5	0	0	1	0	98	49	0.039248064	0.170760496	NA		1		1		1	0.714	NA		0.143
SE	9383	PECAM1	chr17	-	64354932	64355040	64353490	64353518	64356110	64356398	0	1	38	0	0	8	0	5	0	0	1	0	98	49	0.039248064	0.170760496	NA		1		0.704	NA		0.714	NA		0.138
SE	11702	POLR2A	chr17	+	7496209	7496346	7495940	7496073	7496439	7496613	0	1	9	0	0	0	0	5	0	0	1	0	98	49	0.039248064	0.170760496	NA		1		1	NA		0.714	NA		0.286
SE	11979	TSEN34	chr19	+	54191720	54191964	54191213	54191607	54192115	54192373	0	5	17	0	0	1	0	8	4	0	3	1	98	49	0.039311924	0.170760496	NA		1		0.895	NA		0.571		0.667	0.329
SE	7521	AQP9	chr15	+	58173067	58173205	58166672	58166799	58179127	58179345	0	1	40	0	0	0	0	21	1	0	2	0	98	49	0.039675176	0.170760496	NA		1		1	NA		0.84		1	0.08
A3SS	462	BTN3A1	chr6	+	26412704	26415216	26413168	26415216	26411554	26411581	0	1	5	0	0	2	0	6	0	0	1	0	98	49	0.040279671	0.197717869	NA		1		0.556	NA		0.75	NA		0.028
SE	1761	FAM53C	chr5	+	138341808	138341866	138341183	138341413	138344824	138345609	0	1	7	0	0	0	0	6	0	0	1	0	98	49	0.040279671	0.170760496	NA		1		1	NA		0.75	NA		0.25
SE	3245	SULF2	chr20	-	47665198	47665293	47664129	47664189	47665856	47665953	0	1	14	0	0	0	0	6	0	0	1	0	98	49	0.040279671	0.170760496	NA		1		1	NA		0.75	NA		0.25
SE	8677	MANBA	chr4	-	102714437	102714561	102690595	102690771	102722870	102723041	0	1	4	0	0	0	0	6	0	0	1	0	98	49	0.040279671	0.170760496	NA		1		1	NA		0.75	NA		0.25
SE	12664	STXBP2	chr19	+	7643164	7643245	7642982	7643048	7644613	7644752	0	1	4	0	0	0	1	6	0	0	1	0	98	49	0.040279671	0.170760496	NA		1		1		1	0.75	NA		0.125
SE	19	FLNA	chrX	-	154357250	154357274	154354824	154355072	154357433	154357623	0	1	4	0	7	5	2	10	0	0	4	0	73	49	0.040614468	0.170760496	NA		0.087		0.349		1	0.627	NA		-0.596
SE	12838	PTTG1IP	chr21	-	44865394	44865447	44861162	44861271	44873501	44873903	0	2	4	0	0	0	0	3	1	0	2	0	98	49	0.040702408	0.170760496	NA		1		1	NA		0.429		1	0.285
SE	4624	SORL1	chr11	+	121543547	121543726	121532463	121532552	121545242	121545429	0	4	58	0	0	0	0	34	0	0	1	0	98	49	0.04109126	0.170760496	NA		1		1	NA		0.944	NA		0.056
SE	1140	IGF2R	chr6	+	160063414	160063630	160062531	160062619	160064400	160064531	0	2	31	0	0	0	0	16	0	0	1	0	98	49	0.041121367	0.170760496	NA		1		1	NA		0.889	NA		0.111
SE	5495	ADAM8	chr10	-	133269444	133269529	133267928	133268118	133269896	133269974	0	2	12	0	0	0	0	16	0	0	1	0	98	49	0.041121367	0.170760496	NA		1		1	NA		0.889	NA		0.111
SE	2907	RAF1	chr3	-	12600151	12600279	12599690	12599808	12600387	12600415	0	2	19	0	0	0	0	22	3	0	2	0	98	49	0.041304338	0.170760496	NA		1		1	NA		0.846		1	0.077
SE	5124	DNM2	chr19	+	10759737	10759811	10718052	10718403	10772478	10772628	0	11	23	0	0	0	0	12	3	0	1	0	98	49	0.041331042	0.170760496	NA		1		1	NA		0.857		1	0.072
A3SS	3207	ANKRD11	chr16	-	89316932	89317078	89316932	89317075	89418283	89418368	0	1	2	0	0	0	0	1	0	0	1	0	52	49	0.041336327	0.197717869	NA		1		1	NA		0.485	NA		0.515
SE	8601	PYGL	chr14	-	50912155	50912303	50911977	50912036	50913028	50913130	0	1	14	0	0	1	0	7	0	0	1	0	98	49	0.041336891	0.170760496	NA		1		0.875	NA		0.778	NA		0.159
SE	9493	SAP30BP	chr17	+	75668515	75668625	75667115	75667478	75671815	75671863	0	1	1	0	0	0	0	7	0	0	1	0	98	49	0.041336891	0.170760496	NA		1		1	NA		0.778	NA		0.222
SE	10364	SOS2	chr14	-	50158564	50158646	50156998	50157121	50159430	50160086	0	1	14	0	0	2	0	7	0	0	1	0	98	49	0.041336891	0.170760496	NA		1		0.778	NA		0.778	NA		0.111
SE	11078	TCEB2	chr16	-	2775450	2775556	2771413	2772102	2776992	2777127	0	1	12	0	0	0	1	7	0	0	1	0	98	49	0.041336891	0.170760496	NA		1		1		1	0.778	NA		0.111
SE	10594	RNF213	chr17	+	80390011	80390196	80389827	80389917	80393344	80398781	0	0	79	0	1	1	0	30	3	0	0	0	98	49	0.041591024	0.170760496	NA		0		0.975	NA		1		1	-0.512
SE	1662	FAM193B	chr5	-	177531952	177532200	177531281	177531521	177532442	177532641	0	2	0	0	0	1	0	0	1	0	0	2	98	49	0.041785228	0.170760496	NA		1		0	NA	NA			0.2	0.3
SE	9538	CAMK2G	chr10	-	73839538	73839601	73837467	73837511	73842168	73842211	0	0	0	0	1	4	0	1	0	3	2	0	98	49	0.041785228	0.170760496	NA		0		0		0	0.2	NA		-0.1
A5SS	2638	BPTF	chr17	+	67945408	67946325	67945408	67945896	67947725	67947808	0	0	0	0	1	1	0	1	0	0	1	0	98	49	0.041803869	0.170414363	NA		0		0	NA		0.333	NA		-0.333
RI	287	EDEM2	chr20	-	35146824	35147358	35146824	35146935	35147151	35147358	0	0	0	0	0	1	0	0	1	0	0	1	98	49	0.041803869	0.297702651	NA	NA			0	NA	NA			0.333	-0.333
SE	888	GIGYF1	chr7	-	100683549	100683632	100683303	100683444	100683817	100683918	0	0	0	0	1	0	0	1	0	0	1	0	98	49	0.041803869	0.170760496	NA		0	NA		NA		0.333	NA		-0.333
SE	6999	AGTRAP	chr1	+	11746126	11746223	11745802	11745837	11747439	11747545	0	0	0	0	1	2	0	1	0	0	1	0	98	49	0.041803869	0.170760496	NA		0		0	NA		0.333	NA		-0.333
SE	8430	GS1-124K5.11	chr7	-	66560578	66560675	66553467	66554837	66573384	66573550	0	0	1	0	1	0	0	1	0	0	1	0	98	49	0.041803869	0.170760496	NA		0		1	NA		0.333	NA		0.167
SE	9926	PPP4R3B	chr2	-	55578246	55578342	55577314	55577356	55579678	55579781	0	0	0	0	1	1	0	1	0	0	1	0	98	49	0.041803869	0.170760496	NA		0		0	NA		0.333	NA		-0.333
SE	7544	TKT	chr3	-	53237796	53237820	53234982	53235174	53240250	53240348	0	2	1	0	1	33	0	0	0	0	10	6	73	49	0.041838974	0.170760496	NA		0.573		0.02	NA		0		0	0.296
SE	2150	YTHDC1	chr4	-	68322748	68322915	68320122	68320205	68324138	68324223	0	1	3	0	0	0	0	8	0	0	1	0	98	49	0.04224465	0.170760496	NA		1		1	NA		0.8	NA		0.2
SE	2915	LPCAT1	chr5	-	1483427	1483486	1480941	1480976	1488390	1488451	0	1	2	0	0	0	0	8	0	0	1	0	98	49	0.04224465	0.170760496	NA		1		1	NA		0.8	NA		0.2
SE	3672	CALCOCO2	chr17	+	48848063	48848166	48841697	48841887	48848321	48848455	0	1	8	0	0	0	0	8	0	0	1	0	98	49	0.04224465	0.170760496	NA		1		1	NA		0.8	NA		0.2

SE	1138	IGF2R	chr6	+	160061502	160061646	160060546	160060717	160062531	160062619	0	5	19	0	0	0	2	9	3	0	4	0	98	49	0.042663026	0.170760496	NA	1	1	1	0.529	1	0.157
SE	5043	CFL1	chr11	-	65855653	65855730	65854813	65855448	65855934	65856242	0	9	66	0	0	0	10	26	1	0	1	0	98	49	0.042685592	0.170760496	NA	1	1	1	0.929	1	0.024
SE	7672	SF3B1	chr2	-	197392971	197393188	197391973	197392461	197396055	197396328	0	5	39	0	0	0	0	9	1	0	1	0	98	49	0.042920223	0.170760496	NA	1	1	NA	0.818	1	0.091
SE	9384	PECAM1	chr17	-	64375050	64375356	64369749	64370025	64377823	64378117	0	2	51	0	0	0	8	20	0	0	1	0	98	49	0.043140749	0.170760496	NA	1	1	1	0.909	NA	0.045
SE	8137	ITGAX	chr16	+	31356624	31356728	31355892	31355998	31357030	31357101	0	26	76	0	0	0	8	82	30	0	2	0	98	49	0.043231591	0.170760496	NA	1	1	1	0.953	1	0.016
SE	22	ARHGAP4	chrX	-	153919566	153919686	153919154	153919283	153920625	153920808	0	2	0	2	2	2	0	0	0	0	6	3	98	49	0.043694816	0.170760496	0	0.333	0	NA	0	0	0.111
A3SS	4015	PLEKHB2	chr2	+	131140166	131140275	131140169	131140275	131132901	131132991	0	1	3	0	0	0	0	4	0	0	1	0	52	49	0.043807749	0.197717869	NA	1	1	NA	0.79	NA	0.21
A3SS	461	BTN3A1	chr6	+	26412411	26415216	26413168	26415216	26411554	26411581	0	1	5	0	0	2	0	10	0	0	1	0	98	49	0.044092288	0.197717869	NA	1	0.556	NA	0.833	NA	-0.055
SE	7268	IVNS1ABP	chr1	-	185309002	185309172	185308799	185308875	185309382	185309511	0	1	10	0	0	0	0	10	0	0	1	0	98	49	0.044092288	0.170760496	NA	1	1	NA	0.833	NA	0.167
SE	5494	ADAM8	chr10	-	133268747	133268862	133267928	133268118	133269444	133269529	0	2	19	0	0	0	0	25	4	0	2	0	98	49	0.044237698	0.170760496	NA	1	1	NA	0.862	1	0.069
SE	1818	CHD1	chr5	-	98881974	98882123	98881278	98881375	98883087	98883237	0	2	14	0	0	0	0	23	0	0	1	0	98	49	0.044532178	0.170760496	NA	1	1	NA	0.92	NA	0.08
SE	814	LINC-PINT	chr7	-	130959868	130959970	130944159	130945835	130984052	130984109	0	0	1	0	1	2	0	1	0	0	4	0	98	49	0.044611886	0.170760496	NA	0	0.2	NA	0.111	NA	-0.011
SE	815	LINC-PINT	chr7	-	130967388	130967517	130944159	130945835	130984052	130984109	0	0	5	0	1	2	0	1	0	0	4	0	98	49	0.044611886	0.170760496	NA	0	0.556	NA	0.111	NA	0.167
A5SS	3046	PLEKHB2	chr2	+	131132901	131133015	131132901	131132991	131140166	131140275	0	0	4	0	1	3	0	1	0	0	4	0	73	49	0.044612593	0.170414363	NA	0	0.472	NA	0.144	NA	0.092
SE	11394	ADGRG3	chr16	+	57679176	57679311	57678169	57678316	57679815	57679855	0	0	15	0	1	0	0	11	3	0	0	0	98	49	0.044729667	0.170760496	NA	0	1	NA	1	1	-0.5
SE	2103	WDFY3	chr4	-	84836928	84837090	84831412	84831605	84841153	84841263	0	1	20	0	0	0	0	11	0	0	1	0	98	49	0.044972537	0.170760496	NA	1	1	NA	0.846	NA	0.154
SE	7942	IFI44L	chr1	+	78629719	78629915	78628950	78628999	78635336	78635489	0	3	61	0	0	2	4	38	0	0	1	0	98	49	0.045281167	0.170760496	NA	1	0.938	1	0.95	NA	-0.006
SE	7117	MED13L	chr12	-	116007410	116007636	116006305	116006411	116008400	116009132	0	2	11	0	0	0	0	25	0	0	1	0	98	49	0.045416917	0.170760496	NA	1	1	NA	0.926	NA	0.074
SE	4382	STK38L	chr12	+	27308338	27308461	27302136	27302188	27309113	27309197	0	3	8	0	0	0	0	5	1	0	1	0	98	49	0.045453113	0.170760496	NA	1	1	NA	0.714	1	0.143
SE	1691	DOCK2	chr5	+	169761518	169761625	169759704	169759775	169803057	169803206	0	4	19	0	0	0	0	12	1	0	1	0	98	49	0.045620082	0.170760496	NA	1	1	NA	0.857	1	0.072
SE	6818	OAS1	chr12	+	112917546	112917700	112916508	112916738	112919388	112919907	0	3	8	0	0	0	0	8	1	0	1	0	98	49	0.045786327	0.170760496	NA	1	1	NA	0.8	1	0.1
SE	7899	IFIT3	chr10	+	89337420	89337542	89332478	89332627	89338660	89340968	0	0	0	0	1	1	0	1	0	0	5	0	98	49	0.046158728	0.170760496	NA	0	0	NA	0.091	NA	-0.091
SE	9005	LYST	chr1	-	235709090	235709308	235702746	235702977	235712056	235712197	0	3	10	0	0	0	0	4	1	0	1	0	98	49	0.046507952	0.170760496	NA	1	1	NA	0.667	1	0.166
A5SS	2803	TYROBP	chr19	-	35908167	35908309	35908174	35908309	35907445	35907580	0	0	5	0	3	1	0	1	2	0	0	0	56	49	0.046582587	0.170414363	NA	0	0.814	NA	1	1	-0.593
SE	5273	NIPBL	chr5	+	37036378	37036487	37027358	37027412	37038601	37038738	0	1	11	0	0	0	2	13	0	0	1	0	98	49	0.046638092	0.170760496	NA	1	1	1	0.867	NA	0.067
SE	9385	CLTC	chr17	+	59682270	59682428	59681646	59681839	59682628	59682793	0	1	12	0	0	0	0	13	0	0	1	0	98	49	0.046638092	0.170760496	NA	1	1	NA	0.867	NA	0.133
SE	4948	ARAP1	chr11	-	72712195	72712339	72711429	72711499	72712437	72712568	0	7	9	0	0	0	0	6	3	0	1	0	98	49	0.047039397	0.170760496	NA	1	1	NA	0.75	1	0.125
SE	9916	CASP8	chr2	+	201274888	201274953	201272897	201272942	201276826	201276968	0	1	6	0	0	2	0	14	0	0	1	0	98	49	0.047441935	0.170760496	NA	1	0.6	NA	0.875	NA	-0.075
SE	11995	STAT3	chr17	-	42348388	42348517	42346568	42346713	42388278	42388495	0	1	28	0	0	0	0	14	0	0	1	0	98	49	0.047441935	0.170760496	NA	1	1	NA	0.875	NA	0.125
SE	9286	VIM	chr10	+	17236293	17236379	17235168	17235389	17237229	17237593	0	7	135	0	0	0	15	37	1	0	1	0	98	49	0.0475366	0.170760496	NA	1	1	1	0.949	1	0.017
SE	510	ST3GAL1	chr8	-	133475718	133476600	133465893	133466090	133499134	133499189	0	1	1	0	0	3	0	3	1	0	0	0	98	49	0.047617	0.170760496	NA	1	0.143	NA	1	1	-0.428
SE	12755	ADGRE5	chr19	+	14396341	14396473	14390923	14391079	14397657	14397804	0	0	4	0	1	11	5	1	0	0	6	0	98	49	0.047680582	0.170760496	NA	0	0.154	1	0.077	NA	-0.461
SE	5193	PICALM	chr11	-	85978069	85978093	85976622	85976682	85981128	85981228	0	0	12	0	2	14	0	1	0	0	14	0	73	49	0.047713059	0.170760496	NA	0	0.365	NA	0.046	NA	0.137
SE	12573	R3HDM4	chr19	-	899600	899686	899439	899495	900060	900146	0	6	13	0	0	0	0	32	2	0	1	0	98	49	0.047912713	0.170760496	NA	1	1	NA	0.941	1	0.03
SE	6173	ABCA1	chr9	-	104804625	104804720	104803283	104803316	104806240	104806430	0	4	7	0	0	0	0	25	1	0	1	0	98	49	0.048012831	0.170760496	NA	1	1	NA	0.926	1	0.037
SE	11306	SP100	chr2	+	230461261	230461414	230450171	230450255	230462434	230462518	0	3	35	0	0	0	8	15	5	0	2	0	98	49	0.048106877	0.170760496	NA	1	1	1	0.789	1	0.07
A3SS	1145	FBXL5	chr4	-	15644492	15644708	15644492	15644657	15655203	15655412	0	1	40	0	0	0	0	15	0	0	1	0	98	49	0.048230843	0.197717869	NA	1	1	NA	0.882	NA	0.118
SE	8157	KMT2E	chr7	+	105078845	105078963	105077302	105077433	105081687	105081797	0	1	12	0	0	0	0	15	0	0	1	0	98	49	0.048230843	0.170760496	NA	1	1	NA	0.882	NA	0.118
SE	10588	RNF213	chr17	+	80363608	80363790	80363101	80363314	80364432	80364553	0	15	83	0	0	0	0	31	8	0	1	0	98	49	0.048276428	0.170760496	NA	1	1	NA	0.939	1	0.03
SE	12401	CSF2RB	chr22	+	36936548	36936652	36935629	36935687	36937376	36940437	0	7	34	0	0	0	4	32	2	0	1	0	98	49	0.048373702	0.170760496	NA	1	1	1	0.941	1	0.02
SE	7883	HIF1A	chr14	+	61740504	61740627	61738086	61738373	61740754	61741188	0	0	14	0	1	0	0	18	1	0	0	0	98	49	0.048559241	0.170760496	NA	0	1	NA	1	1	-0.5
SE	4361	LRRK2	chr12	+	40293544	40293663	40287350	40287539	40294844	40294914	0	2	74	0	0	0	0	33	0	0	1	0	98	49	0.048704034	0.170760496	NA	1	1	NA	0.943	NA	0.057
SE	11375	ARHGEF1	chr19	+	41894625	41894661	41894450	41894547	41895348	41895486	0	5	22	0	0	0	0	16	2	0	1	0	85	49	0.048992317	0.170760496	NA	1	1	NA	0.902	1	0.049
A3SS	555	RICTOR	chr5	-	38945490	38945724	38945490	38945610	38946467	38946552	0	1	15	0	0	1	0	16	0	0	1	0	98	49	0.048997106	0.197717869	NA	1	0.882	NA	0.889	NA	0.052
SE	9006	LYST	chr1	-	235728075	235728131	235724027	235724180	235729595	235729657	0	1	35	0	0	0	2	16	0	0	1	0	98	49	0.048997106	0.170760496	NA	1	1	1	0.889	NA	0.055
SE	6618	AMPD2	chr1	+	109629326	109629490	109629108	109629235	109629795	109629916	0	2	5	0	0	0	0	6	1	0	1	0	98	49	0.049093709	0.170760496	NA	1	1	NA	0.75	1	0.125
SE	6896	ITGB2	chr21	-	44889275	44889495	44888692	44888895	44889977	44890222	0	4	78	0	0	0	3	11	2	0	1	0	98	49	0.049219655	0.170760496	NA	1	1	1	0.846	1	0.051
SE	11046	HLA-B	chr6	-	31355106	31355223	31354478	31354526	31355316	31355592	0	40	238	0	0	0	10	193	14	0	1	0	98	49	0.049293349	0.170760496	NA	1	1	1	0.99	1	0.003
SE	9374	FMNL1	chr17	+	45233647	45233731	45233223	45233297	45234071	45234200	0	4	23	0	0	0	0	21	1	0	1	0	98	49	0.049821364	0.170760496	NA	1	1	NA	0.913	1	0.043
SE	7884	HIF1A	chr14	+	61740754	61741188	61738086	61738373	61744704	61744813	0	1	13	0	0	3	0	34	1	0	0	0	98	49	0.049963886	0.170760496	NA	1	0.684	NA	1	1	-0.158
SE	9513	SIPA1	chr11	+	65644954	65645129	65642462	65642639	65646220	65646378	0	1	11	0	2	0	0	7	7	0	0	0	98	49	0.050052195	0.170760496	NA	0.2	1	NA	1	1	-0.4
SE	6178	ABCA1	chr9	-	104828915	104829138	104826947	104827169	104830924	104831101	0	4	14	0	0	0	0	25	1	0	1	0	98	49	0.050181472	0.170760496	NA	1	1	NA	0.926	1	0.037
SE	1146	IGF2R	chr6	+	160102518	160102671	160096438	160096625	160103745	160103815	0	6	13	0	0	0	0	14	7	0	2	0	98	49	0.050333626	0.170760496	NA	1	1	NA	0.778	1	0.111
SE	10067	ACTN1	chr14	-	68884774	68884883	68884167	68884308	68885424	68885575	0	1	15	0	0	0	0	18	0	0	1	0	98	49	0.050460196	0.170760496	NA	1	1	NA	0.9	NA	0.1
SE	4427	PREX1	chr20	-	48734545	48734650	48726289	48726391	48745024	48745147	0	12	55	0	0	0	3	75	3	0	1	0	98	49	0.050469046	0.170760496	NA	1	1	1	0.974	1	0.009
SE	12942	SMCHD1	chr18	+	2703691	2703886	2700734	2700918	2705693	2705807	1	4	139	0	0	0	0	77	0	0	1	0	98	49	0.05052881	0.170760496	1	1	1	NA	0.975	NA	0.025
SE	7030	ARPC2	chr2	+	218217462	218217544	218217093	218217254	218225919	218225954	0	4	9	0	0	0	0	24	2	0	1	0	98	49	0.050622824	0.170760496	NA	1	1	NA	0.923	1	0.038
SE	12325	MAPKAPK2	chr1	+	206731838	206731919	206731639	206731725	206732574	206734285	0	1	14	0	2	0	5	16	3	0	0	0	98	49	0.050687516	0.170760496	NA	0.2	1	1	1	1	-0.4
SE	11256	SRSF7	chr2	-	38746143	38746179	38743598	38745187	38746693	38746747	0	2	5	0	0	0	0	7	1	0	1	0	85	49	0.05078876	0.170760496	NA	1	1	NA	0.801	1	0.099
SE	5864	NFKBID	chr19	-	35896912	35897058	35896725	35896831	35897650	35897856	0	6	7	0	0	0	0	4	7	0	0	1	98	49	0.050792748	0.170760496	NA	1	1	NA	1	0.778	0.111
SE	9455	PFKFB3	chr10	+	6216124	6216191	6215220	6215317	6216705	6216780	0	19	68	0	1	1	1	72	24	0	0	0	98	49	0.050850888	0.170760496	NA	0.905	0.971	1	1	1	-0.062
SE	8552	EHBP1L1	chr11	+	65581538	65583765	65581210	65581373	65584240	65584398	0	2	12	0	0	0	0	5	1	0	1	0	98	49	0.051092621	0.170760496	NA	1	1	NA	0.714	1	0.143
SE	5410	TAGAP	chr6	-	159043588	159043655	159042077	159042244	159043977	159044031	0	1	14	0	0	0	0	19	0	0	1	0	98	49	0.051175801	0.170760496	NA	1	1	NA	0.905	NA	0.095
RI	1060	SEC14L1	chr17	+	77213917	77217099	77213917	77214019	77216548	77217099	0	13	22	0	0	0	6	16	9	0	1	0	98	49	0.051358786	0.297702651	NA	1	1	1	0.889	1	0.037
SE	4670	TNFRSF1B	chr1	+	12201966	12202171	12194583	12194618	12206739	12209220	0	5	27	0	0	0	0	27	2	0	1	0	98	49	0.051369383	0.170760496	NA	1	1	NA	0.931	1	0.034
SE	4501	PTPN6	chr12	+	6951608	6951731	6951270	6951520	6951982	6952177	0	5	25	0	0	0	0	8	3	0	1	0	98	49	0.051433279	0.170760496	NA	1	1	NA	0.8	1	0.1
SE	7417	VAV1	chr19	+	6836984	6837050	6836431	6836568	6843134	6843166	0	1	12	0	0	0	3	20	0	0	1	0	98	49	0.051859277	0.170760496	NA	1	1	1	0.909	NA	0.045
SE	12684	HNRNPK	chr9	-	83973901	83973973	83973285	83973399	83974516	83974589	0	1	53	0	0	3	1	20	0	0	1	0	98	49	0.051859277	0.170760496	NA	1	0.898	1	0.909	NA	-0.005
SE	3134	IFNGR2	chr21	+	33414887	33415020	33402895	33403616	33421479	33421685	9	14	31	0	0	0	0	15	47	0	0	1	98	49	0.052010728	0.170760496	1	1	1	NA	1	0.959	0.02
SE	7031	ARPC2	chr2	+	218239390	218239484	218238663	218238850	218245419	218245546	0	0	56	0	0	1	1	33	2	0	1	2	98	49	0.052035219	0.170760496	NA	NA	0.966	1	0.943	0.333	0.207
SE	1737	ARHGAP26	chr5	+	143014079	143014116	142932046	142932125	143037195	143037261	0	1	47	0	0	0	0	23	4	0	2	0	86	49	0.052066191	0.170760496	NA	1	1	NA	0.868	1	0.066
SE	13867	ACSL1	chr4	-	184776483	184776662	184773842	184773875	184776883	184776983	0	3	33	0	0	1	1	36	1	0	6	0	98	49	0.052282739	0.170760496	NA	1	0.943	1	0.75	1	0.055
SE	4669	TNFRSF1B	chr1	+	12188795	12188895	12167002	12167169	12190956	12191085	0	7	37	0	2	3	5	25	32	0	0	1	98	49	0.052384975	0.170760496	NA	0.636	0.86	1	1	0.941	-0.232
SE	9376	FMNL1	chr17	+	45245252	45245416	45244978	45245108	45245877	45245973	0	3	24	0	0	0	0	14	0	0	1	2	98	49	0.052499618	0.170760496	NA	1	1	NA	0.875	0	0.562
SE	10927	DEDD2	chr19	-	42201988	42202072	42198592	42199829	42209699	42209840	0	1	2	0	3	5	0	1	0	0	16	3	98	49	0.052585138	0.170760496	NA	0.143	0.167	NA	0.03	0	0.14
SE	1854	DBNL	chr7	+	44058431	44058480	44058128	44058280	44058901	44058983	0	4	5	0	0	0	0	3	2	0	1	0	98	49	0.052858149	0.170760496	NA	1	1	NA	0.6	1	0.2
RI	576	SRSF2	chr17	-	76734114	76736464	76734114	76735158	76736153	76736464	0	7	12	0	2	3	0	16	1	0	12	1	98	49	0.052909456	0.297702651	NA	0.636	0.667	NA	0.4	0.333	0.285
A3SS	459	BTN3A1	chr6	+	26407670	26407952	26407826	26407952	26405908	26406256	0	2	3	0	0	0	2	2	1	0	1	0	98	49	0.05291102	0.197717869	NA	1	1	1	0.5	1	0.167
SE	8417	GRAMD1A	chr19	+	35021950	35022038	35021690	35021864	35023235	35023343	0	4	17	0	0	0	0	4	12	0	0	1	98	49	0.052978196	0.170760496	NA	1	1	NA	1	0.857	0.072
SE	7910	ATP6V0B	chr1	+	43977025	43977216	43976772	43976824	43977980	43978300	0	4	22	0	0	0	1	11	3	0	1	0	98	49	0.053042143	0.170760496	NA	1	1	1	0.846	1	0.051
SE	10586	RNF213	chr17	+	80360060	80360206	80358287	80358479	80361733	80361888	0	1	46	0	0	0	2	41	4	0	2	0	98	49	0.053139356	0.170760496	NA	1	1	1	0.911	1	0.03
SE	6938	CD55	chr1	+	207331107	207331296	207326751	207326837	207336692	207336818	0	2	28	0	0	0	0	46	0	0	1	0	98	49	0.053445903	0.170760496	NA	1	1	NA	0.958	NA	0.042
SE	7392	IRAK3	chr12	+	66211445	66211597	66210146	66210201	66217170	66217235	0	2	30	0	0	0	2	10	1	0	1	0	98	49	0.053461329	0.170760496	NA	1	1	1	0.833	1	0.056
SE	9740	PXN	chr12	-	120222548	120222750	120221622	120221758	120222862	120222999	0	3	23	0	0	0	0	6	2	0	1	0	98	49	0.053502796	0.170760496	NA	1	1	NA	0.75	1	0.125
SE	4088	TNFRSF1A	chr12	-	6334090	6334244	6333736	6333865	6341775	6342117	0	2	17	0	3	1	0	5	3	0	0	0	98	49	0.053505644	0.170760496	NA	0.25	0.895	NA	1	1	-0.427
SE	13863	ACSL1	chr4	-	184765890	184765986	184764852	184764925	184766621	184766756	0	2	59	0	0	3	4	48	12	0	0	0	98	49	0.053534738	0.170760496	NA	1	0.908	1	1	1	-0.046
SE	1144	IGF2R	chr6	+	160084994	160085131	160083949	160084184	160088032	160088147	0	4	17	0	0	0	1	20	3	0	1	0	98	49	0.05376828	0.170760496	NA	1	1	1	0.909	1	0.03
SE	8590	LTB	chr6	-	31581813	31581859	31581558	31581630	31582255	31582425	0	0	5	0	3	0	0	11	0	0	0	0	95	49	0.054362012	0.170760496	NA	0	1	NA	1	NA	-0.5
SE	4241	ITGA5	chr12	-	54403624	54403779	54403186	54403324	54403910	54403966	0	0	12	0	0	0	0	7	0	0	0	2	98	49	0.054536123	0.170760496	NA	NA	1	NA	1	0	0.5
A3SS	690	CNBP	chr3	-	129171078	129171283	129171078	129171277	129171445	129171538	0	0	0	0	1	4	0	1	0	0	11	0	55	49	0.054675117	0.197717869	NA	0	0	NA	0.075	NA	-0.075
SE	5196	PICALM	chr11	-	86001034	86001158	86000642	86000779	86003365	86003451	0	6	50	0	0	0	2	55	1	0	1	0	98	49	0.054703796	0.170760496	NA	1	1	1	0.965	1	0.012
SE	11996	STAT3	chr17	-	42348388	42348539	42346568	42346713	42388278	42388387	0	2	29	0	0	0	0	12	1	0	1	0	98	49	0.054806328	0.170760496	NA	1	1	NA	0.857	1	0.072
SE	7497	IFI44	chr1	+	78660553	78660654	78659311	78659483	78663764	78664078	0	0	31	0	1	1	0	20	1	0	0	0	98	49	0.054840236	0.170760496	NA	0	0.939	NA	1	1	-0.53
SE	7726	FGD3	chr9	+	93002924	93003014	92976207	92976709	93004000	93004137	0	10	22	0	0	0	0	16	9	0	1	0	98	49	0.054857277	0.170760496	NA	1	1	NA	0.889	1	0.055
SE	4940	ARAP1	chr11	-	72687438	72687502	72686041	72686191	72687687	72687738	0	3	10	0	0	0	0	4	2	0	1	0	98	49	0.055117945	0.170760496	NA	1	1	NA	0.667	1	0.166
SE	12992	PLAUR	chr19	-	43656478	43656640	43655438	43655573	43665315	43665459	0	14	45	0	0	0	0	84	14	0	1	0	98	49	0.055245993	0.170760496	NA	1	1	NA	0.977	1	0.012
SE	11304	SP100	chr2	+	230449560	230449710	230449087	230449150	230450171	230450255	0	7	33	0	0	0	0	28	7	0	1	0	98	49	0.055357429	0.170760496	NA	1	1	NA	0.933	1	0.033
SE	3739	BRD2	chr6	+	32976561	32976936	32976249	32976464	32977441	32977570	0	0	8	0	0	0	0	8	0	0	0	2	98	49	0.055397548	0.170760496	NA	NA	1	NA	1	0	0.5
A3SS	1302	GMPR2	chr14	+	24238245	24239241	24238588	24239241	24237519	24237562	1	0	3	0	2	0	0	10	0	0	0	0	98	49	0.055620739	0.197717869	1	0	1	NA	1	NA	-0.333
SE	2096	WDFY3	chr4	-	84678918	84679242	84678167	84678279	84682373	84682470	0	2	8	0	0	0	0	2	1	0	1	0	98	49	0.055836208	0.170760496	NA	1	1	NA	0.5	1	0.25
SE	6103	GLUL	chr1	-	182385759	182385887	182385356	182385556	182386255	182386402	0	16	127	0	0	0	6	106	11	0	1	0	98	49	0.055843452	0.170760496	NA	1	1	1	0.981	1	0.006
SE	10624	RRP12	chr10	-	97366107	97366233	97363853	97363903	97366445	97366621	0	0	2	0	0	0	0	1	0	0	0	5	98	49	0.055847068	0.170760496	NA	NA	1	NA	1	0	0.5
SE	2782	ATG7	chr3	+	11299361	11299416	11298685	11298855	11306942	11307060	0	0	7	0	1	0	0	9	1	0	0	0	98	49	0.055867249	0.170760496	NA	0	1	NA	1	1	-0.5
SE	11397	ADGRG3	chr16	+	57684389	57684483	57680504	57680617	57685642	57685926	0	0	7	0	2	0	0	9	0	0	0	0	98	49	0.055954714	0.170760496	NA	0	1	NA	1	NA	-0.5
SE	2283	IL1RAP	chr3	+	190550341	190550505	190514050	190514219	190556129	190556216	0	0	0	0	3	3	0	3	0	0	4	2	98	49	0.05597701	0.170760496	NA	0	0	NA	0.273	0	-0.137
SE	1506	HLA-E	chr6	+	30491536	30491653	30491136	30491412	30492540	30492583	0	12	185	0	0	0	11	70	10	0	1	0	98	49	0.056110759	0.170760496	NA	1	1	1	0.972	1	0.009
SE	3382	HCK	chr20	+	32084390	32084543	32083893	32084043	32086627	32086807	0	3	55	0	1	5	5	17	4	0	0	0	98	49	0.056127965	0.170760496	NA	0.6	0.846	1	1	1	-0.277
SE	3057	COL18A1	chr21	+	45505133	45505278	45504521	45504556	45505357	45505431	0	0	6	0	0	0	0	2	0	0	0	2	98	49	0.056129395	0.170760496	NA	NA	1	NA	1	0	0.5
SE	10626	RRP12	chr10	-	97379292	97379414	97373829	97373894	97379627	97379770	0	2	7	0	0	0	0	9	2	0	1	0	98	49	0.056251461	0.170760496	NA	1	1	NA	0.818	1	0.091
SE	1507	HLA-E	chr6	+	30492403	30492436	30491136	30491412	30492540	30492583	0	7	64	0	0	0	0	48	3	0	1	0	82	49	0.05627258	0.170760496	NA	1	1	NA	0.966	1	0.017
SE	9587	ZNF467	chr7	-	149770439	149770556	149769089	149769200	149770998	149771074	0	0	1	0	1	0	0	2	3	0	0	0	98	49	0.056299876	0.170760496	NA	0	1	NA	1	1	-0.5
SE	3981	NCOR2	chr12	-	124374412	124374463	124372021	124372610	124378236	124378384	0	0	2	0	0	0	0	0	0	0	0	4	98	49	0.056348041	0.170760496	NA	NA	1	NA	NA	0	1
SE	6199	RPRD2	chr1	+	150441880	150441958	150440922	150441023	150443230	150443283	0	2	0	0	0	0	0	0	0	0	4	0	98	49	0.056348041	0.170760496	NA	1	NA	NA	0	NA	1
SE	636	RB1CC1	chr8	-	52660595	52660657	52658872	52658976	52660925	52661007	0	0	3	0	0	0	0	4	0	0	0	3	98	49	0.056460428	0.170760496	NA	NA	1	NA	1	0	0.5
SE	12437	SPAG9	chr17	-	51014231	51014353	51007268	51007326	51020158	51020258	0	0	8	0	1	0	0	14	0	0	0	0	98	49	0.056479947	0.170760496	NA	0	1	NA	1	NA	-0.5
SE	6672	FGR	chr1	-	27621558	27621657	27615688	27615844	27623041	27623144	0	3	15	0	0	0	0	10	3	0	1	0	98	49	0.0565412	0.170760496	NA	1	1	NA	0.833	1	0.084
SE	3944	ZDHHC20	chr13	-	21421060	21421164	21413651	21413772	21425651	21425678	0	0	5	0	0	0	0	0	0	0	1	2	98	49	0.056609827	0.170760496	NA	NA	1	NA	0	0	1
SE	10040	SYNE2	chr14	+	64029894	64030059	64027483	64027793	64031015	64031357	0	0	5	0	2	0	0	7	0	0	0	0	98	49	0.056662448	0.170760496	NA	0	1	NA	1	NA	-0.5
SE	3801	ZCCHC2	chr18	+	62544801	62544873	62542497	62542574	62550347	62550460	0	0	29	0	1	2	3	13	0	0	0	0	98	49	0.056686045	0.170760496	NA	0	0.879	1	1	NA	-0.56
SE	1817	CHD1	chr5	-	98876397	98876558	98875071	98875113	98879551	98879728	0	1	2	0	0	0	0	8	2	0	9	0	98	49	0.056740038	0.170760496	NA	1	1	NA	0.308	1	0.346
SE	2275	VNN3	chr6	-	132728740	132728979	132724605	132724979	132734252	132734383	0	1	2	0	0	0	0	7	1	0	1	0	98	49	0.056900121	0.170760496	NA	1	1	NA	0.778	1	0.111
A5SS	2964	USF1	chr1	-	161042554	161042670	161042585	161042670	161042115	161042217	0	0	9	0	2	2	0	6	0	0	0	0	80	49	0.057135855	0.170414363	NA	0	0.734	NA	1	NA	-0.633
SE	3101	MX1	chr21	+	41451166	41451243	41445999	41446141	41452620	41452869	0	2	24	0	0	0	1	15	1	0	1	0	98	49	0.057325035	0.170760496	NA	1	1	1	0.882	1	0.039

SE	12164	USP32	chr17	-	60180544	60180637	60177329	60179428	60181323	60181748	0	0	12	0	1	0	0	10	0	0	0	0	98	49	0.057462192	0.170760496	NA	0	1	NA	1	NA	-0.5
SE	6865	ATG2A	chr11	-	64901961	64902176	64900493	64900629	64902259	64902386	0	0	2	0	0	0	0	2	0	0	1	3	98	49	0.057547789	0.170760496	NA	NA	1	NA	0.5	0	0.75
SE	8248	PRRC2C	chr1	+	171513000	171513172	171485526	171485735	171514535	171514645	0	1	1	0	0	0	2	2	1	0	1	0	98	49	0.057579548	0.170760496	NA	1	1	1	0.5	1	0.167
SE	8660	DUSP1	chr5	-	172770160	172770306	172769574	172769794	172770585	172771200	4	105	424	0	1	1	27	197	285	0	2	6	98	49	0.057728535	0.170760496	1	0.981	0.995	1	0.98	0.96	0.012
SE	5304	CD82	chr11	+	44605354	44605429	44605057	44605182	44615271	44615373	0	0	1	0	2	0	0	5	0	0	0	0	98	49	0.057729	0.170760496	NA	0	1	NA	1	NA	-0.5
SE	4803	CASP4	chr11	-	104950924	104951098	104949542	104949777	104954746	104955001	0	0	17	0	1	2	4	9	0	0	0	0	98	49	0.057774487	0.170760496	NA	0	0.81	1	1	NA	-0.595
SE	5518	DENND5A	chr11	-	9145669	9145815	9144994	9145113	9147029	9147151	0	0	13	0	1	0	2	9	0	0	0	0	98	49	0.057774487	0.170760496	NA	0	1	1	1	NA	-0.5
SE	5966	15-Sep	chr1	-	86868052	86868102	86862444	86863605	86880661	86880725	0	0	12	0	1	0	0	9	0	0	0	0	98	49	0.057774487	0.170760496	NA	0	1	NA	1	NA	-0.5
SE	6091	SRRM2	chr16	+	2756333	2756606	2752328	2752846	2757471	2757579	0	0	7	0	1	0	0	9	0	0	0	0	98	49	0.057774487	0.170760496	NA	0	1	NA	1	NA	-0.5
SE	11055	ARFGAP3	chr22	-	42834241	42834325	42831548	42831636	42835361	42835493	0	0	8	0	1	0	0	9	0	0	0	0	98	49	0.057774487	0.170760496	NA	0	1	NA	1	NA	-0.5
SE	15	FLNA	chrX	-	154352180	154352447	154351883	154352021	154352771	154352924	0	1	15	0	0	0	5	8	3	0	2	0	98	49	0.058033181	0.170760496	NA	1	1	1	0.667	1	0.111
SE	9987	TRANK1	chr3	-	36838371	36838504	36834761	36834907	36838612	36838716	0	0	3	0	0	0	0	3	0	0	0	2	98	49	0.058049942	0.170760496	NA	NA	1	NA	1	0	0.5
SE	8136	ITGAX	chr16	+	31355892	31355998	31355133	31355291	31356624	31356728	0	17	42	0	0	0	8	52	24	0	1	0	98	49	0.058165611	0.170760496	NA	1	1	1	0.963	1	0.012
SE	4886	DCAF11	chr14	+	24120837	24120991	24119710	24119896	24121364	24121517	0	0	4	0	1	0	0	8	0	0	0	0	98	49	0.058181256	0.170760496	NA	0	1	NA	1	NA	-0.5
RI	523	DDAH2	chr6	-	31728864	31730265	31728864	31729225	31730050	31730265	0	1	0	0	0	3	0	0	3	0	0	0	98	49	0.058431081	0.297702651	NA	1	0	NA	NA	1	-0.5
SE	4634	GMPR2	chr14	+	24238245	24238405	24237519	24237562	24238588	24239241	0	0	1	0	2	0	0	4	0	0	0	0	98	49	0.058521801	0.170760496	NA	0	1	NA	1	NA	-0.5
SE	11517	CMIP	chr16	+	81664268	81664349	81660883	81660946	81670141	81670245	0	0	5	0	2	0	0	4	0	0	0	0	98	49	0.058521801	0.170760496	NA	0	1	NA	1	NA	-0.5
SE	3630	USP4	chr3	-	49286097	49286325	49284848	49284919	49292509	49292598	0	2	22	0	0	0	6	10	2	0	1	0	98	49	0.058749844	0.170760496	NA	1	1	1	0.833	1	0.056
SE	14092	NLRP6	chr11	+	279833	279872	279326	279607	280083	281839	0	1	1	0	0	0	0	0	0	0	0	4	88	49	0.058768543	0.170760496	NA	1	1	NA	NA	0	1
SE	13371	CCT7	chr2	+	73243996	73244049	73243003	73243129	73244543	73244715	1	0	0	0	1	0	6	1	0	0	0	0	98	49	0.058830341	0.170760496	1	0	NA	1	1	NA	-0.5
SE	1853	DBNL	chr7	+	44058428	44058480	44058128	44058280	44058901	44058983	0	1	3	0	0	0	0	3	1	0	1	0	98	49	0.058903155	0.170760496	NA	1	1	NA	0.6	1	0.2
SE	3097	MX1	chr21	+	41437014	41437152	41435836	41436029	41439693	41439848	0	4	56	0	0	0	7	31	3	0	1	0	98	49	0.05891182	0.170760496	NA	1	1	1	0.939	1	0.02
SE	2611	KIAA0922	chr4	+	153592474	153592584	153591052	153591194	153593798	153593871	0	0	7	0	0	0	0	0	0	0	0	1	98	49	0.05894622	0.170760496	NA	NA	1	NA	NA	0	1
SE	6096	CLN3	chr16	-	28491481	28491560	28489289	28489386	28491713	28491835	0	0	7	0	0	0	0	0	0	0	0	1	98	49	0.05894622	0.170760496	NA	NA	1	NA	NA	0	1
SE	9258	MOV10	chr1	+	112694748	112694896	112694452	112694629	112695415	112695574	0	5	7	0	0	0	0	0	0	0	0	1	98	49	0.05894622	0.170760496	NA	1	1	NA	NA	0	1
SE	12991	PLAUR	chr19	-	43655438	43655573	43652224	43652371	43656478	43656640	0	8	42	0	0	6	0	48	10	0	1	0	98	49	0.059088669	0.170760496	NA	1	0.778	NA	0.96	1	-0.091
SE	13915	CD300A	chr17	+	74473535	74473874	74466382	74466743	74474531	74474685	0	0	1	0	1	1	0	6	0	0	0	0	98	49	0.05910855	0.170760496	NA	0	0.333	NA	1	NA	-0.834
SE	4341	SLC38A2	chr12	-	46370511	46370627	46367266	46367340	46370775	46370857	0	2	6	0	0	1	0	0	0	0	2	0	98	49	0.05919998	0.170760496	NA	1	0.75	NA	0	NA	0.875
SE	5039	RELT	chr11	+	73391143	73391223	73390754	73390921	73392210	73392468	0	0	2	0	0	0	0	6	0	0	1	2	98	49	0.05919998	0.170760496	NA	NA	1	NA	0.75	0	0.625
SE	7302	PPP6R2	chr22	+	50436987	50437068	50436366	50436452	50437505	50437603	0	0	2	0	0	0	0	0	0	0	0	2	98	49	0.05919998	0.170760496	NA	NA	1	NA	NA	0	1
SE	12511	WDR7	chr18	+	56717963	56718159	56696241	56696462	56731382	56731597	0	0	2	0	0	0	0	3	0	0	0	2	98	49	0.05919998	0.170760496	NA	NA	1	NA	1	0	0.5
SE	8184	EVI2A	chr17	-	31320372	31320513	31316409	31319023	31321494	31321749	0	0	0	0	0	4	0	0	2	0	0	0	98	49	0.059276699	0.170760496	NA	NA	0	NA	NA	1	-1
SE	9171	KLF6	chr10	-	3780105	3780229	3775995	3779590	3781640	3782214	0	3	14	1	0	0	0	6	3	0	1	0	98	49	0.059302042	0.170760496	0	1	1	NA	0.75	1	-0.208
SE	7343	FAM129A	chr1	-	184803584	184803692	184798078	184798190	184805945	184806056	0	1	38	0	0	0	0	7	1	0	1	0	98	49	0.059353638	0.170760496	NA	1	1	NA	0.778	1	0.111
A5SS	553	HMGA1	chr6	+	34240736	34240915	34240736	34240882	34242711	34242795	0	1	4	0	0	3	0	5	1	0	0	0	82	49	0.059398863	0.170414363	NA	1	0.443	NA	1	1	-0.278
A3SS	414	DCTN3	chr9	-	34614041	34614198	34614041	34614101	34614709	34614768	1	1	0	0	0	0	0	0	0	0	3	0	98	49	0.059555994	0.197717869	1	1	NA	NA	0	NA	1
A5SS	2673	RNF31	chr14	+	24148257	24148477	24148257	24148413	24148641	24148701	0	1	1	0	0	3	0	0	0	0	3	0	98	49	0.059555994	0.170414363	NA	1	0.143	NA	0	NA	0.572
SE	6361	ZSWIM8	chr10	+	73789366	73789539	73789095	73789190	73789716	73789824	0	1	4	0	0	0	0	0	0	0	3	0	98	49	0.059555994	0.170760496	NA	1	1	NA	0	NA	1
SE	7469	SLTM	chr15	-	58899468	58899883	58898802	58898852	58901259	58901287	0	1	0	0	0	0	0	0	0	0	3	0	98	49	0.059555994	0.170760496	NA	1	NA	NA	0	NA	1
SE	7470	SLTM	chr15	-	58899468	58899937	58898802	58898852	58901259	58901287	0	1	0	0	0	0	0	0	0	0	3	0	98	49	0.059555994	0.170760496	NA	1	NA	NA	0	NA	1
SE	9472	USP33	chr1	-	77745420	77745599	77741616	77741748	77759642	77759879	0	0	1	0	0	0	0	0	0	0	0	3	98	49	0.059555994	0.170760496	NA	NA	1	NA	NA	0	1
SE	13888	RIF1	chr2	+	151410413	151410527	151409882	151410033	151411259	151411338	0	0	1	0	0	0	0	2	0	0	0	3	98	49	0.059555994	0.170760496	NA	NA	1	NA	1	0	0.5
SE	4181	DDIT3	chr12	-	57517268	57517595	57516587	57517180	57517705	57517753	0	1	5	0	0	0	0	4	1	0	1	0	98	49	0.059568296	0.170760496	NA	1	1	NA	0.667	1	0.166
SE	12744	DYSF	chr2	+	71561751	71561944	71555964	71556071	71564057	71564213	0	5	41	0	0	0	2	10	26	0	0	1	98	49	0.059610573	0.170760496	NA	1	1	1	1	0.929	0.024
SE	2865	POLDIP3	chr22	-	42591954	42592084	42587505	42587572	42595536	42595603	0	0	0	0	0	2	0	3	3	0	0	0	98	49	0.059642855	0.170760496	NA	NA	0	NA	1	1	-1
SE	7379	RNF19B	chr1	-	32949568	32949774	32948221	32948363	32964050	32964813	0	0	7	0	2	0	0	3	0	0	0	0	98	49	0.059642855	0.170760496	NA	0	1	NA	1	NA	-0.5
SE	7408	RBM23	chr14	-	22905605	22905659	22905335	22905453	22906194	22906368	0	0	2	0	2	1	0	3	0	0	0	0	98	49	0.059642855	0.170760496	NA	0	0.5	NA	1	NA	-0.75
SE	9772	FRYL	chr4	-	48512480	48512688	48510834	48510984	48515027	48515275	0	0	2	0	2	0	7	3	0	0	0	0	98	49	0.059642855	0.170760496	NA	0	1	1	1	NA	-0.5
SE	11703	POLR2A	chr17	+	7511810	7512014	7511405	7511588	7512157	7512344	0	2	19	0	0	0	0	5	2	0	1	0	98	49	0.059650539	0.170760496	NA	1	1	NA	0.714	1	0.143
SE	6864	ATG2A	chr11	-	64900883	64901092	64900493	64900629	64901961	64902176	0	0	0	0	0	1	0	5	5	0	0	0	98	49	0.059786145	0.170760496	NA	NA	0	NA	1	1	-1
A5SS	2457	SCARF1	chr17	-	1645559	1645789	1645596	1645789	1645177	1645239	0	0	0	0	0	1	0	3	5	0	3	0	86	49	0.059797532	0.170414363	NA	NA	0	NA	0.363	1	-0.681
A3SS	1375	ARHGAP25	chr2	+	68807272	68807480	68807275	68807480	68787839	68787956	0	0	5	0	1	0	0	5	0	0	0	0	52	49	0.059800864	0.197717869	NA	0	1	NA	1	NA	-0.5
SE	12948	SMCHD1	chr18	+	2778168	2778239	2777805	2777915	2784449	2784621	0	2	47	0	0	1	2	28	1	0	4	0	98	49	0.059897198	0.170760496	NA	1	0.959	1	0.778	1	0.053
SE	5027	OSBPL8	chr12	-	76459858	76459895	76450850	76450988	76487509	76487618	0	1	0	0	0	2	0	0	0	0	3	0	86	49	0.059993631	0.170760496	NA	1	0	NA	0	NA	0.5
SE	9705	ABTB1	chr3	+	127676966	127677083	127676535	127676581	127677167	127677286	0	1	20	0	0	0	5	4	1	0	1	0	98	49	0.060016261	0.170760496	NA	1	1	1	0.667	1	0.111
SE	4178	DDIT3	chr12	-	57517268	57517438	57516587	57517180	57517705	57517753	0	1	6	0	0	0	0	5	1	0	1	0	98	49	0.060034631	0.170760496	NA	1	1	NA	0.714	1	0.143
SE	364	SEMA4D	chr9	-	89418055	89418118	89405350	89405699	89455887	89455953	0	0	5	0	0	0	0	7	0	1	0	1	98	49	0.060038159	0.170760496	NA	NA	1	0	1	0	0.667
A5SS	1404	RBMS1	chr2	-	160286968	160287084	160286977	160287084	160284994	160285044	1	0	2	0	0	2	0	1	0	4	0	0	58	49	0.060055861	0.170414363	1	NA	0.458	0	1	NA	0.229
A3SS	413	DCTN3	chr9	-	34614041	34614132	34614041	34614101	34614709	34614768	1	1	0	0	0	0	0	0	0	0	3	0	80	49	0.060211792	0.197717869	1	1	NA	NA	0	NA	1
SE	12010	GPR108	chr19	-	6731890	6731934	6731472	6731522	6732024	6732155	0	0	5	0	0	0	0	5	0	0	0	1	93	49	0.060224876	0.170760496	NA	NA	1	NA	1	0	0.5
SE	8079	NADK	chr1	-	1757180	1757310	1756257	1756343	1761951	1762035	0	1	24	0	0	0	0	6	1	0	1	0	98	49	0.060230863	0.170760496	NA	1	1	NA	0.75	1	0.125
SE	2349	FNDC3B	chr3	+	172346326	172346440	172344085	172344258	172347211	172347361	0	1	6	0	0	0	0	7	3	0	2	0	98	49	0.060484453	0.170760496	NA	1	1	NA	0.636	1	0.182
SE	4177	DDIT3	chr12	-	57517268	57517424	57516587	57517180	57517705	57517753	0	1	5	0	0	0	0	3	1	0	1	0	98	49	0.060546566	0.170760496	NA	1	1	NA	0.6	1	0.2
SE	4179	DDIT3	chr12	-	57517268	57517519	57516587	57517180	57517705	57517753	0	1	5	0	0	0	0	3	1	0	1	0	98	49	0.060546566	0.170760496	NA	1	1	NA	0.6	1	0.2
SE	4180	DDIT3	chr12	-	57517268	57517542	57516587	57517180	57517705	57517753	0	1	5	0	0	0	0	3	1	0	1	0	98	49	0.060546566	0.170760496	NA	1	1	NA	0.6	1	0.2
SE	13577	STK17B	chr2	-	196163261	196163427	196156438	196156651	196171332	196171612	0	21	136	0	0	0	6	68	29	0	1	0	98	49	0.060570323	0.170760496	NA	1	1	1	0.971	1	0.01
SE	1540	HLA-F	chr6	+	29725030	29725306	29724172	29724448	29725446	29725563	0	4	52	0	0	0	0	23	5	0	1	0	98	49	0.06060798	0.170760496	NA	1	1	NA	0.92	1	0.04
SE	11556	KIAA0513	chr16	+	85078414	85078455	85077424	85077632	85078924	85079003	0	0	9	0	1	0	0	4	0	0	0	0	90	49	0.060658984	0.170760496	NA	0	1	NA	1	NA	-0.5
A3SS	3134	STX4	chr16	+	31034203	31034325	31034225	31034325	31034012	31034114	0	0	3	0	1	2	1	4	0	0	0	0	71	49	0.06066392	0.197717869	NA	0	0.509	1	1	NA	-0.746
SE	2842	HDAC10	chr22	-	50247691	50247776	50246874	50246966	50247889	50248145	0	0	0	0	0	1	0	0	4	0	2	0	98	49	0.060672385	0.170760496	NA	NA	0	NA	0	1	-0.5
SE	6993	MATR3	chr5	+	139307238	139308327	139293643	139293805	139314674	139314736	0	0	0	0	1	0	0	4	0	0	0	0	98	49	0.060672385	0.170760496	NA	0	NA	NA	1	NA	-1
SE	7078	ERBB2IP	chr5	+	66075023	66075230	66053405	66054951	66076315	66076408	0	0	2	0	1	0	0	4	0	0	0	0	98	49	0.060672385	0.170760496	NA	0	1	NA	1	NA	-0.5
SE	1858	AP3B1	chr5	-	78228143	78228239	78227371	78227532	78240861	78240936	0	1	13	0	0	0	0	5	1	0	1	0	98	49	0.060716183	0.170760496	NA	1	1	NA	0.714	1	0.143
A3SS	2183	PPP4R1	chr18	-	9595017	9595153	9595017	9595102	9614225	9614270	0	0	4	0	0	0	0	2	0	0	2	1	98	49	0.060734355	0.197717869	NA	NA	1	NA	0.333	0	0.834
SE	788	PARP10	chr8	-	143984007	143984104	143983166	143983811	143984209	143984431	0	0	4	0	0	0	0	4	0	0	0	1	98	49	0.060734355	0.170760496	NA	NA	1	NA	1	0	0.5
SE	5889	SIPA1L1	chr14	+	71723646	71723886	71709221	71709664	71724669	71724835	0	0	4	0	0	0	0	0	0	0	0	1	98	49	0.060734355	0.170760496	NA	NA	1	NA	NA	0	1
A5SS	3026	ABCG1	chr21	+	42287888	42288073	42287888	42288037	42288210	42288312	0	2	3	0	0	2	0	8	2	0	0	0	85	49	0.060789913	0.170414363	NA	1	0.464	NA	1	1	-0.268
SE	4502	PTPN6	chr12	+	6954804	6954994	6951982	6952177	6955150	6955267	0	2	31	0	0	0	0	21	1	0	1	0	98	49	0.061131927	0.170760496	NA	1	1	NA	0.913	1	0.043
SE	10395	HES4	chr1	-	999691	999787	999525	999613	999865	1000172	0	0	0	0	1	1	0	1	0	0	4	1	98	49	0.061306069	0.170760496	NA	0	0	NA	0.111	0	-0.056
RI	672	PDE4D	chr5	-	59038858	59039512	59038858	59038971	59039057	59039512	0	0	1	0	0	0	3	3	0	0	0	2	98	49	0.061401334	0.297702651	NA	NA	1	1	1	0	0.333
SE	7868	VRK3	chr19	-	49989638	49989771	49988371	49988492	49992859	49992952	0	1	2	0	0	0	0	0	0	0	2	0	98	49	0.061401334	0.170760496	NA	1	1	NA	0	NA	1
SE	8054	CUL4B	chrX	-	120544480	120544643	120544113	120544203	120545443	120545517	0	0	1	0	0	0	0	1	0	0	0	2	98	49	0.061401334	0.170760496	NA	NA	1	NA	1	0	0.5
SE	11362	RSRC2	chr12	-	122520503	122520581	122518838	122519029	122521384	122521428	0	1	3	0	0	1	0	0	0	0	2	0	98	49	0.061401334	0.170760496	NA	1	0.6	NA	0	NA	0.8
SE	12007	DHX8	chr17	+	43508338	43508520	43507808	43508019	43513361	43513502	0	1	1	0	0	0	0	0	0	0	2	0	98	49	0.061401334	0.170760496	NA	1	1	NA	0	NA	1
SE	9457	PFKFB3	chr10	+	6221640	6221745	6221380	6221527	6222854	6222984	0	16	35	0	0	0	0	44	26	0	1	0	98	49	0.0614161	0.170760496	NA	1	1	NA	0.957	1	0.022
A3SS	3638	MAP2K7	chr19	+	7911414	7911578	7911435	7911578	7911249	7911330	0	0	0	0	1	2	0	0	2	0	0	0	70	49	0.061460457	0.197717869	NA	0	0	NA	NA	1	-1
A5SS	725	FAM193A	chr4	+	2694945	2695142	2694945	2695129	2696362	2696593	0	0	0	0	2	0	0	2	0	0	0	0	62	49	0.061467502	0.170414363	NA	0	NA	NA	1	NA	-1
A3SS	16	ALG13	chrX	+	111682131	111682294	111682235	111682294	111681169	111681424	0	0	0	0	2	0	0	2	0	0	0	0	98	49	0.061489123	0.197717869	NA	0	NA	NA	1	NA	-1
A5SS	1081	WIPI2	chr7	+	5193117	5193345	5193117	5193171	5199575	5199658	0	0	0	0	0	2	0	0	2	0	0	0	98	49	0.061489123	0.170414363	NA	NA	0	NA	NA	1	-1
RI	843	FAM96A	chr15	-	64072558	64075537	64072558	64073028	64075491	64075537	0	0	0	0	0	2	1	0	2	0	1	0	98	49	0.061489123	0.297702651	NA	NA	0	1	0	1	-0.667
SE	4053	CASC3	chr17	+	40167849	40167948	40167497	40167612	40168202	40168417	0	0	5	0	2	0	0	2	0	0	0	0	98	49	0.061489123	0.170760496	NA	0	1	NA	1	NA	-0.5
SE	10886	AKAP13	chr15	+	85662387	85662453	85658536	85658590	85664562	85664755	0	0	2	0	2	11	0	2	0	0	0	0	98	49	0.061489123	0.170760496	NA	0	0.083	NA	1	NA	-0.959
SE	11013	RHOT2	chr16	+	669552	669606	668655	668699	670122	670175	0	0	1	0	2	0	0	2	0	0	0	0	98	49	0.061489123	0.170760496	NA	0	1	NA	1	NA	-0.5
SE	11296	CREBBP	chr16	-	3791980	3792094	3782683	3782926	3793385	3793626	0	1	9	0	0	0	0	3	1	0	1	0	98	49	0.061515403	0.170760496	NA	1	1	NA	0.6	1	0.2
A3SS	3506	DYRK1B	chr19	-	39826671	39826987	39826671	39826867	39827284	39827425	0	0	3	0	0	0	0	0	0	0	2	1	98	49	0.06170155	0.197717869	NA	NA	1	NA	0	0	1
A5SS	1533	BTF3	chr5	+	73498424	73498799	73498424	73498558	73499133	73499202	3	0	2	0	0	2	0	2	0	1	3	0	98	49	0.06170155	0.170414363	1	NA	0.333	0	0.25	NA	0.541
SE	7580	MMP8	chr11	-	102722990	102723078	102722428	102722673	102724753	102724954	0	3	3	0	0	1	0	0	0	0	1	0	98	49	0.06170155	0.170760496	NA	1	0.6	NA	0	NA	0.8
SE	7581	MMP8	chr11	-	102722990	102723081	102722428	102722673	102724753	102724954	0	3	3	0	0	1	0	0	0	0	1	0	98	49	0.06170155	0.170760496	NA	1	0.6	NA	0	NA	0.8
SE	8154	CD302	chr2	-	159783358	159783469	159780881	159780998	159798131	159798255	0	0	3	0	0	0	0	3	0	0	0	1	98	49	0.06170155	0.170760496	NA	NA	1	NA	1	0	0.5
SE	9121	KDM4B	chr19	+	5071009	5071059	5047475	5047669	5077366	5077470	0	0	3	0	0	0	0	4	0	0	0	1	98	49	0.06170155	0.170760496	NA	NA	1	NA	1	0	0.5
SE	10516	ZNF316	chr7	+	6642381	6642764	6641824	6641962	6642963	6643073	0	3	0	0	0	0	0	0	0	0	1	0	98	49	0.06170155	0.170760496	NA	1	NA	NA	0	NA	1
SE	11106	MGRN1	chr16	+	4680031	4680097	4677462	4677572	4681549	4681776	0	0	3	0	0	0	0	1	0	0	0	1	98	49	0.06170155	0.170760496	NA	NA	1	NA	1	0	0.5
A3SS	910	LIME1	chr20	+	63737972	63738060	63738007	63738060	63737820	63737902	0	0	1	0	1	0	0	3	0	0	0	0	84	49	0.061899751	0.197717869	NA	0	1	NA	1	NA	-0.5
A3SS	2475	ZBTB17	chr1	-	15946153	15946316	15946153	15946294	15946934	15947123	0	0	1	0	1	2	0	3	0	0	0	0	71	49	0.061903	0.197717869	NA	0	0.257	NA	1	NA	-0.871
A3SS	122	FAM219A	chr9	-	34398183	34401122	34398183	34401119	34401665	34401720	0	0	0	0	1	0	0	3	0	0	0	0	52	49	0.061903774	0.197717869	NA	0	NA	NA	1	NA	-1
A3SS	3899	PPP1R12C	chr19	-	55092782	55092868	55092782	55092865	55093015	55093076	0	0	0	0	0	1	0	0	3	0	1	0	52	49	0.061903774	0.197717869	NA	NA	0	NA	0	1	-0.5
RI	528	NFKB2	chr10	+	102395595	102395980	102395595	102395796	102395887	102395980	0	0	0	0	0	1	0	5	3	0	0	0	98	49	0.061911422	0.297702651	NA	NA	0	NA	1	1	-1
SE	4260	SLC22A4	chr5	+	132312160	132312264	132294451	132295009	132313613	132313768	0	0	4	0	1	0	0	3	0	0	0	0	98	49	0.061911422	0.170760496	NA	0	1	NA	1	NA	-0.5
SE	5368	BAZ1A	chr14	-	34826012	34826156	34810934	34811036	34874491	34874662	0	0	13	0	1	0	2	3	0	0	0	0	98	49	0.061911422	0.170760496	NA	0	1	1	1	NA	-0.5
SE	6045	GADD45A	chr1	+	67686349	67686587	67685176	67685538	67687660	67688338	0	0	0	0	0	1	0	1	3	0	0	0	98	49	0.061911422	0.170760496	NA	NA	0	NA	1	1	-1
SE	12169	HDAC7	chr12	-	47786584	47786703	47785751	47785885	47787711	47787809	0	0	4	0	1	1	0	3	0	0	0	0	98	49	0.061911422	0.170760496	NA	0	0.667	NA	1	NA	-0.666
SE	12235	FAM104A	chr17	-	73227164	73227264	73207352	73209768	73232085	73232394	0	0	1	0	1	0	0	3	0	0	0	0	98	49	0.061911422	0.170760496	NA	0	1	NA	1	NA	-0.5
A5SS	2118	CSNK1A1	chr5	-	149505446	149505595	149505482	149505595	149495893	149496860	3	0	11	0	0	1	0	5	0	1	1	0	85	49	0.062188913	0.170414363	1	NA	0.864	0	0.742	NA	0.561
SE	12245	DYRK1B	chr19	-	39826951	39826987	39826671	39826867	39827284	39827425	0	0	3	0	0	0	0	0	0	0	2	1	85	49	0.062188913	0.170760496	NA	NA	1	NA	0	0	1
A3SS	2080	AP2S1	chr19	-	46839464	46839620	46839464	46839578	46845992	46846142	0	0	0	0	0	5	0	0	1	5	2	0	91	49	0.062394347	0.197717869	NA	NA	0	0	0	1	-0.333
SE	4424	PREX1	chr20	-	48658135	48658228	48657039	48657188	48666282	48666355	0	2	10	0	0	0	0	20	2	0	1	0	98	49	0.062524268	0.170760496	NA	1	1	NA	0.909	1	0.045
SE	2274	VNN3	chr6	-	132728740	132728933	132724605	132724979	132734252	132734383	0	1	5	0	0	0	0	9	1	0	1	0	98	49	0.062727245	0.170760496	NA	1	1	NA	0.818	1	0.091
SE	13163	PPP1CB	chr2	+	28752037	28752176	28751747	28751820	28776850	28776982	0	2	2	0	0	0	1	4	3	0	1	0	98	49	0.06302451	0.170760496	NA	1	1	1	0.667	1	0.111
A3SS	2776	MYADM	chr19	+	53869689	53869838	53869753	53869838	53867864	53867943	0	0	2	0	0	0	3	0	0	0	0	1	98	49	0.063117334	0.197717869	NA	NA	1	1	NA	0	0.5
A5SS	1568	POLD4	chr11	-	67353302	67353596	67353363	67353596	67352987	67353077	0	1	2	0	0	0	0	0	0	0	0	1	98	49	0.063117334	0.170414363	NA	1	1	NA	NA	0	1
SE	498	NAPRT	chr8	-	143577061	143577177	143576645	143576842	143577268	143577399	0	2	4	0	0	0	0	0	0	0	1	0	98	49	0.063117334	0.170760496	NA	1	1	NA	0	NA	1
SE	3230	TMEM189	chr20	-	50130855	50130947	50129545	50129690	50143501	50143621	0	2	1	0	0	0	0	0	0	0	1	0	98	49	0.063117334	0.170760496	NA	1	1	NA	0	NA	1
SE	3231	TMEM189	chr20	-	50130864	50130947	50129545	50129690	50143501	50143621	0	2	0	0	0	0	0	0	0	0	1	0	98	49	0.063117334	0.170760496	NA	1	NA	NA	0	NA	1

SE	3810	CPNE1	chr20	-	35658929	35658982	35632794	35632923	35664759	35664937	0	0	2	0	0	0	0	0	0	0	0	1	98	49	0.063117334	0.170760496	NA	NA	1	NA	NA	0	1
SE	3811	CPNE1	chr20	-	35658929	35659014	35632794	35632923	35664759	35664956	0	0	2	0	0	0	0	0	0	0	0	1	98	49	0.063117334	0.170760496	NA	NA	1	NA	NA	0	1
SE	6736	MTOR	chr1	-	11256932	11257165	11255991	11256192	11258484	11258593	0	2	4	0	0	0	0	0	0	0	1	0	98	49	0.063117334	0.170760496	NA	1	1	NA	0	NA	1
SE	6925	DMTN	chr8	+	22067084	22067159	22066704	22066893	22067526	22067682	0	2	1	0	0	0	0	0	0	0	1	0	98	49	0.063117334	0.170760496	NA	1	1	NA	0	NA	1
SE	9827	SHISA5	chr3	-	48503094	48503193	48501136	48501293	48504422	48504814	0	0	2	0	0	0	0	0	0	0	0	1	98	49	0.063117334	0.170760496	NA	NA	1	NA	NA	0	1
SE	10041	SYNE2	chr14	+	64031015	64031357	64027483	64027793	64047999	64048155	0	2	0	0	0	0	0	0	0	0	1	0	98	49	0.063117334	0.170760496	NA	1	NA	NA	0	NA	1
SE	10233	SYNE3	chr14	-	95467794	95467967	95465930	95466240	95475677	95475836	0	2	0	0	0	0	0	0	0	0	1	0	98	49	0.063117334	0.170760496	NA	1	NA	NA	0	NA	1
SE	12575	ARID3A	chr19	+	964832	965080	964247	964431	966571	966868	0	2	4	0	0	0	0	0	0	0	1	0	98	49	0.063117334	0.170760496	NA	1	1	NA	0	NA	1
SE	13045	CNOT7	chr8	-	17232426	17232537	17229230	17230848	17234715	17234860	0	2	2	0	0	0	0	0	0	0	1	0	98	49	0.063117334	0.170760496	NA	1	1	NA	0	NA	1
A3SS	1438	FGD4	chr12	+	32607937	32608095	32607956	32608095	32602160	32602317	1	0	0	0	0	4	0	2	1	0	0	0	68	49	0.063176479	0.197717869	1	NA	0	NA	1	1	-0.5
A5SS	1600	TRAPPC3	chr1	-	36149091	36149514	36149336	36149514	36140068	36140166	0	0	0	0	4	0	8	1	0	0	0	0	98	49	0.063182848	0.170414363	NA	0	NA	1	1	NA	-1
SE	5519	DENND5A	chr11	-	9203659	9204317	9193493	9193681	9206672	9206782	0	2	20	0	0	0	0	18	2	0	1	0	98	49	0.063221519	0.170760496	NA	1	1	NA	0.9	1	0.05
SE	7498	IFI44	chr1	+	78662703	78662878	78659311	78659483	78663764	78664078	0	6	51	0	1	1	0	87	6	0	0	0	98	49	0.063259916	0.170760496	NA	0.75	0.962	NA	1	1	-0.144
SE	12385	JAK3	chr19	-	17831227	17831400	17830502	17830620	17831673	17831798	0	2	10	0	0	0	0	7	3	0	1	0	98	49	0.063527186	0.170760496	NA	1	1	NA	0.778	1	0.111
A3SS	3618	CYB561D1	chr1	+	109495678	109500441	109495755	109500441	109495142	109495180	0	0	0	0	0	1	0	0	2	0	0	0	98	49	0.063770311	0.197717869	NA	NA	0	NA	NA	1	-1
A5SS	1161	GAPDH	chr12	+	6534404	6534661	6534404	6534569	6534809	6534861	0	0	0	0	0	1	0	0	2	0	1	0	98	49	0.063770311	0.170414363	NA	NA	0	NA	0	1	-0.5
A5SS	2679	OXLD1	chr17	-	81666145	81666637	81666517	81666637	81665035	81665584	0	0	0	0	0	1	0	0	2	0	0	0	98	49	0.063770311	0.170414363	NA	NA	0	NA	NA	1	-1
A5SS	2683	OXLD1	chr17	-	81666149	81666637	81666517	81666637	81665035	81665584	0	0	0	0	0	1	0	0	2	0	0	0	98	49	0.063770311	0.170414363	NA	NA	0	NA	NA	1	-1
A5SS	2688	OXLD1	chr17	-	81666163	81666637	81666517	81666637	81665035	81665584	0	0	0	0	0	1	0	0	2	0	0	0	98	49	0.063770311	0.170414363	NA	NA	0	NA	NA	1	-1
A5SS	2689	OXLD1	chr17	-	81666276	81666637	81666517	81666637	81665035	81665584	0	0	0	0	0	1	0	0	2	0	0	0	98	49	0.063770311	0.170414363	NA	NA	0	NA	NA	1	-1
A5SS	2944	UNKL	chr16	-	1369933	1370357	1370129	1370357	1367655	1367858	0	1	0	0	0	1	0	0	2	0	0	0	98	49	0.063770311	0.170414363	NA	1	0	NA	NA	1	-0.5
RI	746	PTPMT1	chr11	+	47565429	47565986	47565429	47565796	47565905	47565986	1	0	2	0	1	0	0	2	0	0	0	0	98	49	0.063770311	0.297702651	1	0	1	NA	1	NA	-0.333
RI	790	CCDC125	chr5	-	69320236	69320580	69320236	69320345	69320464	69320580	0	0	0	0	0	1	1	1	2	0	0	0	98	49	0.063770311	0.297702651	NA	NA	0	1	1	1	-1
SE	255	SEC16A	chr9	-	136468414	136468512	136466956	136467083	136471974	136472111	0	0	1	0	1	0	0	2	0	0	0	0	98	49	0.063770311	0.170760496	NA	0	1	NA	1	NA	-0.5
SE	506	SLC45A4	chr8	-	141221576	141221765	141219649	141219829	141253988	141254629	0	0	5	0	1	0	2	2	0	0	0	0	98	49	0.063770311	0.170760496	NA	0	1	1	1	NA	-0.5
SE	3157	XBP1	chr22	-	28796046	28796192	28794559	28795732	28797076	28797205	0	0	6	0	1	0	0	2	0	0	0	0	98	49	0.063770311	0.170760496	NA	0	1	NA	1	NA	-0.5
SE	3781	MYCBP2	chr13	-	77126317	77126542	77125335	77125468	77139195	77139336	0	0	2	0	1	0	0	2	0	0	0	0	98	49	0.063770311	0.170760496	NA	0	1	NA	1	NA	-0.5
SE	5029	BRMS1	chr11	-	66340120	66340213	66338720	66338785	66340773	66340870	0	0	2	0	1	0	0	2	0	0	0	0	98	49	0.063770311	0.170760496	NA	0	1	NA	1	NA	-0.5
SE	10606	ANKFY1	chr17	-	4189381	4189479	4184817	4185046	4194977	4195177	0	0	0	0	0	1	0	0	2	0	0	0	98	49	0.063770311	0.170760496	NA	NA	0	NA	NA	1	-1
SE	13383	ZNF805	chr19	+	57243922	57244049	57240684	57240921	57248604	57248700	0	0	0	0	1	0	0	2	0	0	0	0	98	49	0.063770311	0.170760496	NA	0	NA	NA	1	NA	-1
SE	12368	NBEAL2	chr3	+	47007065	47007155	47006332	47006449	47007240	47007350	0	2	16	0	0	0	0	8	3	0	1	0	98	49	0.063873063	0.170760496	NA	1	1	NA	0.8	1	0.1
SE	8540	ARAP3	chr5	-	141655361	141655400	141653400	141654435	141655620	141655758	0	0	0	0	1	2	0	2	0	0	0	0	88	49	0.063945674	0.170760496	NA	0	0	NA	1	NA	-1
A3SS	530	GLRX	chr5	-	95813848	95814807	95813848	95814389	95816506	95816626	0	0	0	0	0	3	0	0	1	0	1	0	98	49	0.064268453	0.197717869	NA	NA	0	NA	0	1	-0.5
A3SS	531	GLRX	chr5	-	95813848	95814957	95813848	95814389	95816506	95816626	0	0	0	0	0	3	0	0	1	0	1	0	98	49	0.064268453	0.197717869	NA	NA	0	NA	0	1	-0.5
SE	5261	PCSK7	chr11	-	117208896	117209053	117207969	117208064	117218465	117218568	0	0	0	0	0	3	0	0	1	0	0	0	98	49	0.064268453	0.170760496	NA	NA	0	NA	NA	1	-1
SE	6387	PSRC1	chr1	-	109280395	109280489	109279553	109280156	109280776	109280916	0	0	1	0	3	0	0	1	0	0	0	0	98	49	0.064268453	0.170760496	NA	0	1	NA	1	NA	-0.5
A5SS	1969	SLC35A2	chrX	-	48911506	48911958	48911545	48911958	48909813	48909996	0	0	0	0	0	3	0	0	1	0	0	0	88	49	0.064272896	0.170414363	NA	NA	0	NA	NA	1	-1
A5SS	1774	SF1	chr11	-	64767566	64767844	64767570	64767844	64767191	64767251	0	0	0	0	0	3	6	0	1	0	2	0	53	49	0.064284969	0.170414363	NA	NA	0	1	0	1	-0.667
SE	10332	CYTIP	chr2	-	157434369	157434424	157430859	157430962	157434697	157434747	0	9	93	0	0	0	18	103	8	0	1	0	98	49	0.064528457	0.170760496	NA	1	1	1	0.981	1	0.006
SE	11921	RBM39	chr20	-	35729461	35729527	35729311	35729365	35731940	35732135	0	3	27	0	0	0	2	37	2	0	1	0	98	49	0.064578009	0.170760496	NA	1	1	1	0.949	1	0.017
SE	8709	CD74	chr5	-	150403120	150403312	150402562	150402625	150404679	150404767	0	2	7	0	2	32	1	1	0	0	20	0	98	49	0.064910289	0.170760496	NA	0.333	0.099	1	0.024	NA	-0.296
SE	250	NOTCH1	chr9	-	136515980	136516094	136515482	136515716	136517271	136517385	1	1	10	0	0	0	0	2	1	0	1	0	98	49	0.065113604	0.170760496	1	1	1	NA	0.5	1	0.25
SE	6697	FCGR2A	chr1	+	161506333	161506591	161505986	161506007	161509819	161510074	0	2	42	0	0	0	1	17	3	0	1	0	98	49	0.065339874	0.170760496	NA	1	1	1	0.895	1	0.035
SE	12941	SMCHD1	chr18	+	2694526	2694693	2688627	2688747	2697031	2697122	0	3	97	0	0	0	0	55	1	0	1	0	98	49	0.065369701	0.170760496	NA	1	1	NA	0.965	1	0.018
A3SS	2057	FBXO7	chr22	+	32478980	32479275	32479132	32479275	32474719	32475124	0	1	1	0	0	0	0	0	0	0	1	0	98	49	0.065790241	0.197717869	NA	1	1	NA	0	NA	1
A3SS	2058	FBXO7	chr22	+	32478980	32479275	32479132	32479275	32475236	32475398	0	1	1	0	0	0	0	0	0	0	1	0	98	49	0.065790241	0.197717869	NA	1	1	NA	0	NA	1
A3SS	2827	APOL3	chr22	-	36149045	36149371	36149045	36149142	36160668	36160929	1	1	0	0	0	0	0	0	0	0	1	0	98	49	0.065790241	0.197717869	1	1	NA	NA	0	NA	1
A3SS	3029	POLM	chr7	-	44072061	44073430	44072061	44073377	44073624	44073708	1	0	1	0	0	0	0	3	0	0	0	1	98	49	0.065790241	0.197717869	1	NA	1	NA	1	0	0.5
A5SS	1566	POLD4	chr11	-	67353293	67353596	67353363	67353596	67352987	67353077	0	1	1	0	0	0	0	0	0	0	0	1	98	49	0.065790241	0.170414363	NA	1	1	NA	NA	0	1
A5SS	2228	USP8	chr15	+	50424376	50424654	50424376	50424514	50439008	50439177	0	0	1	0	0	0	0	0	0	0	3	1	98	49	0.065790241	0.170414363	NA	NA	1	NA	0	0	1
A5SS	2719	NBPF11	chr1	-	148110377	148110541	148110457	148110541	148109283	148109335	0	1	0	0	0	0	0	0	0	0	1	1	98	49	0.065790241	0.170414363	NA	1	NA	NA	0	0	1
SE	1021	PPIA	chr7	+	44798747	44798904	44796635	44796793	44799246	44799277	0	0	1	0	0	0	0	0	0	0	1	1	98	49	0.065790241	0.170760496	NA	NA	1	NA	0	0	1
SE	2879	ACO2	chr22	+	41522829	41522987	41520170	41520276	41523204	41523278	0	1	2	0	0	0	0	0	0	0	1	0	98	49	0.065790241	0.170760496	NA	1	1	NA	0	NA	1
SE	4101	FAM13B	chr5	-	138018954	138019146	138018301	138018514	138021030	138021197	0	0	1	0	0	0	0	1	0	3	0	1	98	49	0.065790241	0.170760496	NA	NA	1	0	1	0	0.667
SE	4610	HSPA8	chr11	-	123060592	123060798	123060115	123060268	123061119	123061329	0	0	1	0	0	0	0	0	0	0	2	1	98	49	0.065790241	0.170760496	NA	NA	1	NA	0	0	1
SE	6531	FRMD8	chr11	+	65393572	65393674	65389360	65389528	65394040	65394099	0	1	3	0	0	0	0	0	0	0	1	0	98	49	0.065790241	0.170760496	NA	1	1	NA	0	NA	1
SE	7131	GGA3	chr17	-	75243062	75243166	75242830	75242911	75243446	75243570	0	1	5	0	0	0	0	0	0	0	1	0	98	49	0.065790241	0.170760496	NA	1	1	NA	0	NA	1
SE	7595	ATG16L2	chr11	+	72826702	72826823	72826172	72826243	72828358	72828508	0	0	1	0	0	0	0	2	0	0	1	1	98	49	0.065790241	0.170760496	NA	NA	1	NA	0.5	0	0.75
SE	8118	POLE	chr12	-	132648928	132649072	132643836	132643977	132649305	132649515	0	1	1	0	0	0	0	0	0	0	1	0	98	49	0.065790241	0.170760496	NA	1	1	NA	0	NA	1
SE	8179	MLXIP	chr12	+	122137468	122137590	122135488	122135666	122138193	122138295	0	0	1	0	0	0	0	0	0	0	0	1	98	49	0.065790241	0.170760496	NA	NA	1	NA	NA	0	1
SE	8364	STRAP	chr12	+	15883540	15883676	15882353	15882819	15889927	15890009	0	0	1	0	0	0	0	2	0	0	1	1	98	49	0.065790241	0.170760496	NA	NA	1	NA	0.5	0	0.75
SE	10100	GGA1	chr22	+	37625796	37625949	37624968	37625076	37629461	37629526	0	1	1	0	0	0	0	0	0	0	1	0	98	49	0.065790241	0.170760496	NA	1	1	NA	0	NA	1
SE	10460	OSBPL11	chr3	-	125547405	125547592	125538450	125538633	125560378	125560519	0	1	2	0	0	0	0	0	0	0	1	0	98	49	0.065790241	0.170760496	NA	1	1	NA	0	NA	1
SE	10539	P4HA1	chr10	-	73051052	73051249	73046924	73047101	73053350	73053590	0	0	1	0	0	0	0	0	0	0	0	1	98	49	0.065790241	0.170760496	NA	NA	1	NA	NA	0	1
SE	11584	FANCA	chr16	-	89742799	89742938	89740803	89740866	89744958	89745071	0	1	1	0	0	0	0	0	0	0	1	0	98	49	0.065790241	0.170760496	NA	1	1	NA	0	NA	1
SE	13943	PDK1	chr2	+	172562740	172562800	172562219	172562291	172564977	172565073	0	1	0	0	0	1	0	0	0	0	1	0	98	49	0.065790241	0.170760496	NA	1	0	NA	0	NA	0.5
A3SS	2853	TGOLN2	chr2	-	85318017	85322799	85318017	85322741	85324914	85324998	0	0	0	0	0	2	0	2	1	2	4	0	98	49	0.0658956	0.197717869	NA	NA	0	0	0.2	1	-0.4
A5SS	1409	PHYKPL	chr5	-	178231668	178232822	178232491	178232822	178231404	178231523	0	0	3	0	2	0	0	1	0	0	0	0	98	49	0.0658956	0.170414363	NA	0	1	NA	1	NA	-0.5
SE	145	SNX12	chrX	-	71061842	71061967	71059246	71061118	71062853	71062949	0	0	3	0	2	0	0	1	0	0	0	0	98	49	0.0658956	0.170760496	NA	0	1	NA	1	NA	-0.5
SE	239	NDOR1	chr9	+	137215406	137215521	137215094	137215202	137215658	137215805	0	0	0	0	2	0	0	1	0	0	0	0	98	49	0.0658956	0.170760496	NA	0	NA	NA	1	NA	-1
SE	436	UBE2R2	chr9	+	33911963	33912098	33900173	33900271	33917017	33920403	0	0	6	0	2	1	0	1	0	0	0	0	98	49	0.0658956	0.170760496	NA	0	0.75	NA	1	NA	-0.625
SE	4582	ST3GAL4	chr11	+	126409267	126409411	126408306	126408496	126413504	126413648	0	0	3	0	2	0	0	1	0	0	0	0	98	49	0.0658956	0.170760496	NA	0	1	NA	1	NA	-0.5
SE	7326	SREBF1	chr17	-	17819012	17819234	17818259	17818374	17819319	17819454	0	0	0	0	2	0	0	1	0	0	0	0	98	49	0.0658956	0.170760496	NA	0	NA	NA	1	NA	-1
SE	8223	MAPK3	chr16	-	30118046	30118163	30117669	30117784	30118348	30118538	0	0	0	0	0	2	0	0	1	0	0	0	98	49	0.0658956	0.170760496	NA	NA	0	NA	NA	1	-1
SE	11719	CHD3	chr17	+	7907600	7907702	7907352	7907488	7907893	7908019	0	0	0	0	0	2	0	0	1	0	0	0	98	49	0.0658956	0.170760496	NA	NA	0	NA	NA	1	-1
A3SS	81	GBGT1	chr9	-	133156014	133156102	133156014	133156065	133161466	133161532	0	0	0	0	0	2	0	0	1	0	1	0	86	49	0.065901144	0.197717869	NA	NA	0	NA	0	1	-0.5
SE	4442	CORO7	chr16	-	4357967	4358103	4357167	4357259	4358366	4358483	0	1	4	0	0	0	5	8	2	0	1	0	98	49	0.067252723	0.171142152	NA	1	1	1	0.8	1	0.067
SE	8028	GNAS	chr20	+	58898940	58898985	58895611	58895684	58903530	58903585	0	1	3	0	0	2	0	0	0	0	1	0	94	49	0.067458351	0.171142152	NA	1	0.439	NA	0	NA	0.72
SE	911	LMTK2	chr7	+	98171540	98171674	98159337	98159425	98185050	98185135	0	1	2	0	0	0	0	3	2	0	2	0	98	49	0.068013122	0.171142152	NA	1	1	NA	0.429	1	0.285
SE	2940	LIMK2	chr22	+	31273451	31273507	31272529	31272704	31275150	31275308	0	13	40	0	0	0	3	93	36	0	1	0	98	49	0.068060835	0.171142152	NA	1	1	1	0.979	1	0.007
SE	2968	AP1B1	chr22	-	29329711	29329720	29327679	29328895	29330377	29330532	0	0	1	0	0	0	0	0	0	0	0	1	58	49	0.068063814	0.171142152	NA	NA	1	NA	NA	0	1
SE	12329	ARHGDIA	chr17	-	81869329	81869406	81869172	81869236	81869541	81869625	0	0	21	0	0	1	0	11	1	0	0	2	98	49	0.068249658	0.171142152	NA	NA	0.913	NA	1	0.2	0.313
SE	5783	DENND3	chr8	+	141138021	141138137	141136557	141136791	141151618	141151837	0	3	15	0	0	0	0	19	7	0	1	0	98	49	0.068459154	0.171142152	NA	1	1	NA	0.905	1	0.047
A3SS	1319	P2RX7	chr12	+	121162270	121162520	121162423	121162520	121160901	121160974	0	0	0	0	0	1	0	0	1	0	0	0	98	49	0.068700612	0.197717869	NA	NA	0	NA	NA	1	-1
A3SS	3364	CCL4L2	chr17	+	36212292	36212878	36212496	36212878	36211775	36211890	0	0	0	0	0	1	2	4	1	0	0	0	98	49	0.068700612	0.197717869	NA	NA	0	1	1	1	-1
A3SS	3368	CCL4L2	chr17	+	36212302	36212878	36212496	36212878	36211775	36211890	0	0	0	0	0	1	2	4	1	0	0	0	98	49	0.068700612	0.197717869	NA	NA	0	1	1	1	-1
A3SS	3371	CCL4L2	chr17	+	36212312	36212878	36212496	36212878	36211775	36211890	0	0	0	0	0	1	2	4	1	0	0	0	98	49	0.068700612	0.197717869	NA	NA	0	1	1	1	-1
A3SS	3373	CCL4L2	chr17	+	36212333	36212878	36212496	36212878	36211775	36211890	0	0	0	0	0	1	2	4	1	0	0	0	98	49	0.068700612	0.197717869	NA	NA	0	1	1	1	-1
A3SS	3374	CCL4L2	chr17	+	36212363	36212878	36212496	36212878	36211775	36211890	0	0	0	0	0	1	2	4	1	0	0	0	98	49	0.068700612	0.197717869	NA	NA	0	1	1	1	-1
A3SS	3842	CARKD	chr13	+	110638241	110639995	110638377	110639995	110637128	110637249	0	0	0	0	0	1	0	0	1	0	0	0	98	49	0.068700612	0.197717869	NA	NA	0	NA	NA	1	-1
A3SS	3925	C10orf128	chr10	-	49154723	49156259	49154723	49155909	49161598	49161622	0	0	0	0	0	1	7	0	1	0	0	0	98	49	0.068700612	0.197717869	NA	NA	0	1	NA	1	-1
A5SS	407	ARHGAP26	chr5	+	143207197	143207473	143207197	143207308	143213996	143214088	0	0	0	0	0	1	0	0	1	0	1	0	98	49	0.068700612	0.170414363	NA	NA	0	NA	0	1	-0.5
A5SS	1221	C6orf203	chr6	+	107028171	107028797	107028171	107028271	107039748	107040230	0	0	0	0	0	1	0	0	1	0	0	0	98	49	0.068700612	0.170414363	NA	NA	0	NA	NA	1	-1
A5SS	1816	POLR2F	chr22	+	37956772	37956901	37956772	37956842	37959345	37959476	0	0	0	0	0	1	0	0	1	2	0	0	98	49	0.068700612	0.170414363	NA	NA	0	0	NA	1	-0.5
A5SS	2218	TRIM69	chr15	+	44758612	44758854	44758612	44758803	44759639	44759662	0	0	0	0	0	1	0	2	1	0	0	0	98	49	0.068700612	0.170414363	NA	NA	0	NA	1	1	-1
RI	334	ARL6IP4	chr12	+	122982468	122982913	122982468	122982538	122982619	122982913	0	0	0	0	0	1	0	0	1	0	1	0	98	49	0.068700612	0.297702651	NA	NA	0	NA	0	1	-0.5
RI	369	RECQL	chr12	-	21501169	21501669	21501169	21501215	21501412	21501669	0	0	0	0	0	1	2	1	1	0	0	0	98	49	0.068700612	0.297702651	NA	NA	0	1	0.333	1	-0.778
RI	581	TK2	chr16	-	66548977	66550412	66548977	66549009	66549937	66550412	0	0	0	0	0	1	0	3	1	0	0	0	98	49	0.068700612	0.297702651	NA	NA	0	NA	1	1	-1
RI	682	NAP1L1	chr12	-	76045382	76048464	76045382	76047718	76048392	76048464	0	0	0	0	0	1	0	2	1	0	2	0	98	49	0.068700612	0.297702651	NA	NA	0	NA	0.333	1	-0.666
RI	968	CCL4L2	chr17	+	36211775	36212878	36211775	36211890	36212496	36212878	0	0	0	0	0	1	2	4	1	0	0	0	98	49	0.068700612	0.297702651	NA	NA	0	1	1	1	-1
RI	1065	CPT1B	chr22	-	50568860	50569421	50568860	50569081	50569335	50569421	0	0	0	0	2	1	0	0	1	2	0	0	98	49	0.068700612	0.297702651	NA	0	0	0	NA	1	-0.5
RI	1071	NFKBIB	chr19	+	38907398	38908894	38907398	38907659	38908730	38908894	0	0	0	0	0	1	0	2	1	0	0	0	98	49	0.068700612	0.297702651	NA	NA	0	NA	1	1	-1
RI	1115	DCTN1	chr2	-	74366243	74366620	74366243	74366375	74366458	74366620	0	0	1	0	1	1	1	1	0	0	0	0	98	49	0.068700612	0.297702651	NA	0	0.333	1	1	NA	-0.834
SE	689	PTK2B	chr8	+	27445793	27445919	27444205	27444271	27450748	27450895	0	0	0	0	1	2	0	1	0	0	0	0	98	49	0.068700612	0.171142152	NA	0	0	NA	1	NA	-1
SE	969	RHBDD2	chr7	+	75881364	75881486	75878998	75879260	75881828	75882236	0	0	2	0	1	0	2	1	0	0	0	0	98	49	0.068700612	0.171142152	NA	0	1	1	1	NA	-0.5
SE	3923	HAGH	chr16	-	1826506	1826602	1822864	1823037	1826711	1827194	0	0	0	0	1	0	0	1	0	0	0	0	98	49	0.068700612	0.171142152	NA	0	NA	NA	1	NA	-1
SE	3999	ASL	chr7	+	66086743	66086821	66086584	66086662	66087333	66087386	0	0	2	0	1	0	0	1	0	0	0	0	98	49	0.068700612	0.171142152	NA	0	1	NA	1	NA	-0.5
SE	4928	FCRL6	chr1	+	159808960	159809245	159808177	159808444	159809401	159809683	0	0	0	0	0	1	0	0	1	0	0	0	98	49	0.068700612	0.171142152	NA	NA	0	NA	NA	1	-1
SE	5379	SYNE1	chr6	-	152455885	152456044	152455425	152455590	152458756	152458930	0	0	1	0	1	0	0	1	0	0	0	0	98	49	0.068700612	0.171142152	NA	0	1	NA	1	NA	-0.5
SE	6602	CCDC88B	chr11	+	64351159	64351255	64349550	64349668	64351475	64351616	0	0	0	0	0	1	0	5	1	0	0	0	98	49	0.068700612	0.171142152	NA	NA	0	NA	1	1	-1
SE	6680	TTN-AS1	chr2	+	178571299	178571449	178542221	178542361	178597595	178597681	0	0	2	0	1	0	0	1	0	0	0	0	98	49	0.068700612	0.171142152	NA	0	1	NA	1	NA	-0.5
SE	7590	UBE4B	chr1	+	10158355	10158482	10151325	10151561	10161141	10161286	0	0	0	0	0	1	0	0	1	0	1	0	98	49	0.068700612	0.171142152	NA	NA	0	NA	0	1	-0.5
SE	10301	MARK3	chr14	+	103491776	103492034	103480386	103480490	103502881	103503829	0	0	0	0	0	1	0	1	1	0	0	0	98	49	0.068700612	0.171142152	NA	NA	0	NA	1	1	-1
SE	11137	CLEC16A	chr16	+	10977224	10977399	10972931	10973061	10979328	10979382	0	0	0	0	1	0	0	1	0	0	0	0	98	49	0.068700612	0.171142152	NA	0	NA	NA	1	NA	-1
SE	11367	LONP2	chr16	+	48256609	48256741	48252130	48252365	48258617	48258740	0	0	0	0	0	1	0	0	1	0	1	0	98	49	0.068700612	0.171142152	NA	NA	0	NA	0	1	-0.5
SE	12842	SMARCA4	chr19	+	11033766	11033865	11033289	11033517	11034122	11034200	0	0	0	0	0	1	0	0	1	0	0	0	98	49	0.068700612	0.171142152	NA	NA	0	NA	NA	1	-1
SE	13356	TMEM86B	chr19	-	55228190	55228437	55226633	55227563	55228690	55229264	0	0	1	0	1	0	0	1	0	0	0	0	98	49	0.068700612	0.171142152	NA	0	1	NA	1	NA	-0.5
SE	13996	QRICH1	chr3	-	49092396	49092456	49076708	49077038	49093911	49094071	0	0	0	0	1	0	0	1	0	0	0	0	98	49	0.068700612	0.171142152	NA	0	NA	NA	1	NA	-1
SE	14058	ATG2B	chr14	-	96322111	96322254	96317697	96317855	96322539	96322735	0	0	0	0	1	0	0	1	0	0	0	0	98	49	0.068700612	0.171142152	NA	0	NA	NA	1	NA	-1
A5SS	1424	C7orf49	chr7	-	135168748	135169060	135168785	135169060	135167731	135167807	0	0	0	0	0	1	0	6	1	0	0	0	86	49	0.068703243	0.170414363	NA	NA	0	NA	1	1	-1
A5SS	3076	NCEH1	chr3	-	172647861	172648114	172647885	172648114	172645622	172645692	0	0	0	0	0	1	0	0	1	0	0	0	73	49	0.06870403	0.170414363	NA	NA	0	NA	NA	1	-1
A5SS	903	ATF7	chr12	-	53543296	53543448	53543329	53543448	53537414	53537552	1	0	0	0	0	1	0	0	1	0	0	0	82	49	0.068705526	0.170414363	1	NA	0	NA	NA	1	-0.5
A3SS	2808	SRRT	chr7	+	100885212	100885370	100885215	100885370	100884922	100885040	0	0	0	0	0	1	0	4	1	0	2	0	52	49	0.068726673	0.197717869	NA	NA	0	NA	0.653	1	-0.827

A3SS	3999	SERPINA1	chr14	-	94388559	94388663	94388559	94388660	94390456	94390692	0	0	5	0	1	3	0	1	0	4	0	0	52	49	0.068726673	0.197717869	NA	0	0.611	0	1	NA	-0.195
SE	9686	IFRD1	chr7	+	112450507	112450782	112423143	112423432	112455762	112455867	0	3	58	0	0	0	0	33	5	0	1	0	98	49	0.068775688	0.171142152	NA	1	1	NA	0.943	1	0.028
A5SS	2089	CLASRP	chr19	+	45068014	45068058	45068014	45068054	45068419	45068480	0	0	0	0	0	1	0	1	1	0	0	0	53	49	0.068946269	0.170414363	NA	NA	0	NA	1	1	-1
SE	11969	RARA	chr17	+	40348315	40348464	40342018	40342867	40349783	40349925	0	2	18	0	0	0	0	9	4	0	1	0	98	49	0.069039705	0.17126162	NA	1	1	NA	0.818	1	0.091
SE	3617	NIN	chr14	-	50772300	50772468	50771331	50771468	50772948	50773095	0	1	12	0	0	0	0	13	1	0	1	0	98	49	0.069097631	0.17126162	NA	1	1	NA	0.867	1	0.067
SE	784	ZYX	chr7	+	143388218	143388339	143382800	143383322	143388488	143388658	0	5	34	0	0	0	0	9	7	0	1	0	98	49	0.069165538	0.17126162	NA	1	1	NA	0.818	1	0.091
A3SS	2597	FERMT3	chr11	+	64219878	64220015	64219890	64220015	64219739	64219789	0	2	1	2	8	9	0	0	0	3	6	6	61	49	0.069201254	0.197717869	0	0.167	0.082	0	0	0	0.083
SE	2329	ATP11B	chr3	+	182842074	182842122	182837070	182837174	182845457	182845522	0	1	22	0	0	0	0	6	2	0	1	0	97	49	0.069320212	0.171362302	NA	1	1	NA	0.752	1	0.124
SE	5624	ETF1	chr5	-	138517560	138517700	138513567	138513706	138518691	138518867	0	0	12	0	2	0	0	10	1	0	1	0	98	49	0.069479149	0.17144651	NA	0	1	NA	0.833	1	-0.416
SE	1355	MAPK14	chr6	+	36102570	36102649	36095986	36096066	36107454	36107628	0	1	19	0	0	0	0	14	1	0	1	0	98	49	0.069582416	0.17144651	NA	1	1	NA	0.875	1	0.062
SE	4942	ARAP1	chr11	-	72695717	72695865	72695541	72695628	72696548	72696654	0	1	24	0	0	0	0	15	1	0	1	0	98	49	0.06991611	0.171507349	NA	1	1	NA	0.882	1	0.059
SE	10851	IRF1	chr5	-	132489391	132489483	132486953	132487130	132490544	132490773	0	0	6	0	0	2	0	8	2	0	2	7	98	49	0.069995497	0.171507349	NA	NA	0.6	NA	0.667	0.125	0.204
SE	4877	TPP1	chr11	-	6618775	6618915	6617625	6617776	6619195	6619267	1	2	13	0	0	0	0	1	1	0	1	0	98	49	0.07001504	0.171507349	1	1	1	NA	0.333	1	0.334
SE	6551	SESN2	chr1	+	28271673	28271871	28269182	28269248	28272283	28272466	0	1	1	0	0	0	0	7	4	0	1	0	98	49	0.070207347	0.171507349	NA	1	1	NA	0.778	1	0.111
SE	8748	ADIPOR1	chr1	-	202954240	202954307	202950929	202951164	202958184	202958396	0	3	1	0	1	1	0	2	2	0	0	0	98	49	0.070257706	0.171507349	NA	0.6	0.333	NA	1	1	-0.533
SE	13036	VASP	chr19	+	45523840	45523877	45523643	45523695	45524096	45524142	0	5	37	0	0	3	0	33	3	0	0	0	86	49	0.070291768	0.171507349	NA	1	0.875	NA	1	1	-0.062
SE	7499	IFI44	chr1	+	78662703	78662878	78660553	78660654	78663764	78664078	0	6	52	0	0	2	0	85	6	0	0	0	98	49	0.070496338	0.171727708	NA	1	0.929	NA	1	1	-0.035
SE	12903	TYROBP	chr19	-	35907729	35907762	35907445	35907580	35908174	35908309	0	3	6	0	3	1	0	5	1	0	0	0	82	49	0.070693174	0.171928544	NA	0.374	0.782	NA	1	1	-0.422
A3SS	581	LRCH4	chr7	-	100574010	100575304	100574010	100574356	100575704	100575782	0	1	2	0	0	4	0	3	9	0	1	1	98	49	0.070727262	0.198669639	NA	1	0.2	NA	0.6	0.818	-0.109
SE	12640	CYTH4	chr22	+	37310987	37311064	37309211	37309323	37311455	37311527	0	2	30	0	0	0	0	38	1	0	1	0	98	49	0.07087784	0.172099182	NA	1	1	NA	0.95	1	0.025
SE	12746	DYSF	chr2	+	71656161	71656290	71643964	71644063	71658877	71659033	0	2	17	0	2	0	0	7	6	0	0	0	98	49	0.071270681	0.172773926	NA	0.333	1	NA	1	1	-0.334
SE	333	AKNA	chr9	-	114356862	114356969	114355924	114356136	114357920	114358167	0	1	3	0	0	0	6	8	3	0	1	0	98	49	0.071722008	0.173368597	NA	1	1	1	0.8	1	0.067
A3SS	3730	RASGRP4	chr19	-	38414847	38415123	38414847	38414916	38417051	38417168	0	2	16	0	0	0	1	4	3	0	1	0	98	49	0.071741814	0.198669639	NA	1	1	1	0.667	1	0.111
SE	4363	LRRK2	chr12	+	40313971	40314173	40310430	40310649	40315211	40315300	0	3	44	0	0	1	1	19	1	0	3	0	98	49	0.071746685	0.173368597	NA	1	0.957	1	0.76	1	0.058
A3SS	590	UBE2D3	chr4	-	102826484	102826844	102826484	102826636	102827591	102827948	0	5	24	0	0	2	2	32	7	0	0	0	98	49	0.073395949	0.200170771	NA	1	0.857	1	1	1	-0.072
SE	4359	LRRK2	chr12	+	40274582	40274727	40263788	40263901	40274853	40274993	0	1	45	0	0	0	0	7	3	0	1	0	98	49	0.073501365	0.177323519	NA	1	1	NA	0.778	1	0.111
SE	6869	ATG2A	chr11	-	64910615	64910708	64910039	64910195	64910806	64910954	0	8	7	0	0	0	8	26	23	0	1	0	98	49	0.07395786	0.178138883	NA	1	1	1	0.929	1	0.024
SE	1736	ARHGAP26	chr5	+	142901934	142902039	142894237	142894348	142903539	142903669	0	2	101	0	0	0	0	61	1	0	1	0	98	49	0.074241382	0.178535676	NA	1	1	NA	0.968	1	0.016
SE	8074	BCL2A1	chr15	-	79967636	79967692	79960889	79961174	79970699	79971301	0	0	2	0	1	19	0	0	0	1	40	1	98	49	0.074407684	0.178649759	NA	0	0.05	0	0	0	0.025
SE	13869	ACSL1	chr4	-	184788378	184788513	184783926	184783991	184788616	184788731	0	0	0	0	1	55	0	2	0	1	37	9	98	49	0.074750662	0.178951805	NA	0	0	0	0.026	0	-0.009
SE	11135	ECE1	chr1	-	21279190	21279332	21272698	21272911	21290069	21290156	0	2	12	0	0	0	0	8	5	0	1	0	98	49	0.074771613	0.178951805	NA	1	1	NA	0.8	1	0.1
SE	2078	HERC5	chr4	+	88472408	88472502	88470613	88470673	88475840	88476030	0	2	29	0	0	0	0	34	3	0	1	0	98	49	0.075329785	0.180001061	NA	1	1	NA	0.944	1	0.028
A5SS	2702	IFI6	chr1	-	27669220	27669346	27669244	27669346	27668457	27668535	0	0	5	0	1	20	0	0	0	0	9	1	73	49	0.075687381	0.183611979	NA	0	0.144	NA	0	0	0.072
SE	801	TBXAS1	chr7	+	139955458	139955607	139953367	139953456	139957633	139957764	0	1	15	0	0	0	0	3	2	0	1	0	98	49	0.07582917	0.180642255	NA	1	1	NA	0.6	1	0.2
SE	12943	SMCHD1	chr18	+	2722518	2722663	2718314	2718434	2724898	2724995	0	1	43	0	0	5	1	37	1	0	12	0	98	49	0.075958015	0.180642255	NA	1	0.811	1	0.607	1	0.036
SE	6058	HCLS1	chr3	-	121637145	121637256	121636433	121636489	121642926	121642981	0	0	9	0	1	0	1	4	1	0	0	0	98	49	0.075958686	0.180642255	NA	0	1	1	1	1	-0.5
RI	461	WTAP	chr6	+	159748190	159749421	159748190	159748369	159748589	159749421	0	1	13	0	0	0	3	9	5	0	2	0	98	49	0.076502744	0.314063896	NA	1	1	1	0.692	1	0.103
SE	11482	AP1G1	chr16	-	71789278	71789482	71774467	71774592	71808762	71809073	0	1	7	0	0	0	0	7	3	0	1	0	98	49	0.076633988	0.181960321	NA	1	1	NA	0.778	1	0.111
SE	4413	MEGF6	chr1	-	3496987	3497119	3496654	3496783	3497232	3497361	0	3	0	0	0	2	0	1	1	0	0	0	98	49	0.076772626	0.182001983	NA	1	0	NA	1	1	-0.5
SE	5396	MTMR14	chr3	+	9662266	9662375	9653620	9653769	9668718	9668794	0	0	1	0	1	0	0	1	3	0	0	0	98	49	0.077074472	0.182348728	NA	0	1	NA	1	1	-0.5
SE	5680	GMIP	chr19	-	19641809	19641867	19640445	19640571	19641975	19642051	0	1	27	0	0	3	0	15	6	0	0	0	98	49	0.077161537	0.182348728	NA	1	0.818	NA	1	1	-0.091
SE	6871	ATG2A	chr11	-	64914080	64914233	64913820	64913923	64914337	64914500	0	2	8	0	0	0	4	20	8	0	1	0	98	49	0.077718556	0.183327985	NA	1	1	1	0.909	1	0.03
SE	10716	GADD45B	chr19	+	2477028	2477251	2476528	2476630	2477487	2478259	0	1	6	0	0	1	0	22	4	0	0	0	98	49	0.077819863	0.183327985	NA	1	0.75	NA	1	1	-0.125
SE	10342	PDCD4	chr10	+	110887664	110887886	110885252	110885366	110889532	110889630	0	1	4	0	0	0	0	21	2	0	1	0	98	49	0.078019098	0.183509709	NA	1	1	NA	0.913	1	0.043
A3SS	1541	GMIP	chr19	-	19636706	19636796	19636706	19636787	19636916	19637029	0	1	9	0	0	0	0	5	2	0	1	0	58	49	0.078029018	0.208872272	NA	1	1	NA	0.809	1	0.095
SE	11436	PLK3	chr1	+	44803267	44803391	44802954	44803153	44803599	44803691	0	2	8	0	0	0	0	6	5	0	1	0	98	49	0.078722929	0.184875878	NA	1	1	NA	0.75	1	0.125
A3SS	1062	ATXN2L	chr16	+	28832186	28832399	28832204	28832399	28830961	28831072	0	0	1	0	1	0	0	0	0	0	1	2	67	49	0.078907303	0.208872272	NA	0	1	NA	0	0	0.5
SE	9029	MYD88	chr3	+	38140387	38140568	38138477	38139028	38140756	38140848	0	3	7	0	0	0	0	2	2	0	2	0	98	49	0.079080192	0.185334725	NA	1	1	NA	0.333	1	0.334
SE	9375	FMNL1	chr17	+	45236135	45236244	45234071	45234200	45237280	45237357	0	8	42	0	2	1	3	17	7	0	0	0	98	49	0.079164932	0.185334725	NA	0.667	0.955	1	1	1	-0.189
SE	4098	SETD1B	chr12	+	121819403	121819895	121817798	121817904	121822489	121823749	0	3	1	0	0	3	1	6	1	0	0	0	98	49	0.079530423	0.185900818	NA	1	0.143	1	1	1	-0.428
SE	5185	MYO9B	chr19	+	17159394	17159484	17156908	17157038	17162349	17162466	0	4	24	0	0	0	1	16	12	0	1	0	98	49	0.079811629	0.186229709	NA	1	1	1	0.889	1	0.037
SE	6698	FCGR2A	chr1	+	161506336	161506591	161505986	161506007	161509819	161510074	0	1	45	0	0	0	1	23	2	0	1	0	98	49	0.079918937	0.186229709	NA	1	1	1	0.92	1	0.027
SE	7345	FAM129A	chr1	-	184823637	184823742	184823166	184823329	184831846	184831962	0	2	32	0	0	4	1	19	1	0	0	0	98	49	0.080074419	0.186303176	NA	1	0.8	1	1	1	-0.1
SE	7416	VAV1	chr19	+	6822418	6822514	6822220	6822329	6825052	6825121	0	0	15	0	2	0	0	11	4	0	2	0	98	49	0.080433731	0.18684992	NA	0	1	NA	0.733	1	-0.367
SE	12584	SBNO2	chr19	-	1111018	1111093	1110744	1110888	1111505	1111614	0	1	6	0	0	1	1	26	2	0	0	0	98	49	0.080803043	0.187418168	NA	1	0.75	1	1	1	-0.125
SE	10297	TNFAIP2	chr14	+	103127004	103127629	103126326	103126692	103129739	103129854	0	2	36	0	0	0	0	35	5	0	1	0	98	49	0.081018716	0.187601066	NA	1	1	NA	0.946	1	0.027
SE	4657	PRKD2	chr19	-	46681649	46681748	46678395	46678663	46689536	46689698	0	1	4	0	0	0	1	17	4	0	1	0	98	49	0.081231413	0.187601066	NA	1	1	1	0.895	1	0.035
SE	11395	ADGRG3	chr16	+	57680504	57680617	57680264	57680365	57684389	57684483	0	3	11	0	0	0	0	12	14	0	0	1	98	49	0.081296035	0.187601066	NA	1	1	NA	1	0.875	0.062
SE	5698	RPLP2	chr11	+	812534	812633	811596	811645	812759	812876	0	2	43	0	0	0	0	41	4	0	1	0	98	49	0.081381168	0.187601066	NA	1	1	NA	0.953	1	0.024
SE	10951	IQGAP1	chr15	+	90482196	90482281	90481959	90482100	90483360	90483593	0	1	25	0	0	0	0	12	4	0	1	0	98	49	0.083074196	0.191210592	NA	1	1	NA	0.857	1	0.072
SE	3399	ELOVL5	chr6	-	53273219	53273344	53270592	53270727	53275089	53275261	0	1	21	0	0	0	3	9	4	0	1	0	98	49	0.083349562	0.191551057	NA	1	1	1	0.818	1	0.061
SE	5156	PSAP	chr10	-	71819030	71819111	71818616	71818724	71819464	71819622	1	15	181	0	0	0	10	44	9	0	1	0	98	49	0.084300652	0.193441038	1	1	1	1	0.957	1	0.014
SE	9929	PTPRE	chr10	+	128056122	128056213	128049529	128049666	128060938	128061015	1	1	31	0	0	0	0	31	1	0	1	0	98	49	0.084566773	0.193502494	1	1	1	NA	0.939	1	0.03
SE	6870	ATG2A	chr11	-	64912326	64912423	64912084	64912249	64913037	64913136	0	2	5	0	0	1	4	16	7	0	0	0	98	49	0.084584923	0.193502494	NA	1	0.714	1	1	1	-0.143
A5SS	886	DDIT3	chr12	-	57517268	57517753	57517705	57517753	57516587	57517180	1	3	7	0	0	0	2	20	3	0	1	0	98	49	0.084700702	0.201741673	1	1	1	1	0.909	1	0.03
SE	10384	SIGLEC5	chr19	-	51627461	51627746	51627148	51627248	51627833	51628091	0	2	8	0	2	0	0	4	5	0	0	0	98	49	0.084994123	0.19414311	NA	0.333	1	NA	1	1	-0.334
SE	12730	PILRA	chr7	+	100397878	100397912	100374043	100374433	100399290	100399340	0	1	14	0	0	0	0	3	2	0	3	0	83	49	0.085434257	0.194790215	NA	1	1	NA	0.371	1	0.315
SE	4324	KMT2D	chr12	-	49026181	49027322	49024809	49024946	49027802	49027930	0	1	7	0	0	0	0	2	2	0	1	0	98	49	0.085536621	0.194790215	NA	1	1	NA	0.5	1	0.25
SE	11837	RAB11FIP4	chr17	+	31517650	31517877	31488000	31488272	31521165	31521360	0	1	2	0	0	0	0	6	5	0	1	0	98	49	0.086321731	0.196280728	NA	1	1	NA	0.75	1	0.125
RI	359	DDIT3	chr12	-	57517268	57517753	57517268	57517595	57517705	57517753	4	2	10	0	0	0	8	27	2	0	1	0	98	49	0.086408407	0.336992786	1	1	1	1	0.931	1	0.023
SE	10863	ARHGAP9	chr12	-	57473602	57473708	57472254	57472688	57474041	57474176	0	0	32	0	0	3	0	6	1	0	2	2	98	49	0.086690204	0.196820811	NA	NA	0.842	NA	0.6	0.2	0.442
SE	9932	PTPRE	chr10	+	128072137	128072214	128070807	128070901	128073336	128073471	0	1	29	0	0	0	0	27	2	0	1	0	98	49	0.087167679	0.197606366	NA	1	1	NA	0.931	1	0.034
SE	10447	ELL	chr19	-	18465796	18465918	18465411	18465575	18472834	18472882	0	2	13	0	4	0	0	10	0	0	0	0	98	49	0.088641831	0.200598284	NA	0.2	1	NA	1	NA	-0.4
SE	10822	C16orf72	chr16	+	9092920	9093001	9091679	9092270	9102983	9103259	0	1	22	0	1	1	0	20	0	0	0	0	98	49	0.088754397	0.200598284	NA	0.333	0.917	NA	1	NA	-0.375
SE	13628	SUN2	chr22	-	38754641	38754706	38752506	38752665	38755762	38756019	0	0	7	0	1	2	0	0	0	0	0	6	98	49	0.088928004	0.200688873	NA	0	0.636	NA	NA	0	0.318
SE	1903	MAP3K1	chr5	+	56875031	56875310	56872824	56873005	56878979	56879101	0	0	5	0	1	1	1	3	2	0	0	0	98	49	0.089518138	0.201717783	NA	0	0.714	1	1	1	-0.643
SE	7640	IRF7	chr11	-	614173	614399	613950	614037	614475	614534	0	1	2	0	7	10	0	5	1	0	3	3	98	49	0.089929548	0.202173556	NA	0.067	0.091	NA	0.455	0.143	-0.22
SE	5219	TPM4	chr19	+	16081912	16082046	16076324	16076697	16086422	16086540	0	4	6	0	0	0	0	10	7	0	0	1	98	49	0.09016239	0.202173556	NA	1	1	NA	1	0.778	0.111
SE	6816	OAS1	chr12	+	112908535	112908824	112906933	112907219	112911050	112911235	0	1	25	0	0	0	5	3	3	0	1	0	98	49	0.090232466	0.202173556	NA	1	1	1	0.6	1	0.133
SE	2081	HERC5	chr4	+	88499925	88499992	88494164	88494331	88500914	88500985	0	3	48	0	1	1	0	33	0	0	2	0	98	49	0.090258454	0.202173556	NA	0.6	0.96	NA	0.892	NA	-0.112
A3SS	872	DGCR2	chr22	-	19064847	19065075	19064847	19065067	19068099	19068225	0	0	3	0	1	4	0	0	0	0	2	2	57	49	0.092162455	0.240423795	NA	0	0.392	NA	0	0	0.196
RI	832	HES4	chr1	-	999691	1000172	999691	999787	999865	1000172	1	2	1	0	0	0	3	13	4	0	1	0	98	49	0.092178476	0.342377196	1	1	1	1	0.867	1	0.044
SE	1266	MICAL1	chr6	-	109447356	109447440	109447072	109447229	109447680	109447722	0	3	5	0	0	0	0	2	1	0	0	1	98	49	0.094107379	0.210481236	NA	1	1	NA	1	0.333	0.334
SE	4124	XPO6	chr16	-	28135215	28135324	28133840	28133933	28146093	28146203	0	5	50	0	0	2	3	60	4	0	0	0	98	49	0.094351496	0.210563613	NA	1	0.926	1	1	1	-0.037
SE	3589	SP110	chr2	-	230202578	230202728	230200884	230200965	230207990	230208059	0	1	8	0	0	0	0	10	6	0	1	0	98	49	0.094424401	0.210563613	NA	1	1	NA	0.833	1	0.084
SE	10651	SLC43A2	chr17	-	1616561	1616769	1614978	1615034	1627714	1627920	0	0	9	0	0	1	0	9	0	0	0	2	98	49	0.094824344	0.211028906	NA	NA	0.818	NA	1	0	0.318
SE	7418	VAV1	chr19	+	6847997	6848114	6843134	6843166	6850669	6850757	0	3	13	0	4	0	3	10	0	0	0	0	98	49	0.095128464	0.211028906	NA	0.273	1	1	1	NA	-0.363
SE	10849	IRF1	chr5	-	132486794	132486844	132486556	132486686	132486953	132487130	0	2	21	0	0	1	0	36	5	0	8	0	98	49	0.095196923	0.211028906	NA	1	0.913	NA	0.692	1	0.111
SE	4963	DDX3X	chrX	+	41343215	41343351	41342736	41342836	41343736	41343822	0	2	31	0	0	1	0	24	1	0	3	0	98	49	0.095320071	0.211028906	NA	1	0.939	NA	0.8	1	0.07
SE	1143	IGF2R	chr6	+	160079579	160079787	160078200	160078362	160080128	160080275	0	4	40	0	0	1	0	6	2	0	3	0	98	49	0.095335081	0.211028906	NA	1	0.952	NA	0.5	1	0.226
SE	10701	TLE3	chr15	-	70059409	70059460	70058662	70058815	70060529	70060649	0	4	31	0	2	0	0	21	0	0	0	0	98	49	0.095684167	0.211266553	NA	0.5	1	NA	1	NA	-0.25
SE	6176	ABCA1	chr9	-	104819585	104819723	104818662	104818883	104819926	104820069	0	1	17	0	0	0	0	29	3	0	1	0	98	49	0.095723568	0.211266553	NA	1	1	NA	0.935	1	0.032
SE	8187	EPSTI1	chr13	-	42895008	42895108	42886387	42888502	42900309	42900383	0	1	9	0	0	2	4	11	1	0	0	0	98	49	0.096411782	0.212473472	NA	1	0.692	1	1	1	-0.154
SE	12365	NBEAL2	chr3	+	46995931	46996051	46995713	46995846	46996270	46996592	0	8	19	0	0	0	0	5	4	0	0	2	98	49	0.09699006	0.213434934	NA	1	1	NA	1	0.5	0.25
SE	8710	CD74	chr5	-	150406880	150406960	150406258	150406321	150407151	150407324	0	7	68	0	0	1	2	12	4	0	3	0	98	49	0.097385214	0.213991193	NA	1	0.971	1	0.667	1	0.097
SE	11126	PSEN1	chr14	+	73198030	73198129	73192643	73192864	73206385	73206472	0	1	8	0	2	1	0	5	0	0	0	0	98	49	0.098181291	0.215425518	NA	0.2	0.8	NA	1	NA	-0.5
A3SS	1986	PGS1	chr17	+	78414878	78415027	78414951	78415027	78403567	78404089	0	1	5	0	0	0	3	5	5	0	1	0	98	49	0.098812002	0.253724157	NA	1	1	1	0.714	1	0.095
SE	13029	FOSB	chr19	+	45471193	45471301	45470628	45470949	45472550	45475179	0	3	9	0	2	0	0	13	0	0	0	0	98	49	0.099118539	0.217164962	NA	0.429	1	NA	1	NA	-0.285
SE	13040	MAP3K8	chr10	+	30437175	30437406	30434020	30434378	30438915	30439274	0	0	6	0	0	1	0	2	0	0	2	2	98	49	0.099502086	0.217687969	NA	NA	0.75	NA	0.333	0	0.584
SE	12128	LRRC59	chr17	-	50392738	50392897	50392397	50392502	50394928	50394988	1	1	3	0	0	0	0	1	1	0	3	0	98	49	0.099753465	0.217812352	1	1	1	NA	0.143	1	0.428
SE	55	ELF4	chrX	-	130081255	130081539	130074580	130074752	130110324	130110500	0	2	5	0	1	0	0	0	1	0	1	4	98	49	0.100072297	0.217812352	NA	0.5	1	NA	0	0.111	0.695
A3SS	2860	TPM3	chr1	-	154155303	154159048	154155303	154157721	154170399	154170469	1	0	1	0	0	20	0	2	1	0	14	0	98	49	0.100080084	0.253724157	1	NA	0.024	NA	0.067	1	-0.021
SE	9378	FMNL1	chr17	+	45246504	45246604	45246209	45246330	45246866	45247316	0	1	0	0	1	15	5	0	0	0	13	2	98	49	0.100134734	0.217812352	NA	0.333	0	1	0	0	-0.167
SE	6719	KIF1B	chr1	+	10345844	10345953	10343231	10343287	10348648	10348733	0	4	2	0	0	0	0	1	2	0	1	0	98	49	0.100138613	0.217812352	NA	1	1	NA	0.333	1	0.334
SE	13866	ACSL1	chr4	-	184773080	184773154	184768315	184768450	184773662	184773714	0	2	38	0	0	1	3	28	2	0	3	0	98	49	0.100289399	0.217825096	NA	1	0.95	1	0.824	1	0.034
SE	13870	ACSL1	chr4	-	184803319	184803546	184788616	184788731	184825915	184826114	0	4	91	0	0	0	8	83	44	0	3	0	98	49	0.100452402	0.217864301	NA	1	1	1	0.933	1	0.022
SE	13051	NAPA	chr19	-	47500632	47500749	47495549	47495596	47514842	47515258	0	1	13	0	0	0	0	11	7	0	1	0	98	49	0.100911104	0.218543788	NA	1	1	NA	0.846	1	0.077
SE	7577	SPATS2L	chr2	+	200439121	200439328	200419249	200419496	200440648	200440784	0	0	3	0	0	1	0	1	0	0	0	3	98	49	0.101162648	0.218773323	NA	NA	0.6	NA	1	0	0.1
SE	12742	DYSF	chr2	+	71520177	71520208	71516988	71517039	71520788	71520904	0	1	12	0	1	0	2	7	0	0	0	0	80	49	0.10199177	0.220129306	NA	0.38	1	1	1	NA	-0.31
SE	10929	DEDD2	chr19	-	42204461	42204559	42198592	42199829	42209699	42209840	0	1	0	0	3	5	0	0	0	0	16	3	98	49	0.102192668	0.220129306	NA	0.143	0	NA	0	0	0.071
SE	3914	ALOX5AP	chr13	+	30755943	30756025	30752051	30752122	30763943	30764428	0	2	74	0	0	0	0	36	17	0	1	0	98	49	0.102229046	0.220129306	NA	1	1	NA	0.947	1	0.026
SE	3886	LCP1	chr13	-	46146907	46147103	46144441	46144520	46148351	46148447	0	9	167	0	0	1	8	67	3	0	2	0	98	49	0.102410835	0.220205272	NA	1	0.988	1	0.944	1	0.013
SE	3619	NIN	chr14	-	50830463	50830517	50821873	50822077	50831005	50831121	0	3	3	0	0	1	2	0	18	0	0	0	98	49	0.10387195	0.223027916	NA	1	0.6	1	NA	1	-0.2
SE	803	TBXAS1	chr7	+	140017670	140017833	140015722	140015860	140020024	140020325	0	0	14	0	0	1	0	2	0	0	1	1	98	49	0.104215706	0.223185153	NA	NA	0.875	NA	0.5	0	0.625
SE	10226	ACAP1	chr17	+	7337311	7337369	7336528	7336787	7341947	7342067	2	1	3	0	0	0	0	2	3	0	1	0	98	49	0.104242167	0.223185153	1	1	1	NA	0.5	1	0.25
SE	6858	SELL	chr1	-	169701559	169701688	169696534	169696553	169703254	169703440	0	2	95	0	1	0	12	29	3	0	0	0	98	49	0.105494926	0.225138011	NA	0.5	1	1	1	1	-0.25
SE	796	PARP12	chr7	-	140057898	140058034	140056855	140057153	140062521	140063721	0	1	8	0	1	0	0	6	0	0	0	0	98	49	0.105498898	0.225138011	NA	0.333	1	NA	1	NA	-0.334
SE	4946	ARAP1	chr11	-	72705804	72705890	72702904	72703079	72707174	72707374	0	2	13	0	0	0	0	5	7	0	1	0	98	49	0.105603658	0.225138011	NA	1	1	NA	0.714	1	0.143
SE	7968	OAS3	chr12	+	112967417	112967593	112964234	112964408	112967935	112968174	0	0	27	0	0	1	0	7	1	0	2	1	98	49	0.106158162	0.225817599	NA	NA	0.931	NA	0.636	0.333	0.447
SE	4056	FOS	chr14	+	75280559	75280667	75279876	75280128	75280782	75282234	0	25	189	0	1	0	3	248	16	0	0	0	98	49	0.106325146	0.225817599	NA	0.926	1	1	1	1	-0.037
SE	1509	HLA-E	chr6	+	30492540	30492583	30491536	30491653	30492748	30494205	0	3	55	0	1	0	0	33	4	0	0	0	92	49	0.106659671	0.225817599	NA	0.615	1	NA	1	1	-0.193
SE	11045	HLA-B	chr6	-	31354632	31354665	31354478	31354526	31355316	31355592	1	12	113	0	0	0	8	112	7	0	1	0	82	49	0.106709745	0.225817599	1	1	1	1	0.985	1	0.005
SE	7902	PACSIN2	chr22	-	42890946	42891182	42888642	42888798	42893456	42893613	0	1	8	0	0	0	0	10	8	0	1	0	98	49	0.106828817	0.225817599	NA	1	1	NA	0.833	1	0.084

SE	7882	HIF1A	chr14	+	61738086	61738373	61736888	61737109	61740504	61740627	0	1	10	0	0	1	0	34	1	0	0	0	98	49	0.106940257	0.225817599	NA	1	0.833	NA	1	1	-0.084
SE	7143	CPD	chr17	+	30438974	30439077	30431771	30431881	30442307	30442450	0	1	10	0	0	2	0	11	1	0	0	0	98	49	0.107022882	0.225817599	NA	1	0.714	NA	1	1	-0.143
SE	5742	ENTPD1	chr10	+	95860468	95860582	95847445	95847706	95864723	95864861	0	1	5	0	0	0	0	2	3	0	1	0	98	49	0.107124383	0.225817599	NA	1	1	NA	0.5	1	0.25
A3SS	166	SDCBP	chr8	+	58575899	58576061	58575902	58576061	58572204	58572314	0	3	20	0	0	2	0	9	1	0	4	0	52	49	0.10775843	0.269396074	NA	1	0.904	NA	0.68	1	0.112
SE	4628	SORL1	chr11	+	121577280	121577400	121574240	121574363	121583457	121583583	0	8	61	0	0	3	0	33	7	0	0	0	98	49	0.107936364	0.227210581	NA	1	0.91	NA	1	1	-0.045
SE	9584	ANXA11	chr10	-	80176106	80176155	80172806	80172869	80205342	80205444	0	0	4	0	0	2	0	4	0	0	0	3	98	49	0.108478756	0.228032965	NA	NA	0.5	NA	1	0	0
SE	10587	RNF213	chr17	+	80361733	80361888	80358287	80358479	80363101	80363314	0	1	25	0	0	0	0	32	6	0	1	0	98	49	0.108649764	0.228073457	NA	1	1	NA	0.941	1	0.03
SE	9787	HERC4	chr10	-	67990210	67990400	67988662	67988835	67990903	67991015	0	0	1	0	0	2	0	2	3	0	0	0	98	49	0.108971078	0.228428912	NA	NA	0.2	NA	1	1	-0.8
A5SS	1408	HNRNPC	chr14	-	21230957	21231072	21230996	21231072	21230318	21230366	0	6	6	0	1	5	4	15	0	1	7	0	88	49	0.108976627	0.254927467	NA	0.77	0.401	0.69	0.544	NA	-0.031
SE	11640	ARRB2	chr17	+	4716411	4716608	4715972	4716033	4717216	4717276	0	0	2	0	0	3	0	5	4	0	0	0	98	49	0.1093759	0.228660806	NA	NA	0.25	NA	1	1	-0.75
SE	7570	PGD	chr1	+	10403070	10403136	10400392	10400572	10404160	10404279	0	7	13	0	4	0	0	8	0	0	0	0	98	49	0.109641955	0.228660806	NA	0.467	1	NA	1	NA	-0.266
SE	5385	CD44	chr11	+	35180273	35180407	35176574	35176740	35186831	35186900	1	4	16	0	2	1	0	9	0	0	0	0	98	49	0.109674831	0.228660806	1	0.5	0.889	NA	1	NA	-0.204
SE	1986	DDX60	chr4	-	168272042	168272138	168268853	168268969	168273278	168273398	0	1	7	0	1	0	0	5	0	0	0	0	98	49	0.109690247	0.228660806	NA	0.333	1	NA	1	NA	-0.334
SE	1038	AOAH	chr7	-	36659165	36659265	36637850	36637910	36673942	36674009	0	0	10	0	0	1	1	8	0	0	1	1	98	49	0.110142389	0.229285332	NA	NA	0.833	1	0.8	0	0.233
SE	5785	DENND3	chr8	+	141144147	141144259	141138021	141138137	141150833	141150953	0	0	6	0	0	2	1	0	0	0	4	2	98	49	0.111042654	0.230839709	NA	NA	0.6	1	0	0	0.267
SE	5585	WTAP	chr6	+	159736257	159736295	159726997	159727333	159738989	159739045	0	3	10	0	0	0	0	6	3	0	0	1	87	49	0.111205631	0.230859204	NA	1	1	NA	1	0.628	0.186
SE	13176	ZNF274	chr19	+	58209960	58210073	58206719	58207202	58211559	58211686	0	1	0	0	3	0	0	2	0	0	0	0	98	49	0.111677163	0.231518312	NA	0.143	NA	NA	1	NA	-0.857
SE	422	FAM214B	chr9	-	35106537	35106643	35106251	35106410	35106832	35106891	0	1	10	0	0	0	0	4	4	0	2	0	98	49	0.112112283	0.232100222	NA	1	1	NA	0.5	1	0.25
SE	13719	HMHA1	chr19	+	1073673	1073746	1073505	1073590	1073947	1074014	0	0	23	0	0	1	0	6	1	0	3	1	98	49	0.112614145	0.232818515	NA	NA	0.92	NA	0.5	0.333	0.504
SE	8977	RPS9	chr19	+	54206275	54206462	54201486	54201609	54207397	54207647	0	3	56	0	0	1	0	22	2	0	2	0	98	49	0.113459253	0.234243486	NA	1	0.966	NA	0.846	1	0.06
SE	12370	NBEAL2	chr3	+	47007524	47007697	47006332	47006449	47007815	47007910	0	14	33	0	0	0	2	7	17	0	1	0	98	49	0.114232158	0.235336193	NA	1	1	1	0.778	1	0.074
SE	4364	LRRK2	chr12	+	40319987	40320175	40315211	40315300	40321033	40321188	0	2	64	0	4	2	0	16	1	0	3	0	98	49	0.114457785	0.235336193	NA	0.2	0.941	NA	0.727	1	-0.293
SE	11194	PARP14	chr3	+	122703741	122703978	122699389	122701635	122704526	122704748	0	6	21	0	1	0	0	22	0	0	0	0	98	49	0.114458255	0.235336193	NA	0.75	1	NA	1	NA	-0.125
A5SS	1123	MYO9B	chr19	+	17211646	17211786	17211646	17211774	17211894	17213295	0	0	2	0	0	2	0	0	0	0	0	4	61	49	0.11468766	0.263580411	NA	NA	0.445	NA	NA	0	0.445
SE	10702	TLE3	chr15	-	70064453	70064470	70060529	70060649	70066013	70066218	0	3	13	0	0	1	0	11	1	0	3	0	66	49	0.114960195	0.235829003	NA	1	0.906	NA	0.731	1	0.088
SE	2899	GTPBP1	chr22	+	38708844	38708956	38705801	38706147	38715906	38716087	0	0	8	0	0	1	0	9	0	0	6	1	98	49	0.115011749	0.235829003	NA	NA	0.8	NA	0.429	0	0.586
SE	7731	KIAA1033	chr12	+	105162748	105162845	105160000	105160148	105164110	105164307	0	1	7	0	1	0	0	4	0	0	0	0	98	49	0.115352023	0.235883116	NA	0.333	1	NA	1	NA	-0.334
SE	13111	AP2A1	chr19	+	49800960	49801058	49799967	49800150	49801389	49801621	0	1	3	0	1	0	0	4	0	0	0	0	98	49	0.115352023	0.235883116	NA	0.333	1	NA	1	NA	-0.334
SE	3524	DYRK1A	chr21	+	37420298	37420384	37367556	37367628	37472683	37472880	0	0	2	0	0	2	0	2	4	0	0	0	98	49	0.115941495	0.236766395	NA	NA	0.333	NA	1	1	-0.667
SE	7378	RNF19B	chr1	-	32945513	32945628	32944018	32944159	32946401	32946564	0	1	24	0	0	0	1	30	9	0	1	0	98	49	0.116142758	0.236855585	NA	1	1	1	0.937	1	0.021
SE	6360	ZSWIM8	chr10	+	73788669	73788823	73785623	73786086	73789095	73789190	0	2	3	0	5	0	0	2	0	0	0	0	98	49	0.116399553	0.237028277	NA	0.167	1	NA	1	NA	-0.416
SE	12686	MYO1F	chr19	-	8541905	8541991	8539946	8540028	8544296	8544464	0	11	31	0	0	3	0	21	4	0	0	0	98	49	0.116542846	0.237028277	NA	1	0.838	NA	1	1	-0.081
SE	6861	CARD8	chr19	-	48238382	48238532	48234402	48234543	48240961	48241063	0	0	7	0	0	1	0	0	0	0	0	1	98	49	0.11681427	0.237259253	NA	NA	0.778	NA	NA	0	0.778
A3SS	412	PRRC2A	chr6	+	31622689	31622901	31622726	31622901	31620672	31620858	0	0	2	0	0	2	0	0	0	0	3	4	86	49	0.117982531	0.29091583	NA	NA	0.363	NA	0	0	0.363
SE	11993	STAT5A	chr17	+	42291971	42292061	42289865	42290022	42295618	42295793	0	1	2	0	2	0	0	4	0	0	1	0	98	49	0.118118135	0.239583747	NA	0.2	1	NA	0.667	NA	-0.067
SE	12348	CSNK1D	chr17	-	82255428	82255577	82253015	82253244	82265685	82265796	0	0	3	0	0	2	0	2	5	0	0	0	98	49	0.118962034	0.240970265	NA	NA	0.429	NA	1	1	-0.571
SE	4455	GABARAPL1	chr12	+	10218062	10218141	10212889	10213219	10220439	10220558	0	0	8	0	2	0	0	6	3	0	2	0	98	49	0.119908107	0.242559736	NA	0	1	NA	0.6	1	-0.3
SE	11089	IQSEC1	chr3	-	12902772	12902822	12897042	12901522	12908348	12908525	0	0	2	0	0	3	0	0	0	0	5	5	98	49	0.120556389	0.24354335	NA	NA	0.25	NA	0	0	0.25
SE	7230	NFKBIZ	chr3	+	101855394	101855458	101855061	101855208	101855732	101855902	0	1	28	0	0	0	5	65	2	0	1	0	98	49	0.120755712	0.243618571	NA	1	1	1	0.97	1	0.01
SE	6272	BAZ2B	chr2	-	159430862	159431156	159429199	159429260	159432756	159433363	0	0	18	0	0	3	0	5	0	0	0	1	98	49	0.12105318	0.243891326	NA	NA	0.75	NA	1	0	0.25
SE	4944	ARAP1	chr11	-	72699004	72699107	72697910	72698106	72699416	72699552	2	8	34	0	0	2	0	30	7	0	0	0	98	49	0.121497957	0.24431631	1	1	0.895	NA	1	1	-0.035
SE	11629	ATP2A3	chr17	-	3953346	3953429	3951580	3951685	3953692	3953710	0	2	4	0	0	0	0	8	4	0	0	1	98	49	0.121589221	0.24431631	NA	1	1	NA	1	0.667	0.166
SE	3974	GLT1D1	chr12	+	128875913	128876062	128853535	128853649	128888638	128888744	0	3	18	0	0	0	0	7	3	0	0	1	98	49	0.122907287	0.246635051	NA	1	1	NA	1	0.6	0.2
SE	3455	GPCPD1	chr20	-	5586193	5586269	5584280	5584322	5593326	5593411	0	0	11	0	0	2	0	7	0	0	6	1	98	49	0.123901232	0.24829807	NA	NA	0.733	NA	0.368	0	0.549
SE	812	MKLN1	chr7	+	131445773	131445903	131443480	131443702	131463216	131463364	0	1	9	0	1	0	2	3	0	0	0	0	98	49	0.124958526	0.24977771	NA	0.333	1	1	1	NA	-0.334
SE	8076	NADK	chr1	-	1754050	1754208	1753566	1753649	1754283	1754383	0	4	21	0	0	2	4	17	5	0	0	0	98	49	0.125009625	0.24977771	NA	1	0.84	1	1	1	-0.08
SE	6057	HCLS1	chr3	-	121632331	121632563	121632100	121632184	121633066	121633171	0	10	51	0	0	2	0	41	11	0	0	0	98	49	0.125175468	0.24977771	NA	1	0.927	NA	1	1	-0.036
SE	11932	FBRS	chr16	+	30661303	30661333	30661179	30661215	30662419	30662468	0	0	1	0	0	1	0	0	3	0	0	0	79	49	0.12530432	0.24977771	NA	NA	0.383	NA	NA	1	-0.617
A3SS	1733	ZSWIM8	chr10	+	73794540	73794639	73794555	73794639	73794146	73794330	0	1	7	0	1	0	0	3	0	5	0	0	64	49	0.125310145	0.30480846	NA	0.434	1	0	1	NA	0.217
SE	7879	ALPL	chr1	+	21573664	21573799	21570304	21570374	21575732	21575924	0	0	18	0	0	1	0	14	1	0	1	1	98	49	0.125632388	0.250099972	NA	NA	0.9	NA	0.875	0.333	0.296
SE	8247	PRRC2C	chr1	+	171512031	171512200	171485526	171485735	171513000	171513172	0	1	4	0	1	0	0	8	2	0	0	0	98	49	0.127922639	0.254322389	NA	0.333	1	NA	1	1	-0.334
SE	10699	TLE3	chr15	-	70054437	70054685	70053226	70053374	70055048	70055298	0	4	15	0	0	4	0	21	6	0	1	0	98	49	0.128234493	0.254605606	NA	1	0.652	NA	0.913	1	-0.13
SE	3888	LCP1	chr13	-	46158825	46158989	46158521	46158651	46159598	46159686	0	1	110	0	0	5	0	59	2	0	6	0	98	49	0.129117546	0.256020675	NA	1	0.917	NA	0.831	1	0.043
SE	6628	PCBP2	chr12	+	53465931	53465973	53462480	53462567	53467220	53467293	0	2	30	0	1	3	0	11	0	0	1	0	91	49	0.129961836	0.257034707	NA	0.519	0.843	NA	0.856	NA	-0.175
SE	7727	FGD3	chr9	+	93019830	93019861	93018135	93018215	93020316	93020424	0	3	10	0	0	1	0	10	1	0	3	0	80	49	0.129970976	0.257034707	NA	1	0.86	NA	0.671	1	0.094
SE	9562	OASL	chr12	-	121027575	121027817	121023989	121024137	121031441	121031617	0	0	26	0	1	2	1	10	1	0	0	0	98	49	0.130217062	0.257182975	NA	0	0.867	1	1	1	-0.567
SE	8139	ITGAX	chr16	+	31372377	31372509	31371629	31371784	31372596	31372670	0	16	38	0	0	2	0	24	19	0	0	0	98	49	0.132864223	0.262066834	NA	1	0.905	NA	1	1	-0.047
SE	6225	AHCTF1	chr1	-	246916141	246916395	246913231	246913412	246918249	246918377	0	0	4	0	0	1	0	0	0	0	0	1	98	49	0.133877166	0.263718717	NA	NA	0.667	NA	NA	0	0.667
SE	5499	ADAM8	chr10	-	133275483	133275587	133274158	133274235	133276771	133276903	0	7	13	0	0	0	1	20	14	0	0	2	98	49	0.134548885	0.264504128	NA	1	1	1	1	0.778	0.074
SE	12648	TLN1	chr9	-	35704668	35704815	35704331	35704498	35705550	35705670	0	3	28	0	0	0	1	6	8	0	2	0	98	49	0.134879669	0.264504128	NA	1	1	1	0.6	1	0.133
SE	5934	RASGRP2	chr11	-	64727295	64727360	64726910	64727131	64728862	64729042	0	0	7	0	1	0	0	1	1	0	0	0	98	49	0.134979818	0.264504128	NA	0	1	NA	1	1	-0.5
SE	9259	MOV10	chr1	+	112695415	112695574	112694452	112694629	112696147	112696251	0	0	7	0	1	0	0	1	1	0	0	0	98	49	0.134979818	0.264504128	NA	0	1	NA	1	1	-0.5
SE	7950	PPP4R1	chr18	-	9562921	9563046	9561979	9562075	9563377	9563550	0	0	4	0	0	5	0	2	2	0	1	0	98	49	0.135910558	0.265981209	NA	NA	0.286	NA	0.5	1	-0.464
A3SS	2930	LRRC75A-AS1	chr17	+	16440110	16440253	16440184	16440253	16439326	16439414	0	0	2	0	0	1	0	1	4	0	1	0	98	49	0.136565787	0.324235571	NA	NA	0.5	NA	0.333	1	-0.166
SE	865	NAMPT	chr7	-	106274945	106275049	106272529	106272658	106277022	106277179	0	15	313	0	0	2	3	467	4	0	6	0	98	49	0.136632841	0.267047022	NA	1	0.987	1	0.975	1	0.002
A3SS	1281	CHD4	chr12	-	6601959	6602175	6601959	6602154	6602375	6602497	0	0	2	0	0	1	0	17	4	4	4	0	70	49	0.136899463	0.324235571	NA	NA	0.583	0	0.748	1	0
A5SS	1981	C12orf57	chr12	+	6944379	6944652	6944379	6944565	6945770	6946003	0	2	1	0	0	1	0	4	3	0	0	0	98	49	0.137146559	0.309762055	NA	1	0.333	NA	1	1	-0.334
SE	8155	KMT2E	chr7	+	105038125	105038199	105014189	105014535	105040838	105041023	0	0	7	0	0	2	0	12	0	0	2	1	98	49	0.137637656	0.268661555	NA	NA	0.636	NA	0.75	0	0.261
SE	11184	MEF2D	chr1	-	156480833	156480971	156479585	156479796	156482436	156482640	1	4	3	0	0	0	0	3	5	0	2	0	98	49	0.139889993	0.272539469	1	1	1	NA	0.429	1	0.285
SE	1447	BAG6	chr6	-	31643889	31643974	31643100	31643115	31644081	31644194	0	1	2	0	0	1	0	6	1	0	0	0	98	49	0.139987006	0.272539469	NA	1	0.5	NA	1	1	-0.25
SE	1213	VNN2	chr6	-	132753417	132753491	132752672	132752749	132755842	132756035	0	0	3	0	0	10	0	2	1	0	3	0	98	49	0.140487467	0.273115403	NA	NA	0.13	NA	0.25	1	-0.495
SE	4623	SORL1	chr11	+	121470006	121470123	121452202	121452616	121478117	121478243	0	9	67	0	0	1	1	40	3	0	2	0	98	49	0.140646255	0.273115403	NA	1	0.971	1	0.909	1	0.016
SE	2848	PIM3	chr22	+	49961124	49961234	49960494	49961032	49961317	49961368	0	8	15	0	1	0	0	32	7	0	0	0	98	49	0.14293986	0.277211109	NA	0.8	1	NA	1	1	-0.1
RI	341	CDKN1C	chr11	-	2884669	2885765	2884669	2885466	2885633	2885765	1	1	2	0	0	2	2	0	1	0	0	0	98	49	0.143188949	0.496482453	1	1	0.333	1	NA	1	-0.222
SE	2268	TNK2	chr3	-	195882050	195882328	195879048	195879175	195883156	195883309	0	0	3	0	0	1	2	2	0	0	2	1	98	49	0.143961591	0.278473967	NA	NA	0.6	1	0.333	0	0.156
SE	8090	SCLT1	chr4	-	128965226	128965318	128959599	128959777	128970377	128970468	0	0	3	0	0	1	0	6	0	0	0	1	98	49	0.143961591	0.278473967	NA	NA	0.6	NA	1	0	0.1
SE	9704	ABTB1	chr3	+	127676271	127676431	127675969	127676114	127676535	127676581	0	3	20	0	0	0	1	11	4	0	0	1	98	49	0.145196396	0.280100163	NA	1	1	1	1	0.667	0.111
A5SS	1928	PIGG	chr4	+	499199	499725	499199	499489	500395	500601	0	0	1	0	0	3	0	0	1	0	0	0	98	49	0.145772881	0.312928566	NA	NA	0.143	NA	NA	1	-0.857
A5SS	1929	PIGG	chr4	+	499199	500047	499199	499489	500395	500601	0	0	1	0	0	3	0	0	1	0	0	0	98	49	0.145772881	0.312928566	NA	NA	0.143	NA	NA	1	-0.857
SE	7876	TLR4	chr9	+	117708562	117708729	117704174	117704565	117712388	117717491	0	0	10	0	0	10	0	2	0	0	6	2	98	49	0.146224332	0.280100163	NA	NA	0.333	NA	0.143	0	0.262
SE	324	STOM	chr9	-	121366115	121366245	121356052	121356156	121370126	121370304	0	2	0	0	6	0	0	1	0	0	0	0	98	49	0.146368947	0.280100163	NA	0.143	NA	NA	1	NA	-0.857
A5SS	468	ARFIP1	chr4	+	152779936	152780226	152779936	152780091	152829624	152829726	0	0	1	0	0	1	0	2	2	0	0	0	98	49	0.146398672	0.312928566	NA	NA	0.333	NA	1	1	-0.667
RI	1023	OXLD1	chr17	-	81665035	81666637	81665035	81665584	81666517	81666637	0	0	1	0	0	1	0	0	2	0	0	0	98	49	0.146398672	0.496482453	NA	NA	0.333	NA	NA	1	-0.667
SE	3770	KIAA1109	chr4	+	122226706	122226835	122224497	122224677	122229120	122229240	0	0	1	0	0	1	0	9	2	0	1	0	98	49	0.146398672	0.280100163	NA	NA	0.333	NA	0.818	1	-0.576
SE	6502	GAS7	chr17	-	9969676	9969762	9959201	9959255	9981803	9981884	0	1	9	0	1	0	0	2	0	0	0	0	98	49	0.146398672	0.280100163	NA	0.333	1	NA	1	NA	-0.334
SE	11396	ADGRG3	chr16	+	57683931	57684212	57680504	57680617	57684389	57684483	0	0	1	0	0	1	0	3	2	0	3	0	98	49	0.146398672	0.280100163	NA	NA	0.333	NA	0.333	1	-0.333
SE	13361	SMARCA5	chr4	+	143540362	143540495	143538785	143538938	143543508	143543657	0	0	1	0	0	1	0	3	2	0	0	0	98	49	0.146398672	0.280100163	NA	NA	0.333	NA	1	1	-0.667
SE	13734	SERPINA1	chr14	-	94388800	94389010	94388559	94388660	94390456	94390692	0	1	17	0	1	3	0	2	0	4	0	0	98	49	0.146398672	0.280100163	NA	0.333	0.739	0	1	NA	0.036
SE	4332	NCF2	chr1	-	183567203	183567345	183566919	183566988	183569141	183569185	0	1	39	0	0	2	2	30	2	0	0	0	98	49	0.146479526	0.280100163	NA	1	0.907	1	1	1	-0.046
SE	9574	FAS	chr10	+	89010752	89010815	89010538	89010600	89011998	89012081	0	0	2	0	0	2	0	6	2	0	0	0	98	49	0.147583451	0.281852512	NA	NA	0.333	NA	1	1	-0.667
A5SS	2262	TLE3	chr15	-	70074502	70074607	70074532	70074607	70066013	70066218	0	0	7	0	0	4	0	2	3	0	2	0	79	49	0.148103596	0.312928566	NA	NA	0.52	NA	0.383	1	-0.171
SE	2581	PRKCD	chr3	+	53165103	53165215	53161206	53161428	53178403	53178537	0	4	3	0	2	1	0	1	2	0	1	3	98	49	0.148640738	0.283511458	NA	0.5	0.6	NA	0.333	0.25	0.259
SE	12586	SBNO2	chr19	-	1113534	1113704	1112817	1112949	1114230	1114422	0	9	21	0	0	1	0	40	18	0	0	0	98	49	0.149179961	0.284179318	NA	1	0.913	NA	1	1	-0.043
SE	8708	CTSS	chr1	-	150764637	150764764	150757857	150757980	150765697	150765957	0	1	129	0	0	0	20	18	6	0	0	1	98	49	0.150561932	0.28644884	NA	1	1	1	1	0.75	0.083
SE	759	RBM33	chr7	+	155665174	155665253	155644508	155644919	155672866	155672915	0	4	8	0	0	0	0	7	2	0	0	1	98	49	0.15316134	0.291025909	NA	1	1	NA	1	0.5	0.25
SE	6532	CARD16	chr11	-	105043482	105043545	105041325	105041719	105044391	105044658	0	1	14	0	1	5	4	1	0	2	5	0	98	49	0.15394085	0.292137749	NA	0.333	0.583	0.5	0.091	NA	0.162
SE	7042	SRSF2	chr17	-	76735771	76735875	76734114	76735158	76736153	76736464	0	1	2	0	2	3	0	3	0	0	12	1	98	49	0.154541762	0.292908283	NA	0.2	0.25	NA	0.111	0	0.17
SE	9278	PIK3CD	chr1	+	9721443	9721587	9721126	9721248	9721760	9721860	0	1	19	0	0	3	0	8	1	0	0	0	98	49	0.156377277	0.296013914	NA	1	0.76	NA	1	1	-0.12
SE	6507	TRIM33	chr1	-	114397939	114397990	114392776	114397860	114399456	114399609	0	0	7	0	0	2	0	4	5	0	0	0	98	49	0.156576702	0.296018595	NA	NA	0.636	NA	1	1	-0.364
A3SS	3032	NME4	chr16	+	400108	400754	400218	400754	399626	399739	2	0	0	1	0	0	3	0	0	0	0	0	98	49	0.156977715	0.366960892	0.5	NA	NA	1	NA	NA	-0.5
SE	5476	MICAL2	chr11	+	12138389	12138460	12110575	12110726	12162078	12162419	0	0	2	0	0	1	1	2	3	0	2	0	98	49	0.156977715	0.296403901	NA	NA	0.5	1	0.333	1	-0.278
SE	4367	LRRK2	chr12	+	40367005	40367077	40364841	40365050	40367643	40369284	0	0	13	0	0	3	0	13	6	0	3	0	98	49	0.157632777	0.297267333	NA	NA	0.684	NA	0.684	1	-0.158
SE	9609	TAPBP	chr6	-	33303954	33303989	33299694	33301771	33304127	33304217	0	2	23	0	1	0	4	10	1	0	0	0	84	49	0.158787547	0.299069779	NA	0.538	1	1	1	1	-0.231
A5SS	3020	SERPINA1	chr14	-	94390419	94390692	94390456	94390692	94388818	94389010	0	4	5	0	1	15	3	3	2	0	1	0	86	49	0.159752672	0.332184127	NA	0.695	0.16	1	0.631	1	-0.45
SE	8027	GNAS	chr20	+	58898940	58898985	58895611	58895684	58903527	58903585	0	1	2	0	3	2	0	0	0	0	4	0	94	49	0.16039283	0.301490206	NA	0.148	0.343	NA	0	NA	0.245
SE	1448	BAG6	chr6	-	31643889	31643977	31642828	31643115	31644081	31644194	0	1	3	0	0	1	0	7	1	0	0	0	98	49	0.160473829	0.301490206	NA	1	0.6	NA	1	1	-0.2
SE	4626	SORL1	chr11	+	121557313	121557405	121555186	121555318	121558590	121558837	0	10	73	0	0	2	0	39	5	0	3	0	98	49	0.162347432	0.304629451	NA	1	0.948	NA	0.867	1	0.04
A3SS	1989	PPP6R2	chr22	+	50419345	50419462	50419348	50419462	50418866	50418979	0	1	0	0	0	1	0	2	1	0	0	0	52	49	0.163314358	0.376879289	NA	1	0	NA	1	1	-0.5
SE	8529	CKLF	chr16	+	66558189	66558348	66552562	66552793	66563121	66563217	0	0	8	0	0	2	0	3	5	0	0	0	98	49	0.164123685	0.307578427	NA	NA	0.667	NA	1	1	-0.333
SE	695	DOCK5	chr8	+	25368191	25368250	25366869	25366970	25368570	25368725	0	0	11	0	0	5	0	5	3	0	1	0	98	49	0.166703165	0.311886731	NA	NA	0.524	NA	0.714	1	-0.333
SE	7636	IRF7	chr11	-	612998	613117	612554	612800	613205	613595	0	9	20	0	0	0	0	20	5	0	0	1	98	49	0.16703421	0.311886731	NA	1	1	NA	1	0.714	0.143
SE	2487	ATG3	chr3	-	112553279	112553329	112550191	112550262	112558375	112558417	0	0	4	0	0	3	1	4	2	0	0	0	98	49	0.167045122	0.311886731	NA	NA	0.4	1	1	1	-0.6
SE	471	DOCK8	chr9	+	317042	317128	311953	312166	325670	325737	0	10	33	0	0	0	2	15	7	0	0	2	98	49	0.167661893	0.311930048	NA	1	1	1	1	0.636	0.121
SE	4637	ARID5A	chr2	+	96549320	96549459	96547401	96547517	96549752	96549805	0	2	2	0	0	3	0	1	0	0	6	4	98	49	0.167831186	0.311930048	NA	1	0.25	NA	0.077	0	0.587
RI	566	SPRTN	chr1	+	231351303	231355027	231351303	231351571	231352609	231355027	0	0	1	0	0	2	2	2	1	0	0	0	98	49	0.167898476	0.545670046	NA	NA	0.2	1	1	1	-0.8
SE	5473	ARNTL	chr11	+	13365499	13365592	13360344	13360424	13366664	13366782	0	0	1	0	0	2	2	4	1	0	3	0	98	49	0.167898476	0.311930048	NA	NA	0.2	1	0.4	1	-0.6
SE	11688	CDK11B	chr1	-	1637080	1637202	1636896	1637004	1637407	1637513	0	1	0	0	2	0	0	1	1	0	0	0	98	49	0.167898476	0.311930048	NA	0.2	NA	NA	1	1	-0.8
A3SS	3932	OTOF	chr2	-	26472518	26472649	26472518	26472589	26473131	26473294	0	0	1	0	1	1	0	0	0	0	2	2	98	49	0.167980116	0.379975466	NA	0	0.333	NA	0	0	0.167
A3SS	2018	RNF19B	chr1	-	32948221	32948366	32948221	32948363	32949568	32949774	0	0	1	0	0	2	0	1	1	0	1	0	52	49	0.168877985	0.379975466	NA	NA	0.32	NA	0.485	1	-0.422
SE	7215	LSP1	chr11	+	1880086	1880224	1852969	1853197	1881431	1881596	0	7	39	0	0	0	2	37	11	0	0	1	98	49	0.17099036	0.31689431	NA	1	1	1	1	0.846	0.051
SE	1069	HNRNPA2B1	chr7	-	26197830	26197866	26197621	26197732	26200571	26200793	0	0	1	0	3	7	0	2	0	0	2	1	85	49	0.170992206	0.31689431	NA	0	0.076	NA	0.366	0	-0.145
SE	4615	PTPRJ	chr11	+	48112746	48112983	48110057	48110076	48121002	48121266	2	11	32	0	2	0	0	9	5	0	0	0	98	49	0.171767102	0.317938368	1	0.733	1	NA	1	1	-0.089
A3SS	502	ARG1	chr6	+	131579086	131579285	131579110	131579285	131576662	131576735	0	0	2	0	0	4	0	0	1	0	0	0	73	49	0.173440158	0.385422574	NA	NA	0.251	NA	NA	1	-0.749

SE	11874	MGAM	chr7	+	142036168	142036285	142034669	142034841	142036822	142036977	0	2	23	0	0	0	6	6	3	0	0	2	98	49	0.173570524	0.3208813	NA	1	1	1	1	0.429	0.19
A5SS	1004	GMPR2	chr14	+	24232421	24232847	24232421	24232788	24233218	24233340	3	0	2	0	0	4	0	4	1	0	1	0	98	49	0.174787188	0.357767525	1	NA	0.2	NA	0.667	1	-0.234
SE	7320	IL18R1	chr2	+	102373936	102373993	102371952	102372118	102375906	102376063	0	0	2	0	0	4	0	0	1	0	0	0	98	49	0.174787188	0.322733591	NA	NA	0.2	NA	NA	1	-0.8
SE	4120	XPO6	chr16	-	28104545	28104707	28101846	28101945	28106042	28106214	0	7	58	0	2	0	1	60	4	0	2	0	98	49	0.176059763	0.324652462	NA	0.636	1	1	0.937	1	-0.161
SE	235	CSF2RA	chrX	+	1303922	1304019	1300490	1300626	1305445	1305527	0	1	11	0	0	1	0	2	0	0	5	1	98	49	0.176258423	0.324652462	NA	1	0.846	NA	0.167	0	0.84
SE	12687	MYO1F	chr19	-	8548035	8548122	8545649	8545736	8548236	8548317	0	9	61	0	0	2	0	38	5	0	0	0	98	49	0.177681081	0.326872294	NA	1	0.938	NA	1	1	-0.031
SE	10039	SYNE2	chr14	+	64009965	64010116	64007042	64007222	64016472	64016631	0	0	3	0	0	2	0	7	2	0	0	0	98	49	0.179363678	0.329564313	NA	NA	0.429	NA	1	1	-0.571
SE	5557	SPATA13	chr13	+	24278613	24278821	24270686	24270912	24284134	24284271	0	0	1	0	1	3	0	0	0	0	3	4	98	49	0.18048442	0.331070043	NA	0	0.143	NA	0	0	0.071
SE	10589	RNF213	chr17	+	80369767	80369867	80369501	80369671	80371873	80371985	0	10	63	0	0	2	3	33	13	0	0	0	98	49	0.180942085	0.331070043	NA	1	0.94	1	1	1	-0.03
SE	10850	IRF1	chr5	-	132487925	132488025	132486953	132487130	132489391	132489483	0	7	14	0	0	1	0	12	1	0	2	0	98	49	0.181005184	0.331070043	NA	1	0.875	NA	0.75	1	0.062
SE	4941	ARAP1	chr11	-	72692752	72692785	72688454	72688537	72693324	72693470	0	0	2	0	1	16	0	2	0	0	2	1	82	49	0.181064255	0.331070043	NA	0	0.07	NA	0.374	0	-0.152
SE	6699	FCGR2A	chr1	+	161510833	161510956	161509819	161510074	161513894	161513932	0	1	66	0	0	2	0	40	2	0	0	0	98	49	0.181721355	0.331867797	NA	1	0.943	NA	1	1	-0.028
SE	7908	ATP6V0B	chr1	+	43975799	43975848	43974647	43975107	43976089	43976173	0	5	19	0	1	4	1	10	5	0	3	3	98	49	0.183348939	0.334433805	NA	0.714	0.704	1	0.625	0.455	0.016
A3SS	457	BTN2A1	chr6	+	26467718	26469653	26467947	26469653	26466061	26466088	0	2	4	0	0	2	0	3	1	0	0	0	98	49	0.184424674	0.403832852	NA	1	0.5	NA	1	1	-0.25
SE	2077	HERC5	chr4	+	88470613	88470673	88469156	88469260	88472408	88472502	0	2	23	0	0	1	0	41	1	0	3	0	98	49	0.18526689	0.337522588	NA	1	0.92	NA	0.872	1	0.024
SE	8553	EHBP1L1	chr11	+	65589935	65589991	65589750	65589820	65590086	65590210	0	1	10	0	0	0	3	7	2	0	0	1	98	49	0.185747044	0.337684951	NA	1	1	1	1	0.5	0.167
SE	5679	GMIP	chr19	-	19640295	19640360	19640084	19640192	19640445	19640571	0	9	25	0	0	2	2	12	3	0	3	0	98	49	0.185805359	0.337684951	NA	1	0.862	1	0.667	1	0.042
SE	9611	TAPBP	chr6	-	33313693	33313864	33313216	33313477	33314004	33314387	0	4	1	0	0	1	0	9	3	0	0	0	98	49	0.186099817	0.337811625	NA	1	0.333	NA	1	1	-0.334
SE	6577	RILPL2	chr12	-	123430507	123430659	123423043	123423157	123436081	123436733	0	1	12	0	4	4	0	13	6	0	3	3	98	49	0.189327441	0.343255903	NA	0.111	0.6	NA	0.684	0.5	-0.237
SE	6105	GLUL	chr1	-	182390496	182390852	182388571	182388750	182391678	182392206	0	2	1	0	5	18	0	1	1	3	24	22	98	49	0.189877016	0.343454188	NA	0.167	0.027	0	0.02	0.022	0.083
SE	2138	CXCL8	chr4	+	73741541	73741677	73740505	73740722	73741948	73742032	1	26	133	0	1	0	6	985	18	0	1	0	98	49	0.189893833	0.343454188	1	0.929	1	1	0.998	1	-0.023
RI	624	MCL1	chr1	-	150578842	150579738	150578842	150578963	150579422	150579738	0	41	264	0	0	2	13	148	29	0	0	0	98	49	0.190371362	0.586344893	NA	1	0.985	1	1	1	-0.008
A3SS	504	NDUFA2	chr5	-	140645362	140645725	140645362	140645678	140647255	140647362	0	0	3	0	1	2	0	0	0	0	0	1	96	49	0.190583999	0.403832852	NA	0	0.434	NA	NA	0	0.217
A5SS	1894	MYD88	chr3	+	38140387	38140592	38140387	38140568	38140756	38140848	0	0	4	0	1	7	0	0	0	0	0	2	73	49	0.190693809	0.381630589	NA	0	0.277	NA	NA	0	0.139
SE	12366	NBEAL2	chr3	+	46996750	46996833	46996270	46996592	46996953	46997046	0	7	12	0	0	0	0	4	6	0	4	0	98	49	0.19095347	0.344955607	NA	1	1	NA	0.333	1	0.334
A3SS	2130	FGD3	chr9	+	93034540	93034681	93034543	93034681	93032768	93032873	0	6	5	0	0	1	0	11	5	0	0	0	52	49	0.19141247	0.403832852	NA	1	0.825	NA	1	1	-0.088
SE	5865	NFKBID	chr19	-	35897650	35897856	35896912	35897058	35898471	35898532	0	7	10	0	2	0	0	4	6	0	0	0	98	49	0.192464506	0.34726789	NA	0.636	1	NA	1	1	-0.182
A3SS	4000	MAP4K4	chr2	+	101859642	101859864	101859735	101859864	101858995	101859082	0	0	4	0	0	1	0	1	4	0	0	0	98	49	0.193038976	0.403832852	NA	NA	0.667	NA	1	1	-0.333
SE	3456	GPCPD1	chr20	-	5593326	5593411	5584280	5584322	5598724	5598821	0	1	27	0	0	1	0	38	4	0	0	0	98	49	0.193523387	0.348759773	NA	1	0.931	NA	1	1	-0.034
A3SS	1806	PCBP2	chr12	+	53462480	53462567	53462492	53462567	53461014	53461143	1	1	8	0	1	19	0	9	0	4	7	0	61	49	0.194077531	0.403832852	1	0.445	0.253	0	0.508	NA	0.312
SE	8686	PTPRC	chr1	+	198702386	198702530	198699563	198699704	198703297	198703372	1	0	4	0	3	6	4	3	2	1	11	0	98	49	0.195041125	0.351074025	1	0	0.25	0.667	0.12	1	-0.179
A5SS	1245	TUBA1A	chr12	-	49188416	49189324	49188976	49189324	49186610	49186833	0	1	1	0	1	3	0	1	0	0	4	0	98	49	0.195185111	0.381630589	NA	0.333	0.143	NA	0.111	NA	0.127
A5SS	1246	TUBA1A	chr12	-	49188888	49189324	49188976	49189324	49186610	49186833	0	1	1	0	1	3	0	1	0	0	4	0	98	49	0.195185111	0.381630589	NA	0.333	0.143	NA	0.111	NA	0.127
A3SS	570	DDX60L	chr4	-	168395958	168396127	168395958	168396124	168400825	168400978	0	0	9	0	2	12	0	16	0	0	5	1	52	49	0.195185878	0.403832852	NA	0	0.414	NA	0.751	0	-0.169
RI	52	GSTK1	chr7	+	143264991	143265296	143264991	143265092	143265260	143265296	0	2	3	0	1	6	2	4	1	9	2	0	98	49	0.195448298	0.586344893	NA	0.5	0.2	0.1	0.5	1	-0.183
SE	5839	TUBA1A	chr12	-	49186309	49186458	49184794	49185990	49186610	49186833	0	5	29	0	1	2	6	17	0	0	1	0	98	49	0.195483437	0.35144929	NA	0.714	0.879	1	0.895	NA	-0.151
SE	12944	SMCHD1	chr18	+	2724898	2724995	2722518	2722663	2726451	2726524	0	2	53	0	0	1	0	50	1	0	2	0	98	49	0.196703531	0.353220319	NA	1	0.964	NA	0.926	1	0.019
SE	2289	BCL6	chr3	-	187726730	187726898	187725498	187725629	187728359	187728544	0	1	25	0	0	2	3	7	3	0	3	0	98	49	0.197315011	0.353895539	NA	1	0.862	1	0.538	1	0.085
SE	12685	MYO1F	chr19	-	8539946	8540028	8536948	8537055	8541905	8541991	0	6	55	0	0	2	1	30	5	0	0	0	98	49	0.197961525	0.354631909	NA	1	0.932	1	1	1	-0.034
SE	5477	MICAL2	chr11	+	12138389	12138464	12110575	12110726	12162078	12162419	0	0	3	0	0	1	1	3	3	0	2	0	98	49	0.199033101	0.354859729	NA	NA	0.6	1	0.429	1	-0.21
SE	5479	MICAL2	chr11	+	12220200	12220458	12216218	12216319	12221643	12221759	0	0	3	0	0	1	0	1	3	0	0	0	98	49	0.199033101	0.354859729	NA	NA	0.6	NA	1	1	-0.4
SE	7788	CLK3	chr15	+	74627969	74628052	74627538	74627668	74628603	74628683	0	0	3	0	0	1	0	0	3	0	0	0	98	49	0.199033101	0.354859729	NA	NA	0.6	NA	NA	1	-0.4
SE	8840	C1orf43	chr1	-	154213892	154213946	154212457	154212624	154214456	154214574	0	0	3	0	0	1	0	5	3	0	0	0	98	49	0.199033101	0.354859729	NA	NA	0.6	NA	1	1	-0.4
SE	9582	ANXA11	chr10	-	80168968	80169358	80167225	80167313	80170799	80170915	0	0	6	0	0	2	0	2	3	0	0	0	98	49	0.200611555	0.357250198	NA	NA	0.6	NA	1	1	-0.4
SE	10668	LAPTM5	chr1	-	30741639	30741716	30739808	30739937	30742455	30742549	0	4	37	0	0	1	6	52	6	0	0	0	98	49	0.201003059	0.357523784	NA	1	0.949	1	1	1	-0.026
SE	1136	IGF2R	chr6	+	160048374	160048543	160047791	160047907	160050472	160050652	0	3	42	0	0	2	0	21	4	0	0	0	98	49	0.201533872	0.357884259	NA	1	0.913	NA	1	1	-0.043
SE	5119	SLC3A2	chr11	+	62883988	62884112	62882907	62882999	62884456	62884525	0	0	1	0	0	4	0	0	0	0	5	4	98	49	0.201681948	0.357884259	NA	NA	0.111	NA	0	0	0.111
A3SS	3948	ALOX5	chr10	+	45443037	45443216	45443133	45443216	45441343	45441430	2	7	33	0	0	0	1	6	7	0	3	0	98	49	0.202413488	0.410636839	1	1	1	1	0.5	1	0.167
SE	13916	CD300A	chr17	+	74481288	74481326	74477435	74477530	74484000	74484798	0	2	17	0	0	2	0	5	6	0	0	0	87	49	0.203130618	0.359943165	NA	1	0.827	NA	1	1	-0.087
SE	11717	KDM6B	chr17	+	7844900	7845020	7839916	7840024	7845313	7845456	0	3	14	0	0	1	0	15	9	0	0	0	98	49	0.203321189	0.359943165	NA	1	0.875	NA	1	1	-0.062
A5SS	519	TNFAIP3	chr6	+	137867187	137867518	137867187	137867494	137871212	137871522	0	3	1	0	2	0	0	8	4	0	1	0	73	49	0.204508982	0.388363884	NA	0.502	1	NA	0.843	1	-0.17
A3SS	1274	PTPN6	chr12	+	6960662	6960945	6960805	6960945	6960343	6960435	0	1	8	0	1	17	0	2	0	3	6	3	98	49	0.204565043	0.410636839	NA	0.333	0.19	0	0.143	0	0.214
SE	13219	LILRA5	chr19	-	54312334	54312370	54311863	54312148	54312536	54312621	0	0	15	0	0	2	1	2	1	0	3	1	85	49	0.205560222	0.36347884	NA	NA	0.812	1	0.278	0.366	0.264
SE	5498	ADAM8	chr10	-	133274158	133274235	133273950	133274029	133276771	133276903	0	8	19	0	0	0	1	26	10	0	0	2	98	49	0.206600072	0.364888259	NA	1	1	1	1	0.714	0.095
A3SS	3540	MAPKAPK2	chr1	+	206732037	206734285	206732574	206734285	206731838	206731919	0	0	4	0	1	9	0	8	9	5	10	2	98	49	0.208792326	0.410636839	NA	0	0.182	0	0.286	0.692	-0.235
A3SS	1990	PPP6R2	chr22	+	50439697	50439857	50439700	50439857	50438598	50438762	0	0	1	0	0	2	0	0	0	0	3	2	52	49	0.209323809	0.410636839	NA	NA	0.32	NA	0	0	0.32
A5SS	1572	ACAD9	chr3	+	128879489	128880017	128879489	128879841	128884652	128884746	0	0	4	0	0	2	0	0	2	0	0	0	98	49	0.209418448	0.388363884	NA	NA	0.5	NA	NA	1	-0.5
SE	3047	FAM157A	chr3	+	198169784	198169872	198167675	198167871	198180754	198180857	0	4	48	0	0	3	0	7	4	0	1	4	98	49	0.209675253	0.368087771	NA	1	0.889	NA	0.778	0.333	0.389
A3SS	2544	TRIM26	chr6	-	30198665	30199264	30198665	30199153	30201034	30201138	0	0	2	0	0	1	1	2	2	0	2	0	98	49	0.209881051	0.410636839	NA	NA	0.5	1	0.333	1	-0.278
A5SS	2686	OXLD1	chr17	-	81666154	81666637	81666517	81666637	81665035	81665584	0	0	2	0	0	1	0	0	2	0	0	0	98	49	0.209881051	0.388363884	NA	NA	0.5	NA	NA	1	-0.5
SE	533	PRRC2B	chr9	+	131446400	131446642	131444184	131444328	131447084	131447206	0	0	2	0	0	1	0	1	2	0	0	0	98	49	0.209881051	0.368087771	NA	NA	0.5	NA	1	1	-0.5
SE	3379	TM9SF4	chr20	+	32159991	32160111	32158450	32158514	32161275	32161365	0	2	4	0	1	0	0	2	0	0	0	0	98	49	0.209881051	0.368087771	NA	0.5	1	NA	1	NA	-0.25
SE	3413	RALGAPA2	chr20	-	20524405	20524543	20520916	20521100	20524829	20524898	0	0	2	0	0	1	0	0	2	0	0	0	98	49	0.209881051	0.368087771	NA	NA	0.5	NA	NA	1	-0.5
SE	10410	RBM42	chr19	+	35633686	35634019	35633076	35633252	35634255	35634373	0	2	0	0	1	0	0	2	2	0	0	0	98	49	0.209881051	0.368087771	NA	0.5	NA	NA	1	1	-0.5
SE	12955	MAN2A2	chr15	+	90912541	90912664	90912042	90912279	90912876	90912991	0	0	2	0	0	1	0	2	2	0	0	0	98	49	0.209881051	0.368087771	NA	NA	0.5	NA	1	1	-0.5
A5SS	2442	RNASEK	chr17	+	7012416	7012761	7012416	7012757	7013665	7013742	0	0	4	0	0	2	1	2	2	0	1	0	53	49	0.210487296	0.388363884	NA	NA	0.649	1	0.649	1	-0.234
SE	5409	TAGAP	chr6	-	159042077	159042244	159041353	159041515	159043588	159043655	0	4	19	0	0	1	0	28	2	0	0	0	98	49	0.21465984	0.37603	NA	1	0.905	NA	1	1	-0.047
SE	4197	CPPED1	chr16	-	12704623	12705049	12659798	12665115	12781184	12781403	0	1	12	0	0	3	0	2	1	0	3	0	98	49	0.215298398	0.376709536	NA	1	0.667	NA	0.25	1	0.209
SE	3187	RGS19	chr20	-	64076856	64076954	64076524	64076646	64079293	64079492	0	0	7	0	0	2	0	1	3	0	0	0	98	49	0.220448488	0.384909961	NA	NA	0.636	NA	1	1	-0.364
SE	8747	ADIPOR1	chr1	-	202943757	202943945	202942024	202942218	202944982	202945169	0	1	9	0	0	0	0	5	1	0	0	1	98	49	0.220497323	0.384909961	NA	1	1	NA	1	0.333	0.334
SE	6274	BAZ2B	chr2	-	159453612	159453801	159448241	159448409	159478574	159478721	0	0	11	0	0	5	0	3	1	0	0	2	98	49	0.220936482	0.385229157	NA	NA	0.524	NA	1	0.2	-0.076
SE	838	CAPZA2	chr7	+	116906262	116906342	116904176	116904383	116910232	116910311	0	0	23	0	0	3	0	10	6	0	1	0	98	49	0.221735441	0.386174239	NA	NA	0.793	NA	0.833	1	-0.123
SE	276	RAPGEF1	chr9	-	131650128	131650242	131643238	131643417	131650809	131650949	0	2	1	0	0	1	3	10	1	0	7	0	98	49	0.223239446	0.388343619	NA	1	0.333	1	0.417	1	-0.139
SE	7716	THEMIS2	chr1	+	27876587	27876728	27872542	27872665	27885294	27885451	0	10	8	0	1	0	6	6	15	3	0	0	98	49	0.223535788	0.388409582	NA	0.833	1	0.5	1	1	0.083
SE	7875	TLR4	chr9	+	117708198	117708318	117704174	117704565	117708562	117708729	0	0	18	0	0	5	0	9	3	0	0	0	98	49	0.224282921	0.389257772	NA	NA	0.643	NA	1	1	-0.357
SE	4169	RNF10	chr12	+	120552498	120552698	120546404	120546601	120554717	120554808	0	1	5	0	0	1	0	6	2	0	0	0	98	49	0.225232695	0.390455294	NA	1	0.714	NA	1	1	-0.143
RI	991	HSH2D	chr19	+	16152551	16153208	16152551	16152641	16153042	16153208	0	5	21	0	1	1	4	8	1	0	0	0	98	49	0.226524618	0.654404451	NA	0.714	0.913	1	1	1	-0.186
A5SS	1015	AMICA1	chr11	-	118197967	118198091	118197997	118198091	118196734	118196821	0	0	14	0	0	6	0	9	2	0	1	0	79	49	0.228281084	0.409690921	NA	NA	0.591	NA	0.848	1	-0.333
SE	12396	TMEM127	chr2	-	96265137	96265512	96254832	96254997	96265868	96266013	0	3	11	0	0	1	0	13	4	0	0	0	98	49	0.229343062	0.397122837	NA	1	0.846	NA	1	1	-0.077
SE	10331	CYTIP	chr2	-	157418522	157418589	157414618	157416143	157427350	157427420	0	5	36	0	0	1	4	50	1	0	0	0	98	49	0.231211633	0.398828525	NA	1	0.947	1	1	1	-0.026
SE	6671	FGR	chr1	-	27617192	27617296	27616856	27617006	27621558	27621657	0	3	47	0	0	1	5	12	2	0	1	0	98	49	0.231633345	0.398828525	NA	1	0.959	1	0.857	1	0.027
A3SS	2782	GCH1	chr14	-	54842005	54844143	54842005	54843054	54845767	54845852	0	0	2	0	0	2	0	2	0	1	0	1	98	49	0.232250281	0.43959786	NA	NA	0.333	0	1	0	0
SE	3236	SLC9A8	chr20	+	49850761	49850844	49849578	49849680	49855437	49855581	0	0	4	0	0	4	0	0	0	0	4	1	98	49	0.23228173	0.398828525	NA	NA	0.333	NA	0	0	0.333
SE	10641	USP3	chr15	+	63533802	63533918	63532646	63532707	63537024	63537156	0	0	6	0	0	6	0	3	0	0	2	1	98	49	0.232454602	0.398828525	NA	NA	0.333	NA	0.429	0	0.119
SE	4196	FAM49B	chr8	-	129904498	129904585	129903311	129903350	129970942	129970995	0	0	3	0	0	3	0	1	0	0	3	1	98	49	0.232595516	0.398828525	NA	NA	0.333	NA	0.143	0	0.262
SE	3237	SLC9A8	chr20	+	49850809	49850844	49849578	49849680	49855437	49855581	0	0	4	0	0	4	0	0	0	0	4	1	84	49	0.232676511	0.398828525	NA	NA	0.368	NA	0	0	0.368
SE	4286	RASSF3	chr12	+	64688215	64688453	64684786	64684894	64691469	64691579	0	1	21	0	0	2	1	10	1	0	0	0	98	49	0.233640332	0.398828525	NA	1	0.84	1	1	1	-0.08
SE	10884	AKAP13	chr15	+	85658536	85658590	85655416	85655787	85664562	85664755	0	2	13	0	0	1	0	4	3	0	2	0	98	49	0.233666262	0.398828525	NA	1	0.867	NA	0.5	1	0.183
A3SS	650	TMEM175	chr4	+	951645	951717	951681	951717	951206	951258	0	0	1	0	0	1	0	0	1	0	0	0	85	49	0.234374295	0.43959786	NA	NA	0.366	NA	NA	1	-0.634
A3SS	1341	FAM89B	chr11	+	65573352	65574198	65573362	65574198	65572348	65572960	0	0	1	0	0	1	0	0	1	0	1	0	59	49	0.234434278	0.43959786	NA	NA	0.454	NA	0	1	-0.046
A3SS	193	ZYX	chr7	+	143381556	143381779	143381559	143381779	143381266	143381409	2	1	1	0	1	0	0	1	0	0	0	0	52	49	0.234452192	0.43959786	1	0.485	1	NA	1	NA	-0.172
A5SS	696	PDCD6	chr5	+	306601	306760	306601	306754	311292	311402	0	0	1	0	0	1	0	0	1	0	0	0	55	49	0.234460292	0.409690921	NA	NA	0.471	NA	NA	1	-0.529
A5SS	2321	ISG20	chr15	+	88638742	88639178	88638742	88639076	88639342	88639594	0	0	1	0	0	1	0	1	1	0	2	0	98	49	0.234545893	0.409690921	NA	NA	0.333	NA	0.2	1	-0.267
SE	739	CTSB	chr8	-	11864375	11864463	11853328	11853479	11868000	11868137	0	0	1	0	2	1	0	0	1	0	0	0	98	49	0.234545893	0.398828525	NA	0	0.333	NA	NA	1	-0.834
SE	5272	NIPBL	chr5	+	37000372	37000570	36995621	36995804	37000816	37000888	0	0	1	0	0	1	0	2	1	0	0	0	98	49	0.234545893	0.398828525	NA	NA	0.333	NA	1	1	-0.667
SE	7330	NEDD9	chr6	-	11305065	11305159	11213280	11213727	11305991	11306155	0	0	1	0	0	1	0	2	1	0	0	0	98	49	0.234545893	0.398828525	NA	NA	0.333	NA	1	1	-0.667
SE	9136	TRIM5	chr11	-	5665980	5666081	5663194	5665682	5667688	5667711	0	0	1	0	0	1	0	0	1	0	2	0	98	49	0.234545893	0.398828525	NA	NA	0.333	NA	0	1	-0.167
SE	10646	HERC1	chr15	-	63662983	63663204	63661752	63662021	63664469	63664594	0	0	1	0	0	1	0	2	1	0	1	0	98	49	0.234545893	0.398828525	NA	NA	0.333	NA	0.5	1	-0.417
SE	11342	BCKDK	chr16	+	31110687	31110761	31110400	31110499	31111090	31111219	0	0	1	0	0	1	0	0	1	0	0	0	98	49	0.234545893	0.398828525	NA	NA	0.333	NA	NA	1	-0.667
SE	13736	SERPINA1	chr14	-	94388818	94389010	94388559	94388660	94390456	94390692	0	1	16	0	1	3	0	1	0	4	0	0	98	49	0.234545893	0.398828525	NA	0.333	0.727	0	1	NA	0.03
A5SS	2440	SMARCB1	chr22	+	23791755	23791894	23791755	23791867	23793558	23793688	2	0	1	0	0	1	0	2	1	0	0	0	76	49	0.234555871	0.409690921	1	NA	0.392	NA	1	1	-0.304
SE	11000	TBC1D14	chr4	+	7004843	7004924	7001144	7001251	7010652	7010781	0	0	3	0	0	3	0	1	1	0	0	0	98	49	0.234573797	0.398828525	NA	NA	0.333	NA	1	1	-0.667
SE	2249	PCGF3	chr4	+	764983	765064	761278	761416	766031	770639	0	0	2	0	1	0	0	1	1	0	0	0	98	49	0.236755697	0.402083405	NA	0	1	NA	1	1	-0.5
SE	12650	TLN1	chr9	-	35725564	35725727	35725223	35725321	35732074	35732395	0	1	14	0	0	1	0	15	3	0	0	0	98	49	0.239010624	0.405454817	NA	1	0.875	NA	1	1	-0.062
SE	9492	SLC15A3	chr11	-	60941121	60941290	60939479	60939638	60942034	60942145	0	2	5	0	1	1	0	2	3	0	2	2	98	49	0.239519623	0.405735216	NA	0.5	0.714	NA	0.333	0.429	0.226
SE	13178	NLRP12	chr19	-	53807494	53807665	53805279	53805450	53809586	53811288	0	1	13	0	0	2	0	3	4	0	0	0	98	49	0.23984512	0.405735216	NA	1	0.765	NA	1	1	-0.117
SE	6632	MRVI1	chr11	-	10600917	10601059	10594145	10594195	10603119	10603251	0	0	4	0	0	1	0	6	3	0	0	0	98	49	0.239985767	0.405735216	NA	NA	0.667	NA	1	1	-0.333
SE	13627	SUN2	chr22	-	38750897	38751035	38750224	38750320	38751209	38751373	0	1	6	0	0	0	0	1	3	0	2	0	98	49	0.241094036	0.407150939	NA	1	1	NA	0.2	1	0.4
SE	3099	MX1	chr21	+	41443787	41443866	41441715	41441914	41445447	41445570	0	4	62	0	0	2	18	50	6	0	3	0	98	49	0.244079828	0.411554843	NA	1	0.939	1	0.893	1	0.005
SE	11814	FLOT2	chr17	-	28881191	28881375	28880712	28880862	28881813	28882028	0	3	27	0	0	2	0	12	1	0	2	0	98	49	0.244249447	0.411554843	NA	1	0.871	NA	0.75	1	0.06
A5SS	1373	FCAR	chr19	+	54888006	54888294	54888006	54888228	54889648	54891420	1	5	40	0	2	6	9	17	4	0	2	0	98	49	0.244859012	0.422059613	1	0.556	0.769	1	0.81	1	-0.162
SE	1039	AOAH	chr7	-	36686698	36686794	36673942	36674009	36724021	36724549	0	5	37	0	0	1	0	10	2	0	1	0	98	49	0.245974583	0.413770577	NA	1	0.949	NA	0.833	1	0.058
SE	12364	NBEAL2	chr3	+	46992474	46992555	46991839	46991946	46993936	46994020	0	0	10	0	0	9	0	2	1	0	1	0	98	49	0.246115034	0.413770577	NA	NA	0.357	NA	0.5	1	-0.393
SE	12574	R3HDM4	chr19	-	901421	901546	900828	900952	901975	902130	0	6	19	0	0	1	5	27	1	0	0	0	98	49	0.247240838	0.41489777	NA	1	0.905	1	1	1	-0.047
SE	516	SLA	chr8	-	133044983	133045115	133039997	133040130	133047829	133047933	0	0	35	0	0	6	0	8	4	0	1	0	98	49	0.247337593	0.41489777	NA	NA	0.745	NA	0.8	1	-0.155
SE	4618	PTPRJ	chr11	+	48164379	48164515	48163457	48163618	48167203	48170842	0	1	10	0	0	1	0	10	3	0	0	0	98	49	0.248230356	0.415931131	NA	1	0.833	NA	1	1	-0.084
SE	4055	FOS	chr14	+	75279876	75280128	75278777	75279123	75280559	75280667	0	18	153	0	0	1	11	176	10	0	0	0	98	49	0.249706448	0.417938521	NA	1	0.987	1	1	1	-0.006
A3SS	3814	NLRP12	chr19	-	53807494	53807668	53807494	53807665	53809586	53811288	0	0	1	0	0	6	0	0	0	0	2	4	52	49	0.250107194	0.464116443	NA	NA	0.136	NA	0	0	0.136
SE	2700	MBNL1	chr3	+	152432716	152432920	152414940	152415111	152445281	152445539	0	1	24	0	0	1	7	7	1	0	1	0	98	49	0.252076189	0.421435498	NA	1	0.923	1	0.778	1	0.035
SE	12940	SMCHD1	chr18	+	2674014	2674145	2673280	2673363	2688393	2688508	0	7	143	0	0	2	5	100	1	0	3	0	98	49	0.254061338	0.424282435	NA	1	0.973	1	0.943	1	0.005
A3SS	3504	FCGR3A	chr1	-	161548420	161548678	161548420	161548675	161549010	161549031	0	1	5	0	0	2	0	2	1	0	0	0	52	49	0.25427504	0.467035788	NA	1	0.702	NA	1	1	-0.149
SE	4164	NT5C2	chr10	-	103090610	103090787	103088016	103089908	103090935	103090996	0	0	14	0	0	5	0	2	2	0	0	0	98	49	0.254640733	0.424778049	NA	NA	0.583	NA	1	1	-0.417
A5SS	2258	TLE3	chr15	-	70057458	70057658	70057473	70057658	70056297	70056374	0	1	9	0	0	3	0	3	1	0	3	0	64	49	0.254834903	0.430953855	NA	1	0.697	NA	0.434	1	0.132

SE	13858	MMP25	chr16	+	3050253	3050546	3050008	3050144	3057032	3057209	0	7	19	0	0	1	0	17	10	0	0	0	98	49	0.255901705	0.426408273	NA	1	0.905	NA	1	1	-0.047
A5SS	403	CNOT8	chr5	+	154858518	154858816	154858518	154858768	154863206	154863395	1	0	3	0	0	2	1	0	1	0	0	0	97	49	0.256598479	0.430953855	1	NA	0.431	1	NA	1	-0.284
SE	12369	NBEAL2	chr3	+	47007240	47007350	47006332	47006449	47007524	47007697	0	1	7	0	1	0	0	5	0	0	2	0	98	49	0.256741576	0.427007803	NA	0.333	1	NA	0.556	NA	0.11
SE	12947	SMCHD1	chr18	+	2751277	2751393	2750349	2750507	2752487	2752552	1	8	40	0	0	1	0	46	4	0	0	0	98	49	0.25682971	0.427007803	1	1	0.952	NA	1	1	-0.016
SE	2166	FYB	chr5	-	39125997	39126135	39124252	39124278	39127740	39127807	0	0	17	0	0	9	6	25	1	0	6	2	98	49	0.257579272	0.427780824	NA	NA	0.486	1	0.676	0.2	-0.139
SE	7217	LSP1	chr11	+	1887236	1887314	1886731	1886866	1887473	1887576	0	6	18	0	0	1	0	22	2	0	0	0	98	49	0.260907854	0.432830579	NA	1	0.9	NA	1	1	-0.05
SE	7231	NFKBIZ	chr3	+	101857072	101857183	101855732	101855902	101857291	101857459	0	0	19	0	0	3	5	34	4	0	7	0	98	49	0.261478977	0.432929688	NA	NA	0.76	1	0.708	1	-0.143
SE	5891	CSF1	chr1	+	109914258	109914381	109910610	109911062	109915633	109915696	0	1	1	0	3	0	0	0	0	0	2	0	98	49	0.261543684	0.432929688	NA	0.143	1	NA	0	NA	0.572
SE	6056	HCLS1	chr3	-	121632100	121632184	121631397	121631982	121632331	121632563	0	3	39	0	0	0	1	28	4	0	0	1	98	49	0.261910932	0.433060651	NA	1	1	1	1	0.667	0.111
SE	1667	GRK6	chr5	+	177431994	177432107	177430871	177430967	177432232	177432310	0	7	9	0	0	1	0	4	3	0	3	0	98	49	0.26379167	0.435653879	NA	1	0.818	NA	0.4	1	0.209
SE	10928	DEDD2	chr19	-	42202050	42202072	42198592	42199829	42209699	42209840	0	0	3	0	3	5	0	2	0	0	16	3	71	49	0.264511909	0.435653879	NA	0	0.293	NA	0.079	0	0.107
RI	607	TAF1C	chr16	-	84181327	84181663	84181327	84181463	84181591	84181663	0	0	3	0	0	1	0	1	2	0	0	0	98	49	0.264638717	0.737207854	NA	NA	0.6	NA	1	1	-0.4
SE	3872	WDFY2	chr13	+	51719197	51719348	51703595	51703650	51727677	51727790	0	0	3	0	0	1	3	2	2	0	0	0	98	49	0.264638717	0.435653879	NA	NA	0.6	1	1	1	-0.4
SE	7725	ZFC3H1	chr12	-	71636849	71637059	71636489	71636654	71638417	71638515	0	0	3	0	0	1	0	9	2	0	0	0	98	49	0.264638717	0.435653879	NA	NA	0.6	NA	1	1	-0.4
A5SS	1432	ADPGK	chr15	-	72783245	72783785	72783458	72783785	72774871	72775097	0	3	7	0	0	1	2	2	8	0	0	0	98	49	0.265624158	0.440465375	NA	1	0.778	1	1	1	-0.111
SE	5576	SAT1	chrX	+	23784293	23784403	23783799	23783883	23785327	23785429	0	5	44	0	10	78	0	46	0	0	89	9	98	49	0.267669304	0.440160792	NA	0.2	0.22	NA	0.205	0	0.108
A3SS	1360	DDX3X	chrX	+	41347311	41347451	41347314	41347451	41346858	41347012	0	0	8	0	0	6	0	16	1	0	2	0	52	49	0.267844114	0.486989299	NA	NA	0.557	NA	0.883	1	-0.384
SE	7628	RALGAPB	chr20	+	38548688	38548795	38546242	38546430	38551070	38551223	0	0	4	0	0	3	0	0	1	0	1	0	98	49	0.268677998	0.441336646	NA	NA	0.4	NA	0	1	-0.1
SE	6197	TPCN2	chr11	+	69072626	69072708	69071922	69072023	69072914	69073001	0	2	0	0	0	1	0	3	1	0	0	0	98	49	0.27131636	0.444793103	NA	1	0	NA	1	1	-0.5
A5SS	435	PAPD4	chr5	+	79612419	79612769	79612419	79612735	79613010	79613075	0	2	1	0	0	1	0	2	1	0	0	0	83	49	0.271326485	0.444297119	NA	1	0.371	NA	1	1	-0.315
SE	1339	CMTR1	chr6	+	37462828	37463008	37461548	37461645	37471020	37471077	0	0	3	0	1	0	0	2	5	0	0	0	98	49	0.271849638	0.444793103	NA	0	1	NA	1	1	-0.5
SE	9456	PFKFB3	chr10	+	6217134	6217191	6216705	6216780	6219568	6219693	0	2	31	0	0	1	0	32	1	0	0	0	98	49	0.271902353	0.444793103	NA	1	0.939	NA	1	1	-0.03
SE	9007	LYST	chr1	-	235804503	235804665	235802907	235803064	235805742	235806772	0	4	25	0	0	2	0	11	1	0	0	0	98	49	0.271988796	0.444793103	NA	1	0.862	NA	1	1	-0.069
SE	12740	DYSF	chr2	+	71511806	71511921	71503213	71503319	71513715	71513921	0	0	5	0	0	1	0	4	3	0	0	0	98	49	0.272557849	0.444793103	NA	NA	0.714	NA	1	1	-0.286
SE	13209	LILRB2	chr19	-	54276380	54276456	54276263	54276301	54276806	54276929	0	0	5	0	0	1	0	2	3	0	0	0	98	49	0.272557849	0.444793103	NA	NA	0.714	NA	1	1	-0.286
A3SS	831	TOM1	chr22	+	35322998	35323177	35323027	35323177	35317876	35317961	0	1	5	0	0	1	0	0	3	0	0	0	78	49	0.272864991	0.491156984	NA	1	0.759	NA	NA	1	-0.121
SE	12823	SEC14L1	chr17	+	77193420	77193549	77191180	77191312	77194676	77194911	0	5	49	0	0	1	2	50	3	0	0	0	98	49	0.274578151	0.44760408	NA	1	0.961	1	1	1	-0.02
SE	4882	DCAF11	chr14	+	24116916	24117044	24115381	24115749	24117265	24117393	0	0	3	0	0	1	0	2	2	0	2	2	98	49	0.277220408	0.450933196	NA	NA	0.6	NA	0.333	0.333	0.267
SE	4883	DCAF11	chr14	+	24116971	24117044	24115282	24115749	24117265	24117393	0	0	3	0	0	1	0	0	2	0	2	2	98	49	0.277220408	0.450933196	NA	NA	0.6	NA	0	0.333	0.433
A3SS	3597	EIF4G1	chr3	+	184323404	184323593	184323407	184323593	184323082	184323241	0	0	2	0	0	3	0	0	0	0	1	1	52	49	0.278939638	0.491656631	NA	NA	0.386	NA	0	0	0.386
SE	8081	CSF3R	chr1	-	36472065	36472139	36471432	36471646	36472237	36472391	0	94	256	0	0	1	16	183	131	0	0	0	98	49	0.278963874	0.453278598	NA	1	0.992	1	1	1	-0.004
A3SS	1548	PPP4C	chr16	+	30081199	30081310	30081258	30081310	30076314	30076475	0	0	3	0	0	2	0	2	1	0	5	0	98	49	0.280935793	0.491656631	NA	NA	0.429	NA	0.167	1	-0.155
SE	3522	HDAC4	chr2	-	239144582	239144714	239139683	239139796	239156651	239156773	0	0	3	0	0	2	0	0	1	0	0	0	98	49	0.280935793	0.454548838	NA	NA	0.429	NA	NA	1	-0.571
SE	4254	MAPKAP1	chr9	-	125672315	125672643	125669817	125669907	125706970	125707234	0	0	3	0	0	2	8	0	1	0	0	0	98	49	0.280935793	0.454548838	NA	NA	0.429	1	NA	1	-0.571
SE	4531	DUSP6	chr12	-	89350587	89351025	89347824	89349561	89351639	89352859	0	3	21	0	2	1	0	1	0	0	0	0	98	49	0.280935793	0.454548838	NA	0.429	0.913	NA	1	NA	-0.329
SE	10627	RRP12	chr10	-	97390422	97390539	97388488	97388624	97390738	97390844	0	7	7	0	0	1	5	9	3	0	0	0	98	49	0.281057395	0.454548838	NA	1	0.778	1	1	1	-0.111
SE	13626	SUN2	chr22	-	38738872	38738988	38738586	38738754	38739341	38739426	0	0	6	0	0	4	0	6	1	0	0	0	98	49	0.281257764	0.454548838	NA	NA	0.429	NA	1	1	-0.571
A3SS	1827	FCGR2A	chr1	+	161506333	161506591	161506336	161506591	161505986	161506007	0	1	20	0	0	17	0	6	2	0	10	1	52	49	0.28133685	0.491656631	NA	1	0.526	NA	0.361	0.653	0.256
SE	12951	GMFG	chr19	-	39335260	39335310	39333076	39333126	39335434	39335531	0	1	18	0	0	1	13	16	2	0	0	0	98	49	0.283872459	0.458281747	NA	1	0.9	1	1	1	-0.05
SE	3214	RTFDC1	chr20	+	56516934	56516989	56513314	56513428	56517105	56517201	0	0	7	0	0	2	0	13	2	0	0	0	98	49	0.286074243	0.461340759	NA	NA	0.636	NA	1	1	-0.364
SE	9377	FMNL1	chr17	+	45245631	45245733	45245252	45245416	45245877	45245973	0	5	21	0	3	6	0	24	7	0	7	0	98	49	0.287117418	0.462526773	NA	0.455	0.636	NA	0.632	1	-0.271
SE	9458	PFKFB3	chr10	+	6222854	6222984	6221380	6221527	6223957	6224020	0	3	22	0	0	1	0	21	7	0	2	0	98	49	0.28811154	0.463631312	NA	1	0.917	NA	0.84	1	0.039
SE	10611	PIK3R5	chr17	-	8889972	8890126	8889138	8889222	8890737	8890912	0	5	10	0	0	1	0	3	10	0	0	0	98	49	0.288581572	0.463891019	NA	1	0.833	NA	1	1	-0.084
SE	816	LINC-PINT	chr7	-	131002196	131002320	130984052	130984109	131052424	131052555	0	0	0	0	2	9	0	0	1	0	8	1	98	49	0.290996266	0.46727285	NA	0	0	NA	0	0.333	-0.167
A3SS	2039	H2AFY	chr5	-	135360496	135360607	135360496	135360604	135369405	135369603	0	0	3	0	0	5	6	1	0	0	0	1	52	49	0.292512865	0.506272265	NA	NA	0.361	1	1	0	-0.306
SE	7721	THEMIS2	chr1	+	27881970	27883043	27879643	27880054	27885294	27885451	0	1	19	0	0	1	0	15	3	0	0	0	98	49	0.292531456	0.469236689	NA	1	0.905	NA	1	1	-0.047
SE	11044	HLA-B	chr6	-	31354632	31354665	31354478	31354526	31355106	31355223	1	16	132	0	0	1	8	135	6	0	0	0	82	49	0.294858598	0.472465323	1	1	0.987	1	1	1	-0.004
SE	2178	WAS	chrX	+	48685733	48685836	48685546	48685633	48685945	48685987	0	5	20	0	0	0	0	20	2	0	0	1	98	49	0.297924203	0.476869091	NA	1	1	NA	1	0.5	0.25
RI	186	PAPD4	chr5	+	79612419	79613075	79612419	79612735	79613010	79613075	2	3	7	0	0	1	2	7	2	0	0	0	98	49	0.299346388	0.80513856	1	1	0.778	1	1	1	-0.074
A3SS	2683	PXN	chr12	-	120215102	120215417	120215102	120215273	120221622	120221758	0	1	7	0	1	3	0	5	3	0	2	0	98	49	0.29950383	0.513435137	NA	0.333	0.538	NA	0.556	1	-0.343
SE	8361	TNFRSF10C	chr8	+	23111719	23111825	23102813	23103181	23114656	23114770	0	0	51	0	0	4	0	12	6	0	1	0	98	49	0.302014034	0.482901162	NA	NA	0.864	NA	0.857	1	-0.065
SE	9507	FOXP1	chr3	-	71359149	71359244	71299819	71299880	71493425	71493555	0	0	4	0	0	1	0	5	2	0	0	0	98	49	0.303158096	0.484215322	NA	NA	0.667	NA	1	1	-0.333
A5SS	2161	SIRPB2	chr20	-	1479699	1480065	1479993	1480065	1478265	1478607	0	0	8	0	0	2	0	1	2	0	0	0	98	49	0.304701638	0.492789069	NA	NA	0.667	NA	1	1	-0.333
SE	7847	RPS6KA1	chr1	+	26555536	26555625	26555150	26555221	26556653	26556718	0	0	27	0	1	4	4	8	0	0	3	1	98	49	0.305107208	0.48651819	NA	0	0.771	1	0.571	0	-0.138
SE	4904	PAK1	chr11	-	77325327	77325376	77322014	77323360	77332729	77332867	0	0	3	0	1	12	0	0	0	0	2	1	98	49	0.305247274	0.48651819	NA	0	0.111	NA	0	0	0.056
A3SS	3600	CNN2	chr19	+	1036066	1036246	1036129	1036246	1032558	1032696	0	2	9	0	1	10	11	5	10	0	5	1	98	49	0.309557191	0.525663154	NA	0.5	0.31	1	0.333	0.833	-0.317
SE	8217	FURIN	chr15	+	90877526	90877615	90877134	90877211	90878131	90878304	1	1	6	0	0	1	0	2	4	0	0	0	98	49	0.310862931	0.494727732	1	1	0.75	NA	1	1	-0.083
SE	12945	SMCHD1	chr18	+	2732264	2732492	2729274	2729409	2738396	2738545	0	1	52	0	0	1	1	43	2	0	0	0	98	49	0.311165715	0.494727732	NA	1	0.963	1	1	1	-0.018
SE	5935	RASGRP2	chr11	-	64735902	64735980	64735541	64735664	64736752	64737034	0	1	5	0	0	0	1	3	1	0	0	3	98	49	0.311385518	0.494727732	NA	1	1	1	1	0.143	0.286
SE	13180	NLRP12	chr19	-	53809586	53811288	53807494	53807665	53814907	53814988	0	1	12	0	0	1	0	5	6	0	0	0	98	49	0.311762966	0.49480437	NA	1	0.857	NA	1	1	-0.072
SE	3534	LRRFIP1	chr2	+	237751199	237751271	237749198	237749324	237753308	237753479	0	0	2	0	1	13	0	7	0	0	4	2	98	49	0.314453037	0.498547379	NA	0	0.071	NA	0.467	0	-0.198
SE	6694	RBCK1	chr20	+	420870	421031	419557	419731	422126	422238	0	2	3	0	0	1	0	1	3	0	0	0	98	49	0.315518625	0.499709688	NA	1	0.6	NA	1	1	-0.2
SE	1664	PDLIM7	chr5	-	177489392	177489627	177488067	177488248	177489770	177489832	0	3	11	0	0	1	0	6	5	0	0	0	98	49	0.31710227	0.50058381	NA	1	0.846	NA	1	1	-0.077
SE	3246	SULF2	chr20	-	47666259	47666488	47665856	47665953	47672197	47672393	0	1	5	0	0	1	0	3	2	0	0	0	98	49	0.317618048	0.50058381	NA	1	0.714	NA	1	1	-0.143
A5SS	1014	PRKD2	chr19	-	46716814	46717127	46716950	46717127	46716130	46716535	0	0	2	0	0	1	0	0	1	0	0	0	98	49	0.318735	0.509198598	NA	NA	0.5	NA	NA	1	-0.5
RI	727	TRIM5	chr11	-	5663194	5665682	5663194	5665395	5665655	5665682	0	0	2	0	0	1	4	0	1	0	0	0	98	49	0.318735	0.805848647	NA	NA	0.5	1	NA	1	-0.5
SE	702	ENTPD4	chr8	-	23442006	23442066	23441568	23441723	23443849	23443953	0	0	2	0	0	1	0	2	1	0	1	0	98	49	0.318735	0.50058381	NA	NA	0.5	NA	0.5	1	-0.25
SE	2612	UBA7	chr3	-	49809389	49809464	49808975	49809159	49809541	49809725	0	0	2	0	0	1	0	1	1	0	0	0	98	49	0.318735	0.50058381	NA	NA	0.5	NA	1	1	-0.5
SE	2722	KAT2B	chr3	+	20125904	20126113	20122667	20122804	20136941	20137052	0	0	2	0	0	1	2	1	1	0	0	0	98	49	0.318735	0.50058381	NA	NA	0.5	1	1	1	-0.5
SE	4806	GNB1	chr1	-	1839189	1839238	1825396	1825499	1890819	1891117	0	2	5	0	1	0	0	1	0	0	0	0	98	49	0.318735	0.50058381	NA	0.5	1	NA	1	NA	-0.25
SE	6222	DNAJB12	chr10	-	72338201	72338311	72336523	72336696	72340788	72340868	0	0	2	0	0	1	0	0	1	0	0	0	98	49	0.318735	0.50058381	NA	NA	0.5	NA	NA	1	-0.5
SE	12813	SRP54	chr14	+	35013345	35013494	35011508	35011659	35013801	35013902	0	0	2	0	0	1	0	1	1	0	0	0	98	49	0.318735	0.50058381	NA	NA	0.5	NA	1	1	-0.5
SE	10593	RNF213	chr17	+	80389172	80389367	80388611	80388689	80389827	80389917	0	7	56	0	0	1	2	23	1	0	1	0	98	49	0.320121262	0.502236176	NA	1	0.966	1	0.92	1	0.01
RI	389	GMPR2	chr14	+	24238245	24239241	24238245	24238405	24238588	24239241	1	0	7	0	0	1	0	8	3	0	0	0	98	49	0.32027318	0.805848647	1	NA	0.778	NA	1	1	-0.111
A3SS	3968	SUN2	chr22	-	38750224	38750383	38750224	38750320	38750897	38751035	0	0	2	0	1	1	0	2	1	0	0	1	98	49	0.320307546	0.538835124	NA	0	0.5	NA	1	0.333	-0.416
SE	7718	THEMIS2	chr1	+	27879643	27880054	27876587	27876728	27881970	27882125	0	7	42	0	0	1	1	25	11	0	0	0	98	49	0.322009305	0.504671517	NA	1	0.955	1	1	1	-0.022
SE	372	AGTPBP1	chr9	-	85692688	85692813	85681267	85681335	85741774	85742029	0	3	9	0	0	1	3	8	1	0	0	0	98	49	0.326777752	0.511611417	NA	1	0.818	1	1	1	-0.091
SE	13193	MBOAT7	chr19	-	54187160	54187287	54183520	54183680	54188216	54188346	0	10	24	0	0	1	2	13	11	0	0	0	98	49	0.327855035	0.51276391	NA	1	0.923	1	1	1	-0.038
SE	2938	LIMK2	chr22	+	31225719	31225819	31212263	31212424	31258290	31258426	0	14	29	0	0	0	3	19	8	0	0	5	98	49	0.328408932	0.512945188	NA	1	1	1	1	0.444	0.185
SE	3975	GLT1D1	chr12	+	128888638	128888744	128875913	128876062	128899235	128899287	0	6	18	0	0	1	0	7	2	0	1	0	98	49	0.328653504	0.512945188	NA	1	0.9	NA	0.778	1	0.061
SE	2767	LST1	chr6	+	31587943	31587966	31587199	31587318	31588517	31588909	0	0	7	0	0	4	0	0	1	0	4	0	72	49	0.329153667	0.513192906	NA	NA	0.544	NA	0	1	0.044
SE	4457	CLEC7A	chr12	-	10123244	10123363	10116780	10118590	10125296	10125448	0	0	38	0	0	8	0	16	2	0	1	0	98	49	0.329686827	0.513491504	NA	NA	0.704	NA	0.889	1	-0.241
SE	10068	ACTN1	chr14	-	68909954	68910042	68909317	68909396	68912155	68912242	0	9	25	0	2	0	0	18	1	0	2	1	98	49	0.331082125	0.515130884	NA	0.692	1	NA	0.818	0.333	0.27
A3SS	1213	CIB1	chr15	-	90232218	90232447	90232218	90232327	90233668	90233703	0	0	5	0	0	1	2	0	2	0	0	0	98	49	0.332444824	0.545043142	NA	NA	0.714	1	NA	1	-0.286
A3SS	1850	OAS1	chr12	+	112919388	112919907	112919486	112919907	112916508	112916738	0	0	5	0	0	1	0	12	2	0	1	0	98	49	0.332444824	0.545043142	NA	NA	0.714	NA	0.857	1	-0.215
RI	641	ATPIF1	chr1	+	28236360	28238105	28236360	28236452	28237836	28238105	0	0	5	0	0	1	0	0	2	0	0	0	98	49	0.332444824	0.810334259	NA	NA	0.714	NA	NA	1	-0.286
SE	7846	RPS6KA1	chr1	+	26546866	26546983	26545851	26546035	26547188	26547270	0	3	19	0	0	1	0	10	6	0	0	0	98	49	0.332491859	0.516037235	NA	1	0.905	NA	1	1	-0.047
SE	9991	TRANK1	chr3	-	36892900	36892984	36892201	36892340	36899108	36899259	0	0	15	0	0	2	1	3	3	0	0	0	98	49	0.332635761	0.516037235	NA	NA	0.789	1	1	1	-0.211
SE	10092	NUMB	chr14	-	73279280	73279424	73275209	73277293	73282358	73282505	0	0	2	0	0	10	0	0	0	0	0	2	98	49	0.332694665	0.516037235	NA	NA	0.091	NA	NA	0	0.091
A3SS	653	VNN3	chr6	-	132728740	132728979	132728740	132728933	132734252	132734383	0	0	9	0	0	4	0	23	1	0	2	0	95	49	0.33308192	0.545043142	NA	NA	0.537	NA	0.856	1	-0.391
SE	13633	CCND3	chr6	-	41936558	41936695	41934932	41936107	41937234	41937394	0	3	19	0	2	0	0	2	2	0	0	2	98	49	0.333586462	0.516887065	NA	0.429	1	NA	1	0.333	0.048
SE	9234	RPS24	chr10	+	78040203	78040225	78037193	78037304	78040614	78040715	0	0	11	0	0	26	5	22	0	0	6	1	71	49	0.336551286	0.520943957	NA	NA	0.226	1	0.717	0	-0.346
SE	2900	DDX17	chr22	-	38495795	38495937	38494885	38495046	38498084	38498150	0	1	31	0	0	1	3	22	2	0	0	0	98	49	0.337825803	0.522378788	NA	1	0.939	1	1	1	-0.03
SE	12582	CNN2	chr19	+	1036129	1036246	1032558	1032696	1036415	1036562	0	1	25	0	0	1	0	17	2	0	0	0	98	49	0.338322411	0.522609027	NA	1	0.926	NA	1	1	-0.037
SE	7966	OAS3	chr12	+	112941569	112941852	112938432	112938707	112944475	112944651	0	7	34	0	0	1	0	17	11	0	0	0	98	49	0.338676564	0.52261897	NA	1	0.944	NA	1	1	-0.028
SE	12901	TYROBP	chr19	-	35907217	35907264	35904400	35904634	35907445	35907580	0	18	77	0	0	1	0	30	10	0	1	0	96	49	0.342984435	0.527529386	NA	1	0.975	NA	0.939	1	0.018
SE	4627	SORL1	chr11	+	121570156	121570270	121566939	121567113	121574240	121574363	0	0	56	0	1	12	0	27	2	4	1	1	98	49	0.343393791	0.527529386	NA	0	0.7	0	0.931	0.5	-0.127
SE	5558	SPATA13	chr13	+	24286764	24286950	24286213	24286393	24288998	24289178	0	0	5	0	0	2	0	1	1	0	1	0	98	49	0.34342743	0.527529386	NA	NA	0.556	NA	0.333	1	-0.11
SE	11729	PER1	chr17	-	8146896	8147002	8146593	8146765	8147249	8147381	0	0	5	0	0	2	0	1	1	0	2	0	98	49	0.34342743	0.527529386	NA	NA	0.556	NA	0.2	1	-0.044
SE	12395	TMEM127	chr2	-	96265137	96265489	96254832	96254997	96265868	96266013	0	3	13	0	0	1	0	7	4	0	0	0	98	49	0.343613619	0.527529386	NA	1	0.867	NA	1	1	-0.067
SE	13844	C10orf54	chr10	-	71755358	71755466	71752974	71753002	71760867	71760924	0	3	48	0	0	1	2	32	4	0	0	0	98	49	0.344174583	0.527680329	NA	1	0.96	1	1	1	-0.02
SE	13536	ALOX5	chr10	+	45382482	45382681	45374175	45374429	45395854	45395936	1	6	67	0	0	2	1	16	6	0	0	0	98	49	0.344414107	0.527680329	1	1	0.944	1	1	1	-0.019
SE	12400	CSF2RB	chr22	+	36925986	36926177	36923243	36923367	36929401	36929559	0	1	39	0	0	1	1	24	4	0	0	0	98	49	0.345199125	0.528344486	NA	1	0.951	1	1	1	-0.024
SE	4506	PTPN6	chr12	+	6960805	6960945	6960343	6960435	6961125	6961316	0	1	28	0	0	1	7	18	3	0	0	0	98	49	0.346027303	0.528422573	NA	1	0.933	1	1	1	-0.033
SE	9933	PTPRE	chr10	+	128076602	128076728	128073336	128073471	128077616	128077783	0	3	39	0	0	0	0	24	2	0	0	1	98	49	0.346119905	0.528422573	NA	1	1	NA	1	0.5	0.25
SE	7719	THEMIS2	chr1	+	27879643	27880054	27876587	27876728	27885294	27885451	0	6	25	0	3	1	1	12	8	0	1	0	98	49	0.346439311	0.528422573	NA	0.5	0.926	1	0.857	1	-0.239
SE	12745	DYSF	chr2	+	71620546	71620609	71613333	71613410	71643964	71644063	0	0	13	0	0	3	0	3	2	0	2	0	98	49	0.346656459	0.528422573	NA	NA	0.684	NA	0.429	1	-0.03
SE	13437	HBP1	chr7	+	107189291	107189448	107186568	107186681	107190172	107190317	0	1	1	0	0	1	0	1	1	0	3	0	98	49	0.348964996	0.531353191	NA	1	0.333	NA	0.143	1	0.095
SE	9997	GCH1	chr14	-	54844000	54844143	54842005	54843054	54845767	54845852	0	0	1	0	0	2	0	0	0	1	0	1	98	49	0.349286063	0.531353191	NA	NA	0.2	0	NA	0	0.2
SE	3661	ETS2	chr21	+	38821585	38821704	38819502	38819766	38822673	38824954	0	0	8	0	0	3	3	13	1	0	0	0	98	49	0.35298525	0.535895789	NA	NA	0.571	1	1	1	-0.429
SE	9992	TRANK1	chr3	-	36895639	36895758	36892900	36892984	36899108	36899259	0	0	8	0	0	3	0	4	1	0	1	0	98	49	0.35298525	0.535895789	NA	NA	0.571	NA	0.667	1	-0.263
SE	11324	ITGAL	chr16	+	30479330	30479461	30479090	30479208	30481438	30481584	0	0	11	0	0	4	0	6	1	0	0	0	98	49	0.355559836	0.539081642	NA	NA	0.579	NA	1	1	-0.421
SE	2458	MPP1	chrX	-	154799744	154799893	154792141	154792285	154805271	154805527	0	0	1	0	0	6	0	0	0	0	0	2	98	49	0.355801057	0.539081642	NA	NA	0.077	NA	NA	0	0.077
A3SS	564	PHF20L1	chr8	+	132777791	132777911	132777795	132777911	132775357	132775645	0	0	1	0	3	3	0	3	0	2	5	3	53	49	0.358798866	0.579927566	NA	0	0.236	0	0.357	0	-0.001
SE	2884	MKL1	chr22	-	40594673	40594735	40552105	40552367	40636477	40636719	0	0	4	0	0	2	0	0	1	0	0	0	98	49	0.359545146	0.544205795	NA	NA	0.5	NA	NA	1	-0.5
SE	3751	ARHGAP27	chr17	-	45396486	45396585	45396206	45396284	45396667	45396790	0	4	14	0	0	1	0	8	3	0	0	0	98	49	0.360380165	0.544920914	NA	1	0.875	NA	1	1	-0.062
A3SS	140	LY6E	chr8	+	143020882	143020991	143020887	143020991	143018484	143018586	0	0	2	0	0	1	2	1	1	0	1	0	54	49	0.360843819	0.579927566	NA	NA	0.645	1	0.476	1	-0.18
SE	18	FLNA	chrX	-	154354615	154354711	154354380	154354483	154354824	154355072	0	2	25	0	0	1	0	15	2	0	0	0	98	49	0.361294614	0.545754578	NA	1	0.926	NA	1	1	-0.037
SE	12741	DYSF	chr2	+	71513239	71513332	71511806	71511921	71513715	71513921	0	1	10	0	0	1	0	3	2	0	1	0	98	49	0.362088102	0.546301307	NA	1	0.833	NA	0.6	1	0.116
SE	12142	TRIM25	chr17	-	56895520	56895604	56895342	56895441	56895925	56895952	0	0	13	0	0	1	0	15	4	0	0	0	98	49	0.362383502	0.546301307	NA	NA	0.867	NA	1	1	-0.133
SE	275	RAPGEF1	chr9	-	131630235	131630324	131629101	131629254	131638634	131638791	0	0	6	0	0	2	0	8	1	0	1	0	98	49	0.365054683	0.547059405	NA	NA	0.6	NA	0.8	1	-0.3
SE	5253	SEC31A	chr4	-	82848803	82848977	82844385	82844509	82851430	82851604	0	0	3	0	0	1	0	4	1	0	0	0	98	49	0.365118555	0.547059405	NA	NA	0.6	NA	1	1	-0.4
SE	6132	ANKRD13D	chr11	+	67299057	67299124	67291990	67292180	67299529	67299611	0	0	3	0	0	1	0	8	1	0	0	0	98	49	0.365118555	0.547059405	NA	NA	0.6	NA	1	1	-0.4

SE	7202	MDM4	chr1	+	204544534	204544684	204542783	204542944	204546796	204546877	0	0	3	0	0	1	0	7	1	0	0	0	98	49	0.365118555	0.547059405	NA	NA	0.6	NA	1	1	-0.4
SE	13919	LUCAT1	chr5	-	91313077	91313229	91303872	91303971	91313637	91313777	0	2	3	0	0	1	0	0	1	0	0	0	98	49	0.365118555	0.547059405	NA	1	0.6	NA	NA	1	-0.2
SE	1533	HLA-A	chr6	+	29944499	29944616	29944121	29944397	29945058	29945091	1	41	187	0	1	2	5	230	19	0	6	0	98	49	0.3653902	0.547059405	1	0.953	0.979	1	0.95	1	-0.006
SE	3397	APMAP	chr20	-	24978766	24978882	24973644	24973737	24983902	24984019	0	1	5	0	0	1	0	2	2	0	0	0	98	49	0.365434227	0.547059405	NA	1	0.714	NA	1	1	-0.143
SE	11877	MGAM	chr7	+	142036822	142036977	142036168	142036285	142038530	142038615	0	0	20	0	0	1	4	3	6	0	0	0	98	49	0.366623914	0.548294271	NA	NA	0.909	1	1	1	-0.091
SE	2284	IL1RAP	chr3	+	190556129	190556216	190514050	190514219	190564288	190564353	0	3	7	0	0	1	0	4	2	0	0	0	98	49	0.367396868	0.548904067	NA	1	0.778	NA	1	1	-0.111
SE	12976	RPS19	chr19	+	41869030	41869214	41861111	41861212	41869698	41869753	1	6	58	0	0	1	3	38	4	0	0	0	98	49	0.368911011	0.550618918	1	1	0.967	1	1	1	-0.011
SE	8375	OAS2	chr12	+	112987037	112987308	112978468	112978785	112995295	112995474	0	6	42	0	0	3	3	7	1	0	0	0	98	49	0.370767489	0.552840809	NA	1	0.875	1	1	1	-0.062
SE	6279	IDS	chrX	-	149491537	149491667	149490313	149490440	149496345	149496516	0	0	1	0	0	7	0	0	0	0	2	2	98	49	0.37479931	0.558298674	NA	NA	0.067	NA	0	0	0.067
SE	6619	AMPD2	chr1	+	109629795	109629916	109629326	109629490	109630232	109630406	0	1	7	0	0	3	0	7	1	0	4	0	98	49	0.376779701	0.560692961	NA	1	0.538	NA	0.467	1	0.035
SE	12898	TXNIP	chr1	-	145994543	145994800	145994280	145994437	145994928	145995031	0	17	121	0	0	1	4	61	19	0	0	0	98	49	0.378921937	0.563323118	NA	1	0.984	1	1	1	-0.008
A3SS	3632	STXBP2	chr19	+	7640697	7640809	7640730	7640809	7639730	7639807	0	0	7	0	1	1	2	0	2	0	0	0	82	49	0.379879433	0.605117681	NA	0	0.807	1	NA	1	-0.597
RI	545	CDK5RAP3	chr17	+	47974399	47975337	47974399	47974448	47975158	47975337	0	7	10	0	0	1	0	4	5	0	0	0	98	49	0.38169658	0.878475733	NA	1	0.833	NA	1	1	-0.084
SE	8578	RC3H1	chr1	-	173983417	173983657	173982726	173982902	173984498	173984619	0	1	7	0	0	1	0	3	1	0	1	0	98	49	0.382084955	0.567464117	NA	1	0.778	NA	0.6	1	0.089
SE	11360	TATDN2	chr3	+	10249194	10249614	10248492	10249067	10260136	10260670	0	3	6	0	0	1	2	4	1	0	0	0	98	49	0.382895649	0.568106772	NA	1	0.75	1	1	1	-0.125
RI	660	CSF3R	chr1	-	36466042	36466827	36466042	36466200	36466620	36466827	1	19	165	0	0	1	7	83	24	0	0	0	98	49	0.382925319	0.878475733	1	1	0.988	1	1	1	-0.004
SE	7522	AQP9	chr15	+	58174917	58175036	58173067	58173205	58179127	58179345	0	1	43	0	0	1	3	20	6	0	0	0	98	49	0.383614897	0.568612613	NA	1	0.956	1	1	1	-0.022
SE	802	TBXAS1	chr7	+	139957633	139957764	139955458	139955607	139961918	139962233	0	3	22	0	1	0	2	4	2	0	0	0	98	49	0.385851284	0.571364019	NA	0.6	1	1	1	1	-0.2
SE	8267	DPYD	chr1	-	97549559	97549744	97515725	97515941	97573759	97573970	0	4	25	0	0	1	0	11	4	0	1	0	98	49	0.389051763	0.57553622	NA	1	0.926	NA	0.846	1	0.04
A5SS	1166	IRF2BP2	chr1	-	234608446	234609525	234608494	234609525	234604268	234607852	0	0	5	0	1	5	0	4	0	0	10	1	97	49	0.389300363	0.614437923	NA	0	0.336	NA	0.168	0	0.084
SE	306	SH2D3C	chr9	-	127741787	127741959	127740257	127740369	127742848	127742964	0	2	7	0	1	0	0	2	2	0	0	0	98	49	0.390828967	0.577596791	NA	0.5	1	NA	1	1	-0.25
SE	4458	CLEC7A	chr12	-	10125086	10125173	10116780	10118590	10125296	10125448	0	3	18	0	0	8	0	4	3	0	1	0	98	49	0.391525682	0.578058056	NA	1	0.529	NA	0.667	1	-0.069
SE	6890	EP300	chr22	+	41135812	41135906	41131387	41131633	41137652	41137790	0	2	14	0	0	1	0	8	1	0	0	0	98	49	0.392944473	0.579315862	NA	1	0.875	NA	1	1	-0.062
SE	3233	SLC9A8	chr20	+	49839540	49839599	49823060	49823141	49845035	49845119	0	0	8	0	0	1	0	0	2	0	0	0	98	49	0.393533929	0.579315862	NA	NA	0.8	NA	NA	1	-0.2
SE	11134	ECE1	chr1	-	21225249	21225440	21221746	21221842	21227158	21227226	1	0	8	0	0	1	0	12	2	0	0	0	98	49	0.393533929	0.579315862	1	NA	0.8	NA	1	1	-0.1
SE	11656	NLRP1	chr17	-	5517745	5517887	5515472	5515517	5530480	5530704	0	0	7	0	1	0	0	2	3	0	0	0	98	49	0.39950276	0.587426728	NA	0	1	NA	1	1	-0.5
SE	1505	HLA-E	chr6	+	30491536	30491653	30491136	30491412	30492403	30492436	0	33	293	0	0	1	21	137	29	0	0	0	98	49	0.399825378	0.587426728	NA	1	0.993	1	1	1	-0.004
SE	5153	FTH1	chr11	-	61964986	61965112	61964284	61964891	61965368	61965515	0	8	114	0	0	1	7	66	2	0	0	0	98	49	0.400407723	0.587707819	NA	1	0.983	1	1	1	-0.008
SE	11835	NCOA1	chr2	+	24768078	24768135	24762686	24762776	24768220	24770701	0	0	8	0	0	2	0	2	1	0	0	0	98	49	0.401159139	0.58823628	NA	NA	0.667	NA	1	1	-0.333
A3SS	909	TPD52L2	chr20	+	63882718	63882820	63882749	63882820	63875815	63875875	0	2	11	0	0	1	3	5	2	0	0	0	80	49	0.401186798	0.63162252	NA	1	0.871	1	1	1	-0.065
SE	9689	IFRD1	chr7	+	112462269	112462378	112462000	112462179	112467980	112468115	0	0	10	0	3	37	4	3	0	2	22	2	98	49	0.40162165	0.588340487	NA	0	0.119	0.5	0.064	0	-0.129
A3SS	4073	CFLAR	chr2	+	201162995	201172688	201163834	201172688	201160431	201160942	0	0	4	0	3	38	2	3	2	7	14	3	98	49	0.40353661	0.63162252	NA	0	0.05	0.125	0.097	0.25	-0.132
SE	13946	AP3D1	chr19	-	2108686	2108766	2100987	2102268	2109085	2109207	0	2	2	0	0	1	0	1	1	0	0	0	98	49	0.409859466	0.599823542	NA	1	0.5	NA	1	1	-0.25
SE	13218	LILRA5	chr19	-	54311863	54312148	54311164	54311716	54312536	54312621	0	1	18	0	0	1	0	9	1	0	0	0	98	49	0.410993926	0.600898707	NA	1	0.9	NA	1	1	-0.05
SE	6789	RAB4B-EGLN2	chr19	+	40806554	40806674	40800338	40801415	40807137	40807274	0	2	3	0	0	0	0	1	0	0	0	1	98	49	0.412150794	0.601420042	NA	1	1	NA	1	0	0.5
SE	7891	FHL3	chr1	-	37999256	37999432	37998973	37999148	38005356	38005515	0	2	3	0	0	0	0	1	0	0	0	1	98	49	0.412150794	0.601420042	NA	1	1	NA	1	0	0.5
SE	12902	TYROBP	chr19	-	35907729	35907762	35907445	35907580	35908167	35908309	0	4	15	0	0	5	0	7	3	0	1	0	82	49	0.416321308	0.606916515	NA	1	0.642	NA	0.807	1	-0.083
RI	981	ACAA1	chr3	-	38126161	38126700	38126161	38126341	38126509	38126700	0	0	9	0	0	1	0	1	3	0	1	0	98	49	0.421251047	0.934008656	NA	NA	0.818	NA	0.333	1	0.151
SE	5079	WDR13	chrX	+	48599586	48599717	48599352	48599462	48600318	48600626	0	1	4	0	0	0	0	1	0	0	0	1	98	49	0.421888846	0.613929698	NA	1	1	NA	1	0	0.5
SE	11377	ARHGEF1	chr19	+	41906456	41906620	41905938	41906025	41906702	41906803	0	0	18	0	0	2	0	5	3	0	0	0	98	49	0.422105527	0.613929698	NA	NA	0.818	NA	1	1	-0.182
SE	3692	DOK3	chr5	-	177508236	177508542	177505010	177505110	177509474	177509575	0	7	32	0	0	1	0	13	5	0	0	0	98	49	0.42235749	0.613929698	NA	1	0.941	NA	1	1	-0.03
SE	13638	RNF114	chr20	+	49941560	49941711	49936376	49936552	49945381	49945488	0	1	2	0	0	0	0	0	1	0	1	0	98	49	0.423875417	0.615287988	NA	1	1	NA	0	1	0.5
SE	5214	SUCO	chr1	+	172551511	172551626	172533117	172533497	172553259	172553370	0	1	1	0	0	0	1	1	0	0	0	1	98	49	0.424110682	0.615287988	NA	1	1	1	1	0	0.333
SE	5013	ADRBK1	chr11	+	67283706	67283773	67283127	67283228	67283853	67283949	0	13	34	0	5	0	4	7	17	0	1	0	98	49	0.425222253	0.6162202	NA	0.565	1	1	0.778	1	-0.144
SE	2290	BCL6	chr3	-	187728359	187728544	187726730	187726898	187729049	187730021	0	2	24	0	0	1	3	8	4	0	0	0	98	49	0.425573232	0.6162202	NA	1	0.923	1	1	1	-0.038
A5SS	1299	WDR26	chr1	-	224431476	224431581	224431524	224431581	224424517	224424654	0	0	11	0	0	4	4	5	1	0	2	0	97	49	0.426432576	0.665031756	NA	NA	0.581	1	0.558	1	-0.272
SE	6186	HERC3	chr4	+	88681158	88681325	88680092	88680236	88686735	88686802	0	0	12	0	0	2	0	8	2	0	0	0	98	49	0.429038403	0.620451665	NA	NA	0.75	NA	1	1	-0.25
SE	2247	MAEA	chr4	+	1327626	1327703	1315396	1315600	1332756	1332865	0	3	2	0	1	1	0	1	0	0	1	0	98	49	0.429432607	0.620451665	NA	0.6	0.5	NA	0.333	NA	0.217
SE	12900	TYROBP	chr19	-	35907217	35907261	35904400	35904634	35907445	35907580	0	7	39	0	0	1	0	22	1	0	1	0	93	49	0.429733987	0.620451665	NA	1	0.954	NA	0.921	1	0.016
SE	679	SARAF	chr8	-	30073876	30074055	30069641	30070059	30082846	30083208	0	3	13	0	1	5	0	17	4	0	5	6	98	49	0.430454253	0.620561858	NA	0.6	0.565	NA	0.63	0.25	0.143
SE	5660	KCNQ1	chr11	+	2585211	2585307	2583434	2583545	2587569	2587692	0	0	4	0	1	0	0	1	1	0	0	0	98	49	0.430636073	0.620561858	NA	0	1	NA	1	1	-0.5
RI	694	UNC119	chr17	-	28546706	28547849	28546706	28547409	28547676	28547849	0	1	10	0	0	1	2	3	2	1	0	0	98	49	0.431080918	0.934008656	NA	1	0.833	0.5	1	1	0.083
A3SS	3011	ISG20	chr15	+	88650234	88652310	88652109	88652310	88639342	88639594	0	0	2	0	0	11	0	3	1	0	1	2	98	49	0.432135137	0.670554523	NA	NA	0.083	NA	0.6	0.2	-0.317
SE	13731	SERPINA1	chr14	-	94388559	94388660	94382591	94383241	94390456	94390692	0	1	16	0	2	20	4	5	1	0	5	0	98	49	0.432495196	0.621784006	NA	0.2	0.286	1	0.333	1	-0.535
SE	1921	PARP8	chr5	+	50821212	50821338	50815431	50815524	50822334	50822400	0	2	9	0	0	1	0	4	1	0	0	0	98	49	0.432725263	0.621784006	NA	1	0.818	NA	1	1	-0.091
SE	2181	RBM47	chr4	-	40437770	40438924	40432650	40432862	40466576	40466699	0	2	9	0	0	1	0	4	1	0	0	0	98	49	0.432725263	0.621784006	NA	1	0.818	NA	1	1	-0.091
A5SS	1440	MLKL	chr16	-	74675323	74675404	74675354	74675404	74674959	74675100	1	1	1	0	5	3	0	3	0	1	1	0	80	49	0.432994858	0.667321487	1	0.109	0.17	0	0.648	NA	0.102
SE	4557	SLC6A6	chr3	+	14443623	14443863	14416411	14416453	14445716	14445851	0	5	30	0	0	1	0	16	5	0	1	0	98	49	0.434955535	0.624391756	NA	1	0.937	NA	0.889	1	0.024
SE	5786	DENND3	chr8	+	141150833	141150953	141138021	141138137	141151618	141151837	0	0	22	0	0	1	0	10	4	0	0	0	98	49	0.435694354	0.624855547	NA	NA	0.917	NA	1	1	-0.083
SE	3704	LYZ	chr12	+	69352219	69352298	69350107	69350272	69353152	69354233	0	40	456	0	0	1	43	267	1	0	0	0	98	49	0.441484717	0.632034884	NA	1	0.996	1	1	1	-0.002
SE	2699	MBNL1	chr3	+	152299404	152300367	152268039	152269092	152414940	152415111	0	0	6	0	0	1	5	3	1	0	1	0	98	49	0.441541336	0.632034884	NA	NA	0.75	1	0.6	1	-0.117
SE	13538	ALOX5	chr10	+	45428617	45428764	45424959	45425132	45440429	45440633	0	9	46	0	0	1	0	19	4	0	0	0	98	49	0.442803979	0.633239183	NA	1	0.958	NA	1	1	-0.021
SE	3207	STX16	chr20	+	58669290	58669453	58667986	58668127	58670511	58670603	0	0	9	0	0	3	0	9	1	0	4	0	98	49	0.44356605	0.633726019	NA	NA	0.6	NA	0.529	1	-0.164
SE	10590	RNF213	chr17	+	80379619	80379714	80377761	80377796	80380830	80380987	0	3	53	0	0	1	0	22	3	0	0	0	98	49	0.44486502	0.634978277	NA	1	0.964	NA	1	1	-0.018
A5SS	2190	HES4	chr1	-	999691	1000172	999865	1000172	999525	999613	1	1	1	0	1	1	3	11	4	0	4	1	98	49	0.445770867	0.679023064	1	0.333	0.333	1	0.579	0.667	-0.193
SE	1983	DDX60L	chr4	-	168448637	168448779	168441336	168441492	168453123	168453282	0	2	53	0	0	1	0	35	2	0	1	0	98	49	0.445818676	0.63573574	NA	1	0.964	NA	0.946	1	0.009
SE	4191	FAM49B	chr8	-	129879388	129879471	129871374	129871496	129903311	129903350	0	0	14	0	0	1	4	13	4	0	4	0	98	49	0.449034561	0.639399819	NA	NA	0.875	1	0.619	1	0.002
SE	13835	SPG21	chr15	-	64983506	64983593	64980863	64981025	64989664	64989946	0	1	6	0	0	1	0	2	1	0	0	0	98	49	0.449609977	0.639399819	NA	1	0.75	NA	1	1	-0.125
SE	11826	ARHGDIB	chr12	-	14949801	14949885	14947872	14947949	14950531	14950724	0	13	90	0	0	1	12	34	7	0	0	0	98	49	0.44966441	0.639399819	NA	1	0.978	1	1	1	-0.011
RI	780	SHISA5	chr3	-	48467797	48469573	48467797	48469186	48469360	48469573	0	3	1	0	5	13	5	0	1	0	10	2	98	49	0.455153121	0.94773154	NA	0.231	0.037	1	0	0.2	-0.266
SE	2102	WDFY3	chr4	-	84817391	84817585	84809886	84810344	84820084	84820186	0	1	19	0	1	0	1	6	3	0	0	0	98	49	0.458220849	0.650950791	NA	0.333	1	1	1	1	-0.334
SE	13718	HMHA1	chr19	+	1068413	1068744	1067165	1067495	1073148	1073292	0	8	14	0	0	1	0	2	6	0	0	0	98	49	0.460039403	0.652917113	NA	1	0.875	NA	1	1	-0.062
SE	8333	NCSTN	chr1	+	160355654	160355757	160354117	160354290	160355862	160355958	0	1	4	0	0	1	0	1	1	0	0	0	98	49	0.46501066	0.659350021	NA	1	0.667	NA	1	1	-0.166
A3SS	2675	ABTB1	chr3	+	127674344	127674418	127674390	127674418	127672937	127673081	0	0	8	0	3	0	0	2	0	0	3	2	95	49	0.468058768	0.720090412	NA	0	1	NA	0.256	0	0.372
SE	1195	MAP3K5	chr6	-	136720449	136720589	136705109	136705133	136791709	136792518	0	1	12	0	0	1	0	4	1	0	0	0	98	49	0.46879248	0.66408586	NA	1	0.857	NA	1	1	-0.072
A5SS	3016	SERPINA1	chr14	-	94390419	94390692	94390456	94390692	94382591	94383241	0	4	5	0	2	20	3	3	2	0	5	0	86	49	0.47090828	0.709068789	NA	0.533	0.125	1	0.255	1	-0.423
SE	9545	PRKCB	chr16	+	24172269	24172361	24154683	24154857	24174517	24174580	0	9	27	0	0	1	0	11	2	0	0	0	98	49	0.472851524	0.669205122	NA	1	0.931	NA	1	1	-0.034
A3SS	1443	TBC1D10C	chr11	+	67405902	67406017	67405906	67406017	67405594	67405701	0	0	7	0	0	2	0	0	1	0	0	0	53	49	0.472854846	0.721304002	NA	NA	0.764	NA	NA	1	-0.236
RI	556	CDC16	chr13	+	114272183	114272675	114272183	114272481	114272563	114272675	0	0	7	0	0	2	0	1	1	0	0	0	98	49	0.47352014	0.94773154	NA	NA	0.636	NA	1	1	-0.364
SE	11966	MSL1	chr17	-	40131536	40131584	40129244	40129627	40132033	40132098	0	0	7	0	0	2	0	4	1	0	1	0	97	49	0.474814393	0.671350925	NA	NA	0.639	NA	0.669	1	-0.196
SE	2939	LIMK2	chr22	+	31266983	31267070	31265945	31266132	31267775	31267907	0	0	20	0	0	1	0	34	5	0	0	0	98	49	0.475908434	0.671934183	NA	NA	0.909	NA	1	1	-0.091
SE	1139	IGF2R	chr6	+	160061752	160061928	160061502	160061646	160062531	160062619	0	5	27	0	0	1	0	13	8	0	1	0	98	49	0.476121028	0.671934183	NA	1	0.931	NA	0.867	1	0.032
SE	3270	CTSA	chr20	+	45893219	45893311	45892724	45892880	45893987	45894072	0	1	14	0	1	0	0	4	2	2	0	0	98	49	0.476656296	0.672058549	NA	0.333	1	0	1	1	0
SE	5812	HAUS4	chr14	-	22951554	22951689	22950313	22950410	22952327	22952459	0	0	16	0	0	1	0	3	2	0	0	0	98	49	0.480086309	0.676260283	NA	NA	0.889	NA	1	1	-0.111
RI	1055	STX10	chr19	-	13144057	13144671	13144057	13144486	13144576	13144671	0	1	6	0	0	1	0	0	1	0	1	0	98	49	0.482241355	0.94773154	NA	1	0.75	NA	0	1	0.375
A5SS	2804	TYROBP	chr19	-	35908167	35908309	35908174	35908309	35907729	35907762	0	1	10	0	0	1	0	3	4	0	1	0	56	49	0.482508271	0.718279358	NA	1	0.897	NA	0.724	1	0.087
SE	8138	ITGAX	chr16	+	31371333	31371497	31371083	31371214	31371629	31371784	0	1	13	0	1	0	0	10	6	0	3	1	98	49	0.482836455	0.67949737	NA	0.333	1	NA	0.625	0.75	-0.021
SE	12743	DYSF	chr2	+	71549349	71549391	71539156	71539239	71551040	71551156	0	2	2	0	1	11	0	2	1	0	4	0	91	49	0.489990933	0.688920834	NA	0.519	0.089	NA	0.212	1	-0.302
A3SS	1013	INPP5D	chr2	+	233125744	233125919	233125747	233125919	233122106	233122257	1	0	7	0	1	5	0	5	0	1	2	0	52	49	0.490380444	0.74107425	1	0	0.569	0	0.702	NA	0.172
SE	10208	RFWD2	chr1	-	176135009	176135086	176116623	176116681	176149005	176149074	0	1	21	0	0	1	0	7	1	0	0	0	98	49	0.493056216	0.692582704	NA	1	0.913	NA	1	1	-0.043
RI	322	MAP7D1	chr1	+	36176198	36176581	36176198	36176321	36176417	36176581	0	1	8	0	1	1	3	5	6	0	1	0	98	49	0.496928876	0.94773154	NA	0.333	0.8	1	0.714	1	-0.338
SE	4467	CLEC12A	chr12	+	9978965	9979064	9971408	9971687	9979335	9979524	0	0	25	0	0	3	0	0	2	0	0	0	98	49	0.497638055	0.696506389	NA	NA	0.806	NA	NA	1	-0.194
SE	4625	SORL1	chr11	+	121545242	121545429	121543547	121543726	121549959	121550088	0	6	54	0	0	1	0	38	2	0	1	0	98	49	0.497995567	0.696506389	NA	1	0.964	NA	0.95	1	0.007
A3SS	1731	SECISBP2	chr9	+	89325426	89325676	89325547	89325676	89319651	89319797	0	0	4	0	0	1	0	4	1	0	0	0	98	49	0.498166579	0.74107425	NA	NA	0.667	NA	1	1	-0.333
A3SS	3445	PHOSPHO1	chr17	-	49223369	49225131	49223369	49225004	49226646	49226758	0	0	4	0	0	1	0	2	1	0	0	0	98	49	0.498166579	0.74107425	NA	NA	0.667	NA	1	1	-0.333
RI	392	PRKD2	chr19	-	46716130	46717127	46716130	46716535	46716950	46717127	0	0	4	0	0	1	0	0	1	0	0	0	98	49	0.498166579	0.94773154	NA	NA	0.667	NA	NA	1	-0.333
SE	3691	ARHGEF40	chr14	+	21087319	21087463	21087000	21087105	21087967	21088098	0	0	4	0	0	1	0	0	1	0	0	0	98	49	0.498166579	0.696506389	NA	NA	0.667	NA	NA	1	-0.333
SE	6752	UBR4	chr1	-	19165655	19165757	19165248	19165349	19167021	19167231	0	0	4	0	0	1	0	4	1	0	1	0	98	49	0.498166579	0.696506389	NA	NA	0.667	NA	0.667	1	-0.166
SE	7087	PRDX5	chr11	+	64320660	64320792	64319733	64319868	64320864	64320903	0	0	4	0	0	1	0	5	1	0	0	0	98	49	0.498166579	0.696506389	NA	NA	0.667	NA	1	1	-0.333
SE	4125	XPO6	chr16	-	28177219	28177332	28175897	28176095	28180940	28181031	0	3	53	0	0	1	0	35	8	0	1	0	98	49	0.499162452	0.69697095	NA	1	0.964	NA	0.946	1	0.009
SE	13537	ALOX5	chr10	+	45395854	45395936	45382482	45382681	45412190	45412313	0	5	67	0	0	1	1	17	7	0	0	0	98	49	0.49942629	0.69697095	NA	1	0.971	1	1	1	-0.014
A5SS	2491	ZNF276	chr16	+	89727278	89727357	89727278	89727325	89729234	89729318	0	0	8	0	0	2	0	3	1	0	4	0	81	49	0.50024699	0.736318604	NA	NA	0.708	NA	0.312	1	0.052
SE	10650	SLC43A2	chr17	-	1591268	1591471	1590801	1590948	1591565	1591699	0	5	19	0	0	1	0	5	8	0	1	0	98	49	0.500994407	0.698510756	NA	1	0.905	NA	0.714	1	0.096
A3SS	1434	PICALM	chr11	-	85990249	85990399	85990249	85990378	85996825	85996929	0	0	18	0	0	1	0	21	4	0	1	0	70	49	0.504274672	0.744011811	NA	NA	0.926	NA	0.936	1	-0.042
SE	12757	PKN1	chr19	+	14469751	14469814	14469340	14469517	14469911	14469988	0	1	5	0	0	1	2	1	1	0	0	0	98	49	0.507272183	0.706608055	NA	1	0.714	1	1	1	-0.143
SE	6543	NRD1	chr1	-	51800555	51800683	51798248	51798411	51803813	51803964	0	0	18	0	0	1	0	10	4	0	0	0	98	49	0.507742839	0.706608784	NA	NA	0.9	NA	1	1	-0.1
SE	1150	SOD2	chr6	-	159688125	159688242	159684853	159685033	159692660	159692863	3	7	82	0	2	0	18	158	13	0	13	1	98	49	0.509005729	0.707237006	1	0.636	1	1	0.859	0.867	-0.03
SE	13859	MMP25	chr16	+	3057309	3057394	3057032	3057209	3057530	3057613	0	1	33	0	0	1	3	8	3	0	0	0	98	49	0.509135356	0.707237006	NA	1	0.943	1	1	1	-0.028
SE	8685	PTPRC	chr1	+	198699563	198699704	198692346	198692373	198703297	198703372	0	3	18	0	0	25	2	15	1	0	11	6	98	49	0.509721028	0.707396773	NA	1	0.265	1	0.405	0.077	0.139
SE	8376	OAS2	chr12	+	113004933	113005222	113002931	113003102	113006412	113006600	0	2	43	0	0	1	0	11	3	0	0	0	98	49	0.512035658	0.709568442	NA	1	0.956	NA	1	1	-0.022
SE	4905	PAK1	chr11	-	77332729	77332867	77322014	77323360	77336085	77336282	0	2	22	0	1	0	0	7	1	0	0	0	98	49	0.512585144	0.709568442	NA	0.5	1	NA	1	1	-0.25
SE	5056	SRSF3	chr6	+	36599820	36600276	36598848	36598983	36601151	36601190	0	1	11	0	0	4	0	7	1	0	1	0	98	49	0.512702147	0.709568442	NA	1	0.579	NA	0.778	1	-0.1
A5SS	3021	MAP4K4	chr2	+	101887087	101887261	101887087	101887237	101887777	101887937	0	0	2	0	0	5	0	5	1	0	2	1	73	49	0.51637545	0.751613155	NA	NA	0.212	NA	0.627	0.402	-0.302
SE	2564	HK3	chr5	-	176887200	176887337	176887001	176887121	176887450	176887746	0	10	17	0	1	0	2	5	2	0	0	0	98	49	0.517806019	0.715972812	NA	0.833	1	1	1	1	-0.084
SE	12585	SBNO2	chr19	-	1112188	1112301	1111995	1112067	1112401	1112537	0	8	15	0	4	0	0	8	3	0	2	1	98	49	0.521310981	0.720156622	NA	0.5	1	NA	0.667	0.6	0.117
SE	4558	SLC6A6	chr3	+	14443627	14443863	14416411	14416453	14445716	14445851	0	5	14	0	0	1	0	9	4	0	1	0	98	49	0.523615544	0.722675999	NA	1	0.875	NA	0.818	1	0.028
SE	5960	LILRA2	chr19	+	54574075	54574111	54573697	54573912	54574300	54574582	0	0	5	0	0	1	1	1	1	0	0	0	85	49	0.5248053	0.723653547	NA	NA	0.742	1	1	1	-0.258
SE	7023	HPCAL1	chr2	+	10408561	10408734	10396834	10396920	10419733	10420135	0	0	2	0	2	7	0	2	0	0	1	2	98	49	0.527686141	0.726959	NA	0	0.125	NA	0.5	0	-0.188
SE	9067	ERGIC1	chr5	+	172909666	172909761	172897001	172897074	172914713	172914838	0	1	13	0	0	1	0	1	3	0	0	0	98	49	0.528398477	0.727273728	NA	1	0.867	NA	1	1	-0.067
SE	11047	HLA-B	chr6	-	31355106	31355223	31354632	31354665	31355316	31355592	0	46	261	0	2	4	10	220	15	0	4	2	98	49	0.529614267	0.728280186	NA	0.92	0.97	1	0.965	0.789	0.027
SE	7715	THEMIS2	chr1	+	27876587	27876728	27872542	27872665	27879643	27879963	0	13	31	0	0	1	7	17	23	0	0	2	98	49	0.530434112	0.728740832	NA	1	0.939	1	1	0.852	0.019
A5SS	2259	TLE3	chr15	-	70058149	70058291	70058158	70058291	70057458	70057658	0	0	2	0	1	4	0	2	0	0	3	1	58	49	0.531185703	0.764673924	NA	0	0.297	NA	0.36	0	-0.032
A3SS	1208	SDF4	chr1	-	1216907	1217804	1216907	1217688	1218457	1218633	1	0	0	0	0	4	1	0	1	2	0	0	98	49	0.53213915	0.762573929	1	NA	0	0.2	NA	1	-0.1

SE	4662	AMICA1	chr11	-	118214823	118214886	118212406	118212561	118224940	118225094	0	7	17	0	1	5	0	13	2	0	2	0	98	49	0.537451897	0.737707946	NA	0.778	0.63	NA	0.765	1	-0.179
A3SS	1184	DDIT3	chr12	-	57517268	57517519	57517268	57517438	57517705	57517753	0	0	5	0	0	1	0	11	1	0	2	0	98	49	0.538906714	0.762573929	NA	NA	0.714	NA	0.733	1	-0.153
A3SS	1185	DDIT3	chr12	-	57517268	57517542	57517268	57517438	57517705	57517753	0	0	5	0	0	1	0	11	1	0	2	0	98	49	0.538906714	0.762573929	NA	NA	0.714	NA	0.733	1	-0.153
A3SS	1186	DDIT3	chr12	-	57517268	57517595	57517268	57517438	57517705	57517753	0	0	5	0	0	1	0	12	1	0	2	0	98	49	0.538906714	0.762573929	NA	NA	0.714	NA	0.75	1	-0.161
SE	8577	RC3H1	chr1	-	173964838	173965120	173963972	173964187	173970504	173970617	0	0	5	0	0	1	0	5	1	0	0	0	98	49	0.538906714	0.737743862	NA	NA	0.714	NA	1	1	-0.286
SE	1576	TRIM38	chr6	+	25973053	25973076	25971868	25972099	25973172	25973285	0	0	5	0	0	1	0	9	1	0	1	0	72	49	0.539026433	0.737743862	NA	NA	0.773	NA	0.86	1	-0.157
SE	3235	SLC9A8	chr20	+	49849578	49849680	49845035	49845119	49855437	49855581	0	0	10	0	0	2	0	6	1	0	2	0	98	49	0.539441453	0.737743862	NA	NA	0.714	NA	0.6	1	-0.086
SE	5886	RASSF5	chr1	+	206585179	206585295	206584386	206584684	206586825	206589284	0	0	10	0	0	2	0	10	1	0	6	0	98	49	0.539441453	0.737743862	NA	NA	0.714	NA	0.455	1	-0.014
A3SS	3256	FLII	chr17	-	18251679	18251816	18251679	18251813	18251998	18252146	0	2	6	0	2	3	0	1	0	0	2	0	52	49	0.542191316	0.762573929	NA	0.485	0.653	NA	0.32	NA	0.249
A3SS	116	AGTPBP1	chr9	-	85655142	85655320	85655142	85655200	85657434	85657643	0	0	20	0	0	4	0	8	1	0	2	0	98	49	0.542274794	0.762573929	NA	NA	0.714	NA	0.667	1	-0.12
SE	4629	SORL1	chr11	+	121605401	121605571	121605112	121605239	121606844	121606957	0	11	57	0	0	1	0	45	4	0	1	0	98	49	0.544440604	0.743903843	NA	1	0.966	NA	0.957	1	0.005
SE	2099	WDFY3	chr4	-	84726860	84726911	84724425	84724594	84733381	84733609	0	5	2	0	0	3	0	5	3	0	1	0	98	49	0.545895829	0.745214742	NA	1	0.25	NA	0.714	1	-0.232
SE	6349	FLI1	chr11	+	128807179	128807239	128805365	128805431	128809156	128809204	0	1	11	0	0	1	1	2	1	0	0	0	98	49	0.547491166	0.746714358	NA	1	0.846	1	1	1	-0.077
A5SS	1194	ETF1	chr5	-	138542633	138542936	138542832	138542936	138518691	138518867	0	0	2	0	2	4	0	4	3	0	3	0	98	49	0.547780994	0.779992502	NA	0	0.2	NA	0.4	1	-0.6
SE	1134	IGF2R	chr6	+	160029549	160029655	160027184	160027314	160032550	160032713	0	14	45	0	0	4	0	27	6	0	1	0	98	49	0.549943569	0.749379133	NA	1	0.849	NA	0.931	1	-0.041
SE	11237	APLP2	chr11	+	130123611	130123779	130122304	130122513	130126699	130126830	0	0	33	0	0	3	0	5	2	0	0	0	98	49	0.550569813	0.749552925	NA	NA	0.846	NA	1	1	-0.154
SE	8917	IRF9	chr14	+	24163877	24163959	24163377	24163508	24164062	24164134	0	3	25	0	1	5	0	7	3	0	8	0	98	49	0.551132735	0.749640272	NA	0.6	0.714	NA	0.304	1	0.005
SE	5788	DENND3	chr8	+	141160631	141160787	141155848	141155970	141165185	141165289	0	0	11	0	0	1	0	5	2	0	0	0	98	49	0.551645974	0.749659945	NA	NA	0.846	NA	1	1	-0.154
RI	675	PSME1	chr14	+	24138473	24138967	24138473	24138560	24138735	24138967	0	0	2	0	0	11	3	0	0	0	6	1	98	49	0.557006469	0.999202116	NA	NA	0.083	1	0	0	-0.25
A3SS	2017	RNF19B	chr1	-	32936445	32937309	32936445	32937259	32938396	32938528	0	1	9	0	0	5	0	3	2	0	5	0	98	49	0.558523697	0.779335391	NA	1	0.474	NA	0.231	1	0.121
SE	13539	ALOX5	chr10	+	45444115	45444286	45443727	45443828	45445507	45446119	0	2	41	0	0	2	1	4	7	0	0	1	98	49	0.559152751	0.759174873	NA	1	0.911	1	1	0.778	0.029
SE	4964	DDX3X	chrX	+	41343736	41343822	41343215	41343351	41344029	41344128	0	4	36	0	2	0	0	34	1	0	4	1	98	49	0.565342278	0.76688578	NA	0.5	1	NA	0.81	0.333	0.178
A5SS	520	TNFAIP3	chr6	+	137867187	137867542	137867187	137867494	137871212	137871522	0	3	6	0	2	0	0	9	4	0	1	0	97	49	0.570399439	0.803465876	NA	0.431	1	NA	0.82	1	-0.194
A3SS	1102	FKBP8	chr19	-	18538215	18538436	18538215	18538433	18539370	18539459	0	2	2	0	0	5	0	2	1	2	5	0	52	49	0.570761829	0.79028561	NA	1	0.274	0	0.274	1	0.212
SE	7496	IFI44	chr1	+	78660553	78660654	78659311	78659483	78662703	78662878	0	0	36	0	0	6	0	23	1	0	5	0	98	49	0.575224839	0.778766917	NA	NA	0.75	NA	0.697	1	-0.099
SE	8238	LBR	chr1	-	225410290	225410416	225406663	225406832	225411336	225411440	0	0	18	0	0	3	1	10	1	0	1	0	98	49	0.575643233	0.778766917	NA	NA	0.75	1	0.833	1	-0.194
SE	11307	SP100	chr2	+	230464066	230464150	230462434	230462518	230466300	230466354	0	0	25	0	0	2	0	12	2	0	2	0	98	49	0.575655386	0.778766917	NA	NA	0.862	NA	0.75	1	-0.013
A5SS	3019	SERPINA1	chr14	-	94390419	94390692	94390456	94390692	94388559	94388663	0	4	5	0	0	4	3	3	2	0	1	0	86	49	0.578375981	0.804333654	NA	1	0.416	1	0.631	1	-0.169
A5SS	1552	PGS1	chr17	+	78419545	78419725	78419545	78419675	78424060	78424659	0	10	32	0	0	1	0	27	6	0	1	0	98	49	0.583295398	0.804333654	NA	1	0.941	NA	0.931	1	0.005
SE	6362	ZSWIM8	chr10	+	73791323	73791499	73790974	73791176	73791858	73792852	0	0	6	0	1	0	0	1	0	0	0	1	98	49	0.584480703	0.789995051	NA	0	1	NA	1	0	0
SE	3417	RALGAPA2	chr20	-	20676235	20676288	20653529	20653587	20680690	20680801	0	0	13	0	0	2	0	5	1	0	0	0	98	49	0.588967448	0.794630228	NA	NA	0.765	NA	1	1	-0.235
SE	3803	ZCCHC2	chr18	+	62558686	62558770	62556202	62556297	62560586	62560644	0	0	13	0	0	2	5	11	1	0	0	0	98	49	0.588967448	0.794630228	NA	NA	0.765	1	1	1	-0.235
SE	1922	PARP8	chr5	+	50824907	50824975	50822334	50822400	50826754	50826803	0	0	14	0	0	1	0	5	2	0	0	0	98	49	0.592731575	0.797560929	NA	NA	0.875	NA	1	1	-0.125
SE	3771	KIAA1109	chr4	+	122356620	122356755	122355795	122355990	122359556	122359722	0	0	14	0	0	1	0	7	2	0	0	0	98	49	0.592731575	0.797560929	NA	NA	0.875	NA	1	1	-0.125
SE	4732	CDA	chr1	+	20604927	20605039	20588950	20589283	20618451	20618907	0	0	14	0	0	1	0	5	2	0	0	0	98	49	0.592731575	0.797560929	NA	NA	0.875	NA	1	1	-0.125
SE	7937	TMC6	chr17	-	78124888	78124985	78124523	78124781	78125157	78125263	1	4	2	0	1	0	0	3	3	0	2	0	98	49	0.596766039	0.802271339	1	0.667	1	NA	0.429	1	0.174
A5SS	2516	NLRP1	chr17	-	5533291	5533384	5533303	5533384	5532821	5532984	0	0	1	0	0	7	0	5	0	0	4	1	61	49	0.597621998	0.815505018	NA	NA	0.103	NA	0.501	0	-0.148
SE	11312	ALDOA	chr16	+	30066884	30067038	30065672	30065927	30067233	30067366	0	0	1	0	0	7	0	0	0	0	5	1	98	49	0.59822268	0.803510892	NA	NA	0.067	NA	0	0	0.067
A3SS	1568	ENTPD1	chr10	+	95847445	95847706	95847568	95847706	95845356	95845596	0	4	26	0	0	1	2	4	1	0	0	0	98	49	0.600161541	0.824649445	NA	1	0.929	1	1	1	-0.035
SE	4503	PTPN6	chr12	+	6955150	6955267	6954804	6954994	6955371	6955485	0	2	28	0	0	1	0	24	1	0	1	0	98	49	0.600780878	0.805924887	NA	1	0.933	NA	0.923	1	0.005
SE	2258	KIAA0226	chr3	-	197717976	197718130	197705091	197705175	197736654	197736926	0	0	2	0	1	0	0	3	1	0	0	1	98	49	0.601092347	0.805924887	NA	0	1	NA	1	0.333	-0.166
SE	2636	PFKFB4	chr3	-	48539253	48539310	48538497	48538619	48539696	48539771	0	0	7	0	0	1	0	1	1	0	0	0	98	49	0.602141516	0.806612031	NA	NA	0.778	NA	1	1	-0.222
SE	9030	MYD88	chr3	+	38140387	38140568	38139863	38139998	38140756	38140848	0	1	15	0	1	1	0	0	2	0	0	0	98	49	0.608707381	0.814681384	NA	0.333	0.882	NA	NA	1	-0.392
SE	4423	PREX1	chr20	-	48650893	48651055	48649995	48650206	48651395	48651583	0	3	32	0	0	2	0	37	5	0	1	0	98	49	0.611249391	0.817355724	NA	1	0.889	NA	0.949	1	-0.03
SE	8705	CTSS	chr1	-	150750005	150750171	150747776	150747879	150751780	150752008	0	1	129	0	0	1	5	24	1	0	0	0	98	49	0.612568689	0.818391768	NA	1	0.985	1	1	1	-0.008
SE	8707	CTSS	chr1	-	150755000	150755150	150751780	150752008	150757857	150757980	0	4	234	0	0	1	6	39	7	0	0	0	98	49	0.614855148	0.820716951	NA	1	0.992	1	1	1	-0.004
SE	56	SASH3	chrX	+	129788430	129788577	129787974	129788070	129790939	129791081	0	0	8	0	0	1	0	12	1	0	1	0	98	49	0.622026252	0.829552314	NA	NA	0.8	NA	0.857	1	-0.128
SE	11031	STAT1	chr2	-	190998216	190998308	190997855	190998007	190999625	190999704	0	0	33	0	0	2	0	14	2	0	4	0	98	49	0.624060418	0.831527312	NA	NA	0.892	NA	0.636	1	0.074
RI	30	PLIN2	chr9	-	19126113	19126448	19126113	19126309	19126396	19126448	2	0	1	0	1	0	4	1	0	0	1	0	98	49	0.627465042	0.999202116	1	0	1	1	0.333	NA	0
SE	12347	CSNK1D	chr17	-	82253015	82253244	82252433	82252604	82255428	82255577	0	2	3	0	1	0	0	4	7	0	1	0	98	49	0.630476403	0.839332182	NA	0.5	1	NA	0.667	1	-0.084
SE	11030	STAT1	chr2	-	190979771	190979866	190978855	190979001	190980619	190980669	0	1	20	0	1	2	0	2	0	0	3	0	98	49	0.633284155	0.842323969	NA	0.333	0.833	NA	0.25	NA	0.333
SE	381	GNAQ	chr9	-	77794462	77794592	77728513	77728667	77797519	77797648	0	0	9	0	0	1	0	2	1	0	0	0	98	49	0.642008309	0.852419159	NA	NA	0.818	NA	1	1	-0.182
SE	8334	NCSTN	chr1	+	160355862	160355958	160354117	160354290	160356259	160356347	0	0	9	0	0	1	0	2	1	0	2	0	98	49	0.642008309	0.852419159	NA	NA	0.818	NA	0.333	1	0.151
SE	5678	GMIP	chr19	-	19635643	19635721	19635414	19635569	19636706	19636796	0	9	8	1	1	0	0	5	1	0	0	1	98	49	0.642808803	0.852728712	0	0.818	1	NA	1	0.333	-0.06
SE	8358	ARHGAP30	chr1	-	161056387	161056532	161054622	161054705	161069527	161069970	0	0	18	0	0	2	0	11	1	0	2	0	98	49	0.643507131	0.852902309	NA	NA	0.818	NA	0.733	1	-0.049
SE	4961	DDX3X	chrX	+	41337407	41337465	41333307	41334297	41341483	41341616	0	2	10	0	0	2	0	23	2	0	2	0	98	49	0.648092299	0.858222665	NA	1	0.714	NA	0.852	1	-0.069
SE	11305	SP100	chr2	+	230450171	230450255	230449087	230449150	230461261	230461414	0	2	23	0	1	0	8	15	5	0	1	0	98	49	0.652122993	0.862079722	NA	0.5	1	1	0.882	1	-0.211
SE	4428	PREX1	chr20	-	48747808	48747880	48745024	48745147	48827641	48827883	0	20	45	0	0	1	2	45	9	0	1	0	98	49	0.652532646	0.862079722	NA	1	0.957	1	0.957	1	-0.007
SE	9514	SIPA1	chr11	+	65645853	65645957	65644954	65645129	65646220	65646378	0	0	19	0	0	2	0	5	1	0	0	0	98	49	0.652725698	0.862079722	NA	NA	0.826	NA	1	1	-0.174
RI	230	ATG3	chr3	-	112532506	112534337	112532506	112532780	112534268	112534337	0	0	3	0	3	5	0	0	1	0	4	0	98	49	0.654603078	0.999202116	NA	0	0.231	NA	0	1	-0.385
SE	6617	AMPD2	chr1	+	109628642	109628806	109628363	109628495	109629108	109629235	0	1	2	0	1	0	0	7	2	0	5	1	98	49	0.656411303	0.866186294	NA	0.333	1	NA	0.412	0.5	0.211
RI	644	ATP6V0B	chr1	+	43977025	43978300	43977025	43977216	43977980	43978300	3	0	3	0	4	15	0	0	4	1	7	3	98	49	0.657605162	0.999202116	1	0	0.091	0	0	0.4	0.23
SE	2839	TYMP	chr22	-	50526000	50526141	50525752	50525918	50526245	50526476	1	8	30	0	1	0	2	8	14	0	0	1	98	49	0.661103457	0.871612716	1	0.8	1	1	1	0.875	-0.025
SE	3332	SAMHD1	chr20	-	36935028	36935189	36930759	36930875	36941038	36941111	0	0	21	0	0	1	0	3	2	0	1	0	98	49	0.661813092	0.871783591	NA	NA	0.913	NA	0.6	1	0.113
SE	8706	CTSS	chr1	-	150751780	150752008	150750005	150750171	150755000	150755150	0	3	181	0	0	1	7	24	1	0	0	0	98	49	0.66301558	0.872602817	NA	1	0.989	1	1	1	-0.006
SE	11325	ITGAL	chr16	+	30510880	30510960	30510360	30510471	30511049	30511136	0	2	13	0	0	1	0	1	1	0	0	0	98	49	0.665452763	0.875044185	NA	1	0.867	NA	1	1	-0.067
SE	7596	ATG16L2	chr11	+	72827187	72827293	72826702	72826823	72828358	72828508	0	1	41	0	0	1	1	4	1	0	0	0	98	49	0.668079787	0.8777307	NA	1	0.953	1	1	1	-0.024
SE	9411	WDFY4	chr10	+	48760346	48760440	48742967	48743548	48774457	48774672	0	0	11	0	0	1	0	1	1	0	0	0	98	49	0.668749011	0.877842588	NA	NA	0.846	NA	1	1	-0.154
A5SS	1756	PSME1	chr14	+	24138473	24138568	24138473	24138560	24138735	24138967	0	0	1	0	0	11	0	0	0	0	6	1	57	49	0.670811132	0.905940807	NA	NA	0.072	NA	0	0	0.072
SE	4459	CLEC7A	chr12	-	10125086	10125173	10123244	10123363	10125296	10125448	0	1	25	0	0	8	0	7	4	0	4	0	98	49	0.674199264	0.884224689	NA	1	0.61	NA	0.467	1	0.071
SE	7535	MCL1	chr1	-	150578243	150578491	150574550	150577491	150578842	150578963	0	5	123	1	4	15	1	70	5	0	30	0	98	49	0.678459838	0.889036737	0	0.385	0.804	1	0.538	1	-0.45
SE	8082	CSF3R	chr1	-	36473434	36473622	36472516	36472686	36473763	36473887	0	19	197	0	1	3	20	148	32	0	2	1	98	49	0.679743226	0.889942569	NA	0.905	0.97	1	0.974	0.941	-0.034
A5SS	2261	TLE3	chr15	-	70058158	70058291	70058185	70058291	70057458	70057658	1	1	4	0	0	1	0	4	1	0	1	0	76	49	0.682465604	0.91227545	1	1	0.721	NA	0.721	1	0.046
SE	6482	PADI2	chr1	-	17079263	17079415	17075678	17075823	17082544	17082652	0	0	12	0	0	1	0	0	1	0	0	0	98	49	0.685696462	0.895643369	NA	NA	0.857	NA	NA	1	-0.143
SE	6819	OAS1	chr12	+	112917546	112917700	112916508	112916738	112919486	112919907	0	0	12	0	0	1	0	4	1	0	1	0	98	49	0.685696462	0.895643369	NA	NA	0.857	NA	0.667	1	0.023
SE	8684	PTPRC	chr1	+	198696711	198696909	198692346	198692373	198699563	198699704	0	0	1	0	0	12	0	3	0	1	4	1	98	49	0.685885241	0.895643369	NA	NA	0.04	0	0.273	0	-0.051
SE	3887	LCP1	chr13	-	46148351	46148447	46144441	46144520	46150935	46151078	0	0	37	0	0	3	0	8	1	0	0	0	98	49	0.687307489	0.89672149	NA	NA	0.86	NA	1	1	-0.14
A3SS	803	TYMP	chr22	-	50526000	50526156	50526000	50526141	50526245	50526476	0	2	0	0	5	13	1	0	6	2	5	5	64	49	0.687426061	0.937399174	NA	0.234	0	0.277	0	0.479	-0.135
SE	13917	CD300A	chr17	+	74481725	74481833	74481288	74481326	74484000	74484798	0	4	15	0	0	1	0	6	0	0	0	1	98	49	0.688861707	0.89796977	NA	1	0.882	NA	1	0	0.441
SE	2765	LST1	chr6	+	31587640	31587733	31587199	31587318	31588562	31588909	0	0	2	0	1	6	0	1	0	0	2	1	98	49	0.690552858	0.899394233	NA	0	0.143	NA	0.2	0	-0.029
SE	1145	IGF2R	chr6	+	160088032	160088147	160083949	160084184	160089106	160089253	0	0	13	0	0	1	0	2	1	0	0	0	98	49	0.699215366	0.909888048	NA	NA	0.867	NA	1	1	-0.133
SE	13865	ACSL1	chr4	-	184770398	184770476	184768315	184768450	184773080	184773154	0	1	55	0	0	1	4	29	0	0	0	0	98	49	0.700770989	0.911123526	NA	1	0.965	1	1	NA	-0.018
SE	12581	CNN2	chr19	+	1036066	1036246	1032558	1032696	1036415	1036562	0	0	15	0	0	1	0	12	1	0	0	0	98	49	0.713986072	0.927503083	NA	NA	0.882	NA	1	1	-0.118
SE	8658	DUSP1	chr5	-	172769574	172769794	172768089	172769132	172770585	172771200	0	6	110	0	1	0	3	47	10	0	2	0	98	49	0.7163398	0.929757097	NA	0.75	1	1	0.922	1	-0.099
A3SS	775	LST1	chr6	+	31588496	31588909	31588562	31588909	31587199	31587318	0	3	13	0	1	6	4	11	0	0	2	1	98	49	0.718836501	0.97286143	NA	0.6	0.52	1	0.733	0	-0.018
SE	11238	APLP2	chr11	+	130137255	130137291	130135562	130135715	130140397	130140483	0	1	13	0	1	11	0	3	3	0	10	0	85	49	0.719023331	0.932434915	NA	0.366	0.405	NA	0.147	1	-0.188
SE	1142	IGF2R	chr6	+	160072764	160072884	160071909	160072036	160073212	160073469	0	1	16	0	0	1	0	18	2	0	1	0	98	49	0.724649909	0.938921391	NA	1	0.889	NA	0.9	1	-0.005
SE	1037	AOAH	chr7	-	36548611	36548686	36540318	36540491	36549438	36549475	0	0	16	0	0	1	0	3	1	0	0	0	98	49	0.727128809	0.941321792	NA	NA	0.889	NA	1	1	-0.111
RI	251	TYMP	chr22	-	50529495	50530085	50529495	50529731	50529903	50530085	1	7	17	0	0	1	7	16	4	0	1	0	98	49	0.727141068	0.999202116	1	1	0.895	1	0.889	1	0.002
SE	5784	DENND3	chr8	+	141141202	141141324	141138021	141138137	141144147	141144259	0	0	17	0	0	1	2	10	1	0	0	0	98	49	0.73240269	0.947333256	NA	NA	0.895	1	1	1	-0.105
SE	5221	CLEC4E	chr12	-	8536089	8536205	8533304	8534809	8539216	8539306	0	0	35	0	0	1	0	8	2	0	0	0	98	49	0.736969319	0.952420366	NA	NA	0.946	NA	1	1	-0.054
A5SS	2690	ARHGDIA	chr17	-	81869329	81869625	81869541	81869625	81869172	81869236	1	3	16	0	0	1	0	7	3	0	0	2	98	49	0.739861649	0.968908208	1	1	0.889	NA	1	0.429	0.249
A5SS	2132	RFWD2	chr1	-	176163814	176163891	176163826	176163891	176162868	176162988	0	1	17	0	1	5	0	5	0	0	6	0	61	49	0.745584037	0.968908208	NA	0.445	0.732	NA	0.401	NA	0.188
A3SS	1291	SLC6A6	chr3	+	14443623	14443863	14443627	14443863	14416411	14416453	0	0	19	0	0	1	0	9	1	0	0	0	53	49	0.746035627	0.999202116	NA	NA	0.946	NA	1	1	-0.054
A5SS	2703	IFI6	chr1	-	27669232	27669346	27669244	27669346	27668457	27668535	0	0	2	0	1	20	0	1	0	0	9	1	61	49	0.747020832	0.968908208	NA	0	0.074	NA	0.082	0	-0.004
SE	862	NAMPT	chr7	-	106254363	106254504	106253016	106253151	106261587	106261707	0	24	228	0	0	4	14	336	15	0	3	0	98	49	0.748257683	0.966178091	NA	1	0.966	1	0.982	1	-0.011
SE	7082	UBR2	chr6	+	42676055	42676191	42674125	42674193	42676782	42676873	0	0	20	0	0	1	0	6	1	0	0	0	98	49	0.751735186	0.96983518	NA	NA	0.909	NA	1	1	-0.091
SE	8416	GRAMD1A	chr19	+	35014188	35014387	35013540	35013691	35015823	35015967	0	3	13	0	0	0	0	1	0	0	0	2	98	49	0.752841225	0.970277462	NA	1	1	NA	1	0	0.5
SE	4184	STAT6	chr12	-	57107604	57107743	57107230	57107314	57108162	57108299	0	1	13	0	1	5	0	3	1	0	3	0	98	49	0.753369127	0.970277462	NA	0.333	0.565	NA	0.333	1	-0.218
SE	3752	ARHGAP27	chr17	-	45404268	45404334	45404028	45404096	45404444	45404528	0	2	4	0	0	3	0	0	1	0	5	0	98	49	0.759645396	0.976832296	NA	1	0.4	NA	0	1	0.2
SE	9027	MYD88	chr3	+	38139863	38139998	38138477	38139028	38140387	38140568	0	5	19	0	2	0	0	11	3	0	2	0	98	49	0.759758452	0.976832296	NA	0.556	1	NA	0.733	1	-0.089
SE	2840	TYMP	chr22	-	50526000	50526156	50525752	50525918	50526245	50526476	1	3	17	0	1	0	0	3	9	0	0	1	98	49	0.762114536	0.979024058	1	0.6	1	NA	1	0.818	-0.042
A3SS	3416	STAT3	chr17	-	42348388	42348539	42348388	42348517	42388278	42388495	0	2	14	0	1	6	0	2	1	0	2	0	71	49	0.764799772	0.999202116	NA	0.58	0.617	NA	0.408	1	-0.105
RI	356	DDIT3	chr12	-	57517268	57517753	57517268	57517438	57517705	57517753	1	2	7	0	0	1	2	28	3	0	2	0	98	49	0.777903504	0.999202116	1	1	0.778	1	0.875	1	-0.032
SE	9233	RPS24	chr10	+	78040200	78040225	78037193	78037304	78040614	78040715	0	0	1	0	0	26	0	8	0	0	6	1	74	49	0.781756708	0.999202116	NA	NA	0.025	NA	0.469	0	-0.209
RI	733	CELF2	chr10	+	11328925	11336673	11328925	11329097	11330455	11336673	0	0	29	0	0	1	1	10	1	0	1	0	98	49	0.794160768	0.999202116	NA	NA	0.935	1	0.833	1	-0.009
SE	8415	GRAMD1A	chr19	+	35013255	35013368	35011473	35011554	35013540	35013691	0	1	5	0	0	4	0	0	1	0	1	0	98	49	0.795544674	0.999202116	NA	1	0.385	NA	0	1	0.193
RI	1027	ARHGDIA	chr17	-	81869329	81869625	81869329	81869406	81869541	81869625	1	5	15	0	0	13	5	7	6	0	8	1	98	49	0.797337051	0.999202116	1	1	0.366	1	0.304	0.75	0.104
SE	11387	NLRC5	chr16	+	57054750	57054840	57051537	57051621	57055031	57055094	0	3	12	0	0	1	0	9	11	0	1	0	98	49	0.799295763	0.999202116	NA	1	0.857	NA	0.818	1	0.019
A5SS	2652	ACOX1	chr17	-	75978790	75979434	75978964	75979434	75978533	75978693	0	0	0	0	2	2	1	2	0	0	0	1	98	49	0.808907745	0.999208248	NA	0	0	1	1	0	-0.667
A3SS	3230	ARRB2	chr17	+	4720549	4720640	4720585	4720640	4720392	4720472	0	0	2	0	2	13	0	1	0	2	5	8	85	49	0.814988864	0.999202116	NA	0	0.081	0	0.103	0	0.006
SE	1331	TREM1	chr6	-	41280960	41281153	41275260	41276230	41282394	41282751	0	0	37	0	0	1	0	38	1	0	3	0	98	49	0.817902743	0.999202116	NA	NA	0.949	NA	0.864	1	0.017
SE	8687	PTPRC	chr1	+	198749415	198749549	198748108	198748199	198750491	198750626	0	1	72	0	0	1	9	51	0	0	0	0	98	49	0.818606032	0.999202116	NA	1	0.973	1	1	NA	-0.014
SE	6603	CCDC88B	chr11	+	64354003	64354170	64353714	64353813	64355193	64355400	0	4	4	0	1	2	0	2	2	0	0	1	98	49	0.823294092	0.999202116	NA	0.667	0.5	NA	1	0.5	-0.166
A5SS	1623	ADAR	chr1	-	154590183	154590409	154590261	154590409	154589756	154589928	0	4	24	0	0	3	0	17	2	0	1	0	98	49	0.823441962	0.999208248	NA	1	0.8	NA	0.895	1	-0.047
SE	5377	SYNE1	chr6	-	152300640	152300781	152293959	152294127	152301868	152302063	0	0	1	0	1	1	0	5	0	0	0	1	98	49	0.827502283	0.999202116	NA	0	0.333	NA	1	0	-0.334
SE	4122	XPO6	chr16	-	28117317	28117462	28112903	28113050	28121669	28121762	0	1	55	0	0	1	8	46	0	0	0	0	98	49	0.827647446	0.999202116	NA	1	0.965	1	1	NA	-0.018
SE	12729	PILRA	chr7	+	100389887	100390106	100374043	100374433	100397878	100397912	0	4	21	0	0	5	7	11	3	0	2	0	98	49	0.837221542	0.999202116	NA	1	0.677	1	0.733	1	-0.073
SE	11878	MGAM	chr7	+	142064383	142064522	142063498	142063586	142065334	142065468	0	4	8	0	0	2	3	8	2	0	1	0	98	49	0.840992744	0.999202116	NA	1	0.667	1	0.8	1	-0.1
A3SS	1500	ADAM8	chr10	-	133273253	133273443	133273253	133273430	133273761	133273838	0	6	11	0	0	2	2	5	3	0	1	0	62	49	0.846278337	0.999202116	NA	1	0.813	1	0.798	1	-0.026
A3SS	537	DBNL	chr7	+	44058428	44058480	44058431	44058480	44058128	44058280	0	0	6	0	3	2	0	1	0	0	1	1	52	49	0.847104365	0.999202116	NA	0	0.739	NA	0.485	0	0.127
SE	9515	SIPA1	chr11	+	65650400	65650479	65650137	65650192	65650568	65650920	0	0	9	0	1	0	0	4	2	0	0	2	98	49	0.852675535	0.999202116	NA	0	1	NA	1	0.333	-0.166
SE	6906	FCGR3B	chr1	-	161626144	161626402	161623197	161624639	161629777	161630032	0	0	59	0	0	1	2	19	1	0	2	0	98	49	0.85537265	0.999202116	NA	NA	0.967	1	0.826	1	0.025

A3SS	3415	STAT3	chr17	-	42316788	42316901	42316788	42316851	42317181	42317224	3	2	12	0	0	4	0	8	2	0	2	0	98	49	0.866784393	0.999202116	1	1	0.6	NA	0.667	1	0.033
SE	2906	RAF1	chr3	-	12590797	12590974	12587590	12587637	12591707	12591792	1	1	24	0	0	2	4	25	0	0	0	0	98	49	0.867186844	0.999202116	1	1	0.857	1	1	NA	-0.048
SE	144	IL2RG	chrX	-	71108598	71108695	71108276	71108346	71109227	71109390	0	1	4	0	2	0	0	12	1	0	10	0	98	49	0.876241607	0.999202116	NA	0.2	1	NA	0.375	1	-0.088
SE	4365	LRRK2	chr12	+	40322034	40322181	40321033	40321188	40322318	40322510	0	1	45	0	0	2	0	21	0	0	0	0	98	49	0.881723484	0.999202116	NA	1	0.918	NA	1	NA	-0.041
RI	809	ZFP36L1	chr14	-	68787654	68790492	68787654	68788809	68789526	68790492	0	4	17	0	0	2	8	27	5	0	2	0	98	49	0.883440896	0.999202116	NA	1	0.81	1	0.871	1	-0.052
SE	9293	ABCA7	chr19	+	1054569	1054694	1054192	1054341	1054779	1054878	0	1	4	0	0	3	0	4	1	0	2	0	98	49	0.891129146	0.999202116	NA	1	0.4	NA	0.5	1	-0.05
SE	6059	HCLS1	chr3	-	121647318	121647448	121644817	121644928	121657278	121657352	0	1	33	0	0	1	3	18	0	0	0	0	98	49	0.89175776	0.999202116	NA	1	0.943	1	1	NA	-0.028
SE	9137	CLK1	chr2	-	200854615	200854695	200853902	200853993	200855003	200855086	0	1	8	0	0	2	0	18	0	0	0	0	98	49	0.89175776	0.999202116	NA	1	0.667	NA	1	NA	-0.166
A3SS	1625	RPS3	chr11	+	75402303	75402446	75402351	75402446	75401639	75401733	0	0	2	0	2	7	3	0	0	0	15	0	97	49	0.906255181	0.999202116	NA	0	0.126	1	0	NA	-0.437
SE	1126	RNASET2	chr6	-	166948569	166948625	166946681	166946739	166952487	166952548	0	8	51	0	1	0	0	18	2	0	1	0	98	49	0.909429153	0.999202116	NA	0.8	1	NA	0.9	1	-0.05
SE	11303	SP100	chr2	+	230440475	230440557	230417590	230417665	230442936	230443099	0	0	1	0	1	3	0	0	0	0	7	0	98	49	0.909814497	0.999202116	NA	0	0.143	NA	0	NA	0.071
SE	1834	ARRDC3	chr5	-	91376620	91376768	91375510	91375613	91378693	91378775	0	1	13	0	0	6	0	13	0	0	0	0	98	49	0.910129019	0.999202116	NA	1	0.52	NA	1	NA	-0.24
SE	11821	SSH2	chr17	-	29650653	29650800	29648143	29648344	29655560	29655607	0	1	32	0	0	3	0	13	0	0	0	0	98	49	0.910129019	0.999202116	NA	1	0.842	NA	1	NA	-0.079
A3SS	3715	TYROBP	chr19	-	35907217	35907264	35907217	35907261	35907445	35907580	0	11	42	0	0	4	0	9	9	0	1	0	52	49	0.912303287	0.999202116	NA	1	0.908	NA	0.895	1	0.006
SE	3136	IFNAR2	chr21	+	33248708	33248854	33246717	33246890	33252661	33252830	0	1	7	0	0	1	0	12	0	0	0	0	98	49	0.91423345	0.999202116	NA	1	0.778	NA	1	NA	-0.111
SE	6130	WDR26	chr1	-	224431681	224431781	224431476	224431581	224433683	224434299	0	1	6	0	0	1	0	12	0	0	0	0	98	49	0.91423345	0.999202116	NA	1	0.75	NA	1	NA	-0.125
SE	12143	TRIM25	chr17	-	56895925	56895952	56895520	56895604	56899114	56899180	0	1	3	0	0	1	0	9	0	0	0	0	76	49	0.917397557	0.999202116	NA	1	0.659	NA	1	NA	-0.17
A3SS	1626	RPS3	chr11	+	75405582	75406913	75405613	75406913	75404671	75404868	1	0	4	0	1	4	0	0	0	0	6	0	80	49	0.917875723	0.999202116	1	0	0.38	NA	0	NA	0.46
SE	3618	NIN	chr14	-	50821873	50822077	50806736	50806818	50830463	50830517	0	3	8	0	0	1	0	7	6	0	0	1	98	49	0.918267371	0.999202116	NA	1	0.8	NA	1	0.75	0.025
SE	8078	NADK	chr1	-	1756502	1756608	1756257	1756343	1757180	1757310	0	1	24	0	0	3	0	11	0	0	0	0	98	49	0.918460091	0.999202116	NA	1	0.8	NA	1	NA	-0.1
A3SS	2809	SRRT	chr7	+	100888041	100888143	100888053	100888143	100887702	100887859	0	1	1	0	0	3	0	7	0	0	0	0	61	49	0.919108663	0.999202116	NA	1	0.211	NA	1	NA	-0.394
SE	10061	TPT1	chr13	-	45339496	45339602	45338659	45338776	45339993	45340184	0	6	79	0	0	3	10	61	0	0	0	0	98	49	0.922183161	0.999202116	NA	1	0.929	1	1	NA	-0.035
RI	894	STX4	chr16	+	31034225	31034536	31034225	31034325	31034461	31034536	0	1	5	0	0	3	6	10	0	0	0	0	98	49	0.922793683	0.999202116	NA	1	0.455	1	1	NA	-0.272
SE	5493	ADAM8	chr10	-	133263687	133263765	133262415	133263233	133267351	133267417	0	4	10	0	2	2	3	19	13	0	5	4	98	49	0.923886696	0.999202116	NA	0.5	0.714	1	0.655	0.619	-0.151
SE	1583	SYTL3	chr6	+	158663204	158663378	158662749	158662812	158665394	158665613	0	1	1	0	0	1	0	2	0	0	0	1	98	49	0.925071736	0.999202116	NA	1	0.333	NA	1	0	0.166
SE	4333	NCF2	chr1	-	183573184	183573292	183570779	183570839	183574486	183574621	2	5	63	0	0	1	0	47	0	0	0	0	98	49	0.925576519	0.999202116	1	1	0.969	NA	1	NA	-0.01
SE	8261	ARHGEF11	chr1	-	156957791	156957815	156956419	156956564	156958741	156958864	0	2	7	0	0	0	0	2	0	0	0	1	73	49	0.92640979	0.999202116	NA	1	1	NA	1	0	0.5
A5SS	2405	SP100	chr2	+	230467119	230467215	230467119	230467206	230469042	230469096	0	3	13	0	0	1	0	15	0	0	0	0	58	49	0.926498131	0.999208248	NA	1	0.917	NA	1	NA	-0.041
RI	1099	RPL28	chr19	+	55387929	55392083	55387929	55388048	55388242	55392083	1	0	1	0	0	13	0	0	1	4	0	0	98	49	0.927341297	0.999202116	1	NA	0.037	0	NA	1	0.018
SE	11228	PTPN12	chr7	+	77585542	77585581	77583554	77583650	77592184	77592256	0	1	9	0	0	1	0	8	0	0	0	0	88	49	0.9284209	0.999202116	NA	1	0.834	NA	1	NA	-0.083
SE	10669	LAPTM5	chr1	-	30742455	30742549	30741639	30741716	30757658	30757836	0	3	22	0	0	2	1	26	0	0	0	0	98	49	0.928473035	0.999202116	NA	1	0.846	1	1	NA	-0.077
SE	4334	NCF2	chr1	-	183573184	183573292	183570779	183570839	183577598	183577707	2	5	53	0	0	1	0	44	0	0	0	0	98	49	0.928484533	0.999202116	1	1	0.964	NA	1	NA	-0.012
SE	11373	CYLD	chr16	+	50744729	50744866	50742761	50742841	50749575	50750202	0	0	0	0	2	1	2	0	0	0	7	2	98	49	0.928492447	0.999202116	NA	0	0	1	0	0	-0.333
A5SS	2858	RPL13A	chr19	+	49489849	49489922	49489849	49489912	49490231	49490297	0	3	11	0	0	1	4	17	0	0	0	0	59	49	0.928715497	0.999208248	NA	1	0.901	1	1	NA	-0.049
SE	1923	EMB	chr5	-	50410876	50410965	50405724	50405852	50411196	50411383	1	1	22	0	0	1	1	9	0	0	0	0	98	49	0.928729465	0.999202116	1	1	0.917	1	1	NA	-0.028
SE	3671	CALCOCO2	chr17	+	48846442	48846514	48841697	48841887	48848063	48848166	0	0	1	0	1	5	0	0	0	0	4	0	98	49	0.930162674	0.999202116	NA	0	0.091	NA	0	NA	0.045
SE	10146	GALC	chr14	-	87988454	87988523	87988143	87988207	87992969	87993271	0	1	5	0	1	0	0	5	12	0	0	2	98	49	0.93135784	0.999202116	NA	0.333	1	NA	1	0.75	-0.209
A3SS	538	DBNL	chr7	+	44058877	44058983	44058901	44058983	44058428	44058480	0	0	2	0	1	3	3	1	0	0	2	1	73	49	0.931399982	0.999202116	NA	0	0.309	1	0.251	0	-0.262
SE	10209	RFWD2	chr1	-	176136487	176136547	176135009	176135086	176149005	176149074	0	1	16	0	0	4	3	15	3	0	3	0	98	49	0.931793753	0.999202116	NA	1	0.667	1	0.714	1	-0.071
SE	5478	MICAL2	chr11	+	12204249	12204457	12162078	12162419	12208022	12208139	0	1	17	0	0	1	0	8	0	0	0	0	98	49	0.932280727	0.999202116	NA	1	0.895	NA	1	NA	-0.052
SE	11480	AP1G1	chr16	-	71748250	71748378	71746587	71746692	71749893	71749983	0	1	4	0	0	1	0	8	0	0	0	0	98	49	0.932280727	0.999202116	NA	1	0.667	NA	1	NA	-0.166
SE	13003	HNRNPU	chr1	-	244858010	244858274	244857597	244857717	244858728	244858841	0	2	18	0	0	2	2	13	0	0	0	0	98	49	0.932958395	0.999202116	NA	1	0.818	1	1	NA	-0.091
SE	7216	LSP1	chr11	+	1884279	1884323	1883931	1884024	1884499	1884581	0	5	71	0	0	1	0	36	0	0	0	0	93	49	0.933472812	0.999202116	NA	1	0.974	NA	1	NA	-0.013
SE	4366	LRRK2	chr12	+	40354298	40354492	40351538	40351733	40356114	40356187	0	2	26	0	0	2	0	15	0	0	0	0	98	49	0.934689043	0.999202116	NA	1	0.867	NA	1	NA	-0.067
SE	13049	DHX34	chr19	+	47357865	47358120	47355038	47355350	47359967	47360070	0	2	24	0	0	1	0	15	0	0	0	0	98	49	0.934689043	0.999202116	NA	1	0.923	NA	1	NA	-0.038
SE	3908	MYL6	chr12	+	56160625	56160670	56160242	56160320	56161386	56161582	0	0	1	0	3	15	0	0	0	0	11	0	94	49	0.935467898	0.999202116	NA	0	0.034	NA	0	NA	0.017
A3SS	2959	MTCH1	chr6	-	36977633	36977784	36977633	36977691	36978077	36978155	0	1	1	0	0	3	2	6	0	0	0	0	98	49	0.936414148	0.999202116	NA	1	0.143	1	1	NA	-0.428
RI	864	SUMO1	chr2	-	202206179	202207321	202206179	202206803	202207252	202207321	0	1	10	0	0	1	0	6	0	0	0	0	98	49	0.936414148	0.999202116	NA	1	0.833	NA	1	NA	-0.084
SE	11834	NCOA1	chr2	+	24705085	24705233	24697657	24697798	24706567	24707888	0	1	11	0	0	1	0	6	0	0	0	0	98	49	0.936414148	0.999202116	NA	1	0.846	NA	1	NA	-0.077
SE	12434	SPAG9	chr17	-	50984922	50984990	50982523	50982672	50985697	50985778	0	1	8	0	0	1	0	6	0	0	0	0	98	49	0.936414148	0.999202116	NA	1	0.8	NA	1	NA	-0.1
SE	13879	ZEB2	chr2	-	144404835	144405024	144403915	144404130	144429768	144430026	0	1	3	0	0	1	0	6	0	0	0	0	98	49	0.936414148	0.999202116	NA	1	0.6	NA	1	NA	-0.2
A3SS	3220	LILRB3	chr19	-	54217318	54217477	54217318	54217474	54218360	54218413	0	2	8	0	3	11	0	1	3	4	6	1	52	49	0.937019423	0.999202116	NA	0.386	0.407	0	0.136	0.739	0.105
RI	712	CD164	chr6	-	109366513	109369017	109366513	109368325	109368921	109369017	0	1	31	0	0	1	4	7	0	0	0	0	98	49	0.937374675	0.999202116	NA	1	0.939	1	1	NA	-0.03
SE	2080	HERC5	chr4	+	88487068	88487179	88486114	88486228	88489165	88489336	0	1	19	0	0	1	0	7	0	0	0	0	98	49	0.937374675	0.999202116	NA	1	0.905	NA	1	NA	-0.047
SE	2091	HSD17B11	chr4	-	87374698	87374830	87372708	87372815	87382254	87382362	0	3	26	0	0	1	2	20	0	0	0	0	98	49	0.939243248	0.999202116	NA	1	0.929	1	1	NA	-0.035
RI	861	JUP	chr17	-	41754606	41755895	41754606	41755432	41755729	41755895	1	0	0	0	0	0	5	1	0	0	1	0	98	49	0.94318207	0.999202116	1	NA	NA	1	0.333	NA	0.334
SE	8746	BEST1	chr11	+	61959891	61960043	61959497	61959578	61962254	61962893	0	1	3	0	0	1	0	5	0	0	0	0	98	49	0.94318207	0.999202116	NA	1	0.6	NA	1	NA	-0.2
SE	9188	GIT2	chr12	-	109951166	109951316	109947255	109947504	109953091	109953234	0	1	5	0	0	1	0	5	0	0	0	0	98	49	0.94318207	0.999202116	NA	1	0.714	NA	1	NA	-0.143
SE	1843	VCAN	chr5	+	83537006	83542268	83512102	83512396	83545536	83545650	0	2	51	0	0	1	0	10	0	0	0	0	98	49	0.944027198	0.999202116	NA	1	0.962	NA	1	NA	-0.019
SE	3459	RASSF2	chr20	-	4795814	4795966	4792538	4792627	4798009	4798085	0	4	29	0	0	1	0	20	0	0	0	0	98	49	0.944343057	0.999202116	NA	1	0.935	NA	1	NA	-0.032
SE	3950	ARID4B	chr1	-	235213768	235214026	235196030	235196115	235219792	235219968	0	0	1	0	1	2	0	0	0	0	3	0	98	49	0.94451324	0.999202116	NA	0	0.2	NA	0	NA	0.1
SE	8841	C1orf43	chr1	-	154219835	154219937	154214456	154214574	154220341	154220797	0	0	1	0	1	1	0	0	0	0	3	0	98	49	0.94451324	0.999202116	NA	0	0.333	NA	0	NA	0.167
SE	12024	KLHL2	chr4	+	165238777	165238899	165228806	165228913	165263196	165263359	0	0	4	0	1	2	0	0	0	0	3	0	98	49	0.94451324	0.999202116	NA	0	0.5	NA	0	NA	0.25
A5SS	192	TBXAS1	chr7	+	139875584	139875666	139875584	139875637	139911224	139911321	0	0	1	0	1	15	5	0	0	0	3	0	78	49	0.945027512	0.999208248	NA	0	0.04	1	0	NA	-0.48
[truncated: 17,473,833 more chars]
